# Construction of a high-density genetic linkage map and mapping of quantitative trait loci for growth-related traits in silver carp (*Hypophthalmichthys molitrix*)

**DOI:** 10.1038/s41598-019-53469-8

**Published:** 2019-11-25

**Authors:** Xinhua Wang, Haiyang Liu, Meixia Pang, Beide Fu, Xiaomu Yu, Shunping He, Jingou Tong

**Affiliations:** 1 College of Animal Science and Technology, Henan University of Animal Husbandry and Economy, Zhengzhou, 450046 China; 2 State Key Laboratory of Freshwater Ecology and Biotechnology, Institute of Hydrobiology, Chinese Academy of Sciences, Innovation Academy of Seed Design, Chinese Academy of Sciences, Wuhan, 430072 China; 3 Key Laboratory of Aquatic Biodiversity and Conservation of the CAS, Institute of Hydrobiology, the Chinese Academy of Sciences, Wuhan, 430072 China

**Keywords:** Reverse transcription polymerase chain reaction, Reverse transcription polymerase chain reaction, Comparative genomics, Genetic markers, Genetic markers

## Abstract

High-density genetic map and quantitative trait loci (QTL) mapping are powerful tools for identifying genomic regions that may be responsible for such polygenic trait as growth. A high-density genetic linkage map was constructed by sequencing 198 individuals in a F_1_ family of silver carp (*Hypophthalmichthys molitrix*) in this study. This genetic map spans a length of 2,721.07 cM with 3,134 SNPs distributed on 24 linkage groups (LGs). Comparative genomic mapping presented a high level of syntenic relationship between silver carp and zebrafish. We detected one major and nineteen suggestive QTL for 4 growth-related traits (body length, body height, head length and body weight) at 6, 12 and 18 months post hatch (mph), explaining 10.2~19.5% of phenotypic variation. All six QTL for growth traits of 12 mph generally overlapped with QTL for 6 mph, while the majority of QTL for 18 mph were identified on two additional LGs, which may reveal a different genetic modulation during early and late muscle growth stages. Four potential candidate genes were identified from the QTL regions by homology searching of marker sequences against zebrafish genome. *Hepcidin*, a potential candidate gene identified from a QTL interval on LG16, was significantly associated with growth traits in the analyses of both phenotype-SNP association and mRNA expression between small-size and large-size groups of silver carp. These results provide a basis for elucidating the genetic mechanisms for growth and body formation in silver carp, a world aquaculture fish.

## Introduction

Molecular markers are the basis for high-resolution genetic linkage map construction and quantitative trait loci (QTL) fine-mapping, which provide powerful tools for genetic analyses of economic traits in fish^1,2^. With the rapid development of the biotechnology, dominant DNA markers were gradually replaced by co-dominant markers in genetic mapping^1,3^, including single nucleotide polymorphisms (SNPs) and microsatellites (SSRs). As the second-generation marker, microsatellites have the merits of high information content and transferability. However, low efficiency and relatively small amount of SSRs impeded the high-density genetic map construction, which limits their application in fine-scale QTL mapping and other studies. Next generation sequencing technologies (NGS) created a new path for developing SNP markers and made them currently preferable genetic markers by virtue of abundance, simplicity, stability and possibly direct relatedness with phenotype^4,5^. Among the multiple genotyping-by-sequencing (GBS) methods, restriction site-associated DNA (RAD)^6^ and its derivative methods, such as double-digest restriction site-associated DNA (dd-RAD)^7^, 2b-restriction site-associated DNA (2b-RAD)^8^, genotyping by genome reducing and sequencing (GGRS)^9^, specific-locus amplified fragments sequencing (SLAF)^10^ and genome wide sampling sequencing (GWSS)^11^, have been widely used for the construction of high-density genetic maps. Among these RAD-related methods, 2b-RAD technology possesses the benefits of simplest protocol, even distribution on genome, uniform fragments and tunable genome coverage^8^, and this method has been successfully used for genetic maps construction in aquatic animals, such as pearl oyster (*Pinctada fucata martensii*)^5^, Zhikong scallop (*Chlamys farreri*)^12^, sea cucumber (*Apostichopus japonicus*)^13^, bighead carp (*Hypophthalmichthys nobilis*)^14^, crucian carp (*Carassius auratus*)^15^, etc.

Marker-assisted selection (MAS) is an efficient approach for increasing the accuracy and efficiency of selection by using markers tightly linked to quantitative trait loci (QTL) as a substitute to assist phenotypic screening^16^. Identification of QTL related to phenotypic traits is the basis for the application of genetic markers in fish breeding. Fuji *et al*.^17^ have successfully applied a microsatellite marker locating in a major QTL for resistance to lymphocystis disease in Japanese flounder (*Paralichthys olivaceus*). As a major interest in aquaculture, growth is one of the most complex traits that may be affected by multi-loci across the genome and environmental factors. Growth-related QTL mapping have been reported in many aquatic animals, such as sea cucumber^13^, bighead carp^14,18^, Asian seabass (*Lates calcarifer*)^19^, Japanese flounder (*Paralichthys olivaceus*)^20^, common carp (*Cyprinus carpio*)^21^, large yellow croaker (*Larimichthys crocea*)^22^, rainbow trout (*Oncorhynchus mykiss*)^23^, tilapia (*Oreochromis niloticus*)^24^, and so on. QTL-related markers and candidate genes were also identified, some of which showed potentials as candidate genes for marker-assisted selection^19,21,24^.

As a filter-feeding fish, silver carp (*Hypophthalmichthys molitrix*) has great value for biological control of bloom-forming cyanobacteria in ponds, reservoirs and lakes^25^. In China and some Asian countries, silver carp is also one of the most important food fish. In pond culture, silver carp usually takes 3 years to reach sexual maturity^26^. According to the recent statistics, annual production of silver carp had exceeded 5.12 million metric tons in the world, of which more than 80% were produced in China (FAO, 2015). However, during past two decades, both population resources in natural waters and production traits in aquaculture (such as growth rate) of silver carp declined in China, and genetic breeding programs for economically important traits are thus required^27,28^. To date, very limited genetic and genomic studies have been conducted in silver carp^27,29,30^, and the resolution of current genetic maps is not high enough to perform QTL fine mapping.

Nowadays, rapid and cost-effective manner for SNP developing and genotyping makes the construction of high-density genetic maps easier to achieve^4^. This study aims to construct a high-density genetic linkage map using 2b-RAD technology and detect QTL intervals associating with growth traits of different stages in silver carp. Comparative mapping between silver carp genetic map and zebrafish (*Danio rerio*, a model fish of Cyprinidae) genome was also performed to identify potential candidate genes for growth traits. *Hepcidin*, a potential candidate gene, was selected for expression analysis and association study for growth. This study would facilitate the genetic analysis of growth traits and provide valuable genomic resources for potential molecular breeding programs in silver carp.

## Results

### Characterization of the phenotypic traits

In our study, the growth traits (GT) for 6, 12 and 18 months post hatch (mph) were abbreviated as GT1, GT2 and GT3, respectively, and four phenotypic parameters, including body length (BL), body height (BH), head length (HL) and body weight (BW), were measured for all progenies (Supplementary Table A1). Accordingly, a total of 12 morphometric traits were recorded and named as BL1, BH1, HL1, BW1, BL2, BH2, HL2, BW2, BL3, BH3, HL3 and BW3, respectively. The average values of BW1, BW2 and BW3 were 92.2 ± 8.6 g, 194.2 ± 17.6 g and 686.2 ± 57.8 g, respectively. All 12 growth and body shape traits showed significant correlation with each other (*P* < 0.05; Table 1). The highest correlation value (*r* = 0.893) was observed between BW2 and BL2, and the lowest correlation value (*r* = 0.190) was observed between HL2 and HL3. Highly significant correlations were detected between different growth traits measured at the same stage, and the correlation values were ranged from 0.666 to 0.815 (within GT1), 0.700 to 0.893 (within GT2) and 0.544 to 0.883 (within GT3), respectively. For the traits of different growth stages, the correlations between GT1 and GT2 (*r* = 0.519–0.868) were significantly higher than that between GT1/GT2 and GT3 (*r* = 0.190–0.572).

**Table 1:** Pearson correlation coefficients (*r*) between 12 growth traits of silver carp.

Traits	BL1	BH1	HL1	BW1	BL2	BH2	HL2	BW2	BL3	BH3	HL3	BW3
BL1	1											
BH1	0.740**	1										
HL1	0.715**	0.728**	1									
BW1	0.815**	0.796**	0.666**	1								
BL2	0.868**	0.676**	0.665**	0.726**	1							
BH2	0.629**	0.720**	0.627**	0.595**	0.774**	1						
HL2	0.560**	0.624**	0.594**	0.519**	0.700**	0.844**	1					
BW2	0.759**	0.706**	0.655**	0.716**	0.893**	0.833**	0.745**	1				
BL3	0.555**	0.285**	0.273**	0.403**	0.557**	0.383**	0.290**	0.522**	1			
BH3	0.199*	0.372**	0.311**	0.298**	0.250**	0.482**	0.312**	0.389**	0.611**	1		
HL3	0.248**	0.255**	0.310**	0.211*	0.255**	0.284**	0.190*	0.301**	0.544**	0.622**	1	
BW3	0.441**	0.402**	0.305**	0.455**	0.492**	0.487**	0.292**	0.572**	0.798**	0.883**	0.623**	1

BL, body length; BH, body height; HL, head length; BW, body weight. The digits of suffix (1, 2 and 3) indicated the growth traits measured at 6 months, 12 months and 18 months after hatch, respectively. “*”, Significant at *P* < 0.05; “**”, Significant at *P* < 0.01.

### 2b-RAD genotyping

Based on the digital-enzyme-cut analysis, about 0.098 million *BcgI* restriction sites were identified from the genome sequence of silver carp genome sequence. The amount of the raw reads generated from the male parent, the female parent and every progeny were 6.3 million, 6.7 million and 1.41 million, respectively. After initial trimming, the male parent, the female parent and the progenies obtained 5.9 million reads, 6.4 million reads and 267.6 million reads for further analysis. A total of 73,629 representative tags were produced after clustering of parental reads, including 58,351 co-dominant tags and 15,278 dominant tags. The statistical sequencing depth corresponded to 85-fold in the male, 91-fold in the female and 19-fold in the progenies. After filtering low-quality tags, 52,472 co-dominant tags and 11,599 dominant tags were obtained and used to construct reference tags. High-quality sequences of the 198 progenies were mapped on the reference tags and a total of 6,603 markers (2,175 co-dominant markers and 4,428 dominant markers) showed heterozygous in at least one parent.

### Construction of the high-density linkage map

**Figure 1:**
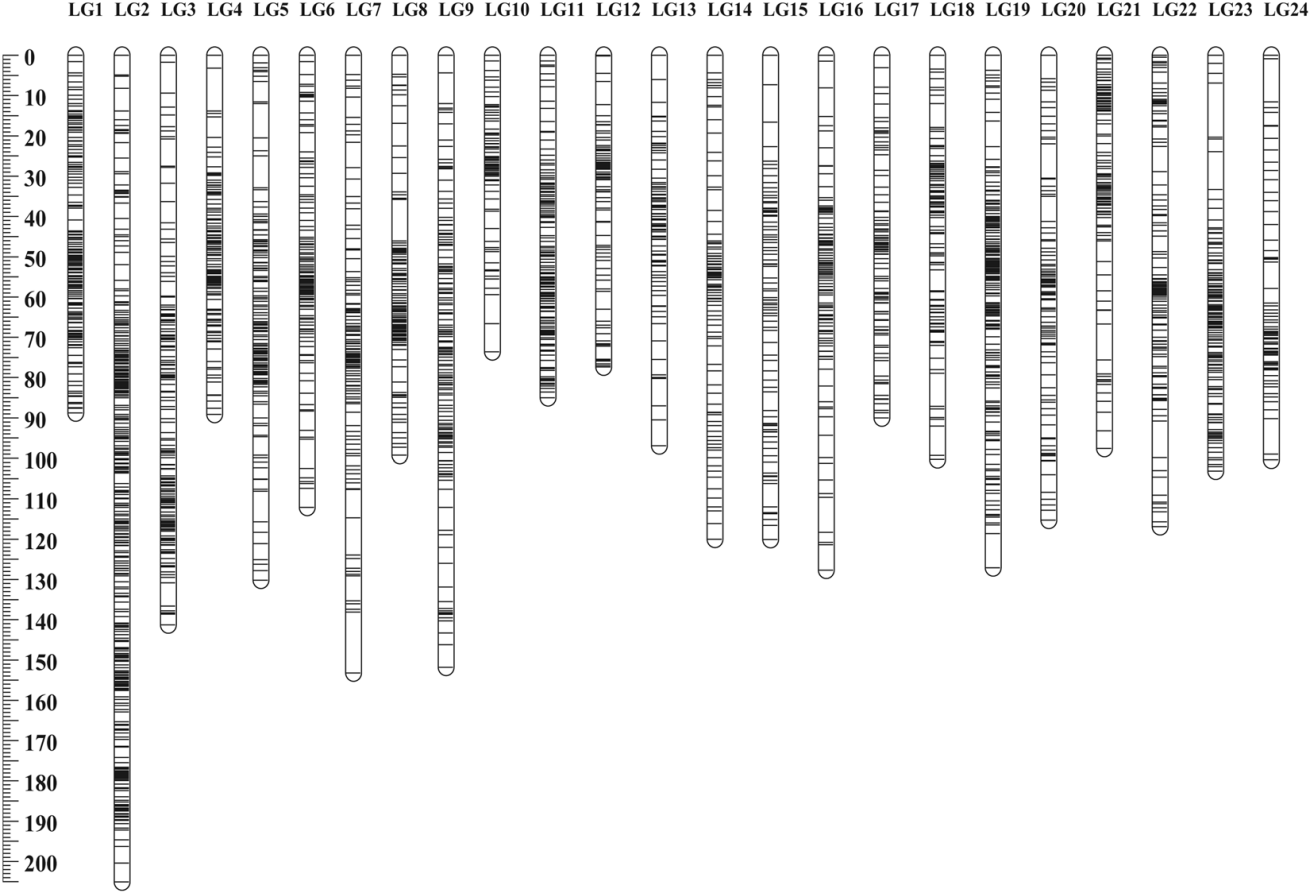
The high-density sex-averaged genetic linkage map for silver carp. A black bar indicated a 2b-RAD marker. The scaleplate on the left indicated genetic distance (centiMorgan as unit).

After removing those sequences with low-quality and highly significant deviation from the segregation ratio, 3,253 polymorphic markers including 2,114 dominant markers and 1,139 co-dominant markers were obtained for linkage map construction. Finally, 3,134 markers were successfully grouped into 24 linkage groups (LGs), corresponding to the haploid chromosome number of *H*. *molitrix*^31^. The sex-averaged genetic linkage map contained 2,013 dominant markers and 1,121 co-dominant markers (Fig. 1 and Supplementary Table A2) and spanned a genetic length of 2,721.07 centi-Morgans (cM) with an average marker interval of 0.86 cM (Table 2). The length of each linkage group ranged from 73.5 to 205.1 cM with an average of 113.3 cM. According to the two different algorithms^32,33^, the expected map length was estimated to be 2,762.3 cM (*G*_*e1*_) and 2,766.2 cM (*G*_*e2*_) with an average of 2,764.2 cM (*G*_*e*_). The map coverage (*C*_*oa*_) of this sex-averaged map was 98.4%.

**Table 2:** Information of the sex-averaged genetic linkage map for silver carp.

Linkage group	Number of markers	Genetic length (cM)	Average marker interval (cM)
1	167	88.7	0.53
2	358	205.1	0.57
3	168	141.3	0.84
4	116	89.1	0.76
5	146	130.1	0.89
6	127	112	0.88
7	128	153.2	1.19
8	119	99.2	0.83
9	154	151.8	0.98
10	72	73.5	1.02
11	155	85	0.54
12	88	77.4	0.87
13	86	96.8	1.12
14	101	119.9	1.18
15	85	120	1.41
16	113	127.7	1.13
17	98	89.9	0.91
18	101	100.3	0.99
19	199	127.06	0.63
20	104	115.3	1.1
21	108	97.55	0.9
22	129	116.86	0.9
23	140	103	0.73
24	72	100.3	1.39
Total	3,134	2,721.07	0.86

**Figure 2:**
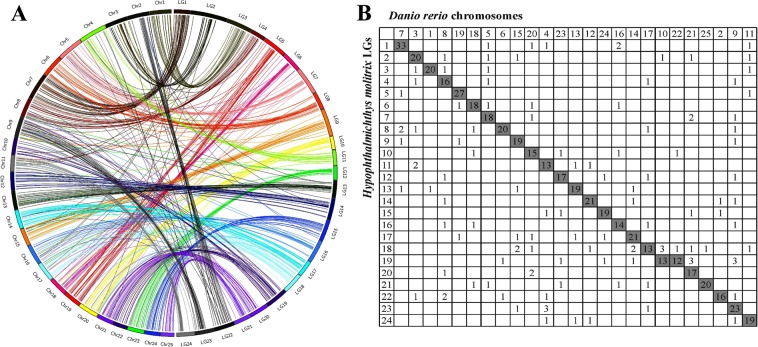
Genomic synteny between linkage groups (LG) of silver carp and chromosomes (Chr) of zebrafish. Genomic synteny was visualized using Circos diagram (**A**) and Oxford grids (**B**).

A total of 1,731 markers were exclusively aligned to the genome sequence of silver carp and obtained extended sequence for comparative genome analysis with zebrafish. Finally, 581 markers were uniquely aligned to the chromosomes of zebrafish (Fig. 2A,B), with 464 (80%) markers assigned into 25 syntenic boxes. On the orthologous map, the LG19 of silver carp corresponded to two chromosomes of zebrafish (Chr10 and Chr22), and other LGs apparently corresponded to a particular chromosome (Fig. 2A,B).

### QTL detection and identification of potential candidate gene

**Figure 3:**
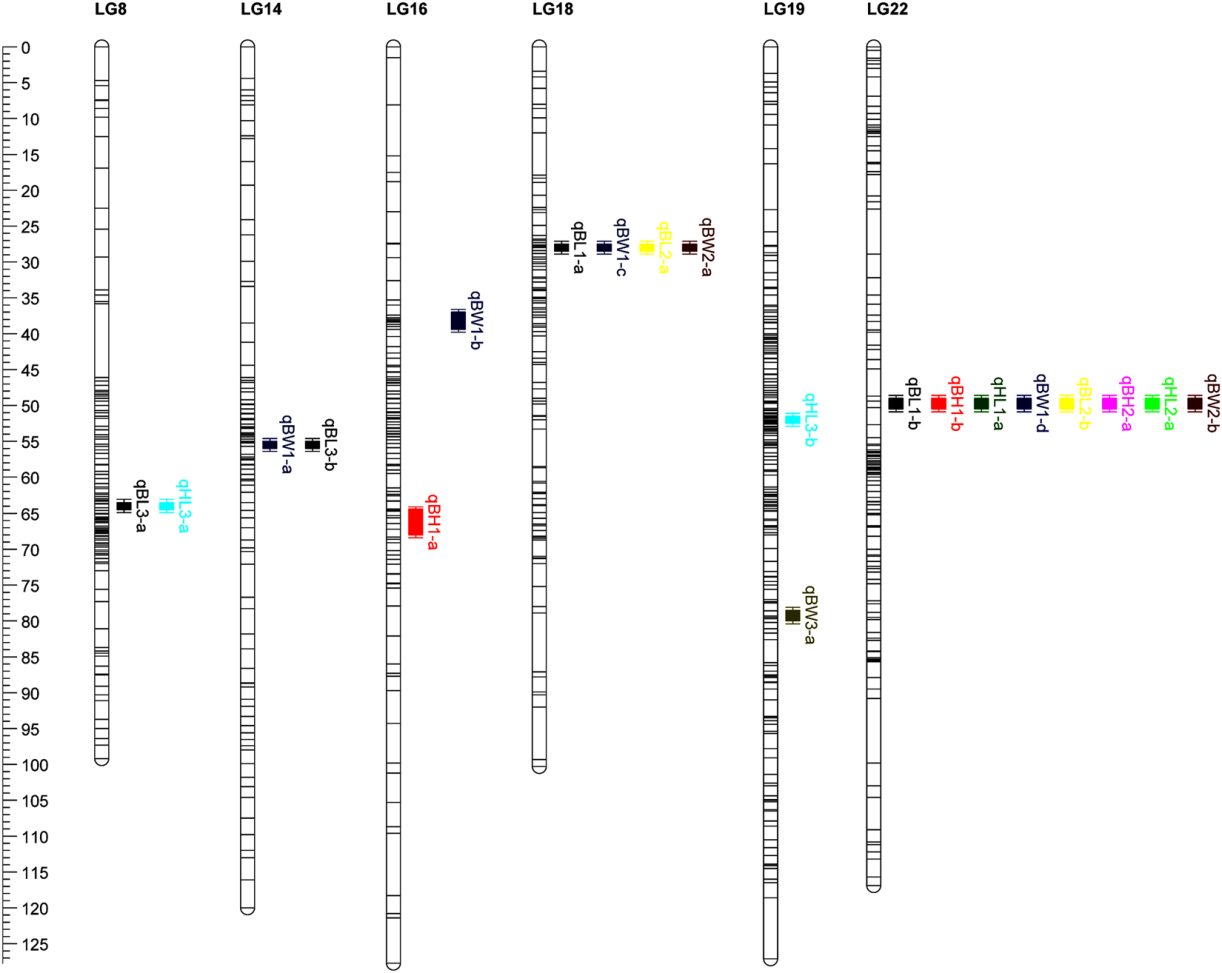
Distribution of growth-related QTL on 6 different linkage groups of silver carp.

Genome-wide (GW) and chromosome-wide (CW) LOD (logarithm of odds) threshold for each growth trait were listed in Table 3. One major and nineteen suggestive growth-related QTL were detected on six LGs (Fig. 3) and the detailed information were described as follows. Eight chromosome-wide and one genome-wide QTL associated with GT1 were detected on 4 LGs (LG14, LG16, LG18 and LG22) and the LOD scores ranging from 4.17 to 8.28 (Supplementary Fig. A1 and Table 3). The phenotypic variance explained (PVE) of the genome-wide QTL on LG18 reached to 19.5% (Table 3). A genome scan showed that the different growth traits exhibited quite similar LOD profiles. For example, two QTL associating with BL1 (qBL1-a and qBL1-b) were distributed on two single LGs, and two of the four QTL for BW1 (qBW1-c and qBW1-d) were also separated onto the same two LGs. Besides, one confidence interval on LG22 was associated with all growth traits of 6 mph. For GT2, six QTL were detected on two LGs (LG18 and LG22), which were overlapped with that of GT1 (Fig. 3, Supplementary Figs A1 and A2). In detail, two QTL (qBL2-a and qBW2-a) on LG18 were overlapped with that for GT1 (qBL1-a and qBW1-c), and four QTL (qBL2-b, qBH2-a, qHL2-a and qBW2-b) on LG22 were also overlapped with that for GT1 (qBL1-b, qBH1-b, qHL1-a and qBW1-d). For GT3, five QTL were detected on three LGs, of which just one QTL on LG14 (qBL3-b) was overlapped with that for GT1 (qBW1-a), and other four QTL (qBL3-a, qHL3-a, qHL3-b and qBW3-a) were distributed on two additional LGs (LG8 and LG19). Meanwhile, other four QTL with no overlapping with that for GT1 and GT2 presented higher PVE (14.5~18.8%) than that of GT1 and GT2 (Supplementary Fig. A3 and Table 3).

**Table 3:** Growth-related QTL for 12 morphometric traits of silver carp.

QTL	Linkage group	Nearest marker	Marker position (cM)	Confidence interval (cM)	LOD	LOD threshold		PVE (%)
						CW	GW	
qBL1-a	18	ref-16553	27.9	27.5–28.0	8.28**	5.1	8.2	19.5
qBL1-b	22	ref-46998_8	49.4	49.1–49.9	5.03*	4.9		14.0
qBH1-a	16	ref-24639	65.3	65.3–65.6	4.21*	4.1	7.9	10.3
qBH1-b	22	ref-46998_8	49.4	49.1–49.9	5.48*	4.9		13.0
qHL1-a	22	ref-46998_8	49.4	49.1–49.9	5.51*	4.5	7.2	13.0
qBW1-a	14	ref-5067_7	55.7	55.3–55.9	5.02*	4.8	7.2	12.0
qBW1-b	16	ref-29748_27	38.2	37.3–49.7	4.17*	3.7		10.2
qBW1-c	18	ref-16553	27.9	27.5–28.0	5.94*	4.6		13.9
qBW1-d	22	ref-46998_8	49.4	49.1–49.9	4.43*	4.1		11.8
qBL2-a	18	ref-16553	27.9	27.5–28.0	6.71*	5.1	8.0	14.5
qBL2-b	22	ref-46998_8	49.4	49.1–49.9	5.81*	4.9		12.6
qBH2-a	22	ref-46998_8	49.4	49.1–49.9	6.75*	4.4	7.6	14.5
qHL2-a	22	ref-46998_8	49.4	49.1–49.9	6.72*	4.2	7.0	14.5
qBW2-a	18	ref-16553	27.9	27.5–28.0	5.63*	4.6	7.5	12.3
qBW2-b	22	ref-46998_8	49.4	49.1–49.9	6.09*	4.3		13.2
qBL3-a	8	ref-68151	64.2	63.9–64.5	5.33*	4.0	5.5	18.8
qBL3-b	14	ref-30367_1	55.0	54.7–55.4	5.04*	4.1		17.9
qHL3-a	8	ref-68151	64.2	63.9–64.5	4.96*	3.6	5.2	17.6
qHL3-b	19	ref-29737	52.0	51.8–52.4	4.08*	3.8		14.7
qBW3-a	19	ref-10649	79.5	79.1–79.6	4.00*	3.8	4.8	14.5

BL, body length; BH, body height; HL, head length; BW, body weight. The digits of suffix (1, 2 and 3) indicated the growth traits measured at 6 months, 12 months and 18 months after hatch respectively. “*”, chromosome-wide (CW) significance; “**”, genome-wide (GW) significance; PVE, phenotypic variance explained.

Four potential candidate genes were identified from growth-related QTL intervals after BLAST searching against the genome of zebrafish (Supplementary Table A3), including *hepcidin*, phosphodiesterase 11a (*PDE11a*), fas-activated serine/threonine kinase (*FASTK*) and solute carrier family 35 member F3a (*SLC35f3a*). Three of the four markers are directly matched within the sequences of the correspondent candidate genes in zebrafish, and *hepcidin* gene was identified in the vicinity of the marker ref-68349_2. As a known multi-functional gene, the potential candidate gene *hepcidin* was selected for further mRNA expression analysis and association study in this study.

### Association analysis of *hepcidin* gene

**Figure 4:**
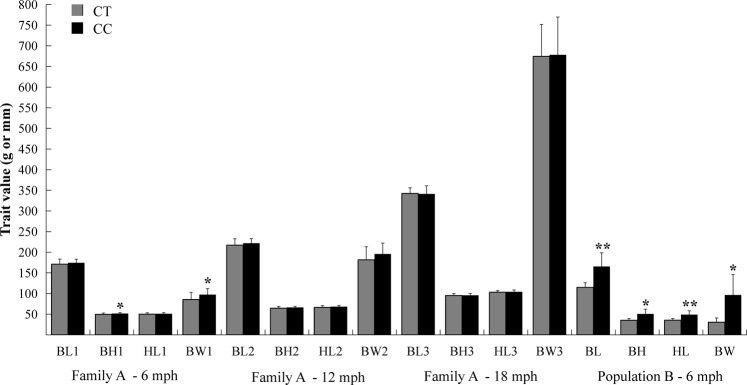
Differences of observed growth traits between genotypes (CC/CT) of hepcidin-g.752 C > T in two silver carp populations. Significant differences at *P* < 0.05 and *P* < 0.01 are labeled with “*” and “**”, respectively.

The nucleotide sequences of *hepcidin* gene in silver carp is available from the GenBank database (accession no. KF270356). Primers designed for polymorphism identification and expression analysis of *hepcidin* were listed in Supplementary Table A4. One polymorphic locus (hepcidin-g.752 C > T) identified from exon2 of *hepcidin* gene showed significant growth associations in two silver carp populations (Fig. 4, Supplementary Table A5). In detail, hepcidin-g.752 C > T significantly associated with BH1 and BW1 (*P* < 0.05) in family A, and the individuals with genotype CC had a higher BW (13.8%) than those with genotype CT. No significant association was detected between genotypes of this SNP and the GT2 and GT3. Only four individuals were identified as genotype CT in population B and the segregation ratio was significantly deviated from the HWE after Bonferroni corrections. However, the locus hepcidin-g.752 C > T still significantly associated (*P* < 0.05 for BH and BW, *P* < 0.01 for BL and HL) with all four growth traits of population B, and the BW of the individuals with genotype CC were 217.3% higher than that with genotype CT.

**Figure 5:**
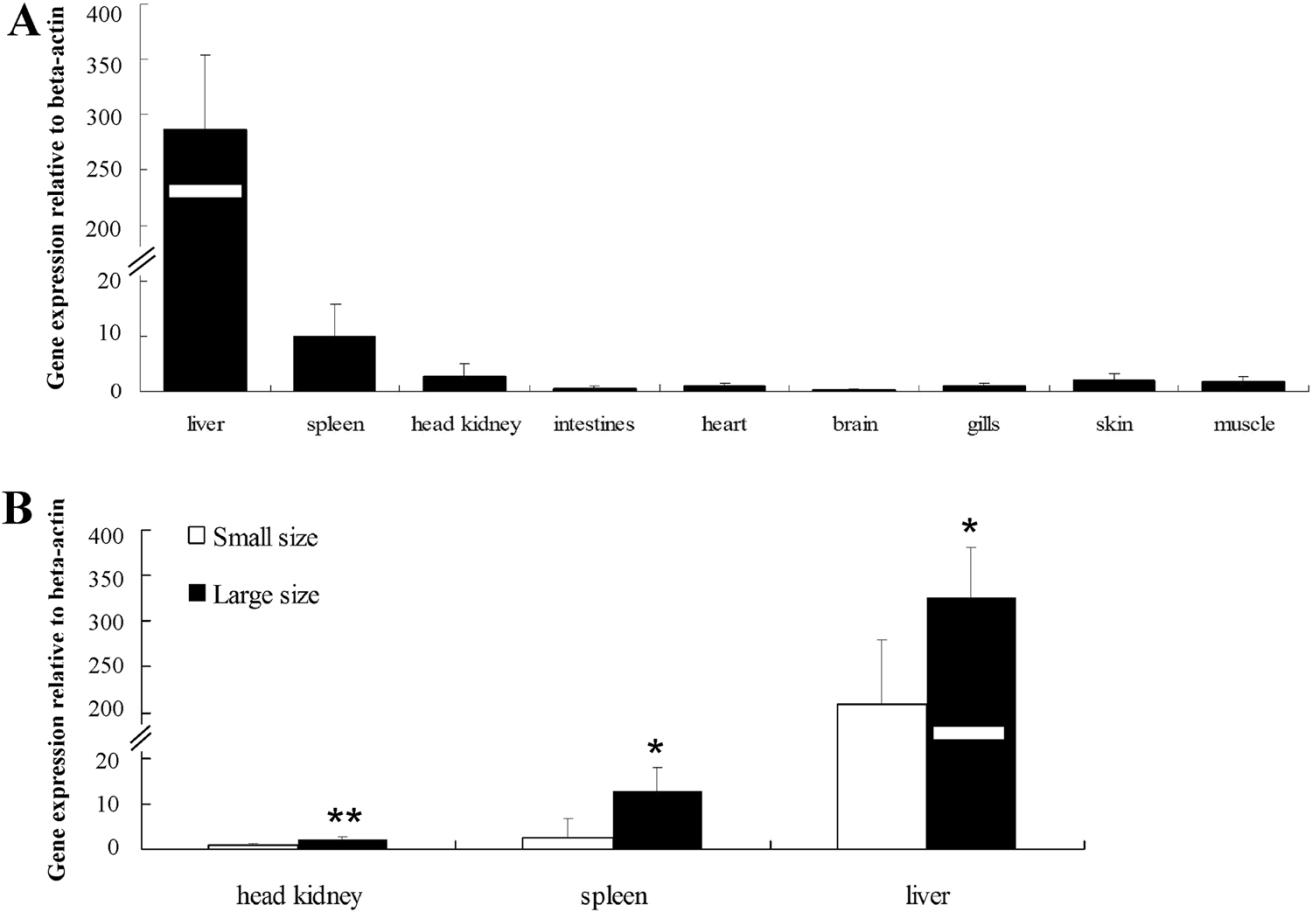
Spatial expression analysis of *hepcidin* gene in silver carp (**A**) and comparative expression analysis between small size and large size groups in head kidney, spleen and liver (**B**). Significant differences at *P* < 0.05 and *P* < 0.01 are labeled with “*” and “**”, respectively.

A ubiquitous expression pattern of the *hepcidin* gene was observed in all analyzed tissues, with the highest level in the liver, followed by the spleen and the head kidney (Fig. 5A). Then the liver, spleen and head kidney were selected for comparative expression analysis between small-size and large-size groups from the population B. Significantly different expression levels were detected between these two groups of extreme weights in three tissues analyzed (*P* < 0.05 for the spleen and the liver, *P* < 0.01 for the head kidney) (Fig. 5B).

## Discussion

### 2b-RAD as an effective way of SNP development

To date, RAD sequencing approaches, which can balance the marker quantity, sequencing cost, sequencing coverage and genotyping accuracy, have become more and more popular in the construction of high-density linkage maps in non-model species without the whole reference genome^34^. Here, we used the 2b-RAD approach to develop thousands of SNPs in silver carp and constructed a high-density linkage map containing 3,134 markers. Compared with other RAD-based technology, 2b-RAD provided a streamlined library construction method and an adjustable marker density by utilizing selective adaptors^8^. This approach has been successfully applied for linkage map construction of several non-model species^5,12–15^, which indicated that 2b-RAD is an appropriate and powerful method for genotyping by sequencing (GBS) in silver carp and/or other fish.

Different species and marker systems may lead to various levels of segregation distortion^35,36^. With the same protocol (2b-RAD) for SNP development, the proportion of segregation-distorted markers was 50.5% in this study, which was similar to that in pearl oyster^5^ but smaller than that in bivalve mollusk^12^. In addition, the levels of segregation distortion of 2b-RAD markers were much higher than that of SSRs and AFLP markers in previous studies, such as the earlier studies for pearl oyster and silver carp^27,37^. Segregating distortion may result from special selection, such as double recessive of some lethal genes and zygotic selection^38^, and may be also from sampling and genotyping errors^39,40^. The application of techniques like 2b-RAD, which generated quite small read length in aquatic animals, could also lead to a huge percentage of segregating distortions.

### High-density genetic map for silver carp and comparative genomics

A linkage map with a small marker interval (< 2 cM), high map coverage and even marker distribution was commonly known as high-density map, which usually possess thousands of polymorphic markers^41^. A few years ago, we reported a second-generation genetic map for silver carp using a strategy of interspecific hybridization between silver carp and bighead carp, and 703 microsatellites were grouped into 24 LGs with an average resolution of 2.2 cM^27^. This medium-density map was used to comparative genomics studies and genome assembly, but it is not sufficient for QTL fine mapping for growth related traits in this aquaculture species. Therefore, in present study, a sex-averaged genetic map for silver carp was constructed using a crossing strategy of a sire and a dam and their 198 progenies. The LGs of the current map were numbered according to our previous map^27^, of which the number of LGs is equal to the haploid chromosome number of silver carp (*n* = 24)^31^. The number of markers (3,134 SNPs) of current high-density linkage map is almost 4.5 times higher than previous genetic map constructed by SSRs, and the average distance between markers dropped significantly from 2.2 cM to 0.86 cM (Table 2). The total length of the present genetic map (2,721.07 cM) is 74% longer than previous microsatellite map (1,561.1 cM)^27^, which may because more markers chained in our study or other possible reasons. The improved genetic map provides a base for QTL fine mapping of economic traits in silver carp.

Comparative mapping with model species provide the possibility to evaluate the accuracy of the genetic maps of non-model species and to locate genes of interest. Zebrafish and commonly known carps in aquaculture (e.g. common carp, silver carp, bighead carp, grass carp, and so on) are all members of the Cyprinidae, the largest family among all fish species. In this study, a high level of conserved genomic synteny was observed between silver carp and zebrafish (80%) with a clear 1:1 relationship between LGs and chromosomes except for LG19 of silver carp (homologous to Chr10 and Chr22 of zebrafish; Fig. 2). The same synteny results were also detected for bighead carp^14,42^ and grass carp^43^. It is believed that Chr10 and Chr22 of zebrafish were fused into one chromosome in grass carp^43^. According to our evidence of comparative genomics, this is also the case in silver carp. The high genomic synteny with zebrafish, whose genome and gene annotations are available online, made the identification of potential candidate genes convenient in silver carp.

### QTL for growth and comparative studies

Growth traits are important quantitative trait and generally influenced by multiple genes and biological pathways, and QTL mapping is an efficient approach to uncover potential genomic regions underlying these traits. To date, QTL mapping for growth-related traits have been performed in many aquatic species^13,19–24^, and only one QTL study was reported in the hybrids of bighead carp and silver carp^44^. In this study, a total of 20 QTL for growth traits measured at three growth stages were identified on 6 LGs (Fig. 3, Table 3). The comparative analysis of QTL between silver carp and bighead carp^14,18^ showed that two of the 6 LGs (LG18 and LG19) both identified with QTL in our study and in bighead carp (homologous with LG11 and LG9)^14,18^. The common QTL regions are valuable resource for the exploration of trans-species conservation of growth QTL regions among both carp species. Other specific QTL intervals may be responsible to different species, mapping populations and/or growth stages. Similar phenomenon was also reported in other fish species^45–47^. These QTL detected in the given silver carp family need further validation and association analyses in other genetic families or backgrounds to insure their availability in MAS breeding of silver carp. In addition, similar distribution patterns of QTL were detected not only on multiple LGs but also between different growth traits and/or growth periods. In our results, the markers “ref-16553” on LG18 and “ref-46998_8” on LG22 were associated with four and eight growth traits, respectively. This co-localization QTL may be a reflection of the high correlation coefficients among the different growth traits (Table 1), and these two markers would be valuable genetic resources for future determining and physical cloning of candidate growth genes and MAS program in silver carp. Similar distribution patterns for growth-related QTL had also been detected in different species, such as scallop^12^, bighead carp^14,18^, Asian seabass^47^, Japanese flounder^20^.

Comparative analysis of QTL detected in different growth stages would be conductive to understanding the regulation mechanism underlying the traits of interest. In consideration of the mature time of silver carp (3–4 years)^26^, 6 mph and 12 mph could be judged as the early muscle growth stage, 18 mph and 24 mph as the middle and later stage of muscle growth, and after that as the gonad development stage. In this study, all six QTL for GT2 were overlapped with QTL for GT1, while only one of the five QTL for GT3 were co-localized on the same linkage group with QTL for GT1. Therefore, we speculate that silver carp at the middle and later muscle growth stage (18 mph) may start a different development mode, which might be influenced by different set of QTL or genomic regions compared with early muscle growth stage (6 and 12 mph). Similar phenomenon was also observed in rainbow trout^46^, Atlantic salmon^45^ and Asian seabass^47^, and the period specific QTL suggested the existence of dynamic expression for the traits investigated. Atchley & Zhu^48^ had pointed out that individual growth at different stages were controlled by two different patterns (cell division and cell size increasing respectively), which made the existence of these specific QTL reasonable. These growth-related QTL detected in different growth stages are valuable genomic resources for potential candidate gene discovery and potential MAS program in silver carp. In addition, we found that the average PVE of GT3 was higher than that of GT1 and GT2. Generally speaking, the value of the phenotype variations was closely related to the trait variance within family^49^, and various levels of PVE had been detected in previous studies of fish^19–22,44,47^.

### Potential candidate genes for growth

Comparative mapping with model species is an effective way to identify potential candidate genes within QTL intervals, and several potential candidate genes for growth traits had been identified in aquatic animals, such as *IFABP-a* and *ACOX1* in Asian seabass^19,47^. *Hepcidin* gene, as one of the antibacterial peptides, plays an important role in innate immunity and iron regulation in animals^50–52^, and its potential positive effects on growth have also been proved by adding supplemental antibacterial peptide into daily diets, in which the individuals from experiment group grown faster than control individuals^53,54^. As a hydrolase super family, phosphodiesterase (*PDE*) mainly functioned on the hydrolysis of the second messenger cGMP and cAMP, and the latter plays an important role in regulating growth, proliferation, phenotypic transformation and other pathways^55,56^. *FASTK*, a constitutively phosphorylated Ser/Thr kinase, plays an important role in the regulation of T-cell apoptosis^57,58^, and this potential candidate gene needs to further functional studies considering its relationship with eight growth traits in our present study. The members of solute-carrier family 35 (*SLC35*) characterized encode nucleotide sugar transporters localizing at the golgi apparatus and the endoplasmic reticulum^59,60^.

### *Hepcidin* as a potential candidate gene for growth in silver carp

In our study, one polymorphic locus of the *hepcidin* gene (hepcidin-g.752 C > T) showed significant associations with growth traits in two tested populations of silver carp, which confirmed its significant effect on growth in previous studies^53,54^. Interestingly, hepcidin-g.752 C > T significantly associated with GT1 of the family A, but not with GT2 and GT3 (Fig. 4). This specific association pattern was consistent with the results of QTL mapping on LG16, further demonstrated that some genes controlling growth traits just functioned at specific stages and presented a dynamic expression pattern of different development stages^48^. Furthermore, the mRNA expression levels of *hepcidin* in the large-size individuals were significantly higher than those in the small-size individuals in all three tested tissues (Fig. 5B). These results strongly indicate that *hepcidin* may play an important role in the modulation of growth and development in silver carp.

In summary, based on a high-density genetic linkage map, one major and nineteen suggestive QTL were first identified at three growth stages of silver carp. The QTL for early muscle growth stage (6 and 12 mph) were generally overlapped in several LGs, but the QTL for the middle and later muscle growth stage (18 mph) located at other positions of those LGs, indicating a different genetic modulation during early and late muscle growth stages in silver carp. As one of the potential candidate genes from the QTL intervals, *hepcidin* was significantly associated with growth and body shape traits by the evidence from both mRNA expression between large and small size fish and genic SNP associations with phenotypes. This study provides a basis for elucidating the genetic mechanism for growth-related traits in silver carp, which would help to improve production performance via future MAS programs.

## Materials and Methods

### Ethics statement

All experimental procedures involving the fish in our study were approved by the Committee for Animal Experiments of the Institute of Hydrobiology and carried out in accordance with the Laboratory Animal Management Principles of China. And the informed consent was obtained from all participants.

### Mapping family

The broodstock fish of silver carp used for genetic mapping were collected from wild populations of the Yangtze River. A full-sib family was established in May 2014 by mating a pair of sire and dam (with the weight of 11.2 kg and 9.3 kg, respectively) which showed a larger genetic distance based on microsatellite markers. One thousand progenies were randomly selected and raised in a 5000 m^2^ muddy pond at the Seed Farm of Four Major Chinese Carps of Laohe Oxbow (Shishou, China). PIT tags were applied for individual fish throughout all growth stages in this study to ensure that we get the phenotypes of growth traits precisely for each fish. Growth traits, including BL, BH, HL and BW, were measured for all progenies at 6, 12 and 18 mph, respectively. Finally, 198 progenies were randomly selected and used for map construction and QTL detection. Pearson correlation coefficients, which is the ratio of the covariance of the two random variables to the product of their standard deviation, were calculated for all 12 traits using the SPSS 19.0 software (IBM, USA). Fin clips of the parents and progenies were sampled and ethanol-preserved, and genomic DNA was extracted following a traditional phenol-chloroform protocol^61^. The quality of DNA was evaluated by visualization on a 1% agarose gel electrophoresis and by spectrophotometric analysis using a NanoDrop 2000 UV-Vis Spectrophotometer (Thermo Scientific, USA).

### 2b-RAD sequencing

Previous studies reported that the genome size of silver carp was about 1 Gb^62,63^. Before the experiment, a digital-enzyme-cut analysis was conducted on the draft genome assembly of silver carp (unpublished results) to confirm the sufficient amount of restriction sites. 2b-RAD libraries were prepared for two parents and 198 progenies following the protocol^8^ with some modifications. The detailed information had been described in our previous studies by Fu *et al*.^14^ and Liu *et al*.^15^. The raw read data were archived at the NCBI Sequence Read Archive (SRA) database (accession no. PRJNA328416).

### Preprocessing and genotyping of sequence data

Raw reads were processed for initial trimming using a homemade Perl script according to the method described in our previous studies^14,15^. The remaining trimmed reads with a length of 32 bp were used for subsequent analysis. *De novo* genotyping was performed using the RADtyping program v1.3.0^34^. This software used stringent criteria in filtering candidate markers, and only those loci with at least 4 reads supporting were kept for the following analysis^14,15^. A co-dominant marker was determined when a tag sequence was detected in reads of both parents with one SNP between them, and a dominant marker was determined from a parent-specific tag which only appeared in reads of one parent and absent in the other parent, and the dominant markers were generated by the variations of a single site within the sequence of the recognition site of the restriction enzyme (*BcgI*).

### Linkage map construction and comparative genome analysis

Segregating markers with missing data exceeding 20% of the progenies were removed. A Chi-square test was performed to assess the goodness-of-fit to expected segregation ratio for each locus at the confidence level of 0.05. Subsequently, markers showing significant departure (*P* < 0.05) from the expected segregation ratios were excluded from further linkage analyses. The sex-averaged genetic linkage map was constructed using software JoinMap 4.0^64^ under the CP (cross pollinator) algorithm. The logarithm of odds (LOD) threshold of 9.0 was used to assign markers into linkage groups (LGs). Graphical visualization of the LGs were achieved by MapChart 2.2 software^65^.

The expected map length (*G*_*e*_) of silver carp was calculated by the methods proposed by Chakravarti *et al*.^32^ and Fishman *et al*.^33^. The estimate expected map length *G*_*e1*_ and *G*_*e2*_ were obtained and averaged as the final expected map length (*G*_*e*_). The observed map coverage (*C*_*oa*_) was calculated by the formula of *G*_*of*_/*G*_*e*,_ where *G*_*of*_ represents the total observed length of the sex-averaged map^66^. Comparative mapping was performed between our genetic map and the reference genome of zebrafish (*Danio rerio)* based on the method described in our previous study^66^. The genomic synteny between our linkage map and the reference genome of zebrafish (http://www.ncbi.nlm.nih.gov/genome/50) was visualized using the software Circos^67^ and Oxford grids.

### QTL mapping and potential candidate genes identification

QTL analysis was performed for all 12 morphometric traits of silver carp using software MapQTL 6.0^68^. As the method described previously^66^, the genome-wide and chromosome-wide LOD significance thresholds for each trait were calculated by a permutation test of α < 0.05 and *n* = 1,000 within the software MapQTL 6.0. Then the LOD scores were calculated through the multiple QTL model (MQM mapping). The regions with LOD scores greater than the LOD threshold were the confidence intervals. The phenotypic variance explained (PVE) effected by a QTL was calculated based on the population variance found within the progenies^68^. The extended sequences of QTL-associated markers were used for BLAST searching against the genomes of zebrafish for the identification of potential candidate genes.

### mRNA expression and growth association analysis of potential candidate gene

In order to investigate the associations between growth traits and genotypes of the candidate genes, forty progenies with extreme body weight phenotypes (defined as family A, including 20 small size individuals and 20 large size individuals, with an average body weight of 77.2 g and 106.1 g, respectively), were selected from the mapping family of silver carp described above, and they were used for SNP genotyping. The SNP was genotyped by direct sequencing of the amplification products. Another set of 40 extreme body weight individuals (defined as population B, including 20 small-size individuals and 20 large-size individuals, with an average body weight of 37.5 g and 137.5 g, respectively), were selected from a population generated by crossing 13 males and 15 females in the Zhangdu Lake Fish Farm (Wuhan, China) in May 2015, and they were used for the verification of potential growth-associated loci detected from family A. A one-way analysis of variance (ANOVA) function in the SPSS19.0 software was applied to the phenotypic data, and a critical value of *P* < 0.05 was set as the criterion for statistical significance.

Five individuals randomly selected from population B were sacrificed and nine tissues (including liver, spleen, head kidney, intestine, heart, brain, gill, skin and muscle) were sampled for spatial expression analysis. In addition, five small-size individuals and five large-size individuals (with a mean body weight of 66.5 g and 304.5 g, respectively) were sampled for comparative expression analysis. Total RNA were extracted from tissues using TRIZOL method (Thermo Fisher Scientific). The quality of isolated RNA was also evaluated by 1.5% agarose gel electrophoresis and NanoDrop 2000 spectrophotometer. Reverse transcription reactions and qRT-PCR amplifications were carried out following the specifications of the Reverse Transcriptase M-MLV kit (TaKaRa, Japan) and the SYBR Green PCR kit (QIAGEN, Germany). The expression of silver carp *β-actin* gene (Hy-β-actin-qPCR; Supplementary Table A4) was served as the internal control. Reactions were repeated three times for each sample. The relative expression levels were normalized to the quantification of *β-actin* using the 2^−ΔΔCT^ method^69^.

## Supplementary information

Supplementary information
