# Supplementary material for: Construction of a high-density genetic linkage map and mapping of quantitative trait loci for growth-related traits in silver carp (Hypophthalmichthys molitrix)
Source: Sci Rep. 2019 Nov 25;9:17506. doi: 10.1038/s41598-019-53469-8 (PMC6877629; doi:10.1038/s41598-019-53469-8)
Supplement: Supplementary file 1 — Supplementary information [file 41598_2019_53469_MOESM1_ESM.pdf]

Construction of a high-density genetic linkage map and mapping of quantitative trait loci for growth-related traits in silver carp (*Hypophthalmichthys molitrix*)

Xinhua Wang[1,2], Haiyang Liu[2], Meixia Pang[2], Beide Fu[2], Xiaomu Yu[2], Shunping He[3] & Jingou Tong[2]

[1]College of Animal Science and Technology, Henan University of Animal Husbandry and Economy, Zhengzhou, 450046, China.

[2]State Key Laboratory of Freshwater Ecology and Biotechnology, Institute of Hydrobiology, Chinese Academy of Sciences, Innovation Academy of Seed Design, Chinese Academy of Sciences, Wuhan, 430072, China.

[3]Key Laboratory of Aquatic Biodiversity and Conservation of the CAS, Institute of Hydrobiology, the Chinese Academy of Sciences, Wuhan, 430072, China.

Correspondence and requests for materials should be addressed to Tong JG (email: jgtong@ihb.ac.cn).

Supplementary information legends:

Supplementary Fig. A1 A genome scan of LOD profiles for body length (BL), body height (BH), head length (HL) and body weight (BW) measured at 6 months post hatch for silver carp. The red solid line indicated the genome-wide significance thresholds.

Supplementary Fig. A2 A genome scan of LOD profiles for body length (BL), body height (BH), head length (HL) and body weight (BW) measured at 12 months post hatch for silver carp.

Supplementary Fig. A3 A genome scan of LOD profiles for body length (BL), body height (BH), head length (HL) and body weight (BW) measured at 18 months post hatch for silver carp.

Supplementary Table A1 Phenotypic parameters of silver carp at different growth stages.

Supplementary Table A2 Detail information of markers located in the high-resolution genetic linkage map of silver carp and the genome of zebrafish.

Supplementary Table A3 The markers within confidence intervals of QTLs for growth traits in silver carp and the potential candidate genes detected from the genome of zebrafish.

Supplementary Table A4 Primers for polymorphism identification and expression analysis of *hepcidin* in silver carp.

Supplementary Table A5 Association analysis between genotypes of hepcidin-g.752C>T and growth traits in two silver carp populations.

Supplementary Fig. A1 A genome scan of LOD profiles for body length (BL), body height (BH), head length (HL) and body weight (BW) measured at 6 months post hatch for silver carp. The red solid line indicated the genome-wide significance thresholds.

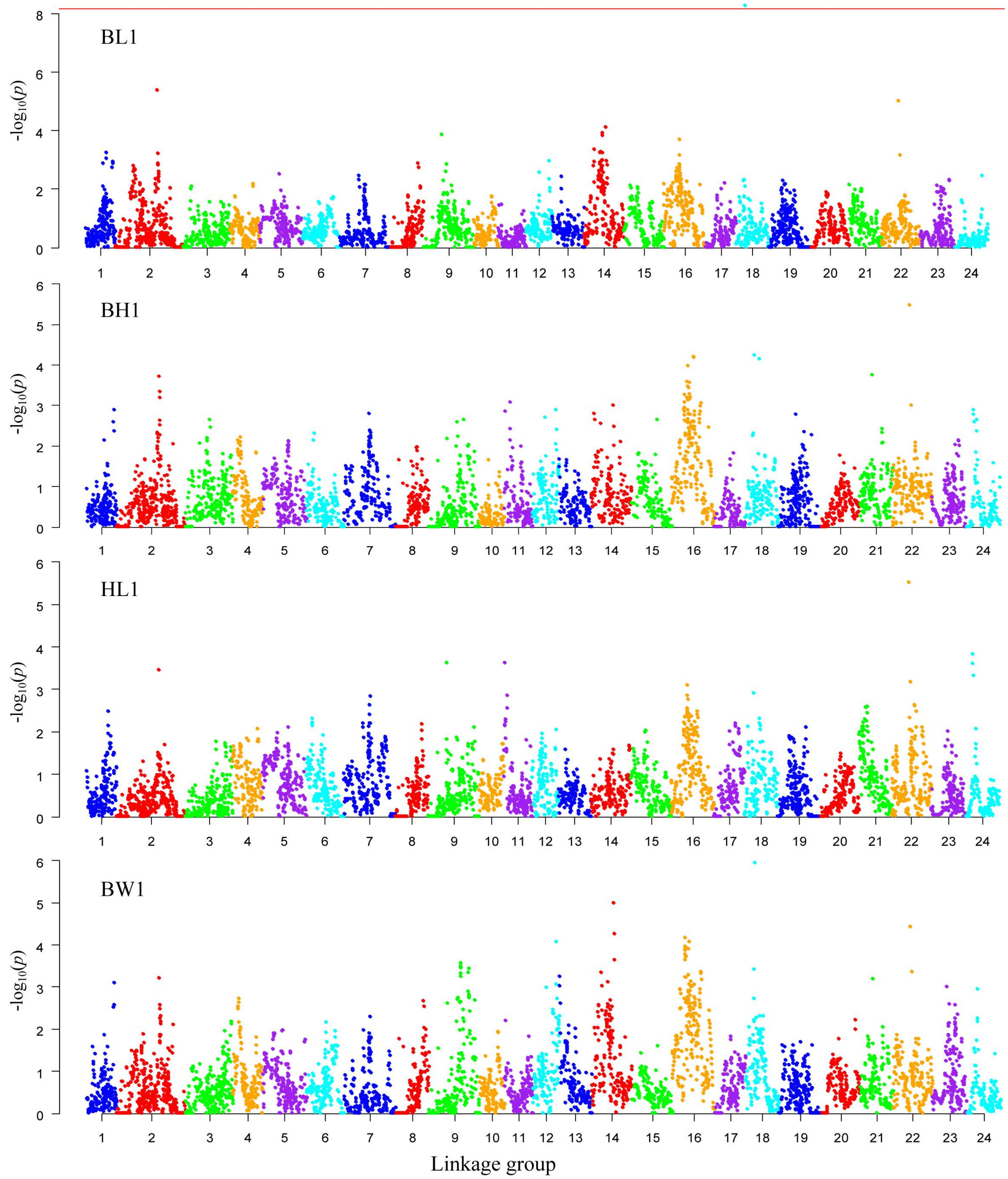

Supplementary Fig. A2 A genome scan of LOD profiles for body length (BL), body height (BH), head length (HL) and body weight (BW) measured at 12 months post hatch for silver carp.

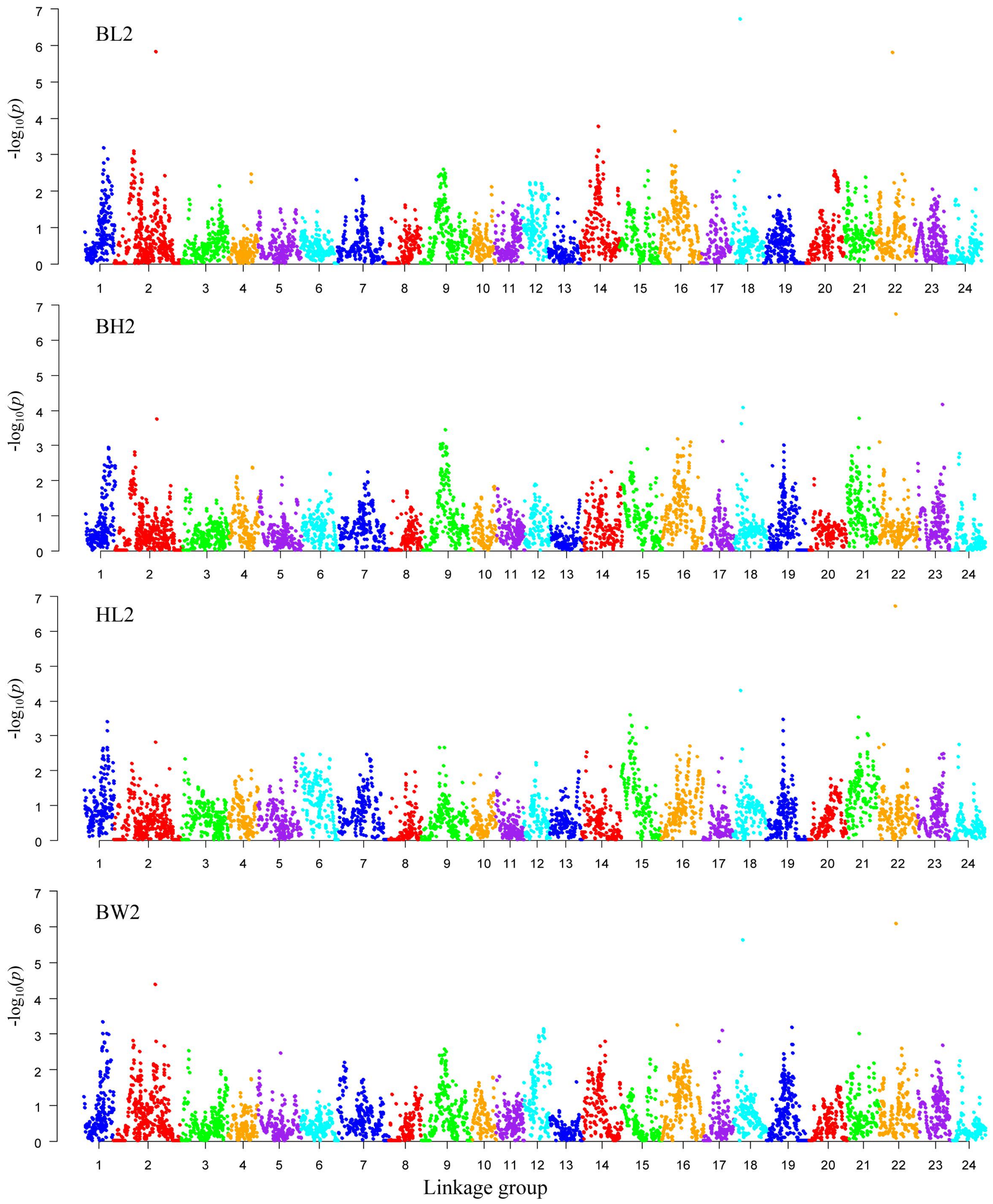

Supplementary Fig. A3 A genome scan of LOD profiles for body length (BL), body height (BH), head length (HL) and body weight (BW) measured at 18 months post hatch for silver carp.

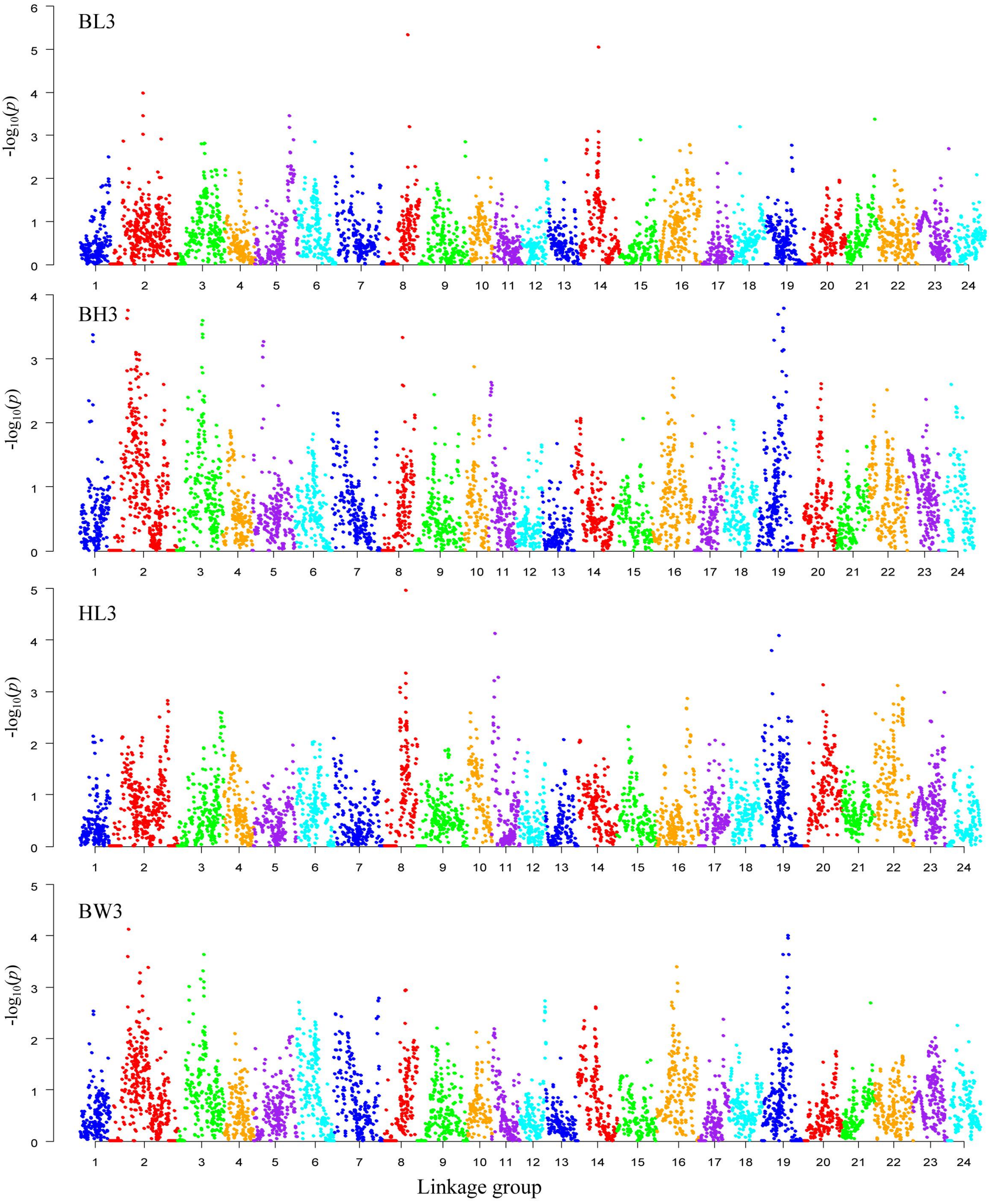

Supplementary Table A1 Phenotypic parameters of silver carp at different growth stages.

Progeny	PIT tags No.	Growth traits at 6 months post hatch				Growth traits at 12 months post hatch				Growth traits at 18 months post hatch			
		BL1, cm	BH1, cm	HL1, cm	BW1, g	BL2, cm	BH2, cm	HL2, cm	BW2, g	BL3, cm	BH3, cm	HL3, cm	BW3, g
1	618000619001962	17.6	5.3	5.3	105	22.4	6.6	7	190.4	--	--	--	--
2	618000619001984	18.3	5.4	5.3	115	23.3	7	7.2	221	--	--	--	--
3	618000619001951	17.5	5	5.2	104	22.3	6.3	6.3	182.5	--	--	--	--
4	618000619001902	18.1	5.2	5.3	100	23.1	6.7	6.8	203.6	--	--	--	--
5	618000619001996	17.8	5.1	5.2	98	22.1	6.5	6.5	193.3	35.3	9.9	10.3	732.7
6	618000619001943	18.4	5.4	5.3	109	23.6	6.7	7	215.7	36	9.9	11.3	760.3
7	618000619001987	18.2	5.3	5	107	22.7	6.6	6.7	211.5	35.1	9.7	9.9	744
8	618000619001944	18.3	5.4	5.4	111	23.5	6.9	6.9	227.5	35	9.9	10.2	736.1
9	618000619001979	17.8	5.4	5.2	109	22.8	7	7.2	231.7	--	--	--	--
10	618000619001233	16.3	4.8	4.7	73	21.4	6.3	6.5	175.2	--	--	--	--
11	618000619001926	16.7	5	5.1	84	21.3	6.4	6.7	179	--	--	--	--
12	618000619001916	17.3	5	5.1	81	21.7	6.5	6.7	183.6	33.4	9.3	10.3	625
13	618000619001977	18.1	5	5.2	99	22.7	6.7	7	204.1	34.3	9.2	10.5	663.4
14	618000619001955	17.9	5.1	5.3	94	22.8	6.6	7.1	194.2	35.3	9.7	10.2	682.1
15	618000619001995	18.1	5.3	5.2	101	23.3	6.8	7.1	212.1	34.1	9.4	10.2	648.9
16	618000619001609	17.2	5.2	5	95	21.8	6.6	6.9	189	--	--	--	--
17	618000619001498	17.5	5	4.9	91	21.3	6.3	6.3	170.9	33.9	9.3	10.1	660.5
18	618000619001494	17.7	5	5.2	92	23.1	6.8	7	216.8	--	--	--	--
19	618000619001696	17.2	4.9	4.8	84	21.6	6.4	6.4	173.7	33	8.8	10	577
20	618000619001658	17.3	5.1	4.9	80	21.7	6.4	6.6	180.2	--	--	--	--
21	618000619001495	16.7	5.1	5.2	93	21.9	6.4	6.5	199.1	34.3	9.7	10.3	685.8
22	618000619001497	17.8	5.3	5.2	93	23.1	6.8	7	204.4	--	--	--	--
23	618000619001453	17.4	5	5.1	88	21.8	6.4	6.5	171.8	--	--	--	--
24	618000619001607	17.9	5	5.2	97	22.8	6.9	6.9	207.5	34.9	9.5	10.4	700.1
25	618000619001481	18	5.1	5.1	99	22.7	6.6	6.8	193.5	--	--	--	--
26	618000619001496	15.3	4.8	4.7	67	19.4	6	6.2	139.4	29.5	8.6	9.6	467.4

27	618000619001470	18.1	5.3	5.4	104	23.4	6.8	7.1	218.7	35.4	9.6	10.3	714.5
28	618000619001458	16.9	4.9	5	85	21.3	6.4	6.6	168.8	--	--	--	--
29	618000619001468	17.3	5	5.1	92	22.3	6.7	6.7	200.1	34.5	10	10.8	722.7
30	618000619001591	17.7	5	5.2	96	23.3	7	7.4	223.7	--	--	--	--
31	618000619001602	17.8	5.1	5.2	97	22.3	6.5	6.5	191.6	32.8	8.9	10.2	594.7
32	618000619001624	17.5	4.9	4.9	90	22.3	6.5	6.7	180.9	33.8	9.1	9.7	611.3
33	618000619001667	17.7	5	5.1	95	22.8	6.6	6.8	217.9	--	--	--	--
34	618000619001518	17.2	5	5.1	90	21.4	6.3	6.5	175.6	34.8	9.8	11.2	679.3
35	618000619001519	17.8	5.1	5.3	95	22.1	6.5	6.8	174.9	34	9.6	10.3	654.2
36	618000619001577	17.6	5.1	5	94	22.7	6.7	6.9	184.6	34.3	9.4	9.9	653
37	618000619001585	18	5.2	5.2	98	21.7	6.5	6.7	179.2	35	9.7	10.3	697.1
38	618000619001588	17.6	5.1	5	93	22.4	6.6	6.6	194.9	34.1	9.7	10.3	660.4
39	618000619001570	17.6	4.9	4.8	89	22.5	6.4	6.7	191.8	--	--	--	--
40	618000619001545	16.2	4.7	4.6	76	21.3	6.4	6.7	172.3	--	--	--	--
41	618000619001640	17.3	4.8	5	87	22.2	6.5	6.7	189.9	--	--	--	--
42	618000619001368	17.3	4.9	4.9	88	22	6.4	6.7	182.1	34.5	9.9	10.3	707.8
43	618000619001655	18	5.2	5.2	105	23.1	6.8	7	214.9	33.6	9.3	10.2	654.1
44	618000619001482	17.5	4.9	5.1	101	22.3	6.6	6.6	187.9	34.2	9.4	10.1	661.5
45	618000619001522	17.3	5	5.1	94	21.7	6.4	6.8	184.2	34.2	9.7	10.1	692.5
46	618000619001618	17.7	5.1	5.2	99	22.6	6.7	6.9	200.2	34	9.5	10.6	656.4
47	618000619001537	17.9	5.1	5.2	96	23.1	6.8	6.9	211.4	35.8	9.9	10.7	782.3
48	618000619001598	17.2	4.8	5	85	21.7	6.3	6.7	173.6	34.5	9.6	10.7	663.3
49	618000619001534	18.4	5.2	5.3	103	23.2	6.8	6.9	203.5	--	--	--	--
50	618000619001574	17.5	5	5.4	92	22.5	6.3	6.7	186.5	--	--	--	--
51	618000619001685	18.3	5.2	5.4	104	23.2	6.7	7.2	206.4	36.2	10	10.9	761.7
52	618000619001590	17.4	5	5.1	82	21.2	6.4	6.4	166.6	32.1	8.9	10	561.9
53	618000619001692	17.3	5	5.2	87	22.1	6.6	6.7	192.2	34.4	9.9	10.1	722.7
54	618000619001507	16.7	4.9	5	82	21.3	6.4	6.6	164.6	--	--	--	--
55	618000619001663	17.9	5.1	5.2	92	22.9	6.8	6.9	199.6	--	--	--	--

56	618000619001516	17.2	5	5.1	83	22	6.3	6.3	173.9	--	--	--	--
57	618000619001565	18.2	5.3	5.4	102	23.2	6.8	6.8	220.1	34.5	9.6	10.1	706.2
58	618000619001517	17.2	5	5	87	22.1	6.5	6.6	177.1	--	--	--	--
59	618000619001596	17.3	4.9	5.1	84	21.7	6.4	6.6	186.1	33.7	9.1	10.2	638
60	618000619001463	17.5	5	5.2	93	22.4	6.7	7	195.4	34.4	9.7	10.1	704.5
61	618000619001600	17.1	5	5.1	83	22.4	6.8	7.1	202.5	33.7	9.8	10.2	662.8
62	618000619001657	17.3	4.9	5.2	92	22.3	6.6	6.9	193.8	33.5	9.3	10.5	651.5
63	618000619001462	18.1	5.3	5.2	103	23.4	7	7.4	229.1	--	--	--	--
64	618000619001459	17.9	5.3	5.3	98	23	6.8	7.2	208.5	32.4	8.9	9.8	572.3
65	618000619001668	18.1	5.2	5.3	99	23.4	6.8	6.9	226	36.3	9.8	10.8	779
66	618000619001611	17.2	4.9	4.9	86	21.8	6.4	6.6	175.3	--	--	--	--
67	618000619001505	17.3	5	5.1	86	22.1	6.7	6.6	179.9	33.7	9.7	10.3	675.9
68	618000619001572	17.4	5.1	5.2	89	21.6	6.5	6.7	167.7	33.7	9.6	10.1	635.4
69	618000619001589	17.3	5	5.1	90	22.3	6.7	6.8	195.2	33.7	9.3	10.3	634.1
70	618000619001671	17.9	5.1	5.4	91	22.9	6.7	6.7	210.7	34.8	9.7	11.1	714.1
71	618000619001622	17.5	5.1	5.2	91	22.7	6.8	6.8	198.8	--	--	--	--
72	618000619001464	17.7	5	5.1	89	22.9	6.5	6.9	194.1	34.3	9.2	10	669
73	618000619001443	17.7	5	5	94	22.7	6.6	6.9	212.5	34.7	9.5	10.1	694
74	618000619001434	16.3	4.7	4.9	74	20.9	6.1	6.4	167.9	--	--	--	--
75	618000619001444	17.6	5.1	5.2	92	22.8	6.9	7	215	--	--	--	--
76	618000619001421	17.7	5.1	5	94	23.2	6.8	7	214.2	--	--	--	--
77	618000619001429	17.6	5.1	5	94	22.3	6.5	6.6	191.9	--	--	--	--
78	618000619001447	17.6	5.1	5.3	85	22.4	6.7	7.1	190.8	33.6	9.5	10.7	651.8
79	618000619001760	12.7	3.8	3.9	32	16.1	4.9	5.3	70.5	--	--	--	--
80	618000619001780	17.8	5.1	5.2	97	23.1	6.8	6.9	209.7	--	--	--	--
81	618000619001436	17.4	5.1	5	93	22	6.6	6.7	192.9	34.5	9.8	10.2	724
82	618000619001412	17.8	5.2	5.1	98	23.2	7	7.4	211	--	--	--	--
83	618000619001753	18.1	5.1	5.3	92	22.3	6.5	6.6	197.6	--	--	--	--
84	618000619001433	17.6	5.2	5.1	91	22.3	6.9	6.9	211.5	34.3	10	10.5	750

85	618000619001415	17.5	4.9	4.8	92	21.4	6.2	6.5	166.2	34.4	9.2	10.2	669.3
86	618000619001437	17.9	5.1	5.2	99	22.7	6.8	6.8	208	35.8	9.8	11.4	736.4
87	618000619001411	17.2	4.8	4.9	84	22.3	6.6	6.8	188	--	--	--	--
88	618000619001449	17.7	5	5.1	93	22.8	6.8	6.9	206	--	--	--	--
89	618000619001414	17.1	4.9	5.1	81	21.7	6.6	6.7	183.9	33.3	9.6	10.2	653.1
90	618000619001423	17.5	5.1	4.9	93	22.5	6.9	6.9	201.1	35	9.8	10.2	712.6
91	618000619001446	17	4.9	4.8	74	21.7	6.5	7	179.5	--	--	--	--
92	618000619001416	17.5	5	5.1	94	22.3	6.5	6.8	185.3	--	--	--	--
93	618000619001417	18.2	5.2	5.1	97	23.4	6.8	6.8	223.8	36.5	10.2	10.8	802
94	618000619001774	17.7	5.1	5.2	94	22.8	6.7	7	215.6	34.8	9.7	10.3	724.7
95	618000619001770	17.8	5	5.1	85	22	6.5	6.7	189.4	33.8	9.7	10.5	673
96	618000619001772	17.8	5.1	5	90	23.3	6.7	6.7	210.4	36.3	10	10.6	787.4
97	618000619001759	17.8	5	5.2	94	22.3	6.5	6.9	196.5	34	9.3	10.2	658.7
98	618000619001768	17.6	5.1	5	95	22.3	6.5	6.7	190.9	31.8	9.6	10.8	699.2
99	618000619001432	18.3	5.4	5.3	102	23.3	7.1	7.1	223.9	35.8	10.1	10.9	780.3
100	618000619001493	17.6	5.1	5.2	89	22.3	6.6	6.7	198.2	--	--	--	--
101	618000619001769	17.4	5.2	5.3	91	22.4	6.9	7.1	197.4	--	--	--	--
102	618000619001788	17.2	5	5.2	87	22.3	6.6	6.6	203	34	9.6	10.5	683.3
103	618000619001792	16.3	5	4.9	80	20.8	6.6	6.6	165.1	--	--	--	--
104	618000619001785	17.6	5.1	5.2	91	23.4	7.1	7.1	220.6	35.4	9.8	10.5	721.3
105	618000619001791	17.8	5.1	5.3	99	22.3	6.5	6.7	188.2	34.9	9.8	10.8	706.9
106	618000619001729	17.5	5	5.1	90	22.5	6.7	6.9	203.2	34.6	9.4	10.7	657.4
107	618000619001786	17.9	5.2	5.3	99	22.8	6.8	7.2	216.3	34.2	9.7	9.9	687.1
108	618000619001723	18.2	5.2	5.3	99	23.3	6.8	7.1	217.3	35.9	10	10.6	765
109	618000619001728	17.3	5	5.1	87	22.3	6.4	6.7	190.1	34.9	9.7	11.1	697.3
110	618000619001708	17.4	5	5.2	90	22	6.6	6.8	196.1	34.1	9.6	10.2	668.5
111	618000619001746	17.1	4.9	4.9	84	22.4	6.6	6.6	197.2	33.8	9.5	10.1	681.8
112	618000619001714	17.2	5	5.2	86	22	6.7	6.8	182	--	--	--	--
113	618000619001797	17.2	5	5	90	21.5	6.4	6.8	194.3	--	--	--	--

114	618000619001418	17.1	4.9	5	85	21.7	6.3	6.5	168	35.2	8.8	9.9	569.9
115	618000619001430	18.3	5.1	5.3	98	22.4	6.5	6.8	201.5	--	--	--	--
116	618000619001727	16.8	5.3	5.4	91	21.4	7.1	7.4	195.6	33.9	10.7	11	758.5
117	618000619001736	18	5.2	5.2	102	22.9	6.9	7	212.4	--	--	--	--
118	618000619001741	16.8	4.9	4.7	81	20.6	6	6.3	142	--	--	--	--
119	618000619001703	17.9	5	5.1	96	23.2	6.8	6.8	212.3	36.9	10.1	10.9	830.5
120	618000619001747	17.9	4.9	5.1	93	23.2	6.7	7	206.2	--	--	--	--
121	618000619001725	17.7	5.1	5.1	91	22.2	6.6	6.7	182.4	35.1	9.7	10.3	696
122	618000619001722	17.8	5.2	5.3	90	22.7	6.8	7	202.9	35.6	9.4	11	693.3
123	618000619001758	17.3	4.9	5	87	22.3	6.5	6.6	182.7	--	--	--	--
124	618000619001773	17.6	4.9	5.1	88	22.3	6.6	6.8	189.8	--	--	--	--
125	618000619001237	17.7	5.3	5.4	105	21.4	6.7	7	191.4	33.5	9.4	10.2	645
126	618000619001976	17.2	5	5.1	102	22	6.7	6.9	207.2	--	--	--	--
127	618000619001998	17.3	5.2	5	104	22	6.5	6.6	206.2	--	--	--	--
128	618000619001929	17.7	5.2	5	111	23	6.8	6.8	211	35.9	10.1	11	783.8
129	618000619001957	17.4	5.1	5.2	108	21.9	6.6	6.7	185.6	--	--	--	--
130	618000619001994	16.9	4.9	4.9	87	21.7	6.3	6.6	171.1	33	9.2	10.3	626.3
131	618000619001974	18.3	5.2	5.3	102	23.3	6.8	7	203.4	35.4	9.8	10.3	756.3
132	618000619001913	18.5	5.2	5.1	104	22.4	6.5	6.5	195.5	35.8	9.8	10.6	740.7
133	618000619001970	17.6	5.2	5.3	107	22.7	6.7	6.9	204.1	--	--	--	--
134	618000619001954	17.6	5.1	5	99	22.3	6.5	6.6	197.4	32.8	9.2	9.8	609.3
135	618000619001912	17.6	5.1	5	91	23.1	6.8	6.9	203.6	33.2	9.1	9.9	627.4
136	618000619001939	17.5	5.1	5.1	90	22.2	6.5	6.7	197.6	33.2	8.9	10.2	622.4
137	618000619001928	17.3	5.4	5.2	93	22.3	7	7	201.7	--	--	--	--
138	618000619001931	16.7	5.1	5	87	22	6.6	6.9	182.2	--	--	--	--
139	618000619001502	17.8	5	5.1	92	22.1	6.4	6.6	193.2	33.9	9.1	10.2	637.9
140	618000619001457	17.4	5	5	90	22.4	6.8	7	193.5	--	--	--	--
141	618000619001616	17.7	5.1	5.3	91	22.4	6.4	6.8	190	33.8	9.4	10	660.3
142	618000619001485	17.5	5.1	5.2	94	22.4	6.7	6.9	194.8	33.3	9.1	9.9	615

143	618000619001686	17.7	5.2	5.1	99	22.4	6.7	6.9	208.2	33.9	9.7	10.2	690.6
144	618000619001490	17.1	4.9	5	85	21.8	6.4	6.6	184.2	--	--	--	--
145	618000619001499	18.1	5.2	5	98	22.4	6.6	6.8	185.9	--	--	--	--
146	618000619001642	18.2	5.1	5.2	98	23.3	6.7	6.7	202.5	33.2	9	10.1	575.8
147	618000619001592	17.2	4.9	5	89	22	6.3	6.6	185.6	33.4	9.1	10	619.4
148	618000619001467	17.7	5	5.1	91	22.7	6.6	6.8	211.2	--	--	--	--
149	618000619001575	17.7	5.1	5	101	22.2	6.5	6.7	190.2	--	--	--	--
150	618000619001557	17.2	4.9	5.1	91	22.2	6.3	6.6	186.5	--	--	--	--
151	618000619001526	17.7	5	5.1	99	22.3	6.7	6.9	197.6	34.3	9.6	10.7	683
152	618000619001501	17.8	5.1	5.1	101	22.4	6.7	6.9	190.4	34.1	9.8	10.2	700.1
153	618000619001531	17.5	5.1	5.3	92	22.6	6.8	7	205.8	--	--	--	--
154	618000619001465	17.5	5.1	5.3	95	22.3	6.5	6.8	193.3	--	--	--	--
155	618000619001695	17.6	5.1	5.3	96	22.3	6.7	6.7	197.4	34.9	9.7	10.2	708.7
156	618000619001599	17.6	5.1	5.2	98	22.8	6.8	7.1	205.5	34.5	9.9	10.5	710.8
157	618000619001561	17.1	4.9	4.9	88	21.7	6.6	6.8	190.4	--	--	--	--
158	618000619001550	18	5.2	5.2	96	23	6.5	7	200.6	34.8	9.8	10.5	708.2
159	618000619001455	17.1	5.2	5.3	93	21.7	6.6	6.7	196.7	34.5	10.4	10.9	778.7
160	618000619001650	17.9	5.1	5.3	92	22.6	6.8	6.9	203.7	35.9	10.2	10.9	761
161	618000619001699	17.7	5.2	5.1	94	22.3	6.8	6.9	205.5	34.9	10.2	10.8	744.5
162	618000619001492	17.3	5.2	5.1	89	21.7	6.7	6.8	193.3	33.8	9.7	10.3	675
163	618000619001514	17.7	5	5.2	91	22.4	6.7	6.7	192	35.3	10	10.6	752.7
164	618000619001511	17.5	5.1	5.3	85	22.7	6.7	7	205.3	31.9	8.7	9.9	544.1
165	618000619001610	17.7	5.2	5.1	94	22.7	6.7	6.9	200.6	34.1	9.4	10.2	671
166	618000619001552	16.8	4.9	5	85	22.4	6.5	6.7	182.4	--	--	--	--
167	618000619001403	17.6	5.1	5.1	92	22.6	6.9	7	201.2	35.6	9.8	10.6	752.2
168	618000619001408	16.4	5.1	5.1	81	21.3	7	7.1	188.2	33.2	9.9	10.4	652.5
169	618000619001407	17.7	5	5.1	87	23.1	6.8	7.2	203.7	34.8	9.4	10.3	680
170	618000619001450	17.4	4.9	5.1	82	22.1	6.4	6.6	184.3	35.2	9.7	10.6	691.1
171	618000619001528	17.4	5.1	5.2	98	22.3	6.5	6.5	184.8	30.7	9.7	10.2	693

172	618000619001420	17.6	5.1	5.2	88	22.9	6.7	6.7	205.8	35.3	9.4	10.1	721.4
173	618000619001428	16.8	5	4.9	85	21.7	6.7	6.9	186.6	--	--	--	--
174	618000619001427	16.8	5	5.1	84	21.6	6.5	6.8	180.2	33.9	9.7	10.3	691.5
175	618000619001401	17.4	5	5.1	87	22.2	6.5	7	199.1	34.1	9	9.9	610.7
176	618000619001424	18	5.2	5.3	94	22.4	6.7	6.9	196.4	--	--	--	--
177	618000619001435	17.7	5	5.2	93	21.7	6.4	6.7	184.5	34.4	9.5	10.2	679.6
178	618000619001402	17.8	5.1	5.3	98	22.7	6.7	7.2	209.1	--	--	--	--
179	618000619001799	17	5	4.9	91	21.8	6.5	6.7	193.1	--	--	--	--
180	618000619001777	17.3	5	5	90	21.3	6.3	6.6	154.3	--	--	--	--
181	618000619001660	17.7	5.1	5.2	94	22.3	6.5	6.7	180.7	--	--	--	--
182	618000619001697	17.5	5	5.2	92	22.4	6.7	6.9	201.5	34.8	9.6	10.1	700
183	618000619001779	17.5	5	5.1	93	22.7	6.6	7	201.4	34.9	9.6	10.5	721.6
184	618000619001787	17.3	5	5	91	21.9	6.6	6.8	185.4	--	--	--	--
185	618000619001776	17.9	5.1	5.2	94	22.7	6.7	6.8	206	34.5	9.6	10.2	708
186	618000619001625	17.4	5	5	85	22.5	6.8	6.9	190	--	--	--	--
187	618000619001440	17.6	5.1	5.2	92	22.3	6.7	6.9	184.1	--	--	--	--
188	618000619001790	17.4	5.1	5.1	91	21.7	6.5	6.7	180.2	--	--	--	--
189	618000619001794	17.3	5	5.1	89	22.4	6.7	6.7	198.1	35	9.9	10.7	732.7
190	618000619001796	17.8	5.1	5.3	90	22.7	6.8	7.1	199.5	34	9.6	10.5	643.1
191	618000619001764	17.6	5	5.2	93	22.4	6.6	6.9	196.7	35	9.9	10.3	724
192	618000619001733	16.9	4.9	5	84	22	6.8	6.9	191.5	34.3	9.9	10.2	709.8
193	618000619001711	17.4	5.1	5.2	92	22.5	6.8	6.9	189.3	--	--	--	--
194	618000619001734	17.2	5	5.2	87	21.9	6.6	6.7	184.7	--	--	--	--
195	618000619001439	17.7	4.9	5	85	22.5	6.6	6.8	195.5	34.9	9.5	9.9	682.2
196	618000619001755	18.1	5.2	5.2	92	23	6.6	6.9	200.6	35.9	9.8	11.4	736.4
197	618000619001748	17.9	5	5.1	89	22.3	6.7	6.9	194.1	34.7	9.7	10.6	687.5
198	618000619001795	17.4	5.3	5.1	96	22	6.7	6.9	189.9	--	--	--	--

Supplementary Table A2 Detail information of markers located in the high-resolution genetic linkage map of silver carp and the genome of zebrafish.

Linkage group	Marker	Map position (cM)	Genome of zebrafish		Marker sequence
			Chromosome	Start position	
LG1	ref-4252	0			CTCCAGCGCAGCGAGCGAGTCAGTACCTGAATCTCTGGGCCGGACGCATCAGTCTACTGTCCAACACGAATGCCGGGCTGCTCTTCTGACTGGGATTGTTGGCGAACTTGA<br>CTGTGATTGGCTCGGTGGAGCCCGGCGGTTTCTGTGCGTTCAGCCCTTTAATGGCCTCCTCGGCCTCCGCGCGCCGGTCGAAGCGGATGAAGCCCACGCCTCTGGACACGC<br>CTGAACAACACAAACACAATCCAGTCAGAACAGTTTGGTACACAGTTTTGTAGACACCAAACACACAAAACACTTCACACACACACACACAATAAATAAATATACACACA<br>CAATGGTTGTGTTGGCAGATATTTCAGTGTAGGGCTG
LG1	ref-57997	1.587	Chr7	73945846	GCCATTCGTCAAAGATGGCGTCAGTGAAGGTGTGCAGCAGCACGAATATGGGGTCGTTGGGGGAGAGATGCGTCTGTCCGCCCGTCCCGTTCAGAAACAGATGAGCCAGG<br>TTATGCAGACTGCGCACCGCGGGGTCGTAGTTCCCCTGAGGGGCGCTGTAACCTGACAAAACAACACCACACCTGAACACACGGCACACACAAACACTGACGCTGAAGCT<br>GCGACTCACCCTCAACGCTGTTCCTGAAGCTCTCAGATGAGGTGGAGTAGAAGGGCGGCGTGTCGAACGCGTTGATCTCCAAACACGCCGCCACGTCCTGCGCCTCCGGCA<br>GCCGCTGCACCATCGGACGCGCAACATTACCGGCAGGGTTTCTGCGGATGGGACTGCTCTCCGAACCTCACCCAACAATAACAACCATATTAATAACAAA
LG1	ref-46353	4.372			GTGACCGAGACGATCTCCGTGCAAATGAAAA
LG1	ref-56617	5.066			ACTCGCTACTGCTAATAGATACACAGACACACTGCTGTGAGACTGTAAGATCTGCTGCTGCTGCATGAATAGACATACATCAATCCTGTAGCAGATCTAGGCAGGTGGAGC<br>TGGGGAGGTGGAGGGGTTCAGAGGAACTGCAGGACTAGATTACAGCAAAAACTGAACCAGTTGATCAAGCAATCTCATTGGCTGTAGGATCAACAGAGGCCAATCAGCTT<br>GTGATGTGATGGCTTGCTTGCTGACTGCATGTAAAGGACTGATTAGCCTAACCGTCACGTATGTCCCAAACAGGGTAGAAATCCCCCACAATTCATTGGAAACATTGGAAA<br>TTCACAAGAGTGAACTACGCCTGACTAAAACACCAAA
LG1	ref-6188	6.63			ATTTACTTCACGATTGAGCTGCATGACGTTT
LG1	ref-4612	7.87			GTTCACTACTGTTTTGGACACCACTACAAATCAGGGGTGTTTCCTCTGAGGAGGCAAGGGAGGCAGTGCCTCCTCAAGAAACTGGATGAGAAAATAACCCATACTATTTGT<br>ATTTGAAGTAGATTCATTTACATTTACCTGGCACTGGAAGTCCTTTCACAAACTTGTCTATTCTTAGTGCAGAACCATGGTGTCACAGAATGAATGAAGCACACGAAGAAG<br>GATAATTCCAGGAAATAGTCTTTTAATCCAAACACGTGGAAATACAAAGAAACACATGGGAAAGCTGGGTCAACTGAAGGTGAGCAAACAGAAGGTGAATGCAATGCAA<br>ACAAAGAACAAAACCCTGACACATGCATCATTCATATA
LG1	ref-44550	8.528			TAGTGTTGGACGACAGCTCTGCACCATCATT
LG1	ref-47616	9.908	Chr7	66950545	ACTGCAATTTCTCCAGAGCATCCCATTTGTCCCATTCCAAGCATTCTGGAGGGATTCCCTCTTCCGCTGCTTCCCTCTCTATTCCTCCACACTGGGAGGCATCTATATTTGGA<br>GAGAATTATGAGACAGCCCCAGTCCAGATGTTTTGCCAGGCCGACACCTGCTCTTAATACAAGTCAGCGGGGAGCAACTAGCCCACATGCACGTCCCCGAAGCTCTTGCA<br>AAGCTCAGTCCCATCAGCTTTTTTTTCCTCGGAGCTGAGAGGCGCTGCAGTGTCTCGCCTCAAAGGCGCCTGTGGCCTCAATTACTTACATATTACTGCAAGTGAGACCACT<br>TGGCTGTGATTGTTAGATGCAACCCCCAACAAGGATCTGTAGACGTTTCCTCTTACCGTTCATTAAGTGAGATGTCCTGTTCGGAAGAAAGGCGAG
LG1	ref-57066_6	10.886			TTCACAATTTGTGTTTTTACTGCAGGTTTTTGTGTAATTCTTACTTTGGCGTTCAGCACCACTTAACTTCGTCAAGTGGGCGAGGGAAGAGTATTTGCACAGACCCTCGACCC<br>CTTGATTTTGACCAAGGGAGTGAGTCTACTCATAAGCACACTTCAGGCAGATCCATAGGCCA[C/T]AATGCAACACGTTCGTGACGTCACAGCAACAACTCGCACTCAGTTT<br>ACCCACACGTGATCAATGGCTCATGTTCCATTTAAACACACCGCAAGGGCTCCGCCAGCAGTGGGCGCTCGTGAGCAATGACGTACATCAAGTGAACGAGACGGAGGGAA<br>GTTAGTGAGGGAAGAGCATAAGCGGACCTAAACACAGG
LG1	ref-72183_3	12.003			TG[C/T]GGGGGTACGAATGTGCTGCTGAACTAGTC
LG1	ref-72183_10	12.564			TGTGGGGGT[A/G]CGAATGTGCTGCTGAACTAGTC
LG1	ref-22224_31	13.689			CAATTTGAGGCGACACAAGTGACTCAACTTTATGCAAATGGCCAGTGACGCGATATGTCGGCTGCTAATGGGAGTCCGCGCAAATATGATGTGATCGGTCACATGATCTGT<br>CGCAGAAATCAGCAGTTGAGTAGGAATGCCTGCTAGTAACCCACAGTACAGCAAGCGGTTGATCTCCTGCAATGTAATCGCTTTCAGT[A/G]TGAACACTTCTTCCTCTAGA<br>GCAGTGGTTCTCAAGCAGGGGCCGGGGCCACTAGGGGGCATCAGGATGAGTGGGGGGAGGAGCTTCAAATATTGTTGTGGACAATGGGGGGATTTAAGTCAAAAAGGT<br>TGAGATCCACTGCTCTAGAGACCAGTGTTGGGGTAACGCAT
LG1	ref-24364	14.036	Chr7	710900	ATGCAGGTATGACATCATCATATATGCACATATTTGTAAATTTGATCTCACACACAAGTTGAAGTGTATTAATAATTGTTGCATTATTTTCTTCACAGTTCAGATCTCTATTG<br>ACCGTGAGCATTTACATCCAGATCTGAGGGTTTCTAAGAACTGTAAAACTGTGAGAGACGCTCCAGATTATAATCACACTGGAGAGGGATTTCCATATGAATTATGTGCGT<br>TTGGAGCTCAAAGATTTACATCTGGTCGTCACTATTGGGAGGTTTTATTGGCACGGGAAAATATACCTCCTAAAAAGTACTGGTTAATTGGTGTGTCAAAAGATGAAAATT

					TTAAAGCAAGAGACAAAGCTGCTTTAACTCCATCAGATGGTTTCTGGTTTCTGTGTTCAGACGGTCCTAATGGCTTTTACACCAACTCTGATCGAT
LG1	ref-41299	14.604			ACTCCCTCTACGAAGACCTTGCCCTACAGGT
LG1	ref-35626	14.966			AGATTGTCATGTGCTTTTATTAAATTACAAACACATGAAAAAACAATCGTGTGCATCTTGATATCATTTTTGATTGTTTTTCTTGAGTCCCTCTGATAAACACTAGATGGAG<br>CCACAGCGGTGTGCATCTTGATGTCATGAGTCTTGAGTTCCTCTGATAAACACTAGATGGAGCCACAGCATTATTTTCCCTTGAGTTGAGTCAGTGTATACTATAGTATGTA<br>TACTCTGCTCAGAATTTGCTTTATGTGTGGAATACCTACATTTTAACATGCAGTGTGATGAATGAAATTATTTGATCAGACCTAAAATTGGATTAAAAGCAGTTGAATTAAA<br>CCCTCAGTTCAATGAGTTCACTGACAGCAGTGT
LG1	ref-69433	15.244			TCTTTCTCACCGATGGCTCTGCTAATTCAAC
LG1	ref-58923	15.389			ACAGGTATGACGAACGTGTTGCAACACTGAC
LG1	ref-61049_16	15.643	Chr7	65926333	GAAATTATGAATATGCAACCCCACATGCATTTCAGCTATTCTTTTTAAGACATAAATAATGAATGGCTGGAGATGAAACATATTACTCTGTAAACGTCTTACAGCCGAAAG<br>TGATTTTACGACCTCGTGCTGCTCTGCAGTTCACTAGCAAAATCCTGGGCCCCTACATTTGGGTTTGGTTGGGATATTTTATATTTCATGAACTTCCCGCGATC[A/G]TATTG<br>CCGTTCTCTTGTCCCTTTTTTTATTTGAGAGCTGTTTGCTTCACATTTGTCCCACCACAAGAACAGATGTACAGTATGTAGAAATGTAGTGTACAGTATGTGTCACACTTAAG<br>TCAAAGTAAATTAATGTGCAGCTCACTGATGATATTTTGACCTTTTGTGAAAATGTGCCATATAGCAGTGCATGTTGATGCACTACAGGATGTGTTGT
LG1	ref-42284	15.721			TCACCAGACCCGACAGCAGTGCAGTAACGTC
LG1	ref-62193_1	16.209			GTAGATGAAGTAAGTCATGCTCGCATCAGCTGACTCTTAGCTGATCCACGTCTACTTATAGGAATACAATGCCTATCACAGCATCCCTATATATAAGGTCCATTCTGTATTA<br>TCGGCAGGTGTATCGTGTTGTTCTGAAGCTGATTAGACAGTGAATTCGGCAGGCTAAACCACATCATATCAATAGGAATTGTGCAATC[A/G]GGAATTTTGCGATGCTTTTG<br>CTCCCCACACGAATTTCACATCAGATTCAAGTTTGTGAACTTGAACTTGCAAATTCTCTGCATTGATCAATAAGCTGCTTGATTTTGGAGGTGACCCAAGTAAGCATTGCTT<br>CTCGCATCACTACATTATTAACATGGACTCTTTGCCCGTAAATCTCGCTGAAGTTATACTAAGGTGACTGCCTCAAGAACAAAGTTTAGACAAGATCTG
LG1	ref-395	16.778			GGTCTGAAAACGAGGGGTGTGCTGGTCTGAA
LG1	ref-17331_31	17.133			ATATGGAGACCGAACTGCATGCTTTGATGA[C/T]T
LG1	ref-55780	17.774			TGCTGTCTAACGAGGAACATGCAGAACAAGA
LG1	ref-28255	17.982			CTGAGCAACACGATACACCTGCCGATAATAC
LG1	ref-68407	18.451			TCGCAGGAGACGAGGGAAGTGCGGAGGGGTT
LG1	ref-55848	18.643			AGTGAAACCTCAATGAGACAAAAAAAAAAGTGTAGGGCAGGACTTGACTGGATTGTTAAAAGTGTTCCATACTAGAGTTTGCAGTCAATTTTAGGACTTCTTCCCTTCCTG<br>AAACACTTAATTGGTTGAAACATATGCAGCTTTGGCCAAAAAGGAAACTCATTTCAGAGTCTTTTTGATGAAAGATTACAAAGACAAACACTTTTCTTTCGAAGAGCGTGC<br>ACTGATGAACTGTTTATATCAAGATGATGTGTTAAGATACCTGAAGAACTTTTTCCACTATCAAGAACCTTTTGTGCATTGGAAAGGTACCATAAATGTTAAGGGTTCTTCA<br>TGGAACCATAAATGCTAATAAAGATACTTTATTTTTTAAGAGTGTTTAGACTCTTTTCCACCATCAGGCCGAACGGATCCAAGAACTGTAAGGAATA
LG1	ref-47642	19.083			GATAGTCTCTCGAGCCGCCTGCTTCCAGCTT
LG1	ref-20485	20.443			TAATGCGATCCGAGACAGATGCTTGATGATC
LG1	ref-48101	21.194			ATAAAACTGACTCTTTCTAATTTTTAATGATTCAAAGTTAAAACAGGCCTAAGTGTTAAATTCTAAGCCTATAAGCTTTGGGTTAAAAACTGTTGGATGGGTATAATTTCGC<br>ACAATGCTTTCAGTTGACATTCTGATAGTAGGGCTTGACTGTGAAGGTTGACTGGACTGTTCAAAGTGCATGTGAGTCGGGGAGTGGGCACTTTTTGGAATGAATCTAGGC<br>AGGGAATTGTCGCACAGCAGGAAAGGGCGCTTGAAGCAGGGAGAGAGACCACTCTGGTGTTTTTTCCATATCAAAGCAGTCACTGCAAGAAAAAAATATCTGGAGCCACA<br>GCAGTGGTTCTGAAGACAGCTCGGCGAAGCTGTCTG
LG1	ref-43710	21.298			AGAAGATGGCCGACACTGGTGCGGGTAGCGT
LG1	ref-56650	22.21			ACGCTTAATGCAGGCAGCATCTGAGGAGGGAAATTCCAACTTTTTGGGAAATTCCAAGTTCATATTACTCATCAAATAATCAATGCCTATGATACAATCCTCCTGCTGTAG<br>GCGTATCCAGCTCTGTATCTCTTGACGGATGTCCTTCCTCTGTCACCTATGCTGTCGTCGTCTTCTAGCACTCTTCGTCTTTTTCACCGTAGTCTTCGTCTGGCTCACCTCTGC<br>TTGA
LG1	ref-49544	22.646	Chr11	24012135	TATAATATAAGTATCCGGTGTTCCCAGAATGTGTCTGTGAAGTTTCAGCTCAAAATACCCCACAGATTATTTATTATAACTTGTCAAATTTGCTTCTATTTGGGTGTGAACA<br>ACTATTTGCTGTTGAGTTGATTCAACTCATCGACTAGCATGTGCCGTCATGTTAATCTTTTGTGCAAATCAGCGTTGAATTGAACCTCGTTTGTAAAGCGAAAGCACTGCAA<br>CAACACTCTACAACAACAACTCTTCCTCTTCTCTAAAGCAGCCCAACATGGCCTCACCCCCTCTGTTGTGTGTTCTCGGGGGCAGGGTTTATGTCAAATTTGGGGTTTGTGA

					TGTCACCAACCCAGGAAGAAACTCATTGTAGTCCCTACCAGGCGTTTGTTGTAGCCCGTAAAAAGCAATTTCTGTAAAAGAAAATATCTCCCTTT
LG1	ref-22500	23.359			CTTACCAGAACGACTCCTCTGCCAAACAACA
LG1	ref-27610	24.273			CCAACTCCAACGAGCCCTATGCAGGCGCAGA
LG1	ref-13500	24.955			GTCCGCTCCCCGATTCAGGTGCCCAGTGATG
LG1	ref-73551	25.134			GTCACAAATTAGAAGTCTAAAATGCTAATGTTGGAGGATTTTGATATAAGAGAAGAAGAGCTTGAGTTTGTTGCACAGCCCTATTTGTTTGAACCGTGAGAGGCGTCTAAT<br>CTTACGCTACTCCTACATCCTGCGTCATACGTTGCTTCAGGGGTTACTCGTTTGGCGCAAGCCGATTTGCGCAAAATTCGTACAGTCGTCCGCCGGAAGCTAGTTATTTGAA<br>TCTGTAAAATTTTAAATATGGATATTTTTCTTACAAAAACACACTTCAAAAGGCCTTTATTAACCCCCTGGAGCTGTATGGATTATTTTTTTTTGTAATGGATGGATGAAT<br>TTTTTGGGCTTCAAAATCTGGCCCCCCTTCACA
LG1	ref-2708	25.289			TCCTGTTCCTCGACCTCCATGCTCCGATCCA
LG1	ref-20571	26.594			TGGAAGGCTTCGACTGTGCTGCCCGCACCGG
LG1	ref-37503	27.136			GCGCCTACTTCGACATCATTGCTTCTTTAGC
LG1	ref-28496_3	27.614			AAAGTCTTGATTTTGTCTTTGGGGTCTACAAGAATAGGCTTTCATGCTTGAATGTTCAAAAAACAAATTTTTCACATATTTGTGGTGTGTTTCCCATACAACGATGTAACTC<br>GCTAATTAACTACCATATGTATAATGCATCATTGGAGAAACTAACCCAAAACCATAGTG[A/G]CATAGTCGCACCCCTGTCGTTTGAAGTACATTGGTTCAGCAATATCGTT<br>AATGACGTCGCGCAGGTGGAGGAGTGATAACGAGATCTCTGAGATGCTGCTGTAACGATCATGGCACCTAATGATGACCTCACTGTTCTTTGTTCCATGTTATTTTGCTTTA<br>TTTGATGTTTGACTTTGTAGTTTCCTTGCGCATTTTA
LG1	ref-41152	27.774			ATTCAATGTACGAAGAAACTGCCGTCGCGTC
LG1	ref-17162_17	28.259	Chr7	69466221	TTTATTTGTTGTAACATATATATGATTATGGATTACTCACTACACAATACATTCAAACCATAAATCTTAAAAACATTATAGCCTATTTTCCAAAACGAATAAGTAACATTAA<br>ATGGTAATTTTCTGTGCAAAAGAAAAACACACCATAAGTTATTTGCATTGTTTCCTCAGGAACACTGGCATTG[C/T]GATCGAGGCTGGGGAGTCCAGACTGATCTCCAGTG<br>CTGTGCTTAGCGCTCACGACAAAGACACTTCTTCTTCGGACATCATCTATGTGTTTGAGAGCATACCGAGTCATGGTGTCATTCAGATGAAGGTCAGTTTGCTTAATATGCA<br>GGGAGAAATATATCTAATCTCTCACTGCCAAAAANNN
LG1	ref-1601_5	28.844	Chr20	4547766	CTGTTTTATTGATGTCTTTCCGCAGTTGAAACACTGGTTGAGAGATCACAAGTAAACATCTATGCATAGGTCTTCTTCTGCGCTTTTTAGCAAACAGAGTTGCATTACTGCG<br>CGCATCCCCTTCTGGATTGGAGTGTGGATCATCTGGGACTGGCTGTATTCATCGTCTGACTGTATGCACTGAACCGTGACGTCCGTAATGTGA[A/T]GATTCGAAAGGAATG<br>CAATGTACCATTACACCCCCAGTAAATATGTACTGTGTGCTTTCCTCTCAATAACAAAATAAACACACAACAAAAATGACAGTTAAGAAAGCATCTGATCATAGGGATGTG<br>GACTTGTTTACACAAGCTTTGCAATTTGGCACTCCAGTCAAGTCACATTTATTTATATAGCATTTTATACAATAGATTGCTTAAAATCAAGCAGCTTTAC
LG1	ref-21951	29.472			CCTGTAAGTCCGATTGAAATGCCATCCATTC
LG1	ref-50307_27	30.325	Chr7	71116896	CCATCTGGACAGGGGCGGGAGCTGCGCCACATGTTCGGGAAGCTGTCATTGCCAGCTGTGTCCTGCTCCCATGGCGATGGCCCTCCTCTTCCTGTCCTGTCATCTGAGCCAA<br>TCAGGGGGACAAAGAAGATTAATGGACATCGTGTTAGTGCCATGATAATGGAATGGAGCCCTTTGAGGCAAATGTGTCGTTTA[A/C]CACAGTAGTGAAGCTCAGTTAAGA<br>ATTCATACCATGGTGAGTGCGCTTGGCCAATGGAGATCACATTAGGGGTTTTAGTGGTTATTAGTTTCTGTGGCACTAGCGTAATTACAAAAATAAAGCAACTTTGCAAAT<br>GCCACTTTCATGTGTCTATCCTCGCCAACTCAAACATCA
LG1	ref-5482	30.976	Chr7	11471411	AACAGTCAAGGATCTACCTGGACATTTTCAAGCAGTTTGTCTACTGTATCCCGCCCCCAACTCCTTATCTGCACATTTTATGATCCTTGTGATTTGATCTGACTGCGAAACGT<br>CTCTCGCACTCGGATGCATATGCTCAAACGCAGCAGAACTGTGCAGGGTAGCTTGCTGGATAAGATGCATTTACATTGTCAAGAGTCTGACAGTAATAATTTGGTCTGTCT<br>GAAAAGTGATGTTCACTCGCAGGTGTTAGACTCAGTCCCAGCAGTGAGGGTGTCCAAGAAACAGGCTTTCCTAACTGAGCCCAGACATGGAGAGGAATTCACCAGAGAGA<br>GGAATTTTAAGAAAGCAAAGGCAATGCAAATTAAT
LG1	ref-17331_25	31.77			AACTAATGAAGAACTATCAAACCTTGCCAAGCATCTGTCACTTGCACATCCTGTCTTGTACAAGACATTAATTGATCTAAATTGATCGTAGAGATCGTTGGTGGCTATGATA<br>AACTTACAATGCTTTTGCGAAAAGCAGCCCAGAGCCATCTCTCTCAACGGCTCTATACATACCAACCTCGACGACTGTGTAAACAATAATATGGAGACCGAACTGCATGCT<br>TC[C/T]ATGACTCTGTTGTCCTTTTTAGCAGCCATTGTGAAGTTAAACTCTGCATTTTATAATACTGATACAGCTGTAATAACCTACAATCAGAGGCAGCTCTATAGCTTTCA<br>TTCCGAGTAAATTAATTGAGCAATTACATTTAACTTTTTATTTTTTGTCATTTTTAGTTAAAATTGGAATCTGGCAAATATTGGATTAATGACTAAAAC
LG1	ref-53350	32.807	Chr16	15682888	CATTTGTAGGGGTGTCCGAAACGATAGTGGACAATATCGAGTGCACACATTCAATCCCGCAATGCACTGCAATAACGAGTGTACAACTGATGTACGCTCAACGGCTAGAG<br>AATACCCATATTTCACTGTGATAGTCGCGCGCTGAATGAATTCCCGCTTCCCACCAGAAAATGCCACCTGCAGCTGAATCGTTTTCCAAACCACTCTGGTCCAATGTGACTA<br>CAGTGTAATATCATACAGGTGTAAATTCATTTCCAGGTGATTATTGTTTAATTAAGTCAAGTCAAGTCAAGTCACCTTTATTTATATAGCGCTTTTTACAATGCAGATTGTGT

					CAAAGCAGCTTAACATTGATAACTGGTATATAAT
LG1	ref-56348	34.706			TCCACTATATGGAGAAAAATCCTGGAATGTTTTCCTCAAAAGCCTTAACTGCTCATTGATGGAATGTTTTTTTTTGTTTGTTTGTTTTTTACCTATTTTTTCTTTTCAACTGGT<br>TCATTCCTGTTTATAATTTTGATCAAATAATTTGTTCACAAATTGAATTGAATTATTTATTAACAAATCAGACAGATTTATTCATGAATTGGAGTCCGACACACTTGCATTTA<br>AATCAGTGTGGTGACAACATTCAACATATATGAGGTTATGTGACAAAAATAGCATATAATATATATAAGCATAGAATAGCATACTACTGTTTGTATTGTTTAGTACTTAAC<br>ATACACTATCATTCAAAAGTTTGGGGTTATTTATTTATTTATTAAATTAATACTTTTTATTCAACAAGAATGCATTAAATTGATCAAAAGCGA
LG1	ref-44508	36.503	Chr7	66905921	CACTTCAAGATCTAAACAATCAGTCACAATGGGCGTTCTGACTGTGGCTAAGTGTCTGCATGGCTCGCGTCTTGGATTTTATGAAACACTCTGTGTACTTTGGAGGATTGTG<br>GTAACAAATATCTGCAAGTTCAGCAGAACCATCACCACAAAAAATTAAAGAGCGTGCCAAAGTCAAAGCAGCATGATCATGTGCATGTGTGTGGGAAGAGTTGTGGTCTG<br>TGACAGAACACAGATAATGAGATATCAAGACAGAAGACAGTCAACTAAAGATATCAGATGAAGGATGGGGAATTTGTTTCACGCTAAATATCTCCTTGGAGTCAACGGAA<br>ATGAAGAATAGAGGCTCACTCACCTCCTCTGGTGAAG
LG1	ref-15030	37.216			TGGGTCCATTGTGAAAAAGAAGTACACATTAGCATATTTTTAGTAACTTATTAAAGAAAGAATATACTTAAGTGTGAACTGAATGTAACTGTAACACAGTTTGTAGTTTGG<br>GGAAGAAGGAAGTGTGAACCAGCTAATCTTTAACATGAATTTTAATGACAAAATCAAATTCAAAACTAAAAGCAAAAGGTGCTGCAGCCACTTTTCAGCCGACTGCCGCG<br>CATGAACACAAAAGAAACAACATAAATACCACACATCTGTGACATGCACTTCATGCACTTGTTCTTTCCTTAATCATTAAATTCATTCTTTTGTTTTTGTTCTTCCTCAGACC<br>TTCACGAGGCTAGTGGAGTGGATGCCTAGCGACAAGAGAAAGGTGTCCTCTGGAACTTTAACCCCGCGTTATGCCACCGCTGTACCAGCCAGAGATC
LG1	ref-15296	37.533	Chr7	12475736	TCCCCGGACGCATCACACCAAGTTCTGAGAAAACCAAAAAGTTCATTTGAAATATATTTACATTTTCATAAAACATGTATATATAAAATATATATATAAATTATACAACGT<br>ATATTAGGTAAAATAATAAATTTGATTTAACATTTTTATCAAGGTAGCTCACTAGGTGAGCCTATTAGCTAGTATTAAATTCAAGAAGCTAATGAACCACGAGCATTTCGC<br>AGAGCAGCGGTTTTACAGCGCGCAATTTAACCACCACAATATGAAGTGGCAAGTATGACTTTAATTAGAAATCATTTCAGTTGAATAGACACAATCCAGATGAATATTCTG<br>AGTGTGTGATTCTGTGATTCTGACCTGGTCATCATAGCGATAGGTGAGATACTGCTCTCCTGTTGTGTCTTCCAGAAAGCTTCCTTGTGGAGAAAGTG
LG1	ref-45007	37.905			ATGTTGCTGCCGATGTTATTGCTGCTGTTAT
LG1	ref-22727	37.929			AAGGTGAGCTCTTGAAGACAGTGCTATCATTGAAGGACTTTACAAGGGCCATCGTCTGCTTGCTTTGGCTGCATGAGGCTTTGATAACAATCCTTGAATGTTAGTCCAGCTA<br>GCTTTTGTGTCCCCCTGACCTCTCGGGCACCTTCAGACAGCTGAAGAAAGGATGAAGAGTAAAAGCTGCAGCTGAATCGCTGGATTGCTGTGGGTTGATTACCTACAAAAG<br>AGAGTGCTTGTGAACTTTTTCTTGCCATACTTTCTTTGGACAGCAAAGTCAAATCTTTGTGAGTAAAATAAAAGAAACAGCTGTGAGATGTAACTGTTTGTTGAACATTACA<br>GTATGAATATTTCACCTACGCATGATGATCAAAC
LG1	ref-19919	40.885			CCTGTCTACACTGAACACGACAAACCGCAATTGGACTACGTCAGAAACAGAACGGGGAATCCATTTACTGTCGGGAATTTGTTGTGTCGCGTCACGTCACGCCGCATCCAG<br>TGTAGACTAGGGGCCCTATCATACACCCAGTGCAATGCAACAAACGCAGACGCAACGCAAAAACACATGCATTTGATTCGTTGAACAACGAGGGCGTAATCAGCTGGCAA<br>GTCCTAAGGGGAAGGCTTGGCTCATTAATATTAATTAGGTTATAAGACAGAACTTCAGGCGATGGAATGTTTATAAGTTTTGTTGGACTCGCGCACGTGTTTTGAAAGCAA<br>ATCTCTTAATATTCGGACGATAGACGGCAGTGCGTTC
LG1	ref-47123	43.607			TTTTTTATTAACTTCCTCAGTAATTGTTTTTTGTTGATTTTAACCAAATAAATAAATAAATAATCTGTACTCCAGCTTTAAACCATTTAATAAATAAAGGGATAATCTTTAAC<br>AGTCAATCCTGCTACCATAACCTTACTGAAAACTCAATATTTGCAGAAATATTAAAATTCATATAAAAAATTAGCTTGAGATTGCACCATGTAATTGCAACTTCTATGCATT<br>CCGGTTGGAATGAAAGAATCCTGATAGAATGCTTAAAATCGTGAAGACATTTATCTCAAAACAGAGTAAATGAATGAATTGGTTAATACAATTTAAAGTAACCATTATTAT<br>TTATACATTTCTGAAACTGGAAACTCAGCATCGATGAGAAGTTAAACCACAACATAAAAGTAAGTAATTAACACTTTAAATCTCAAAACATTCAT
LG1	ref-57775_14	43.901			GAGTATGGTGAGTAGACAGTTATGTGTAATATTCCAAAAAGTAGCTTACAGAGATCGCTGGAGAATCGCTAGGAGATTCAAAAGGTAAGTAGAGTGGGTAAGTAGTCTGT<br>TGTCTGTAGTTGAGACCAGACAAAAGCTGCATCCCAATTCAGAGGCCACATCCTTCGTAGGCCGCATTCGCAG[C/T]CCAATCGCATCACTGTAGTGTGATGAAGGCTGTCC<br>CAGTTCAAAGGCTCCTTCAAATGTGGCCTCAAAATGCATCCTTCGGGCAATGAAGGATACAACAGATGGATCCTTCGCAGCCTAGCCTATCCCAGAATTCATTGTGCGCTG<br>GTGACGACAGGATTTTTTTAAGAGAAGAATGGTATATATA
LG1	ref-24994	43.92	Chr5	13287113	AGCCCAGAGGAGAGGGAGCTGCTGGCGGCTGTGACCGAGCAGGGCTACTCTCTCCGTACGGCCATCATCGCCCTCCAGAAAACTGGCCAGCAAAGTCCCGAGCAGGTCAG<br>TGGGCTTCACTGTGAAATATGAGCTGGGATTCGTTTGGGTGTCGAGATGCAATGCTGAGATTCTCTCCACCATCATTGGTGTATCACAGGGGAAGGTCAACGAAGTGCATG<br>CTGGGTTGCCAGTACAGAACATGCACCAGTTGGTCACACTGACCACATTTATTTTTCGCACAATCACAAATAACTTTTGCTGCTAGTAACACTTCACAATAAGGTTTCATTA<br>GTTAACTACATTAGTTAACATGAATTAAGAATGAACAATAATTCTACAGCATTTATTAATATTAGTTAATGTTCATTTCAACATTTACTAATACATTA
LG1	ref-5457	44.422			GGAATCAGAACGAAGGCAATGCAAAGACTGT
LG1	ref-9798_17	44.509			AATGGTGATACGAGTG[C/T]GATGCAACCCTGATG

LG1	ref-9798_10	44.607			TACGAACCCCCTGCAGTTCCTACAAAAAACGTACAAAAAAGTAATCAGATTACATTACATAAAATGTGTAATGTAATGGATTACGTTACTAACTACACTTTTCTTCATGTAATTTGTAATCAGTATGGACTACAGTTTGTAAGTAATCTACCCAGCACTGCTTATCAATATCATTTAATGTGTCTGAAAATATTAATATAAATGGTGAT[A/G]CGAGTGCGATGCAACCCTGATGCATAAATATTTGAGATTAAAAACCCTTATCAAGTCAAAATAAAGACCCTGTTATTGTTGTTAAAGGGTTAGTTCACCCCAAAATTAAAATTCTGTCATTAATTACTCACCCTCATGTCATTCCACACCCGTAAGACCTTCGTTCATCTTCACAACACAAATTAAGATATTTTTGATGAAATCCGATGGCTCAGTGAGGCC
LG1	ref-41386_25	45.174			GTTTACCATGCGACTTTGCTGCCA[A/T]CGAGTCA
LG1	ref-41386_17	45.346			GTTTACCATGCGACTT[C/T]GCTGCCATCGAGTCA
LG1	ref-58640	45.395			AGCATGCTTACGATGTTCGTGCGTGGACGCG
LG1	ref-21759	45.821			ACAGCCTTCAGAAATAAGGAAACAGCCCCCTTAGTCCCCTTGAGTCGGCTCAATGTGTCTTTTGCCGCTTGCAGTGCTTCGTGTTATTCCTGATTTGCGTATTTAATCTCTTAAGCTATCACAGATCTGTCATGGTTACCATCTGAGACACTCCCTGCTAATACTCCATCACTGAAAAGGCATTAGTGTCGTGATTTGGATGTTTCTTATGAGCTGGTACATTAGGAGACGGCAATACATATAGCAGTGGAAGTGATGACAAGTGCTTTCCAAATCTGACTGTGCAAGCGAATTTAAATGACTCAGACTGGGTGGTGTATTTTCTGTCCTGTTCTAAATCAGGATATACTAGATTTTCTATAGGGAGTC
LG1	ref-49576	46.472			AACTTGGGTACGACATGCATGCATTATGACA
LG1	ref-40568	46.943	Chr7	53012369	TAAAGCTAATGAGACTAAGAGGGTGTGGGAACGCTGGCGGTGGCAGTCACATATGTTTGCGCTGTCAATTTCGTCTTCAAATAAAACCGCAAGACAAAAGAGGGACACTCTTGCTCATGCTCGGAGATTAATGCTGTCATACAATCCATTAGATCTACAAACAATGAAACAGTAACCATGCACCACTATCGAATGTTGGATGAATTTATCCAACAAACTCGCTAGCCTTGAGTAGCTGGACTATCAGCATACAGTCTAGTCCTCTTTGTAGTCTGTTCCACCCGCCCACTTGGACAAGAAGGGGCGTCTGATAAGCGAAGTGACCAGAGTTTGCTATAGGGGTGGGCAGTGATAATCTTGTCAGCAAAA
LG1	ref-61350	47.458			AAACTCAAGTCGAGTGCAGTGCAAAGTTACT
LG1	ref-41758	48.123			AACCTTCTACCGATGAAAGTGCACTGGAACG
LG1	ref-39509_10	48.2			TGCGATAGG[C/T]CGAGGGATCTGCCCAATGCTCT
LG1	ref-42265_8	48.218			TTTTTAA[A/T]GACGACTCTTATGCTCACCAAGGC
LG1	ref-42265_19	48.229			TTTTTAATGACGACTCTT[A/C]TGCTCACCAAGGC
LG1	ref-23888	48.373	Chr7	11461035	CGCAATCACAAATAAAACTGTTTATCTTTACAATATTTTATCGGAATGTAATAAACTTGATACACCTATCCTTTTTGCCTGTGAATCTTTCACCTACAAACCGTCTGGCGTGTTACACCCATCTTACACATTCCAATCAATTCTCAATGGATAAAATCATTAAATGAACTGTTGCATACTCAGCGGATTTATTTGTTGAGTATGCAAAAACGACCAAATTGCACATCCCTAATGGATATTCCAGATACTGAGGATGACTCTGTGGACCGTTATTACCCATCCTGGTATGTTCCCAAGACCACTGAACCAGATTTCTGGCTGTTTGTATAGTGAGTGTCACCTTCTGACTCCTGACCTACATGTAAAGTAGTATGTAACACATTTAGAGTCACAAACAGGTGCATGCTTCCTCAGACACAATGATATATTGA
LG1	ref-34656	48.758			AAACAGGAAACGAGTTGAATGCCCACTTTAG
LG1	ref-10481_10	49.099	Chr7	70265960	GCTGGAAGTGAATTAACGCTGAACAAAACCCCCAATTCAGCAGAAGCCAAAGAAAGAAGTTTTAAACAAAAATTGGAGAGAAAGAACGAAAAAAAGAAGCGCCGACATTCACAGATAAAGCTGAATCCAGAGCCCTTTTCACTCTCTTTCTCTCCCTCTCATTCCTCCCTATCATATAGCCGGTCCTTTACTGTGCTATAGCTGGAATTA[A/G]GAGTGTGGTGCTTCCAATACCTTTTTTGTAGCGAGGTTAATTACTGGCGAGGCTTGAGAAAATTACATTCTCAAAATAAAGTATTATGATTGGCTCTCCACTGGCTATTTTAAGTCGTGCATGCTTTTTCATGCAAGATTGCTAGGAAACCAGATTTGCGGACTTGTCCAATATCAAGTAACCTCATTTACAGTCCCTATTAAACGCATCATTCTGACATG
LG1	ref-7283_8	49.568			TTCAACT[C/G]ATCGACTGATGTGCCGTCATGTTA
LG1	ref-7283_16	49.812			TCAAAATCCCCCACAGATCATTTATTATTGCTTGTCAAATTTGCCCCTATTGGATTGTCAGTAATGGTGTTCAGCCTTACATTGTTCAAACCGGAGTCGACACTGATGGAGAGACTCAGGAAGAAGTTACAACTTTTAGAATGAAACTGGATGTTAATGAATGGCTAGTGGATAAATTTATGTAGTTGCTGTGGAGTTGATTCAACTGATCGACT[A/G]ATGTGCCGTCATGTTAATCTTTTGTGCAAATCCAGCGTTGAATTGACCCTCGTTTGTAAAGCAGTCTGACGTAAAATGATGCCATGTTAACAACACTCTACTACAACAACTCTTCCTCTTCTTTAAAACAGCCCAACATGGCCTCACCCCCTTTGTTGCATGTTCCCGGGGGCAGGGTTTATGTAAATTTTGATGTCATCAACTCAGGAAGAAGCT
LG1	ref-28244	49.954			CTACGCACTACGAGACATATGCAAGACATGG
LG1	ref-17765_19	50.137			CTTTACAGAGAATGATCTATTTAATAGATAAGACTTGAACCACTAGAACCACCAGCACTTGCAGGTTCACCACCTGATCTCGAAAGGGAGTGGTAGGAACCTTGGGCACATATCCAGGCCGGGGTCTCAGGATAACGTGAGAGTCACCGGGCCCGAATTCCAGGCACGATTCGTCAACGAAAATGCGTGCAGGTCCCCTACCCAATGCAGCGAGGCCA[A/G]TGCAACCAGGAGGACGGTCTTTAGGGACAGTACTTTTAACTCGCTGATTGCAAAGGCTCGAAGGGACGACCCCGGAGAGATGTAAGAACCAGGGTCAGATCCCAAGAGGGTACAGAGGGGGGCCGGGGAGGATTCAGTCTCCTCGCCCCCCTCAAGAACCTGACGATCAGATCGTGCTTCCCTAGTTTGAAAATGGTTTTAGCTGATAGAAAG

LG1	ref-48026	50.312			AACCGTTCGACGAACAATCTGCAAGCGGTTT
LG1	ref-17006_19	50.439			CAAACAAAGGAGACATATTTCTGTGCAAATCATCTCTGTCTGCCCATACAGCTGAAAGTAAATGTAATAACGCCATAAAGGTTCCCAGGTTTGGCAGACCCGTATAGTACA<br>TTAACATCATCTCCAAAACAAACATCAGACATCCTGTACATATCAAGTCCATGCTGCAGCTGTCAGGTTAACTTCTGCACATGCAGATCACCATCTTCTCGAGCCTC[A/C]T<br>GCATCTTCTCAGGGTGAAGCCGATCACTTTCTCCAGCCTGATGGACCATAGTGTCAGCTGACTGCATTGTTTTTCCTTACTTACTGTCTGTGTGTGCATCTCAATCAGCTCTG<br>AGCTCTTGAATCAGTATGTTGTGTACAGAAATTTTGCGCCTGAGACGGTCCTGATCAGTGCCCTAACTACTGAACTAGGGAGCTGATTGAGACGCACCC
LG1	ref-33076_7	50.534			CGTACGCCTATGTGTCTATGAGACTGTAGTCGACAAGTTTTATTAAACCATTGTTGAAAGAGCTGATAATAAAGGTGTTAAGCAAGGTAAAATGTTTAAGGTAAAATGGAA<br>AATGTAAAATGCAATAAATGCAGGGTATTGAAGTTATTAAGTTACTGATGTACATTTTTTTTTC[A/G]TAGCACCCCCTTTGAACCCCCTTCACCCTCTGCTCTTTCTGATG<br>GAATGTAAATTAGTGAAGATTGGCCACTGTGCATATAGGTGTGAAACCTCCAAAACTATATTGGCCATTGTTTCAGTTTCATTGTCTAACCCCTGTAGTTTTCAAGTTTTAG<br>CACGTTTACAGGACAAGCATGTATAAAATCATTTTAA
LG1	ref-33775	50.534			CCTTCTTCGGCGATTTCTTTGCTGCTGCAGT
LG1	ref-716	50.659			CCAAAGGGTATGCGGGGATACCCACAGTGGAGCGCTCTGTTGAGATGCAACACTCCACCTGGCGTGGCGATCCCAAGCACCCATCCAAAGCCTGCAAGTTCTAATCCACG<br>CTTTGGGCAAGGCTTACAGAGCTGCAGATCAGGCCGCTTCCGCTTTGCATGCCATGGCCATTCTGCAAGTTCATCAGGCCAAAGTGCTTAAACAACTGCACGAGGGCAGTG<br>CTGACCCAGGGCTTATGCAGGAAGTGCGCACTGCTACTGCAAGTTAGGTTACTGCGTGGTCCCTGGGCCAGGTGATGTCCACTCTAGTGGTCCAGGAACGCCACCTATGGC<br>TTAACCTGGCAGATATGAGAGAGTCTGAGCAGACCCGTTTCCTCAACTCCCCGATCTCCCAGGCTGGCCTCTTCGGCGACACGGTCGAGAATTTTGCCC
LG1	ref-60720	50.876			TATATGGTGCCCAAGTTCCTTTCGGTGCCCTCAGGAGGCTGTTCTGTCAACCCTGCCACCTTGTGTGCTTCGGGGCACAGTGTTCTCTGGTGAGCTCTTATCTCAGGGTCCGC<br>CCGGAAACGTAGCAGCACTGGGATGCTTATCCCCTTTGAGGAGGGTCCCATAGCAATCAGTTTGTCTGTTCCCTGCCGGTTTGCCACTTCAGGGCACCGAATTGGTCGCTTG<br>AGTAATACTGGAGACCAGTCTTGAAAGGCTGGTTCTCTGGGCCTCCAGGGAGTAAGTGGTTCACATATCATCATACAGTGCCCACTTCTCCTTGGTGCCCTCAGGAGACTG<br>GTTTGCTAACCGCGCCACCTCTGGTGCTTCAGGACACAGTGGTCTCCTGCGAGTTCAGGTCTCAGTGTCTGCCCAGTGACGTTGCGGCACTGGGA
LG1	ref-9369_14	51.012			TGATGGTCTCGCGCCATTTCCGGAACCATGTTTCCCTGAGACGGCAGTTGCCGAAGCAACAGAGACTGCTGCCCGTTCTCGCTTTAGCTTCACTTTTTTGCGCTTCGAAAGC<br>AAACTCGGACTTTGTGGTGTCTGGCCTGGGGAGGATGACGAAGAGGACGAGGAATCACTACAGTTGGTGGTGCTCGTAGACGAAGGGGCACCTACTACCGA[C/T]GTCCCT<br>GCACCTGTACTGAGTGGCAGAGTCACTTCAGCGACTGCCAGGCACACCTCCATCAGGGCATGGAAGTATGTCGAGAAGCGGCTCCGCTCGCCAGACAGCGCAAGCGCTCC<br>ACCACTACCCGTTGCGGAAGTGCAGGAACCGCCGCTAACAAACCCCCAACTTTGGGTCTCGTGGTCATAAAGCTTTCGTAGTTCAGTTTGAAGAACCATGAG
LG1	ref-2454_27	51.043			TGAATGAGTATAAATTCTATGAATGAACTGAACACAAATCAAGGAGCTGCGTTCCTGTTGCCTTTTCCATACTTTCCACTATTTAAATATATTTAAATAGCCTATTTATTTAT<br>CGGATCACGAAAGTTCCCCAAGCTGGGATTCGAACTTGTGTCATCCGAAGCACAACTGCACAAGATGCACTTCCATCGAGACT[C/T]TTGGCTCAGAAAGTTAGAAGATAT<br>TTAAATAAATGTTATAATCATAAAAAATAAAAATAAAAATCAGTTCAAGGCATATTTTAGTGATATCTACAAAGAAAACAGATATATCTGTAATAGACCATAGACAAAAC<br>GGTCTTCTCAAGTAATTCTCAAGTAAAACTTCCGGGTAA
LG1	ref-18055	51.302			CGGCCGATACCGATACTAGTGCATCCCTAAA
LG1	ref-60496_10	51.586			ACACAGCTATTGACCAATCAGAATCAAGGACTGGAACTAACTGTTTTATAAAAACATTCTGAACACCAAACTGAACCAAAGTCTTAGCATTATTTGAAAAAGACCAGCGG<br>GTGTGTTTGATTAGTGATGGAGCTGAAACTTCCAGAACTGCTATTGAAGACATCAACTGGATAATCACGAACATTGAATGCACAACTGGAAACTCAAGCG[G/T]GATGGTA<br>GTGCTCAGATTCCAGAAGGTTCCAGAGTTTCTCTAAACTCCAATCCCTCTCATTTTTCTTCAGCGCAATTGCTTGATTTCTTTCTTCCTAGGGACAAACTGCTTTTATTTATTT<br>CTGGGGTGGTGATAAAATATCCATGCTGCTCACCGCGGGCCTGGTGGCCGGCAGTGGAGCTGAAATTAGATATCTATAATTCAGTTCACACTTCAGACGG
LG1	ref-57267_2	51.913			C[A/G]GACGTATTCGAATGGATTGCTTTCGTAGTG
LG1	ref-30392_5	51.951			ATGAGAGGTGGCTTTATGTGCACACTTTGGATGTGAGCGCGGGAATGAAAATTATGCGCAATCCTGCACCGAAATTCAGACCAGCCTTTCTTATGATTTGCATTTTTACTAC<br>CCATTTGTGTGTGCTCTTTTGTTGTTATTAAAGACATCAGAGATTGGTGGTACACTCAAAAAAATGATACGTTGAAAATGTTTGAAGGAATT[C/T]TTACACGACACCCCTG<br>CTCTCACTAGACGGCTCCCCAGTTAGGAATTTGGTCTAGGGTTTTGGCTATGAGATGAAGGTCTGGCTTTGACGAGTAAATATAACAATTCACTGGTACTTTATTAGGAAG<br>ATCCCCAAACGAAAATATGAAAAACAGCTAAATAATTCCAACTAAAAAAACCCCATTGTCAAAAGGATAAAATATATAAACTACCAATCAAACGTTTGGG
LG1	ref-33497_25	51.955	Chr7	7372142	CTGTCAACATGAGGAGGTTAAACAACAAAGTTGTGTTTGTAATTTATTAGATTTTTTTAATGTATTTTATAATTTATAAGATATTCAGTTTAAATAAACGAAACAAATTCTA<br>ATCATATAAAAATAAATACATTTTATTTTACAATACAACACAAATGTTTTGTAACTTTGCAAAAGGCTTCGCTTCCAAAAAAATGCACAACTTTGAAGCGACTGCTCTGCCC<br>C[C/T]TGTGCTTAGGATGCTTCATTGCTTATGTGAAATGATAAACAAAAAAAAATAAATTATTTAATACTTCAAACTGCCCCACTAATCGATCTTTCAAAACGTTTTTATAAT<br>CATAACCTGATGTCTTTCTCCCTCTTAGATTAAAACCATCCTGAGTGGCTTTATTATCCGAGGGTATCTGGGAAAGTGGACCCTGATGATAAAGACCA

LG1	ref-51675	52.297			GAATGGGCAGGCGAACTCGTGAAATGTCCTCATCTGTCAACATGATGCTGGTGAATCACAGAGATGGTTCCACATCGCTATGATCAGTTGCTTTCTGAACTGCCGCTGTCAATTTTGTTGTATGTTAAAGCACAGCACATACTATTTATATATTCATCTAATTAACGTAACTCTCAGCAGGATGGACGTCGGGATGTTCGCAGCAAAGCGATTTCCAACTTTTTACTTCTAAGCGAAGAACGGAAAAGAACTTCAACTTGAGTTGTATTGGGGACAGTTACTTTTAAAAGTAATGCTTTACAATACTGTGTTACTACCTTAAAAAGTTATTAAACTGCGGAACTTTTTATGGAAAGTAATGCGTTACAT
LG1	ref-69537	52.321			CCTTGACCGCCGACAGCCCTGCATATTGTGG
LG1	ref-50108	52.811			AATTTTCCCTCGAATCAGTTGCCGTGTGACA
LG1	ref-19190_32	52.998	Chr7	15758300	CTTAACCTTTATCATACCTCTGTCCTCTTCACAAAGCACCAGTAAATCTAGTAAGGGATTTGCATCCTAAACATGGAGCGCCCCCCTCATTTCACATTCTCAATGGAGACCCCAGATCAAAGTTCAGTCAGTCAAAACCCATCCCGTAACGTTAAAGGGGTCGGCACGACCTGCCCACTAAACGAGGATGAGTGCCAAGCTGTCAATCTCCGAAGGTGGTGCTGGAACACA[C/T]AAGAGTCGCTGATCCAGATCAGGAGAAGATTAGGGGTTCACCTCACTGCTCATTAGAAACTCTGGAGCGAAAGCCAGACCTCAGGAAAACATCTTCCTGTGTGCAAGTGTGTTTTTCTAAGCTCAGTCTTGTCAGAAAGTCATTATGTTCTCAATGGCTTAACGAATCATCTGGGTTTCTTACAGTTCAACAATGGTTAA
LG1	ref-19472	53.345			CCGGCGTTGCCGACGCCTGTGCTCAATCACC
LG1	ref-13703_6	53.806	Chr7	20534720	GACAGTCAGACATGGCTGTTGAGTCACAAACCAGTATTGGCACTGGCGACAGTAGGAGTTTTGTCCTTGATAAGGAACCTATTTTATCCGAGGAGAAGGAACCTTCGCAAAGTTCTGCCGTAATGAGTGAGAACTCGATCTCCAGCAGTTCCATGTCAGATGGAGACGAGACACAGCTGGCCAGGAGCTGGCTGGACTATGAGCA[A/G]GATTCGACTCTCTTGCAGACATGGTATAAGCTGGATGAAAACACAATTCCTGCTTACTTCCTGTCAGGTAAGTGTATGCCTTAGTGACGTAATCCTGCATATCACATAAGTATCTTTTCACTCTGACTCACTATTTTATATTACTTGCATGATCCTTCAGTGAATCGTACCAAAGCACTGTTCGCCAGAGGAGAACTGGCCCCTGACTAAGCCTGGTTTCTCCCAA
LG1	ref-63537	53.962			CCAAAATAAACGATGAAACTGCAACCGAAAC
LG1	ref-33465	54.687			AAATAAAAGAAAATTCTGCCATCATTCACTCACCTTTATATAGAGATGCACCGGTTGACCGGTTTGTTTTATTTTGACTGATCTCTGTTCTGGACTTGCACACAAACTGCCTTGCAATAACTTCCCAAAATGTTGTTTGTGTGGAAATAGTCTGTAAACAGGACATTAACACAGGCCAGAGACGGGGCGCGCTGAATACAGATGCAAAGACGAGAGGAGAGCACAAATACTGTACCAGGTACAGCAAGCTCACTGTTTGCGATGCTCAAAATATTGGAAGAAAGAAAAAATATAAATATTGAATTGCGGGTGGGGGATTATATGAATGTATCTCTAAAGGGAACTGACTGTCTTCAGAAAAGTTTCCATGGCATTTTCTTTTCCTCTACCTTCATATAATGCCTATTAAAGTAGTGTTTATAAGCACAGTT
LG1	ref-53975	54.9	Chr7	9613479	ACAGCATCTGAGCACACAGCACATCGTCTCTGAGGGCCGACAGGTGCAGCGGCGTCTGTCCGTCCTCCAGCCGGCCCAGGTTAGTGTCCGCTCCCCGCTGCAGGAACATGCGACAGTACGAGTGGTTGCCCTTAATGACGGCGTAACGCAACAAGAAGCCGTTCTGAATGTCTATGTTGGCGTTGTGATCGAGCAGAATGCGAACGCAGCTGGAACGCTCGCGGATAATCGCCAGCTGAAGCGGCGTTGTGCCTTTGTCGCTGAGCGGGTCCACCTCGGCTTTGAACTCTAAGAGGAGACGCACGAAGGAGTCACGGCCGTAGTGGGCCGCGACGTGCAAAGGGGTCCAGCCATCGTTGCTCTTGGCGT
LG1	ref-28129_28	55.021			CGGCGCAATCAAAATGCAGTCTTTGCAAGGGACTTGCTCAACGGTCCGTGGCCAGGTCAGATTACGTCAGAGTTTGTTGTCGTTTGTTGAAAAATCATACCACACTGCTCAGACATTCCCCGACCCAACAAACGCAGATTAAACATGTTCAGTCAGGTGAAAACAAACTCCGAACAACGGCAACAGACTCGACACG[G/T]GTATCACGCTAGCTGTGTCTCATTTCGGAGGCTGCGTCCTGATCCAACAAACTCCAACAGACGTTAATTAAAATTAATCCACTACATCTTCAGTGGCTAATTTGTCGAGAGTACAGGACGACTGCTATGTTCATTCTTATATCCAAATACAAAACGCCTCAGTTGCTTTGGAG
LG1	ref-49163	55.44	Chr7	46735315	GAAATTACTTATTCATAAGTACCTTACTCAGAAAAAAGGCTATTTCAGACACAGCCAAAGTTCTTCCTGTCATTCCAATTCAACTTCATGTAGGTGTGGCCAATTCAGTTCAAATTCACACTCATAAATTGAAAGGGAGCAATTTCTTCAGTTCTGAATTTTGCACAACCATTGTGTTAGACTGTCAAATGGTCAAGGGCCCTGTAAATGCGATTCATCTGCCATCATACCTCTCTCTCTCCACAGGTCTAATTTCACTGTTGAATGAGACAGAGCTTAATGAACAGGCACTGCTAAAACATTTCATCCCCCCCACCCATCTTTTTTTTTTTTTTTTCTCCCCAATAACCTCTCTAACCTGAGGGAAAGCACACCTTGAGCTTATTCAGCAAAAGTCAAATGCCAGCACCTCAGTCTCTTTTGCAGAGCT
LG1	ref-44344	55.517			CGGAACAAGACGACAAATGTGCTGTTAGTCA
LG1	ref-6038_26	56.018	Chr7	63473983	GCCTAAAAGCAAAGTAGTTATGCCTCCACTACTTTTGAAATGTAAAACAGAAACAGCCACAAAACTAACACCTTAAAATCATGGATTTTCAAAAGTGTTACAGTGTTACAGTGTTTTTTAAAAGTGAAAAATCCATTAATTTTATTATTGGCTTTCACAGTCTGTCCATTGCTCTAACAGCAAGCCCTTCGGAA[A/T]GGACACATTAATGCAACTCGACCTGACTTGTTTTGATTTAAAACATCTAACCAGACATTTGTCTCGAGTCTTTACTAGAAACATAAACTGAAGGTTAGGATTTGAAGCACGACTCTGACCTGAAAACCAATGACTCTGACCCATGTCTGAGGACTATCAATAAGGAACTAGCTTA
LG1	ref-50178	56.031			AAGAAGACTGAGATATGTGAAAGACACATACTATTTAGACTTAATCATCACTGACAAAGCATGTGATATTTCCAGTCTGGTTTCAGACTGCTTCTGTAATATAATACTCATGACAAAAGTCAAGCTCACAATCATCTGGAACGTTTATTTACTGTATAATACCATTTATCAACCAACATGCAGCCCACTCATTCATTGAGGTTGTTGAGGAGGTGTTATTGGTGTTATGTGACTTCAGCTCCTGTTGCCATTTTGATCGGAAAGCTCTTTGTCTCATATTTTATTTACTTTCCTACTGTGTGGGAGATTCTGGGAGTAACATTATATAATTATGACAGAGCTTGCTTGATCTTGTAAATGAAAGCATTT

LG1	ref-55265_27	56.156			GCCCGAGACTCGAGCTTGATGCTCGA[C/T]TGCCT
LG1	ref-32953	56.238	Chr7	46267964	ACACATACACGCACACAGGCTGATTTTAAGAGAATCAAAATTGTTTTGTCTTACACATTGAACTGCAGTTCATAATTTTCATTTCTGCTTGGATGAAATATAGTGGACTATATGTATCTGGAAAAACACACCACACAAATTCACTCTTCGTCATACATTCAAACTCCACCCTGAACTGTTGCAAATGCACTCTTCAAAACATTACTGCACGGCTGGAGTGCTGCTCTCCCTGGCAGTTTGCTTGGCGGTGCTGGTGGGTGGGATAATGGCCCGGGAGGACCGTGATTATGAGAGGGGAGGGGCCAAGAGAACACTTCCTCTTGCCGCGACATCATTTTTATTATCCGCTGCCCATCAGAGGTCCTTGAGAAACATGTACTTTCAGCCCAGGCAATCATTCATCTGGGGTCCTGCTCCAAGAGACATGGAG
LG1	ref-31235	56.409	Chr7	47825645	CTTTCTATCGCAGCTAAATGCAAAATGCCACCGTCATCCCAGGCCCCCCAAAAATCCCTAATTGACAGCTTTCAAAAACTCAACCTGGATATTCATTGACCTGAAAGATGAACAAAACCAAACAAGACTCCTCAATTGCTCAGTTGGGTATTGTGAAATTAAATCAGTAACGTTGATGTGCACATTGTTCGTTTTAACACTCCATGGCGAACTTGCATTAACACATTAACCTTCCAGTCACGTCTTATCAACACTCTTCAGCGTCAGAAAGAAGTTTCAACTCTAAACTATCGTAAGGCTGTTCCAGATGGAAACTCAATACAAAGAACAAAGCATCCATGAACAAAGTTAGCGAGAGTTTAAAGTCAT
LG1	ref-1263_17	56.918			ATGCTCTGCACTCATTGTGTTATGTCTTCCGGTTTGAATTTCCACAGCATGAAGGTAAGATTTTATGGATGTATGAATGAGCCATTCATTTTGAAATCTTCCCGGTCTACTGACATTTGTTATGACATATTCTTCAAACATATTCTTCAAACATTACAATTCCCATGATAACTTAAATGAAAATCCACCTCTCTAGCTCTCAGCACTCTTCGACAG[C/T]GATGCCATAGGAGCTACCACAAAAACGGAAGTTCAAACACAATATTCTAAAATGGCTGAGGGCTGGTTTCTCTGGCACAAAAGGTCTGTAGAGGTTATTTTTCATTTTAATTCAATAATTAAAAAGGCTTGCTTAAAATGTTTGTTCTTTATTTTTCAGTGTATTTTAATATACACATATTGGTGTGGATGCTTGAAGTTTGTTAATGGCTACCA
LG1	ref-43579	57.104			AAAACATTATTTTCTATTTATCAGCAAATAACTCACCAATACTTTGGAGTTAATCGAATTTACATGCATTGTAAAGTTCAGACTCTAAGCTTTCAAACAACACCTACTTTTTGTTGATCAAGGTAGTAGTTTTGAAAAATATGATTATTCATTTTTTCATTGCTAGGTGATGCCCGCGGGCGGTATACTCGGGTTTATAGAGATTACTCAGCGCTCCTTAAGCTGAGTAACTAAGCTTTAAAATGATACCTATTTTGTGTTATTCTATGTTGGAAAACTCAATTTGTGCAGACTCATTAAAGGGTGAAAACAAGGGAGGGATGATCTGAATGTGTAAAGTCTTTGATTTTGTATGCAAAAAGAATTATT
LG1	ref-30056	57.226			AACTGGCAGGAAATAACCAATTTGACTGCTTTTCAACCCTCCTCAGAGGTTGCGAGCCTGACGCAATGTTTTGTGGTGGACCTTGAGTCAAAAGCTCGCTGGAGACCGCTGCGCCCTGAGGCACAGAAGATGGCAGGATTGGCAGAATGGCCCCCTGAGGGCACCGAAGTGGCGTGGGCACCGTATAATGAGGTGTACACCACTCCACCCTGAAGGGGCTGCCCTCGCAAGTCTGACACTGGTGGCATCAGGACTTCTTCTTAGCTGAAGCCTTCTTTGCGAGAAGAACGGTTCTCAGATCCGCCTTCTGCTTAGGAGGCTTCGGCGAGGAAGATACTGCTTAAACGCTTCTGCCTGGAACTTTCCTGGAACCTCTCGATGACATTATTCATGGCGTCGCCAAAGAGACCAGGCAGTGAAAGCGGAGCATC
LG1	ref-63127	57.324			CTTTAAGCAACGAGAGGGTTGCATTGGCCCC
LG1	ref-10621	57.363			CAGTCTCCTACGAAGTTGATGCAATAAACGC
LG1	ref-69758	57.662			TGATTGTGACCGAATCAGTTGCATATGGGGC
LG1	ref-71191	57.909			CCAAGCAGTACGACTCCACTGCTCACCAGAG
LG1	ref-57834_15	58.387			TTCACTGCTTCGAA[A/G]CATTTGCAACACCACAT
LG1	ref-57834_16	58.487			TTCACTGCTTCGAAG[C/G]ATTTGCAACACCACAT
LG1	ref-35702_32	58.857	Chr16	12000005	GACAAGGCAGCCACTGGATTAACCCCGTTTCAATGCATACTTGGATTTCAACCACCTCTCTTCCCCTGGTCTGGAGAACCCACTGACGTACCCGCAGTCAACGACTGGCTGAACAGGAGTGAAGCCACCTGGAATCGAGCTCATACTCACCTCCAGCATGCTGTACGCCGTCTCAAAGTGCAAGCTGATCGTCATCGTCGTC[C/T]TGGTCCAGAATACCGTCCTGGTCAGTGGGTGTGGCTCTCGACTAGAGACTTATGTCTCAAACTCCCTTGCAAGAAACTTAGTCCAAGGTACGTGGGTCCATTCCAAATAACACGTCAAATTACACCGGTCTCATTCCGTCTTGCATTACCTAACCATTTCCGTATCTC
LG1	ref-24121_28	59.341			TTCAGCCTCCTAGAACCTCTCAAAAATCACACAACTAAGTTGTTTCTCCCCACCGACTGGCCAGCAATAGGGGCATGAAACGCTGCTATGTGGAGGGTGTGAGACCTTTGTCCAGACGCTCCTGGAGAAATGACAATATCGAAGATATGTCACACTCAATGGGGTCCTCGTTACGAGTGGCACACCAGTCGACGAA[A/G]ACCGACCATTTTTGGGCATAGAGGCATCTAGTAGCACGTCCTCGGGGAGGTTGACAGGCTCTCATCGAGAGACCAGAGGTGCTGGGCCCACAGATCTGGCTGGGGGTGCCAAATCGTCCTGTTCACCTGAGAGAGGAGGTCCTGTCTCAGGGGGATCGGCCACAGGGCTTTTG
LG1	ref-4484	59.825			CTGTGTCCGTCGAAAAAAGTGCCCCCCCCTC
LG1	ref-67331	60.264			CAAGACAAGGCGATGTAGCTGCTGTGGAAAA
LG1	ref-6297	60.363	Chr7	31559990	AATTACTTTAATTATTTATATTGTTATACATTTTAAAAGTAGCCTAATTATTTTCGACATGAGACTTGGAAGAAACTTTTATTTTGGAACGAAAGGAGCTTTAATATTTGCCCGGAAGTATTATTCTGCAATCGCATAATCGCCGTAGAAGAACATCGGATCTGTATATGATAAGACACGCACGAGAGTCGTGTAAATACTCTAGAGATCAGTAAACCAGCATTGACTTCGTGCATGTCCTGTGTTTCATTGTGATTTCCTATCAGGTTTTCCGGACACGTTATTGTTGCATCAGATAGAGGAAAATGGCAAACACGGGAGATATAGGTAATATT

					TCTCTATTCAGTTTTCTTGCGATGTAACAGTCAT
LG1	ref-46424	61.437	Chr7	19313621	TTTAGACAGTCTTATTTGTATCATACAATCTATTAAAACGATGGCATCCTACAGTCCTGCACCCACAGAAGAGCCTTCCTCCGAGGTATCGAATGGAGACCTGAAGGCAAGCAACTCGCAGGAGTCCATGAAACTGAAAAAAGAAATATCTTTGCTCAATGGCGTGTGTCTCATCGTGGGAAACATGATCGGCTCCGGGATCTTCGTCTCACCCAAAGGCGTACTGATGTATAGCGGCTCCTACGGGTTATCTCTGGTAGTGTGGACAATCGGTGGGATCTTCTCTGTGTTTGGTGCGCTCTGCTATGCCGAGCTCGGAACCACCATCACCAAGTCGGGCGCCAGCTACGCCTACATCCTGGAGGCCTT
LG1	ref-27945_7	61.638			CCTTAAAAATCCTTTACTATTTGGAGTGTCATTTAAACAGTCCAGTTCCTTTCATATAATTAATTTCCTGCTTTGTATTTTTGTCTTGTTTTCCAGTACAAATATCTAAACATTCTTAAAATAAGATACATTTTTTAAAATAAGATAAGAATGCTTTCGCGTTCGCTCACAAAAGTTTTGCATTCCCCCGAGTAAGTTTGCATTCCC[C/T]CGCGAAACCTTTGCATTCCCCAGAGAAACTTTGCATTTGCTCGCAAAAAACTCAAAAGTTTCAACATTTCTCAGGGGAACGCAAAAGCGTTGACTTATATTTTTCCTCCACCATGTCCCTTTAGATGCTCCATACTTATCTGTTCAACTTTTTACTAAAAACATGTTAATTGCTCATCTGTAGAGTATCAGAGTCCGTTTGTGTTAGAGCAGTGCACTTATAGC
LG1	ref-71881	61.84			CTACATTATATGCCACACTAGACAGTGCATTTCCTTCTCCTTCCTCGGTATTATCTGAGAAGGTCAAAACAAATACTTATATTCACTACTACATTATATGCCACACTTGACAGTGCATTTCCTTCTCCTTCCTCCTGTAAATATTGCTCATTTTGTTTTGTTTATCAAGCCAACCCTGAGCATTTAGATCACATAAGATTTAGAATAAATAAAGTTCATAACATACACCTCAGCCCATTTTGTCAGAGGATGCTCTGCTGCTGCTTTGGTTTTGTGCACAAGATGCAGCATCAGAGCAGCCTTGAACTTTGCTATTGTCATCACAGAATATATGAAAGGAAAGAAAAGAGTAAATTCTTGAAAAAAGAA
LG1	ref-15874	61.899			TGGACTCCCACGATACACGTGCTTGTGACAG
LG1	ref-25546_4	62.039			ACG[C/T]ACCTATCGACTGAGGTGCCTAACGTAAT
LG1	ref-1136_31	62.845			CTGATTGTCTCGATGGAAGTGCTTCTGTAC[A/G]T
LG1	ref-16037_16	63.718			AACATAGCATAATCAGATGCAACTTTATTTTTAGTAACGGTAATACAGAATTTTCTCTATCATACAATACGTTTTAAAATTAAAAGCATGCCATTTATCAACAAGCCATCCAGCATTTAATGTGGTATTCTAAAAATCGTTTTATCTTACTGCAGTGTGTCTCAAACAAGTGTCTCGCAGCACTC[C/T]TATCGTATCCATGGCATTTTAGCTTGTTTTTTTAACTCTTGTCTTTGAGCTCTTTTACCGTGATTCGTCTTTCACGTGATCCGTACCTAAGCTCTTCGCACCACAAGTACAATTCTGTACCAGCTAAGCTACCGAGCAACCTTAAAAAGCAAAACTAAAACTTAGTAAAACTGAACAAATGGAG
LG1	ref-44268_25	63.999	Chr4	14695866	CAGTGCAGTGCTTCCGGATTCTACATCAGAACGCCAGCTCAGTATTGGCCGATGCTATTCATGTGAGCAAAAAGTATTCTTGATGCTTCATAACATTAAGGTTGAACCACTGTAGTCACATTGACTATTTTAACGATGTCTTTACTACATTTTTGGCCCTTGAAAGTGGTAATAAGGTTGCAGTCTTATCGGA[A/C]GCCAAAAAGCTCACGGATTTCATCAAAAATATCTTAATTTGTGTTCCAAATATGAACTAAGGATTGAACGACATGAGAGTAATTAATGACAGAATTTTCATTTTTGGGTGAAATAACCCTTTAATATTTTTGTGAACACCTTGTTTGAAGAATAGAAGGAAAATCATTTATTTGAA
LG1	ref-10254_30	64.338	Chr7	35416648	GTAAATCTATTCAGTCGGGACCTCACTACACCACTCATTTGCTTTACTCAAGCAACTAGAATGAGAATAGCAATAAAAAGCACTTGCAAAAAAGGGAGAGACACCACACACCAAGCAATATTGTTTAAGAATTAAACGCCCCAAATATCTTCGGGAAATAGGAGTGTTTTGTCATGGATGCAGCCTAATCGCTTAAGT[A/G]ATGGTGGTTTGAGAGGGAATGTTTTCATGTTTTAAGAGGCTTGGTTCCACTTTCCTCCACCACAGAAATCATTCTACACAGGAGCTTCTTTCCCTCTACTCTCTCTCTCTCTCTCTCTCTCTCTCTCTCTCCATGGATGTATGGATGAGTATATGACTCAAATGTAGAGCT
LG1	ref-37914_26	64.537	Chr7	30441360	ACCACAGTAAACCATCCTGCTGAGTCTCATGGCTCATTCCACCCTGTCTGTACAATTACATTAGATGTTAGAACTGTACTGATATTAGATCAGTTTGAAAGGATGATGTCAACAACCACTAGTGCCATAGACCTGTCCGACTGGCCCTTCCATTCCCTGTATAGGGTAGTGCATTTAAGCGCATTCTCCTCATATTTATGTAGGTGTGCTCGAGGGTGTTGCTCA[G/T]TCAGGCCTCTTGATTCTTAAAGATCAGCTTAAAATAGACAGCAGTGAAATAGCTTGAGGTTACTTGGGCTTCGCTGTTGTTTAATCAAGTTCCCACTTGCCTGACTTTGATTAATGACCTCATTACAAGCTTCTCATGTGGGCTGGTAATGTGGCCCCTATGTTGTGTTGTATCTCTGTGAATCTGTGGAAAATTTGTTTAGACGG
LG1	ref-61914	65.004			GGCCTTCAGACGATACTCCTGCCGAATGTGG
LG1	ref-33517	65.273			AGGTATGGTACGACACTCATGCCAAAGAAAG
LG1	ref-10722	65.5	Chr7	60724347	AGGCACAAAAATGGAACAGTTGTTTATTGAATTCACCTGTTTGCTCTTTCCTCTCTGTCTCTGAGAACAGAGGTTATTTTTGGCCGACTTTGTCGACTCTCATATTTCCTTTTTCAATTCACTCAATCACAAAAGGGGACAATAGCTGAGCGAAATAATGAGACATAGGAGCATGAAAACACATGCACACACAGCTAATCAGTGGAACAGTGAAGACTGTGCTTGCTAATGAGTTTCCACTGGACAAACCTGGTAGTTTGACAAAGAGTGTACAGAAACACACACTTACACACACACACACACATTCATATAACATGGAGTTAAAGCATGTACAACTTATTTTGGCCGACTCTCTTTATCTTTGCGTGCACACACACACACACACACACACATTACTGCCAAGGTGTTCATCAGTTTAATTAGCCTTATAC
LG1	ref-62472	66.321			GCATGATCTTACATTATTTCCTGTATGGCTTCTGGCTTGTGTTTGGTTGTGAAACGTAATCTGTAGACCAGACAGAGTTGTTGGCGATTCTTCCTATTGAAATGTTGTGTAAGGTGGGACCCCGTCACTGATCCGTTGTGCACACAGCAGCAACTAAATAGTATACGCAGACAGTCATGCAGTGTGAGAAGAACAGTGATTTGATTACTTGGAAAATCGTGCAGTGTGTACCCAGCATTATTAAAAACAGATTTTTGGGTGAACTGTTCCTTTAAGCAAATTCTTTGTACATTCTTCTCACTGTAATCACTTGGCATTTACACTCAGGCACGGGCC

					TACATGCTTGTGCTTGTTATAAAGACTAGTATTGCAGCATTTTTGCAGGTGAAAGAGGGACATTTTTTCACATAAATAAATCCTGTTTATGGCTA
LG1	ref-67362_15	66.396	Chr7	52639416	AAATCCCAACCTTGAGCTGTGGAAATGGGTCATGCTCTGTTGTACGCCTCTATACAGCCCTGACCAAGCAGGAGAGGCACTATCAAACCTACTTTAAGATTCTAAAGATAT<br>CCAACACCAAGGGAACTCTATTTATAACCTCAAGAAAAAAAGAATTCAAGGTCAGTTGTGCCAAAGACTATTCTTATGCAGAGTCTAGTGACTTGAGAACAAGC[A/C]TTA<br>TGCTTACACTGTTTAGGAGCACTCATACCCCTCCAACAGCAGCACTGATCACCTTATGAACATCTGAGCAACAAATATATACTGATTACACTTTCACTACTGATAATTTTCA<br>TTAGTCAGATACAAATATTATATTGAATTAATATTAACTGCATCACAATGTAACAGTAGGCCTAATATTTTGGCGGATAAAGGGTTGCAAACTCATCAATG
LG1	ref-29806_10	67.475			AAATTTTGTTCCGTTATTATGCTTGTTGTCATCGGTTGACGTTGTACAAGCAGAATGTGGTGGTTGGTCGGTGTTGTATTTGTCCCGCCCCTCCTCCACTGTGATTGGACGGC<br>TGGGTACAAAGTGACAGTGATGAGCGCTCCATTTTACTCAAAAGTTCTTCAACTCTTGCCGACCGGTAAAAAACCCAGAGCGTTCAGCGCTTCAGC[A/G]CGAAATAGATG<br>CTAAGCACCTCGTTACGCTCCTGCCTTTTTACAACAATTGGAAAAATTGGAAAAAACACTTTGGTTGGCACATGGCCTTTAAGTGGGACCTGAAGTAGCCTGAAAAGTTTG<br>GACAATATTTTATCGAGACATCCAAATTCTTTAGCTTTACAGTCTCTGAGGGGCCCGATTCCAGCTGTAGTCTAAATGAAGGGCAAGCAGCAAATCTCTA
LG1	ref-61849	68.336			GTCATTTTTCTATAAAACATAGTAACGGTTGATCTTTTTAAGGTGACTTCTGTTTTTGTCCTCGGTTTAACAATTTTTTTCTAGGTGAAAATATTTCTCATGACAAAACCTTTA<br>AAAGCTATAAACACTGATGTTATCAAATGTATCAAATGTTAAACTTTATTGTAGCATTGTAGTAGTTTTTCTATTATACAAAAAAAATGTTTGTCTCGACCCACATGCCCTA<br>AAGGTTTTTTCCATTTGGCCCTTTTGTTGATATATAGCCCTGCTGTACAGTATAATACAGTTTTAAAAAAAAAATAGTATATGAAACAACTCATTATACTGAGTTATTGTCCA<br>GTTATCCTTTCATACTTCATTTTGAAAACTTAATCCTTTGAAAATGTCCACATACAGGCCTACTAAATATTATTTATAATTCCATATGCATTA
LG1	ref-68673_17	68.644			ACAAAACTCGATTTTTTTTTTACGTGACACGCGAGTAAGCAGTCTGCAGAGGAAGCATCAACTTGACACACTACGCATATGCCTAGCCTACAGGAAAGCACAGTTTATGTT<br>TAATCTATCTAATGTTGTGTTGTTCATACTATACAATAGAGATGGGAGAAAAGATTCTCTCTCTAACGATTCTGAGCTTAGTTTTAACCACAGGTGCTCGATGG[C/T]GCTG<br>CATGTGCTTTAGAAACTTATTCTGTTTGCTTCCAGTTGCTCTCTCTCTCTCTCTCTCACTCACACACACACACCACTCGAACCATCTACACAATAATCTTTAATAAAGTTC<br>AGAAAGTTTGAAGCGAATTGCAAGGACATTCGCGCGCTTCATTGCACAGGATGTGAGAAACGTGTGCTTTGCTGATGCTTGCATTGTGTCGTCTCGCC
LG1	ref-63353	69.039			CGATACACATCGACCTGAGTGCTGTACACGA
LG1	ref-34781_2	69.323			G[A/T]TTTGAAGTCGAGTCCGGTGCCAAGAGAGAA
LG1	ref-33329_30	69.622			AATCACAAAGCGAGGTCAGTGCATGCTGA[A/G]GC
LG1	ref-28698	69.859			CGATGCATTGCGATACAAATGCCACTTGTAT
LG1	ref-34781_1	70.073			[G/T]TTTTGAAGTCGAGTCCGGTGCCAAGAGAGAA
LG1	ref-39246_26	70.482			ACTTCCACCTCACCTCAGAATCTCACTTTCATTTCACGTGTCGCTACTGAAACCCCACACTGAACTTTTGTCTTCTCCCCTCACAGGATTTGGTGGTGATGAGATGCCTCCTC<br>CTCCCGAACTGGCTGACGAGGAGGTCATCTACCGGGTCCGCTCCATCATTGACTTGCGGCGATAGTGCAATCATATCGAATA[A/G]CTCATTGACTGGTAAGGTTACGGCCC<br>TGAAGAACAATCCTGGGTTGCTCGTGATGATGTGCTCGATCCATCTATTTCACCCAATTCCATCCTGATCATTCAAATCGTCCAGCACCAAGATGTCATGGCAGGCCCCATC<br>ATCGCACCATCTGGATGTCAGGAGCCAATTGAGGAG
LG1	ref-66535	71.129			TGAGCATAGACGACAAAACTGCAGCGTGTGC
LG1	ref-48985	71.771			AAGCTGTCTGCGAATTGCTTGCCCAGGAGGA
LG1	ref-54338	71.977			CAGCTGCTGAATTCGGAACCTCATAGATTCTAGTATACTACACTTACTACTTTTACAATATCTTTCTATGACAGAAACTGTAGTACGCTAGTGTGCAGTTCCATATGCTGCT<br>CCAGTCAGGATAAGCCAGTACCAGGGAGGTGTCTCCCTCTAGTGGCTGAGCAGTTCAAGAACATAAAGCAGCAAGGTCATACGTATTAGGTCTACGCCTAGTTACTAGAG<br>TTAGTTGCATACTAAGCTCTCTTCTGTCCATTACAATTGTATTTAAAAGGATAGGAAAGGTAGAAAATAATCTGATATCAACAGTTTAATTAAAAAATATTTACAGAAGTC<br>CTTGTGTGGCTTTTAATTTAAATTGTATATTTAGTG
LG1	ref-68306_24	72.581	Chr7	35560350	GGTACATGATTAAAGAGACAGGATTTTTGTGCTTTCAAACTTTTTAAGAAGCTCGATGACGTCTGTTCTTTGTGCTTGTATCACTTTCCCATCGATGGGTTGGTCGTGAGTGT<br>CACGGTTGGATTAGAAGCAGACAGCAGGACACATGTTTGTGCGCTCGGTAACGAACTGTGACCAAGGCACATCTCTCGCT[G/T]TTTCTCGTGAGGTTGGCCCTGCCCTCTC<br>ACCAGGGCCGTTACAGGGCCAGGCAGCAGGGAGGCGCTGTGATAATTTGATGTGAACTGGTTGGATAAATCACCCCTCCATTACTCTGTCTTTAGTATCTGTTTATGCCTCT<br>ACAAATTGAAACCCCACTGGCACAGGCCTGCATGGC
LG1	ref-68970_27	74.542			CAGAAAGCAAGAGAAGAGCCCTCCCCCGCTCCACTAACGCTGTCAATCTATAAAGCGCAAGCTCTCTGAATTCTGCACTCAAACTCTAATTAAAAAAATGCTGTCTTAATT<br>GTACAATAAATCTCTTATTTACATCATGTTTTTTCACACTTTATTGGTTACAGTGAAAGGATTTGTGTGCACGTGGCTCGCATTT[A/G]ACAAAACGGAACCTTGTTCTTTTTC<br>TGTGGTGTTAAGCAGATTCTGACTAACTGGACTGACTAGCACCTCTGATAGGCCATTACGTTCACGCGCTCAACAGATTTGATTGTGATTAGCTACAGTCCTCAACACTTGT<br>AAAAACACGCTGTAAATAGAAAACTTTGACTCTCAG

LG1	ref-29956_17	76.246	Chr7	49787319	CCATTGATGTTTTTAGCTCGAACACATTTTAGATTCTTTATCTTCTCTTTTATTATGCAATGAATCAGTTGCTGATGTAGACCTATCAGTCTGATTTATAATTTGTCTGCCTGTGGCCATTGCAAGGAGCAGATCGCCTAATGTGAATGCAAAAGATGTAGGTCTTGGGGAACAGGATGCAGCCCAAATGGCTACAGATAAATGATGGAGCGAACAC[C/T]CTGCACTGGTTTTAGTTCTGCAGCAGATGTCTAATTCCATACACGGACACAAAAACCCTTTCTGAGAGACATTTGGCGCACTGGAGAGGAGCCAGGGTGTTGCACAGATGTATAGGCACAGAGAGGTTAACGTTCATGCATTTTAAATCTATAGAAATAGGTCCTTTGAGCAGCTTATTAAGCAAACCTCTCATTTCAGTGAGAATGAGAATTG
LG1	ref-32274_5	76.522			ATAATTGGATTAGAGAGGCTAGAGCACGGTTTGAAAATACTGATCAAAAAGAATGTCTTGTTGGAACGCATTTGAAGTCTATCAATAATAGGTTGTCTTGTATTATCCAATATTCCGCTTCAAGTAAAGAGTTTGCACATATAGTATGAAAACATTGGCATATTTTAAGTTCGG[C/T]TCCTGCAAATGAGTCGTGTGTATAAAGATCCTCCACGATTTGTACATAAACAAGCTCTTAATAGGCTATACATCAAATGGTGGTGAAAGCTGACCAAGGATTGGAATGTTCCAGTACGCCTAATCCCAGTCCCTGACGGTAATTATAGGTGTGGACAATGTGCACAATGTAACTTTACAACTAAATGTTATGTTT
LG1	ref-52247	77.287	Chr7	46860973	CCCGCCCCATTGTTCTGGCTCATACCCCATCGCACTCCAGTTCATATTAATAAAACGAGATCACTCACCACTAATTAATACAAGCATGCACTGCTAATTACTGCTCTGTACAACAAGCTGGTTGATGAACCTCCTTTAGAAAGAAGGAGAGGAGAAAAGAGGACGAAAGAGACAGGAAGGCAAGCACACCGTAAGTAGACTGTCGGAAAGGAAAGAGCAAAATGGACAGGGAAAGACAGGGAAAGAAAGATCAAGATGATAAAGTCACAGACAGGGAAAGAAAGATCAAGATGATAAATAGATAATCATGATGGATTGATAAAATGTGCTGTTGATGTAATTCAGTGGTAACACTTTGATTCTTTTAAT
LG1	ref-32594	79.119			CCGCAATATGCGATCTCATTGCATATGAGGA
LG1	ref-1624	80.925	Chr7	44463211	ATAATGACAGCGGTAACAGGTGAATGAAGCACGCTGTGATTGATTGACAGATGAGCTGATCCGCTCAGGCCCCGCCTCCTCCAAAGAAGATATCACCATACCGACTTCTGTTCTGTCTTGTACTTTTTAGTTGCATTTAATTGAATTGTATAGGCTAAATGAACTCTGAAGGGAGCATGGCATTAACGTCATCATGTGGCTGTAGATATTTAGCTCTGATACTTCATTTGAGTTGCTGACAGGATTCATTGTTGCAATTAATTAACATAACCTATGCCGCAAATAACCGACTGTATTATACAATTTATTTCAAAAACAAACTAAATATATGTAAGGAATATTTCCTGTTAGGTCTGAATAACTGCAGT
LG1	ref-57741	81.964			GTTGTCATACCGATTACCATGCCAAACACTC
LG1	ref-41374	83.364			ACTGATACCACGATGCTGCTGCTGCTGCTGC
LG1	ref-21617	83.899			TGACGAGCTCCGACGCCGATGCCTTAAGACA
LG1	ref-37423	84.477	Chr7	42752243	GACTTACTTAAACACTGTCATGTCATTTAACCTTAAGAAATGTACAGCGGCAGGGCCCGGGAGCAGAAGTGACCCTCATTACTGTTTAGCTTCAGAGTTAAAGTCAGGCTTCCATTGTTGCTTTGGAAGAGGATGTTGTGTAGAATTCATGCTGCCATCTGTACACACCATTTCATCTTTCTGCTCTCTAGATTTGGTAGGTTATGATGACGAACAAGGCGCCGCTAATTATGATGCATAAACACGAGAATGCTCCCTTTCTCTATCTTGCTCTTTCGCTCACACACAAGCACAGAAAAACCGAGACCCCACATCTGCTTGGAGCGGAAACAAAATGGATGCATGCCCTTTGGGAGCCCAGGCAAATGAATACTGTTGGCAGAACAATTAGCATTTAAAAGGGAGAGTAACCTTTGCTCAATACATCATTT
LG1	ref-51980	84.685			TCATTCATTTTTTATTTATTTTTTAAAAGTTCATTTAAAAGTCTAATTTTATTAAAAACAAACTAAACTAGACTGTTGCCTGGAGGCAACAATGTACCATAGAGGATTAAACAATATATTTTATTTGTGTATTGATTTTCAGCTGTTCTTTTTAATAGTCAACTTATTTCGGATCATCAGTCATGCTCGGTAAACACTCTGTCACAGACACGAAGATGTTGCCTATCTTTAATTTCACCAAGCTGATTGCTCACTAAAACCCCTGCAGTCAGCATGTTTTCAAGTCTTCTGACGTTTGAATTTGACGTGACAAAGCTGTGATATTTATTTTAATTAGTCCTACTGATGCCATAATTTGTTCATTCAGACTGACGCGCGGAACGCGCATGTAAACAAGAGGCGGCATTTATCGCACAAAAAAATCGTATCCA
LG1	ref-32208_29	86.209			CGAACATGCCCACCGGCCTCCATTAACCTTGTCTAATAGGTGCTCAAACTTCATTGGCTGATGGGAGGCATTGTTTTCTCATAGACTATCATGCCCCCTTGGACCAAGACACTGCATACCAAATTTCAAGTCAATCGGACTAACGGTTGCATAGTTACAGCTGTTTTTTCATGTTATAGCGCCACCTAGTGTTCCATCGACACGGTATACCGAGTTTGGTGCAAATAT[C/G]TCATTTCGTTCTTGAGTTATAGCCATTTTAGTAAAAGTGGCTCCTCCCACACCCTACGTTTTAGCGCCCCTTAGCGACCATGAATCAAAATTTCAACTTTTTTTTGATAATTATTGATATTCAGACTCCAGAGAACATTTCTGCCTAGGAACTAGGACTAGTTCGCAAAAGTAGGTTTTTGACAAAATTCAAAATGGAGATAT
LG1	ref-22633_32	86.543	Chr7	36907982	GGGGAAGGCTCTGCGCTAATCCAATTGTGCAATTAGAGGAGGAATAGGGGGAGAGGAGAGACAAGGGGAGAGGATCTTGGTCTGTACCATTGACCTTTGACCCCAACATGAGCTGAGCTAATGCCGTGTAGGAGATGAGCAACTTCATCGACTTTCTTACATTGTGACCGTAATGTGTAAAGAAACTATGTAGACTGGTCAAGGACAGACCGAAGCTGATGCCACCATTTT[A/G]ACACAATTACATTTAAGCAAAGAATTGTTATGTGTGATCTATTTTTGATACCCAATGCGTTATAATAATTAAACTTTATCTGATTTTTAACATCTGTGGAACTCTATTTTTCATTGAAAAAAATTAATAGTATTTTTGTCACATTTAAATACAACATTTAAAAAAAAAAAAAAAAGTCAAAAGTCAAAAATTTAAAGTCA
LG1	ref-34822_25	87.492			AATAACATTTCGAGACTTGTGCTG[C/T]AAACTCG
LG1	ref-47905_29	87.622	Chr7	15629599	TTGATCAGGCAAACCGAGTCGGATTAGAGAGGGGAAAAAAGGCTGAGTTTTTGCATTGCGCTGTCAGTGTAACTGACAATAACTGTGCACATCAAATATTTAAATGCACAACAGCAAAGGAAACTTTATACTTATTTTTTGAATGCTCACTACCCAACAAATCAACACAGGAATGTCATGCACTAGACTCGAATCAA[A/T]CAACACACAGAATCGACTGATGAACCGACCTCTCCGTTCAAGTGAACTGAAATTGTCATAATCAAACTGTCCTCAAGAGAACCGTGCGTCAGTGTTTGGAAACTCTCATTCACACCAGACCAACAACAAATCCCTAATAAATAAGCACACGTCGCCATTGAGGAACAACAGA

LG1	ref-3951	88.765	CCATTAAACGCGAGATGTGTGCATGTGTAAA
LG2	ref-8043_18	0	AAATGAAAAACGAAAAA[A/G]GTGCCGTTTTTTCG
LG2	ref-8043_29	0	AAATGAAAAACGAAAAAGGTGCCGTTTT[C/T]TCG
LG2	ref-16713_4	0.065	GAG[A/G]TGTTTACGAATCCACTGCTCAGCGCTCA
LG2	ref-48628	0.362	ATCTCTGGTTCGACCACGTTGCTGAATCATC
LG2	ref-11281_24	0.436	CTCCCCGGGGCGAGACTGTTGCC[A/G]AGAAAGCT
LG2	ref-43069	0.446	TTCAGATAAACGATCCAGATGCTGCTTTGTG
LG2	ref-16713_15	1.089	TCAATGGCGCTCTTTACCGAGCGCTTACACCGATACACACAGGAGCGTTTGAAAGCAGGAATCTATCGTGGACCAGTCTGCTGATAGACTCCTGCTTTCAAATTCAAGCACATCCGTGTGCTTTCAATCGCTCCTGCGCGTTGATCATTGTAGCCAATCACAGACATATCTGATGAGCATGTCAAAACAAAGGCCAATCAGAGATGTTTACGAA[C/T]CCACTGCTCAGCGCTCAAAGCATCATATTTTTAAAATTTGGTATCACAGTCGATACTTCTGACAACACTATAAATTGCCTTTTTTTTTTTTTTACTTTGTTTTCTTTGCAATTAATTTTGAGTAATGGACAGAAATAATCTGCAAGCATTCATGAACAAAGCAAGGAAATACTGAACTATGTTCCATATAACAAAAAAACAGGGGAACATAAAACC
LG2	ref-49966_18	1.298	GTTGCCTTCTCGAACCA[C/T]GTGCTGCTGCCCGC
LG2	ref-15673	2.021	CAAACGTATCCGATTTCACTGCAAAATGACA
LG2	ref-40795_15	2.192	AAAAACCTGTCGAT[A/G]TCGCTGCGGTTTTTGCG
LG2	ref-40795_24	2.426	AAAAACCTGTCGATATCGCTGCG[G/T]TTTTTGCG
LG2	ref-54663	2.73	TTCCCTCGATCGAAACCTATGCATTTTTGCC
LG2	ref-48122	3.206	CCTTTTGACCTGAGCTAGTAAGCAATCATCTAGCAACCACATAGAAATTCACTATATCCTCTCACAATCTCTATAGCAACCATATAGCAACACCCTAGCCTAGCTTCATGACATCAAGTTTTGCATGAACCCACAGAGCAACATGCTAAACACCACTAAGAACATCTTAACAACCATATGCAATCCTATCGGTCTGGAAACGACATGTTTCACTCACAATTTCCAAAAAGTAAACATGTAGGCCTTCTTATTTTTGCATTAAAGGGATATTTCACCCAAAAATGAACGCAAAACATACACAAAGTATTGACCTATTATTTTTCTCATATTTTTTCCCACCACCATGTCCCTTTAGGGGTTCCATAAGA
LG2	ref-73208_14	3.346	TTTATGAAGCGACTTGTGTGCTTTGTTTGCGCAAAAAAAACATAATTTACCACTTCACTTACAAAAATATTGATCTGCAACATGCGTTCACAAGAGCTTCTTGACCCATTTTTGCTTGAACACGCTTTGGATAATATTATTTTGTAAAAAAAAAAAAAAAAAGTGGTAAATTATTTTTTTATTGCTCAAACAAAAACTCAATTTTATGAAGCGAT[C/T]TGAGTGCTGAGTCACTGGAGTCACATGGAGTACTTTAATGATGTCTTTTTCCTAGTTGTAGTTGCATTGGATCTCTATGGAGAGACAGAAACCGCTCAGACTTCATCAAAAATATCTTAATTTGTGTTCTGAAGATGAACGAAGGGATTACGGATGTGGAACGACATGAGTGATTAATGACAGAATTTACATTTTTTTTGGGTGAACTAACCCTTTA
LG2	ref-44884	3.561	GTAGTGCACTCGACTTGCATGCCAACAGCGA
LG2	ref-23241_31	4.902	CACTGGGAGACGAGGAGAATGCTGGAGACG[A/G]G
LG2	ref-30933	5.141	ACTGAAGAACTGGAATATCGCTAGAGAGCCCTTTCGCCTTCGAGTGTAACTAAACGAGCCAATGGACATTGGCATGCGAGCTTTGCGTCACATGCACCGCCCCCTTAGCGCGGGTATAAATGCGGAAGTGAGAGCATTCACACTTTGTCTTTGCTTCGGAGCCAAGCATTTGTGTTCCTGCAAAAAGATCGCCTTTTTGTGCAAACTCTATGAGTTTTGGAAAGCTTCCTTTTAGGATCTGTGCTGGTGTTAAAGCACTATACAACGGTGGCCATGAGCTTTACAGAGCTCTCATCTTGCTGTTTTTGTTCTGGTTGGTTTGCGATTTGGGCTGGTTCCTCTCTGTGTGTGTTGACTGTAGGCGATCAG
LG2	ref-23241_30	5.18	CACTGGGAGACGAGGAGAATGCTGGAGAC[A/G]AG
LG2	ref-11970	6.171	TAATAAAAGAATTCTTACTCATTCTCTCTCATTTTCAGATCTGTGCCAATCTGGATTCGGTAATTGTGTCCACCAAGAATTATGAACCAGTGCTGAAGGAAACCGTCCCTAAACCCAGAAAGCAATATGACATTGTTGAAAAAAGAGCTCTGGTACTGTCCTCCGTTCGCTTTCAGACTGCACCTCAGTCATAATGTCATTCTTCTGCACCAACTGCTTGAACATTCAATACCAAAATGTGTGTATGTTTGTTTGTGTGCAACAGGATCCATTTGGCGACCACTTGAAGTTAATGATGTCATTGATTCATGAGTTCATGCCATCATCGGTGAGCCGTAGCCTGCGGGAGGTGGGCACCCAGGAATATGA
LG2	ref-10666	7.091	GATGGCGACGCACCATCTGTCAAAACTATTACTAATTACTTTATGCCCAAGCCAGTGGAGAAACCGTTCTCACCTCCTCGGTCTAACAACATCATGGACTACTTTAAGAGAACCTCGCCAGCGCAAGAAATAAAAAGCTGCTTTCAGGTAGCTAAAGAGAACTCCCCGCAAACTTCAGAGCAAACAAGTCAAGAAGTCCCTGGAAAACCTATAAGAGGACAGGGGCAGAAGCGAACTAGGAAGGTCAAGGACAACAAGAAGTCCAAAGAGGAGGATGCACAGATGGTCACAGATGATGTTGTCTTGATAGAGAGTCCCAGTGAATCAGCTGTAAAGGAAAGTGACACTGCTGCTGTTCCAGCTCAAACC

LG2	ref-2772	7.884			GGCATTATATACACACATGTAGTTAATGATACAGTGTTTCCACAGCACTTCATTCTGAAACAAATTATGCCATATTTTTATTTGCCGGCAACCTGTTAAAATAAAAGCATGATGATTTACTATTTGTAAATGAAATAATAGGATTCAGGGCTATGTTCGATACAATGATACAACAATGTATCGTCACATACTACTTGTTGATATGAGTATCTATACAGCTGCTTCTGACGTTTAAAAGGAAAAGTGCTGAAATTAATGAATAAAACATAATATATAGGCTACAACAAGGCTTAATCTGGCCCACAGAGGAACATAACTGGGATTGCCTGATTTCAAGAGACCCCAATCAGAGCTTCACACTTGCATATCTGCTAAAGTGTTTTACTTATTCTTCTCAACCTGGCAACCATGCGCATGGGCTCTGTCTCCGC
LG2	ref-68735	8.101			GTTTCGTCTGCGATCTCGATGCTGAATAGAA
LG2	ref-13369_8	8.222			TTTTCCCTTTAACTTCATTTCACTTTACTATATTTCTGTATGATTAAGAGCTTGCTTAGCTAGCTTATCAGCCACTTCATTAGCCTCCACTCCCACATGAGCAGGAACCCACAAAAATGTTACTAAGCTTACTTTACTTATTCTTAACAAACTTGCAAGAATCTCATATATAATGTCTTGCCTTGATGATTTTAGTCCACTTGCAAGG[A/G]TTGATAATGCTGCGTAAGAATCAGAACAAATTACAACTAAGCTTGNNNNNNNNNNNNNNNNNNNNNNNNNNNNNNNNNNNNNNNNNNNNNNNNNNNNNNNNNNNNNNNNNGGCATAGGAAAAATCCACGGTGGAATTCTGGATACTATCACAGTTGGGCTGACATCACAGTCTGCAATACCAACTACTCGTGCTTCCCTTTTAGCAATCCATCCAAAGCTTTTGATGT
LG2	ref-43873	8.377			AGAGTCAAGTCTGACTGGGAAAAATTGAGATCCACACAACTACATATGTCCAACCATGTTAACAATATTGTCAGGCAAGTTTATTATAGTAATAATATATATATATTTTCAATGTATTAATGTATCAAGTCAAAACATAAAAATACTAACCTGAAATCCGAAACGCTGTAGCCAACCCCTTGCATTGAGGTTGGTATACACCGATTTTTTTTTTTTTTTTTGATGCGACCCATAGGCCTATACAACCATGCAAAGCTAAACAAGCCCAAACGAATTCATTTAAAACTAAACTGAAGGTAAACCTGTATTGATCTCATGCTTATACTTCTCTCTTCGGCACAAATCCAAATACTTCCATTTCGCGATGTATT
LG2	ref-8362	8.901			TCAGTATGTGATTAAGTGTGGCCAGGTTTGACATTAAACTGAATATTTGTGGAATGGCACTAGCAACTCCAAGGTCATGGATTTGCTTCCCAGAGAACAAACGTTGATAAAAAATGTATACTTTGAATGCACTGTAAATTGCTTTTGATAAAAGCATCTGCCAAAAGCATAAATATAAATATATAAGCAGTACTTTAAAAAAACGAGTCTCGAGATCCCTGTGTTCTGTTCCACTCCATTGCACTCCATCCAGAATCCTGTGTGTGCGGCCTCTGGCACTAAACTCAAAAAATGCATGTCAATGTTAAGTATGAGCCGCAAGCATATCAACATAAACAAGTTCAGGCCGAGTACCCCAAGCTATTTGGAAATACATGTACTATCATACAAACAATGCTAAATACATAACCCCATAAAAAATGTTCCACTTG
LG2	ref-4383	9.843	Chr3	30262306	TTGTGAATTTTCACTAGTATCTAAAATTGGTGTCATAACTGCCTGTTTTTGTTTCATGGTTGGTAAAAAAATAGTTTTGGCCAGTAAATTTTATTAAGTCACCGGCCATTGGCAGGTGTGGCAAAAAGTTTGTTTTAGGCCCTGGGTCCAGGGCTCCTGAAAACACATTTGCGGCTATAGCTCAATAGTCGGGACTGGTTGCCCACCTGGTTACCAGCAGATGCAGTAGGGAACAACAGAGGCAGGTATTGCTGCAGGTAGCTGTGACTATTAGTGATGCTTGAGTTGCAGTGTTTATAGAAGAGGGCCTGGGGCATGGCTTCCTGTTACATGACAGCTCTACAAATGGCTTTTGCCCATAACGGCATG
LG2	ref-38664_25	9.995	Chr3	29120223	AGCTTTCTACATCTTCCAAAGATAATTAACTGCACTTTCATGGCAAGAAAAAATCCAACAAGACTCCAAAGGCCGTTCCACACATTCTCATTACTTTGAATGGCAGGCCCTGTGAAGACTCTATCAGCACTGCAGAAAGAGCCGGCTATTACAGTAATATGTGACATGATGAGGTTGAACACTTCTTCAGAGTGGGTTCATTATGTGAACCGACTGCACTGCGG[A/G]TGAAATTACCTGGTTATGGTGCTCGATGCCCATGCTGATTGTATTGATGAGGATGGCGATCATAATCCCTCTGTTGAAGTACTTGCTTTCAACGATACCCCAAAGCTTCACTCTCATTTCATCCCAGACGTCTTTACATTTCCCAAAACAGGTACGTTTCTTTCTCGGCGTGTCCTCGGAGTCTTCGTCCCCCCTCTCGTCCAAGTG
LG2	ref-22048_32	11.111			ACAAATTTCTCCTTTCTAGAGATGAGTGAAAGGGTTTATACAATCAGAGCCCTCTGTTGAGTGCAGGTCAACTTACAAGAAAAAAGCAAAGCTTTTGATTTCATTCAGAGACTGACACAAACACATCTACACTCTAAGAAAAGTAAAATAGGGCCCACTGTACTGTTAATATGAAGGATTCCCTTTCGAAGTGGAACTTTAACACTGCGTCGACAGTGATGCTATGGGAAA[C/T]GCCCTCAGCATGACCAGTGCCTGAATATGTGTAACAAACACAGCAAATTTATTGGCTGATGGCAGTCCATGACGTCACAAGCGGAGCACCCGTGAATATGAGAGGGCACCTGCAAGACACATCATTCAGCTTTTTTGTCTACAGAAGCCACTTTTTATTTGTATGCATGTGTAAGCCGAGAATCTGTTGGAGTGCGTTCG
LG2	ref-16713_24	11.476			GAGATGTTTACGAATCCACTGCT[C/G]AGCGCTCA
LG2	ref-3421	11.706			TAAGGTTTTGCGAGCGTTTTGCACATAGATA
LG2	ref-24880_5	12.07	Chr11	19059526	TACATCAACCATCAGGGGGGTCTGCGGTCTCGCCGACTTTGCAACTTGGCCCGTCGGATCCTCCTTTGGGCCCAGGGAAAGCTCCTCTCATTAAAAGCAGCTTATATCCCGGGGCATCAAAATGTCGAGACAGGGGCCGAGGCCCGGGGAGTGGAGACTCCACACCGAGGTGGTGGAGCTTATCTGGAAAACCTTTGGTCGAGC[A/G]GAAGTCGACATTTTTGCCTCAAAGGAGTCAACCCAGTGTCCTCTGTGGTACTCCCTAACGCACCCAGCACCTCTGGGACTGGATGCCATGCTGCAGACATGGCCGAGGCTATGTCTGTATGCTTTTCTGGAAAAGGTACGCCAGGAAGGGGTCAGTCTAATACTGGTGGCCCCATACTGGCTGACCAGGATTTGGTTTGCAGACCTAATGTCATTGTTAGACGGCTC
LG2	ref-73208_15	13.133			TTTTATGAAGCGAC[A/G]TGAGTGCTGAGTCACTG
LG2	ref-14084	13.797			AGTGATTGAGCGAGACGCCTGCCAGCCGAAG
LG2	ref-16301	13.799			ACGAGGAGTTTCTTAGAGCTATTCGTGAGTTTTTCCGTCCCATTTACCAGAGACGTTAACATGATCAGAGTTGCAGTTCCCAAAAGCATCGCATGGTGGCAGCGGTTTCCACAATCAGCGCACGAAGCGTGTTTCCGCCACGAAGCACGGAGCACCAGAGCAAGGCCCCAAGAGGGTAAGGAGGGGGGTCGAGGAGGATTTAATCGTCTCGCCCCCCTAAGGAACCTGACGACCAAGTCGCGCTTACCAACAGACTTGCCATCGATGTGGTCGTGGTATGCGGAAATTGCGGCAGCATGAACTTTGAGGGTAGAGGGGGACAGCCTATGCTCCAACCCTTGCTGCAAGAATGAAAGCACGACTCTGATC

LG2	ref-70780	14.157			TATTAATTTCATGCCATAACACTTCTTTATTGAAAATCCTAAAAAAAAAATGTTATTTCCAGGTTAAATTAGTGAAGCACTAAAATTAATGTTTTATAGAAAGATCCAAACT<br>GACATTACAGTTTAACTTTAGTTGAACTGAAAATAAAGTTTGATTAATTTAATTTGGGCATCTGAAATTAAACCAGTTATTTGCAATGAGCTGCCTCTCGATATTTCTGCTA<br>AACAAATTGTACATATTGCTATGTTAACCTGCTTTTTGGTCCTGTGCCTGTTGTAGCTTATTCAAAATAGTTGAGCACAAAACAGGCGTTGGCATAGTCAAAATGCATCATT<br>GGAGAAGTGCTAATTAAACATTTCAGGTCAGTGTTTACAACCCTCCAAAACTACTTTGGCTGAAACCAAACCCATGTTTTTGCGGTTTCGGCCAA
LG2	ref-39917_24	15.031	Chr3	30172525	CTCCTGCATCCTCAGGCTTCACATCCTCTATCACCAGCTTGTGAGTCCTGAGACATCAGGATCAGAAATGTTACCACAACATAAGAAGTGTATGCCAATGATGGACATTTC<br>GATGACTAATCTGACTTAGTGTCCTGACATAAAACAATATTAGTGAGGTCAAGGTGAGCTTGCTTATGCATGAATCGATAAGGGTGGCTGTAAGTGGACCGACATACCTGC<br>CG[A/G]TGTGTGTCATTTGATGCGGTTACTGGGAATGACCTCCACACCATCCTTGTACCACTTGCCAGTCACTTTCTCATCAGACACTTCACACTTGAAGACAGCCTGCTCT<br>GCTGCCTTCACTGTCAGATCAGCTATACTCTGCAAAACCTCCAGTTCCTTCTCTATAATAGAGAAAAAGAGGAGAAAATGGGGCCATGAGGCAATTAATA
LG2	ref-40123_29	15.48			GCGCGAGGAGAACTGATTGCGCGCTGTCTGAGAGATTTGGGATCGCGCGAAAGAAGGAGAGGGAGCACGCAAGAGGGAGCGCGTGAGAGAGAGCGCTTCTGGTCTGAAA<br>GAAGGAGAGGGAGCGCGCAAGAGGGAGCGCGCAAGAGAGAGCGCTTCTGGTCTGTCATTTAACACTATTTGCATGCAGTTCGCAACGT[C/T]TTGGTAGTCATCATTCATT<br>TTTCAAGTATTTCCACACCCCTGAGACTGACATAGTTTCTGCTGCTGCTGTCGGTGTCTCTGTGCACGGAAAATTCTGTCAGTTACGTCACGTGAGGTATCAGTCTTTGGTAT<br>CGGGGGTATTTTTACGAGTATGAGGACAAGTACATGAGCT
LG2	ref-11152	15.749			AAATGTTGTGCGAAAAAGTTGCTATAGGAAG
LG2	ref-71802_25	15.967			CATTTGTGAACGAATGGGTTGCTC[A/T]CAGAAGC
LG2	ref-68620	15.994			CTAAGAAATGCGATGGATCTGCGTGTCTTGA
LG2	ref-46910	16.188			GGAAAGGGCTCGAGTGGATTGCACATATTTC
LG2	ref-59673_24	16.829			GCATACAAGCCGGCCGCTACGCACTCAAGTTCGCAGATTGCAACGCACTCAAGTTCGCGGGTTGCAACGCACTCAAGTTCGCCGGTTGCAATGCACTCTGGATGCTATGGA<br>CGAGATGGCCGCTTCGCCAGTGCCTACGGGCAAGATGGCCGCCCCTCCTGTGTCTGTGACCACAGGGGTCGTTCCTGCTATTGAGTCCGCTCCAGAGCCCGAGCTCCTTGC<br>C[C/T]TGCCGGCGCCGCCCAAGAACAAACTGTCATATTAGTAAGACAGCATATTAGAATAAATAAAAATAATTATTCCAGGGATACGCATTTCACAGATGTCAAGTAAAAC<br>TATATTGTCAGAACATGGTTAAAGAAATTTAATGGTTATACATTCAAAAGTATGGAAACGGATAAGCTTAAAAATAAGGAAAATAAGCTTAAAATATTCCTT
LG2	ref-11116	17.088			GAGATTAAAGCGAAGGTATTGCCGCAAATTT
LG2	ref-225	17.448			AGTAATACATCGATAAGGATGCATGCTCGAA
LG2	ref-25354	17.541			GCAGTGCATCCTTATTATTATGAGCTTGTGTTATGGGTGTTTATTTAGGGGGATGAAACTGTGTGCAGTGACAGCAGACCATAAAACTGTGAACCCTACTGAAATCACACC<br>ATGTCTTAACAAAGAAGGATTAATCCAGATTTACATATTTCTCCGAGTTGTAGAAATCATATTAGTAAGCAATGGCCTCGAAATAAAGCCTAACATTTATATAACATTCTC<br>ATAAATGTTTCAACATTCTGTAATGAGCCGGAAAAAAAAAAAAAATGCAATGGTGCAACTTGAGCGCAATGATAAAGGGAACTACTGTTAGTCTGTGGTAAAAGTGCAAGC<br>AGAGCTCGTTACATCCAAGGTTTTCTGTTATTGATCG
LG2	ref-45116_31	17.942	Chr21	13125323	AACTTATTTCACCATTCAAATTATTATATACCATTACTGCTGGGGTAGTGAAGGGATACTTTTCTGTCCCTACAGAAACTGTTTCGAGAGTGGAGGCTGAAATCCCTGCTGC<br>GGCAGTGAGAAATAATTTTAGAGAGCAGTGGGGGAGATATCATTGCTGTGATAGATAGAAATTAAATTTAGTCTGAAAGGTGTTGCGAGATCAGTGGTCGAAATTTCTGC<br>TGCAGCAG[C/T]GGGGAATAAAGTTTTAGTCTGAAGGCTGTTGAGAGAGCTGTGGCTGGAATCACTGCTGCGACAGTGGGGAATAAAGCGGTTGCAAGAGCACAGGCCAA<br>AATACCTGCTGTGGTAGTAAAATTCAATTCACATTTATTTGTATAGCGCTTTTCACAATACGTATTGTTTCAAAGCAGCTTTACAGAAATTCATGTATCTACA
LG2	ref-28808_17	18.374			TGTCTAATTATTTGAATGATGCTTTGCTGCACCTGACCTAACATTAGTTCGTCCGTTGCATCACCTTTTGCATGCTTTTTCGACGG[A/C]GGTGCTTTTGGATGTAGTATTTGG<br>AGTCGAATCACATTCTCCTCCCTTTTTCTGTTGACAAGTGTAAGAGTGAGTTTGGCTAATAAGTCTTTTTTCCTTCATTCTTTGAAGTTCGATATCCATCTCTTCGTGGGCTGA<br>CAAGTCCATGATAACTTCTAGACTCT
LG2	ref-58282	18.625	Chr3	39659032	TTCTTTTACCTCTAATAAAGAAAAAAAGCATCTGATCCCTGCCAAGAGGCATGCCTGTCTCTATTTATTCTGGAAGCGCTCACGCTCAGATGAAATATGCATACATTTCTCA<br>AGTGCTGTTTTGTTTAAGCTCTGAAAGGGACAAGCTTTGGTCCAGGAGGCCTTGTGTGTTCCTCACTGCATCCGCCTCAATCTGCCTGTGTCCTTACTTGGAGGACAACGTG<br>AGCACAGGAAGTGAGAAAATATCACTTTCTCAGAAACACGGGGGCAGTTCTGAAATGCTTCACTGGCAAGTTAAATCTGGTTGACAGAGGAAGATATATTTGCAGCACCT<br>CTGTGAATGGATCTGGCAGCAAACTATTAACACCC
LG2	ref-37413	18.67			GATACTTTGCAAATTAGGAAAGTGACCCGACTGCGTGTTTTCCCCAACTACTTTTATAGAATAAAACATAACACATATTAAATAACATGTGTCTGTATTAACAATCACATG<br>GCTCTCTCACATGCCACAAACAACCCAGAGTACACATTCATTATTCTGTTCCCTGTGGCAAAATTATAGCGTTTCCACTCCTTTATTAAATTTTGCATTCAATATAGGTGCAA<br>ATCACGCCTCTTAATACTGCTGCCCCAGAAGTGGGGCATGAAAATAACTACTAATAATCCTGAAACAAAAATATCTTAGCATGGATATTTATGACAGATATTTGCAGCTCA<br>AATCTGATTGGACAAGGGTGCCCTGTGGGTCACATGTGAAAGCCTAGGCTTATTAAATCTGATTAAATACCTTTTATTTATTCATATGCAGATTCT

LG2	ref-42126	18.685			GACAGACGGCATTAGTGGTTGCTGTCCCTTTAAGAGCTGCGGCTGTTGCACATGACGCGTGTCCAACTTTCTCACAGCTCTTTACTTTCGTTTTAAGACTTTAACTCATTCAAGGCTGCTCTTATTCGACAAAACGGATATTTTTGGCATTATTGTGCGTGTTACTGTTCAAGCATGAAAGAAACAGAGAACTCGTCCATGCGCGAGATGCGATGGTCTTGCATTTGTGTGTGTGACAGGACATTCACACATTCACAGACAGCACATTCTCTTTTTCTCTTTTGGCGCGTTT
LG2	ref-55044	18.75			CGTGTTTCCACTGAAAAAACCTGAAACAATTTGTCCCAGGAAATGGATTTCGTCATCCGCATATAAGCTTAATAATGCCTGAACCTCGTCGTCTGTCCATTGGTCGTATTTTTTAAACTCAATTGTTGATTCGAATAGCAAATAACTCTTACTGCACGCACCGCAACAGACTCTTAAAAATCGTGGTTGAAATAAAATGCTGCGTGGAGACAATCAAAATGCTGCGTGAACTCAACCAATCAGCATGTTCAGCACCCAAGTCCCATCCCCAAAAGTTCCTGAACTTTGAAAAAGTACTACCTTGCGAGCAGGGACTTTCTGAGGTGGAAATTTTTTCTTCATTTAGACCCTGGTTCCTGCGGTCGAAACACACCAAGTACCACCCCAAAGTCCCTAGTTCATGTGGTGCAAACGCGGCCTCCGATGACGTG
LG2	ref-6959	19.192			CATGCTTTTTCGACAGAGGTGCTTTGGATGT
LG2	ref-43119	19.372	Chr5	39344539	ATTCAAAGGGCTGATGAAAAAGAGCACAGGAGATTCCATTGAAGCCAGGCTGGCAAGAGCATTGTTCAGCTATCGGATTACACCGCAGTCGACTACGGGAAAATCACCCACAGAACTGTGTGGACAGAAACTGAGGTCTACCCTAGATCTCATTCACCCTGACTTCAAAAGTCAAGTGCAAAACAAGCAAGTGAAGCAGAAGTGGGACCACGACCAGCATGCTAAAGAGAGACATGTGAGTGTAGGTGATCATGTGTATACCAGGAACTTCAGTTCAGGCCCTACCTGGATTCCAGGAACAGTTCAGAAAAGGACTGGTCCTCTGTCGTATACAGTCGCACTTGGAAATGACCAGGTAGCGCGTCGGCACATCGATCAGGTGAGGAACAGACATGTGGTGTCTTCTGCTGGAACAGTTGTGTTGTTGGAGC
LG2	ref-35558	19.385			CGTAAAGCAAAACTTCACGACTGAAGTGTATCTCCTGCAGAGACAAAAGTTGTAAAGCCGTGTTGTCACCAACAATGTCTCAGCATAGCTCAGTGGATGACTAACATATTTGCACTTTAAGGTTTCTAGGTGCATTTTTAGGCATATTTTAAGCAAAGGGAAACTGCACGACACACCTTTAATGACTAGAAAATGCTGTTGTGTATTTAACAAAACAGGTGCTGACACTCCTGCCATTGAGCCTTAAATAATCACACCCGGAAAAAACCGACACTCCTCTTTCATTAGACATCAGAAGATTGATTAATGTGATTTCTACATATATTTTAGAAATATATGGATCAATATAGTATAAGTCACTTGCTTTTTTGCTGTTTTCCAATCAAATGTTATACTGTATTGGTTTGCCTATTAGTGGAGGATTTTAAAAA
LG2	ref-65145_7	19.568	Chr3	38624620	GCGAGAAAGCCGTAATCATGCACGAGTACTCGCGTTTTCTTCTACAAGCACTAGAGCGGACCAGTCACAACGCAAGCTTCCCATTGGGTCCTGGTTCTGCCGTGACTACCACGGGACCATTTCCCGGGCATAGCCCGCATGTGTATACTTCCTCACCTCTTGAGTACTCGTTCTC[A/G]CAAGCAGTCTCGTCGTCGCCCTCCCTTTCATCTGTGCCTAAGTCTCACTTTGGTCTCCTAGAGGGCTCGTCGCCTCCAACGGGCTTCCACATGAGCAGCCTGGAGCAGTCTGCGCACACGCAGCAGCTCACACCTTCCCAGGAGCTCACCGAACAGCTGGACAAGCAGCACACCTCCCCTCCGCCACCTCCACA
LG2	ref-53438	20.286	Chr3	41314726	GCAGTCAGTCTCTTAGGTGCCCTGTTTCTGATGCGATATGCTGTCGAGCACCGCACACCAAAGCTGCATGAATGTTGTTGATGCAGCTCTGACGGAGACTGATAGTTTCTTTTGAGGTGCAGTCATTAGAATTTAATAAGAACAGCTCAGGTGAAGCGCTTTTCATGTAGCTTACCATCGCACGGATCTCGTGTTCGAATCTCAGTTATATATCTGGTTCAGGTCAAACTTCTAAAGAAACACTGAAGCATTTCAGGTGAACGAACCAATCATAAGTGACCTGCTTCGCTAGCGTCTCAGCAGTTTCCACAGTGATGGGATGCATTCGGGTATTAGGTACAATGCCAGTCAAAAGTTTGGACACAACTA
LG2	ref-33156	20.736			ATTACTTAACAATTAGTCAATAGTCAATAATACAGCTGTTTATGGCACAATCTTCTGACTATTTCTTTGATGAATTAATCAGATACAAGTTTAATTTAATTTACTGTATTTCATTTAAAACAAATGTCATTCCACATCCTGTCCGAAGTTGCCCTCCAAACAATGTGTTTGGTTTCTGCAGTAGAATCACTGTTTTGTACCTCAAACATTCGAGGATGATGCAGTCGTTTGGCCCTTAGACCCTGTAATCACATCTACATCGGTATGTTGATAAAACACTATGATTCTCAATGCTGATTGGTTTTCTCACCTCTGTGCCCCTTTACCTCCTCACTGTCTGATTGGTCTGTGTTGTGTTGCATTTCTATGACGATTAGCTGATGAGGGTCAGGGTGAATGAGGTTTTTGCTTGTTCATTACGGACATGCAAC
LG2	ref-57077_26	20.761	Chr3	35086217	CTCTAGATTGGCCTTAGTCATGGCGTATCAGCTCGTATCAGGCTTGTGTCCCAAGCCTCTCTCAGTGGTGTGCTGGGCTGTGTTCCATATCAAAGAACCACAGCATCCCACTTTAATCCTATGATATCAGAGCTGTACACTCCATCAACACTGATAATACTCTTCAATTAGCACTCAGAGCACCGCAGTCGCAC[A/G]TATCTGCTGTTACCGCTGCTGCATGAGCGATTCCTGCAGTACGCCATCCTCTACCTCTGTCAGCTTCATTAAACGAGTCCTTAAACACATCCAGGAGGTTATTTTTTTTAGAATTAATGCATTTTAATTCATATAAATACTGAGCTGTCTATCTACACTTACCAAAATGGTACGG
LG2	ref-57077_28	20.888			CAGATATGTGCGACTGCGGTGCTCTGA[A/G]TGCT
LG2	ref-70638	21.508	Chr3	37287765	CAGGATTGAAGAGCTGTGTTTACTGAGCGACTGATGACTGGCTTTTGGCTGCTGTTTTTTGTTGATGTGTTTTCAGTATTGTCCCTGGGGTTGAATAGAGGTTAATTCTGTCATTTAAAAGCAAGGATCAACCTTTAGCACGTGTGTGTGTGTGTGTGTGTGTGTGTGTGTGTGAGAACAGATTATTGAAACATTGCCGCATATTTTGAACGGCTGCCATGCTTACAAGGGAATGTACATAGCACGCCATGACAGAATAGTGGACTTTATAGTGAAGGACATATCAAATTATTTCTCACCTTCAGTAAGAATGTATAAACATTGCTGTGTAAAATCATACATGTTCCAGTATTGCAATAGTAACTCTGAAACCTTTTCACATCTGTTTGCTAATACACCTGATGTTGTGATTAATGAGGAGTCTAGAGAAG
LG2	ref-8922_30	21.562			CCATGTTATGCTAATTAGTCCCTGTATGTATTTAAGCCCTTAGTTACTTCTGTTCATTGTCGGTGTCACTTATGTTTATGTGTTTGTACTATTATTATTACAATCTCCAGAGTTTTACTAGTTTGATTTTTATTAATTAATAGCTTTTCTGTGGTATTCTCCTCTGTCGTGCGATTGCCGCAGCCACATCGTTACAAG[A/G]TCTTGGTTCATTTTAAACTGGACAGGACAAAAGTAATAATTTAAAGTAATTCAGTAACTGTAGAGCGATTCTGTTTCTAACTCACTAAAAAGAAACAGCTTATAAGAATCCTTCATTTGGGAATCTGATGGAATTAGTCATGCTGTGTGTATATATATTATAGCATTAATA

LG2	ref-11554	21.749			GTGAGTCCAGCGAGTCATGTGCAGACCAGTA
LG2	ref-61623	22.929	Chr15	35121391	GAATGGAACGCAGGGCTTGTCTACACTGGATGCGACCATTGTCGCTCGCGTCACTACAGCTAAAAGCGGTCTACACTGGACGCGACAAAGTGCCCGTTGCAAAGCATTTGT<br>CCTTCCTGTCAGTATGTCATTAATAGAATGAGGCATCAGTTTACTGTTGGAGATTTGTCGCTCGAGTCGCACCGCTCTCGTTCAGTGAAGATACAGTGTTAGAGTGTAAACA<br>AATCTGCATTTCAGATTCCAGCCAGAAAAGGTAGAACATTCATGTATCTTTGGCAGGGCCGGCCCAAGCCTTTATGGGGCACTAAGCAGAATTTTATTTGGGGGCCCCTCT<br>GTGACTATTGTCAATGCTTCATTATTCGCACACTA
LG2	ref-50152	23.344			CGTGTTTTTACGAGCCTTGTGCAATGTAGCC
LG2	ref-26701	24.227	Chr10	10643351	GAATGCAAACTTAAGATTTAGAAATAAGAGTGTTTGAACTTCCTTCTCCTGTATTCTATTCTTCTTTAAAACAGAATGGAGCACAGCAGAAAGGTTTCTTTGAGCTGTGTCC<br>TCTACTGTACAGGCGTAAATATGCATATCCTTCAGACTGAGGCTTATTCATTTCACTTTTGGTGTGAAAGGGCCTTAACATTTGCCATAAATAGCAAGCGAGCTCAGCGCAG<br>GTGAAAAAAAGTAATGCAAAAGTAATGTAATGCATTACTTTTCATAAAAAGTAACTAAGTAATGCAATTAGTTACTTTTTTAGGGAGTACCGCAATATAGTAACGCATTAC<br>TCTTAAATCTTAAAAATTAAATATTACAATAAAGAGGAAGTATAGAAATGTGAAAACATGTAAACAAAAAGGAATATCAAATAAGACTATTAGCCT
LG2	ref-18944_19	24.32			GTATTGTTACTATGTCAGTAATATATGTAAATGAACAATGTTTGTGTTACCTGGACTGAGAAATTCACATTTATTTGTCAGCAAAAGTCTGCTGGATGACACATTTTGCTTTT<br>GACAGCTCCAAGGCTTTCATGAAAACTATAGATTATTCTTCCACATTTTTGAATCCCCGTCCACGGGCAATCGGA[C/T]CGACAGAACAGTTATTGTAAGGGTAGATTTATC<br>ACTGAAACCTGTATCGTATTTTGTCTACTCTTTAAACAGCATTATGGTAAAAACGAAACAGCTTTATCTGTACACAGCGCCTTTTTGCTTCAGTGGTTTGGGAGGAACTTTTT<br>TCCACTCCAATTGGCATAAAACACTGTTGTTTTGT
LG2	ref-10942_30	24.455			ACACCGGTATCGACCCAATTGCGTACCAG[C/T]CC
LG2	ref-32981_15	25.402	Chr3	37596265	AACATTTGGGGTTTGTACAGTTTTTTCAAAGTTCTTTTATGCTCACCAAGTCTGCATTCATTTGAATATCACATGATCGGATGTGATGGGCTGCTCACCTTTCTCCAGTCTTC<br>TCATCTCTAGTTCTGGCTTGGTTTTATAGATGATTTGCGGCTCAGTTGAAGTGGCTTGAATGTGGAGCAGT[A/G]ATTTCGGAGGAGAGTTGTCATCGTTTCTTGTCTCCTCT<br>ATGCGTTTCCACTGTTTTCCCTTGTGTGGTTTGTCATCAAGTTCCCCATCACCCTCTTTCCTCTTCTGCTCTGAGCTCATCCACTGCAGCTTAGAGTTATGAGAGAATGTGGT<br>GTGGTGGGATGTGGTGGGTGTGGCTGAATGGGCG
LG2	ref-29128	25.498			TGCGCTCGCACGAGACACATGCGCGTTAGCT
LG2	ref-38530	25.918	Chr3	40562691	TCATAACCTGGTGTATCCTTTTAACTGGGTGTGTAGTTTTAATGTATTTACTTTAATGCTAAATAATAAGAATAAGGGGGTGCTTTATTTATAATGGTAATAACATCATGCA<br>AAAAAACATTGAATTTTAGCCATACATGTCATCATATTCTGGCTTGTGCACTTTCAAAGTGTGACGGGCACAATAGTCATGTAGAAAAGTCAGTGAGAGAGAAAAGTGCTC<br>TCTTTTAATTTAATTGTATTGAAACTGAATCAGACATTTCTTATGAAAATCACTCACAGGATCCAGACGAGGCTCTTGTGCAGCTCCGGGTCCACAGTCTCAAGGTCACAAA<br>GCTGGATGGGTTTCCCCAGGAGCTGCTTATAGAA
LG2	ref-63139	26.643			ACCTTATTGTCGATGCTTCTGCTATGATGAC
LG2	ref-27826_4	27.239			TGG[A/G]TGGTTTCGAGGGCGTTGCTAGAATGTTC
LG2	ref-31448_15	27.317			ACATAGCTGTCATATCATGATTATCTCGAGATAATGGGTCGCGTCAGATTCAGCCAATCAGAAGGCTTTTGGGGCACTTTTTGCCAGCCTGTCAATCATTTACCGCGCCAAC<br>ATAAGGGAAACTGGACGCTGAGAGGATGATAGACAAATTTAAAGTACTTTTCAGAAGCTTGTAGAGTGCAAA[C/T]TATTCGCGATTGAAGTAAATAATAAATAAATAAAT<br>GTAAGCCGTGACTTACAAAGCCAAAACAGAACAAAGACAAGAAGACAGAGGAAAATAAATAGACCAAAAAGCGACGAAACCAAAAAAGAGAAGCAAGATGTGAGTGA<br>ACATCAACTTCATCGCTGGAGGGTGTTGAGAACGTCACAGCGT
LG2	ref-1363	27.817	Chr3	40326100	ACATTTGTCGGACAAGAAGGCCTCCCACTGTGAGTGTCATGTTGTTGGACACAAAAGGATGGCTGCCGCTTGCCAGGCTACAATTACCAGCCATCACCGTTCCTCCCTCCC<br>GTTTTTTTTTTTTTTAGTGTTCAGTACATGGCTGCCTAATAATGTTTACTTGTCTATCCAATGTTGTTGAATGGCTGTAATGTCTGGGAGAACTGAGCACGATTGTCATGCAT<br>TTGTATGAGGCTTTTCACTCTTGTCTGTGCTGATGGTTTGGCTTTGTATCTGGTGTGAATTTTCCAGAAGAAGAGGATTAAGGAGACCCCCTTGTGTCTGCCCCCACCCCAG<br>CTCTCCAGCACACCCTCGGAAAGTGCCGGAGCTCTGCCTGTCAGCCCAGGCCGCAAGAGCAAGGTGAAGCCTAAAGCCAGAGAGGTAACTATTGC
LG2	ref-50419_6	28.541			TTTAC[A/G]AACCCGACCGAGCTGCTTTCAAGTCT
LG2	ref-50419_32	28.669			TAAAAAAATCTAAGTCAATTTAGAAACAATTATAATGTATTTATAATACAAATTCTAAAATTATATATATATATATATATCAGGACTATTACTGAAATGTTCTCATTATCAG<br>CTCATTCATCGGTCCTTGGCCTACAACACAGATATAATTAACAGATTTGGCGACAGTAGACTTGAAAGCAGCTCGGTCGGGTTCGTAA[A/T]TGAATCGATCAGTCCGGTTT<br>TGCAAACCAATTCATTCAAAACACACTCCAATCCTGCTCCTGGAGGGCCACTGTCCTGCAAAGTTAGCTCCAACCCCAATTAAACACACCTGAACCAGCTAATCAAGTTCT<br>TACTAGGCATACTAGAAACTAGGGGTGCGTCTCAATCA

LG2	ref-45046	28.897	GACACATTGCAGATGGCAAAAGGCTAAAACACTTCCTGGTAGGAAATTTTACATTGTCCTTTTACACTGTTAAAATAATTACTGCTCAAGGAAAGGCAAACACTTAAGGTCTGTGAATGACTTTGCATTTCAGTTCCCCAATAAAATCTTTGTGATTCAGCCTGTGTTTGTAGGCTGCAAGCACGCGTATCGTTGTGTCGATCTTTCTTGCTTTCTCAATATGTCATGACATGAGTGTATAGATTAATCAATTTAAAAATTATGTAAAAAAAAATAAAATAAAAACGGGATTAATAAGCGCCCTCTCATGGTACGCACAGCTGTGTTTCACAATCTGGTGTCTGACTTTTAAAAAACACATTAATGATCA
LG2	ref-34945	29.11	GTACGAGGCACGAGTTTGTTGCCCAATTTGT
LG2	ref-61844	29.131	TTTTGCTAAGCGACAAAGTTGCACAAGCGCT
LG2	ref-12412	29.425	TGATATTTGGCAGCTCAGTCAAGGGTGCTGAACATGCTGTCATAACTTGGAATGCAAATGTAGTCGGGGCTCTGAGAAATTCGCTTTGTTACTTTCGTCCCGGTTCTCCCCTACACTTGAACCCATAAGTGTACTGCAAGCAGGGGTGTAGCCAGAACTTATTTCAAGGGTAAGACCCAGCAATTTCTTTGGGGTGGCGAAATCCATCTGATCTAACTGTGACCACGTAGCCTGCGTGGAATAGCCTCACTTTCCCATATGGCCACGGAGGAGAATCGGAGGGTCACATTCAGCGGACAGACACCGACACACTGTATTTTTTTGTATTTATTTAAAAAATGACAATCAAACTCATATAGAAGCTCGTGA
LG2	ref-5546	30.648	GTGTAAAATGCGATTCGAGTGCAACTCGAGT
LG2	ref-27826_18	31.897	GTTAGGTGGAAAGTCTTGAAAGCCTGCGAAGACAAAATCAATAGAAATGGTCCAACATTGTTCATAACTTTAGTTATTTAACCTTATCTAATGCTTTTTTTCACTAACTAGATGAGTGTTAGTCAGACTGTGATGTTGGCTTGACAAAGCCAACATCACAAAGCAGTTGCTAGGATGTTCTGAATAGTTGTTAGGTGTTCTGGGTGGTTTCGAGGGC[A/G]TTGCTAGAATGTTCTTGGTGGTTGCTAAGGTGTTTTAATAGGTGGTTACTAGGGGTTGAACGATTTAAGATTTTTTAAAGTTGACATTTATGTCACTGATGACATCAATAATGAACAAATAAGCCTGAACCTCAAGCTGAAACTCGGTTTGCCTTGTACACAGCTAAGGCATATGACATTATTGCTTCTAGATAACAGCTTATTTTTATTCGTT
LG2	ref-25434	32.159	CTGCCTCAGCCGAAGAGGATGCTAGGACGGA
LG2	ref-4226	32.272	TTGAACATGTTTAGATCTGGCCTTTCTGTCTGCCTGGTGTTCCTTGTATGGCTTAAAATGGCAGACAGACACTAGATTTTCATTTAGCAGCATACCATCTAGCATGGAATTGCAAATTCTGCAGCTTCGCCTGTGGCATAATGAGGCATAATGTGGCATAAAACATACCCTTTAGCTTTGCATGACTGTTGCACACCATTCTTCTTGACAGGATTTCAACAAAACACAAAAAAGTTTTGAATAAACAAGGTCATTGATGTCCCTTGCTAGCCCCTTAATAATTATATTATCCACTTTTTCTCACAGCAAAATCAGATTGGGCCAACATTGGTTTGCCTAGATTTCTAATGATAACTCTCAGAGGGTTTG
LG2	ref-49791_15	33.412	TGGTGTTGACCGAT[A/G]AATTTGCACATGGTTTC
LG2	ref-41295	33.471	GTTCATTCAGCGAGTGCTCTGCACGTCTCAG
LG2	ref-63253	33.613	CGCTCTCCCTCGACACTGCTGCCAAGCCTAC
LG2	ref-38825	33.796	TAAAGCTAGTGAGGACCAGTCAAACGTCCTCACTAGTGGGTATTTCGAAGTATTTCATAATATAAATGAGGACATTTGGTCCTCACAAGTATAGTCAAAACAAGTACACACTCATATACACACACACACACATTACTTCAACATCTTGTGTTTGCTTTGTTCAGGACGGGCGTCTGATCGCAGATCCATTGTTTCCTCTCAGACTGGCCAATCCTGCGACATCTGTGTCATCTGATCGTCTTGTCACTTCTCACTGTGTGAATCTGAATCATGAGATCATCTGTCATTCTAAAAGAGTAAATCAACTCAATCTCTCTCAACATTCATCTGCTTTCATATTTAAATCTTCATGAAGTCTAAAAAAGCTGC
LG2	ref-54150_23	34.053	TGACTAAGATGGACGATCTTCTTTCGGGGGGATTATCAGAGCCGCTTCGTTCACACATGCCACTATGGGACCCTAACTGGACGCTGGAATGCTACATGGGTCTTGCACTGGAACTGAGCGGCTCAGTATTGACTGTGGGAGTTGATACAGTATAGCACTGATGCCAGAGTCTTCAGCCATCATGGACACCAAGTCACAGTTCCTAGTAGCCGAGATCATTGC[A/T]GATGAAGACATTAGGGTGGTTCATAGGTATTTAAAACTGGCATTGAGCCTGGAGGACACATGGCTTTGTTTGGTGCAAGCTGCTGGCATCTCTGCGATGGGGTCAAGTCTGGGGGCTTCAGCCAAAATGGCCACCACGCCTGAGTCTTCACTCAGAATGGCCGCCACACCTGAGTCTTCAGTCAGAATGACTGCCATGCCTGAGCCCTC
LG2	ref-35433	34.057	CTCTTCGTCCCACAAAATAGAAGTCTTCATACTCTTGCTTGAATGCTGATCCGTTTTCTCATCCGTGCTGCATGGAATAAATGTCTTCTATCAGTGAACAAGCAGCTCCTGGAAGGGCTTTTCTGAACTTCACCACTATTTACAGAATCTGACCAACATCAGACTGTCAGCGATTCCCTGCAGTTCATTCGTTGAAGAAGTGTTGATGGACCGTGTCACTAAAATGGAGACCAGGAAATTTTCTGATGATGTATGCTTTCAAATCCTGATGAATCTGCCAGTGAATAAGTCCAGAACACATGATATTCATGAGATTTGTGCAATGCCTTTGTGTCCAGTAGCCTTGCTACTTCTCATCAGCATTTCCCT
LG2	ref-55875	34.123	AGGTGCATCTCGAGCCTATTGCCATAGCAGA
LG2	ref-9438	34.156	AGACGCGGGGCGACTGATATGCCCGACCTTC
LG2	ref-65588_1	34.272	TGTTAGCTTGACAACAGGAGAGACTGAAGACTCGCCACTTCATTCTCCTGCGAGCGAGGAGCTTATGGTGGTGCTTTATCGCGCTATTGCCAAGCTCAACATCGACTGGCCAGCCGAGAAGCAAGAGCAGCAGACCAGAAGTAAACTTGATGAACGCTTTTTACCATCTAGGTCTGCTCTACTTCCACGTCAGGCGCTGCC[C/T]TTTTTTCCCGATCTCCACGCTGAGGTGTCGAGATTGTGGAAGAAACCGGTTTCCTACCGTGTTTTCACCCCGCATACCTTCACTTACAGCAATGTGGCTGGTATGAGAAAGCACGGTTATGGGGCGATG

			CCAAAAGCGGAAGAGTCAATTGCGAGCCATCTCTCTCCGCAGGCGGCATCGTCCCTCAAAGTCCCGACGTTGCCCACTAAGCCTTTGAAGACTACTTCAGC
LG2	ref-43500	34.281	CTTGCACAGACGATTGTTTTGCTTCTTAAGA
LG2	ref-24665_6	35.136	TTTCAACTCCCCTCACTTCCATGCTCCGTCAGAAACCCAAGTCTCTGTCCTGGAACCCCGAAGCTCGAGCTGTCTTTAAGAAACTCCAAGAGGCCTTTTGCACTGCTCCCAT<br>TCTCACACAGCCTGAACCCAAGCTCCCGTTCATCGTGGAGGTGGACGCCTCCATCACAGGTG[G/T]GGGAGCAGTGCTGTCGCAGTCCCACGGAGAGCCTCCCGACTTCAC<br>CCTTGCGCTTACTTCTCACGGAAGCTTTCCCCAGTAGAGTGGAACTATGACATCGGTAATCGGGAGTTGCTCACTATCAAACTGGCGTCGGAGAAATGGAGACATTGGCTC<br>ACCCTGTTCCAGTGCATACTCAGGTACCAACCCCCTCTG
LG2	ref-24665_8	35.184	CGTGGGA[C/T]TGCGACAGCACTGCTCCCACACCT
LG2	ref-5640	35.584	TTCATAGTGTCGACACTCATGCAGTCGAATG
LG2	ref-15607	36.668	ATTAGAGGACCGAGATGGGTGCTGGTGTAAT
LG2	ref-46934	37.448	CCACACCGGTGGATACTGTACAGTAGGGGTGTAACGATTCACTCATTTTCTCGATGCATCGATTACAAATCCTGACGATGCACATGCACCGATCTTAAAACATGTTTTTTGA<br>ATCATGAATCGTTAATTTTGGCCGATAATCGATGTAAAATGCTAAGTATCGGATCAATCTTGATTGTGCACTGTCATCGCGCTCATCCAGCTCTCCTTCACAGATACGCGCT<br>GCACTCTTTACCGCCTCTCACTAGATTGCGCTCGCGCTCCATCCGGAGCTCTCATTGACACATTTTTGAGCAGCCCACATTTGAGAATACCTGGACACATCTGTGCCTCAGA<br>GTTTGGTCTCCGCAGTCATTTATGTGTCCATTC
LG2	ref-10942_28	38.027	GCAGTTGGGAGACGATGGAGACATAATACATTGCTTGAGGAAGATGGCGTGTTTTGTTTGTAAATGTTTCATTATATTTCTAGTGTCTCCTTTGTTGAAAAATGACAAGCAG<br>CAGCTTCTAATTGCCATTAACACTGAAGTATACGGAGATAAAACAACACGAGTATCAATAGCAGTATTGTTTGTCCAAGCTTATCAGTACACCGGTATCGACCCAATTGCG<br>TACC[A/G]GTCCAAAAAAATACCGGTTCTCGGTACCCATCTTTGCTTAGGAATATCACAGTATTATTGACTGATTAATTTCACTCTGAATTCTTTCACAATGAAAGTATTTAT<br>AAATGTGGACTTACCTTCAGCAAAGTGCAGCATAAACAGGAAAAGACAGAAACACTCCTTCATGACTGATCCTCTGAACTCACACACTGATGGTCCAAA
LG2	ref-27014	39.165	ATGATCAATTAATCGTTAACCTTAAAACTGAAAGATGTGTCTACTGCCATGGCATATATGGACCGGCACCGTTTTTTTTTATTTTTTTTATTTTTTATTATTATTATTTTTTGC<br>TCTAAACAACTGTCACGGGTGGTTGCAGGCAAACAGACAAAGATGAAGAAGGAGGTTCCAACATAAAGCTTTAATAACAATAATCCAAATGACAGGCGAACAGGATGCA<br>ACACAATTAACTTAGGGGCTGTTTACACAAAACTAAACTAAAAATGGAAAACTTTTTAGGTGTTTTGGCTGTTCATTTACAAGACAACAGCATTTTGGTGGCCTGATAACA<br>CAAACTTTTGAAAACGGGTTTCAAAGTGCAAGTTTTTTTAAATGATACCATTGTCATCTCTGTATAAAATGCAAAAACACTAATCTGTGAACACGGT
LG2	ref-128	40.546	TAGTAACTAACAGAGCTGCAGTTATGACCTATGACCTGCTCTAAAACCTGACTGGTGATCATTAAGAATATTATTATCTGTTAAAAAGTGTTTTTATTGGTCGTTTATCAAT<br>GATTCGAATACGTTAGTTAAAACTGGTAATCTAGAAATATGACGATAATTACTAGGATCTGAAGGATCTCCACTTTTTAATAATGGAATAACAGATGCCGACTTCCATGCT<br>CACTGTATAATATTTTAAATCAGACTTAAATTAAAAATAGATGCAGTTGGTTCTGCTACAATACCTTCCATTTTTAGAAAATAAGGCTCAATTTTATCAACCCCTGCAGATT<br>TTTTGACTTCTAGTTTCACTAAAGCATTATAAACCTGGGATGTTGTAATAGGAGAGAAATTAAACACCTGCCTGGGACAATGTTTATCAGGAACAA
LG2	ref-53264_18	40.837	TGTAATCAGTATCCAAAATCAAATATCACTACACGTATTGCAATTAGGGGCCGTATCCACAAAACATTTTAGCTTACCACTAAGAGTTCTCCTAAATAGCAGTAAGTTCTTA<br>AGGTAAGAGTTTCCTCTTAAAACCTATTCACAAAGCTGCTGAGACAAACTTTTACTAAGGAATAGAGAGAAGTCTTAACCTAAGAGAAAGGGCAGGGTCGACCTC[A/G]TT<br>GCTATGGATGATGTCAACACGCTTCAGCCCTGGATATACTTTGTTTTTTTTAGACATGCGCGAACGTACACGCACAGTTAAATGCACAGCCTTCCAAAGTATACTTCATTTG<br>ACTCGTATGCATACACAGGTGTTTGACACATGCACAGTTTTGATCTCTCGCCAACATATCTTTGTATTCTTTCATGCTAGAGTTGTACAAATGAGGGCAC
LG2	ref-65548_19	40.843	TTAGTCTTTTCAATGTATAAGTCTTTGCTACTGACTGACACACTCATAAAGGCGTAATAATATAACTTCTGTTGCTGTTCACGGCGAGGGACTATTGGTGGAACGAAGGCTT<br>TTCGTGAAAGTTAACTTCATGAAAGTTGCATTGATACATATTTTTGGCTTTAATATTTGTATTGTGTGGTAACCGTTTTATAAAAGCAATAAGGTACTCGAGGCTA[C/G]TGC<br>GCCGCTTTGCATCATGCCTAACAACGCCCTTCAGCTGTGACTTATTCACGATACAGCACAGCCTCTCGTACCTTATTGCTTACGTATATAACAGTATTATATATATTTAACAT<br>GCAAGCAATTAGTAATTTAGGATTGAGTCCACATTCACTTTTAAGTCACCAAACTTGCTGGTATTGGCACCAAAAAACATGTTTCTTATTGCACAAG
LG2	ref-41678	41.027	TTTTTAAACAAAGAATGTAACTTTTTATTATTCAACAAAACACTGAAAACTGAAAACTGGAAATATATAAAATGTTGCACCATTTCCTGGCTCCTCCTTTGATACTTCCTTTT<br>ATAAAACACCCAGGCTTTGCATTCATATTTTTTCATTTTCATGAACAGTACATCCTCTCATAGTTGGCAACAGGGTCGTGCCCTGAATAATATCTTAAATGCTATTGCAGAA<br>TAATTCATAAAGTAATGACTTTAACGACTGAATGTAAAGTAAGTCATGTATTAAAAATTAAATCTGCTACTGTACACTAGTATGCTTGCTATGGCTTTCATATCAGCAGTGC<br>TTAGATTTATTACAAGACTCACTGAAATTGAG
LG2	ref-3159	41.383	TTCTTGGTCACGAAGAAGATGCTCGAAGCAG
LG2	ref-18871	41.545	AACTTTGTACCGATATGCCTGCCCCTCGACA
LG2	ref-33957_32	41.638	CTGAGAGTGGCGAGAGAGCTGCAGGCTGCTC[A/G]

LG2	ref-6522	41.672			GCTTGACGCTCGACTTGCTTGCTGCTCTTAT
LG2	ref-72342	41.883			TTTCAAACTACGAAGGCGGTGCCTCAGCACA
LG2	ref-20809	42.391			GCCACTTCCTCGACCTTCCTGCACCTTCCTT
LG2	ref-23597	42.888			TGGCAGGTCCCGAGGCAGATGCATATAACGG
LG2	ref-30413	43.206			AATCGACTACCGACGGAGCTGCCCGAGCGGC
LG2	ref-1974	43.727			TGGCTCAAATCGAAACTAATGCTGAAACAAC
LG2	ref-50686	44.567			AACGTAGTTGCGATGATACTGCTTTTCTAGT
LG2	ref-37779	44.591			ATGTCAGGTACGAGTCCTATGCAAACTGTGC
LG2	ref-33505	45.015			AGAGCCATCTCGATTAGCGTGCTTCATCTAC
LG2	ref-57845_26	45.073			TCCCGTGTTCCTGTGGGAAGAGTGAGCCGGCACGTCCTAAATAAGATCTTGGGGCTTGCGTGTTGATCTGACTGATGAACAAGAGACGGGTCCGGCCCTTTCTTTTGCTCTT<br>TCACCAGATCGAGAGGTCCTTCAGTCTGCTTCTGAAGCATGCTTCGGCGCTTCTTCAGACCGTGCTGAGGACGCTGGTTCTCTTGTAGATATGGATCTCGAGTGCACTGCCT<br>C[C/T]GAGCGCAACTCTCATGATGATAGATCTTACGATGAGTTACTGGAGGTAATCACTTGCGCTGTGTACAGTCTTCAGTTAGACTGGCCACAGGAACAAGAGACTCCCA<br>GACGATCCAAGTGAGATGACCAAAGGGAAACATCAGAAAGACAATCCCTTCATTTCTTTCCAGATTTACATGACAAATGATCTAATTCTTGGAATAAGCC
LG2	ref-22129_2	45.255			C[G/T]ATCTGGTACGATTTGCTTGCCGCAACTCTG
LG2	ref-57845_30	45.427			ATATGGATCTCGAGTGCACTGCCTCTGAG[C/T]GC
LG2	ref-21818	45.99			TGCTGATTTGCGAGTATAATGCATGCAATGC
LG2	ref-70582_4	46.931			TAT[C/T]AGAGAGCGACTTCAGTGCTGATTCTCCT
LG2	ref-11267	47.125	Chr3	61799269	CTGAACATTGTTGGAGATGTTTTCAACCTGAGATGTCAGGGCATTGAGGCTCCACAAACTGGGGTTGGACAGGAGCTTTTCTGAGAATCGTTCTTTCATAGAGCCAGAATA<br>GCTCTGGGACTGGTGGTTCACACTGGGTGATGAATGGGTATTAGGGGGCATCAGCATATTTTGGTCTTGCAAACCTGTGGATGAGGAAGCCCCAGGGTTGATGGGAGGTTG<br>GCCGTACTGGAAAGAATCAGGATTTGGCATTAAGGGGGAAGGTGTAGAGTTATATGAGGGAGATCTTCCCACAGACCTTGCAGGTGAATGACTGGAACTTGGACTGCAGG<br>TCTGGTAGTATTGATCTGGTGACCTTACAGAAATATC
LG2	ref-56124	47.253			AAACTTTGTGCGAGAGTTATGCCGATCCCCA
LG2	ref-36377	47.282			AAAACCCTATCGATGTCTTTGCACAGGTATG
LG2	ref-44610	47.743			AGTTTTGACACGAGTGAACTGCTTTGGGAGA
LG2	ref-18583	48.399			TTTGTGTGTATAATCATCTAATGCAGAGTTCACACTGCATGATTTTAGCCCTATTTTTTACTCGCTGACAGATTTTGCGAAATCGCCAACAAATGCCCGAAATCATAGGCAA<br>ACTGGTGTGCATTCACGCGGATGACAATCACGCAATGTGAATTTTCAAAGATGTGATCTGAGAGAATCGCAGATGAGTCGCCGACGCCCGTCACCTGTCGGCGATTCAAA<br>ATCCTACTGTGTGAAATGTGCTCTGACCTCCAGCCAAGATACAGGGCAGCAGGAAGTTCGGAGAGGAGTTATCGACCTGAATATCAGTACGTTAATAGATATATTTAGAA<br>ATATATCAATCAGAAACAAAGCACAAACATTTGCTTGA
LG2	ref-64196	48.449			GTCACAATTGCGAGGGAATTGCAATTTCCTC
LG2	ref-25582_17	48.712			CTGTTTCATCCGAAAC[C/T]AGTGCTTTCGGAGGA
LG2	ref-39239	48.876	Chr8	16850534	TTCTAAGATCAACAAAATGTTAATTAGTGGATGTCACTGATGCCAAACTTTTGATTTGAGAGCTCACAGAAACCCTACTCTTGAAAGGCCAATGTCCTGCAGAGTTCAGCT<br>TCAACCCCAATTAAACACACCTGAACCATCTAATCAAGGTCTTACTTAGGGATACTAGAAACTTTCAGGCACGTGTGTCAAGGTAACAGAAGCAGATTTGAACACAGATG<br>CACAAAGATTTTGTTACATTTAGCCTGATCTGTTCTGTTTATTTTGCCAAGGACTTTTGGGGGCTGTTTACACAACACCATTTCCAACTAAAATTGGAAAACTGTTTATGCGT<br>TTTGGCCAATCATTTACACAACAACAGCGTTTTGG
LG2	ref-40210	48.92			AATAAGAAAATACATGGTAAAAAATAAAGGAAGAAATAAAGAAATCAATAGATCAGTCACAGTATTCTACGAACTTGATGCAACGATCGTTAAGTATTAATAATCAGAG<br>AAGAGTTATCTTACATTTAAAATAATAACAAATGAATATATATATATTTTTTTTCACTATGTTTACTTTTGTGAGCGTTGACTTTCAATGATAAGAACACGCAACAAACTCG<br>ATTTTCTAATGAATTACTTAAACTCATTTTATCAAATGTCTGGAGTGGTTTGACACATCAGACAGTTAA

LG2	ref-44053	49.242	GAGTTACAGGTTTGGGAAGTTTACTGTATTATAGCTTACCTGTACCATATTTAGAATTACACAAAATGTTCAAATGTATACCTATATATGTGCATTTTTATTTTAATTCATGT TTATGATGTAAGGAACCCCCCATCGGCCCAATGAACGTAATCCGACGGCCTGGGCAAAGGTTTAATGCATGTATGTCGTTTCAGCAGCCCTGTGAAGTTCACTCAATTTTT AGAATTTAGTTTCAATTTTACTTTAACTGCTCGTTTAATCTGACTCCAGTTTCAAATGATTATTACTCTTAGATCATTTTTCTAATTAAGGAATTAATCATTGCTCATGTATTC ATTTAGATATTTGATTATTCATGTATGTCGT
LG2	ref-62681	49.286	CTATATTGCTGCACTGTCAGGACATAAATATGCAAAAATTATACCTTTAAGGGTTCAACAGTCTGTCATTGGGGTAGTATTCTTAAAGGCACAACTTTGTACCTTGTCTACT CCTTAAAAGGTTGAATATTAGTATTATTAACTTCAAGTAATAATATGTACACTTAATGTACCACTATGAAGGGTACGTAAGGTACAAAGATGTCCCTTCGAGGGTACTGCC CCAGTGATAAGCTCATGTTGTAACATCAGATGAACTCAGGCCCTGTTTCTACCTGGTATTAAGATGCGTTTTGGTCAATCGGATCACAAGTGGATGAGGGAGACACATTCC GTTTACACTTGGTTTTTTAATCAGTCTCTTTTGTCCACTTTTAACCATTTCTGTCCTGATTTCTCCAAGGAGAGGGTCTATGGGCGGGTAAATGTAT
LG2	ref-30040	49.525	ACTCTTGAATCGAATCGAATGCCTATGAATC
LG2	ref-53036	49.958	ATCACCTGCACGAGAGGCATGCGCTAACAAG
LG2	ref-31246	49.991	CGCTTCGTCCCGAGCCGCCTGCAGTTTGTGC
LG2	ref-65541	50.146	TGCACAAAACCGACATGCCTGCAAAAACGGA
LG2	ref-3966_6	50.262	ACTTA[A/G]CTCCCGACAAGCCTGCTGGTCCCTGT
LG2	ref-65055_16	51.166	TCACATGCTTCGAAA[C/T]GACTGCTGAACAGATA
LG2	ref-62099	51.873	AGTCATTATCCGAACACATTGCCAAACCACT
LG2	ref-16246_28	52.012	CAGTCGCTCCACCTCGGCTCATCGTCCNNNNNNNNNNNNNNNNNNNNNNNNNNNNNNNNNNNNNNNNNNNNNNNNNNNNNNNNNNNNNNNNNNNNNNNNNNNNNNN NNNNNNNNNNNNNNNNNNNNNNNNNCTGCCCCCCCCCCCCCAGCTCCGCCTGGGACCATCGGCCATTTGGCTGCACCTTGGTCGGACGTC[A/T]CCACGCCTTCGCCACGG ACTTGCGAGCCGTCTGCTGCGTGCCGTCTCTCCACCCCATGGCTCCGTCTGGCTCCGCCCTCCCTCCGGCTCCGCCTCGGTCCTCAGTCGCTCCGGTTCCACCTCAGACCTCT GGCACCCCAGCTCCTTCTCAAGGGATCATCGCCACGCCTCAC
LG2	ref-65368	52.661	AATACCATAACGATTGGTATGCTACTTCCGC
LG2	ref-31381	52.975	ATCCCGGTGGATGACAGTGTTGGTATGGAGGAAGTCAAGTCCACTGAGCAGCTGACGCATCACATCCTACATAACGACACAGAGAGAAAAGCACATCAATCTAAAGGCCC GTTCACAGCAAAAAAGATTAACGTCCATGCTAACACACAATATCATTCTGTTTATTCAAAGCCGGTACTGCAGTTTTGTCGTCTAGCATTTTAAATGCTCTTTAAAACTGAG CTCTGATTGGCTGTCAATATTTATATACAGTAGTCAACATCTGAAGTGGATCAAAAAAGTTGTCCTAAGAACTAAGCTGTCCTAAGAAGAAGGTTTTGATCAACTTCAAAT GTTGACTACTGTAGTTGGAAAAAATAATTCTGAAAT
LG2	ref-830	53.107	ACGTTTATACCGAGCGCTGTGCTGATCCTGA
LG2	ref-58061_32	53.219	CTTACCAGAACGACGCATTTGCTGCAGATCC[A/G]
LG2	ref-46009	53.559	TTCACAGGAACGAATAGAGTGCAGCCGATAG
LG2	ref-39280	53.762	CTCACTGGTTCGAAACCCGTGCTTCTCAAAT
LG2	ref-37608	53.766	GGAAGCTCGACGAAAAAGGTGCTAATTGCTA
LG2	ref-30276	53.823	AGTTGTGGTGGAGGGATGCTGATAAACTGTCAATGAACAGAAGTAAGTCTTCTAATTAGGTTAATTATCTGAACCTGCTCCTCCAAAACTTTGTAATTTGGAGTTTGACAG ACTTGTTTTATTCAAACAGGTGTTTTTTATTATAAACAGGTGAGTGCTAAAATCAATGTAGATTGATGGCACTTAGCTCGAGCATTAACAGTTCTTGGTTTGTTGCAGGTGC TGACGTCCATGTGGTGTTGGCTTGGAGAAGACTGGAACTGAAGACTTGTAAGTAATTCCATGTGGAGAGACAAGACCAGACTCTGTGTGTGTGTGGGTGGGTGGGTGGAT GGTGTCTATATATCTGGTGTTGGGCTTATAAGGTGA
LG2	ref-27675	53.831	TCTTTTATTTCTTTTTTTTTTCTAGTAGGGATAATAAAATTCCTTGTGTCTGCACAGTAACAAATGCATTGCTTATTTTGTGAGGTGTGTTTTTACTGTTTGTTCAATAAATCG CATTGTCGCAGGCTTTGATTCCTAACGGATGAAACCTCTTTCCCCACAACCCCAGCCTTTGTGCTGTTTGAACTCAAGACCCAGAGATGGACATACAGAGTTTGTTGCTGAT TTGTGATGAATGAGTTAATAAACATGTTTGAACAAAATACATTTATTCCAGATAAAATAATAAATGAATTACTATTTATTATACACATCTACAAAAACACGCATATCTACT AAATTATATATAATTGAGATTTAACACTTTGGTCAGAGGATCCATTAAATACACAATGGTTTTATATTCAGACAATTGTGATGCAACAATTGAA
LG2	ref-60719	54.192	AATAGGTAAGCTATGTGTACCAAAGTAAGCAAACAAATGAGTAATTGGTTACCTTCATGGCAGTCCTGTAGTAAATCCGAGTCTCCCACAGCAAGGACACAGGTTGTAAG TTCATCGACCCCTTTGCCACTGCCCATACTTTGTCGGTGCACAGCAGTTCTCTGTTTAACTTCCACCTTTACATCAGACCATTTCTTTTTAAGATCATTTACAGCGCGATTTTC AGATCCCACCGCATTGACGCCGTCAGCCAAACTCTCCCACTCATTTTTTTCCCTTTTGTTATTAATTCTGGAAGACAAAGTTCCAAATAAAATATTTTTTCTCGTTTCTAC

LG2	ref-25303	54.224	AGAACGGCCCCGATACCGGTGCTGGAGGCAT
LG2	ref-40044	54.315	GAGGATACAGGAGGAGGTTCCCAGTCAACAGGGAGGAGGTGGAACTGAAGGGTCCAGGGCGGGTCCAGGGAGCAGCATTGAGCAGGGGCATGATGGATGGTGCCAGGCCACAGCCAGAACTGTGCTCCATGGTGGAGGAGTCCTGACAGCCAGAGGCCATGACACTGGAGTAGTAGCCCACTGCATAGTCAAAGTCCCAAGGTGATGGAGCGACTGAGCTGCAGCGACAATCTCCCACGATGACGGTGTGGCTCCGGAGATCAGAGGTTGAACTGGGGCAACCGAGGGCAGTGGCGGTGCCTGAGTGGGCCGTAGTCAAAGGGGTGGAGGGCCAAAGTGTAGAGGAGGCCCACTAGTCCGCAGCGCAGGTGTGGCGAGGTCTGACCGACTGACCGAGGGAGCTCGGCGAGGCCGAATGGCCGATGGTCCAT
LG2	ref-48526	54.386	TCACGACCTCCGATGCTGCTGCCTAATGTTG
LG2	ref-2207	54.388	AGGTACATTAACAAACAATGCAGTCCTTCTAAATAACATCATCAAAAAGGGAGTAATACTGGTTAATCCTTCCACAGTATCAAAACATAACAGATTATAGACCAATTACTATGTAAACTGTAACATTTAACTCTGTATTTAGCATAAATTATGTAGCATATCTTGCTTATCAAGCGATCGCACATGAATCGTTACATGTCCGTACATGCCTGTGCAAACGTACAGTTAAATAATACACTTAATTTTTTTTTAAAAACATAGAAGAAAACTTACTTTATGGTCATGTACTAACACTTTGAAACACCAAACACAGACAAAAGTCCCAATTCATAGTTTTCAGTCACGTGACTGTCGCGGAGCCCTGGATT
LG2	ref-67779	54.431	ACACACCTCACGATCCGGATGCCATGCATGT
LG2	ref-56488	54.504	CACACTAATGCGATCTGCCTGCCGACTTGGA
LG2	ref-64205	54.826	TGGACTGCCTCGATCTCATTGCCTCAAGTTT
LG2	ref-7750	55.29	GGTGTGTCAGCGATCGCTGTGCCATCATCGC
LG2	ref-2781	55.476	TTGCTTAGCTCCAATAGTCCTCAATGGAAACACCTCTGGATACTTGGTAGCGTAATCACTCACCACTAACATAAACCTGTGGCCTGCTCTACTGTTCTCAACTGGACCAACAATATCCATAGCAAGTCGCTCGAACGGAACTCCAACAACTGGAAGAGGTTGAAGGGGAGCTTTAGAGGGCAGTCTACTGGAGGTCAACTGACACTCTGGGCAAGTTCTACAGAATTTGACCACATCTCTTCTAATGCCAGGCCAATAAAAATGTTTCCTAATTCGGGCCAACGATTTCTGCCTACCCAGGTGGCCTGCCCAGGGAATAGAATGACCCAAAGATAGGACTACATTTTGAACTGCCTTAGGTACCACCAT
LG2	ref-61407	55.821	AACTTTTAAGCGACACAGTTGCTTTTCCGGT
LG2	ref-37228	55.923	CAGGACTCAGCGAAGTACTTGCAAGCAAGGG
LG2	ref-49959	56.146	CACACAGGTTCGATTCTCTTGCAAAGAAGTG
LG2	ref-19714	56.316	TTTCTGTTAATAATTATTCACTGTGTATATAAGCTCTTAGCTTTCCCAGTTATGTTTGTCATTAATAGTAAATCTATAGTGTGTTTCGTGTTCATTAAAGTATCTGTACTTCCTGTTCATAGAGACGTCCAGAGACCAGCCAGACTTCAGCCATGCCCACCACAGACCTTGAACCAGAGTCCACTGCAGACTCAAAGAAAGAGCCCACCTCGAAGCCAGTGCCAGAGCCAGTGCCTGAGCCCATTTTCGTCCTGAAGCCCAAGCATATAACAGTTTGACCAGAAGCCTGAAGTATGTTAACCATGAAATGAGGGAAACTCTCCATTTCAGAATGGGAGGTATTTTATCAAACCCAAGATAAAATACCAAATATTTTAAAATTTTAAACTTTCGACCAACTGTCACCTGTGCGTGCATGAGAGGGTTGAGAG
LG2	ref-34614	56.524	GTTCAAATTTCGAGGCCATTGCGAATTCCCT
LG2	ref-59689_4	57.023	CATTAATGACTTGAGACATTTCTACCTGATTTGAAATCAAAAAAGATCAAACTAAAGCAACTTTAGTTAGCCTGGACGCAGAGAAGGCATTTGATTGTGTGAACTGGGTATATTTATTTCAGGTTCTATCAAGGTTTGGTTTTTCAGAAAATTCCAATCCCTGTATTTCAGACCAGAAGCTAGGAAAAAAATTAATTAGAAAG[G/T]GGAACTCGACAGGGATGCCCTCTTTCTCCCTTATTATTTGCAATTTATATTGAGCTTCTGGCCCAGGCAATAAGGGAAAACAATACAATTGGAGAGATTACTATTAATAAAGTAGAACATAAAATGGCATTATATGATGATGATGTGTTAATTTATCTTTCTAATCCAGACATATCACTTAAGATATTTGATTTTCTTGAAGAGTATGGATGGTGCTCAGCATATAAA
LG2	ref-19121_25	57.29	GCTTCCTCGTCGATGGTAGTGCCC[A/G]CACTCAG
LG2	ref-23570	57.551	ACTTTCAGAACGATTTTTTTGCCAGCTGTTG
LG2	ref-40289_32	58.031	AAGTTCCTACCGATTTCTGTGCTTTTCGTTT[A/G]
LG2	ref-72037_29	58.43	AATACAACAGAAGGATTTTAGTGAAAAAGCAATGTGTTACATTCAAATTGTATTTACTTTAACTCGTTACACTTAGGCTTCTAGTCATTTTCAGAAAAAATGTTTGTGCAAGCTTTTGCGATCGTATAGGCAATGTGCCTTACTTATCTGTTGTCATTGGCAACCGGCTCGATTGCTGAGCACGCTGGTCGGGTCGC[A/T]TTCGTTTGTATAGCAACATACTATACTGTACATGCTGGTCGGATCGCCTGTGTTTAGCGCTATACATAGTAGATTCAAGCAGCTTTGCAGTAGAGACAGGAAAACGATTCATGGTGCTAACTGATTTGGCTGCTCTGGCGCAGCCCATAGTAAACATCGTTCTTCTTAAAGC
LG2	ref-37389	59.059	AAAAATCACAATGTGACTCCAGATTACTGTCAACATACTGAAGACCACAAGAAAACTGCTCTGAAATGCTGATATCTGTTGTACAATCCGCTATTAGTCCGAAAATATCACAGTCTGAGGCACGAGATATAATTTTTCTCTGAATAGCATGCGCAAACTGGGATATAACACCATCTCTAGGATGTTCCTCGCTGTTTTCCTTGGTTGCAATATCAGACAAAATTGGGATTATTGCCATTTGCTTTAATCCAGCAAGTACTCTTACAATGTCTTTGTGAATTGGCGATTTTTCATGCAGAGTAAATTTTGATGTAGCTTTGCGCCAATTGGAAAAA

			CCTCTTTCCACAAAGAGCCTATCTGACTGCCCTC
LG2	ref-48716	59.734	GTCTACTTGAGATTGGCCTACTTTCGACTACATGTCAGGCGATGTGAGGCGCATCTCAAACGAGCCTAATTTATCATTAGTGAATACACCATTCTGCTGTTGCCAGTTATTG<br>CGTTCGGTCGTTGATGAAGGAACAATGTAAATATCCACGAAACTCACACTTGGCTTTTCTACTATGGGCACTGGTTTCGACAAAATCGTTACTCTGCTTTGTGTTTTTCTTCT<br>TTATGGGATTTTGTCTTCCGTCCAAGCAGGTACATGATTAACTTTTACTGGGTTTATCAGAGAATGAAAATGAATGTAAAATGTTTACGCTTCCTGATGGGGCTACACTGGG<br>TTCGATTGCTGCGCTCTGAGCCCGGGCGCGAT
LG2	ref-29544	59.822	TCCTCTCTTGCGATTGAAATGCTTAATCCAC
LG2	ref-60767_30	60.71	GGACTTTTCACTCAACTGAGAGAGCACATCCTATTGTATTATAATGACTATTTAATATTACTACTAAACTTACATATTTTAATATTTTATTTTTTGTCATTTGTAGTGTAGGG<br>TCCAAACTCAAGTCCAGGGGCCCCCAGACCCCTAAGTCCTGCCCTGTTTATAATACTCCAAATGTAGCATGGCGCTCGGGAGTAA[G/T]CCTGATGAATGCATTAATCAAGT<br>CCTTTATATTCAGTTGTTCAATGGCCCTGTCCCAAATGGCACCCTAAACCAGAAGTGCGCATCGAGGGAGCATAGCAGCCGCAAAGGGGGCGCTCACGAGCATCCTTCTTT<br>AAGCTTAAATGACGAATGGGACACCCTACGGTCTTGT
LG2	ref-35422	61.185	GAAATAAACGCGATACATCTGCCTGTGTGTG
LG2	ref-25351	61.252	TTAGAGCGCCCGACTCCCATGCCAGCAACCT
LG2	ref-15031	61.397	AAATTTATATCGAGCGCAGTGCAACAGCTGA
LG2	ref-3521	61.934	GCCTCGATGATCTCTAATGTTTGAGGAGCAACAACACTGGGTTAATTTAATAGATTAGATTGTTAGATATGTGCTGTAAATTCTGCATAGCGCTTTAATGTTTGTGATTTGT<br>GAATCTCTCTATTTGCTGTTGTGCCTGATCTGATCCAGCAGGTGGGGTTGTGACTTCTGTGTGTGTGCGTGTGTGGCGCATGCGCACAGCTGCCCTCTCGAGTTTTTTGCTCA<br>ACCACAACCTGAAAGGAACTGTTTTTACCTTTTCAGACTTAAAGCTCGTGCAGCAGTGTGTTTGTAGATAATTTTGTTTTCAGACATGTCCTGATGAAGTGTTTCTTTTGGAG<br>TATTTTATGAGAATAAAATCTCATCTGTTGGATTTACAGCGCAAGTTTTTTTGGATCTGTATGTTTATTTGCTTCTGTGAACGCGATCATTGT
LG2	ref-14557	61.997	TGCAAGGAGGCGAGACTCATGCTTCTCACAA
LG2	ref-51511	62.342	AACAGTTCATCGAGAACTATGCTGACATTGC
LG2	ref-36779	62.861	CACTGTTATGATTGGCTAACATCAATGCATAGGAAACAGTATCAATACCCGCTCATTATTGCACGCGAGTAAGTGTTTTGAAAACATTATCCATATAAAACTCTCATTTACA<br>GACAAAGCATTATGAAATCATCTACTTATGGTATGTGAGGCAGCTGCTGTCAGCAACAGTTTCTTTGTGAACTCTCCATTACACTGTCTTATGGTTTGCGAGGCAGTTGCTT<br>TCAGCAAGTTTCTTTCTGAACTCTCCATTACACTGTGCCTCGTTTACAAAACAATCTGCAGTCAAATGCTCTGAACAAACATATAATCCTTCACCACAATCCGGGACGCCGT<br>TAAAATAAACCATCCACTTGTTTCTGACACTGGGATCCTTCTGAAGCCTGTACAGAAATGCAAACAAGCCGTTTAAACTGAACGCGTCAAAGCGA
LG2	ref-37819_31	63.282	AGAGCAGGACCGAGTGCTGTGCGAGCTGAA[C/T]G
LG2	ref-2994	63.792	CTTCAATGCTCGAGCTGTGTGCTCTTCTGTC
LG2	ref-372	64.448	GAGACAAATTCGATCTTCTTGCACTGTTAGC
LG2	ref-4781	65.078	TGCACTTTCGCGAAGATACTGCCTAATATAT
LG2	ref-30579	65.284	GGATCATTATCGATCCATCTGCTATTTTCGC
LG2	ref-60861	65.599	ACCAGCCGAACACAGTGAGTGTGTGTTAGGAATGGTGAAAGAACAATGTGCTTGGTAAAGTTTAAATCTTTAAACCAAACTTGGGACTTCAGCCGACAAAGTCTGGCTAG<br>AACGCTTCCCATAATGTTTACATGTTAGGTCAGTAGGCAGAGAGCAGGCGGTCTTTTGTGCCTTATCGTGCACACTTATCGTGAAATTAAGCATACACCAAATATCTTGTTA<br>AACACGTCTATTTGTGCCTGTTTAAACACATTGAGCACATGAGCATTTACACTACAAAAGCAATCCGGTCTAATGCATTTTCAACTACCTCTGGAAGTGGTTGAAAGTGGA<br>CAACTCAAGAGTCTTACACCCTGTTTACATCCGTAT
LG2	ref-71360	65.992	GCCCCCCTTTCGAAGATATTGCTTCTAGTGT
LG2	ref-63575	66.015	ACATGTCCAGCGAGTGGCTTGCCATCCAAAT
LG2	ref-53780_14	66.16	GTCTACCTTCATGCTTTGAATCTTGCTTTCCTCCATGATGGAGATCTGTTTTGGGTGTTGTTGGGGGTGTCGGGGGTCCATGAGGCTGACCTAAGGATAATCACCACAAAAT<br>TATGTTTTGAGTTATACATATTCATTTATTTTGCATGCATACTATAAATTGCTCCACATGAGTGTGTGTATCTCACCTGTATGATCAGAGATATGATCCGA[C/G]GAACTTGC<br>CAATCGCCCCATTCCAGGTTCAGCTTTATACATGTGATGGGTTAGATCAACGCCGCCATCAGACTCACTCTGACCTGGCTTCACACTCTGGTATTTATAATCCGGGTGATCT<br>TTCTTGTGCTGAACTCTCAATCTTTCGGCTTCTTCCACAAACGGTCTCTTTTCATTCTCCGTCAGCAGTCTGTTGAGTGACACAATTATCAGTCATAA
LG2	ref-33085	66.351	GTTGGCTGGACGATTATTCTGCCCTTCATAG

LG2	ref-58667	66.499	ATTAATATTTAGATAGATAGCTTCTTTAGCCTGCTAACATGATCACATCGTGCTAGGCGGGCGTGGTTTCAGCAACCAGGCACCTCAGTTCCAACCACGTCCCGCCTCTTTGCCCATTTTCAGATATCCGGGAGTGACGTGCGGTGACGCGTCGACTGTGCAGATATCAACAGCGCAACAGGGTTGTAGGGGATGAGACAGAGACAGAGCGAAACATCTGCAGTGAATTATACCCGGGCCTTTAATTTAAAGGTGCTCTAGAATTGACAATTTACCTCGGCNNNNNNNNNNNNNNNNNNNNNNNNNNNNNNNNNNNNNNNNNNNNNNNNNNNNNNNNNNNNNNNNNNNNNNNNNNNNNNNNNNNNNNNNNNNNNNNNNNNNNNNNNNNNNNNNNNNNNNNNNNNNNNNNNNNNNNNNNNNNNNNNNNNNNNNN
LG2	ref-36324	66.776	TGGAAGACTTCGACTATGTTGCCCGTCCCGG
LG2	ref-17277_3	67.027	TG[C/T]CAGGATCCGAACACCATGCTCCGCACACA
LG2	ref-58061_7	67.061	CTTACC[A/G]GAACGACGCATTTGCTGCAGATCCG
LG2	ref-24197_14	67.355	GCTGGAGCCCCGA[A/C]TGAAATGCTAGGTTCCCC
LG2	ref-6076_31	67.649	CTGCCGTGGACGATGAGGGTGCGGAGGTGG[A/G]A
LG2	ref-39045	68.148	CTCACAGGATCGATCGTTGTGCAGTGAAGAA
LG2	ref-17586	68.359	ATAATTTTTTCACAGCTCATGTTGTATATTTTTTGCAGTGTTTCGTTAGCAGAAAACCCCAAAACACGTCTTCCCTCGACAGCTTTCATTCCCGTTAAAAATCCAAGATGGCGCTGCTGTGAATAAGGTCTTCTGCTTCACATTACACAGATATGGGACAACAGAATGACAGACATTTTTAGGACAAATATATAGCGTATCTTATCATATCGTAAACTATGCTGCGTTCACGCCATTAACCATGACAACCCGTGACGTTCTACTCGGAGCTGTTCACATCCTCGAACTTGGAGTTGTGCGTTTCTAGGGCAACACTAACAAGGCCAAAGAGAACCTGCAGCGGTCTCGTGGTTCAGCACAGTCACGTGGTACAAGTGGGCTCAGAAAATAGCCTACCGAGTTTACAAGTTGTAATCTACGAGGACGTGAAT
LG2	ref-53963	68.839	CTACGGTCGCAGTATTGTCCGTACGGACCAGAACGTGCTTGTCTCTTAGCAGGGCCTTGAAGCGGTGCAGAGTAAGCCATACTGATAGCAACTCCAGGCAATTGACATGCAACTGAAGTCGGGGTCCTGTCCAGGAGCCTGATGCAGCATGACCGTTGCACATGGCACCCCAACCCGTGGCAGAGGCATCGTTGACACAACAACATGTTTGGACTATGGTTCTAGGGGAACACCCGCCCGTAGAAACGACAGGTCCCGCCACGGAGTGAGGGCTTGACGGCAGGCCGGAGTGATGGTCACCCGAAGCGTGCCCTGTTGCCATGCCCATCTCGGGACTCGGCTGTGAAGCCAGTGCTGAACAAAAATGAA
LG2	ref-7985	69.127	GGCCTAGTGCCGAGTGCTGTGCATCTCTGAT
LG2	ref-50498	69.574	GCCACATGCTAAAAAGTCAATGGCCTTACTGAGCTAGTCCACAGTGGGCAAAGCATCAAGTTCTTCCATCTCGGGCAAGGGTGGTGCTCTCCATTACAATGTCAGTGATGACGTTCTCCCTTGAGTATAACTGCTGGTAGTGCTCCACCCATCTCTCCATCTGCTTACCGCGGTCTGTGATGACGTCGCCTGTGGCGGACCTCAGGGAAGCGATCTTGATGCTACTTGGGCCAAATGCTTTCTTCATGCCCTCATACATGGCGCGGATGTTCCCACAGTCTGCAGAGAGCTGAATGCTCTGACAGAGGTTCAGCCAATAATCATTTGTGCATTTCCTGGCAATCCGCTGGGCATCATTTCTGGCCTTCCGGAGTGCAGCTAAAGCCTTCTCTGAAGGCTCGTGCTTGAAGTCTAGCAGAGCTATACGTTTG
LG2	ref-7234	69.61	GGGCCCCGGAGGGGGGGGGGGGGAACTTTGGCCCCGAATCCAGGCCGGGGTCTCAAGACAATGTGTGAGTCAGCCAGCCCGAATTCCAGGCACGCTTTGTCAGTGGAAAAAGCCTGTAGGTCCCCAACCCTCTTAACTGAAGCCAAAGCTGTCAGGAGCGCAGTTTTCAAAGATAAAAATTTCAGCTTGACTGAAAGCAAGGGTTCAAAGCGAGCTCTCTGCAAAGCGGACAGAACCACCGAGAGGTCCCAAGAGGGAACTTGTGAAGGGCGAGGCGGATTAAGTCTCCTAGCCCCCCTCAGGAACCTGATAACCAGGTCGTGCTTTCCAAGAAACTTACCGTCAACAAGATCATGGTGAGTAGCGATGGCAGCCACATAGACCTTAAGGGTGGAAGGAGACAGCCCTCGGTCCAACTTTTCTTGGAGAAA
LG2	ref-20198	69.691	AAAGAAGAGGGCTGAGGACACTGGCCTGGTACACTTTCATCTTAGTGTTGATGGTGAGCATGCTGTTGTTCCACACTCTCTTGGTCAGGCGGGCCATCGCTATTGATGCCTTGCCGATTCTGGTGTTCAGCTCGTTGTCCAGAGAGAGGTTGCTGAAGATAGCGATGCCAAGATATGTAAAGTCCTCCACTACCTCGAGGGTGTAATCGCCGATGGAGATGCAGGGAATGCTGCTGACATCCTGGCCCATGACGTTTATTTTCTTCAGGCTAACGGTCAGCCCAAAATCATCAGAGGCACGAGCGAAGCTGCTGATGAGATGCTGTAGTGCTTCCTCGGTATGGGCAGTCAGGGCGGCATCATCAGCAAACAGTATCTCCCTAATCAGGACCCTGCGCACCTTGGTCTTTGCGCAAACATGCAAGGTTGAA
LG2	ref-71066_2	69.704	GCCTATCAGCCAAAAAGCGAGTCTTCAATCAGACTGGAGAATTTCTTTTACCACTCCTTAGCGTCACAGTACCCTTTATTTCCAGCTTAATATCTTCTAGAAGGGGTTGAGAATGGAGTATGCGGAGAAAATGTATTTAGTACCCCAGCAGCAGCTGGAAAAGCTGAAAAATGAAAATGTTCACGAGAATATTCAACAGGT[C/T]GTAGAGAACGATCTGGATGCATCCATAAGAAATATCTTACTCAGGTCTGATTTGAATCAGTTCGAAAAAGCCAAACTCTACACAAACATCCTGAGCAGGTTTTTGACAATCATTAAACAAGGTGATCGAGAGAGTAACGTTTTAACGCTATCGCTACCATATTCTAAACACGATCATAAAGACGAAGGACCACCAACTATACCTGAAGACAACGGCAGTGTGGATGACGT
LG2	ref-68957	70.378	TTTTTTATAACGAACAAGCTGCGATGTCACG
LG2	ref-11391	70.874	TAAACAAAGATATAATGAATCTTTACAAACTGAGGATGGAATCGAAACTGTCTTGTGTGTGTGTCTCTCTCTCTCTCTCTCTCTCTGTGCGCATGTGCGGAATGTTCGGGAGGAGTGTGTGAGTGACTGAGTGGCGTGTTTGAGTGAGTTTCAAACTTTTTTCAGCAAAAAAGAGAAGCAATGGGATCGAATTCTGATGGCTTTGCATCCTTGACTGCACGCCACGGGTGCAAATGTTTATCAGATGAGACTGTGTCAGTGGAAGATTGTTTAATTGCTGTTAGTAATACAGTTGGTGCGTTAAACATCTTGTCTGCGTCGAGAATGAACAAGGCAGTTGTTATTTTTCTGAGAGAAACGTCTATGG

LG2	ref-61442	71.407			AATGAGAGACCGATTTCAGTGCTACACGCGA
LG2	ref-30804	71.525			ATCTCATCAACGACAACACTGCTTCAGTGTT
LG2	ref-27126_32	71.637			AAACGATGACGTCATACACATGCGCATTATACGTTCAGTCTATAGTGATTCATCGCCATCTAATGGCCTGCCAGCAGAATACAGCGTTTTTAGTTATTTCATAGATTTGTATGAATGGGGATCGTTACCAATGGTGACGTTTGTACGCGGAAAACAAAGGGAAAACTTCTCCGTTTTAAGCATCGTTGTCGTGTAAACGT[A/G]CCCTTAAGTGGGGAAAATGTAAACCCCGCCATACAAATTTTCACAGGAGAATAATGGGAATATGCATGGCTGAGCTTTTGCTCAAGGCCTATTCCTCCGATTATAAATCTATTCTTGCCTATTTGGAGAGACATGTTTATTACATCCAGAGAGGGATTTCTATGTTAAG
LG2	ref-73031	72.039			AACGACTGAACGATTCTGTTGCCTTATAGAG
LG2	ref-63447	72.706			TGGAGCAGATCTGAAAACATCTCAATTCAAACATACAGAGGAATCAGTATCAGAAAGTCAAACTTCTAGCACAGATGTGGCTTCTTCACCTACTGAATTGAGGGAAAAAATCGCTAATCCAAACAACAGTGAAACCAGCGATGCTGAATTGGTGAATGAGAACGAGGATACTTTAGTTAACGTTTGTGAGCAGCAAAAATATGACAATTACGAGTTGTGTGCAGCAGATGTGAATGATAAAGAGAATGTAAGAATGGATGATGGTCTTCAGTCAACTGTTCATCAAAGCAATGCTGATCTGGAGACAGTGGGAGCTGCTGGGGGAGATAAGCAGCATTCTGGAGCCTATAATAATTCCAGTGCTAGCCAGTACAGACCTGGTATAGCATTAGGAAATGAAGAATATAAGCATAGGGCAGATGAAACTATCT
LG2	ref-14610	73.191			CCTTTAAATGCGAATGAGCTGCTTTGCCCCG
LG2	ref-43940	73.437			GTCACAGAGACGAACTCCGTGCCAGCAGTTT
LG2	ref-42400	73.752			TGTTGCTAGGCGATTGCTATGCGGTTTCTAT
LG2	ref-20070	73.835	Chr3	11294772	GTGTAGTGTAAATGGCCTCTAACACAATTATGTATTATTAAAGCATTTTTCTTACTCAGTTCTAATTTTGTGCTGTCAGAAATCAGAAGATCGGAGTTTCTGAATACTTCACATGTGTATTCTCCTTCGTCTCCAGCTCTCAGATTGTCCAGACGGAGGGAAAAGTTTCCATGCTGAATCTCATCAGTAAAGAAATGAGCTCTATCATGACGATCCTGCTGCTGGGCCTTTGGATGATTGTCACTATCTTCATATAGATAAACCAGTTGCGAGTTATTTCGTTTCCATTTTATCTTCAGTTTCTCTTTAAATTGGCCTAACTTTCTGTCAGCATAACAGGGCAAAATCACTGAACCTCCCAGAGGAACAAACAGGAAATTAGAGGGTCCTTTCACAATGACACCTGCAAATGAAAAGATATTTTGTTTTTT
LG2	ref-45920	74.151			ATCAGCACACCGAGTTCTGTGCCACAGATGT
LG2	ref-48028_9	74.163			AGTTTGTTTGTTTCATTCATAACGTGTTCCTTTGTTTAGATTCCCACCACTCATCCATTACCATGGACACTAACACTAATCATGACAGCTGTATTTCTTTTAGTTAATCTCCATTGTATACTTAATTCCCTCCTGTTCACTTAGTGTTTGTCCGGTCACCATTGTATTAAGTGTGTG[C/T]TGCATCCTCTTCGCATATCTTTTGGATTATTGCTATTAAAGCTTTGTCTGATTACCTGCAATCACCCATGACAGATGACCAGATCCAACATGGATTATGAGACAAAATTTCACACTGTGGCTCAGATGAGCCACAAACTTATATATTTCACCCAGTGTTTTTGTCATCTTGCCATCACCTGTCCATATGACA
LG2	ref-33341	74.203			ACTCTCTCTTCGATGGAAATGCTTTCTCAGT
LG2	ref-11744	74.245			AATACAAATGTTATTTCATTTTGTAAAAATAATTTATTTTGAAAACATTTCTAGTTTTACAAAAACACATTTTAGCATTATCACTAATCTGTGTGTGCATTTTCCTTAAAAACCCATCAAGTTGACTCATGATACCATTTATTATAATCGCAATTGCATATTGCAATATTGACCTCAATAACTGCACTATGAAAATTTTCCCAAATCGTGCGACCCTAGTGCAGGCTGACCAACGTGCTTTACAAAACATCATAACACAAACACACATAAAACGGAGAGAAAAAAAAAAAAGAAAAAAGAAAAATTTAAAAACAGACAACATTCATAAAATACATAGCACAAATTACTGACTATAAAAAGCAAGTGAACAGAAGTAAGTTTTCAGCAGTGACTTGAAAATTGGTAAGGAAGGTGCAGATCTAATCTGAAAT
LG2	ref-52460	74.406			AATCGTAAATCGATAGTACTGCGCTTCACTA
LG2	ref-13607	74.715	Chr3	10164685	GACTTGTTCCTTTGTTAGACAATAACACTGCTTTGAATATATCACTAATGTCTTCAGATGTGTCAGTGTCTCTTTGTTGTGTTCTATCATGTTCACTGGCCTTATTAACATTAATGTATTTTGCTCTGTAGTTTATCATGAGTAGTTTATGTATGAAAGTCTGAATCAGCTCATTTTCAGCACAAGACTCGTGGGTCTGTAATGAATGTTTAGTTATCTGAAGAACATCTGCAGCTCTCAGTTTGTTGTTCCTATGAAGATGAAGTCTGTGAAATAGGTGCTCTATTTCTTCACTTTTGGTCTTTTCCTGCTGTAGGTCTTGATCTGAACTGACTCCTGCTGCTCCAGTGTCTATGTTCTCTCTCTGTT
LG2	ref-30038	74.819			GGTGATTGCACGAGATCCATGCGAATTGGAA
LG2	ref-35293	74.847			AGCTCTATCACGATACTCCTGCTGCTGGGCC
LG2	ref-55927	75.1			ATCGCTACTTCGAATGGTATGCAACGTGGTG
LG2	ref-68235	75.121	Chr3	9987401	TGCAGTATACGCTATGAAGTGTGCATTATGCAGTGCACTAGTATTCCATTCTGAACACATCCTCTGATTCAATCAGGTTTCTCTCTTATTAAAATTATTTTCAGTGTCGCCTACGTAATTCGGTAACATGAACCATATTAATCTGATAACTCATGCTGTATGTTCAGGACCTTCATACTGCGCAGGGCTCGTGGAAGGGCCGTGTGAGTTTGGAGGTTGTTCGAGGAGCGGGAGGCCTTCAGAACGTCTAAGAGTCGAGAGAGAGACGACTCATTAGGATTATAAATAGCGTGTGTCATGTCTGCCATCGTTTCAATCATGACTTAATGGGGTGAAACCCGAGGAAGGGCGTCACTGACAAACACACAT

LG2	ref-52335	75.432			GGTCTTTTTGCGATGTATATGCGGGTTGTTT
LG2	ref-32128	75.475			CAAGTTCTGTCGATTCTATTGCTACCAAGGT
LG2	ref-32541	75.545			AACGTTGTTGCGATGATACTGCCTATTCTAA
LG2	ref-41062	75.617			AAGTTCGGAACGACATGAGTGCAAGTAATTA
LG2	ref-17131	75.878			TTATAATACACGATGCATTTGCTGTGTCCAT
LG2	ref-71180_8	75.895			CAGCAACATTTTTTTGAGCGTTATAGGCGTTTGCAGCAGCTTTTTTGCGCATCATAGGAGTTTGCAGCAGCATTTTTGGATATCATAGGCATTCGCAGCAGCATTTTCGGGCATCATAGGTATATGTAGCAGCATTTTTGGGCATCATAGTGCTGCATTTTGGGGGATAAGCGGTGT[A/G]TGCAGTGGTTTCGGGCGTTGCACATGCAGCAGCACCATTTTTTTTTTTAGCATCGTAGAGGTGTGCAGCAGTTTTAGGCATCATAGGCAATGCTATCTACATAGGCAGTTTACTAGGTTTTCAAACACAGCTTCTAATTGATATGCTTATTCAAGCATATGCATGCAAATATTGCCTGTCAGAAATGTGCAT
LG2	ref-30141	76.319	Chr3	10194201	AGATTTGCATGTAGGTGTTTTGGGATCGACTCCTCCGCTGCCCATATCCGAGACTGAGAGCTTGAAATCTGAAGCGGTTTTTGTTTGTGCTGGCCGACTTAGTGTAGGAGGCGATGAGGCCGACCCGACCCGTCCGAGGGAACTTAAAGAACTCTTCTGGGTAAAAGGACTTCTGTTTTGCAGTGCCATTGGCTCCCTTCCGGAGGTTCGGTTCAATCCGGTGTAGGTGAAGCTTGCAGGCATGAGCGGTTTCTTTATGCTCCCTCTGAGCTCCGAGTCTCTGGAAATGGGCTTGAGGATCTTCCGAGGACCTTTGGCCTGACGCAACGCCTTCAGCTCCGGAGGCAACGCTTTCCTCCCTCGCTTCTT
LG2	ref-18651	76.603			CCAGGGGGTTAATAAAGGCTTTTTGAAGCGAAGCGATGGGTTTTTGTCATATTTAAAACTTTATAAATTCAAACTAGCTTCCGGAGAACGACCGTACGCATACTGCGCAAGTCGACTTGTGCCAAGAGAGTAACCTAAGAAGCAACGAATGAAGCAAAATGTAGGAGAATTGTAATCTTACATGCCTCTCGTGGTTCAAACAAATAGGGCTGGGCAACAAACTCAAGCTCCTCTTCTCTTATATCGATATCCTTTGACATTTCTCTTTAAAATTTCTCATTTTAGACTTCGAATTAATGACCGGTGTTTTGTTTTGATCTATCATCTGCGCTTCAGCGATCGTCATTACGTCATGCGTTGAGTCAGAGT
LG2	ref-21419_1	76.603			[A/C]AACATCATCCGATCAACCTGCAGGTACGTCA
LG2	ref-27662	76.925			AGGAAGTGTCCGATTTGCCTGCACTGACTCC
LG2	ref-30595	77.101			ATTCATTAGTTCCGCCGCACAAACCATATTAGCTGCATTGTCGGTAACCAGAGCCGGTTCTTTCTCTGTACTATTCCACTCATTTAAGGCTGTATTTAACAATGCTGCGATGTTGCTGGCTGTGTGGCTGGTGTGGACAGCCCGGGTTTGTAGAACATGAGACATGAGTGTCCAGACACATGCGATAAAGTGACACGTGATGGTGACATATGAGTCTGTTGCCCTGGATGTCCAGCTGTCACAGGTAAGGGCAACTCTTTTTGCCGACTGGAGAGACATTGCTCTTAACCTCTGCATACATTTTCGGTATTGCCATTTCACTGAGGTACTTGCGTGTTGCCATTACGTAGCGTGGTTCCAAAACATTAAAGGGGCTCTATGTAGGAATGACACTCAGTGGTCGAAATAGGTACTGCAGTCCAAATTCAAAA
LG2	ref-54756	77.194			ATCCATTCAGCGAGCCAACTGCTCGATATTA
LG2	ref-6042	77.45			TTTGACCCTTCGATGACAATGCTACTGATCA
LG2	ref-33960_19	77.525			TTGTTTTTCAGTAAAATATCTAAACATTCTTAAGTCAAGTAAGAAGCAGAATGACGCAGACACTGTAAGACTTTTTCAGTAAATCAAACATGTCTGTTGTCATAATGTCAGATGTGTTTATACATTCACAGGAAACTGAGTGCCTCCATTCTCCAGAGACCGATAGGTGGATGGGATACATGATCACATGACACAAACCAGCAGGAAGAGCGAAGCAC[A/G]TGCCCAGAAACGTGCGTGTTTTGGCAAAGAATGGCACACACTCACATCTGGATACCTGTTGATACTGGAACCTGTTGGTATTAGAACTTTACATCACATTCATGACATTTATTCTTTTTATCCTCAGTGACTTACAGTTGAGGAATACGACGAGTGATTCATCTAAAGAGGAACATGAATGAACATGAGAAATGCTAAAGTTTCAGCCATCGT
LG2	ref-14417	77.526			TATCTGGAGGCGATTCCATTGCGCACAATCA
LG2	ref-44537	77.614			TCGGCGCCGACGATGAGTGTGCAGTTGTTTT
LG2	ref-27616	77.711			CAACTGGACACGATTTCAGTGCTTCGGACAC
LG2	ref-17046	77.824			GTGACAATACCGAGCGTTGTGCAACAGTTGA
LG2	ref-48722	77.929			GTAAAGATCCCGAGATGTATGCCATGAGATT
LG2	ref-71439	78.01			GCCGTGAGGCCGAGCCCTCTGCAAGAAGGGA
LG2	ref-34249	78.064			ATTTGGACTGACAGTGTGAACATAGCCATTGATGTTATACACGATTGGTCTGCGCAGAGAGCCGCATCAAGTCGAACACACCTAATGAGCGATTTATTGGTAAGAAAGAAGGATTCTGGGCAGACGAAATAGGGGTGTGGCAG
LG2	ref-61698	78.153			ACCTGTATCCCGATGACCGTGCTAAGGTAGC
LG2	ref-48518	78.234			ACGGCATGAACGAGCCAAATGCCCTTTCCTA

LG2	ref-36808	78.252	TGGCCTTGGACGACATTTGTGCAGCTGTTAA
LG2	ref-3716	78.309	CTGTCCCAGACGAAAGAGGTGCATCGTGAAA
LG2	ref-8566	78.343	CAATCGACTACGACCAGGATGCAGGTTTTAT
LG2	ref-58061_10	78.35	ACGCCGAAAAAAATACACGCTGCTCACGTGTCACTGATGCCTGCAGTGTGAAACTGCCGTTAGGCTTTGTGCAACCCTTTTTGAGCCATATGAATTACTATTACCTGTATGC GGTAGTGATTGCCTGAAATGGAACGCTTCTGGATAGATTGGATTGTGTTTTCCTCTCCAAGCTGCCCTCTTCGACCACTCTTCCGCCTCTTACCAGAG[A/G]GACGCATTTGC TGCAGATCCATATGTAACCTTAATCCAATTGTTTGACGACCTCATTTCAGCGAACGAGGATCTCCTAGACGTCCGGAATGATAGCACGTTTACAGTCTGTGGTGACGCATTT AAATTTAGTAGTATCAAGTATTTATTGACCTTAGGACTTGAGGTGGGGTTCAGTGGCGAGAAGCTACATGTTTGTCACTACTAAATATGTTTTTGCT
LG2	ref-21471	78.35	AGCATGCACTCGAGGGAGCTGCCTGTGTGCA
LG2	ref-13271	78.425	CCAGAGGTTGCGACATGGGTGCCATATGGTG
LG2	ref-34552	78.432	TGCCATTACACGATAGAGGTGCTGCACGTGA
LG2	ref-45463	78.505	TCGTCCATCCCGATGCCACTGCTATCGATGT
LG2	ref-18534	78.552	AGGTAAGGTCCGATGGCAGTGCCGGACAGGG
LG2	ref-13984	78.645	CAGGAGACCACGATGTCCATGCCAACCTACA
LG2	ref-14250	78.667	CAGTAGTGACATTGCTGGTGAATGCTATTTCTGGCCACTTACTGTAGTAATCAGTTAAAGTGAGTGCATACCTGCAATCCCAGGTCCCTAACTCAAATGGTCCAACAATGTC AAGTCCAACCTTTTCCTATGGTGCAGAAGGAAGTGGTATAGGCTGAAGGGGAGGCGTATGTGTTTTGGCACTTTTGTCATGTGTTTGGCAAAGCTGTCATGAAGAGATTAT GGTCTGGGCTGAGTGGTCCATGCCAGGCCACCAATACAAGTCACGAAGACGCTGTTTGGTCCTGACCATGCCCTGGTGACCCTGATGAGCTAAATTAATTAGGACCCTGCG CAGAGATACAGGTACCACCAAACGGTATGAACCTC
LG2	ref-54972	78.777	ACAACTTGCTCGAGGCCTATGCTTCTACAGT
LG2	ref-12048	78.783	AGTAACTTGGCGACAATATTGCATGGCTTAC
LG2	ref-36329	78.798	AAATAATATCTCAATACTGTCAACTGGAAACTCAAAAATAAGGCTACATTAGTTAACTACATTTGTTAAAATTAATATCAATAAGCTATACTTTTCACAGCATGGTTCTGTA ATCTTGGTTAATACTAATTTAAAAATGTATTAATATATTTTTAAAATGGAATGTTGTGGCTCTGGTTATGGGCAGTCCCTCATTTCTTTTTTCAGCTACGACACACTTGCCTG TCAGTTTCTTCAAAGTGTCTGTTTTTAGGACCGCGGTAAGATTTTCTTTCACTGTCATCTCATATATGGAACAATTTGGTAAATTCTAAAAAATGAATAATTGAAATTGAAA TTGAAATACACTTTATTGTCCCCAAAGGGGAAATTTGTGTTGGACTCAAGGTGTTTACATTGACAAAATCAATAAAAACATTAAAAAAAAAAAC
LG2	ref-45696	78.837	ACTCCCCTTATAAAGCATGGATTAGCCTGGACATTATTTAATATAACTCGGATTGTGTTCGTCTGAGAGAAGATAGTCATATACACCTAGGATGGCTTGAGGGTGAGTAAA TCAGGGGGTAATTTTCAGTTTTGTGTGAACTATCCCTTTAAGTTTCCTATGGTAACGCTTAGATCTGTGCGACCAGATCGCAGGTATTGGCGAAGGAGAGTTGTAATCATAA ATCCATCAACGTTTTGCAGACCGATGGATGTAACGTGCACAACAAGCAATTCTGAGGGTAGTACACCAGTCAAATGTAGAGGATTTAGGTTATGTAGCTGCAATGAGATAT AATAAAGATCCATGCTATCAATGAGAACTAAAACA
LG2	ref-7200	79.038	ATGCAGCGCTGCCTCCTCTGAGACTCCAGGGCATTCGCGTACTAAATTATATCGACGATTGGTTGATTTTAGCTCAATCCGAGAGTTTGGCCGTTCAACATCGAGATGTAGT TCTCGCCCATATGAGAAGGTTGGGGTTCAGTCTCAACGCCAAAAAGAGTGTGCTCATTCCATCTCAGACGACCACTTTTCTAGGCGTGGTATGGGACTCGAAGTCCATGCG GGCTCAGCTTTCGCCTGCCCATATTGCTTCAGTTATATCAGCTGTGAATGCGGTAAAACTAGGCCAGTCACTCACTGTAAAACAGTTTCAGAGATTGTTAGGTCTCATGGCA GCAGCGTCCAACGTGATTCCTTTTGGTCTACTGTACGAGACCTTTACAGTGGTGGCTCAGGACCAAGGGGTTCTCCCCAAGGGGAAATCCTTTTCG
LG2	ref-56753	79.041	AAGCCAAATGCGAACGAAGTGCTTACCACGA
LG2	ref-66750	79.178	GCTAGTAACTCGAGGCCAATGCAGTTGGGGG
LG2	ref-68968	79.288	AAACTTACCACGAAGAACATGCAGATCAACA
LG2	ref-46281	79.391	ACGTGTTAGTCGACTGTTTTGCTCAGACTGG
LG2	ref-13970	79.568	TAGCCACAAACGAGGCTGTTGCTGTCTTGGG
LG2	ref-21148	79.672	TCTGACGATTCGACTGTGCTGCTCCAGACGT
LG2	ref-2949	79.82	CATCGTTATCCGACTAGAGTGCGGAAACCAC
LG2	ref-25485	79.931	ACTAACAGTGCGAACACAATGCAGAATTGTC

LG2	ref-55346	79.952			GGAGGAAACATGAAATAGCCTATATTGTTAATGTTATTATTGATTATAAAAGATTTTATATTTAAATAAAGGTAATTAAAAGAAAGATAAAAAATTACGGCAGTGATTTACGCTCCACCCCATATTCGTCACGGGGTGTTTAGGTTTAGGTTAGGGACTCACAAACTCCTACACTGTACTAGAGGCTCTGAACAGAAGCGAAACTTTTATCGATTCTCTTGCTGCTCACGACGTTCACTGTCAGCAAAATGGACATTCCCTTTGAAGAGGAAATGTTGCAAAACTGCAAGTACACCCTGAGAATGACTTGAAAGAGTTTATGTCCTAAATTTATTCCACGGTTTTGTTAGGAGAGATTGATTGTCGATTACAACCCAGTTTACTTCCTTAGGCTATGTAGTTTCGAGTTTAATGTGATATACGCAATAGGA
LG2	ref-17738	80.392			CGTTCATATTCGACCTGCTTGCCTGTATGTA
LG2	ref-30841	80.71			TGCCATGCCTCGAGCTGCCTGCTGAACAGTC
LG2	ref-60950	80.71			ACGGCTTATCCGATCTGTGTGCGATGTGGTC
LG2	ref-57127	80.753			AAATCTTCTGCGAAACGGATGCCAGCTTCTT
LG2	ref-72132	81.047			CTTCTACTGACGAGATGGGTGCAAGGTGGGT
LG2	ref-18390	81.21			CTGTCTACTACGACCAGGATGCAGGTGCAAT
LG2	ref-60718	81.361			AAGCAGTTCGCGAGTCTCATGCCTTTATAGA
LG2	ref-1744	81.381			TACAGTCTCACGAGTCTTGTGCTGAAGAAGA
LG2	ref-20469_31	81.469			AGACTCAAAGAATTTGGACAATTTTCATCCCATCTCGATTTTGCCGTTCTTGTCTAAAATTCTTGAAAAAAATCTCTCTTGTAAGAGCTTATATGAGCAGTTCCAATCAGGGTTTCGCTCCCATCACAGTGTTGAGACTGCACTTGTAAAGATTTCCAATGATCTGCTACTAGCTGCTGATTCTGGTCTTTTATCTATACTTATCCTTCTCGATCTTAGTGCAGCATTCG[A/G]TACCCTTTCACGCTCCATACTCTTAAGCAGATTGGAATCTATTGGAATTACTGGTATGGCTGTAAGTTGGTTTCATTCCTATCTCACAAACCGCAGTCAATTTGTACAACTTACACAGTTTAAATCTAAACCCACTTCTGTTGTGTTCCACAGGGTTCTGTCTTGGGACTTCTTTTGTTTCTTATTTATCTTTTGCCAGTT
LG2	ref-43933	81.526			AGGCAGAACACGAAGGAATTGCTCTAGGATG
LG2	ref-14784_16	81.605	Chr3	14643952	TCACGGTTATATTTTTGACTCCACAACATCAATAACAATGCTGAAGGAATAATGGAGACGTACCCTGTAGGAATCAGACACCACGAAGCACTCTGGTATCCCAGGACCTCTGGATGGGTCTTCATTCACCAGAAACGGTTCGGTGGCCTGAAACATAATATAACACATATAAGTCATCAGCATT[A/G]CGATCGCAAGACTGTTGCTACTATTTACCCTTCACTCTTAGTTTTTTGCAACTATTTTAGCAGAGTTCTTAATCTGCTATATGGTTCTTTGGAGGTTCAAAAAGTTCTTAACCATATTATAAGTTCATATCAACGGTTTCTGGGTTCTTCGAACACAAATCATAGATAGTTGTATTTAAAAAAAA
LG2	ref-40400	81.635			GGGGGACCCTCGACACAGATGCAAATATGCG
LG2	ref-8761	81.704			CCCCCACTAACGACGCCCCTGCATACAGCAC
LG2	ref-57846	81.828			AGGGACAGACCGAGACCCTTGCATTAAGAAC
LG2	ref-48929	81.872			CACGAGGAGACGAGGAACATGCAGCTGGATC
LG2	ref-44984	82.088			GATCCTAATGCGAAAGGTCTGCTGTCATCCT
LG2	ref-28850	82.134			GGTGAGACTACGACCCTGATGCAGGTCAAGA
LG2	ref-9732	82.31			ATTTGGGTTTCGACAATTCTGCTACAGCTCA
LG2	ref-53222	82.392			TTTTCCCCAACGAGTAGCTTGCATAACGTAT
LG2	ref-67685	82.395			CAAAAGCCATTTCACTGCCTGAAGTTTAAATAATGACAAACTAGAAAGCAATATGCTACCATTTAAGCTGTTTAATTGTAAATAAAATACTTCCTAAAGCAACAAGAATGATAAATGCTGCTATGCAATTCTAGAACTAGTTCAGCAGCTGTTTGATTATCAACAGAGAGGATTGTTTATTGTGTTTATTGTGCATTACAGCTGTAACAACCACAGTGATGCTCCCTAGCCTGCGTGAATTACCTGCTCAGACCATGTGCCCTCACTGTAAGCACCAGGTGATCACTATAACAAATCACTACTCCGGACTCATGACCTGGATGGTCTGCGGCTGCCTTGCCCTCATAGGGTAAAATTCAACCTTCTTATTCCTTGATAATTTCATAAAGCATACTATGCACTCATAAAAATGAAGGTTCTTTATTGCATCG
LG2	ref-30543	82.404			TCAGAAAGGCCGAGTTCTTTGCGCATGCGTC
LG2	ref-40797	82.743			ACCGAGGACACGACCACAGTGCAACCCTGAA
LG2	ref-42943	82.751			ATTGCTGGCGCGATCTTAATGCCATGCCTGC
LG2	ref-39078	83.178			GGTGGTAGTACGATGTGTGTGCCAGACTGAT

LG2	ref-23485	83.35	Chr3	50721860	GATGATCAAAAATTAGTTACTTCATAAAGTGCATATGTGATTTTGCCTGTAATGAAGTGATCGTTTTTTTGAACAGTGAAATGTTACTAATAAAGTTATTGTGTGTGTTTTGTGTGTAGGTGCTGGTTGAGGGTGGATAACTACTTCATCTGGAGTTTTATCGGCCCTGTGTCCTTCATTATCATGGTATTGTTCTCTCCAAACATTCAGCGATCTCACTGCTTTGCTCACAGAGTTTGTTCTTTTTTCCCTCTGAAAAGTGCTGCGCTAGTCATATCAAACAAAGCACTTTTGTTCACCTATGGATCTGTTGCTGCTGACGGCTATGTTTTGGCTGTAATATGCTTTATATGAGTGTGGAGTTTAGATTAGGTTTAGATACAGGAACTTGCTGACATTTTTGTAATTACAATATATTTTACTAATACGCT
LG2	ref-25720	83.64			CTCACCAGCACACGATCATTAACTTCCAAGAAGGAATTTGTCACACGATTGTCAAACCTGGTTTTGTTCCTTTCTGCACTCTTTAATGCTCTGCGTGAAGCAAGCAAATAGCTTTCTTCCAGGTGAGACTTCAGATGCTTGACATACTCAGAATGGGATATCTGTGGACCGTCCTGGTATGGCAACCCAAAGGCAAGGTCGACAGGCAACCGAGGCTGGCGCCCAAACATCAATTCATAGGGGGTGTATCCTGTAACTTCATGCTTCGTGCAGTTATATGCATGAACTAATGGCTTGACAAAATCTCTCCAATGAGTTTTGTCTTTCTCATCCAATGTGCCAAGCATGTCTAGTAGGGTCCTATTGAAGCGTTCAACAGGGTTTCCCCTAGGATGGTAAGGCGTTGTTCGAATTTTCTCAATGCCTGATAT
LG2	ref-2046	83.811			CGGCCCAACCCGACTGGTTTGCAACCAGTTC
LG2	ref-31993	83.921			CTACTGATCTCGATTGTTGTGCAGCACTCTA
LG2	ref-11471	84.197			TGCGAGCTCCAGGCCGTCAGCGGCCGTTCAGTGCTCGTGTACCCGCCGAGAACAGTGCTTCGGCCAGTGAAGTTCTACACAAGACTGGCAAACAGGACAAACACCAACTGATTTTTATTATTCACTTTTTCTGATGGAAAACAAAAGCCATCAGAAGAAAAAAATAAAAAATAAAAACAGCAGCCGCCTCGTAAAGGACAGTGAGAAATTTGAGTCTTTGATCTTTCACTAATGTGTACCTCAATCCACAGGTCAAGATGTTGTTCCAGAATGTCCCAACGTTCCTTATGGCGAGAGAGTGGACTGTTTCCCAGAACCCAACGCTTCTGAGGTAGACTGCAGTGGATATGGCCTACATTCCATACAAAC
LG2	ref-36003	84.22			TAAGTAGCAAAAAAACTGGAATGATATTAGCGTCACGGGGTTGCTTGCGCAGGAAACACACTGCTGTAATTTGAGTGGACTTCACTTCAGAGTTGGTCTCAGTGGACACCTTTCAGCCAGCGTTTCACTAAGCCCAGATGCGTTTGGGCAAATCCGATCCCTTCCAAATCTTTCATTGGTGCACCGCTGTCGCTATCAGAGCGATTCCTGCAGCTGTGTGACAGCCTGATGCAGTGATAGGGATGTGTGTGCTGAAGACATAGTTTCAAACAGGAAGCCAGGGCTAATGATTCACAGATGGTTGGGATGGCTGCAGCGCCGAGGCCTGGGCTTCGTCATGGTTGTTTGTGATTTATTCGGCTGTTACTCT
LG2	ref-2945_14	84.589			AGGGTCATACCGA[A/T]TGAACTGCCACGTCTCCC
LG2	ref-2091	84.786			CAGTTTATTTCGACCAGCTTGCTCCTCTGAA
LG2	ref-34368	84.98			GACGACTGAACGAATCTGTTGCAAGTTATCC
LG2	ref-2945_19	84.982			AGGGTCATACCGAATGAA[C/T]TGCCACGTCTCCC
LG2	ref-33277_24	85.187			TACGTGCGTATGGCCCCGACTACACCACTGACACTGACGTGAAGCTCTAGAAAAGGGTCAGATGTCAATTGCTTTGGAAAGAGGGCTATTGTCCTCTGGAAAGAAAGATGACACTGATTCTTGCTACAGCAGTGTTTGTTCATGCTGAATCAGATTCAGAGTTGACAGCCATGCTGCGTGCATTAGACTCGTT[C/T]TGACAGACTGTGGCTGAATGATTTGTTTCTGGGCTCGGAGTGGGACGCGAAAATCACCTTGCCCTGCCCCGTGCCTTTCTTCCTGGAGGTGCATGAAAAACTTTGTAGTATGGAACAAGTGTGCCAATATGGAAGGCCACATGTACCAAAATCTGCTTCAAAAGTTTTTCCTCTCT
LG2	ref-13769	85.903			CCTTGTACAACGAACACACTGCCAAAGATTC
LG2	ref-35815	86.061			TCAAGTGAGACGAGTCTGTTGCATAAATCTG
LG2	ref-68427	86.349			CCGGGGGGCCCGACCCCCCTGCCCGCAACAG
LG2	ref-17326	86.656			TCACCCCACCAAGGAACTTGACGACCAGGTTGCACTTCCCAACCGACTTGCCTCCTATGGGGTCATGATAAGCAGATATTGCAGCAGCATAGACTTTGAGGGTGGAGGGAGAGAGCCTTCGCTCCAACCCATGCTACAGGAAGGTAAGCACAACACCGATCGAACACTTTTGGGGGTCTTCCACTTCAGAGCATAAACTTGTCTCGTAGACGGTGCTCGTGCTGAAGTGATGTTGTTAAGTACCTCTTGGGGTAAGTCACCTAGAACCTCCGGGTCCCATCCAGGGGCCAGACATGGAGTTTCCAGAGGTCTGGGCACGGGTGCTACAGGGTGCCCCACCTCTGAGAAAGCAGATCCTTCCTCAGAGGAATTGGCCAAGTAGGGGCTGTCGCTAGGAGCATCAGTTCTGGAAACCAGGTCCGGTTGGGCC
LG2	ref-26253	86.761			CACTGTGTCCCGAGGGGGGTGCTATGGGAAA
LG2	ref-47747	86.851			GCCTGAACCTCGATAACACTGCCACGAGCTG
LG2	ref-52787	86.919			ACAAGGAGCACGAGGTCGATGCATCCACTAC
LG2	ref-45600	86.943			GATTCTGTTAGCATTTAGCTAACAGAATAATAGCAACCTTACCTGTAGCAGCTAAAGTATTTTGTCAGTAAATTAATGTGGTAAATTTGTCTTGCTTACTGATAAAACACACTGAACGGTTGCTAACAGTTTCTTAAATGTTACATAGCCTACATTTACAATTTTATACAAAGCAACATGCAGTGCATTCAGGATGTACATGATCTTTCTAGCACCTTGCTGTTCCTTTTAAGCTACATTTTGGGGTGATTACAATTTGAGCATGTATCTAAGAAATACAAAAGCAACATGAGCACATAAATACAATGCCATCATCTCTACTGCTGTACTACTGTTGGCCTTTGAGTTTGGTTTGGTGATGTAAAGGCC

LG2	ref-56250_7	87.034	AGATGT[G/T]TGACGATGAGGATGCGGATGGTGTC
LG2	ref-62177	87.2	GCTCTCATAGCGAGAGGAGTGCATGACTGAA
LG2	ref-36577	87.326	CTGCTCCGCGCGATGGACATGCACCATATAC
LG2	ref-15895	87.356	TTTAGTTTTTCCCCTCCGTTTGGCCTAAAATACTGTGTTCGTTTTCATCCAGTTTGAAATGTTGACTGCAAAGATTTTCCGTTACAACGTTCTCTCTGTATTTAGGATTCTTTGTAGCCTTTATCCGTTGCCTACAATGTGCTGGATCCTTCACTGGAAACCAAAGTAACTTACTCCTGCACAACCTTCGCTCTTTAAATTCTTGCACCCAGGAGCAATACAAGTCGACACTATTTTGACTAAAAGTTTGCTCAGGATTATTTCCTACTAAAGCAAGGCAGTATTGGACTCTGGGAGGACACTGGTGGGAGTGGCCTTGAACAGCAACATGCTTAGTGCATTATGAATGTTGATTTTTTTTCTTACCT
LG2	ref-34218	87.488	AGCTCACCTGCGAGCCCTATGCCTGCAGCCA
LG2	ref-8445_15	87.95	AAGTGAAGTTGAAGAGATTTTACCTCGGCCATCCCACTCAGATGTGTCCTGAGTCCAGTTTTTCTGGGCTGCACACAGACACACCAGCGACAGCAGGTTCAGAGCGCTCTTCATCCTCATCCTCCTCAGAGAAACAACAATGATGTCATTCAGGACAGTGCAAAGCTGCAGCATTGAGCGCAG[G/T]GACTTCGTCTGACATTATTTTATAAGAGTTCATGAATTTCAGCATATGCTTCACACATTTATGAACATTTCACTGCTGGAATGAGTCAAATAAAATCTGATGAAGGGCTTCATTGACCGTCTGGTAAACAGATTGTTGACGGACACACTGTGAGAGCATGCAGGGTAAATACCGAGCAGATGCAGA
LG2	ref-16317	88.121	TGGGTCAAGACGAATCGATTGCATCCCTATG
LG2	ref-18487	88.309	ATATGTGTACCGAGTTTCATGCTGATACATG
LG2	ref-7925	88.694	GTGCATCCAACGATAGCCGTGCTGATGTCTG
LG2	ref-53445	89.036	ACAAAAGACACGATACTGATGCACAATGAAT
LG2	ref-19121_1	89.098	[A/G]CTTCCTCGTCGATGGTAGTGCCCGCACTCAG
LG2	ref-58582	89.562	CCCCCTTCAGCGATGTCTTTGCAAGCTGCCT
LG2	ref-7014	89.574	TTCGGAGATGCGAGCTCCATGCCATCAACGG
LG2	ref-25723	89.75	CGAACATTCCCGAGTGTATTGCTAGTGGCCT
LG2	ref-348	89.965	ATATTTTGATTTATAGATTTGAATCTATGATAAAACAGCATAAATTAATCTTTAATAAATAAATACATAATGTCCGAATACCTGTGGTGGTTGTTTTCACAAGTTCACACTTACAATCGAATAGAGTAAAAAAATATAATAAGCGTATTTGGTGTGAAAATTAGCCCAATTTAGCTCATGCAGAAAACTCGAGGTAACTACAGGTGAGTTGGCTCTCTCCAGCTGATTAGATAGACCGTTTGAACGCGTTCTTCCCAAACTTTGTTTATAAAACATCATTTGTTGCTTGAATTCTGCATTCTCTTGAGATGCAGACATGTAAGAGGCTGACAATTAGCTGAAAATTTAATTAGTGACATTTAGCCTACA
LG2	ref-24103	90.285	GTTTCAAGATCGATGCATATGCATGAGCTCT
LG2	ref-31481	90.455	TGACGTGTTACGACTATATTGCAAGTAGCGT
LG2	ref-26845	90.564	GACTGGTATCCGATATCTGTGCTTACATTAC
LG2	ref-6526_15	91.327	CCACATTATCAACAACTCAGACAATAAAAAACTGTAAAGAATTTTTTGTTGTTGTTTGATCTCAAATGACCCGTTTTAAGGGCCTGCGCTGTAGCCCAAGACAGCCCTCCCAATTCCAATTCAATAGAAGAAGACACTAGTGATGGTAGAAATAAAGCGTTTTGAAATGTAAAGCCAAGGCAT[C/T]TCGATCGCTCCTTGCTGGCTGCATTATAGGTCATAAACCCGCCCTCTCCATGTAGGCTGGTAATTGAATGGGATGCAAGCCAAACAAAGTAATTTCAACAAAGTAATTTCTTTTCTACAGTCTATGATACACCTTTATAAACCACGGATCCTTAAAAAGTCAAAGTATTAAATTAGATTTTTAAAA
LG2	ref-6526_29	91.327	CAGCAAGGAGCGATCGAAATGCCTTGGC[A/T]TTA
LG2	ref-55831	91.736	ACATTTGCAGCGATTAATGTGCCAGGTTATT
LG2	ref-32178	92.206	AACCCAGTTCCGACAACAATGCATTGGCTCC
LG2	ref-7791	92.419	CTCTGACCGGCTGGGCCGCGGTCTTTCTTGGCACATAAACAGCTTGGAGCTGAGAGATGTCAACCTGGCCCTACTATACTTTCTCCCCTTTCTCACCACGTCATCATCAGGACGGACAAGATGGCAGTGGTGTCTCACATAAATCGCCAAGGGGGCTCACGGTCACGCACCCTAGACAGGCATGCGCATCGTCTCCTCCTTTAGGCTCAGGACAGGCTCCTTTCCCTGAGAGCGCTTCACGTTCCCGGGACCTTGAACCTCGCAGCAGATTTTCTGTTGAGGCAAAAGCTCAGATCGGGGGAATGGATGCAAAACCCTCAGAAAGTAGCCCGGATTTGGGAAGTGGTCAGCAGAGCAGAAGTGGACCTC
LG2	ref-70648	92.855	ACAGTCAAAACGAACTTTTTGCCACACCTGC

LG2	ref-13906	93.353			CATGCATATTCGAGCTGAATGCACTGCTAAC
LG2	ref-12268	93.769			TGTGAATTAACGAGCCTGTTGCGGTGAGTTT
LG2	ref-14709_5	94.261			GATA[C/T]GGAATCGATATTGATGCCGGTGAAATT
LG2	ref-14709_17	94.297			GATACGGAATCGATAT[C/T]GATGCCGGTGAAATT
LG2	ref-7760	94.605			GCAATTATCGGTAAATATTGACGCATGAATCAAAACATTAATTGAAATATATCGGAATAAGATCTTTTGAAATGTATATATAATACATATTGTGTATAAGAAATATCTTGCGATTTTCTGCTAAAGTTGGGAATCTTTAGCACTTTTCACAGATAGAGAATGGGAAACGATTCAGAGCTATTTCTATATCTATTTCCTATTCCCTATTCACTATATATGTGCCATTCACCATGTAGAATCTAGTAAACGTGTGAGCAAATGACAGAGCTATAACAACCCAGCCACGACACATAATTGCGAATAACATGCCTTACCTTAACACAGGCCTGGGGTCAGCTTCCTTTGCTGTTGGAGTTAACATGTACTAAACATGAGTGGAATTTGCACCGCAGCGCCCCCAACCTTGATGCTCATCACAAGAATAGCATGTA
LG2	ref-10502	94.656			GTAAAGTTCTCGACTGCGTTGCCGAAGAGGC
LG2	ref-57455	94.954			GGGTGGTGGCCGATGGAAATGCTGTCACTGA
LG2	ref-35107	95.169			AGTTGGCTAACGATGCTTTTGCGAAACATTC
LG2	ref-55175	95.783	Chr3	6675028	AGTTGAAGCCCTTCAGGGTATTTTTCACAACACCCTGAATATAATCAGATGAAAAGTTGTTCATAATCATGGACACATAAAATGTTTCAGTGTTTGTGTTGGTCTTCTTAAACTCCTCCATCTTTTCCCTAAATAGTTCCAGTTCCTTCTCGGATAAATAGCTTGGAATGAAAACTGCATCGCCAGTTCCTTCAGTCTCTTCACAGGATTCGACTCTTATGCAGTTGACAATCATGACTTGTGGATATTTTGAAAAAATGTTTTGCTTCAAACATTCCTCCCCAATTTGATGTTGGAGTGTTAGTACTTTGCGAATGCCTTGGAAGTCATCGATCATGAGCAAAACGGGAAGTCGTGTTGGTTGCTCTTTTGTCTCATATGTTAGTAGCTGCACAACCTGTTTGGCTACAGTAGAATAATCATCTGTTGTG
LG2	ref-12302	96.285			CTCACATGATCGAACATGCTGCATGACGAAC
LG2	ref-12520_1	96.934			[C/G]TAGTATTGCCGATACCGATGCCTATACATTT
LG2	ref-12587	97.488	Chr3	10544331	GCTAATACAAACCTGCCTGCTGCGGGAACAAGGGAGCGACCCACACCAACCACCCTGAATACTAATATCCTCCGCCATGCCGCCCGTAACGCAATGCATGCATGAGTCGGACGCCTGCCTCTTTAAATTTCTAAGAAGGCCATATTCGTGTAGTTGCTTGAGCTATCAAAGACCTGCCTGCAATGGCTTCACTCGCTCGCTCTTCTGTGATGAGTCTGTCTTTTCTCTAGAAACAAGACATGAGATTCCAGCGAGCAGAGGTGTGACGTGTCTCCAGTTTGACGCTGATCTGTAACTGACGGACAGGTCAGGTAGTTTTGGAGTCTACCAGCAATTACCGGCCTGTCCAGGTGAGCTGAAGTGCTGTGG
LG2	ref-16109	97.811			TTTTGAGCATCAAAGGCATTTGCAGCAGCAATTTTTGGGAATCATAGACAGCAACAGCATTTTTTGCACATCATAGGCATTTGTAGCAGCGTTTTTTGGGCACCATGGGCATTTGCAGCAGCATTTTTGGGCATCATAGGCATTCGCAGCAGCATTTTCGGGCATCATAGGCATATGTAGCAGCATTTTTGGGCATCATAGGCATTTGCAGCAGCAATTTTGGGGCATCATAGTCAGCAGCAGCATTTTTTGAGCATCATAGGCATTTGCAGCAGCATTTTTGGGCATCATAGGAGTTTGCAGCAACATTTTTTTGAGCGTTATAGGCGTTTGCAGCAGCTTTTTTGCGCATCATAGGAGTTTGCAGCA
LG2	ref-55678_10	98.367			AGTTTCTTTTTTGTCCCAATAGAAATTAAACTTAATTTTGTTAAACATGAGTACACTGGAAACAGTTGTTAGTGCACAATGATGTTCTCCAGTGAATCTCGATCAGAGTTCAGCGTTCAGATCCGGGAAGTGAGTGAGAGAGCAACTCACCAGAAAAAAAAAACCTTCATGAATAATGCA[A/C]CATGTCGCTCGGTGTAAATGCTACTTTATAATAAACCGTGATATCTGTATATGCGGCTTCCAGGAATATGACAGCGAGAGGGCTGAGGGTAATTATTTTACCCCGGCTCAGACTGCTTGCACTGCACAAATCCTCTAAGCGCCGCGTTACAGAACGACGTACAACAACAAATCCAAATGCAGCTTCTCTG
LG2	ref-56713	98.679			TTATTTGTTTGTTTTTTATTATTATTTATGATTAATTATTATTATTATTATTATTTTAATTGTTTTTTTTTTACTTTAATTTAGTAATTTTTTAAATTTTTTAAATTTTTTAAATTTAGTCTCATCTTTTTTCATCAACGATATTTCATGTTGATATAGGCACATTTATCATTTAATGGCATTGGTGTCATCTTGTCATCGTCTCGTCTTAGTCATGGGAAAAAAAGGTAGCTGACAAACATTTCTTTTCATTAACGAAATTAACACTCACATGCGATCAACCAATAGCAAACCACAATCATCCAATCAATTCCTGATGGACAAAGTCAAGCCCCGCCCTACATTTGTTCTTATTTCAGAAGCCATTT
LG2	ref-34482	98.991			GGTGAAGAATCGACCCGCTTGCTGAAAGAGA
LG2	ref-21564	99.334			GTGTTAAATGCGAAAGCAATGCAAACAAAAC
LG3	ref-62608	0			AAACTTAATGCGATGCACTTGCCTCAGATGC
LG3	ref-12002_15	1.451			ATGTTCTGCACTGCAGTATAGACAGATGGCTTTGGGCTTGGTAGGTACTTTGGGGTATACTGCCTTGGGTTTGGTTAGTGGTTCTATGATCTGTCTTTGTTCCATGCCATGCAGTATGGTGGTTTGTTTGCTTTGTCCCCAAATTGCTGCCCTTTGATCTTGCTTTGTGGCTCTTGTCTCGTCAGTTGGGTCGAATTGGGTCAGTTGGGTCGAA[A/G]GGCTTGCAGGGCATTGGTGTATGACATAACTGACTCTGCATGTGCCAACACCAGTCTGTGAGCGTTAAGGACCTTCACATGCTTAAGAAGGATGGAGTACTTATAATGCTCTGGTAATTGGGGGTGGAGCAGGTTATCCAGAGCAATCCTAAGTTCCACATACTGGGCTCTGTCCTCTTTAATGAAATCTGGGAAGGTGGGACCTTCTACTCTTGGA

LG3	ref-28172	1.591			GAGATGAACTCGAAATGCATGCTTTAGTCTC
LG3	ref-13312	1.733			GTCATAGACTGATTCATTAAAATTATAATGTACTATACTACAATATAACATAACTTATTTAACAAGCATTTAAAAAGTCCAAAATTCAGTGGTAAATGTTTTTTTTTTTTTTTTTTTTTTGAAAAGTGATATGTACAGATTGATGAGAATAAACGTATTAAATGTACCTTGTCCCTGTAGCAGATGGATCGCCTCGTTTTTCCAGCCTCACCCTTTATCTCCACTTCACACACTCGAGCGAACTTCAGCAGCCGCTGATGGTCAAAGCTCTTTGAAATGCCAAGATGATGACAGTCTCTGTGAAATAAAACTCAATGATGATTGAAGTACAATGATTCAGATTTAAAGAATTATAATTTCTTTGAACTA
LG3	ref-63062	2.512	Chr1	40738028	CTGGGTGTTTCTTCATGGGAACATCAAACCCAACTTGGTCATTCCATTGAATATCACTATAGAGGAGCTGGTTATTTGCATCCATTGTATAATAGACTTAATTATTACCAGCTATCCCACTGTGACAGGTGACCATTTGCTTGGTCTCTGCTGGACTGTCAGACCTCTTCTCCAGATCTGCATGTGCATTGACCATTACCTGGCAGTGATTCATCCAGTAGCATTCCTGAGATATAAGGGCATCCAGTACAGGATTGCATTGGCAGCAGCAGCTTGGCTAATTGCAGTGGGAGACAGCGTGAGGCTCATTTTCATCAGACTAGAATATTTCCCAGATCATGTATTCTTTCTGTTTTTTTACATTGCAGT
LG3	ref-40868	2.584			TTTCCATTTGCGATGCCTTTGCTGACCTCTG
LG3	ref-32121_3	4.316			GGATCACGCTAC[A/T]AACATAACGATCTTATTGCTGTGTCCGTTAACCCATAGGCTAATTCCCACTTTTGGACGCACAGCTGATGTTTGCACGCGCAGTAAATCACTTCCGCGAGAGGATAACAGATCTACACGCGAAACGGGAGCCCGCGAGCTCATTTTAAACCCGCGCGTGCATGACATCTCCTCTGCGCGCGAATCAAGCCGTGTGCTCGGACACGACTCGCAGCACGCGCGTCATCATT
LG3	ref-15588	4.844			AGAGTTTGAGGAAGACATAACATTTCTGGGGGGGAAATAAAACATCCTCAGATACACCACCAACACCAAGAATAGTGCATGTGAGGCAAATTGCAGAAGCATGAAAAAAGTTATGTAATACTGTAAGTGATTAGTGGAGGAGTTTGTTTTGGTTAAAATTTATGTCAAAAATCCTGCAAGCACAACATGCGGTCTAATCCAGGTTTAATCAATGGGTGGTGCAAAATGGAATGTCTAGATTTCAGTAAAGGCAGGTTTAAAAATTTAAAAGCATTTGACCCACTATTTATTTGCTTGGAGCTATAAATGTCTGGAGAGGAAACGGATTTGTGCCATTATAACACTGTTGCTGTCTGCCCAAGCAAACTG
LG3	ref-26631_9	4.958			AAGTCTTAAATTCAGTTTTATCAAATTTATGGTCATAAAAATATCTTAAATATTTTAAATGGTATAAGAAAAGTCTTAATTATAATTTCAAGAGGACTTAAATTTGGCAACGGAACAACAGAAATCGTGATTATGGCATATGTGATTTTTAAACTTTAAAAGGCTATGATTTAATCACACATGAGCGCTGCATGTTCCAAAGTAAAAA[C/T]ACGACACAGTTGCTTGATCTCACGGTTTCTGCACATGATGTGAATCTCCCATTCCACCACATCCCAAAGGTGCTCAGTTGGATTGAGATCTGGTGACTCTGTAGGCCATTTGAGTATGAACTCATTGTCATATTCAAGAAACCAGTTTGAGATGATTTGAGCTTTGTGTTATGGCGTGTTATCTGCTGAAAGTAGTCATCAGAAGTTCAGGACATAAAGGGAT
LG3	ref-5993_17	5.173			TCGCGATATGCATATCACAATGGCATGCAATATCTGAATGATTTTAATAGACTACTTCAAAACATGCAAAAAGTCAAAGTTTTAGGTCGACGCATATTGCAGAGAATAGACTGACCACGCGACTGTTACTTACGTGATGTTAAAAAGAGTTAAGCTCAATAAGTTGTGGAAAACAAATCCTTGCGGTCTCGCGCTGTCTGCCACACACACCGAAGCG[A/G]ATGCACATGCTTTCTGACAGCGTGTGATCCCTTCAGTTCTCTTTCGCACTCGAACGGTCAAATACACACACATGTCAAAATGCCTGTATTGACGAGCATTCATGTAAACACAGTTGGTTATGTCTTAAGTGAACGTTTTCAGTTGGGAAAAAAAATTAGATGTGTGTCAATAGGGCTGCATTTACACTGCAAGTCTTAATGCACAGATCCGATC
LG3	ref-38179	5.797			GAACCACCTCCGAAAGGGCTGCAGAATGACT
LG3	ref-8640	6.03			GTCACCCTCGTTATGACTAAAGACGGAACGGAAGATCAGTAATCATCTGATACACCTTCCACATGGAGATGACGGCCACATGTTCTACAACAACACAGAACAAAGTGGTCTCTTACAGCCAAACACCGCTGAAAACTCAGTAGTACCAGGAAACACAGTTCTGGTTACTCATCACCCACCAATAGATCCAACGAGTTCCCATCTCAAGAACCATACTTGTGCAACAGTAACAACAGTAAGCACAGAAGGTCAGACAGTCTGTCACAACCAGGAGGCGTTACAAAAGGAATGCAACGTAGGAGACAAAGTTTTGCACCCACAGCTCAACGCAGCTATGTCAAACTACCTCTCACTTTCTGCCAAAGAACTTCCTGCCTCACTGGACTGACTCCCATCAGATTATGGACAATCCCTCATCCCAAGTCCAAGATG
LG3	ref-40859	6.516			ACGAGTTGAGCGATTAAGGTGCTAGTCATGA
LG3	ref-42960_28	6.724			AAATTCTGTCATAATTTACTAAACACAGAAGACTTTTGATAATATTCGGAAGCATTCGTCAAGCAAGGACGTCGGAGCTTCTGTTTAGCACATACAGTACATAGAGTGCATAAAATATGGTAAATAAAACATGCATGCTTGATGTGCAAGAAGCAATGAGGATTTTTCTCATGTGTCAAGCAAGGACGTCGGAGCT[A/G]CTGTTTACCATATTTGATGTGCTCTATGTATATCCATTAATCAGCGTTTATTTGTGAATAAAAGTCTAAATTAAATCTGTTCATCAAAGCTTGGATTAAAACTCTTGATTCATATGGATTACGCTTAGAGTGTCTTTATGTGTCTTGAAGCTTGAAAATATTGATTGTGTATA
LG3	ref-42960_29	6.768			ACAGAAGCTCCGACGTCCTTGCTTGACA[A/C]ATG
LG3	ref-18462_23	6.848	Chr1	24192481	CCACAACACACAGGGCTTTCAAGCGTTTTAACGTGCCTTGCTTTGTTCTATCGCCATGTGTGGCAATTGATGGAACAAAAGAGCAACTACTGAGGAAATCAAGCCAATTAACAATCGTTTCAACCATTACTGTACCTGCAAAAATGACAAATGGGACGATAAAACGAAGGGGTTAAAAGCAGAACGGCTCTTCATAGGGAACTGGAGGAACGACAGGGATGC[A/C]GTCTCTGAACAATCCCGTATTAAAACGTGCCCAAAGCCAATTTCTTATCTTGTGATCAAAAGCTACTCCACTCAAATTTGTAGTGTCCATAACACGCCAGAGAGAAAAAGGAGGGAGGTTATATCATGAATATTAATGTACTTCTTACTGCATTATGCATGAGGAGTTGTGTTCCATAATGAGTATGTGTTACTAAGGCGATAGATCTT

LG3	ref-27025_19	7.484			AATCCCATAATGCACCTCAATCAGGGGTGTCCAATCCTGCTCCTCGTGGACAGCCAATGCCAACCCTAATTAAGAACACCTGAAACAGCTAATTAAGTTCCTCAGAACCTCGGCTGCATCGGAAATCTTGAGTAGATTAAAGCAATAAGCCCCAAGAAGCCGTGGTTTACAGTAAATTTATAACAGCAAGGGGCGTTGTTAGGCACGACACGAGCGGA[A/G]TGCCTAAAACCCCTTTAGCTGTTATAAATTCACTGTAAACCACGGCTTCTTGGGGCTTATTGCATTTTAAAATTTGAATAACTAATTACTTCACAGCAGCTAAATAAGTCAGTTCTATGTAGTATCAATGTGTGTAGTATGAATGTCATCTGGACGGACTACATTCGCCATGTTGTCATTACCATGTGACCTACCAGCGTCAGTTACGTCGCT
LG3	ref-27025_24	7.486			AGGCACGACACGAGCGGAGTGCC[A/T]AAAACCCC
LG3	ref-70847	7.897	Chr1	22254137	CGGATCGTGGAGAGCGTGTAAACAGACTGTGGAAAACTAGCTGCCATTTTAGATGACCGTGTATGATAATGAGACCACCGACCCAGACTCTGAACTCTTCTGTCGTACTGAAATGCATTACACCCACAAGTCTCTAGACTTCTCTATGATTCTCTGCTGCACACATTCCTTCTCGTTTAGCACCGATCTTGTTTCTTGAGCGTGAAATCACAAAAGGCGCATCTAATCTTAAAGACTGAGGACGCATCTTGTTTTCTTATGTTTATTTTTCAGTAATAATCTCAGTTATGGGAAAGGTAATCTTCACTAATTACAAAGAAATGGAACAGTTTTCCATGTAAAATGTACAGATTCCCAATATTGCATATG
LG3	ref-8886	8.181			CCTGCACCAGCGAACAATATGCTAGGATGAA
LG3	ref-62863_31	8.81			AGTTTGTGTTCATGGTTTGTTTCATGTCTTGGATTTTGTTCATACTTTATGTTTCATGTGTTTGGATTTTTGGACACTGTAAATAAACTGCACTTGGGTTCTCTCCCCCATCATCTTCATCTGCTTCCACGTCAGCACCATTAAAATTGGATGAGGGATGCAGGATACATTGTTATACTAGTAGGTGGCAGCAAGATTATTAGGCTTGTTCGACTTCAGTGCTGCACAGA[C/T]CGATTGGCGGCTGACTTGAAGCAGTGCATGCCAGTAAGAAATTTTGTCCGACTTGAGACAGCGCCAATGCCATGTGACTGTGACATATGGCTTCAAAGTACCGCAAGAGCAATTCGAGATCGGCCGCCTGAGTCAGCCAGTGCAGTTCTCAAGAGGAGTTGCACGAGCGCAGATGACGACACAGCTGTTCCATGATTGGCC
LG3	ref-13814	9.284			TTCTAATACCCGATGCTGTTGCACTCTATCT
LG3	ref-55080	9.354			GTTCTTTTACCGAGTGCACTGCCTCGTTGGA
LG3	ref-58240_4	9.831	Chr1	16805450	AGCCCGGCTCGATCCAGCAGCGCTACGGTCAATGTAGACGTCTCAGACATAAACGATAACCCACCTCTCTTCTCTCAGGCCAACTACAGCATCATTATACAGGTCTGAGTCCTCAAAGGAGACAATACAGTGATTTCATTGTCAATAATTTCTTATCTTAAGGAAGTCCAGTTCCACTTGTTTAGAGTCATGGTTTGGGGTCA[A/G]TAGACACGAGTCAGATGCATTTCACATTTCATATGCTTTATATTGACTTGTGTCCTAACACTGCAACACTCATGACATGAGACTCTAAAAAAAAAAATACAGTACAGTGTTATTGTCAGCTAAAAATAGTTTTTATTTAAGTTAAACTGAAATTAATTAAAACTGACATAAAAATAATAAAAACTAAATAGAAATTTATAAAATGAATAAAAAAGATATTGAAATGAC
LG3	ref-72812	10.149			CCCTCTTGAGCGAGGCCAATGCAAAGTCAGG
LG3	ref-72102_27	10.329			CCCTGTCATTCTTCTATGTAGTTTGCGTTGAAGACACATTGCAGAGCTTCCATGTCCTACATCAGAACGCCGACTCAGTATTGGCTGGCTCCTGCGTCAGCAACACACGCATGCGTCGTGCTGCTCATGTGAACAGCGTCGGCCAATAATGAGACGGTGTTCTAACGTAAAACCTGGAAGCATTCTTGTCGCTTCA[A/G]AACATTAACGTTGAACCACTGTAGTCATGTTGCCTTTTTTAATGATGTCCTTGAATGTGGTCATTTCGTTGGTTTCTATGAGAGATAAAAAAAAAAAAAAAAAAACTTCTCATATTTCATCAAAAAATATCTTAATTTGTGTTCCGAAGATGAAGGTCTTACAGGTTTGGAACG
LG3	ref-72102_7	10.52			TGTTAT[A/G]AAGCGACAAGAATGCTTCCAGGTTT
LG3	ref-67338_29	10.703	Chr1	18435130	ACTGAAACTGCCGGACTGTTCTGGATTGGCCGTCCTAGAAGAGAGGCACACCAATGAGAGACAGACAGCAGCAGTATTTTTAACCAGAGTTCTGTGTGGAGAAGTTATATAAAACATGGAATCAAATTTTCATGACATTTTGGTCTGGAAATTTGGAAATCCCAATGCTAATCATTCATGCAGACCAATCGTTCTGA[C/G]TGAATCATTCAAAAGAACTGACTCAAAGAATCAGGCTGCACTAATGCAAGTTTCACACAAAAGCAATAAAAGCCAATTAACACTTTCCCCGCCATTGACAAATTTTTCTGGCAATCCGTGTTTTCACTGTTATATGGTACAGCATCTCAGGATTCAAAAACAAGTGAAGAAC
LG3	ref-5423_31	10.712			CATATCGTCCTATTGTCAGCCTGTGAGATCGCTCATACATGATATTATCACACAGGGGTGGGGGTTCAATAGTTTACTTATCTGATCATTACTTGATTATTTACTTATTTCTTTAATTATTTACTTTAATTATTTACCAGAAGTCGCGTAGGAATATTTCACCCAAAAGTCAAGCCTGTGGATCTCACATAGGCAGTTTGCAAATTTGCACGAGAGTTATGCTGGTGAAG[C/G]AGTATCAAACCACAATCAATTATTCACTAAAATGAAAGCAAAAACTGGCCGAGCTTTTGGTATCTGTCACTGCATTAACGGCACATTAACTCTCTCAGAGACGACGAGAGCTGAATCTACCTTAATATATGCTGAAAACAGTAGGCTATATCAACTATCTAATTTATTAGCTCAATTTTTCTCTTGTAGCCCATACATTAA
LG3	ref-43719	10.828			TCTCACACCCCGACACACATGCTGGTAGTAA
LG3	ref-19558	11.016			TGTTGTGCCACGAATGTGGTGCATGCAGGCG
LG3	ref-11857	11.041			GCACACAAAACGAGGCGTTTGCACTTGCAAA
LG3	ref-69699	11.438			TTAAAATGTAATGAAATGCAAAGAAATCAAAATTCATTTTTAATAATAACAATTAAAAAAAACAATCAAGACAAAGACTGAATGTTGGCTTGACAAAGCCTAGTTTGAAATTTCTCCAATTTAAAAAAAAAAAAAAAAAAAAAAATCTTAGATAGGAAGAACGATAGAGCGACAGGGTAAAAAGAATAAAGCAACAAAAGCACAAAGCGACGGCAAGTATGCCTTCACACTAGAATTTGTGGTACATCAAATACAGGTACAGATCCATTGAGCTGAATTGAAATAGTACAGACCACTTGACGGACAGCGAAACTCTCAAGCGCTCAATC

			AGAGTGTTCAGCGAGTGAAAAAAATATCTAGTTGCATGAAAAAGTATGGGATCCACCTGCAGAATCTGTGAAAATGTGAATAATTTTAACAAAATAAGAGAA
LG3	ref-67490_18	11.91	CAAAAACAATTTCTAAAGCTTGGGCTCCTAAACATTACATCGTTCACAACTAAGGCAGTTATTGAAAATGAAATGATCGCAGATAATAGTTTTAATGTACTCTGCTTTACTG<br>AAACCTGGCTTAAACCAAATGATTATTTCGGTCTTAATGAGTCTACTCCACCAGTCTACTGTTATAAGCACAAG[C/T]CTCGTCTGAATGGTTGTGGCAATCTATAGAGATA<br>TTCTTAATGTCACTCAGAAAACAGGAAACAGGTTTAATTCAATACACTTTCAGATATAAGAAAAATCTCTTCCTCTGACTACCATGTATAGACCTCAATGGCATTTTCTAAA<br>CAAAATTTGCAGATTTTCAGTCAGACCTACTTGTCAA
LG3	ref-67490_5	11.96	ACCA[A/T]TCAGACGAGGCTTGTGCTTATAACAGT
LG3	ref-57371	12.334	AAATATCTGACGATATCAATGCATGGGGGAG
LG3	ref-55539	12.906	ATTTATGTAGTATTTATAGTAATTATAATTATAATCATTTACAGTGGTAAACTGTAAACAGACTGCATGGTCACAATTATGAATATGGGACTATACTAATATAAATTTATTG<br>TATTTTATATGTACAATAAGCAGAATAATTGCAGCAGAAAAAAAAAAAATGGAGATAAGATTACAAAGCATGGAGCTCGAGGTGTCATAATGCCAGTCTGTCAGTCAGAC<br>ACAACCCATAGCAACATGTTATGGCAATGTCATACAGTCTGATGGCAGATAAAAATAAACAGTAAATAAATTACTTTTGAGTGATTAATTACAACTGTGCACATCAAATAT<br>TATAAACATGAGTCACTTTTCCACCATCGGGCCAAA
LG3	ref-60903_7	13.293	GGTACTCAACATGTTGACTCTGAGCCTAAATAGTTTCATGAACCAGGATGGACCAGATGGACCGGTGCTTGCGCTTGTAATGTAATGATAAATGATGGCAAAGACCACGCT<br>CTGTTTATCGTTCATCTTTAATGTTAAGCATTTTATGTACTGATTCCATTTATATTTAGTCTTTTTCTTCTAAAATGTTTGTTTGTAGCAGTAATG[A/G]AGCGACAAAGTTGC<br>TGGCAGTGTGAATGCAGCTTTTAGAATGATTTCATGTGAGACTGAAGACTGGAGTAATGGCTGTGAAAATTCAGCTTTGCCATCACAGGAATAGATTACATTTTAAATATA<br>TTGAAATAGAAAACAGTTATTTTAAATTCACAATATTGATGTTTTTTATACTTTAATTAAATAAATGCAGCCTTATAATAAACATAGACTTCTTCTTTC
LG3	ref-39937	13.992	CAGAAAAACCTGGAGAGAAAACTTATGATCAAATAGTAAGCACTCTTAAAGCGCATTTCTCCCCAAAGCCTTTAGTAATTGCAGAGCGATTTCGGTTCCACAGACGAAATC<br>AGCTAGAAGGGGAAACTGTGACAGTGTTTGTGGCAGTGCTAAAAAAATTAGCTGAACACTGCGAATTCGGTGATGTGCTTAACGATACGCTCCGGGACCGATTGGTATGC<br>GGACTCCGCTGCGAGGGCATACAAAAAAGGCTGTTAACCGAGAGCAACCTGACACTACAAAAAGCAATTGAGCTGAGTGTGTCTATGGAATTGGCTGCTAAGGAAGCCCA<br>GCAATTGAGCTCAAACAGTAAAGTGTACAAGATGGAGACCGAAAAACAAACTGAAAATAAAGGCCCATGTTGCCGTTGTGGTAAGACTGGACACTCACC
LG3	ref-23274_4	14.041	TGT[A/T]ACGAAGCGATGGGAATGCTTTTTGTACA
LG3	ref-73276	14.381	CATTTCTGTTCGATGGAAATGCCATTTGTCG
LG3	ref-40162	14.844	CCTAAATAAGCGAATCTCTTGCCGCAGATGG
LG3	ref-37260	15.023	ATGAAGTATACCTGCATGGATTTACAGCGCCATGAAGTTTAATACTAAATATAAAAGCTAAATATAACTGTCAGAAATGACAGTTTTGTTTCTTAAAATGAAATTCAGGAT<br>TTCTATGCACAGATACACACCTACACAGGATCGTAGAAGCCCACCAGGCAGTAAAATCTATGCTTTCTGCATCTACATCGTGCTCTCAGGGTCTTGGCTGACATTAGAGCT<br>GCGGCGTGAAGGTGTTTTGAGTTGTGTCTGTTTGTTGGCAGATGCTAATGAGCTTTATAGAGGAGCTGTGACTGCAGGAGAGCTGTAGGATCAGATACAGCCAGAAGGCA<br>GTGAACAAAACCAATAACGTACATCCTCGCTGTCTCA
LG3	ref-45089	15.38	TTTTGCTTTGCGACACAATTGCAGCATATAT
LG3	ref-28744	15.568	GTGTATGCAGCGACATGTATGCAAAAGTACG
LG3	ref-24093	15.886	GATGGAGTGTGATTGCACCAGAGATCCACTGTCCCCATCAGACAAGAGTCTTACTTACTTCAGACACCACCTATGAATGACTGGATCTTGATCTTCAGACACCTGATATTAT<br>GCAAACATTTTAATATCTTTGGAGTTCTAGATTAAAACAAACACAGTTCACAAATACACAAGAGGTTGCACTTCATTCGTATTAGTCTGATCTTGGATTAGCATACCAACA<br>ACATATGAATGTTAAGGGGATAGTTCACCCCAAAAATAAAAATTTTCACATATTTTACTCACTCTCATGCTAAACAAAAGATTTTTTCTTTAACCTTATGGAGCCATTATTT<br>TTCATGCACACCAAAATAAGATTATACGAAACAA
LG3	ref-59466	15.992	TTTTAGGCTCCGATCTGAGTGCTGAGATACT
LG3	ref-2486_32	16.265	ATTTTTTAGCAAGTACATAACCAGCCAGTGTTCAAAACTATCTCCTTATCTTAGCTCGATTCACAATGGTAAGCTTGTAATAATGTTTTATAATAAGAGCGACCTGGTGGAT<br>TTCCACGGGAAATTTGAGCATGCAGCAGTTCGTCTCTGCGTCATTATGTCACGTCTGTAAACGGAAAGAAGGAGTCCAGGCTAGTTGACTTTATCATGCGAGGAGGATGCT<br>GCTTGTAT[C/T]GGATCATTTATAGCCTGTTCTCACAGCAGCTGAAATAATTAAACGTATCATTTTGATGGTGGGTTGTAATCCAGAAAGGTTCAAATGACAATCATCAGTG<br>ACAACTGGAGATCCTGTAGTCAAAAGCAAAAGGCTTTGGACTGTGGAGTGGCTACAGAAATTGAAATCTACAGGTAATGATAATACACACTAAATACACA
LG3	ref-41754	16.459	AATTGCTAATGGTTGAAAATCTGGAGTAGAGTGGACTGACTACAAAATGACTTGGAAAACCCTTCCCAGACTGATGGGCAATCTTTTTGTAAGGGCCTCTAAGAGCCCTTT<br>TAATCTCAGCATGATGTTCCACACTGAGACCAAATCAGACCGCCTGATGTTCAAACATGGCAGTGTGAGCAAGTTACTCGCAAATTATGTTAGAATCCTTTTAATCCTTAAT<br>AGATGAAACATCTGCAATGATTTTGTTGGAGATATAAAATTATGATAATACCACAGTTTTTTTTTTGGCCATCCAGTTTCATAAAGACCGTCATTAGTTTTCCAATCTTTATA

					TTGTATCTACACGAATGCAAAAATGCAACCATT
LG3	ref-36832	16.726			GTCACGATCACGATTCAGTTGCGGGCGCCAT
LG3	ref-34900_5	16.794	Chr11	30214956	CTTTCAGTACAATTTTTTTCCAGCTGATAAACAATAAAAATAAAAGAATTGCAGAGTCCACAACACAACTATAAAGATAATGGCACTGAGAGATGATATCGCTGAAATCA<br>CTTTCAGTACAATTTTTTTCCAGCTGATAAACAATAAAAACGTTGAGAGCCAATCAGAATCCATCCTGCTTTAAAGAGCTCGAACATTTAAAGC[A/G]GCAGACGACAAAA<br>CTGCAATGCAATTATAATAAACGGAATGTTATTGTCTGTTAATGTGGACGCTAATATAGTTATCGTAATAAATATCTAGTTATTTTTCTTGGTGTAAACGGGCCTTTATAGA<br>CAAAAAGAGAAGAGTGAAAGGTATGTGCATGTTTGTTTGTGAGTGTGCATCCTTTTGTGCATCAATATTTTGCTTGCAGGGTGTTTTTATGAATACCTCCTA
LG3	ref-40882_24	17.048	Chr1	12190672	TAATGTATTTACCAGCACTACACACGACACGCACACACCTTAAACAAGTGCAAGATGGCATATGGAGAGCTTGTATCATCAGGGAGCTGTAGTGAAGGCATGTTAAACTC<br>ATGACTCCCATGCTGAACATGTTCACAGACATCTACCGCCCAGGGCTGCGTTTCCCAAAAGCACTGAGAGCAAGTTGATCGTG[C/T]AGAATGTTGCCACCAATGTTCTCTA<br>TATGATCAACTTGGCTCACAATGCTTTTGGGAAACAGCCTAAAACAGATACATTAGAGCCTCCTGCAACATGATTTGAAGTTTTAACTTCTCTTCAGGATGACATATTACCC<br>ACGTCCAGCCAAGCAGAAAAAACTGTACCCAGGACGACA
LG3	ref-14167	17.39			TACAAGATGCCGATGCCGATGCCGCGGCCGC
LG3	ref-35419_2	17.567			AAAACTATTAACCTCTCCATTGTTAACATACAGAGCTGTCTGAACCACTGCCAGAGGGAAATTAACCCCTTAAGTCCAATTTCCCCCATCAGAGGTTTTTTTTTTTTTTTTT<br>TTTTTGTGGTATGCTCTATTTTTCAGGTATAAAATGAACTGTGCATGAGCTGCAATGTGCACCAGACTTTATGCCGATAGTCAAACGC[A/G]TGGCTGTGCGAGACTAGTGC<br>AGCTTTAAATTCTAAAGGCTATAAGCTTCCTCAAGCATAAACAGGTAGGTTCGAATTGCATGATTTCCCATTTTCTTATCACACATTTTGAAGGCATTCAGCGTGCACACCC<br>AGCTAGTCCCAGAGTCCCCGAAATTCTCAGACTCCTTATAGTCGCTCCAATTTACATGCATCCTCTGTTCCCAAGGCGTCTCCTCCTCGTCTGTACAA
LG3	ref-12743	17.735			GGAATGCCCACGAGAGATATGCATTTTATTT
LG3	ref-51944	17.887	Chr1	5038313	ACAGATCTGTCCTTTCATGTAAGGAGACAGACGGGGAGAGGGGCTACAGACAGCCTCCAGAAGAGATTTCAGAACTGGCTGCTGGAATGAACAAAGAATAAAAATAAGG<br>TTTTGTGTCATTTTAAACAGTTTGTTCATCCTAAAATAAAATCCATAAACATGATCCAAATGATTGGGCAGCATCTTTTTTGTGAGGAACACACTGAAATTTAAGTCATTAA<br>CAGTAAGCTGTGACTGGATTACTGGATTGAGTCTTTGTAAACATTTCAGATCAGGTTTCTTTACTGATTCATTCAAAAGATCTGACATCTGACTGAATGATCTGTCCATACT<br>GAACAGTGCCACTTCTCTCACAAACTTAAAGGGTTA
LG3	ref-457_1	17.964	Chr1	8764407	CGTCAGCAGAGAATCATAGTCACGATGATGGACAGGGCCAAAATCAGACCTTGCTTCACATGTTGGGTCACAGTCGCCTGCGTTCAGACAGACAGACTGCAAAGTGCTGG<br>ACGAAACCACAACATTCAATCGCACACAAAACAAAAATACCAAGAGAGAAACTTCACTTGCTACTGACGTCAGTTTGTGCACAAACAGAC[A/G]TCTTTAAAGCGATAATT<br>ATGCCAGCGCCATGTTAGGCTTGGCAACCGGTCCTTCTTCACCATGATTTTGAATTATCGCTTTGGAGAGTCTATATACGTGTGAGTGACAGTGAGACACCAATGACAAATT<br>GTTCATGCTCTCTGACACTCGCGTCAATGGGGGTGATTCACAACCTTTTAGATCGAAAAAGCAGAACCGCTTTGAATTACACAACAGAACACACACATCTAA
LG3	ref-30010	18.828			TCACTTATACCGATACCGATGCAACACCTCA
LG3	ref-61595_17	19.152			AATAAGTATGCGAACC[A/C]CTTGCAGAATCTGTG
LG3	ref-61385_3	19.721			TGTCTTTATAGTGCGTACAATCGCTGAGCTTAAGAAAGCATACAGAGCTGAATCAGTTATGTAATAGGTGTTTGTTTCCGACTTGCGCTGTAAGTGGTAGACCAGTCACAA<br>CAGACTGGGCCGTCTGGCCAATCAGAGCAGAGCAGGCTCATGGAAAGGAGGGATTTAGAG[C/T]GACTGAAGCATCGGAGTCGTTTGAGAGTCATTGAAAAATTTGGTGC<br>TGTGCAATGTATATTATGAGAAAATGAGAGTGTTTTTTGACCTTTGATGCATGTACACCTGTTGTAGGAGACTCCAAAACTAAATTAAGAACCTTTAAAATAGCATAATAA<br>GGGCACTTTAAGGAGTTGAGACCAAGTCTTAACACTCATGG
LG3	ref-61385_8	19.759			GACTCTC[A/C]AACGACTCCGATGCTTCAGTCTCT
LG3	ref-55934	20.083			AACAGGAGAAAAGAATGAAAGAAAATCTCAGTGCATCTCAATGAATTACCGCAGAGTACACAAAAGAAACATGTCTCTAATAGACCATCTTCTCTTTAAATTTAAAAACA<br>TTTATTCTATCTAGCCAGGTTTCATATGTGAGTTTCTCATAGCATATGTAAAAGTCAGGAAGTAAACATGCACTTGCATCGCCGAAGCTCACAGGCACCATCTTGTGCACAT<br>AGCACTGATTTATTTAAATTATGTCATTATAGATCGGTGGCACCTAGCCAATATAGAGCTGTTTATTCAAGATGCCATCAAAGGAGCTACCCAAAGATTAACATAACCGAT<br>GTCATTTGCATATGCAGATTGATATCTAAAGTAGTG
LG3	ref-53893	20.177			ATTTGGGGAACGAAATACCTGCCATGCTGAG
LG3	ref-16986	20.28			AATGTAACTAAAATGTATGCCTTTATTTAATCAATTAATAATGTTTATTTTTGTACCTTCACAGTCATCTCAGCCACAAAGATCACAGTGAAGATGTAGTTGGATACTTTAA<br>GAAAAATCCGCTCCTGTCAAACAATGATAAATGAATAACGAAAATGTCATTTATAAAGGCAACAAATGCACCATATTCGTTACAGAACAACACAAAACCAGCTTTGAGAT<br>GTTGCTGATGAGAAGTCACCCAGTGACTTTTTAAAGCATTTATTGTGCAGCATATTTTCATTACGGAGTTATAATGGATATTGTAATTTCGAAGTTGTACAGTATGTGGGGT<br>AATCTTTTTTTGATTATGATAATGGGTTTATAAGT

LG3	ref-65222_27	20.763			TTATTGCAGTGATTATTATGTTTCTATCATGTTTGGCAACAGTTCTTCTTACCCTAATTGATGGAGTGTGAAGCTTTTCATTTCTTAAATGACATGTAGGAAGACACATCATGGCCATATTCCAGGATGACAAAGCTAAGATTCATCAGGCTAGATTGGAGATTTTTTTGCAGGGAGGCAATTATTAATATTATTATTGATTTACTGAGACGAACCATGTGCATTT[C/T]TGCTTTGACAACAATTTAATTAAATTACAACTACTAAATGATGCATCATGAAATTTGATGCAATCTGATTTCACTTATCACGTGACAAGAGTAAGGCGAGATGACTGACAAGACAATGTCGGAGGATATGGTGCACAACAGATCCTGAGTGTCATTGATGTATATCTTGTATTATTATTTTTTTTTCTCTCATCTCATTTTTTTC
LG3	ref-14922	20.843			GACCGTTATTCGAGTGTCTTGCTGATATACA
LG3	ref-57766	21.273			TAATGATGACAACGCACACATTGCGAACTGCATGCTTTCAATCACAATTCGTTATCGATTAGTGAAGGCGGGATCGGCGGAATCGCGCTGAATGGCACAGACCGTATTTATTTATTAATTTCTTTCTTTATTTTTTCTGCTATATTGTACCACCATAAACAGCTGCCATCTTTCAAAATACAGTGGCCTCGAAAAGTATTTTAACACTTAAAATATATCATTATATTTTGGAGAGTGACTTAAGTCTCCAAATTAATTTTAGGGGCCACTGTACATTTTAATGATAGTCAGAATAGTCAGATACATTTTTGCAAATAGTTCTTCAACTTAGGCTATTATATATTCACATTTACTTCAACAGCTTTTTAT
LG3	ref-64989	21.45			AGAACCAGTTCGATATCAGTGCAGTAAACCA
LG3	ref-13830_23	22.557			GACGTCTGCGCGAACGGGGTGC[A/G]CGAGGGTAT
LG3	ref-13830_16	23.072	Chr1	55427561	ATTCGAGTCACAAACTGCTCCGAGTATTACGTCATGGGGTTTCCTTCTCTTCCAAATTACGTAAATTATGGTGTGAACGTAGCATAAGCTCATTGTTTTGGTTCAGAAGGAACTGTAAAACATGGTTGCTGTTTTGGATGCAATGCTTGTGACTGCTGTTGTCTTAACTCTGCTTAGCATGAAACGCACTGATGACGCATGACGTCTGTGCGAACG[A/G]GGTGCACGAGGGTATGTAAAACCTCAGACCGAGGGATATGATTGGACAGGGCTACAGGGCTGTTGAATGCTTAATTCTAACTGTTTGATGGACATTCTTAGGTGTGCAATTATTTTTCAGGAATTAATTATTACAGTTTTACAGTTCCATCTCACTTCACCAAATTATTTCAGCTATTTCAAAAAGAGTTGTTGCCTGGTACAGCAAACAAACCA
LG3	ref-3044_3	23.162	Chr1	2692214	GCTCTCTATTTCAGTAATCATTGTTGTTTATTTCACAAAAGATCGAATACAAGTACAGAAATCTGCAGGCGATGACACAAATCAGATTTGTGCATTGCCCTACTAATCCAAATGAAGCTCAATCTCTCTAAAAATCTACATTAGCACTCACACCAGCATCACACGCCCTTTCTTCGGACATCAGGCATGTTCGGCCAGCATC[A/G]TTAACCTCGATGGCACTGCCAGTGAACTAACACGGCAAAAGTCAACACGCCATCTGTCATCTAGAAAGGCACCTAAAAAAGTGGGTAAAGAGGAAATGTGAACCCAGTAGTGGACTGCATCCTCTGAACTTTGCACAGTACTACAATACTAACAAATTTTACAATACTAATACTTTTATACACACATTGGAAAGCTAATGTGATATAGATAAACTTGATTAATCATAAT
LG3	ref-7317	23.533			ATTAAGCGGTCGACATTACTGCACACCATGA
LG3	ref-68220_31	24.782			AGGTTGTGCTGCTGCTAGCTTCGGAGACTAGTAAGTTTTTTGTTTTTTTTGTTTTTTCTCCTTAATTCCTTGTGGAAAGTCATTACTGCATACATTTAAAAACGAGCATTTTTTTATACAGTTATTCCCCCTTCAGAATTGACACTCATGAGTGAATGACTGATGCTCATCATGAACCTCTTCAAAAGCAAAGCCACACGTGGATCTTCTCGACACATTTGCCTGGCCAT[C/G]TGCTCTAAGTCCCAGCTTTACTAAAACACCTGCTCTTAACCGTCTGTGCCTTCAGAACTTTGACTGTTGTTTTCTGACCTGTCTGCTGAGCCGAGAAGATGTAAAAATGAAACTTTCTTCTCTGAATTTGAATCTTTAAATCTTCTCTAACATATGCAAATACTTTTACAATCAGGGACAAGATGAGAATATATTACAGGC
LG3	ref-60844	24.787			GATCACGGTTCGAGAGTGATGCATATTAATG
LG3	ref-22970	25.936			GTTGCCTACACGATGCTGATGCTAGATGTGT
LG3	ref-56439	26.591			AAAAGAAATAGAAAATTCATTTTACTTCTACTGTGCTTTTTGTCCTTTTTAAGCTTGGCAGTTGTGAATCTTATTGAAAATCTGATGATTTTAGGGTGTAATTTTTCTACCTCTTAGTGCCTCTTAGTGTAATAACTTGGGGCACATTGAAGAACTGAATGCAAGTGCGTCAGACATGTGGGAAGAAAATCCATATTTGAGCATTCAGAACGAGCCACGTGCTGTGCAAAGCCTGGTCTTCGTTTTCAGTTGTAATTGGCCTATTCTTTATGGGAACAGTTTTAGGCTATTAAATACCATAAGCAGGGACTGGATATGCTGTGTGCCTATGTGGTTTGTGTCTGATGTTAAGATGGAATTTCAGGCTTTATTATAAAGTATAAATTATTTATAAGAATAATGCACATAATTTCAGTATGCACTCAATTATT
LG3	ref-54571_25	26.888			TTAGCTTGCTGCTTAGTGGTTGTTAGGGTGTTCTGGGTTGTTGTTGAATGGTTACTAAGGAGTTCTGAGTTTATTTATCCTGTTGCTTAGTGGTTATGAAGTTGCTAAGGAGTTGTTGCTTAGCGGTTGCTAGGTCGTTACTAAGGAGTTCTGAGTGTATGTATCTTGTTTCTTAGTGGTTGCTAGGTGTCTAGCACAAACGCTGCATTACGACGCACATGCTC[A/G]AGGTTAACACTTCGAATACAAGTATCCATTAGCAATCTGCTCAGTTTAGTTCTTGTATATTTCTGTTTCTCTGCCAGATTTAGTGTGATCACAGCTGATGCAACTGTGCATTAACACCCCTCTGAGTGTCTCTCCTGCTCTTCCTCACAGCTGAACAGAGATTTCACCGCGCTATTATCTGTTTACTAACAGGACGGCTGCGCTGAA
LG3	ref-12242	27.473			AACAACCAGCCGAAAGTCTTGCTCTCTGATA
LG3	ref-45256	27.849			GTACAAACCTGCAAACATGAAGACCTGAGGCATTTGCAGGCAAGTCTCTTCAACCTAGTAACCAATACTGTAAAAAAGCAATTTATTTTGAAATTACACACCTAAATTAAAAAACAAAGATAACCAAACTTATGTTTATTTAAAAGATTCTAGCAAACGTTCAATCACAACTGTATAGACTCATAATTTCACTGATTATCGTCTGTCTGTTGTCTGATAATGATCTCACTCATATCACAACACAAA
LG3	ref-18287	28.056			GATGCTTTTTGTTTCAAAAACACATGGCATTATTTTACTTCTATATATTTGTATGTAACATTTTGGTTGCTAAATTATTATTCACTATGTAAACAATTAAGGCTTTGTACTTCCAATATCCACGTAAAACAGCATCCTTCACATGCGCAAAAATGTTAGTTTGATGATGAATGGCTGAAAATGCTAGAACTAATGCTGTGTGTGTGTGCGCGAGACGCCTGCGTG

					ATCACTTGGATTTAATAAAATAAAGATATAAAGAAAACAATCCGTTAGAGTGTCACAAAAACGAGCATCACAGTTTTTTTTTTTTTTTGTTAATCTGTTAATTATTTAATTTA<br>ATTATTAATATGTCGTGTAGTTATTCCTTTTATATTTATTCAGTTTTTCTCGGTTCACAGAAGCGCTGAAGATGATGTGACGATGTGTGAGTCC
LG3	ref-44660_32	28.842			GCACGAGATCCGATTTTTTTGCATCCGATCT[G/T]
LG3	ref-68258_19	29.532			GTATAAAATCAATATCACGTTTTTAATCGTGCTCATATTCGTGAAAACAGCTCTCTTGCTCTTGTGATATTGTTTAATTCTATCCTACGTTATAATATAAAAAACAAGATCTC<br>ATAGAAGTACTTTTTTGTAATCTTATCTTAAATTTGTGGGCATTTGACTTTTATTGAGACAATCCTGCACTGATT[A/G]CGATCCCGACACAACAGTAACAAACGAAATCAC<br>ATTTGAAATAAGTTATTGATTGCATTTTAATCAGTAAGTTTTACATCAAATCACATTAGTGATTTCCTCGAGGTTGTGGTCTTGTCCGGTCTTAAGATAAAATCCTGAGTCCT<br>CTTATTCTGAGACCGAGACAAGACCAAGACTTTCT
LG3	ref-44660_2	30.771			G[C/T]ACGAGATCCGATTTTTTTGCATCCGATCTG
LG3	ref-29274	31.79			CTGTCATGTCCGAAGAGGTTGCCCTTTCTCT
LG3	ref-20652	36.29	Chr5	18135213	TGCGCAATTCACTTTTACATGGTGTGTGAACACTGATGTGTGTCCACAGTGTGGGTCAACAACCAGCCTATAATGGTAAAAACCCACGCACTCCTTTTTTTTTATAATCTCC<br>ATAAATCATAAGCAGTGTCTGAAAATACACTGTTCCAGATTCCCACAAGGCTTACCCATGACTGGTGACAATCTGCTCTATCATCATAGATCCCATCCTGAGTGAGCTGCA<br>CACAGTCCGCCATGTTTATATCCTCGCTATAGCACCTATTTATATAATATCTGCGCCAGATAAACATTACAATTTTCTGTTTTTGGATGTACCAATGAACATAAGAGTCTCC<br>ATAGACTCCCTGAGCCACTGAGAGGACACAGTGGATGAATTGCAGAAGAATTTAGGTAAAGTCTTGTATGTTTGTGAAAATAATTTTATGCTTCGC
LG3	ref-36722	36.609	Chr1	5768103	GTTGGGTAAGCTGGCAGTGAACATCCCTCTACAGTGAGCCATTGTGTTGTTATGCAGCGCACATTCGACACAGTGCAACAGGCCTGGTGTGGGGAGATTCCCAGTGCGGCT<br>CTGAGGAATCCGACGCAGCCAGTTTCTGGCCAAAAGACGAGGAATTTTGGGGGTGTAATGTGGTGATTGCACAAAATTCTTTGGCGGTGTGCTCACATGCAAGTTATAAAT<br>AATTAATAGGTGATTTCACACATTCATATGCCTTTTTCATTAAATTAACGGTATTTTTCTGTGTCAAGTGTGAACATGGCAAATGTTCCATGTTGATATACAGTAACATAA<br>GTTGCCTGACATGTTTCTGACAATCTCATACATTT
LG3	ref-12825_28	37.789			ACATGTGTTCATGAACTTATTTTATGCTGCTCAGATGCAAATTGGGCCTGTGCACACCCTGTATTGAACACACACACACACGACAGTGTATTCAAGCACAGAGATCTGTAG<br>ATTTCTCATGTAATGTGAGTGTTCTGTAATTAGTTTCTGAACAGAACTGGACTCAACAGCTCATTAATGCAGAGATGTCGCTCAG[C/T]TTTGGACTCTGAAGGTACGTGTCT<br>CAATTCCCTCACAAAGTTTTGACCCAAACCTGAGGAAAGCAGTCTGTTTCTCCACTGTAGTTTCAGTCTGTGACTCAAAGCGTCCAGATGGGCCTGTGACAGAGAGCGATT<br>GCAGCCACAGGATCCTGTGTTTTTTGTTTCTTCCTGCC
LG3	ref-61595_27	38.333			AATAAGTATGCGAACCACTTGCAGAA[C/T]CTGTG
LG3	ref-50036_32	38.438			TGAAACGTGCCGGCCGACAGCCCCACCTGCTGGCCAGAACAGGGCTTGAACACTACAGTGAGCTGCTCACACAGAAGGAGCTTGTGCTTTTAAAACGCGAACAAAATGGA<br>CAAGAGCGCGCAGGGTCTCGAAGATGTGTTTGAAAAGTTGAACGTTTTAAAAACGTGAAGCTCAAAATGCAGACGAGGCACAAAAAATCCTAGGCTACGGCGAACGATG<br>TGCAAGAATTTAA[A/T]TTAGTTCACGTTATATGAATCTTCAAAAAGATCACAGGCTTTAGACAATGAAGTACTCACCAGTGGCAGATTACAAGAGGACAGTGCGACTGTT<br>GTTTGCTTCCGAGTTACTCTGATGTGGGAGATGAGGGAGATGATATTCTGAGGTGTGACTGAAATTCGCGGTCTTTTTTGTTTGACCGTTAATGCGCGCCGTAT
LG3	ref-50036_8	38.585			TAGGCTA[C/G]GGCGAACGATGTGCAAGAATTTAT
LG3	ref-59183	41.306			CCGTCCTTCTCGACAATGATGCTGAATTCAG
LG3	ref-23393	41.643			GCCAGATGAACGACTCGTTTGCTCAGAGAGA
LG3	ref-50845	43.21			CTCTGACTTTCGAAGCTGCTGCCATTTTGGT
LG3	ref-21075	45.628			AATGAAAATTTTGCTTAAGGGAACACTAAAGTCTGTTCTGCTCTATTGATATTTCATGTGGATTTTAATGTAGCATCAGCATGTAGCATAAGGTAGTATTGCAAGGGCTGCA<br>CAATTAATCAGAACTGATAATGTTATATGGCTTAGCAAAGTGTGTGATTTAATTAAATATATATTTAAAACATAATAAATGTATACGCTGCATGTTTCCGAGTGAAGTGCA<br>GCATTATTTTACATGAGCATCTCATGATACATTTATTATTGGTTTGTTTAACTTGACTCCAATAGTGTAAACTCCATGTGTTAATGACGTATACTCCCTGTTGACTGATTGTG<br>TTCATTTTGTTTTTTTCTTCTTTATTTAGTAACCGCCACCGTCGGACTGAGCAGGAGGAGGACGAGGAGCTGCTAAACGAGAGCAGCAAAGCCAC
LG3	ref-43108	46.494	Chr1	54072875	CTAAACACAACTAAAATCTTGTGGTTGTTAAAGACTCACCTGCCCCCTCATTACTGAGACCTGCTTCTTGAACTGTCCTCTGTCGAAACCCGGATCTTTCTGTCAACGTTAA<br>TGTACATATAATGTCAAAACTAAGCATTAATTTAGTAAAATAGTACTAATAAAAGACAAACACATCAGCAAGTCCCTCGCTGAATTTCAAAGTGATCTCACCTTAAAAAGC<br>TCATATAGATCTTCTTCAAGGTCTTTGATGAAGTTTGGGTCGGAAAATTTGGGAAGAACTAAATCTCGGATCTCTTGTGAAAAGGCAACTTTAGCCTGTGGCAGCCATGCC<br>CAGTAAAACGGGTCTACAGGAAACACATTCAACAA
LG3	ref-28876	49.944			CATGAGTCAACGAGAGATGTGCTCTAGTGAG
LG3	ref-42757_3	51.24			CC[A/G]CCATGCTCGAGGAGAGTGCATGCCAAAGA

LG3	ref-10416	52.259			TCTAGAAGATCGACCAAGATGCATCTTAATA
LG3	ref-8493	54.019			GGGGAAAAAAATGGCTTTGCATCTACAACCACCATTTATTAACCCAGTAAGTGACCAATTATTTTGAAATTGATTATTAAATTCAGGTTAATTGAAAATATGCTGAAATGTTATAACATGAACAATATAACACTTGTAGCTGTGTGATGTATGGCTGTATATCAGCACGGCTGTGATTCGGCAGTAGTGTCGTAGGTAATCTCAAGTCGACAGTTTAACAGTTGAGCCCAAGCCTCCGTTACTTATTCTAAAACATCACTTTAAAACTAGTGATGAAGGAAAGCTGAGTCGCTTCATTGAAGCTTATTGTTTTATGTAGAGTATTATGAGAGAGATCGACTAAACGAGTATTACCTGCATCTGATATAGC
LG3	ref-47198	54.941			TTTTAGTCATCGATTGTCGTGCCGTGCGGGT
LG3	ref-96_26	56.092			TGCTGGGATCCGAACACCATGCTCT[A/G]CACACA
LG3	ref-96_4	56.092			TGC[C/T]GGGATCCGAACACCATGCTCTGCACACA
LG3	ref-42757_16	59.771	Chr8	22411683	AGGAGCGCCTAGGGAAGCAGTCAGTATCCATCGTCTTTGAGGGACTGTTCCGCAGGCAGCCCGAAGCATGGCAAGGAAACGCAGAGTCTCGTTCCCTGTTCTCAGGGAACCATGGTTACATACGTAACCTGAGACGTTTCCCTTTCAAAAGGGAACTCAACTCTGCGTTCGAAAATGCAATGGGGAACGATATACCCACGCCGCCATGCTTGAGGA[A/G]AGTGCATGCCAAAGAATGGCTGGGACAAGAAAACAGTTTCCGTAGAAACTGCAGCAACTCCCCCTCGGGTCACACCAGACTGTCAGTGACAGCATTCCCTATGGCCAACAGCCCAAGCTGAGCCTACGGAAGGCCCTCCAGAGGAAAAAGGCCTACCAAGACCGCTAGAGGCATCTGAGACCAACTTTTCCGCAGATAAAGTGGTCCCTTTAAGG
LG3	ref-2101	59.964			CCAGCACATAAGGGACCTGGAGGATTTCTGCCGCAGATGTAATGCCTGTGCAGCCTATAAGGGCCCTCTTGATCAGTGCCATGCCAATCTTCAGAAGGCTGTAGGAGCATCAATGGAAAGAACAGCTGTGGACATCATGGGCCCCTTCCCCTTCACAGACAGAAGTAACTGTTTTGTGCTCACTGCTATGGACTATTTTACAAAGTGGCCCGAGGCCAGTGCGATACCAAACGAGGAAGCAGAGACGGTGACTGATGCACTGCTGGACGGGGATGTTCCAGACGTCATTTATAGTGATCGGGGAGGACTTTTGAATCCAAGCTGTTTGTGGCCCTGTGTGAATGGCTTGCCATGGAGAAAACACGCACAACACCCCTGCACTCCCAAAGTGATGGCCTTGTTGAAAGTTTTAATAGGACCTTGGCACAGCA
LG3	ref-12342	61.973			CGAAATAATACGAGTCTGCTGCAACTTAATG
LG3	ref-17482	62.469			TCACTCCCTTCGAAGTAGCTGCCTTCACAGA
LG3	ref-40629	63.109			GACCAGTCTCCGACCCTTCTGCATTGTAACA
LG3	ref-43727	63.212			ACAGAACATGTGGAATTGTTGTGCATGGTGACTGTGCTCAGATTTTATTCTATACCAGCGAGGAATGACGATCTGGTGGATCTGTGGAAGTTCTGAACACCATCCTTGCCATTTTTGTGCAAGATCTGTGGGCAGCTTCTCATTCCAGCCAAGGCCTCGTATCCACATCTCTTGGAAAAGGCATTTCACTTGCACAGTGAAATCCAATCACAATCCAGGGCTGTCTACTCTTAAGAGAACTCTCATCGCAGACACATGAGTGGGACACACCACTGCCTGAAAACAGACATGAAGAGTGGACACAGTGGTGTCATTCGCTGCAAGACCTAAGAAGTCTGAACATTCCACGCATGTATGTATCCATTCCTAT
LG3	ref-32321	64.227			TGGACAGATTCGATGATTGTGCTGCAGTGGA
LG3	ref-18655	64.386			GTTTTTGCAACGATGCTTGTGCGTAGGGCTG
LG3	ref-14183	64.416			AGGGCTATCCCGACAACATTGCTCAAAAAAG
LG3	ref-71587	64.573			CCTGGCCCCCCGAGTGGCCTGCACCTCCATG
LG3	ref-67528	64.801			AGTAAGAATGCGATTTACTTGCTCCATTGCC
LG3	ref-47499	64.83			AATGTGAATGCGATGCATTTGCTGTCTCAGA
LG3	ref-58878	65.593			GGCAAGATGGCGAGCTAGGTGCACATGTTTT
LG3	ref-48102	65.741			ACAGGTTATCCGATGCTATTGCCTTACAAAC
LG3	ref-58122	65.901	Chr1	54028063	ATGTTCAGATTTCGTCGAGATATGTGGAAAACAACTGATGTTGATTTAAGTTTTGCGACCCTACTGTATAGTTGATATGCTGTAAATAAGTGTAATTCTGGAGGTGTTTGTAGGGTTTAAAGACTCGTTTGGCGAGCCCTGCCCACCGCCCGGGGCGCGAGCGCTGCTGCCATGCATACCTGCCCTGAAACACACATCTGTGCCTCTGCACGAACTCGCTGCTAATGAATGACTCGGGTTGTTTTGATCACCTTGATTCCCCTGAAAAGCTCATTTGCTTAAAGCACAAGAGATGCAGTTTTAAATTCATTTTCTATGCATTTGTGACAGAAAATAATTTAGGCCCCTCCTTCAGTTTGGAAAAAAAAATCGCGTGTATTCACTTTCAATAAAACTAAATGGGGCAAAAGTGTAAAGTAAGGGGTTTGAAG
LG3	ref-57593	66.311			TCCTCTCCAGTGTAGTTCTTGGGGTGGGTGGAGTTAAAACGAAAGTGAAAGAAGAGCTACGCCATCCAGAAGCCATTATGTTTAAACATCGTTTTATTTAAACGGCTGCATATTTTGTGGTAAGTTCATAAATTATTCTTCATGTAAACTATCAGTCATATAAAGCTGTTATTTAGGTTAGTGTTCCTTATGTTTCGACAGTTAAACCGTCGAAACATATGCCCAGCTGTCCAAACGAACACGGGTATTTCCAAAACTGCAGCTTTCATGTGGTTTGGCCGTTAGTCCACACGCAAGCGCAGTATCCGGTAACTGAAACCAAACTTTTTTGAAAACTCTGGCCAGGGTGAAGATTTTTAGAGATTTTGGCCAGTGACATTGTAGTACGCGCTGTTATCTCATTTTGTTTGCATCTCATTTTTTGTCCTTTG

LG3	ref-59013	66.93			GACTGAACATCGAGAGAGATGCAACAGAGAG
LG3	ref-37027_9	67.941			TTGAACCAGTGTGGGGTCTAGGGCCGCTA[G/T]GCAAGCCTGTCGGCAAGCACGAGGTGGAACCCGAGGTTTCAGCCAAGACTGTAGGGGCCGCATTTTCAGCAGGCCCA
LG3	ref-72369_6	68.559			ATCGAAAGCTCCAGTCTCTGTGTTTAGAGTGAAAGCTCATGCGATTGCCGGCGGCTCAACATGTCCAGTACAGTCGTGCCATGTACCAGCTGCTTCAACACCATTATTAGG<br>TTAACTGTGAAAATATAGTTTCAAACCTGTATGACTTTTTTCTTTTGTCGAACATAATAAGGGCCGTGACATCCCAAGCCGACCGTCAGCCAAC[C/G]TCACCGACAGAATT<br>GCTAGTTGACAGTCGCCTGTAGTCCTTTTGCTGTGTTCAAGCATAGATTTTTGGCTGAAACTCCACCGACTCGAGGTGACAACACGGTCAGCTTTTGTTGCCGCTAGTTCTTT<br>TATGTCAACTTGGTGTGTCACAGCCTTAAAAGAAGATATTTTGAAAAATGTCTTGGCGGGTTTTTTCGTGTTCATACAATGAAAGTCAATAGGGTCCAA
LG3	ref-20345	69.354			ATAGGTTGCTAAATCAGTGTATTAGTGCCTAGGTTCTTAAAGCCTAGGAAAAAAAGGCATTAAACTCTTTGTGGTTCTGATTGTAACCTTTTTTTTTTTTTTTACAAATTGAA<br>GGTGCCGGCCAGAAGTTTAAAGGTTTAAAGAAAGATAAAGATATTTACTAGAAGCTAAAATGATACTTTAATGTACTGTAAATCAATTTCTCAAACACGAAGAATATGCA<br>GGTGCATCTCAATAAATTAGAATGTCGTGGAAAAATTCATTTATTTCAGTAATTCAACTCAAATTGTGGAACTCGTGTACTAAATAAATTCAATGCACACAGACTGAAGTA<br>CTTTAAGTCTTTGGTTCTTTTAATTGTGATGATTTTGGCTCATATTTAACAAAAACCCACCAATTCACTTTCTCAATAAATTAGAATACTTCATAAG
LG3	ref-34588	69.7			ACTCATCGCAGGGAGGAATCATTCCTTTGCCTTGTTTTCAAAAGTAAGACAGTTTTCATTAATAAACAGTTAAACTATTAGTGCATGGAGTACTTATTTTGTACTCTTTTGTA<br>TTTTGTAGGAAGTTGGAAATGAGGTAGAAAAGCCCAAATCCAATCCAAGCAGAGAGATTTTGACATTAAATGACAGAATGATTGACTGCTGGATGTCTGAAAGTGATGCA<br>AATTCATGGAAAGCCATTACGAAGTGAGTTTGTCTCAGTTTTACACACATTGCTGTCATTGACATAATTTTTCATGCTGTTGTTAAGGATTTATGTGCAGTATGTTTATGAGT<br>TTATGTACAGTAAATGTCCATGTCACTTTTGAAATGTTCTTTCTTAATATCTTTTATAAAATAATAAGTGGAATTTGAGGTAATAAATTCTAAGC
LG3	ref-43039	70.122			ACACTAGATTCGACCAGACTGCAAGACCACA
LG3	ref-69197	70.182			CTGAAAGTTGCGATTGGTGTGCAAATGTACG
LG3	ref-24641	70.531			CTGATAGCCTCGATGGCAGTGCTTCTAGATG
LG3	ref-36424	71.019			AGATTTAATGTCTTTTTTTTAGTTGATTTCATCTAAGGCTGTGGCTGAAAGTCAGTTGTAGTGTAGATGGAAACTCTATATGCAATTCAAATGCATACATCTTAGATTTACAC<br>ATAAATGTTTTTTTTTTTTTTTTGGATTGTGGTGTTACTCTTTCCAGTCTTAGTCAGTAACACAATGCAATTCTGTCGTCCTGATTCTAATGAATGTAAAATTATACACAAAAT<br>GATAATTTACATATGTTTTATTGTTGTAGGCCTACTACACACATAAAGAATTAAATATCATTTGAGGTTTGTGACTATATTTGAAAACAAAAACAAACAAACAAATTATATT<br>GTTTATAATGCATTATAAATTGAAACAATA
LG3	ref-23661	72.086			CTGCAGCAGCAAAACAAACTGAAAAACTGTTGGCCGAGGCATGCTTATATTGTCGGAGTAAATGCCAATCATTTGCGAAAATCACGTTAGCCAAACCCCGTAAGTCACAT<br>CACCAAAAACAGCAGGCTAAGAGACAACACACCCAGATAGCAAGCAGTAAACAAACCGCGCAAATGATAGAGAGAGCGGTAACTCACCCATCTGATGAGCCGAAAGTG<br>GTGCACAGGGTCGAGAGCTCGCAGCAGCCCGATGGCAAAACTTGCAGGCAGTAAAAATTGCAGTCAACAATCAAGTCCAAAGCACCAACAGTTAGAATTGGAGTAATCT<br>GAAAAGCACAGTAATCCGATGGTGATATAGAAATCCTAATAAAGCAGTAATCCGATCAGTGCAGCAAAACATACAAACAAACAATCCAGGTTTGAAGCGGCAGC
LG3	ref-63191	72.448	Chr1	56489066	GAAGAGCTGCAGGTTCAGACACTCACTGATGGATTCTCATGTTTAGAAAAGCTCTGAGGATCAAGAGTCCTGCAGGAGTCTTCAGGAGCTCTGGGATGAACCGTCTGTGTG<br>AAAGAGAGCCAGAGATCGGTCATGTGATCATCTGTGATGGATTATCTGACTGTGACGTCTCACTTACTGCGAGTGTGTCGTACAGGTCAGAGGTCGTGGACTTGAGCTCAA<br>GAGCTGTATATGTGTCTTCATTGGGATCAGATTTTTTATAGTGTTTCCATTTTTATGGAAAAAAGATCCCTGACAATGTTCAAGTATAAAGGACTTTTTCATGGGACGCTGA<br>ACGCTTTTCTTGGCTTCTCACCACCTTTCATGTGT
LG3	ref-34232_25	73.104			TCCGTCAAAACGACCCTAATGCTA[C/G]ACTACTG
LG3	ref-62764_8	73.195			TTCAGGT[A/G]GACGAGGTGGATGCCTTATTTTGT
LG3	ref-21385	74.779			GCTGATAGGTCGACTTGTTTGCAATCACATC
LG3	ref-12116	75.729			TTGCTAGGCAACGCTAACAAGCAATCAAACAAGTGACAACAGCAGACAGACATGCAGAGTTGAACACACAGACCCATGAACCGTGAAAGCTGTGGACACTAAATGACTG<br>TATTCGTCGTCTGACTGAACCGTGACGTCTGTACCATGACAATTCGAGATGAATACATGAACCGTTACACCCCTAATAAATATATATGATGCAAAGATGTTCGAAGTGGTT<br>GCTAGGCAGTTGCTAAGATGTTTTTGGTGGATTCTAAGGCATTGCTAGGGGGTTGTGAGGAAATGACAATGGAACTGGACTTTGGGGGCATTTTGATTGTCTGCTATGGGA<br>AAAACACTCTTGTTCTCTCGCTGTCCTTCTTTCTCCTTCCCTTACAACAAACACACTCTAGTCTATAACATACATTCTTAATATTTACAGTCCGTCAGTA
LG3	ref-1316	76.235			CGCAAACATCCGAAGCATGTGCCCAGAAATA
LG3	ref-71023	76.69			TGCAAATAGACGAATGCGCTGCAAAAGCCAT
LG3	ref-50551_2	76.697			C[A/G]GTTTCCCCCGAAATGCATGCAAGTGGCTGG

LG3	ref-64259	76.851	Chr1	47884711	TCACTTTACCTATAGGTTATGATTACAGTGTCACCTTAAGAGCTGTTTGTGGCTCGTCTGCATATACTGTCTCATGTCGCTTGGCCTTATGGGTATTTCCATCCTCGAATATT<br>TTCAGCAGGTTAAGTGAAGAAACGGATGTATGATTTGACAGTAATTGTTATTTAAACAAAGGGAGACATATCTGTAAGACAGGCTGGGGGAGACTGATGATACCAATGCC<br>CACCACCACTTAACCACTTTTTTCTTTCTCCTCTAGAACAGTCATTTATGTGGAAGATGAACATGTGCAGGTGGTCCCACATCTCCCCCTGTAGAGAGCACATGGTACTGCA<br>ACTGATGCACTGGGGAGAAAGTTAAGGTAAGTTTCAGCAAACTATCATACATCATGTTTAGGCACATGATGCAATCACAGCGGTTTCAAGTGGAAT
LG3	ref-68767	77.753			TGTTGTTCTAAAGCCACATGACCTCCTTTTTTTNNTGAAACAAAAAAAAAAAAAAAAAAAACCCTGCTTGCAACTGTTTAATAGCAGTTAAACTGAACATAAAATGAAATGT<br>GAATGGTGATGTTCTTTAGGCATCAAAAAACATTCCCACACAACTTGTACCACACAATTCAAGAGTTCACAAGGCATACGACAGGGTTTGATGAGAAAGTGACCAAAGTG<br>CAGTTTATTACTGAGTGGAAATCTTGACTTTGCATATATGAGTTTTTGAGAACAGCAGTTCAGACTGAGAACTGTCATTCTGACCGACAACAAGAAAACATCTTTTCCTTTC<br>TTTTTTACAATTTTATTTATTTTTTGTGGCTGAGCACCCTCATTGAGCTTTTTTGCCCATTAGTGCATTTTGTAAAGACGTAGAGCTGTCAAGCTCCT
LG3	ref-54080	77.928			CGATGACAGGCGAAACAAATGCAGGAGATGA
LG3	ref-68474	78.802			GAATTTATGGGTAGGGTTATGTTTAGGTGTAGGTATATGGCTATGATTAAATTTTCGGACAGGAATGTTGTTCCAGGATCAACAAAATATGTTGACCCAGGAACGCGTCTA<br>AATCTGCAAAATTAGAATGTGTGTGGAGTGAGGAGAGCAGATCTCTGGCAGAGGGTGTAGCATGTAGAAAGGTGTAGCTGAACAGAAAGGACTGGTCCATGAGGTTTCTG<br>CTGGTTGTCTTGAAGATGTTTTATCAGTTTACCATAAAATAGTAGGCCATTGTGTGGTACGTATAAGATTCTTGTTGTTTTTTTCTATGTGTGTGTTTGTGTGCTGTAGTGAA<br>TAAGTGCAGAAGTGAGGAGAATGTTGGGATTCAGGGATTTTGAAAAAGTGGTTGTTGCAAGTGCAGCCTTTTAACAACAACAACAGAAGAAAAAAGG
LG3	ref-13280	79.329			GTAGAATCTGCGATTTGTTTGCTGGTTTCCA
LG3	ref-54093_1	79.534	Chr1	40023493	GACATTCCATGGACTTCGGCCAAATGACGAGCCTGCTCGATTAACACAGATGTAATTGTTGAAGAGACCAGATCGGCCTTGTTGCAAACAAGGGCCCGGGGTATATCTGG<br>ACCAAGAGAGTGCTGACGGCACTCGTCCATCCACAGTGGCAGATTGCGGAAACTAATGGGGTTAGTGACATCAAACACGAAGACAATCCC[A/G]TGCACATTGCGATAGT<br>AGTGCTGCACCATGCTTTTACGAAAACGCTCCTGACCAGCCGTGTCCCACAGCTGTACCTAAAGTGAGATGGAAAAGGACAAGATTTATATTTTTGTACACATTCAGGCTA<br>CATATATATTATACAGATTTTTTTACTCATGACTTAATAACCTTGGCATAACAATTAAGCTACAGAAACATTAAAAATTTAAACTTGAGTGTCAATTGAGTTAT
LG3	ref-52601	79.702			GCGGACCGTACGAGGCTTATGCTCATTTACA
LG3	ref-16325	79.773			CGTTCGGGTTACGGAAGCCGAGAGATTTTTCAACAATGGCTGTGTCCCAATTCAGGGGCTGCATCCTTCGAAAAATAACCATTAGATTGTAAAGTAAGAAAGAAATTACA<br>GTATTTTACACCTCACAATGAATATCAGTCAATTTTATTATGGTTAATTTTCCTAAATAAGACATCCTTGATGTCGTATGCAGCCTTCAAACGCAGCCTCCGAATGAGATGC<br>AGAAACTGTGTGTTACCCTTTAAAAGCCTCACATAGGCTTTTTAAAAATGTTGAGTACAAATTAAAATGCCAAAAATAATGTTACCCCTGTTTTTCTCCCTTGTGTAATACC<br>ATAAGTAAGTGCAATAATCTTTGCATTGTTTTGTAAAGCATTGTAAATTGGTATCTGCAAGTATTATAATACATTGCATATACACGTGGGACACCTT
LG3	ref-24950_3	79.984			CT[A/G]GAAAACACGAGCACATTGCACCGCATGCG
LG3	ref-6829	80.11	Chr1	9841741	TTTTTTTGCAAACTTTGAAAAGATTTGCTTCATGTTTGGATTGAAACCCAGCTAGTGTGTGAACAAAACCACCCTACAATGATAAAAATCCACCCACTCCTTTTTTTAAATC<br>CCCATTAAACCAAACCAGTCTCTTTAGACATGCCGTTTTGATTCTCTGAGCAGCGTGACGTCACACTGCCCAGGCCTCGCCAAACGGCCGCCATTTTCTCAACAGTTGTAGC<br>GACAACAGTGTCTTGTAAGCAATCCTGGTGTTCTGTTTTTGGATGTAAGAGTCAGCATAAGACTCATTTTCTCAAAACATCTGAGCCACTGAGGACGCAGTGGATTATTTTT<br>ATGTTTGAAGATAATACGCCCCAAAATCTACCT
LG3	ref-4618	80.505			AACTCACAACTCACAACTCATTACGATCTTCTATGCAGGGGCGGAGCCAGGGGGTGCTGTTTCTGTCTCCTTTTTTCTTGACAAGATTTGCGTTTATTTAAACGCAGAACCG<br>TCTCTTTAAGACCACAAAAACGGGAGACTTTTCAGACACATACTCCAACTTCGCCTCAGCACCGGGCGCAAGTCAATCGCGTGCGGAAAGGATACAGGTACCGAATGAGC<br>TTCAGCAGAACAACAGTTGAACGGGGAACAATATATAGGCTAGCCTACGTCAATGTTTCTCAACTGGTGGGTTGCAAGACCGTTTTGAGTGGGTTGAAATGTGCAGTCAAA<br>GAAACAACAACAACAACACTCTGTTTTCTGATGCC
LG3	ref-44774	81.735			CCTTTGACAGTTTGTAAAAAAAGGATACCAAAGGACACCATATGGTCAGAACTGCAATTATTATCTTTTGTGCCTCTCAGCTAAGAATAGGGAATATTCTTTTCTGTGCCTA<br>ATATCACCACACCAATAAAACAATACCGTTCCAAGCGAGTTCATCCAGTAACGCAGCAACAATTACAGCAAGTGACTCATTGGTGTGAATCTCTCGCTCATTAAGTTTGAA<br>TTGCAGAGACAATGGTAATTTTAGCCCCTCCGTAGCTCTCATATCAAAGAAACAGGCTCTAATGAAGTACTGTTCAGAGAGTCTCACATCTCACCAGTGTCGATTTGAATG<br>TGATATGAAGCGAAGGGTGCGTGGAAGAAGTGCGT
LG3	ref-55450	83.379			TGAGGGCAGAATACGTCGCTGAAGGGAGCATAACACTCAGGGATATAGACAAAGCGTTGGTCAACTGGGCTCTCGATGGTCGTGGCACAGACAGGAAGAGACTTGGAGC<br>GTGGAGAACATGGAATTGGAAGCCCAGGGAAACAGTTGTGGAAACAGGTTTTGCCCCACTTCAGGACCTTGCTGGTTTTCCAAGAGAGAGTGGGGTTGTGCTGCTCCAAC<br>CAGGAGCACCCTAGGATCATGTCAGCGGTGGATCCCTCCAGAACCAGCAGATGGAGGTGTTCGACGTGTAGAAGG

LG3	ref-62112_29	83.648			GGCATACCAATACACAGTCCTTCCATTTGGACTCTCTCTAGCTCTCAACCGTCTAGAAATGATGGTGGTAATTCTGGCCTTCAAGGAGTTCCAGATGGTCCACTTGGACAACAGGACAGTGACACCTTCATAAACCACCAAGGAGTTATCGGGTCTTGCCCTCTGCACAGACTCTCCTATGGGCACAGAAGAACCTCCGCTCATTGAGGGCGATTCACATGCCAGGCT[C/T]CTTGAACATAGGAGCAGACATTCTTTCACATGCCGGACAGCCCCCGGGGGGAATGGAGACTTCACCCCAATGCGGTCAATGCGATATGGTGCACCTTCAGTTGGATCAGGGAGATGTTCAGAACCAGCCTTGGTTCCCAGAATTGATTCAGCTGCTGTCAGCATCTCCATGGCCCATACCGCTGAGAAGAGTCCTCCTCTCCC
LG3	ref-50226	84.863	Chr1	34253847	GCCAATATGAGACATACAAAAGGCTTTTCTTGGTCTACATTTTTTTCCTGTTTCCTTTATTCTTTTAGGGTCTGAGCAGCATTGTAATCCAGTGATCAAAGTGCCACGAAACACAGTGTTTAAAGCTTGTGTTATGACAGAGTTGAAGATAAACTGTACTGTGAATCTAACTGGATGTCACAGAAATCCAAGAGTAAAATGGTGCAAAATCGATGGAAATGCCTGTAAAGCTTTAAATCACTCAAATCACATACAAACTCAGTGGAAGAATACAATAGGGAATGAATGGATAGTTTTCCTGATTTTCCTGAACATCTCAATGGAGGATGCAGGTTTATACAGATGTAAAGAAAGCGAGACGTCTGTAAGCCATACTATTAATGTCACTGTGACATGTAAGTGACATTATCTTTTATTTCATTGGCAGCATG
LG3	ref-28111	84.936			ACTCTGGCTCCGCCTTTGACGGTTGTCACCTCATTACCATCGGCTCTCCGTCTGCGCCAGCAAATCCATCGGCTCCGTCAGTCGTCCCCCAGCTGTCGTCA
LG3	ref-9997	85.661	Chr1	33683429	TATGCATGTGCTTCTTCTTGGAGACTTTTTTTTGCAATCATCTCATATGGATGGCTCTTTCTTGGAAAGCAGTGTGTCCTCAATTCCTTCTGAAAATGGACCGCCAGGATGTGAATCTGCTACTTTGGAGGAAGAGTTTCTTCAGCGTTCCCTGAACAATGGCATGAGTGACTCTGATGAGGACACAGGTTATGCCGATCCTGTGCTGGACGATCTTTATGCACGTCGGGTTCTCATCTCCGAGCATCAGACCTCCGTCAATGCTGATTCAGACAAGTTCCTGCCCAAGTATTGGACCCCTGAGGAAGATCTACATGTGAGAAAGATCAGGCTAGGGTCCCAGCGCAGGCCATGGTATAAGAAGATGCAGGGCTTCAGGTTTGTCTTATAATAGAGAGANNNNNNNNNNNNNNNNNNNNNNNNNNNNNNNNN
LG3	ref-17831_3	87.231			CT[A/G]AGCTCAGCGAAAGCCTTGCAACTAACTTC
LG3	ref-50593	87.942			CATCTGATGCCGAACACAGTGCAGTGCTTAA
LG3	ref-59448_3	90.15			GGAACGGCAGCGGAAATGGTCTTGTTCGCCTTCAAGGAGTAGACCCACTGTCTCCTTTAGAGCTGGATGCCATGGTACAGATGTGGCCAAAGCTTTGTCTGTACACTTTTCCTATGACAGCACTGCTCCTGGGAGTTCTAGTGAGGGTTTGCCAAGACCAAGACCACCTAT[A/G]ATTAATGGCACCATTTTCGCCAGCCTTAGTCTGGTTCTAGGATATAGTGTCTCTCCAGGACAGCACTCCTTGGGAGGTTATCTGGAGAAACCTCCTGTCTCAAGTGCATGGTTTGATCTATCACTGCCGGCTGGAGCTCTGGAAACTTTGGGTCAGCTCATAGATTCCGGTCTCTCAACTGAGGTTGTTGAGATGATT
LG3	ref-1238	91.121			GTGCTTCCTTGTCGTTCGTTCATCAACTGCGCTTTATTTTTCGTGAAGCATTTTGTTGCGCGTCAGCTAAGATAAACAGACATCCCTCGCATTGCGTGTGTAATCAGTGAGAGTTTTGCAGTTAAACTACTGCACACTTACGATTACTAGAGAATGCGGTGTTTAGCTTCATTAGTAGTGCATTCGCTTCGCATGATCGATCGAGCTGGGGTTCGAGACTGACTTGGAGCAGGGTGGTTAGGATTGGAGGATGAGTTTTGGAGGCGGAGCAATGAAGAGAGGGGTGGGTTTGTTAGGGTTGATTTCAAATATCAACAGTGTTCAACAACGAACTTGAGAAATCGCATACAGTACCTTTAACATGAACTA
LG3	ref-29957_4	93.685			ATT[A/T]TCTCTACGATGGCCATGCCCCGCATGGG
LG3	ref-29957_9	93.685			ATTTTCTC[C/T]ACGATGGCCATGCCCCGCATGGG
LG3	ref-52603	95.127	Chr3	20964756	TATGATTTTAAAAAGATAGATTGATAAGTAAGTTATTTTGAAGAATGTTGATAATAAAACTATTGTGTTTTATTGTATGGATAAAATAAAAAAAATAAAAACACTGAAGCTGCGTCCAAAATATAGTATACTATATAGTACGCGAAAAACAGTATGCGAACAGAGAAGTATGTCCGAATGCATAGTATTCGTAGAGCAGGAGGTGAAAAGTACTCTGATGAATCACTACTTCTGGTGAGATTCTGAAGTGCGCATGCGATGGACACTTTACTATCCCATTAAGCCACGGGAGACGATTTGTGAATGGAGTTAAAGGGACGTAACTGACGCTGGTAGGTCATGTGACAGTAACAAAATGGCAGATGTAGT
LG3	ref-32008	95.495			ATCGTCTTTGCGATGCTCATGCTTACTGTCA
LG3	ref-5206_4	96.817			TGA[C/G]ATATCACGATTCTTGTGCAACCCCGGAT
LG3	ref-7354_7	97.718			GAAAGC[C/T]TGCCGAACATAATGCTGAATCTGCA
LG3	ref-57180	98.143	Chr1	48468953	ATCTTTTGTGTTACATGCAAGAAAAAGAGATCTAGAATGACATGAGTAACTCATACCAAGTCCTGTCCCTTTAAGATGCATTCTGTAATCTATTTAAACCCTGTGATATTATTACATGTCAGTTTAGCCATAATTAGCTAACAGTGTGACATAACATGTAGGCATGATTGCACTAATTGGCAGACACTTCGCATTTAGAGTAGTCTCAGATGTAAAGAAGTTGGATGGCAGTTCCCAGAGCACATCTGGTGTGGATATAGTCCCCTCACACTAAAATGCCCACAGGGCCACCAGTGATTACTTTTCAGGAAACCATGGAGTATTACCTGGCCTATGGCCATATACACTTCCAAGGCTTCAAGTGACAGT
LG3	ref-73373_30	98.367			AAGGGGAGAGGCTGAGGTTGTGGCCCTTCCCTTTTGCCACCGGACAGAAACCTATCCTCAAGCTTAGTGTTTGGGGATCTCGTTCCTGTGGCCAGTCTAGCTGTAATCTGGCTACAGCTCGAATTACTACCTCGAGTAATTCCTCAACGGATTTATCATCACGTGAGGAACGCTCAGAGGCAGCACACTCGTAAAGAG[C/T]ATCGTCATCCCCTGCGTAAACTAAAGAAGCACCGGAGTGCACTTTAGGATTGCGCGAAGGGATGCATGGATCAGGTGACAGATCGGACGAAAGGGCAGGACCCGTCTCCTGCTCGTCAGCCAAATCCATTCGTGAGCCCCACGATCGTACTGTGGGTGCTGGCCCTTGGAA

LG3	ref-37188_14	98.738			ACGTGTTGAACGA[C/T]AGGTTTGCGACAATACAG
LG3	ref-36794	99.152			AAAAAGCGCTATATAAATAAAGGTGACTTGACTTGTAACAGTTTAGTTAAATTCATGCATAATTTGGGAAAACTCGCCATTGGTTGTTGCATCAATGCACACATGATATTG<br>TGGGGTGGAGCTACGTCATCAGCAGCAGTCTCATATCTCTCCCTGCTGACTTCTCATGCAACATCAAAGCACTGTACTCGTGTTTGTCCCAGTTTTGTCCCAGGCCCAGTAA<br>AGCAACTTTGAGTGACCATCCACCTGATCTCTGTGAATTAATCCATCAACACACACACACAGTCACATTGGTGCCGTGACTCACTGTTTCTTCATTCAAGATGGTATCTGAA<br>GCGGCCATGGGTTCATCTTAAAGTTTTCGCTGGA
LG3	ref-7354_23	99.867			GAAAGCCTGCCGAACATAATGC[C/T]GAATCTGCA
LG4	ref-68806	0	Chr8	3018918	TTTTAGGCTGGTGGGTGGAAGGGGGGGTGGGTGGTAAGACAAGGTTTAAACCAACAAAACCAGAGAAAAGGCCCTTTTCACTCCCATGGATAAATCCCACTAAGTCTACG<br>TTTCCACATCAGCTTTACTTTCCCCCGACAACAATCCAGCGACTCAATAAAATGATCAAGAACAAATAAGCGAGGCCTTCGAGAGGACGGGGCCACCGGTTTAGCAAATA<br>GACCATGCCGAGCTAATCAGTGGAGAACAGAGGAGCTGATACCATATAGATATTTGGGAAATTTACACAACATTAGCGTTCTCATCTAATCCTCCATGACGTCCTGAGCTG<br>CAACTTCTACAGGAGGCCAAACTGGGTGCAGCACAGGG
LG4	ref-67961	3.246			GATAGTCATTGATAGCTTTGCATGCACATGGTAGACCCTTGGGTTTTAAATGGCCATGCTATTAAAATTATTTATATATTTCACGTCGGGTGCTCATGGTTACCTGCCAGAA<br>CTGATAAGTTAACAGCTGTCAGTGTCATGGATGTTTTGCAGCGCAAATTCTGACTGAACACACGGCGATCTCTTTTTAAATTAACCAGGCAGAGGTTGCGATTCAGCTGCT<br>GAATGTGTGTGTGTGTGCGTGCGCGCAATTTCTTTCAAGGAAAAAATAGATAGACATATAAATTTCCTACCTCAATAATAAAGCTGTGAATTTTCAGTGCGAGTGCATACA<br>GACACGTCTGCTTCATAATGCAACATTAAAGCTACCTTTTAGTAAGCAAACGTGTCGCCCTCGCCTTGTGGTGGCTTGGAATTCCAATGGGAATCGG
LG4	ref-34976_25	13.793			GTCTTTTTTACGATATAGATGCAC[C/T]GGCACCA
LG4	ref-34976_19	14.42	Chr9	45028831	TGTTGACAATAAGTACGTTTTTTTTGTTTTTCAGTGGACACTCATTGGACATTTTACCTAAGTGCCACGGATTGGATTCATCTCGTGATCGCCATTTCTTTATAAAAGTATGA<br>AGTCCCGTATGATTTTTCGTGTTGTCATTTGAACACCCGACACAAATTACAATTACTGGTGCCGGTGCATCTATA[A/G]CGTAAAAAAGACACCGTAGATTGACAGTAAACC<br>TTTAGAACTTTCTAATGATAAAACTTATGTTTATTAATATTTTAGATAGTATCTAAGATAGATCGATAGCTCCTTTGCCTGCTAACATGATCATGTCTACGGGTGACGTCAC<br>GGACACTACGTCCATATTTTTTTCAGTCTTTGGCCA
LG4	ref-44704	15.265			TATTGCAATGTATTTGGACAAATTTCATGATGAAACCTGAAAAGTGACCCACAGTCTCTGTTCAACTTTCTTTCCATATGCATAATCTAATCTAGCTCGTCCAGTAACTTAA<br>ATTTGTGAATAAAAACAAAAACAGAGCCAATTAAATAGCTTATTATCATTAAAATATGAAAATTAAAATGATGGGTAATAATAGATTAAGATTTTTTACAACCCCAATGCA<br>GAAGTCTTAAGGCAGGAACACATCAAGCCGATGGTCGGCCGTCGGGCGGGTTTGTTCGTCGGCCGCCTANNNNNNNNNNNNNNNNNNNNNNNNNNNNNNNNNNNNNNN<br>NNNNNNNNNNNNNNNNNNNNNNNNNNNNNNNNNNNNNNNNNNNNNNNNNNNNNNNNNNNNNNNNNNNNNNNNNNNNNNNNNNNNNNNNNNNNNNNNNNNN
LG4	ref-4200	20.397			TCGTCTGCTGAAGTTTTCTCTCTCGCAACGGCCTGCCTCTGTGACAACAGCAGGTGCACGATTATTCTGCAGAGTAAAGCTCAGGGAAATTGGCAAGAAATATCCTGGTC
LG4	ref-50703	22.814	Chr8	4867866	GCGTAGTTCTCTGTTTTACAATCAGTTGTTTAGACTCTGGGGGCCACATCCACAGGCTTTATCCACTCAATGGCCCACGATAGTTCCTGTTGTTGTTTCTCCAAAAGGTGCCT<br>GCAAAAGGCTGATTTCCTAGGCAATTCACTAAGAGCTCCCTTGAAACAACTTTCAAGGCTGTGTGGGTGCTTTACTCGTTCTCGGAGAGGTGCTTGGCGGTGTAAATGCAG<br>CCGTTTGCCCCAGTGCACGTGAAGGAGGGGTCGTGCTCTGGAATTTATTGAAGGGGGAGGGGCATTGTGTTGTTAAACGAATATCCCCATATAAATGACCCACCCAAACA<br>AGTGCCCTGTAATTATGCAGAGTGTGTGTCTGCGTTTGGTACAGATGCTGGAGGGAGAAACGGGAAGGGGGTAGTGGGGTGCTCAGACGTTATGGCC
LG4	ref-11729_6	24.073			TGTTTGAATACATGACATGATGGATGCTGTGCACATATTTCTACCCTGTAATATATTCAAATTACCGTCCATCCTGTCATTTATTCAAATATTTCATTACTGCGAGAAATGGA<br>AAGTTGTTGATTTGGTAACAGCTCATGAGTCTTTAGACTCTTTTGATTTATGACTCATTAAAAGGACTGGTTTAAAAGAGTCACTTGTTTGT[A/G]AATCCGACCACACTGCT<br>TGTGTCTGATGATTTTGATTCACTGAAGAAAACTGGTTCATAAGAATCATTTGTTTGTGAATCAGACTGCTTTCTTGGTATAATTTTGAGTACAACCAAATTAATTCTTATGT<br>AAAAGTATAGTGTAGAATTTCCCTAATGTTTTTAAACACAAAAATTAAGACTTTTTTATTGGTTTTATAATTGGTGTTTAAAACTAGAATGACTTT
LG4	ref-30057	25.168			GTTAGTCAAACGATTGCTGTGCCGAGCCCAC
LG4	ref-32189	27.724	Chr8	10375956	CATTATCAAATGTTTAGTGAATGTTTTTTCGCAAGCTGTTTATAAATCTTAAATATTTCATTATAGTAGTATTTTATTTTTTATTATTATATTAATCATTTCAATTATTCAAGT<br>TTTTTTTTTTTTTTTAAATACCACACTGAATGAAAATTGCCTTTCTATAATGCTTTTCATTTGTAGGTCAAAATGTCACATGATGCTTGCATTGAGCGATTCAAGCGCTGACA<br>CATGTCATGTGAAAGAGCCTTAACAAGATGATGAAATAATGATTTCATGTTCTAAATACTACCTTTGTTTTTCTCCATCAGGACAACTACACGTTCGCCCAGCCTGGCATTC<br>AGATTAAAGTGAAGGCGCTGGAAGAGCTTGTCAGCAGAATAGATGGTAAGATCATGTTCACAAACATATCCTTTCACTCTGCATAGAGAAAA
LG4	ref-63244	29.15			GCCGGTAACTCGAGCAATGTGCAAGAACCAC
LG4	ref-27521	29.423			AATTACTCTTCGACAGCGCTGCCACAGAATA

LG4	ref-13167	29.481	Chr8	10273246	TATTGACAACTTATCTTGCACTCGGGGGCGTATACAAATATTTACCCAATAAAATGCTCTCTCAAACCACCTGATTCCATTTAGCAATAGCAAATAGCAAAAAAAATTTTTT TGAAAAAGCCCTTCAATATGGCCCACCCAAAAAAAATATGTCAGGAAAGAAAACTGTGAGTGTCATTAAAAATCCAATTAAATTATCCTTCTGGAACCCTACTGTCATGCC ACATCAAGGTTAAATATAATAATAAATTCTCTGCCAGTGATCAGTTTCAGATTGTGCTCAGATTCAGAACATTTAAGGTGAATCTTATCAGAGAAATAAGCCTGATTTGTTG ATTGCTGTTTGTCTCTTATGTACCATACAACCCAGGACAGCATGGCCCCCAGTATGGGGCGTCCTGACCTGATCCCATCGTGGGCCCATAAATGCT
LG4	ref-9956_24	29.609			TTCTGCAGATTCATTTCAGCATAGATAAGTGTTGACAGAAATGCATGAGGACGTCACGGGTTGCAATCTTGTAATTACAGATAGGACATGGTGTGAATGCAGCATAAGCTC ATTGTTTTGGTTCAGGAGGAACTGTAAAAGGCAGCTGCTGTTTGTGGCGCTCGGTGACTGTCTGTCTACAACTCTGCATAGCCTTATGGAGCGTCTGCACGAGCAGAGTGC A[C/T]GGAGGTATGGAAATGCATATATTGACGGGCAGGTAGAACTATTGCATTCGGACCAAATGCAATGATTGGACGAACATTTTTTGGTTCTGACACTTCCACAGAAGAT ATATGTAGCCTATATGTTTTAATATTTAGACCACTTATATTATTGTTTGCTATCAGGATGTGAAGAGAGTTTCAAAAAGTGTTTATAAAGCAAATTGCCTAC
LG4	ref-55054	29.813			CATAAGGGAGCGATGCTGATGCTGATGCTGG
LG4	ref-12468_14	30.983			TTTGCTTGGGCGA[A/G]GAGCATGCCCGCTCAGTC
LG4	ref-25314	31.643			TGACAGGCCACGAGGCTGCTGCAGATACAAT
LG4	ref-62627	32.205			GCTCCGATGCCGAATGCGTTGCTGAATGCCA
LG4	ref-32792	32.282			AATGGCATTCAGCAACGCATTCGGCATCGGAGCACATGCTCGAATGGCATTCAGCAACGCATTCGGCATCGGAGCACATGCTCGAATGGCATTCAGCAACGCATTCGGCA TCGGAGCACATGCTCGAATGGCATTCAGCAACGCATTCGGCATCGGAGCACATGCGCGAATGGCTTTCAGCAACGCATTCGCCGATGGCGTACGGCATGCCGATGGCGTA TGAAAATGTTCTTGGCGTCGAAACTTCACATGCTTGGATGGTGTACGGCAACGTGTTAGGCGTCGGAGCACATGCACGAATAGCATTCAGCAACGTGTTCGGCTGTAACTG CTGTCTGTAATTTCTGATTACTTTGAAATAAATATGTT
LG4	ref-35825	32.72	Chr8	13968644	ATCATGGGAACAACGAAAGCAATGGTTGACTGTTGTAAACCTGCGAGTATAAGCCCATAATGGTTTCTGCTGCCATTTTCATATTAGGGATATTAGCATTAATAATGCTAA CATTTTTCTATCCCGTTCAGGACAAAAAGCATACTTACGGTGTGCCATGTTTTTTGCTCATGTCTTTTGCACTGTCTTTGTGCGACTTGTTCAGATTCTCCTCTCTCTCTTCAC AGGTTTCGTGTTATAACCTGCCAAACTTGTCATCTAATGAAAATGTCCTGCTAATTGTTGCTGCTTGAGTTTCTACACATTAGAAAATCTTTCTTTCTATCTGCTGCTATGTC ACAAAACAGCTGTAGTTTTGGACACAATCTT
LG4	ref-17707	33.325			GGTGTACGGTCAAGAGTTTAGCGTCAGAGCTTCATATGGACGAATGGCATATGGCAACATGTTCGGCATGGCAGCACATGTGTTGATGGCATTCCGCCACATGTTCGCTGT CGGACCACATGCGCAGATGGAACACAGCCATGTGTTCGGCGTCGAAGCTCATGTGCGAATGGCATTCAGCAACGTGTTCGCCTTCAGAGCACACGTGCGGATGGCAGTCA GCCATGTGTACGGCGTCGGAGCACATGCGGCGCTGGCCTCCAGCCATGTGTTCGCCCTCGGAGCACATGCGCGAATGGGATTCAGCAACGCGTTCGGCGTCGGAGCACCT GCGGCGATGGCGTCCAGCCATGTGTTCACCTTCGGCGC
LG4	ref-71648	33.884			GCATTCAGCAACGCATTCGGCATCGGAGCACATGCGCGAATGGCTTTCAGCAACGCATTCGCCGATGGCGTACGGCATGCCGATGGCGTATGAAAATGTTCTTGGCGTCGA AACTTCACATGCTTGGATGGTGTACGGCAACGTGTTAGGCGTCGGAGCACATGCACGAATAGCATTCAGCAACGTGTTCGGCTGTAACTGCTGTCTGTAATTTCTGATTACT TTGAAATAAATATGTTTGTTGCAGGCAGACTGAATCAACAAAAGCCAGCCGAGAACTTCAACACCATCACAATGCCTCGAATGCCTAAACAATCTTGTTTTCTATTACCTT CTAACAGGTTTGTAAAAATGGTAGATTCACTTTTC
LG4	ref-48756	34.165			CGTCGGAGCACATGCGGCGCTGGCCTCCAGCCATGTGTTTGCCCTCGGCGCACATGCGCGAATGGCATTCAGCGCACATGCGCGAATGGCTTTCAGCAACGCGTTCGGCGT CGGAGCACATGCGCCGCTGGCGTCCAGCCATGTGTTCACCCTCCGCGCACATGTGCGAATGGCATTCAGCAACGTGTTCGCCGTTGGACCACATGCGCCGATGGAGTCCAG CCATGTGTATATGGCTGTAACTGCTATCTGTAATTTCTGAATACTTTTAAATAAATATGTTAAGCATTTTTTGTGTTTTCATTTGCTTAAATGTGTCTCTACCATGCATGTGGT CTCTACCACGACCACATGCAGAGATGTCACGTA
LG4	ref-4772_30	34.317			CGGGAACGAACGAAGGTCTTGCAGGTATG[A/G]AA
LG4	ref-4772_23	34.589			TTTCTGTTTCCTTTGTTCCTTGTTCCAGCTTCTGGTTAGTCTCCTTGGTTACTCATTGTTTGATTTGTGTTCACACCTGATATTCATTGCATTGATTGGTCTGGTCTGTGTATTT AATGGGTTCACCCAAAAATGAAATTTCTGTCATTAATTAGTCGCTCTAATGTCGTTTCATACCTGCAAGACCTTCG[A/G]TCGTTCCCGGAACACAAATTAAGATTTTTGATT GAATCCAAGAAGTTTTTTATTTCCCACAGAAAGCAACAAAATTACCACATTCAAGGTCCAGAGAAAGACATTGTTGAAATAGTCAACGTGACTACAGCGGTTCAACCTTAA TGTTATGAAGCGTGCAAAAACAAAACACAAATAA
LG4	ref-43662	34.621			AGAATCTGATGACGTTTTGACCTTCTATATTAAAGTGGCTTGTCTGCTCTCTCTCATTTCAGATGCACATCAAGGAAGAGAAGGAACTTCAAGGAACGGATCATCTGCTCTT TCATTTGTTTAGATGGTGTACGGTCAAGAGTTTAGCGTCAGAGCTTCATATGGACGAATGGCATATGGCAACATGTTCGGCATGGCAGCACATGTGTTGATGGCATTCCGC CACATGTTCGCTGTCGGACCACATGCGCAGATGGAACACAGCCATGTGTTCGGCGTCGAAGCTCATGTGCGAATGGCATTCAGCAACGTGTTCGCCTTCAGAGCACACGTG CGGATGGCAGTCAGCCATGTGTACGGCGTCGGAGC

LG4	ref-60401	34.708			ATTGTGTTTGGCGTCTGAGCTTCACATACACGGAAGGCATTCAGTAATGCGTTGGCTGGACACCGGCGTTGCATGTGCTCCGACGCCGGTGTCCAGCCATGTGTTCACCCTC<br>GGCGCACATGCGCGAATGGCATTCAGCAACTCATTTGCCGTTGGAGCACATGCACCGATGGCATCCAGCAATGTGTTCGCTCTCGGAGCACATGCTCGAATGGCATTCAGC<br>AACGCATTCGGCATCGGAGCACATGCGCGAATGGCTTTCAGCAACGCATTCGGCATCGGAGCACATGCGCGAATGGCTTTCAGCAACGCATTCGGCATCGGAGCACATGC<br>TCAAATGGCTTTCAGCAACGCATTTGGCATCGGAGC
LG4	ref-32865_17	35.774			TGAAGACCCAATTTATACCAATTTTAATTGTATACTATACTAATATAAAGTATTATTTGGAAAATATTTTTACGTTCGTTTTCTACTCACTATGGTACTCTACTGTTAAATGT<br>ATTATATATTTATTTAAAGCCCCCCTGTGGTGAAAATCAAGTTTTTAATGTTTTTTATTTGTCTATGTGGTGTTTTTAAGACAAATCATGTGCAAGTCGACACC[A/C]TTGCTA<br>AGTATTTTCTCTTTAAAGTGATCTAAGGACAGTTTAAAAAATGCGCTGGTGTTTCTGACGTCACAAACTACCTTGTAACCAATCACGTCAAGGTGTGGGCGGGCTTTAGCAT<br>ATCATTAACTATGACTGCTCTGAAGCAGGAGAGTCTCAAGAGAAGCCAGGTCATCCTGGTATATTTTCTGTTAATATGAAAAATCAGAATATAGCG
LG4	ref-47650_10	36.873			TCGAGACTC[A/G]CGACAGATGTGCGTCTGTGTAG
LG4	ref-12610	38.006	Chr17	30496092	TCGCCATCTTTAATATTACGTTGGTCATTGTTTATGGTTACTAGTAAGAGAATACACTCTTGATGCACGTTCATACAGACAGTGTTCCAGATGCTACTGTAAGTTTGTGTGA<br>ACAGGACATTTCAAGACTCACACCTGTAAGTAAATGCAGTTGTCAATCCCCAAAACATACAGTGTGAGCATGACCTTCGTTATAAATGGGAGAAACTGCAACACGCAATA<br>TTGCGGAATACGTCGCGCCTTCTAAGTAAAAGAGCCAATCGCTGATTGATAAACAGTGCGTCCCAGTTTGTGTACTTATGCACTATTCTATGCTGTTTTGTAGTTTAAACAG<br>TTTGAGTAGTGTGTTCACACTGAAAATTCCAGAAA
LG4	ref-63429	38.427			ATTGTTCGAACGATGAATGTGCGACAAAGTT
LG4	ref-5659	38.894	Chr8	16880779	AGGTTGTTAGATGCCATATAATGTCATTTCTTTTCCCAGTGCCCTTTTGTTTTCCCACTCAACCTATAGTTTAAGGCTGAAATCAGCTGAAATTGTCCGTAAAGAGCTTGTGC<br>CCTCTGCCAACTCTGCCAAGTCTTTTTCGGTTCTTGTCTCAGAATAGCTGATAAATGTCTGGTGCTGCATTACCTCCGCCTCTGCACACACAGAATTAGCCCCCCACATTAGT<br>GCTGTGGGTTATTTATTTCTGCTCCTGCCGGACTGACACTCTAATCTACGGAGAATAGAGACAACATTAACGTCAACCTATCTGTTCCCTTATGTGTTTTTGGGGGAGGGAG<br>GGGTGACAGAATAACCTTTGCGCTGACAAAT
LG4	ref-15885_14	39.729			TCGCTAAACTGCGTACATATCAACAGACATATTGGCCAAAACACAAAAAGGATGTTGATCCAGACTTACAGGGATACTTTCCTGTCCGACATGAGCTTGCTGTCGTGGATG<br>GATGTATTGTCAGGGGAACATACCATCTCTTGGTACCTGAGTCATTACAGAGGAAACTCATTGAACTGGCA[A/T]ATGAATCGCATCAAGGTATAGTCTGTACGAAGCAAA<br>GACTTAGGCAATTTTACTGGTGGCCTAAGATTGACAACTAGATTGAAGCTCTGATAATCAACTGTCATACGTGCAAACAAAATGACAAGTCTGCAGTTACACATGATGCTC<br>TTCTCAACCCTGTTGAATTGCCTTCTGCTGCATGGGTAAA
LG4	ref-56126	40.601			TTATAACAGTATAACAGTTCCAACAGGCCGAGTTGCGCAGTATAATTTTTTCTATACTCTTTACCAGACATTGTGTCATCAACTGGACATTCGCTGGGCAGGATTGTGGGCA<br>ATTGTGCTCTGTACTGTGCGTATTGCCACCTAGTGGACTCTTCTGTCCTGCTTGCGCATGCGCTGTTGCACACGCACCGTCCGCGCGGTCTCGAAATTTGGGCTGCACGCGT<br>AATGTAATTTTATTGACATCAGTAACTGTAATCAAATTACATAAATTTATGTAATGCGTTACATTCAGGGTTCTAAATTAACACCCGCCAACCGCAGGTTAAAATTCATTTT<br>GGCGGGTGTTAATAAAAACTTACTAGCCAGTTT
LG4	ref-2700_27	40.923			TTCGAAGCTTCATGAAGCAGTGTTTTGAAATCGGCAATCACTAGATATTGTTGAAAAGTCGTTATTTTGTTTGAATGAATGTTCAAAAAACATTATTTTTCATATACTTTAC<br>ATTGTTGCATTTTGCGTATACAGACAGAGCTCATTTATTCTAATCTGAAAACCACGCCCACCGGGGGGCAAAACAATCGGAAC[C/T]TAATCGGTGACAACATATGCTAAA<br>CAAACAAACAAAAACAAACCATTCATTATTTTTAACCATGTCAAAGAATTACTTCACCTGTCGCCGAATACGTTCCCCTCCAATGTAGTTTTTATAGTAATATGACCCGTTG<br>CGCTCTGTCTCACTGCCTGTATCCACTTTTGTGTCCTT
LG4	ref-46223_18	41.667			CACCTTCTACAGATTATTTTTCAGATCCACATCAACTTGCTTTTATATTATATAGAGATGCTTTAAGTAGAAATGCTGAACATCCTCCTCCACAGACCGCTTGATATAATCA<br>ATTTAAGTTAGAAGTTTGAATTCTCTTACCCACTAAATGCCAGGTCTTTCTTTTTCCATATTTTCCACATCCTGCAGTCCGGTCCGATTCGTATCCGACGAGCGG[A/G]GTGC<br>AGATCCCGTCCGCGATCTGCCCGACTAGCAGCAGAGCACCTGCGTTCGTATCCTTGAAACCGAGAACCGAGTGATAATACACCAGTAAATACGTGAACCACATAGACGCG<br>CAGAGGTCGTTAAAGAAATGTCCCACGGCGTACGTCAGTCGCCTGCACATGGGCAAAGTTCCCGGCTGATCAGACATCGTGAATGAAGAATCGATAAACG
LG4	ref-29931	42.379			TTATAAAGTCCCTCCTTCAGTTTAAGTCTACAGGATTTTTTTTTTGGCCTATTTAATGTCAAAACATATAAGTATAGTAATAGGTATAGTGATGCTATCCTTAGTAAAAAGGT<br>ACAGGTAGAAGAATCACTCAGTGAAACGATGCATGAGATGGCAGACATTCTGAAATGTTCATCACTGTCAGAGAAAAGCAGTGGTTGTGTACCTGAGCCACAGAGATGCT<br>GATAGCAGCTGTGATTAACTGTCCACCCACTCACTATAAAGTTCTGTTCCAAAAACAAGTGAGCTGTCAACCTAAACATCACACGGAAGCTGTTCCGATTGCCTTCCAAGT<br>CTCATTTTGGACAAATTTTAAGGAAACGTCCTTCACCAAAATCCAAAAATGTGCAATAAATGTAATCCATCTAAGACGGCGGCAACTGGAATCAAGA
LG4	ref-52344	42.853	Chr8	30686759	CTAGCTCAGGGTAACTTGTGCCAAAGTCATACACTGCAGAATACTTAGGACCTTCACTGCCTTCACCTCCTGCACAAGAGAACATACAGTGTTTTTACTGATTTTTCTCACA<br>GTTGTTTTGGTATCTGATGTACTTGAAGTGGGTAAGGGGAAACTCACTTGACACACGTAATGGAATATAGCTCTCTCTCTCCTTCAGCAAACTGCTGACGAGGTCTGTGCTC<br>TGCGCTCTCTCTGCCAAGTTCACCAGCTCTCCCGTCCTGTCCATCAAGTCCACAGTCTCTACAGAGAACAGTACTTTTTTAGATCTCCTACACAGATGAGTCTGTTTATTTTC

					AAATCAACAGTTTCTGATTTTTATTGTTATTTGGAGTTGAACAAACCCCAAACCTGAATAAAACTTCAGGTTTGGGGTTGCCAGAAAGCAACCA
LG4	ref-56195	43.479			GGCGTGAAGTCGATGTCCCTGCAATAACAGA
LG4	ref-19774	43.653			GTACTCCATGTGACTCCAGTGGCTTAAACTCAATTTTATGAAGCAACGCAAGTTCTTTGTTTGCCCAAAAAAATATCATCTACCAATTCTTGTACAAAAATATTGATCTGCA<br>AAGCGTTCACGAGAGCATCATGACGCACGCGTTTTGCCTTCACGCAAGAGCTGACATTTTCAAGACACATGCGTTGTGTTCAGACGCGCACATCAACTCGACACACATGCG<br>TGAGTGTTCACGAGAGCATCATGACGCATGCGTTTTGCCTTCACGCAAGAGCTGACATTGTTGCGTGAACACGCTTTGGATAATATTATTCTGTAAATAAAGTGGTAAATT<br>ATGTTTTTTGTGCAAACAAAGCACTCGCGTCGCTTCATAAAATCGAGTTTAGGCCACTGGAGTCACATGGAGTACTTTAATGATGACTTTACTACTT
LG4	ref-3418	43.786			TGACAACACACGAACTGCATGCTTTTGTTAT
LG4	ref-27591_19	44.605			CAGACTGGGCCGAAGACA[A/G]TGCATTGTCTTCC
LG4	ref-6351	44.692			GCACAGCAATGAAGGGGTGACACTCTAAATGACTAGAGTGCAAAATACAGAGCAGGTTTTTGCAAATGTGTCCAGCCACAATGCAGGAAAAAAATCATTTATACACGCAC<br>GCACACACGCATGCATTTCGCTCGCATTTGCGCCTGAAAATAACTGCGTGCCAGTGTGAAAAAAATATTTACGCGCACTGGTGCGAGTGACCTGATCGTTTCTCATGAATAA<br>AACTATAATACAATTGGAACAAAAAGTACACCTCCTAAAATGCATCCACACTGAACAACAGTTATGTTTCGTACTGCAATCGGCTGC
LG4	ref-57591_25	45.189			TTGTGGCATATGTTATTTCACTGCATTTTTTGCATGCTACAAAATTAGTAGGAATAGTATGGTAGTATGCCATTCCAAACATACTTTGTTTCCTCTCTCTTCCTCTACGACTCT<br>CCAATTCCTATGGTTCTTTTGAAAATCCTAAGAAGAGGATTAGCCTATATGTTTGTTTTGCATTTGTTTGTTCAGACCTCCAATCCACCCTTCATCCGAACACCCTGCCC[A/T]<br>CCCATCGGCACTCACATTCCACTTGCGCTTACCGGAACTGTCCTACGTGACAGCTCCTTCTCTATTCACGGGCATGAATATTAATGCACCGCCCCAGCCAGCTTTTGTATCA<br>ATGTTAACAGTGCTACATCTGAAGTCACCTCTGTGCTGAAGGGCTCTATGATTAAAAGTAAGATCTTCATAGGCATTGCATCACATCACAGCCAG
LG4	ref-56860_1	45.495			TTTGGTAACTAACTGATCTCATTTGGGCACTTACCACTCTAACCCTAACTCTAACTGATCTCATTTGGGCACTTACCACTTAACTTAACCCTAAGTTCAATAATTAATGCTTT<br>TAAACTGGGTTAAAGCCAGTTTTAACCTTGGGTTGAGCAATGTTTGACACTTCTAC[C/T]TGGGGATTAGCAGGTCAATCGCATGAAAACAGGTCCAGATCACATTGAAGTA<br>TAAATATATAATTGATTTAATATAAATATTATGTATAAATTTTTAGGCTTTTGCCATGTGAAATTATTTGAAATTATGAGATTTCTTGTTCAAATAGGCTTTGTTTTCTTTATG<br>CGAAATATAATTTCTAATGTTTTTCATTCTTGTG
LG4	ref-51124_18	45.968			GCACTAATTTAAAATATAGCTGTATGCAGCAACTATGGGGCCAAGCCCTTTATGGACATTTGATATTTTTGGATGCAAGATTTACTTTATGCACATTGAACAATTCACATGG<br>CACGAACTCTTGCTACACACTGGATTTAATCAAACAGTCTTCAAACATACTGCCTGTTGCTTAAGCAGCACACC[G/T]CTCGTACACCTTGATTTGCTTAATTGATTTGCACA<br>GAGACTTGAAATGTTGAAGGCAAAACTGTAGTTTTTGGCAGTAAAGCAACAGTAAGTAGTAAGGCAGTAAGTAATTAAGTCTCCAAATGTAATAATGACATTTGGTCCTA<br>AAAGTCTTAAAACTACTGTATTAAAAACTTAGGCCTCA
LG4	ref-29224	46.236			TTCCTGCTGACGACAAGCTTGCTCAAGGCCG
LG4	ref-34043_7	46.472	Chr8	12530235	TTACTAAGATTTTGAGCATCCTGATAAAGACACAGTGGGGGCCAGGCTGACATCTGCAGTAAAAGGAAAGAAGATATTTGATGACACATCGTCTTTATGGTCATCAGTCGC<br>TGAATGTGTTTCGTTAAGGTCAGTGGTAGAGGCGGAGAGGACATGAGGCCACAGAGTTGTCCTTGTGTTATGAACACAGACACACAGTTGCCTCT[A/G]TTCCGATACAAG<br>TGCCAGGACATGTATTTTCATATTTGCTCTCACTGGATAACATACATGGTGTTGAAAAGGGTGACATATTGTATTCCCTGATTACAGTCAAAGCTGCTTTGTGGCTGTGAGA<br>GCATTAGCTTAGCACCGCTAAGCGTGCCGGGAGCACTGGGAATCTGAAGAGGCAGAAAGGAACTGTTACACGGAATTAATAGATCCAGCGCCCCTATTCAT
LG4	ref-58567_25	46.586			TCGTAACTTTGCAGATGTTGTTATGCTCAAACAGCAACATTACACACTAACTAAAGTTAAAAAAGTGAAATCATAATCAAGGACCCCTTTAAAAGGACAGTTCAACCAAA<br>AATGAAAACTCACCATTTAATTCCAAATCCATATGATTCTGTGGACTACAAAAGAAGATGTTATGCTGGATGTTCACATTTTTTTTCTATTCAATGAAAGCGAATAGTGTGC<br>AG[C/T]GGCTGTCAAGCTCTAAAACTGACAAAAAGCACAATAAAAGAATCATAACGGGTCCATAAGGCTCAATATTCCAAGGTCTTCTTCTGAAGTAGCTTTGTGTGAGGA<br>ACAGACCGAAATAGCTGCTATTCACTGAAAAATTTCCCTTATATCGATTAAAAAAATGTACATATTCAACATATCTTGATTGGTTACAAGACATGAGAGCCA
LG4	ref-62227_31	46.93			GCCTAGGCCTATTCGATTCCTGGCCTTAAGTAAATGTGATTCCAGAATAGATTTTTTTATCCCTAACCCTATTAATTAGCAACTATAGGCAACAAAATATTTCATTTATGCTA<br>TTGCATGTGAATAAATATGTGAATGGTAACCACAGGACTATGTTAAATAAATTTGTTTGTTTTTTTCGTCTGTAACTGACTTTTATTATCGACATCGCGACATACTTGCAAG<br>ACATC[A/G]TTGATATACAATAATATCGTCATATCGCCCAGCCCTAGAACAGAGCTCACCAAACTCAATCTTTAGTACACAAAATCCTACTCATATGCTTGCATTTACATGG<br>GTATGAATGGAAGAACACAACGTCTAATGTGGCTTCCCTTTCAAAGCACGACTGGAAGAAAACTCTGGAAGAAGGTAGACCTTAAGGAGCTGCAGCAA
LG4	ref-9453	47.386			TGAATTGAATTATAGAAGTAGATAATGTGGCATGTGTATGCAAGTTTAAACAGATGTGTTTTTAGTCTAGATTTAAACTGACAGAGTGTCAAAAGCAGCACTGAGATCGGA<br>TGATCTCAACCATGATCGGATGATAAGAGCAAATCATTTGTAACTCTAATGAGAGCAGTCTCAGTACTATGGTACGGTCTAAATCCTGACTGGAAATCCTCACAGATACTA<br>TTTCTTTCCAAGAAGGAGCGTAGTTGCGATGATACTGCCTTTTCTAGTATTTTCGACAGAAAAGG

LG4	ref-65228	47.473	TTTAGGATTCTCTGAAAAATTGAATTTAAAAAACACACACACACACACACACACACACACACACAAAGCCAGGAGTTTCCAAGCAGAGCTGATGTAATGCATTGGCACAGCTGCACTTACACTTAAAAAAAAATAAATAAAAAAAATAAAACAAAACACATGGACATAAAAAAAAAAGTACTAAACGACTCATAAATCCTGAATTATGAACGGTCCTTGTGCAGCAGTTGCTAACGTGCTCTGATGTAGGGAGACATGATGATTTGCAAACCCGCAGGACATAATTCAGCAACTTACAACCAATTTGCTCTGATGCCATTTTATTTTACATTTATGAAGAAAATGCAAATGGAGTTTATCCATGCAAAAGGTTTATCAGATTGTTTTTGATCACAGATTAACATCTTATCTGCACCTAGAGTAGTAATGC
LG4	ref-52908	47.52	GGTCCGCCATCGATGGTTTTGCCGAGTGCTT
LG4	ref-16838	47.932	GTGGTTTCAGCCAGAGCTGCAGAGGGCCGGACATGCTGAACTCATCAGACCTAGCATTCTCTGAAACGTTCTGAGCGTCAGAGAAGCCCCGGGCGGAAAGGTTTGTATGAGCTGTTGAATGGCCAAAGAATGCTCTGGAGTGACTGTGGCCCTCAAGTGGATAGAGTCTAAAATTATCCCCAGGAACGACACACATTGGCTAGGGGATAACGAGCTTTTTGCAGAATTGACCCTGAGCCCCAAGCACTCTAAGTGGTTGAGGATGATGGATCTGTGGGATATTAGCTCCGCCTCCGACTGGGCCAGAATGAGCCAGTCGTNNNNNNNNNNNNNNNNNNNNNNNNNNNNNNNNNNNNNNNNNNNNNNNNNNNNNNNNNNNNNNNNNNNNNNNNNNNNNNNNNNNNNNNNNNNNNNNNNNNNNNNNNNNNNNNNNN
LG4	ref-20243_2	47.958	CCTAACTTTCACAACTGTTGCTTAGTCATCTTATAACAAACAAAGGAAGTTTTGCAGTTACTTATTTCCATAATCATTCAAGCATTCAAGCAAATATGGCCTTAACTTAAAAGCAAGCACATAACAACATTGAAACAGGTAAATAGCAATATCAACATCAAAATCCAAAACACTGTCAAACATCACCCGAGGTGCAGAAGAA[A/C]AGCGCTGCGATTCCAGTGCCTGGATCCTGTTGTCCATGTTGGAAAGGGTTCCTTGAATTGACAGCAGCGCTGAGAGAACCCTGTCATCTCCCTGTTGTGCTGGAGCTTGAGAGGGATGAGATGATTTTACCTTCTTTGGAGTTGGAGACGCTTGATCGGTGGAGATATTTCAACATGTTCTCCAACCAATACACCTATATAGGTCCTTTGTCACTTCCCTGAAATACGA
LG4	ref-67668_32	48.381	TCAACAAGCTCATTTTCAAATTTTCTTGAATTTTTCCCTCACTTTTTCAACAGCTATCAATATGAGGAGTGTGTGACCGGCGTCTGCTCTGCAAAATGATTGGAGAACATGAACGGATCATAGATGTCCCAAAACCCTGTGTGTACACAAGGGCTGTGCAAATCATGTCAGCCACACCTTTAACAACACAAACATCCTGTAATTTACTGCCGATGCTTCTGCCGTGCTGCC[C/T]AAGCCTGCGTTAACACAGGACTCAACAGGAGGACGGGAAAGGAGGGTTTTCCACACAGCTGATGCTGGCAGCAACTGCCGGCTACGCTCAGCCCCCACCCGAAAATTACCATCTGTATGCCGGCACAGAGAGCTGGGTTCCTGCTCAGTATAACAGGAGAATGTAGAGGATACTTAAAGTTTCCATCGCCCATGTTCTCA
LG4	ref-1265	48.396	AAAGTAACATTTACACTTAACTTAGATTTTCAGTGCAATATTAATAAATAACTGTAATAAGATGTTCAAAAATGTGGGAATTTTTCATTAATTTGTAGCAGGATTTGTTCAAAAAAGTCACTGTGTCACATTCTGTCCTTCTGTTGTATCCATAGGTTCTCACTCACTGATGTTGTTTGCAACATATATAACTGGACAGACTTCACTTCCCGAGTTCAGTGCTGTAGTGATGCTGGATGATCTGCAAGTGATGTACATTGATTCAACTACGTGGAAACTTGTCCATCGCAGAAACAACATTTCAAAGTACTATGAAGAGGAGCAGATTGATGCTGATTATATTTTTAGGCATATATATGACAGCATGACACATAAAACATCCAATGCAAAGATACAGTTTAATGACACAAATGGTGAGTGCTATTCTAAAT
LG4	ref-32547	48.855	TTTCGCAAACCTGTCTTTCAGCCACGTTGGTGAATGACGCAGGCGCAATAACTTATTTTATTGAATCTTTGTGAGATCTAATTAATGCTAGATTTTTTGCTATAATTTACGGACATGGAATCTGAGGGCTAATTCAAATTTCTCTGTTATTTTGCTTTATTTCCAGAATCAAGTCTTACGTACCAGAGCACGTACGCGCATGCGCACTCTCGAAAACGGTGCTTAATCTCTTGACCGACTAAAATATGTAAATAACATCTGTATATATTCGAAATAAAAGACATACATTGTACTTAATGTCAGCTTGCTTATTTTGCTCAAGATAACGATTTAATAAGATCACATGTCATAAAAATAAGAAACCGCTTCTTAACGATTTAATAAAGTCACATGTCATAAAAATAAGAAACCGCTTCTAAACTGTCGTTCCA
LG4	ref-31770	49.423	TTGGCCGATAACCGATATATTTGAAAGCCGATAACCGATATATTGGCCGACAAATCTGAATCAAAATTGTTATATAATTTTTCAAAGCCTGATTGCAAAGACAAAAGTTTCACCATTCTTACTGGGCCGATAACCATAACATTAAAAATTAACATATATGGGCCGATACCGATATGTTGGCAGATATATCGTGCATCTCTACTCAAAAGGGATATAGTTTGGTGACACATTTAAACATACATTTTTTAAAAACTACTAAAAATGCTAAGGAATATAGTTTGGTGACACATATATACACATTTAAACGTACATTTTTTAAAAACTACTAAAAATGCTAAAAACACAATAAACACTGCACCTTTAACTAAA
LG4	ref-23239	49.697	GCACAGCAATGAAGGGGTGACACTCTAAATGACTAGAGTGCAAAATACAGAGCAGGTTTTTGCAAATGTGTCCAGCCACAATGCAGGAAAAAAATCATTTATACACGCACGCACACACGCATGCATTTCGCTCGCATTTGCGCCTGAAAATAACTGCGTGCCAGTGTGAAAAAATATTTACGCGCACTGGTGCGAGTGACCTGATCGTTTCTCATGAATAAAACTATAATACAATTGGAACAAAAAGTACACCTCCTAAAATGCATCCACACTGAACAACAGTTATGTTTCGTACTGCAATCGGCTGCTTTT
LG4	ref-50582_7	50.45	CTGTTCAGAGAGGGAAGCACTCAAACCAGAGTCCTGCACTGATATGAGATCTGGTGAGATGCGCATTGTCTTACACGGAAATGGGGAATGAGTCTCATATGCAAAGTGGCTTTGCGTGAGTGAATCAGAGCATGCTGTGTTCAAAGCTGAAAACACAAATTAGGTAAAATGAGAG[A/G]AATGCACCATGTTCGTTACAGTTCAGTTTACACAAAACCAAATTAAAACAACAGAATTTATTATATTGTCTTCACTGGTGGTAAATATACACAACTCATGCTTGTCATGTACACATTGAGACTACATTTGTTTTTACATGTATATGCAACAATCATTATCAAATGTTCATGTTATTGTTGTTCAGAATTTGCCT
LG4	ref-26182_4	51.006	CGG[G/T]GACCGACGACTGCTATGCCAAAGCCTTG
LG4	ref-69459_18	51.902	AAAATGCATTCGAACAG[A/G]CTGCTTTTGTGGTA
LG4	ref-69459_9	52.525	AAAATGCA[A/T]TCGAACAGACTGCTTTTGTGGTA

LG4	ref-1149	53.188	Chr8	42572229	ACAGAAAAGACATTTGTTTTCGCCGTCGAGTGGGCTGAACTGGAGGACTGGATCCAAAAACTGTGTGAAATCGCCTTTCCGGTATTTAACACTGAATTTAAATTGTGTGATCGTCTCTTTCCAGTATTTTTTCCTGTTATAGAATATTGTGGTGTAACAGCTCACTTGACAACCGGATTGCAACAGTGTCGAACGGTTATATCAAAGTTTCCAGGTTTTGTCCTGTCTTGTGTTCCTGGCTTTGTAACCACAGTTGCGTTTGTACTTTGAAACTCTTGTTGCCTTCTTGTTACTTCTGCCTTCTATCTTTGCTGAAAGCTTGTCAGCTCCTTATGCTAAAATTTCTGCAGACGTGAGATTCAGATTGCA
LG4	ref-65768_29	53.445	Chr8	42612573	AATTTTCCCAGTGACTTTAAAGAGGGGATTTATGACCTTTAAACTGCTCTTCAAGTCAGGCTGACATAGGAAGTGTTTTTTTAATGTGATCCCTGATATGACCTCTTTACCTATTTCTGTTTCTCTGTCTATTTCTGTTAAGTCCCCGTCATCAAAGTGCCACCACATAATCTGGTAAATGTAACAGGCAGCAGTATAGCATTTCTCTGCGAGGTGTTTGCATTTCC[A/C]ATGGCACTAGTGGAATGGAGGAAAGATGGAAAAGAGGTCATTTTACCGGGCGATGATCCCCACATATCTGTTCAGGTAAAGTATTTCCAAATGTAAGAGCGCTGTTTTAAACAGAAACTCTGTGAAGTAATATAGCTATAGCCTTGACTTACATGTAGGGCCACACATCTGCAGCCACAGAACTCTACAAGCTCTACAGATTT
LG4	ref-49302	53.739			GCGATTGATTCGAGGCTCGTGCCGTTCCCTC
LG4	ref-61218	53.984			TGCCCGTTGACGACAACATTGCTCGACAACA
LG4	ref-45389	54.183			TTTTAAGGACCGATGCCGGTGCCAATGCCTA
LG4	ref-67443	54.203	Chr8	35819380	AATTGCGCTCTCACGCTAATTTAGCAAATCTGGCCCTTATTATTTTAAACTTTGACAATCCCCTTTTTCATTTTTCACTCTGAGGTGGAAACAGACCTCCATATGTGTATATTTATAAGCCATATGATTATAAGCGTTTGTGTGTAAGCTTCGCTCCCATGCACAGACATAAGATGGGGTTTGCATTGTTACCTCTGTGCTCTGTCTTTTCGAGCGGGGTGGCAATTTGGTTAGCTGTGACAATAATAATACATCAAAGCGGAGAATCAATTCCACTACAAAGGCTTTGTAGCCTAAACCTACAACTAGGGGAGACAGCTCGAGCATTGCGTGTGTGTGGCGCACACTTTGCAAAAGCATCTGTGTGCCCTCCTGTGCGTGTGGCTTCTTTAGTATTCAGTTTGTGCATCTAATGTACTAAATGTGTTTTT
LG4	ref-52830	54.785	Chr8	41284378	TGTCCCTCAAGACAGACAGCACAGCCGTCATTTCCTAAGAAAACCATGTTTCAAAACTGCCCCTGATAGGGTGTGTGAGTGTGTTTTGGGCTCGGTGCCTAGTATGGTAATTGGAGTCAAGTTCTGCTGGGGTTAAAAACAATATATGTCTGCTTACACAGCTGTAGCACTTAAATGTAGCAGCCCTGTCGGTCCCACAGAAACATGCCCCACCCTTTTATGACTGGGTGTGTGTGTGTCTGTGTGTGTGAGAGAGAGAGAGAGGTCTTGACTCATTGTAAATGATAGTCACCAACTCAGTAACCCTAACTAATTGTGTTATGTTGTGAAATTTTTAAAAGCATCTTTGTTGTTGGTTAAATGTGTGAA
LG4	ref-37325	55.125			CAACATTGCTCGACAACATTGCTCGGCAACA
LG4	ref-24182_32	55.255			TAGTGGTGGTGACACTGAAGTCAAGTGATTGTGTAGTTTTTAATTGACTAACTTTAGCTTTTTACTTCTGGTGATTGTATTTAAGCTTCAAAATACATAAATATTGTGTTAATTTGTGAAGATTATAAACAAAACGTGAAAGAATCATAAACTTTTGTTGGTCAGGGAGCTTATTTCCTGCATATGCATCGATCTTGAAA[A/G]GATATGTTTTTTGAATCGTTAATTTCGGCCGATAATCGATGTAAAATGCTAAGAATCGGATTAATCGTGATTCTATAGCGATTCAGTGCTTTAAAATACATAAACATTAAATTACTACATGCGTGAATTGATGGAGACCATAGACTGCTCTCCTCTTTACCGCCTCTCA
LG4	ref-3004_25	55.463			GTGCGTGTGACTCACAACACAGGGGAGTGCGAGAATAGCTTTGACGGCTCTGATAACGGGGATCTTCTTAAGATGTAGTGCGATCATCAATAGAGCGCCGGCGTTGCGAGGGGCTGTAGTCATCATAAGATTGCTTGGCGTCGCTGTTGCAGATGAGGGTTGCTGATGCACAGTCACGGGCATGATAGTCGTTG[A/G]ATTGGCTACGCTGATGGCCAACTGTGCACGTTTACACTGACTGGCTATTTTTAAAGCACTGTCCAAAGTCACTGCTCCATGCTCATGTATTTTCAATTGAAGTGCAGGATCTAAGCCAGTAACAAATCTACGAAAAATCTCACAATTACAAAGCCGTGACGTCATACTGAGGAAA
LG4	ref-60104_2	55.653			GCTAATGCGTATTCATATTCTTGAATAAATAAACAGGTGATTGGCTCTTTTAACTCTAAAGGTGTGGTCCAATTTACCATGCAAATTTTTGTAGGTGAAATGTGATTTCAATAGGAATTCACACAATCAGGCATTTCACATGAGGGTGAAAGATTTCCCCACACAGATTTCGCAGCGGGTTCAAGTTGGTCATGAGGATTC[A/G]CCACATGACGAACAGAATGCTGCTTGGGTTTGAAAGTGTCTTCTGTGTGAGCTCTGCTTATTCACTAATGACAATAGACAATGGACTTTTTGGCCTGCAAAATTGTACAGAAATGTTGCTAAAGTGGCCGCACCTACAGAAGACATATTGATCTAGCTTTACTATTTTGGCTATTTATATATGTGCAAATAGGCTGTTTCTGCCCAGTCTCTGATGATACCCAATATTGC
LG4	ref-21958	56.022			ATTTTGCTCACGATAGTCATGCAGCCTGTCC
LG4	ref-9478	56.219			GTACATGTGTCGATGAGTGTGCAATCACAGC
LG4	ref-5897_32	56.221			TTGGTTAGATCGCCTGTCAATCAAACTCCTGGCAAAGGGTCAGTTGGTCCATATAGTCTATAAAAATACAAATTTTAGTGTAAAAGTAGTAAACTTAGACTACACAACAAGTTTACATTTACGTTTTTCTTCTGTATTGCAGCTATCTGTGAACTTAACTACAAGTAAACTTATAGGTATAATGTTACTGTTTACTAGTGCAACCTGTTCCGATTGAGCTGCTCAAACTGG[C/T]GACAGCTGGGATGGGAGGTGATGAGTGAGGTTTACCAGCACAACTCTTACCAGCTGTGCTCCGTTACACTACCTGTATACTTATAGCCCATGTCTGAGTTTATGAAAGTACTGTAAATTGACCTTTAAAAGACTCCTATTTAAGTATTCATTTTAAATTGAAATATACCTTTATGCTTTAAGTAGACTTGAATTTAACTC
LG4	ref-8666_15	56.387	Chr8	36661426	GCTGTGTAGATCACTCTGCCTGACCCTGAAGGGGCTTTAGTTTGGCTAAGCAGAGTAGACAGGAAGGCCTGCCGAGGCCTCCGGCTGGGAGCTCTAGTTTCATCTGACAGAGATGGAAAATAGCAGAGTAAACAGTCTGGATGCCTCCAGCTTGGTAGTCATACTTTACCTGTCCAATAAATGCAGCAGTAATGTTGACAGTGATTGGGGCGAC[G/T]TGAATGCATAAACCATCACGCCTTGATCTAAGGTCAACAATGAAATAAATAAATCAATGCAAATAGCCAGAGTCAAAGTCTGACCTTGAATCTGCTAACAAAAATGAAAAGTGT

					TAAATCCTTATGTTGTTTTTACTAAGTAATGTATGTTATATTATGGTGAGCATATTTGTTTTGTCATATTTTAGCCACTGAATTGTGTGAGTTCACTCACTGT
LG4	ref-13517_25	56.487	Chr8	49921048	CTCTACTCGTTGGCCTAAATGGGGTTAAGAATATTTGTAAAAGAGCCATCAAAGTTCTGGAAAATTGATGTTTATCAGGTTTGTTATGCACTACAGAATGATGACATAGAGACCGATATCAACAAACTGCGGCCCAGACAGATCAGCCTGCGTCCATTTGATGAGCTCAGAGTCTACGAGCATTTCTGTCGGG[A/G]ATTCACCGATAATTTTGAGGTGAGGATATTTCTGATTAACTGATGAAAGGTCAGTAAAAATGCATATAAATTAAAAATGACTTTGATTGCAAAATAGCAGACACAAAAATGCTTGATAAAAGGTTTTAAGAACTTAAGTTAATCTTGCTCATCTAAGGTAGCCTTGCTTTTTATTT
LG4	ref-54796	56.52			GATGATTTACCAGCATTAGGGATGTGACAGTGAGGAAATTTTACCACCGGTTAACCGACGTGTGACAACACAGATAATACACCACAGGTGGGTGGGGGTTTCCCTCTTTTGCGCCTCGCTTTAAATGGCTAATTCAAAAGCGAATGTATAATGTGCACAGCCATTCATAACAGTGTGGAGCAGAGCAAGCGTTTTCAAATAATTCCTGTGGAAGTTGAGCATCAATAGGAATGAACTGAAAACACTCCGGCCAGTGAACACGCAGTTATGAATGCCTGTGCGTTATGAATTTTACTTTCGCTTTCAAATTAGCACCAATTCAGGGGCAATAATTGCTTTAATGGTGAGTTCATTATTATTCTTAATGAA
LG4	ref-39126_6	56.774			TAGCCCTGGCTGCATCTAAAAAGGTCGCCCAGGGAATTGGCCGCAATATGGACAGCCTTGTGGTTCTGCTTCGTCACCTGTGGCTGACGCTTACTGAGATGCAAAACGTAGAGAAGAGGCAGCTCCTCGACACCCCGGTGAGCCCACAGGGTCTCTTTGGAGTGGCAGTGGAGGCTTTTTCTGAGAAGTATACAGAAGCACTAAA[A/G]CAGTCGAAGATGCTGCCAGGTCAAGTTCCAGATCGTCATCAGCTCAGTGATTGGCCGGATATGCTTGTCTCGCTCCACCCGTGAGCGCGAGCCTGAGCCGCCGTTTCATCAGACACCACAGCAGGTATGGCCTGCGGAGAAAAAAGAGCAGCATTCCTGATGTTCGTGCCAGGAGGAAAAGGGCACGGTCCATGCACGACAACGGGCCAGCGGCCAAAAGAGCACG
LG4	ref-10997_7	57.09			ACCGAGAGTCAAGGGAGCTGAGCAACATCGCTGGTGCAGGAGACCAGGCGACGTCGTGGCGACGAGCATCGTAGCCAGAGTATCTGAGGACAGGGACGACGCTGGTGAGACAGGACAGAGGTGACCCCGGAGGGAGGAAGGAGCCCACTGTAGCCGTAGGGGAGGAGGGACAGATC[A/C]CAGCATAGGGCTCGGATGTCCGTGGTGTAGGCAGAGCGACGACAGACCAAGGGGGAGCCGGCGGGACGAGGGTCTCTGGAGGAGCCAGAGTGGAGCTGTCAGGTCGATGGGCCGAGATGGAACGAAGGGGTCTGAGGCCGGAGGCGGAGCCGCTGGATCCTCCTTGACAGGTGCTGCTGGAGGTTGCTCGACC
LG4	ref-13293	57.514			ACATTTCATTGGCCATGTCAATCATAGTAACGATTACCTACACCCAACCCAGATCCCTAAACCTACTCTTCACTGTAACCTGTAACCTGTAAGCAGGGTGGGGATTTCCGCTGAACCTTTTTGGAAAAAAACATCACGCGTTAGCAACATGTTAAATCACAACTTGCTAAAACATTTTAGCAATGTGCTCGAACAAAGACGGGAATACACTACATGACTTTTAAAATCGGAACGGATTTTTAAACAGCAGGCATCATACACTTGCCGCCTTTGTAAATGGTTACAGAGAAAAGCTGGGCATCATACACTACACGACAGAGGATCACACACTATCAGACTTTAAAATCACAAAAACAAACAGAAAACATG
LG4	ref-22377	57.856			TGGTGGGAACCTTGGGCACATATCTGGGTCGGGTCTCAGGACAACGTGAGAGTAGACCAGCTTGAATTCCAGGTATGATTTGTCGACCGCAAATGTCTGCAGGTCCCCCACCATCTTGACTGAAGCCAGTGCAGTCAGGAGCACAGGTTTTAGAGACAGGATTTTGAGCTCAACTGACTGCAGAGGTTTCGAAGGGTTCTCCTTGTAAGGCTGTAAGAGCCAATGAGATATCCTAAGAGGACACGAGATCCTCTCGCACCCCTAAGGAACCTGATGATCAGGTCATGCTTCCCTGGTGACTTAGAGTCTACTGCATCGTGATGTGCAGCGATAGCTCAACATACAGTTTCAGGGTGGAGGGAAACAGCC
LG4	ref-18811	58.694	Chr8	38016358	TGCGTGAGGTCAGTAGAAGATGGTATTTAGCAGTGTATATATTGCACCGTGTATTTGTGTCCTTACATACACATGCACTCCTTTGTCCCAGCTGTCCGAGGTGCTGGTTGAAATGGGCAGTAACCTGATGCAGGTAGATGATCAGATCCTGGCCCGGGCCCAGAGAGAGGAAAGAGCCTGCAGTTCAGTTGTCCACACTCTGGAGACTCTCGCCTGGCCTCAGCTGCACTCTCACGCACAGGACCTTTCCAAAGTATGTTTTTAATCATGTCAGCTTCATATTATTACTATTTCAACTAAACTTGTTCTTTTGAACTTTCTGTTAATCAAAGAATCCTAAAAAAAAACATCATAGTTTCCACAAAAATAC
LG4	ref-37523	59.192			AGAAAGTTGGCGACTTCTGTGCATGCGCTGA
LG4	ref-65165_10	59.466			ACTCAGTAA[C/T]CGATAAACATGCGCTGTTTGAT
LG4	ref-19121_24	60.609			GCTTCCTCGTCGATGGTAGTGCC[C/T]GCACTCAG
LG4	ref-51506	62.958	Chr5	29658437	AAACTCAGGACTTCTAAATCAATTCTTTCCAAAAATATATCACAAAGTGATGAGACTGCAGACATTTGACTGTCCTCAGCGTGTGCTGAAGATGCGTCGTCATCTCTCGCTTATTAGTGACTGAAGTTCAGTGTGATGTAAAATTGAATTTGCTATTGTTCAGTCACTGTGAAGATGGAGCGATTGTGTCGTGTTCTGCATCTGTGTGTGTGTGTGTGTGTGTGCGCGTGTTTTTGTGACATATCAGGACACAAATTTGTATAATGACATGGGTATGACATAGGTATTACAAGGAGAGGGTGACTTATGAGGACATTACCCCATGTCCCCATTTTTCAGTTCACAAAAACAAATATGTGTGTGTGTGTG
LG4	ref-47792	63.313	Chr8	53796051	ATCAATGTTAGTGTGCATATGCACTCAACTCACACTTCAGATGTGGTTCTCCATTAAAATAGAGCTTAATAGCATCGATCTGAGATAAGAAATGATGTTCTGGATGTTCCTCTGTGTTACAGTATGTCCTGAAATGTCAAACCAATCAGTCACACACATGCAAACATCATACACATACTGCAGACACTTCGATAAAGCACCAAATAAAACCTCTAAAGATACTACTGTACCATTCATCTGCTAACACAACATCTGGATGCTCCAAATCATGTAGTCCTGAAACAGAAGAAACACCCATCATGCTTAGTGCTGCTTCAGATTCATATCCAAGCAATATCCAATTCATATCATAATCCTTGATCAGCAACA

LG4	ref-54308	64.094	TCACAATATTTTTGTTTCAAATATTACTTTTCCATTATTTTTCAATGTAAATGCACTAGACAGACGTCATTAACCATTGCGCTCGCGTCTTTTGCAGCTTCCCACACATGAAACCGCATTCTAAAAACATGCTCAAATTAAATTAAGTTCAGTTTTTAAAAAATGCGTCTCTAGACCTTGCGCTTCTTTCGTTCTACTGACCTGTGTCTTTGCGCTTTTAACAGCAAAAAGGCGTTCTGTGTGAACCAGCCGTTGAACAAAATGTTGGGTTTGAATGAATGATTCAATGACTCTCATGAATATTAAAACTTGTTTAATTACTGGATGAATCAGTGAATGATTCAATGACATACTTTTTTTTTTAACTGT
LG4	ref-46448	64.366	AAAGTTATTGCTGCGTTCACCCCTCATCACCCTAGTTGTTTTCTACCAGGTGACAGAAAACCTTCACTAAAGCATGGCACATCTCCATGAATGAAAATGAGAAATGTAGATCTTCTTTAAAGTAAATTATACTGCACAAATGTAGAGCGCAAAATAGTGTTTAAAACATTACATGTTGAAAAAAAAGTGTAAAAAAAAAAACTTTACTCCTGATGTGTTTGCATCTCTAAACTGAAACAACATGTGGAGTTATTTACTAAATAATGACCCAGCAGCTGATTGTGACATACATTTATTAGCATTACAGTAAAGGCATTTGTGCTTTTTGATGCAAGTTTGAATTATAGATTCATGTGCTGTTATTTTTTAATAAATAGAAAACAAACAAACAAAAAAGGAATGATTTAATATGGGACAGATTAAACATACAT
LG4	ref-14106_28	65.499	CATGCATAATATCCCTTACAGCAGATCTAGACAGGTGGTGCTGGGGGAGGTGGAGGGGTTCAGAGGAACTCTGATAACATGCTTCAGGCCTGGCTTACAGCGAACACTAAAGCAGACTCTTTAAATGTTGAGCAAGCAAGCTGCAGATACAAGATACGTGATTGAAATGTAATCGGCCAGCAGTCACATCGTACAA[G/T]GACATATCAGTATGCACCCGACTGAACTGACTGAGCACATTAGATGACAGAATGATTATATTGGGGCCTTTTTACTGATCAGATGGCTACACGATTTGCTAAAACTGTTCTATTTACACTTGGCAACAAATGTGTCTCTGCAAAACCCGATCTTCAATTCCCACGCTATAGGC
LG4	ref-18379_9	65.815	ATAATGAGTTAAAATAGCTGTTTCTGCATGTACAGTATTATTTTGATATATTGATGATAACGTACATATTTATGTTTTTGTGTTTAATTAAAAAAACATTTGCTTGACAATTGATATTGACAAAACAGTTAATAATTAAAAAGCTGTATCCATATACGTATGGATCCAGCACAGCTGACTATATTCTACATCAATAGAACGCAATTATG[G/T]CGAGTGCTGTGCAGCAGGTATACGAACATTCTTATGTAGAATTGTTTTGGCGATTGTTGCAGTTGGTTGCAGTTGTTAACCCTAACCGTGATAGGCAACATGTGTTTGGAGCAGTTAGCGATCTATGGTTGTTAACCCTAACCGTGATAGGCAACATCATGACTATGATGAGAAAAAACAGGTATGCTTGAGCAGTCTGTGCATATTAGGGTAGGATCTTGT
LG4	ref-42337_32	66.202	TAAAAATGCGAATGGATGAGCTTGAGAAAATTAATAAGGAGTTAATGAACAAAGTGATGGACCTGGACATCAGATAACATCAGACTATTAAATCTAAAAGAAGCTACCGAAGGAAGTGACCCGATTGTGTTCCTTGAAGAATTCATTCCCAAATTGTTAAAACTACCGATCACCTCAGTAGCCCATCGGGGCTTTGGAGTACCTGGAGATCGACGTCCATGCCCAGTATTC[A/T]TCAAATTACAACGCTCGAGAGATGTCAGGATGATACTGTCGGCGGCCAAACGACACAGGAATATATCGTACGAGGGCACTAACCTTCAGATTGTTCCCGATATTCCACCAAGTGTTCGAATGGCCAGGAGAGCGTTTAACACTGTGTGCACAGATCTTATTGGGAGGAACATACGCTTCCAGATGGCATATCCAGCAGTT
LG4	ref-59579_27	66.927	CCAAAGAATTCGAGAGGCTTGCTGCC[A/G]TCGCC
LG4	ref-26904	67.223	TGCTTACACCCAGGGCATCCAAGATGTAGGTGACTTTGTTTCTTCAGTAGAACACAAATGATGATTTTTAACTCCAACCGTTGCAGTCTGTCAGTCGTATAATGCATGTCAATGGTAACAAAATCTATGAGAGTAAAAAAAACATGCACAGACAAATCCAAATTAAACCCTGCGGCTCGTGACGACACATTGATGTCCTAAGACACAAAACAATCGGTTTGCGCAAACTACGCCAGAGCGCGTTCACACCTCACACACCAGATGCCGTGCGCGCACTTAAGACAGTTGGACATAGTGGTGTACATCAAGAGTGACAATGAGTGACGTATACGTGCGAGAGATCACTTCCGGCGCTTGCGCAAACTACGCCAGAGCGCGTTCACACCTCACACACCAGATGCTGTGAACGCTCAAGACAGTTGGACATAGTG
LG4	ref-50051	68.461	CAAAACATAATGGAAGACATATCAGAGTTCAAAAGAGGACAAATTATTGGTACGTGTCTCACTGGCGCATCTGTGACCAAGAGCCTATACCATGAAGCCGGTTTATGTGGCTAGCCAGGTATGTTTCAGTTTAGTTTGTGGCAACCCTGGGTTTTAGGCACCATGAAAGTGCTTTTAGCGGTGATCATAGCTAGAGTAACTTACACACCACGATTAACCTGCTCAGGGGCAAGATATGTTCTGTGTAAGAGATCTCTAACTGAAATTGGACCAATCAGCTGTGAGCAAAGTGACATGTCTATAATCAACTATATAAACTAACTTCACAACTTTCATTTCCACTGATGTAGCAAGATATTTTCTTCTTGCTGTGTAAATGCATTTATGTACTTCATATTCTTCAATCTCTTAATCTTCAGAAGAAGAGAAAT
LG4	ref-33037_28	68.78	TTGAGGAACACGAGAGAGCTGCCAGAA[A/T]CATT
LG4	ref-52303	69.506	CACAGAAGAACGACTGGGGTGCTCTCGCGGC
LG4	ref-47851	70.317	CTTTCAATTTCGATTTAACTGCAATTGGCGA
LG4	ref-18482_1	70.922	TTTCAGGAAGTGCATAAGGAGCTTGTAAAGCCGTGGAAGGCAACTTTTACTGCCAGGGCCCATTTCATGAGCTCTTCCACCCTCTCTACCCTTGACGGTGGGGCGGCCAGGGGATATACATTGGTTCCACAG[C/G]TGGAGCATGCGATTGCAATGCATTTATACCTGCAGAATGCCACCACCTGGTGGAATTGTCCCAGGTTCTTGTCCAGGGCCTGTAAATTCACTTCGGGCCCTATCATACACCCGGCGCAACGCA
LG4	ref-61897	71.102	CGAAAAGTGACGAATTGCTTGCTTGAACCAG
LG4	ref-22870	72.891	CCAGCATACACGACTTTCATGCTGAAACCTT
LG4	ref-28714_16	75.993	GTTTTTGGCCAAAAGAGAAAAAAGACAGAAAATATGAGCTGAAAATATATGCCACCCCGTATACATGTTCAGAGTGTTCAGAGCTCAGGATTCACTCTCACACAGCATGTTTTGTAACTTTGCGAGCGGCTCCGCTTCAGAAGATGCACAACTCTGGACGAGCAGGTGCGTCTGTACCGGCACT[G/T]CGTTCGCTCCACTCGTGTGCCACTCACGCTCACAAGAAAAGCAAAGTCCGCTCAAATAACGTGCCACATGACATTTCTCTTTAGCAGACTTGTTTCTGTTTGCAGAAATGTTTCTGTTCTGTTAGCTGGTAAAATAACGCTGCCCTCTTGTTCACTCGTTCTGCTCACATGCTCTGACAGGACTA

LG4	ref-25117	77.489			GTATACTCACACTGACAGGGGAATACCTTGATGCGACGCACATATGCTGCTTTTGTGTGTAATCCATAGATGGAGGAAAAATACACAATACAAAACACACTTCATGATGTCCAGACCTCCTGAAGAGATGAAGAATTATCCGTGCACAAACTCAACTGCAGATTAACACTGTGTGTGGAAATGTCGCGTGCGCTCTAGTCAGAGATGTGACGAGGGAAGTGCATGAATGTATTTGTGAAGTAAAGCTGCCGCTCATGACGCTTTATATACTCCAATCGTTGCTACTGCAGTTTCACTCAGATGAAGTGCGCTGAAAGAGAGAGGTGAGAGCAGAGCGGACGTGTCATGTCTTCCCTTTCATCCTCTTTCTCTTCTCGTGGGAATGAGAAGCTGCTGCTGTCGTTATTGTTCACCACAGGAGAATCCGCTAT
LG4	ref-40013	77.947			ACCACTAATGTTCAGACACATGTGCCTTGAGTGTATGTTTTCTCAGCAGCGCAGTAGCACAAAAGTGTTCATTCATTTTCCCCACGATGCTGTCAGAGCTGGAGTTTGACTAACACCACATGGAAAATATGACTTGTGACAATGTTTAGTATGTAACAACAGGCAAAGTGCGCATGAGAGCGGTTTCTTCGTGGAACGCCGCGATGAGATTAACAACACTCGCAACGTGGACCATAGATCATATACGGACTGGAGTAAGTGCGGTTCAAGTTTTTATAGACATTGCATATAGCGTTGTCTCCGTTTGCATCTAGTTTTGTGAAATGTAACCACACTTCAGAATGTTTAGCATTGCCCTGTGTGTTGTGT
LG4	ref-50634	79.391			AAGTCAAAGACGATCCTTGTGCCATCCATAA
LG4	ref-50127_7	80.126			TGAAAAAAAAAACTGTTTTGTGCAAAGAGAAAGTCATATTGGAACAACATGAGTAAAATATGTAGTTATGTAGAAATATAGTTATGTGTCAAAAGTACTGACTATACTATATTTATCCCAAAATTCCATATAAAATCATACAAAATGAGATAATTGTTGACATACAGTAAAAAT[A/G]TAAGCAGCGTCATCGACTTGTATAGTTTCTATTATCATTCCAATTGCAGATTATATTCAATACTCTCTTTTCCAACTTCTGACTACTAGGTACAATCACAAATCACTGTATACATGGCAAACATCTCTGCCTGGATGAACAGGCATCATCTGAAGCGGAATTTATGAAAAACTGTTCATTCCAGCCAAGAACTA
LG4	ref-3190	81.133			CATTTGGCTCGTTCAGTTTCTCTCCTCTTTTTTTTTTCAGTCAACATTTGTAGTGAAACCTGCTCCGTTAGGTGTGGCGCTCAGAGCTTCTGAGATTGTTTGTTTAGAGGGTAATGCTGGGAGCAAAGAGATGTCCTGACCTCTGGGTAGCACATGATCAACCACACATCTCTCAAGCTGCAAAAAGGTCACAGAACATTACAATAGCTGCATATGACTCATACTCACAAATCTTCTGAGGCCATATGATCCAATTTTGAGTCCTGTCCTTTTTTGAAGTTTGACAGTTGTGGTCATTATCTATGGCCTGTAAAGGTTACAAGCTTTCTAGGCTTGAGACAAGTGACACATGAGAATGGCCATGATTAA
LG4	ref-27670_23	84.342			CATCCCGAAACGACTCCGGTGC[A/G]TCCGAAAAT
LG4	ref-72619	84.391	Chr3	49375023	NNNNNNNNNNNNNNNNNNNNNNNNNNNNNNNNNNNNNNNNNNNNNNNNNNNNNNNNNNNNNNNNNNNNNNNNNNNNNNNNNNNNNNNNNNNNNNNNNNNNNNNNNNNNNNNNNNNNNNNNNNNNNNNNNNNNNNNNNNNNNNNNNNNNNNNNNNNNNNNNNNNNNNNNNNNNNNNNGGGCTTTCTTGTGCATCATCTTTAGAAGAGGCTTCCTTCTGGGACGACAGCCATGCAGAATTTGAATGCAGTGTGCAGCGTATGGTCTGAGCACTGACAGGCTGACCCCCCACCCCTTCAACCTCTGCAGCAATGCTGGACAGGACATGGTTCCTTAGTCTGCTTGTCTTCAGCAAACTGTTTGCGGGCTGTGCATAAATTGTTGTGCATCATCTTTAGAAGAGGCTTTGGAACCTGATCTTTGGAACCTGTCCTGTTAAACCGC
LG4	ref-13092_15	85.804			TTGCCTCGAATTGATGGCTGTAATTCTGGCCTTGAAATACTTCCTCCGGCAGTTAAGGACTACCATGTTTTAGTGTGGGTGGACAACACGGCAGAAGTTGCTTATATAAACCACCAGGGCAGACAGTTCCTGTCCCTCAGGGTGGTTTACATTCCTGGGAAAATGAATGTGGGAGCAGATCTATTGTCCAGACAGGGTTAGGGTTTCCACCGATT[C/T]CTTTGCTCCCGGGAGTCCTGGCCAAGGTCCGTCAACAAGGGTCATACCTCTTGCTGACAGCACCTTGTTGGCCGACCAGGGTCTGGTTCTCAGAAATAATATCTCTCCTCGACGGCTCGCCCTGGTCAATACCGAACAGGAGGGATCTCCTGTTTCAGGCGCAGGGGACGATACTCCATCCCTGGTCCGAACTTATGGAGCCTTCATGTTTGGCCC
LG4	ref-54481	87.593			GCCACAGTTTCGATATTGGTGCATACCTGAA
LG4	ref-57313	89.14			CATCAGGAGGGATTACTTGCAAGGTCATGACTTGATCCCTGAAAGGGGTAGTGGGAACCTTAGGCACATAGCCGGGGTCTCAGGACCCGGCCCATGTGTCTTCAACAGAGAGGGCTTGAAGGTCCCCCACCCTCTTGACAGAAGTGAACGCAGTCAGGAGGGCCTTCTTCATAGAGAGGGCACTTAACTTGACTGACTTTAAGGGCTCAAATTAATCTTTCTGTAGGGCCTCTAAGACCACTGAGAGATCCCAAGAGGGGACGAGGCGTGGCCTTGGAGGGTTCAACCTCCGTGCGCATCTTAGGAACCTAATAATTATGTTGTGCTTCCCCACCAATTTGCCATCCATGGTGTCGTGATTGGCTGCTA
LG5	ref-36093	0			ACCCTGTTGGAACATTTCTAATGTTGTTCATGTAATATTTATATTTTATTTTATTACAACTTTATTTTAATTGGCAAAAACAACTTTTATTCATTTTAGTTTTAGTTAAAAATAACAACACTGCAATTAAGCAATTAAAAAAATTTTGTAAAACACGTTTGAGTTCTTCTCTTAAGTTTGCAGTGGTTTTGGCGCAGGTGTGGTAGGGTTTTAAGTTTTAGTTATAAAAATAACCCTGTTGGAACATTTCTAATGTTGTTCATGTAATATTTATATTTTATTTTATTACAACTTTATTTTAATTGGCAAAAACAACTTTTATTCATTTTAGTTTTAGTTAAAAATAACAACACTGCAATTAAGCAATTA
LG5	ref-13450	0.859	Chr19	41056608	ACCTCTGTAGACTCATTGGGAGGAAAGCAAGGCTTAATGGGGTTCGCTGATTAGATAACAACCACAATGATATGACTGATTGGGTTAGACAGGCTTGGTTGGAAGGAGGCTGCTGGGGTTAGACATTCAACACCTAGGAACAGTAGGCTTGATCACAAGAACAGAGTCAACCAGCTCTTTTATGTAAATGTGTTCATTTTTGTGCTTCAGCTACGGAGATGCCACGCATACAGCTGGATATTTGATGCATATGGCTGGTATCGCTTTTTAAATGCACTCTGCAGAAATTTCTTAAAACAATAGTTCACACAAAATGTTTATGTAAAGTGTTTATGTGCACTTCCAGCACGGGTTTAGTATTTTTTTTTAAAGAAGGGTGAGTAAATAAAATAATAAATACAACGTTCATATTATGTGGGGCTATTGCTTTA
LG5	ref-32563	1.858			ACTGCCATGCCGAAAAAAATGCGGGTTTAGA
LG5	ref-58702	2.284			ATTGTGGATTCGAAGATCTTGCGTAGTCTGT

LG5	ref-27571	3.079			CTCCTTGCACAGTTTAAGACAGCACTCACATAAAGCAGTATAGCCACCCTAAACAACACCACGTTAGCCAGATCCAGCTAGGTATGATCCACAAAACCATACAGGACACA<br>ACTCACCCAATAAGTGGAGACTTGTCTACTGGTGGTGTCAAGCGGTATGGGACCTACGGATAGGTAAGTGCAGAGGGCTCGCGAGAAACAGTAAAGTGAGGCCACAGCAT<br>TCCACGTTAGCCAAGCCTGGATAGGCAGGACCACAAAGAAACACTACTCGATGAAAAAAAGGAAACGTACTCACCGAGAGTGCAGAGTGGTAATGCCAAAACAACTGGC<br>AGAGGGCTTGCGGGGATTTCAAAAAGTGAGACGCTGAACA
LG5	ref-21478	3.714			CACAGACCGTGTTCACGGTTCCGTTGCACGGCCGAACGACCCGCTGATGATCCGAACACGTGTCGCACAGCAGACAGTGCGGGATGTAGTTCCAGAACTCCGTGAACAGA<br>CCCGCGCCGCACGGGACACAGTCCGTGTCGCGGGAGCTCGTGCAGTGAGCGCGCAGCCGGAATCCCGGCGCACAGCGATTGCACTGAAGCGCCTCTCCAGTGACCGGATC<br>GATGCGCCGGTAAGTGGGAACACCCCCCGCCGCCACTGCCGATAGAGCCGGCAAAACCAACACTGTGAAGAACAACTAGAGAAAGGAAATGACTGTTAATGATGGATAC<br>ACTTTGACACACTGATCATAGAATACTTCCTAAATTGTAA
LG5	ref-62631	4.112			CTGCAGAATCCGATGAGGCTGCCTCGCTGTC
LG5	ref-69424	5.117			AAAGAAGGGAAGTTTTTTTTTTTTTTTTTTAACTTGTATTCCGGAGTTGACAGCAGGCTGTACAATCAACTTGAGCGTCACGTGACAATCATGGAGGCACCAAAACAAAG<br>CGTCTAATCTTGAAAATTCATCTTTATATAAAACAAATCCTTCCAAAAATCGAATTACGTCCAACATTAACAACAAGTTCCTAAGTAATTCATATAACCCGTGACAGATGC<br>AACAACTAGCTGTGCAAGCTAGCTACACAGTAAGTGACCTCTTACCTACATTCACTTGCTGAAAAATATTTGACCTGCACATTCACATTCACAGCGTGATATTCAGGTAGC<br>AAGAAACTTAATCAACATTCATCTCGACAGAGAATTATATATGTGTTAAGGATTCAACGCAATAGCACGGTGTCATATCTAACGTTACCTTCCTCAAA
LG5	ref-48915	5.166			AGTAGTACAATCAGTTGAATGCTATTATGCTAGTTGTATCACTATTTCGTTCAATATAATCAAATCTTTCTCATTTTTGTGTTCTCATCCCAAGTTAACCAGAGGCAAAAGTG<br>TCCCGAAATAAACTATAGTAATGGTTTATGATCTGTGCAGTAGTTGTTCATTGGCAGTTTTAAGGAGCACAGATATCGCCTTGGAACAAAACGTTATAATGATAAATTACA<br>GGTCTTATAATGACGACCAATCTGTTTTGGGAAAATGTGCACTTCATAGTAAAGTTTCTGCTTTATCTAAAACTTCAAATTATCAGTCAGTCATATGAAATATTCTCTTGAC<br>AAATAAAACCATATATTAGAATATTAATAACTT
LG5	ref-15770	5.227			CTTACCCAAACGATCTGCTTGCTCCTTATTT
LG5	ref-3815_10	6.474	Chr19	1581181	ATTCCCTTTTGCCTCAGTTTTAAAGGATTGGGACTATGACGCTTTAACACCTGTTGCTCTTTCACGTCACACCTGGTTTTCAGTCGTGAAGACCTTTTGGTTGCCACACATCT<br>GAGGTACAGATGCTGATTTTAGCGAGACATCTGTCATTTAAGTTTTTCTTTGAGGCAGAATGCTTT[A/G]CAATATGCTCGCAGGCATATTTTTGCACATTTTGAGAAGTTGT<br>CAGGATCCTGCTGGTGAGGTTCTGGTTACAGACCACCTATCCCATCTGCCTCTCACCTTGCTGTCAGATTCGCCGGTCCCTTCCACTCACGTCGTTATTCCTTCATATTTATT<br>CTGTGGAAAAGCATCTGTTGATGGATTTTTGGTT
LG5	ref-47582_24	7.589			GAAAACACCACGAACATCATGCA[C/T]GCTTCAGT
LG5	ref-47582_14	8.101			GCAATAATATATTTCTGTCACAATTTCTTTAAGTTAAACCAAACTTTTATTTTGACAGGTTGCCATGAGTTTCTCTGTGTTTATGATATGACAATAGTTTTTCCTCAAATGAA<br>ATGGTAAAATGTTCATGAAGTGACTCTCAGAGCGGCTCTGGAGATGTTCATGCATTCATGTCCTCATATAGTGAGACGGCAGAGGTTGAAAACACCACGA[A/G]CATCATG<br>CATGCTTCAGTGTGTGTGTGTGTGTGTGTGTGTGTGTGTAGTGAACAAAACCGCCCGTCTGCGCCATTCATTCACACAGAGATGCAGAACATGCAGGATTCATATTTAAATAG<br>TCTTTTGCGGTTTAATATTCACAGACACTAGTTTATATTGCGATTTGATTTAAGTGTACTGCCCTATTTATTATTTATTCATCAAAAATTTGAAAAATTC
LG5	ref-29063	11.638			AAACTAAATGAAGAAAATAGCATAATGGGGCATTTGAAAAGGCTCATTGGTGACAAGAAACAATCAAACAATTACAGGAAACAACTAAATGCCACAGGATAAGAAATGG<br>AAAAACATTCCTTCGGTCTCAAGTAATGAGGATGAAGCATGAATCTGTCTCAGTGTGATTCTGTTACTGAAGGAAGGGGAAGTTAAAAACTATATTTAACACGACAGCAGT<br>GCAGACCAGAGAAAACACTGTGCTCAGTTCACATGTGAAGGCATTGTGTGCAGACCAGACAAAACACTGTGCTCAGTTCACATGTGAAGGAGTTCAAAGGCATTGCAGTC<br>AAAAATGCCCCGTTTGATTCTAAGGCATCAGAGAGAGTGAGTGTAACATGGGCAAAAAACTAAAGTATGATTTATCAGTATGTAATGCATGTTTGGCCTGA
LG5	ref-70048	11.981			CTGCAAAGCACGATGGACTTGCAAAACAAGA
LG5	ref-44969	15.746			TTAAGCGCCGCGAGTGTAGTGCTGTCCAGAA
LG5	ref-47676	18.343			TTGCACATAATTTATTTGCATCTCGCGAAGAGAAAACATGGACTGCAAATATTGAGACTAGCGTGGAATTTTCTCACTCAAACCACTGCCTGCTACTGCTTCTGATTGCTTT<br>TAGAGGGAAAAGCTGCCCATAACTGCTGGTTTTGGATCAGTTTTCTCCGTAATAGCATATTGGTTTGGCAAATGATGCGTAAGGTTTCTATATACAACATGTTTTTCAGCGT<br>TGAGCAATGTAGCCGATCACAAAGGTATCTGTTGATTTCTTGAACTCAATGGCCAATCAGAGATCTTTAAGTTAGCATTGAACAAAAACTTGGTGCTCGGCGGCACCTATG<br>ACTAATACCACCGTCATCCTCTCAAACAACTTGA
LG5	ref-58489	20.491	Chr19	2502754	TCAATAAGCTGTTATGTCATTGACACCTCAAAAACTCTTTCAGATTCATGGCGGTAGCCATTTTGTTTTCTCACACTCTTAATTAAAGGCAATTGAGACGATGAGTTGTATT<br>GAATCTTATTGATTTGATACAAGGATCCTTCAGGGTTTCAGAGTCAAGCTGAGAGACTGTTGCAGTTTATGTTATTTATTTTGTCAGGGTTTCAGAGTCGAGACTGTTGCAG<br>TTTATGTTATTTATTTTCCTTTGGATGGATGAATTTATGGATGGTCTGATTGATGGAAGGATGTATTAATAGGCTGATTGAAACAGTTTTTCCTGTCACATTCATCACCTTGT<br>TTTACAGTAACATCAATGGTGGGTTGTTCATGCATACTGTAGCTAGACACGTCTAGGGTCCTGCTGTCACGATCCCTAACCAATAAATTCCACC

LG5	ref-40855	21.114	Chr19	45576389	CGTCTGGATTGAAAAAAGAAGATGAATTTGTAATGGCCTGTCAGTGAAGGGAAAAAAATCTAATCAGCATAAGCTGTCACTGCTAGGAGCTAGCTCTCTGCCCTATTTATATTCATGCCTCATTTGATTGGCATCACCTCCTCTGGTATGCCCATCATTTTCTACCATGTTGCATTGCCCACGTTCTTTCAGCCATGTTCGACGGCCCACCGAAGGACACGCATACCTGTCTTTCAACTGCTAATCGTCAAAATTTCACAGAGCTGTTTCCCTAAAACTGATAAAAGAATAAATGACAAGAAATACCTTGACACCATTGACTCTGTTTAGTCCATGTTTGAATAGGCTGACATTTTTAAAGGATGGGTAATTCATGTACGGCTCGAAAATACCTTTTAACATTAAATTTAAGTATATGTCAAACACGTGGA
LG5	ref-38844	23.655			GACGGCACATGCTAGTGGATGAGTTGAATCAACTCCACAGCAACTACATAAATTTATCCACTAACCATTCAGAAACGTCTAAAAGTTGTAACTTCTTCCTGAGTCTCTCCATCAGTGTCGACTCCGGTTTGAACAATGTAAGGCTGAACACCGTTACTGACAACCCTCATTTTCGCTGTGTGAGATTCTCCAGCTTTGTTGTTGAGCAACCGAAGCATGAGCTGTTCTGGAAAGGGTAGCTCATTTGCATTTAAAGGGACACACACAAATGGTTTTTGCTCACACCCAAAATAGGGGCAAATTTGACAAGCTATAATAAATGATCTGTGGGGTATTTTGAGCTGAAACTTCACAGAAACATTCTGGGGACACCAGAGACTTATATTACATCTTGTGATGTATAGGCATTATAGGTCCCCTTTAAGCAATAC
LG5	ref-11524	24.978			TAATCAGTACATTTGTTCCAGTATTTATTTATCTTTTTCAATAAAAAATACAACTGTTCATTGTTAGTTCATGTTAGCTCAGGTCCATTAATGGATCCAACTTTTGATTTTAATAATGTGTTCATAATACGGAGCCCTGCACATGACATACGAACAGATATAGCTGCAGTTTTGTCGCCTGCACTTTTATCGCTCAAACTCTTCAAAGCAGGATGTGTTCTGATTGGCTGTCAATGTTTTTATCGGGAACGAATTAGTAAAATGTGCTGACATATTAGTAAGTCGTGGGAACGATTTGTTATTTCGTAGGCCATGATTTTCCGCATGTGGGCTCAGTATTGGCCGGCTCTTGCATCAGCATCATACGCAT
LG5	ref-104	25.105			GCTGACGGAGAGCTGCGTCACTCGAGGCCGCGTTCTCAAGCTCCTGTAGTGCTCGGATCAGATCTGCCGTCTGGGACACGCAAAACAAGTCCTGACACGGCTGACGTATAGCTAAATATGTGCATGATGTAAGGCTGTTCTTTCATACCTGTGGAGGCTCACCAGGGGAGCTTTGGGAGGCGAAATCATCGTTGTTCACCTTGATTTCTTCATAAGGCCTTTTCTTGGCCTTCTTTTCTACCTCTGTAGGTCATGAGGAGACGTTCTACATGTGAAGGACTTTCTCACTATCAGCAAACACTTATCAAAACAGAATACTGAGTCTTTCTGAACGACTATATAATATTTCCAGTAACCTTCTCAGTCAGT
LG5	ref-39603_4	26.2			TGC[A/G]TCGAAGCGACGCATGTGCGTTCCAATTT
LG5	ref-65446_7	27.779			TGTATAAATGTACTAGTCAGGAGTTGTTTGGGGCTGCTGACCCCCACGCCACAGTGTGACCTATATACAGGCCTGGTTTCTGCTCTCTACAGAGCATTGAGAGTGGACCACCTGCTCCATTTTGTCTCCTATTTCTTTTAAGGACATAATTGTTTGTCAATACCACCCAACTTTT[A/C]CCAGCAGGGTCATCGGGGTCAAGCTTATTGAGGACAGCTGTGTGTTTCTAGGTCAGGGTCCAAGTAGAGCAGGGAAAGTATAAGTCTGTTTATGCACTTAACTCATTACCTTAGGTCGCCTATGACGCCTTCCGCAGGTCATCTCCAACCAAACACACATGGCTGAACTCGTGCAGTGCAGTTCATGTTCAATC
LG5	ref-61907	30.159			GATTCAGTTGCGAATGCAGTGCATTTGCAGA
LG5	ref-46638	32.91			GTGTGCTGAGAGAAGAGTTTTCCTGCTGTCAAAGCAAGTCACGTGAGACCCGGTCTGAACTTCAGACAAACTTGGATAGGGAAAGCATGCGCACAGCAGATGCCACAGAACAGCGGAATGGAAGTGTTCTCACCCTCTAATAGTCGAAACTTATTAGTTTCTATTCAGACGTTTCCTTTGCACAACAGTTGCATGCGGTGAGAATATTCCTACAACTGTGACTGTATTCACTTTTATGCGCTGTTTGAATAAAGCCACACTTCACATTCAACACAAGTTCAAGCACAGATTGAATCCAGTCAGAAACAATGAGAGTCGCGCGGCCTGCTATATTCACACGCATGCGCAGGACCGCTCGTGCAGGGAGTG
LG5	ref-50375	33.423			TGATGGTAACACATTACAAGTAACTTAAGTTACAGATCACTTTTTTCAAGTAACCAGTAAAGTAATGCATTACTTTTTTAAATTTACAACAAAATATCTGTTACTTTTTCAAATAAGTAACACAAGTAACTTTTTCACATTTATTGACTGACAGCTCTCCTGTCCCCATGTTGAGAAGTGCAGAGGTGTTGTGTGCGCTGTGTGAACATGATGGTCATTGTAGTTCTAGACTAAATGTGAGCATAAATTTACTCAAAACATTCAAAAACAGTGAAATGCAATCTCAGAATTTTATGCAAACCTGTAATAATTAACTGTATTAAATTATTAAATTACACAAATATGATCTAATTCAACCATACTAATTAT
LG5	ref-71189	36.306			AAGGGTTATGCGAGGGCGATGCGGTTCTAAA
LG5	ref-3427	37.695			CGAAATAATACGAGTCTGCTGCTAATCTACG
LG5	ref-29234	39.42			TGGGATGTAACCTTTAAAACAGCTGTATGTGTATGTAACTTCCTTATTATTATGTTTAAACGTTAAATTGCAGATAACTCAATGATTACTTCAGGACACTCCCACAAGCTGTGATGGTCCTTGAATGTCTTCTCACCCAAACCAAAGAAAATGTTTGTATTTCATATAGCCAAATTTTAGAAGTCTCTTTGATAATTTTAGGAGTCTCTTCGATAATTCTGCCCAATTGGATGGAGCTGTAACCAGTGTGCATGAGGTGATGACCCAATGATGGGTAAGATTTTCCGGTGGCCAGTGGAGGACAACCTAAGGCAGGGCTTCACCGCCACTGGGCACCTGCCCAAAAGGGAAGGGAGGTGTAAAAAAGCTCTGTGAAAAGGGAGTGGATGTGATGCTTCTT
LG5	ref-12780	39.9			GACCCCAAATAACATGATATTTAACCCCTGGAATGCGAATTTTACCAGGGGAACCCCACCAAAAATGTGAATTTTACTCCCCGGAACACGATTTTTACCATAATACCTTAATTGGGATAGTTTTGGGATAGTTTTGAGTAGCAATTGGGTGGGTTTTGTTGTGAAAACCTGGCAACCCTGCACTCAACTCGTATTTACTTGCATGTACTTGTGCTGCTGGTATGTTGTTTAATATAGATTTGTGTGTATGATTATAACATGATTCTTTTCTGCCAGTGTAAACTTATTAAACATATTTACAAATATTCACAAATTGTAAGCTACACTGCATATATTAAAAAAGGGGCTTTTAATGCATTAGTCATAAAT

LG5	ref-1292	40.001			GGGCGGCTCCCAACGCCCAACATAAACCGAAACAAGACAGCCCAGGAGGGTGGTGTAGCGGAGGTGGTCTTGGGGGAGGGATGGTGGGCCAGGCCCGTGCAGGGGAACAGGACAAGGACCTGGGCGTGGCAGAGGCAGGCATGGGCCATGGGGCATGGGTGGACTTGGCAGGCTAGGCCACCTCCAGGGCGGTACTGGCAGTTCAGGGAACGAAGGTGGCGCCGGCAGCTCAGGGGGCCATGGAGGGGCAGGCAAGGGACCATGAGGGTGCAGGCAGTTCAAGGGACTGGAGGCTGAAGCCAATGTGTGTGCAGCCCACACACATAAAATGGCCGTTGCCATAATGACAGCATTTTCAGTAGACTCTGGTGTGGTAGTCATCTTGGCTGAGGGCTCAGGCGTGGCGGCCCTGGCATGGCAGATTTGACGTG
LG5	ref-18045_30	40.838			CTTTATATATAATGGCTATTGACAGACACTGAGGAAGCATGCAAGCTTTTAAAAAAAAAACATTATTCATTCTCTTAGTTTTGAATGGGTTATCAAAGCCTTGTTTAAAAAAAAAAAAAAAAAAAAAAAAAAGTATTTGGACACTTAAGTCACACTTAAATCAAACCAAATGGCATTTGCACAAATATCGAGTTTTT[A/G]CTCTTCATTTTGAACAGCATTTATTTGTATTATTTACCATAAATGTCATTTTTGTAAAATGTTTTACTTGGAAATATATTTCTTTAAAATAAATGCCATTTGGTTTGACAAATTGTACGTTCATTTTAAGAGTGGCTTAAGCGTGTGAGTATTTTTTATGGAGCCATTTTA
LG5	ref-62518	41.218			ATGTGCTCAACGATTGGCATGCGGGCTCCGT
LG5	ref-10740_24	41.622			GCGAAGCTCTCGACCGCGTTGCT[A/G]AATAGGCC
LG5	ref-10740_18	43.329			GGGTCTGAGCGGGCTGGGTGCCGCGTCCCCAACCGGCTCCCTGCTGTCTGGTGGGTGGAGGCTGCTGCTTCGCAGACTTAGCAGGGGCGGAGGACGACGCAGGCAGCACCCTCGGCGACGGGGTGGAGGCAGCAGCGGACCGCCGGGGCAAGGTGTGTTTGATAGCCTCAGGCTGCTTCTGTGCGGCCGAGAACTGCTGGGCGAAGCTCTCGACCGCG[A/G]TGCTAAATAGGCTGACCTGAGACACAGGGGAGTTCAGAAACTGATACTTATCGGTCTCCCTCATGTCCGCCAGGGTCAGCCAGAGGTGGCGCTGCTGGACTACAAGCGTAGCCATCGCTTGACCAACAGAGCGAGCCGTCACCTTAGTCGCCCAGAGGGCGAGGTCGGTGGCAGCGCGCAGCTCCCCCAGCAGCTGTTGGTCAAGACCACCC
LG5	ref-68935	43.363			CCAGAATAGTCGATGGGAATGCATCTCTATC
LG5	ref-72037_8	44.572			GAAAGCG[A/C]CCCGACCAGCGTGCTCAGCAATCG
LG5	ref-33374	45.798			ATATAAGGATCGAATCCTATGCAGCAGACCC
LG5	ref-835	46.098	Chr19	6166750	ATAAAACTTGATGAAGCATATTAATTCTCTATTTAATTATAGATATTTAGTGCTGTTTTACATTCTAATTTACAAACAAGACCAGATTCTGCATTAAAAAAAAAGTATGTAACAAAAAATACATAATTTTCAACATATTGACAACATGAGCATCTAAAATTTCAAAACCATATCTATATCGTACACCACTTTGCTTGTAAAAATCAATCACGAAAGAACTGCACAAAAACCAAGCGTTGTGTTAGGTCTTTAATTTTTTCAATAATCTCTTGTTTAGAAATATTGCACATGGTCAACATGCCTGTTGATGAGTTAACACTCACCTTTCTCATTCTAAAACTCCTTCTCCAAGTAAAGACAGTTTATTGGATACACAATCATAAGGTGTACACGCATACATGTGCAGGTATGTGTATGTGTGTGTGTGTG
LG5	ref-14244	46.263			AGCTTACTTACGATCTACATGCGAGCAGCAG
LG5	ref-54647	46.745			GGAATACAGTCGATGGGAATGCATCTCTATC
LG5	ref-37807	47.183			ACACCAACGCCGACTGTACTGCTCAAGATGC
LG5	ref-24339	47.486			CCCCAAACACCGATCGAGTTGCGTAATGCGC
LG5	ref-17847	48.31			AAAGTGGGACCGAATTTTGTGCTAATTGTCA
LG5	ref-1026	48.637			TGTTTCTTACCGAGTACGTTGCCTGTTGGAA
LG5	ref-845	48.954			GTCAGCAACACGACATCATTGCATGGCATCA
LG5	ref-3199	49.623			GGAGACAGATGAGTGGCGGAAAACTAAACAAAGGAACATGTGACAGGTACAAACAAACTGAAAGTCCAATCAAACTGAAACTAAACAGTAATGAACATGACAGTTCTTCTGGGCTTTGGAGGAGGATGAGGTGATGGAGAAGGGATGGTGAGAGGTAGACAAGGAGGGATGATTGCAGGATGTGGTGAAAGGATGGTGATGGTGGGACGACGATCCCCTAGCAGCGATGATGGTGACTGGCCGATGAAACAGACCCAGAGCTCCCCAGTGCCAACAGCCCAAGGCGGAGACGAGGGAGGGAGGAGTCCAGGTGGTGACGAGGCTGACAACAGCTGGGGGACAACCTACGGAGACAGAGACAGAGCCGGAGGATCCGCAGGTGCAGACTGAGGGTCGAGGGAGCTAAGAGACATCGCTGGTGCAGGAGACCC
LG5	ref-47918_23	50.228	Chr19	9767003	AAACACATTTATTTAAATGAAGTGAGACTACTCTCACTACTCTGTCAAACCTCAAAGTGAGTCAGTGCAGCACATATGTAACGCTGGTACACTAAAACACGGGAAATTCAATAGCATGATTAACGGTCATAATCTAGCAAATTTCCTCCCTGATCACATCATTTGCTAAAAATCTTTATCGTGGAAATCTGAAATTCATTGAACACTGGTCGATGCATTTGC[G/T]TCCTCTATAATGCACAGTAAAGTGATTTTAGATGAATGAGTGACATTTATCTGAAGAGCTTGTTTCTTTAAATGTTTTGGTAGTAAACTAGCGATTTTATTCTTGGCATAAGCTTTATTAGCTAAGCACTTCCTATAAATATATATATTTTTAATTAATCTTCTAATTAGATGTTTAAGTGTTTTGGATAAATGGTAGTAGTGATCTGA
LG5	ref-42661	51.429			ATAAATACGTAATGTAAAACTAGTATTGGATAGATATATAAATATTGCCTACAACTTTATCAAGTTCAGAAAGTGATTCTATCAGAGACTTGTGACAACAGTTAAAGTTCCCCTATTATGCTTCTTTCAGATATTACCTTTCATGTAGTGTTGTGTAACTGTAAATGTAAACTGTCAGCAAATTTTCAGAGATCGAATTGCAAAATAATTAGATGTTATTGCCACCTGCACTTTATGCTGATCATCTGCTTAGCAGCTGGTAAAATTATGTCTTCATCTATAGCATTAGTCTTTCCAGATGTATCAGTCTTGATACATTATATGATGCGGTCACTGACTTGAGAGATTTACAAGATTATTTACGTCTTTTATAAGGCTCTTATTATATTTACAAATAATTTCGAAAATATTTTGATACAAAAATTACAATA

LG5	ref-41704	51.511			AGCAGCCCTGTGTATATATATATATATATATATNNNNNNNNNNNNNNNNNNNNNNNNNNNNNNATGTGTGTGTTCTAGAAATAGTGCAGAATGCTAAATATTTATTACTCATTTCATTTCATGTCATAACATTTCTTCATCACATTTCTAAAACATTTACAGGTTTAAATTATTTTTGGAATCCTGGTTTTCAATGTGGTCTCAGGCTTTTGGACACCACTGCATATACCATGGTACTAAATTATTACCATAGTATTTTAATAAAATACTATTGTGCTTTATGTACAAAAAAAACAAAATTCCATGTTAAACGATGGTACTTTTGTTACTTTTTGTTCTGTTGACATATCGCATTAATGTGATTGCAGTAATTTGCACATAGTTTTTACACTATTTTATTTTACTTTATAGTTCAAATATACAGGATTGTAT
LG5	ref-27134	52.287			TGGGGTTACACGAAAAAAATGCTGAGTTTTG
LG5	ref-58051	52.772			GTAACATCTACGAGTCCGCTGCCACCACGCT
LG5	ref-8450_30	53.029			TATACTTACAGTATAGGCCTATTTGTGTGTGTGATTTTATACAACAGTTCAATAACCAAGAAGTTTATACATTAAGTAACCTACATTTTAGATACAGTAATGCCGGTTACTTTGTTGTTGCTCCACCAATGAAAATTGTTCCAAACAAAAACACAACAGAACCGTTACAACACCGAACAGCAGTGGTTTCGTTCCTGG[C/T]TGATTCAGTATTTTTAAATGAAGTGATTTAATGAATGATTCAATGGCTCATGGTAATACTTAATTTTACAGTGTCCTTGTTACACGTTACATGTACTTATTATAGTCATAGCAGTAAATTATGCATAATTACATGCAACTAACCCTAAACCAAACTAATTCTAACCCTATA
LG5	ref-16735_24	53.388	Chr19	20487033	CTTGTCTATATGTTTACCCATTTCTTTCTTCTGTCTCTATATCTCAGATACATCTCGGCGGCCTCGCGATGACGAGCGAGATGGTCAGTCGTATAGATTTGTAACGCGTCTAGAGATGGAGACCGACATCAAGGCGGGCCGGTTCTTGGAACACGGAGAGTACGACGGCAACCTCTATGGCACTAAAATCGAC[A/G]CCATCCATGAGGTGGTGAACAAAGGTCGTACGTGTATCCTGGACGTCAATCCTCAGGTGAGTGTCTTGACATGCTAATCTCTCAACAGCTCTCAAAGTCAGAGAGGTTCAAAGATATTATATTATAATATATGAAATATACTGTACCGTTCAGTAAGTTTTTCCCACATTGATGAA
LG5	ref-35758	54.9			TCCTTATCCTTGCCCCTTTTTCCTGCTTCTAATACATCAACTTTAAGGAGTCAGTGTTATTCATTTCACCTGTCAGTGGTCATAATGTTACGCCTGATGGGATATATATATATATATACACACACACAAGTAACAAATCAAACATACAGTATCTGCACTGGGCACAGTTTTGACATAACAGCATACCCATCGCTCGCCACTTATATATATATATATATATATATATATGTGATGTGTGTGTGGGAGATCTTAATTGTTCATTAAGAAAAACAACTGGTTTTTGAAAGCATTTAATAAGATATGTGATTTTATAACATGTTTTATAAGATAATTAATAATTAATTCTAATAAATAAGGGTGGATCTTACTT
LG5	ref-13841	55.18			CACGTACAGACGACAATGTTGCCAAAACAAT
LG5	ref-44354_16	55.775			AATGCAAAGGAATTTTGATGAAAACAGTTTCACTAAAAGGACCCTGGAAAAACACATTAGGCAGTGCCTTGAAATAGAAAGGGCTAGTACTGAATTCCTGAAATCAGCTCTCAAAACCACCTATGGAAATAACAGAAGAAGCAAGTTGGTTGATTTTCCAGCCTTTGACCTTATTGAGTAAACTCCACAGGACATAATGCACATCATTCTCGAGG[G/T]TGTTGCACCCATGGAAATTAAATGGGTGTTAAAATACCTTGTTCTTTCGGGACAGATGGAGCTGGACATGTTCAATTCAGCCATACTGAGTTTTCCCTTTTCACCAGTAGACATACGGGACAAACCTTGTACGATCAGTGTCAGTACTTTGGCATCTAATGATAACAAAATTAAACAGTCCTCTGGTCAAATGTTAATACTGTTGAAAATTATGCC
LG5	ref-20777	55.974			ACATTTGCTTACCTTCTCTAATAACGTGCAAGAGCAGTGTCGAGCTCTGTCAAGTCAAAGTTGTCGCAAAGCTCTCATCATCTCGGAAACGCCACGCCGATATTGATTCTCGTTTTATTTCTCCTTTTGTCCCAAAGTTTGCTTGCCATGGTCCATAATGTTTGAACGAAACAACAACAGCATGTCTGTCGCTAGACGTTATGCAGCTGTCCGCGTCTGTTGCCATGGTTACTATGCAGTACAAGGAAACCAGGGATAACACAGATATTATGTCTCTGACAGGCGACAAGTGACAAGGGAGGGATGGGCACTATTTAAAATTATGAATTTCTCTGAATTTAAAAATTCTTGGAAACATTTAGGATAATG
LG5	ref-57432	56.105			ATTAGAGCAGCGACATCACTGCCTGACCTCA
LG5	ref-24754	56.157			TACGTCCACACGAAGCCGGTGCTTACCCTAT
LG5	ref-41876	57.806			ATGGATAATTAGACTGAGAAGTAATGGAAACCTGAGGTGGAAGGGCGTTGCCTTGATATGTGGCATCAGCTGTCTGATGCTGAGGTACAGTGAGATCAGAAGTTGTGTCTTGGAAGGGAGATGTAGTCCCTGTTTTGGAGTTAGAAGAGACATGCTTTGAATGCCTGAAGTGAGATTTGGCAGATTCTTTGCCTGTCTTCTCGGATGGTTGATTTATGGAATGATTGAAACATACAGATTAAACATCTGTACTTTGCTGGCTCTGTGAGAAAGCAGGATGTTAGCAGCTGCTAGTGCATGACGCAGACCATTTAACATCCATTTTGCAATAGAAGGTATATAATGGCTTGCCAAATTCATTCGTTGGGA
LG5	ref-54052	58.598			CTCTAATGCGATCCTTCGCGTGAATACCACGATTATATATTCAGAAAGAATAGGAAAAGGGATGGGCAGCGAAAACAATAGCGAAACGGATGAAGCACAGCTCGTTTTTCCAGCCTGTCAGAATCAAGGACAGGATACATGTATAAATAAAGCCGCTAGGTCGCTTCATTAATACTCGAGCGACATGATCGCAGTTAGGCAGCTTAATCCTGTTCCGCCTGTATTATTGTCTCAATGAATCCCCAGCCTCATAGATGGAGCTATCTGGATCTCTCTACCCCCGCGATTAAGCCCACTATAACGCTGCTCAGCAGCAAGCTTGCGCCTTCATAGCGATCGATAAAGCCAAGGCCAGGGGGTAGACTAAAT
LG5	ref-60200	59.709			CTTTAATTAACTAACTATTACTTTTATGCATTCAGCAGACGCTTCTATCCAATGCAACTTATTTTAAACATTGTTGGATTTTGGCATGATTAACAGAGTTGATGTTGAATCATTGCAAGGGACAACATTCAGAGAGAATGTGTTTGCCAATTAATAGCAGTGCTACAGAGCAGATGTCTGCATCTCTCTCGTCTCTCGTCTAGACACACACCAGTTCTCACACCTCCACCTCCTCACAGAGTGAGCAAAAACAAGAGACATCAGCATATCTGAAAGATAAATGATGCAGTGAGAGATGAAAAATAGTTTTTTTCTTAATAAAGAATAAATTAATAACAAATGGAGAATAACTTTGAGTTTATAAGTAAA

LG5	ref-60340	60.124	Chr19	42799021	ATGTTTTCTTGTTGTAGGCCTCTGCCTCCTCTGATGGAATGCTGATGCTGGACTTGGTGAATGAGGAAGATGCTGGTATGCTCGATGACGATCAGGATGAGTGGTTGAAGG ACCATCGGGTCAACTTGGATAAACTGGCTCCTGGCCGTCGCCGCGCTGGCACCGATGCATCCAGGCCGCCGTCCTCCATTACAGAGGCCAAGGTGAAAACCAGCAGCTTG CCACGCAGGACAGAGTTCTCCTGGAGCCATGAAAACCAGCCTCAGACACCTCAAAGTGAGAAGAAGTTAGCTCAGGGCCGTAACCGATGTGCTTCAATGGATGACGCTCT GTCTCGAGGAGAGCGAAAACTAACTAGGAGGAATGGGCACCAGGCGACGCTGGCCCAGAGTCCCACGGGCCACCTGCAGGATCTGATCACCCAGCGGCTG
LG5	ref-12650	60.458			GCGTGAAGAAGTGTCCGCTTTGCAATCTTCTAACGCTTCCGTAAGTCTTCTTTATGCATCTTTATCTACTCTGTGGAATTTAAGTTGACTAGAAGCCTAGTTGTGTTGTCAGA AATGCGCAGGGAGAATAATAACGGAATGGCATTTGTGCTTCTTGTATATCTTACATAACCGTGAATGTTATACCAGTCTGCCTGAACTTCAAAACGGTGATTCCCATGCAG GTGCACGTGTAATCCCGCAGGATTCAGGGGATATATGGAAATATTTCATTTGCTTAAACATACAAACCCTACTGAAATGATGTAATAATTGTCAGTTTGTTTGGATTGTTAT TAATGATAATGTAATGTGCATACAACTATAGGCTACACATTGTCATGTGAGATAAAAGAAAAATATATTGTATAGTATAGTATGTGAGATTTACT
LG5	ref-47412	61.13			AAAACGCATTCGACCAGATTGCAAATTGCAT
LG5	ref-35811	61.983			ATATGACTTGTTTATTCCCGGAAGAAAACTTAAGACTACAATGGACAGGGAGTTCAGAAACAGTGCGCACTGATATAGAGAATAACTCTCTTTGGAGTGACTTTGTGCTTT GTATTTTTGCTTCGTGATCAATCAGCAACATTACACACTAAAAAACACACTTTTTTTGTGTGTGTAAAAAAGCATAATAGGTCCTTTTTAAAGAACAGACGACATAACTGCA GCATATAGGCCTAATAAACAGAACATTATCATGTGTTGGTGTGGATGTATGTATAGTTATCGTTCTTGGTGTTAAAGGACCTCATGCTGTTTCAAACCCATATGTATATCTT TTTTCTGTTGAACACAGAAGTAGATATTTTGAAAAATCAATTACAATGGGGACTGATGCCTTCAAGCTTCAAAAAGGACACAAAGCACTATAAAAG
LG5	ref-23770_9	62.35	Chr19	7546617	CACATGCTGTAATTGTTGGAATTGTTAATGAAGGGATCACAAGCCTTCATTCATAGACTGAAGGCCTCAATCACAATATCTGCTTGTATATGTGTTTCGGTTCTGAGGTTTC CAGCACAGCCCGTCTGCTTAACATGTTAATTAAATTCACTAGACATGTGTTGAAGATCTGTGCGGAGCGGAGCCTGTTACACTTGTTCTCCAACAC[A/T]ACGATTCACCTG CTGGAAATTGAGCTCCATGCCCTCCGCTTTCTCTCGCGGTTTGATGGACAGCACACATTCACCTAGAACGAGAACAGAATCAGACTTTTAACACTTCAGCTTTCTGTCAAAG GTTGTTGTCATTATGTGATTAGTGTCACCTTTTCGTGTCAAGCATGTTTGTATAGTTTGTGAAAGCACTTCTGGTTTATCTCCCTATCGCTCAACAACA
LG5	ref-49332	62.974			CATCTGCTCTCGATGATCTTGCAGTTTGAAA
LG5	ref-24311	63.909			AAGTCCATAGAATAAAACAGTCTCTTCTTCATGTAAATGAATTAAAAATTAACAAGTTTTCCTTTCGCTGTTTGATATTTGCATTCAAAAATCGTTCATTTGCACTTACAATC TTGTGGACTTACTGTACAATAGTGCATGTGATCGTGAGATCACGAGACCGTAGGGTGTCCTAATTATTAATTAAGGTTTCAGAAGAGTGAATGCGAGCGACCCCTTTGCGG TTGATACACACCCCTCGATGCGTACTTTTTCTGAGCACTCACTGAAGTGAGCTCCATAGCAGACTTCTACCCATCAGACAAAGCAGCATTACGTCACGAAACTGCAGACTC GTGGGAAGGCCCCGAGTGTTTAGGTGAGTCCCGTTTGGGACAGGGCCCAGGTACTACATTTGGTTAAGAACTTATTTGAGAAACTAATGTTCTAT
LG5	ref-1038_30	64.458	Chr19	21786071	CGCGTTTTATTTTTCTTCTTTCGAGCGTGTTTGTGTGCGCTTGTGTTTTTTTTTTTTTTCAATCTGAATATGGTCTCAAATGTGAATCTAAAAGGAATACAATTTAATAGTCAC GCTTACATTTAGAAAAGAGACAGTTATCGAAGATGTAAAGTTAAAATGAAGGAAAAGTAAATGCAGCAAAAATATCGCAAACCA[A/T]CTCAGGGCACTCAGTGTTTGCG CCCCGGCTATCCAGAGGACGGAGTTGCGTGGAATGGATGCAAACCGAGCGTGTCTCTATCTGCGTGGGAGGTTGTGAAAACACAAAAACAAATGAAACCTGGAGCAGCG GAGAGATACCGCATTCATCGCCGAGTCCTCCCCTGGCTCC
LG5	ref-1038_32	64.473			AGGTGGTTTGCGATATTTTTGCTGCATTTAC[C/T]
LG5	ref-23081_9	65.37			GGGGCAGACCGAGGGGAAACAGGGAGGGTGGAGAAGAACAATCGACTGAAAGTGTTTGTAGGCATTTAAATCATAGGCTGGCAATGGTGCTGCATGGCTCAGAGTCTGG CTGACTACAACTGATCACATTAAATGGCCTTACAGTCATGCAGCGGTGCCAGCGCTCCCAGTGTCAACACACACACACACAGCTCTCTCCCAGAGGGACG[A/G]CGAAGCT GATGCTGAGTGGATTAACCATGCAACACAGAGACAAATCTGCAGACAGACATTCCTGTAGTATTCTCTGAAAACAAGTTGTTGCTGCTGTTTGCCAATCACATTGTTAAGA AATACATTCAAATATAAACGTTTCTTCAACTACGGGCACTTGTGGGCCAGTGGAAAAACTCTTTTGTTCATAATTTCACTAGTTTTCTACTAACAATGTCCAA
LG5	ref-58895	66.127			GACGCTACGTCGAATGACGTGCTTGTAAATC
LG5	ref-5094_14	66.267			CACTCTTACCCGA[A/C]TGCAATGCTCCAGCAGAA
LG5	ref-13910	66.611			TTCACAGATGCGAGCTCCATGCCGTCAGCGG
LG5	ref-22954	66.849	Chr19	35531838	CAGGAGGCACTTGGGCCACAAGCTTGTAGCAACAAAAGCCTGGAGCGTTTGCGTAGCCAACAGGAACTGTTAAACTCTGTACAGCCACAGGTGGTGTACCTGCGAAATCT GGCTCAGGGGCTGCTACAGGATGCACCCGAGATATCCAGGGCTGGCGAAGATGGCGGCCAGAGGCTAATGCAGCAAGCTTGTGATACAGAAAAAGAGTTTGAAGAAGTT ACTGAGAAGGTGAGAGAATCTGCACATGATCAGGAGCCACTGGAATGTGTTAGGACTCAGGCGGTAGTTGGGATGTTATATATAACCTGATACGTAGTTTATGTCTGCGCT CATGTTGTGTCTGCTGAAATAAGACACAGTCTTTTTCTG
LG5	ref-32875	67.039			TGATGGTCGTGGAGACAGAATGAGCACATGGGAGATTTTTGAAAAAAAAAAAAACGCAGAAAGAGAAATGATGAGACACAATACAAAAGAGCTCAAAAGAATATCACT GGAATGAAGGTTTATGACTGGGCAAGTGCTTTAGGACCTGCATTATTATTAGAAAAGCTTTCCAGTGCTGGAGAGAGCTAAGCGAGAAAGCCTGAAAACAGACAAGAGTT GTGCACACTGATAAACGCTAGAGAAAGCGATGCCAGAAGCGAACGGGCCAGGGTTCAAGACCAGCTCGGAGCAGGGTGGTAAGGATTGTAGAAGAGTTTTGGAGGTGGA

					GCAATGAAGAGAGGGGTGGGTTTGTTTGGGTTGATTTCAAATATCAACAATGTCCAACAGTGTTGCTGAAAAAAATGGATACCCTACCTTAAAGAAATGTATAT
LG5	ref-26044_4	67.472	Chr19	36379023	ACCAGCCTGAGAAGAATTCTTGGTGGTTGCCAGTGATGTTGTTGGTCTTCATATCACACATCTAGTTTCAAACGATGCCAAAAGTGGAACAGAGCCGCCTCCTCGCTGTTTG CCCATTTCACGGTAAATTGTGCTAGCGTATGCATGGTGATTCTGAGAGCTGTAGCCCACAG[C/T]ACTGTGCACACACATCACACTACAGCTCCAGGGACCAAGTGAGGAG AAAAGCAAAAAATATTGTGTAAATACATGCTTTTGCAACCAGTTTCAGAGATATGAGATTCTTCGGCAAATACACTGGATTTGTGGGTATCTATCTATAGAATACATACAA GTAACAAACAGCTTACCTGTAACATATAGTCTAGTATGT
LG5	ref-28546	67.685			AGCATTAGACCGAGCTGCTTGCCAGGGCTTC
LG5	ref-59973_3	67.817	Chr19	38948338	AATGATTCCTCATTGACAGATAGCACACAGCATTTTAAAAACTTCTGCATCACTTCCTCCCATAAGTCCTGAAGTGCAGTAAATTACAATGCAAACAGTTTTTGATATCCAC TTTAGTTTTTGCTCATGAGTACGCAGGGATGCATATATATTCGCTCATGCATACAAGAC[C/G]ACTCCACGCATTTGGCTCGGATATACCCTTCAGGCACAAATGCTTTTAAA AAATTATTGTAATGACAGATTGGGGAATTAACCAGCCAAGGCTTTCATTTCATGTCTGATAAACAAGAGAATATGCACTGATCTTCTCTGATCTTCTTTTTCTTTCCTTTTGT TTTACTTGATATTCATATATAGGGAGAACCAGGCC
LG5	ref-72781	68.316			GAACCCAGGTCGACTGCTGTGCCTGCGTCAC
LG5	ref-62162_15	68.634			CTGAAGTCACACGATAGCTTTATGTGAGAAACAGACATGCAAGATATTTGCATCATTATTCACTCCAATGAGCCGTTCTATCAGGGAGTTGATCTTGAGAAGTGGATCAGC CAGAACAAATCTTTCTGACTGGTTTGGTGAAAGAATTTCTTCATGAACTTCTTCACTTCCCTCATGAACTCTCAAAAATGCACCAACGGACTCTATGTACGAT[A/C]GTTTTG CCATTTAGCCTTAGTGATTATTGCAACTTGTGGTGCAGTTCAGTGTATGGGACCTGAGGAAAAATTAGCTTAAAATTAAATCTTGTGGAAGAATCCCACAACAGGACAAAG CAATATTGATATGATCCAAAGGTTTTTAAAGCTTCTGGAACTCATTACTAAAATCTTATCTTTTAATATATACTAAAATGTTATTTTTTACTATAGTTGA
LG5	ref-12264	69.426			AGGTGAGTTGAATAGAGTGTTGTATGATGGTATAATGAAGATGAATTGTGATACTGTCCTTGAGGTGATTGCAGGAGTGAAGATCCGTGGAGATGATGAGTTGAGGCAGA CGTGGATGACAGAAGACACTCACACACACACACGATGGAGACGAGGAGACTAGGAGACGTTGGAGACAGGCAAGTAAATCGGTTTGAGTCCTTGAGGTAAGCATATAGG GTATCCTTGATGCAAACGAGACCGGACAGTGACTGTGAGTGTGTGAGTGTGTTTTATGGTGGCAGTGATGAGTGGCTAGATGAGGTGCAGGTGCGGATGATCAATACTCA GGAGAAGGGGTGCGCTGTGATTGGATGGTGCTGGAACCTG
LG5	ref-59636_23	69.79			CAGCAACTAGCGATCTACATGC[A/G]TATCAAATG
LG5	ref-57856	70.603			TCCTCGCTGATCTCCGAGCTGAAGCCTTCGCCGCCTGGAACGAGCTCTCGCCGCCGTCGAAGAGGCTCCCGCCGCGGATTGAGCTGAGCTCACTGGACGAACAGCGCCGGC GCCCTCGTAGACTGCCGCCTCGTGCGCCGCTCTCGAGACGCCCGCTTCCCGCTTCCGGCCGTCCCTCAGCACTCTCCTCGTGTTTTCTTCGCGGTTTTTCGACCTCCGCTCTC TGGACGAGTCCGGCATTGATGCACCTGCCTTTCGTGACCTCCGCAGCGCCACCGACTTAGCCCTGCGTGCCATAAAAGCCACAGCCCAGGCCATTGGACGATCCATGGCCA GCCTGGTTGTGCTTGAGCGCCATTTGTGGCTCAA
LG5	ref-9893	71.527			GTGTTCAAAACGAACTCCTTGCCTTAGCCCG
LG5	ref-45444_9	71.919	Chr19	37363342	GTACTGATTCATTAAGGAATTTGTCTTAGTAGTGTAGAAGTGCATGAGCACATGGCTTGTACATGCAGGTATGCCTGTGTTGTATGTTGGGGGAGGGCTCTTTCTCTGCTCA AGTAAAATTTGCAGTCTTCCTGAAGTCAAGCTTTGGCAGCCTCCCTTTCCATTTAATTAGAGAGA[A/C]AGCATCTGCATCGGGGTTCTGACCTCAGAAAGGGAGGTGTAGT GGGAGGCAGAGAAGAAGGGAGTGATACAAAGAGAGACAGTCAAGAGAGGCAGACTGATAGACTGTCAGAACAGAGATTATATGGAACTGGATTTTCTTAGTTATAGGCC TACTGGTCACTTCATGATAAACTATTGCAGAGTCATAGAG
LG5	ref-6081	72.228			GGGACCATCTCGAGATGGGTGCCATGACCAA
LG5	ref-14262_7	72.678	Chr19	37430399	TGGTTTCAGCAGACTGCATTTGCAAATTATGCTGACGTTTTAGCATATTATTGATCTTAATATTGTCGTGACTGACTTGTTGAAATGTCGAGAATAATTATGTACTATTGCAT TCTGACTGAGAGAAATGTTTGTGGCAGAAGATCGATTATTTTAATTTTGCTTCTTTACCCATCGTGTTCCGTCTGGCAGTGAGTGATGTTTGAG[A/G]GTCGACAAGCTTGCG GCTTCTGCTGCGTCTCATTGTCCTCATCTACTTACTGAAGGGTGAGATGACCCTGACAAAACAAGCGATTGGGATTCAAGATAGAGGAGTATTATCTTTCCCCTGCAATCAG ATTTTAAAGGCTTGCAGTCAATGAGGCCCATTCATCATAAGGCACCATAGCCTGACAGCTGGCAGTGCCCCCCCTGTGATGAACCATGACCCCATGA
LG5	ref-7100_7	72.946			CTGTTTGTGTGCTTCAGATGTGTGCGCTCAGAAGACGATAAATTAGAAGTTTAAACTGATATAGCTTTAAAGCACATGAAAATAATAAACTTTCATGATGAAGAAGAACTG CACTGTCCATGAGTGTGATCACGATATAGATGATAATCAAAACCAAGCAGATGTTTTGTTAGTA[A/G]TTGGCACAGTAATCGATGCAGGAGCTGTGATTGTGGAAAGGGG GCGGGGAGCAGCAGCTCGGTTGCATTTAAAGGCACAGGCACGAAATGGTATGTTTTTCCGTGCACTCAGTCTTTACAACATGGTATAATAAAAGGTCTTTTAAGCTGAAAC TTCACACACACATTCTGGGACAGCTGGGACTTAAATTACA
LG5	ref-11908_6	73.255			CTACTGGCAACCACCCATCCACAACACCTTAATGATCTCATAGGTATGCCTAGACAACACTAAAATCATGACAGTAAATTCTGCATAGACCAACACTTCACAACACAATCT TTCTTTCTGTATATTTGAAAAAAGAAAAAATGTTTTGCAGAAGTCACGTCTTTTTTTTCATGT[C/T]CTTTGCAGCACAGTCGAGGGGTATTGATTTGCACATTACAGGCCGC GGGAAGAACTCAACGCAAGCTTATGTAACAATGAAATACTGTATGAATATGTATTTCTCACAGTATCAATGACTTAACCCTGTAAAGCCTGACATAGGAAATAGGCCTAA

					ATTAAGAAAAGTCTATGTTTAAAATCTCTATTTTTGAAA
LG5	ref-64621	73.522	Chr19	19887805	CCTGAGCGACTCACCTGGACAAAAACGATGAGAACAAGATAAACGCAGCCCGAGGTTCCTCTAAATGAAAGGTGTGTTGAAACACCAGAGACGCTGAATCCTCAGAAGAAATCACGGTGCATGAAAATCATCTTTCACTCCCACGTATCCGAAGACTTCACTCACTGAGACGCAGTTTTGCACCTAATTCACCTGAAAGGATATATTTTGTCTCTGAGAAAGCGTAACATCATCCTTTGCCCAACATCACCTGAAGAAGGTTCTTAATATACCACGCAAAATCTGAACTTACGTGAAAGACTTTATAGTGCTGTCGCAGTTTACAAGTGTACCTGAGATTCCACCTCCGAAACTACAATGAGTGCACCT
LG5	ref-11516	73.745			TCCTTTGTTACGACAGGACTGCTGTGTGTGT
LG5	ref-36416	73.873	Chr19	21763557	TTTGTGTTGGTGCTCCATTCTGACTGATGTGCTCGACATGTCATGCTGCAGGTTTGAGTTTATGGATATGAAGTGTTAATACATCCCCCGCTGGATAAGTCATTTTGACTCATTCTTGCATGCACACGCGTGTCGGAAAGTCTATGTGCCTGTAAGAGAGAGAGAGTGAGAAAGAGAGAGAGCGATTGACTCGGCACACCGCCTGCCCTCATACAACAATTACCGCAGTCAAGGCCCACTGCAAAGATAAATTAATGCCTCCTCACAAGACAAAAGATTTTCTGTTCACTAAAATACAGAAACTATCATGTACCAGTACGTGTTTAGAGCATGTTTTACATTTAAAACGATTCTTGGAATTAAAGTAAACA
LG5	ref-49269	73.982			CGTCAGCTGTTTTCAAATCGACTTTGATATTTTTATTATGTTTTTCCAATAATTGACACAAAAACACTATGCTCTCATTGTGTCATGGTGACATCATCTGGTCAACATGTGAACTCACCGAGGTAACGTTACTGGGACATTTAACTTGGGATCCCAGGTCCAACCTCGTCTGGAACGCAGCATAAGTCTTGCGTCGGAGACTGTACACCGCAAGTTTTTAAATACTTAGCTTAAAGGTGTAATATGAATAAATAGTGCTCATAAACGGCTGCTGAGATGGTATTCTGAAGTTAAACGAGATGCCGCATTTTGCGCCAGCCCCTGCTGTAAACGAGTGGAGGCTTGGACCCTTAAGTCGTGACTTAAGCG
LG5	ref-73099	74.024	Chr19	12678055	CCTTTTATTGTCTGTGCGCAAAAGCAGAACATTTAAGGTACAGCATAATTTGTTTTACAGTGAAGTTTATAGAAGGAAAAACATTTTTTTTTATGCAGACATAGACATTTGATTAAAAAAAAATCCTGCGTGTGGTTTTCAAAACAGTCACGGCAGAACGCAGACCTATCTAAAGCAGCTTCGCTGTTTAGTTTTTACCCAGGAATGTAAACGATTTGTCTGCATAACACACACACAGCATCTGGCCTCCCTTCCGCTGCCTTTTTTGTGTTGTTGTTGTAAAATGGTGCATTGGTGCTCCGGGCAAGAGAAGACTGCCGAGCAGACAGCAGACGCAACAGAATCAGATCAGACGTGATGTTATCAGACTGTGTGTCTCACACCCATCCCCTCTCATCAGGCATGCATGCACTGGGTCCTGCTCTCAGACAT
LG5	ref-36035_25	74.131			GAGCTTCATAAGCATGCCGCTGACGCAAAAGCTGTCACACTTAAAAATGTAATTGAATTAGTTAACTATAAAATATCAAAGCAAGTAGCTTTTATTGTAAAGAATGATAGCACAAGCACACTTTACGAGAACAACAATAGTTTTTTTTTTTTTTTTTGGTTTGAAATGTTAGAGGACCATGTGCATAGTTAATGTGCTGAACTACACCAGCGATTACTTTGCTA[A/G]GACATGTTTGTAAACCAAGAAGGTGTAAAATAGCTGTTAGCATTCCTATCCATCAAAAGGGAGCTGTTCGGCTAGTGCATGCAGCCTTGCAAGTATGTGACCTGCAAGATGTTACCATTAACGATGGTCACTTAGTACTGATGCATTTTTTTTATTTTTTTACCTTAGTTTTAAATTCCATGTGACCAACTACACCAGAGAAAGGTG
LG5	ref-71707_9	74.559			GAGATGAC[A/G]ACGAAGTGAGTGCAGGTGTGGAA
LG5	ref-32637_2	75.078			GAGTGGCCTTGATAGCCACCAGAGATTCCACCCCAGACTGTTGTCACTCCATCAAAGACTACATTACCCATAATCCTTTGCCCAGAACTCATCAGCACTCATTGTTTTCAGCTGTGTCTCATTAGCCTGTTTAGCTCACCCTATATAAGCCTGGTTTACTGTCATAACTG[C/T]ATTTCTGAGCATTCTCCTCGTTTCATGTGTCTTGTTTTTTTTTGTTGTTGTTGTTTATTTATCTTTTGCCTGTTATTCTTGGGTTTCTCTGTCTCTTTATTTTGCCTGTACCCCTTTGACCATGTTTTATGTTTGGATTGTTTTGACCTAAATACTTCATTTGGATCCTCAACCTAGTGATGGGAGAAAGCTTTCCACA
LG5	ref-25131_24	75.313			CCTTGAAAGTTTGTTTCTCTGGTGGAACTTTTTGATCTACATACTTTCATTATATGTCCTTGTTTCCCACATTTGTGACATTTCTCTGAAACAAATCTGCACTCATTGGCCAAATGTTGCAAATTCCCACATCTGTAACATGCTCTACTTTGACTATCTTCTTTCTTTACTGACGTTTTATGCACTCTTATTGAAATGAATGCTTCTGAGCGATGAGCTTGCA[A/T]ATCCTTCACATCCTTGTTGGCTGTTTCCAATGCTTGAGCTATTTTCAACGCCTTTTCAAACGTTAAATCTGCTTCTGCCAACAGTCTATGTTGAATATGGTCATCATTTATGCCACATACCAGTCTATCCCGAAGCATCTCTGTTAATTTTTCTCCATAATTACAATCATGAGCCAATTTCCTCAATACTGCCACATACTCAAGAACA
LG5	ref-32480	75.344			TAACAATGGGCGACACTGTTGCAAGACCTAT
LG5	ref-51541	75.405			AGCTTGGCAACGACGTCACTGCGCAGTGCAT
LG5	ref-64924_25	75.407			AAGTATGAATGCAATCCGGACATACTACATTCACCATGTTGTCTTTATCATGTGACCTACCAGTGTCATTTGCGTCACTTGCGTCACACGGGATAGTAAAGTGCCCATCATATGCACACTTAAGAATCTCGCTGGAAGTTGTAGGTCATCCTGGTACTTTTTACCTACTGTAAATTTGGGCATACTACTCGTC[A/G]CATAGCCTACTGTTTTTCACCTACTATATAGCAGGGAAGTATGCAATTTTGTCCTCAAAGGTGATGTGTTAGAAGCAGGAAAAATGGGCAAGCGTAAGGATTTGAGCGAGTTTGACAAGGGCCAAATTGTGATGGCTAGACGACTGGGTCAGAGCATCTCCAAAACTGCAGCTCTT

LG5	ref-52279_3	75.579	Chr11	36483127	TGAAGGTTATTGGACACTGTGAAATTTGTGTTCTTTAAGTTAAAAATTATTACTTTTTTCATTAGAGTAATAATAAAGAAGCTGTCCTACATTCATTAGTCTCACTGTGNNN<br>NNNNNNNNNNNNNNNNNNNNNNNNNNNNNNNNNNNAGAGAGAGAGAGAGAAATTAATAA[C/T]TGTATAGGCATCGATATCGGTTCGGCGAGTACTAGAAAAAAGTAT<br>CGGTACTCGTACTCGGTCCTTAAAAAATGGTATCGGTGCATCCCTACCTTTTACATTCACAAACAGCTATATTACACACTACATGAAAGGTAATATCTAAAAAAACACTTTA<br>AGTTATTCAGTTCATGATCATGTTTTTTAATTGGTGTACAA
LG5	ref-52279_6	75.579			CTCGC[C/G]GAACCGATATCGATGCCTATACATTT
LG5	ref-72582_4	75.642			CTTTCTTGTTCTTCCGAGCTTTAACTGGAGATTCCGGGGTATATCTCCAGCCATTTAATGTTTCAAGTTCACCACTAAAGTATCGACGCCATTAACCCTGTTCAACGCGTTTT<br>TGTTTATAACGCTAGATGGCGCAATTTTTAACTTGGGATTTCGTTAAGAGAGACCGCGGATACAAAAACATAAATGTTGCGTCAGTGAGT[A/G]GGATGTCGATTGGTTTGC<br>ATACAGTTGGTTGAACGGTTGGTCATTATTCATAAAATATTGCTATAAAATAATGCTGTATTCAGTTAATACAGTGTACCATGAAATAAATAATATTATTACAACCTTTTCT<br>CAGTTTTTATTATGCACTCAATATTGTGCCCTTGCAATGATATTTGAAGGACATTCACAGAAATATGCCTGACATTTGTTCAATGGAAGCCAAACTGT
LG5	ref-64928	75.785			CTGGAAAATGAACAATGGTCTACTCAGAAAAGAAAATCTCAAGCTGCTCAAGAAAAAAAAAAATAATAATGAAGCAGAGCTCATTTAAACTAAAATATCACCAACAGGTC<br>TACTTCAACGGCGTAAATCTGACTTCTACTAAAACTTATGGACTTCAGCACCATGGACCGAGACATATGCTCTCTCACACACATACTTCAGCACCATGGACAGAGACACAT<br>GCTCCCTCGCTCATGCACAAACAAACTTTAGCACCATGGACAGAGACACATACACTTGCTCACACATACTTCAGCACCATGGACAAGGACTCTCTCTCTCTCTCTCTCTCTC<br>TCTCTCTCNNNNNNNNNNNNNNNNNNNNNNNNNNNNNNNNNNNNNNNNNNNNNNNNNNNNNNNNNNNNNNNNNNNNNNNNNNNNNNNNNNNNNNN
LG5	ref-45635	75.806	Chr19	4713513	TGTCGCCTCTCACCAGCTCCAGTCAGGGTTAGGAAAGGAGATCTCCCTCTCCAAATTAAACATGGCTCCCGTAATGTAAGATCTCCAATAAAAGATGCCGAGCTAAGCAGC<br>TTCCACAGGATTTGGGCTGAAATCAGTTAATTACTTTGTGATATGGAAAATCTTAAGCCTGTGTAATTGGAGGCCCCCGCAGCAGACAGAAAGAGAGAACGATAGAGCCG<br>CAGGTTAGGCTTTTTACAGCGTGGCACAATGCCGGCACACAGAGGCATATTGTCCACGTCCGTCTTTGTTCCAGACAAACACACACACACACACACACACACAGGCTCTGT<br>TCCAAACTCTATATGGCTATGTAGAGGGCAGCTGTCTTCAAAGGCAGCATCCTAACTGAAGTGCAACCTCATAAAAGTGACCAATTTGGAACGCTCTAA
LG5	ref-68238	76.385			GTAAGACTGCATAATTTTGTCTCTTGTTCACAGTCCATTTGGTAGTTTGCAAATAGCGATGACGTTTTGTGGCGGAGACAGTTCTTTTTGAATCGTTAATAGTGGTAGAAGT<br>AACTCCTCTAGTGCAGTAGGTGGCAGTAATCGGCTCTCAGATGTTTTTATCTGCAGTAAAGCAGCGAGCAACGATTTCGAGTCAAAAAAAAAAAAAAAAAAAAAAAAAACA<br>CTTTACCGGTAGAGTTAGAATTTGTGCTGAAGTTTGACCAAGCGCCTATCTGCTGTTTTATTTACAATTTGTTTGAGATATTTTCTTGGTGGCGTCAAGAAATAGTTTTACTA<br>GAAGATGTTCGTGGCGAAGAGCATCGCAGCGGAT
LG5	ref-52136	76.426			CCAGATGAACCGAAAAATGTGCATGCTGAAT
LG5	ref-21749_9	76.807			AAACCCTG[C/T]GCGAACAGGGTGCTCGAGGGTAT
LG5	ref-18265	76.954			GTATCATATAACAAAGACTTACAAAGTATATTGGATAAACAAATGGACAGATTATCATAAGGCCTCCTTTCTACTTTTTACCATAATTGTAAATGTTTTTACCCTTTGCAAG<br>GTGCTTGAAAAGGGTAAAAACAAATACAAAAGGTGTGCCAGATAAGGCAAAGTGGATACCTTTTTTTCCACACTTTTCTGAAAACAACAGATGTTTCCCGAATGAGATGCT<br>CATACTTTTTCAGGCTCCTCATTATGTTCTGATCTTAGGCTTACACAGCAAAAACAGAAGACAGTAGAGAAATGAGAGGGAGTTTACATCATGTAACCTTAAAATTCCTGC<br>AAGCTGAGAGCCTATTTATTCTTACAGTTAAATGTCATTTCCAAGTCTTGCTGTAAAACAGTTCAAATGAAAACAGAGCAACAAATCAATATATATT
LG5	ref-26447_30	77.178	Chr19	8203112	TGAAAGGCTGCATGAGGTCTAGTGGGGTCAGTTATGTGTGTGTGGCTAGATAAAGTCTGAAAGAAGTTTATCTCTCTTATCATCTGGTACGGTGATCATAAAACCATGAAG<br>GATACTGTGTGAATATGATGCAGCCTTAGAATTTGACCAAAAGTGCAGTTCCCTTAAAAACGATATAGGCAGCTCACTCGGTTTTGG[A/G]AAAAAGCCATGATGTCCACC<br>TCCATTTATATCTCTTTTTCAGCCTGGTAGACAGCTGTGTTTGAGAAAGCAGCAGGAGCTATCAACTGTCCAGGCCTAAGGAAGCATGTCAGAGAAGTAAGATGCTACGCA<br>TAAGCCAAAGTCACATTTTTTAACCCGATTTCAGTGCAAA
LG5	ref-21749_5	77.45	Chr7	49975653	ATCTCTTCACTTCCCAATAGCAATCACTAATTGAAGTGGTCTAAATGTATTTATATGTATTTATATCGTCTTTGGAAGGTGTAGGACCATAAAATGTTCATGCAATCATATC<br>ACTTGCTCCGAGGGATACGATAGGATGTCCTACCTGCCTGGTAACATATGTATTTCCATAC[C/G]CTCGAGCACCCTGTTCGCACAGGGTTTTCAACATTAATAATAATAAG<br>AAATTTTTTTTGAGCAGATAATCTGCTTATTTGAATGATTTGAAATGATTGAAAATTTGAGTAATGGCTGCTGAAAATTCAGCTTTAACATCACAGGAATAAATTACATTTT<br>AAAATATATTAAAATACAAAACTGTAAATTGTAATGT
LG5	ref-57354_16	77.906			GTAGTTTATTAAAAGAAGCTATAAAGAAGAGTTGCAAATCATTTTCATTTTAAAACTACAGCATGGTTTTTAAATGCATCTAACAAAACTACTGCCATGTTAAAGGAATGA<br>TTTACAGTACTCATTATACTGGTAGATTAATATTTCTACCATAAACCCTTGCCTATTTACCCACCTCTGCACAC[C/T]CTTCGCTCTTTCTTTCTACTCTGCACAACATGCTCTC<br>TTAAACTCAATTGACCTTCATATTTTCCCGCAAGCAAATCTCTGACAAGTGTCTGACATGACCTTACCTCTCTTCACTTCATTCTTTTCATAACACATGTAGCAAATCCTTCC<br>ATGCCCTGTTTCCCATCAGTTAAAATGAGGTACA
LG5	ref-59705	77.968			AGCGAGGGGGCGATGATACTGCAATGACGAT
LG5	ref-13490	78.467			TACAGTATCTCGATTGTGATGCTTCTGCCAC

LG5	ref-68461_32	78.676	Chr19	26358476	GTTATAACACTTACCAGCAGTTAATTATCTTTTCAGTTTTGACCACTTCACCTTTGCTTGTGGGATTTGCTATATACAGAGCTAAGTTCCAGCCAATTACACACATGAAGAGGAAANNNNNNNNNNNATATATATATATATATATATATATATGTTTGTGTGTGTGTGTGTCTGGTGTGTCACAGTCACAACCCATCTGTTTTTTACACAGCGAACACTGTGCCTAAAACAC[C/T]GGTCATAAATCAGAAGTCTAAAACGATCACTTTTAAAGAGAAATGTCAGAGGATTTCGATATAAAAGAAGAGGTGCTTGAGTTTGTTGCACAGCCCTATTTGTTTGAACTGCGAGAGGCGTTTAAGCTCACAATACTTCTACATCCTACGTCATAAATCGCGTCAGAGGGTTACTCATTTGGCGGGTTACTCATTTTCTC
LG5	ref-44296_4	78.9			CAG[C/G]ATCACACGATTGTGCTGCTCACATGAAC
LG5	ref-57505	79.062			TATGTGAAACCGATGATGGTGCCTCTTTTAA
LG5	ref-12493_17	79.291	Chr19	25321094	GGATGGTTTTAATTATTCAGGACCCAGCAACAGTTCTCAATCTGGGTCTTCAGATGGAGGAGTTGATCTTTGAGCTTGCAGACACACATCTCTTCTTCAATGACCTGGAGGTAAGTGTAGTGTAACACAAGGGTCAGTTTGCTGTCTGTTAGGTTCATGTAAATAACATTTACCTCTGCTGTCAAAACAGGAATGTGATCAAGTCCATGTCGATGA[C/T]GTTGCCTCAGACGACAATGGGCAGGATTTGAGGTAGTGAAACAGTCAAATTTTGTGATGAGCTTTCCAGCAATTTCTTAGACAAGCAAGTTGCTTTTCAATTGTTTTGCTTATATCCTCAAAACACACACATATTACTGTTATATTTATGACAAAAGTTATGTGTAAAAGTTTTTTTTTTGTAATAGTTTTTTGTTTTGTCTATATTAACACATC
LG5	ref-34806_4	80.206	Chr19	28216678	TCATCAGTACTCAAAACCACAACTTCTCAATGACAAATCATCACTAAAGGATGTTTCATCCAAAACATGTTGCTTACGATGAGAATTGGAAGTGTGCACAAAGGATTTGAGGTGCTGAGGTGTCGTCAGTCATCTATGCAAAACCTCAGAAGCTAAAGATGAATGTTTCCTC[A/G]TGCCAGGCAGATTGTTCGACTTCTCCATTTTACGGAGTACGCTGAAGCCTGCGGCTGGGAAGCAATTTATATAACTTCATTAAAATGTCCTTTCTCATCCTTCTGCCATCATGTCTATTTTTGAATGGTGTCACACAAAGTGTAACCGCATTGATTATAGTTTATGGCTGTACTTGTTTCTTTAGAAAACAGAGCAA
LG5	ref-34806_7	80.206			ATGGAG[A/C]AGTCGAACAATCTGCCTGGCATGAG
LG5	ref-27540	80.716			CTCCTTTGCACGAAGTTGTTGCCGCACGCTT
LG5	ref-30530	80.784			GCACTTTAACCGATGCATGTGCTGTTTAAAT
LG5	ref-52655_25	80.977			CTAGCATTCCAATTCATCTCAATGGGATGAATGTCAAAGGGCCCTGGAAGTATGTGATTTGAAATCTGTCTTTGCTCATGGGACCAAAGAACTTGTCAATCAAAATAAACCCATGAAAACTATTGCCTAGTATAGCCTAAAGTCACAGAACAGGGTTACCGAGTGCTGAGGCGCATAGTGCATAAAAGTCGTC[C/T]GAGTTCTGAACTTCCAGTGGCATTAATATCAGCTCAAAAACTGTGCAGCGGGAGCTTCAAGGAATGGATTTCCATGGCTGAGCAGCTGCATGCAAGCCTCACATCACCAAGTACAATGCCAAGCGTCGGACGGAGTTGTGTAAAGCACACGGCTACTGGACTCTGGAGCAGTGGAA
LG5	ref-52655_7	81.265			AGAACT[C/T]TGACGACTTTTATGCACTATGCGCC
LG5	ref-4977	81.366			TACTGTGTTACGATTGTGTTGCTGTAGAGAC
LG5	ref-72196	81.741			CACATGCCCACGACATTCATGCTCCACATTC
LG5	ref-68583_27	82.289	Chr19	27034665	GAAGGAGATGCACGGAAATAATTATAGGCTAATTTTATAGTTTACTGCGACAATCATGAGTGCAGTATTGAGTTATCTATTTGTCAATTTGTACATGCCACAGGTGGTGACACTGCTGAATGATCTCTACACTTGCTTTGATGCAATCATCGACAATTTTGATGTGTACAAGGTAATTCCAGACAGTCATCACATTCAATGTCTGTTCAACGACATGAGTGCTCGG[A/G]TTAGTTTAAGTGGCTGAGATGCATCTCACAATCAGTGAGCCGACTCTTGTGAAAAAGTAGTATACTTTTGTATACTTTTAAAAAGAGTACTCATTACAGAAATAATATACTTTAAATGAATATACTTCAAGTCAGTGTAATTAAATTAAAGACATGTTATAAGTACATGTTGTTTATTAATAAAGTCAGTGTAATTAAAGTTGCG
LG5	ref-29687_30	82.34	Chr19	7765709	AGTTCCCCCAATGGCACCTGTTAGTACCGGTTACAGTGAGGAGGTGAGTAAGTTGAAAGATGGGATACTTAACATTTTTAAGGCAACAAAAGTACCAGCACAGAATTTCATGGAATTTGAAGAATGGATACGAAGTCTCTGGAAGGCTGTCCAGTTTGAAAACTTTATTTTCAGTTTTCGCAACAGCCTCGTCGCTGA[A/G]GCCTACTCCAAACTTTGTGTTGAGTTTAACACTTGGGAATGGGCCTTCAAGAAGTACATGATTACCTGGTACACAAAATCTGAAACCAAAATCAATAACTTGAGTGCAATGTCAGTGCGGTCAAGAAACACACTTAATGTTGACACAGTAGTGGCTGATTTAAAACATGAA
LG5	ref-11539_10	83.447	Chr19	42718391	GAGCAATGCATTGCGCAAAATTTTTAGGAAAAAGAGAAGAAAGAGCTTTAGTCACTTGTTAAATCAGACAGTGTAATCCTGCTCATTTTAAAGTAACACTTGAGACTTGGTTCGGAAATTCCCAGGGAAAAGGACACAAAAGTGCATCCTTATGGAGTTGTATGACAGCGCAATCCAGC[C/T]CAAACTCTTCGGCATGACAGACGGCCCCAGGGGGTCATAACAATCACGAGGGCAAAGTAATCCCACCGTGTTCCCTGTGCAGCTGTTTTCTGTCGACGCACACTCACAGCTCTGACTTCCTTCTCTTGTTCTGTTCATTGACTACCAGCAGAAACAAGCAGGAATTTCTTCTTTTGTCTTGAGGAAGTGA
LG5	ref-27647_16	83.922			TGGGTGGTTGCGAGG[A/T]CATTGCTAGGGAGATG
LG5	ref-23149	84.451			GGAAACAGGTAAGTGATGTTTATTGAACAACCAGATGAACATGCAGGTGAGTAAATGGCAATGTGGCAGGTTGAATGGAAGATAACTGAATGGTAATACTGTCCTTTGTGTTGTACGAATGGAGATCGTGGATGGTAGAGGAATGGAGATCGTAGCAGAGATGAGAAGACACTTGGGAACTCACACGAAGACTGGAACACGGAAGGAAACGAGGAGCACGCTGGAGACGAGACAGCAAGTATACTTAAGGTAAGCATAGAAGACGTTCTATGTGCAAACAAGACCGGACGCTGACTGGAGTGTGTGTGAGTCCTTTATGTCATGACTG

					ATTGTGGTGCTGATTGAAGACAGGTGCGTGTGCTCAGTACTCTGGTGAGGGAGTGCGCTGTGATTGGGTGATGGTGGAGCCTGGTGTGTCTGTGACAGTTAT
LG5	ref-53027_25	84.543			CTGATGAACGATAAAAACACTGACAGCCAATCAGAATCAATCCTGCTATAACAAGCTTGAGCATTTAAAGCAGCAGACAAGCACGCTCTTAGAATAAATAGAACAATATC GTCTGCTGGTGTGGACGCAAATATAGTTATCGTTATAATTATCTTTACAGTTATCGTTGTTGGTGTGAACGAGCCTTAAGATCGTCCTGGAGCCCAAGCCCGACAAAAATGC AA[C/T]AGCGTCCATGCCAGTGCCGTGAATATATTGGTGGAATGAAGGAGTGGAATGAAGCCCTGTCCCTTCGATTTCAGCAGATGTAAGTGCGCTGAACTCTTAATATTGT TGGAGTATGAAGAGAGCACTATTCTTGTTTTGTTGCCCTGCATCAAGCCTGTTTTGTTGCCCAGCACTGAATTATTGATGTCTGTGATGTTCCCGCCCTGC
LG5	ref-59352	85.955			GATAAATCTTTCCTTAAATAAATAGATAAATAAATAAATAATGCAATTAATCACAGCTCACTTTTAACTTCGTCATTTTCTATTTTCAGTAGGATGCCATTTTTTAAAGAAT GTCTATTCTTTGCCAATAATTTGATTTCATCCTAACAAATCTTTGTTTATTTTTATTAGTAAAACATAATTTGATACTAAATATATCAGTTTGAGTCACGACCTGCATGTTTTG CTTCAGATAGAGAACATTTTAAGACACAGCAACTTTACTCCTCCATCTTTTTAAGTGAGTCTTTAAAATTATTATTATAATTATAATTATATAATTTTATAATTATTAAAATC ATTTTACGTAAAAGAAAAAAACTTTTGTATGTATGTTTCTATGTTCTGTTTCTCATACATGAGGGGTCTTCAAAGTTATTCAGGCCAAGGAT
LG5	ref-73084	86.611			ACGACGGATAGAACTATTGCCCTTGTTCAAGGGCACCTCAGTCATTTCCTGTCAGTATTGAGAATCAAACCCACAACCTTCGGGTTACCAGTCTGACTCTCTAACCATTAGG CTACGACCGTGCAGATAAACCTCACTCCAATGGCTTCAAAAGGCAAACTAGCGACTGATGTGAGATGCCACAGTGTTTAGCATCCTTGTTAGTGTGTCCGCCTCCCGTGCC AGTGACCCTGGTTCGAATCCTGTTTGGAGCAGGTGTGAGTAGGACCAGATACACAGGGGAACCAAAAATAGTGATTTAGCCCTGTCACACTTCCCTGCAACTTCTTACTGG AAAAGCTGCACTATAACATCTGAGCTATTGTCATTCAAAAAAAAATGCAGATAGGTTAGCAGCAGTGTCACTATATACGTTTAGTTAGTTACTCCTC
LG5	ref-70517_27	89.961			TTCAACTCATCGACAAGCGTGCATTG[A/G]CTCCC
LG5	ref-45588	91.358			TGTAAGGCTTCGATGCAGCTGCTCGGCCATG
LG5	ref-71442	91.916			ATTCGTATCTCGATAAGCGTGCTGTTGATTA
LG5	ref-56866	94.325			GGGAGTCACACGAGTACTCTGCCTGTGCTGG
LG5	ref-31622	94.741			TTGTGACCTTTCCTGCAAATATATACTTCACACTTTGGTACAAAAAGTGACAAAAATAGATTTTTAATGTTATAATAGTGATTTGATCATCTCTAAAAATAAATTCCAAAGC AAAACGTGTGGAAAAAATATATTAAGGAGTGGGATAAAGGGTGAAAAAATAAACATCTTTTTGGTTTGCAATACCTTCGCTTTTGGTGAATTGACCCCACTGCTTTGGGAT CAGCAAGATTTAATATTTGTTTGTTTTTTTTTTCCAATTTTAAAACAAGTGTTTGTTTTACACCAAATAATAGTTTTCCTACACTGTGAAACTATTAGTTTTACAAATAGGGA CAGTCTTTGAAAGATAAAAAAATAAACATCTTT
LG5	ref-60455_16	99.208			TATTATTGTTAATAAACTTTGAAATATGGCCTCTCCAGCTCTGCCATCTGTGGTATGACTTTTTCAGCTTGGTTATGAAGAGAAGACGCCACAGAGATTTGCTGACTTTACA TGTTTATAGACTGCTGACGGCAGCAGCTCTTTCCCCCCACTTTGTTTTCACTCTCTTTCACTTTATAAATTTACTACAATCTTATTTGAAAATGGACACGATT[A/C]AGCTGCA TTTAGGAAGGTGGCCACCTCTACTGCCCAGCAATGTTTGTTTATCTGAATGTTCATCCATCACAGAAAATAGCGATTAGCCCTGAAACAACCCCAGTGATGTCTTTAAGACT GTAGGGAGAAAAAGCAAGAGTACTTGAAATAAGTTTTTCATGTTTTTTGGATGAAATTCCCAGTTTACCATTCAGTGAAGTCACTGCTGACCTGAGT
LG5	ref-15973_2	99.86	Chr19	698571	ACTATTGCTTCAAGTTACAAGTTTTCTTAAATGTAATGTTTAAATATGCAAATGACGCATTCATTAAACATGAAGTAATTTGCATACATTTCCAGAACGATAACGGCACAGA GAAACAATATCATTGGAATCACTTTCAGAGCGATTTTACCAGCTGTAAACTTTGACAGCCAATTAGCTTTAAAGATCACGAGTATTTAA[A/T]GAGGCAGACGACAAAACT GCAGTGCATGATTACAATAAACAGAATGATATCATGCATTGATGTGGACGCTGATATAGTGATGGTTCTTGGTGTGAACAGGCCTTAAATGTGATTCTCACCCTCAGGAAT CGCCTCCATAGTGGTGTCCTTCATGGTGGGGATGTACTACAGCACCATCATCGCCTGGGTCATGTGGTACTTCTTCAACTCCTTTCAGGACCCTTTACCCT
LG6	ref-47037	0			ACATCTGTCTCGAATATTATGCAGCAAGCCA
LG6	ref-65552	1.592			CCCGACCACACGCCTCAACCGTTTAGTTTTGACCTCCTTTGGGAGGGTTCAAATCCCCCACACACCACCCTTATACACCCCTACAAACTCATAACCTGTATTTTTGACTCCG AGTTCATATATCATACGCACCTGCCTGCACAGTGTTCAGTAATCTGACAGGTTGGAGAAGAAACCCCGCAGATTCACCGTGAACTGAAGGGCAACCCCTGACATGAATCCC ACCCTCCAAAATAGTAGCATGCACATGCATCCCAACACCTCCTATTGGATTGCATCCATTATCAATCTCACCCCTTTAATGAGCTGTTCACAAAGACCTGATGAATTATTAA AATCATACCTTGACACGCTGGTCGGTTTGCTTTT
LG6	ref-71678	2.517			ACGTTTACATGCACCGTCATAATGCAGTTATAATGAGATTCAGGCAATATTGCAATTAAACTTTAACTCATGTAAACACAATACTTAGATTAAATCAATGTGATGAAGCTC ATAAACGTAGCAACCATAATCGTAGTAAACCATGTGATGTACGGTGACTTAATCACATTAAACAGGCATGTAAACACTTTAATGGCATATATTCCACTCCGAACAAAGTGC ACATGTGCATATGTTTTGGCTGCTGAAGAAACAATGACATGAAGTTCATGTTGACAGTGTTGCATGAATATAATGTAGTACGCTGATGGTCAAAACATTGAAATGGAGACT GAATTGAAATGGTTTCCCCATGTCATGTAAACATCTTACTGTGATTAAGTCCTTACTCTGATTATTGGAAATAATCTCATTATTGGAGCACAAGTAAA
LG6	ref-50221	4.751			TCCTGTGTCAGCATCACACGCTTGCGTCTAATTGCACTGGATATGCCTCATCACCGGCTGCAGTCATGACCTCATCTCATAAATCAAACACCTGTGATTCAGCAACTACCTG CTAATGCACTTGCATTCATGTAAAGTAGCCTTGCTGACAAGTTTGGGTGAGTAAAGGCAAAACACACCAGATATAGTACCTTCAATGTCCTTAGATGGCGATATTGCTGCC ATTTTGGGGATTTCCAACTGGATTGGCCAACCCAAACTGAAATGCTTTCCATACCCACACACAGTGGTCTAAATACAAAATCTTTTTACTTTTTCCATCATCATTTTACAGG

					AAACCCTTTGAGGTTACATAAAACACTGCTGAAAGGGATACAAATTATATATTGTCTGTGCTGTAATCAGCAGTTTTGGTTGTTTCCTCTAGATTT
LG6	ref-6821_8	4.848			CGTACGAGAGCGACCGCAGCTCCGCCAGAGCGTCAGCTTCAGCGGCTTCTCCTCTCAACCTCAAACCCTCGGTGGTTTCCACGCCGTACCTGATGCACTGGCCTTCTCCAGATCGTCGTCAGTGTCCCCGCCGCCGTCCACAGGTAGTCCCGACCTGCTGTCTCCTCTGTTCCCCGAGCCCGGGACCCTAAAGCACAGCCACCCGTT[C/T]GCCGATCTGGGTGCCAACGGGAGCTTCTACGCCATCAGCGACTCGGAGAGCGTGCAAAACCTGGCATACGCGCTCAAGGGCCTGTCGGCTCTGCAGCGGAGCGCGTCTGCAGACTCTCTTTCCGACCAGGACGACTACACCAGCTCCAGCAGCCTGAGCGGCTGCGACTCTCCCGGCATCGAGGGCAGGCGTCTGCCCATCTTCAGCCGCCTGTCTGTCTCAGA
LG6	ref-54967	5.66	Chr20	45439254	AATTTTCTCATAATATCCACTGCAGCATCAGCTCTTTTCTCTCAGTGTCTGAAACGGTTCGATCAAGGATTCGGTCTCTCTAAACCCCGCCTTTCTGAGAGCTGCTCTGCTCTGATTGGTCCAGTCTGCTGTGATTAGTCGGAAACCAAACACTCATTATCATATCTGAATACTCCTCAGCACTTGATTCGCAGTGATACGAACAGTAACGATGGCATTGGATTCAATCCGATCTCATCAAAGTGGATTTGCTTTGGCAGCAGAAAACAGCGTCTTCTAGACATGAACAACACGAACCAAACTCTTCCAGTCTCAGATACAACTACAGTGATTGAGGGCGGGGCAAAGAGACGCTGTTCAGCCAATGAA
LG6	ref-3929	6.163			GATTTTGGATCGATTCGGGTGCAGTGCGCTT
LG6	ref-67990_3	7.218			CA[C/G]ACACACACGACAATTTTGCCTCCGGTGAT
LG6	ref-21252	7.415	Chr18	49238492	CGATGCAGAAGACGCCAACACCCCAATGCGGGAGCTTCCCGGCCTCTGTGGAAACTACTGTGGCGATTACTGGCTCCATTGCCGTTACACTCTCAGCCTTCTGATCAATAACAATAAAACGTATGCTATTGAAGAAGACCAAAGCAAGTTCTGCAAATACCTTGAGCTGAAAGATCCCGAGTACTGTTACCCAAACGTCCTCTCTAATGATGAGCTCAATGCCAACCTGGGAGATGTGAAGGCGGATCCGCAAGGCTGCATACAGTTATGCCTGGAAGAAGTCGCGAACGGTCTTCGCAATCCTGTCGCGATGATACATGCGAATGACGGAACTCACCGCTTCTTTGTAGCCGAGCAGCTGGGATACGTGTGGACTTACTTGCCGAATGGTTCTAGAATTGACAGGCCCTTCCTGAATCTGACCAAGGCGG
LG6	ref-67990_1	7.709			[C/T]ACACACACACGACAATTTTGCCTCCGGTGAT
LG6	ref-66480	9.2			TCAAGTGAGACGAGTAAAGTGCATCTGTTGC
LG6	ref-24733_18	9.711			ATCTATTTTATATTATATACTTGTTTTAATTTTTTAGTAATTTTTTTCTTCTCTTTCATAATTTTTTACAGTTTCTTACCTTTTCCACAGATCCCCAGAATTTCCTCCAGCACCTATTTTATTTATTTTTTATTTTTATTGTATATTTAATTTGTATTATTTATTTAAATTTGTATTTTATTTTTTGTTACTGTTTTCTCTAAACATTTCGACTTC[C/T]TTGCAGCCCCTGGTTTATTAATGTATTTTAGTTAATTTTTTATTGTATACTTAAATATAAAATTAATACAAAAATTATTTATTTTTACATTTTTTTATATGATTTTACTCAGAATAAATATTCNNNNNNNNNNNNNNNNNNNNNNNNNNNNNNNNNNNNNNNNNNNNNNNNNNNNNNNNNNNNNNNNNNNNNNNNNNNNNN
LG6	ref-49306	9.909			TCATCCACCCCGACCCACCTGCAATTAACTG
LG6	ref-3644	9.918			TGTCATACGCCGATAGGCCTGCATTTGCTGA
LG6	ref-30415	10.129			TGACAACACTCGACAGAAATGCCAGCTAGAT
LG6	ref-60842	10.376			GTGCAGCTTACGAATGCAGTGCCAGTTACAT
LG6	ref-21213_25	11.449	Chr19	2894379	TATCTACTCTGGAATGGCTTAAAGGGATCCTATTATGCTTTTTCACTTTTTCAACTTTAGTCAGTGTGTAATGTTGCTGTTTGAGCATAAAAAAGATCTGCAAGGTTACAAAGCTCAAAGTCCACTCCAAAGGGAGTTATTTTATTTAGGCTAACAGAATACACTTTTCAAGAACTACAGCAAACGGCTCGTT[C/T]GGACTACAATGCGTTTTTTCCGGATCTTGTGAAGTCACAATGTTACTCATTGGGTGAGGCCTAATTGAGCTGTGCTAGAGAAGATGAAGAGTGTTGTTGCTGTTTTTTCCACCCCGGAAAAAGTTTGTCCTAAGGCAGTTAAAATTGGTTAAAATTTATTTTTAACACTGTTCCTG
LG6	ref-34353	12.067			GTTCCCCTTGGCGCAGGAATCGAGTTCAGCCAGGTCGCGACCTTTTCACCTCTGCGTGACACTGGAAGCAGAAGCAGCAGAAACGGTTTCTATGACAACCACTACATTCCTTCCACCGTCACTATGACAAACACAGTGATGGGCAGGAGGACATGAGCTTCACATCACTAAACAAATGTGCACAAGAATCGACCTGAATTAAAGAGCAAACTTTATACACACATATACAGTTCTCAGAGTAAATGAGTACAGCCCCTTTGAAAAGTAACATTTTAAACAATATCTCAATGAACACAAAAACAATTTCCAAAATGTTGACAAGACTAAGTTTAATATAACATCTGTTTAACTTATAACATGAAAGTAAGG
LG6	ref-13457	14.325			ATGGACAGCACGACAAATATGCTTTTATTCT
LG6	ref-2306	16.015			TCTCAAGAGGAATTTCCCTATCCTTTTCTTGTATGGTCAGAGCTACATTTGTCACGGTGTGTTCGCTTAGCGGAATTATCTCCGGTAAGGCCTGTGTGGGCCTTGGGACCGTTTTTTCGGCACCAGTAACACCATTTTCGGCCTGTATGGGCCCTGCGGTCGTGTTTTCGGTATTAATGACACCAGTGTCGGCCTGTATGGGCCTCTGTTCCGGCATGGGAACCTCATACGCTTCGTACAAAAATTTCGTGAGCGGCGAGAGGACCTTAGCTGGTTCTTCCTCCGAGTCGGGGAATAAGATTATGCTAAGGGGGGAGGAATGGTCTGATCCTCTGAACTCCGCTTGTGTGAACGGTCTGTTTCGTGGCG

LG6	ref-18936_26	17.201	CTTTTGAACCAATAAAACATGCGTTAATGAGAGGCCTTCGTTAATTAAAACTGTAAAATAATTATTTATATTTTGTATTTTGGTAATTTGGTATTTTCTGAATACCAAACGC ACATTGCACCAGACAGTGTGTGTGATTGATTGGCAGACGCGCTGTAAACTCATTCCGCGCAGCGCGTGCAGGTCGCTCGCTC[A/C]CGCGAGGTGGGGGTGCGCGGCGAGG GGAGGAAACGCCAGACAGACGCGAGCGGAGCGGAAAAGAGCTGCAGGGAGTCGAGCGCTGAACGCCGGGCGGCTCGGAGGTCTGTGATCGCGGTTCTGCTCGCTCGAAA CGGAGCGCACCGGATTGTCACGCGCTCGCTGGCTGGAGGAA
LG6	ref-22419_18	17.633	CGTCTGTCTCCGAGAGG[A/G]GTGCAGAGATGAGT
LG6	ref-11897_4	19.2	CAG[A/G]CCAGCTCGATTGTTCTGCCATGTGACTC
LG6	ref-59918	23.955	CAGTTACCCCCGATTTCACTGCTGATTTGAA
LG6	ref-71082_9	25.522	TTTAGAAAACACACTCAATCCTCCACCACTAACTACACTGATATTTTCAGATAAATCTATAATCTATCTATAATTGTAAAAAGGGCATTCAGAGGATTATATTTTTCTATGT ATTTGTAAAACGGCTTTAGATGTGATCGATGCTTTTGTGCAAATTCATCTCTGAAGTCACTGACTGTCTGTTGGGTGTGTGAGTATGAAACGATCG[A/C]TCGATGTGTGTGC ACTAGATCCTAAAAATGATGTTTATTAGACTAACACAAGGATGCGCGTGTGTGTGTGTATGTGTGTGTGTGTGCACGTGTGTGTTTCCAACACTACGCTGATGTCTTGTACG TGACCTAATTGAGTTTATTTCCATACACAGCATATTAAATGACTACTGAACAGATTTTATTTATCCAATGCAAAACTCACAGTTTAAGAGCTATATAT
LG6	ref-2485	26.244	ACTGTTATTACGAGGCGCATGCGCTTCAAAA
LG6	ref-39150_7	26.435	GCTGCAAAGTGAACTACAAAGTTTCTCTTCTTCACCTGTGGCGTGGGTGGAGCCGTGTGTTCGGGAGGCGTGTCCTTCACGCACGCTCAGTTCTGCAGTAGGTGAGAACGG CCCATCTCCCATCTGATTGGACGCCGAGACCTTCACCAGATACACCTGACCAGACTGAAGATTCT[A/G]CAGCAGGGCCATCGTGATGCTCCCTGACAACGACACAGTGAG GTGAACATTAGTATCCAAAATAATGAATACACCTATGAATAATTTACATCATAAATCTTTTGGGAATGTTTTGTAATGACATTTATCTGTATTCTGTAAATGGGTGAATCTC ACAAAGACAATAAAAAATTTATATTTCTACATTTTTCAT
LG6	ref-56056	27.041	AGTTTGGAGGCGACATCATTGCTCGCTTAAC
LG6	ref-71014	27.795	GGTGTGAATGCAACAAGGAAAATGGCCCGAATGAAGAATTGGTTCTCCGGGTACTACCCTAGTGGCTAGTTCCTATAACTTAGTTCCTAGAACTATATGGTGCGAAAGCCC CTTCTGACACTCTTCTTCTACTGCTGCTTCCTGACACCTCTCTGAAACGCCACAACACCCACAAGGTGGCACAAGTTTCGAACCCCCTTTATCTGGAAGTGACACTCAAACC AAAACGATGACGTATTCCACACAAACCAAGGCCTGGTTTGTCATTGGTCATGTGATTTCTACGTTAACAAAAGTCTCAAATCAGGGTTTCATATGGTATGCCCATTTATTCT CGACACAAGCTTCGATGCCTCAGTTCAAATGTGA
LG6	ref-64799_8	29.4	ACCCATATCACTCACAACAAATCCCCATTCACCCAAAATAGAAAGTTAACACTTTCACTCCATCTACTTTCATTGTATGATGGTCAAACTTTGCAATTAATCCGAGAATCAT GTGCGTTTTGAACAGTGGTATCTGGTCCGCACGGATTAACGATCTCTATACTTGACATTGCACATCCAGCGATGTGACTTTCACAGGTGTGCACA[A/C]TGCGATTTCCATG CTAAAACCATATATCATGCAGCCCTAATTTAGTGCTGTCAAATGATTATAATTTTTTTTTTTTTTTGGATGCGATTAATCACGATTTTAAACATTTGACAGCACTAATATAT ACTAAAACTAAAAGTAAAAATCAATCATTTCAAATGCTACATAACAGTTATTCAAATACTACATAAAAAATGTGGTAACACTTTTGTATAAGGAACAC
LG6	ref-30766	30.433	TAACACTTCCTTTAATCCCCCTCTGACTACTTCCACACACTCCGAGACTGTTACTCACACAAAGTGCTAACATAAAGTCCTGATTCAGACGTTGCCCTCTGACACCCCACTG CCTCAGAGAAAGCAAATCCCTTACGACGGCAGAGAAACAAACTGCCCTGTCACTCTACGCTGGGAGTCCAGTCAGATCGACTCAGCTGACAGGACGCTGAGTCATTCCGC TGCCAAAGGGGAGGCGAAAACTGTGTGTGTGAGCTTGTTTAGGACGGGGTGATGATTCCAACATTTCATGCTTTATCTCTTGACAACCAGCACAGAAAGGAAGGGGCTGTT TGACCGCAGAGACTGAGGACATTTTAACCCTTCCAT
LG6	ref-45946_4	32.455	GCT[A/G]ATACACCGAAAGGGATGCACAAAATCAC
LG6	ref-16681	34.599	CTAGCTAAACGATCAGTAGTATATAAACAAATGATTGCCTTCCAAGTGCTTCCGCTGTACTTCCTACGCCTTCCCTATTCAACTTACGGAAAAAATGGAACTGGCGCTGCAT TCGTTCCGTAAGTAGAATAGGGAAGGCTTAGGAAGTACAGCGTAAGCTTTTTGAAGAATACGAAAATGCAGTTTTGTCGGAAGCACCTGGAAGGCGATCATTTGTTTATAT AAAGCATATACATTTACATTTTTTTTCGAAAATGACCAATCGTTTCGCTAGATAAGACCCTTATTCCTCGTCTGGTATCAATCAAATAATAGCATGTTTTCATTTCAAAATAG CGAAATTTCATTAAAATGTTGAGTAGTTTGCAT
LG6	ref-67050_26	35.107	CGGCATCTAGCGATGAGGTTGCAAC[C/T]GACGGC
LG6	ref-24052	35.774	CTTTATTAACCGATGTCTTTGCTTCGATGAT
LG6	ref-45523_30	36.494	GAGTCATTTATCAAATGTTGTGATAAGTGACGTAGAGGACGGATGAAAATGGCAAAGGCTACCTGACGAACGTCAAGCTTGAGCCGGTTGTCAAAGCGGGCTCTGCGCAT TTTACAGTGGGTCACCCAGCTGTTAGCGAGTTAATCATCCACTCTAGTACTAGGGAAGGGCGCAGACAGGCAGTCACCTCGCACACAC[C/T]CATCTGTGCGCTCGCACAC AAACATACAGGAACATTTCCTGAATTTCTTGGCATGTCTGAACCTGTTCTGCCACCATTTGACTTGAAATGAAAGCATCTCGCAGTGAGCCACTTCTGAGAGGAACTGCAG TGGCAGAACAGAGACATGCTGTTTATTTTTCTTTTATGCAA

LG6	ref-66590	37.86			TATGCATCTCCGATAGCAGTGCTTTCAATTC
LG6	ref-4380	39.062			AAGGAAAACTTTCCCTCAAGTTTTGTATAGAGCTGTCACAGTAATACTATTAATAATATAGCAATAGTATACTGTTGCTCTACAGAATAACTTTGCAGCCGTCTTTCATATT<br>CACTGAGTGACCAAAGAATTACCGGTATGTGAAATCTCAAATGTCGATCTAGAGTGGCGTAAAAGTTGCATGAATTCCGTCAGACCTGTATCTCGTTTAGGACCACATATA<br>AAAGTGGCCCAAATTTGTGCTGTTCACACTATGAAAAAAACTTACCTAATCTAAAAGATTGAATATGAGCAAAATAATTGGATTGCCTCCAATCTGAATGTATCTGAATAA<br>CGAAAGATACACTGCACTAAATGCATCCACAACAA
LG6	ref-52302	41			ATGTGTTTTACGAGGCAATTGCATTGATAAG
LG6	ref-37666_17	42.533			GAGCCATTCATTTGCTACTCTGATTGCACTCACTATGTGCAATTACACTGCATGCTCACTCTTGCACATATCCAAGGTAAATCATCACCACTTTCATTTCTTAAGTGTGTTTT<br>GTTGAACTTATGATGTCGTTATCAGCTAAAATGCATGTGCAGATTGCTTTACATCCTGAACAAGTGAGCACCCGAGTTCGAGTTGCTTTCACATTCACGAGAA[C/T]TGTGC<br>CACAGTTACAGTGCAACCGAACTCAGACCACCTACTACAGGTAGTCTCGGGTTCAGTACCCTGGTGCACTCCTGGGTCTGCATGACAGCTTTTAAATTATCAATATAAATC<br>ATGCACTGTTACGAACTAAACTGCCAGTGTGAAAGCAACCTATTTAAAAAAAAAAAAAAAAAAAGGCCTTAAAAGCAACAAAAAATTTAATTCCCCTTTA
LG6	ref-30675	43.449			CTGAGGGAGTTTTGACAGTGTTTGTGGCATGTTATCTTCCTCAACTAGTCATAGTCAAGAATGAGGATCAAACCATAGAAAACAGACCGAATCTCACATAGGTAGTGGTGA<br>TCTTGGAAAGTAAGATTAAAAACAGCAGATATTCTAAATGATAGTTAGCACCATGATCCAATCCACCTGTATGGCCATCGTCAACCCCCACACTGGGTGGTGGAAAGAGA<br>ATATTGTTCTTAGTAGTACTGAAACTACTAGGATGAACTAACTGTCATAGAGATTAGTGGTCAACTGATATTTTTTTCAATGGCTGATGCTGGTATAAAATTGTGAAAATGG<br>CGATGGCCAATACAATAAAATACAAAAACTATGACC
LG6	ref-32144	45.167			ATGTCTGCACCGACTTGGATGCCAAGCAGCT
LG6	ref-49029	45.577			ACGGGGGTAACGAGTTTAGTGCATCCATCCA
LG6	ref-13947	46.179	Chr18	19147436	TGTCATTGTCTCGGCGGTCAGACATCTGCTTTTGGGATACGATCCGATAGATTTCTACGGGACAGAAAAAGATATTGATGCTAAGACAACGTACACAGTGATAACGATCTT<br>CGAACTGGTAATGAATACGGACTTTGTGTTGCCGTAAAAGCCTATAAACCCTACAGTTATTACTTAATGTCAGGGCAAACACAATTACAGCTTTGCAACTGACTGTGATGC<br>CTTTGCCTTTGAAACATTTATTATTTGTTCTTCCTCTGCAGATGCCAGGAGCCAAACAGAGGAGCAAAAGACGACGGAAACTCTCTATTGCAGTCACGTCAGGCTAGAGAA<br>ACCTACCCGACCAAGTCAGGGCTTTTGATGAGTCATTCATAAGGATATGGCCTTCAGCCAATCAATCTTTCTTTTAGACAAAAACACAGTCATTTCCT
LG6	ref-54843_17	46.776			CCATGAAATAATGCCGTTTTTCTCTGCTCGCTTCTCGACGACTGGTGCTTCCCCTCAGCACAAGTTTGGTATCGTTACAAAGCGCAGCATTCAAACTTTGTGAATATTCATA<br>GCATATTCTCGATGGCATAAGTCTGCACCAATGAAATGTGATTTTCAAACTCATGCCGATGTAAACAAAATGATCTTACTTGCGCTGACCGACAGATCCGAAGCA[A/G]AT<br>GCACAGAAACGCCTCAGGCAGCGTGCGATCCCGATATACACATATTCACAGAATTACAGTATTTCAAAATGTCTGTCTTGGCTAGTATTCACGAAAAACATTATCTGTTAT<br>GTCTTAAGTGAATGTTTGGTTAACTGTTGGGGGAAAACATCTCACGTATAACATTATATTCGATCAGTGCGGTACAGTAGGTCTTAATATATAGGCTGTTT
LG6	ref-8211_32	47.046	Chr18	15151287	TCTTAATGGCTGCATCACGATCCTTCCCGAAGGGCTCAGAGTCGTACTCATATGACAACCGGGTGATATTCCACTCCTATGCAGAAAAAAAAAAAAGAGAGGGAGAAGCG<br>AATCAAAGAAGTGGCACAGCATAAACACACAGAGCATGGCTTCACAGAAAACAGTTGACTAGTCTCTAGGCAGTCTGTTCGCTGACCTTG[A/T]ATAGTCTTGGAAAGACA<br>TCTGTGGGTTGTCCCCTGATGACAAAGAGGCGAGAGTTAAGTTTGCGAAGGCTGGCGTCCAAGTCCTCAAGACACTGCAGCAAAAACCTGTACATAAAACACAAAGAATA<br>ATCCATAAACATTTAATCTAAACAGCTAGTGATGAGCAAATA
LG6	ref-5826_16	47.637			TTCTGCATTGCGGAAATCATAGGACCCTGTATTGTATTTTTGATCATATAAATGCAGCCTTGGTGAGGATAAGAGACTTCTTTCAAAATTTTACTGACCGCAAACTTGTGAC<br>AGTGAACATTGTCATATTTTTTGTCTATTCTTCCAAACAGTCACATGAGACCATGTGTGACTGCTGTGCACC[A/C]TTCTCGCTTCAATTGCTCTGCATAGTGATGCTCACTTT<br>CAAGTGGAATTGGCTCCTTATCTTGTGAGTGACCAAGCCATAAAACCAGGAAGAGTTTAATGTCGCCCAACAATGCTTAACCAGATAACAGTGCAAGTGCGGGAAAACTG<br>CTAAAATTGTGTTGTTTTCCGAGAACGATTACTCAAA
LG6	ref-54816	48.2			ACTGATCAAACGATTGGTCTGCTAGTTAGTC
LG6	ref-3022	48.232			CTCAACCACCTGTAACCACTCCAAAACATATTTCTTAGAAGCTATAACTACATTTAAAAATAACCTGCTGAGCCCAATGCACATATTTACATACTGGCTATGATCTAATGTT<br>AAATGAAGGCCATGCCTTCTTTCCTGGACTAAAGATCACATTAAGTACATACTACACTCAGACGCTGGCACTGTAATCGTACCTCCATAAATAGTGCATTCTTTTGAGCTTC<br>AATAAATAAATATAAGTCACACTTTTAAGTATGAAGTTGTATAATTTTTATTTGTTGAATGTAAAAAAAAAAAATAATAATAATTGTATGAAATGTTTTATAAATGGCCTGTT<br>TCTTTCTGGGGCCTCAGGGTGGCAGTGTTGCCT
LG6	ref-73037	48.988	Chr18	13720304	TCTCATCCCTGCTCTACTAGAAACACTGTGAGAAAACAACATGTTCCTTCTCAACAGATAAGACAGAAGACATCATTTGACACTGCACAGAAAACCGTCCCTATTACAACA<br>CCAGCCTGTCCAAAAGCAGTAAAACACGGGCTCTGTTTTTTGACGCTACATTAAATTGACCAGATCTGACTTTGCTAGGTTAGATTGGTTATTGCATCTCGACAAGAACGCC<br>CGCGAGCCTCCGAGGAACTCTTGAACTCTCTTGCCTGTGTATAGGTTCGTTAAAAGCACCAGGTTTAATGATCCATGAGCATCAGATGCAGGTTTCTGGGTGTGGGGCATT<br>GAAATGATGGATTGATCTGGATATTTACTCATTCAAGCTGCTGAAATGTGCCGGCTCATGCAGCGCTGAAAGAGAGAAATGCATTTGTCCCTGTGCA

LG6	ref-31950_2	49.432			C[A/G]TTTTATGCCGACTTCAATGCCTATTTGACA
LG6	ref-69120	50.107	Chr18	9760749	CAATGCTATAAATCGCAGCCGAGTTATATAGCTATGATGCTCGTTTAAATGAGATACTGTTTATTTTTCTTCCCATTGCAATAAAGTTAATAAGCGTGCGAGCAGGGATGGGAGGCAGGTCGGGGCAGTAAGCGGCGAGTTACCCAGGGATTTCGGCTTTCACCCGTTGCCTAGCAACCGCGCTCTCCGCGGCAACGACGAGTGTCAAAGCGAGGCGCGTGCAACAGGGCGCGAGACTGCGAGAGAGCGCGAGCTCTATAATAGGAGAGGCCTTGTGCTTTTGTTCCCTGGTCTGCATGAAGGTCCATTAGCACCCATCCATCCCATCGAACGGCGGATCCAACAGGGCCTAGAGGAGGTTTTGTACTGATGCACCACCGCAGCCCCTCTGATGGAGAAGAGATACAGTGACCCTGTAGCTTCAAGCACAG
LG6	ref-34174_9	50.505	Chr18	21668714	GTTTTTTGTCTTTCCAGCACTGTGTTGAACAGAGAGTTGGTGAACCCGGCCAGTATGAAACAGGCCCTGATTGCCTCAGCACGCAGGCTGCCAGGTGTCAACATGTTTGAGCAGGGCCATGGCAAACTCGATTTACTCAGAGCCTACCAGATCCTTAACAGCTACAAGCCACAAGCC[C/T]GGCATGTCTCTCGCACACACACAAATATTGCTTTCCTCTCTTTGCCTTCACATTAGCATTGATGTATTTAGCAGACGCTTTTATCCAAAGTGACTTACAAAAGAGGGGAAGCATCCTTCTCTAGCTTTCTGCTCGCATAAAATGCTCAGTTTCTTATTCATGTATGCCAAATTGGATCCATTTAAAATGTAA
LG6	ref-46338_2	50.83			G[C/T]AGTGTCTTCGATGGAGGTGCCATGATATGA
LG6	ref-27765	51.247			AATCTTTTCTAATGAATAAAATCAGCAACCTCTCGAGTTTTGCTACTTTCAAAATTCAGTTTCCTTGTTTATTTTGTATTCTTTTCAGCCTGGTGTTGGCATTTGACACAAATATTTCTATTTGATGGATGCATTCAGATTCTGGTGTTGTTTTGGTGACTCCTTTTCACTTCTTAATCACACTGTGAGTATCACAGTATGAGAATGGTTTGATGTAACTGCTCAACATTTTGAATTCACAATGAGCTCACATATCACAGTATGAGGATCGTTTGAATTCATAATGAGTTCATGTATTAGACACACTGTATCACGAATATTGCTCATGAATTTATGATGAAAAATTTTGTTTGACTACTTGTACACTGAAAAGATACAGACCAAAATGTGATAGATAGATAGATCTGTATGCATTAATGTACTCATAATGC
LG6	ref-28728	52.037	Chr16	4298174	AAAAAGCCCATAAATACTTGCAAAAAAATCATTTTATTCCCTGTTTTTATTGATCACAAATGTGAAAAAGACAAACGTTTCAGCTCTCAAACTTCAGTGTGAATTCCATTATTTTATGATACTGTCAAAGACTTTGCAAAGAGATTATTACTGATGGATGGAGCAGTTACTCTGTATATTAGTATCAGTATTACACCTGACAGAAAACGTCCAGCCATGTGCAAAACAGCGATGCTTATTTTAACTGAAATTTTGATGCTTGTATAGTAGCCTTAAAGGGAAAGTTCACCCAAAAATAAATGTTGTCATTACTCACCTCTATGAATTTCTTTCTTTTGCTGAACACAAAAGAAGATATTTTGAAGAATATGATTTAACAGTTAATGCTAATTAAAACAAAAGAAGATATTTTGAAGAATATGATTAAACAG
LG6	ref-15654_16	52.369			AGAGGAGTTCAAGATAATGCAAGTGAGAGAGAAGTGCTGCAGTTGGTCTTAACATAGACAATTTACTCTGTTTGAACACTGCTTTGAGCATCTTCTCTTGCTTTGGCATATAATCTCACTGACATTTATCATACAAAATGATATCACACAAGACATATTTTTTAAACATTAATAACTCAATTGGCTTTTTCTCTTTACATTAACTTCCAGCGACAC[C/T]AGTGCATCTATTGTCAGTAAAGTAATAAATGTTTGTCTTTGAAGTGGTTTGTTAATAGTTTTCAATAAAGGTTATTTTAGACATCAAAAGTGTGTACTGACAGAATAGAGGCCTATATTTTGCATCAACTCTGAAATTTCAAAAAATCTGTCACTAAAGTAGCACAAGACGATAAGTTGAAGACATTCATCTTTATTTCTGAAATAGAGAATACA
LG6	ref-68385	52.785			CATTCATTTTACCATGGTGAATAAGCAGAGGGTAACAGCTGCTTCAAAACCATTTTGAAAGTTCCATACGACTGCCACTAGGGGCATTCATATGCGATCAGACACTTTAGTTTAGAATTAATTTACACATACACCTGTGTTTGTATTTCTACAATGAATTTGAAAACCAAGTCTTTGGTGCACTGCAGTCGAATCGTCTTGAGCCAAGGAATCAGAGAAAAAAGAATTTTGTTTCTAGCCCTTACGGTTCAAAAGCTATTAGCATAAACATGAGTGCATCTTTGAATAGTTGGTGGCGCTAAAGAGATTGAGTTAGATACTCCAAAATTGCTGTGGTTAAAGTTCAGACTGTCCTCTATCTGTGTGCCA
LG6	ref-2268	52.831			ACAATATGTGCGATTTTAGTGCAGGACCAAT
LG6	ref-32621	53.27	Chr18	14484609	CATTAAACAAACCCAGTGATTTATCACAGGCTGACCTATGGTGACACTCAGGCTCTCCCGCACCTAAGGGGAGATTGATGGGACCACCAACATAAAGCCTTTGAAGTTGTGACCTTTCATTGTCCTTTCTCTGATTTCCCCTCTTTCTTTAGGTTTTATTAGTAGTGGAATGTGAAAAAGCATTTGGATCCTCCATTCTACACTTGAACAGTGCTGTGGTTTTTGTTGGTAACTGCTTTAAGATCCCAAATGTCCATGCCTGTGATAATCTGGTGAGTCCAACAGGTGGGCACTTACAACCAACCAAAGTAACAAATAGATGTATATTCTAAATACTCCTAAGTGTATTGTATTCTATTTTAAACATGT
LG6	ref-24330_26	53.661	Chr5	15874094	CTTAATTCTTTTCTTTTTCAGCCATTCTGGTGTAGATTTGCTGGTGTGCTTGGGATCATTGTTCTGACTCCTGTTGCATGACCCAATATCGGTCAAGCTTTAGTTGCTAGATAGATGGTCTTATATTTGACTCTAGAATGCTTTGGTATACAGAGGAGTTCATGGTCGACTCAATGACTGCAAGGCACTCGGGT[C/T]CTGTGGCTGCAAAACAAGCTAAAAATCAACAACCCTCCATCTGTGTGATGGACAATTGGTATGAGGTGTTTGTGCTGTTATGCTGTGTTTTCACCTAATTTGGCGCTGTGCATTATGGCCAAACATCTCCACTTTGGTCTTGTCTGTCCAAAGGACACTGTTCCAGAAGTCTTTA
LG6	ref-66674	53.759			GTCCATCGCTCGACGACATTGCCCGGCAACA
LG6	ref-17287	53.975	Chr18	4697407	ATCCTTCTTTATTAAGATCTCAGCTAGATGGCGGCTTTGGTGTAAGGGAGAAAATGACAACACAGGAAGAAAACTATTCATTTCTTCTCTATTTCATTAAAAGTGGACCATTGACAGTACTCAGAATTTAGCTCGAAAAATGAACTGCGTCCCATGATCTCAAACGCATTATGGATCCAGCAAGCTTTCCGGCCTCATTAATGCTGGAACATCTGCAGTCCACTCACAGATGGCCCATGGGACTCCAGTGGACGCCTTTAAACAAAGACGCCCGGTGGAAGAATAAAGGTTGGCTGAATTTTCTTCCTTTTTAGTGGTTTAGTCTCTGTTAATGCACGCATTTCACACTGGGAGATTGTCTATGTTTCC
LG6	ref-37692	54.662			CGTTTGAACCCGAAAGCGCTGCGGCGTGAAG

LG6	ref-15055_24	54.677			CTTTAGCGACCGACTCCCATGCC[A/G]GTGGACCC
LG6	ref-26146	54.92	Chr18	10953188	TTTCGCTCCCAGCATACACCAATATCTGTAATAACAGGTGGCTACTGACGAATTCGAAAATTTCACAATTGTCAATGCTACAAACACGCGTGCCGGTCACTGAGGGTGTTCTGTGAAAACTACAGAAGTCCATCACATGCTTATGTGTGAACCACACATAAAGACTTCATTCTTCTGTTGCATGGAGGTCTGATGATTATAGTGCGTTATATTATACAGGGGAGGAGTGTGATTGGAAAGAAACAGCTGAACTTTTTCAGTCAGTAGTTAAGGTAGAAAATGTATTATGGGAAATCAAGTCCAAATGCCTTGTCGTCACTGAAAACGTAACAGTTGATTGACCAGACATTCTCTGAAAAACAAGACTGG
LG6	ref-14655_8	55.129			ATGACATAAAAAAGTCAAACACTTCAGAAACAGTAAAACAATACCGAAATCACGCCATGAAATGTTTACAGATCTTTTACACACTTTAAAGGGGGATAATAATAATAATAATAATAAAACACACACATAGCACATACAAAAGCTGAGGAGTTTTTAACGATCGCTCACAGTAAACAAGTCTCGAAAACGAACTCACCCACGTCCATCC[C/T]ACGATAACGATGCAATGTTGGAATCACTCACGGCACAAATTCACTGTTTCCAGTCCCGTTCGATCCACCCCTGGCACACGAAAGAGCACTTTGGGCCTTATATTACAAAGAATATAACTAGTCTCCAAAATGACGACACATTTTTCACGCAAACAATTCACATGCAGCTGCTTCTCGCCGTGTGCGATGCTCCTCCCCTCCTCCTTCCGCACGTAACTCACGT
LG6	ref-61766	55.149			AACGAGCGAACGAATGATATGCAGACAGAAC
LG6	ref-72067	55.482			TGCTGCCACACGAAAAAGATGCCCACATCTT
LG6	ref-15319	55.57			AGAAAGAGAAAACATGACAGAATAACACGACGGATATCGCAGTCTGTGCAAAATTACTTTTCAATACCAACCAGATACGATACTGAATTTGGCAGAAACTAAAATTTTTTTTAAAAATGCCATTTATCACGTTTTGGAACCAAACTCCTCATTTGCAACTTGGATATATGCATATGCAAATTGGATACACAGAAAAATGCAGGTTAAACTCGCAGCACATGCAGTGATGAAGCTTTGATATGTGAATCATGCGCTTCTTGCGTGAACATAGTGCATTTCATTCTCAGGAATGCAAACAGGTAAGGGGGAAAAAAGGTGAGAACATTGCACGGGGCTGGGGCATGCTGCGCACTTAATTTTTTAAAGTTATTTGCTTTACATTCTCATTTGTAGTTACTTATAGGGTGATAAAAAATAAATGAAAAAAATCC
LG6	ref-42188	55.596	Chr18	11832437	CCAGAGTTTGACCCAGGTCTGAATACTCATCCCACTCTCTGGCCACGGTACACAGGCCTCTCAACAGGAGGCTGTTTGTCACGGCTCGAGTCCCTCTGGCCGGAGTGCAATAAATTAGCTGGACTTTGTTCCTGCATGTTTGTGGAGCTTCTTTGTCTTCCCATGGAGCGAGAGAGTGGGCGGCCTGCTCGTTGTGTTTGACTGCAAGGCCCATCCTGACCACCGATAGGATGGTTTATCATTCGTGCGGCTCTTGTTGACCCACAGGAGCACGTGGGAAGGAAAATGAGGAGAATGACATTGGATGGATGAGAACCAGCTGAAAGAATGCAGAGAGGAACATGTGCTACAGGGATGGGAATGTATACC
LG6	ref-51112	55.74			TGGTCCCAGACCCTCTCGCTCTCTTGGAACCAGTAGTCGACCGCTGGCATGTCCGAGGGTTCCCCTGACCAGGGGAACAGTGGGGGTTGGTAGCTGAGTACGCACTGGAACGGGGTGAGTCTGGTGGTAGGCTGCCACAGAGAGTTTTGGGCATACTCGGCCCAACCCAGGAACTGGTTCCAAGAGTCCTGGTGGCCGTGGCAGAAGGTACGAAGGAAGCGCCCTATCTCTTGTATCTTCCACTCCGTCTGCCCATTCGTCTGTGGGTGGTATCCTGAGGAGAGGCTAACGGTCACACCTAGGAGTGTGAAGAAGGCCTTCAAGATAAACTGTGATCCTCTATCCGATACGATGTCTTCAGGAATTCCAAAATATCTGAAAATATGATTGAACATTAGTTCAGCAGTCTCCATGGCCGTGGGCAGTCCTTT
LG6	ref-40966_19	55.904	Chr18	9821613	TTCATCTTTATTGTTAAAACAATATTAAATTATACATTATAGGCAAAAACACCAGTTCAAATGTCACTGAAAGTGCAAAAACACTGATTTCAGGACACACAAGCCACGCTTTTCATTATATCAAAAGTTTGTCGGGAGCTATATAGTCCTCAGTTCATTTCCCGCGCTCTCAATGCCATGCAGCGTA[C/G]TCGTATCTGTTCTGTGATATTCGGCGAGAAGTGTTTGATGCATATGGATGTAGAGCCGCTCAGACAGCTGCGACTCGGTAGCAAACAGAGTGTGCCGAGCATCCATCGCTCTTGCCCTGCCCTCATCAGCGGGCCTCTGGCTACCTTCACACACATGCAGTTATCGACAGCCCCTCGCTAAT
LG6	ref-41336	56.031			AGATCTCAAACGAACTTACTGCTCGGCGAGA
LG6	ref-34704	56.063			TGTTGTTGCTCGATGCACATGCTATTCCCAT
LG6	ref-19618_2	56.246			T[C/T]ACTCTGGACGATAGGGCTGCTCTTGCTCTT
LG6	ref-57199	56.343	Chr18	35795414	TTTTTTTCCTGCATGTCATGTGGGGCTCTGTAATGAAGGGACAGAAAGCTCTTAGATTTCATCAAAAAGATCTTCATTTGCGTTCTGAAGATGAGCGAAAGTCTTACGGGTTTTTGAAGGACAAGAGGGTGAGTAATTAATGACAGAAATTTCATTTTTGGGTAAACTGTCCCTTTAAATTGGAAGAAATCAAATAAAATCTCTAGTCGTCGACATTTATGCACTCTGTTTCACCTCATAGCGGCTCTCTCATGAATTGGACACTGAAGTTTAACCTTTGCATCTCCGCAATGCCTCCAATGCTGTCGGTTCTAACTACGTGGTATATAAATCTTATTTTGCCTGAGAAGTCACCTTGTCATTGCCGTATGCACTGTACGTGATTCAGTGTTGTGCGTTGGTGTTCTGAAATGAGGAGGCAAAGTCCTTAG
LG6	ref-45591	56.504			GCGGTGATGCGGGAGCACATTGAGCTGTGATCAGTGCAAACAACGCGATTATACACATTAAATCCATCATCTGATATGTAGCTTACAAAAGCTTTATAATTTAAATATATAAAACGTTAAATATTCCTGCTCGATTTCTTATGAACAACCGCTTTGTTATGAGCTTAGGTCATTTCCTCGCAGCTTCATCGCTGTCATTCACATCTCGCTGTAGTCTTTCATGTTGTTTCATGTGAGTCATATGACCACCGACACGTGATCCAGTGAGAAGAGGGCTGATGGGATATGTCGTTTTTTAGCCAGATAGGCGTAATGCATGATGAAGTTTTCATTGAATGGTTGTTTAGTGTGGATTATTACTACTTTTGA
LG6	ref-70875	56.823			AATGCAGTTACCTGAAATGAGCCCAGGCCACAAGCATGTTACCAGTACCTGAAGCTTAAGCAACAGCATGTTGCCAACAATACCTGAAGCCTTAAGCAATGAGCATGTTGCCCTTACCTAGATGACCTAAACAAGTATGTTGCCAATACCTGCACATAAGGCATAGCAACAAGCATGATGCCAAAAGCTTAGCACAACAAGCATATCCCATGAATGACATGCTGTCTGAAATGAAGAAAGTGAAGATTGAGTTATCAAAATGAATCGACACTGCTTCCTTAAGGTTTGTATCCGCAGTCCAAGTTCCAGTTCTTTGATGAATGTTACTACCA

					CCTGCTGGTATTGACAGTTAATCCTCTCATGCAGGTGCAGAAGTGCATGCTTGTTGGCTGTAGTCTTGTGGTAAACCTTCATCCGTGTGAGTTGTCTTA
LG6	ref-7063_32	57.179	Chr18	2465680	ATCTTCTTCTTTAACTGAGGCAACGGCCCCATGGAGAGGAAAATAGGGTTTTATTTGGTGCTACAGGAATGGGTACATTGAATATTCCTCCTCAGGATGTCATATATAAAA<br>TATGGCGGGATTTCTAAGAGGTGGGTTGGTATGTTATATAATCATGTCAAAAAGCTCTTATGACACGTGCAGGGGGGTCGGAAACAACCAA[A/G]TCCTGTTAGTGTGATG<br>TGAATGAAGGAGATCCATGGAAACTAGAGATAGAGAGAGAGAGAGAGAGAGAGAAGGGCTGGAAGAGAACAAAGAAACAAGGTTGGTGGAAATTAGATGAGACACAG<br>AGTCAGAACAATATTCAGAGTGATAAAGAGATGAAGAAACTGACA
LG6	ref-41310	57.428			CTCTTCGATACGACCAATGTGCTGTTTTCCT
LG6	ref-56412	57.59			CCCTTTAAAGCGACATACTTGCCCCAAGTGG
LG6	ref-32452	57.695			TAAAGGAAAGCGAAGTTTGTGCCATGCAATA
LG6	ref-21625	57.936			GGGTCTTTCCCGAAGTTTATGCACTTACTGG
LG6	ref-9982_19	58.248			GTTACCGATTATAGATCTTACGTTTCGATAATTGCAAAAAGATTAGAAAATATAATCTATAAAGGCAGACACAAGATAATGTGAAACAAGCATTATTTTTGATTGACAGCA<br>TGAGCAAAATTCTCTGGTAATTAGCCTTGATGCCGAAAAGGCTTTTGACTTTTGGCTCACCCCCACATGCAATGCG[C/T]TCGCTCCAATGCTCTCGTTACCATTCAGGATCA<br>TAGGTACCTGCGCAGTGACACTGAGAGCACGAGCACCAAGACAACAATGAAACAATCTGCCCCTTACCTTTAGCTGAGGTAGCATAGACATGCCTAATGAGTCACCTATG<br>ACAACAGCGTTGTGTTCCGTCTCGCGAAGGACAGGATAA
LG6	ref-55589_29	58.412	Chr18	5166572	AGATGACACCAACTGATGAATTCCCAACACTGACGGTGGATTTCAACTCAGCGCCCGCCTCCTGTTTCGCCTCGACAGCCCACGGCGCCACCTGGACCTTCATCTCCATCTC<br>CTCCATCATCTGGTCCACCTGAGCGATCTCGGTCTCCAGCTCTTTGAGGTCCACCGTGTCGATATTCGCATCGGCTTCGTACTTC[C/T]TGTTTTGGACACTCATTGCTCGAGC<br>AGCCACGGTGGTGGTGCTGCCGGTCATTCCCGTCTGGATGAGGTGTCTCGTTGGCACTTTGAGAGGGAAATCCTGTCCTATCTCCAAGGATCGCCTCATGTCCACCTCCAGG<br>AGTTCCATGCTGCTGGTGAAGAGCACCCAGAGGCG
LG6	ref-33812	58.69			AGAAATGGATCTTTTGCTCCCTCCAGCCAATGCCTCCACTCTTCAAGTGCCAACTTGATGGCCAGAAGTTCTCTGTTGCCGATGTCATAATTCATCTCTGCCGGGTTGAGCT<br>TGCGGGAGAAGAAGGCACATGGATGGAGTCTGCTGGGATTCCCCTGCTGCTGCGAAAGTACGCTCCTACTCCTGTGGTTGAGCCGTCGACCTCCACGACGAAGGATTTGTC<br>AGGATCGGGATGGACCAGGAGAGGAGCGGTAGTGAAGGCTTCTCAGAGGGTGTTGAAGGCCTCTGTGGCAGCTGGTGTCCAGGACAGAGACT
LG6	ref-56522	58.718			GTTTAATTATATTTTATTTAATATTTTGTTAGGAGGTACAGTTTCATCATGGCCTGCGTCTGAATGGAGATTGAACTGTACTGATCCCAGCAAATGTTCTGACCATGATTCCA<br>GTTTGGTCGTTTCTTTGGGAACTGTTCCACAAATGTTCTTTGTGGGTTTTGAATTTTTATTTAGCTGCACGTAAGTCGCAGTTGGGTGAGTTTGAAGGGAAAAAGAACAACA<br>CAAAACAGATAAACACCTTTTAAGATAAGTTGTTAGTGTTTAGTGTTTTCTGCATTTAATGAGGACCAGTTAATTTTGTGCTATTCTGTAATGTTTATGACAAAGCTGCAAT<br>CAAAAGTTTAAACTCTGATAATATTATTTAAA
LG6	ref-5145	58.925			TGTCTTGCTGCGAGCCCACTGCTGCATTGCT
LG6	ref-21018	59.011			GATCGGCTCACGATGCCACTGCACTGACATT
LG6	ref-31875	59.324			TCAATGCCGCCACATGTGCACAGCTGTCCAGGGAGGGTCCCCGAGGGAGCTTCGCTTCCTATGTGGAGTGGGTGCTGGCATCCTGCGGATCAGCATTCATCGTCGAGGTTG<br>CTGCCAGCCCCACTCCCCATCCAGTGCCCAGCCAGAACCATCCAGACGTCGAGGATGAACAACCTGAGGCCACCGCAGGTAGTGCGATGGAGACCGGCGCGACTGATGTG<br>CCCACTAGAGCGATCGGGGGTTACATCGCCATGGAGCCTGAGCAGCATGCACCTGACCAGGTGTGTGAGCCGGCCGAGCCATCCATCGCCGAGGGAGAGTTGGTGGACTT<br>CGAGGGTTCGGAAGTGAGCCCCACCCACCCACGTCACTGCTGGACTCTAGGTGTGGAGAGGACATTTTTCTAGCCCCAGTACTGTTGCCTGAACCTATCT
LG6	ref-25282	59.357			ACGGGAAATTCGAGCATGTTGCCATTGCGTT
LG6	ref-57833_28	59.427	Chr18	33276381	AGGAACAGGAGGAGAAGGAAACTTAACTCCATATTGTTTCCTCGTATGACTGGTGTTTTCCTGTACATTATAAACACACCAAGCACTGCTAATGCTGCACAGGCCCCGAAT<br>GCTGAAAATGCCCATAGGATTATTCCCAAGGGCTCCTGAAAGGATAAAAATTCTAATGTTTTGGGAATGCACTGGTCTCGGGCTTG[A/C]TTTGGCCATGTTTCCTTTGAAC<br>ATGTCAGGCAATCGTTTGAGTCTGTGGAAAAATTTGTGAAAAGGGGCAGCATAAAATGTTTTTTTTTTTTTTACATTAATTTATTGATTGATTAATTTCTATATTTTGTATTA<br>GTGTTCTTGTTATTATTGCTATTCAAAATTACTTATT
LG6	ref-43459	59.893			TGCTTTGCAATGTATTTTTCACTGTGTGAGAACATGATGGGTGGCGTGAGAGCGGCATGGCTTGTCGGACGTAGCAACAGTAACTAAGAGGGAAGGGTCTTTGCAAAAAG<br>TTAATTGCAATTTCAAATCATTTAAATCTTTCTGGACTTGGCCGGACTTGACTCAGACTCGGATGCTAAGGACAAAGGTGGACTCGGACCCAACACTAGACGACAGCGTTC<br>CCCGGGTCTGAACCTTAGCTGACGACCATGCTTGCCTGGGCAGCTGAAAGCATCGGGCTTGAGTAGAACCCTCCACTGTGTCCCGAGCGCTCAAGGCTAGATGATTGGTTC<br>CTGGGGGCTGCACGCATTGATCGTCAGCATCCCCTGCCAGTTCCTTTCTTTCCGGTAGTGCATGAGGAAGTCACCAGTTCATGGAAAGGCACCTTTATC
LG6	ref-4859	60.292			ATGACACAGTCGACGGCGCTGCTTCCGCTTT

LG6	ref-70213	60.55			AGGGTATGCGGGGATGCGTCCAGTGGAGTGCTCTGTTGTGAGGCAGTTGTGTCCTCAGAGCGCCGCCACCTGGCGTAGAGATCCCAAGCACCTGTCCAAAGCCTGTAAGTTCTCATCCACGCTTATGGGCAAGGCTTACAGAGATGCAGGTCAGGCTGCTTCTGCCTTGCATGCCATGGCCATCCTCCAAGTACACCAGGCCAAGGTGTACAAGGGCAGTGCTGACCCAGGGCTTGTGCAGGAGGTGTGCACTGCTAACAACCTTGCCCTATGGGCAGCCTCCACCCATCCTGAGATGGGCGACCAAGAGTCAGAGACGTCAGCTCTCCAGGAGATGGTAGCAGGACCACTCCTTCCCCCAGAGGAGGGCGGGGTAATCCTTTGTTTTCCTTTTCTTTCTGTTTCGCCACTGGCCTCTTGGCCAGCGGTAC
LG6	ref-3927	60.721			TTGGACTCTGAAGAGGATGAGGAAGCCGAATTTCCGCCCTCCGGGATGGTCGTTCAGGCTGAATCGAATTCAGAGCTAACAGCCATGCTTGCCTGGGCCGTCGTGGGCATCGGGCTCGAGTGGAACATTGGGCTCCACCATGTCCTGAGCTCTTGCAGTTGGATGATTGGTTTCTGTCCAGGAACGACATCTCTGGTTGGACCTGGCCGAAATGAAGGATGCCGACAAAGTTCGATTTCTTGTCGCCCCCATTTCCCAGGCCAGCCTCTTC
LG6	ref-70496	61.305			AAGGCATGAAATGTTATTAGAAATGTCTCTGCATAATGCCAGACTGGTTTTTATTGTGGATCTGCTTCCAGATGTGCATGTTGATGATGTACTGTTAACACTGAGGGTAATTATTTCAGACAGTGTGATGTGATTGCTAATTGTGGGTAATGAGTCCAGGTTGGAATTATGGGTGTTATGTATTTGCCTCGGAATAATGGGTAGAGCTCAAAGCTGTTACAATACATGATTTACAGTGCATTAAGAATTGCATTCTGACACTTTTGCATTGCTAGAAATAGTTTCATGCACTTGCGTAGTCACTTTGGAGAAAAGCATTAGCTAAATGATTAATTGATTACAGTCTTTGGGCACAGCATTTCAGGAAAG
LG6	ref-30839	62.117			ACAAACGAACCGAAATCATTGCTCTGAAAGA
LG6	ref-58548	62.377	Chr18	7660146	AGCACAATTATTGCATCTTGGAGCCTATTTTTTTTTTTTTTTTTTTTTAACTGAAACACCATTATTTCAAGTTATATATTAGCAATATTAAGACCTGCTATGACAGACTGTAGCTTGTTGGACAGAATTTATTAGAACAGAAAGGACCTGTTTAATCACCACCATCCACAATAATGGGAGCAATGTACTCGGAATGACGAATTCCAAAGGAGAAGCTGGGAGCTTTGATTCCTGTGAAAGTAACCTGTTGAGAGAGACAAAGGAAACAGTTTTAGTATACTAAAATACAGTAGTTTTAATAGAGAGCAATTCTTAACTAGAAGCTGAATATATATACACTACATTACCATTCAAAAGTTTGGGGTCTGT
LG6	ref-57160	62.994			ACCACAGCAACGAATTTGATGCCAAAATGAC
LG6	ref-35047_28	64.568			AAATGTGCAATTCATAATGCAACGCAACATTCTCAGGGCTGCCACAAGGGGTACTATAGCGGGCATTAGCACCCTGTCTTCTTCTATGCTCAGTTTCCATATTTGGAACAACAGTTTAAAGAGAATTTTCTTAGTGAGATAGGTTAGTAAATGGCATAAGTAAGCAATAAGGTACTCGAGGCTAGTGCTATATCGTGAATAAGTCACAGACGAAGGGGTTGCAGGCG[A/C]TCTGCTTCGGCCCTTCAGCCGTGACTTATTCACGATACAGCACTAGCCTCGAGTACCTTATTGCTTACTTATGCCATTTACTAACCTATCTCACTAAGAAAATTCTCTTTAAACTGTTGTTCCAAATATGGAAACTGAGCATAGAAGAAGACAGGGTGCTAATGCCCGCTATAGTACCCCTTGTGGCAGCCCTGAGAATGTTGC
LG6	ref-38335	65.189			TTTTTGCCATATCGCCCAGCCCTAATCCGTAGTATGTCATAGGGACATAAATGATTCAGAAATGGCACCATTTCTTGAAAATGACCTAGTTGTTGTATTATGTTCAACTGTTTTTTTACTCTTCTTTATGTCAAAACCTCTATTCTGTTATCACTTAGCATTTGTAATACAAACATGTTGCACTTTCATTGTAAAAGCAGTGCTATTGTTCTTCTAAGACGACCCACCACCTGACGTCTGTATCTGCCCTCCATTTCACTGTTATTGACCAGGCAGCTGCTCCTATAGACAGCATTGAATTTAAAATGTTTTTCCATAGAGGCATAACAAAGAAACTCGCTGAGAATCCACAGTCACTGATCTGAATGC
LG6	ref-72600	65.497			ATTTTAAGCTCGAAGCTAATGCATCTCAGCT
LG6	ref-35327	66.206			CGTAGCCGTGCGACATGGCTGCATATATCAG
LG6	ref-23333_29	67.015			TAAGCCGACCCGAGGCTCATGCTAGGGA[A/G]GGA
LG6	ref-17807_30	67.869			TTTACCGCAGCGATTGAGTTGCTTTAACC[C/T]AG
LG6	ref-17807_29	67.884			GTTTAGTTGAGTTGAGTTAAAACTACCCAGCTGGTTGGGTCAAACATTTCACCCAACCATTGGGTTAAAACAACCCAACTGCTGGGTTAAAACAACCCAAACGCTGGGTTAAAGCAACACAATTGCTGTGTTAAAGCAACCCAATCGCTGGTTTAAAACAGCCCAATTGCTGGGTTAAAGCAACTCAATCGCTGCGG[C/T]AAAACATCCCAATCACTGGGTTAAAGCAACCTAATCGCTGGGTTAAAGCAACCCATCCCAACCCAATTGCTGGGTTAAAGCAATGCAATCGCTTTAAAGCAACGCAATCGCTGGGTTAGCAACCCAACTGCTGGGTTAAAGCAACCCAATCGCTGGGTTAAAGCAACACATT
LG6	ref-23448	68.081			GAGAGACAGATTTGTTATAAATTGCAGTTTTCGCATACATGCTAAAAACCCAGTATGGACACTGCTGCAGCAATACAGATTTATAATTTATAATTTTTTAATAGCTCAAGCTGGACCCTTTCAGAGCAATTAATCATTGGGAAAATCATTTCAACTCTCTCTTGCCCGATCTCGTTCCCTTCTCCACAGCTATGTTTCAGGAGAGGTGGACGACAAAGCCGCTTCCTTCACATGAAGGAAAGGTGGAGAATCAGTAAATAAAGAACAAATCTGATCAAACTGAGAATTCAGATATATTTAATAAGACATTTCTTTATGTGCACCTATTTTTTGTATATGTCTTGAGTTTTTTTTTTTTTTTTATAATGATTTTTAATGCTGATTTTTAATACTAATGTCCTTACATCAGTGGTGGTCAGATCGTGACCTTGG
LG6	ref-4327	68.644			ATAGCATTAGCGAGCTCACTGCAGATAACTA

LG6	ref-45426_31	69.144	Chr18	49382184	GAAAGGAAAGAAAAAGAAATGAATGAGACTACATGAAGCAGGAAGATGACAAAAAAGAAATGAAGAGAAATGTGCTGGACAGAATAGGAAAAATATGAAACTGTACAGAAAAGATAAAAGGAAAATAATAGAAAAGAAACTAGACAGGACAAGGACAGGAGATGTACAGAACAAGGAATTGGCGGAACAGGAGAGACAACAATAAAACCTCGAACCCGGTGCTTGAGGAC[A/C]GCATGTGGGATATGATGTATATATAGCGAGACCACCAACATGAACAGAGAAGAGGAAAGATTGATGGCAAGTACAGAAACATGAAAACATAGACTCAGAGAAGGGAATGGATAAGTGACCGATGTGTTGTGAGAACAAAGGAAGAGATCAGAGGAAGAAGTGGAGTGTAGAGGAGGAATGATGGAGGGGGAAAGCAATCAC
LG6	ref-26164_25	70.044			TCAGCCTCGAGAGAGTGCCTGTTCCATATAATCAAGGTAACGTGCAGGTGCCTTTGTTCCTTGATCATATTGAAGAAACCTTGGTTCATGTCCAAAGGCCCTGTGCTAGGAGCATCGTGTCATCGCAAGATAATATCGACAGATGCATCCCGCACAGGGTGGGGTGCAGTCATGGAAGGGCACTCAAATCGGG[G/T]TTTGTGGAGCGAACATCACCTCCTTGGCACATATACTGCCTAGAGATGTTAGCTGTATTGCTAGCCCTGAGGCAGCCTGGAGATGTGGAAATTATGGGTCTGGCCCCTGAGGGGGATCACTTCCTAGATGCAGGTCTCTCTGCTGAGGTAGTAGAGACCATTTTAAACTCTAGAGC
LG6	ref-5576	70.975			TGTAAAGTACTAAAACCTAAGTTAAACTGTGCGGTCACATTAGCGAAATTTCGCATAAGTTTCATTGACAAAAAAATTAGCATAATTAGCATCATTAGCCATTAATCATCAATACTGGGATAGAATAAATATGTGTCCATTTCTCTATGACAGATGCATACGTAAACATATGTAAAGTGCTAAAACATTTGTTAAATGCAGTCACATTAGCGATATTTCGGCGAAGTTTAACATGCAAAAAAGGTTCCATTGTCAATCATTGGCTGTTAATCATCCATTTAGGCTAATGAAATCATGACGGCATAGATTTAATATATTATGTGGCCGTTTCTCTATAACAGATGTATATGTAAAGATATGTAAAGTGCTAAAACTTTCATTTAACTGCAGCATAAAAATGAAGTCACATTCATATTTCGACAAAATTATGC
LG6	ref-20427	72.257			GATGTCAGACCGACACCGGTGCCGCTGCAAT
LG6	ref-20039	74.308	Chr18	35735740	TACATGATTGCTCAAGCAAATTCAACTCTTAAAAAGATACCTAAGGAAGGGGCCCCAGATTTTATCAAATTTTTTAGAAGCATCGATCCTGGAAAAGCAAATCCTTTCGAGCTTGAGGGCGGCAACCATCTCCGCCAACCATTTTATGAAAACTGGGGGGTAAAGTGGACTTCCACTCCATCAAAATCACCCTTTTAGCTACCACCATACCGAGACAATTGCTGAGAAAGTGTCAATGCCAGTGTAGAGTCAGAACACCCAAGCAAAGCTAATTCAAAGTCTGGACTGATATCTTCTCCAAAAACATTTGAATACCACTGAAAAATATTCTTCCAAAACTCAGAAATCTCAGGGCAAAACCAAAAAGTACGAGCATGCGTGCCATCTGAGCTCTTATATCTATCACATAATGGGGAAACTGAAGGGTAAAT
LG6	ref-33957_31	74.481			CTGAGAGTGGCGAGAGAGCTGCAGGCTGCT[C/T]G
LG6	ref-48120	75.801			AATATAGAATATTCTGCAAACGCACCTTTCGCATGTTGCGCCTCCCTTGACTCAAACATCACAAAAGCTCTTACGGTTGCTTGTGAAGACACGTCTCAACAGGTAAATGTAGGCTACTCTCAATAAAAAGTCCAACATTAGAAATACTTTTCGCTGTGTTGCTATGTGTCATGAGTAAGCAACTGAGTAAACTAACACTGAGAGAGAACGCGAGTGTCTTGCTTATTAGAAATAAACCCATTCAATGTAACTGCATTGGCCAAATTTGTTTTTTTGTACCTGAAGTCACCACTATATATATATATATGTGTGTGTGTGTGTGTGTGTGTGTGTGTGTGTATCCACCCCCTTGTTCAGGGACTCATTATTCCACTTCTCTTCATCAAACGTCTGTTAAATTTGTTCAATATATGTATACATTTTTTTATGTT
LG6	ref-59930_4	76.335			GTG[C/T]GCACAGCGAAAATGCTGCCTGACATGTT
LG6	ref-62129	78.888			ATATACAGTATCTGTGTCATCATCTGCAACTGGTCTGTTAGCGTTTATAGTGAAAAGTCTGGCTCTGTGAGACTAGCCAAATCTAACTTTGACCCTAAAGCCTGATCTGATCCTCACCATGACCAGAACAGCATCATGCCAACTCTATCTACTGAACTCTAACCTCAATAGCAAAATGGGCAATGACTTCGCAGACAGCGTTTGCATGCACAGGTACCTCCTAAACCTAAAGACAGGCTTCTGAGGCAGCATAACAATCTAAAATTTTACAACAAAATAGAGAGGCCTTTGGTATTAACTAGGCAAATATTACTACAGTACTCATTTCATAGAAATTAAATTAAAAGTTGAAAAAGTTATTCAAAAACA
LG6	ref-2572_1	80.767			AGAGCCCTCGCTGCTAAAAGGGGGGATCATGGTGCTGAGGAGGTTGGGCAGCATGAAGTAGCGGAGACCACCAGTGGCTGCCCAAGGTGGTGGTGCTCAAGCAATTGAGCAACAGGTGGGATGGAGAGCTTGCTGTGGTACTCCTGGATTGAGGAGGGGTGGCTGCGAT[C/G]CTAGAGTTAGCAGAGGAGTCGCCTCCGTTTGCTGAGGCCGGAGCCTGATGCCATGTGCCATGATAGGGGATGAGCAGGGAACAGGGGAGGAGCTGCCTGTTGTCCAAAACCAGAGGAGCCATGCTGTCCGCCAGGAGGCAGAGGAGTATTAAGCTGTCCACCGGGGAGTGTCCAGTAACATTGCATGGCATCACGAGTGA
LG6	ref-29102_17	83.861			TGGTTGAAATCGAGAG[C/G]TGTGCTGATACTTTT
LG6	ref-15698	86.742			ACTTACGGAACGAACGTGGTGCCAGTAGTCC
LG6	ref-31095	87.993			CGGACGTATACGAGGGGTTTGCCTGACGAGC
LG6	ref-28451	88.277			CCTATTGGGCCGAAAAGGCTGCCTAGGTAGG
LG6	ref-49206_1	93.122			AATCGGGGACTTCACATGCACTGCTGTTTTTTTTCACTGCCCTCGCACACAAAATTGAGAAGATTTTGAATTTGTTTTCCTCTCAATTGATCTACAATGCTTATAATGGCAAATTTTAAGCATCTATGAAACTATTTATTATACCTCAGAAGTGGCATTTATGTTAATAAA[A/G]AAAAGCAAGCACCTTTGTCGTTGCTGCAGCAGAAGCACAACAGGAGATCCATTTGGTGGTTCTTCCAAATATTGTCTTGGACAATATGGGGCCTCAGAGTTACGAAGGTCAGGATCCACTGCTTGTGCAGGTCATCCATTACGACTCTGACCGCCAAACAGTGATGTGTGCGCTGCCGCAGAGGAGAGCAGGTCTGAGT

LG6	ref-55698_26	94.782			AGGGCAGGACTTTATCCATCAGGTTTGCTATTATTGATGAATATTATTTTTCCTTATTCACATGATCTTGGTTATGTAAAGTTGTTCCATATTATTAAATTGTGATGAAGGATGACTGTAATGTGAGCTGGTGTCTTTATATTCTAATGAACAGCTCTCTTGATATATCTCAGATCCCAGCAGGAGGATCGTCTC[C/G]TCTTTCACAGTAAATGATTTATTTGCATTTATAGCTTGTCAGCTTGTAATGCAAATGCTGCCATTTTCACTAGGATTAATTTACAGAATATGATGTTCCAATAAAAATAATTTAGTATGATCTCTTTACAAGTCCTTCAACTGTTACTTTTTAAATGTCCATACTAGAGTTCT
LG6	ref-8883	95.284			TATCATTTAGAAAAAAAATAAAAATGTATATACTTTTTAACCACAAATGCTCGTCTTGCTCTGCGATTTGCCACGCATTACGTAATCATGATGGAAGGGTCACGTGGAAGTACCGATCCAGTGTCTACAAAGCCAAATGCCCTTTACAAAAGAAGGTAAAACAACGATGTCGGACGATTTTGAAGTTGGAGGAGAAAATGAGGAGTACTGCGGTACTTCTGCCTACCTTTCCAGTGTGATTATGTAATACGTGGCACATCACAGAGAAGTGCAAGATGAGCATTTATGGTTAAAAAGTATATTTATTTAGAAAATGACGAATCATTTTGGTAGATAAGACTCTTATTCCTCGTCTGGGATCGTGTAGAGCCAATTGAAGCGGCACTGAAACTGCAATTTGGACCTTCAACCCGTTGAACCCCAGTGAAGTC
LG7	ref-8010	0			CAGGTTCAGGCTGGCTTTAATTATCCATCTCTACAGCACGTCTCCCACCGGCTGCATCAGTGAGGACGTGTTATATTGCACAAAAAAATTAATGATTTTTGCTATACCATAACCTACACTTAACTCAACAAGGCCCCGCCCCTTTATTCTGTGTATGAATTATTTAAATGAGGAATATTGTAATGTGTTCGTTCCTGGAAGAAAACTCAAGACTACATTGGAGGCATTTAAGGGAGTTACCATCCATCCATTATCACACTTCAAACTTACTTGTAGTTGCTGTTGATATCAGACACCCTCCAGGCGTTTTGCATGTCAAAGCCCATCCGCTTCACCTCCACCTCCATCCTGTGCCGCACATGATCACCT
LG7	ref-42014_30	1.757	Chr5	63093423	TTTCAATTTAAAATGACTTTCATTATAAATGTCTCTGTCACTTTTGATCTAATTAATGCAGAATATCTAAAAACCTAACTTTCTTTCATTGTAACACCACTTAGTTTTAAAATGTATATTTGTCTAGAGAAATGTTCCTCTTTATTTCATTCTCCTCTTGTCAAAGCATTTCCACCACACCATCGTCTAATTACACGGTGGGGAAGCGGCGACTGGACTGCTCATTGA[A/G]GCTTGAAAAATGACCTACAATTACCAAGAGCAGCATACTGCAAACAAGCTGGCATAGTGACTTTATTCTGCATGCTTCCCTGCACGAACAAACAAGAAATATCCCATCTGAGATCCCTGTTCCTTTACATTATGTCCCGTTGTCAAATTCAAGAGTGTTATACTCATGTGTTAAGACAGTAAATGCTTTCTCGGAGGGTGAT
LG7	ref-15779	2.82			AAGCTTCAACCGACTGAGTTGCATTTTGTTC
LG7	ref-15212	3.782	Chr21	21999231	AAAACATCTAAACATTTTGACCAGTACGGCTCACACGATGGCAAAAACACTTGTTATTTTGGTGGCGCTGGACAAATTACTGACATAATAATTTTGAAGCATGAATTAAATGGTTTGGGTGAGTAACTACATTTTGCAGACATGCAATAGAGTTTTGATGTATCAGCGAACAGTTGTGACAATTGCACTTACAGTTTAGAGAATGCTAATCGAAAAATGTGCCAAAGCGATTGAGAAAAACTGTAACAATTTGAACTATTGTCATATGCAGTGCAGGCTTCCACGTTTACGTCTGAACGCCAGCTCAGTATTTTGGGTGAACTAACCCTTTAAGAGAATGAATGTCAGATCCAGGGAGAACTAAAACTATTAAAACTTTTTTGTTAACTGAAATAAAACTGAAATAAAATAAAATAAAATATAAATATTA
LG7	ref-27330	4.769			TTTGTCTGGGCGAAGAGCATGCACACTGTTC
LG7	ref-50594_4	5.146			GAACATGTCCCGCCCAAAGTCGTCCGCCTCCTCCAGAACCTCTACGACTGCTCGACAAGCTGTGTACGCGTTCGCAATGAAGTGTCTGAGGAGTTTTCTGTCCGTACCAGTGTGCGGCAAGGTGACGTAGCCTCCCCGCTTTTGCAATCATGAGGAAGAACAAGCAGGGAGTCCAGTATGGCATCGATGGCTATGTGATCTTAT[A/T]TTCACCGACGACAGTGCCATCCTGGCCAATACGGGTACGGAGGCCACCGACGCCCTCTACAACGTTGATTGTTTTGCCAAGCCATACAGATTGAAAATCAACATCGATAAGACCAAAGTTCAGTCAGGACCACTTAAGTCAAAAAATTTACCAGAGACAAACTTTTTAGTCAATTTCTTTATTGGCATATTTTGCTGAAGTTACATTTGGCGGTATGGCTCTGTTCT
LG7	ref-68949	6.021			GAGAAAAACGCGACGTTTGTGCTTGTGTTGG
LG7	ref-41860	6.207			TAAGGCATCGCGAGGGTGTTGCTCAGTGGTT
LG7	ref-48399	7.523			CTACGCCATACGAAAAAAATGCCTGAATGTA
LG7	ref-63045	7.715	Chr9	30213960	CCATCTCTGCTGTCCAAATCCATTTAACTTTGTGTTGCGCCATGATTGCATTTGTTATGCTTCAGTCTTCTGTCAGCTCACTTTCGGATTGGATATTGTTTCGTTTCATGTCATAATCTCCAGAGCGTGCGCATGTTTGTCTTCGGGGTTCCCCCCACACCTCAGGATTTCTGATCGTAAATATTCAACATGTTGAATATTTACAATTTGCGATTGGGGCGCCTCCGACATTCTTCAGAGCAGACACCTCACACCACAGGAAAATCTGATAAGATAATCTGTAGAACCATCAAGATAATCGGGACATTACCTAGGATTGTCGGAAGAGGAGAAATCGGCCCAAAATCAGATCGATTGTCTTGTAGTGTATACAAAACAAAGGAGAACATGTGACAAACAGAACATGGTGCAAAAACAGACATACATGTGAC
LG7	ref-43855_27	7.733			CCTCTCAGAGCGAGCGGTATGCACAT[A/T]CAAAA
LG7	ref-43855_23	8.171			GATCCTGGCACAAATCAGTCCAGGGGACTGGTTTGTATCTGTGGATCTGAAGGACGCATACTTCCATATTCAGATGGCGATGCGCCACAGACACTTCCAAAGTTTCGCTTTCGAGGGCATTTACCAGTATTCAGTGCACCTGTTCAGGCTGGCCTTAACCCCCTCGCACATTCTCGAAGTGCGTAGATGCAGCGCTTTCCCCTCTCAGAGCGAGCGGTATGCG[A/G]ATTCAAAATTATCTGGACGACTGGCTGATTTTAGCCCATTCATGGGATGTGCTTATCAGTCACACAAATTCGCTGTTTTGCCACCTGGAGTACCTAGGGCTGTGTTAATATGCAGAAGAGCATGTTCACCCCGAGCCACTCGGTGGTGATGCGAGCCCGCCTCTCTCAAGAGCGCATAGAGACCATTTCATCAACTGTGTGCCTCTTC
LG7	ref-67543_9	10.365			CACTCTAC[C/T]GCGATACTTCTGCATGACCTTTC

LG7	ref-25601	14.746			CAGATTGTCCTGTACTCTAAAGCATTAAATGACTTTTCACAGTGAAGTAGCTCACCAATACCAACAGCTAAAAAGGCTCCCCACACATTGACACATTTTCCTCCATGCTTTACTGTGGTACTGTAGAGATGCATTTATTATTATATTTCTCACCAAATGTTCGAAGACACCGTTCCGGAGCATTACCATCGTGTTTAACTCGTGTTTCATTGTGATTCAGAACCACTCTGAATCAAACTTCCCATATTCTGCCAAATCTTTATCCTGCCTTTGATATTTGTCTGAGACAACAATGGCTTTTTAAGCTAATTGCCTGACCAATAATTTGTGGTTAAGACAATTAAAAATTAAATTAAAATTACATTTAAA
LG7	ref-22204	15.427			CATGTGTTGACGACTCCATTGCTCTAGATCA
LG7	ref-59120_2	17.1	Chr5	1542210	TTAAACACAGTAAGAGTGTGTGTGTGTGTGTGTGTGTGTTTCTGCGGGCTGGAGAGGAAACTGTACACACATTTATCATTTCAAACCAATCCCATGGTGAGCATATGTCTTTTCATCTGTCCTCTAAAGGATAAAACACACGTCTGGCCTTTTAAACGAGCAGCAAAACACAAAAACTGCTCGATGCAGCACAACTGCGGC[G/T]GAAGGACACGAGAGAGATGCTCCGCAAACACGTCCTCGGTATAATGAGGTTACAGAACGAAAAGAGACAAATTAACACAACTCAAAGTGAGCAGTCCTACACAAATGGAGCGATCGTTCTGACCTGACGACAGGTTTGTTTGTTCTGACCTCGCCTAAAGCAAAGCAAAAACGTGTCATTTCAGAAACAAACAAAAATAGCAATAATATAAACTGCTAGAATACCAAAGA
LG7	ref-14606	18.803			AAGACAAAGAACAATCACTCATTACTCTTAACTAAATAACTTCTCTAACTTTAATAAGGATTAATCTATGTTTCATGGCTGGTAAAAATATTTTTGGCCAGTAAATAAGTTAGTTTTAGGCCAATATTTTTAATTTGAAAAAATTCAAGGAATCCAATTGTTTTTTTTGAAAAAAATTGAAAAAATTAAAGGAATTGCTGCATAGTGTCATGATCCATGTGCTGTGTCTTGTGTTTTGTAGGACTTTTATGTTGAAGTGTAATCTTAGTTCTCTTTGATTCTGTCTTCACTTCCTGTGCTTGGTTCCTGTTTTGCCTTGTACCTTGTGTGTCGCTATAGTTACCCTCATTAGTTACCATAACATCAAATCACCTACACCTGTCCCTCATCAGCCCATTGTTAACATGTCTATTTAAACCTCTGTGCTTTAG
LG7	ref-37725_3	19.737			TT[A/G]TCACATTCGAGTGCTTTGCTGATGGAAAG
LG7	ref-57263_28	21.604			ACCGGTAGATCGAGAGCTTTGCCTTAC[A/G]ACTT
LG7	ref-41714_31	23.89			GCTAGGAAGTCGATCCACATGCAGTAAGAT[A/T]T
LG7	ref-6394	24.834			CCATCCTGAGCGAACAGTTTGCAGCACGAGC
LG7	ref-60688_3	27.228			AG[A/C]GGCAAAGCGAACAAACTGCTCAGGAACGC
LG7	ref-14906	27.886			AGATTGGGGCCGATGAGCGTGCCGATAAAAA
LG7	ref-26436	27.972			GAATAAGCCACGATGGAAATGCATTTACCGG
LG7	ref-30856	28.698			GTTGGTTATACGATCTCTGTGCAATATTTGC
LG7	ref-1019	29.1			AATTCATTCATTATTCATTAATTCTTACCTACTGTCCTGTCCGTACACAGGAGGGCCCTAAATGTCAACCAAAACCTGCAAGTACACATGAATTTGAAATCACATGAAGACAATAAAAAAAGCTTATTAAAAAAAAAAATATATATAGTGGGTTGCAAGAGAAGGGAATTGTAATTTTTGCACTGCACTAGTTCCTAAGCCCACTTTGACTAGACTGTGACTAGTGCTGTGCAAAACTTTCTAAGAAAAACTATCCAAACTACTTACCATACTGCATATGTCGGTTACAACAGGCTTCAAACCACATGGGGAGACTCAAGAACTCCCTGGAGGGGGAAAGAAAAGAGTCTTCANNNNNNNNNNNNNNNN
LG7	ref-19865	30.733			GCCCGTCTCAAGGCCGCTTAGATGGATGCTTGGAAGTCTTAGCAGCGGTGTGAGGGAAGGGCTGCGTTGCCTTTCTGCAGGATACTCAACGTTGCAGCCTCTCCAGAGCAGGTGTAGACACTGCAGGAGGATGCCCTCAACAATGAGCAGACTGGGGGGAAGCCCGCAGCGGCCTGGTGGCAGAGGTGTCGCACCGGTGCAGGATTTGCTTGATGGTCTCTCTCAACTCTGCATCTGTCTCAGCATGACCGAACTCCCCCGAAAGGGGGAAGCTCAGCATCATCTTCTGTAGAGGACATTAGCCCACTCTCCAATGCAGCCATCAAATGCACTTTGTAGAGCAGTTTGTCCGTTTAGGGCTACTGTCGA
LG7	ref-35481_2	35.109			T[C/T]GAGAGTGTCGAAAACAGTGCCATCTGGTGT
LG7	ref-34558	35.259			AGTGGTCGACCGATTATATTGCCAAGGCCAA
LG7	ref-63347	36.018			AAGCTATTACAGCTCTTCAGAAGTCATACGATGGCTTTGCGTCAAAAACTTCATATACCGCATAAAGACGCACGGCAGCAGGTTTGCTCTCAATTACTCTG
LG7	ref-5237_17	36.657			AAGCACAAGACGACTC[A/G]CGTGCTGTTTCCGTG
LG7	ref-5237_23	36.657			AAGCACAAGACGACTCACGTGC[A/T]GTTTCCGTG
LG7	ref-43895_23	37.411			ATTATGGAAACGATCCGGCTGC[A/G]TCTGTGTTC
LG7	ref-1627	38.12			TGCGCACATGCGACCTATGTGCACAAAGTAT
LG7	ref-22264	38.122	Chr5	9173423	GAGGAGCGTGGCGCATTATGCCACATGCAACTCTCCTCCAGACAAGACAGGCTTCCTCTTTAAGAAAGGGGAACGGAACACGGCGTACCATCGCCGCTGGTTTATTCTGAAAGGCAACATGCTGTTCTACTTCGAGGAGAGGGAAAGTCGAGAGCCCATTGGCGTCATCGTCCTGGAAGGCTGTACGGTGGAGCTCTGCGAGTCTGAGTCCGAAGAGTTTGCCTTCGCCGTTAAATTTGACTGCGTCAAAGCGAGGGTGTACAAGATGGCAGCGGAGAGCCAGGCCGCCATGGAATCTTGGGTCAAGGCTCTGTCACGAGCCAGTTTCGA

					CTACATGCGACTGGTTGTGAAGGAGCTAGAGAGGCAGCTGGAGGAGGTAAAAGGAGAGAGATTGTTACAAGCGAAAAACAAGGCGAAGAAACACCAGTCTC
LG7	ref-35481_18	42.16			TTGAGAGTGTCGAAAAC[A/C]GTGCCATCTGGTGT
LG7	ref-52274_5	43.152			AACATATTATAGATACAAATAAGAGTGAATACCATTGTAATATAAATACCATTTGCAAATAATTTAAATTGATTACTTAATAACTATAGACCTGGAACGTTCTTATTTCTTT<br>TTCATTATTCCCGAAATCCCGGACTTGTAATGTATTAAAGATTTATTAACATTATTGTAGTATGAAGCAGGGTGTGGCTGAAAGCCATTGGA[A/G]CTGAACGAGGCCATTG<br>CTAATGAGAGACGAGCGCGACACACCTCGAGAGCAGCTGAACTTTTTTTATGCCGCAGATGAGCACTTCCGCTTCATCCGGTCATGCATATGTGGGGTAAGGCAGCGCAGT<br>TTTATCATATTATATACATTTGTGTGTTGAAATTTGTTATAATGCTACTCTGTGCATTTACACGGCGACTGTTATGAGACACTTGCTGCACACTGCAGTA
LG7	ref-68174	45.513			GGTCTCTTGGGCTGCCATGGTAATAAATCTCCAGGTCATCCACTGCTAGAGCTGCTAGAAATTAAAATAAAATTAAAATCAAAGTAAAATATAGATTCATCTCAGATGAAA<br>GAAGCTTTATTTAGTGTCACTTGTTAAGGAAAATAACACAATTATACTTGCTTATACATAGGGTTAATGCAAACACACCGTAACACACTTAAACCCACTTAGAGCACCTTA<br>GCAAATGCTTAGCAACGCCCTGGCAACCACTCGTACTGTGATGGTGAGCTATGGACAAGCACTACTCTTTTTACTTAAGAAAATGTAAAAAATGTATATGATTTATCAAGA<br>GTATTATATCATTTACAATATTTGTCAAAAGTTTGG
LG7	ref-43025	47.982			CCTGTTTTCTCGACACCCTTGCTGCCTGCCC
LG7	ref-41086	48.26			GACTGCGACCCGAACCAGGTGCATTAAATCA
LG7	ref-5686_30	50.54			AGAAAGCCCGCGAACAGTTTGCTGAAGAC[A/G]AG
LG7	ref-33523	53.247			AGACTAGGGAATGTTGCGACTGACGTCACTGCCATTACACGATAGGCTGAGAGGTGCCGCACGTGATTAAGTTAGCCATTACGCTTTCTCCGATGGTAAGAGATACAGACC<br>CTCTCATCTCCCTTTATTATACAATCTCTGGCTGCAACAGAACTGAGCATGTGACTGCCTATCTGAGTATCAACTATGAGGCGCCGGACTGGACAAAAGCGAAGCGAACGC<br>CGCGAGAGAAACCGAGCATGTCCGAGAGCGGGTGAACCTGCGCGAGAGAGATCGAGTTGTCCGAGAACGAGCGACACTGCGCGAGAGAGACCGAGTGTGCGCGCGAGA<br>GAAACCAAGCGTGTACAAGAGCGGGTGAACCTATAATCATGATAATCAAAACCAAGCAGATGGCTTGTTTGTATTTGGCAGAGAATCCGATTGAAGCAGG
LG7	ref-46918	53.693			CAAACTCTTTGCTCTAGCGGTGCAGCTTCATCTCCCTCTGGTTTTTAATCGAAGTGACTCGAGAGCCGTGCAGTCAAGCAGCAGGTGGGTGGAGGTGCTTCAAGCCGCCTC<br>CGGCCTGCATCAGGATTTAGCTTCATTTCTAGAGCAATTACAGGACATAAAATGCAATGTTCCAGCTCATCAAAGAGGAGTTCATCAAACGGTGATGTACGAGGAAAATG<br>CACATACGTGTGGACACCTGTAAGCTGATCCAATCACTTTCCCTTAGAAACCTCCACAAATTAAAGGCATAATAAGATTAATAATAAGAAGAAATGTTTTTTGAGCACCAA<br>ATCAGCATATTAAAGTGATTTGTAAAAGATCAAGTGACAACTCAAGTGAAACTGGAGTTACATGGGAATAACATACCATGATACAAGTCCATTTCCACC
LG7	ref-57438	53.703			AACTCAATTTAGCTCTGTAGGATTGAGGCGAAATGTGTACTTGTTATTTTAACACTTGCCTTAAAGTGTGTGAGAAGGGTAAAGATCCTGAGATACCCCTCACTTTCTTAGA<br>AAATCACATTTACTTTGGGAAAAAGAAAAACCCATAATGCTTTTTGGTGATCATATATATCAGGGATGCACCGATACCGTTTTTAAGGACCGAGTACGAGTACCAATACTT<br>TTTTTCTAGTACTTGCCGATACCTATGCCTATACATTTGTTAACTTCTCTCTCTCTCTCTCTCTCTCTCTCTCTTTTTTGGTGATGNNNNNNNNNNNNNNNNNNNNNNNNN<br>NNNNNNNNNNNNNNNNNNNNNNNNNNNNNNNNN
LG7	ref-29910	55.184			CAGCTTGAAACGAACCTTCTGCCACTTTCTA
LG7	ref-7312_24	55.709			AGCATGATGGCGACTGAACTGCC[A/G]TTCTGTTC
LG7	ref-15578_18	56.225			TCGTGAAGATCTGAGCACCTCGTAAATTGTTCCAGGGCTGCTGGGACCAGGATGATGTCAGCAGTTGATCCCTCCAGCACCAAGAACAAGATGATAGGTGATAGATGAGA<br>AGTTACAATAACATCAGTTCTGGTTAAAGATGCAGTCATGGGTGGTAGTTGAATTGTACTCACTGCTGGGCATTGTG[A/C]TCGAACTGGACAGAATGGTAATTAATGTCCT<br>TCATCCCCACAGTACAAACAGAGACGGTTCAGAATCTGTCTCTGACACTCCGCATTCGACAGATGGAAAGTGCTGCCAAGGAATTCATCACAGGTCCACTAGCTTCCCAGA<br>TCGAATTCGCCCACTGTAAAACTATGCCAGATAACAAAGA
LG7	ref-14119_5	57.122			TTGTTTCTCTTAGGAGGATGAAAATAATCTGTTATTTAAAATGAGATTAAATTATATATAGAAAGCAATCAAAGAACGCTCCCTTTACAATCGCTGGAGCTGTCAATCAAA<br>CTGCGCGAGTTTATCAGAGTTCAGGACTCGCACCAAAATTCCAGTTTTATTCAATTAATCAC[A/C]TAGTTGCATCGCATTCGCACTGGTAGTTATGATGAAAGCATTCATTA<br>CTGTGCAGAAACTGAGTTGCTATGACGAGATCACTGCTTTCATGCGCTCAACAACATGTCTGTGATTGGCTGCAATGTTCATCACTGCAACCTTTCATGCCACGATCTAAAT<br>ATAATGCTATAATCAACACTTCACACGATTAGTTAAT
LG7	ref-4386	58.282	Chr21	30384484	CTGGTTTTATTCAGGTTACAGATTTTACGTTGCACATTAAGTGGTAGTAAAATGTTTTTAAAGGGTTAGTTCACCCAAAAATGAAAATGTCTGTTATTAATTACTCACCCTC<br>ATGTCGTTCCAAACCCGTAAAACCTTCGTTCATCTTCTGAACACAAATTAAGATATTTTTGATGAAATCCGAGAGGTTTGTGAACCACCCATAGGCAGCAACATTATTGCAC<br>CTAAGGCCCAGAAAGGTAGTAAAGACATTGTTAAAATAGTCCATGTGACTGCAGTGATTCAACCTTAATGTTATGAAGCAATGTTTTTGTGCGCAAAAACAAAACAAAAA<br>TAATGACTTTATTTAAAGCTGCAATCCATAACTTTTTTTGGTTATAATGATCCAATTTTTGAGCAAGTACACAACCAGCCAGTGTTCAAAACTATCT

LG7	ref-25512	59.139	Chr5	6550997	GTAACTGCAGAAGAAAAGGAGTTTAAATTGCTGATTGAAAGTGGCTTTATTTATGGTTATGGATTAGCTTTAAGAGCTGGAGGAGGAGGAGGAGGAGGTGTGTCTTCAGC TGTTTTTGAAGGTTGTGATGGTCTCAGCAGATCGTGTGGAGGCTAGAAGTGAATGGGACATTATTCAATGCAACTCTTCCGCTCTGTGTCACAGCATTAGATAATCACATAT TTAGCCATCTGCATGTTTGAAATGTTGCAGGATCTTTTGTTTTCTTAAACATTTTTTGCTCAATGTGTTTTAATCAGCAGGTGTTTGGGTGATTTTCAGTGGGTGTATGAAGT CAGATTTCCTCAAGTTCACACAGGTGTACATCTA
LG7	ref-69599_16	59.486			AAACGCGGAAGCACAGAGGATAGAGCAAAACAAAACAGGGGGTTAGACTTGGGGTTAGACTTGCAAGAATGGAATATAAACAGTTGTGTATAATATACCGTTATACTGTT ATCCTTCAGACTATGAGATCAGAGCAGCACAGAGTGAGGACCGTTTCTGACTTCAGAGATCCAGGGGCCGCACT[A/G]CAGTCGCTCTCATGCCTGACATACCACTGAGAC TATAATTACAGGCAACATCTGTCATGTTTATCTCAAACATACAGTTATCTACCTGAAATGCAGCTGACCTGTCACAAATCTTCTTGAGTTTTGTCAAAAATACACCGGCTAC GACGTCAGAAGCCTGCTATCATAGATTTGTCATGTGAAAT
LG7	ref-52655_5	61.581			AGAA[A/C]TCTGACGACTTTTATGCACTATGCGCC
LG7	ref-648	61.763	Chr5	22520184	AATAACTCTCAGTGAAGTGTTTACGATCCCAGCGACAGTAAAGTGAATCAGGACCGGGCCCGACCCCTCAGGTCCGCAAGGATACTCTCCATGGTCGACATAAAAATCCG GTTATGCTCCTGGTTCTACTTGAGGAAGTGTGACGATTAGACGGGAATAGTTTTCAGTCTAGTTTTTCCCCAAGCTCTTCGTGTAAATCAGTAACTGTCCCCGCTCTGTTTGT GGTAATTTCCCTGTATTCACTTCCGGCTTCTCTAAACAACTGGAGTGATGTCACAGTCATGCGCTCAGGTCAGCGATCGCGATGATGTCACGACTCTGTCAAAACAGCGAG CGAGATATATATATACACACACACACACACACACA
LG7	ref-6736	62.888			ATCAGCTGCAGAATTAATTTGAGAAACAATGTTTTTAAGCTGTCTTGGTGATTCGTGCTTTGGCCATGAAAACTTCTCACCATTAAATTTAAATAGGAGAAATGCAATTAGA CAAAGCTTTTAGCATTCCATTGTGAAAGAGTACAGACATTGCTTTTGCACAAGAAACACAGAAAACTCTTTCATTTCAATATTAGAGTAAAGCTCTCACGCCTGTAATGCA GGAAAAGTGATGACTATGTTTTTTCCGAAGGCTTTTCTGCTGTTTAAGTAAGAGTTAGTAAAAGAAGGATGTTAAAGTCACAGAAAAATATGAAAATGTTCCAATGGTGTG TGTTTAGTCTGTGATTCAAAAGCTTAGATGCTTTTGTTGATTGTAGACATGATTTATTCAGTAATATGATTATGAAAGCTGAATCTGTCCCCAAACA
LG7	ref-7086_32	63.048			CGTGCACACACGAAGCTGCTGCTCATAGAGC[A/G]
LG7	ref-30769	63.076			GCCAGATAAACGACCTCAGTGCTCAAAGAGC
LG7	ref-7086_3	63.152			TGCCTCGGGTCTTCCGGTCTCCAGCTCTGCCAAGGCACGAGGATCCCCGGCTCCGTCTCCGGTCACTGATCGCGGTCACTAAAGCTTATCATTTACATCTGTTTTTAAGCAT TTCGGTTTAAAAATAATTGATTTTAAACTGCTAAAGCGCAGTAAGCAGCAAAAGAGAACTCAATTCGCTATTTGCGCGCACTGACAGACG[A/T]GCACACACGAAGCTGCT GCTCATAGAGCATGTGAGTACAGAACCAAGTTCTTTTTGAAGCCTAATATGGCTTGAATGGTTAAATATACATGTGTCAAAACACCGTCTTTTCAAGCATCCTTGTAAACAC AGCCATTTATGTCTTAAGTGAACATAAACAGTTAAGAAAACGCACAAGTATATTGGATCAATGCATTTGGTCTTAAAGCAACAGTAGCCTAATAAACCTC
LG7	ref-8960_23	63.513			TTATATGGTCGTTGTCATTATTCACTTTATGGGGGTAATCTTTGCAGATGTAATGTCTAAATATTTAAATTAAAAACGTATGAACTGTTCAAGTTAAATAAGAATAATAAAG CGTATGCAATAGTAATAATAAATGTATATAAACGTATATATATAATAAAGGTTTAAAACTAATGTGTGCATAAAATTCG[A/T]CCTGCGCTTGTCTCATATCTCGCGATGGT TGTTGAAAAGTACGAGACTGAACGGCGTATAAATCCGCTATTTCAGTTTCAAGTGCGTCATTAGAGATATCATGGCGGTTCTACTGGTGAAAAAGTGCGCGTCTCCGCTAT TTCTCAAAACGCTTTCAGCTCCACAATTGAGGATGCCA
LG7	ref-56665	63.756	Chr5	32194248	TAGCACAGACACCTTTTCCTTAATATGAGCCTATTCAGAACAAAACAAACAAAAGCGTAACTTTTACGCTCATTAAGCCGACATCAGTTTAACCTTGTTGAATCATTCAGTT ATTTTAAAGACGTGTCCGTCGCACTACAAACCAAGCTACAACGTATTTTAACGGTTTTAAATGCCTCGTCGTAATGTAGTACAAACATACCGCGCGACCGAATTACCTGCG TTTTAGCGACATTTAAAAATACGCGAAACGGCTATATTCGTCGAAAACAGCCAAGACAACGACCTGATAATAGCCTATGTATGTTGGACGTTGATGTGTCGGGCGTACTGC GGTGCAGTATCTCAGATTGAGAGGAGTCGAGAGGTTTCGGCTACAGATGAGAGAGAGAGAGAGACCGGAGAGAGGACAGGGAGGGGAGGGGTGGAGG
LG7	ref-71081	63.923			TGACTGAAGACGAGAGCAATGCTGCTGAAAT
LG7	ref-26515	64.834			AAGGATCATGTGACACTGAAACTTCTCTTTGTAAACAACAACAGAATTATAAACGATTCTAATGACGAATGTTGCGTTCCCAAATGCAAATTTAAACATCACAAACAAAGT AGCATTTACAGTGAATCGAAAATGAAAACAGCAAGCATGCAGCGCGTCAGACTATTATTTATATAACTGCATCACAGCCGTTGCGAATTCTATATTAAATTCTCACCACCA TTCAACCGGGCGAGTAACACGTTTCAGCACTGATTCGACTCGCTTTTGGCGCGTTGAACCATTTGAACTCTTTTAGGGACTGTTCATATGTCGCGTCTAAAAACGCATAGAA AACGCGTCTGCGCCGCTTTCTCCTTTTCCAAAGCA
LG7	ref-26731_15	65.665	Chr5	28841318	AGGGCACGCTTACTCCATAATTGTAGAGAGGCTCATAGCTGTGCAGATTCAAACACAAACTACACAGCTGCATCTTCCCTGTCCTTTTATTTTCCTCATCCAAACACAGAGA CATGAATGAAACATGCATGACAGACAGTGGTAATCCATGAAGCATCACAAATGTAAACTTATCCTGGGGCTAACATGCGCTTTCTAGACATGAGGGAGCGACG[A/G]TGTT GCTTGGAACAGGTTTGTATACTAAAAATGCATCTGCGTGTCTCCTTTTCCTGTAATTATTACTTTATCAGAATGTCTAGTGGCTGCTTTAAGCCTCAGTAGAAGCCATAAGA ATTATTCTTTTAAATTGGCCACACTCTAGATTAAATAAGTGTTGTTTTAGAACCTGTATTGACTCATCAGGGGAGAAAGTAAAGGGCTTTTCCAACCACT
LG7	ref-13754_32	66.577			GACCACTCTGCGATACTTTTGCATCTTTTTT[A/T]

LG7	ref-30687_14	66.609			TGTGGTGGAACGA[A/G]AGATTTGCATCATGGATG
LG7	ref-2174	67.216			CAGCCAGCTCCGATCTTTTTGCTCTTTACTA
LG7	ref-25847_9	67.479	Chr5	17767734	ATAATGCCACAGTGACTCACACTTCTAGTCTGGTTGAAGAGATACAGAAAGACAGCAATTGAAAATGATATCCTTACAAATAGGCCCCTGCTGCAGTAAACATTTGGTGTC CTGTCCATTAAAGGAACCGTTTTTTTTTAGCTCAAGTATCTCAGATAATGAGTTCTAGATTGGGCCTTAGAGCAAGTAAACCGGCTGTATCTGTATC[C/T]ACGAAGGCCGT GCGAGCGATACAGCAGTTATCTTCAGGAGGTCTGTGATGAATCAGAGCCGGTCTATGTGTGTAGTGCGGAAGGCATTGCCGACATTCATTCTGCGTGCAACAAGAACAGC CACAGTCTAGCTGCTGGAACATGCCCACTTCACAATCCATTAAATCACCCCAGCCCTTCTGAGGAAATAGCAAATGAGAAAAGTACAAAAAAGGAGGAGAAA
LG7	ref-14407	67.911			AAGAGTAATCCGAGCCAAATGCTATGGTCCT
LG7	ref-35033	68.308	Chr5	32645817	AAACAGTCAAATACACGCAGAAATATATGCATATATGTATATGTGTGTTATTTTTTTTCTCATCTGTCCTGCCTTTATCCTCTCCTTTTGCAGTCGCAATCACCAGGATCCTC TTGCTGTGGCCTATCACTTGATCATAGACAACCGCCGCATTATGAGTGAAGCTAAAGACTTCTATTTAGCCTCCAGCCCCCCAGACAGCTTTCTAGACGATCTGCCTGCTCA TCACTCTGCTAAGATTCACCCTGAAAGAGTGCCATTCCTAGTGACCGAGACTCAACCGCGTCCTCGACACACACTGGATGAGCTTAACCCCCAAAAGTCCAAGCATCTCGG TGTTCGCCGGGCCAAGTGGCACCTGGGAATCCGTAGCCAGAGCAGACCGAATGATATCATGAGTGAGGTCTGTCGTGCTATGAAGCAGCTGGACT
LG7	ref-26270_24	68.542			GATGTAAACACGAAAGCACTGCA[C/T]TGCGTCAA
LG7	ref-30426_14	69.29	Chr5	20437083	CTAGCTGTTTATGCATGTTCTTTTTCTGTCATCTGCCTTTGGCAATAATGCATCGCCAAACTCCAGTGTCACGTTTGACTGACCTAGTGAGCAGTGTGCGCAGACCTACAGCT CCCCAGACAGACAGTGAGGCATCAGCAGCCAGCAGACCCCTTACTGCCCCAGTATCTGCCCCATCACACCCCTCCCCTTCCCCTGCCCAACCATCCTCGAG[A/G]CCCCTGC CGTCAGCACATTGTGAGTATGTGGTGTCAGTGTCTGTGCTGGCATTACTGGCACAGCAGCACGAGTTTTTTTTATTTCCTGGGAGTGTAAAAAGGCAGCAGTGATGCTAGT GGACTAACATGCACACAGTGTGGATTTCTCTGGTCATTTGATCACATCACCGTGTACACCTGGGCAATAACATGGGTCTCAAATGCGTCATCTGTGAG
LG7	ref-37020	69.516			ATTTTCTGGCCGAAGACGTTGCATTGGCCAG
LG7	ref-29269	70.55			GCAGGAGACACGAGAAAAGTGCAGAAAAGTC
LG7	ref-17810_3	71.017			GTGATGGCTGGTTGCAGGCCATTACAAGAATGTGGACTTAAAATGAGACTCCAAAGACCTCCACTTACCTGCTGATTTAGAGTTTTGTAGCCACAAAGTGTGTTCACAACT TCTCCGACCTGATAAAATTAACACACAGGACTCATTAGTAATTTGCATATGAAGTTTTTGAAAACCTTCTCAGTTGAGATGTGACATTAGT[C/G]AGCTTGACGATGTTGGT GCAGAAGAGTATTGGTGAACTGCATGTTGTGTTTTGATTACCTTTGACCCTTAAAATAAATTTTATATTATAAAATTCCATATGGAATTCCAGCACACACTTATAACCATGT TGATGTAACCTTTAAAGCAGAGGGTCATTGCAATGATGGAATCCAATAGGACTGACGAGCAGGAAGCCCATCTAATCAGATTACAAATAGACTGCAAATC
LG7	ref-18307	71.355			ATTCAATAATCGAGCTTTGTGCGTTGTTACT
LG7	ref-57745	71.56	Chr5	25431162	AGGTTGTGATAGAGGGTCCGAAATCTCTTCCTGGTGTAATTGCAGCGATAGGCTCCACCCATGAGGTATTCAATTACCAATCCGATGTCAATCAGGCTGATGCGGTAGTCA GGGGGCAGGTTACCCTGTAAACAAAAAGGTTATTTATGGCAACACTGAACCCGTGCCAACAGTTATGGCTATGAAAATGACAAACGGTAAAGTCAGACACGAACCACGTG CTCAACTACATACACACCCATCCACTCTCACTCCACAAACACACTGACATATTGCTAGTAGGAATCAAGATAATTGAAGAAGATTGTCTGATTCACCTTGAGGTAGATCCA ACTGAACCCTGGATATTCTTGCTTGGAAAGAACACACAAGGTCAGTGAGACAGAATCATATTAAAGGGACAGACATCCCACTGAGACCAAGCAAGTCTA
LG7	ref-18990_27	72.213	Chr5	21103824	AGCTCCTGCTGCCACACCCCCTTTCTCACGAGCGCTGAATGGTACGTCCCGGTGATGCCGAACGTGTCAGACAGTAAGAAATCCGTGAAGACTTGAATTTGAATCCTAACC GTTCAGAATACGACGCTCCCTCCGCGTGCGAGTGTGCTTGTATATGAGCTGCCGCATCCCCTTTCACTCAGCATCCGCTCTCGCGCGTTTCCCAGTAACCGAGCACCGTGCT TCT[A/G]CCGTCTCACCGGAGTCACCGCGTCTCCGACAGAGGCGCTCTGCTTTCTGAAGGATAAACGCCTGGATTCGGTGCCGGACCCTTTTCCCACATATCTACAAGAGAT TAAATTATCTATCCGGGGATTTTACACTGAATCATGCTTAGCATTGACCAAGGTAAGCAGCAGAATCCCGGTAAACATAACCGGTTCTCCATAACTGCGT
LG7	ref-46773_30	72.783			ACTGGTGTGACGACCACGTTGCCCAACTG[A/T]TA
LG7	ref-68362_8	72.988	Chr5	37799153	GGGGAGGGGGGTTTCAGGACACTTTAAAATTCCCTAAAAGTTTCAGTTTTTTTCATGACTGTAGAAGACTCTGATGCAAGTTTGATGTAAGTTTCATATATGTGTTTGATTA ACAGAAATACAATATATGCTCTGGTGCATCTCTGTCGCTGCTTTAGACACATGTATACACACCT[C/T]AAGCACTCCTTTCGTAAGGCGAGTGCTAGTGGTCCAGCCTGGTA CTCCTCTCCGTTCATAACTGATGGAACTCTTTCCTCATCTTCAACCAGCACTGCCAGCTCACTGTCACGACTGCCCAGCATGCTCCTGTCATTGATGTTGGCTGACCCTGAAT AACAACAGGAGAGGAAGGACATTTTGAAGAACTTTA
LG7	ref-64250_23	73.619			AGGCTTTTCATGGAGTCCCACTGGCCATATGTGACCTCGCCGCCCATCAAACACATGTGCCTCCATTGTTTTTCTCAACAGCATGGTGTGAGGTGTTAGGGGAGGCTGCCTG TTTTATAATCTATGATATTTTGTGAGGAACTTGGTGTAATCTGAATGTAGACGGGCAACGTAAAAGGGCACAGCACGTGTGTATGCCATGAACTAAAGCGACAAAACTGC[A/C]ACCTTACCATAATCCAATTCACAACAATCCTGGAGATGAGTTGTTTCTGCTGGTTGCTTGTTGAAATGCAATTAGCTTGTCACCAGACAATCCCCTTAAAACCTGGTGC AAGTGACCCAATGCAACTGAATTTACAGCCATGCCCTCAAAAAGAGCAGACGAAGAAGAAGAAAAAAATGCTAGTATACTTCACAATTAATTACAAAGAA

LG7	ref-51424	74.033			TTAAAGGATACGATCCTCGTGCTTCGTTCTG
LG7	ref-43796_27	74.192			TATATAGATTCGAGGCCGGTGCAACA[C/T]GCAGC
LG7	ref-43796_26	74.232			TATATAGATTCGAGGCCGGTGCAAC[A/G]CGCAGC
LG7	ref-31115	74.349			ATTAAACATTAATATAATGTTATTCTAAATGAATAATGATGAATATGATCTGGTTAGTTAAAATGATTTGAAATGAAATCAATATTCAGGGATTGACTTGATTGGTAGTATA<br>GAACCTATTGTGTGTATTGTTTCTGTCTACCCTAGTATACAAACAATGTATTAAATGTTTTCTAATCACACCATACATGAATGTGTAGACTGGCAAGAGGAAACATGTGCAA<br>ACCATAAACTTTCACTCTATGGTGATTAAACTTGCAATTTATCCACAAAGCGTGTGGGGCCTGGTCCGTACGGATCACGGTTCAACGACTATCCGTTCAACCCCTAAATAAT<br>CTTGTAAATGTAAAAATGAAAATATTCTAATAAAATCTGGTCTTCTGCTGCATTTCTACAAATTAACTTCCAGAAAAAGAGTAACTCCGATTGAT
LG7	ref-51011	74.531			CAAATGGGTCCGATACCTTTGCTGGTCTGAG
LG7	ref-50790	74.538			CACAGTCCCTCGACCGACTTGCTGCTAGAAC
LG7	ref-45127	74.582			TTTAAGCTTACGAACCTGTTGCTATGGCAGC
LG7	ref-13409_1	74.654	Chr5	55539088	CCTCAGTGGCTGAAAGGCTGTAGATGGAGGTGTTTTGACAGAGATTGAGGGAGCTGTGAAAGCCCTGGAACATTTGTGTGGATGTCTGTGGAGATTGAGAGCAGATAATA<br>GCCTCCGTGTTGACATTCCCTCTCTAACTGTGACCAATTAATGATGGCTGGCTAAAAGAGCATTTCATTAGTTTTTGCCCTTGGCTGTTG[A/C]GCATGCTTGCGAGGGCTCT<br>GCTGAAAACAGATTAGGACATTAACTCATGAAGGGCTGAAGGACTTGTGGAGTCATGCCTTCAGGCTCTGCCAGGCTTCAAAGAGCATGGCGCTATCGTATCACTTGCTCC<br>ACTCTATCATCCGCCGGCCTGCCAAACGCATTCATCACATGGCTGCTTTAAAGGTACAGACCATTGCTTTCACCTGGGCTTCCTTTTTATGCATGGAGGAG
LG7	ref-25895_17	74.702			TTGAAGGCTGTTTTGTAGCGTAACTTTTCTGGACTGATGTTGATGTGCTTACATTTAAACCTTCTAAATCCTTAACCCATAGAAACTGTCTGTATGACAGACATTCATACTTA<br>GAATCTCTAGAGAGGAACCCGTCAAATGAGATTCTACAGATGGTTTTATTTGATGTATCTAAAAGTGCATTT[A/G]CATCGGACTTGTACAAACTAAATATACGCATAATAC<br>GCAATCCTTCACTGTTTTATGCTGGTTTGGTGCTTGTCGACAAGTATAGTGTATATGCATTTATCATTTACCAAGTGCTTTTACCCAAAGTAACTTTTTAAATGAGGAACATC<br>ACAAGCAATCTACCATACAAGAACCAACAATATTT
LG7	ref-38289	74.79			AAAATGAACAACAGAAAAACAGAAATAACTAGCATTCATGCAATGCTCAAGTCGCACATACAATACAGACAGTTCAACAGGGAGCGCGCAGCTCTTAAAGAGGCCACAC<br>CATATTTCTGCTCGTTACACTGCCTTTATTAGAGAGAAGAATATGTTCAGCCCCCTCTCTCCGAAGCTTAGAATATTCAGTGTTGATTACTACCGAAGCTTCGAAGCTTGTG<br>CAAATCTAGTAATATGTTTTCCTAATATTCCAATTCAATGATTCTTGATTTTTTTCCCCCTGTACTTCAAGAAAATGGAGAATGCCAGTAGTGCCAGATAAAACCAGAGTGC<br>CTGCTTTGACGGAAATGCATATTTTTGACAGACATTGCATGCATTGTGCAGTGTCAAAATAAAGGTAAAAATAAACAGATTTTTTTATGCATTGCAT
LG7	ref-64250_26	74.841			TGAACTAAAGCGACAAAACTGCAAC[C/T]TTACCA
LG7	ref-32462	75.025			GATTCTGGATCGATTCAGATGCGCGATGACG
LG7	ref-380	75.107			ATTTTGGCTACGAGTGAAATGCAGGGCAGTT
LG7	ref-40095	75.422			GGGATACACACGATTTATGTGCTGTTTGCTG
LG7	ref-68130	75.695			TTGCCTAACAAGTGTTTAATAATAATAAAATAATTTACACTCTGTTATTATTGCATCCTGTTATTTACAACAATACTGAGGTGCAAATAGTATGTATCTGCCAGTATAAAAT<br>TTAAAAAGCATCTAATTACTATTTACACTTATTGCAAAGAACTGCTACTTTTAGCCAACATGATTGAGCAATGGAATCGCTAAACACAATTCTACCAGTTAAGAAGTCCAC<br>ATTAGATAGTACACTTTGATTCAACATGCAACAACTAGATGCTGATGCAGACACGTTCCTGACAGGCTGGATGTACACAACAATAGTGCAGACACATGATTAAGTAAAAC<br>GTCTTGTTTCCTCCTTCAATGAAAATACCCTCAGCG
LG7	ref-56080	75.906			CAGAATCTGCCGAAAGCTCTGCAGATGTCCA
LG7	ref-61476_1	76.084			[C/G]TCTTTACTCCGAGCGTGGTGCAAGGGAAAGA
LG7	ref-47455	76.25			ACCTCCCTTCCGACCCCTCTGCCGCCTCTCC
LG7	ref-22806	76.74			TCGAGTTCTCACTCTCTGGTCGCATTCAGGTGCAGAAGTAACCCTCAAACTGTCCTGGAGGAATATCCTGCTCTTCTCACGTCATGTAGACCTCGCGCACAGCACGGCGCGC<br>GCATGTTCGACCGTGTTAACAGACAGATAAATCAACGCTTGCGCGTAAAATCAGAGTGGTGGACCGCGCAGCGCCCTCGCGCCTCACCGGGTCAGCGTCACAGGTTAATT<br>GAATCTGAAAAAAAGCTGAGAGCTCTGCTTTGTCTATGGCAAGCCTTTTTGGCATTTATTCCTTAAAATAGATTTATGTGTTACTAATGCTTATTTTTTATTTACTAGTGAGT<br>TATCCAATGGCTATATTTCAATTTCGCTATTGCT
LG7	ref-4842	77.104			CATCCAACATCGAACACGCTGCAGCTCCTGA

LG7	ref-26695_30	77.364	Chr20	6217262	AGTGTTTCTGTGGGAATTTTCCACAGAACGTGTTTTGCTCTAGAGTCCACTCTATCCGACGCTTGGCATTGTACTTGTGATTTGAGGCTTGCATGCAGCTGCTTGGCCATGGAAACCCATTCCATGAAGCTCCCGCTGCACAGATTTTGTGCTAATTTCAATGCCTGTGGTAGTTTGGAACTCTTCAGCTATGGAATCAGCAGAGGGATCCGACTTTTATGCACCATGCG[A/G]CTCAGCAGTCTTCCGCTTAATGGCTGAGTTGCTGCTGTTCCTAAACGCTTCAGCTTTCTAATAATACCACTTACTCTTGACCTTGGTATATCTAGCAGGGATGAAATTTCATGAACTGACTTATTACAAAGGTGGCATCCTAACACAGTATCATGCTTGAATTCAATCTTCAGAATGACCCTTTTTTCACAAATGTTTGTA
LG7	ref-53362_24	77.718			ATTAGTCTAGCAGAAAAAAAGTCTACACACTTCATCTTTGAAGAAAAAACCTCTGAAAAACTCTACTCCAACAGTCACAAAAAACAAAACAAAACAAAAAAATAATGGTACAAAAGGTGTCAATGGGGTGGTACCCTTTCAAAAGGCCATTTAGGTATAGGTCCAAATAAAGCACAACTGCAAACCTTTCGA[A/G]AAGGGTATCGCCCAAGTGACAGTTTATTTTTTATTTTTTTTATTTTTTTTTAAGTGATTAAATGAAGAATTTGCTAATGTCTATCAAACACTGAACAGTCATAACATTACCAACATTCACTGGAAGGAAAACACTCTCACACCCACACAAACAAACAAACACAGATGCTGTTTCAT
LG7	ref-53362_31	77.718			ATACCCTTTTCGAAAGGTTTGCAGTTGTGC[C/T]T
LG7	ref-38960_5	78.318			CAGAGCAGAGAAAGGAGGAGCAGGAGGATGACCCAGAGCACAGACATGAAGAAGGTCCACAGAGGAGCCAATGGAGGGAGGAGCTATGGTGAAGCCTTGGTAGAGAGTCTGGACGTAACCAGTGAGATGACTGACTGAAGTGGAGATGGTGGAGGTAGAGACCCAGGCAGAGCCGAAGAGCCAGGGTGGAGCCAAAGGCATCGA[A/G]GGCCACGATAAAGCTGCAGGTGGGCGTGACCAGGGTGCAGGCATGGGCTTGGCAGACTGGGACAATGGTGGAGCTGGAGGAAAGGAGTCAGAAGGGTGGAGTCTCAAGTCGCAGCTGCTGACTCAGAAGTCTGAGGCAGAGCCAGAGCTACGATGGTCCAAGGTGGAGCCAGAGGGACGAGGGAGCCCAGCAGACCTGAGGGATGACAGTCCATGGTGGAGCTGAGG
LG7	ref-35602	79.024			TAAAAAGTTAATGCTCTATCGCATTATAAAATAGCATACCACCTACATTAAACCCAACCATAAACCTAATCAACAGTGTTAACAAAAGCAAGCGTGAGATAAAAACACATTTGCTGAATGTTAGCTTGCCTCTACGAGCTCTTTTATCGTGAATTATGTTTCACAGGACTGAGCTTTCCGCATCACAATCGCAATTCCGTACCAAGCAGGGTTGCCAGGTCTGTGCAACTAAAGTAGCCCAAAAATAGCCCAATGTGATTTTCTCCGTCGGGCACCAAATTCTCCACGGCAACAACTTAAAAGTAGCCCAAACACTGGTACCAAGTGAGCTACCGAGCAACTTTACATGTCATACATATGGAGCTGTTT
LG7	ref-63676_2	79.502			A[A/T]AATCAGCCCGACGCACTTGCCAGTGCCTTG
LG7	ref-54327	79.51	Chr5	37912406	CAAAGTCCCCAGTGGAAATTATGCCCAGACTGTTGTTGTTACTGCACCTTGTATTCATGATGCTGACACGTGAGTTAAGGAACAATAATTGCTCACAAACTGGATACTGAAACAACAAGCCTAGAAGGTCAGGATAAAGAGAAAGTGAATGAATGAATAAAAACATTACCACATCAGACTGTGATAGCAGATATCCAATTGGTCGTATCATGAGTGGTCTGCAGGACAAGAAGACATTAAATCCATGTGGGTTGCAACAGTTTCTTTCTCTCTCTCTCTCTCTCTCTCTCTCTCACACACACACACACTTTTACTCACACACATGATATGTTCATCATATAAAATTTATTTCATGACGATATCAATGCAAATATAGCACTACAGTGATTCCAAGGTACACCGTATCTTACCATACCAATATATTATGGCAC
LG7	ref-18145_8	80.336	Chr5	40383653	CATGCTTTTTATGGTTCTTTTTTTAATTTCAGAGATGGCCGTCTGAAGGCAGGCAATGAGTTACTAATGATAAACGGTCACTCCCTGGTTGGTCTCTCACATCAGGAAGCTGTAGCTATCCTGCGCTCTACTGCTGGACTCGTGCAGCTGGTGGTGGCCAGCAGGGTGAGTTTGAG[G/T]TTGCAAACAAATCGGCCTCATACCCTATCAATAATTGTGTGTCAAGTGTCTTCACACACAAATCATGAGCTGTTCTTGATGTGTTCTTCATATAAAGTGTGTCAGCTGTTCAAGCACATAAAGATGTCACAGGCAGATGCTTGTAGCAGCTAAAAAATTGAAGAGTGAAGCGCTGATGGGCTCGCCGCTCGGC
LG7	ref-60454	80.902	Chr5	43192688	TAAACATATTACCAGTAGATTTAAAGCATTCTACCATTTCTCCGTAGGATTACCAGGAATATTCTACATGGAAAGATTTCTACAGGCCCACCTTCTTGGAGGTGCTCGAAGAATTTCCCTCTCTAAAAGTCCCCACCGCCTTCCTTCTCAGCCAGATTCCTCTGCTGAAACCCCGTCTGTACTCTGTCAGCTCCTCACTGGACCGTCATCCAAACGAGCTGCACCTCACGGTGTCTGTGGTGGAGTACCACACCCAGGGTATGATACTAAACATATAACAAACATACAAAATAAAAACATTGGGTAATTGTTTTATAATCAAACACCATGTTTCACTAGGCATGAGGATAACGTTCTGTTCTGTTTTATCGTAGGAGGAAAGGGCCCAATGCATTTTGGCACCTGTAGCACATGGCTTAACACCATTAAAA
LG7	ref-55812_7	80.985			CATCAT[C/T]GCTCGACGACATTGCCCGTCAACAT
LG7	ref-55197	81.853			GCAGGAGGCCAGGCAAAGCTCACTCTCAGCAGCAGGAGTATGGGCGGGGCTCCAATCCATCCCCTCGTACTCCACAAGCACACCAACTGGGATAAGCGATGTTGCTGGCTCATGCACCTGGTCAGACTTTCCGTTGGGCTCAGGCTCCGGGGCAACGATTGGCTCAGGCGTCCACTTTGGCGATGACATCGCGGCAGGCTCAGGCTTTGTGTCTGCTGTGGATCCTGGCATGTGCTCCATGCAGGGTGTGAGAGGTGGCTGGCAGGGCTCTGGGTCCGGAGTGCGGCTGCTGATGTTGTCCGCAGACAGTGAATGCCGATCCGTTGTTCACCAGTACCCACTCTAGAAATGCTGCGAAATCCCTGCGAG
LG7	ref-34723	82.038			TGTTCGGCCATCTCTTCACTTGCCTTATAGTTCTCACTCTTAGTTGGTGATGTTGCTGAACATTTTCCCTTCAGGATCAGTTGATTGTCTTTCTCTCTATTGATCCAATAGTTTTGCACTCCCCTGGGTTTGATTAGCAAAGTGAGAGGGGACAGATGGTACCACTGTGATTTTTTTCCAGAGCACTCACTGCAGATAGAACTAAGGATGCAAATAAAATGCTTTGTCAGACCAGTCTTTAAAACAACAGCCCTAAGTTTGTTGATGCTTGTTTTCGAATGCACGTCCTCAAATTGCTAAATATGAGAATACATACAATAACCAGTGGTCCAAAGTTTGTTGCTATTGTTGTTGCTTTCTGGCATTTTTTTAATTTATAAATCCTGAAATCAGGATTTCATTCACTCAAAGGTAACATTAGTATAAACATT
LG7	ref-43198	82.112			GCCGTAAAAACGAGTCCCATGCAAGCTCCAG

LG7	ref-65771_10	82.75			AGACCCACCCTGCTTCACACCACGTTTTTTTTTTCTTTTCACTTTACATTTAAATATTTGATTACAGTTTAGAACGTCTGATAACCAGAAGCTAAATCTATAAACAAGTGACATTTTCAAGGGCCTGCAGAAACTCTTAACAGAACAAACATATTTAAGCAATCTTAAGCTATATTC[A/G]GCATACAGCTCGCTTTCAGGGTAAATTTTGAGTTGTTATGCATGACTCTGCATGAATCTAGTTTTAAACTTTGAATAAACAATGGGTAGGAAACCACCAAAGAACTAAAAAAGGTCAAGAAAAAGAAACTCTTCTTGTCACTGTTATAGATCATTCTGAGATGGCCGATGTTTTGTCTTGGATGATGTTCA
LG7	ref-31524	83.337			TGCAATACGCTATCGTTTGCGCACGTGAAAAATGCGAAGGCGCCCTCTGGTGGCCGATATCTTTTGAATTTCTCATAGACGTCTAGGGCTGTGATTCAAACAGTCCCAGTGAGTTTCGTTCCGATCGGCCTCCGTTAACCTTGTTTGATAGCTGCTCAGAATTCATTGGCCGATGGCAAACATGTTTTTCGAGATACGTCAGTGTCCTCATAGACAATCATGGCACCTCGGACAAAGACACTGCATACCAATTTTCAAGTCGATCGGACTAACTTTGAGCTTGTTTTTGATAATTATTGACAATCAGACTCCAGAGAATCTTGCTGCACTGATTTGGCTCCGACCGGGCCAAAAACCTATAGGACTAGT
LG7	ref-247_15	83.987			ATGTTTACTTCTCACCAGATGCTATGTTAAATATCCTATTTTTTTTTTTCAGGTTTAGTTTTTTTAAGTAATTTTAGTACTTCAATTTAAACTTTTATTTCAGTTAGTTACCAAGGCAAGAATTTTTAAGTTTAAACAACAGCAGATCAGACGATAACAGAGGAACGTATTTTTAAAGACTTGCGCTAAGCTAACAGCTGTAACGGCTAACGAC[A/T]TTGGTGCTGTCTGTAGTCAAAGGAGTTTTTCAGCCATATCAGAGATGTGACTCCATCAGCTTGGGAATAAATGCTGCTCTGCACACTAATGTGACTGACAGTAGAAATATGAGGAGGACGTGCTTTTGGATATGACAGATATTCTTGACATTGCTACAACCAAGCAAGTGAAAATTACTTTTGTATCACAATCCCTGTGAGTCTAAATGGAATTTGC
LG7	ref-62012_17	84.793			AGTACCTGAGCGAGCT[C/T]AATGCATTATAGTCC
LG7	ref-62012_14	85.345			AGTACCTGAGCGA[A/G]CTTAATGCATTATAGTCC
LG7	ref-1028_25	86.059			CGCCAGCATGCGAGGCAAGTGCAC[A/T]AACAATG
LG7	ref-8569_23	86.451			CAGGCCGATTCGACGAGAATGC[A/G]CACGAAAAC
LG7	ref-60058	88.58			GAGCCTTCTGGAATTATCATTAGCCATAGTTTATTATACAATTTGACCTTCATCTAAGCTGTTGCAATGGATATTGAAGATTAGATTTGCATTAAATTTCAATTCCTGACAGATGTCTGCTTGCTAAAGCCTGATGTGCTTTTGCCACAATTAGTTTTTCTTGGTGTCTGAGACTAATTTCAGGGATAATAAAAGATTTCTTTTGTCATACGACACATCTGCCAGTCCTAGATAACATGGATCACATTGTCATTGACCTTGTCTGACCAGTTCTGCAGTAAGATCTCAGCAGTTGATGATATGTGTTCATAAGAGCTCAACAGAGAGACATTTAGTTTAAAATGTTTATACTTGCAGTGCCCTCCATAATTATTGGAACAGTAAAGACAAAATTGCTCTGTTAGCTGTGGTGTCAAGACATCTATAAATAT
LG7	ref-72858_10	91.902			GGGCAGATTATCACAGACTTATTTTCACACAGTTCCATGCACAATGCTATGTTATGACCTCAAAACAATTTTACCATAGTGCACGGTTTGTAAACTAATAGATCTTGACATTTGCTTCACATGACTTTACTTAAGTAGTAAAATTGCTTGCTCACCTACTTCAGAATTGCACTTGTGTT[A/T]CAGAGTGCTCGAGTCCTGTCAAGCACAAGTCATGATAAAAGAGCTCAAAAACTTGTAGAAGCAAGCTAAAATGGCACGATTGCTTCAGCAAATGTGTTTTTTTCTCACGTTTATTAATGTTAACACTTTCGGTTAGGTTTAGGGTTGGTGGTACGTTATTTTCAAACAGAGCTTTAGGGACATTTAATTT
LG7	ref-16542_10	93.398			GATGAAGAACAAGAAGAAAGGACGTGCTGAATGAGAGCAGAAAATAATGAGGTGACAGAGAGCAACACACTTGTGGAATCATTATTCAGAAGGACTTTTTTTTAGAGGGACTAAAAGCATAATTTTTGCAGGAAAAAAAAAAAAACATCAACCACCAAATTAGAGTAAAAGTCACCTTT[C/T]GCATGTCATTCGGGTTACTCAACAGTTCACTTTATAAGGAACAGCAACATAATACATGCTTTAAATGATAGTCTGACTGATGTAAACCAAAGACAAACACCACCTGCAGAAGAAAACTCTTCATCTTAATGTATCACCATCATTTTATCAATAGGCCACTAAAACAAAAATATGAACAAGACGTGGCATAA
LG7	ref-51670	94.48			AGCAACGAGACGACGCAAGTGCCTGCTGATA
LG7	ref-5857	95.418			GCCAAAATTCCGAGCCCTCTGCCCGGACAGC
LG7	ref-63323	96.52			CCCAGATGTTCATTGGGAATCGAAAAAGTCTAAACAATGCCGCTCTGTATTATTTAGCAGCTTAAAAAAATGGTTGGAAATATTCTTAAAATACTCCACATTGTGTCATGAAACTGAAAGATTTGTCAGAGCCCTGGAGGATATGATGTCATGCCTGACTAATGGACTCCCTCTTGGCCGCACAACACTCAGCAGACAGTTTGAGAGCATGTTTTCATTTTCCAAACTTGGAAAGCAGTTTGGAAATTCAAAATGAGACGAGCAGCAGGAGCTCGAGGCACTTTCCAGCAGTGAAGCTGGGCTTTGTGTAGATTCACGCTGCCAGATCTTTGAATTCCAGCATAAAGTCCAGTCCAGATTGACCATTTC
LG7	ref-8532	97.806			TGAAAAGTGTCGAATCAAATGCCATACGTTC
LG7	ref-46131	98.787			GAGCCCACCTCGATGCCAGTGCCAGAGCCAA
LG7	ref-39264_8	99.431	Chr5	64876800	ACACATCTATGAATTTCCTCATCCTGCACCTTCCCAAAATACATAAAGGAGAACCTCATATCTCATGGCTCATTTGTATTGTATTGCAGGAACGGTGGCAATTGACCTGCAGAATCCAGATCCCCCGGCTGACAGCAGTAAACTGGCTCTGCCCACGGAGAACGGAGCTGGGAGTCGTAGACCCAGCATTGCCCCTGTGCTGGAGATC[C/T]CCGATTCTTCTGCCATCCTGCCTTGTGATCTTCTCAGTGACCCCTCAGAAGATGAGACCAACCAATCAGATGAGGAAGGAGCACTGGCCTTAGAGTAAATAATGTTGTCAAAAGTACAAAATGAATGAGGTTTCTCAGGGGAAGAGCAATAGAGTCATTTGTCTTTACTAAACAATACTTGGAGAAAAATAAGGCTAATAAAATGGCCAGTGGATGATAAAAT

LG8	ref-6384_1	0			[C/T]GTTGATCCACGACCATGGTGCGCTGTAGTCC
LG8	ref-68142_30	4.689			ATTAAAGAATTTAATGTGAGTTTGAATATTTATAGCCGTATTGAAAGCGACAATGAATAATTCTTGAAAATAAATGAAACAAGAACGTTCAAACTGTGAACAGACAAGTGTATTCTATGTGAATAGAGATTAAGTATTTTTAATATATTTTTAATATGTTAATATGGTGTCATATTTTTGAATTTGATTATGTACTTAACCATTTGATTCCGAGTGCAGTGCGCTTGCA[C/G]AGACTCCGCCTACACGCTCTTCTGATTGGCTGTGATTTTTGATAAATTTGAATGCAAGTGCAGTGCGCTTAGAGGCTCCGCCCCCACATGCTCTTCTGATTGGCTGCGATTTTTAATTGATTTGATTGCGATTGCAGAGAGACTCCGCCCACACACGCTTCTGATTGGCTGGCTTTTTATTATTATTGTTTTTTTTATTTGT
LG8	ref-68142_32	5.352			CATTTGATTCCGAGTGCAGTGCGCTTGCACA[C/G]
LG8	ref-4420_3	7.444			GAATCTCTACTGTCCATTCCAGCCCAGATCAGCGGTCAGACAAATGAATGCAAGGACATCCGCATGGACGTCTCGCTGTATCAGCACCGTCTCCATCTGTTACGCCGTCAGCTCATCTCCACCGTACGCTACCATCACCCTCTCTGACCCTTGACCTCTGCTCACGGTCACTTCAGCGCCAGACTCATGTATCGGTACAGGA[C/G]CATTGACCGACCCAAATGCTGGATCAATGATGATTTCACAACATGGATCATTGATTTCACTGCTGTCACAGAAATGGCAAGTTTGAGCCGCACAAGATTAAATATGAGCTTTCAAACACGACCATGTGACTGAATGAGAGGAAACTTCAGCTCACATCTGGGTCTTAAAGGGACAGTGTATCAGTTCACAACTCTGCAAGATAGTTCCTTTAAAGTCACCATGAAATCA
LG8	ref-4420_7	7.466			GACCAT[C/T]GACCGACCCAAATGCTGGATCAATG
LG8	ref-61780_28	8.604	Chr6	58065571	CTAGTTAAATTTAATTAGTCCTACATCCTATAAAAAATATATCATTTAAAATATTTTCAGATTTTATCCTTTAATTATATAATAACTGCATTATTAATTTACCAACAGGCATTTAAGGCAATTTAATAAAGAAGATTTGAAACGTTTCCTAAAGGTTTGCATGTGTTATACTGACAGACGCATCTATATCGCTGTG[C/T]AATGTTTAGCCTTGAAGGAAAGTCAGAAAGGAAAGTGGTCTTGAATATCAGAATACAGGCAGCACTCTAATCTGGTTCAGGTCCATCTTGCTCCAGCGATAAGCTTTGCCAATATGGTATCCGTTTCTGCTTGTTTCCTTGGCCGGTTGCCCAGCGTCCATTGTTTTCCCTCC
LG8	ref-36960_16	9.76	Chr6	57647898	TGATACCATAACTTTATTTTAAAGCAAACCTCAAGGTCTTATGTCTCGGAGCATGTCTGCATGTGTTCATACCGGTTAGGACTGTGTCGTTTGCTGCTGTCATTGGGCTCCAGCAGGAGTTCGGAGGAGTCGGCCGGTGACGGGACGGTGGTGCTGAGCAGGAGGTGTTCGTGCTGGGACAGATACTGCGCTGGAGTCTGTTTGGCGGCCCGAGC[A/G]CAGTGCAGCAGCTCCCTCTGCAGTAGGGGCAGGTTGGCCTGTGTGGAGGACACAAATCTCACTGCATAACAAGCATCAACTAAATGGAAGATTGCTAGTAATCAAACCCACTTTGGAAGCAAGTGGTCTAAACTAATCAAAGACCACCACATTTTTTGCATTTTTCCTGATAAGAGTGGAGATAAAGGTCAAAAAGATTTCTTCCTCGACAGACTC
LG8	ref-57461	12.494			ATCATGCCAGCGACTCTTATGCATGAGATAT
LG8	ref-28278	16.898	Chr18	22402061	AATTGCAGCAGTGTTGCCAACTATTTTCAATGGAAAGTAGCTAAAGCCTGCTCAAAAAATTGCTAAATGTCACTAGATGACTTAATGCGCCAACTGTTGATACTTTACATCAGTTTTCTCTCCTAATCCGTGCTCACATACTGTATTTTAGTTTCTGTTTAGACAACGCTATGTTGGCAACACTGAATTGCACAATCCAAGCAACTGAGATGAATGCATTGCAGGTTCAATTCCAGATATCTTCTCTCTTAAAGGTGAAGCATGATGTTAAAATACATCCAGTTTTTGTTCACTGACTACTATTAATATGCCATACATGGGTTTATTTCTCCCAAAAGTGTGAACATTGAGCCTCTCAGGCACCATCTAAACATTGAGAACTGCCTGCTCAGATCTGTCAATCTCTTTCCAGCTCAACCTATAGCGTGAGT
LG8	ref-61591	22.514			AAAGATGAAGCGATGCGAGTGCTTTTTTTGA
LG8	ref-39362	25.429			AAGACAGACTCGAGCTAATTGCATGAGCTTG
LG8	ref-8251	29.324			ACTTTTGCATCGAAATATTTGCCAGGGCTCG
LG8	ref-43495	33.865			CAAACATATTAACTCTGTTACCATGCCAAACACTCCTATTTGGGTGCTACACTTGTCATAACAGAATGTGCAATGTCACCCACCCATTGTGTACTGAATTTTAACTGCTTCCCTAAGGCTGGAGATTTCATTTTTGATCTGGCTTACCCTACAGTTTCGTGGACTTCATGTTGTCATTTGCAGCATTTTCGTATTCAGTGAGTTTTTAAAAAAAAACTTGCTGAAAGCTTTTGTTGCATCCCGTCTGGTCAGGAAATGGCATTAAACTGCACACATCTTGAAGAAATGATCCAGAAAAACCTACATAAGCCAAAAAAAAAAAAAAAAAAATTGAGTATTTTTATCATATTTTTCCCTTGAAAATGCAAA
LG8	ref-14408	34.561			TGTTCAGTAGCTCAAGACGTTAAGGATTGTGTGTCTAAATAGAAATTGACTTTAAAAAAATAAACCTGCCTTGAGAAATTTTCACTGGAGTGAAAAGTCTGACAAATGACCTGCTCTTCTCTGCTTTCTAAAATAATGCCACATCTGCCCCCTGCTGGTTGTATATTAGCATCACACTTGCATCTGGTTCCACCAGGATGTTGGATTTGAGGTTTAGGCTTGAGAACACAAAGAAAGAAAAATAAAGTTTGTAGTATGGCAAGATGAACTGCAGAAGTGATTATGGTTTGAATATTCTTGAAGCAATGAACTTCTTCAACTCCTGTCAGAGACACTCTGGGCTCAGAGCCAATGAAGGAGAGGCAGGGA
LG8	ref-23753	35.463			ATGCAGCGCGCGACTCCACTGCAGATCTCTG
LG8	ref-16790_32	35.81			CCCCTATGGCACAAGCCCAGACCGCAAGAGGTACCGTAGCCAGAAGTGGTGAAAAAGTTGCTTATGTTCCAGACATTGTGCCTTTCTAAGAATGATCTAATCTGAACTTATTAAATATAACTCGCAGTGCATTTATATACCAAATCTTCATTTTGAGTTAATTCTCACCTGAACTACTGATCCCCGGGAGCTGGGTGAAAATGTGTCTTCCGAGTGGCGTGCAGCGATGAG[A/C]AACAAGCATGTGATTTAAAGATTGTGGGTTCAATTTCTGCCTGGTACAACTTTATAAAAGTGTGTGTAATGTTGTGTCACTTCAATTCCTTTATTATCTGAGTAAGCATTGTACTAATCTTCCTTCCTGTGTTTTTTGTTCATTTTGAGTTCATTCTCATCTGAACTTCTGTTCAGTTTGAGTTCATTCTCACCTGTACT

LG8	ref-16391	46.073			TGGTCCTGATCGAGAATGTTGCTTCTCTTTG
LG8	ref-24745_31	46.619			TTTTACTGCCCGAAGTTGCTGCCCGGAGGC[A/G]T
LG8	ref-71550_19	47.197			AATTAATTTGCGAATCAC[A/G]TGCGTGCCGAACT
LG8	ref-71550_23	47.21			AATTAATTTGCGAATCACATGC[A/G]TGCCGAACT
LG8	ref-69899	47.917			TAGTGGTGTTCGAAGTAGATGCATATGGGAA
LG8	ref-50683_27	48.053	Chr17	24434816	TGATGAGAGAACATTGTGTTTGGTACATTTTTCATCTTCAAACCAAATTTGGATTTTGGTTTAAATCCCGTCTGGCTAGCATGCTTCCCATAAAGTTTATGCATTAGGTCTGTAAAGGTGGAGAGTAGGTGGTCTTTTGCGCTGTTGAACGCATTTGACCACATGAGCATTTATGCTAGCAATCCAGTCGAATGC[A/G]TTTTCGACTACCTCTGGAAGTGGTTGAAAGTGGACAAGCTCAAAATGTTTTACAGCCCGTTTACACCTGTATTTAGCGTTGTGATCCAGTCAACCAAAACGCATCTTACTACAAGGTATAAACAGAGCCTAAACTATAGGGTACTATAGGGTAAATTCAGGATGATTGGGACA
LG8	ref-66907_19	48.431			TAGGGATGCGCGAGACGA[C/T]TGCTCTGTAAAAT
LG8	ref-66907_15	48.509			TAGGGATGCGCGAG[A/G]CGATTGCTCTGTAAAAT
LG8	ref-54299_17	48.692			ACTAGCAAGACGAAAC[A/G]CGTGCAAATGATAAC
LG8	ref-54299_32	48.707			ACTAGCAAGACGAAACACGTGCAAATGATAA[C/T]
LG8	ref-39898	48.962			GCCCAGAGATGTGTGTGACGAGTGGATGAGTGGAGTGCCCTGGGTTCTGATGACGTACTGCAAACCAGGTGGACAGCCCAGCAGGGCGTTGGTAGAGGCATTGGATGAGGGCACAGAGGTCTGAGACCACTGGATGGGACTTACAATGATGTTCTCCGGGAGAATAGGGTCAGGTTCTTCGTTGGTCTCCTCCGAGGTATAGAGAAGGAACGAAGCGTCTGCTTTCACATTCTTTGGGCCAGGACGATAGGAAATGTTGAAGTGGAAACGTGTGAAAAACAATGCTCAACGAGCTTGGTGGGGGTTCAGTCTCTTAGCATCCCTCAAGTACTCTAAGTTCTTGTGGTTAATGAGTACCGTGAAAGGGTGGTTAGCGCCCTCTAACCAATGCCTCCACTCCTCTAGTACCAGCTTGATGGCTAGAAGCTCCC
LG8	ref-13830_9	49.422			GACGTCTG[C/T]GCGAACGGGGTGCACGAGGGTAT
LG8	ref-71595	49.976			TTGAGACGCGCGAGCGCGTTGCGCGCCTGTT
LG8	ref-38882_6	50.657			CCCAAATGCAGTGCATTTTTCTTGCCCTCTTTTGATTGACAGGATATATCAGCCGATGCAAATCATCGATGCAAAATTATGAAATTATGAAAAAAATACTAAATTGTTATGAAATCAGTTGCTAAAATTACAGATGCACCGATATATCAGCCAATAATCGGTATCGGCCGATAAAAGCAAATTTTCACACTATCAGCTATCAACCA[A/G]TGCCGATATCAGTGCATCTCTAATTTTAGCCACTGATTTCATAACAATTTAGCATTTTTTTTTTTTTCATAATTCCCATTTTTGCATTGATGATTCGAGTAGCAAATTCTTACTGTTTGAACAGCAAATACAACCATAGTCTATAAATACTTTACAATTTAAATCTGCATCTTTCGTGCATTGCAGTAATTTGAATGAAAAGGCAAATTGTGACACAAGCTTTGT
LG8	ref-43905	51.001	Chr6	38933135	ATTTTGTGATTTGGTAAGAAAACAATTACACTTTTTTTCTCACTTGAATGTATTTTGACTTTCCAATCCAGAACAACCTTTTACTGTACATTAGATGGTAAGTGTGGCACCAGATAATAGCGCGTGCCCTGCCTTTCTTATCTTCCAAATACACACTCTTCCTTTACGTGACTCTGGCGGCAAACCAGTCATTGTGGTTATTGCAGACATCCATTAAGATATCCATCATTTGCATGCTCAGCTTATGGATTCTAAATGAAATGATCGATAGATTTGTTCGTAGAGAGAAGAAAACAACTCTTGATGTAGTGAGAAGAGAGGAAGCTCAACAGGAGGGGCTTCACAGTTGGCAGTGTGAACGTAAATGTA
LG8	ref-68005_14	51.505			CTGCCTCCTAATGTCCTTCCTGTGCCTGGTTCTCTTGTGCCAGTCTTTCAGTGCGCTGTTGCTGAGTTCTAAAAACTATTTACACGGCGTAAAGGTGGATGAAACTGTCACACCTGTGCACCAAAAAATTTCACAGTCTACCACTCTCTTTTCGTAGTGCTGTGTCAGAGGAATTAAATCGCTTGCAAACATCTGGGGCAATAGAATGCATCGA[C/T]ACCTCTGCATGGGTGTCACCAATTGTTGTTACGAAGAAAGAGTCAGGTAAAATTAGGATGTGCGCTGACCTCAGAGAGCCGAATAAAGCAGTGATCATGGACTCTTTTCCATTGCCACACACAGATGAACTACTCTCTGCTCTAAAAGTTTCTACTGTTTTCTCTACAATTGACCAGACTAATGCCTATTACCAAGTGTCGCTGCATGAGGACAGTCG
LG8	ref-58408	51.548			ATTCTGATGACGAAATTCATGCCATGCACAC
LG8	ref-11594	51.792			CCAGTCCTGAGCACTCTCGGTTGGATGATTGGTATCGGGGCTCGGAGCGTGACTCACAGCTGCACCGCACCCTAGTGCCTTTCTTCACAGAAGTGCATGAGGAGTTTACAAAATCGTGGAACACACCTTTTACTTCCTGGTCCCACTTCACAAGCTCTTCTGCCCTCACTATCCTCAACATTTTTTCCACCTTTTCCCAGATGGAGAGTGCAATTGCGGTGCATTTATGGATGCAAAATCCCGCCACTTGGTGGAATTGTCCTAGGCTCCAGTCCAAAGCCTGTAAGTTAATATCGTCTCTGACGGCAAAGGCTTACAGTGCTGCTGGAAGGGATGCCTCTGCTCTGCATGCCGTGGCCATCCTGCAGGTCCATCAGGCCAAGGCACTCAAACAGCTGCACGAGGGTTGTTCTAGGCTAGGATTGATGCAG
LG8	ref-27015	52.863			TTTTTGCAGCCGACATTATTGCATTTGTAGG
LG8	ref-55271	53.375			TCTAGTGAACCGGCAGTTTACACAGAAGTACTCTTATCTGTACTTTTCACAGGTAAATTGAAATTGAAACCGTTGATTCACTTGCTTTCTCACACGCTGGGTCTCTCTCGTGAGCATAGTTTTATTTATTTAGATATAAAGAGCAATATAGTCTAATGTTATTAGATTGCTCAACAGCAGCACTTCTTTCCGGCCCGGAACCAGCTTTTCTGCAGTGGAAATGCGTGGAATGGTTCGAGAATGGAGACAGGCCGGGAACCCGCACTGTCAATGGAAAAGGGGTATCTGAGGGCAATGTTTGCTCTTTGGCTGCCAAATAAGCTTAAATAAGCAG

					GGGCCTTTGCAAAAAAAAAAGAGGCTGTGATCAGA
LG8	ref-28745	54.083			TATAACCACACGACTCAATTGCACCCATGTG
LG8	ref-68729	54.38			ATTACTTCTTAAACAGCATTTGCTTGGTGATGAAATGTGCAAGATGAAATGAGAGTGAGGTGTTTTCCACCTGGCAATGGTTTGAGAAATGTTTGTAGGTCACCCAGCCATTGGTTCTGTAATGAAACAAGCTTTTATCTGTAAGTTACAGCTGTGTCACAAGGCTTTGTTGTACCTCAGCATCATCTTCTTGGGTCTCTGACAGTGGTGAGAGAGAGAAAGAGAAAAGTATGAATGTGAAGAGGCTGTATTCCCCAGTGGGCTTTTATACTGAGAGGCTCTTCACAGCCAGCCTAATTATAATGTTCAGAGTCATTGTGTCATTATGCCCCCTTCCCCCTTTTTTTGTGGAAAAATAGCTGATGAGC
LG8	ref-61585	54.38			AGAAAACAAGCGAATCATCTGCGTACTCAAT
LG8	ref-47586_26	54.741	Chr7	42037467	GTTTTCATTAGAGTAATAATAAAGAAGCTCTCCTACATTCATTGTCTCACTGTGCTTGGAAAACTGCAACATCAAAAAAAAAAAAGAGAGAGAAATTAATAAATGTATAGGTATCGGTATCGGCGAGTACTAGAAAAAAGTATCGTACTCCTGCTCGGTCCTTAAAAAATGGTATTGGTGCATCCCTATCGGTG[A/G]ATGTTATGGACAACGAGCACAGGTGCATTTTATTGATGGCATTTTGAATGCACAGAGATACCGTGACAAGATCCTGAGGCCCATTGTTGTGCCATTCATCCACGACCATCACCTCATGTTGCAGCATGATAATGCACGGCCCCATGTTGCAAAGATCTGTACACAATTCCTGGAA
LG8	ref-66922	55.379	Chr6	32970944	TACATCAACGGGAATCGTATTTATTCAGCTTTTAGATTTCAAAAAATGTAGTGTAGTTGCATGATAATGTGGCCTTGTGTATTATGTTCCACTGGTGACACCTGTAGGCATCAGATGGGAAAAAAATATATATTTTTTTTTTTATAGGAAATTTTCCCAAGAAATCTCAATCACAAAATTATTGCTCTTACAGAAGATACGAAACCTACTCGATAACATTTCAGCAGAGTAACAAACATGTTTTACAATTTGATGACATTTTATTTGTTTATTCAATGCCTCTGTCTGTATCCTCTAGGTCTACCAACAATAATACTGATCATCTGGGTTCTAGCAAGGAATTTTTTTGATAATAGAGGGTAAGTCCAGATTGATCTAAACTCATATAGACACAAACTTCACGTTTCTGACAGTAACACTAGTGTGTGATA
LG8	ref-41037	55.442			ATCCAGTTTAAATGTCTTCCACGATGGGTAAAAGTAAAATGAATATAAATAAACAAATGTAATCTTAATAAATACTACAATAGCAATCTGAACGATCAAAATGCAAAGTGGCTAATTGTATATATATTCAGAAATTGGTTTATACTTAAGAGAGCGTAACAAATTACTCGCTGAAACAGTCACTGGGGTCGTCTTGCTTCCTCATAATTTCAAAAAGAATCATTAATTGATTCAAAAATATAGGACCGTCTGGTGGATGAAAAATAAGAGGGAAGCTGAAAAGGAAAGCAATTTCAGAGCAGAGCAAGTTTTACATGTGTTAAAGAAAGATGAAGACAAAAAAAAAATCTTTAGAATATTGTGCACTGA
LG8	ref-68035	55.671			TTAAACACACAATTAAAATTCATTTGATATCTTTGGCTTTGAACTGAACTTTTTTTTTTCTTTTCATTTGTTTTATATTGTGTTGTATGTTTTATTAAGCACCCTTTTTTGTGCAGCACTTTAAAACTATGTTTAACTGTAAAGAAAGTAGGTAAGAAAACATCAACACCATGGAATCGCATCAGTATCAGGACTCAGTTAACAGGCATGATGTACAAAACCAGCTTGTTTCTCACATCCCACACAGCTCGCCTCCAATCCCAAACCCCTCTCCCCCATCTGAAGAGCAGCCACTCCGATCTAGGAGCTCTCCCACTTACACCACCACTCTTACTACAAAGAGTGAAGGCACAGATCCTCAGTAAAGA
LG8	ref-51877_31	56.159	Chr6	31204299	ACATAAGATGACCTACTTAGGTCTGGGGCAGGGCGGGTGCGTTAAATCGTTTATTTGAATTATTTTTCCATAACTAGTTAAGTAAATTAAAGTGTTAAATTGACAGCCCCAGATTATTTTAATCGTTATTATTTTAATCAGCTTAAGATATTTGGCAGACCCCTGCAGTTCCTCACTGGGCACTCTAGTCGCAGACAAA[A/G]CCTATGATACAGTAAATATCTTTATCTTGACCACGAGAAGATAGTTGTAGGTCTTACTGGTTTTGTGACATCATAAGTGTGACAAACAGCACCACTGACAGGAACGCAATGATCAGTAGCAGTATGTATGCTGCAGGGTCGCTCCTAGTAGCTGGATGACAAAACAAAAG
LG8	ref-32815_17	56.222			TCGGCCGAGGGACTCCGCACGTGAGGAACGACAGATGGTCAACATTAACGTCTACAGCACTTCAAGGTCATCTGCAGTCATTCAGGAGGTAAAGTGGGCATGATTGTGCATGTCTTTCACCAGCTGGTGTAACACTAGAGTAATACCACTTTCACCGGGTGCTTGTCAGAATCAAGGGTGTTTTAATTGCTTGTAAGAGAGGTGAAGGGCCGATAT[C/T]GTTGCAGGAGTGGGTTCGATTACTGAGGCCGGTGAGCGCCTGCACTAACAAACCTCACAAGAATCGATCATCTATATTGATAGACGGTTGAATGAGCTCCCCAGTGTTGACAGCTATGTGCCTGATCCAGATTTGTACACTATCAACTGTAAATAGTGTGCAAGATTGGAATTAGAGCATCCCAAATCGTAATATATTGAAAATATTATACCAAA
LG8	ref-36216	56.733			CTCCTGAACACGATGTTGATGCTGTTTACTT
LG8	ref-48409_24	57.357			TATTTAGATGAAAACTGCATTTTGGTGGCCTGAAAACGCTAACTTTGGAAAACAGGTTTCAAAGGTCAAGTTTTTGAAAACGATACCATGTAAACTACAAAAACACATGCGCAGTCTATTCTTTACAAAGTGATATCGCCAACTACTGGCCTGGCAGCATAATACAGTGTTATAGTCGTTTTTTGCAGATCTGTGTGAACAGGATCATTTCGACAGCATTGCTG[A/G]CTGTACGCAATAAATGCACAGAAAAAACTTTTCCATTTTTAGTACATCGCTGTCATGTAAATGTCAGTTGTTGTCTTTCTAAGGGTATGTTTACATGACAACGATGTACTAAAAAAAACCAAAAAGTTTTTACTTTGCGTTTTTCGCGTACAGACAACAATGTTGTCAAAATGATCCCCGTTCACACGGATCCACGAAAACAATGCTG
LG8	ref-45322_17	58.19			GGCTCCTCCATCCCTCGGGCTCCACCGTGATCCTTGCTCACACCGACGTCGCATCAGTCCTCCGGCCCCCTGGCTCTACATCAGCCACTCGTCGCCATGACTCCACCTTGGTCTCCCAGTCCTTTGTCACCAGGTCTCTTGGTCCCACGGATGCGCCTGGGCCTCTACATCTCCAGGCGGCATCGC[C/T]GTTGGTTGGGCTCAAACTACCACCTTGGCTCCTCCCTCCATCACTGCCGCCATGGATTGCCTCCCTCATGGCTGTCTCCTGGGTCATTCTGAGGTCTTGCCGGCCATTCGTTCTTCCCTGGTCCTGCCATCTTCCACTCCTCCATGGACCCTTCCACCATTCACTCCTCCATGCTCCTCCCAC
LG8	ref-33372	58.297			ATCCAACAGACGAAGTTAATGCTGGTTCTGA

LG8	ref-9407	59.193			ATTATCTTGCCGATGCTGATGCAGCTCTCTC
LG8	ref-47967	59.569			CCCTGGACCTCGAAGCATGTGCTCTACCACT
LG8	ref-57855	60.216	Chr6	27524668	ATTGCGTTTCTTTGACAGAGTGGCAAAAAGTTCTTGTTCTAAGCAAAACTTTGGACAGAGCAGAAATAAAGTGGAGCAGACGGGAAAATAAAGTAACGTATACACACCCA<br>GGAGTTTCTTGACCTCTTCCTCGCTCATGAAGACGTTTACATTCGGCCTGTTGTTATTGTTTATGCCTGGCGCATTGCTCGGGTTGGCGAGGCATCGGGCCCCTTCGAGAAG<br>CAGGAGGAGAAAGAAACCCCCTAGTCTCCACAGTACCGTATGCTGCCAACCCCGGGACACCGCCGACGCCGCGCCTCCACCGGAGCCCCGCGCTGGCAGAAACATCACAG<br>CCCACCGTCGCGTCAAAAAGTCTTTGAGATCGAACTG
LG8	ref-40411	60.88	Chr3	36455863	TATCGCCTATATATATATAGTCCTTGTTTGAAGTATCAGCTAGATGAATACAGGTAAATGATGTAAGTGGGTTTGTTTTTGAATTATTTAAAGGGATAGTTTACCCAAAAAT<br>GAAAATTCTGTCATCAATTACTTAGGGGTAGTTCACCACTGGCAAAATGGACTTTCAATAAAACTTGGCTGCCTATGCAGAAGAAAAGAGGGGAAAAAAGAAATTAGTGC<br>TACCTGACTAGAGAAGGGAAAAAAAAGGCTGTTGTCTTCTCTTTTGCCAAATAGGTAAAACTTCAACACCCATAATGCACTGTACCATAATATGCATGTTGCCATCCAAGG<br>CCACGCCCACTAGTCTGCAATGTATATGCTTTACCAGAGTGAAATACCACCTTGCTTTAGTAGGAAATAATTCTTAGCAAACTTTTAGTTATAATGGT
LG8	ref-22710_6	61.361	Chr9	1601283	CTTTTATGACCACAGTGTATGCATCTTGTGATGGCTACTTCCAGAAGGATAATGCACCATGTCACAAAGCTCAAATAATTTAAACTTTTTCTTAAACATGACAGTGAGTTC<br>ACTATACTTAAATGGCCTCCACAGTCACCAGATCTCAATCCAATAGAGCAACTTTGGTATGTGGTGGAACGGGAGATTCAGGTGTGATGGATGC[C/T]AGACGAAAAATCT<br>GCAGCAACTGCGTGATGTCATCATGTCAATATGGACCAAAATCTCTGTGGAATGTTTCAAGCACCTTGTTGAATCTACTTCATGAATAATTAAGGCAGTTCTGAAGGCAAA<br>AGGGGGTACTAGCAAGGTGTACCTAATAAAGTGGCCGGTGAGTGTATAAACATATTTGCCTAAGAGTTTTTCAGTGTTTGTAGATGAATAATCCCTTCCTT
LG8	ref-22710_7	61.579			GGATGC[A/G]AGACGAAAAATCTGCAGCAACTGCG
LG8	ref-434	62.214			AAATGGTAAACGATACCAGTGCTGATACTGG
LG8	ref-411_16	62.423	Chr6	26111082	AGAGCCAGGTGCGCTGAGATAATGTACTGCCTCCCGGCTCCTGTCCTCAATGCTCTTCCTCTATGACAGATGGGTGATAAATATGCTTGTATCTAATTAAATCAGTAGAGGT<br>TATTGGGATCAATCCATCTCACCTGCCCCTCCCCACCTGCCCCCTCCTATAGTCCTATCAGCCATTAATCTCAGCTTTGTTTTATGTTCCGATAATGTCGACA[C/T]TCCTGCC<br>TGCCTTGAATTACATCAACTTGATTACCACTGAGATGCTGCCACCTGGCACCCTGTTTTTTGTTTGTTTAAAAGGTTAAAAAAAAACGGTTTGACCTGCGACATAGGTTTCC<br>TGTAAAACTTTAGCATCATGCAGTTCTATTTGACTCTGATGACTTCCTGCAATTTTTTTGAGCATGGATTCTGTTTAAAGGTAGGGTAGGCAATATT
LG8	ref-69403	62.665			ATTGTTTGCTCACCTTAATGTTGTTCCAAACAGATTTCTTTTTTCTGCAGACCACAAAAACAGATATTTTGAAGAATCTTTCAAATACATAGTTCAGGCATGAAATTCTAACT<br>GAACGCTGATTGGTTATTTCAAAGCCGTGGCACCAGCCAATCTGAAGTACTCCTAAATCCACGTAACCATATATATCGCACCCTAGCCTGCGATCGGCATGAAAGCTTCTG<br>TTTTCCCTCCTCCTCCTCGTCTCCAGCTCCACCTGAGATCTGCTACGGGGGTTCTACCATTTTCTCGCAGCAGCCTGTCCTTTTGTTTTTTATTTTAACTAAGCTGAACAAATT<br>ATTGCTGAAAAATTATTATTTGCTCAGCAGC
LG8	ref-9210	63.039			GGAGCAGGTGGAGAACAGCATCCTTTACATGATCAGACATGAAGTCAATTAGATGTGTGAGGAACGAAGAAAAGCATATGAATGCAAGGCACGGAGCAGGGGGTGAGGC<br>AAGTCTGATGGCTAAAATGGAGGTCAGCAGAGGCAAAGTCAAAATAAGATTCATCGTAAATGGAAATGCAGCAAAGAGCTTGGGGGGAAGGAGGCTGGAGACTTTTCAT<br>AGATAGAAAAAAAAATGTAATTGTATGGTCTCAACAGTTGATTTTAGAGTGTAAATGGAATCAAATCTGAATTACTTTTGTCATCCAAATAGATAATCCTCTAATTCTAAAT<br>AGAGTTATCAGCCATATTTACAAGCATCATAAGCATTTTT
LG8	ref-41495	63.169			CAAAACGATTCGAAAACTTTGCTGGAAGCTG
LG8	ref-37382_17	63.349			GTTGCTAGACCGATTG[C/T]TTTGCAAGTTGTAAT
LG8	ref-26840_7	63.631			AAAGTATTATCACCACTAAGATTATTGGGAACATCAGTGGTTTTTGTTTACAATCTGAGGGCAGTTATGAGATGAATCTTTATTGGTAGATGTGAGCCTCACTTCCTTCCTT<br>TCTGATGCTTTGCATGACTGCAAAACAGCAACAGTAGGCCTTGTCATTTGCTGGCAGACTTTTCTTATCTGAGCGCTGGAGTCTGGCAATCTGAT[C/T]GGCGAAGAGCTTG<br>CACCTTCTGTGCTAGATTTAATTGAAGCATTTGGTTTGTTATATTAGAGCCTGTCATCTGACTCTTATACCTAAACATGAAAATAGTGGTGGTTCTTTGAAACAGCAATTTCT<br>TCAGCTTTAGTGCTTGTGTTGCAGTTCCACAACTGACACCTACATAAATGCTACTAGTTATGTACTCTTTATCAAAGTTTAGCACACAGCAAACAAAA
LG8	ref-39679	63.728			ATTTGCTCTGATTTGACTCTAGCCACAAAGTAAGTGAGTAACCCATTCGTGGAGACACCAACAGCACATATCTTTAACAGCTCTCTGAACTGACGCATCAATTTTTTGTTTA<br>CTTATATGTAATTTTAATGGTCATTAATTATATGGACTAACATATGGTAGTTTACCATGGCATGTAGGTCAATATTTGTATTATGTATAATCACAATGCGATGTCTGTGCATT<br>AACACAATGCACAGATATTGTGGACCAAAAAACGAAAAACAAATAAATAAATAGGTGTGTAGAGTGATTCCAATAACATTTGTATGTTTTGAAATTTATTTGTCAGGAAC<br>AAGCATTCCTTCCCTCAAATCATAAGTTTTTGGCAGTCTATAGAAATATATCCAAATATGCATTGATTAAAGACAGTACAAAGCAGTTCAACAGAA
LG8	ref-68151	64.166			GGGATCATTTCGACACCGTTGCTGCCTGTAC
LG8	ref-30062	64.55			CCTCCCGAGTCCCCACCCTCAATTTGATGGCTCCCCCACCAGTCCTGTCCAGCTGTCTGGATGCTCTGGTTCCGTCCAGAGACCTTCATCCTCCGATGCTCCTGCCCAGCTTC

					CTGGTCCCATCTCATTTGCTGGTTCTGTCCAGTCCCTTGGATACACCAGCTCCACTGGTATCATCCAG
LG8	ref-4422	64.605			AGTCAGAGCACGAGATGTTTGCAAACAATAA
LG8	ref-60017_32	64.969			AACATTGCTACGAACCAATTGCTCTAGACTT[A/C]
LG8	ref-38994	65.149			AGATCTGCTACGATGCTGCTGCAGAGCAAGT
LG8	ref-29047	65.486	Chr6	46834151	CCTACTGAGGACTCAGTTTTGGCCCCCAAGGTGTCCTTTATCAAAAGCGATGTTCATCCCCGCCTGACAGCGGTCAGAATGTATTGGTGTTCTTGCTGGGGACCTTAAAAAC<br>ACTTGTCATTACTTCTCATTGCCTCTAGCCTGTGGTAAGAATCAAATAAATTCAGTTTCACTGAGAGGAGTTTCCAGCCAATCCCGCGTGTCCTCGTGCGAACCCTGTGCTT<br>GGCATCTTACAGGCAGCTTAATGAAATTAATATATCGGATTACCTTCAGTGATTGATATGCTACATCCAAATGTAAACCATAATCTACTCTGCTGAAACTTGGACATCAGC<br>AAATCTGATTTATTGCAGGTACTTTAAGAAGAACTTATTGCATCTCTCCTTTCCCATGAAATTATAAAATGAACCTTTTGGATGTCAGCCATTATA
LG8	ref-21664	65.748			AGTTTTCCTCACAAATGACAACAAAGGATCATTTTTACCTCAGTTTTCTTCCATACTCAAACGTCTGGTCACTTCAGCTATAGAAAACAACATAAAAATAAGTAGTAAATA<br>ACTAACGTCTTGACGTCATTTTATTTTGTGTATCAGTCAAGACATGTTTACACATTCTTAATTCCTCCGCCCCCTTTCTCTCCTTGTTGTGAACAAGATTGACTCACATGCCA<br>AATGAATTAACGAAAAGAAATGCCCCCTCTCATCAGACGGCCAACAGACCCTCGTTCACGAACGAAATGTCGTGTTCAGTGCCGAATCTTTTGTAAACAAATCGTTTAAAA<br>CGCCGACTCGTTCAGTGAAGGGGGCGAGCTCGTTCGGTAAGTCCAAATGCTCCTTCACAGCCCACACTGGAATGCTATTGGCTGGAACTAGAGACA
LG8	ref-70052	65.842	Chr6	45068286	ACAGAATTGTCATTATTGTGTGAACAACTCTTTTAAAAAGAGCAACACAGAAACTCCAGCATAACAATTTACTCCCGTTTCAAACACACAGTGCATGTGCCCATGAAACCA<br>CCAGCCCCAAACCCAAATGGAAGGACTAATATGATAGAAATATTAAAAAATTGTGTGAGGATGGGTGCGTTTGAGCATGCATTAACTTCACTCAAACTGTGATGCTCATGC<br>ATTCACACTGTTTGATGTCTGTATAAAACAAGCCTTGTGTTTCAAATAGTGGAATGTAAGACTCTGAGTCAGGCAGTTCAACCTCATTTATCATAAACATGTCTCGGATGAT<br>CTGCTCAAAATATTTCAGATTTATAATTGTCACTACAAACACTGTTCTCAAAGAGATTGTTTTAAATTGCATCAAAAAATGATTAATGAAACAAGAA
LG8	ref-47022	65.918			GAATGCGAATTTTACCAGGGGAGCCCCGCCAAAATGCCTTTGGGTTAGTTTTGAGGAGCAATTGGGCGGGTTTTGTTGTGAAAACCTGGCAACCCTGGTTGTAGCTGAGGC<br>CTGGAAGAGTTTGTGTTGTCGGCATGACAAGAAGACGCTGTTTTCAGCGCTATTAATCCACTTTGCATGCACTTCATTCGAAGCACTTCATGCAATTCTCACGCTGAAATTC<br>ATATGGGAAAAACCAACGCCACTGTTACTGTTCGTATCACTAGCTATGTTTCTATCACCTTGTTTTTTGTTTTTTGCGCTTTTTCATGTATCGCATCAGAAACAAATAATAAT<br>GGAAACGTCCGATTTCGTGAAAAAAATTTCCCT
LG8	ref-73427	66.112	Chr6	23212178	TGTCCTCTAAAAAAAAAAAAAACAGTTCAAATGAATCGATTCAGTGAAATGAACTCTCTTTACGCTACTAAGCAACGAGGCCAAACACAGCGTGCCACTGTTGCCTAGATA<br>CGTGTGTTTGGCTCCTGAAGCTAGCAGCCTTTTCAACACATATCAGAAGCTGAAAGTTGTATCACGATGGCAGAAGACACGCAAGAAGTCCCTCAACTCGCATTAGAAGCT<br>GTTATTGGGTTTAATGGTAAGGTGAACTGTGAACCCTCTCGCATTTGATTGTTTGGTCTTTAAAATTTAAACAATACTCTCTGTATTTGTTAAGATGAAAGTTATTCAACGGT<br>ATCAAAGCATCTACATTGTTTATACAGGGCATGTG
LG8	ref-60419_29	66.285			GCTTCACAAACGAGGTCAATGCAACGCT[A/G]GAT
LG8	ref-60419_2	66.286			G[C/T]TTCACAAACGAGGTCAATGCAACGCTGGAT
LG8	ref-57516	66.31			TGTGCTTGTTGGATGAATTTGATTTAGTTCATTATTCCTTTTGTTATTATTGGTGTGTTTCCAGATATGTCATAACATGCAGCATTTTTGAAGGAGCTGCCATTTTTGCCACAT<br>TGTGCACTATTTTTGATATACTATGCTATCATTGGTTGTTTGATTGGGCTTACCCCAAGTGCCTCAAAAATATCTGCGGTTGGTAACTTTCCATGTCGATCAGATTGCTTCTC<br>CCAACAGTGGCATATCTAATCTGCAAGACTCTGAATGTAAAGCCAAGCCTGTTCGCTTGAAGTGGAGAGTCTGTGGAGAGACTGCTGTCCTAATCTGATCCCAGCTGTCCG<br>GACTTTCCTTCAGATTGGTAGGAAACTTTGCAATGTTGATTTTGCAACGTTGTTTCATCGTACTTTTCGTACAGACCTTTCAATGTTGAAGTA
LG8	ref-11563_25	66.772	Chr6	34242308	CACGGGCACATGTGAAACCCTTTTTCACATATGCCAATGTGTGTAGAGAGGCTGGAGGGGCAAATCTGGAGCCTGTGAAGAACTTGGAAACGTGTGTGCAGTGGCAAGGC<br>TTTCCACGCCGCTTAATGAAATGCTTAATGACCTGAAAAGAATCCCTAACCCCAGAAGTCACTCACCAGTCAATTTGAGCTGACATGCACTGTTGTTGCTCGAACCACTTGC<br>TC[A/G]ATTTTATTTCTTTTTACAATTCTTTTCTTCTCTCACTCTGTTTTTCTGCTCAACAACATCCATGGGGCTTGAGTTTTATGCTAAAATGTTATGACAGGAGAAGTTGGT<br>GGGAAAAGGTCTTCAAGGGTTTAAAATTTTCTAGAGGTCAATCTGTTATTGTGTGTTTTATGTGGTTGCTGTATGTGTCCTTTGGATAAATACATAAAA
LG8	ref-40712	66.972			GCACTTTGATATTCTTCTCGTTATTTCTCTATAGCGACTAATAAATCGGAAGTCTCACACATTCACAGAAAACACCTTATTTCCGCACTGAAAAATAAGGATGGACTAAACA<br>TGTAATACGCATGTGCATGCCGTCGCCGTTTTCACAGATTTGTATTTTTGCAGTTTACACGTACATGGTAACGGTATCGTTTTCAAAAACTTCCACTTTGAAACCTGTTTTCA<br>AAAGTTTGCATTTTCAGGCCACCATAATGCCGTTGTCAAGTAAATGAACAGCCAAAATGCGTAAAAACGTTTTCAGTTGAAAATGGTGTTGTGTAAACAGCCCTCAAGTTC<br>CTCTTGGTTTTCAGCTCCTTGTTGTGTTATGTT
LG8	ref-10740_32	67.14			GCGAAGCTCTCGACCGCGTTGCTAAATAGGC[C/T]

LG8	ref-29210	67.324	Chr7	3608351	CCCCCCCGGTGGGCGTGGTTTTCAGATTATAATAAATGCGCTCTGTCTCTATAGTTTCCCTAGCGTGTTTTACACAGCATTGAGTTCCTTTTTGAAAGGGAACGTCTCAGGTTACGCATGTAACCACGGTTCCCTGGGAACGGAACGAGACGCTGCGTCTCTTTGCCATGCTTCGGGGCCCGCCTACTTACAGTCCCTTCAGATGATAAGCGGATGACATGCTGTTTGGGCACCCCTTTTATACTTGCGGGTCACATTATGATGATGTTATGGGCTGTCGCCGGTCAATATGATATTGCCGAGATTGGATATGTGTTTCAGACAGCGGTTCCCGCGGAGGCGGTTCCCCTAACGTGTTTTACACAGCGTCTCGCTCGTATGCTAATTGTAAATCATCATTGATGATAGTGATCCTGACAGAATTAACTTTT
LG8	ref-56899	67.404			CTGTCGCTCATTCCCTTTTCCGTCATATTCTCTGCTGTGTCTGCCCCTTGTTGTCAGTAGATTTTTGTGTGCCTCATCTTATGTGCTGTTGGATCTGTGCTGTGTTTTTGTGTTTTACCTTTGTTTTCTACTGTGCCCAGGATGCCTAGACGAGGAGTTACACAGCTGGCTGAGTGCTCGCTGTTCTTAGTCTCGTCTCTTTCCCCGTCCCGATGGGCATGCACCTGTAGTGGTCTCCACAGCCTGTGTCCGCAGGTGTCTGTACAGACGGCTCTGGAGCTTGCACACATTGCGCTTGTCCATGTGTGTATCACTACACTTTGGATGTTGTTTATTCACAAATGTGTCTAATAAACTGTTGCATGAAACCTTTTGAGTCCTCCATTTCTCACATTCTGACACATCGGACACACCCAATTCAAGCCTTGGGG
LG8	ref-32838_24	67.451			GTGAATGGGGGTCAAGCTTTTGAAGCCCAAATAAATGCATCCATCCATCATAAAAATAATCCATAGAGCTCCAAGAGGTTAACAAAGGCCTTCTGAAGCAAAGTGTTGGGTTTTTGTAAGAAAAATATCCATATTTAAAACTTTATTAACTATAATAAAAAGCTTCCGGCAGACGGCTGGCATATGCATCGAT[A/G]TGTGGCGGAAGAGTAACCCCTGACCCGACGCATGATGCAATGATGAACGCGGAAACACAGAGGATAGAGCAAAACAAAACAGAAAAGAGGAGCTTGAGTTTGTTGCACAGCCCTAATTGTTTAAACCGCAAGATCTTACGATACTCATACATCCTACGTCATAGTACATTGCATCA
LG8	ref-55454	67.74			CTCTGATGTGTCGAAAGCATTCGAAAAGCCACTAAAGACCGCCTACTCTGTGCCTACTGGCCTAATGCATAAACATTATGGGAAGCACGCTAGCCAGATGGGATTTAAACTTTGTCGGCTGAAGACTCAAGTTTGGTTTGAAGGCTAAAAATGTACCAAGCACAATGTTCTCTCACCATTCCTGATTTCTAACAAGCACTCAATGCGTTCAGATGGTCTTGCAGCTGTCAGAGCAGAAACGAAAGCTATGCTCTCTGTATGTTTTCCGTCGTCTCTGGTAGCGTTCATATGTAAATATTATTTTGTCCATTAGATCGAAAGATCTTAAAGAACCCATACATTTACCCACCCATAGACCCTCCCCTCAAAGAAATCGGTTGAAAGTGGACAAAATAGATGGATTAAAATAAAAGTGTAAACGGGTATCTGTC
LG8	ref-43053_28	67.813	Chr6	27422984	TTCAAAATCCATGACCCTATTCAAGCAGCCCCTCTGCATTATGTTTATCATGACTTACGCTTATGTCAATAACTCTTGTCATGTTAAGTACATCTGGCCCAAAATATCATAATCATGTTCAGTAAACCTGATTAATGTTGTGGAGTATCTGTTGCAGACTGCTGATCTTGCTGTGCAAGGCATCCCTCTCGGTTCTG[A/G]CTTCGGACCTATGTATTTCCTGTGCAATAAAGCATTCAGGTCAAAGTGCAGCTTCTTGAACAGCCATGACACACACCAGACAAGACAACTGCATCCAGACAAGTCATTAAGGAAGACTACCCTTCCCAAATAATGATTCTAGCAACACCATTATTCTACTGTATCTCTGCTC
LG8	ref-3624	68.055			GTCCAAAAGACGAACTCATTGCATACATTGC
LG8	ref-28618_6	68.228			ACAGAAGGGCAGAGACACTGGACGTATATAAGCAAGGTGTTTATTTTCACAATCAGATGAGCAACATTTAAGCAGGTGAGTGAATGGTGAAACTTCAGGTGAATGGTGATAAATGGAATGGTAATACTGTCCATTGTTTTGTAGGTGAAGGATGAAGGCTGAATGCAGGTTTCCAGACACAACAACGGAGCTGGACTTCACACAC[A/G]AAGGCGATGAGTGTGCTGATGACGTGCAGGTGTGCGTGATTAGTATTCGGGTGACGGTGAAGCCTGGCGTTTCTGTGACAGACAGCACTACTTATAGGATATAACCGATGTGTTTATGTGAATATGAGGTAAGACATACAGTACATAGTGACATACAGTACATAGTGACAATATTTTGGTGTGTATTTGACCACAAGTGCAATAAGCCATGAAAAAGAGGCAGCTT
LG8	ref-24547_3	68.535			TAAAGGTGACTTGACTTGACATGAGCATTTGTCCTACTGAGAACAGTATTCGAACGATTAGCAAATGCATCCTTAACCATAAATCTGGTGAAATTTGAGTTTGGTAAGGCAACAATTACATAACTGGGTAAGTTAGTCGGTCAAGGTCAAGTGCAACCTGTCAGTGCAAAATTCACCGCTATTGTTGATTTTCCTGTGCCTA[C/T]CACACGACGAGAATTATGCCGCTTTTTAAGCATGGCAGGGTATTATCGTAATTTTTGTCAAAATTTTTCAACTGTTGTACATCCCCTTACCAGTGTGTTAAGTCCTGCCAATGATTTCGTCTGGTCTGATTCTTGCCAACATGCTTCTTGCCTTACTAATCAGTGCACCTCCCGCTCCGCAGTTCTCTCGTCCATTTAAATTAGAAGTGGAAGCACTGGCGCCGTAGTC
LG8	ref-2783	68.657			GCCCAACGAACGACTCCTGTGCCTGTTTGTG
LG8	ref-59821_3	69.087	Chr6	1740915	ACATTATACTGTATATTATTCACTATCAATCGCCTCTTACACTAAATGTAATAAAATCAGAATCATTCTCACGTTTTGGTGATTCCCTAGTATTTTTTGTTATTGATGTTTTAATCAGGCCACTTGTCCAATCGGCCAAGTAAAATTCTCTTTTACTTGCCCCTTCAAAAATTGTCGAGCCCTGCTCAAGAAAGCCTTCAAT[A/T]TTTTACCCGATATAGTTGCAGATCCGCTGGTGATCATGGCGGTCCGTCCCACTCCGAGACCCATTCCCATAGACATAGACGTCCCCAGCGTCCCTGTGCTGATTCCCCCGATACTGTCGGCTGCCGAGTGACCGTACTCCCCCTGATCAAAGTGCGAGTTATAGTGGATCCGGGGGAAGGTGCTGCTGGAGGACAGCTGGTTGTTAAGAGGCATCAGGCAGTCGGGTGG
LG8	ref-67683	69.27			CGGAATTTGCAAGCGGAAAGACTGAGCACTGAGCACTTCTCCAAAAAGGAATCCTTTTATTTTTAGTATTTAAAAATGTTTGTGTGCTGCTGCGTCCCTGTGTGTGTAACAAGCAGAGTGTACGCGTGTTGTGCGCCTGCCTATAGGTGCATATAGGTGCACACCTCGTTTTCAGACCAGCACGCCCATCGGGTGAACAGATGGCCGTGAGTGCATTTGCTATTTAAACAACGTGACGCTGGACGTGAAAATGATAACTGCGTCAGGCTGAAACTAGCAAAAAACACTTGTTGCGCCTGGTGTCGCATTGCGCCAGGTGTATGATAGGGCCCATTAACTTGCAGTTGCGAGTTATAAAGTCCGAATTGT
LG8	ref-40017	69.473			GCTTTGTGTTCGAACATGTTGCAAGTGTATG

LG8	ref-27381	69.706	Chr6	42188792	TCACGTAGCTTTAGTAATCCTGAAGACATTCAAGTGTTTGATCAGGGTTGTAGCTAAACTGCTGCTGCTGCACTCCCCTTCCTACGTCACAAGGGGAGCAAAATCAGAACGGCTCGATTTTTCACATGCTTGTAGAAAAAGGCTTACCAAAACAAAGTTACTGGGTGGTCCTTTTTCACATTTTCTGTGCTGGTAGATGTACCAGAGACCCGATTATAGTGCTTAAACACGGAAAAAGTCAGATTTTCATGATATGACCCCTTTAGGAAAGCCAGGTTCCTGCTTTTGCTTAAGTGTAAGGGGAGGTCACTAAATAGGCTACTTGCCACTTCAGCCTATAGAAGCCGTTTTTTTCCGATTTTGTTTTTTTCCTTCTGTTTTTTAATACTGCATACAAATTTCATGTATTTTTTGGTTATATTCTAATAATG
LG8	ref-35931	69.719			CATACACAATAATTGCATGCATTCAATTCATTTAACTATATTAGATTAATAATAATTGCACTGTTTGCATCACGATTTTAATGTGATTATTTGTGCAACCTACATTTTGAATAAAGGTTTTGGCAGAGAACACAAATCTGGCTTGTTAATGATTTTTTACTTGTTCCTCTGATTGAAAAACAATGAACTCGCTGCTGTGATAAAGATTTCTTTGCAAAACTCAGCGGTGAGTTCAAAAGCACTGGAAGCAGCCCAGATCTAAACTAGAACAAGCACTTTCAAGTTATTTCTGGCTATAGTTCAGCTAGTTTTCTTAATTAGTACAGTATAGACTCAGGTATTATATTGTGGCTAGCAGCCAAATGCATG
LG8	ref-27700	70.114			AAATTTAAATTATTTCAATATAGACACACACACACAAGGTGCAATAATGAAACTCTGTATTTCATATGATAGTAATGAGAATTAGGGGGTAAATGAGCATAATTTTCCTGAAAATAATAATTAATTTATTATTTTAATTTATTTAATTAAAATATCTTTGTCTGATACCTACGATGGCTGCATTCCACTCGACTTTTAGACATGCACTCGCAAACTTCCCAAAGCACTTCCCCTCAGGGGAATCCCTGCCGCCATTTTGTGCGTTCCACTTTGTGAAATGGATGAGGGAAGTATATATGGACAGACCCTCGCTCTTTCGATTTTGGCCGAGGGAGCAAGTCTACTTCATATGTACACTTCAGGCAGCTC
LG8	ref-45771	70.252	Chr6	5956210	AAGTTTATTGGGCCTAACTTATACATTTTCTAACAACCTTTATGTAAGTAGAAAATTTACCATTAAACCTTTATCTAGAATGCAGAAAGTTTTCCCCCCAACCAGATCATCATGTAGCATCACATGTTCTATATAGCTGACATTAACACATCCCTCCTGCTGTGCGACTTTCCAACTGTTTCTGCATTTAAAAATAGTCCTCCTGCTGTGCGACTTATATGCAGCTTTCCAACTGTTTCTGCATTTAAAAATAGATAAATGCATAATACTAATGAGAAAATGAACACAGAAACATAAACTATCAAGCAGTTTCCACAGTACCACAGTAATCTGACATAATAGTTTGGCCTATTATTCAAACGGAAGCAGCATGTGTGAGGTATGACATCACACTCAAAACTCAACAAACTTTTGTCCGTGGGTGCCATCCA
LG8	ref-4540	70.558			ACACCCTTTTCGAACACCATGCTAATTGACC
LG8	ref-67884	70.783			TTCTGTCGGAGACACTTCTGGAAGAGTTCTTGGTTCTAGTAGACTGAGGTAGAGAGAACGGCGTTTCCATGTTGCTATATATATGTAATCCATCCCTACAGTAAACACTTTAACTGCAACAAGTAGCAGTCACAGTTGTATCTTCATGTATCATGATTAACCGTTCCCATTTCTCGCTTTGGATAAAAACTTATTGCTCTACATTAATAATGAATACCGTGCGCGTTTTGTGCCTTATCGCGCTTTTAAATTCACGGAATTGTTATGGATTTGTACTAACATGGAGCAAGGTTTAACGGTCTAATATTAAATCTCGCGCCAAGCAGAAAGACGATTGCTGCTGCTCATGGGATGCGTCGTCTTAGATGCCTTCTCACGTGACGTTCACTCTCCCCATAGAGACAGAGCGCGTTTATTCTAATCTGAAAACC
LG8	ref-38161_2	71.275			C[G/T]GTAGTAAACGATCGTTGTGCGTGGAGTCGT
LG8	ref-47955_1	71.829			[C/T]TTAATGAAGCGACAAGAATGCTTTTTGTGCG
LG8	ref-47955_4	72.033			CTT[A/T]ATGAAGCGACAAGAATGCTTTTTGTGCG
LG8	ref-31917_8	72.985			GTTTGGGGTCAGATTTTTCTTTTTGAAAGAAATTAATATTTTTATTTAGCAAGGATGCTTTAAAGGGGACCTATTATGCAAAATTCACTTTTGCATGGTGTTTGGACATAAATGTGTGTTGGCAGTGTGTGTACACAACCACCCTATAATGATAAAAATCCACCCACTCCTCTTTTTTAATCCCCATAAATCATAAGCAGTGTCTCA[A/G]AACGAGCCGTTTGCAGGTTTCTGGCAATGTGACGTCACATTAGCCACAGGCCCCGCCCACGAATGTTGACAGACACTGCTGTTTTAACATAGAACCGCCCTGAGCGAGTTCACAGCGTGTTGTACGCAGTCCGCCATTACTGCACTGACGAGAATGTCTCCCAAGCATTTTAGGTGTTCTGTAGCTGGATGGATTAATCCACATAGCTCTCTCCATTTACTCCC
LG8	ref-36996_1	75.611	Chr6	5657560	GCCATGACAGAAAATCAAAGAGCAGCCATGGCTACCGCTTCCCCTGGGAGAGATTACATTCTGATGGGTCGCTCTGAAGTATTTGTGGATGAAGGTGAGAATAATACAATAATCAAGGCATTAGGAGCGCATCCTTTCCGGCTGGTCTACTGGTAGAGAACGACCCGTCGATACACACCACCTGCTTTCAATGAGTTGCGC[A/G]GGCTTACCGAACGCAATGCCTCCGCCGATAAATTTCCCTCATTCATGTTTTTAAATGCCCATTATAGTTTTAGTGGTTGCTGTGCACTGTGACCTCCTGAAATAGATAGATATTTACAATTCTATTCATCTTACTCTCTCTGTCTCGCTCCCTTTTTATCACTTTGCACATCTTATCAGCTCTATAACATTTGCCTTTTTATCTGCCTTTTCAGTAATGCATTTTCCTC
LG8	ref-21089	77.32			TGAGAGAACTCGATGCTGGTGCGCGTCGTGT
LG8	ref-44406	81.071			TTCTGAAATGCGAGCTCCATGCCATCACCGG
LG8	ref-4706	83.738	Chr6	3900335	ATAATTTTAGTCAGCCTCTCACGGACGGGCTGCGTCCAGAGTAAAGGGGGGAGATGAAGGGGCTTCAGTAATGTGTGCAGTGAAGCATTCAGCCCTGTGAAAAACAAACTGTTAACTTTCTCAATGGAGGGAGGCTGCTGGGGGCATGACGGCTGTGCACGAGTACGTATGTGTGTGCGGCATCTGTATCGAGCAGACGCAGGGAAAGAAAGGGTTAAAATTCCTCTAACTGTTCCTAAAAACTGAGGCTGGTGCACCTCAGATGCGTACATCAGCCTCAGTAAGTATTTGAACACTGATGCTACACTTAAAAATGTCGGGATGCCACTGCATTCGATACAAAATAAGAAAGCAAGTAACATCTGTGCT
LG8	ref-41858_2	84.201			C[A/T]CAGACGTGCGACAAGGCTGCATATCAACAC
LG8	ref-41858_3	84.517			CT[C/T]AGACGTGCGACAAGGCTGCATATCAACAC

LG8	ref-52359	84.934			AAGTTGAACACGATTTAAGTGCCACTCACTG
LG8	ref-68091_15	86.341			CAACCAATATGGATGTGCAACCGCAGTGTGAACAGAAAAATTAATGTACATTAATTAGTGAATAATCTCAATGCTTATTAAGAGACAACATATTTATAGCTACAATTTGTA TAAAAGTATCAAACTAGTGCTTAGAAACATTTTTGAGTGTTTAGTGGGGGAAGGGGGCACCAATAGGGATCTCGCATAGGGCACCAAAATGGTCTGAAACGAC[C/T]CCAA TGCCATTAATGCTCCTTGAAGTTAAATAAACACTATTTAGCAGTCTAACAGCATAAGCCAAGAGATTAATTTAAATCAGCACACTCGAATTCTGACCTGCTTTGGAGAAAT TAACACATCTAATGAAGCAATGTGGCATCACAGAAGGGCTTGACAGCTGTCATTTACACAATGCCTATTTTAAACAAAATCAGTAAAAATGGATGGTTGTTT
LG8	ref-70820_7	87.378			TCCAGC[A/G]CCCCGAGTGAGCTGCCTGGTCCCCC
LG8	ref-70820_3	87.524			GGCACCGTGCAGATACTCACTTTAAATTTTCCACTGAGGAGTCAAGATGGAACCCGGCTGAGCCAGAGGAGGTACATGAGTTGTGGTGAAGGGACATAATGTAACCTAGA CATTCCCTATCAGTCAGTCACATTCTACGTTACGTCAGAACGACTGACGAATTGGGATCCCTATGGAAGACCCCCCCAACTGCTGCCCTTTC[C/T]AGCACCCCGAGTGAGC TGCCTGGTCCCCCCGCACCTGCTGGGGTGGAAGAAAGGACAGGCCTATGCACGGAGAATGTCAATACTTGATGTTCCAATCAACCCTGTACAGTGAGACTACTGGATAAC ACTGAGAAAGTATGTCTATTCCAACTGGAATTCCAGCTGTAATCTTACCTACACCAAACTTTTGAACAAATAGCCTATATGTTCTAAAATAAAGATAGAGATA
LG8	ref-53917	89.13	Chr6	6993442	GCAGACCTGGTGAGCATAAGAGACATAAAACATAAAAAAAAATTTTACTGACTCCAAACTTTTGAATGATAGTGCAGGAAAAAGTATGCAGACATTCCTTTCAAATGAAC AGCTTTGGCTATTCCAGTATTTTCAGTGAAAACAAATCTAAACACAACACCAATAGTGTATATTTACATACAGTAAAGATGTCACACACCACTGCAGAATCGACAGAGCTG TTGGATTTCCTCATTCAGAATTTCATTTTCCTTTCATCCTCCTTAATTCTGTTTCAGAGCTCGCTTCTGCTGTCTATTTTGGGAGCCCATTTCTGAAGGTTTCAGGAGATTAGA GCTGTGAGGCTGGAAGCTCACAACAGCTCAACAGCTCGTCTGTGCATGACCACATACAGCTCACCAGAGACCACACATTTCTGCATGCCCAGACCC
LG8	ref-53852_18	90.338			AAACTATATAGACACTATATATTTTTTTAAAAAATGACAAAAAAAATGAAATTACTAAAACTAACTAAAATCAAAATGAAAACAGAAAATATAACAATAAAATTTTTTT AAAATATTAATAACGTTTTTTTTTTTGACAGCTCCAGTGCTTTTGTGAAAACTACAGATCGTACTACCACATTTTTGTATCCCCACTCGCGTAGTCTGATCGAGCCC[A/C]GT GCATTATGGGTGTTGAAGTTTTCCTACGATTTTTGAAAAAGGGAACGGCACAGTCCTTGTTCTCCATTTAATCTAGTCATATTGCACCGATTCTAAAAAAAAATTATGTTTC TACTTCATAGACAGCCAAGTTTCATTAAAAGCCCATCTTGCCAATGGTAAATCACCCCTTTAAGGGTACAAACAAGTTTTTTATAGTGGAAGTCTAAAA
LG8	ref-47458	91.058	Chr6	8405312	TAATTTAATTTCAAAAACCATAAGAAACTTCTTTCAAAAACATTAAATAATCGTAATGATTTCAAAATTTTGACCAGTGATGTATGAACAAGTCTATTGATGATGATATTCT CTTGATTCAGCCGCACTACTCCATTATAGGACCAACCTGTTCGGTGATTGGCCACAGCCAAAAAAGCTTCATTATTAATATGGAAGGCTTCCCAGTCACGAGCACCGTGTG TCTCCAGGGTCTGATAACGCACAAACTTCAGCCTCTTATTACTCCACCTATAGATCACAGAGAGCTCTCGCTGTCCGTCCGCTGGGCCCACCGAGTTCGATACCGCCAAGA ACATCTGGCACACATACAACACACATAGTGATAAATGAGCCTGATACAGACAAACAGAGATAATGTTACACTTATTAGTCAGTCTGTCATTTGGCTA
LG8	ref-64582	93.704			CATTTGTGGATCGCTGCACGCATTTGTGTCAAGACTGCAGACATATCCACAAATAGGTCGACTGACTCCAAGGCCACATTGCGCTGACCAATCGCAGCACACTCGCTACAA CCAATCACGTTTTGCTTTACAATCGTGGGCAATCACGTTGGCATAGAAAGGAAGTATAGAGAGTATGGACGTGAAGTCACCGTCGACCATTATGACTGCGGTTACACCCAC TGAGTGGCAGCATTGGATTTCAGCATGAATACCGCGAAAAACACATCCAAAACGGGAATGA
LG8	ref-38600	94.968			TATATCAAATCATTCTGTTATATATTTCGTAACATAGTTGAGAATCATCTTTATACAATGTTTGGTTAAAGAAAATCCTGAAACCTAAAAATTAATTGGTGAATGAAAAAAA TTTCCATTGTTTTTGCCTATACATGTCATTTTGTCACAGTTGTGTTCAGGCAGGATGATGAAGACAGGCGACCAGATCGAAATACTCAAAGTTTAATTACTAAATCCAAACA GGCAGGGGAAACACAACATAATGCAACGTAGAACTGACAACTGAACAGAACACACACAGTGAACAGAAGAACTAACGAGACTAATTAATAAGACACAGCTGAACTGAA GACACTAATCAAATGAGTAACCAAGGATACAAACAAG
LG8	ref-13147_3	96.355			TC[A/C]GTTAGATCGAAAGATCTGCAAAAGCCCAT
LG8	ref-38884	97.263	Chr6	10523675	TTAAATATGCTTTGTCTCCATAAGGGTTTTGTTTCCTTTGGAATTTTTGGGTTGGACGAGCACCTGATTATTCTTCCATTTAAAAAGAGGTGAATCAGCCGTGTTTGTGTTAT TAATTTATTATTTGTTTATTTTGGATGTAGTTTCAGCAGCTTCTTTGTGTGTTTGTAGGCTGGCTACTCTGTGTGGTTTTGGTCAGGATGAAAGCCTTGAGTCTGCTGCTGTTC CTGAGGAAATACGACTGACCTGTACACAGTTTGTGAGATACCACAAAGATCAGTGCGTTCAGGATATTGTTCGCAATAGAGGGTGAGTATGTGTATGTTAAAATGTTATAT TTGGTTAAAAAAAAAATAAAAATAATTTTAAACGAAATAGTGCCACAGACATATTTTGTTGATCCTGGAACAACATTGCTGCCCGAAAATGTA
LG8	ref-46429_30	99.203			AGCCCGAACTTGATCACTGAAACCTAATTTACCTACGGGTATAAAATAAAGGAACCTGAACCTGAAATATTACTGCCATGTAGAACCAGCACTCTTACTGTAGTACAATTT TAGCAGCATTTGTATGGGTTTGATCATACTGCTCTCTTTCAAATTCTGACAAGTTTCTCCGAGCAGCAGCAGCTGCATCGATCAGGC[C/T]ACCATCTCAGCATGAAAGCAA AAGTAAATCTGCTCAGGTACCGCTCGGGTTGCAATCTTCATTTCCCAGACATAAGTGCGGGAAATGTAAGTGTCCTGAGGTGAACTGTGTAATGTGTAGGAGAAGAGTAAC AGAATTTTGCAGCTTTTCCACATTGGTTGTCCCCTCCAG
LG9	ref-15578_28	0			CTGTCCAGTTCGACCACCATGCCCAGC[A/G]GTGA

LG9	ref-38777_10	0.612	Chr15	42996169	ACCACAGGTATCATTACGGTGCCACAGTTCTCCATGCACTGGCTGTGCGCACTCTCAAAGGAAATGCGGCTGCAGACGGCCAGGTCGTCGTCCTCCGAAGCCTCGGTGCCA<br>CCCACCACAACAGTGCGACGAGAATGATCAGCAGCATGTTTCTTCAGCACATTACCGGCCCCGATCATCATGCGTGTGGCCTGGACACGATAAAAGGC[A/G]CGACTTTTC<br>TGCTGATGAAGGAGAGCATAGTAGTTGGCCAAGTCTACCAGCTGATCCAGCTCCTTATCTGGATATTTCTGCTTCAGGTCCTTCAGGATACGGATCACCTGCCAAGCAACA<br>TTAAAAGGTCAAGGAAAGGGGAAAATATGGGTTGCCTGTTGTAATTTTAGTTCCAAGTCCAATTTTAGTTCCATCATCATTTATTCACCCTCATGCTGTTCC
LG9	ref-53765	1.472	Chr15	44699064	TTCCCCTTGGTTGATAACATGGGGATCCTTCTCTACTCCCATTGATTAGCACTTCAATCCTGCAGTACCTGCTATAAATTATCAGAGGAACATCAAACCAATCTTTACAGGT<br>TCATTAGAGCACACTTTACAGGCAAGAGCCTCGACTACCTCCATCTTTTGCTCGCTCTTGATTTTCTGCATCTCACTCGCTGACTCCCCCTTTAATGATTGTTGTTTGTTTCTG<br>TCTTTCCCTCGATGTTAATTGGATGTTATTATGCCTCTGCAACAACCATAAATCTTCATTTCCGCTGATCAAGTTCATTCATAATGTCTCGTGTCAGTGCAGACCCAGCCGCT<br>GAGACTCGATATGCAAATAGCATGCTTTTG
LG9	ref-56494_16	2.343			GTCTGAGCACCGATA[A/G]TTTTGCAAACACATAT
LG9	ref-56494_17	2.343			GTCTGAGCACCGATAG[C/T]TTTGCAAACACATAT
LG9	ref-40140	3.171			AATACGGTCTCATTCTGCTATATAATTCTTTAATTATTGTTGTTTTATAAGAATCATGAATTGACTCATTAAAAATAGGCTAAATTTATTTCAAAGACACTAAGTAATTTGTC<br>TTTTAGTCAAATATTTTAATACAGAAGCAGAAACAGATTTTAAAATAGATTTCAAAGGTGATGATCCTGCAGTTTGTCTGTTGATGCCAATGAAATGCGAATAATGTGCGG<br>AAGATGTGTCATTTCTGTCAGAATATTGACAGGAATCAAACAAGTATTCCAAGATAATCCGGAAGAATGAATTGCACACATGAGCCGACTGTTTTTAGTGAATCGATACAG<br>CCTAATTCACAGATAATTGACTCATAACAGCTGATTCTTTTAGTGAATCAGATCAGTAAACCCGGCTAACGGGGAACGTTCTGGCAAGGTTCTCGC
LG9	ref-62701	3.702			AAAAACTCTCCGAAACGTATGCGAAACGGGA
LG9	ref-15107	4.034			TCTTTGGCCTCGAGTTCTCTGCCACCGCCAC
LG9	ref-29087_28	4.376			CGACTAACAACGAACACTTTGCATTTA[C/T]TCTT
LG9	ref-50573	4.529			TCAATTAATGCGAACAAGGTGCATAAATGTG
LG9	ref-34765	5.385			AAAAAAACAAAAAGCTTAAGCGTTTATATGACCTTATATGTTCTTGACACTCAATAACATTACACAGACTCTATAAATAACAATGAAACAAATGCCATTAAAACAAGAAA<br>AGTCATTTTTGATTCGTTGTTCTAGAACAGTGCAGATTAATCTGCAGTAAAACCAATCGCCTCTAGATGGCAGTACAATAGCAGTTTTGCACCACGACTGAATCTCTGTTCC<br>TGTTTGAGAAAGTAAAAATGTCTCTCACACCTGTGAAAGCTTTTTGAGGCCAAAGCAGATTGATTTATTAAGCATAAAAATATAACAGCCATCTTTAGAATAAGGCTACTG<br>TACCGTTAAAATCCACAGACATCTGAAATAGTAATT
LG9	ref-8905	7.568			CTAATTCCCCCGAATGGTTTGCCCACACCCA
LG9	ref-163_18	12.012			ACAACAGTTTAGCCTAGAATACTCTATGCAAGTTCATAAACGGGCCTAATACTCATTTTTGTTGTTTGATTGGTTGCAGTTTTACTCAAGATGAAATCTCATTGGTCTAAAA<br>ATCTGCTTTTTCATAACTGTACATAACACATCAAATTATGTTCTGATTGGCTGTGATTGTGAATCACGCAGCAG[A/G]TTCGGTGTGGACAGAAAAATTGCTTGTTGGAAAC<br>TTGTCATAACTAGTTAGGATCAAGAGGGTTTTTTTCATTACCACACTTGTTCATCGTGATAGCAGTCGCTCGGCTGTCGTGAAAAGAGGAGTCAAAGTAGTCTTTATCTTA<br>GGTGTCTAACTACTATATGTCAAAATAATTCATTCTT
LG9	ref-39454	12.059			AACGATAGATCGATTTTTCTGCTTCAGCTCA
LG9	ref-28489	13.135			GTTTGAGACACGAGCCTGGTGCAAATTGGTT
LG9	ref-17883	13.489			TACCTTTATGGACCTTGAGAGTTTGAATGGCTTTACTCTCAATGCAGGCCTCACTGAGTCATCGGATTTCAACAAAAAATATCTTAATTTGTGTTCTGAAGATTAATGAAGG<br>TCTTACGGGTGTGGAACGACATGAAGGTGAGTAATAAATGACAGTATTTTCATTTTTGGGTAATCTAACCTTTTAAGGCCCATACCATTGATCCGTGACGAATGGTGTGCTG<br>TAGTCCGTTTATAGCTTACTGTTAGCGCTTTATATCTCATGACATTATTTAGGCTTCAAAATGTATAAATGTTGTTTTAACTCGTAAAGATTATCTTGATAGACAAAACATTT<br>AAGTGTCAAAACCCTTTAAACACAGAGCTTAAATCGCAATTTTTTTTCCAAAAGTCTATGGGAAAAATGCATAGGCTTTCGATCGAGGGAAACC
LG9	ref-24006	14.162			CGGTCAAAAACGACCAGCCTGCAAAAAATTG
LG9	ref-69755	17.029			AAGAAAACAAATGTNNNNNNNNNNNNNNNNNNNNNNNNNNNNNNNNNNNNNNNNNCTTTCCCCGCCAGTGTTTTATGCACCAGCATTTGTGATCATATTCACAAAA<br>CTTTCATGGCCCCCAGAATATTTTGTTGTATGAATATGTAAATGGTCAAAATTAAAAAAAAATGATCATGTTAGCTTACTTGTGCGTAAGCAACTTTTGTCCGACCTTTATG<br>CAGATTCTGTACTCTTTCAGAAGATGCGTAATAACGCCCCCTACCGTATAACAGTGAAAACACAAATTGTCGAAAAAAACTTGTCCATGGCGGAANNNNNNNNNNNNNNNN<br>NNNNNNNNNNNNNNNNNNNNNNNNNNNNNNNNNNNNNNNNNNAAGAGTTAAGAAGGTCTATTCTGATTTCTTGCTGAAAAAATGATTTAAAAAAAACAAAC
LG9	ref-27581	17.807			AATCCAATCTCGAGATTTGTGCCGTATGAAC

LG9	ref-13410_18	18.871	Chr15	45893426	TGTTTTATGTGCTAGTGTTCGGCGCGCTGGTGAAGGCTCAGCATTGCCCGGGTCGCTGCATCTGTCAGACCATCTCACCCACGCTCACGCTCCTCTGCGCCAAGACCGGACT<br>GCTGTTCGTGCCGCCGACCATCGACCGCAAGACCGTGGAGCTCCGGCTGACCGACAACTTCATCACGGCTGTCCGCAGAAAAGACTTTCTGAACATGACGAGCCT[A/G]GT<br>GCATCTCACGCTGTCACGCAACACCATCAGCCAGATTGCTCCCCACGCCTTTGTGGGTTTAAAATCCCTCAGAGCATTGCACATGGATGGAAACCGGCTGACTGTCATCAA<br>CAGCGACCAGCTGAAGGGTTTGATCAACCTGAGACACCTTATACTGGGCAATAATCAGATTCACCACGTCGAGCTGTCCTCCTTCGACGAGTTTGTGTCCA
LG9	ref-47363	20.963			GATGCACGTACGACGTCAGTGCTTGCCTGTC
LG9	ref-11913_7	22.022			CAGTCGCCACGTCCTTTTTCATGCGACTACAGAATGCGGTCTTGCATAGGTGTGCTGAGTTTTTTCCGCCCCCCTTTGAATATTTTGGCAGCCATTAGGGACCGTGAATTTCA<br>ACTTTTTTTTGTTAACTATTGACAATCAGACTCAACTGGGGAGCAGCGGTGCTGAGCGTGCAAGATAAGACATTTATCAGATGCTTGGCTCTGT[C/T]GTGCGAATCCTCTG<br>CCATATTAATAAAACACACACACATACAAAAAGCCGGATTGACTCTTGTCACGTGATCAGTGGACTCTGATTTTCACCTTGACGAGAAATCTCGTCACATTTTAATCTCGTG<br>GCACGAGAGCTCGTCACACCCCTACTGCTCATTAATAGTTAGTAAGGTAGCTGTTAAGTTTAGTTATTGGGTAGGATTATGGGATGTAGAATATGGTCA
LG9	ref-71967_27	22.617	Chr19	43682643	GATGTTGTTAATCCATGATATTTGCTGAAGCCGTCAGCCTGCATTATTTCAGCTTATTTCTTTTAATAGTGGAATTCCTGGTGAAAAACTACATTACCCATGATGCTGTACAA<br>ACATTACACCAATCAGAGAGTGGAAAATACAACATGCTCCAAAACATCTCTTTAGTTCCGCCCACTCCCATGAAGCACTGCAAATATGTAATCGAGTCGATCTCTATGCTT<br>TC[A/T]AATGTGTGTATGTATGTATGTNNNNNNNNNNNNNNNNNNNNNNNNNNNNNNNNNNNNNNNNNNNNNNNNNNNNNNNNNNNNNNNNNNNNNNNNNNNNNNNNNNNN<br>NNNNNNNNNNNNNNNNNNNNNNNNNNNNNNNNNNNNNNNNNNNNNNNNNNNNNNNNNNNNNNNNNNNNNNNNNNNNNNNNNNNNNNNNNNNNNNNNNNNNNNNNNNNNNNNNN
LG9	ref-69605	25.785			TATATAAGAAGAAAATAAGTATTTATGGACAGAAGAAATAACATTATTTATGGACAGATAAGATTTATTGTGCTTTATTCTTAGAGCTCTTACATCATTGAGATAACTTCCC<br>ATCCTCTCTTCTTCCAGAGGTGGACCCTGCTCATAGTTTTTAAGCTCCCTTTGCAAATCGAGTCGCTTATTCTGAATCTGAACAGTCAAAGTTGGAAGCGATTTCTGTGCCCA<br>TAAGAAAACATAGTTTTAGATTACAGTTTGTTTTTACAATCACTAGGACACATTGCTAAATACTAAGTTCATTTTTTCAAATCTGTTCACACAGTTCTCCTAACAAAGTTTTA<br>ACTTGGCATGTCAGTTCATTTCACATTCAAAATGGTTAACACTTTATTCTGATAGTTCACTTTAGAGATGGTACTAACTATATGTAACTTTGC
LG9	ref-70246	26.032			CTGGAATTAACGAGCATGTTGCAATGCAGGG
LG9	ref-40022	26.701			GTTTGCGACTCGACTATGATGCAGACACTTC
LG9	ref-67329	27.556			CGTCTGATCACGACCGCGATGCCACGATGGG
LG9	ref-24500	27.895			GCCTCTACGTGATTGCGTCGTGAAGCACGTGAGAAATCAGTCCAACATAACATGACGTGACGTCATAAAGTCAGCCGAGGAGGGACACGAGGCCCAAGAACAAACTTGG<br>CCCGGCCAGAATGGGGCTACTAACAGAAGATGGGCCCCATCCTTGCACACTCTCTCCAGAACTCCCTGAGGCAGAGCAATCGGGGAAATTACGTACAGACGATCCTCAGC<br>CATGTCTGTACCATGGCATTTAACCCCGGGGGGCTGAATGCAAGAAGGAGTACCACAGTGGACAGTGAGTCGTCTCAAGAAAAGATCTCGCAAACAGATCCACCCCTGCA<br>TGACTACTCCCATAGGAGCTCCACTGCCTTGGGGTGGAGT
LG9	ref-21160	28.081			TTAAAACTTATTTACCAAAGTGCATGATCTATTAATACATTTTGATGTTTGTGTCGCATTACCAGCTCCATGTGTATGACTTTTCTAATGCAGTGAACTGAATTATCCAACCC<br>AGGCTCATTGGAAAAAAACATGCTGTGGTGGTGACATTTCTCGAAAACGACATTATGTTACTTCTTGCACGTTTGTCGATAGAGTGGCACTAAAAGAACATTTAGTTTTATC<br>TGAAAGCTGATTAATGTGAATTTAGGAACTTTTTATTATGTTAGTTGTTGTATGTATCACAGCAGTTCGGAGCTGAATAAGTGTGATGAGTGGTACTAAAAGCTCAGTCACA<br>TTTGCGTTGCATCAGCCCCGTACATGACGTGG
LG9	ref-4835	28.245			ACAACAGGGGGGAGTAGTCTGGGTACGGGCCAAAATTTCCGAGCTCGGAGCCTTCCCCCAGACAGCACGCCAAATACACATAACTCTTACTGTACTTAATTATATGTAAATG<br>TGAACTTGTGAAACATAACATGAACACACAGCCAGTGAGCTCACTCACAGACCGCATGTTCACACAAAACACGACATGAAAGCACACGGCAGATGTAAACGAACCGCGT<br>GCTACACAGAACACAACAACACACGCGGACAGAGTGCACGGCCCGAGCGAACTTGAAACCGTACGCTCACACAACAGGACAGAGTGCATGAGAACAAGCGAATGCTCAT<br>TCTGTGCGAAACGAGACAAACACATGGAGCATGAATGTCCGAGTCCCGACACAAAACCGAAATCAAACTAGACCGAAGTGCCGGGATCCGAACACCACGCCC
LG9	ref-14303	31.032			AGCCGTAGAGTGGAAACACTCTGTTATCCTTGCACTACTCTGGGATCTGAGCATGCAGCTGGGGTTGAGTTGATGCCCGGAGGGAAGAAGTTAGTGTCAGGAGAGTTTAAT<br>GAAGAAGCTGGAAAGTGGCGCTTGTCATCGATCTTTACACATTGAGAACAATGGATATGCCAAAATAGACCCTAGCTAGCCCACTTACAGCCAGAACCTCTACTCTGTTGC<br>AGGCAAACACAGCATAAACTTGGTCATAATCAATTTTTCGATTAATTGTTTGTTGTTGTTGTTGTTGTTTTTTTAATGTGGTCGATTCGATATCGATTTTCAAAGCCATGAAT<br>AAAAAATTTTTCTGGATTTATTTCCGCAAACGTTGAACATAATATCTGATTGACGTTAATCTTATTACTAGTCTTCTCCACTAGATGTCATCCTCT
LG9	ref-36558_10	31.863			TGCGTCGGT[A/G]CGAAACAGCTGCATTTGAACAC
LG9	ref-36558_16	31.863			TGCGTCGGTGCGAAA[A/C]AGCTGCATTTGAACAC
LG9	ref-16675	33.822			CGCCTCTCCAAAAATGTAAGAAAGCGTCAAATTTACGTGGACCGCAAGCACTGTGATTGGTCTGCTAGAACCCCTCTCTCAGGTCAAAAAACTGGTACGGAGTGAGCGGA<br>GAAGAGTGAGCGTTTACAACATCCATAGGAATGAAAAACTCTTGGCTCAGGGGACACGTGCCGTCAAGTTGCAACAAAGTCGTTGCCTATTGGCCGCCATCACTGCTAAA<br>GCTTCTCTGTCGACTGCAGAGGCTCCGCCGTAGGTCCGACGCTGAAGTATAAATCAGCCTTTACATTTTGGACAGGTGCACGTCAGGCTAGGCCTTAGGCTATGTGTGCTA

					CACACAGCCTGTGCCGTAGCTACAGCATATACTCGACG
LG9	ref-67046	35.472			GTGAGTTCTTCGAAATAGATGCTTGCAGACA
LG9	ref-44097	35.69			ATATCCTTCTTGTGGTTTGTTACCCACAAACTAGGAAAGACCTTCAGTCACTTCTAACTTTCTCTGAGTGATCATTTTACATGAAAATATATTTCGCGATGCAAAAATGTGGTGGCGATACTTTTACGATATGACAGCAGTCTAATCCTATTGGTTGAGCTGCCGACGTGACCTACTCCGCACCAGCATCGCGTGTTGCAGAAATCTCTTGAACTGAACATTCACGAGTTCACACTTTACAAATAATCAGATAGTAAATAGTAAGCGTAATAGTATGCGTATTTGGCGTGCTGTCCGAGGAGAGGGCTCCGAGCTCGGTATTTGGCCCAAGCCCAGAGTACTCCCCCCGTAATCTGGTTAGGAAAGTAA
LG9	ref-25213	36.658			AACCCTGGTGCGATTGCTCTGCGGACCGAGA
LG9	ref-5100	37.182			CGGATGTGAGCGAGAAATCTGCGGGAAATGA
LG9	ref-73168	37.787			CACATACACCCGAAGCACGTGCCCTTTTCAC
LG9	ref-8653_28	38.042			GTGACACATACCTGCACATAATCATGGCGCAGATCCTTGACTCTGACACTGTTCCTCTCAAATAGCACCCTGTACACCTGCTTCATTCTGAAGTTATGTTTCTGTAGGATGCGACTGTGTAGAAATGCGGACCCTGTTGATATTGTTGAATACATTTCTATTTGCAATTATGCTTTGTTGCAACGAAGTCGGATTG[A/C]ATTATTTTCTCGCACCATTTAAATGATGGAAAGCTCTTGTTGTTGAGTAAAGAGCTGTTCCCTTCCACCTTGAGGTCGTCCAACCATTCTGTAGAAAATGCCACCACAAGATGTCATTGGTTAGGTTTTTTTCACATACTGTACTTTCAAACTGTACACAGTACTGTAACAT
LG9	ref-32983	38.172			AACTATGTCCCGACTGTCTTGCCTGCACTCG
LG9	ref-9806	38.301			ACTTTACTGCCGATGGTTTTGCAGTCCGTTC
LG9	ref-30890	38.372	Chr7	51281163	GAGTTACACTTTCATAAACTTTTTTATTCTCCTCTGATTATATAATGGTAAATATTACAATATCATAGCACAACAATAGTGATCATCAATAATGACAAGCCTAATACTTACAATAAAAAATAATAAGGTCATATAGATCATACAGTGATAATCCACATACAAGAAGTGGATTATCCTTCACCTGAAATGTTTGTCTTTTCATCCTGAAATGCGAAGCAAATGCTGGATCACAATAATTAGGAATCACAGTTTCCACAAATCCCTTCACTTGATTGGGATGTTCTCTTTTATTCTGGATGGCAAGGGCAGTGACTGTAACATTGAGCGTATTTTGCTGTATTAAACACGTATTTATACTGATTTCATCAGGTTCTGAAAATTCTTCAAAAAAAAAAAAACACAAAGAATGGCCTTCTCAGCTGCCATAGTTGC
LG9	ref-8273	38.548			ACCTGGAGATCGATGGGACTGCTCTTCAGGA
LG9	ref-66773	38.625			TGTACTCATACTAGGCGCTCTGAACCGTGTCCGAGGGCGTTTGACCCCCAAAGCCCGGTTCGTTTGACAAGTGTGATCGCTCTGTTCCGTGCCCGGGCGCGGCTCATTTAGCCGTCCCTGGCCCACTTGGAAGAGGTGAGCCAAAGCACGGTTCGGTTGGGCTCGAGCGCGGTTCGTATGCAGTGTGAGCGCTAACTGTGCCAGAGCACGGAACAGCTACTACTTTTATGTGCGCTACTGACAGCAAACATCTTTATTTCTACTTTGATAGTACACTTTTCAATTCATGTACTTTACTTGAGTGTATTTGGGTTTATAACGGGTCTGGTTCTCACCTTATTGATGAACAGGAATTGTTGTTTGCAAGTA
LG9	ref-72355	38.745			CCTCTGCCGCCGACTGGGGTGCCACATACAA
LG9	ref-35965	39.483			AGTCTGCACACGATGTACCTGCAGCACGCAA
LG9	ref-51596	40.215			TTCTTGCGCGCGATCCCAGTGCTCTCAGACA
LG9	ref-309_28	40.335			CTTGGATTAAAATGCTCCATTCATATGGATTCATTTTATTCGGGTGAACTGACAGAAATCTCTTAATTGTGTTCTGAAGATGAATGAAAGGATATCTTTAATTGTGTTCTGAAGATGAATGAAAGTTTAAATGATGACAGGATTTACTTTGTTGGGTGAACCGCCCCTTTAAGCGTGTGGCACGCAGGTCGTGACC[A/G]CTGACCTCGAGCAGCGTAGGCCGCAGACAGATCCTCCTCTTCCTCGTCCACGCGTCTCCTCTCCAGTGTGTTCGGTCTGGAGATCTTCTGCCCTGAAACACGTCAGCTTGAGTTAGTCGTGTTGCTCAGGTGATGATGTTATATTCCAGATAAAGTGGCAGAAGGCTGTGGAC
LG9	ref-28166	41.027			TTTTTTTCCCCGACATCACTGCTAATGATGC
LG9	ref-48240	42.04			TAAGGTGAGACGACCCTGATGCAGGTAGAGT
LG9	ref-48930_2	42.113			AACTTAACATTCTGCCAAGATAACCAGCTTATTTTAAGGAGATTTTATGCCTGCTACGTGTCAAAATAATTTGGTCTTTGGATGCTGATGCGCTGGGTTGATGAATTTCCTCACTGAAATGGCAGCATGGAGCACAAGGAACAGAAAGAGAGTGCAATCCCGAATTCAAC[C/T]GTTTAGCAGCATGCATTTCGCTTGAACGGTCAAGGGAAACACATCTGATGCATAACATTAAGCATGATTTTGCATCACATAACCAGCTCCATTTGACTTTTCTGACATAGCTACTAAGCTGGATCGGTAGCTCAGCTGGTATAGAACTATAGCCTACTTGTGATGCTGAGGGCTCGGGTACCGGACGTTTTAATTCAGA
LG9	ref-22641	43.342			TTGTGCTGTGCGATAAAACTGCACATTTTAG
LG9	ref-55754	43.531			ATACTGTTCTCGAACCTTTTGCACACTTAGA

LG9	ref-21322	44.236			CAAGGCCTGAAATAAAAATTCCTTCTCTCTCCAATACTCCTAGATCTTTCAAAAGGGGTTCTAGCACAACTGAATATCCATGCTGTTTTACATCTTCCGCTTTGCACAGAAC<br>TGCCAAATAAATGGACGTCAACTCGGAACGGAGTAGTGATGGTAGATTTCCTAGCACCCAATATACTGCAGTGATTTTATGTTTTTCCAGGACGTTCCGAGTGGATTGCA<br>AATCTCAAATTCATCAATGTAAAAAAAAAAAATCAGGGAAATTTTAACTTCTCCTTCAGATAGAAAAGGGTTTTGCTGAAAATATAGCCCATCCTGAAAAGATCTGCCGCT<br>GACACAGTAAATAGAGCACCATAATGATAAAAAAAATAGCAAAACCGTCGTCTCAGCCAGTATAGCGGTCACGTTCTTCTACGAAGAGGAGCTGGTG
LG9	ref-32336	44.468			TTGGAATTTGGAATGCGCGTCTGAAAAGTGTCCCATCTGTTGTGTTGTTCGTAAAGAGACAGTTCTATATAAACGCAGCGTTTTACTTGGCGATGTTTTAAAAAATATTTTT<br>ATTTTTGGTATTTATATGCTCTGGTGGTGGTTTGGTTCACCGGGGATAAAAATAAATCAGCAGAGGCAGGGATACATAGACCAGAAGGGGGGAAATCCCGAGAACACTGC<br>GAAACACAAGATCCAGGGGGACCTAGATCCAACTCCTCCCACTTTACATATCCCTAAACCTACCCGTCTCCGCCCCCTGGATCACGAGTGTTCTGCAGTGAGGGGCTACTG<br>GAAATCCCCACCATCCTCCCTACAAATCCCCACCATCCTCCCTACAAATCGTACCCTGTATATATATACATACATACATATATGTCCTGTTGTCCTCT
LG9	ref-28493	45.136			AAAACAGACACGAGGTCAGTGCTCAGACCAT
LG9	ref-67896	45.822			ACAAACTTGACGATGCTAATGCACCTTTTAT
LG9	ref-44501	46.249			TATAAACATTTTAAATGTATATACAGGGTTCATACACATACTGTAAAATGAAATTTCAGGACTTTCGAGGACTTTTCAAGCACTACTTATTTTTTGTTTTCAAGGACTACAA<br>TCAGAGATCTCACTTATCAAAACAGTATCTTTATTATCTTTCAAATTCCAAAGAAGGTAAATAGATAAATAAAAATATACTAATAAAACAAAATGGTACAAATAAAGTGCA<br>AGGGAACATTAAAAAACCCTAGATCTTTCAGTGCATAGAAGAGCAAATAGGAAGAGGGCATAAACTTCTTACACGATTCTTATTAAAAGATGTAATAACAGAAGAAAAA<br>GGTTTATAATGGCTATTACTGTTCAATAAATAATCTGTTATATTATAATGTTGGTGTACTTGGTATTGAGAAACGGTCTGTATGTTAATGATGGGGGTT
LG9	ref-49384_7	46.444			TGACTTTAATGAGATTGCCCTCAAGGACATTTTTAGAGGGGTCTCAATGAACCTATCTGTTCATGGATGCCAGGGGAAAGATCCATTTTTCCTTGGCCCAATACATCGACCA<br>TGCCCTTCTGCTTAGTGGTTCCCCATTTACTGTTGGATTTGCGAACAAGGAGCCCCGTAATCCCACAGTACCCTCCACACCAGAGTGTTTTCACG[A/G]CCCGACTGTCATGC<br>CAGGATTTATTCACACCATGGCCGCCACACCTGAGCCTGTTCACGTCATGCCTGCCAAGCCTGAGTTTCTTCACATCATGCTTGCCAAGCCAGAGACTATTCACACCATGGC<br>CGCCACACCTGAGCCTGTTCACGTCATGCCTGCCAACCCAGAGACTGTTTATGTCATGCCTGTCAAGCCTGATTCTCTTCATGTCAGGCGTGCCAATC
LG9	ref-13121	46.896			TGTTTGTAGTCGAGTACATTGCCTGTTGGAA
LG9	ref-49097	47.166			GGGTCGTCTTCGATTCTAGTGCACAATATGA
LG9	ref-71571_29	48.277			GCCGTGGGAACGAAGCGAGTGCTAATAA[A/G]CAT
LG9	ref-51830	48.284			AAGTCCGAAACGATAAAGATGCATTCAATCA
LG9	ref-71679_16	50.196			AGTAAATGAGCATGAGGAAAGACTAGATCCTCAATAAGAGAGAGAGGGGGAGGAAAGCATGACAGAAAGGTGTTTCTGTGTCTGTTAAGGGTCAGTTCTTGGGAGGAAT<br>CTTCTAGTTTTGACACTGTTGACCTCTGTGTTTGGCGTGGGCAGTCACAGACTAACTGATGATGTCAGAATGTCATGTTCTGTTGTAGAACGGCTCTATTGCGAAC[A/G]CGC<br>TGCATGCCTGGTTGCGTGTGATTAGGAGAAATGCAAAACAAACCAACTGGAGGCTGGAGGTGCTTTTTCAACCTGCGTGAAACATCCCTTCCCCCAACACACACACATACC<br>TGAAATATATATGTATATACATTTGCTCTTTTCTAGTGAGCAGTCTACACAACACAAGCAGCTGCTTTCTAAGACAGAGTTTGATGGTAGCGTTTTTGCTAA
LG9	ref-47815_32	51.834	Chr15	35733638	ACCCGTGAGCTTCATTAGGAGTGAGTTATGAACGCGAGCTGTTTTCAGCAGGTGTGTCTGACAGAACCCAGTCTCGTGGCACCAGGAGTCCGACGGGACGTAATAGAGTC<br>ATAATGTGACGGCTCAGAACGAAACAGACTGGTTTTCATTTCAAGATTGATTAGTGCACGAAAAGTCATGCAATGGAATCGGTATGAACGTT[C/T]CATGGTATGATTTTGA<br>ACTCTTAATTTGAGGATAATTATTAGAGACGGCTGCAAAAAATAATGCTAAAATAAAGTTTTATTGGAATACGGTAATAGAGCCCACACGGATGCAGTTTTGGTAACATTG<br>CTAGAAGTGTTCAACAATTACAGCTTGCTAGATTTGATCTG
LG9	ref-2736_14	51.836			ACACACTAAGTAAAGTTAAAAAAGTGACAACATAAACCACCCCTTTAACAAAGATATTAATTTCTCTCTCCCTGTCACCATCCTGTCACTTCTGTACTGTGCTATGTAATAA<br>AACAGGACAAAGAAAAAATATTTTAAAAGATACTTACAGTAAGCATGCATTTGTAAAACGAGCAAAACACATTGGGAGGGGAAGACAACGAATCTTCACGA[C/T]GCGCT<br>TGCCATTTCCTAAGAGTACAAGTAAAAGAATAACACTGAAGAGAAAGGAAGACACAGGACATTTGATAGAAAATAGTAAAGCAGTTTTAATTACTTGTATATCTGAAACC<br>TGTTGTGTTCTCAGAATGTTTTTTTTTTTTTTTGCACAATTATAATAATGTGGTGTTAAAATCAACTCCAAATAAAAGACATTAAATCTCTCAAAAGATCTCA
LG9	ref-23859	52.403			AGCATCATTAAAATGACATTGATTTTTTAATTGTTTTCAGTTCTGATTTATATGTATTTTTTCTTGACTGCAAGGTCTTGGATTTGCAGCAGTACAAGTAAAGCAGTACTTTC<br>AAATGAAATATCAAGCAGGTGATTCAGCAATTCTCTATTGCAACCTTACCAATGACAAAGAAACGGTTAGAAGATGTACAATGATGTGGCAGATTTACGATGAAAATGCA<br>AAGACCCATGTACGTATCACACAAATTCCAAAATATCACCAAAGAGTCAACATTAAATCCCAAGGCACTTTCACTTCCATGACAATAAAAGACCTGATGGTTACAGACAC<br>AGAAACGATATTGTGTTCAGCAGTATGTTTTGTTAATGAGAAGATACAATTCATCATCGATAATGGGACAGTTCTAAAAGTTACAGGTATGTTTGAAA
LG9	ref-45697	52.674			GTATGTCAGGCCAAGGCACTCTTAATGCATGAGGGTAGTTCTGAGCCGGGGTTGAGCTGCGCTTGTTGACTAACCATGATCAAAGGCCCTCGGCCAGACGATGACCTTGCG<br>GAGGTGCGAGAGGTCGTCAAGCTAAAAACGCTTTCTCGATGCTCCCATCTCACAAGATGGCCTTTTTGGCAACGCTGTCGGGGACTGAGCCCAGCAGTTCTC

LG9	ref-30930	53.118	CTCAGTACCACGAGGCTGTTGCCCGAACTTT
LG9	ref-32591	53.372	CCGCTCTCCCCGAATTCCCTGCACCTGGTCT
LG9	ref-65043	53.415	CCACCGCTGACGAGGCTTATGCTTCCGATGC
LG9	ref-9238	53.712	CTTCAGTCAAACTGCCAGTTCAATCTCCATACATGACCAATTCTACCGGTTTTGTCAAGGGACATCCACAGTTAACGACTGTGCTCTCCAGTTCCACACTTTGGCTCACATA CGAAGAATAAAATCCACAATAGTTTGTGCATAGACTGTGGTGCAGAGGGACAACTTCTTCTGCCTGTCCAGTCAGACCACCATGCCCAGCGGTTAGTACAATCCATATGCT TCCTTCCGTTTCCTCTCTTGCATGTATTGATGTCGTAGTAAATTCTTTCAGACGAATACAATCGGAGATATATTAATAATCACCCTTACGCTCCAAAGCTTTATAATGGCAGT GCACGGAAACCACCTGTTTGAAGCTCAAAAAAAAAAAAAATGCATCCATCCATCATAAACGCATGCCACACGGCTCTGTGGGGTTAATAAAGACCT
LG9	ref-41562	53.728	TTTTTGCTGCTAGAGGTCACTTATTCAAAACAAAAGCATAGTTTGATGACGCTGTGAGTGTGGAATCATGGAAGTTGTTGTCTTTATCCCCACAGCCGAAGCAATCCGATTA GAAATCATGTTCATGAGCTAATGTATTAAAGATTTATTCACATCACTGTATGAAGCAGGGTGGAGCTGAAAGCAGTAGAAGCAAAGCCGCGAACCTGACAATGTCACTGC AAGTTACTTAAAACAGCTTAAACGTGTTTTTTAGTCCAGGACTAACTTAAGCCTTATCTGTAAAACCGGGGGATACCCAGGGGATGTTTTGATACCCCTACTGAAGTCACA TGGAAACCATTAAAACATCTGAATCTGAAGACTGATTGTTCAGAAGACACAATGGTAATTCATGTACCAGCAGAAGGTGACATGTTTCATGAAGACAA
LG9	ref-3012_28	54.154	GCTGTATGAACGAAGTAGATGCTGACA[C/G]ATTT
LG9	ref-31484	55.442	GGCAAAAGATAACAGCGGCAGTAAATGAAGCTGAGGGTGGACTAGGAAATCGGACAGTCGATGAAGTAAAAAAAAGAAATGGAAAGACCTTCTTCAGGCAGCCAGGAG AGACTCTAGCCTGCTCAAAAATCCCCCTCCTAAAATCTCACCCTACAGTAGGCTACTATTATGATGTGTTTGGAGAGGACTCGCCAGCTTTTACCGACCTAAAAGGCTGTG ACAGTGCCAGTTACGTCTAACGGAAGAAAAGCAAGCTGAAGCTGAAGAGGACTCAAGTTTTTCTTCCATAACAAATGTTTCATCAGGCACCAGTTATGAAGGTATTTACTA ATACATAGGCTAGTTTTAGGCCTGTTTACACTATGGATT
LG9	ref-11874	56.499	GCTTTATATTCATTCCCTGAAAATGGGCTTTATTTAGAATTTTTCACCATTGTTACAATAATGATAATGTCCAAAAGACATGATATGTCTTGATATAACCCATTGCGTTTCTA CTAGTAGAATGAAGATATAATATAGCCGTATTATCAGTAGTGTATTCGCTCTTGATTTTAAATTTGACAAGCCCCTTTTCTCTATGGTACATGCCCTCGAAACTGATGCAAA ACTTATGCATTTTTTTCAGTCAAAAGCTCTAGAAGAGCGCTCTCTAGTGTGTAGGAATCTTAACAATCCGTGTGGACCACACAAACAAGTGGAGTGATAGCATGGTGTGTC CCAATTCGTGTACTTAAGGACTATTCTATGTCATTTTATAGCATAAATAGTGTGAAGAGCATTCACACTAAAAATTCCAACAAGAAAAGGTCACT
LG9	ref-30012_1	56.724	ACCCTTACAGACAAATCTTCCTGGACTAACTCTGTCAGAGCTCCATCCAGGGATAACAAATGCTTACTTCCTAATTCTGCCAGCTTTTTCAACCAGACAGCAAGTATGTAAG AATAGGGGAAATAAGTATGCACAAAATAAAATTTCTGTCATGACAACTCTCAAAGTGTTTGGCATGGTAATGGATATGACATTGTTGA[C/T]AGGTCTTGGCGAAAGAGCT GCTGTACAATATATGCTGTACAATATAGCATCTGCTATTACCCATAGCAGATCTGTACATTTATACAGGTGGAGCTGGGGAAGGTGGAGGTTTCCTGAAAGCACGCTTCAA ATGCTGACCAAGTATTTGAAGGTTGAACAGCGAGTTCATTGGCTACTGATACAGCAGGAACCAATCAGCTGTGCCCTATAGAGAATGATGTGATTGCAAGC
LG9	ref-27921	57.82	TGAACTTAAACGAGCAGCCTGCAACTACAGT
LG9	ref-9287_19	58.22	GAATGCCGCTAAAGGTACAATGATGGCCTTTGAATACATTGCAGAAATGCCTAATTGTTGTACATCAGTGATATCCAGCTGATATTCCTGTAGCTCAAACTGTAGAGCATG ATGCTAGCAACGCCAAGGTCATGGGTTTGATTCCCAGGGAATGCATGAGCTGATAAAATGTATATCTTGCACGCAAG[A/G]CGTGATGCCAGTGTAATGTAAAAACGGGAA ATGAGATGCATGGCTAAATATCCAATGTCATCTGATATAATAAATAAACAACTGCAACATTGGATGATAGCCCTCATAGTATTGATATTGTGCATCTCTCTCATTGAAATTT TCCTTTAATCATCAAAGGTCTTGTCTTTTTCTTAGGTAC
LG9	ref-27393	58.814	CAGGACAAACCGATGGAGATGCTCCCGGAGC
LG9	ref-58988	59.017	CTAGCCTCTCCGAAAATATTGCACCTTTAAC
LG9	ref-52047_27	59.112	GTTTCAGACCAGCTGATGGAACTCACGATTATCTTTTCAGGTAGGATAGATTCGGGCTCCTCAGTGTTCTCTTCAGGATTATAGAGACGAGATAAGGCGTCCGCCTTAACAT TATTGGGGCCTGGACGATAGGAGATGAAGATTGGAGTGAAGTTAAAACTGGTGAAGAATAGCGCCCAGCACGCTTGTCGAGGA[C/T]TCAATCTCTTGGCATCTCTGAGAT GCTCCAGGTTTTTATGGTCAGTAAGAACCAAGAAGGGATGCTGTGCACCCTCCAACCAATGCCTCCACTCTTCCAGGGCCAACTTGATGGCTAGAAGTTCCCTGTTTCCGAT GTCGTAGTTTCTCTCCGCCGAGTTGAGTTTGTGTGAGA
LG9	ref-36604_26	60.217	CGATTCCTAGGGTTTGCCAATTTCTACCACAAGTTAATTTCCAATTTCAGCAAGATTACCGCTCCACTCCCTTCTTACCTCCCTTCTTCATACCCTGCCCAAGTCTCTGTCCTG GAATCCTTCTGCCACGGAGGCCTTCAAACTTCTGAAGGATGTATTCTGTCACGTTCGCACTCTTGCTTATCCCAGTCCTGAACTACCATTCATCATCGAAGTTGATGCCTCC[C/T]CCAGCGGTGTGGGAGTGGTGTACTATCACAGCGGCAGGGAGATCCATCCAAACCTGTGCCTTCTCCAAGAAACTGTCCCCGGCAGAGCAGAATTAAGACATCGGTGA CTGCTGGCGATCAAATTAGCACTGCAGGAGTTGAGACATTGGCTGGAGGGAGCAAAACATTTGTTCAAGTTAATCACTGACCATCGTAATCTGGAGTA
LG9	ref-56724	60.812	CGATTCGATTCGATTCACATGCTTGCGATTT

LG9	ref-72936	61.456			AAATGCTCCTCGAAATTTGTGCAAGTTAGAT
LG9	ref-34754_17	61.869			GGGAGGCCCCCGACCA[C/T]AGTGCACAGGACAAA
LG9	ref-46657_4	63.2	Chr15	30573058	TACGTTAGAGCAGACTCAAGGTTTGGGCTGAGGTGAGCAGTCAGCAGACTAGAGGTGGATAATTAATGAGTGATTGGAGAAGGTCAGAGCAATGGCCTGTGAGAGAATC AACTTTCCTTTCTCATTTCTTTTATTTATATTTCTTTATCACTTTCTCAACTCTTCTTAAATG[A/G]AGGCTGGCATAGACCTCGTTTGTTTTGCTGTGTATATCTGAGATGCTC TGAAACTAATTAAGGAAGAGGAAAAGGAAGAAGAATCGAATATAGTACCTCACCAAGGTTGCTCTAATGAGACGTTCTCTGGGCTGATTGGTTGTCTGATATAAAACCCT AGCAGAGGGGCTATATTGACTATTTGTAAACAGTTAGCA
LG9	ref-4480	63.249	Chr9	9791911	TTCATTTTTATTTTGGAAACCTTTTATTTCATTACATTTCAAAGAATATTATTGTACTTGTTTCATACACATTACAATTCCTCATTATTTTGAGCTGAAGAAATCGTCACCCAC TTCGAAACCAGATAGGCTGTGTCCGATTCGTGAACAAGCTGATTCTTTTCAATTAACCTGTTAAATTACATTGAAAGATTCATTCGTGAATTGGGCCGATCTGATTGCATCT GTTCTCGAGTCAACAACTCACTGATTCAAATGATCTGGTCAGAGTGAGTCTCAAGTTAACAATTAACTGAATTTACAAGTTTAACTCAAAATGAGATGAGAACAGAAGATC CTCAGAGCTTGAAGTAACTTAAAGGATTAGTTCACTTCAGATTTAAAATTTCCTGCATGCGTGACCTTTCCAACATCATTACATAATGTGTGGC
LG9	ref-54781	63.877			TTATTAAATTATTTATTTTTATTAATATTCATTGATATTGATTATATGAGTGTATTGGTACATTACACTCATATACATTCATAATATTAATAATAACTGCAATAGTACTGATA TAGAGCTCCGGAGGTTGACGTCACGCAATCTAAACTAGAGCGGTAGAGCGCATTGGTAGCATTGTAGCATTGGTATCGATAGTGGTCGAAGAATAATTTAAAACCAGATT TAGACCTTACAAACCTTACTATTTCAACATGGTGGACAGCTGCTGTGTGCCAGGATGCCAGAACGGGCGGGGGAAAGCAAAGGGGAAATCATTTTATCGTATTCCTAAGG ATCCCGGTAGATATCTGAAATGGTTCACTGCAATAA
LG9	ref-50537_31	64.175	Chr15	40003602	TGTTATTTTTTTGTTTTTCATTGTCACACGCTGTTTATGTGTTAGATATTTGTTAAGGTCTCCACAGAGTGTTGATAATAGCCTCATAAATTATCACTAACGGTCTCCATCTTC AACCATTATGATGTCATAAGGCTGTGAGTAATGGGAGGAAAGAGAGGCCCGACGCTCTATCACGGAAGATAACACAACACAGCTGATGGACATTTGCGAATAAAGTGCC AGTGCCA[C/T]TCTAAGACCACTTCGCCCACTGTGATTGTGTTCTCTGTTCATCACGGCAGAAGAAGATAGAGAGGGAACCAATCAATAGCCATTTCACATCTGCACCCTTG ATGCAATCTTAATCAAAGGGCAGAACCTCTCTTTCACCTGTCATTCCTTTACTGACATCAGACCATCCTCTCACATGGTCTTGTCTGTCTGTTGCCATGG
LG9	ref-46521	65.568			CTCAGATCTTCGACACCGCTGCCCGAAGCAA
LG9	ref-61183	66.957			TTTTTCCATCCGAATTGCTTGCACTGCCATA
LG9	ref-9782	67.541	Chr15	27971042	AGCGAGAGCCATAGGGAGTGATATCTGATCCAGCGGTTTCAATAAACACCCCGGATTCACAGGGAGCATCAGGAGACCCAATTACTTGCAGGAGCACTAAATGACTGCGT GTGCGGCTGGAACATGGTGAGAGGAAGTAGGATGTGTGAGATTAAGGAAAGACTGAGAGAACTGTGTCAGCAACTTCGTTGCGCTCTCATTTCTGCCCAGGGTTTGGAAA AAGAAACAGGAGCAGCGTGGCGGATAACAGCAGGTGGTGTTTAATAAGGGACTGACAGTGACTGTATACTGAAGAAATGTACCTGTTAATCTGCCTGAAGTCTGAGGGGA ACTGCTGGAAAGAAGTAGGTTTTCTGGCTGAATGAAAGA
LG9	ref-70528	68.057			CTGGAAAATTCGAAAACTGTGCAGCTGGTAC
LG9	ref-2936	68.5			TAACATGCTCCTTTCATGGGCCTTTAGTTTAAATCTATCCTGACGCAGATTTGCGGAAATATGAACTCCTATAATCTACTGGTCATAATGATTTCAGAAGAATCCAGAGTAA ATCAAATATAACATTTTATTTGTCAAGTAAAAATGTATCATGCCAATTACGTAGTTGGACAATTAGAAGCCAATTCAGAAATAATGAATGCATAACATCAATTGAAATGCA CATGCACACAGTGGTGGATTAGTGTTTTTAAGACACTTGCCCATCACTGCGTGGGGGCTTCTGGCTACAGATGATCCCACATGACTGCTTTGCACTTTACCTTATATACNNN NNNNNNNNNNNNNNNNNNNNNNNNNNNNNNNNNNNNNNNNNNNNNNNNNNNNNNNNNNNNNNNNNNNNNNNNNNNNNNNNNNNNNNNAAAAACAATAG
LG9	ref-26812_30	68.675	Chr15	27848760	TTCTCCAGCCTGCACCACTGGGACCAGTGCGGCATTGTCCGCTCTAGCCGTAAAAGGCCTATAAAAAGCTAACACGGCGAAGAAAGGGTAATGATGAAATTTAACTGCTT GCCTTGGCGCACCTGGGGTCTGGCGTGGCCCGGGGCCTTAAATCGCTTCCCCCTTCGCTGCCACCGGAGGGCCCAGTGCACTGAACCAGAACTGCCCCGCCGAGGTCAATG CCACTGCA[A/G]AGAGTTATTTCGTTTTAAAGATGTGATATAGATTTTGTGGGATCTCATTTAAGCCTGAATAAAAGAGCTGGCCAGAGAAAGTTTGAAATACGGTGGAGG AGAGACTCTCCCTGAGAAGGACAGTAACTGTGGTGGTGTTTAACCTGTAGTGGGGCTGATGACTGCTGGAATTAACATTGCATGCTGGTCCTTAGACTTGGCC
LG9	ref-33295	69.066			TCTCTGGGGTCGACAAACGTGCTGGCGCGTT
LG9	ref-38217_25	69.32			ATCTAGTGCACCTGGCTAAAATGGTGTCTAATACTTGAGGCCTAATGGTTAGAGACTTGTTACCTGAAGGTCGCGGGTTCGAGCAAAGCACATGTACACACACATACTCAC ACCAGCACATGAAATCGCAATCCCTCTGTATGAACACAAGTGTAGACAGACATCCATACAATCATGGAGCATATGGCTCGCA[C/T]ACACACGCGATCACAAGAGATCGGT CTTGTATTCCATCAGCCTGATAAGATCTTGTAAAGCGCGTGTGTGTGAGCGCGTAACGGCAGCGGATGGTTGTGATCGGGCTGCAGCCAGTAAAATGTGTTTCACGGCTGA GGCCCTCAGAGATGGAGACCGATGATAGCACTCAAGAGCT
LG9	ref-71136	69.33			GGAGTTGGAGCGATGCTCTTGCAGAAGCAGG
LG9	ref-51195	69.792			TCTGGATTTTCGAGTGTCGTGCTCCGCTGTG

LG9	ref-52900_6	70.91			TGGCC[A/G]CCCACGACCCTGTTGCCGGTTCACAG
LG9	ref-52900_31	71.035			TGGCCACCCACGACCCTGTTGCCGGTTCAC[A/T]G
LG9	ref-7211_26	71.166	Chr15	23009908	CCCGGCATCCAACTCCATCATCAGTAATATCTGCGGTGAGAAAGAGGTGAGTGCTGGCATTGGCTTTCTTTTGTCATCAGTGCAACATACCAAAGCCCAGATCATATTACCAAGTAATCATTCATATCATTACTGTAATGACCGGAGTGTTCAGCGACATGCTCTAAAAATGATCCCTAACCACAGCTGATGAACACAACGACCAAACAACGAGTGTTATGCTGTA[A/G]CTACAATGTGCAGCTCTAATTAATAAATACATAAATGATTATTGACACATCATGCTCAGCAATCCCACTGTTCTGAAGTCAAACTTTCCTAGAAGCATTTGTTCTGCCGTCAGCAAACATGAAGAGGATGTGAACAGACTAAGTAAATTACTAAAGGGAAGAATTAAATAACGTGGATTAACGGACAATTTATATTGATTAATTG
LG9	ref-38894_23	72.482			CTCCACAGGTAGAGCTCTTGTAATGCATCTTACAAACGTTTGACACTTTGGATTCAGTGTTTACAAACTCTGTGATCATGAGGGACCTTAAACACAAGTTCTCTGGAGAGCTGTTCACACATGTGTGCCAAGCCTTTCCCTCAGAGAACACCTGTTAAATTGAACAAAACCATGAGTCAGTTTATCAGATATCTGCAGACAAGTCTGAACCGAATCCTGTGC[A/G]TTTCTGTGACCATAAACCCTGAAGGGCGAAAATAATTTTCGGAATGAGAGAGATTAATGCCTCTGCATTCAGTACACAATGAAGTTACTGTGTGAACAGTATTATTTGTCTCGCTGTAGCTTAGTCCCAGAAGTTATTTCTGAAAGTTTGTGCTGAACCAAGTTTATAGTCACAAAGTTTAAATTACCAGAGAAGAGGTAAGTACAGTA
LG9	ref-15272	72.675			ACTGTGCGTGCGATCTGACTGCAGGTAATTA
LG9	ref-33761	72.94			TACCGCTGTAAACCTGCGTTGACAAAGAGGTAGTTAAAGCTAACACATACCCTATAACTTAGGCAAAAGTGCAACTGTATGGGAGGGCATAACTTACTTATGATGAAGGAACCACAACAGAGTACAAAAACTTTCATGTTGCTCCATAAAGCGGTCTGCAATTAGTTTGTCCATGCACAGCACAGGAGTCAAGTTCGACTGCAGATGCTCCACGAAGGTAGTGTGCAAATGAAGGAATTAGTCTGTCCGAGCACAAATGAAACGTTCAATTGGAATGCTCTTTGAAGGCTGTTTGTAAATGAGAAACTGATCAAGTGCTTACACATATTCACATATTCATATTCATGTATAGATTATTAAACACACATT
LG9	ref-52021	73.252	Chr15	29218781	GAAGAACCTATTTCAGCTCCTAATGCTTCTTGTTTTGGTTATTATGTTATAAACAGAGAAAACTTTTTAGTCCTGTCAGCTCTGTAACTCTCAATATTTAAGATATTTAAAGATTGCCATATACTCTAGTGTCACAATATCCTCAGCTTATTGTATTTTCTGTGCCATCAGCACTCACTGCTTTTGTAACTTGCTCCCAGTTGTAGCTGTCGATAGTGTTGCCGTGTTCAGAAACTATGGATGTCCGTTGAACTTGTCCTGCAGGAGAGTCAAGGCGTTGGAGGAGAAGTCACGCAGGACATGGAGTGTTTCACGCTGCATTTGTAAGGTGTCACGTGAAAACTGCTGTTGGGTGTGGACCAATCTCTGCACCGACTCCGCAAGCGACTCCAGCGAGCGAGACAGCGACCCTATCAGCAGTGTGGTAGCCT
LG9	ref-46047	73.612			GAGATGGATAGAGTGAAAAAAGGGGGAGAGAGTGGGGGTGTAATGCACCATATTCTTTAATAGGGCACCACTGGCGCTGCTTTATATCAGATCAGACACGACAAAAGGAATGTATATATATATATACCACTACACTACTGTTTGTGTGAAGATATAGTAGGGCTGCACAATATATCGTTTAGCATTGATATCGCGATGTGCACATCATTTATTTCAAACAATTTATAGATAAAAAAAAAAATACGCTTCCTTCTGTAGACACCACTGGTGTCGATTTTTTTTGCAGTGTTATTTCCGAATAATAGCTTTACGCACTATTTGAGCTTGTATTTTGTGATATTTGTTTTGTTTTAGTTTGCATGTTCTCTCCTC
LG9	ref-54707	74.027	Chr15	15987440	ACTTGCCAAATTTGGGCTTGTCTCCTTGAAAGGCTCCAAACTCGTCTGTCATTTCGCCCTGGTTGCCCTCTGCTTCTGTGATTCCAGGAGGGGAGTCGGATTTCTCAGAAATCATGCTGGCAAAGTCGCCAAAGTCATCATCGTTGCCTTGGCTGGTCTGTTCAATGGAGCCAAAATCAGCAAAGGCTTCGAACCGGTTTTCAACAGTGGAGCCACCATCTTCGATTGGGAAGGTGGTCACCTTAGCAACTGTGGTGTGGGTGATGTTCTCAGAGCTCCCGAAAGATGTCTCTTTCCTATGGACAACAGCTGAAGATGGCAGTCCACCTTGAGACTCTCCCTGGAGCTTCTTACCTTGACCAGATGGGA
LG9	ref-34444_30	74.347	Chr15	25598827	AGCAGGATGAGCTCCCTGCTGATGCAGCACTGAACCAGGCTGTTTCCAGCATCACCAGTGACCACATCCCCACCATCTTCCTGTCCTCACCTGCCAAATCGCCTGCCAAGACTTTGCCTGCAACCTCTACGGTCATAACACAAGAAGAAACGGCTCAGGCTGTGAGCTGCTTGCAGGGGGTGGAGGGCGTTGGATCATTTGGAACACCTACGAGGAGCGTGCAAAATAA[C/T]GGACAGGCTGCACTGGCACGTCCCGCAGGTCAGGAAACCGGTTTCATTCAGTTGTTGCAAGCCAACCCCACCTTTGGGCCTTCAGGCGGCTATTTTGTCGTGACCGATCCGGCCTCTGCAGAGCAGCGGTCAAATGTCATGCTGCTTTCAAGTAATGTGCCTCAAGGGACTCTGTCTTCAATGCAGCATGTGGTAGCGACGC
LG9	ref-5203	74.859			TTAGTTTACGCGATCTCAGTGCTGGTGGGCT
LG9	ref-900	75.673			TTCGAGCACAAAGCAGTACATTGGGTGATTTTGCCTACGTATTAACAGCAGTTGAAGTCAAGCAAACTGGGAATTAACACATGGGAAACAAAAGATTCCTGAGATTTGACGACACGCTGCACTATTCTCATGCATCTAAATCAAGACTTATTACTGCATATTTTTAGACAACAAGTTCTGCAGTTTTGTTGAAAATAAAACCAAAATAATTTATGGTTTTAAAAAAAATTTACTAAAAATTATTTTGCCTTTTCTTTTTTGACTTATCGGCCAGTGTAATTAGTGTATTTTTTAATATATTATAATAATATTTTTAAATCTTTCTAAGCCACTGAACAGGGAATTTATACAGTAATTCTGGGTTCCTAATT
LG9	ref-62198	76.668			GGTGTATTGGCGAACAATATGCAGGCCTTGT
LG9	ref-12190	77.051			CGACACCTAATCCCAACTGATTCACGCAAATAATTCAATCTCATCAAGCCTGATTTGTTTTTGATTGATTCATGCTCTGTGGATGTTTAAACCAGCTTTATTTTTGACAGAGTGATTTATTTGATGACGCATTTAATACACAAGATCAGCCCATAAATGCTGATATGTTCATTTGGTTTGGGTTGTAGTTATCGGTGGGGCCAAGGAAATAGACTGGTTTGCTCCAACTGGGGAGAAAATCGAGCCCGGCAAACAAGACGTCAGCGTGATTCGCAACGACGAGACGTCTTCCACTCTCACCATCTACAATGCAAAGGTCGACAATGCCGGGACGTATAAATGTGTGGCCAGCAATGGTGATCAGGACGCTGAAGCTACAGTCAATCTAAAGATATACCGTAAGATACTTGTTTATGATTCATCTGTGCTGA

LG9	ref-43980	77.962			GAATTAAACTCGAGGCTTGTGCGTGAACAGT
LG9	ref-60387	78.766			GCATCTATATCCACACAATTTTCATTTGCACTCACATTGCTTGCTGAGTTCAATTTGATGTTCCCTATCATCAGACAGAGTCAACATTTCAATTTGTTCATCTTTTCCATTGCTGTTCTCAACCAGTCTATGGTTTTCTGGAGCTGGAGTTACTTGCTGTACATTAGCAGGTTCAACCTCATTCTCAACTACACTATCAGCCTCAATAGTCAATGGACATGCCTGCTTTATATGTCCATATCTACCACAATTGAAACATGTAATTGAGTCAGTGCTAATAAATATTGTGTAATCCTTGCCTTGTAAAGTCATCTTCGCAGAAACATTTAAGGATTGACAATCTGCATTCAACACCATGTGTGTTTGGCGCCTGAATGACATTACATGTTTCAAATCAGGGTTTTTTAGGCCAAGAGGAATCATTTTTATCG
LG9	ref-63114	79.417			AAGTCCCACTCGAAGGGGGTGCAAGTAGAAC
LG9	ref-3514	79.769			GGATCTGGGACGACAAAGTTGCTATCTGGGT
LG9	ref-11012_7	80.06			GTCAAC[A/G]TGCCGACAGAGATGCATGGGAAACA
LG9	ref-11012_8	80.705			GTCAACG[C/T]GCCGACAGAGATGCATGGGAAACA
LG9	ref-71816	81.254	Chr15	8648702	CTTGTGCGTTATTCATGTTCAGTTGAGCTCTTAAGTTCGTTGATCATTGTTTTCATGCGAGTAAAAGCCTAAATTCAATCTGTTCACCATAAAAAACGATCGTGTCTCTTCAGAAAATTTGGACTAAACCGCTCAATTCATATGGATTAGTTTTACGATCTCTTTATGATCTTTTTGAAGCAAAGTGGTCGTTGCAAAGACAGTCAATGAAGGAACAGACATCTCTCAGATTTCATCAAAAAGATCTTCATTTGTGTTCCGAAGATGAACGAAAGTCTTACAAGTTTGGAACGACATGAGGGTGAGTAATTAATGACAGAATTTTCATTTTAGGGTGAACTAACCCTTTAAGAGACTTCTTTCAAAAAC
LG9	ref-60512	81.604			TGACGAATGACGAATGATTTGCACACAAATT
LG9	ref-47880_32	81.846	Chr15	14367113	AAATATTTTTATTTCAGACAGATGGGGAAAATAAAGTGGCTGGAGTAGCTTTGCAGCACAACAAAAGTAGGAACAAAAGAGGACAAAACAGGGAGTCGACTGCTGCACTGGGCACTTCCAGTTAGCTTTGTTGGTTATAATGAACGATCTAGTTCTGTCATGAGTTGTTGTTTTGGAAACTTTAAGTGGTCTCCCGCCCCTGGTCCCTGGCGATAATGGTGCCTGGTTTAA[C/G]ATTAAGAGGCGGAGTCAATTTCATCCAGGCCAGAATCACCTTTAATTCATTGGAAATGCTTGGAACAGGGCCAGATTGAACAGCGGCTCCAGCACCAGCACCGGGGTCAGTGGAAAAGGGGTATGAGAGTGGGTAAATTGTGTTAGAATAGTGGTACTCTATGGGTTGTGGTATGAGTGTGTAATCCATGGCTGAGGCTG
LG9	ref-6135_15	82.597			TCTACAGGTGTGCTTACAAATTTGTACGCATATTTGGCACCCATAAATCTGTTAAAGCTTCCTTTTTAGGACATCCTCGCGTGTACTAATATATATATATTATATTTAAATAAAATATCCCAATTTAGATATCTAAGTAAACATATGTTTGTATTTCTATAGACAAATAACTGTCTTATTTCTTCCAGATGAATGCGGTGACACAAGTAGCGAGA[A/G]TCTTGCATACTGTGATGCTCAAACTCGGGATGGATCCTACACTGGAAGACCATAATAATGTGCTTTGCTGAAATTCAACATCAATACAAGAAAGTGTTTGGTTTCAAACAGTATGTCTGTGTCTATTTCAGTGCCTCCAGAAGAGCTATTGAATTTTAGATTCTTTTTTTTTAATATAGAATGTTAATGACGTTCTTTAGTTGAAGCTTAATTAAT
LG9	ref-61947	82.982	Chr15	14565979	CCTCAGCTGCCCTGCATTCAGTCCCATCACCATGGAGGGGAGCCCCAGGCTTATGTGAACACACTGGCCCTGTCTGCTTGTTCTCAAAGCCTCGAGGCATAAAAAGCAGCCCATCTGACGCAGAATCACTGATATCAAGCAGGCGAGAGCCTCCAGCGAGGCCCAAAGAGGCACTGAGACCGGCAATGCTACTTCCTTGTTTGTTCTCTCCGACTCCACTGCATTCGATTACTAAATCTTGGTCTCAAACAACTCATTATCCCTAATGCGGTTTCCTCCTGCCCTCTCCTCTTTCACTCAGCCTGTTTGCCACCCTCGTTCTTATTTTTGCTACAGGCATGATTGTGGTCCCTGTTGGATCTGACAACAGACCCAGAGCGCAATCATACCCAGAGGTCCGTCACGCTAAGAATCCTCACTGTTCGTCATAC
LG9	ref-63665_17	83.897			ACTTGGACTAGTAATTAGTCACGATGGCCGACTCGTCAATTAATGTTCATAAAATATATTTTCATCACACAAAGATCTGCCTGTAGCTCTGCCTCAAACTAGCTTAGTCCAGCTGATCCATCACCCAAATAAACTTGGACTAGTAATTAGTCACGATGGCCGACTCGTCAATTAATGAAGTGTTTTCACACTTGAAACACTCACAAAGGTCGACCA[A/T]GGTGCTTACTGTGATTTAGCCAACTGGGATGTGTTCCTGGGTCAATATTGTTGTTTGTTCTGAAGCAACATTCCCCTTAACCAATCAGATTTTAGGGTTAGGCTTAAGGCGGGGCTTGGGATTTGAATGACATGCTTATCAACCTATTATTTCCTTACAATTTAAGGGTTAGTTAATTAGTTGGGGTTAGGACTATGTTTGTTGACATGAATGTT
LG9	ref-64789_31	84.297			ATCAGTTCCATATAATAAAGTTCTCATGCTGTTTTTCTGGCAGGATCTTTTTTTTTTTTTTTTTTTAGCAAAATATCTCCCCATTCACTGATACATCCAGGTCACACTCAGTCACCAGTTAATTGTTTGGTCTAAATTGCAACTGCCCTCATTTTGCCGCAGGACAGCAATTGTGAGTAAATTTTCTGTTATCTTTGCAGGACTGGATACGAAGAGTGTGCAAAGTAAC[A/G]TATCTTTAGCAACAGTGTGAAAGTGAATAGCACATAACAGGCACAATAGCGGTATAAAACATACATATCTATCGGTTCAATATGCGTCACAACTAAACGCGTCACACTAAATTGTGAAAATGCTGTTTTCAAATTTATCCACTTGGGAAAAGTGGATCGGACCGAGCAGCACAACACACTTGTACCCAATCAGCAGTAGGG
LG9	ref-25974_31	85.625			ATTGATTTCACGAAGAAGATGCTTGTTTGC[C/T]T
LG9	ref-30941	86.949			AATAGATCAATCCAAAACTAATGTTTAGGAGCAAACATGTTGAAGATTAGCACTGGGCTGTCGATTGATTAAATGATAAGCCATTTTCTCACAAAAGCAGTTTATTCAGCATAGTGAACCCTCTACTCCATTGACATCCATTCAAAGAAAAAGAAAAAAGGCCTCTTGTCTCCTTCCCTGCATGCGTACCGCAGCATTAGCAGGCATTACACTTTTTTTGCTAACCTTGCAGGCTTGCCTTCAAGTGAATTTGCAACAACATCCCAGCAAGCAGCTAGAAAACCTTATTAACCATCCATAATACCCTATAGCAACTGCCTAGCAAGCAGCATATCATCCAGACCAACCTAGAAATGCTTAAAAACAACA

LG9	ref-62471	87.554			TTTGTTAAAGAGTTATAAGCCATGAACCAAGCCATTCAGCAATGAGGTGAATCACAACACTACAAACTTGGATTTGAAGCCAAACATTTTTGAAAATCAGATATAAAGAC<br>AAAGGTAGAAGACTGCGTACTTAACGTCTTTAGTGATGGAAACAACTACAATCCAATGAAGCACTGTGAGCAGCATAATCGAATTAAAATCCATGAAGAAATATAAAACA<br>ATTTCATGGGAGTGGGTAGAGCTAAAGAGCTATTTTGGAGTGTTGCTTTTTTCGACTGATTGGTGTGATTTTCTGTACAGCATCATGGGTAATGTGGTTTTTCACCAGGAAT<br>TCCACTTTTTAAACATGAAAAAGTTCACAGTAGATCT
LG9	ref-23707	88.378	Chr15	42251606	ATAAATATAAAATGAAAATATATTAATCTGACAGGAAAATAATGCAATAGAAATAATCGTGAAATAATGCAATTAATGCATCGAATAGAAACTCATTTACAAACACACAC<br>ACGCATTCATTGTTAACTTCTCCATTAAATAGTTAGGTCGGTGTCTCCAGGGTCCAGGCTATTTGGCGTTGATTTAGCTTTTTAAGTAAGAGAGAATAATCTAGTGTTTTGC<br>ACGTGAAAGGACCCCCGTTTCCCCCGCGTGTTTCCCTCCCGCGTGCGCCCCATTGTCCCCGCGCGCAGGCAGAAAGAAAACATTTGAAGTTTCTTTTTAGTCTAACAAAAG<br>AGCCACATGTCATTGAAGTCAGTGTGTGTGTGTGTGTGTGTCAGAACTGAGAGAGTTAAATTACTAAGTCAATCATCTTAAGTTATTTTGTTTAAATG
LG9	ref-60194	89.555			GGATTCAAGTGCTGGTTTAATGTAACTGACCGACATTTTTACAGGGGGAGTAGACGCTGTAGATGACAACACAGCTGTCATTTCTATGTTTTAAGAGTTCATTAAATGCTTT<br>TTGGAGCAGGTAAGACACTGGGTTACTTTTGGGACCTTCTGATGTTTTTAAAGGACGAGGTCCTGCTGCTATGAGGTCGTTACCCTCGTAAAAGTGAATGTCATTCTTAAAA<br>ATGGCACATCTCACACGTGATGCATCATCCTCCTGAGTCAGGAAGTTTGAATGATTAAAGCTCCCATTATGTTGATTCTTCGGTACATTGATGGCTACAGCAAACGGCTCAG<br>GATCCCATGTAGCTGTAAAACATCAAATGGAGC
LG9	ref-42806	90.576			GAGTCACAGACGAAGGTTGTGCTGCTCTGAC
LG9	ref-60450	90.61	Chr15	11386844	AACAAATCTCTAATATGACAAATACATTGGCTCTCAAGTCAGCAGGGAAATCCCAACTGTTGCATTGTATAGAAGTGTAGAAAATCTCATTGTCTGACCTACTAGCAAGCA<br>TTATTGTGTAACGAGGCCCTTTTTCTGGTCCAAAAGCATCTAGAAGCTTCAGACCCACCTCTTTAAAAGCAGCATCATCGTGTTATCATGTTTTTCCCGAATACAAGTTTTA<br>AAATACCCAGGGATTTAAGAAGGGTAAAAGAAGTAGACGTCAACAACAGTGGCAAAGTGGAACCACTGCAACTACGTAGTGGTCACCAACAATACAACCACTTTGTATG<br>CAATCCACGTTGCGTCCTCCTCAGCCAGACATGGAGG
LG9	ref-48584	91.377	Chr15	42811869	AAAAAACTATGACATTCAAAAGAAAAAATAAGTTAAAGGCTCCGTGTTATTTCAATTTCCACCTGCTCTTTACTTTTCAAGCCTAAACACAAGCATGCATCATCATCATCAT<br>AGTGAGATCTGAAGGATGAAAACGCATATCAGAGGATGAAAGTTTATGAGACAAGTTGTGGATGAACGCATTTCAATCGGGTTTCGTATGTTGTCATACCTTGTATCCTAG<br>TTTGCTGTGTTGTTCAGAGCAGGCGTGGCTGTGAAGCGCATGTTGCTCATGTGTAATTCATTGAAAAACTGCTCCGGTTGTGCTGATGTTTGTCTCTCTCTCTCTGCGGTTTG<br>ACTCTGTTGTATTTTACTCTAGAGCGCTCACTC
LG9	ref-39380_30	91.739			CGACTGGTATCGACAGTGTTGCCACATCT[C/T]GC
LG9	ref-65706_4	92.321			GTG[C/G]ATTAAGCGATTAAGCTGCAGTGTGGACT
LG9	ref-10648	92.748			TGAAAAAGGGCGCGAGAGCGAGCTCAATGCTGACAGCGCTCAGCTGTATATGACATGATGCTCTCCCCTGACTCAGGTGAGACTCTTGGAATATATATTCACTCTAAGAGC<br>GTACTTCTCCAGCATTTGTTGTAAAATGCCCTATCTCTGAGTGTGTCGTTTGCTGGCCACACCCCCGCGAGCCGCATTCATTGAGGGCGTGTGCTCTCACGATGAGAGCGCT<br>ACAACGAGCTCAATACTGACAGCACTCAGCTGTATATGACATGAAGCTCTCCCCTGACTCAGGTGAGACTCTTGTAATATATTAACTCTGAAAGAGCATATTTCTCTGGTGT<br>TTGTTATTAAATGCCAAACTTCTGAGAGCTTCACTTACCGGCCCTGCCCCCGCCCCCACAAGCCGCGTTTATTGAGGGCGCATGTTCTCACTACAA
LG9	ref-60614	92.929			ACATTTTCATGGCATTTCCCCCCAAATCTTTCTTATTATTTAATAATTGTCATTATTTACATTATTCTCAATACTAAAATACAGTAAAAAATAATCTATTACAGTACAGTGAT<br>TTTTAAAATTTAAATCCACATACAAAACTGCTTTAAAATGTAAATTTTTGTATTTAAATGAGCATAGCATTTTTGTCGGGTTACAGTAATGAGCACTTAAAGGAATAATGTG<br>CTTAATTGTTATTAAAATTAAATCAAATTGTTAAACAACTCTTAATGGTCCTAGAAATATCTTTCATAACTTTAAGCAAAACATGAATTCCTTCTTTTAAATTTGGGGTGAA<br>ATATGTTCCAGACATGCAGACTCGTCATGCCA
LG9	ref-42970	93.551			ACTATACCGTCGAAAGAAATGCAGACAGAGT
LG9	ref-15815	93.783			ACTTGACAGAGGGGGCGTAGCGGTGGTTCAGTTCAACACAAGCAGTCAGAAATTAGTTAAGTAAAGTTTCACGAACCTTCGGTAAGTTACTCGTTTTGAAACGAGCATCGTT<br>GAAAGAACGAAAACTTGCTGTTCAGTAAGTGAGTCAGATGTTGATGGTGAATTCCTGAAGTTCATTCGGCAAAGGATTCGTTGTAACGTGAGTGAAAATAAAATGAATCA<br>TGAAAAGCTCAAACGAGTCATTTGTTCGTGAATCGGACTACAGCGTTGTTTGTTTTGGCATGCAGCTCGCGAGGACAGGACAGCTCAATAGAAGGACGAACGCTATGTTTT<br>TATCATCACATTAAATGCAGACTTCAGAATTACATTC
LG9	ref-7642	94.174			ATTGTTAGGAAAAGTGCATAGCCCCTCTGCTTTTTAAAAATTTTTTTTTTTTTTTTTAAATATAATTATTATTTGGGACTATTGTACTCTTTTCATCACAAGCACAACAGCAA<br>ATATACAATGGCATCTTGGATCTTGGAAAACATATATACTTTTACCCTTTAAGCAGACGACGCCTGCATCTTCTTCGTGACCACAGTAATGGTTTCCAAAAAGATTGTGTGA<br>GCACTGTGTGAGGGATGATTCACTTCCAGAACATCTCACATTATCCAACCAGATCGGATCACTTCCCTGGCCAAAATGAGCGTTGGAGAGTGCTCTAACAGCTTCTCCACA<br>TCCCAACTGTCTGCACACCACATCAGCATCTT
LG9	ref-37216	94.251			CAATGACACACGATGAGTTTGCCCTAATGTT

LG9	ref-6977	94.324			AAAGTTTATCCGAAGCTGCTGCCTGTGATTA
LG9	ref-4144	94.473			TACCCTCAAACGAAGGCAATGCTGTCTACAT
LG9	ref-68783	94.789			TATGTGTCAGCGATCAGGTTGCAGCAACAAA
LG9	ref-14685	95.078			TTTGTGTCACCGATCCTATTGCTAGGAGCAG
LG9	ref-22145_9	95.163			ACAGACATTTAGATTTTTCTAAATTCAGAGCACACTATCCATACTCACTCACCCTTCTTTTCTTTCTATTGTCTCTTTCTAAATGGCCTTATTCCATCACAGTGTAGCTCTGTG TCTAAAACAAAGTTTGAACTGCACTCTCTGCTCTCACTCAGAATACTAAACAGCAAGACAGAG[C/T]TGCATTGTGCTCGTATTTCACGCAGTGATGGGTTGCACGGAGGTT AGTGGGCTGCCCCATCATTTCCCCTCATTAACAGAGCATCTCTCTCTCTTGACGTAACAGCATTCGTTTGACTGCGAGCTTGTCATTTGCGGAGAGCCAAACCCCCCAATAT AGCAGCCTGGCAAAGACTGAGTCATAACACGGTAT
LG9	ref-41249_4	95.87			ACT[A/T]TTTGCGCGATTTGGATGCATGTGCGCGA
LG9	ref-43875	96.337			TACGAGATGCCGATGCCGATGCCGCTGGAGA
LG9	ref-62004_7	96.88	Chr15	42720367	TGTTCAAAATTTATAAAATCTATATAATGAGCATTGAGAGAGTACATTATGAATCCATCTTCCAAACTGTGGTTTTGTCTTATCCTGAATCACTACTATAATAAGTGTTTAT ATTCTGAATATTTTAGTATCCCAGTACGATGTTACCACATAATGCATTTTTTTTTTTATATATATTTCTGACCAATCATTGTTCTATGTGAGC[A/G]TACCGATAGGAGTGCC CACTCCATAGCCCTTCGAGTCGATGAGGCCGCCGATCTGTGTGAGGTTGCAGTTTCTCTGGCTGATGTACTCGATGCTGGTGGACTCCATCAGAAGAGCGTAGTCTGTGGTC AGCACACGCTGGATGCCCTCTCTGTTATTCTTCACCAGCGCCGTGTTCTTCCGGCTGCTCATGAACGCCCACATCTTCTCATAGGTGGAGATCTTTG
LG9	ref-62215	97.324			TAACACTTTACGAAGCTAATGCCAGTTGTCA
LG9	ref-21382	98.555			TTTATGTTAATTAAATTTACGTACAAATACCCACTGTATATAGCAGTATGTGCATCAGTGTGTATACAACAGTCATAGCGAGATTTGTGTCAGCTTATTTACAGCATCAAAG GGATGTAGAAACTTCTACTATAAATGCTGCACATACTTTTCTACAGGCTCTCTGTGAGAAAGTTGGTCCTTTCCTGGTATATGTAACCTTGGACAGCGTGAAGAAATTGCTG TGCTCTATTTGGTTGTTGGCAAGCCAAGAGAGCTTCAGGGGTGTTGCAGACCAGTTTGACATGTCAAAAGGAACCCTTCAATATATTTTTTTCTGACATTTGCAAGGTCCTT TGTACACTGAGAAATGAATACATTTGATGGCCCAGAGAGGATGAGTTTGCAAACCTGGCAAATGGCTTTCAGGACAAAACTGGCTTTCCTGATGT
LG10	ref-42771_28	0			CACAGATGAACGATAATGCTGCGTGTG[C/T]TTTA
LG10	ref-19419	1.418	Chr20	41592960	AAATGGAATTTTCCTTCACTAAAACTAAAACCTTTCCAGTGCAGTAAAAGTCGTTTTTAATGGACCAAAGGTGGGGGAAAAAATGAATATTGAAGACTTTGGGAAGTGGT AGATTCTCTAGGGGACATATTCTTTTTATTTTGTGGGATGCTGTGGAGTGATGTCAAGATGTCATTCCCCTGTACAGTGTTGAATTAAATGTTGCACCTGCGAACCTTCTACT GTTGCCATGAGCAACGACATTTAGCCAATCACGAATGCAAGTGTCGAAGAAGCAAAAACATCCACGAGTGATTAAAAATAATCAGGGTGAACACAAAAGGGCGAGAGCA CCTAAGAAAGATCCTTTCTTAGGTAGGTGATCTGTCTATCGTCAAAGTAATGACATCATTTTATAGTAATCATGGCATTTGCAGCTCAGGTCTAATTGT
LG10	ref-45329_31	3.785	Chr20	41225212	TGATGGGATGGTGAGAGATCTAGTGGAGCTACAGTTAAGACGGAAGTGCTGAAACCTAGATATAAATAAGTGACAGCCTGAGCTTTTTGCACTTTTACATCTAATGTTCCC TGTCAAAGAAGGCCAGCGGAGAGATGGATCGAGATCACAGACCTGAATACCTGCACTTATCGCCACTTATCACCTGCGCTTACGTTTCTGAGGTTAATGCGAGAGGCATGC TGGGGCCA[A/T]GGGCTGCCATCTGAACACTTCACAAGTTTGTGAGTTTCAAAAGACAGACTTCAAAAAATTTTTGCACTGAGGTTCTGTGTTTATTCCTAGTCTAAGGTAT GCACAGAATAACTTTTGAAGTTCACAGTGACTGGTCCCGACTGGAAGTAAAGTAAACTCACTTCTTTTGTAAGCCAACATGAGGCCCCGTGTTATAGGGCC
LG10	ref-71884	5.424			GAGTGAGTAACGATGCAGCTGCGGTTGAGTG
LG10	ref-60255_9	6.203	Chr16	23118408	AATGGCATTGAACAGTGTCAGAGTTTTTGAAGCTTCAAAAAGTGCATCCATCCATCATAAAAGTAATCCATACGACTCCAGTGGGTTAATAAATGTCTTTTGAAGTGAAGT GTGTGAGTTTTTGTAAGAAAAATATTCATATCTAAAATTTTATAAACTATAACTGCATGGCAAAATATGATAGTTGCGGTTTAAACTCGATTTTGAC[C/T]GCGATCTGGAT GCAACAGTGAGTGATGCGATATTATGCTAATAAAATGATCGTGCCAGAAGAATAGCGTGAGAACTTAATTATATCTGCTCTCTCTGCATCATCTTGTGAGCTTTTAGTATGT TTTGCATACACAGTATAAGGCAATTTAAAGTGCCACATTATGGTATTTGAAAGATTCCTAATTTTGTTTTGGAGGTCTCCTACAACAGGTTTACATGCAT
LG10	ref-2113_8	7.85	Chr20	39797437	TTACTGTAGATCCAACACCTCATTACTTTATTTTACTTCAGCATGTTTGGCCTTCAGGTACTGTATCCCAGGAGGTATGTGTTTTTGGTTGCCAGTGTGTGACGCTGTCTCTC TGCATGTGTCTGGGGGTTTTCTACCTCATGTTTGCAGTGAACATCCAAAGAACAATGTGTAGC[A/G]TTGCATGCTCATCGGCTCCTATAGCAGCAGAGCGCACGATTACAG TTCATACTGTTTGGAACTAATATGACAAGTAATGCTTTTTACTTCTCAGGAGTTCATCAATATCTTATACTTCAAAAATTGGCTTTTTTGTTTAAATAATGATACATCTTATT GTAATGTTGTGCAGTTTCTCTGTGTGTGTCCTGAG
LG10	ref-1969_8	9.123			CGAGAACCATTAGTGAAACAGCAGAAACATTCTTTTAGTTGACATTTTTTAATATTCCTTGTCATATTATTACATTGTGTATGCCTACTTATCAGTCTTGTTACTTTATTCTAT AAAATGAGCAGTAAAAAAGAGAATATTTAGAATACAACAACCAAATCCTTTATTTGAGCAAT[A/G]ATGCATTGACATCGAACTTGCCTTCAGTAAGCAAGTCATATGTAA GTTCAGTAGCCAGATTCTGATTCAAACCAAAAATGTATGAGTTTGTGAGTCTTAATTCATTTAAAGAACCGGCTTGGTGCAGGAAACAATGCTGTGATCTGTGATATACTT

					GTGGAGTGTCATTCAAGTTATTTAACTTATACAAGTT
LG10	ref-64729_25	10.277			CGAAGAGGCCATCTTGCGAGATGGGGGCGTTGAGAAAGCAGACTTTGTTGGCAGCATCTCTGCAAGGTTCAGCCAGAGATGTTGTTCCTGGACCACCAAGGTGGACATCA<br>TATGGCCGAGGGCCTGCTCTGTGACTTTTGTTGCCCGGAGGGCGAGGTCAGTAGCCGTACACAGTTTCTGCATCAACCTCGGC[A/G]CAGAACTACCCTTGTGCAGTTTTGA<br>GTGCTTTAGCCTGATGGACTTGCAGGATGGCCATCGCATGCAGGGCGGAGGTGGCCTGCCCAGTGGCATGTTTCCGCACATTTCCGGGAGGTGGGGGCACTTTGCGGGCAC<br>AAGTGCACCACAATTGCACACTGCACCTGGTTAATCTCCA
LG10	ref-50699	12.197			AGTACCGAGTATATTCGGTACCCACTGATAGGGCTGTGCCAGTATTTACTTGAACATGACACAAGTGAAGCACAAAGCTTTGTTCACAAAAGAAATAACAGGTTAGCAATT<br>AAATGTGACATCGCAGCCATGGAAACATTTTGGTTCATGCCGAATGAGAGAGGTACGTGAGTCCATAAATTTAAGCTTTGTATTCTGTTTTGCGAATCACGATCTGGTTGCC<br>CGATTAGATATTCCACTGAACATGGAAACTCTTTCTTTTCCCAAATCTTCACTCACGCAGGGAATTATAAATGTTTCTTCCTTTCGTTCACTTTTAGGCCTAATATAGGCTAT<br>TCGCATTCTTTACTGTGGTAAAGTAGAAAAAACACTGTAGGACAGAAACCTTTTTGCTTGCACATCAATTTCTATTTAACACAGTTTGTGCTTGT
LG10	ref-42771_18	12.583			CACAGATGAACGATAAT[A/G]CTGCGTGTGTTTTA
LG10	ref-42771_17	12.885			AGTTTTTTTTTTTTTTAAATTCAAATTAGAAAATTATGTTAAATATTGAGATATTAAAAGTGTTTTTTCCTTTCTGGATATAGATGAATGTCTGTTCAATTCAACTGTATGTAC<br>ATTTTGAGTGGTGTGTATGTGTGAGTATTTGGAAGCAAGCAGATACGTCTGTTTCTAAAGCACACGCAGCAT[C/T]ATCGTTCATCTGTGTTTACAAACTAAGCTCAGAATC<br>GATTCTGAATTCTCAGAATCGATTCTGAATTCTCAGAATCGGTCCAGAATCTTTGTAGTAAATGAATGAAACTATTAATAGCAATAACACATGCATAGATGCGATTAATTA<br>TTTGTATTATCATGTTTGTTAATTTTTTTTTTTTTAT
LG10	ref-3971_27	13.614	Chr20	33075348	CGCATTGTTTGTGTGTGGATTTTCAAAGCCCAATAAAAACCGACTGCAAAACTATTTAAAAAAACAAAGAGCTTCTTGCCTTTGCTACTAAACTGTAAGTCTGGCAAATCA<br>CACTGACCGATCGACTGGAAGCCAGTCACTGCGATCGATCCGTGCAATCTTCTGCTTGTGCCTCTAGAGCACAGGGGTCGCAGG[C/G]TCTTGACCCTCACATCGAGGAAAT<br>AAAAACTGTTACTTGTGGAAGCCACAGACATTGCTTCTAACTGAGGTTTGCAGGCTCATTCCTCCGGAAAAAGCATTGTGTGTGCACTAAATCTTTTTTCAATTTGAGCACT<br>GCTAATGTCTGCTGTGTGTGTGTGTGTGTGTGTGTGTG
LG10	ref-51902_4	14.187			ATGTAGGGAACCACTCAATATACATAAATCACATAACAGTTCAAGTTTTACTTACATTTATGATTAAGTTTAAATCCCAATTGATGCATGTCCGGTTTTTACTGTCAGTTAA<br>CCTTAGTAATATGTTCCTCCTTATTTCTGCTTTGCTATTGAGATATCTGACCAAACTACAAACTTTCCAGCAACGTCTGTGAAAAAGGGATA[A/G]GTGATCGACAAGCTTCC<br>ATGTCTGTGCTGCTGCGCGCTCAATCCACTCCGAGTTTCGCTTATCAGCTTGTGCACAGCAGCTATATAGGCATAAATATTTAATTTGTTTAACATTCTATTAATTTGATCAT<br>CCCTAGTTCCAGGGCTACAACTTAAGTAGTTGGAGTGACTTAAATTTTTTGAGTAATCAACTTAAAAATTTGTTTATGCCATAAAGTTCCTCTAACT
LG10	ref-45632	14.859			TATGTCAATAAAATTAAACGAATTCTGAACCTGGCTTTGTACAGTGTTCAGAAATCTTTTCTGGAGCTTTATGCAAATGAGTGCATATTTAATTAGATAATGCATCATTTGC<br>ATTTTTAAAAACGCATTTCAGAAAATTTAATACAAAACAAAACACGCTGTCTGAGAGGTGGCTTTCTGCACACGCTTCGGATGTGTGCACAGAAAATTCTCTGTGCTTGAA<br>TGTCTTAAAACTTCTTGAATGTTGAAATTGGCAAGACTTAAAAATGTATGATGCAAATGATAAACTTGGCCATTGGGCTGTCCTTATTGCCAAGCCTTGTAATAGTACCATC<br>CCAATGTTTTGTCTTGTAGCTGTTCTGTGTGTAT
LG10	ref-19998	15.664	Chr20	31792223	ATATATTTGAAATAGCAATCTTTAGTCACATTATAAATGTCTTTACTGTCACTTTTGATCAATTTAATGTATTAATTTAATTAATTTAATGCATTAAGGCATTTTTTTTTATCG<br>TAATGACCCCAAACTTGTGAATGGTTGTGTACTATATGTCAGATCACCAAGGACAAATACATGCTGCATCAGCCTCATTGAGTTGGTAGAGGACACATCATATCAAAAGGG<br>ACAAAAGGCTGAAAATAAACATAAAAAACACAGACACTTGATATGTGCGTGTGAAGCTGGAAACTAGGTCTATGGTTTTCAAAGCTCCGCCATGACAAGAACACTGGACC<br>AGAATAATAACAGAAATGATGACCCCCAACTCTG
LG10	ref-47475_24	16.318			GTCATTTATTAGCAAATTCTCTCATGATAATCTATAAATTGCTCATGTGGGAACTGTTGTAAGATCCTACAGAGCCAGATAATATATGCTGCATGAAGATAGAACAGGTAT<br>AGTTTAGTTTAATTACGGTTATAATCTTGCTTTTATGACATGCAATTGCATTTGTGTTATGCACTATTGCAATAACATCGC[A/G]TCATCCCTTTGTTGCCTGTTCTCGGGGGC<br>AGGATTTATGTAAATTTTAGGGTTAGTGATGTCACTAACCCGGGAAGAAGCTTGTTGTAGTCCCTACCAGCCATTTGTTGTAGTCCTTAAAAAGCAATTTCTTTAAAAGAAA<br>ATATCTCCCTTTGCTTTAAACTTTGAGCATCGTAAC
LG10	ref-58535	18.288	Chr23	43144781	CCGTCAAGCTCCGACGCCGATTCAACATGTCGAATCAGCCGAAAAAAAGCTGCCGAGGACCAACTTCAGCCGACGGTGTGGAACACACTGAGAAAACTTAGTCGGTCGAC<br>GAACAAAAACTGCCCAACGGCCGTCGGCTTGGTGTGTTCCTGCCTTCACCTGTCAGTGACATTGTATCTGCATTACCCTCAATTTCCAGTCCTACTTGTGTAAAAACTATGG<br>AAACACCAAAGACACTTTAATATATTATGTGGCTATAGTGCCAGGGCTGAATTTTTGTCCCAGTCCGCCCCTGCTCTCACGTGACTCGTTGTTTAGAGAATTGTTTAGAGCT<br>GCAAAACACGTCCAGCGGAGGCTGATCTCAAAAGA
LG10	ref-59490	19.319			ACCGAGGCTTCGAAGCTTGTGCCGAGCATAA

LG10	ref-1728	19.452			GCAGTTTTTCCTGTATTGATTGTTTTTATTTTTGAGTAAAACTGTGTCCACACAAAAATACAGTGTGTCCATAATAGTCTGATTCATGAAATAATGACTCTTATGAGCAGATTCTTTTAATGAATCTCTGAAATTCACTCTTATGAGCAGATTCTTTTAATGAATCATTTAAGACAAGAGCTCAGGAGGTACCAATTTGAATCAGTATGACAAATCCTTTGCTGAATGAACTAATCAACATCTAAATATCCATCTATCGACCTATCTTTCAGTCTATGTATCGTTCTGTCTGTCTATGTGTCTTTCTGTCTGTCTACCAGTCTGTCTACCTGTGTGTCTGTCTATCTATATGTCAGTCAATCCATCCTTGTAATATAGAGTATTACACATTAGACAGATAGGCAGATAGAGAGATAGATGTGTGTCAGAGT
LG10	ref-19645_7	19.833			TGCTGT[C/G]AAACGACGTGCTTGCAAAGTTGGTT
LG10	ref-58012_26	20.637	Chr20	45861039	ACATTACAGTAGCTGCAGAATCACACAAACACGATGCGATACATGAATGTAAAAGTCATAAAAACAAAACTCTTACCCTCTGTTCTCTCAGTTTTTCTCTCTCTGCTCTCCTCATTTCTTCCTGCGGGTCGACGCTGACCTCCATACTCTGAGGTTCTGAGTTCTTCTCCTCAGACACACCTGAGGTTTCCTGAGAGGAGTCAGATCCACCGATCCTGCTGCCATC[C/T]GTCTGAGAATCTGTGTCCTTCTCTGACAAATCCAGGGTTCCTGCCAGCAGAGCGTTAACTTTGCATAAAGGGAACTCGTGCAGCGTCTCGTGGAAGTGTGACGGAAAGCCGAAGCTCTCCATGCCAGCTCTCGTTGGCCCTCCACCGGGATACATCTGAATCACTGAGCCAAAACCTGAGAAAGGAAGCATTTAGATCATCAGCA
LG10	ref-19645_15	21.202			TGTCATGTCGAGGAAACGCTGTGTGTTCAAATGCGAAAGCTTTTGCTTTCAGAGGTTAAGATTTATTAATAAAGCTCTTACTGAGCAGTACGACCCAAAGTTTGCTTGTGCACAGCGCATTTTACTGAGGACAGCTTCATAAACCTGGGACAGCACAACGCAGGCGATATACAAAGACAACTTACCACTGTCAAACGTTCTGCTGTGAAACGAC[A/G]TGCTTGCAAAGTTGGTTCCGATCAATTGGCTTGATTGTCCACATTTTCAGAATCCGACTTGGGCTCGGACTGATACAGTAAAACTGATGACATTATTAACTGTGTGTTTAAAATTGCTTTAGAAGCCTTGGAGGGGCTTTGTGAAAATCAGCACGTTTCAGACAGACTGGGAATAGAGGAGCTGCAATAATGTAAAGCATGTGAAAAATGTGTTTTT
LG10	ref-69647	21.866			GCCAATGCCAAGATGATCCAACAACATGTTCTGTTTATCTTCTGCATGTGTCTCGACCATAAACCGCACAGCAAGACATATTATTTGATAATTGTATGAA
LG10	ref-51848_8	21.998			TGTCAAGTACAGTTGAGCTTCTGTTAATGTTCGCTAATCAGTGTTTATATGTGAATAAAAGCCTAAATTCAATCTGTTCATCATATAAAGTTATCCAGTTTCTTTAGAAAATGATGACAGAATTTTCATTTTTGGGTGAACTAACCCTTTAATATGAATGTACACCAGGAACAGGT[A/G]TTGCAACGGCTTCGAATGCGGCTTGTCCAATCAGAATTTTACATCAAAAAACAACTAAAGTGAGATACAGGGGGGGAAAACATGGGACTTTCACATGTGTGAGAAAACATTTGCGCGTACAAGAGGAAACGTATGTGCGTATGAAAAACTTGCTCGTGCTCACATGGTGGTGTTTCCATTTTATGTAGGTCTA
LG10	ref-38253	22.748	Chr18	29939068	TTGTTAATGTTAACAACATCAGCATTGTGTGACTATGAGTATTTAGTGTGTATTAGCGTTACCTGTAGATTTCAATTTCTATACAGTCTAATATTTAATTGTCATACAATCCGCAATCAAAATTATAAGTTTAATTATTCCAGCTGCTGTGAGAAAAGGCTATAAATAATCCGCCACCTGCAGCATCCTCGACATTATATAGCCTGGGACTCATTCTTTATGTAAACAGATGTGATGTAATGACGCAAAGATGAACGCCTGCATGCTTGAATTTCCCGCGGAAACCTACCAGTACCACACGAGGTACAAAACAATACAAGCATAATGTAGTAAATCAGGCTAAATTAAGGAGATAGTTTTGAACACTGG
LG10	ref-45001	22.873			AACGTTCATCGATGGAAATTTTAAGTGCTTTTTCTTGTCTTTCAAGTAATTATGTTTGTAAATTGGCCTCTTTAAATGATTTTCTGCCTAACAGTCATTTTGCACTCATTGGTAGATTTTCTGTGAAAATCTTTGCCTTGAGTCGTACACTTGTTGTGACAGCTCTTTTCATTCACACCGCACACCTCTCATTACAAAAAATACACTTAATCTCAGTGCGTCACACTATTGCCTGACTGACCATCATTCTCTGAAGTCTGTGACACTCTCATCTTTCTCATCAAAGACTCTGTCAGACCTACAAGGTCACCATTGATTCCACATCCTTCATTTGGATGGGTGTTTATAATGCTTCATTGCACCCTTTGG
LG10	ref-25046	23.336			AACCTCTTTAAAAAATAATAATGTACTAAAATGTATGGAAGGGATTTTGTAAGCTTTAAGGTTTAGTTACAATGGCTGCCTTGGATCAATTTAAGCTGGATTACTTAATATTATGTGTGAGCTATGTTTACATTTTTTAGAGTATTGCAGTGTTAGCTTAGCAACATGCTAATACCAGACTTGATATCAATTGTTAAATCTTCCATGCTTCGAGTGAAGTGCACTTTTTGTCACTTATAAGGTTCCATTTCTGTTAGGATGCTGCTTTTGAAGGTTGCTGCCTAAGTAGACAGTAGACAGCAATGTAGCTTGCTAAGTTGTCGAACTGAGGCCCAGCTTAATCATGGTCTGTTAATAGTTACATTTAATAATAATGCTTTTTCAGCATCGCAGGATCAGATCATGTGAATTCTGAATCCTAGAATCCAGCA
LG10	ref-57266_18	24.148	Chr22	17083014	TGCTTTGGTTGAACGAAGATCTTCTTGGTGTAGAACGACATGAGGGTGAGTAATTAATGACAGAATTTTCATTTTTGGGTGAACTAACCCTTTAATGTTTACTATTTGCTTTTGTTTTTGCTGTTGTTTTTGTCATGACTTTTTAGTGGCTTGTTTTGTGATGTTCAGAGCTCATCTGTTTATCTATTTTGTTGTTTGTCACCATCGTTGCGAATGAG[G/T]TGCTGAATCTTGATGTCTGTGTGTGTCGAAGTGGCACAAGTAGCTCAAAGGTTTTTTTGTATGAATGTGTTTAAAAGCATTTCATAACTGATTGCTCAAACACTGCATCTTTTGCTATAGAATAACAGTGGTGGTAGCAACATAAAATTAAAATGCATTAATTTAATACTTTTTATTTAAAAATATTGAATCAAAGCAGTGAATCATGCTTTA
LG10	ref-63259	25.103			AGCTTAAGGACGACACAGCTGCTGGGTTTGA
LG10	ref-59946_24	25.22			CCAGAAAGCCGCATAACCTCAAACTAGTCATGCTTCAGACACTCCAAGTCATAATAGCAACTGTTTGCCCACACTGCCCTTTCAGTGTCTGTCTGTCCTATTATGACTGGGTTGTCACACAGATGTAGAGCAAATGATGATTTAAATGCAAAGTGCAGCTCCTCATTCTACTTGAGCATGCAGATGCTTCGA[C/T]ATGCATCTTACTGCTGTCCAGCTTCCCGGTTCTTCATAACTGGTGATCGTAAACTTTGTTTGCGTGGGAGCTGGAGAAACCCTAGCAATGGTTGCAATAATATACGTAATGTTCATTCTCATTACGGTATATCAGGAAACCATAATTCTCAGTATACAATGTAAAGGTATAGACCAC

LG10	ref-38403	25.524			TAGGAACACCTTAGATGTACCCTAAAAACTGCATAGCAACACCCTGACAACCACACACAACATTTTAGCATCGTATCAGTAGGCTCTATTGATTTGAAAAAGTAACCAACCACCTAGCAATACTCAGAACACATTTTAAGTGCCCTAGCAATCACCCAGAACACCCTAACAACTGTATAGCAACACCATCGGAACCACATGACACATCTTTGCTTAGTGGCAGTGGGCTTATTTAATTTGAGCAAATAAGCAACCATCAATACCCAGAACACCTTAGATTTCCCTTAGAAAACCACATTATAATTTCTCTAGAAAGCACCCAGAACACCCTAGCAACGACGTAGCAACACCCTGGCAACCACAAACATT
LG10	ref-33098_26	25.786			AAAGCTCTACAGACAGCACCGGCGTGACAGGGTCATGCTGGCCATGAAGAAATTGCTGCACATGGAGAAACCTTCCTCCCTCCCTTCCTCTCCTCATGAGGTACCACCTATTAGTGCTTTTGTAACCTCTCTGGTCCAACTTGCCCCGCTCTGAGCGGGATTCGAACCGGCGTTTCTGGGCAAGGGAGTCGTGC[G/T]CACTAACAAGGACGCTAAAGACTGTGGTCTCTAGTGTCTGTCGCTAGTGAGCCTTTTGAGGCCAGGGGAGTGAGGTTTATTCACACAGCCCTTACTAGCTGGCCTTCATTACACTTTTTCCACTGGTCGTGCAGTTGTGTGAGAAATAGTAAAGTACATATTTGATGCAGATGTC
LG10	ref-13300_31	26.061			TATATAACAGTATGTTACAGCGAGAATGAGCTTCCTTTACACACAGCTCTAAGAGAGAAAGAGAGAGAAATGCAGTAGCCAAGATTTACTCAAGCTGTTTGTGAAATTACAATCTAAAAGAAATATTTAAAATGATTATTATTATTATTATTATTATTATTATATCCCACTAAAATTATGCACAACACTCGCAACCCCA[A/T]GCCAAAATTCAGGCCCTGAAAAAAATCAATAATTGGTGTCTGTGTATTAATTCAATATCGCTACAGAAAATATCACTATATTATGTTGTATAGATTATTTTCCTCACCCCTACCATCCACCAACAGTGACTTTCTTTTCAAACCTTTTAAGGCACAATGGACAGAGTGAA
LG10	ref-58715	26.509			CAATTACGTACGATGAAGTTGCAGAGACAAC
LG10	ref-21593	26.967	Chr20	24621350	TTTGAAATAAAACACAAAGTTAGACAATGGTAATTTAATACATATAGTTTGTATATATGTGTGTGTGTGTGTGTGTGTGTTTGTGTGTGAGCGTCCCCTTTCTAAAATGTCCTGGTTTCTCTAAAATCTTTATTGATTGTACATTAACTCCTTCCCCTGTGTCCTCATCCTGGAAGAGTGCAGCACAGTCAGGATGGGAGTTTGCTTGTTGTGGTGCACGGCTGTAATACCTCTGCTCCCTACAGATTGTTCTGGAACTATAAGATGACTATTAAACATGTTTTAAAGGACTGGAAACTTCATTAAATCATTTGGCCCATGCAAAATTATGTCTCATCCAGGCAAAACCAGCCGGAATTGTGGTTGAAA
LG10	ref-63780_17	27.22	Chr20	4318121	CTTTGCTTTAATTGGTAATTATTTTTCAGGGTTAATGCCTTCATTCAGACTGTCAGTGTGTCGTCAGGAGCAAAGGATTAGGAGCCCCAGGGAGACGAGCAGGGAGATGAGACACTGGCAACGGGAGGACAGCTCATTTTTACACAGGCAGAGAGGGAGGACAGCTCATCTTTGATCCGGCAGGG[C/T]CTTCGCAGGCCAGAGGGGTGACAGCTGCATTTTCTTTAGCCCACATTTGAATTTGAATTCTATCTTATGATGCAGCTATGTGCCGGCTTGGCAGGGCAGTGGGGTAATCTGCTCCACACAAACGCCCGGGGTTTCTTCCTGTGTGCCCTCACAGAGATTTAAGTAATTAAACTTAAATTGAATG
LG10	ref-6234_7	27.356			TAATTAAAATAATATACACTTGCTGTTCTAAAATAAAAGTGTATGATGTACTGTTAACGAAATAAAGCTCCTTCTCCAGTCCACCCAGTCCCTTTATTGAAACTAAACTGCAAAACTTCTTCAGACCTCTACATAGTGTTGATGTCACTGAACACATTAACATATAACCATCAGCATTTACTTTACATATTAACACCTCTTAGTAA[A/G]CAGCGACTTGGCTGCCCTGATGAATGACAATAACAGTGTACAGAGATTAGCCAATCCTAACAGATTCCATTTACATAGAGAACTAAAAAAAACTGTCTTTAAACTTAATTTTGAATACATGGACATTTTTACTTTATTTTTGTTTTACTATTATTTTACTTCTTAAACTTGTTCTTCTAAAAGAGGGTGGATTATGGCCTCGACTAAATTATGTTTTTGATGCTG
LG10	ref-34849	27.557			TCGTTCAGACCGATTTTTGTGCAAATCCTAT
LG10	ref-16926	27.908	Chr20	5269094	TCTTAAGGCTCAAGACCTGTGTATAGAAACAGCTAATACTGTATGTAAGAGAACGCTCACCATAATAGCTTTGAGTCCATGGGCTTTCCCAATTGCCATCTGTCTTGTCCAGACCACTAGGGAGGGGCTCCCCACTGTCATATTCCAGTGTGTAGTCATCCTCTTCCTCCACTGCTTGGCCAGTACCATCGTCACTGGAATCTGCATCCGTGTAGCCCCAAAATAAAGGCCAGAAAAGCTTTTTTCTACCTAGCCATTCTACAGTAGAGGAGGGAGAAGATGTGGGCCAGTCATTGCTCCATTCTTTAACCAGGTCCATCTCAACTTCCATTGGGGGGTCATCCAACACCTCGATGGTCACCTACAAC
LG10	ref-27271_6	28.003			CTCTTTGAATGGGGAAACATAAAATTCTCAAAATCTGTTTGCCAAGTTTTCGATTAAATTTCATATTTGAAATCACCAATGAAATCTGACAACAACTGTTTCATAAATTTTGTTTCTAAACGCTCAAATCATGACAAAAAAAAAACATATTTTTCAGGTTGGATCAAGCTAATG[A/G]AAATGCACCTCTATCGGAAGCGTGCGTCTGACTGTTTCAATAGGAACCAGACCTTCTAACGGCCGCTGCAGTGATGCGATGACTTTACCAATTGGTGATTGGCTCTTATTTAGAAGGCGGGACTTATTCCGCCATTTTGCGCATTTCTTTGGTGTAACTAATCAAGAACCATGACAACTAATAATGAACAAGAAC
LG10	ref-1556_9	28.027			AAATAAGACTTAATTCTTAATTGATTTCAATAGGTTTTGACGTTAGCATGTTGCTAAGCTAACGTGATACCAAAGACTATAGAATAACACAAGACACTTCACTCGTATTGTTTTGAATGGGAGAAAGTGCAACGCGCAATATGGCGGAATAAGTCCCGCCTTCTAAATAAGAGCCAA[C/T]TGCAAAGTCATCGCGTCACTGCAGCGGCCGTTAGAAGCTCCAGTTCCTAGAGAAACAGTCAGATGCACGCCTCCGAAATGAGACACAAGAGATGTGCATTTAGGACTGCGCATTTCAGCCTGAAAAATACAGTTTTTTGTCATGATTCCAGCATTAGAAACAAAATTTATGAGACAGTTGTTGTCAGATTTC
LG10	ref-12254	28.093			TTCCCTTAAGCGAAGCAAGTGCCAGGAAGAA

LG10	ref-72089	28.285			TTCTGGAAACCAAAGAGTAAACCAAAGACGCACGTTTGATGCCTATGATGGGCAAAGTCGCAACTATAACAAGAAAAAAACGATATGCGTGAGGATTCTGTCCTTTTTAGGGTCTCATCTTCCTGTTTTTGGTTTGTTTACATGTCTTTGGTCCATGTTGCGTTCATATATCACCACAGGCACCAAGGTCAGTTTGGAAGTGGACCGAGACCCATCTTTTCAGTGGTCTCGGTCTGCTTGTTTGGTGCGCACCAGGGTTCGGATGGCAGCATTCACACATGTTCAAATGAACCGCACTAACAGAGCAATTGCACCAGGGTTCGTTTTAATCGAACCAAACATGAAAAGTGTGAACACACCCTTAGAGTT
LG10	ref-30721	28.643			AATGTCGTGTCGACTCGTTTGCACAGAAAGT
LG10	ref-21544_25	29.018	Chr20	9579688	GATGTGAGGGATGAGTACAGATCACCATGGATTTTAGACTCGTGTCAGGATCTCATGACTCTGCTCTGATTTTTAAATTCACCCTGAATGTTTTATTCTCCAGAGCCTTCTGTTTTGCAGGGAAAGAAGGAGTTTTTTTGGAAAGAAGGAGAGTTTTCCTCCCCTCGGTACAGGCACAGGCAGCGATATCGCC[A/G]CAGGTCAACCATTAAACGATCTGTCAAAACATGTGAACTCTCAGCCTATCAGCCTGCTTATGGGGGGAAGGGAGGAGAGATATACAGAAAGAGGATGAAAGAGAAAGACAGACAGAAAGAACACCTACTCAGCATTACGGGTTGGGTTGTCAAAACTGACTTTATCTGCTGGCGGA
LG10	ref-36679_25	29.299			TATTGTTTCAGTCTGTTGTAAGCTGACTAAGGGCTTGTACCTGGAATATGTTGGAGAGATCGCGGAGATTGAAGATGTAGTGGAACTTGATGGCGGTGGGAAGGAAGGTGGTGGTGATGCGATGATGGAAGTCGAGTGCCAGCTGGACCAGCTGTGAGCTGCTCTTCTGCAGAACTGCTGCAAATCCCTCGCT[C/T]CGCAGGTGCTGGTTTAAGATGCTGCAGTAGATGGTTTTCAGTGCGTCCCCTCCAGGGAAGGAGAGCGCAAACACCGAGAAATGCCTCTGAGATACATGTTTCAACAGTTTTACATTAAGCCTTTGCTTAAGCTTTGAGAGGTGACACTAGCTAAATGAAACATTTTTCAAATACAA
LG10	ref-25626_24	29.623	Chr20	38248429	ACGTGCTTACAAGCAGAGTGATCAGCAAGAAGGGGTGAAGAACAAAGGCAGAGAAATTACTGCCAAATCCTCAATTCTTCATTTCATGACAGCAATTCATCCCCGCTCATCTTAGTCTTTTTGGCAGGATCTCACATTTTCTTGCAGCAGTAATTTCCTTGACCTTTGTCTCTGGCAGAGCAATTTCATCGT[C/T]CTTCTTCCAATATCTGAGAGAAATATCAGCGGGGTTATGCAGTTGACAAAGGAGCCAGCACATAACAAGCACTGCCAAACTCCTTGACATGAACTCAGCAATAACAGAGAAAATCGATAATGGCCCCACTGTTCCTGATCTTTTAAACGATGTGGACTGATGGGAGATGTGGGTGCT
LG10	ref-21623	29.659			ACGCAGCACAACTCTTAGTTTTTGTTCGCTTAATACGGAATAAATTAGTGATTGTATGCAATACAGTCACAAATGCATATCACGTGTTGTTCTTCAACGCAAGTCAACATAACATGAAAAAACACATGAAATGCAATGCAGCGCAAACCGCACATAAAGTTAAATTGTGTGTATGCATTGCAGCAGCATCATGTTTTATTTCTTCAAAGCGCAATGGAATGTTAATTCTTCTGTGCACTGCAATGTAAAAGCACATAGAGAAGCATAGTTCATGTTTTATTCTTTACTGCACAACGGATTAAGTTTGTGTATGCAATTGGAATGAGTTAGATCTTGAAATCGGTACAATGCAACAAACTCATATGTTAT
LG10	ref-48828	30.006			AGTCATGTCTCGAGACACTTGCACTCGATTC
LG10	ref-58505	30.895	Chr20	3321886	GAAATTTACAGTGTATGATCACGCGATGTTTCTGCTCAACTAATGATCAACGCCAATTTTATATTCAGTGTTTACTGTTGTTTTTCCTAAATAAAACTATTATAGTGCGTTGATAGTCACGAATAGGTCAAATGTGGGTCATTTTAGGTCGGCCATAGATCCACGAAACGCTTCAGCGCAGCGTCGACAATTTTTACGTACACAACAGCTCGAGAGTCGTACTACCATGACACCTGCTGAAGCAGCGCCGAAGAAAAAACACGTTCGGTTTGCCAGGATGGAAGAGGACATTGACCGCGACGAACAGGAAGAGGATACCCTTTTCGACAAGCGTAAAGACATTAAGGAGGGGCTGAAAAGTGCTCTGAAGGGGGGTAAAGGGGTACTAAACCCAGGGTCCGATCAGGTTGATGTAATTCGACCCGGACGGA
LG10	ref-55938	31.113			CCTAATTTAGCGATATGTGTGCCAGGGCTGG
LG10	ref-4719	32.235			CCCAGAGACATGTCAACTTTGGTCCACTGTTGTCTGTCCTACATGGAAACAAACACCTCTTGTATTGGACTTCTCCATTGGGGAAACTTCAGTACATAGCCTGTTATTAACATGCAACATTGTTACCGTTTGTCTTGCTTCTTTTTTTTTTTTTTACAGAAGTAGGTCTCTTCTGTTGTGCATAATGATTGGGATAGTCCCTGCTGACATACCCACAGACTGCACATGGATCTCCATGAGCCATTTGATTTTGTTGTGGGGCCGGTGAAAATTGAATCATTGAATTTATTGAATCGAATCGAGTATCGAATCGAATTGAGATCTTGTGAATCGGAATTGAATCAAGACACCTGTATCGATATCCAGTCC
LG10	ref-25713_6	33.795			CAGTATTATATGGTAAGTCATAATTAAAAGGTTTCAGTTTTGTGAGATACTGCAAGAAAGTTTCTCATTACCAGATACTTGTGAGAAATTCATACCAATCACTTAAAGGGTTAGTTCAGCCAAAAATGAAAATAATGTCATTTATTACTCACCCTCATGTCGTTCCACACCCGTAAGTACCCTCGTACAAATGAGTGAAAACATT[A/T]ATGACGATCCGCATGCTTCCAAAACAGTGTCGACCCGCCGTGGACCGAACTTGTTGGTCCAGCACATGCCACAGATGCTCAACTGGATTGAGATATGGGGAATTTGGAGGCCAGGGCGTGTACCTGGTCTGCAACCGTGTTTAGGTAGGTAGCATGTATCAAATTTACATCCACATGAATGGCCACACCCAGGGTTTCCCAGCTGAACACTGCCCAGATCATCACA
LG10	ref-66916_6	35.741			TCCAGTAGCCTATTTAATATGATATTCTAAAATCGAATTACTGCAGTGTGCAACAAGTATCTCACAGCAGCCGCTGAGCGAATGCACAGAGTAACGTTATAACATCTTTTTCAACACAATGCATCTAAATGTATCTGTATTGTAAACATTAAGTAGTAATTTTTTTTTTTTTCTCA[C/T]AAAGCACTCGCGTCGCCTCATAAAATTGAGGTTAGACCACTGGAGTCACACGAGCCGCTTTTCCACCGTCGGGCCGAACTGTTGTCATTGAATAACCGTTCCATTCAGGGCCAGCCGGTTTCATTTTCCACTGTGAGGCTGATAACGGCCGCTCTTTGTAATGTAATACAAAAGCTTCAGTTGTTGCCTTGGTA
LG10	ref-24702_28	38.225			CGCTTGTGGGCGATCCTAGTGCTGTCG[C/T]TCTA

LG10	ref-49307_15	38.723			GTTTTTGTTTTATTTTATCCTTTTTGGTAGGTCTCTGTCCCCTTTTGAATAAATCCCTATTTTTACCACGTTAAAGAAGGTTAGTCGTTTATTTCCGATCCTCGAACACGTGATTTGGATCGCTGGTTTGTTTAGTTTAGATCGGTAAAGCGCCTCCGTTGACGTGTATGAGGCAGATGGCAC[C/T]GAACTCGTGGACATCACTGCATCTAATACAGTTAAAATGAAGAGCATTTGTACATAGAGATGGAGGATGATGTAAAGAAAGTGAAAAAGGTAAGTTAACATTGTAATGTTACACACTTGCATGATCCGCTAAGCTGGTTAGTAGCTGAAAAGTGTTGGCCTAAACAGCTGCTTTAACGCCCTTCAG
LG10	ref-52315	43.005			CCAACGAGAAAACAGTAAAGTCACACTTCATCAGCTCGACAGACTCCAAACAGAAGCCGCTGCACTTGTTGCTTACCTGCGCGTGCGCCGTTCATCTTCACCGGAGTTTCACGGTCTCTCATCTGACGACAGTGTTTCCAGAAAAACTCCTCCAGGAAACTTTATAACAAACTGATTCTGCAGCGGGATAGAGAGACAGCAAGAGCCGGAGTAGGTACGAGAGGTTCCGCCATGTTGAGCCCATTCAGCGCGGATCACACGGATGAGTGATCCGGAGCTGTGCAGCCGGCGCGAGCGGACCTCATTTATAATGTACAGCTGGTGGGCGGGGCGACGGGAACACACGCCCACAAACACACGCGCGCGCGA
LG10	ref-2128_23	46.234			TCCTGGAGGGCGACTGTCCTGC[A/G]GAGTTTAGC
LG10	ref-2128_24	46.234			TCCTGGAGGGCGACTGTCCTGCA[A/G]AGTTTAGC
LG10	ref-45474	47.725			TGTGTCAACACATTGAAACACAAAATGTTACTTGTGTGACAGGCATAGGCCTATTGCTGATTATTGCCTTTCATTCAATTAAGTTAAATTATCTTGTGGTCACAGTTTCATTCGCAGATCCTTTTATGTCAGATATGACTAACCGATACTGAGTAGCCTCTTGGATAAGATTCCTTAAAGTTTCGCACCTACGGCACATATTAGGCTAACGACATTGATGCACAAACGCGGATATGTTCGAAATGTCTAAAGTATATAGATAGAATTTTATATGTATTGAGTTGGCTTGTGCATTTAATGTGAGGTAATTTCTGGTAAGGTACCATTGTGAGCATCGATGCTCCCTGGTTTTTGCGGTGCATTGTGAGGTTTTTTTGCATTGTGCACTTGAAGGATTTTGCGATTGAGACAGCCCTTATAATGGCTGAC
LG10	ref-55470	48.232			TTGTGCGGCGGCCGGCTGGGCCTTGCCATCCACTTTTGCCAGATTTTATAACCTAGGGTTCTTCTCTTTGCATAGTAGAGTTGACCTCTCCTGAGCTCAACTCACAAGGAGCCCACACTCATGACCTGTTGCAGGATCAATTCTGTAGCTGAGCATTACCCTTGACGAGTTCAAACTCGCTACAGATTCCTTGTGGCTCATATTTAGATACGAGCCACTAGCTTCAGGTGTTCACCTTCTTTTACGTTGTATGCTCTTGGCCCTTCTTTAAGCGCCTAGGCTATACAGGTTTTTCTTGGAACGATTGTCTAGGGCCAGGCTTGACCTCTATGAGTTCAGCCCACTATGCCCCCTTGACGGCTCCGACTTATGTCCGAGCCCCTAGTTAGGATGGCTTCGCCCTATTATGTCTTTGTTCCTCTGGGCCCTTT
LG10	ref-26637	48.674	Chr20	19632553	GATTTAAGCTTTCTTTTTATGTGTGCTGTTACTGAGTTAAACACGTTCATAAGCAGTCGTTTAAAGTGTTAACAGAGAACTTGTGTGACCGCTGGTGACGCCGTTGATCGCCACTCTGCCGTGACACTGCTGACTTCACATGCACACTGGAAATTACATCATGCAGTGTTTGTCATTTTGAACATGTATTAAAATCTGACTATATAGACACGATCACGATGCTCCTTATGGTTTGCATGTACTCCTACTGTGCGTTTGCAAGATCAACTGAATCTTGATGCGACATTAATTAGTTTAGTATAGATCGAGTGAACCAGCATGATGAAATAACACTGCAAATCATCTACCACTAGCTTTTGTGCAAATAAAGGACAATTTTAACATTGGATACGTTTTAAATCATTTTACGTCATAGGTGAAGATGGCAAGTT
LG10	ref-28268	51.451			GATCTCGTCACGATATCTCTGCGCATTCAAA
LG10	ref-28656_8	53.253			CTGTGGTGTTCAATGGTTGTTTTAAAGTGTAGACTTAAACTCTATGCAATTCAGGGTATCTAAGATTTTAGATCTTTTAGTTTTAAACAGCCTGGATTTAAGACTATTTAAGCTATTTAAACTATCTAAGTGTCCACAGATACACTGTAAGGGGTTTGAGTCAGACATGCCAAAAAATAAAAACCTCTCGTCTCAGTCCTGTGGGAG[A/G]GTCGAGGGCGTTGCAGAGAACCATCAAAGACTTGGACGTAAAGGTCTTTTTCCTCAAAGTCAATTACAGCTGCTTCAGGAAATATTGATGAGGGATTTTGAAACGCAGTGGGAATCCAACAAATCCATCCAGTTCACGGCACCGGAGCTGCGGTGAATGTCAGCGAACACAGGTGATGAGAGAGAGCAGGGCTGGAGGTGGAGGTCGTGCACTATACGGCTTTA
LG10	ref-42215	53.274			GTCTAGTATACGACAGATTTGCTTTAGCGGT
LG10	ref-34034	53.536			AAGTACCTGTACACACACATCAGAGGCGGCAAGATCGCAAGAAAAGCTTAGTGCTGGTAGCTATACATTATTTAAGGTTATTTTCTGTTTAAGATATACAGAAACTGTTGTCAAGTAAGCTTCATTCATAAAGCTTCCCCTGTTGCGAGCCTGTGCGCTCTCTAAAGTGTCCGGGTGATGCACTGCTGTCACAGTTGACTGCCGTCTCATTTGTTCCGACAACATCACGGTATTAACAAACATTTCAAAAATCGATTTTTGAATCTGTAGAAGATAGAATCGCGATTCATAAATTGATTTCCCACCCCAAACACACACACACTAAAACAAAGCAAAAGCTATAGGGAAATGAAAGATTAATGAGCATGT
LG10	ref-32708_5	54.796	Chr20	8544028	CATGTGAGAGTCAAAGCGTCTCCATTTGTCTGTCCAGAAAATGCCATGCTCATCATGGGCACTTAGTTAGTGTGCTTACATAGAAATAAAATATAGATTTTGATGTTCCAAATTGGACATTGGCTTGGAAGCACACAGTTAGATGGTGATTCATTCTGTCTATACCGTATTCGGCTGATTTATTTATTTTTTTAATGTAGGGAAC[C/G]TGCACGAACACTGTGCGTGTATTAAAATACACTTTTACCATGCACAGATGAATTATGAAAGAAACTCTCGTTCCTCTGCCCCTCCTCCCTTGATAAGTCCATTACCTTGGCACTTGGCTGTGGCAAATTCACTAACGTGGGCCCGACTTCAAACCATTAATCAGGACTTCAACGCTTAGCGCACCGTAAAAAGGTTATTTGTGTAATAGATAAAAGGGTGAAATGA
LG10	ref-1424	55.505			GGACGAAGGACGAGGCCTATGCCAAAGAACT
LG10	ref-22347	57.777	Chr20	16037585	GGAGATAAGGCACTTGTGTGCTAGTGTTTTTCACAGCATCAATAACCCTCGTTTACTGCTGTTGATGATTTAATTTTATGTAATGTTTTGCTTTAATGTGGTTTTATTTTATTATTTTATTTCAGTATTACTGGGATAGTTGCACATGTCTAGACTATTTTAAATGTCTGTTACATTAACATGTTACATGTTATTTTCTCATTTTCATTTCGAATGTAACGCCACCTCAGCTTGGCTTCAGTAGGATGGTGGAGATGTTTTCTTCTTCTTGTTGAGCCATTAAGGTTCTGTTCATTTAGAGAACAGCTTTATCGATTTCTTTTGGGAACAAGAGATGTCGTCTGTTAAGCTCAGAACAAATGATTTGCGTTAACGGAGCAGGAAACGTTATCACGCCGGACCAGGAAGTATCTGGATGTTTGATTAAATGG

LG10	ref-4848_1	59.35			CAGACCCCCACCTCAGAGCGAAAATCTCTCTCTCTCTCTCTCTCTCGCACTGTGATCGTGTTTCTCAGTTTCGGTGGTTTTTGCTCTACCATTTACAGGTTTTAAGACGTAAGGGGTAAGAGCATGACCCAAGGAGAGAGTTTCGGTTATATCTTTCTATGATACGA[C/T]AGTGACAATGCATGTTACTCGGTCTGGTGAACAACTGAGATGGAGAAACCGACAGACGTTTGATGAGTAAGAGACTTTCAGGACCGCTTGAAATATTATCTATAATGGAATTTTCCAGAAACAGTGGCCTAAAAATGGGGTCATTGTTTATTGTTTTCACTTAAGTCCTCTTTAAAAGACCCATCTCTGACCCTTTCAGG
LG10	ref-25868_18	66.572			AGGGATGCACCGATACC[A/G]ATGCCAGTATCGGG
LG10	ref-45498	73.554			CTTGCAATTGCGAGATTATTGCTCACAATTC
LG11	ref-68486	0			GGTTCAGAGTCGACTCTTTTGCAGACTGTTC
LG11	ref-58332	1.397			ATGTTTCAGCCGAATCTGTTGCTGTCAGGGA
LG11	ref-4750_6	2.372			CGTTT[C/T]TCCACGACCCCCCTGCTTCCTCTTTG
LG11	ref-4750_27	2.75			TTGTGTTTTTCATTAAGAAAAATAATGAACCCACCATTCTAGCAAATCCCCCCCCCAATTGCCGCTAATTAGAAAGCGAAAGTAAAGTGCGCACGGCCGCATTCATAAGCTTCTCTGTGAACCTCTGCTTCAGGGCCGCTGTAATTTGTTGTATCACGCCTGCTATCAGGCGAATATTTTATACACACGTTCAAAATAAACGTTTTTCCACGACCCCCCTGCTTCC[G/T]CTTTGCGACCCCCAGGTTAAGAACCGCTGCATTTAGTGTTTTTAACAGCAAAAACACTCTGAACCGGTTCGTCTAGCACGTTTACATGGTTAACAATGGAAAAGCAGCGCAGTGGAATGGAAAAACGCATTCTGTGCGAACGGCAGCATGAGTCACAACTTGCATTAAACTAGGCCTGAAAAAGAGAGACGGACGGCTGGTGGCT
LG11	ref-71392_19	4.581			AGCTGTCATGCGAACCCG[A/G]TGCGCACAGGGGT
LG11	ref-15375	6.193			CCATGTGATGCAATAAACAGTAAACCAGCCTGTCATTCTCAGCATTCTTCACACGGCTCAACTGCCTTTGAGAACCACCTGACATTGAGCCCCACGTGATCAACGCTCTTCATCTGAGAGAAACACGAGGTCAGCGTTCCTCCGGCACCAGATGCTCAGGTCCTGCAGATGAAGAGGGCCAGAGAGATCTGCATCAGGATGAAGTGATCTCGACTGGAACGCCTGTTTCAGAAGTCTGTAGATCACCAGAGATCATGTTACTGCTCACACTGTTGCTTCTCTGAGTGATTAATCCCGTCCTCTACATGCACACAGTTTAGCTATTTATGGATTAACTCATAATGCTTGACATAGTGATAACCATAGCAATGTTTTGGCATCTTGTGGCTGTAACTATGACACAACATGAAACAATTCAACCCCTTTTGGAA
LG11	ref-53820_27	7.808			TATTTTTTTTTCTTCCACCATATCCCTTTAGTCCCTTTTTTAGTGGCCAATACGATAACCAATAATTCTTTCTATTTGAAAGCTGATAAATCTAAATCCAAATTTGTTATCATTTTTGAGTCTGATTACAAAAACAAAATTCTCACCATTAAAAGCCATGTCCAAAGCACACAATTATAATGTTCTCATTATAATTTTATGTAGCCTATACGACAGAGTTGCACTGT[C/T]CATATTGAACTAAACTCCTTTTTGAAGTGATTCTAATCATATTTCAAGCAATTCAAAACCATCATGGCGAACTTCATATCTGAAGTGTCTCAGCTGAAGGAAATAATCCATTATTTATCAACATATCAGCCGATACCGGTAAGGTGGCCGATATATCTTGCATCCCTAAATAGAAGTAATAAAGTTAGTTACTACAACTATAT
LG11	ref-29826_24	11.444			TTAGTTAATCAATGCTTCAGAAGTATTTTTCAATGTTAGTTCATGTTAACTTATGTAGTTAACTAACATGTTACAAAAATGTAAAGTCTTAGTTTTTTAATATAAAAAAGCATGCATAAATCTAATTCATTTGGATCGCTTTGTCTCTTAATGCTCAGATTCTTCAGTTAACCTTTGGCATCCAAATCTATAAATGCATCGGCCGCAAGACGAGGGAACTGCA[G/T]CCCAATCTGCTGAATGCCATGGAGGAGAATCCTGCAGCGAATACTGAACACCAGCAATACAGCATAGAAAATCACCAGTTTGAAATTCAAACCTATAATAACCCTCAAATGGATAGTGGCAACTATCATCACAGTAGATATGCTGATGAGAATTATGATCAATATAACAATCCTGAATTTTACAAACAGGACAACCGTCAAATGTACA
LG11	ref-49919_15	13.246	Chr4	1764200	GACGCTGACCGTGCCTGTGGTTCTCTTCCCAGTGAGTATTTCTGCACATTCACAGGCTTTGAAACACACATAAACGTTGCAGGTGTGTTGATCGAAGGTTTCTTCAATGTTTCAGATCCGCACCTCAGTCAACCATCTCCTCGGCGCCTCTAAAGACTTCAGCTGGCCGCGTCACATCTGCAT[A/G]ACTGTCGCCCTGCTGGTCTGCGTCAACATCCTCGTCATCTTCGTTCCCACCATCAGAGACATTTTTGGATTTATTGGTAGGTGGGACACAGGATGTGATGTCACAGCTTGTGTCATTTTAGTATCATTAATATACTGTTTTGGTTATTAGCATTATTTTGAAGTTTTAGTAATTTCGTTGTGTGTT
LG11	ref-64118	16.371	Chr4	8215577	TTTGGCATATTTTTTGTTTGTGAGGATTTAGGTCAAATGTAATGGAAACTTACTTTTCCATGAGGCAACATTACATCCCTGTTGAAGAGAGCAGATTGCTGTAATGGAAGGAGATATGGCTGGAAGCAGGGATTGAGAGATAAAGGAATGGAGGGTAGGGAACCTTTTCTTACTGCATCCACTGTCCAGGTGTAATCCACCTGCCTGGGGCGAGCCAGATGTCAGCTGCTTCAATGGCAAGGACTGTCATAGTTCTTAAAGGAACAGTTAACCAAATAATAAACTGTCCTTATTTTACATTATTTACTTCAAGTTACTTCAAGTATACTTTTTTTTGGGCTGTTACTTCAGAGTGAATTATTTTAATCCTAATAGGAGGGTAAGATAATTCACGTACTGTACAAATGGTGTGCCATTTCCAACACATTTCA
LG11	ref-59072	18.863			AGGCTTTACGCGACTATGATGCAACAAGATT
LG11	ref-26908_25	18.924			GACGTGCACGGCGCATGATAGAAAACAGAACGCGGGTCAATAAATTGTGTTTTTATTAATTTGGTGCTATTATGTGCAGCAGAGTAAACAACGCTTGTGTTTACTGTATGTGTGCCGCATGTAGACGTTTTTCCGCTGCTTGCAAACAGACTATTTTGAGGAGATAGCTGATTGCCATTAATTTGCCGCTCTGATTGCACTCGCAAAGCGCGAATACACTGCATT[C/T]TGAGGAGGCGAGGAACGAGGAGTGAGGCAGGTATAAATTCTCCTCCCCCTTCACATTTTTCACAATCATTTTAATTTATATTTTCACCTTTGCAGTTTAAATAAACCATATATTTAACATATTCCAACAGTGTAACCAAACTTCAACAACATAAGAGCAGATCCCTTGAGTGCAGCTGATGATGTTTTGAAATGACGCTTGAGTGA
LG11	ref-71392_18	19.107			AGCTGTCATGCGAACCC[A/G]ATGCGCACAGGGGT

LG11	ref-49098_32	21.006			ATTTACACTACGAAAGCAATGCGGTCAAATG[A/C]
LG11	ref-49098_27	23.338			ATTTACACTACGAAAGCAATGCGGTC[A/G]AATGA
LG11	ref-43918_10	24.824			CCAGTGCAGACCTCGTTGTCCTTACAAACTCTGTTTTGTGTGCAGTTGTTACACTTGTGTGGTATGAAGGATGGGCAGGGCGGCTGTGAGCACTTGTACTCAGCTGGCTGAGGGTGTGTGGCGGAGCCCCAGCCAGACGAGCACGAGGCCTTGCAGACAGTGGATCCACCTTCAGATT[A/G]GCACTCAACTCGCCCGTTTACCTGCTCATCGTAGCACACAGGTAGGGCACTCCACTTCCCTCCAGAGCACACCGCAATCTCGGGGAAGGGCTTGTTTGATCCACCACATTTTATTTCACCTGCTGTTGGGCTGTGCTGTGGTGGGACTTCTCGCAGTAGTTGTTCTGAAGATTCACAGCCTTTAGAGGGC
LG11	ref-23940	25.978			GAATGATGTGGCCATCTCAAAATGTACCATTTCAGACACTAATATCCTATAAAAAAAACTAGATCCATTTTAAATAAACAAGTTCCTTTAATTCAGTATATATAAATATATTTTGTTGTACAATTCACAAATGATTAATCTGCAACTTTAAGATTAAATGTAGCCTAAAAAAAAATGGTGAAAACTGGTTCTTAGTTTTGAGACCAAAGTCGATTTAGTCGCCACCACTTTTTGAAAACCACTGAAAAAAATAGCTTGTTGAAAGCATATGTAACACAAAGAAAAAAAACATCTCCAATCCGTCATAAATTAATAAACATATGTAACACAAAATGCAACTGCACTCACACATATTGTTACAACACGGACTACTCAGAGACAGGAAGTGCAGATAGATGGATTTATTGAACACACAGCAGAGCAATAGGCAA
LG11	ref-66565_30	26.752			CACTCGTTTATATGTCTGGAGCGGGAGGGATGGATACAAAAAGTACAATAACTACCAGGTCTGCTGCAAAGATCTGTGGTACCTGGAAACAGGTGAGAAATAAAGGCTGGCAGTAACAAAGTACACTTTGGAACATTTCAATGAACAAGTTGTCATTTGTGTGCGTAGAGAGGCCTTCCACTCCTGGTGCGGTGTTCCTTGTGAAGTCCACGATCAGTATGCTGCATGT[C/T]GCCTGGCGGCCCCTGCCAGCCGCTGAGTGTTACTTGGTTCAAGTGCAGCCGATCACGTCTCCTCATTCCTCTTCAAGTGAACCACAGTCCACACCAACTCCTCCACCAGACCCGGGAAACAGGAAGGAACATATACAAAAAGGTATTACAACATTTGGTATTCTGGGGTGTTTTGAACATGTTTGTTTTGCAAATTCAGCCA
LG11	ref-13158_8	27.207			TTCTGAGGTAATGCAAGTACACAAGTCAAAAATATATAACATTGTTCTAGTGGTTTTTGGATATTTTTATCCAAAAATCTTACATATTTACATATCTTGCCTTTAATAACCCTCATGTCGAGTTTTCAGTTCATAATCATTTTAATCATATGGCTTCAAAAGATGTTGTAGCAGGC[C/T]GAGCAAAACGATCGTGACAGAATTTTCATTTTTGGAATGAGAGATCCTTTAAAATTTGTATCTTTCCAATAAAGATCCCATTCATTCTTTTAAATGCCACAATGACATTTGTTTCTGTCATTCACTTAATGCTTGTCATCAATCTGTGGTATGGCGGTAAAGTCAAGTTTCTTGTAACGGCAATAGTGCCATG
LG11	ref-38853_5	27.241	Chr4	6146069	AATGCAGCTGGAGTTTTTCATTCGTGAGTGAGCCGAGATGAATATCTCGCTACATAATTTCCCTCATCCAGATTTTTTAAGACCACGGAAATGTATCCCATTCCATATTAATCCTACTCTGTACAGCGATGCTACTAAGGAGCTCATATCTCCGTGCCGCCCTCCCCTTCCTCCTCCTCTTTTTTCCGAAATTAGCACATTGAA[C/T]GTCTCCGAGTTGGCTGCTAATAACAACAGCACCTCGGGATGGCGGGAGCCGTTCTGATCAGCCAGACGACGGCTTAACGTTTGACCTTGGGACACGATATTAAGCTATTGTGGAGACCCGCAACATAAACTTGAGCTTATAATGGTCTAAGCACGCACGTGTAAATCCACATACAAATATTCATAAGATACAAGCGAGTACAATCCTGCTTATTGGGGAAACCTAAA
LG11	ref-8088_23	28.436			AAGGTTCAACTAATATATGTTCAGGGTGACATTGTTACATCAAGTCAAGTCAAGTGTGTTCTGCCCATGTCCATTTCCTATTGTCTCAAATGTCAGTCACCACATTTGTTTCTTGTGTGCTGTCCATAACGGTCCGTGAATGTAAAATGTAATGCTTGTTGTCTGCAAGTATCATGGAAACCCAGGCTCATTGGAAAAACGTACCTCTGGCGACATTTATGC[A/C]AAATGATATTGCATTTCTTCTTGTACATTTCATGACACTTGAAAAGTGAAATATCCAGTGAGTGGCGCTAAAAGCGCATTATCTGAAACCGATTAACGTGAATCTTACAACACTTGGTTGTTGTACATATCGCGGAAGTTCGGAAATGGAATGTTCGTTGAGTGGCGCTAAAAAATTATATAGGCTATCGCATTTGACAATAACGTAGC
LG11	ref-60148	29.15			CCTATTTAGACACTTGTTAGCAACAGCATTTTACAAGAAAAGTAAAAGGTTTTAAAAAATTTCAAGAGTGTATTACTGATGTATTTTATATCGTGAAAATATCTTGAGCTTGTGTTAACCACAGAGCTTATTTCAAACATTTAATCAAAAACCCATTCAAAAACCCCACTGATTACAGGGCGATAGAATCGGAAGTGCTAAAATGCTAACATGTTTCTGGGTTTTGGCCTACAAAAATGCATCATCTCTGCACCCCTCTACACAAGGGAGCTTTAGCAGAAATGTAGCACACCGGCTCTCTCTGCTGGACACAAGGTGAACAACAAGCTCAGTTTCATTTTGACAGTTATGGTTTTTGTGGGCCATTTG
LG11	ref-58940	29.868			ACTCTCTCAGCGAGAGGAGTGCACTATAACA
LG11	ref-66019	30.442			GTTTATGAAGCGACGCGAGTGCTGTTTGCAC
LG11	ref-8581_32	30.828			GCTCTCGAGCCGAGTGTTGTGCTTGTCTCTC[A/T]
LG11	ref-8581_7	30.848			GCTCTC[A/G]AGCCGAGTGTTGTGCTTGTCTCTCA
LG11	ref-71353	31.677			CTGTTCTGGTCGAGTTGAATGCCAGTACTTC
LG11	ref-16402	31.784			CGCTATCTCACGACCAATGTGCACGTATTTT
LG11	ref-45437_24	31.858			CTTTGTGAAGATCTGAATTGGCCACTAAGAGATGGGGGTCTCTAAAAGAGCTCCCGCCACTGATAATGTGTCAGATGAAGGTAGCAAAAAAACCATGAAGGATGATGCAGTTCATCTCAGGAGGAGGATAGGCCTGCTGCCATCCATTTCCTTCATCATTGGCACTGTGGTGGGCAGTGGCATCTTCATCGCC[A/G]CCAAAGGCGTATTGATGAATTCGGGCAGTGTGGGATTGTCCCTGATGGTCTGGGCTTTGTGTGGCGTACTCTCCACATTTGGTGAGGAACGTCTTCGGTGCTTATACACTAATTTTTTTGACTGGCTGTGTATACTATTTTGACTTTTAACTCTCCCATGGATAAGTTCAAAGCAG

LG11	ref-57227_14	32.117	Chr4	11249684	TTGCAACCACACTGTAAATGTATATCCTACACGTATGTGGTCTGAAGCCTGGGTGGTGCGTTTCGGGATAAGTTTGTGGTTTATTAAACTACGAAAGAGGAAAAGGAGATGAGGAAACAGCCGACGGGTGACTTTGAGGCAATAGTGTGGTAAACGAGTGGGCGGTAGGGCGAAAAACAGCA[A/C]ACGTGTCGTTTTATCCGAGGGGTTCAGACTGAAGCGTAATCTTCGTGGCGCAGGAGATCAGTCTGAATGTGAACAATTAAGGAGATGAGACAAAATCATTTATCAAGCTTCCTAAGACAAACCCCCCCTGCCTTTTCTAAAAGGGAAAGACAGAATGAATTCATTTCCTTTATCTCCTCAAGTCGTT
LG11	ref-72244	32.518			CGGCTTCCAGACGCTCCTAATATAGCCAGAGAAGTCCAGGAGGGAGGCGGAAAGGAGGCGGACCAGGGGTGGAGACAAAGAACTGAAGACAAAGGAAGAGCAGGAAGCCAAGGAGGCACAGGCTGGGTAGGAGGCCAGGCAAGGTGTCTTAGGTTAACAAGAAGTTCATGTTCACCTCTGCAGTTGCATCGAGGCAGACAGGAAGTTCATACACAACCTCTGAGGTCACATTGATGCCAACAGGAAGTTCAGGAACAACCTTCATGGTCGCATCAGGGTATACAGGGAACTCAGGGGCGACCTCCTTGATCGTACCAAGGCAGACAAGAAGTTCATATGTCACGAGACTGGGCTCTGGGACAGGAGCGA
LG11	ref-21271	32.882	Chr4	12948243	CCTTTTAGTTTTTAGTAGTTGCAGTCCTTACTTAACTTCTTTTCTAAAAAGAAGTTTGAAATTCCTTAGGTTTGGCATTTGTTTGTTTTTATTTGGATCATTTTAATTTGTGCTGTTATTGTTGTCTGACTAATTTGTTCCTTTCACAGTGTTGATGAAGACGTGTGTAGCTGAACTCACTGAGCTCACAGCGGCTTCCCTCAAAGCCGACGGCACAGATGCATTTGCCAGGATGAAAACACGTCCCTCCATTCAAACAGGTGGTGCTGCAGTTTGCTGTTAATGCAGAAAAAAAAACAAGGCAATGAACAGGTCACAAACTACAAAGAGAAAAAATAACGCACAAACAATTTGTGCTTTTATTTATTTATTTATCTAACAGTTTGGTAGATCATGCTTCGTGGTTTTGGCCTTTTGGTCTAAATCATTTAA
LG11	ref-48410	33.178			GCTAAATGTGCTGGGAATTTCACTTTTAAGTCATGCTTTATGAACTCCCCCCTCGTCTGGTGGAAAAGAATCATGGTACTTTCTTCCAACTAATATGTAAACATGTATTTTTCTCTTACCATTATAGGTAGGACATCTTTGTTGGGCATGTCACCAATCAGAATTTAGTGTGACTGTTTGCATTAGACTTGTGGTGTTTTAGCAGAATTAATTTTAGCACCTCTAGTGGTGCCAAATGGAATTGCAATCTGTTTGTTTGAGTGGCTCAGTTGGGCCCCACATAACATTGGCAATGGCTCAAGTTGGGATGAAGCAGCCTGTTTGTCCAAACAAAGGTTGATGGGGGAATTGTTTGAAATTAGTTTGACA
LG11	ref-23710_25	33.835			TCGAACCGTTCGATAGTGATGCGC[A/G]GATCGCG
LG11	ref-23710_32	33.835			TCGAACCGTTCGATAGTGATGCGCGGATCGC[C/G]
LG11	ref-27433	34.005			TTGTCTTTTTCGACAGATTTGCCAGATGTGT
LG11	ref-25608	34.585			GTTGCGCAAACGAATCCTCTGCCATGGCCTT
LG11	ref-49134_28	35.042			TTGAATATATGTGTGCGTGTTAACGAAAATTAGCTCTTTAGAAATTAGCTCTTTTAGATTCCAACTGTGGCCGCAGAAGGAAACACCGGGATGCACGCACTCGGCGCTGCAGGCGACTCGTGAGT[C/T]ATTTTTACTTTCTCAAAAAAACTCATTCTGTATGATTTATAAGCCTTTTGAAAAATGGGGACATGGGGTAATGTCCTCATAAGTCACCCTCTCCTTGTAATACCTATGTCATACCCATGTCATTATACAAATTTGTGTCCTGATATGTCACAAANNNNNNNNNNNNNNNNNNN
LG11	ref-38899	35.586			TTTAAACTTCTGTTTTTGCAGTAGCTCTGTAAGGCATCTCTATGGCATCACTGTCGAAGAGTAATTAGCTGGTGAAGAGTTAACGAAAGTTATCATGAGCATTGTAATGTTTGGAGCTGATATCATAACAAATGTCACTAGACTGGAAAGAATTTAAAACCAAATCTTACCTTCATGTTGTGTAAATATAAACCTGAAGACAGAATGCAGCGAGTGTAGTGCTGAAAAGGGCCGGGGCTACATAAGGTCTATAATAAATAAATAAAAAAACAAACATTCTTTTTATCGTAATGCCAAAAAACCTCTCATTCCTACGTAACACCTAAAGCGACATGGTGATGGAAAAAATTAGATGGGGTTTTTTTTTTGCGAGTGAACGCAAAGTTTCTCAGGGGAATGCAAGATATTTGCGAGAGAACGCAAAAGCATTT
LG11	ref-54624	36.11			TGTGAGATTTCGATATCGTTGCATCCCTAGT
LG11	ref-16427	36.358			AGTCAGCTGCCGATCAGTGTGCAGCGCTGGA
LG11	ref-16405	36.6			TTTTTCCCCCAAGGATTCTATAAATATCGCGATATATCGATATTGTAAGAAAGGCAGTGCAGATGATGCAGTAATCGTGTATGCACATCTTTGGAGCATATTGATGCGCTCCAACACTCTCTGAGCGCACGCGACGACCACGGGCAGCGCTCTGTTGCGCGCAGATTCAATAAAGATGGAAAAGCAAGTGCGTGACATGGGACTGCGCTGCTGAGAGAACTCACCGTGTTCGGAGAGTTTTATACTTATATTGTGTGCGTCAATGCTAAATAGTAAGTGTTTCTATTTTCATCTGTTAATAATTAAGAAAATCATGTCGGTGTGTTATATTTTGATATTAATCGCTAATGTCTGTCTGACGCCATGCTAGAGTTACTGTTGTAATTCCCTTGTTTTATCTTAATTAATCTCTGTGTACAT
LG11	ref-47856_15	37.045	Chr4	12189827	GGGATTTGATCTAATGACAGTAAACAACAGTGGAGCGTTACAAAACACAGTGGTGTGAAATTCTATGTGGCAGCCAGTCAAAAACCACCTCCTCTTCTTTTAAGTCTAAATTACTTTGGGATTTGCCAGGCAGTGGACAGAGAGACTGTGCAATGTGACTAACCAGATTACTGCTGCACTTTATGGGGGAAGATGGAGCCACGAGTATGACGAC[A/G]ATATTGCTGGAAAAAGCACCAATTGAGTGTCAATATGGAGAACTGTGTCATAATGAGGCAGGGAAAGGGAATTTATGTTCACTTTTGGGAAAGAAACTAAGCTTTATAGAGCACTGCAGGGACTTGTTTAGTCAACTCAGTCAGTGAATCCTTGAAGCCAAGGATCATATTTGTGAAAAATACACAAAGTTCCTTGTGATTAATATTGGAATCTTGT
LG11	ref-65224	37.366			ACAGGTTGTTCGACCCTCGTGCAAAAAAACA
LG11	ref-44114	37.985			TCGACCTTCTCGACTTCCTTGCTCACCTGCC

LG11	ref-25079_32	38.458			ATCACAAAATAAACAAGCAACAAAGTGAAAGAAAAGCGACAGCCCCTCACAGACGATTGCCGCCTAAATGAAAACAAAACAAGAACTTAAAATGTCTCAGGCCTGGTCC TTTCTCGTCTTCCACTGTCATCACTCCTCCTTTTATCCATCCGGAGCTCCTCCATGGGACTCATAGTGTCGCACAGGTGTCGCTCATAAAC[A/G]TTCGCTCCACCCTGCCTCT TGCCATATGGGGGCATAAATTGGGGGCAGTTGTGGCCTAATGGTTAGAGAGTCAGACTTGTAACACAAAAGGTAGCAGGTTTGAGTCCCAGTACCAGCAGGAAATGTAGG TAGGTGGGGGAATGAACAACACTCTCTTCCACTCTCATTA
LG11	ref-49527_28	38.93	Chr12	23145973	CCTACATTTTAAAATCTTTATTTGAATGACAACATTTTTATTTTAAAAGCTTTATTTGAATATGGTAACATGATACCTCATTCTATTAAAATATTTCTATGATTAAATCACCG ACAGAGACCTTTCCCCTATCAGCCGATCACTGTGGACATAGTTAGTAAGCGTGTTGACATGTCATAGCAACAAGGTCGACCCC[C/T]CCCCTTCTCTTAGCTTAACACTTATC TCTTTTCCTTAGTAAAAGTTTGTCTCAGCAGCTTTGTAAATAAGTTTTAAGAGAAAACTTTAGCTAAGAACTTTTACTGCTATTTAGGAGAACTCTTAGTGGTACGATAAAA TGTGTTGTGAATACGGCCCCTGGTGCAAATTTGTG
LG11	ref-54352_10	39.388	Chr4	21060584	TGAACGTGAGAACAGGGAGTGATATATTTTATGAGGCAAATAAGATGTGTCAAAATGTGGTTATTCACCAAAGAATAGAACAAATCTTGCAGGAGAGACGCAAATCATCT CCTCGTCTTTGTCTCTGTGGGTTCACCTTCTCATTTCCCATTATGCCATGCGTTTAGCATCTAACCGT[A/T]GCAGATTGGTCGTGGGGGATATGCATTTGTCATACGTAATGT GTTTTGTGTTTACATGCCAGCACGTAATAGACTTGCTCTGTATGTAAAATCATTCAGCTCCGGCCTCGTAACAATCCAATTAAGCTTTAAATGAAGCCAAACAGAACAATAT CCCAGTGTAAATGGGCCCAGAGGGAGGGCAAAGAACA
LG11	ref-14364_28	39.794	Chr4	22259003	TCTCCGGGGCCGCCCACATAGCCGTCGGGTAATGTGTGGGTAACCCACGAGGAAATGGAGACCCTTGCCGCCCCCTCGAAAACGGTCAGTCACCTCATCCTTTTCAACACA AAAAAGCCCTTTGCTTACTCTTACTGACACTTTTTGCTAATGTTTTTGTTTCACAGAATCAGAATAGAGCATGGAAGTCGACATG[C/T]AATCGAAATGAGCATCATTTACTT TCGTAGTACACGTTCCTGGTGTTACTGTGCTCGATTCAAGCCGCAATAATGGCATACAACAACAAAAAAAAGAGTGTTACGGATAAGTCAAGATGCTCTCGAGGGTGACTT TGACCCACTTCAAAAGCGGAAATACATCAAATTAAGGA
LG11	ref-53202	39.894	Chr4	22469590	TAATGCAGTGAGGAGCCCTCTGTCTTTCGCTGTGTGCATATTGACAGCGTTTATTAAAATAGAAACTTGGGCAAGGACCAAAATAGACACCGGCATCTTCATTCCCTACTTC ATTCTGTTATCAGTTGGCCACTCATATGAATGACTGCAAGGCATGATGGCCACATCCCACAGGCATTCATCAAGCTGTGATTGACAGCCTGGAGAGAACGAAAGGCCTGCA GATATCATCCGCTGCACTCCACAGCTCTCTCAGTTTTTTTTTTATCATGCATTCATACTGGCGACAAAATCTCAGTGTATCGTTTGATGTCAAGGTAAAGGTACAAGAAATT ATACTTAAAGGATAATTGTACCCAATATATGAAAATTTTGTCAACTTTAACCTTAATATTGTTCCTAACCTGCATAACGTACTTTCTTGTGAGGAA
LG11	ref-57969_16	39.938			GGTAATAGTGTTTTGCATCGACTGTGACAGCTGAACTGTGACTGTAGACGATCACAGACTGACACACACACACATCCCAGCAGTCAAACTGCAGTTTAGTTTTTCTTTCTCA AAGTTATTTTTTATGCAGTCCTTCTTAAAGCATCTCCTGAAAAACTACTAAATACACAAAGTCTGGTGCACA[A/C]TGTTCGATTTTGTCCATCTGAAGCATAGTTCATAATC ATATGGTAAAACGTGACGCGATTGTCCTAAGATGTATATTAGACTGTTGAAGTTACACAGGCTACGGTGACTTTTACAGAACCGATCAACGGGATCAGACAATAACGAGC TCACAAGCCAATAAATGAAAAGGAAATCGCGCAGTGTG
LG11	ref-71160	40.001			TCTCTGCTACCGAAATGTGTGCGTGTGTGTG
LG11	ref-51188	40.296			GCAGCGCTTTTTTAATGAAATTAACAATAATCACATTAGGGAGTATCACGATTTGTTTTTCCATATTCCAATATAAGACGCCTTAAAATGATTGTTTATACTCTTGTTTTACT GTTTAAGGCACTGAAGACTTTTAATGACCTTAAATTGATCTGATGAGCATGAGCGGAGTGGAATGATGAAAGTGATCAGCATGGTTGATATTCAAAATGATAAGCATGCG CTGCATTGTGTGTTTATCTGCAGTCTGTAGAGCCGGTTTCCCCTTTTGAATGATGAATACAGATGAATTTATATGAAGAAATAGGCAGTTGTCTCCTTTACGCAGGAGCAGA ACGGTTTGTTCAAATGCAGCTGACGCAGGCGACAACACATTCGGCTATCATCAGCACGAGTCTTTGGCGTCTGTTTGGTGTGTGAAATTTTTCCTA
LG11	ref-1797	41.332			GTTTAGATGTCGATGCCAGTGCAAAATCAGA
LG11	ref-64295_14	41.52			CTTTAAACTTCGA[C/T]ACCGCTGCCCTCACTGGC
LG11	ref-44585	42.683	Chr3	34755147	TGGTCGATCGGATCACAAGTAGACGATGCTAAAGACAGGTGTAAACTTTAGACCACTTCTACAAGTAGTCAAAAACGACCGGATTGCTTTCATAGTGTAAACGCTCTAAA AACGACTGGATTGCTTTCATAGTGTAAACGCTCATGTGGTCGAATGTGTTTGAATGACCACAAAAGACCACCTACTCTCCGCCTACTGACCTAACGGTAAACATTACGGAA AGCGCGCTTGCCAGACGGGATTAAACTTTGTCGGCTGAAGACCCAAGTTTGGTTTGAAGATGAAAAACCTACCAA
LG11	ref-62799	44.78			ATGTCAGTCCCGAAATATATGCAAGTAAGTG
LG11	ref-18052	45.317			CACTGATTATCTCTTCATCACAGCATCTGTTAATGGGTGGGATATATTTTGTCCTCACAGTTGATGTGTTAAAAGCAGGAAAAATGGGCAAGTGTAAGGATTTGAGAGAGT TTGACAAGGGCCAAATTGTGATAGCTAGATGACTGGGTAAGGGCATCTCCAAAACTGAAGCTCTTGTGGCATCAGTATCGATCAAAAGTGGTCCAAGAGTGGTGAACCGG CGACAGGGTCATGGGCGGCCAAGGCTTATTGATGCACGTGGGGAGCAAAGACTGGCCCGTGTGGTCTGATCCAACAGACGAGTTACTGTAGCTCAAATTGCTCAAGAAGG TAATGCTGGTTCTGATAGAAAGGTGTCAGAATACACAG
LG11	ref-3779	46.182			GTGTCTTAGGCGAAAAATGTGCTCCACCAGA
LG11	ref-14074	47.351			TATCTGGTACCGAGTGCCTTGCTCAGGTTCT

LG11	ref-48315	47.388			AGTCAACTGACGACAAACCTGCAGGGAAACT
LG11	ref-21653_32	47.815	Chr4	11570137	TAAAATTGGTAACTTGTTCGCACATTTACTATTTTCAACATGGTGAATAGTGTACTTGTATATAGGGGATAGTTAACGATTCAGACAGTGTAAATATGCTTTCCATTGAGACAAATTAAGTCTGGTTTCATGTTAGTGCCACGCGAACCGCCGTAGCGCACACACACACACACACAGTCTGCTCCGGTCCAGAGACACGAGATCACAGAGCGAATCATATGCACAAGTTGA[C/T]GCAGAACACGAAACGTTTCAGACCCATTTGAATTCTGCACGGAATGCTATATTTTAAAATGAGATTAGGAGGAGAAACTGTTAGAGATTAGAAGGAAACTGAGGCTTTACAGAGCTCAACAGCTCTGGCCGAGCATGATATAACCGAAACGTTATTGGTTGTTTAAAAAAGGAGGAGGAGCTGTTCGATATGTCCCGCCC
LG11	ref-50293	48.41			AGATCAGCAGCGAAGACACTGCTTAAAGGTG
LG11	ref-62286_32	48.821			TAGTCTTGCCAAGTACATTCTCACTACCTGCAAGATTCCCTTTTTGACATGCTGTAAAACCCCATATAGTGTGCTTTAAGTATTATTCTGAATTTTATAAAATATATTGCATGCTTTTCCACATATAGCCATGCATTACCGGACACAAGCTGTTGAACAGTATTCTGGGTGTCAGATGAGCATGGATCTCGCAGTGAAAT[A/C]ATCAGCATCATTAGTGTGGGCGGCAGCTGTATCGTATTTGTACCTTTCAGACAAAAATGGGGATATTTATTAATCCTCAGTATGAATATACTAAATATGAGTATGATGTTATGCTTTTTTTGTCTTATGTATTACTTTACTCAAAACTACATTAAAATGCAATTCACAC
LG11	ref-52123_26	49.022			CTGGCACTGGGGCAGACAGTATCAGTGGAGCCCAGAGAGAGCACACTCGGATCCAAAAGCTCGATCAGCTCGACATCCTCCTGCAACAGCACGTCCTGAGATAACAGACAGCGCAGAGAATACTGGCGGAAGGCAGCGTCCTGCGGGAAAACAAACACAGTTGAACACAATCTCTCTGTGGCTGCAAATCAATCTAATTATCGGATCATCCGACTGCCTTGCAGC[A/C]ATACTGGGATTCTGTACTGTAATAAACAAAAAGATAAGATCTTAGTCTCTGAATCTCACTTTTCTCAAAATGATTTTGAACTAATGTGGCATTAGTTAGATCCTGTGGAGCTGGAATCATGTAAAACTTCATTTGAAAAAAAGGTGACTAAAAATAAAAATAAAATAAATTACTTTAAATTCACTTTATGGTTATACAGAAATCAG
LG11	ref-62604_6	49.546			TACAA[C/G]CCCTCGAACGAAGTGCAAACGTACGG
LG11	ref-62604_30	49.642	Chr4	27733143	ATATATATAAATTAGATGGTCATGAAAATGTATATATTTTTTATTTTTATATAACTGTTGAATATGCAAGAAATTTCTCCTCCTTTCTCTCTCTTTCTTTATCTCTCGCTTCATGTTTAGTTCACTCTCACACTCCACTCTCATTAGGAAACCAAACACACTATTGTCACCGTACGTTTGCACTTCGTTCGAGGGCTT[C/T]TAGTAATTAGCAGTCATATTAGACGATAGCCCATAAGAGTTGTTTAACGCTAAAACAATTCTGTTCGTCAGAAGCGCCTCAGAACGTGAACACTGTTTCCTCTTATCTTGTCTGTTTTGTATGAATACATTGAGGTTCATCAGCATGCAAATTGCTCAAGGTGAATTTATT
LG11	ref-998_16	49.944			TATAAGTTACAACTTACGCATTACTAAATATTTTGGTGTTGCACCATTACACTGCAATTCTACGTTCCAATTAAATATTTACGGAAGCCTCTGCAGTCAGAATGAGATGGCAGCCAGATTAGTTGAGCTTGTAATTGATAACAAAGAACCACATGTTCGTCGTCTAGTTTTATAAGACATACACAATCCTATGGATATTTATGATGATGTCGACA[A/T]ACATGCTCGTTTATGCTTTAAAAGAGAGGGAATAATGCAGATAACAGACTTGATAGCATCTGATATTCAACACGAAACAGACTGAAATGGAACTCTCTCGCCAGGCCTTCAAGTGTGCGTAGCTTTCCATTATTTTGCCACTGAATCAAAGCAAAACTTAGTAGGCGACAGCATACAATTCACAAATCTACAGTATGTAGAGTAATCCGCCACTGG
LG11	ref-35921	50.634			CATTTAAGCACACACTTCACACTGGCACTTTGTCTGGTCTCAACTTCGAGTAAGGACTCACTTACCTGCACCATGCGCCTCCACTACTTGCCTCCATGCATCGTTGTTGCTACGTCTCTCCCAATCATCCTTGTCTCCAGCGTCTTCCCATTTGTCTGTGTCTCCAGCGTCTCCTGTTCCCAGACTCCTTCCATGTTCCATCATGCTCACCGAAGTGTGTGCTTGGCTGCTCGAATCCACATGCTCATAATCAGTATGTATAGTTTATTGCACTCCAGATCTGATTAGATTGTAAAAGGTGAGCTGTGATATTATTGGTTAGTTACTTTTTTCTACTTTTTTTCCTCCTTCGGTGAGTAGAGTGCAGAGACGGGACACTAAGAGAAAGTAAGCGATTTGGTAAACAAGACCGAATTGCAGAGAAGTAACAT
LG11	ref-56269	51.359			AATTTCAAACAGCAGTGAGCTACACTGTCATCACATGACCTCATACAGCGAGTGACGTCCCATTATCATACAGCTTTTATTTCTCACATTTTTAATCCAATTTTATGTTAAAACGTGAAATTCTATGAGTCTGATCAGATGACTCAAGGGATGTTAAACATCATGGTTGATATCACTTCCTGTTCATTCATTACACAGGAAATGGAAGGTCGAGAGCATTGCCTTGTGGGATACGGTGTCTTGTGCAGTGGGCTCTGTTGCAAACAGGCTTTAGCACAGGTCTTTCTGCCAGTATTTTAAGTTATTTGGTTTTTATTTATTTTATAGGATTTTATTTATACATAATTATTAATATTTATTTATATGTATTTATTTTCCTGTCTCAGAAAAGATCTCTGTTACCTGACAAACACATTTTAAATTGCTTATA
LG11	ref-39987	52.28			AAAGTGTTACCGATATCTTTGCAACGAACAC
LG11	ref-16632_24	52.734			AAGACTTGCGCTCTGCGCTGCTGCTTGAACTTTGGTGCAACAGTGATCTTTGCAGTTTATCAACCATCAACAGAGTTAGAATCCACAGAGATTGATGTTTTTTAAAAAATGTTTATTTTATTTTTTTGCATGAATTTGTGTACTCTTTTGGGTGCTCAATGTTGATAGCATTATATACATTGCTCTGGGATATACTGAACATATTGAATCCGAGCCTGTTGCT[C/T]ACCGCCTGTCCTTGTAGCATTTTAATCACTTTTAATTTTAATTGAAGGTTCCGACACCAGGATGTTTCGAAAAAATAAAATTAATTGGGTGTACTTATAAATAGGCTGTATTCTGAAAAGATTACATGAAATAACACAGAGCTTAGACACCGTGCGTCCTAACACCGCATTACCAAACTGGATGTCATCACTGCTATTTTTGATGTC
LG11	ref-30400	53.287			ACTGCATTAACGAACTGCATGCTTTCGTTAT
LG11	ref-19610	53.907			GTGCTATGAGCGAGGAGGATGCACAGGTGCA
LG11	ref-64760	54.017			GGCCAAATACCGAGCTCAGTGCTTAAGCTAG
LG11	ref-45476	54.126			TGAACTGAGGCGAGTGCTGTGCTTAAGTATC

LG11	ref-44198	54.177			CATGTCTTGGCGATAGATCTGCATACCCCCT
LG11	ref-45965	54.977			AGGAGGAGGACGAAAGGCCTGCAGCACAATC
LG11	ref-21563	55.203			TGGCCAGTGCCGAGTGTAGTGCAAGCCTGGT
LG11	ref-7758	55.21			GATCAGGATACGACTGATCTGCGTCAGTATC
LG11	ref-39262	55.232			GGTTGTAGTCAACTCTTGTGTGGGATGTGTCCAGGTACAGCTGTGAATGTGCCCTGGAAGTACAAAGAGAACCTTAACAAGTTATTCTGGAGGCTAAAGGCCATCTGTTTA<br>TTCGTTTGTTTATGTGTTTGATTTTAAGATGGGTAGGAAGTTGCAGAATCAGGTCGTCCACACTTCCGTGGTAGAGGTCGGCCGGTACAGTGTATTGCTCGAAGTCTTTGCA<br>GAGGAGAGTGAGGCTGCCATGTTGTTATGACAATCTCAACTACTACGGTTTCCACCTTCCTCTCAGCAGTCTGAATTTTAACAAAAATACAATTTACACTCAGGTTTACATC<br>CACTTAAAAGCTCAATAAATGTATAATCCACAGTCTAATGATTTGTATACACCAAAAGTGAATTTAATTATTTGTAGATTAGTAGATAAGTAGATT
LG11	ref-48570	55.236			TGGAGGAACTCGACTGGCCTGCACCTTAACC
LG11	ref-50443	55.324			AAACTGCTTACGATCTGAGTGCTCTACGGTC
LG11	ref-40591	55.63			ATGTGGGCATCGACTCTGTTGCATGGATTGA
LG11	ref-71747	55.674			ATTGTTCCCTCGAGTGAATTGCCATGTGTTA
LG11	ref-62280	55.799			TTCAATTGCACGAAGACCTTGCAAACACTTC
LG11	ref-31835	55.915			GCTGAGACCCCGATGAGCATGCAGGACGGTT
LG11	ref-60951	56.281			GTAAAGAAAACGAACAGGGTGCAATTTTTGG
LG11	ref-8764	56.333			CACCAGATTGCGATGTGATTGCATGAGTAAA
LG11	ref-4998	56.579			ACGTTGATTACGACAATACTGCCCTCCTTTC
LG11	ref-8587	56.837			ATCTGATGTCCGATGATGTTGCACATTCAAA
LG11	ref-29570	57.284			CACGCTTCTTCGAGTACTGTGCATATTGCAT
LG11	ref-20914_18	57.536			TATTAAGTCCCGATGAT[A/T]TTGCAGTGGCTCCA
LG11	ref-56869_16	58.064			TTTCTCCTCCCGAAT[A/G]GTCTGCAGGATCCTTC
LG11	ref-44757	58.8			TAAAAATTACTATGATGCTTAGTCCACAGTATATTTAAGCTCCATGCATGTCTCTGTAAACATATCTTACATTTTTAAAGGAAAACATTAAAAAGGTTGTTAATTAGCACTG<br>CATTATACTAAATTGTCAATAAATACCTTGAGATGGCTTGAAAATCACACATAAACCATATCTTCCTGCAGTGTCTTCGTTGTCTTCACGTTTCATCAGTCAATACGTTTCTG<br>CATTTTATCAAAGAGCTAAGAACCCAAGAGGCGACACTCTGATACTTTTTTGGCAACAGTCACTACGGTATCNNNNNNNNNNNNNNNNNNNNNNNNNNNNNNNNNNNNNNNN<br>NNNNNNNNNNNNNNNNNNNNNNNNNNNNNNNNNNNN
LG11	ref-1052	58.951			CCATTTGCTCCGAAGAATATGCCACACCTTA
LG11	ref-5708	59.3			AACCCTCAAACGAGGGGAGTGCAGAGCTCAG
LG11	ref-48087	59.56			AATGCGGCCCAGCAAGAACCACTGTTACAGTGCACGTGGCCTGGGTCTGCTTCCACGGTAGAGGGGGAAACAATGAGGGCTCTGTGGGAGATAATACAAAGGAGCTCCA<br>GTGACCTGGACATGCAACAACAACATGCAACTGTTAGTGGAATTCCAGGACTGTTTTGCCTGTGATGAAGGGGACCTGGGTCAGACTTCCCTGGTGCAACACGACATTGCT<br>GCAGGGGATGCGGCACCTATATGTTAGTGGGTGAAGAGCCCCTGGACATCCCCTATGGTAATGGTCCGAAAGAAGAGGGGGAGTGGCACTTTTGCTTTGATTACTGGAGG<br>CTCAATGAAGTGACACAGAAAAACTCGTTCCCTCTCCCACACGTGGACGAATGCATGGATCTCATGGCTGGCTCTTGCTGGTTTTCATCATTGGACTTTAG
LG11	ref-24921_4	59.616	Chr4	73574791	GGAACTGAGAAAGCAGGGTCATTAGGAGGCACGGCTCGTGATCATCCATTCATTCAGGGATCTGATGAGACGTTTATCAGTGGTGTTTGACACCGGTGCCGAGAATTAAA<br>CTCTTGATTCGAAGTCACTTTTGTGAGAAGGCGAGCAGATTCTACCAATGGAAACTTCTAGGGAAAAAAAATGTCTGTTTTCTTTAATAAAAA[A/T]GGACAACGACACTG<br>ATGCACTTCAGATTAAAGCCTTGAATCTGTCTGTCAACCTATCTTTCATTCTGTCTGTCTGTCAGTCTTTTTGTCTGTCTCTCTGCTTCTGTTTCTAACCATCTGTCTGACTCTC<br>TGAAAGTCTATCTGTTGGTTTGTTTACCTATCAACCTTATGTATTCCTATCTAGTTGTCTGCCTCTATCTCTCTTTTCTTCTCTACAATCGATCCTTCT
LG11	ref-68928	59.76			AATTATTTTTTTTTAGTTGGAAATTAAATGTGCAAAAAGTATGATAATTGATTGATAATTGTTTTCTGAAAGTGGACATTTCTGCTCTTGTCCTAAATCATCTGTGTCATCCA<br>TCCCTCAGCTGGTGGTTCAAGCACTTAATCCTTTCGAACAGTCTTTTCTCTCTGCCAGTGCCTGTGGCATCAGGATCGTCTTTGCTCAATCAACCTTTTCCCTTACCTAACAG<br>CAGCTGATTCTTCCAGGCTACCACAATAACACAGAGATGCCAGCCAGTCATGCCTCCTGACCTAAAGCCTGTTTTCCCCAACGCAGGGTTGCTATATCCTGTCATTCTCCTG<br>AACAAGCAGCCAACTGCTAACATCTGCTCTA

LG11	ref-14699	59.915	Chr13	16331554	CCATTGTGTTCACGGCTCATCGGATATGTCTGTGATTGGCTTCAATGATCAACACTTAAAAACATTATGAATAGTTATCAATGACGCTCTTTACCAAGTGCTTAAGCAGGCAGACCAGAATTCAAATTTCAAACAGTATGTTGATCATTGTAGCCAATCACAGACATATCTGATGAGCGCGTCAACAAAATGGCCAGTCAGAGGTGTTCACGAATCCAGTGCTCAAAGCATCACATTTTTAAAATTTTAGTACCGATTGGTACGGAAGTCGGTACTTTTGACAACTCTAGTTGGAATAAATCTCAATTTCAGGTAGAATATTTACAGGTGCTGGTCATATAATTAGAATATCATCAAAAAGTTGATTTATTTCACTAATTCCATTCAAAAAGTGAAACTTGTATATTATATTCATTCATTACACACAGACT
LG11	ref-61509	59.946			TCTTGGGCGATTGCGGATTGAGAAAATGCGCAAGGCAGATAGAGAAACCTAATTTATTTGATATATTTGTTGGGCAAAATAACGACATCTTTTTGCGTTTGATGACCTGATCAAGCGCTAAATCTGTAAGTTTACGACGCCCGCCTCCTCGTCTGTTCATGGCAACATCAACTTCGACCGCAACTGCATCGTCCGTCAATATCTGTTCATTGCTTTGTAAAACTCTCTCAATTTGATCGGTAAATAGATTAATGTCGTAATTATTCCGATTATCCCGTAAGGGTCATATTGATTACGCTAGCATCACCGCCAATGTCTCTAGCAAAGGACACTATTTCGCTAAAAGCATCGTGTAAAAAAATGTGGTAC
LG11	ref-53266	60.334			TACAGACAGAAAACCAGATGAGCCCAGAAGACCAAACTTAGTAAACACTATACTAATAAAGCATAATATACAGAAAAATTGGTTTGTGCCAAATTTGATCTTTTAATATCACTTATAGGCTACATTAAGCGATGTTAAACGTTTTCTTTATAACAGTTTTCTGAGGAAAACGCTCGTGAATTTAAACACGTTGCGTTCACATTATTGTTACGATCGTGTTGCTGTAGAGACGAGGCGAATACGGAAATCCACAAACATATGGGCTTTATTAAAAAGGGCAGAATGCACAGCTTAACACATATAGATCAACATTAAGAACAGACGAGGAGTGCAGGGAAAGTGTCCATTATAAAGGGAGTGAAGATGATGAGGTCCAGGTGCAGGTGATCAGTGATGATGGGGAGAAGACGAGGAAGTGAGTGCAGGTGTGG
LG11	ref-60163	60.369			AGTCTACCAGTGGCAGCATTTCACAAACCTCTATTTGTGCTTGTCGTGATAACAGAAAGCAGAAAGAACTTCAAATATAATTTTTGGTCATACAGATAAGAGCAATACATCATTGGATACTGTAAAGGTTCTAAGGATACTACTTCTCCAAAGGATTATTTGTGTACATTCACAATACCAACAAAAAGCGCTTTTGTAAAATAAAGAAAACAAATAGGGTGCACATTCTGCCAACTGTTTCCATTTCTCTGGAACACGTCACAAAACTGCTTAAGTTTCCCTTATTTGCTTGACACTAACACTCATGCAACTTAGACACAGATGATAATGTTTTTTGTTTTACATATTCTTTTTTGTTAATCAATCAACGTTGATGCAACCATGCAGATTTCACTGTTCTTTCATTTGTCACGGCTTTAAGCCAGTCATGA
LG11	ref-19860	60.869			GTTTGTTCTGCGACCTGGGTGCACTGCAGTT
LG11	ref-67397	60.943			CATAAATTGTCTTTCTAAATGTTTCGTGTTTAGCTTGTTGCTAATGTACTGTTAAATGTGGTTTAAGTTACCATTGTTTCTTACTGTATTCACAGAGATCAGAGACATGTCATTATTTTCATTTTTAACCTGAAAACGCTTGCAGTCTGTATAATTCATGAATGCATCTTCATTCTTAAGGGAGTCTCTCTAACAGTGTAGCTTTAGCCCCGATAGCCATGCAGCACACTATTAAACTCATTCAGAATCGAATTTTAGCAAACTTCCACAAAATACATGCCATACTTACGTGATCTGATATGCTGCATCACCAACAGTTTGTAAAGATCCATTTTGGGAGTTATATTAGCTGTGTGAACTTCTGTATTATTTATGCAATGTTTAATAGAGTCGTACCCGATAGTGTTAACAAAAGCAAATGCGACATAAAA
LG11	ref-31151	61.03			CTAGCCTGCTCGACATTATTGCTACTGGCAT
LG11	ref-35781	61.647			AGCGTTAATACGATTACTTTGCGTCTATGTC
LG11	ref-7912	62.287			GTTGTCAGCGCGAAACCTATGCGCGAAACGC
LG11	ref-52917	62.367	Chr4	70063281	AGACCATAGAACCGCTTGCGGGAAAGTTGTGAAATTTGGCACACAGTTAGAGGACAGTCTGACCTTTGTCCATAGCAAATTTGGAGTCTCTAACTCAATCCCTCTAGTGCCACCAGCTGTCCAAAGTTGCACTTATGTTTATGTTAATAACTTTTGAACCGTAAGGGTTAGAAACAAAATTCTTTTTTCCTCTGATTCCTTGGGTCAAGACGAATCGATTGCACAACTGTGTCATGAAAATTTTTCCGCCATTTTGAATTTCTGTAAAACCTACTTTTTTGAACTCCTCCTAGGCTGTTACTCCGATTTTCACGAAAATTGAACCAGATCATCTTCGGAATTCAAGTCGATTTCTCAAACCGTTTTCGAAAAAACACACAAATGAATTTTACGAAGCGCTTGCAAAAATAAAAAATAATAAGGCTGTATC
LG11	ref-25359	62.428			CACTCTGTTGCGACTGGTTTGCAACCTTGAC
LG11	ref-69658	62.553			GGAACACAATCTATAAGAGTCAAAAAAACATGCACAAATCCAAATTAAGCTCTGTGGCTCGTGACGACACATTGATGTCCTAAGACACGAAACGATCGGTTTGAGCGAGAAACCAAACAGTATTTATATCATTTTTTACCTCTAATACACCATGTTCAACTGCCTTGAGTGCACGCACGGCACCTGGTTCGTGAGGTGTGTTCAGTGCACAACTCGGCAAAAAACAAGAGTGCTACAGCTGTTCCGGCCTGGAGAACATGGCGGACCGTGTACAGCTTGCTCAGGGTGGGGTCTATGCTAGTCCATCAGCAGTTTTATTCAAATAGCTACCTACCTATAAACCGGCTGCAAATTGAAAAAGCTCGCTGG
LG11	ref-60753	62.809			TACCTGCCAGCGAGGTGTGTGCTTCTGCTAT
LG11	ref-46981_31	63.485			GTTCGAGTGACGAATCCAGTGCATTTGTCT[C/T]T
LG11	ref-46981_16	63.576			TTCTCACACTTGGAAAAATGTGCTTCATTTGAATTAATGGGTGGCTCTTAAAAGAGCCATTGTTGCTGTCCTTAAAAGGATATTTGATGGTTTCAGCTTCAGAGTTCTACCTGCAGAGCTTCTCTGAGGTTGCTCAGCAAAGGCTTATTTCTCCCTGGGCTCAAGTGGACTCCGTCTCTGATCAGATGAGACAAGTCGATGTTCGAGTGACGAAT[C/G]CAGTGCATTTGTCTCTCTGCCAAGAAGCCTGACATCGCACTTTTGAGCCACTGCCGGCTCCATTCGATGCCGTATGCAGTTCTAGTAGTCTGCCTTCACCCAGTTCAGACTGGGCAGAATGAATGAAAAGACAAGCCTGGTTCTGGGTAACATCCTGAAGATTTCAGCCAGGTCTGTCTTCATTTCCTGAAGGAAATCCAGGCGGTTGCGGCCCTC
LG11	ref-65363	65.017			CATCAAATGACGAAGGAGATGCCCACTGCGT

LG11	ref-16487	65.234			GTGGCGGGCCCGATTATGCTGCAGGCCTTCT
LG11	ref-34968_24	65.758			GAACTAGAGCCGATTGAGATGCA[A/C]CCAGAGAT
LG11	ref-1889	66.21			ATAACTGATTTCCTGTTGTGGTCTTGGAAACAATTGGGGCCAACTGAAATGTGTATGTTTTGAAAGAGCTAGAAGTTTAATAGGTTAAAGGTTACTCTTTTTTGCTATGCAA<br>TGCGTTGCACCTGCGAAATACCTACTGGTATTTCCATTTACCTTGCTGATTCTTTAAGATTATGTCTGCACAATGTTCGTCCTGCCAGGTGCAGATGGGATTGGATGATAGG<br>CATGACCGGTGTCCGAGCTGCCTAGGCATAGAGCATCTTAAGGAAGCTCTGGAAGGTCCATGCATTAATTGGAGTATGTTGCCCCTAGCAATACGACGCGCCCGCCTCGCA<br>GGGATAAGTATGAATCAGGAGGGAGGTATGAAAA
LG11	ref-18091	66.649			GTTTTGCTTCCGACACCCATGCATTTGCTAA
LG11	ref-21918	67.273			TATTAAAATGCGACTTGAGTGCCCATCTCAT
LG11	ref-48639	67.331			AAATAGAAGACGACGCTGCTGCAATCGAGAG
LG11	ref-66020	67.725			GGGGGATGGACGAACTCACTGCCAGCTGCCT
LG11	ref-12180	68.185			TCTGTCATCACGAGAGGAGTGCTTTCGAGGC
LG11	ref-17979	68.238			GTGGAGTTAGCGAAACTGATGCCTGCTGTCC
LG11	ref-2955	68.35			TGAATTGCAGCGATCTCTTTGCTTCCGCTAC
LG11	ref-33950	68.644			CTTCAGTTCCAAGTCCACCAGCATCATTTCTCTGTGTATGTTCACCTATCCTGTTTGCATCCCGCTCTTATTCCCTTGTCTCTGCAAATAAAGACTTGTTAATTGGTACTCACC<br>TTGTCTGTCTCTTTCTGTGTATGACAAACGGAGCCTGTTAACTCAAAAAAGGCACAGTGCTTTCCGCAATTGGTTCGGATCGAGGTCAGGTCATGTTCACATCTCAAACAAA<br>CCGTACCAGAGTTCGTTTGGAACCAGACCGAGACAACCTCTTCAGCTGAGTCTCGGTGCGGTTGTTTGGTCCGCACCCCAGTTGCTGTATTCACACCTGCTCAAAAGATCCG<br>CACCAAGGGGGGAAACGAACTTCAGTTTGAT
LG11	ref-2537	68.799			AGTAAGCAGACGACGTAATTGCATGAGAATA
LG11	ref-12879	69.031			GATATTGAATCGAATTGCATGCTTGTGAATC
LG11	ref-27094	69.223			TTCTGCCAGACGACCGTACTGCGCAAGTCGA
LG11	ref-25658	69.386			GATTTCAGAGCGAGTGGTGTGCTAGAGAAAA
LG11	ref-68296	70.07			TGTCCTTCGGCGAGCTCTATGCACCACTCCA
LG11	ref-56911	70.518			ACACCGAACCTAAAGATTTTATGAAGTATTGCCTATTTAGAGACGGAACTTACCAAACATCCAAAACAATGCTGTGTGCTGAGGTAAATGCGCTTGTTGTGTGTGCGTAGT<br>CTGCATATGATGGTATTTTGGCCAGCTGGGAAATCGGGAAGAGCTTATAAAATGTGTAAAGGAGTCAAAACAGCGGCGGGAATCCATCCATCACACACACGAATGATGTG<br>CTGCGTGGAGACGTGTCTGACGTCTGTGATGAGCAAACTCGCGACTATGACGAGAAAAAACTGATATGCGTGAGGATTCTGTCCTTTTTAGGGTCTCATCTTCCTGTTTTTG<br>GTTCGTTTACATGTCTTTGGTCCGTGTTGCGTTCATATATCATTCGAACCGCACCAGAGTTCGTTTGGAAGCGGACTGAGACCCATCTTCTCAGCGGT
LG11	ref-41788	71.739			CGCCGTTAGCATGGCGCGCTAGCTGCTTTCTAACTGTAAAACGAGCTGCGAAAAGCTTACAAAACTACTATAAACAACCCAAGCGGACCCACAAACAATTAGAGAAGCGA<br>TTAAACAGCCATTTAACTTACCATTCAAATAATAGTTGCTTTCATCAGGTCCACAGATGTTTTCTCACGTGCAGGGTTTCTGTCCAATCCAAACAGGAAACGAATGTGATGC<br>TTGACTAGCATGAAGCTTTGATTACCATACGGATAAACGTATGCGTGAACATATAAATAGGATTTAAAATAGATAAATATTACAAATCTGTAATATAAGCTCGTGACTTAA<br>CATGTAGGCATGTTTACATTAATCACAAATATGCTAACGAGCTGTCTGTATAAATCATGAGCAAACAGCTTTCAAGTTATTTTCATACTAAATTATAT
LG11	ref-38783	71.814	Chr3	52435274	GGCATTTTGTCATAGAACCTGGAAGCGCTGCAAAGAAAATAGCATAGGAGAATGACAGGGGAGAGACAAATTTGTTGAATAAAGTCATGTTTTGTTTTGGCACACAGAAA<br>GTATTTTCGTCGCTTCATAACATTAAGGTTGAACCACTTGTAGTCACATGGACTATTTTAACAATGTTTTTAATAACTTTTTGGACCTCAAAAGGTGCAACAATGTAGCTGC<br>CTGTGTGTGATTCAGAAACCCTCGGATTTCATCAAAAATATCTGAAATTGTGTTCCTTACAGGTTTGGGGCAACATGAGGGTGAGTACTTAATGACAGACATTTTATTTTTG<br>GGTGAACTAACCCTTTAAGGATATTACATTGAATACTTACAAATTAGATACAACTGTTACAAATTAGATACAACTGTTACAAAATCTAGATACATGT
LG11	ref-23282_15	72.108			AACCCATTCATTCAGCTTCTGCATTCAGCAAGCGCTACATCTGAAATTGTCTGTTCTATTTATTGTTGTCTGTCACTCATATATAAGAGAATACTATGGCGAGCGAACAACA<br>ATTTAGGAAGTGTGTTCATCCCTGCTCTCATTTCATTACGGGTGGGGATACACACCAAATGTGTGTTGTTTGTCTGGGAGTGCAGCACGCCCAGCTCTCGAG[G/T]GGGCTG<br>CCTGCGAGCACTGTGAGCTTCTCCCACTTGCGTTCCTCATGGCTCGGGTCCCACTTCTGCCAAGGCAGAGCGGCGTCTCAGGTCGTGGGGTTTGCAAATGGATGTGGCAGA<br>GGGGTTAGAGACGGGCGCTGCCCTATCTCAGCCTTCACCTGCCGCATCCAATGTGTAAATTCGGAGTCAGGAAGCACGCGTTGCGGTTTCTTCCGCTCCG
LG11	ref-61472_4	73.232			CCT[C/T]TGTCGGCGAACGGCATGCTGGAGGTGAG

LG11	ref-61472_15	73.388	GCAGAGTACGCCCAAAATTCCATACAGAGGGCCGCTACGGGATTAACTCCCTTTCAATGCATCCTGGGGTTTCAACCTCCCCTATTCCCATGGTCAGGAGAACCCACTAATGTTCCTGCAGTCAAGCACTGGTTGCACAGAAGTGAAGCAACCTGGAACAGGGCCCACACTCACCTCCAGCAT[C/T]CCGTTCGCCGACAAAGGGAACAGGCTGACCGCCATCGTCGTCCTGGTCCGGTATACAGTCCTGGCCAGTGGGTGTGGCTCTCTACCAGAGACTTACGTCTCAGACTTCCCTGCAAAAAGCTCAGTCCAAGGTACGTGGGTCCATTTAAAATAATTCGTCAAATTACACCCGTTTCATTCCGCCTTT
LG11	ref-15177	74.423	GATAACAGGCCGATAACATTGCCATTGTCAC
LG11	ref-24159	75.684	TAAACGAGTTCGACACATGTGCAGTACCCCC
LG11	ref-25242	77.541	AATAAACTGTGTAAATCACGCGAGCAATACTGATATTGTAGATGCCTTTTCACACTATATTAACGTTTTCTCACAGAGGGTTGTGAAAATCGCTCCGTGCAGCTTTCTCTGTCTCTCTCATCTGCTCGAATGCACGAGCCGTGTCATGTGCTCTCAGAGCGGCAGCCCCACGGGCGCTGCATGAGACTCGCGAGAGTGTCTGTACTCATTAAACTAAGCATCTTAGAAGCTCTAAAAGTATGTCATGTTTTCATTGCTTGTATATTAATACAGTCGCTAGAATTGTTTCAAACTGGCAAGCTTTAAAACTCAGAAATGACGAACTGACGAAACCGAAAGTGAACGCATGAGCCAATGTTTTCTTATGT
LG11	ref-14459	77.77	AATTCCTCAGCGATGATGCTGCCGCCGGGGG
LG11	ref-17977	78.203	TCTTGTTGTTCGAGCCCTGTGCATGCCAATG
LG11	ref-20697	79.797	AATAATGACTCGATTGCATTGCACACACCTG
LG11	ref-39034	80.181	AACCGTTTCGCGAGGGGTATGCCAGCCCTGA
LG11	ref-52736_15	80.368	TGGGGGTGGTAACTTTGCAGGAATTAAATTGATTTAGCCTGATTGGCAGCATATTCAATCTGGCTTGCTATCGTAACAGCTCTGTCCAGTGTGAGTCAGTTTCTAGTAAAAGTCTCTCATGTACTTGTGGGCTAAGCACATTCTATACTGACTGATCGTGAATGATTTCGTCATTACTGGCAC[C/T]AAACTCGCATAAAACAACAAGCTCTCAGAAAGCAGCATACTGTACAACTATCTTGTGAGGCGCGTGACAGCATTTATAGAAGGCGTGTCGTTCAGCTACTATGTTAGCTTTGGCACCAAGTAGGCTTTCAATTTGTTAGGCAATGAGTAGAAAATCTGTTGTCCCTGTGTGCCAAGGCAGTGTAG
LG11	ref-52736_4	80.368	TTG[C/T]TTTATGCGAGTTTGGTGCCAGTAATGAC
LG11	ref-53658	80.508	CAGAGAAACACGACAGGGATGCCCACTTTTG
LG11	ref-39893	80.792	AAACAGTTCATGTGAGTACAGTGGTTTAACCTTAATATTATAAAGTGACAAGAATACTTTTTGTTCACCGAAAAAAAAAAAAAAAGAAGCAACTTTATTCAACAATATCTAGTGTTGGGCGATCTGAAAACACTGCTTCATGAAGCTTTATGAATCTTTTGTTTCGAATCTGGTTCGGAGCGCCAAAGTCAAGTGATTTCGTTTTTTAAACGAGGCTGTTACATCATAAGTGTTACGAAATTTCAATAGTTCACGTGACTTTGGCAGTTTGATACATGCTCTGAGTTGAAACAAAAGATTTGTAAAGCTTCGAAGCTTCATGAAGCAGTGTTTTGAGTTCGCCCTTCACTAGATATTGTTGGCACAATATGTAATTTTTTGCGCCACTAGAGGTCACTTATTCAAAAAGGCGTAGCTTGATGACACCTTGA
LG11	ref-72763	81.372	AGAGTTTTCCCGACGGTACTGCTGATATGCT
LG11	ref-40027	81.659	CTTGAGTGGCCACTAGCTGGCGCCACATGTCTGTGAGTTGTAGGCCCTACAAACTATGTGTTATGTCCCCTTTAAAATGAAGATTGATCAGGAAGTCAATATTTTCAACCCAGATCTATTATGCTACTTGCACATCTAACTGTACTGACCTCCGTTCAAGGTCCCGCTGGGCTTTCAAGGCACGGTTGCCATCCCTGTCCCTTGCGTTGATGAACCATTTGCCAAGGGCTGTCTCCACTTCTTTTCTGCTCACATTTTTTAGCAGAGGGTTCAGCTTCAGGCTAACTGTGAAACAAAATAAACAGTTTTAAGTAGAGGATGCTCATCAGAGCATGAGCCTTAATCTATAAATGCAAGTTACATTAGCATAGGACAATGGCTCTCAAACCTGTCCTAGAGTACCCACAGCACTGCATGTTTTGTATGTCTCC
LG11	ref-22968	82.085	ATTAGAACTAGATAAATGGATATGAGAACATATTATACAACTACATTAAACAATAATCTCTTAGATATTTTATAATTTCAACTTTTTCATTTCATTTCATAAACAGTTTTATTAAACATGGAACTTTATTTTTATTAAAAAGGCTTATTTATGCTATTGTAGGCTAATTTTGTAAATGTTAATTAATTTAGACCCATGTAAACTGGGTGCCGATACAGATGCATCTTCTGATGAGAAACTGCGTGTGAAGTTACACGAAATTTTGTTCGCTAAATCGGTTCATGCGCACTAAATTCGCACTTCCGCCAAAACCGTAATGATGCCATTTTTGTCACGTGCACCCAAGCGAACTTGCAGATGCGTCGAGTATAAATCAGAATTCATACTTTGCGCAGTCGCCGTTGTACTATTCTTTATAACGACAGGAGGCG
LG11	ref-36629	82.652	TTTATCTGCCCGAAGTCACTGCACATTAGCC
LG11	ref-40190	83.561	GTGCTGCCTCCGACAGAGATGCAGCGTCGCA
LG11	ref-35645	85.003	CCCCTAAATACGAATAGGTTGCCGAATTTAT
LG12	ref-38316	0	CTCGATCGATCGATCTATCTGCCTATCTATC
LG12	ref-20287_18	0.156	ACATTTGCAGCGATTAA[C/T]GTGCCAGGTTGGGG
LG12	ref-53136	4.468	CACCTATTGCCGACCACTGTGCATTAGCTCT

LG12	ref-19508	6.516			TGGGCGGCGTCGATATGTTTGCTAACAGTAT
LG12	ref-41672	12.229			CTAAGATCCTCGAACAATCTGCCGACCATGA
LG12	ref-50236_25	15.041			ACACAACCTGTGTTCATACAGATGTACATTATGACTGTAGATGTGTGTCTAATGTAGTTCTGATTGAGAAACTGTTATCTCTGCAGGGTAAACCTGGCCCTGCTGGACTTCCTGGAAAGCCTGTGAGTATCTCTGGATGTGTTGTGTGTCGGATCTTCTCATGAAGCACTGATTTACTTTGTTCATGCATTTAACTTTCACAATAGATGCCGAAATGGCTGCTT[C/G]CTCGTATCTCTGATGATTTGTCTGGCAATGCTTCTCTGTTGACTTTGTATCAGTAATGATTCGAGTGTCATGTTTTGAGCCCGTCCAGTGAGGACAGCAGACAAAAAATACATTACATTATTCCCTCACGAAGACGTGAAGCTTTTTTGTGAAGATGCAATAAATAACATGTAAATGGATACACAGATCATATTGTCCAAGAGGCTG
LG12	ref-58618	16.531	Chr23	37577122	TATGATATGATGTATTGATGCAATGTGTAGAATACCTTTATTTTGACACTCTCCACATCTTCACACAATGTCCATATATGCATTACCAGCCTCAACGAGTGCTTTCTCATTTAAACTCACCTGGGCTCGACAATAAGGAGTGCCTGCTGGCCCGCGAACAGCGTGTAAGACTGTCCTGATCAGGGCAATGCTGAATAATTACGAAACTTACAAATCAGTTGCACATGTTTTTAGCCTTGCAAATTTAAACATTCAAGCGCAAACAGACGAAAAGAGAAATTATTTTGGTTCTCAAGCGCTGTGTGAGAAGTTTTCTGTGCGCACACATCAGTAGCGTGCACACAGAAAGCAAGTTCTCTTTCACTACTTTTGTGCTTGAGCGGACAAATACAGACAAAATGATGTCTTGTCGAATATCCTAAACATAGTTG
LG12	ref-73123	17.168			GAATAAAACATATAATATATATATATCTCCCTTCGCCAAGTACATTTCAAACGGCACCATAATGGCACGCAAGTTCCCTGCTCCCTCCAAAGTCCCCACTTCAAGGGCTATGCCCTTCGAAGGGAGTAGGGCATGCTCCCTTCCGTTTGGAATTTGCCCTTTGTCTGAATGTTCGCTGCGCCTTGAGCTCGCTACTTCCTGTTACCCATCGTGAGTCGCTCATCAAAATAAAAGTCCTTACATTGGAACTTAAATTATAGCGGCAGGTAAAACGAAACTTATACATTATATCAAATGATTACCCAGATGTAATATTCATAAATGCAAATGCCCATCATAAATTATACACAGCACCTATCAGGGGCAGAA
LG12	ref-9446	17.325			CCGGCCTTTCCGAGACTTGTGCCAATTAAAA
LG12	ref-2807	18.839			GCATTCCCTGCGATCATGCTGCTCTAAGAGG
LG12	ref-35832_32	19.293	Chr23	10107767	TAGCCTAGCCAATGTGAAGGAAGTCCTCTTCTCTGCCGAGAGACCTGTAGATACGCAGTATGTGGCGGCTAACGACCCTACGGCATCAACGGTTGGGCTTGTGGTGCGACCGCAAGACAAGGATCCAGTCCTGTATGTGGGCCGAGGCTACACCAGCAGTCACCCGCCGGTCTCCACTCGGCAACTTTTGACAGAGCCAGTGTTTTCCTACGAGGAAACTGCCAAACTTGC[A/C]GTTGCCGGACGCCTCTCGGAGTACGATCACAATTTTGTCGCAACCTTTGCGCGACGCAACCACGTCTACTTTTTATTCTATAGGCGCGACCTGAAGGCCTCACGGGAATATCGTACGTATGTGGCACGGGTTTGTCTGGATGATCAGGCATATTACTCTTACGTTGAGGTACCGCTAATGTGTCACTCAAAAGGAAGGAA
LG12	ref-2583	20.421			GCTTGGTACGTTTTTCATCTTCAAAACAAACTTGGGTCTTCAGATGACAAAGTTTAAATGCCGTCTGACTAGAGCGCTTCCCATAATGTTTAGCGTTAGGTCAGTAGATGGAGAGTAAACGTTCTTTAGAACACATAGTTTGAACACATTCGATCTCATGAGCGTCTACACTACGAAAAGCAATCCGGTCGAATGTGTTTTCAACTACCTCTGGAAGAGGTCGAAAGTGGACAAGCTCAATGCGTTTTAGACCCCGTTTACACCTGTATTTAGTGTTGTCCACTTTTGGTTGTGATCCGATCAACCAAAACACATCTTAATACCAGGTGGAAACAGGGCCTTTGAAAGGTTACGTGTTCGACTGTACGC
LG12	ref-40189	21.006	Chr23	10148831	TGTTCAGTTTTGTAATGATTATCAAATGGCCCACTCTTTGATTATTTCTTTTAATATAAATTATAAAAATAATGTAGTAACCCAGAAGTCAGAGTCACTGATACACCGTCATCCTCTTATGTGTGTGATGTGAAACGGGTCTAAGAGTGAGGTTAAAGGGTAACTAGCGCGCGTGGGCTGATAAATTTTCGATTTTCTTGAATGACAGCCTGATGCAGATGCCCAAACGTGCGTCCTTTGTCCTGGATTTGAGGGCAGATGCGAGGGTCCTGGTGGACAGTGCTGTGGAGAGCTCTCGCTGAGAGAGGAGGCATCTGCTGTGCTTTTCAGAGACCACCTCTTGTTCTTCAGCTCCAGTCTCCATCTCTTTTCAGCCTAGGCGGAACTCGTCACCCCTTCACATCCTCGCTTTCTGCCCCTTTCTCACTGTG
LG12	ref-72246_14	22.364			ATCTTGAAAATCCTGTTGTTAGGATATTGCATAGACTTTTAATTTTATTAAAGAGGTCATATTATGCCCTTTTACAAAGTCTTGATTTTGTTTTTGGGGTCTACTAGAATAGTTTTCATGCTTGAATGTTCCAAAAAAAAAACAAAAAAAAACACATTTTATTAAATATTAAAATCGCCTGCTTCATCACAGATTGCGACATGCCTTGTTTCGA[C/T]TGATGTGCGTTTGAATAAGAAATGCTTGCAACGATTAATAACTTAAATTCTGCTCTGTACCTACAAAATTATATACCACCAGAGAGGACTGTGACTGCAACACATCATCATTTGGACTACTTTTGATACCACTTTTGACATTTTTGCACTAAATTACTGTGATTACACTTATCTGTCTGTTATGTTTTCTTTATAACTACATAGGCTATTTTTAAGA
LG12	ref-72246_4	22.802			TGC[C/T]TTGTTTCGATTGATGTGCGTTTGAATAA
LG12	ref-42882	22.832	Chr23	23616248	ACCACAACTATGAAAAGGTGTAAGGACTCCCAGGGGGGTCATTCGCATCACTTCCAGCCCCCACCCAACATTTGCCCGGCGTTTCCATTTCAAGCCCTTTTTACGCTCGTCTCCTCCCAGGGCCGACCAGGAGAGATACAGTTGCATTCACCCATTGTGTGGCAGTGTGGTGTGTGCAGGCATGCTATTCTCTCTCTACTGCGAGTGACCGCTGTGATCTAATTACTGGCAGGAAGATGAAGTCGTCCCCCTTTTCCAGCCCTGATGGCTGAATTTGCTCTCAACAGCGAGGCGCAAAGGCCCTTGCCTTATCCTTTCAAAGTTAACCAACAACAGAGACGTGATCCAGATTACATCAGGAAGGTATAA
LG12	ref-58574	23.341			CAAGGAGATGCGATAGTGATGCCTGTACCGA
LG12	ref-58009	23.482			ATTGGCAGCGCGATTGTAATGCTTTTTCCC

LG12	ref-36248_32	23.731			TCTCTTCACCCATCTCTCCTCATAATCCTTGTCTATATTCCATTCGTATGTGTAGTAACACATTCAGCATCCTCTCAGGTTTGTGAATGTGTGATGATGATGTAATTCTCGCCT<br>GAGGTGCCAATCTATTTGGCCAGCTGATGTACCTGGCGAGGCTAAATTTAAACCTTTGAGTGCTGGCATGATCCTCGCTAGTGCAC[A/C]TTATGACAAATTACTGTGTAAT<br>TCACCACACTCTAAAAATACTCCAAATGAGACTCACTTAAAGGGTGCAGACCAAACCTCAGATAAATGAGTCTCTTTAATACTTTAGGGAGCTGAAAGACCTGAAACTCA<br>GAGTCAACGGTTAAGGGGAAGTACTACAATCCTACGC
LG12	ref-31359_23	24.393			TCATTGGTGAATGTGGTTTGTAATCCTCACTTTTCTGTAAATTCATTTCAGTCAGTCTGTGTTCAGTTTGATTTTCTACCCAAGTCGCTGTCTTACGCCACATAAAACAAAAA<br>CATGTCAGACGTTGTAGGAAATGCACAACCAATACAGATATTCCAGAAAATAAAAGAGTGCCATGAGCAAACAAATCG[C/T]ATTTAAAGAGTTGCAATACACAGTGAAT<br>CATTTAGATCAGATACACAAATAATCAGATTTAGACTGACAGTGTGAACGTAGCCTAGAAGGATGCACTTCACCGATGATGGGGTACGTCAGTATTTGTAAAATCTTGACC<br>ATGTTGATGTTAGCAGCTAGCTAAATTAATTACCCTAAA
LG12	ref-25253	24.937	Chr23	14290930	GCTAACCGGCAAAACACTCCTTTAATTAAAGGATGTTAATGTGTTCTAAGCAAGCGTTCCTTCCTGCATTAATGAGGCCAAGCATTCTGCTTTGTCTTCCTGATTGTTTATA<br>ATGAAATGACCTCCTGTTCCCATTCTCTCCCACTACTTTGTACAGTAGAAGTAAATAGTTTCACAGTGCGCTATGGTCGTACAGTGACCCTAAGGTCAAGACCTCACTCTCC<br>CTCCTTTTATACACACCCACGGACCTCAAAAACTCCTCACACACACACATAAATACACACACACACCCTGGAGAAAAGAACAAAGCGTCCCTTGTGTAGGAGCACAGAGG<br>CTGGGTGACAGCGCTTAACATGATGCTGATATTTT
LG12	ref-32031_15	25.541			ATGAACAGGAACTCACCAACAGCAGGTCACACAAACAAAGCTGTAGACAAGGCACAATGGAAACATGAAGGCTATATACACACACAAGGACTAATTACATAACCTGAAA<br>GCAATCAAACAATGAACCAATCAGAACAAAACGGATGAAGCAAAGTAAGCACACGACGATCACATAAGGGGCAAG[C/T]GGATCGCATGGTGTGACAGGAAACAAGACT<br>ATGAGAAACTTCAAAATAAAAGATGTGAAAACAAGAATGTAAAACGAGCAGCTCCAAATGCCCAAACAGAACACCCACCCAAAACCCAAAACACACAAAATGTCCAGGA<br>GGGTAGTGGAGCAGAGGAGATCCAGAGGGAGAGATGGAGGACCAGG
LG12	ref-56517	25.981			GATCATGGTGAGCCGCAATGGCAGGGTGGAAGGAGAAACGCATACTCGCGCAGGTCCCGCAGCCAGCTGTGTGCCAGAGTGTCCGTGCCAAGAGTGCCCTCGGTCAGGG<br>AGTAAAACAACTGGCAATGGGACACTGGAGAGGCAAACAGGTCCCCGAAGTGTTCCCAAACCAGCTGAACCGACTGGGGATGGAATCTCCACTCCCTGGGGCGAGACTG<br>CTGCCGAGAAAACTCGTCGGCTGCAAGATTGAGCTTGCCTGGGATGTGAATGGCACGAAGATGCTTCTGACTCCATAAAAGGAGATGGCGGGCGAGTTGCGACATGCGAC<br>GGGAGTGTAGACCACCTTGACGGTTGATGTAACAAATCCTTGTCAACCAAAACTTTTTTATTTCTTTGTCTATGTATGTATTGAGACAATCAACTGTTTTTGA
LG12	ref-21095_18	26.11			CAGACATATCCGATGAG[C/T]GTGCGAACACAATG
LG12	ref-15806_16	26.47			GATTCGTTGTCATGGATCAAGGTTGAAACGCTGATACGTTGATAATGCAATATCGTCTTATTTTCGATCTTGTTAAAATTTCAGTGTTGAAAGTATTTTGTTAACCTTTTTAC<br>ATTAATATTACACCGTGCATTGTCTGTCCACACCAGATGCGACAAAATCAGTCAAAAACCACAAGAGCACT[A/G]CACTCGCAACAAAATGGATAAGTAGGATACATAATC<br>AAAATAATCAATATTACACCATTTTGACATTATTAAAAATACATACTGAGTGACTGAAACAATCTTTGTCATAGAATATGCTTCACAATGACTTGACAGTTCTTGTTTCCTT<br>TCATTTATTTTCAAGATTTATTTTCATTTTTGTGTAT
LG12	ref-42381_2	26.581			GGATTTTTGACATTACTTGCTGATGAAGGAAATGGAGTTGTACATCTGAGAAATGGGATTTGTCATTCATTCTGTGTGGCTCAGGGCATTTGCTGGTGCTACGGTCCATGAC<br>TTCTTTAACTGGCTGTCTGTTGTGGTGCTGTTGCCTCTGGAGGTGGCCTCAGGATTCCTCTACAGGCTCACCAAACTTGTGATCGATTC[C/T]TTTAACATCGAGACCGGTGC<br>AGACGCTCCAGAACTGTTAAAGGTCATCACTGAACCCCTCACCAAGAACATCATTGAGGTGGGGTCACCATCAGTGCTTATGAAGTGGACCTGTTTATTTATCTAGTAGGG<br>GTTATTACTCAACTACATCTGTATCGGGCAGCTGGGTTGTTGCCACTCATTTGCATAAACTCTTGTCACATCCAGTATTGTTATTGTCCCATAAATTAA
LG12	ref-18496_29	26.745	Chr24	33565479	TTTTGTCAACTTGTTTCCTTTCCATTAGGACAGGTTTTGTATTTTTTTTGTTTTTGTTTTTTGGAGCTGACTGATCGTAGGCCTCATTGATTGCTTATGCTTTTTTAGGCGAATA<br>AGCTTAAGTGAACTTTTTTCACTCAAACACACTGGCTATGTTTCCATCCACCTATTTTCATGCTAATTTTGGGGTATCACATAAAAAAAAAATGCTCGATGGAAATGCCCAGA<br>T[A/G]CGCATAAATTCTAAAAATGCTCATAAAAAAGGTATGAGCTCAACTGAGGTGGATACATTTTTGTATCTGATAAGAAGACGTGCCTAAACTACTACGGAAACACATT<br>TACTGAATAAATCCCTCAATGTGCAACAAAAAAAAAACTTGTATGACTTTGCCTCAACAGATGATGTGATTGGATAACTTGACTAAAAAGTGGACCA
LG12	ref-48124	26.85			ATACCAATGCCGATGCCAGTGCCTATACATT
LG12	ref-68635_25	26.979	Chr23	4764200	CAATGGTGCACTCTCTCGCCAGATGACCGGTCTTGCCGCAGTTGTAGCAGTTCACCTCAGTGGCCTTGCTGCACTGCACCGCCACGTGACCAATCTCACCACACCTGTGAAT<br>CAACACGCATTTCAGTTTTCTGACAAATTCAAGCCCTTCATCCTGACAAATTCAAGCCCTTCATCCGGCACTCTGTTCGGC[A/G]GTAGTAGTAGTTCAAAATGTAAACATG<br>TTATGGACTATCGCTCATGTCATCCCAAACGTGCGCTGATATCTTTTCTGCTGAACGTTAAAAAGAGTCACATAGCTCTCAGCAACAAAATGAGTCTACAATTACAACATCC<br>ATAGCATACACTCACATGGTGAATACTGTTCGAATTC
LG12	ref-46676	26.998	Chr23	6186140	ACATTCTCACAGTTTGCAGGCTCCTCCATGTATGGGAGTTTCACAGGGGAAACAGCTGTCATCACATGACAGACACTCCCTGATAGACATGTTAGACGCACAAAGTGTACA<br>AACAGATGTCTACAATATGTTTCATCAGGTTGTCCCCCATCAAGTTGTTTTATTTTGTCAATCGAAGATTTTTACGTGTGACATTACATGTGTGCAGTTCGATGTAGTTGCGT<br>GTTGCAAATGAACCGAGTCTAAAGCCAAAAAAAAAAAAAAAAAAAAAAAAACTGCCATTTGAACTTCCATTTAAACCTGATGCTGCATGTCTTTCAAAGATCTGAGTGGTG

					AAACGGCAAAACCTTCCAGGTGGCATAGTGTGCATTCAACAGGATTTACAGACCCTTTAAAAACCAAGTTACTACATAACAGACATGCCTTTGATAC
LG12	ref-18362	27.051	Chr9	3291296	GGTAGGAAATACCAGTTCATATTTCTAAACATTCATTTTGCCATTAATTGTAATAATCCAGTGAGATTTTTGTATGCACAAGGAGTCTGACAACAGGCGGTGCTCCACACG<br>GAGATCTGATCTCACCATCATCGAATCTGTCTGGGATTACATTTTTACACTAGTGCCTAAAACTTCTCACAGTACTGTAGCTTTGCAGGTCGGATTTTACGAAAGCAGTGCA<br>GGAAACACTGTCACAGGGTAAAAAAAAAATCGATCTTGAGCTTTTAAGAATCAAAATTGTGACAAATGACATTCATGAATAATGGAGAAAATCCATATTTTTATCTTTAAG<br>ATCCACATCTGATGTGCAGACAGTGATGCAATTACCATGACTGACTTACCTCAAAGATCTCAAAGCCTGCTATGTCCAGCACTCCTATGAAGAACTG
LG12	ref-21235_18	27.215	Chr23	30307626	AGGTTAAAATGTCAAATGTGCCATTTAAAATGCGACTTTGCGAAACAGGTTGTTTTTAACTTAAATGTTGCTACAGTAGCCTAATATTAATGCTAGAAAGTCTATGCGCTTT<br>TTTAAAAAGGCTATTCTATTTCATCATTAAAATCCATTTTCAAATCTACAGTATCTTGACTTAGGAAGCAGAAT[C/T]GTCGGGTGAGTGGGCAATGCCCACCTCCAGTTCCA<br>GAGGAGGAAATGCGGTTTGGCCTGTACTGACCTCGCTTGACCCCTCCGCCCCAACCTCCCCATCCCATCACCCCTCCACCGCCTTCAGCCTGCGTTTGCACGAGGAAGATTA<br>AGCCACTCTGACGAAGCCGTGTTTACAAATATGCAG
LG12	ref-64302_32	27.385			GTCAACACGGCGAACCCGTTGCTAAACCTTT[C/G]
LG12	ref-64302_29	27.419			GTCAACACGGCGAACCCGTTGCTAAACC[G/T]TTC
LG12	ref-42767	27.551	Chr23	13787	TAAGAATCATATTAGTTTGGTCTAAAGAGAGACTGATCTACTTTGCGTTATAGTTAGTCTAGGGAAAGTGTAAGCACTTCCTGTGTCCACGATATTAAAAGTACAGTGGTC<br>ATATGGATGCGACGTGTGTTTGCGTGCACAGAAATATGGACGTATATGTGTGTTTTGGGTGGGTGTGTGCAATAAACTTGCTGATAGCTGGTTTGTCCCTCTGGCCTTTAAA<br>AACATGAGGCGTAACATTAAATCAGATACACAGAATCAGTACAAAGAAGTATCTTGAAGTATTCAAGTTTACAACCTTCATGCTGGGAATGCAGGTAGAATGTAACTGAA<br>CTTGACAAGAATGCAACTGTTTTTTTGCTTCACTTT
LG12	ref-10630	27.697			ACACACCAGCCGACAGACTTGCACCACCTGA
LG12	ref-69651	27.851			TAACATTTTTAGCACAAAAAGTGGATATATTGTGAATATAGATTAAAAAGAAATTCTGTTTTTTTTAAAACACATTTCATATTTTAAGAAATAGTTGTGTTCCCCAACTTTA<br>TGTCTGTGGTGGATGTCGAAAAGAGGGAATTCTATTTGGACTAGGCTACTTTCTGTGTGTAAAGGTCATGCAGGTGATTGTTGATCTGGCATTAACAGCGAATGATCTGGT<br>GTTTTCAGTGTCTACGGCTGTCCACATTAATCCGGATGCAAATGGAAACACTCGCGTTTTCAAATGTATCCGGATTAGTGTTAAACGCCTCGTTATTGCGGTTCAGTTTGCA<br>GTGAATGCTGCTCCACAAGAACTGCATCTCCCCTTTTAAAAATACAACGCACAACCATCTTCCCAAATTTTTAAAGAAAATTTGTTCAGAAACTTC
LG12	ref-25592	28.133	Chr23	6281352	TACCATTACATAGCTGACATATCCCGTTGAGCTGGTCACACCTGTGATTGTGTGTCCCTCTCCGGAGGGCTGCTTTGGTGTTGGCACTGCTCGGGAGTAAATGAAAACCTTA<br>CTCCCAATCTCCTTCCACGGGGCTTTTCTGTACAATGTGTGGAAAGCTCTTGCAGAAGTATGCGTTTGCATAGGGGTTGTGTGGTCCTTGTGCACTGGTGGAGGCTGAACTC<br>CGACCTGGGTTACAGGAAATTGGAGAGGAATAACTCAATGGTGATTCAGAGCCATGTTCTTAGTTGCATGGACATGTTTGAGGGAATGCTTTGTTTTTCAGCCATCTGTTTC<br>ATCTCCATTGTATCATGAGCCTGTACTGGCGTG
LG12	ref-29468_32	28.173			TCAGAGATCTGTTGGCTTTTTGGGGTTACAACTAAACTGGACAGTCAGGCAGCCAAATATGCAGCAACTCTCACTGAAAGGCTTATAAAACAAAAGCTGAGCTAAATATA<br>GGCTAAAACATTGAAACTTTTACTAATACTATAGCCATGGAAAATAATATATTCATCGTCGTTCTCTTTGCTGGTGTTTCGTGTCAAGTTT[A/G]CAGGGCAAACAGACAAA<br>TAAGTGTTATTTGAAGAAAATTAAAAGGTTATGGCATTCATTTCTGTTGCACTTTTATGAGCTTCCTCAGCTTTGATAGCAGTGAGTGAATTGTACTAATTTAGCTCTTTAAA<br>GCTGAATTTCTGTCAGCAACATTTAGTATAGTTATCTA
LG12	ref-45409	28.329	Chr17	44530350	TCACACTTTCCCAGATGGGGAGCACCATTTGAAAGCAGGACTTCCTTTTCTGACACATGGGGCCCTATCATACACCCAGCGCAATACAACGCCAGGTGCAATGCAAGTGTT<br>TTTTGCTAGTTTCAGCCTGACGCAGTTATCGTTTTCACGTCCAGCGCCACGTTGTTTAAATAGCGAATGCACTCTGTTCGCCCATGGGAGTGCTGGTCTGAATGCCTATAGA<br>CAGGTGCACGATGCACGTACACTCTGCTTGTTACACACACAGGGACACGCAGCAGAACACAAACATGACAAACATTACAAAAAATTTTCCGTGGGGATTCGGATTTGGAC<br>ATGCCCTAAATGCACCTGTGCCATGCTCTTTAGACC
LG12	ref-65080	28.986			TGGTGCCAGATATCACAACACACCTTCAGGGGTCTAGTGGAGTCCATGCCTCGACAGGTCAGGGCTGTTTTGGCAGCAAAAAGGGGATCAACACAATATTAGGAAGGTGG<br>TCATAATGTTATGCCTGATCTGTGTATTTTAAAATGAATTGCATGCCATTTAGCAACACAAGTCATCCAGCTTTTATTAATATGATATTCTAATATCGATCGACATTACTGCA<br>ATGCGCAACAAGTGTCTCATATCAGTTTACACAAGTCACATGAGTTCACACAAATAAGTCAGATTGAATAAATGGTATGCATATTTGGCGTGCTGTCCAGGAGAGGGCTCC<br>GAGCTCGGTATTTGGCCCAAACCCAGAGTATCCACCCACCCCCATTTCAGGTTAGGAAGTAACCAGGTGAGTGTGAGGAGACAGGATGGTGGAGGAA
LG12	ref-4515	29.42	Chr23	33166228	TGAATTTGAAATATTACCAAGTCGGCCAGGGGGAACCAAAATCTCCAAAATGGCTTTGGGATTTATCCAGTGAATCTGATCAACTCAGAGATGTCTGAGATGTGTAAATTA<br>AGCAAGTATAGCAACACTTTAAGAAATAAAATTTACAACAACAACAACAACAAAAATCTGCTGACTAAGCAATCTGCTCGGATAAAGCTATTAAGGAACAAAGAGCCCA<br>AGATAGGACAATTACATTTTAAGGAGCCACTTGGGGACAATAATCTAGCAGATAAGTTTGAAAAACTCTGGAAGAACATGACATGCCCTTCCATCGTTTTCTCTTGTCTTTT<br>CAGACAGTACATTGCATCTGACAGCCTTCAGACTGGC
LG12	ref-9033	29.666			AGCTTTATAACGACAGTAATGCGCTACCAAA

LG12	ref-30987_14	30.172	Chr23	24876719	CAATATTTGCTCTCTATAGAGTGCACCTCTTCTGCTCTAATGATGGGTTTTTCATATGTCTTTTCTTACAGTGGAAAGCTAGAACAGCCAGTTTTGACAGTCAGGGGTCTTGCCCCTCCCCCAAACCTCCTGCCAGCCCTTGATCAGAGCCATATGTAAACCTACAGGCTCGGCATCCTCGCTGTTGTCCGTTTATCTGTCCGATGAAAACGA[A/G]AGCCCTGCTACTTCCACTTTCCCCTACGGGTTGATTTAGATGGTTCCCAAACGCTGATCTAAGATCAGACCTCATGTTCCTCATGTTCAAGCTGTTCCAGTCCATATGATCCTCAGTCAGTTCTTGGAAATGTTGTACATACTCTACAAAGCCAACTTGTGTTCCTAGAATAGCTTGGTATCGTCAGCGGAGATGTTTCTTTCCAATATGAATTTTCC
LG12	ref-73386_28	30.353			CAAAAACAAAACAGTTGGAAATCACCATACATAATTTTAATGTTTTCATTTCTAACAATAATTGCTTTTCAAACCCAAATTTGATTCTTGACAATGACCGACTGTATAATGCTTTAATAACAAAGCAGCACAGTTCTGTATAAGTGAGCACTTGTTTTTTAAAGGTCATGGTTTATGAAGCAACCATCTCGTTCCT[A/T]TCATACCTGTTGAGTATTAGGCAACTCGAATACCATGGTGCTTACTAGTAGTCTGTTTACCAGTCAGTCCAAGAATAGCATGGCTGACAGTGACCAACACTTCGCTTCTCCTGACTTCCTGTCTTGTCATGTCAATAAAGTGTCATGACTCATGATCTATAGTTAGGAGACAT
LG12	ref-59777	30.72			TGCTGTCATGCGATCTCAGTGCACGTTGCTA
LG12	ref-42667	30.878	Chr23	22374303	TGAGCGGTGCAGTATGAGATACAAAGGATACTGTCAATATTTGAGACCGTATAGGATATTTTAGTGGATCTACACAGGATAAAATGACGTACTGAATCAGATATTAAAGCTGACTTGGCTATAACACAAGCTTGTTGACCATCACATCTAAGTGTACGATCCCCCTGGTACAGCAACATGGTGCAGGCACATCATAAATCATGTCTCGAACGAAGAGAGTGCAACGATCCTGCTGGATGTCATTTATAATTTAGAAATCACAATTTGCAAGCCTCATTCAGTTATGGTTCAGTTCATCTCTTAATGAATGAGAAAAACAAAAACAATTGGAATTAAAATAAATTATTAAAACAAATAAAGTGTGCATGTGAATGTGTGTGTGTGTTAGCACTTGGACTGGACGTGTCGAAATTGTTTCCCCCGCCCGCAGC
LG12	ref-72485_30	32.637	Chr8	7897177	TGTTTGTGTGTAAGACAGTTCTGGGAGGTGATTATACCGAACCACTTTGATGACAGACTTTGCTCAGGCATTTCAGATGCTGTGCAACGAGATCTGTCCACTGGTTGTCCCGGTTATGCCGTCCAATCGCAAAGTCACGTGACTTGTTTGGTGCACATCAAGGAATTTATTCGGTAAATGCATTTCCGTCGTAGCACAG[A/G]TTTTCTTATCGGATAAAAAAATTTTCAGATTCAGTTGAGTGCGTATGTTTTTATGCACATCTTGGCATTTTCCATCCAGCATTTTTTGCGATATCCCAAAATGTGCATAAAAATAGGTGCATGGAAACATAGCTAATGTTGTCAGATTTAGACAGTGTAAGTAAGTTATC
LG12	ref-26031	33.442			TATAAACGACCGAAAGCTGTGCAAAATGCAG
LG12	ref-54827	33.772			TGCAATTCTCTGGCGAGAGAGTTCCTTTGTTTGAAAGTGAACTCATTTGTATGAGGGGAGTAAGCACTATACAATATACAAAATAACAATATACAGTAATAATACACAAAATGTACTGGGGAAATGCATGCTCATTCAAGCAAATTTGACATTATGGAGTCTGCATGGGTGAAGGTTTGGAAGAATAACTTTTAAAATTGTGTGTTTGATTGATTCAGATGCTACACAGTCTAAAACCTTGTTAGACTGTGGGCCTGTTCACGCCAAGGACAATAACTATAAGGAAAATTATTTAGAAATAATCATTCTATGAGAATAGTGACGTCCACACCACAGCTATAATGATAACAAAGGAAAATACAGTTGTAATCACTCACTTTTTTTCCATCTGATGAACAATACAAATGTTGACCGCCGATCAGAATCAAGA
LG12	ref-60968	33.903			ATTTAAGTGACGACTTATTTGCAGGCAGGGA
LG12	ref-72798_23	35.349			GGTGTCAAACACTAGTTTTTATGAATTCTAACAATGCAGAATTTTGAGAACATTAACGTGAACGACGCTCGGGACCGTTGACAGGCCAGTGCTGCATCGTCATGAAAGTTTAAGCCTTGCCAATTTAAAAATTCAAGTGCAAGAACACGCGGAAGGGAACTCAATTAATTACTCGCACGCTGTGTGGGAAGATTTCTGTGTGGAATCATCCGAAGCGCATGC[C/T]CAGAAATCCATGTTTCGAACAGCACAAATACAGAATTGAGCTCTCTTTAACGTCTTATTGCGCATGAACAGACATATTCAAAATTGTCAGAATGCCCGTCTTGGCAAGTATCCTTGTAAACATAGTCAGTTATGTCTTAAGTGAACGTAAACAGTTGAGAAATAAAATGCATGTGTAACAGTATAATGGATCTGTGCGCTAGCTCTTAA
LG12	ref-62077	36.332			TGTTTCAAAGCAGCTTTACAGTGATAACAAGAAAATGATGCATTTCGTTTTGATTCAGTTCCATTGTAAAGATAATCAATTATTAAATTAGTTCATTTAATAAAGCAGTTCTGCAACAGCAAAAAAAAAAAAAAAAAATGATGTCATCGTCTAGCTCAGTTCAGTTCTCATACAACAGCGGCAGCACAGTCAGTTCAATAATATCGTTGAATATCATCTATCCACCAACTAAACATGCCAGAGATGACAGTGGCAAGGACCCAAACTCCATCAGGTGACCGAAGAGAGAAAAAAACCTTGGGAGAAACCAGGCTCAGTCGAGGGGCAAATTCTTCTCTGGCAAACGAACGAACACAGTGTGATGATGATT
LG12	ref-33457	37.859	Chr23	18680591	ACAACTTGTAAGCTGGGAATTTTCTGAGAGCTCCTACCACCTGACTGCTGCAGATTCTTTTAGGTGTTGATAGAACGTCACGTTGTCATTTAGAAATTATGACATGGTGTGAATGCATGATTTTAGACAACCTGGACAAAAAGTATGGGATGACTTCAAATATTGTCTTTGACAGTGGGGTGCAATCAATCAAAAACTAACATGTTTAACCCACGGATATGCATAAACCTAACCTATTTATAGAGGTAGGCCTACTGGTTTGGTTCTGCACTGATTTAAAGATGCGTATAAGAGAAATTGCACACAAACACAAAGCGTGTGATCTGCTTTGCAGTCAATATGATGTCAGACGAGTATTTGGTGGAGTGTAATATTGATGATGATGATGATGAAGAAGATGATTATGATGTGAACAAGATCCACCCGTCTCC
LG12	ref-36348	38.406	Chr23	40155476	ATAAATATTGATATTTATTATGAGCGAATGACCAATGAAAATCAATTTAAGGGTCAAATTATTTAGAAATTATGTCTTATGGTAACAATAATGCAGTGAGCACGCATTATGTGGGATATCATAGGCATCATTTAGCAGACTCTTTTCTCCAAAGCGACTTACAAATGAGAACAACAGAAGCAATCAAATCGACATGAGGGCAACAGTATGCAAGTGCCGTGTCAAGTCCAGCAAAGTGCTTGTAGCAAGGATGTTTTTAAAATAAATAAATACAGAAAGAGAGAGAATAGAAATAGAAAAAAGAAAAAGTAAGTGCTAATATTACTGGGTCAAGTGTCAAGGATACTATACAGCCTTCAAAAATGAACC
LG12	ref-52294	41.227			TCCACCATCTGGCTTATGGACCTTAAAAAAGCAAATTTTTGAACTGTTAACTTAATAATACACCAAAGAGACATATGCTAACGTTAGCCACAAGCGTGT

LG12	ref-32494_29	41.369	Chr23	40695925	GCAAACATCCCTTATTTCTCAGCATTAACTGGACACACAGTGATAGGGAAAAACACACACACAGATCAGTTTGTTCTGATTTGTTTGTTTACTGTCATGTCCATCAAACGCTGCTGATTAATTGGATTCAGTTGCATATGTTTAGCCTTGGCGTTCCATTACAGCCATATCAGCCCACCCACTTTCACCTGTCTCTATTACTTTCTCCCGCGATACAGTTGCTGTCAC[C/T]GCCGCTGATGTGTGGAAACAATATTGATCCACAGCCGGGGAGGCGGCGGTCCGTACAGCAACTTTATTGCTTTCTATTAATGATGCGTTTCATGGCCGATGGTACAAGCTCTTACAATGTGAACAAAGACCCAGGGCAAGAAAGACTGTCATGGAGATTTGTACTGTAAATATCGCATATGTTTAATTCAGTCTGGGCATGGG
LG12	ref-68133_26	44.651			ATGCAGCCTTGGTGAGCAGACGAAACTTCTTTCAAAAACATTAAAAAGCTTACAGATTCTAAACTTTTGACTGGTAGTGTATATATATATATATATCATTTGGTATGACAAAAAAGCCCAAGATGTCCTCATTAACATACAGCAGGTCTTGAAACAAATAGACCACATCATAACATGTTTTCAGAAGTATTAGCAAAAAGAAACACACGTCGACTCCAGTGCACA[A/G]CAATGCATTGCTGGCTGTCAGGAGGTTATAACATTAATCACCCTGATGTCAACCCGTTCACTCCTGCTCACGTCCCTTGCAGCACTGAACACAGAGAGATGGAGATCTGCCCTCTCCGATGACTCTTGGTGGGCGGCTGTGTGTTCACGGCCGCTGTGTGTTCGTGCCACTGGCGCAAAAACCTGCACAGAGTGGGAACAACTCCC
LG12	ref-12606_24	47.172			TAATGATCTCATCGTTAATACTTCTCACCATTTATTTCGAGGGTATGACCGATTGTTTTCCATATATCCATTTTCTTTAAAGTAGCCGTGAAAAGGTTGTTTGACTTGTCAAACATTGGTGGCTTTTTCTGGACCTCAGCGATTAACTTCTCTGCGATGTCGTCTGCCATTTTAAACATGCAAACCCTTCGA[C/T]TCGAATTCGAATGGATTCTGATTGGCTGTCAGTGTTTTATCGTTCAACAGTTGGAAAAAATTTTTTTCTGAAAGTCATCCCAACTATATCGTTTCTCTATATCATTATAACTGTTGTGTGAACTCTGCTATTCTTTTATATTTAGAATGATTTTTAGAACTATATCTTTATTGTTAT
LG12	ref-10695_1	47.597	Chr23	40704604	TTAAAAGAACAGTTAGTGACAAATAGACTTGTTTAAAGATAGAGTCTGTATTATTTGTTGAAGTGGTCGAGCTGTAGTGCAGTTTAGAGCCTCTAAAAATGTGCCGCATAAAAGTGAATGAGGATATGGTTTGGTGCCAGAATTAGAAATGGTTTAACTGTAGCCTGTT[A/G]ATATGGATGGCAGTAACCTCGTCCGTACCGCTAGAAACTGCCCTGTAAAATGCTCAATCACGCAAATCAACACGTTTTCAAGACCCAAGTGATTGACTGTTTCAAAGTGCTTAGCCACCTAAAATTCAAGCCAGTAATTACATTTCACAAGCATAAACTTCGTGAGATGGCCAAGAGCAGTTTAAGGAATATTGCAATAT
LG12	ref-21022	48.176			AGTTTTTTTTTTTTTCTTTTCTTTTCTTTTTGTAGAGGTGCTGTTGTTCGGGTTAGAAACATTCACACCTACAGGAGAGACGGGGTCACTATGTACCCACTTATTCACATTATATCATTCACAGTGTGTCTTCATGGTTGTGAGCTTTATAATAACTTCCTTCTCTGTTGTCCCGTGAATATTTTAGAGCATTCAACAATAACATTCGATCAATCCTAGTGCTGCATTATCTACATCTAAGTATTTACATCTATACTGTCTGGTAAACCCCTTCCAAACGGATGAGGCGGAAGGTAGTTTTCACAGATGTGCAGACTGGATATGCTTATTTGAGGACATAGTGCAACGTGTGTCTAAATCTGGTCCAGAACAACAGCACGAGTCACAGCAGAGAACGAGAGGGACGTGAAATCATTGCTGGTGAGATTTAA
LG12	ref-23091	49.057			ATCCAGCCAACGACTTCTTTGCCGTCCTCTT
LG12	ref-10695_7	49.993			GCGGTA[C/T]GGACGAGGTTACTGCCATCCATATT
LG12	ref-53326	50.864			AAAAGGTGTCCGACAAGGATGCATCTTATCA
LG12	ref-15548_6	52.852			GAGCA[A/G]GTGTCGACATTACTGCAGAAAATCAA
LG12	ref-70433	54.629			GCTGGATCAACTCAAAACCTGTCATCCGGCGGTGGGGTTTTGCTGCTTGTGGTTGGCACAACATCTTTTCCTCTCTCTGTCCGCTGGTCGGATTGTGCTCACAGTGCGCGGGCACGTGTCTCGTGTCTCTGTGCGCTCA
LG12	ref-48140	55.762			TACTTCATAACGAGAGAAGTGCTGCACGCGT
LG12	ref-38317	57.984			CACAGAGACACGAGACACGTGCGCGAGCACT
LG12	ref-40303	58.578			ACTTATACAACGAGGGAACTGCCGAAGCTAA
LG12	ref-73381	63.039			CTATAACATAAAAACAAAAACAAATAATTTAAGAAAAATACAATACCTTTTAATTACCGAAACAATGTTAGAAAGAAAAAAAATCACTACCTGGTAACGATAGGCCTGAAACACTGCTTTATCACCAGAGCGCACACAAACCACAGGGTGTGTAGACTAACTTCAGTTCAAAAGCCAACACTGGTTTTACACTCAGTTACACAGAAACCACAAGTCTGGTGCAGGGGAGAAGTTAAACTTCACTCTGCTCTCTTAGTCCACATGAAGCTGGAGCTTACCCTATCCAATCTATTTTTTTTCCTCGTGTCAAGAAATGTCTGCGTCCACATGAAACCACTGAAACGACTCAAAACGATGTAGTATACATGCCAGACCAGTAGAAGTGATTTTTGCCGTTGACACTTACTGCAGATCCCACTATCGGATATGCA
LG12	ref-62521	65.993			AACTGATGGACGATTTACATGCCACTGATGC
LG12	ref-32385	67.021			TGATTTTTTGCGAGTACCATGCCTACACTAT
LG12	ref-39674	67.648			TCATGATGCTGTTGTGCGCAATGGAGAGCAAGTGTATAGGAGGAAGTACATCAGCTCCTAAGGGGTCAAAGGTCATGTTCTTCTGTTGCCTTGAGCGATGAGAACTGCCGAAGCGCAGCGGCGAACTGTGAGGGCTCGTCGTCAGCAGCTCTAGAAGCCCCTCCCTCTTTAACACCGCCTCCACGTCTGCTTCGCTATTGGACCGCAACCCGAAGACCCCGCCCACACCCTCAGGCCCCGCCCACGAGTGACGCTTGTCCTCCGACTCCTTCAGACGTTTGCGCCGAGTCTCCTCCCACATTCCTCGCTCCGCGTTCTCCTGTGAAGAGAAGAAGTGCAGCTGATTAACAAAATGAGTTATAGTTCTAGCTGGAATAAACTACACCGGTGTTTCAAACCAGGGGGGTGAAAAAAGGGAGTGCTGGGGTAAA
LG12	ref-24980	69.093			GATTTGGTTGCGAGTGCAGTGCAGTGCAGTG

LG12	ref-52663	70.92	CAACTCAATGCGATTGATATGCCAATGCAGT
LG12	ref-52882	71.615	TTCTGTGCAGCGATGGATTTGCAGAATGCTT
LG12	ref-73426	71.615	CACCAGAGCCCGAACGAGATGCTGGGACCCT
LG12	ref-19630	71.722	TCCTGCGCTCCGAGCAATTTGCTGGCAAAAA
LG12	ref-304	71.776	TGGTCTACATCGATGCCTCTGCCACCGGGTG
LG12	ref-5749	72.13	ATAAGAGAACAGCCGGACAGACAAAGTATGTGGAGGCAGAGTGCTCTAATGAAAGAAAAGAGGATGAATCAGAGTTATTTGGAGGGTATACAGTGTACACAACCTCAAACGGGAAGAAAGGAATTGTGGTAAATGTGAATTTGGATGGAAAGTGCATGGACATGCAGCTGGACAGGGGGGCAGCGGTGTCGCTGATGTCAGAACTGTCATACAAGGAGTTCCTTTCGCATATACCTTTAAGCAAAATAAGCATGCAGCTAACTACATATTCAGGGGAGAGAATTCCATTGCTGGGGTCCATTGATGTGCCAGTGAAGTATGAAAAGAAAAATGTCACACTGCCATTGGTCATCGTCAAAGGGGACAGAC
LG12	ref-58725	72.185	TCGGCAGATATCTGCTATCGGTATCGGTGGAGAAATTTAGTATCTGTGCATCTCTAATAGCTATTGATGGAGATTCAATATTTTACGTTAGATTTTGTGTCTGCAGATTTTGTTTGGCACAAAGTTCCCTTTGTCTTTCCTAGTTAGTGTATGATAACATTCGTTTACAGTTACTATTGGCACATAGTTCGCAAACAGTTTAATGTCGACTGTAAATATTTTGGCTGATTTGATGCTTTCATCAAGAGAATCATGTTCTGAAATCTTAGGAACACATTTTACTATATTAAATCCATGATGCAGCATGATGAAATGTGTACAGATTCATAATTTCTTTTTGTGTGTATCTGGTTTACAGTGTTTGATGTT
LG12	ref-58216	75.438	GAACACGCCTATGTAGACAGCTGACTAGATTTTGAGACAGCTTCGGTAAATTATCTTTCGTTTCCTTGTTTGTAGTTTCGCTTTTGTTTCTGGATCAGCTGTATGAGCTCTGGCTCAGACAGGACTCTTTCATTCTTCATCATAATCAGTCGTGTCCCGCATCGCCGCGAAAATGTACGAGCTGGAAAAGGAGAATTTGCGGCGCTTGAGCGATAATATCGCGCAGTTTTTACAACCGTCACTTATCAAAACCTTCATGACGACGTACTTTGATAAAGGTGAGTTAATGAACAAATATTAGCATGTAAACACTTCCTTAAAATCTGCAGATAGCCTATTTGTTATCTTAGTCAAAACATCCTTCAGTTGTCATTTCAGAAATATATTTGCATTGTGTTGAATGTGTGTTAAATAATCAGAAAGTGGCTGCA
LG12	ref-50189	75.657	TGCAGCTGGATAATGAACAGATGATGTGATTGTTTGGTTCGTAGACCGACGTGCAGATTCTTGCAACAGACACAAAATTTATGAGTTTGTGAATTAGTGTTATTGCACACTAATACATATTAAGACAATTGCCAAGATGTGGAACAGAGAACACTGCATGAACAACAGCATCCCATAATGCATTGCACTCGATTTCCAGTGTGAGGGCATCAGTTGTTATTTCTCTTTCTTGACTCGTTATCTGCAACGTTTGCTTACAAAACTGAATTAAAAATGCAAAGTGAGCATTGTTATTTTTGAGAGGAAAATGCACCTCTTGCAAAAGTAATGTTTTGTACTACGTTTTGAAACTTTTATGCATACTATTTT
LG12	ref-56893_26	75.854	ACAGAGACATCGATTTTGTTGCCAA[C/T]AGCTGA
LG12	ref-43938	76.576	AGAGTGCCTTTCAACCCTCACAATACTCAAATTTTGTTTTTAATTTTCCTTTCACTTTGTCTTGTTATGAACAGTAGACACTGTTAAAAAAAAAATATTTGGCTGAGCTTCCACACATATTTTTAGATAATTATGGGCAATTATTCGGAGCATAAACCACAAGATGTGTCCACCCTGTGGGCATATCTCTCGTGATCGATCAAACAGTGTTTCAACTGCAGAAAGACAAATTTACCTCAGAAAATCCATTCAGTGACCATAAAATCATTAGATAACTAGTGATATCTTCATTAATGTGTTTGAATAAATCTGTCATGTAAAGATATTACATCGTTGATGTTTAGGCCTATTTCAAAACAAATTGGAGCAG
LG12	ref-49569_25	77.003	ATATCATCTATACAAACAAACAAACAAAAAAAAACCCTTTCATGATCATCTTGTGAGATTTATTTTATAAATAATAAGTTCATCCTTATAAACAGTCCAGCTAGTTGATCAATGAGCCAACAGAACGGAGACGACACGCCAGACCAAACACAGCGAGAATCCAGCACAAGCCAAACAAAACCAAAAAAAAGAGAGGTTCTGTTACAGAAACGAGTGTGCTGCCT[C/T]ACTGTCGAACCCTCACTGAGATGGAACCTCATTAGTGACCACTTTGGAACACACTACATAGGGCACTGAAGCTACAAGAGATGCAACTCACAATTGATTATAATAGTTTCGTGATAAAATACTTGTGATATGTTTAAGTCATTGTTGTTATAGTTTACTAAGACTATTAAAAAAAAAATATATATATAAATATAATAAAATATACAA
LG12	ref-49569_26	77.003	GTTACAGAAACGAGTGTGCTGCCTC[A/G]CTGTCG
LG12	ref-64390	77.432	TCACACGAGCCGATTTCACTGCATGAAAACG
LG13	ref-12968	0	GCGCAATTATCGAGAAGTGTGCGAGTACCCT
LG13	ref-15927	5.966	CAAGGGGCCCTATTGTAACAGTCTAAGCGCATGGTCTAGAGCACACGGCGCAAGTGCGCTTAGGGTGTGTCCGAGTCCATTTTTGCTAATTTAACCAGAGATTGTATTATTATTGGGAGATGAGAGGGTCTGTATCTCTTACCATTGGAGAACGTGTAACGGCTAACTTGTATCACGTGCAGCACCTCTCAGCCTATCGTGTAATGGCAGTGATGTCAATGCCCGCCGTTCAAAGTTTTATGACAGTCAGTTAGAGTTTACGTTAATCGGTTTCTTGTGGATTAGCGCGCTGGTCTTGTGAATAAATGTTTGTGGAATAAATGTGCTTTACTGAGCAGAAAAGAAGAGTGAATATCTCTGTTAGCTGCAAAGTTCACTGCAGGGCGCATTAAACCACATTTAAGAGTACACTAAAAACTTCATTATAATTT
LG13	ref-40395	11.692	TTTTTTTCTGCGATACATATGCAGGGCAGAG

LG13	ref-16150	15.066	Chr7	65975994	CGCAGCTTTCATTGGCCAGGGACCCCACAAGAGGATGTGTAATCGACATGTAAAGCAACCAATCACCCGTTCATTTTGTGTGAAAATGTAAAGTCGATAACAGACTGCCTAAGATAACAGACTGGTGTGCATCTAATAAATTATTCATTTAAGAAGATATCAAGTTTACTGCAACAATGGCACTACAATCGTTACAACAATGGTGCTACAAACTTTACTTTTGAAAATCCAGCGTTTAGTTGATCCTGATAAGCGTTCATTGTCACAGTTGTAAACACAACAGCTTTCTTCTTCCATGAGGGGGTTTAGATGGCGTTGTTTCCAGGTGGACTGTTAAAGAATGTGGCACATACGTCTCCTGGAAATCCT
LG13	ref-28062_31	15.296	Chr13	194924	CTGATGTTGTTTACCTCATAAATACTGACATTTGGTCACATCATTGTCAAAGAGCTGTTTTGAACAAAATGTCCCAGATATTCCTTTAAAATCATCCCGAGAAACCCTACTTGAAGAAGAATCTCCCACAATCTGACCAAACAGTTTACTGAGCATGAGTGAATGTGTAAATGCAAAACGCAGAGACATCGGTGTTCAC[C/T]GTTACCTCGACTGTGGTGGCGGCTTTCTGGGTCTTTTTCCCGATGAGATCCACATGGATGAACTGAGAGCGCATGTATTTCTTGGCCACTTCATAAACCCAGGACGGACACGTGGCGGAGAACAGCAGCGTCTGCGGTTTCTCCTCTGCATCTAAGAGGAAAAGGTCAGT
LG13	ref-72671	16.326	Chr1	17637455	TGTGATAGCTTCACAATGATTCTGAGAGTAAAATGAGCCCAACCCATGTCTCTGATCCAGTTATGTGCAATACAAAATTCCTATGCAGTTTACCATAGTATGTCTATGGGGAAATTTTGGGCAGCTCTTGCCCCCTAGGGGTACAAATTGCAATGTGTGTTCTTACAGAGCCTGCCAGCCTCTTCAAATGTGGTAACTCACATGTTTCTACAAAATCCTTGCTCTGAGCTATGACCAGTCAAACTTTCAAACTGCCAATGTTAAAGGGTTAGTTCACCCAAAAATGAAAATTCTGTCATTAATTACTCACCCTCATGTCGTTCCATACCCGTAAGACCTACGTTCATCTTCGGAACACAAATTAAGATATTTTTGCTAAAATCCGATGGCTCGTGAGGCCTGCACTGACAGCAAATTAATTTATACTTTCA
LG13	ref-25906_32	18.946			CACACCGGGTCGACAGGACTGCAGACTCCGG[C/T]
LG13	ref-25906_10	18.947			CACACCGGG[C/T]CGACAGGACTGCAGACTCCGGT
LG13	ref-65243	20.222			AATGTGGTAATATTCCATGAACACAATGTCACAACTTTAATAGTTTAGATTTGTAGATTTATGGGTGAAATATTAGCCAGGCATTGAGATTGATCCAAACAGCCCTGTGCATGAATATGACAAAAAAAAGTCAGATCACTAATATACACTTGTTTGTTGTATCTGAAACTGGTAATATTGTACTGGCGTCGCATCTATTGCAGTCTATCAATGTTTCAACTAAAGCGTCTGTCTCCAGCCTGCAGATATCGGCGTTAGTGTAATGTCTGTTTTAACTGGCTAGTCTTTGCTGCAAAAGAGAAGCTGGGAAAAGATTGTTTTATTTTCCGGTGTTAAAACCATCATGATGAATCCGCAGTTCAGCGTGTC
LG13	ref-57544	21.753	Chr15	43871320	TAAAGGGCACACACTGAAATGGCACATGGCAACTTTAACATGCTATAAAAAATTATCTGTGTGGTTGAGCTAAAACTTCACATACACACTCTGGGGACATCAGAGACTTAGTTTACATCTTGTAAAATGGGGCATAATAGGTCCCCTTTAAGTCACAGGAGGATGAGAGACTGCACAGGCTTTTTCCTGTTGAGATTTATGTTGACTGCACGAGCCAGTGGCATACATCTGATCAGTTGTTTCTTTGTTTTTGGTAGCCCCTGCAAAGGGCTTGTGGCTACAAAACAAAGGTTGTTGCATTGGGTAATGGATGCCCATGAGGCGCGTGGTTTGACTTTGCCTCTAGGCATTAGGACTGTGGCTTCTTCAGGTGCTATCTAACCATCTTTGGAAGATATTTGTGTGGCGGCAGGCTGGGCCCCTCCGCACAC
LG13	ref-38659	21.971			ATCTAGAACAGGAGCCTGTGCAGGGCCTGATTCAAAATTCGCAGATCTAGGATTGTTGTTACCCACCTCTTTTTTTTTTTTTTTTGGTACAATGAATTAAGGGCTGTAAAAGCCCCTCTTGATCTCAGCTGAAGGGACAGGCTATATTGCATCCTTTGCCAACAGGACCACAATTTCTGCAAGTAAGTCGGGCTTCCTTTCCTGCCACCAAGGTAAAAAGGACGCCATTGAACCTGGGTGGGTGCCTGGTGAATTGAATGACATAGCCGAGTCTTTGACATACCTGCGGTAGGCCGGTGATGCGGAGCATTGTCCGCCATGCCGGGAAGCCGTGCATCCTGTTCTTGTGGCTGTGGGTGTGCTAGAAC
LG13	ref-53921_7	22.409			TTACTACTGAAAACAGCTGGGCAAAGTCCTCGACAGTGTTGTCCAGCCCGATGCCCGCGGCAGCTCACAAAAGCATGGCTGCCAACTCTGCATATAACTCTGACTGAGTTACCACACCTGAGGGTGGGAGTCCAGCCGAGTCCTCAGTGTCGGAAGGCATAAGCCTGTCCTCTGATGCTGCTATTGACAGTTCATCCTCATCTGAA[G/T]CTCCGAATGAGATGCCATGTACATCATATGACGCCATGTCAAACTCATCCGGGAACTCCACCGACGCAACATGCAAGATGAAGAACGGGAGGGCCTGTGGAGATTGACCCTGTGAAAACACTCCCGTTGCTGTCATGTCACCCTCAGAGCTAACTGGCGCAGCCTTGTGCATTGCAGAAGGACAGGGACATGCAACTGACTCGCTTACCAGAAAAGAGAGTCACT
LG13	ref-54808	22.936			GGCCATTAAACGATATGAGTGCACATCCCTT
LG13	ref-21655	23.024			GTTTCAACATCGAGCGAGATGCAAACGCGTT
LG13	ref-3449_14	23.604			TGCGCACAGAAAACAGCGTGTGAGTTCCGAATTGAGTTGTCTTTTGCGTCTTCTTGCGCTAGAACGGTATAAAATCACTTGAATGTTTAAACTGACAGGACCTAAAATGTGCAAATGATGAATTTTCGCTTTGTGATGAATAAACATTGCAATTAATCAGAGAATTACATGTGCTTTGAACCATGTTGCCTGGTCTGTACGGATCAACTACGA[C/T]CCCTATGCTTGACATCGCACATCCTGCAATGTGACCATCGTGGACGCGCACACAGCGATATTGATGCTAAAACTAGGCTATATATCGCACAGCCTTAGCAGGTACTCATTATTTCTGACAAGGTGGTGATTGGTAGAGACATTCATTTATTTAACGGTTGTAATTCAATCAAAATCATCAAGTAATTACAGTTTTCATCTTTTGAAATGCATGTGAAG
LG13	ref-3449_30	23.629			GGATCAACTACGACCCCTATGCTTGACAT[C/T]GC
LG13	ref-42955_8	24.179			CATTGTCCTCAGAGAGGGACAAAGATGATGATGTGGAATTCACAAATCTGGATTGTGTCTAGACTTTCTATACACTGTATTTCTGTATCTGCAGATCTGGAGACTGTAGACGTTGTCGTCTTATTACCAGGAGTAGACTCTGTCTCTCAAACGAAACTTACTCTGTAGCGTGTGTTC[A/G]CGCAGACGATTCGCGTGTGACAGATGATACTCCCATAAACCCTCAGAAGGGCTTTTCACACTTGAAATGCTTATAATAATAATAATAATAATAATAAAAAAAAAAAAAAAAAAATATATATATATATATATATATATATATATATATATCAGTTATCAATCGTTCTTATTGTTTAAATCTCGTTTTCTTGATT

LG13	ref-959_15	25.965			TCCTATGCAGCGAA[C/T]GCAGTGCAGAGCTTCCG
LG13	ref-43811	28.315			ATCCATGAAGCGAATCTTTGCTCCACCACA
LG13	ref-67275	30.538			CCTCTGGGGTCGACGAACGTGCTGACGCGTT
LG13	ref-57526	31.585			ATTCTACCAACGAATGGAATGCCCAAGTCAA
LG13	ref-6120	31.951			ATCCCATATGACACGTGTCGTATATTCCAAGTGGTCTGGGGGTATACGATAGGGTTTTGTAAAATCAAACTGAAATTTAATGAATTATTTAATAAAAATCCTGATCTTGGCCGTTTGACTCGATTGCGTGTTCATGAGACTCTATTAGCGCCACAGTTCACTCCGACTCGCCCAGAAAAGCACACAAGTGGTGAGAAATCAAGATGAATAAAAACACAACATGAAATACAATCCGCTCAAAGTTCAATGCAAAGGGAGATATTTTCTTTTAAAAAGAATTCTGTTTAAGGACTACAACAAACGGCTGGTAGGGACTACAACAAGCTTCTTCCTGGGTTAGTGACATCACTAACCCTAAAATTTACATAA
LG13	ref-43520	32.625			CAGCCGAAATCGAACAATGTGCAGAGTTCAC
LG13	ref-24759	32.785			TAAATGACTGTTACCCACTTCCTAGAATGGAAGATTGCATTAACAATTTAGGATCAGCCAAATTTGTCAGCAAGTTGGATTTACTATAAGGGATTTACAAGGGGTCCCGCTAACTCCTTGCGCCACTGACATTTCTGCTTTCGTAACTCCGGATTATTTTATGCAATATAAGGTGATGCATTACAGAATTTCCCCGTGCCTACTTCACAGCGAGAATTGTGTCATTTTTTGGGTATGGCAGGCTACCATCGTAGCTTCCATAAAAACTTTTCTACGGTGGTCACTCCCTTAACAAATCTGCTTAGCCATGCTTATTCTTTCATCTGACAATTGTCAAAATGCATTTGAAGTGGTGAAAGCTTTAGATTCTTATGCATTGGTCGCTGGAGGTTCAGGGATATAATCTTGATATCAGACATAAGAAAGGGTCA
LG13	ref-66428	33.153			AATGAGTGTTCGATATCATTGCCCGTAAACC
LG13	ref-14344	33.77			AGACCCGAACCCGTGCGGGTTCCTGGTCGGGTCCCGGGTCCTCGGGTCTCGGGTCCTCTTTGTAGACCTCTAGTACACACCTCAAGCACCGGATGCCGTGCGCGCGCTCAAGGCAGTTGGACATAGTGGTGTATTAGAGGTAAAAAATGATATAAATACTGTTCGGTCTCTCGCACAAAGCGATAGTTTCGTGTCTTAGAACATCACTGTGTCGGTGTGTTGGGTCTATAAGCACTTCATATAAACCCAAAACTCAAGATGAAGCTCTCATGCCATGGCTGCCTAAAGCATCATTTATTAATAAATACCTTCTTATATTATGAAATCTGAGCTCAATCTACTTGACCACCTTCCGCAGCTCCGCCAGGA
LG13	ref-68693	34.516			TAAAATCACTTGATGTGCTGCGTGCTTCCTAGAACTCTCTGCAGTGAGTTATGAGTTTTGTCTGTTATTATCTTGCTTTTATTCATAGTGGATTACATGCATTGTGTACAAGGTACAAATTATACATTTAAGTATGCAATTTCGTATGTTCCAGGAATTGAATGACTTTGCTGTTATTAGCACAAGAATTGCACAATTTTGATTAAAAGAGTCTACAATATTTGTTTTATTTTCATGTATATTAATCATTATATTAAAAAAAAATTTAATTAATGTAATACAATATTATAATCAATAGGCTATAATATATAAGTAATAATTAATATCACCAATAATTATTTTGTTATTTTTCAATGTTTTAAAATTTAA
LG13	ref-13688	34.708	Chr13	33713539	TGGAGAGTAAGTGCACACACACACACACAAAGTCTCTTCCGACTGGCTTAATGGGGCCTCACCTGTTGGCGTCCTGCACGAGTGTGTGTTTTAGCAGTTGCTGGCATGTGTTTAAAAAGAAGAACTGACGAAAGCCAAGCCAAACACGGTGGGGGAATGTCAACGACCGTCTTGAGTCAACACAACTCGACTACGGTCGTCTCCGTAGTTCGACCCGCATGCCTTCAGGGGTCAAATGCTATGGTAGGGAATGGCAGGCATACAAACACACACACGCACACTCAATCTGAAGCTAATTCTTAAATCCTGACTTGAATGGTAACAGCAGGTTTAGTGGCGGATCACTCCATTTGAGGCCTATCAACCCTTTTTGGCAAGTTGAGACCAGCTTGATGTCTAAAGTTGGGCTTATGTTGATCTCTGTAGGCTGA
LG13	ref-67239_1	35.415			GGAAAGTATTGTACATTTTTTGTGGTGTCATGTATAATATTTGTTAATAATTTAACGTTATTGGGTGTTTCTCATACTTGTGTGATTTAATGCCATATTGCATACCTTTAATATATACTGTGGATTATTTGTTGCAATTTTATCCTTTGCTTGGTGTTTTCAGGATGCT[A/G]AGACTTTCAGCATTCCACTCGTATTGTTGTTATTTGTATTGTTGAGTGCATGAGATTTTGTGAACAGTGGATTCAAAAAGAATTTGGAGACTTATTATTGTTCAAAACGTTTTTGGTTTATAATGCACCGACAGTATGTCCAAATGCTTTTTGATGCCACTTTATATATTAGGGAAATTTCACATTTTAATTAACATTTA
LG13	ref-15390	35.492			TGCAATTGTAAACAGTATACATACGATGCTTTTCGCCACAGTTTGATGTGCATCTTCGCAAAACATTAGTTAACTTTAGTAGATTGCATATCTTTAGATTAAGTGGTCTCGAATGAGCCCAAACACAACACAAGACAGTGTGTAGATCACAGAGTGACATGACATGCTTCTTGGCTAAAGCTATAGGACTTCCAGGATCTGATTGCTTCACGATCATGACGCAGTGTTTTCAAACGTCATTTGAAAGTTTAACCAATGGAAACTGGATCTCTGGTATAGTGGGTAGGGACGATGTGTCGAGACCAAAGGCCACTGTTTTGGCACTGGTAGATCACTCATCGACCAGTATAGGGCTGCACGATAATGGTTAAACTGATAATTGTGATTATTTTGCTCAAAATTATAATCGCGATTATTAAATCATGATTATT
LG13	ref-23010_2	35.598	Chr13	33668007	ATTAATCATGCCTTTCCTGTGTGTGGCGACACAACAACAGTGGGGGACTGAGGGGGTGGTGGGTGGTGTCTAACGTGACATGGGCCGTCCCACCAAAGCCCTATGAACCTGTATGAAGAGGTCCGTTCACAATAATGCTTACCAGGTCACTAATTGAATTATGTCAAAAA[A/G]AGCATAAGGCACCAAATTCGTTTAGCCATTGTAAGGAGGCCAAGACCTCCAGAGGTCCCTGTTTAAAAAAAAGGAGATGGAAATTATGCACGATTATGTGTGGGTAGTCAGTAACTTTTTGTTGTGAACATTGGTGCCAAACAAAAAAAAAAAAAAGCACTATGCAAATTGATCATGTAAATCTAGTTTCCAACTCCTG
LG13	ref-56016_29	36.002			TACCCAGTAGCGATCAGTTTGCATTTCA[G/T]ACA
LG13	ref-24749	36.04			GAAACAGTGACGACCCTGCTGCAGCTGTTGA

LG13	ref-70250	36.277			GAAGCAGGAGCGACCAGTCTGCTTGGTCTTA
LG13	ref-5716_30	37.157	Chr13	30001089	TGACCAACCCTCCAGTGTCCACCATTTAATGTCAGAAGATAGAGCTGCCTCTGTAGAAATGGGCCACAGAAAGCTACGTCGAACAAGGCGAGAATCGAGTTTCTTCTACCAAATGTATCCACCGTCATCAACCTCCCCCACCTCCCCTTCCTCCTCCTCAGCCTCCTCGCCGTTCTCCTCTGCCTCCTTTTCCTCCTCATCTTCCCATCCGAGCCCGCTGCCAAAATC[A/G]CCCGAGTACGTGACCACCTCATCTGGAAAAGGAAACCGTTCTTTAAAAAAAATATCACATTTCATATATATCATATTGCGAGGTAAACTGAGCGATTTATGCGTGACAAAGGCTAAACAATACTCGCTTGGCGCGAGGACAATAAACAAACAGGAGGTGTGACTTTGACGTTGGGCTTTTTAATCGCTATTTTATGTCCAGT
LG13	ref-47714	37.539			CATGTACCTTCTCTGATGTGAATATATCCGTTGTGTTCATCGCAACGAAGTTATTGCGATCACATCTGTCAGCTGTCATTCTGACTGTCATCAGACAAACTGAGTTCCGGTCCAGAGATATTTAAACTCAGTCAAGAAAGTACACGGATACTTTTTGGGATTTCTAGGCATTGTATTTCGTGTTGCGCTTTTATTAATCTTGATTTCTTACGACTTGTGTGCTTTTTTGAGCGAGTCGGAGTGAACTGCGGTGCTAATAGAGCACGACTTGTGTGCTTTTTCTGGGAGAGTCGGAGTGAACTGCGGTGCTAATAGAGTCTCACATACGCTGACGCTGCGTAGCCATGCATTAAAGTGCAACAGACAAGGTTGGGATTTTCGTTAAAAACTACATTAAATTTCATATCGTATACCCCCAGAACACTTGAAAT
LG13	ref-42949	37.777			CCCTGAGAAACTTTGCCTTCACTTGCAAAACCTTTGCGTTCCCGCACAAAACTTTTGTGTTCCCCTTACAAACTTTGCGTTTCCTCGAGAAACTTTGCGTTCCCTCACAAAACCTTTGCGTCCCCTCAAAAAACTTTCACATTCCACCAAGAAATGTTTGTGTTCCATCGAGAAACTTTGCGTAAAGCTCGGTACTCATACTCGGTCCTTAATAAATGGTATCGGTGCATCCCTAGTTCCTTCACAAAACCTTTATGTTCCCCCCGAGAAACTTTGCGTTCCTTCACAAAACTTTTGCGTTCCCCCGAGAAGCTTTGCGTTCCCTCACAAAATGTTTGCATTCCCCTGAGAAACTCTGCGTTCCCTGAC
LG13	ref-56264_14	38.269			TTTTGAACGACAGTGTACAAATATATTTAGTAATCAACATGTCACAGATGCTTACGTTGCATTTTACATATCTTTAATTTTCAAATTATTTTTTGATCATTAATTACTATCTTTTTCTCTATTATTCAACAAAACGTAAGTGCTAAATTACATATATTAGTAGCTTAGTGTAAGTTTCTGCA[C/G]TCCAGTCGTCCAGCTGCTAAGAGTGGCAGATGCTTTGCTGTAGCATGATAATGGCCCTCATAAATTACTATTTATTTATGCTGACACCACCCATATGTTTTCAAATTCACTGCTTATGGTCCACATTGGAGCTATTCCAGGGTTATGAAGTAAGAAGACAATCTCTCCATATGCAATTAAAGAAAAA
LG13	ref-23355_7	39.28			TCAAAC[G/T]TGCCGATAGTGATGCACTAACAGTT
LG13	ref-38890_5	39.399			ACAATATCTGACCCATCTGGTTATGCTATTGAGTTATTAAATTCTATCACACTTTTGAGGCTTTCACGCTGTTGAGAGAAACACAGCTGAAAAGGAACTGCTGCATGAACCTCTAACATGATTAGCATGAAGCTTGGATATATATTTTTTTTCTGTTGTCTATTGTGAAATTATGCATGTCTGATACAAATCTAGAGATAATGC[C/G]TATACCGACTTGATTGCCGTGTCAAACTCAAATGGCACGTCGAAATGAAATAAAATGTAAGAGAAATGTAAAAATAATAGATTTATTAAGACCATGTAACATGCTAATTTAGTAAAATTAATTAATTACATAAAAAAAGAAGGTAAATTTTAATTTAATTGTGCATCCTGAACCAAACATAAATTCTGTAATAAATCCTTCAACAACAGCCTTCACACCTTGCCAAAG
LG13	ref-38625	40.026			AAAAACAAAACGACCCCTTTGCAGTTAAACA
LG13	ref-20588_27	40.563	Chr13	37023612	CCCATTTCTTCTCTGATTCATAGAATTTATCTGCAGAGCTAAACTCCTTGGGTGCCATATTGACCTCCCAGGGGTCAGAGGTCATAAAGTTGTTAGGTTCGGATTCAGAGGTATCCTCCACCTGTTTTGATCTGCTGACGCAGCATGTATGTGACATTCAAAAGGCTCTGGCGGAAAAACAGGAGCAGCTACAGGAGCAAATAAAACACTCGATTCAGCTGCAGGT[C/T]AGATATCTGTTTGCTTCTATCTATTTATTACCAACACAAACTGATGAGATGGATGTGCAGGCACTTGAAAATTCTCCTAGTTACTAGACTACACTAAATAGATCAGAAAATGTATGCTTATGTGTTTACAGTGTTATGTGTCAGCAGTTAAATTTTCGCAGGTTTTTACACCCCAAACCCTCAGATGTCTCACCCTCATAACTGT
LG13	ref-12446	40.693			TGTCGTACACCGATTGGTCTGCATCTCGAAC
LG13	ref-3438_5	40.893	Chr13	22852602	GATGATCTGAATGTAATACTCACTGTGGGTGCATCTTGTATAGGGAGCCATCGATGCCCACTGTGGTGCGGAGGCGCGGGGTGTTCTTATTGTCCTTGAGACGTGTGAGGATTGCCCCCAGTGTGGCAGCAATTAGATTAGCAGAGCGAAAAGAGACAATAGCGCAAACATGC[C/T]GTACGGCAATGCAGTCGTCTTCAGATGGCTCTACACCCAAGCGTGTCAAAATTTCCTTGGCTTTGGTAAGTCCCTCTTTGCTCCTAGAGGAAAAAGTGGTATTGTTGTAAGGTCGCAAATATCATGATTTTCAGAGTCCACTTGAATTAGCTTTAATTTTAGTCCACTAGTGAATGTTCTTCCATTTTAAAAATTA
LG13	ref-36081	41.796			TGAGCAGGAACGAGGAGAGTGCTGGGACACT
LG13	ref-48950	42.076	Chr13	29293306	TTTAATTGGACTCAGTATCATGTTTGTCTCGACGTGTATTTTTGCCTTCGCAGAGAACTACGCGACTCTGTTCGTCGCGCGCAGTCTGCAAGGGCTCGGCTCGGCGTTCGCAGACACTTCAGGAATTGCCATGATCGCAGACAAGTACACGGAGGAGGCAGAGAGAAGTCGCGCGCTGGGCATTGCCCTCGCGTTCATCTCGTTCGGAAGCCTGGCGGCGCCCCCGTTCGGAGGGGTTCTGTACGAGTTCGCGGGCAAACGCGTCCCGTTCATTGTGCTCGCCTGTATATGTTTGGCAGATGGTATACTATGTCTGACTGTCCTCAAGCCCTTTTCCAGTAGGACTAGAGAGAATATGCCAGTTGGCAC
LG13	ref-2541	42.342			TGTGCTGTTCTCATGTTGTATTTTCTGATATGGCATTCCCACTTCATTGTCACTCTACCCGCTTTATTCTATTTCTAGGAACTATTTTGCACACGCCTGGCCCTTTAAATCCCGCCCACTTGACAGACAACCGCACAGACCAATCATAACACGTTAATCTACAGGCGTGGCCACAGCCCGCACAAACACCGAATCTGCATTTGCTGACGTCACTGAGATCCACTCCGACCTGCCTTTAGCTTAGCCTCTCAAACTCTTCCTTTATTCAACTAGATTTGATTTGGATCAGGTCTGATCTTGTTGTAAGTAGGAAGTAAGCGAGTGTTACGCCGGATAGACAGTTACGAAGGCTGACTGTTTATCCGACAG

LG13	ref-58905	42.526	Chr13	25394569	CGAGCAGCCACTGCCCCTCAAGTGTTACAGGATCTATCAGGTGATGTGGGCCAACAATGGAGACACCATTAGCCGTCAGTATGCTGGCACAGCTGCCCTGAAGGTAAATGGATTGAATGCCTGCCTTTGATTTTATGCTGTCCCCATTGCCTGTTTACAACACCATCTGTCAGCAAGTGGCATTAAGATTGCAGATTTACGAGTCAGTAATCTTTTGACCAGTCATCTTATTGGGAGTGACTATATTATATAAATTCTATAAATAATTTATTCACTTATTTATATAAATTAATGATTAATAAAAAAGAATTCTTAAAGGCACACTACGTAATTTTTTGCCTCTAGAGGTCGCTTATTCAAAACAAAGGC
LG13	ref-27417_5	43.154	Chr13	43451512	TTTGGGAAATCACCGGTCATCATTTAGTCTGGATATTGAGTCTGTTGTGGAGAAGATTAATTCCTAAAGCATTTTTGGCCAAGTTTATGCATTTTTGAGGACTTATTGTGCACAACAGCCATGCCTTCCCTTCCCACTTTGAACAGCCTGGGGAAACTCTGCGGCTCAGGACG[C/T]AGTCAGCAGGTGGATCGCACCCGGATCAAACGCACAAACTCCATCAAACGCATGTCTGTCATAGAAGATGGACGTGTGGCAGAAGTGCTGTACCTCATTCCGAAACAGTCTATGATGCAACAGCTGCCGTTCATCAACCCTGAAGACTACTACCTGACCGAGAGCCTGACACCTGGTACGAACGGTTGTGCTGAAG
LG13	ref-44593	43.35			CCAGGATGGCTCGAGGGTCTCTCACCAACTGTATGAATTTGATATCCAACCGAGGGTCCTCTACTAGAGAACGAAGTGTGTCTAGCTGCCGGACCCTCACTGTTTTGATCACCCTGTGCTGTTTTTCTAGGCAGGCCTCAGACGCCAAGGTCAGATTGAGTGGTCCGCAACGCCTTGTCTTACAGCGGTACCTCTCAAAAATGTCTTTTACAACCGGAGTGCAGACTTGCTCCTCACACAAAGCCATACTGGACTCTCTGCGGAACAGGGCAGGGGTGACGTGGTCCCGTGGAGGAGGGCTAATGAAGCGCTCCAACATGGAGAAGTTACACAAGAAGAGATGCTGCAGGACATCCCTGTACACCCAAACAGAAGTGCTGGCATTTGTGCCACTCGTGCCAATCGATAGCATCCTCTCCACGTGCCAGAGT
LG13	ref-69813	43.757			TCTCAGATCTTTCGATCTAATGGACAAAATAAGCTTGTGCAATTTACATATGAACTCTAACTTTTCATCTTCAAACCAAGCTTGGGTTTCCAGCCAACAAAGTTTAAATCCTATCTGGATAGCTCACTTCCCATATTGTTTACTCATTAGGTCAGTAGGTGGTCTTTTGTTGCTGTTCGAACGCATTCGACCACATGAGCGTTTCCACTACGAACGCAATCCAAATGCGTTTTCGATTACCTCTGCAAGTGGTCGAAAGTGGACAAGTTCAAAATTTTTTAGACCCCCTTTACGGCCGTATTTACTGTCGTCCACTTGTGATCCATTCGACCATCTTGATACCAGGTGTAAACAGGGCCTCTGTTGCTGTAGAACCATACGCACACATAAGGGAAATCAAATGTACCTGTTCTGGTGCCAACTGAAAAATG
LG13	ref-53045	44.926			CAGCAGATGCATGCATGGGTTATCAACACACATGGGAATTAATGGCAAATCAAATGCACAGCAGGCTAAGGCATGCACAGAAGCAGCTCTTTTTTAATGCTATATAGCATAAATTAGGGCTGGGCAAGTCGACATTTTTCAACGTGTTAATATTTCAAAACACACCAAAGTCACACTGGCACTTCAGTCGAAACTAATTAATTGTTGAATTACTAAGTAATGAATCAAATAAACAGTTTGGACTGATTCACAGAAATAAATCAAACTCA
LG13	ref-18856_32	45.121	Chr13	7667130	TTCACAGTGCAAGCTAAAACAGGTCCAAACGAACTAGAATAATAACCAATGATACTGGATGTTGTATATGACATACTTTAGCAATTAAATTAATATTTACAATTACAATAAACATTAATATTGTCCAATCTGACTTGATAGGATCAATATGGACTTTCGCATAAATGATTGGTGTAAAAGCAAAGGTGTCGTACAGTTAG[A/T]TACAAATGTTTAGCTTTAAATGATGGCATGCCACTGAGGATTGATGACCAATTATTCAAGACTACAGTGATATACTACAGACTACCTTGTTGGGCTTAGTGCATATTATCAAATCACTCACCATTCTTCCAGGAATGTAGTGGTCAATGCAAAGTCAAAAGGAAAATCA
LG13	ref-48059_3	45.565	Chr13	11106228	CCAGCCAGTGCTGTTTCATCAAACCATTTTTACTGGCTTCTCAATCGTGAAATTTGTTTCTTTAGATCCAAAACAGTAGGCGGACAGTTGGTCTGGCAGGCGCTGGTGATGATTCTACCCTCAAGTTTCCAGAGCATTTGGTCAAAGTGAACAGTCCTATTGGTTACCATT[A/G]TTACCAAGCACCAGGGTCGTGAACACTTTAGACGTTCAGATTAGTGGTTGACCACTTTGGTTGATTGTTCAATGAATACGCTTGGTTAGAGCCTAAGAATCTGCATAACGATACACTTTCTCAGACTAAAAAGCTTTCAGGTAATACCTGAGCGATCGACGGCAAATATGCAACAGGACATCCAAAGTCCCTCCTGCC
LG13	ref-68791_1	45.731	Chr13	7112235	AGACATGCACTGGGAAGGGTTTCAAGAAAAAGAACGCCCACCGTGGAGGCCGAGGCAGTGTAAACAGAACCACGAGGCTCTGCTTAAACGACTTGTTCAGAGGCGAATGGAAGAAGCACAGTTAGACCCAAGCATTGAGATACATTCCTCCATGCAAAAATCGATCACTAGCAAAGCAAAACAACTGAAAGAAGACTTGTT[C/T]AACGTTGCGACATGGGTGCACAGCTGCTACCCAGAACAAGAGAATGTGTGCCAGTTTTATGCCACACTGTACCATAAGATCTTCAGTGCCAAGCTGAAAGAAGTCGCAGAGTACACACTGTGTGATGAGGACTGCATTCTTTTATTGCAGTGGGTGAAACAGGATTACCCCAAGTAAGAGAAGCAAATAACACTAGACTGTTTTTTCTTATAATCTATCTATTATCTTC
LG13	ref-41831_7	47.262	Chr13	9589256	TGTGTGTTTGTGTAGGGGGTTGGTGGCATTTGTCAGCTCGGTTCTTCCGCTTGGAGTCCAGATTGATGGGTCATCTTTTCCTTCTTGTTTCCTTTCAAAAGGTTTTCCTCAGAAAAGGTCAGATACTTAAAACTATTTGCTTCACAGCATGTCACTTCCCCTTGCAGGGTCAGTAAGGGAAAAGTGAGAGAGGCACGGAGCTTTTTG[A/G]TGCGACACGAGTGCGTCTTAGTGCGCATGGACTTGTTTTAGGAGAGAGCGGGAGGGGGGTAGTACCGACGGTGCACTACCGTGCCTTGCCTTGTGTCGGCTGTCTCTACAGGTTACTCTCTTGTTTCAGACGTTAGCCATTAACCGTGGGGAGAATCTGAAGATTCTGACAGTGAAGGTCAACATCCCCGACTGGGTGAAGAATGATTTCTGTAGGTGGTGCGTC
LG13	ref-49218	48.64	Chr13	19452558	GCGCTCCGTTTCCATAGTGCAAACAAGGTGGAAATTCTCCAATGTTTTGCAAGTGTCACATAGCTGGGTGATGAGCCATCAAGCACAGGCCGGCTGAAATATCACTGACCCTCAGTCACCCCTCTTTTTTTCCCATCCGTGTTTTTCTCCGAAACGTTCTTGATTTGCAAGTAAAATGAGCACAAGAGTCGGCCTTGTAAACTACTCGCAGGTGGTTCGATTTGAAGGGGTGTTTTGATACAATTAGGCAACAGATAGAGGGAGGGGGGGTTCAAAAGGGGTCTCTGACTGTGAGAGTTGGGAAAATATGAGTAATTAATTGTGAGGCATCAGTTTTGACGGGGCCAGAATTAAGCTGTCAGACTGTAAG
LG13	ref-63385_23	49.39			ATATCATCATCCTGTTCAAATGTTCAAATCACACACCCCGGCACTTAAAGGCACAATATGTAATTTTTCACCTCTAGCGGTCACCTATTTAAAACAAAGGCATAGCTTGATGATGCCAAGATTGAGCACAGAATCATGGGAGAAGTCGTCTTAACCTCACAGCTGGTAGAAAAGAATTGGCACAGGACTCG[A/C]ACAGAAATCATGTTCATGGATGAGATT

					ATTAACGTTACTTTACTATGAAGCAGAGCAGGGCCGAGTGTGTATGTGGGGTAACGCAGTGCTGTGTATCATATTAAATTTGAGTGTGTTGAAAATTATTCAATTGTGTTAC TCATCTGCGGCTGCTATGAGACACTTTCTGTACACTGCA
LG13	ref-19317	49.563	Chr13	14081131	ACCATGTAAACACATCCATTTCACAGGTGGGGTGGTGCTACAGTCTGACCGCTGAGATCTCGGAGATTAGCTCCAGCTCTGATCTAGAAGCGCTTCAGTGTGATCTTGCTC CTCAAAGGTATTTCCGGCATTTACAAAACTGCAGCGTCAAGTTTACGCCATCCCTCCCTATTCTATCTGCGGGTCTTGGGGCAGTCCAGAGTGCTGGGTCGGTGTGTATGCG TGCTGATTCAAGCCTAAGCGCAGCCTCTGGTGTCTTGGTAATTGAGCGTAATTTGGCTCCCTGCAGTAAAACTAATGGAGCGGAGACAGCCGGAGAGTAGCAAACAGGAC AAAAGTAGCACAGGATGGAGGGAAACAGCTGAAAGAGCTTTTTGAGAGTGAGTCCACTGAGGCTAGTCTTCACCTCAGCTGTTACTGAACTAGTTTTG
LG13	ref-24431_8	50.922	Chr13	11192146	TAAAGCCTGCTACCAGGGCCTGTCCTTGTACCTCTAGTCATCACTTTAGCTGGTGCGTAAACAAGTTGACAGGCATGGCCAGTGGGCCGTGCTGGACACCCATGCCCTGCT GAAACACACAAACACACACACATCAACAGCAGCACCAGCAACTGGTTCCGTCCTTCCGGATGGCAG[A/G]AGCAGCCCTATCGATCGATGAGCCCCATGCCGTCTGCATGC TGATATGAGGTCCGGCCTGTCCCCGTGTCGCTCTTTGCAGAGGAAATTTTCCCGCCACAGTAATGGGCCAGTAAACTTTTAGTGCTCTGGTGATTGAAATTTAAAGCATAGT CTTTTATAAGGGCAAGAGCCGGCAAAGTGAGGAGGGATG
LG13	ref-66424_29	51.042			ATGTTACAGTCGATGGATGTGCATTCTGTTT
LG13	ref-66424_27	51.148			ATTCACGTTTTTTTCACACACATTCAGGATAATGTTTGGATTTGTCCTGTGTATGTTGCTGTTAGGGATGGAACCGTTCGGTTCTCCACCCACGGTTCGGCACGCGCTTGCAC CGCGGTTCATCCTAAATCTGACGACGCATCTATAATATGGTTTGTTGAAAACAACGTGCAAAATAGACAGACGGATTCGGCTAATGTATGTTACAGTCGATGGATGTGCAT TCATTTCCCAATACGTTACAACAGCGATGATCAAAAGAACATGGACAAAACAGTCACTCTTTGTAAACAAATGTTCAATGCCGTGCCGAATGCTCTATGAGCAGTCATTTA CGCCGTCAACACCCGGGTGTTGATAGCGCGCGAGCTCAGGTGAGACCTGAACATCTCACGTTGCTATCTGTGTTTAAACTAAACTCATTTAAACC
LG13	ref-66424_32	51.148			ATGTTACAGTCGATGGATGTGCATTCTGTTT
LG13	ref-42672	51.253			TAATACACTATTTATAAAATTATGTTTTGCTTTAAAACGTCTTATTCTTTTCCCCCCAGATTTTATGCAAATCTATTTACTGAGCAATACATTCAGTATCTTTTGATGGCAGAT GGGGGCAGCACTGACTCTCTTTGGTTCTGCTCACCCATCCGTGAAGTGAAGAATGAAATGTCAAAAGTTGGAGATGCACATCACTGCTTCAGACTCCGAGGGTGTTCCTTT GCTAGTAAGTGCTTTATGATTAAGATTTAAGATCATTGACCAGTGTTTTTCAGATTAAGCTTGTAACCTTTAAAGTGAAAATGAGATAAAACAATATATGCATTTACGTTAA AATCTATATATGTGTATAAATGGAAAATAGGGCTGGGTGGACAGTATCGATTTTAATCGATCTTCATCTTAATGATTTGTCGTCGGTTGCATGA
LG13	ref-10232	52.129			GATAGTTTTGCTCAGTTAATAAGAGGTGTTGATTCAGAGTGACATTTGTTCTTCATTAACACAGATTTTTGTTTATTGAATGCAGTGAATTGCTGTAATCCCCCTTATTTCAG CACTGTTAACCGCCTTACACGTGGTCAATATGTTGAACAGGTATTTAGCTGTTTTCTGGCACATCAGCACGGTCATAGTGACTTTATCATTAAATCGGTCATAGTGACTTTG TGATCCTTTTGTCAACATCATATGCTCATGCTATCCTTTTGATGATGAATGTCGAGAGGACACTTGTGGGCTGGAAATAAAAGGGAACACTGAATGTAAAAAAGGGGGTGT TGTAAAATATGAATATATGATGCTGATATACAG
LG13	ref-26692_17	52.291			GTACTAATTGCATATAACTCAGCATTCATAGGAGTTGGGGCAGCAGAGCTGCGTCGTGATGTATCATTGACAGCATGGAGTGGTATTTCCTGGCGTTTCATGGGACCTCCA CAACAACGCTGAGAATGAAGACCCTTCACACACCGGACCACAACACAGATAAATCCATCAGCCCATCTGCACCG[C/T]ACTCGACTCAAAACACATCACTCATTGACATGG TAAAGGCTACTGTATATGAGTGCACTAAAAATATGCTGTTCTGGAGTTAGTTAGATACTCTGTATAATGCAATAAGGTACGTTGACTCATGCTGATTAAGCCATTTATTTAT ACAAACATTAAAATGTACACAAAACAAGTCTTTCATATT
LG13	ref-37735_27	53.653			CCCGTGATAGAAGATACAGTAGTTTACTATAAATTAAATAGAAATTAAATTAAATTAAATACAGTTTATTGTGTAAACAAAACGAAAATAATATCCAGGAAAAAATAATA ATTATAAACCATCCAGAAATACACCGTGACTCTTATTTTGAAATGTCTACAGTCTGCACTGTAGCAGTAGCATGATGCTCGTTCA[A/G]ATGAAGGAGATAAAACATGCATT CTTACAACCGTCTAAAACATACTACCATATAAGCATCGTCATAATTCATCAACATTAGCTTATAAGCAATCGCCTGGCATAAATGTGCGCTATTCATTTATTGCGAAGCAGT CTGAAGTGCTGCTGGACGAGCCTGGACGGCGCACAACAG
LG13	ref-51979	54.619	Chr13	7527747	CCTTTATGAACACTATACGAAGTGTTATATTATTGCTATAGTGACTACAGAGGGCAGATCTACTGAAAGCCAGCGTTCAAGGACAATAGGTCAAGAAAGCATATGAGATA CTGGACATGACGCAGGTTAACACAATATAATCTCCAGTTTTGCAATGGACAAACAATGGAGTTTCCGTGGCACTCATGTTGTTACTAGGGATATCCTGCTGATAGAAATAC CACTTTCTTTGAAGCAAAAAATAAAAAGATCGTTAATAGTATCAAACTGTCAAAAGAACCTTGACATTCAGACATCAATCTGACATCATATTGTACTGGTATATGCCCTGA AACTGTCATTTCCTTTTACACACAAAAGAAGAGTTTA
LG13	ref-25070_26	55.171			TGTCTGGCACCGACAACCATGCCAT[A/G]TTCAAA
LG13	ref-51686_8	56.171			CCTTCTTTCGCTGATCAAATAAAAAAATAAAAAAATTGACTGATGTTTATTTATTTCAGGCATCTACTGAACTGATCTATCTTATAATTTTTTATAATCCATTCCCAAACAC AGGGAATGGATTAACCAGGCAATATGCCGGGACTTTCCTGATCCCAGATGGCTTTTACACTCCTTTTATTGAAGGGTGTGGACAAACTGATGAC[C/T]GACGACTGTCTTGC ACAGCACAGAATTGCAAAATGAAAAGCTGAATGTATCTTAAACACTCTAAGTGAACAAATTGGCTGTCATTTTTCAATAAATTATCAGTACAATGTCTTATTTGACCCGTTT GATCTTTTGTCACAGTGGTCAAGTGAGATATATTCATGTATTTTATTTGGACAAACAGTGCTGTATAGAGTAGTCAGTGCTCTGATGCTCCACTTGAA

LG13	ref-69052	57.339			CAGCACCGGATCACAGCACGACACCCTGCTCAACTTCAGATGTCACACGGTTAATGTCTCGGGGTCACCCATGGGTCAGCTCAGCCAGGTGAGAGTCATCGCTAACCAACCAATGTGTCTAAAAACCTTTGAACCAGTTATTCTGTCCAAGTTACTTGAATCAAGGCAAGACACTTCAGGCAAAAGCATCGTTTTTCATGCACTGGTCAGTTTTTTAGGTCTATTTTGCTTCCATAACATGTCTTATTTTAGACTAGTGGAAAGAAAACTTCTAAAATACACATTTAAGTGTTTATTTTATTACAGTTTATTGATTTGCATCTTTAGATTTCAATGTAAAGGCAGTGAGTTTGAGTGAGAAATCTCCA
LG13	ref-36799_14	58.444	Chr13	17338689	CTCCGATGCCGTCAGATGGAAAGGGAGAGAGAGATGAAAAGAAAGAGTCTGATTGTAATAAAAACTGCTCCTCAGATGGATCTGCACCGACCAGCGTCAGCCATAGCAGGATAAGCGTGAGCTGTGCGGGAATTAACGTGGAGGTCAACCAGCACCAGGAGACCACATCAGGATCCAAAGCA[C/T]CGACGTCGGAGTCCTCGTCTGTCACCTCTTCGTCCTCAGCCTCCGTCCTGGGGTCAGGACTTGTAGCCCCCGTTTCTCCATACAAACCCGGCCAGACTGTTTTTCCTCTACCCCCGGCTGGCATGACGTACCCTGGCAGTTTAGCAGGGGCCTACGCCGGCTATCCCCAACACTTCCTGCCCCACGG
LG13	ref-7248	59.553			TGCATGTTTTCGAAGCATATGCCATTGAACA
LG13	ref-41515	62.243			TCCTCGCAGACGAGGGATGTGCCCGAAAACG
LG13	ref-39159_4	62.268			CTG[G/T]ATAGCACGAAGGCACTGCAGATAGGAAC
LG13	ref-67155	63.603	Chr13	13141410	TTTTTGTCGTAATCCTAATCGCCGATTGCTTCTTTCTCTTTTTCTCTCCTGCCAGTCTCATTTAGAGGAGGATCTGGCTCAGAAACATGAGGGTTTTACATCCGGCATCTGCGTCTCAGTGGACGCAGACTTCAGCTCCAGAAAGATAGTCCTTTAACACAAGCAGATTGTAAAATGTTGAGATTGAGTGAGCGGAAAGGAGATTGCATGCGAGAAGTGTGCATGAGACTGTGTGTTGTGTTCAGTCTGAAGGTCAGATGACTGTAGGGGTTGTTGACTAAGTGTGTCACGCCAAACGACTGAAAGCAGCGGGAAACGGCAAAAGAGCAGACTGGCACAGCTCAGGTTGGTGTGTGTGTGTGAGTAGATAAAGGTGCAGACTAATTACCTGCTATATATCAGCTGCATGTATTGATTACTCTGTGTCACAA
LG13	ref-11351_4	64.91			AAACACACACATTTATATAAAATAAAATAAACACATAATATAAAATTATTACATCTGTCAAAGTTAACCTGTGAGATTAATATGTTTAATGGCATGTCAGTTATTTAACGCAGTTAGTGTAGTTCAGATGATTTTCATTCAGTTATGTCTGACTTTTTAAATCAGACTCTTGTTGGTGAATAAATTATGAGGGAAGACCTGTA[G/T]CCGCATCGAGTCATTTGCAGTTTTTGTACTGCAGAGATTTCACTGAAGCTCAAATATCGGCTCCAAATAACACAAATATAATCATATAACGCACATACTATAATAAATCAGTATTATACATATTTATAGCTATTACATATAAAATAAATATAATTAGAGCTATATTCAACTGACACACACACACACACACAAAACAATCTCTATCAGAGTGCTTGGGGTTAGTAAG
LG13	ref-932	66.571			CTTTTCAAAACGAGCGTCATGCGCACTCTAC
LG13	ref-22565	70.851	Chr14	28705986	GCAGAAGTGCGTTCCAAAAAAATTTTTTGGACCCCTACACACTTCATCCCTTCGAAGCTTTCACTCCGGAGGGTAAACCCTTTGAAGGGATTAGGGCATAGGGATGAGCCCTTCCGAATGGAACGCAGGGTGGATGTACTGCATTTGCCAAGTTGTTATTATCATGTGACCTGCCAGCGGCAGTTACGTCGCTTCACTGAAGTAGTAGGTCATCCAGATACGTGTTGTCTGCTCTTTTATCAGTACTGAGAATTTAGACCTATTACTCTTGACTACTACTATTGAGTAGTAGAATTTAGACCTGCTACTATATAGTAGGGAAGAATGTGATTTTGGATGCAGCCCAAGTATCTGCATTATCAATAAGTG
LG13	ref-4388_31	75.451			TCTGACACATGCAACCAGTTATAATATGTCACAATATGTAGGATTTTTAGATTAAAATATCCAAAAACCACTATAACAATGTTATATATTTTGTTGACTTCTGTACTTACATTATCCCAAATGTTTCCATCCTTCAACACGTTTAGTCTTATTGTTTAAATCTTGTTTTCTTGATTCAAAACAAGAACCATGTTTTACCATGCCTAATATCGATCTAGCTGCTGAGCGAA[C/T]GCACAGAGTAGCACTATAACAACTTTCAACACTCAAATGTATCTAATATGATAAAACAGCGCTGCTTTACCCCACGTGCACATGACCAGAAGAAGCGGAAGTAGCGACTGCGGCATAATAAAAGATCCGCTGCTGTCAAGACGTGTGTCGCGCTCTTCTGTTATTAGCAATCGCTCCAGCAGCCTCGTTACTGCTCTCACA
LG13	ref-1216_15	79.298			GCATAATGCGTTGGTTGCGTTATGAATGCGGAGCCGCTGGGGTTTACAGAGTGTCTTTACTTTTTAAGCTTCTTCTATTCCCATGACTCTGATATATTTAGCTGCTCTATCCATTGTTGTCACAAAGCTTCTCATGGGTCCGACCCAGCCGAGAGAAACAAGAGTGTTCTGTTCAATCTGCAG[C/T]GGACTCGAATCAAAAGCGTTGGGTTTCTGGGTCGCACAACTGCTGAGAGGAGCAAGACGTCTTCAAGCTGAAGAGAAACGGACATAAAACCTGAGAGCCTGAGAGAGATCAGTCTGTCCACTATTTCTGTCTTACAAAATTAAAGTTTTTTTTTGCACTGATGCCAAGAACCATTTCTGGTTCCCC
LG13	ref-62975_32	80.02			GGGAATGAGACGAAAGAGTTGCAAGACATTT[A/C]
LG13	ref-62975_23	80.15			GCTCTTCCAGGGCTCTCGGCTCCCGACTGTCCTGCTCTTCCCTCCTCAGTCCCATCAGAAGAGAGGAGCGTCTCCTCCTGCTTCTCCTCAACACCTCCAGACCTCCAGCAAACCCCACAGACGCCTCATCAAACATCAGAGTAGAGCGTCGCCTGCAGACTACAGACGACAAACAGATTCATTAGGGCTTTTCTGATGTTGGGAATGAGACGAAAGAGTTGCG[A/G]GACATTTCTTTCTAACAGCTTCAAAGAAATATACAGTTTTGCAAAATACGACTTCAGTTACAAGAGTGTCATTATTCAGCTCAATTTAGTTCAGTATTGACTCAGTTCAGTTCAATGTCGATTCAGTTTAGTTCAATAACAATGCAAAGTTGCGTTGTCGCAAAGTTTATTGATTATGAAACTAATTTAATTCAGTTGTAAAGCACC
LG13	ref-72362	87.047			TTGTTGTTGTTTTTTGGTGTGTGTTATTTGCGCTTGTATTTTTTTTTTTTTTTTTTTTTTTCCTTCCTTATCTTAGATGTTTTGAACTGTTTAATGTCTTACAGACCCTTTTGTTGTTTCTCTCTCTCTCTCTCTCTCTCAGAGTCTCTGATGAACAACCCTCCTCAAAAGCAGTGAAAAGGGCACCAGTATCACTCCAGAGCGCCAGACGGTGTTTTTCCAGCGAGGAAAACGGTCATCTTGACCCCAATGCAAACCTCTGCTCGGCCCAGCCTTCAGATCGACACACATTAGAGGAAAACAAGCAGAACCAGATCAAGAAAGAACTGAAATGCAAACTGGATGAATTAAAATGCAGAGAAACAGCAGACG

LG13	ref-57773	90.484			TGACATGCCCCGACATGCGTGCTTTTTTCAG
LG13	ref-43887_4	96.867			GAATTACACACACACACACACACACACACACACACATATATATATATATATATATATATATGCACAGAAATAAAAGAGTGAAAAATGCCTACCTGACATCTGAAAGATACATAA<br>TGCTGAGTGTATGGAAGACATTCAGCATTACGAGCTGTTACAACCTCCCTTCTGGAATAATTTAAGGAAAGGTAACACAACATAAACAAAAAT[A/G]ACTGAACGACGCCA<br>CTGCTCAGAACTAGCTTATTAATTCACTCAAGACAACGTATAAACAAAATCAAATGATGTACCATCCAAGAAAATATGTAACATAGTGGACGACAACTAATAAAGAGAGA<br>TATGACATGTAAAGCATAACATAAAGACGCTGCTTTGCTGGCTGGCTCACAAGTCTGTCGTCTTTTATCTGCTGATTTCAATCCGCTAACTCTCAAATGAAAAA
LG14	ref-64417	0			CATCAACTTACGAACCCCCTGCAACCCTGAC
LG14	ref-73378_29	1.769			GAAGGCCAACCCTAACCCTAACCCTAACCCATCATGCCTCGATACTCTCAGGTCAAGGTGCGTCACCTGATGCTTTCCTGTCTTATAAGTTAAAAACGACATACCAGTTTTG<br>AATTCTGCTTTCTCTTTCAGCCTTTTGTGGATGACGGAGTTCAAAAGCGTGAAGTTTCCGTCTCAAAGCACAGTATTCGATTACT[A/C]CATTGATCCGAGAGCAAAACATCT<br>GAGCTCTGGTCAAAAATGGTTCAGAAGTTTGAGATCCTCTGCAAGTTAGAATCGATGATTTTGCTCTGTTTGAGTTTATAAAGGTCTGATTAAACTCACTTGAGCATCAGAC<br>TGATGATCTACATCATCTAGCGATACTTCACTCTCA
LG14	ref-63667	3.146			GTAATGGACACCCCTGAACACCGGGGGATGCCAGGCGAACTGAAGCGCATAGCCGAGTCTGATGGCATGAATGAGCCAGCGGGACGGGCTGGGAAGCGCTAACCAGGCT<br>CCCAGACTCTGAGCCAGCAGGACCAAAGGCACCACAGGCGTACCTGGCGTGGGGCAGCGAGGCAGAGTGCTGGGACCCAACTCGGACGGCATAACGGGACTCGAGACGC<br>GTGCAGCGCACAGTGACTTATAATGGTGTGCCGCGTGATAAGCTGTGTCCCAATTCTAGTTTTTAAGTAGCCTACGGTTCCTTTCACACAGTACAGTTCATAATGATGAAAA<br>TTATTCATTTATTTCATATTCATCATTTATTTATAGGCTACATTATTTATAGGCTACATAATCACAAATAAAGATTTTTGTTACATAAAGCAAACCATGAA
LG14	ref-59934	4.384			TTTAGGAGAGAAAAAAAAAAAAAAAAAGAGGAAATTTGCTAAATCTATTGGAAAAAGTGCAAACTCTATTTGGAATTTTTATTCTTTTCATCCACATGATCCATAAAACAT<br>GAATTAGTGCGTGTACGATGCAGATGAAATGAACAGAGAATCACTATCATTAAAGGTGCAGTAAGTGATTTCTGAGAAACGCTGTTGATCTGAGCACCACCACACTCTTGC<br>AGCCAATCAGCAGTAAGGGGCGTGGCCACTCATGAGGGGGGAGGTGCCTGTGTTATATAGCATGTTAGTGAATTTGGGGGCAGAGCTATGAAGATAAGGGTGTGATTCTT<br>TTTAGTAGAGGCGTGTTTGTTTTGGTGATTTTTAATATCAACTGTGTTTCTCAGAATCGCTTACTGCACCTTTAAAGGGGTGGTTGATTATGATTTCAC
LG14	ref-28953_23	4.605			AGTCACCAAAGTATCATCCCATTCCGGTGAAGCAAAAAATTAAAAAAAATTTTAAACGTAAAATTTTTCATTCATTTCTGACTGAAGAGGCTTGAATTGGTTTTGAACTTG<br>GATGCATGTAAACCTATTGTATTGGAGACACCAAAACAAAATCAGGAACCTTTAAAATAGCACATAGAGCACTTTAATCGT[C/T]TTACCGATAATCTACTTTGTAGGAACA<br>AAATGTTTGAGAATCATGAACATTTAAAGCATTTATTTTAATGGAAAATTATCCTTGCATCTGCACCCTCTCAAAACCCTGATCCTGTCCTGACAGAGCTCTTTATCTTTTGC<br>AGTTGTTTTCTTCACATACAGCCACAGGTCACAGATT
LG14	ref-66508_25	5.989			CCGTCACGAACACAACACACTGGCTGTCTCGTGGTGGCTGGTTCCTTGCTGTCACACACACTTCATATTTCGTAAGCGGGAGCAGGTCACTGACAGAGTAGCTGTTGGTTC<br>CTGGACCCACATGAATGGCGTCTTTCTTAGATGCATCGTAACGGCCGAAATAAACCGTGAACCAGGTTTCTGCAGGTTTCTCAGTCAGTGCAAACCACTCGATGGTGATGC<br>CA[A/T]AGATGGTCTGCTTGGCTATGTGCATGTCTATATACACGCTTTCATCAACCGATGCCAGCGGGAAGGCGGGTGAGGGCGGGAAAGTGGAGTTGTGGTCTAGAATGA<br>CATCAAGCTGGACGCTGACAGAAGAGTTGCCGATGAAATTGTTGGCAGCGCAGGTATAAACGCCTCGATCGGCCACATGAACAGCCGGAATAATCAGCGCAG
LG14	ref-21923	6.772			AGACACCTGACGCTCCAGAAACACAACCCGTGGACTTTCACCCGTAAACATCAGCAACACAAACCTCATCGGTTCAAACAGCCTCTTCTTTTCTCAAACTGGCCGATAGTT<br>TTCTGAATTCTGCTCGGTGTCTTAAGATCCTGAAATCTGGAACTCAATGGAAGGCCTGATGAAGGCCAGCAAGGGGATCGACAGCGCTCAGTTTGCACACGACGGCTCGTA<br>AAGAGACTTTTACACGCTCGTATAATGAGATCTGTCGGGAGAGACCTGGAGGAAACCCAGAGATTTTCCTGATAAACCAGACTGAATGATTCCATGCTTCCATGTTTATCT<br>CTCTGATAAACACACATCTGTTCAGAATAGTATTCT
LG14	ref-21087_18	7.489	Chr8	773074	AAATTTAAACTTTTTGACAATCAGACTCCAGAGAATCTCACTGCACTGGTTTGGTTCCGATTGGGCCAAAAACCTAGGACTAGTTCGCAAAAGTAGTTTTTTTTTTTTATCC<br>ATTCCAACGATATAAGACACTTGATTAAGATTCTCACCTGGATCTGATAGTCCTCACCGATTCCCTCCAGCTTTTGTTTGTTCTCATAAATGGCCTCTCGAATGT[C/T]GTGC<br>ATCTCAGAGCACAGATCTATGGCACGGTTCACCTGAAACACAGCAGGACGCGGTCAACAGCTCATCCGAGATTAATACATATGCTATTTATGTCTTAATGAATACTAGATT<br>GAACATGAGACCAAATACAAACCTCTTCCATGACCTCTTGACCTTTAGGCAGCTTACTGATGAGAGTCTGAATAACCTGGAACTGCAGTTTGGGTTTGT
LG14	ref-24421	7.523			AAACCACCGCCGACACTGATGCTTCACTCGA
LG14	ref-18440_8	8.081			GGAGGAAGTCGCCCTCCAAACGCAGGAACACCACGGGCTCCTCACGCAAACAGAAGAAAATCACCTCCTTTCAAACGAGACAGACGCATGTCAATCTCCACTGCGATCGC<br>AGGACAAATAAAAACACTTTTGCTGTTTAAACTCATTTAACAGTTTCATTTAAAGCGTCAGTTTAA[A/G]CAGCACAACAATCGAGCGAATCAACAGATTTAATCTCTAACA<br>AATCACCTGACAAACAGGATTATTTACACATGGCTTTCCCTGAACAGAAGGTGAACAGGGATCTGCTGGAGATCTCCACACTGACCTGATGACCCTGACCCTGGAGTCTCT<br>GCAGGACCTGTTTGAAGCCACTCAGGCTGGGTTGACCCAT

LG14	ref-5920_9	9.836	Chr12	1874744	TTTCAAGGTTTATCACTGTCGGATTATGATTATGAGGAAATCTTGGGAAGCAGCCACCACCACAGCCTTTCATCTAACCATTTGACCATGTTGTGCCTGAGTTATATTCTGA CTGAGTCTAGCAGGGAAAAGACCCTTTTATTTTGAGCTCTTGCGTCAGCAGTTGCAAAGTACCCTGGCATGATTTGTGAAGCTTTGCCTGGAAACA[A/G]CCGAAGATGTTG CTGGCCCACAGACGGCTAGAGATTAACTTTAGAGTCTAAAATTATGCTGGAGCACACCCAATGCTACCTTATCAAACGAAGTTTGAAACTAAGCATACAAACTGTATAAA GTCAACTGCGCTCCAATAAAAATGACTGACCTGGATTATGCCCTCCTAATAATTCATCTCAGCTTTGGAGGAACCTCATATCATGTGGAGACTTCATACGA
LG14	ref-26347_18	10.306			GGCTAAATGACGACTGT[C/T]TTGCTTTTCTGCAC
LG14	ref-26347_16	10.315			GGCTAAATGACGACT[A/G]TCTTGCTTTTCTGCAC
LG14	ref-72716	11.96			ATGATCTAAGCGATCTAAGTGCACTTAAGGC
LG14	ref-2531	12.395			ATAGGGTAAGCGACGCACGTGCACCCAGCCA
LG14	ref-14161_23	12.826			TGTTACACCAGCAGTGCTGTTGGTTTGTTGGGTAAGATGATCGCTTTGTTACAGTCGAGACTAGGCCATATCTGGTCCTGATTCTACCAGAGTCACACTCACAGGATATCTG AGGTCACCTTCATCTGATCCAGCTAATCACGGTCGAAACGGAGGCTGGAGAGGCTCTTAAATTAACTGCAGGTCCATCG[A/C]CAGATGCTCTTCAGCATCACAGCGACTCT CTCGCCTGCAGACGTCAAGCTGGGCGAACCGAGCGCTGCGGTCTTGAGGGTCGTTTACCTGGAAACGAGTCAGGAAGCGTCTCTAATGAGGTCCGGTCTGAAAACTCAAT AACCCAATCAGAGCTGTGGAGCCGCGGGGAGCCGCTGTT
LG14	ref-66902	12.963	Chr12	2533312	TCACATGACTATGGATATAAACATGTAGAAGGGGGAAGCACAGGCTACGGCTAATGTCCAAATGGGGTTAAGTAGCTTGATAGGCATTTGTACTCAATGTCACAGAGTTT AGAAGACAATACCATCAGACACAAAATCCAACACTGAATCCATTATTCTGTGCTCACCTGCTGCTACATGCATGTCCTTGGGAGGTCAAAAGAAGAAACAGGTTAAAATG TTGTATTACTATGTCTGAATATACAGGGAGGAGTGAATACAGGTCTCCGTTACGGAGGTATTTACATCAAGAGTCTGGTGCCCGGTGGAGTGGCCGAACAAGATGGACGA ATCCAGACAGGTACATGTTACTGTTCTTACCATCATGGA
LG14	ref-61395	15.959			AGAACCAAAACGACACATTTGCCACAATCAA
LG14	ref-23022	16.089			AACCTACAAAAATTTAACAAATTTGCATAATGCCAGCCCATTGTCTGCCCGTTAACAAAGTCTATGTTGCCCGCCCTCAAAACACTGTAGTTGTATCTGCGACTGGAAGAG TTCGGTTCGTGTTGTTCATGTTGAGAAGATGCTGTTTTCAGCGCTGCGAATCCACTTTGATTTCGCCCATGTCTACTGGCTGACATCTCGGACGTGTATTGAATTTCATGCAG TTTGAAGCATGCGAAGAGTCTCAAAAGCATCCAAAATGAATATTAAAGTTTGATACGTTGCCATGGAAACGGTGTTTAAGATATCAAGAATCTTTTCACAGGTCTACATTC GCCATGTCTTGACATTATTCTGATGAAGTTTGAAGCAAAATCGGGTAAAAATAAGAGGGTGATCTCAAAGCATTTTGAAAGTGACACACTTCCTGC
LG14	ref-54167_7	19.267			TGTCCTCTGGTTTTGATTATCATTCCAGCACCGCTCATGCAATGAAATGTCATGCATATAATCTGATAATCGTCGGCTGCGTCTCGAAAGGTCATCTTTGAGCATCATGTTA CGACCCTTGACTTGCGTGTGGTAGCTGAAGCATGTTCTCATGACGCAGCACATGTGCAACATGCAGGAGGAATTTCAGTAACCTGTGACAGCTGC[C/T]GGCGATTTGCATG CCACATCAGTATAAATTTGTGCCACGTGTTTAGAGTTTGATGCATGCGAGTAAACGAGTGAACTGCTTTATGACTGATTCAAATTAGTTTTATAACTGACAAATTTTGCAAC ATCGACACTATCATTGAACTGAATTAAATCAACACTAATCAGAACTTAATTAACACTGAACTGAATCTATATTGAACTGAATTGAATCAACATTGAACT
LG14	ref-71018	19.958	Chr14	34361093	GTGGATTTAAAGATACATGCACAAAAACAGCATGTTTTTCCTTCCAATCAAAAATGGGCATTTACAACATGGTATAATAAATGATCTTTGGGGTATTTTGAGACATATTCTG GGGACACCTGAGACTTATATTACATCTTGTAAAAAGAGGGCATAATAGGTCCCCTTTAATACAAAGATTTTCTTTTTTATCACTTTTTATCATTTTCATGATATATCTGCATG AACGTCCAGATCTGACACAGTCTGACCGTCTTTTCCAGCCGAACTACAAAGATCTGATTTCCAATTCTGCATATTACAGTTGAAATGAAGTTCTATAGTGACCAGAGCACA GCACTGAGATAATACATTAAGTGAAATATGTTTTTATAACTTCAGTGCAATATATATTACATGTATATCATAAAATTGAGCTTCTATTAAACAGA
LG14	ref-67571	24.09			TTCTTTCTTTTCCTTGAATCCCCCATTGGCCAGCACTGTAGCATTCTTATTTGTTCTTCTGTTTTCTGCACCTTTGTCTTTCTATTTCCTGGACATAGACTGGACTCAGAAAAG CATTCTACCATACTTTGCATACTATTTTGTCTGAATACACTGCATAGAATTCTGTAGCACAAAATGCAATGCACTCGACCCAAACATTTCCACTTCTATTTTTTTTAGTATTT TCAATATTTCCATTTTCAACCTCAATCTACAACATCATCTTGACTTAGGCTATCATTCGATTGTAAAGAAAAGAGATTTTTTTAACAAAACTAATTTAAAAGTGCAACATAT AAGTGTTTTTAAAGTCAAAATGGTAACTTA
LG14	ref-73299	26.202	Chr12	47714288	GATGATTGTCTAGTAACGTTAGTCCTTTCCTTCGGGTTTCATGGCGCCATGACATCCAATCCTCCAGGGGGCGCGCTGAGGCTCAGGAGCTCAATCCTCCCTTTAGCCGCAT TATATCTCAGTTTGATTTTACTCATAGTGGAGATACTTGCGCACTATAAAGTATAATCGTTTAAGTCTAACAATGTCAGCGATCAGCACAGAAATCTGCGACAGCCGTGTCT ATAAAATGATTATATAAAGCGCAGCATCCAATAATTGCAACGGAATAATTGCAGACGGCTGTGATTGGTCCATCCTGAAGAGCGCTGAGAAAAGTAACACGCGCGTCATG CGCTTCTGCAGATCTCCACAGCGAAAAATAATAAATCTTTATTTCGATCTATTTAGATCCATCATACCTGACACATAAGCAACAAGATCTGGGCCAC
LG14	ref-34906	29.936	Chr12	48731774	TGCATAATGCTTGGACAGTTTTTACTCAAACGTTTCCATAGCAACAGCTATTTCACAGTCATATCTTTAATGAAAAGGTAATGTGAGACATAGAGTATTCCTCACATCATTA CTGCTGTAAACACACACACACACACACACACACTAACACACAAACAGGTAAATTCAGTGTTCCATTCATAAAATGTCTACACTACACACAGTAGTTTGAGTGAGTAGTATGCT AGTATGCACAGTGTGTCTCTTAATATTGGTGTGTGTGCGTCGCCCTCTGCTGGTGTGTTTGACATCTTCAGAAATTACCTTTTTTTTTCTAACATTGTGAAGTTTCTATTTCTA TTTCTGCAGCACTAAAGAGATGAAACACTCATTAATACTGAGTCCTGAACTACAAAAAATGGCAAACAAATTTAACCTGTAAAACCAGTAAGTG

LG14	ref-43478	32.673	Chr12	48686824	TAATTTCCAACCTATTTTGGGTTCATTTTAAGCCGGACATATAGTCATTTTTAAACAATAGTTGAGTTAAATAAGACTACCCAGCACATTGGGCAAACATTTAACCCAACCACTGGGTTAAAACAACCCAAGCATTGGGTTTGTCCATATTTAACCCAACTTGGGTTGTTTTAACCCAGCATTTTTTGTTAATTTTGATTTTCTGGCAGCGACACTGGTGCTTCTTGAAAATATGAATACGTAACATAAATTTAGGCACAAATGCATCATGTGTCGGCAGTGAGCAACACGCACAGGCTCAAGGTCAGTTAAAAACTTTCTATATTCCTTAAGCTGCGGCAAAGAGTTTCCTACATGTTTATGGAGGCGAGTCTATTCAGGAAGTTCAGCTGGGATCACAAACACTGAATCCCACTAGAAAGGGCATATA
LG14	ref-54990_19	33.433	Chr12	40009457	ATGAAGCTCAATTTCACTTGTCATTTTGTCTGTGCATAAAGAGAGAAACATAATGAGGGACCTTCGAAGAACGAAACAAAGTGGTGTGTTTCCTCCATCCAATAGTGAGCATCTGTGCCTAATGGAGTCACTTAGTGGTCACTCATTCTCAAGGTCGATTTGTCAATCAGAGTGAAAGGGCATTGAG[A/G]TCGTTGATAGCTCATCTTTTAAAACACTCTCCAGTCAGAATAATTGTTTCTGTGCATACAATAGCACAGCAGTCGGGACGACTCCTCTCAGCATGACTCATCCTTTATTTCCTTCCCGGAAGGAAAACAGCAACCGATCACTACACCTCTCCATGTTCTACCATCTAAACTCGAAAAGAAA
LG14	ref-64176	38.521	Chr12	38487288	AGACAGCAGTTGACTAGTATTTGTCATGCTTTGCCACATAAACAAGCTTTTGATAGCGATGGACTCACTGTGACCCGCCTCAAGTGCATATCAAAACCAAATAACATCACATACACACTATGATATGTAAACACATGCAGTGTTTTGGATGGAAATAGTGTGTTTGTCTTGAATTCTCCATATCTCAGATAATGGTCAGAACAGATTCATCGACAGGCTTGTTGGCTGATACTCACCACTGCAGTGGCCACATCAGCTCCGAGAGACGCCCAACTCAGTATCAGAGTCAGAACCCCAAACACCATCATCCTGCACACACACACACCATAAACAATACAGTATAATAAACAATTTTGGTACCGTGCTGGGTCACCACTTGACGCCAAACATAAAATTCAGTTGAGAAACCAGTTAAGAATTAGTATGACAATAT
LG14	ref-41968_8	41.173			TCAAAATTCTTGTGAATAATTGGACCTGTAGCTGGCGTCATGGTATGATACCACATACTGTACTTCCTCAAGCATTAATGATCTCGATTCAAGACAAGTGCTACACGGCTCTAACGTGGTAAGGGGGGTGCAAAGCATCTTCTGCAGTCTCATTCAAACCGCAAAATGCGATGAGCCGTTAGAAGTGTGTTTGTGTATTTCAATGCTC[C/T]AGCGACCAATTTGCTCCATTTTTGTTCCATTTTAAATATAATTTTTGGGCTTTCTGCCCAAATAAATACAGTAAGAGGAGAGACATTAGGGAGAATAGAAGATGGGATTGGAAAGCAACACAAACTAAAAGTTAAAACACAAGAGACATTAGGGAGAATAGAAGATGCGATCAGAAAGCAACACAAACTAAAAGTTAAAACACTTTTTGAAATGGTCAGCTTGG
LG14	ref-62005	44.418	Chr12	36772499	TAATCCACCTGCATAGACCTCCTACAGCGCAGCTGAATTAGTGCTGTGCTACTGCGAGGTTAAATCGCACAGTAATTAAGCAGCTCTTGGGGGATGTTTCCAGAAGTTTGAAATAGAGCACCATTTATCTGCTCACCTCTTTATCTTCTGCTCCAAAATGGTTTTAGAAAACGAGCCTGGATTTAGGCTGAGGAATGTTGTGTTTGAAAGGGAGTCAATTGCACGGCTATACGTTTTACAAACCAAGATAATGCATGACATAACAATAACCTGCAAACCTAGATCACACAAGAACAGCTTAAAGGGCATATTGCAGGTTAAAAAAAAAACATAAAAAAATTCAAGATGAGTATATAACCTTATACGAAAATACCATATGAAAAGGTACTGCAGATTTTAAAAATGTTTTGCAGGTCACAAGGGCACAAATTT
LG14	ref-32491_31	46.06			AGAGAACCACCGACAATGTTGCCAAGTCTG[C/T]G
LG14	ref-46692_1	46.517	Chr9	32060932	TCTGATGAGCATGTGAACACAATGGCCAATCAGAGGTGTTTACGAATCCACTCAACAGCGCTCAAAGCGTCACCTTTTTAAAATTTCGGTCCTGAAGTCGGTACTTTTGACACTACTTTGGAATATTTCCCTGAAAAAAAAAAAAAAAAAAAACTTCCTGAAAAAGTGGG[C/T]GACACTGTTGCACTTTATTCGCAATGCTTCATGCTAATGCTAATTGGATCAATGTAAATGTGTTTTCTGGTCATTTTTGCAAGCTAAGGGATGAGTTAGATGAAATAGAGCTTCGCACTGTGTCCTTAACAGACATAATGCATAGATGTCCGAACCAACCACCAATTTTAAATTAAACCTACTTGTCACTAGAAACATCT
LG14	ref-44346_23	46.809			CCACTCCTCTCGAACCACCTGC[A/G]GGCTTGAGC
LG14	ref-35029	47.593			ATACAGTGAACGAAGAAGTTGCTGTAACTCA
LG14	ref-5632_9	48.077	Chr2	42780123	AGCATTCAGTTTTTCCGCTTGCAAATTCCGCCATGTAAATAGCGAATCCACCATAGCGCGAGTGCAACTGGCTTTTTCAAAGCGAATGGGAGATGAGACTCTGATTCGGTTTATTGCATGTTACGCCCAACACACACCCATGACTCATTAAGAGAATAGGGACAACCCTTTTAGACCA[A/G]GCACAGGGCTCGCCGACCATTTTTTCTGTCTTCAAACTAGCAAAAGTGGATTCGGACACACCCTAAGTGCACTTGCACCGTGAGCTTTAGACCATGCGCTTAGATCGTTAAAATAGGACCCTAACACTAACATTAACATATTAGCATGTGAACCCAATGCCAACTCAAAGAGAGTGTTTTTGATTGACTGC
LG14	ref-12636	49.225			TGATTCAGCACGATTGATGTGCACTCCTGAC
LG14	ref-8419_26	49.76			CCGCAAGTACCATGTTTTACCATGCCTAATATCGATCTAGCTTACTGCAGTGTGCATTAAGTGTCTCAAAGTAGCCGCCTAACGAATGCACAGAATAGCATTATAACAACTTTCAACACACACATGTATCTAATATGATAAAACAGCACTGGTTTAATCCACATACACATGACCGGAAGAAGCGGAAGTGGTTGTCTGCTCCACTGCTTACGAGCCGTGTGCCGCG[A/G]TTGTCCTCATTAGCAAACGCTCCAGTGGCCTCGTTCCGCTCCAACAGCTTTCAGCCCCACCCTGCTTCATAGTACAGTAATGTTAATAAATCTTTAATACATTAGCTCATCCATGAACATGATTTCTGCCAGAGTCCCATCGGACTCTTTTCCACCGGCTGTAGACATGAAGACAACACCTTCCATGATTCTACGAAATCAAGGC
LG14	ref-26811_32	49.854			TTTGTTGGTAAAATGTCTCGGTTCATTTTCTTAATGCAAAATATCATTTTGTATTTTCATTTTATTTTAATAATAGGGTGAAAATTGTTTTCATCAGATGTTTTTTATAACACTATTTTAACTCTCTTAAGTTTAAGAATAGTAATAAAACTACCCACCAGGGAGCAGGCCGGACATCAGCACTGAAGTCGACTGGTCCA[G/T]CTTGTTTAAACAGAATGTAGGCGTATCTGTGAAATCCTGTCCCTCTGGCAGGAAAAGGGCTGATGTAATGACAGATCTCTTCACCTGACGTCACAGAATTCCCAGGAATATTCCCACTGTATTGAATAAGTAGTTAAAAACTGATATCACCACAATTTGTCTCAAACAA

LG14	ref-43767_30	49.903			TCCAACATGACAGCTGTTCCAAAAGGTAACCAGCAATTATATGCTGGCAGATTTTAAGGCAGATTTTACGCAGCAAAGTAGTTCACTAGGTTTTGGATGTATTTCACAATTT TACCCCGTCTGACTCACAATCAGACGGCCTCATAGACCTCAAAGTTCCTCTGTTCTGGACTCATGGACAGACAGCGCTGTCTCTCTCGCAGAATGAACCGAAGTGTGTGCG CGCGCG[C/T]GTGTGTGTGTGTGTGTTACAGTGAGCAGGTCTTCCTCTCAGACTGAAGTTGTAGATTAGTCATCTCAGCTCTGCTTTATGTCTGATACTAGGGTTTCCCACTCAC TGGACACACTGGGAAAAGAAACAGACACTGTGGACAGATAACATTGTGAAATGTCCTGGTCAGCACATTTACAGACTCTCTGAAACACTCAAACAAACA
LG14	ref-10724	50.507	Chr12	41260157	AAATGGATTCGGACACGCCCTAAGTGCACTTGCGCCGTGCACTTTAAATCATGTGCTTAGATCGTTAAAATAGGGCCCGATGTGTCAAAATGAACTGAAATGTCCTGCACA TTAATTATAATGCAATCTGGAAAATGCTGTTTACTACTGACCTCAGTAACAGTTGGCTGTGGAACACTGCAGCAACTTCGCACTTTACTCTGAATGTCCTGTCTTCCAAAAA GGTGACTTTGTCTGATAGCATAACCAGTGAGAGATGAAAACAACATAGTAAATTATAGGCAGCATGTTTTGAGACACTGTTATATAATGCAGTGAAGTCATAGAGGCAGG AAAAAACTATTTGGTGTCATAATACAATTTTCAATT
LG14	ref-52806	51.064			GAGTAAAGTGCGAAGTTGCTGCAGTGTTTGG
LG14	ref-24949_15	51.211	Chr12	27153484	CATGGAGGAGGCTAAGGTGGAGTTGCACAAGATCACTCGTACGTCAGAAAACCAAGGAGTACCAGTGCTGGTTTTAGCTAACAAACAGGACCTTCCAGTCGCACTGCCTG TTTGTGACGTGGAGAAGGTTTTGGCTGTACATGAACTGAGTGCCTCCACCCTACACCACGTGCAAGGGTGCAGT[C/T]CGGTCGACGGGCAGGGGTTGCAGCTGGGTTTGG AAAAACTGTATGACATGATCCTCAAACGAAAGAAAATGGTTAAGCACAGCAAAAAAAAAAGATGAGCTGACTGTGACAGCCTCGGTGGACCCTGAGTTGTTGTCATAGG ATTTTTGCGAATCCCTGAATGAAGAAAATGCTCCAGATGGCCC
LG14	ref-32138	51.834	Chr12	31531631	CAGAACAGTCACAATATAGAAGTTTCCAGGCCTGCATGGGTTTTGCAATAGTAGAGAGGGGCAGGGAGCAGCCCACCTCCCGCTTAACCCCCCCCCTCCACCATTGTTGGC ATGAAATCGGACCTGTTCAATAAATAAAAGCAGACGGTCAGCAGATCAGGTTCCAAAGAGGGAATGCTGCAAGAGAATCGAAAAAGAGGAAAGGGATAGTTAAAGTTTT GAAGGGAAGGTCTTGGATCCAAAAAGGGATGAGGAAGACTGTTGACGTGTAGCCAGGGGAAGGATGTTTTCACTTAGACTCTGTCCAGGATCACAGCTACTTATGACAGC AAATGATACAGAAGAGAAAGGACAGAGAGGCAACATGGA
LG14	ref-55516_23	51.879			AGCCATTCACTTGTTCCTGAACAAATCGTTTGAGTATATGATTATAAAAACAGTTATCTATGCCACCTACTGGCTTAACAATATAACCTGCAGAAAAAGTCAAAGCACAAT TATTATATTATATTATATTATATTTGTAGCGTCCTCTTCCTGTCTGTCTTTGAATTATGTAGGACAGCGCACAAGGTTCG[A/G]TAAATATGTTGAAGTTAGGCCCCTCTGAT GTCACTAAATGAAATACTTCTCCCTTAGAAAATTACTTATAATTAAAATCCATACTCAAACAAGATTAAATTGTCGAGAATAAAACTCTTGGATAACAAAATGTAAATGAT TCAATTAGAATAAAATCAGAATAGTCAAAAGTCAGAAT
LG14	ref-22915_14	52.018	Chr12	23397584	TTGTTACATTCTGTAGCTGATTGCAGACTGGACTAGGGTGTCACATGACTTGAGTGATTCGCTCTCTTCTAACCACCACAGCCCCCCTTCCCATCACCCCACCGTGGCCCTC ATCAGGTCATGTCAGCTCGAGTTTGTTGCTCCTGTAGTTTTTAATTCTGTCTGATAGCTGAACAAAAGCAGAGTGGAGGAACAGAGGGGGAAAGAGGGCGAT[C/T]ATGGT GCATTGTGGGTAATAATACAGCTTGTAGTAGGAAAAAATGGGGAACAAAAGCATTTTGTTATTATGTTTTTTTTATAGCCTGTCATTATTGCAACTCTTTTTTCGTTTTTCTT TCACTCCTTCTCACATGCTCTTCATCTTTCTTGTCTTGACTGAATGGATGTTGGTCCTTGTGGTTTATTTTTTTTTCTTGCTACCCATTCATATTACA
LG14	ref-66729	52.034			GGCGTTTACACGAACAAAATGCATGTTACAT
LG14	ref-36023	52.624			CTGTCCTCTGAACTTCCATAACTGTCTGGTTTGGCTCAGCTACAAAATCAGACATCAGAAGACTACAGAGGATGGTTCGGACTGCTGAGAGGATTATTGGTTCTCCCCTGCC CACCCTCCAAGAACTGTATACATCCAGAGTGAGGAAAAGGGCTCAGAAAATCACTCTGGATCCCTCACATTCAAGTCACTTTCTCTTTGAACTGTTGCCGACTGGTCGGCA CTACAGAGCACTGAGCACCAGAACAACCAGGCACAAGAAAAGTTTCTTCCCCCAGGCAATCTACCTCATGAGCAGTTAAACGTTCTCTCTACTGTGCAAAATATATATATA AAAAAAGTATAGTGTAAAAAAGTAAAAATGTGTACAGTTTTCTGTGATGGGTAGGTTTAGGTGTAGGGTTGGTGTAGGGCGATAGAAAATACGGTTT
LG14	ref-56625_18	52.952			AGGACTTTGCCGATGTG[C/T]TTGCCCTGAGCCAT
LG14	ref-31449_31	52.981			CTACTGATGACGAAATTCATGCCACACACA[C/G]T
LG14	ref-31449_30	52.988			CTACTGATGACGAAATTCATGCCACACAC[A/T]CTGTAACGCTGACCCTTGCAAACGCGTAAAAAGTGAAGTATACATTGGGCTTTAGCCTCTTGTTATTCAGTGTATATTA CACCTTTCTTTTAGCTTTCTTATGTCTGTTC
LG14	ref-65708	53.101	Chr12	38273244	CTACTACATTGCACCATGTTGACTTTCCACTTGTAATATTACTGACATTTTGCGCATATGCCCTCACGTTTGCATCCTGACTTTTATTTTATTTTTTCTTTAGCCCAGTCGTGA CTATAAAATCAGTCCTGTTGGCCCTAATCTAATTCTTCGACACCTAAATAAATGGCCCCTCTGATAGGGAATATGCAGAACTATCTGTCAAGCAGACGAATTGGCTACTGT GGAGGTGAAATAAAATACAGGGCCCTTTTTAATCAATGACAGGCAAAGGGGAAAAAAGGACCACACTCTGTTGTCCCTAATGTGTCCCTGGCTAATGGCTTGACAAAGAC TAAACAGAACAGTAAGGTGTAACTTGGCAGAGGAAGATTAGCAAGCTCCATCACATCACTAAGCAATGACACTGCATGGATACATGGCTAAAAGTA
LG14	ref-39081_31	53.203			TCGATCTTGTACTTTAATACAAAATAAACAAATACAAAGCGAAAGTAAAATGGCACAAAAGAAGGAGGCGGGAACCAGCGAACGTTCACAAACTTTAATACACAATAAA CAAAGCGAAAGTAAAATGGCACACACAAACATAATAAAAACATAGCATAAATCCAGGTCTGGTCCTCTCTTGTCCTTAACTGTCGTCACTCCTCCGTGATACGAGACCGGT GCAGTGCGCA[A/G]GTGATGCTCCTTAGCAATCACGCCACCGGCCTTGCACTGTTCTCTCACGGCTTGACACAACAATAGTTATTTAAACTGTAATAGTAGTTCACAATAT

					TACTGCTTTAACTGTATTTTTTATCAAATAAATGCAGCCGCAGTAAGCATAAGAGCCTTTTTTAAAAAACATTTTAAAATCTTACAGACCCCTAACTTTTTGG
LG14	ref-45783_30	53.759			TCCACTCCACTGAGAGGAGGAGTAGATACAGAGGGAATGGTGAGAGACGGTGACAAGTGGGTGGCAGAAATGGCTGAGGGCTGAGGATGAGACTGGAGTATGAAAAATAGTCTGATGATAAAGGATAAGTCCTCTATGGTGGTTAGGAGAGGTGGTGGAAGAGGGAGGCTGGGTGGGACTGACGGGACAGATTTTTTTAACACGAAGGTCCGAACTTTGTGCTGCCAGA[G/T]TCTTTCTTTTGGTGGCAGGAGGTTGGACAGGTCTCTCCTTCTTGCCCTCCAATTCCACGATGGCTCCCTCAGCGGTGGATGGTACACCTGGTCAGACTCGTGCATCTCAGGCTCCGTGGCGTTGTTGGGCTCACGCTCCCCTGATGGCGTGGACTCGCAGCTTGCGGGCTCAACATCTGCGGTGGGCATGAAATGCTTTATC
LG14	ref-41317_32	53.987			GACAGATTATTAATCAAAATGTCCTCCCAATCTAAAATCAAAACTTGGAAAATTTAACAGTGATATGACTCCTTAGATGTTATTTGATGTTACAACATTTATTTTTATTTTTTTACCTGCAAGAGTCTCCTAGGACAGTGCTACTCAAACTGAGGTACACATAGGCCTACCCCTGGGGGTACGTGAGGTGACAAAAGGGGGTACGCCACGCGAACAAAATGCTGAGTAGTT[A/C]ATTTAACTCTACCATACCTTGAAATAATATTAATACCGAGCATGTATTTCTAACTGGTAAAATGCCGTAAACCCAGTACATTTAATCAAATTACCTCATCTTTTGCAGTCAAAATTGACTGACGTGCAGTCGCTGCCTCAAATAGCGCTAAATTACTACCGTTCTCGACAATGCATCTTTGTAAAAGTGGGGATTTAGCA
LG14	ref-37202_2	54.215			AGCTTTAAGGGGTTTATTTTGGAATAACGAAATACTGATTGAACATTATCCCACTTATTACACAGCTTCTCGCAAATATAAATGTGTGGACATGAAATATTGATTAGAAATTATTGTATAATCATACCTGTATATTATCTTAAATCTTCCGTTAAGGAATAAAAAAGTAC[C/T]CTAAACATGCAAATCAATCGGCCTAGCAGTAAGACGAACACTGCAATTAAAACTGAAGTTCCTATTTAAAAAAAGAAAAGAAAAAAAAGATAACTGAGACTGACGATTGAGTAAGTTGCATTTGTTTGAAACAAAAGAGAGCAGTCAAATTAGCAAAATTTCATGCAGGGGCAAAAAAGATCCATCATCGATTGTCAAT
LG14	ref-8834	54.404	Chr12	21546436	GCATTTTGGCAAACTATGGGCTAGATGATAGAGTGAGTGATGAATTTGCTCCATAAAGTTTGATTTGGCGCAATTTTTTTAAATTGTTATTTAGTCAAATACATATGTACTACTGCAATGCTTTACTAGCTTACCTTCCAGCTAGCACTATCCAGCCTTTACAGCTGATCCAGAATGCAGCAGCACGTTCGGTCTTCAATCAGTCTTAGAGGAGCCATGTCACAGCCCTCTTGGTTGCCAGCATTAAATATAAGTCTCAGAAGGTAGCATATAGAACCGCCTACAGTTCTGCATGCTCTTACCTAAACACTTTACAAAATTTATGATCCTCCTTATTCCCTGCAGTCAGTCAAAAAGTGTGATGCCTGG
LG14	ref-63963	54.662			AAATCGTGTTCGAGTCTGCTGCGTTGCGAGA
LG14	ref-30367_1	55.039	Chr12	30618106	AAAAGTATATTGTAAATGGTAGAATGATAAATAATGGCATAATACATTATTTTCATGTGTGTGGATTCACATTAGAGAGTGTTGTCAGTTCTTGGGAGATGACGGGCTGTCTCTGAGAATGCTGCTTTCTCGCGTGGCCCCTTTTGGAGTGCTGTATACTCTCACCAGCTA[A/G]CTTTACCTGCAAGGCCTTCGCAGAATCCCCGCCACTGATGCCTGTGCCCTTTTCTGCTGTAGTCGTGCCTTTGTCTTCCTAATATCCTGGATTGTCCTGCGGGATCGCTTCATGGGAGTGCGGGTGAGTCTATGATGTGTAAATTGTCATATAAAATGCTTTTATAGTGACTATCCTGTTGTAATTTAGAGTCTACACT
LG14	ref-39225_23	55.148			TATGTGTATGTTTAATGACATGTTAAGAATATTTTATCAAATATTTTGTGTGCATGTATATTCATGTAGAATATTAATAAGCTATACATCTATAATGTAACTCCATTCAATTTGAAACAAATATGAAGAAAACATCACAAAAAAACTGGATTTTTAAGATCAATGAAAAAAGCAAAGCAGCACTGGTTTCG[C/T]ATAGACAAGTGACTGTTTCTGTCTGAAACTCTGCCACACTGGCCATCCCTACTGAGCCTTGATTGTGTATCTTAAATGACGCCATAGCCAAAGCTGTACTTATCAAAAGAGTCTTTCTTCTATGAGGGAAAAAAAAAAAAAAAAAAAAAAAATCCACACAACCGCATACACAGACTGT
LG14	ref-16620	55.227	Chr12	31299874	CCCCCCTGCATAAACAACACCTGCAATGTCGCCGGTGACATGAGACACTCTTCATCTGGCTGAAGGCTGCTTGGTGCTAACGGTGTGATCACATCCACATAATGCGCTTTTAAGTGACACCGCGCGACACATTTCACGCCATTATTGGGGGAAGGCGCCGTGGCAGGTGCCGAAAGGAGGGATGTGTGTCGCATGTAAATTGTCCCATCATGTCATGTTGATTGGACAGTGTAAATCAGCGGGGCCCTTTTGTGCATGCAGGTTTGCTCACATGTGACACGGACACATGTCGTAGTTGTCAGAGCTCAACCGCGCCAGAGGTTTTAATGAAGCGACACCTCCTAACATGACTTGATTTGCCGGGTGCCT
LG14	ref-5067_7	55.73	Chr12	24106076	TTCTCTCCCACTCTCCCTCACCCCCACTTCCCCATTTCTTTTTTTTCCTGCGCTGTCAGCTCTATTAAATCTGTGTGAAAGTGCGTGGAGAAGGAAATGCACGTTTATCTTTACAAATTACGGTCTTTGCATTAAGCATTTCATCTTCTGATGAATTATCTCCAAAGCGAACTGCTGCCTGTGCTAATTTTTTTTGCTCCGCAATT[C/T]GCACGAGGGTAATGCTAGCTCAATTAAATTCCTTTCAGTCTGTCACAGCCGGTCATGATTACGGTACATAAAAGTGTCTGAGAAACGTTTTCAGGAAGATGGACAAATACTGCCAATATTGTCTATCCACTCAAACAGCAGTCAGTCTATTGAGCAGACTATTTGCTGAATACAAAGTGTCTTGACGTATCTTTTTATTATAAAAGTTTTGATGAAACGCATTAT
LG14	ref-67305_24	56.77	Chr12	9445718	CCGTATGGGTAAGTTGGTGCCTGGCAAGATTTTGAGCGATGTTGAATCTTTTTCCGCAAACATTGCAGCCAAATGGTTTTTCGCCAGTGTGGATTATTTCGTGGGTTTTGAGATGTGCGGCTTTCCCGAAGCATTTTCCGCAGACTTTACAAACAAATGGCTTTTCTCCTGAGTGGACTCGATGGTGGTCAGACAGACTCTGTTTGTGGGCGAAAGCCTTGCC[A/G]CAAAGAACACAGCCGAAGGGCTTTTCTCCAGTGTGAATGCGCTGATGTGATTTGAGGTTGCTTTGAGAAGAGAAACTCTTGCCGCACTCTGAACAAATAAATGATCTCTTTCCGAAGAGTGTAACAGCGTCTTGAGGATCTTCACAGCTGTCTTCTAGCAAAACAGATGCTACTCCATCTTCTGAAGAAGTGTTTAGGTGACCTGACA
LG14	ref-53742	57.193			AGCCAGTCATCGATCAGGATGCGAATGCCCA

LG14	ref-52354	57.354	TGTGATTTATTTATTTATTTGAGGAATGTAATGGATTAATCGTCTGCAGTAGTGCCAAAAGAAATATCACCTTTGAAATATCCTAGTCAGCTGGTGAATTAAAGTAATAACATCATATAGCTACAGTACAGCGCGACTCTTATCCTCTTATCAGGGTCAGGAGGATGTGTAAATGGGCGGCACATAACTCGTCATCAGATTTTAACCAATCAACGGGGGGTTCTCTGTTGCGGAGAAGCCAATCAGAAGAGCTATATTCACTTTTCAAAACCTGCCCGCCATCTTGCGTATGTACGTTTAGGACATGTAAACGTTTGTTTACGGAATATAGATATTGCGAATTATTATTTAGTTTTTGGATATAGCATT
LG14	ref-50905	57.356	CCCACGCCCCCGAAGAGACTGCAGCCTTAAT
LG14	ref-22617	57.438	AGGGAATGATACGCGGTGCCATACTTCAGATATGCCTGTGCAATTTCTTCAGACAAGCGAATCTGGTGATGCTCTCGTGCAGGTGTTATTTATAGACTTAATCACACACAACGTTGCATCACCTGCCTTAGTCCTTGGCCATTGGCTTGGCGTGTTCTACATGTGACTCAGACACCGACTCACAGTGAAGGCACTCACATAGCATCAACACGATGATGTTGCGTCTCATTTCCTTTCTCTGGGAACTGGGTTATATACATTAATCTGAGATGATTTGCTTGTGGCAGGGGTTTTTTTTTTTTTTTTTGTGTACTGTAATTATATTAAAAACCACAATACAAGAATGTTAATATATAGGGCTGAGAAAGTAACACGTTAATTTCTGTTATTAATACTTGGTGTTTAACTCATTACAAATAATTAACGCAGC
LG14	ref-59022_27	57.445	TGTTGCAGTACGAATGCGTTGCAGTG[C/T]GCGCT
LG14	ref-21961_4	57.475	AAATTAGCTAATTGAATTCAACTGTTAAAACCAATACTTATAAGCAGGACAACCAAAGTGTGTTGTAAACAAAGTGAGAAGTTGCTTGTCTACAACATCCAGAAGCTTGCATGTGAAGGCCCCACGCAAAGGTTATTGGAATTACAGTAGGTCAATCCAACATGGCATCCAG[A/G]TGGTCAGCAAGATCATCGAACATGTTCCCTATATCATCAAGAATGTTCCCAGCTGACTTCACTGTGTTAATGCCCCTGAGAGAAAGAAGAACAAGAACAAGGTTGAGAAGGTTTAATATGGAGGTATTGTGGTGTATGTCTTAATAAAGGAGTGTTTACACTGACAGCAACCTGCAGTCATTTTACATTTCTATGAG
LG14	ref-41579	57.603	AAACGATCCTCGACTACATTGCGTTTCCACA
LG14	ref-57409_5	58.204	TTGCATTGTGGCTATGGCTATTTTGATAGCTTTTGCTCCCTGCGGTATTCAGAGGAAACAAGAAGCGGGAAATGTGTTTTCATTGCGCAGTGTCAAAAACAAATGCTTCGTCAAGAAAAAAAGTTTTGCTAAATTACAATGGAACACACTCTACCATATCATATACCAATAAAC[A/G]CACGCGCATAACTGTCGCTCTACGCTGCTCTGACTCCCAGCTCTGTACGTTTCCCGCCCGCAGCGTGACGCAAAACGTCAGGTTACCACGTTTTGCATTTGTATGACGTGATCAACTGTTGCCTACTATACAAACTGTACTGTCGGCTGTTTCATCTTTCTTTTTTTATTACTTTGTTATGCTAAGTTAATTTTTA
LG14	ref-28004_30	58.29	AGTGTGCCAGAGCGAGCTTAATGCTGACAGCGCTCAGCTGAATATGACATGACGCTCTCCCCAGATGAGACTCATGGAATATATTCACTCTGAAAGAGCATATCTCTGGTGTTCGTAAAATGTCGAGCCTCTCAGCGCGTCGCTTGCCAGCTCCGCCCCCGCAAGCCGCGTTCATTGAGGACCAATGCTCTCACTGCGAGCACACGAGTGCGAGCTCAATGCTGACAGC[A/G]TTCAGCTGAATATGGCATGACGCTCTCCCCTGACCCAGGCGAGACTCTTGGAATATATTCACTCTGAAAGAGCATATCTCTGGTGTTCGTAAAATGTCGAGCCTCTCAGCGCGTCGCTTGCCGGCTCCACCCCCGCAATCCACGTTCATTGAGGACCAGCGCTCTCACTGAGGGAGCAAGCTCAATGCTGACAGCGCCCAGC
LG14	ref-29678	58.865	TGGCCGGCCACGACCCTGTTGCTGCTTCACC
LG14	ref-33198	59.617	CTTTGTTTGTAACGGTGTATGTATATTTGACTAGGTGAAAGATGCTGCTATCCTGACCGTAAGCCATACGCTTACAGGCATGTCATCCATGTAATAAAAAAAAAAAAAAATCAGATCTCTGAACATGCTTCATGTGTGTACTACAATTATTTATTTCAGAAATGTGGATGTTGTGAAAGCACTCATTTCGGACCACCAAGAGAGATTTTGCATGAAATTGGATTAAAAATTTAAAATAATCATTTTTCAAAACCCAGAGTGAAGCTTGAGCTCGGTGATTACAGAACGATCCATTCTGTAATCGATCCACTCACATACATTGATACAGTTTAGTTTGAGGCTTTGGCTTATTTTGGAACTATTTTCAT
LG14	ref-39677_31	60.313	TCTCCAGCGTCTCCATTCTCCAATGTCTCCATTCATCTCCAGCGATCCATCCATCTGTAACTATATCAAAGACTATATCATCTATCTGCATTCCCAAACTACTCTATCATCAGCACATACTCACCTGCTTCACCATTGTCTCTGCTGTGTGTTCAATAAATCCATCTATTTGTACTTCCTGTCTCCGAGTAGTCCGTGTTGTAACACCTACGACCTGTCTGCCTAAGCAG[A/G]ATGTCTAGATTCAGCACTCTCACAGATTTCTCCAATGAGAAGGTACTGCAAACAGGCAATAACCATTTCAAACCGGATTGAAATGGTTGTAGCTCTAGAGAGAGGAGGCCTAGCAACACTCTACTCTAAAGACAGCAGTCAAGAAGGCTCATAAAGAACCTTAGTGCCACTGTCTAGAAGGAAGAGTTCTAAAGAGCGGAG
LG14	ref-21387	60.514	GCCTGTTATAACCCGCCATAGAAGGTTTTGAACAGGTGCATTGGCCGGATCAATTGTGTCTGCATGTAAGTGCAAGCTGGTCTCCTTCACACCATAGTTATCAAAAAAAGTAGTCCAGGAGACTGATCACACAGTTGGCCCCTTTGCCAGTGATGATTGCCTCATCTAGAACAAGGATCACCATCTGTGGAAGCCCTTCTGTTACAATGCCGAACACAGCGCACTTGCAAGGCTTCTTGAAGAAGATTGGTCCTGGCTGCATGGGATCGTGGGGATAATGCAGCTGTTGAGCAAAGTCAGGGCTGTAATGAACCTGAAAAACAATGTGCAAATGTTTACTCACTTTGTCGATACACCATGTTTTCTGCAAAAATGAAAATCCATTCAAAAACCAGTGCTTAATGGTAGAAATGAAAGGCACTGCAAGATTG
LG14	ref-33834_10	61.126	CAGAGCCTCGTATAAGCTCTGGTATGACTGGGTGCGGTTCATCTCAAAACACAATTTACACAGTGTCCTAACCAGACGCGACGCCTGTTTATAGCCTATTCCGTTGCTAGTGCAGTGCGCTTTAAGAGAGAGCCCGCCCACACGCTCTTCTGATTGGCTGTGATTTTTGATTGGGGCT[A/G]GCAGGCGTGTCGCGTCTGGTGTGGACAGACAAATTTCGTGTCGCTGGAATCTTATCGCATCACGTCTGGTTAACGCTAGTCATTTTCCTCTCTACTGTCTGCCACTACATCCACTTTAGTCGGTCATTCACAGATGTTCGTACAACTAAAGAGGGAAATTAATTATTATGTACGCCTATGTATTTTAAAAT
LG14	ref-11698_6	62.107	GAATG[G/T]TGAGCGAACATTGTGCTTGGTATGTT

LG14	ref-20732_23	63.502			GTTGACTATTTGAGAAGTCATCTAACAACCAACTAGTTGATCAGTGTAGTGGTTGATCGATATATATCGGCCGATATTTGGCAATTTTTTTTATTTTCTGTTTGGTGGTTAAG<br>TGACAAAAGCAGACTTTTATTTTGTTTTGACAGGAATTCACAAATAACTGCAAAATGCACCTTTTGGCATGCTGATCG[C/T]TTGCTATATTTAGCATTTATGTCTATATCTT<br>GTCTTTGTTTATCGCTTTGCACAGTACACAACATTTGTGCAGTTTCTCCATCTTTAAATGCAAGTGTATCCTCCATCTTTGAATGTGTCCTTTCTTTATTGGTTGAACTAATCC<br>TTTAAGTGTGTAAATATTTAATTTTTTTTGTTG
LG14	ref-62265	64.627			GGTTCTGCCCCGAGCAATGTGCTTAACTAGG
LG14	ref-60462	65.616			GATTACAGCACGACGCAGCTGCAAAGCCTGG
LG14	ref-30279_24	66.998	Chr12	13653514	TGAAGAAAGAAAGACATTAACATCTTCACACTCATCATTCATTCCAAACTTTTTTCTGTCCCATCAAACCTGCAATGCTCATGAAGCGATGGGAGAAGATGGACAACTGGA<br>TGGTGTTAAGTTCAGTTCCTAAGACACACTGCGTTCCCATGCCAACAGCAATACACACGTCACCATCCTCTGTCAGATGAGCCACACACGCCGGCTCCACGAGAATTGTGC<br>T[G/T]ACAAAAGAAGAAATATTAATAAAAAAATGAAATTAAAAATATATGGTTACAGAAACAATGTGAAGATTTCTAAACACCTTAGATCAACCCTCAATTGGCAAGTCCC<br>AGAATAGAATTATTACTTAACCGCAGAGCTGGTGACTGAAACAGGTAGTGACAGTTTCCCATCAGAAGGATAGTTCAGAGAAGAGCTAAACCCTCTACTTTT
LG14	ref-30796	68.69			GTGTGCTTTCCGAATGGAGTGCTGTGGCAGT
LG14	ref-66634	69.792			CAGATCCACTTGGTCATTTTACTGAGGTAAACGCTGCACAATAATATCACTTTTTACAACGTCAGACACATAATGGTAATTAATATAATTAGCCTTTTGCTACAACATCATC<br>AAATAGTTTCAAACCACGTTTTTGAAACTGTCTTAAGATCACTGCAGTTTTAAAGAGAAAATACTCAGCAACGGTGTTGACATGAATTTGCACGTTTTTTCTTAAAGCATAT<br>TAAAAACACCACATAGACATATAAACAACATTAAAAACTTGATTTTCACAACAGGGGAACTTTAAAAGTTCCTAATTTTGGTTTGTTTAAAGATTCAGTCTCTCTATAGAG<br>ACAGAGCGACACGGGTCATAATCTGAAAACCACG
LG14	ref-8351	70.255			GTAGAAGGAAAACAGGATGTGAGGACAGAGGGGCTACTCCGGAGCAGGAAGGCCGTTTTACCCTCTGAGGTTCGACGGAAAGAACGGAGCACCGAAGACCCGTGCCGTG<br>GAAGTGCTGACAGCGTGGGGATAAACCCAGGCATCAAGAGCGAGGGTGCTGCAGATGAACCCAGAGGGTGGCGTGGAGGTCGGTCAAGGCGGGAAGTTTACACAGAGCA<br>TGTGTCCCGTCTTCAGGCCGCAGAGTCTATGCAAGCAAGAGCTCGAGAGGCCCATAAAAGGGCTGACGGTATCTGCTACGCCCCAGTTCAGACCTCAAGTGGGAGATATA<br>TGCCAAATTCATCCAAACCCCTGGATGTTGGAAATGTCCTA
LG14	ref-58100_10	72.139			GAGAGCGGCAATCTTTAGTGAACGCGGCTTGTGGGGCGGAGCCGGCAAATGACGCACTAGAGGCTCTGCATTTTGAAACAAATTGCCAGTGAGA[C/T]TAGCTTTATTAAA<br>AATAAGCTCTATATGCTCACCTGACCAAAGATGGCAGGTTGGATGAGCTGAAACAGGTGCTCTAGGACGCTGCAGGCGGCTCGAGAGCGAAGCACTGACCAGGCTGCTTG<br>GGAGTGGCAGGGAGAACAGAGCTGCTTGAGAGTAGCGAACTGATCTGGCTGCTGATGAAGCAGCGCGTTGATCAGAGCTTGCATGAGAGCGGCGAGCTTATCAGAGCCT<br>GCAGGCTGCTTAGAGAGCAGCAGGCTGATCTGACTGCTGCAGA
LG14	ref-48115_29	76.691			TTGCTCATTGCGACTCTTTTGCCAGCAC[A/G]GGT
LG14	ref-41144_24	78.345			TACCATCTATTAACAACCTTGTAGCTCACAGTAGGTCTGTCTTTAAAGGTTTGTAAGCAATCATTCAAAATCAATTCCCCTATGGAGAAAATGAATGGGACTTTTAGAGAC<br>ACTCACCAGTGGACTGGGAGTGAAAAATAATATAAAAAAGCCAGACAAGCATTGATTTTGACTCTACAGCCCAAAGGCATAAGATGACTTGTGCTGGACCGATGTGTTTG<br>CT[A/G]GACTATCTCATATAAATTATCCATGGTGACATAGATTGTTCGTAGTCCAGCCTGAAGCCTCAAGCACCACAGCTTGTGTGTGTGGCCTGTTTCCAAATTTTAAAATC<br>GGATAAATCAGTCAAAATCTGTTTACTTCCCAAACTATTTTTATATATCCAAATAGTTAATACTCCAAAAATCTTTGTTTGACTACAAAAGAGTGCTGGCA
LG14	ref-53018_4	81.809			TTA[A/T]ACAACGCGAACATCATGCAGTATGGCAA
LG14	ref-67456	83.911	Chr12	9547057	AATTTTTAAATAGCCAGTATAATAGGTTGTATCTTGACACAACTGCCTTTTCTGTAAAGAATTAACATTTTTGTGTCTGTGTTTAGGGTACAGCTGTATTCTGGTATAATCTC<br>TTTCCCAGCGGAGAAGGAGATTACAGCACAAGACACGCAGCTTGCCCGGTACTGGTGGGAAACAAGTGGGGTGAGTGTCTCTGACACAGTGTATTATCAGGAGATTTTCT<br>TCATG
LG14	ref-1571	86.649	Chr12	8120834	CAAAACTCTAGTGTTGCATTAAATGTCTGTTTTGCACATTCACACCGAAGACGAGACCCACGGCTCCATCCTGCTGCAATTAGCACAGTCGCTGTCAAAGAGGCATGAACT<br>GATTCAGAAATAACTTGCAGATGTGCAACTGAAATCTCTCCTGAATCTAATTCAGTTCCTTTTACAACACGCCAACAAAAGCCGTTTGTTTGAATGTGTCGAGCGGCGCGCT<br>GATTGGACGTGGAATTGCAGCGCTGCTTCAGAATGGGCCGCTCTGAAATATGATGACACCGCCTTAGAAAATTAAACCCATTTGTCATGAAACGCGAGAGGTTTGAGAGA<br>CAATGCAAATGTTTGCTTGTAATGCTCAGATGAAGGTTAAAGAAAAACAAGAAATATCAATGTCAAACACACTTTGTGGGTTAAAAAGTTCAAATAAG
LG14	ref-60066_28	88.556			TCGCGGCTGCAGTTTGTTTTGTTGCACGGAGAGACAAATCTGTGGTACGGCGGAGCTCCTCCACCACCTCAGGGGGGATCCCCTGGCCCTGGTCCAGGTCCTTCAGCAGGT<br>CAGCCTGGTAGGCTTGCAGCACTGACCCGCTGCCGCATATGCTCTGCCATTCAGCCGTGATGTGACACGCAGTGTCTTAGACGGCAGAGATGGCGTCTTCGATGCGGATGC<br>CTCAC[C/T]GACCGAGAGATATCTAGCCAGCCTCTCTGCACAACTATATTCCTGCATCACCTCCACATCAGCATAGTTTGCCTGCATACGATCGATATGAGCAGAATATGGT<br>TTTCCCCAAGATCTCTGCACCTCAGTGTGAAAATCTGGGAGAACGGGAAGACTCATACGAGCTGGGCAGTTATGTCCTGACAGGAAGCGCTCATCGAGGCG
LG14	ref-66895	88.709			ACCTGTCACTCGATGAGATTGCATCAATACC

LG14	ref-41376	89.158			TGTCTCAGTACGACGTCAGTGCGACGAAAGG
LG14	ref-55694	90.946			GAGTTTTTTGCGAAATAACTGCATTATTGCC
LG14	ref-12065	91.883			TTAAACTGCCCGAAGGCTGTGCCCCCACCCA
LG14	ref-49797	93.307			GTGTTTTTCGCGACATTCATGCTGAAAATCA
LG14	ref-59783	94.558			TAAAGAACAAAAAAGTAGAAAAGTGTTTGCATCTTTTAAGAATTCAAACGTCCTGTATCATATCCATCCAACTGCGACAAGCAAACGCAAATGCAACAAGTGGTTAACGC<br>TGCAAATCAAGAGATGTTTAGCATTAGCAGTGCCGTCGCCTCTTTGAGCAACTCAGTGTAAGTGTAAAAGACTTGTAGCATGAGATCTTTGTGCTTTTTACAAAGCTTTTGC<br>TAATAAGCCATCAGCCATTTTCTAAAAGCTTATTCAAATGAGTTGGTCTTTCAACTCTAAACAAAGCAACTGATATGTATTAAAATGTTTTTCAGTAGTTTAAAGGTGAAGT<br>GTATAATTTTATGGGATGTTAAAATACTTTCTCCCCTGTTAAATATACAGAGAAAGCTATAAGCGAGCCATTTGTGAGTTAATTTAAACCCAAAAAA
LG14	ref-38066	95.562			TTTTGATGGCTGAAATCCTTCTTAATCATTTTCAGATAAGATGTTGGGATATACAGTATTAGATGAATCCCAAAAGCACCAATGGTCAAAAATCAATGGACTTAGTTAGAC<br>ATTAATGGGTTGTTCTCCTCTCTTTATGCTTTCGGTTTCACAGCCTTCAACAATTCAGTGTTTTTTGTGCATCTCTCTCAGTTGATATCTGAATTTTAATGCCTTTGTTTTATTG<br>CTTTGCCACTGAGATGCAAAAGAAAATCTTGTGCAGTATTTTGACATTTGAAAGGATCGAGAAGAAATGCTAAACCTTCTGTTTTTTTGCCGATTTCATTACATGGCGTCCT<br>CTTCTGCACCAGCGAGTGTTTGCGGACTCCT
LG14	ref-33121	96.549			ACCTCTGCTCCGATGGTGTTGCCGCCACCCG
LG14	ref-32288	97.359			TGCAAAAACTCGACTCCAATGCACTTGACAG
LG14	ref-48809	98.011			ATTCATGACCAAGGAAGCTAGGGAGCTGATTGAGATGCAGGGAATGACATATACCTGACCATCTTGTATTGTCTTGTCTTTTGAGCATGCAGTTTCTTGTCTTTAGTTTGTT<br>GTCTTGTTGTGCGAGTGTGCGGTTTCGAGTTCGCTCAGGCCGTCTGTTTGTTGTTATGTTCTGTGTAGCACACGATTCGTGTTTACACTTATGAGTGTTGTCATTAGCTGCGT<br>GTTGTTGTCTTGTTTTGTTTGAGCAAATGGCTTGTGATCGTTTTCGCTGCCCATGTGCATATTTTCGTTTTGTTTAGTGTGAGCACATGGCTTGTCATGTATATTTCTTTGTGC<br>TATGTGCTCTCCTGTCTATTGTCTTAACCC
LG14	ref-71899	99.861			CAGTCCACAGAGTTTCACTAAACGTCATGGAGAATCCTCATGCTCATTGGCTGCCGTCTCTGCAGCTACTGCTGGATAACTCGAATTTAAAGGTCTAAAGCCGGAAGACAG<br>CGTAAACGTGGGTACAGCATACATTTGATGACAGGTAAGGAAATTAATAATTGTGCCTAAAACTCACTACTATCTGTGTAATTTACATAAAGTCAAAAACGAGTTCGCTGA<br>TCCACGTCGATAGATGGCAGTGCAGCGTCGACTCAAACCGTATTGGTACTTTAAGCACACAAAGAAGAACGCACGCTTAACAGACCAGTAGAAGTGTTTACTGTATGTTA<br>AATTCAGAAATTTCATCAGGGTACACATCTAACATTTACAGACTTAAAGTTTTATTAATTTATTTCACATCAAATCACATTTTACACCGCATACAATAG
LG15	ref-32744_17	0			ACACTGATTATCTCGTCACCACGGCATGGGATATATTAGGCAGCAAGTGAACATTTTGTCCTCAAAGTTGATGTGTTAGAAGCAGGAAAAATGGGCAAGTGTAAGGATTTG<br>AGTGAGTCTGACAAGGCCAAATTGTGATGGCTAGACGACTGGGTCAGAGCATCTCCAAAACTATACGATGAGTTAAAATCACTAAATGAGCCTAGATGTCGATGT[C/T]TG<br>TGCGAGTGATGACCGTACTTGGCGGCGCCGCGAGGAATCTGAGGTGAAGACTCTCCACCTCCTCGTCCACAGGCATGCTTAGCAGACCCCAGCGCTGACCCCTCCGCGTGG<br>GCGCCATCTTCACACACACATCCCTGTTCCCAGACGCCCTCACGCTGTCCAGCAGACTCGCCAGCAGAGAGTCCCTGCAGACAGCACAGAGACGGCTCTAAC
LG15	ref-47568	0.873			GGGAAGTATGCGATTCGGATGCAGCCTTTAC
LG15	ref-62831	3.611			TTGAACTGATTTTCATGTCTGAAATACAAGCATAAAGGAATAGTTTAGCCAAAAATAAAAATGTCACTGGAGTTATTTGGTAGAATGTTCATACTGCTCCATATAACAAAT<br>ATATCTATATAACAATACATATAACAAATTGTCATGTGGGGGTTTCAAGCTTCAAAAAGGGCAAAAAAGCAAAAGCATCATGTGTGTTCAAACCCAGGTGAAAAAAAAGT<br>ATATAAAAAAATATATATTTAGTTACCACTTGTAGTACACTTGTTTCATACAAAGATGGGTTCAAATGTACTACAAGTGGTAACTAAATATATTTTTAATACAGAGATAGTA<br>TGTTAAAAGCACATTTTGAGTACACTTAGATGTTCT
LG15	ref-31207	4.253	Chr24	39631152	GCTCTTTGGGTCATTAGATGATCGTAGTTGACATTCCCCCCCGCATCACTTCCTACAGACTCGAACCTGAACCCGAACCGAACCTGCGCTGATCCGGACCGGCTCCGGTAA<br>AACCACCGCCTTTACCAGCTCCCACTCCTCTGGACTGGTGATGGGGGGTCGGAGATGATCCTGATGGACTCGATACGACCGCACGGAACCAAAGAAACGCGAATCGGCTG<br>CTGATTGATCGCAGTTTCTCTTTTTGTTTGGGTTCGAGGGAGTATGCGTAAAAGCCACGCAGACTTAGCTTGCGCAAACATCTCACAGATATGCGAATGCGCAACATGGGA<br>GCGTCCCGGACTAGTTTGAAACAAAACTCCCCGCAGTTCGATACGTCATTCCTCTCAAACACGTGGACGGTGCGGAAGTGCGGAGATGTTCACATAAGC
LG15	ref-61212_18	4.549	Chr24	39467031	GAGAGGGAACACCAGATTGGAAAAATAGGAAGGTGGAGTGCTAAAGAGAATGAGCGTGGGTCGGATCGGTCGCTATAGTATCGTGTCGACGGAGGAGGATGGTCTGCGT<br>TTAACAACCATGCATGGCGGAGGGATGAATGGATATGGCAACGGCAAAATCCACACTCGACGGAAAAGTCGCAACCG[A/G]TTCGTGAAGAAGAATGGACAGTGCAATGT<br>GCAGTTCACCAACATGGACGAGAAGTCCCAGCGCTACCTGGCAGACATATTCACCACTTGCGTTGACATCCGCTGGCGCTACCTGGTGATCATTTTCACGTTCGTCTTCGTG<br>GTTTCCTGGTTGGCGTTCGGCTTGGCGTTTTGGGTTATCGCG

LG15	ref-22977	5.374	Chr24	39594463	TATCTCTATCATCATTATCAACCAGTCGATCATAAAACACACGATAGACCTCATGGATCCATAACCGGATCAGCTTATCTCCTTCCTGAAGAAAAAAGAAGCACAAAACTCAGATTCTGTAAACTATTTAGTATATTTACTTTGCCTTTACAGAGAAATATACACGTGTGCATTTACCTGCATGTGCGCCGATGGACAGAGCATTACGCCCCTAATCACTCTCGCAAAATCTCGCAGGTTGAAAATATAATGAGATTTAGACGGTGTTGGGAGGAAACTCTCGATTGCATCCTTGTACACTGCCATAGTAGCTTGAACCATGATTTTACCCAACCTACAACAAATACACAGTAGTTACAAAAACAATTT
LG15	ref-38516	6.218			TGCCTTTCTCCGAATCTCATGCCTTATCTGC
LG15	ref-28191	7.3			GCATATGATGCGATTGAGCTGCTGGTTTGGG
LG15	ref-6314	11.996			ATACTCCACACGATGGACATGCAGTTCGCAA
LG15	ref-27424_8	13.39			GCTATAACTCGTGAACGGAATGAGGTATTTTCACCAAATTCAGCACAAGAGAGCTCATTCTGTAGTCGCGTGAAAAAGAATGCAGCAACTGGCCACTTGGTGGCGATATAACATGAAAAAAAACATAAAAATGGCTATAACTACTTCACTGTTAGTCCGATCGACTTGAAAATTGG[A/G]ATGCAGTGTCTTCGTCTGGGTGCCATGATTGTCTATGAGGACATTGAAGCATCTCAAAAAATATGTCCGCCATCGACCAATGAAATTTGAGCAGCTATCAGACAAGGTTTACGGAGGCCGATCGGAACGAAACTCGTGGGGCCTGTTTGACTCACAGCCCTAGAGGCCTGTGAGAAATTCGAAAAAAATCAGC
LG15	ref-10059	13.714			AAAATAACCAAATTGCAAAATATTATTATTCAGTATTTCTTATTAAGACAAAAAGGAAGTCTAAGAAGCCCAGATGAAATGAACTTTTTTAATCGTTTCCTCTTTGAAATCAACAAGAGGTTCGTTTAGTTATCTGAGCACTTTTTCATATCTTTGTGTTTCACATATTTTTGCTTCCTGTTTGTGAGTGACGATTCATTTTACACACTTCGATCGCTGTGAATCTGTACTTTCCCAAAGATTTAAAAAAATATTTATTTGAGTCAAAATGTGACGGCCAGTGCCTCAATAAAGACTTAAAACATAAAAACATTTCAACTAAAAATAAACAAAACAGAAAGGATCCAATCAATTTTTCCAGTCGGCTCATTTCATGGAAAATTAAATAATAATAAAAAACCTACATTTTAAAAGTCATCTATGAAGCTTCT
LG15	ref-53851	15.065			CCATGCCAGAGACGCTGGTTCGAATCCCACTCGGAGCAGGTAGAGTAGGGCTGGCTACATTGGTGCCGTGATCCGGATGGGAGTGAGGTTTAGGGGGGTAAGTGTAACGGAGGCCACCTAGTAAAAGATGTGTGAGTAAACATCACTTCCCTGGCCTCAAGAGGCCCACTAGCGATTGACGTTAAAAGGCTGCGTCCTTGTTAGCATGCCCGCCTCCTATGCCAGTGACGCAGGTTCGAATCCCACTCAGAGCGGGTCAAGTAGGGCCGGTTACATTGGTGAGTGTAATGGAGGCCAACTAGTAAAAGATGTGCGAGTAAACCTCGACTGACGTTAGAGGTTGCACCCTTGTTAATGTGACCGCCTCCCATTCCTAGAGATACCGAGTCGAATCCCACTTGGAGGATCAAGTGGGGCCGGTTACATTGGTG
LG15	ref-70505	16.578			GTTGTTTAAGCGACAACTGTGCGATTCAAAA
LG15	ref-72252	16.588	Chr2	41111180	CAAAATGTTTCAAAAATAAACTGAGGGTGTCTGCAGTGTGTACATTCAGGTGAAAACACACACACACACACACTGAAGCTCAGTGCTGTATTAATGGGATCTCTCACTGCACTGATTTCAGCTTTGGGCGACACCGTGACTCTGAACTGAGGACAAAAGAGCCATGAATAAGAAATGTCCTATAAGGACAGACGGGTCGCCCCCTGAGCACGAGACGAGCGCTAATAAAGTCTCTCGTATAATGAAAAATGAGTATGAGTACTGCATGTGCACTTGAGCGGGATGATGCTTTTCAGGGCATTTCAGTGAACTAGCAGCAGAACTTTGGGTTTGAAATTAGAAAACACTTCCAAATGAAGTGGAGACGACCCTTTCACAAGATGTAATATACGTCCCCAGAATGTGTCTGTGAAGTTTCAGCTCAAAATACC
LG15	ref-28486	20.071			GCATTGAGATGTAAAGATCCTCAGTTTGAAATGCATAGTGTTACGACCCTGGTCCAGGTCAGGGAAGCAGCATAAATAAAATGCTCTGGAAAAATCAGTTTCAACAATTTCTTTTTTTTTTTTTTTTTTTTTTTTTTTTTGTTTAGTTTCATCACAAAGTCAAAAAAATAAATGAAGCAATTTATTCGGGGCTGGGCAAAGCCAAAACAATAAACAAAACGGACACTAAACTAAACCCGAATTATAACTAAACATTGTAAAAAAAAAAAAAAAAAAAAAAAAAAAAAAAAAAAAAAAACAAGAAGGGCCTACCTCCTGGCTAATTCAACACAGGAGAAAAGATGTACACAAAAGAAAAAGACACCCA
LG15	ref-54000	22.744			AACGTGCGTGGAGCTAAAGACAGACCAAAGGGAAGGACAGTAAACTGATATGCGATTCCCTCTGGTTCTTGGGAACCAGAAAATAACGACTGTAAAAACCACTCTCGCTCTCTTCAGGACGCACTACCTCTACTGCTCCTTTTGTCAGCAGATTCTAAATTTCCGTTCTCAGAAACATGGCGTTTAGATCGTGAACTGCAGACATTATCACGCCGTTGAAACAGTGTTGTCTGCGAGCAAATTGCAGAGTGTATCCCTTTTCTATGATATTCAAAACCCACCTTGACGCGCCTGGCAGCGCTCTCCATTTCTCTGCGTAGAGTGCGAGAGGTGTCATTGTGAAGTGATGTGGCAGCTTGTTTGCGGGGG
LG15	ref-4722	26.262			AATATTCAAACAGAATTATGTAAATTGTTTGTGAAGTGTAAGGGGACTGCCAATCATTTGAGCCATAAATACCATGTGACAACCTTCTGAAAAGGCCAACGTGAATTTTCACAGTGCTGGTCTAAGATTACTAATGCTTTCTGCACTCTCTTTAACTGTAAATGACAGGAAATAGGCAGAAATGCATGAGGACAGTGGGGTCATGCTGACAGAGAGATCTGCATTAGATCAAAAGTACAAACTTCAAGTGTGACGTGCCTTCACTGTAGCCACAAAAAGGTGAGTCACAATCAGCCAGGGGGTTAAAAATGAGAGAACGAAAGCATCTGCCAGAGAGGAGGAGAAACATAGGTGTGTAGATCAAAACAAAAGAAGCGGGATTCCATCACTGCGTCATTCTTTCTCATCCATCTCTCCTGAAGAGATCACAA
LG15	ref-24218	27.082			TCCCTGATAACGATCTCTTTGCAAAGTTTGC
LG15	ref-70693_15	28.061			TTACTAAAACCGAT[C/T]GCTTTGCTTCAGAAGGC
LG15	ref-18532_23	29.923			ACAAAAACTCACTAATGTGAAAATGCCCTATGGGTAATCTCTTGAGATTCATTTCCACCTTTGTGATACAGCCTTCAAAAAATGCCATCAAAAGCAATAGCACTTTGTCCATCAACAATGTATAACATTTATTACAGCCCGGAAGAACCAAAGCTTTTATGTGAGACACAACAAATTGGAAAACAGACCTCTTGGTCTAGCCGAGACTCCCGACTGCACTGC[A/G]AATCACTGAGCTCAGGACCCCCTACTGGCATCATGAGCTTCTAAATGTCTGCCAAACCTGCTTTATTTCACTACTAGTGGATTCCTCTAAATGGGAAAGGAAGCAAGA

					CCACTGTTCATTCGAGCCTCTACTTTTGAATACATTTCAAACCGCTCATGGTAAAGGTTGTGGTTTCTTTCGTTGGAGAAGGTTGTCAATGCACAGATTTC
LG15	ref-59035	31.58			CATTGAAACACGAGCTGCGTGCTACACAGCA
LG15	ref-32853_7	32.939			AAAAACCTAAATAACTGACATGGGAAACTTACATTGGTTAGAGGTTCTGAATTTCGGTTTTGATTACTTTTCGATTAATCATCCAGCCCTAATTTGCATAGTAGATGCTAGT<br>GCCGTTAAAACTGAGTGGTATTTGGTTTGCAGCTTGTTACCAGTGAGGCATCAGGTGTTTGTGTTGGTCGGATGAGATATATGTGTCTGTTTGT[C/T]TTTCGACATACGTGC<br>TCCCTGACCTGCGTGTCAGTCTTGCAGTAAACGCATATTTGCAGTGACGTTGCAATAACCTCGCTCCAGAAACGGAAACATGAGGGTCGGACACCAGAATCTAAAAACAA<br>ACATTGGTTTCTTTACAGCTGACTTGATTGCCAAGCTGATTTATTTTGATATTGAATAACGCCCATTAAATAATGAATAATACCCATTAATCTCTAATCA
LG15	ref-42415_23	33.917	Chr24	3931906	AATAACGCTTTAAAAGCTATTAAAATGACTAGCACGACTGTAATAACGCCAATGTTAATTTTAATTTCTAATTTAATTGTACAGTAAACAAAATTATAAGAGCAAATCTCG<br>GATGCCAGAGATAAACTTACTGTAAATTAAATGTTCACAGTTAGCGGATATAGTTGCATTTGTCCAAAGCAAAATTATCG[A/T]CCCGCCTACATCATCTTTCCTACCACAC<br>GGAAACGTCGCCGAGAGCGTCTACTATTGGTCAACATTCTGACAAGAATGCGTGATCTGATCAGCCATTGGCTGAGAATGCTGTCAGTCACAGCATGTTGATGCAGGTTGA<br>GGAGAGGACGTGCAGGAGGAGACCGCTGGAAAGAGAAGA
LG15	ref-45178	34.763			CACTGTTTTCCCATTCTGTTTCCTAGGTTCGCTTGTGTTACTATGGTGATTTAATTGTTTGATTCATTCCCTGCACCTGTGTCTCGTTTCCTTGATTAGTGTCTTTATAGCCATG<br>TGTTTGTCTCTTTAATTGTCAGATCTCATCATTACTATTGTGGTTGAAAAACAAAAAGCTGCTTGCAAATAGATCCAGCTCTTTTCCAACCCCAGCGTGGCACTACTAGCAT<br>CTATAAATACTTTATCTTTTAAATAATAATATAGGAGAATGATATTTTGATTACACTGAGAATTTGGGGAAAGAGAACTTAATTGTCATTAGAATAATAATATCAAAATGC<br>AAATTAAAATGGTCCAAATATATGCTTAAAA
LG15	ref-46039_2	35.36	Chr24	1227582	ATCCAGACACATCAGCACAGAATTTAGGACTGCGGAGGATGGGGAAATGTTGAGGCTGAGGTGGGAGACACGCTAATTTATTTACGAGAGGTCACAGACACCAAACAAA<br>CACATATTGGAGTCCTCAATGTAAGGAAATACATGTTGAGCTCATGTTCCCTAATATAACA[G/T]CGAAAGGTGCAAGCAGGTCGGATACACACATACTCAAACAACACAG<br>GCCAAACATGCAGGCTTAAACACATGTAGCAGATGGAGAAGGCATGTGTGTTAAATGGACAGTAGTTTTATTTGTTCACTCACACTGAGCCATTAGCCCTGTTTTGAAAAC<br>ATAGTGAGTCACCAGCCCAGACAGCAGGCATCAAATTTATGT
LG15	ref-53140_24	36.299	Chr24	22015292	AAGCCTCTAGGCTGCGAGAACACACCGTTCCCCTTCGCTGAGCAATCTCCGTCCTGACAGATACGACACTGGATGGTGAGGAACGTCCCAGACTGAGCTCTGTAATGTGTC<br>GTTTTGCCAACTCGGCTCTGAAGAGTAGGTGCTAAAGAATTACAGCCCACGGAGACAGCAACGGATTCCACACTGACCTTTAGAGATGAGAAAAGGCCACGAGCTGACTG<br>CCT[C/T]CACCATCGAGATGAGTTCAGAAGTGTGTCTGCAGGAATAGAGGCATCTCTTCCCCAAACTGCGTTGGAGAGAGAGCGCAGCGCTGGGCTAATTCACAATGCCTG<br>TTTGAAAATGTCTATAATGATATGTAATTATGGATGTTTGCGAGTTTACCCCGCTGAGCCTGCCGCCATCGAAACCAATGTCGAATTACAAGCTTTAATTTCG
LG15	ref-66526	36.576			TGATTGGTTTTGTTTTACCCCCCACAAGACTTTTGAAAAATTACTTGCCACTGGAAACTTGCTGAGTCATGTTTGGTCAAGACTTGGTGTAACAGTCTGTTAAATGAGGCCC<br>TTGACGAGACCAGAGTTCACATTCAAGTTCAAGCCCATGTCTTATGGCAATAGGCATGGATGACTGTCTTTAGCGCTACCTTTTGCACTGAAGCAGGACGAGCCTTCTGCT<br>GAACAGGCTCCCCATTCCAACAATCATGTCATGGAGGGAGTTAGACATATTGTCCATGATGGTCAGCAATTCGGACATCATTCTCCTCTCAGAAGCAAACTTGAGAGGTTC<br>CAAATCCACTCCAATGACAAAACCTGTCTTCCTAATTAGTTTATTTAGCCAGTTGGCATCTCCAACTCTAATTCCAGTACCTCAGCACACTACAGCA
LG15	ref-29197	37.875			CTGTCTCTTTACGACCACAACAAACGGGATACTTTTCAAACGCGCATTCCAACTTCGCCTCAGCACCAGGCGCAAGTCAAACGAGCGCGGAAAAGGTGTTAGGTGACGAT<br>GAGGGAGCTTCAGTTGAACACTCAGAAAAATGAAAATGACTATCCAATCAGCCGTTATACTGTGCTCTCGGGCCGGCGCGCACCCAAGCACGCGCGGCGGCGTGTGGCTG<br>CAGACTATTACTTCATTATAGCTTAAATAAAGCGCAAAAGAAACTACGAGCATGCACCGTCGTTGTTCTGTTGTAAATTAAGTAAATGCGATTAAAGCTGCCTCAAA
LG15	ref-45671_32	38.109			ACTATCACAGACTGATAAAGTCTCTGAGATGAGTCGAGCTGGTGAGCTCTCCTACATGTGGAATCATAAATTCAGTCAGAGTCTTTTGTGTGTCTGTGTGTGCTATAAACCA<br>CCGAGATTATTTCCAGGAACAGAGGGAGAGTGGAGGAGGTAGTCTTGTGATTCACTTCTGCTGTTGGATTTTGCCCCTTTTCAGGGATCTAATGTGATCGAAGTAGCTGCA<br>GATTGGGGT[C/T]GTCACCAGGGGTGGTTCTAGGATTTTATCCTCAGGGGGACTTAGCCTTCAGTGAGAATGTGAAGGCCTCTTTAAATCGGCACCACTAGAGTAAGTCAAA<br>AGTAGCCTAACAGTACTTAAATTGGTACCAAGTCAATACAAACATTTTATAAATGATATATTGATACCACTCTTTCAGCAGTACAGTTCAAATCGGTAT
LG15	ref-52226_24	38.526			GGCAGAAGAGCTGCGCCGCACCACAGACTGGGCCACCAAACAGACTGCTGCATCGGTGGGAAGGTCGATGGCAGCAATGGTGGCCACAGAGAGGCATCTGTGGCTGAAC<br>ATGGCTGACATCGGGGAGAAAGAAAAGGGCTTTCTCCTCATTACACCAGTTTCGTCCTCTGAACTTTTCGGCACCTCCGTCGA[A/G]GCGGGCATACCATTTAAGACGTGCA<br>TTCCGCTGAGATCTGATTCTGGGCCCAGACAGACAGGAGGTCCTGGCCCGTCTCGATTTGAGGATCGGAGGCAGGGTCAAAAGTCCAGTGTTGCCTCTCGGGCACCTCCTC<br>CTCCTAGGAGCAGGTCCCAGAGAAGGCGGGACTCCAGGAAG
LG15	ref-7240_15	38.635			ATCAGTACATTCAACATATGAAGCTTTCAAGAGCAATACTGGCACAAAAGCCAAGATCAAGTTTCCCTGGGTGCACTGCTATCTTTTCTTACTGTACATTTATCTCCTCATG<br>AAACTATCATTACTGATGGATAAAGTCATCATCCTGTGCTGCTGAATCCCTCCAGAAGAGAACATGAGCAC[A/T]GGTGTCGAACAGGAGGAAGCAGCTCCAAGCTCTCAC

					TGCAAAAGTCTGTGAACTGACAGCAGACATTGCTGAAAAAAGCCCAATATCAACAAAAAAAGTCACAATCAACAATAAAACATGCATTTGTTTTCATTATGAAAATTTTG<br>CTTCAATTATTATGCAAGACAATTTCCTTAAAAAGATAGT
LG15	ref-7474	38.801			TTTTATAGTATATTTTTAATAACTGTACTTGCAGTTAATTTAATGTCAATGAAATAAAATGCTGCTTAAGTATACTTAAAGAGAGTACACTTTCATGACTAAGTTTCTTAAA<br>GGATTAGTTCACTTTAAAATGAAAATTACCCCTAAGATCGAACACATTGCTTCACTTCAGAAGGCCTTTATTAACCCCCCGGAGCCGTTTACTTTATTTTTAAAATTAATAT<br>TTTAAATCATTATTTTAAAAATCCTTTGCCATGTTTTAAACACTTATTTTGATGTGTTCATTAACGTACTAAAGCACTAAGTACTTTTAACTGTAATGTATTGTTTGATATTAC<br>ATTTAATTTAATATATATTTTAAATGTACTATATT
LG15	ref-1798	39.035			TACCACTCTTTCAGCAGTACAGTTCAAATCGGTATACATATTTTCTTAACTCTTTCCTCCCCAACATTGATGGAATTTTCCGGCTTTCTGCGTTTTCACTGTTGTACAGTAGG<br>AGGCGCTATTAGCGGCTGTTCACATATCGTGTCTTTTGTGCGCTCAAATTCGTTATTTCAAACGTAGGCGCTCGACAGGCGCGCTCATAATGGAATGCGACACGCATGCAG<br>TGCATTTTCCAGGCGCATCCAGTTGAAAAATTCTCAAGTTTTCAGAATGCCGCAAGTGCACCGCAGGTCATGTGACAAGAACCAACTGATCAGCTTCGCCCTTTCCGTAAC<br>AAAACACCGAAAGCTCAGCCGAACAGCTGATCATAGGGTGGTTTTTATATCTCACCTCCATAAATCTATCTAGTAGTAGATATAATGCAAGGGCTA
LG15	ref-73239	39.722			CATAGATTATCCCAATTATACCATGGTCATTTGTAAGCACTTACAAGTTAAAACTGTTAATGATGTTTGCAGTACAATATTTGACTGGAAGGGTAAAAATGCCCTCATGCA<br>GCTCATTAGGTCTAGCATTCTTGCCCACTTTAAATCAGACACAGAGATGAAGTAGTGCGGTAACACATGCATATTCATCGACAGAAACATTAGCAGAACTCTGTCTGTTTG<br>TTTTAGGTGTTGTTTTTTCACCAAACCATATTATAGATTCGTCATCAGATTTGAGATTAACCGCGGTGCGAATGTATGCCGAACTGGAGAGAACGGTAGGACCGTAGGAAA<br>CAGAAATGATTAGTTCACCTCTCGAGTCTTCTGGTC
LG15	ref-64554	39.928	Chr24	15125308	TACCTCTTTAGCGTATGCTTGCCTTTCCTTTCAAGTCAACAAGTGCATGCTCCCCATTAGCATGCAGGAAAGGCGACCCGCAAGCTAGTCCTTCAAAACCTCAGCTCGCTCC<br>ACATGGAGAGTTCTTAAAGGGTCTGAGAGAGTTTGCATTAATTACACCTGAAACAGCTGGAGCCGCGCAGAATGTAATGAGTCAGCTATAGACTGTCTCGAGCTGAATGC<br>AATTACCAAGTGCTCAAAAAAAAACAAAAAAAAAAACAAGCTGAATGCAATTACCAAGTGCTAAAAAAAAAACAAAAAAAAAAACACCATCGATATTTAGCCTGCTTTA<br>GCTTGTAAAACATGGTTTGCTCATTGTCTTTAAACTGAGTCTATCCTTTTTGGGCCGTTTGGGTGTTAAGAACTCTGTGGATTGTGTTGGGAATGAACAC
LG15	ref-58466	41.367			AGTGGTCTTACGAGCAAACTGCTATCTAAAG
LG15	ref-69422_23	42.484	Chr24	24090813	AATATTCGCAGAAATACTACAACTTTTACTTTAATCTTGCTAATATTTCAAACTGACAATACCGAAGGGCTCCTACCCGCGTGTTTCTCTGCCTGATCCGCAGGGGAAACAT<br>TTTCAGCATGGTTCTGTCCACATGTCAGTCACTCCACGCTGTGGACGGCTAAACCAGAACCCTTGTTCAGCGTGGCAGGAGTGGTCACTTAGGCGGTCCGACTGTGGTGC[A<br>/G]TTCATCCATGCATACAAAAGACAAGCAAAAGGAAACGCCTGCCCAGGAAGGGCCTACAGCTGCAGGTCAGCACCGAGATTAGAGCATGTCCTGCCCTGGTTTCCTAGG<br>CAACACCTGCACAGGAAGAGGCACATCTGTCCAAAGAGAGCATCAATAATATATCACCCTTGAACATACATGCTGTGGGGCTGTATATGAGTGTTTATACT
LG15	ref-51177_4	43.304			AGGAGCCACAAACAGCTCCTTTCTTTCTTTTAGATAGCAAAGGGTGCAATGGCCCTGAGGTGCCCATTTTAAGTTATGCACTAAGCATGATAAAGACCTCATATTTGCAAA<br>AGAGTTGAATGACCAGTCGTGCACTCATCCTGATGGCATTCAGTCGAAGTTATGGGTAATTAATGGCAAACTGGCCAAAATTTCACATGCCA[A/G]TTTCAGCGATGGAGG<br>TGCAATTTAGTTAATAATGAGTGATTCATTTATTTCTATATAGGCTACTAAGTAGGAAGCACCAATGCAATACTAGAACCCCTTAAAATGCCCTAGCAACCACCCCCAACA<br>GTACAGCGTTATGGTAGAGATTTTTGCACAGGTAAAATCTTCTTGGGTCTGACGTCATGTGACAAGCACACTTTGATTACCTTGAGATGGGGACATTTGGCA
LG15	ref-39836_9	44.595	Chr24	28243673	ATGAAATAAATGAGAATTAATGCACGAGATTAGTTATACTACTGTTTGAAACTACTAAAAACATATAAAAGATTATTATTTAATATATATATATATATATAAACAGAATCT<br>ACTGTATACTGCAGTACACATTCAGTACTCTAGTATTTCCTTCCGAACATAGCCAGTGTGTCCTGTGAATCAAAAAGCGTGTTCAGAGTTAAAATAC[A/G]ACGATGAGGAT<br>GCAGCCGAAAGCTGCTGGGATTGTCTCTCGGCCTGATGACATTTAACCATGTGTCACAAAGTCCAGCTGTTCTCCCAGCAGGCACCATCTACATGTCATAGTGACGCCTGT<br>CAAAATCCATTAAACTGACCAATAGGATTGCAATTTCTCATGCGGTGTGTGACATTCACAACACGTCAGAGTTAGCGCTTGCTGTTCTCATTTTCAGCACA
LG15	ref-4352	45.074			TAAGAGCGTCCGACTCCCATGCGGCTGATTG
LG15	ref-6204	45.777			CCATGAGTCACGACACAAATGCCAAATGATT
LG15	ref-54188	46.465			ATACCTTCAGCGACAATTATGCAGGCAGAGT
LG15	ref-5921_10	47.706			GAAAAGTGTGGAACGCCAGACTATAAATAATGGGGGGGGGGGGGGGCCCTTGCCCTTATGAATCGCGCTTCATTTTTTTATTCGCATCGATTCACAAATTTCAAAAATCGA<br>TTTTCTAAAGTGTTACACTTCTTTAACGTTGCAGTTCTACTAAAAGTTATTGACATTAGAGTATTATTAATCACAATAAGCAGTGAAAAAGGACTAAT[A/G]CGATGCTCGT<br>GCTGACGGACATGCAGCCAAAGCATGAACACAGATGTCACCTCTCAGATGCATGCGATGCCAAATTTAGTTCTCTTTCACAGCTTATTGCGCTTGCATGGTCAAATACACA<br>CACAATTATGTCAAAACGCACATCGTGTGGAGTATTCACGTAAACACAGTCTGTTATGTCCAACGTGAACGTAAACAGTTAGGAGAAAATCGGATGTGTAA
LG15	ref-6103_10	48.5	Chr24	33468944	CATTGTCGTGAGGTGGTTCAATATTTCATAGTGATTGTTTGTTTTGGTGTGACTCAAGGTATGAATGACAGTGTTCCTCTGGTCAGAACATGCTTTGTTGGGTTTGTTCACTG<br>CTGTCATATTACAAAGATGTGATTGTCTGCTCCAAGTTAAACACAAAGAGTGTGAGAAACTCAGACTGAGCTTTTGTGTGTCGGTAACCTGAGTCT[A/G]CGAAGCCCCTGC<br>TGGTGCACAGAGAAAACACCTTTTACCCTTTAAACAGGGCTGTTTAGTCTTGAAGTGATAATTAAATAAGCTTAGCGCACCGCTGTTTGAATTTCACCTGTGCTCCTGTCGT

					TTCTGTCTTGCACAACATCAGAATCGTTCGTAAACACGTTTATTGTAGCGCGGCACTTCCAACTTGCTCGCCAGCGCCAGCATTTTTTATCATTTTCA
LG15	ref-52768_15	49.437			ATACGCAAATCGAT[G/T]AATATGCAAATGAGCCC
LG15	ref-26040	50.762			TTTTTTCTCTCGAGATGCTTGCACCGACAAA
LG15	ref-73249	51.792	Chr23	45811650	TTCTGTCATTAATTACTCACCCTCATGTCGTTCCACACCCGTTCATCTTCAGAACATAAATTAAGATATTTTTGATGAAATCCAAGAAGTATCTGACAGCAATTTAACCACC ACTTTCAAGGTCCAGAAAGGTACTAAAAAAACATTCTGTTAGCGCTTCCAGGTTCTACGTCAGAACGCCGGCTCAGTATTGGCCGACATTGTTCACGCCGATGCTGATGCT GACGCTGACGCTGACGCAGGAAATGCTGAATGTAAACAGCGTAAGAGAATGACACAGAAGAGAAGATACTGTTGAATAAAGCACGCAAAGTATTCTCGTCACTTCATAA CATTAAGGTTGAACCACTGCAGTCACGTGGACTATTTTAACAATGGCACTGTTCTTTTACTGTGTACAGCTCATGATCCAGTGTTTTCAGTGTTAGAAA
LG15	ref-12582	51.862			AAACCCCTCTTTCCCGGGTCTGGGGAACCATCCAACTTGCCATCCGTAACAGAGTGGATGCGACGTAGAGAGGAGACCTGGAATCTAGCCCATCACCGTCTACAGTGCGC AGTGAGAAGGCAGGAGGAACAGGCCAATCGGCATCGACGCCCCAATCCCGAGTACACCATGGGGCAATGGGTCTGGATGTCAACAAGGGACCTTCGTCTACGACTCCCAT GCAGAAAACTCAGTCCCAGGTTTGTAGGGCCTTTCCAAATTGTACACCGGAGGGAGCCGTCACCTGAATATTGACTTTCCACCATTCGGACTACGTTTCCCATAGGCCCTC ATTCCTGGGACTGATTGCGCACACACCTGCACCACATCACACTCACACTATTTAAGCCACACACACTCACTCACACTTTGCGAAGTCTTGATTTGCCCCG
LG15	ref-72966	52.395			ATTGACGAACCGACCAGGCTGCCCGTGTTTC
LG15	ref-25368	55.253			GCCGTGTGCACGAAATAGATGCTGACACTGT
LG15	ref-62327_26	56.52	Chr24	10005461	ATATTACAGCCTTATTTAAAGTCTGATTTCGATAGCAATTCTTTCCATATTCTAAACAAAGAAAAGTTTTTAGAAAAGATCACACTCCTGTTCTACCTGCTATTAAAAATGC ATTTTTAAATCATTGTTGTTCTGCCCTTAGCTGCATTCCTGATGTTGATAAGCGCAAGCCTGCGCAGGCAGCGGTGTCGAAA[C/T]GAAGTGATTTAAAGACATCATAAGGC CTTTGTTGTTCGCTGCCTGCATTGTGCTTTGCTTTAGCCTTTGAAAACACAAGACAGAATGTGTACGCATGTAACTGAAAAACCAATTCGGGGTGTAAACCAGGGCTCTGA GAAAGTGTGTAAAGGAAAAAGAAGTTTCAATGGAATGA
LG15	ref-67841	57.994			GCATATGACCCGAGATCCATGCCCTCCACAG
LG15	ref-37374	58.501			CATCTCCAGTCGAAATGCATGCCCAGATATG
LG15	ref-58643	60.145			TGTGAACATACATTTGTAAATTATAACTGTAAGTGTAGAAAATAGTAGTATTTGCTAATTTTAAAAAAATCACAATTTTTTAATTGTAGTTTATTATTTAATTTAATAAATTA AAATTATTATGAATAAAAATGGGGGAGGGCTGATTGTCTTTATGCAAAAACAAAATATCTTGATATTTGGAATATGCTACACACATATGAGATACCTTGATGTTTCTGCAC TTCCTCTTGCAGTATTTGTGTTGGGTTTAGGTGGACAGCATGTGCTTATAGCTCCAGGAGGACCTGTTGCTAACAGTGCCGATTTCTCTGCCTCGTGTCTCAGTTCCAGAATG TTCTGTCTCTCTTGCAGTTGCTGAATCTGCTCCTGTTGCTCTTTCAGAAGGCGCCTCTGCTCTGCTACAGTTTGCTGCAGGGCCACATGGCCGT
LG15	ref-29704	60.674	Chr24	16821502	GAGACGCTGTAGCTGAGTCTTTGTACCTTCTGGACAGTTTAGGAGACAAACGTTTACAGGTGTGACAAGTAATACTAATACTGTTGAAGCTTTTAAACTGTAGGCCAGTGC TTATTTAAGAAGAAAAAAATCCTTTTTAGGCTTTCATTGTAGAAAGGAAGGTTTGTTATTTGTTCCAAGCATTGGCGTTCTCATAAAAAAACGAATACAGACAACCTTTTTGCT CTGACTCATTGCACTGTAATCATCCTGTAGAGCACATAATACTCTTCATGTTTCACCACATTGTCTTCTCTGTGACGTTCTTCTGTATCCCTTCATTCTGTTCACAGGAAGTC ATGTTTTTTGAGTGCGTATTCCACGACAAGTAGTCTGCTTCGTCTAGGAGTGGTTAGAAAAATGAAAAGGAGACTAAATGAAGTGGAGGAACTCA
LG15	ref-18486	60.779	Chr24	21229760	GGCCCACATCCATAACAACTGGTTTTCGGATCACTCTTTCTTTTTGGATGAGGGGAAAGATCGCTTTCATCTGATTTACAGGGAAACATCAGACTGATTTGTTTTACATTGA ACGTGTTTATCAAAGATGGGGTCAGGATTATTTGATTCGTCTTCATTTCAAAACTGGCCGCAAAATGGCATCAGATTCAGTTTCCAGAATTGAAATTTTGCCATTTAATTTA GCAACCAGAGACTTTTTTTATTGGCTACATTAATTTTAGAATAAAGAGCTGTTGAATACTTCTCATATTGATCGTTTTTGTTTGAAGGATTGGAAAGCTTGAGGATTTAGTC CTGTGGAAATATTCAAAGAGATGTTTTTGGATC
LG15	ref-2659	61.502			GATTTTTGCGGTTAATCTGAAGACTGTGCGGAATTCTTCTGAATGAGATTTGTGGACATGTGGGACATATTTAAGGCCCATTCACAAAAAAAAGAACAAGATGCAGGGCA GTGGAACTGGAAAAATGCAAAGTCTAGAGAGACTAATTTTTTTTCAAAAAAATTTTTAGCTTGAGAGCTGCGTTTTTTGAATGATGCGAGACACTGCAAACGATGTGAGTG CAACGGTCAAACACGTCTGTCTAGTGCTTATTTACATAGAAAAACAATCGAAAAGCACCACTGCGGAATGCAAAAACGTATGAGAACGTCCCCTTATAGATTCAGTACTG AAAATGCTGACATGCAAAACAGTTTACACACAAAATGACAAGCTAAGATTTTACACTGATATTGAGCAAAATGCATTTCTCAAAAAAGACAAAATTGATC
LG15	ref-11175	61.888	Chr21	19505683	GTTTATCTATTGTCATTTTTGCTTATTAGTATGGTTGAATTGGATCATCGAAGGACAGCAGCAAAGACATTGGTTAATAAAGTAAGATTAAATACAAAAAGTATATTTGTGT AATTTAATATTCTGAGTTTTTATTCATTTTGAGGAATACTGACTGTTTTTGTGCAAGTGAGATTAGTAAATGCTCACAATAACCGTCATGTTTACACACGACGTCTCTGCACT TACTACTGATTTCTCTTTCAACATGGGGACAGGAGAGCTGTCAGTCAATATAAGGGAAAATAAAGTAACTTGTATTACTTATTTCAAAAAGTAACTCAGATATTTTATTGTA AATTTAAAAGTAATGCGTTACTTACTAGTTACTTGAAAAAAGTAATCTGATTACGTAACTCAAGTTACTTGTAATGAGTTACCCCCAACACTGT

LG15	ref-53661_25	62.635	Chr24	23417958	TAAATATGTAATGCATTTATTAAATATGCAATAAGTAAACATACTTGTATAATATCATAAACATACAGATATATCTAGTAATTATGCTGATTTGGATGAAGCTCGTTTTCAA<br>AGCAAGCTTGACAGCACATCAGACACATGTCAAATATGGACCCGTTTGATACTCTGACATATTTTAACGTCTAATTAAGCTCATTAACAGCTGATTAGCGATTGCCTTGCCC<br>[A/G]TGGCAAAATAGAGCCTCCCATCATTTGGCCGGCTTGAGTTAGCTGGCAGGGGAATAGGGCGTCCAGAGCTTAACAGGCCTGCTTTTGTCCACACTGACAAGCAACAT<br>GAGGAGGCCAATGCTGTTTTATTAATTAGTCCCTTTGAAAGTTGAGCCATGTGGCTACACACATTACGAAATTTGTGAGGCTGCTCCACGCATACACAGT
LG15	ref-70420	63.221			TGTGTAACTTGGGGTGGAAAAAGAGAAGGATAAAAAATGTTACTAATACATACAGGAGGTAACATAAAAGTTTATGTTTTTGAAAGAAGTCTACTATGCTCATCAAGGCA<br>ATTTATTAGATCAAAATGTTAAATGTAATTCATTCCTGTGATGTAGGCTTGCTGCGATAGTTGGTGTTGACAGTATTATCGGTGTCAATGGTTACATCACTTGACCTCTGCG<br>GCACTGCCCTGAATTGGTGCTAATTTGAAAGCAAAAGTAAAATTTGCACGGCCGCACGGGCATTCATAACGGTGCGTGTCTATTGGCGCAACGTGTTTTCAGTTCATTCCT<br>ATGGAAGCTCAACTTGGTTGTGCGCATTTTAGTGCG
LG15	ref-32703_10	64.034			CCACTTGGG[A/G]CGATCGAGATGCTTTGGCTTCA
LG15	ref-12399	64.622			TAGTCCATTGCAACATACAAAACAACTGGTCTAGCAAAAAAAAAAAAAAAAAAAAAAGACAAATATATTACAGTACTATTAATACAGAAGCCCTCTTAGGATATTCACTGC<br>AATCGATGACAGCAGTTGCGTATGAAACAAGAGGGAGCGAGTCTGCAGTGTGGTAAGAGAACCATGAAAGTAACACCAGTGTCTGCATGAGCGGTCTGGTCTACACTGCT<br>GCCCTGGACAAATATTAGTGAATATTAGGAAATATTAGACCACTAATTGTCACAACTGACCAATCAGAATCAAATATTCCAGAGAGATATGTGAGCAGTCGTATGATGGG<br>TAACAATTTACTATCGGGAACACATTAACTATTAACTATGACTTTTGCCTCAATTAACTCCTAATTTACTGCTTATTAATAGATAGTAAGGTAGATGTTAT
LG15	ref-57033_4	66.004	Chr24	20386182	AATTTTGCTTTTCAAGTAAACGTATCTTATTTTGAGGATGTACAGATGTTTTTATTGGAACTCAAGACTCGTTTTTGTAGTGTATGGTTGCCAGCTCTTGACTGAAAATCTCA<br>AAACAAGTGTTTCCAGTTTGCTGCCTTCTGTTCATGTTATTAAGCAGCAATGTATTTAA[A/C]ATGGATGCACTGTACTCGCTCACAGTTATTTTCCTATAAGCATCAATCTT<br>AATGCTTATGCTAGAGCCTCAATAAGCGGATCAAATTATTTAATGCCACTGAACCTGAAATCAAAACACTGAAAAATGGCTTTGCCATTCCCTGCCATTCAGATCCCAGGT<br>TTTGTAGCAATTTCTTCATGTCAAATAGTTATCATG
LG15	ref-4463	67.5			CTGACTCCCGCGACAAAAGTGCCCTGATCAT
LG15	ref-64856_2	68.349			G[A/C]TTTCTTCTCGATCTCAGTGCAGCTTTTGAC
LG15	ref-71743	71.313	Chr24	24350597	CTGAAGCTGTATGCTTGTCTTTAATATTGATAAAGATTAAATATTAATCCTATCGTACAGCATTTCAAGCCAAAATATCTATTAAAAAGATAGTAAGACAGGGGAATACAA<br>AAGAGACATGACTAATGTGTAAAGCAAAATGAGAGGTGTCATCATTTATTCACAGGGTGATAGTTATTAGCAAATTAGTCCTCCTGTCAGATACACCTCATTCATTTACAC<br>AGGATGTTTATCATTATAATTAAGATGTAATTTATCATCAGTAATGAGCCAGATTAAAAATAAAGCATGCTGAATGCTCGACATAAGTGCTTTCTAATGCTGGTATAAATG<br>ATGTTTCAACCACCTATTATCTGAATATATAAATAAA
LG15	ref-48971	74.368			CTTTTGATCAATTTAATGCACCCTTGTTGAATTATATATATGTAAATAGCATGTTTATCAGTACTATGATATATCATAAACACCATGGCATTTCCATCTGATATCACAGTACA<br>CTTTTGTCTCATGTTCTTCTTCCAAAGAACATTAATTACGTCTAAATTGGATTTGTTAATTAAAGTTTCTCTCTCCCAGAAGAAATCCCACAAGCCCCGACATTTCTGCTCAT<br>TAGTTTCCCTTCTGAAACAGACACTTATTCCACTCATTCAGGTAAAGGTCAGGAGGTCATTAGAGTCATTAATGCTAAGAGAAAAGGGAAAGGTCTCCTTACGCAGAGCGT<br>GACCTCTGACGGCGGCCCGTCCTGTCCTCATGCTGCCTGAAATCCAAGAATCGCTCAGCCCTTGACCTTGTCTCACACACACACACACACACAC
LG15	ref-8824_1	75.656	Chr24	31475688	TCCGATCAGCTGTGTAGTTGTTTCTGGTGTGGTGTGTAATGAGCTCTGTATAATGTGCATAATCCCTCTTTTTTCAGTATCAATTATTTTTTGAGTTCAGCGATCGTCTGGAA<br>AGCATCATGACTAAGGCGTATATCTGGAGGTATGTATGAAAATGTGAGATTTTTATATGCAATTCTGAATTTTTTTTTTTTTTTTAGT[G/T]TTTGTGTTGCGATGTTGTTGCTT<br>ACTAGCCTGTCGTAAGTGTGATATTCGGGTATACCCCACTAGAAATTTTACGCTCCCAGAACATTCTCAGAACTGGTGTAAAGGACCTTTTTTTCAGAGTTGGACGCACTAC<br>TGGATATCTGCTCTTCTGTCTCCATATCAACTCCTGCCTTTACTATGTAGCATCAGAATATGAGGGGCTGGGCAGCTCTAAATGGACATATGATGG
LG15	ref-10370	78.266			GCAGTGATTCCGAGGGATTTGCTTTTGATTT
LG15	ref-55224	80.588			CGACAATGATGTACTAAAAACGTTTTTCTTTGTACAAAATGACGTTATCAAAACTATCCCCGTTCACACGGATCCGCGAAAATGACTAAAAATGCTGTATTACGCATGCCA<br>GACAAGCAGTTGGCGATGTCACTTTGCAAAGAAAGACTACGCACCTGCGCATTCGCATTCTTTACAAAGCGCTCGCCTCGCGCATGCCTCGCGCATTTTCATAGTTTACTGC<br>ACTTTGATATTCTTCTCGTTATTAGCTTAAAGCGGATAATAAATCGGAAATCTCGCACATTCACAGAAAACACGTTAAAAAATAAGGCGGACAGACTAAACATAAAATAC<br>GCATGTGCATGACGTCACCGTTTTCACAGATTCGCG
LG15	ref-43265_9	82.427			TAATAAACAGCCAATATGCAAGCTAATAAGCAGCTAGTTAAAAGTGAGAATTGTTCCCCATACTAAAGTGTTATCAGGCTGTTTAGTGCACACACATTAAGAGTAGTGAGT<br>AGTGAACACAGACACCATTTTGTTGTGGTGCCCGGGGAGTGATTGGGGGTTAGGTGCCTTGCTCAA[A/G]GGCACCTCAATCGTTTTCCTGCCATTAATGAGAATCGAACCT<br>GCAACCTTCAGGTTACTAGTCTGACTTTCTAACCATTAGGCCACGACTGCCCCTATCAGCTGTCATTGTCATCACAAAAGTATTAAAATGACAAAATGGTTCTGAAAACCTA<br>CACTTTTTGAAGAATATCCTATATTTATCATGATTTTA

LG15	ref-72482_16	83.511			AATAAGGTTTTATTGAACAACACTGCTAAAATATATACAAAATATTGACAATCATCTTATGTTCATTATGTATTATAAAAAATGCCTGCAGAGGGTGCCAGCGGCCTGGAGATTATTATACTTTACAGCAGATCAAACCCTTGTTTAATATTGGCCAAACAACATTTCATTGAAATGTCCACTTAATATTTAACATTGGGCCACCCCTTACGATA[A/G]AAATGCTACAGCCACTGTAATTTCTTGAACAAGAATATGTGGACACTTTAAAGCATGCAAACTCAGAGTGAGGGTTTAAAATTTATTTGAAATCGTGATGAACAGTTAGCATATTTAGAAAGATATTGATGTACACTCGTGTTAGCGTAGGTTTATAAATGTCTTACCTTACAAATATGGTGCAAACTGCACTCCCAGATGAACGTTATAAGCAA
LG15	ref-4886_5	88.169			AGAATGGATGTATTTATTTCGATCTTTTTCTATCATCTAGGTTTTTGAAAGTTGTTGATTAGTAGTTCATGTAATAAAGCATTTTCATCTTAGATATTTTACACTTTTTAACAGTGTTTGGCACAAATGCTTAAGCCATATTCACACACATTCAAACAGTATAGTACTCACTC[A/T]CTGGTGCAGAGCAGTCGCTTGAAGTTTCTGCCTTCTCACTGCAAGACAAACACATTAAACACATCACACACCTTTTACATCGCTTTATAAGTTGCATGGACGTGCATTAATAAAAGAATCACATTAAAATGAGTGTTGACAGCTTTGACTGCCACTCAAAACTTTCAGGTTTGATAGTGATGTTCAATGGTATTAG
LG15	ref-27195	89.688			GCGGCGACAGAAAGCAAAGGAGGCCAAAGCCATGCTGAAGCTCAAGAGACTCAGCCAGCAGGCGCACTGGGCGGAAGGAAACGGGACGGCCGGCGTCATCGAGCGCAGCGGGGAGGAGGACAGCTACATGACCAACCCCAACCTCACCATCAGCGTGCCCATCGCTCCCGGAGAGTCGGACGTGGAGTTTCCAGAGGAAGAGGAGGAGGATGATGATGGTGCAAGCGAGAGCTCAGAGGAGGAGGAGGAGGAGGAGATAGAAGAAACGAAGCCGGTGAGTGAACGCAGCTAAAGCTGAATCTTGCATACTTACCAACAGGGCTTGACAATAACCAAATGCGGGTGGATTTCAGCAGTGGCGTGTAACGCAGTCACTCCTACTAGCCAATTTGGTGGGTTGAATTTTATATATATGCATTTTCAATAATGGT
LG15	ref-26247	91.376	Chr24	36926085	CTGCCTGGTTCGCTCCATCCTTTCCCTGATCTGTGGCACCAAACTCTGACGAGAGCCTCATGTAGTGGGCTGGGATCACAAGAGTTGGTACCATACTACGGTACATATTCTCCTTCATCTGATCCAATGAGCTGCAGAATATAATCTGACCTGGTTGCGTGTTGAGTGACGTTGGACGAGCAAGGTGATCGGGCCCTTGCAAAACTTGCGAGTACTCCGGAGGCTTGTCAACAATTGGTGGAGAAGGCGGATCAGACGTGTAGCCGCGGTTATCATTGACGACGTGCCTCCGGCGATAGTCGCGATCCTTCTCGTCAGGGCTGTAGTTGCGTCCGTAGTCCTCATGTAGAAGATTGCTGTCCAGATTGC
LG15	ref-13808_28	91.467			CCGTGACCACGTCTGACAATTTCGTCTGCTTAATCTTCTGGATAAAAACGAGTTATTAAACTTTAAAACTGTACAAAAATGAACATATACATAAAAGTTAAACCGAATATCAACTCAAGCCATGTACTTTGAATATTATATCGTCAAAATTTACCTTTAGAAATCTGAGGAGAAACTTCAGTGACGGTTAATGAGATTCCCGCTCTCGAACGATGACGATGCACAGA[C/T]TGCGCATGCGGAGGTTAATGCGACTGACGTCACGTGATCTCGTGATGGACATTAAAATTACTAGCTAATGATCATTTCATTGTAACTTTTACATTTTTGATATGTACTTGTATTTTTGTTACCTTGGCATTAATTAAAAAGAAAAAAAAATAATTACTAGCTTGCGAGCTGACACTATTGTCTTTTATTGTATTAAATAATATT
LG15	ref-3710	91.762			ATTATGACAACGACTGCAGTGCTCCACTGAC
LG15	ref-72482_5	91.919			CCAC[C/T]CCTTACGATAAAAATGCTACAGCCACT
LG15	ref-51016	92.861	Chr4	15119581	TTATTGATAAATGTACAACTTAAGATGAGATAAATACACATCCAACCATGAGATGTTCGTGTTGATGAATCTGTCCTCCACCTGCTGGCCAGAGAAGGAACTGCAGCAGCTGTATCTCTCTCATCTCCTGTTTCTCTGTGTTCTGCAGGTTGACATGGAGAAGATGACCAAGGTCAGCACCATCAGTGGCCGTACCATGTTTTGCCACCTGGATGCTCCTGCCAACGCCATCAGCGTGTGTCGGGATGCTACGCAGGTATTGTATTTTTTGTGGTTGTTCACATGACTTTTTAAATGTAGCCCATTTCAAAGAGAAGCATTGCTACACTGAATTAAAGAAAAAATGGTGTAGGATTTTCTTTTAAACAGTTTTCACTCAGATATTGCTAGTAAATTTCACAAATAATTACAAAGAAACAGCAAGTAACTCAT
LG15	ref-13055	93.357	Chr24	35940085	TAGTGATGGACGTACGTGTCATATTAGCATTTTCCCAAACGTTGTGAGATGCCGGTTCCTGAGAGCGTTTGCATCTGCTACGCGCCTGTTTCCTCTCGCTGCCATGTGAGATGGAATAAACACACATGCACGCCAGAGCAACGCTGTCATGAAGCTTATCTCATCGTGATTAGATTACTGCAGTGGCCTCCTAGCTGACTGCCCAACTAAAACCAACCACCTATGACAGGCTGTGTGGAGCACGCCTGCGCACGCTAGTCCGTGCAAACCTCTCTCTCATCACTATTTTGCATGCCTGCTGTCTATCTCTGATGACAAAGATAGACAACCACATCATTCATACATGCAGAAAATAGCCTTGATGTATAC
LG15	ref-38139	93.545			AGGTCTTGCACGACCCCGGTGCTGATGCTTT
LG15	ref-46864_6	94.722			AATTTTGAGTTGTTCTTTTCAGCAATTTCATTGTTGCATTATAGACAGGGACAAATAAAACGATAGATAGAATGATAGATAGATAGATAGATAGATATTGCATTATATATATCACTACTGCATCTTCTAGAATATTCACCTTCACTGCCAATTGGATCCGTGTTGACTGACGGCTGCGCCATCCAATCCCCTCTCAGCTCGTAGCGAAA[C/T]GCTGCGATGCGTCTGCTGATGTCTTGATGTGGTGTTGCTTGCAGGAAGAGGGCTGTTCGTTCTCCGGGCATGGCACTGAAGCAGCGCACATGGGCTGGGCCGGGCGTTTCGGATGTCTCGTCTCCCACCAGAAGCTCCAGGAACCCATCTCGCTCCAGGTAGAGCTGAGCTCCACCCCAGTGCTGATCCGGCTTGATGCAGGCCGACAGTGGGCTCCTGCTGGACT
LG15	ref-33348	96.001			CAACTGCACAGCAACATACTCACAACCACTCAGAATAACCGATCAACTGCATAACACACTCACAACCACTCAGAACAACCCAGCAACCGAGTAGCAACACATTCATGACCACTCAGAACAACCCACCAACTACATATCAACACCCTCACAACCACTCAAAACACCCCAGCAACCGCACAGCAACACACTCACAACCAGTCAAATCACCCCAGCAACCGCATAGCAACACATTCACAAGCACTCAGAATACCCAAGCAACAGCATAGCAACCCACTTAAAAACACTCAAAAGACCCCAGCAAGTGAGTTTTGCATAGGAAAACACTGCTCACATTTATTTCAAATGTAAAAATCTAGTTTGCATCAGTAC
LG15	ref-24909	97.567			AAGACAGACTCGAGCTGTGTGCCATCAAAAT
LG15	ref-13808_9	99.408			CGCTCTCG[A/G]ACGATGACGATGCACAGACTGCG

LG16	ref-10920	0			CTTCACATCACGAGTGCACTGCTGTATTGAG
LG16	ref-5359	1.167			CGAGTCTGGACGACACTTGTGCAAATCCAAT
LG16	ref-52902_10	1.502			NNNNNNNNNNNNNNNNNNNNNNNNNNNNNNNNNNNNNNNNNNNNNNCCTTGATTATAAAAACAGAAGTCTCACCATTAAAATCCACGTCCCAATCACACAATTATAATGTTCTCATTATATTATGTAGTCTATATGACAGACTTGCGCTGCACGTTGCACTTAACTGTATTTTCCTTTTTGAAGTGATTTCAATCATATTTCAAGCAAT[A/T]CGAACAGTGTGCATCTGAAGTGTCACGGCTGAAGGAAATAATACATTATTTATTAGCTTCTATCATTTTCTTTTTGGGCCGATAACAATAACATTAAAAATGATCATATATCGGCCGATACCGATATGGTGGCCGATATATCATGCATCCCTAAAAATCTCAAATTTCACTCAAACAATGTTGTTTTTCTCTTACTTAAGTGAAGCCCTGAGGCCGTAGGTC
LG16	ref-52902_9	1.515			TCAAGCAA[G/T]TCGAACAGTGTGCATCTGAAGTG
LG16	ref-10198	5.288	Chr16	50960357	TCCGATATGACTGTTCACACTGCGGTCGCATTACAAAACATCTGACCTGTGTCGGATTTAGTACCACATATGACACAAATCGGAATTGAAAAGATCAGATTCCATGTGGTTTGTACTGTTCACACTGTCATGGGAAAAACAGATATGAGTCACATGTGGGCAAAAAAATCAGATTTACCTGCAGCGTGAACGTACCATCTCCATTGAAATCAATGCATTTGCATTGGAAAATCTAGCTTGTAGGCGCCCTATTATCATTTCAAATGTGTACTGTATGTATTGCGATTTTTTTTTCCCCATGCTGTTGATGACTGTAATGTATGATATATTCTGCCGTAATGATGTAATGCTCATGTCTGCCTCGATGACATAACCAAAATCCTCAGTATCCTGCGGTTATGTGCACACATTAACATTTTGGTGCAATTCT
LG16	ref-62754_25	8.135			CCCATCAATGCGAGTCGTATGCCA[C/T]TGAAAAA
LG16	ref-23578_10	8.724	Chr16	50791004	AGCGCGAGGCCGATACCCCTGCGGGAGGTCACGCCGCCATTGATCTGTCTTTCTGCACCCTTCTGACTTTCTTCTTCTTTTCTGTCAGAAGATGACACGGGAAATCAATAGGAAACAGATAGCGAATAAGTTGTTCTTAAAACGCTGCTGAGTGAACTTTTACTTCAAAGGAACTTCTGAAATGAGTTGGAAGGAAGGGCTGTTTGCTG[C/T]CGACTTTGATGCTCAGGCTGTGGGACGTGTATCGTTAGATTGGGGTTAGGAGTCAGAGGAGGATTAGAGGTCAAAAAACACTTTTGATACAATGTCACAAATGAAGGCTGCTCTGTTTTCTAAAGCTCTTTGTGTTGTGTGAGAGCTCACGGACCGCCCACTGGGGAGGGAACCGAAAGGAACTTACCGATTCCGGTTCTGGTTCCGTTTGCGATTCTTTTA
LG16	ref-5725	9.561	Chr16	50680022	TAGCGAGCGGAGCGCTGGGATATTCTCATTCCACCGTTCCCTTTCTCTATTGATCTCACTATCTCATCAAGCACGTTTCCTTGAAGATGCATTTTCCACCACATCAACACTGGGGTTGCAGCACTTTGTTCCAGACATGCAACGGCACAATTGATCTCAGCCTTTCACCACGGCTGCCAGCGCACACCTCGTCCCCGGTGCGAACGCTCTGATGCTCAGATGAAGGTCTGTGAGGTGGCGGTGAAGCAGTCACACGTGAACTCATGCCCTCTGGAGGAATCTCAGCAGAAAGGGCGCGATGACGCAACCACCAGTGAAATTTGCATAAACTAAATCACATGCCGTAGGGATGAAACGTGTTCGCCTGCA
LG16	ref-31412	15.19			AGTTGGACAGCCCATACAGTATAACGAGGGACTCATAGTGGCTTGATGGTGTGATGAACACAGTCTTTCATTCATCAACCTTGTGTGTCCAAACAAGGTTCTAGGCACTCCGCAGGTCCAACTTTGTGAAAATACAAGCTCCACATAACTGCTCCAGGGCTGCAGAGACCAGAGGGAGAGCGAGGTAGTCGATGCAGGGGTACAAGCCTCCGTCCTTTTTCCCAACGAAGAAGTGCACGGGCAGTGGATGGATGGATGAATTCTAGCTTAAGAGCCTCCTCAATGTACTCCTCTATGGCCTTCTGTTCCAGGATGGACTGTGGGTATACTTTACCATCTGGTAATGTGGCCCCAGGCAGCCGGTTGATC
LG16	ref-47266	17.465			GATACTTTGAACAAGAAGAAACTGAACCCATATGGAAGCCCATTTTTTCCACACAAGAAAAAAAAATAATGTAGGTTATAATCATGACATTAAAAAGTAGAAATTATGACTTAAAAATCATAATTATGACATAAAAAGTCAACATGTTGCAGCACAGGCTGCTGTGGACGGAGAGTGGCGCATCTGGCGCGCGCAGAGTCTCTAGTTTCCTTCTCTGACGCGCTTCTGCTGCTTACGAGAAGATTACAGGCGCCATCTTGGATCAGGGGAGGTTTTAAACTGGGAAAAGTCAAAGAGCAGGAAAGAAAAACAACTATTACAACATATGCAAACCCGTCCGTTCGGGCCTGCGGTGAAAATGACACTCG
LG16	ref-65156	18.318			TTGTTTCCTCCGACAGTCGTGCGGTTATAGA
LG16	ref-40371	18.761			TTACACTGTGTTATTGAAAAAGGCTTCAGTATCGAGGAAAAAATGAGTTTTTCCTCATACGCAGTATGAGTGAAGTATCGAAAGGGAACTTGCACTAATCTGTATCACCAAAAATGACGGAGTAGGCCTATCTTAAGTTGTTTCAGCTGTCATGAGGAAAAATCCATCAAAACACGCCGGCGCGTTCATCGGTCAGAGGGTTAAAGAAAACATAGCCTATTTAGTTTCATATTTATGTTTAAATATTAGACTATAGCCTAATATTAGTTTTTAAATTACATTTATGTTGATTATGAAAAGTGAACAGCATGAATAAGATGCCCAATATGTCGGGAGACATGCAGTGGGTCAGACATGATTTTGACCACT
LG16	ref-58873	20.824			ACCCAGCTGGAGAAACACAACATGTGCGTGAGCTGAACACTAGTGAACACACAGTGATTGTTCCCCTCAAACGTTCCAGAATCCAGTCACCTGTATGACGTTTGTTTGTTTGTTTGTTTCAGGTCTCTTATGGCGCAGCTGCATAAACTGCAGGCGTTGATCAAGCAAACGGCCACCAAAGCGGCGCAGACGAGCACATGCATCATGGTACGAGTGCGTCGCTCACTCTTTAACTCATTAACACCAGCATCAGTCTTTCATCTGAACAATGTGTTCTTCCTCCTCACAGATTCTCATCTTCTCTTTGGCTTTACTCATCTTCCCGAGTTACAGTCCGTTCAGCCGGAGTTCCCAGTCACTGGAGGACAGTTATGCTCCGTCCGGAGGTGAGAATAAGTCATTGAAACACCATTCACAATAGGCTTGCCACA
LG16	ref-1900_32	21.403			TGTGGTTTTGCGAAGTGTGTGCCAAATTAGG[C/T]
LG16	ref-26181	23.017	Chr16	887631	CAGAAAAGCTTAGGCCGAAAAGGAAGTAAATTTGCCGATTTGCAATGGACGCAGACAAAACGATAATAAGATTTTGTTCTCCGCACCATCTTTAAAGTTCAGCAAAATACTGGTCTGACGTTATTCGCCATCGTCGCTTTTTCACTGACGTACGATTCGCAATATCCGATCTGTCCACTTACATGGCAGACGCAAATGCACTCATGTTCGCGAACAGTATACCACTGGTCACTTTTTTCGAATACCCCTAAACAAAGATTGAAAATGAACTTTCCGGCGAGTATATTGGGACCTTTAAGGGTTGAGCCGGCATTAAAGAAAGCAATCCCATTT

					GAAATGGTCAAGGTAATTGCTCCTAACACTGAAACACTCCACTCAACAAAGGCCCACAAGGACAGACATTGTCATGCAGAAAACTAGCCTTTGGAGAC
LG16	ref-50727_28	27.393			CTGGTCACAGCGATACGGCTGCACACA[A/G]GAAG
LG16	ref-56846_24	27.469			ACATTGCAGACTCCCTTTGCTAAGCTGCCCTTTGAGTCGTAGGGTTGCACCCTCCCCAACCTTCTTAGGTATTAAAGTAGGGTCCCTTGCTCCCCTTTTTAAACCCCAGTATGTGAACCTAAAAAGCAGAGGCACCTTGCTTAAATACACAGTACTTATTGTTATTCTGCACTTTTCTTAATAAAGTGATGGAAATCTGTCTGGATTCTACGAAGACCATGCAG[A/G]CAGGGTGTGAGCATTCGCCCCTTATTGCCTTGAAGTCATTAAGTAGAGCTGCAGCTCCCCTGAGGTTTCCAGGGGGAGTTTATTGCAAGAATCTGTTGCATCTAAGAATATGTGCCATTCTTGTACTGCAGCCTCTTAATCATTCTTTAAGAGCTGTGTCAAGCGGATAGAGGCTGTCACAGCTGAGCCATTTTATTCCTTAGAGCT
LG16	ref-16827_18	27.723			GAAAGATTTCTATCTCATGCTGAAATTGCTTTATTGTGGTTTGCTTGGAGGGTTGAGGTCAGTCTAATCTTTAACCTAGCAAATAGCATGAAACAGCTGCAGAAATACTCCTTTCTGGAACAGAATCCATTAGAGATCGCTGTAAACCGACACCTCAAACACACTTTCACCCAAACAAAATGAGCCTTGCATGATTTTTAAGGGGCGCACCGATGGCG[A/G]TGCTGGATCACTTAAAGACATGAGCCAAAAAATACAGCAACAATTAAAAAAAAAAAAATCAAATAAAGTAAGTAAATAATACACTACCGTTTAAACCGTTTTTTGTTTGTTTTTTTCAAAGAAATTAATACTTTTATTCAGCAAGGATGCATTAAATTGATCAGAAGTGATAGTAAAGACATTTGCTTTGTTGAAGTTGAAGTTATTTCAGCTT
LG16	ref-44868	29.409			TGGGGTTTATGTGAAAGTTTTTGAATGGTGGCTTGTCAAAAGGCACGATCACGTATCCTTTATTATCTTTTGCCAGAAGTTCATCAACAACCTGGGGTTCTTTTTTCACGGATTGAAGATTGGGACAGTTGAACGAGAAGCCAGGAAGATAGGAAAGACCTGCTGAAAAACCATTGATTAGTCCCATTAACAGGTCAGACACGAAACAACGATTTGGATGCTTTGTAAGAGCTTTGAAGAGCTCTTGGACATTGACAGGAGTTTTTATTTCTTTTTTATGAAGTGTGTGCGACGTGGGCATGCAAATTTCGGGTGGCTGTTCCCGCAGTAACTGCAAACATGAAAGAACTTACAATTAGGTTGTGTACAGACATTTTCATTAAAATTGTAACATAAAGGTGTTTGTCTTTCGGACTTCACCTGATCAGAG
LG16	ref-69995_16	32.591			AAAACGTTTCCTGACTTTCTCCTCCAAAACATCCCAAAGTAGCTCAATAATATTCAGATCTGGTCACTGTGCAGGCCATAGGAGATGTTCAGTTTCATGTTCATCAAACCATTCTGTCACCAGTCTTGCTGTGTGTATTTGTGAATTATTATCTTGATACATGCCACCACCTTCAGGGTGCACA[C/T]GGTTCGCTAGTCCTTGGCAGTGACGTGCCCATCTATCACAAGTAGTGGGCCTAGGGAATGCCATGATATTGCAGCCCAAACCATCACTGATCCGCTCCAGTGCTTTACTCTGGGCAGCAACAGACTGGGTAGTAAGTTCCTTTGGGGCTTCTACACACCATAACTTTCCCGGATGTAGGAAAGAC
LG16	ref-942	35.349			GAGTGACTTCTGAGTAACTCTTAAGAAATTAATCATTTGTGTGCAGATTGGGTTTATGTAAGAGTATTGATCATGATGAGGTTAGTGCACCTCACCTGCTCGACTGGTGAAGACTTGTCATTCTCTGCAATTCTGTCCTCTCCATCATCTGCACAAACACAGACATAAAAGATAAAACAGCAGCGCCATCCAATGGTCAGGATTAAAACTACAACGAAATTGCTGCAGGTCAATAATTACAACTACTTTGCGCATTCACACACGGACACATAAGATCATGTATATGAATCCTAATTCGATGCTTCTATGAAAGCTTTGATAAAAAAAAAAAAAGTAAAATGGCTTTCATGCCTTCATGCTTTACATTTT
LG16	ref-2867	36.032			ACAACAAACAATATGTAATAAAACATTGCCAGATTCACATTCAACTGTTTCTGATAAAACTGTACATGCTTTTAGTGCCACTCACTGGACAATTCACTTTGAACAATTCACAATTTTCACTTTGTAATATAATTTTGCAGAAATGTTGCCACAGGTACATTTCCAAAGAGCCAGTGTTGGCAGATACTTCGGACGGTGGTCTACAACCATAACAAAATGTCAATTGCTCCACAGAGCCCATCACAGGCACCAGCATGCCGAGTTCTGATGATTGATAGTCCTGTATTTTCTGTACCTTCCCATCCTGTGATTTCCCAACCAGAGCCTGTTCATAACATGGCCACCACACCTAAGCCATCAGCCAAGATG
LG16	ref-67451_31	37.391			ATTCTTATTCTTCTGATATCTGATAATACTGTAATAGTCTGTTGTGCATTTTGCATAGCAAACCTGTGGACTCAAGTAATGAAAAATGCAGCAGCGTTGTCTGGCCTCCATTCCTTCATTGGGATCTGTGATCTGTGTGCGCTATTGTTCTTGCACTTCTGCATTATTAACGTGTTGTGGCATTGAAGTCGTTTAAGGT[A/G]GAAAGTGTTTCTAAATCGCCCCTCTGTTGTTCAGGGACACGACAGTGGCAAGTTGAATCAATTGAATGGGTCATTTGAGACGTTTCTTTCCCATCAGCCCCTGTTCCCTCTCCCCTTTGCTTCCTGTGCTTGAAGCAAGCGCCACTATAAGACAAGGGCATTCCTTCACA
LG16	ref-36807_32	37.784			ATCCTCTGAACGATTCATTTGCCTGGTTCGT[A/C]
LG16	ref-36807_30	37.961			ATCCTCTGAACGATTCATTTGCCTGGTTC[C/G]TC
LG16	ref-33817_9	38.207	Chr8	6483515	AACAATCAATGATGCAAACTTCATCAGATGTTAAAATAAAATAAAGCAGCTCTACAGAAGACAATAGTGTCGTTATTCAGCTCATTTCACTTCAAGTTTTGTTCTCATCCAGTCGTGTCAGTGCAGTCAAACCGATAACGTTACTGAATGTTAAGTGTCTTTTACAGCCCAACTAAGCAAGCCAAAGGCGAGCGTGGCGAGATTCATT[C/T]CCGATCCACGTGCTGCGCACACGTTTGGCTGTGACAGCGGGCCTCAGCTACACAATATTAACTCCACCGGTCTTTGTGAGAGCAAACAAAAGAAAGACAGCTCTCTCTGCATGACAGATCCTTTCTTTATCCGTGGAGTTTTCCTTCCTATTATGTCAAAACCTGCTTGCTTGTGATTTGTCCATCACCTGTGTGACTGTTACTAATGGATCATCCTGTACAG
LG16	ref-29748_27	38.258			AGGGCAACTTGCTGCTTGTTTGTGCAGCTACTGCCGTTTTTCCAAGTCCTCAGTACCAGAAGAAGTGTCAGTACTATGACAAGCCTTCTTCCCTCACCTGGAGAGAGGAGATTTAGATTAAGAACAGCAGAAGAGGACTGAGGGAATTTGGAAGAAAAGCCTGCTGAGCGGAAGCGTTTGCACACGCATCGGCTCA[A/G]AATCAGGTGTGAGCTGCACTTCACTGGTTTTTGTGAACCATTCTGCAGATGAGATTGACTTGAAGAAGGGAAGACATGAGGAGTTTCACTGTAGGCTGATGGCTCAGATGTGTGGCTTTAGATGCAGTTTCTTAGCTACTCCGTTCCATGTGTCTGTCATGTTACAGAAATTT

LG16	ref-38964_31	38.433	Chr17	41049147	TCCTATGGATTGGTTTGCAGCGATCGAACTGAAGGATGCATACTTTCATGTCTCCATTCTTCCTCGACACAGACCATTCCTTCGATTTGTTTTAGAGGGGCAGGCATACCAGTACAAGGTTTTCCCATTCGGGTTGTCTTCACGAAGATTGTGGAGGGGCATCTTGCTCCGCTCAGGGAGCAGGGTGTTCGTATCCTCA[C/T]CTACCTCAATGACTGGCTGATTCTAGCTCATTCTTGAGAACAGTTGTGCGAACATAGGGACCTGGTGCTCAGGCACCTCGCCCATCTGGGTCTTCGGGTCAACTGGGAAAAGAGCAAACTCTCCCCCGTGCTTTTCTCTTTTCTCAGTATGGAAATAGACTCGGTTGCCA
LG16	ref-68349_2	38.692	Chr16	50737043	GTTTTCAACATTGATAATAATCTGAAATGTGTCTTGAGCATCAAATCAGCATATTAGAATGATTTCTGAAGGATAATGTGACACTGAAGACTGGAGTAATGATGCTGAAAATCCAAATCTTTCTATATATATATTCACATTTATAATTTGAAAGGGAGACAATTTTGCTTAAACTTGCCGGTTTGTACAGAAAGATGTGTG[G/T]AAAAGAGTCGACCTGAGTGCAATCAAAACAGTATTGGATGGAGGGGTCTGACTCACATGGTCCATCAGTTCTCGGACCATGCCGTTCCACTGGCCCGTGCTCTCTTCCTGCGCTCCGTACTTGCCGTCCTCCACCAGTCGCACCTCATAGGTGAAGCCCAGGATGGCAGCCAGCTCTCGCAGCAGGTCGATGCAGAAGCCCTCGAAACGATCGTTACCGTATAGCGGCTT
LG16	ref-48846	38.73			AATCGATCAGAATTAGTTATAACATATGCTTGAATGTGGGATGTAATGCCATATGAGATTAAAACATGCTATATATTTTTTAAACGTATTAAACTCACTACAATATTATGTATCTCATGACTTTATATCCCCCATTGAAGTATATATTTGGAGTTATTTTTTTTTTAATGAATAAATTATATAAATAATGTTCGTTGTACGTGATTCGATCAAGGGAATTGCTGAATGAGTAGCTGCGTAGCCTATACTGTATGACAGCTGGTAAAATAAACCCACCTGCCGGTAAATTCGAGCAACGGTCTGAGTGGCCGTATAAGCTACAAACCAGTAGAAAGCGGCCACCTTGTAATTCTCTATGCAGGAAAAACCCTAATGTAAAAATGGTAGATGTAATAACTCTCATCGGGAACGAGGAGATGTTATCTACCTCT
LG16	ref-60054	39.015			CAAGCGTACACGAGAATCTTGCGCATGCCAG
LG16	ref-37915	39.35			TAATTTAAACAGGGTTAACAATTAATCCTGGGTATTCATAAGATGACGTTTCACATTGTACATTCGTAAACCCTGGGTTAACATTCTTATTTGCATGTTTGCGGAGTCAGTGTCATTGATTGGATAAACGCAACATGTGACCTTTTTACATAAGCTCAAGCGTTTTGCATCCTCGAATTGCCGAGGCTTCGGAACAATCTGACACCGTCTCCACGGTGACCCCTAGTGGTCAGAACAGTGTAAATGCAACAAAACGCAGGCCAAACATGCATAAAAACACCTTTTACAAATCTATTGCAAAAGGTTCATTGAAAGTAAAATCTCAGACAGAGTAATCAAAGGAACTACAAACAGGTGAGAATCACAGAG
LG16	ref-15392	39.393			CTACGTGAAAATAGCAGTATTTTGTAAGTACTGTAGTTGTTAGATGCTATTTATATTTAGTGTAAATACTGGCAGATACTTTTTGCACCTCAGTGTATTGTAATATAGCACAAAACAGTATGCAATAATAACAACATATTCAGTGTTAATGATTATATTTATTAAAATCGTGAATGGATGCAGCGTTATTGGGACATTACTTGTCACTCCCCTACACTGAATTGGATTGAAAGATGCATGAAGTGTATGCTTCGCATCCTCCAAACACCCACAGTTCTTTGTATGTATTCATATTCACAGAAAAATAAATAAAAAAAAATTGGCAGTGGCTATTTGCACTAAGTGAAAATACTGACAGATGCGATTTAC
LG16	ref-24558	40.435			TGATTTAAGAATGTTCCAAGAAGCATTTTGTGCTTTTTTTTCTTCATTTTTTTCTTTTTTCATTTTAGACCACGTTCTTTTTTTTTCTTTTTTCATTTTAGACCACGTTGTTTCTTTGTTTTATATTGTCAGAAACACTAAAACTCAGTTTATTTTCTCAGTTTATTATTCTCAGATTTGCAATGTGGTCGGAAACTCCTCCACTGTACAAGACAATGTTATTTTATTAGGACAGTCTACAACAGTCTGTCAGGAGACAAAAAGCTCTCAAAAGGACCACAAATGGATTAGTGCATGGCTGGAACCAGGCAATATTTCACTTAAGTTTGGCCACACAGTCCTCCTGCTGCATGACAGAGTACCACACTC
LG16	ref-8240	41.755			GAATTCACATTCTGAGCAACTAAAACACTTGACCTTGCCTTCAAATAAAAAATATGGGACCGTCACACAATATTTCGAGCTAGACAGCATAGAGAGTCTTGTTGTGAATGAATGTTTATTATGCTAAAAATTCTGACTTAAAGCATGGTCACATTTTGTAGAAAATTTGCAAGTACATTTTTTTACACCTTTGTTGCCGTGTAGATTTGTCGAAAGTACCGCCTATCGGTACTGAAATTTTAAAAATGTGACACTTTGACAACAGTTGTGCAGATTCATAAACACCTCTGATTGGCCATTGTGTTGACGCGCTCACCGGATATTTTTGTGATTGGCTACAATGATCAACACACAGTAGCGTTTGAAAGCACACAGAAGTGTTTGAATTTTAAAAGCGGGAGCGTTTGAAAGCAGGCGTCTATCAGCAGACT
LG16	ref-14647_14	42.702			GGAACAAAACCGA[C/T]GTCATTGCAATCTAAAAA
LG16	ref-10784	43.394			AAAGTCAAGCCGACTAAATTGCTGATGAGGT
LG16	ref-51441	44.405			CCGCTCTGTTTCCTTTAGTTCCTGTTGCCTCGCTCTGTTTAGTTTCAGTTTTTTTAATTACTGATTATGTCCTCGTTTGTTTCTATAGTTATGTATTAATCCAACCTGTGTGTCGTTATGTTCACCATGTATATATTCTACCTGTTTTCATTGATTATCTGAGCCATGCATGTTGGATTGCAACCACATCGTGACAGAATATCCAGCTAGAGCCCACTACTGCTATAGAGCTAGAGCCAGCCACCATGTCTGTTCTGAGGCCAGAGCTTGTCACCAAAGCCATCCCAGAGTGTGCGTATCCTGCCTTGTCCATCCTAGAGCCAGGAGCAGACCATTGGCTAATAGATTCATGGTCCGAAGATCCAGGTC
LG16	ref-46065_3	44.633			CC[A/C]GGCACAGCGACATTAATGCAACCAATGGT
LG16	ref-39396	45.286			CAAAGCTATAGTCTGAACTTTGAGCATCACATTATAACACATTAGATTGGGTTGCATTGAGTTTAATGCATTGAGGAACTTTAGCCATGTTTTTAGATGGAATGCGATAGCCCAAATTTGGAATATCGCATAAAACACTTGTGAATAAAGCACCGTTTCCATCCAGTGAGTCTAAGAGAACAAAAGTGTCGCTTCCTGATAAACTGGTGCTAAATATCACTGAGAAAAGTAGGTGAAGTCACTGTATATAATAATTTTCATATATAATGAATTACTTGTGCCTTAGAGCACACAGACGAAACGCAATAAACGCAGTTGCTTTTGGAGGCAGATGCCTGCTGTTGGGGATCACAGTCATGTAAGACAGTC
LG16	ref-13218	45.441			GTTACGATTGCGAGACACTTGCCTTTTTATG

LG16	ref-58432	45.964			AAATCATGTTCGATTGATGTGCAAGAACCAA
LG16	ref-46065_28	46.25			TATATAAAATCAAATATTTCAGCTGAATATTCACAAAATGTCACATTACAGAAGTTAAACGCATTGCAAATGTCGTTTGATGCCTTTAAAGCTGAAATATGTCATTCCTGAA<br>TCTACCCCCCTTAATACCCCAACACAACCCAAATTTACAGTCCCACCCCAAAATTTCACCATTGGTTGCATTAATGTCGCTGTG[A/G]CGGGCTTGCCAGGATGCTTAAACA<br>AACTGAGCAATTTTGATGGCACCAAAAAGCTAAAGCACTACTTAATTGTGTAGTTGTCCTTGCATAGTTTAGTTCACCCAAAAATGCCAATGTCATTCCAACCCTGTATGGG<br>AACATAAAATAAGATATTTTGAGAAATGTATTAGTGT
LG16	ref-40381_18	46.278			GAGCTCGCCACGATTAA[C/T]GTGCCAGCTTCAAG
LG16	ref-51215_26	46.487			AGTTACAACTCCACCACTGAAACCGATGAGCTCCATCAGAAGCAAGCTGTGTTCGCTGTGCGGGTGCGTTTGTGTTAGTCAGACAATCTCGTCTGATTCCAAGCCGTACTC<br>CTCATAGAGAGCGTCCAGGATGGACAGCTGCTTCCCCATAGCTGGCAGGTAGCGGTCCAGATCTCGATGCATGCCGATCGTAA[A/G]ACCCTCCTTCAGCTCATCACCTTCA<br>TGAGAAACCAGTGCAGCCATGAAGCCCTCATAGGACGGGGCGGCCCGTAGAGCCAGCTAACATAACACAAACACACAGAGTCAGAAAGATTTTTCCAAAACTGTATATTG<br>TTATTTTTCAATGGAGCACAAAAAGATATTTTTAAAGGGG
LG16	ref-14788	46.756			AAAACGATAGCGAGAGGCATGCCTTCTGGCT
LG16	ref-66477	46.77			ACACGCGCACATGGCAGCCATTGTAAGTCCACAAGACCGAAAGTGCATATGAAGTGTGCTAATTGGGACTGGGCTTTCAGGAGCTGTTTGTTTGTGTTAGCACGTATAAAG<br>ACAACTATCATGGATAAGCATATTGCTGGCGCCATCCAGCCAGGATGAAGTGGATTTAGTTGATGAGGGCAAAGATATCACTTGCGGTGTGCCCTCTCAGCCTACCAGCCC<br>TGCATACGAGGAACTGCTGGAGGTAATGGCTTGCACCATGACTAGATTGGATCTAGTTTGGGGGCACAAGAGACAGAAAACTTTCTTCTTGATGCGCCAGTCTTGCCCTCA<br>GAACTTTTTGGCACATCTGAAGACGGTAGTCGGAAA
LG16	ref-48743	46.907			ACGTCATTCACGACAGGTTTGCCACAATACA
LG16	ref-47302	47.198			GCTCATAAAGCAATTAAGTTTATAGAGTTATTATATGGGCAAATGCCGGTCGTGATATTTTAGACTTATTTTCAATATAAAGCATTTCAGTTTAGTTTGGTTTATTATGGGCT<br>TGTTCTTGTTTGCTGTTTTTTTTTTTTTTTCTAGCAAGATCTGGCATCACAAACGAGTCAAGGCTTGCATCGCTTTCGCTGTGATTTTCTTGCACTGCGCATACAGTTACGTCT<br>TTAACAACTAGTTGTTCAGTTTGGTTCCATACACACCGTGTACGATTTTTGTAAATGCGGTTGTCACTCATGTAAATTATAAAACTCTGTGTGTACAGAACATGATTAAGCC<br>CTAAAAGGAGAACTGGCAACCGAATTTAAC
LG16	ref-13309	47.976			AATCTTAACCCGACTACTGTGCACTCCCTGA
LG16	ref-59402_15	48.425			GCTTCCTGAACAACAAGAGGATGTTTGTGACCTCTGGACATTGGACAGTCTTATGGAGACCAGTGTGGCTGATAAAAGTTTCTCACAGTGCAAGTCCTTCAACAGTACAGG<br>CACAAATCTGAGAACTGAAAAAACCTACAAAGCTGCAAAGAAAAACAACCCCGGACACACAATGGAAGGCAAC[A/G]GCTTCGAAAGGCAACCAGCATCTACCGATTTA<br>GAAAGCTTCGAGGAAAGCGTGTCCTCCTGGTTGGGAAGCATTGAAAGAACAAGAACGTATTCCAAACCATGTCTCACCAAATACTCTGACATGAAGTTGCTCCCCACGGG<br>TGTTGATGTTTCAGACCTGTCACCTCCTTCTCCGGTGATTTCA
LG16	ref-20466	48.984			GTAACACTGTGAATGCAAGCATCTGTTATAATAAACCCATCCATCAGGTGCACTTTTTGACATGACACATCTGTTTGATGCTGTCTACGCATCAAAGTAAACATTATAAACT<br>GATACATATGTCTTTCTGAATGGGTGCATGATATTTGTTGTATGATATCTATCCTTTAAGGATGAGACTATTGAAGTGTCCATCGTAGTGCATTGCAATGACCCGTTTGCTTT<br>GACGTGTTCATGACCTGTTTAATCTTTGAAGGTCTAAGGGTATATTTACACGACAACAATGTACTAAAAACGGAAAAGTTTTACTTTGTGTTTTTGAAAAGTTTTGCATACA<br>CATGACAACATTGACAACAAATGATCACTGTTCACGCCTATCTGCAAAAACAACAAAATTATGCTTTTTGAATTTTAATTCGCTGGCAAGCCAT
LG16	ref-9875_18	49.089	Chr16	10556995	GTTCCTCCTCCATTCCTGGTAGGCACACACACATATTGTGATATCTTTGACCCTGTTCACACCAGGTATTATTTTAATCTTTTCACAAATATGATGAAAGAGTGACATATTGT<br>TCTTTGGTGTTTTAGGGGATGAAGCGCTTTGAGGCGAGGAAAACTGTGCTCCAGGCACTGAAAGATAGAGGGCAGTTTAAGGAGGTCAAAGACAATCCGATGGT[A/G]GT<br>GCCCGTCTGCAGGTTAGACACTGACTTCCACTTAACTCCTTAACTTTCAGTGGTTGTCAACTGGTAGATCATTGGGCTGGTCTGATAAGGTTGTGGACAACAGGGAAAATC<br>CAGTGCTAAAATTAGAAATAATTGACTAAATGCCCCTTACATTCCCTGAAAGTGTCATATAGCTTGCAAATCTTTATTTGCAATGATTTATAATAAATCAA
LG16	ref-11795	49.731	Chr16	21362867	TAAAGTGTACTTATTTTTCACAAGGGCACTAGTTCACAATGTCATCACCTGAGTTCTTACTTAGCAACAGTGGTAAAAAGATGCTGCTAGCGTGCTAGTTCAGACCTAACTG<br>ACACCATGTGAAGAGACGTGCAGGGAACAGCCAGTTGGAGGTGGAAACAAACTTTTGTGTGAGTCAAGCAATCAAATCGAGTCTGAGAGGGTGTGTACTGCTCACCTCAG<br>CATCAAAGCGGGGATCCTGACAGACGTCACTAATGTTAACAGGCAGTCCGGTTGATGCAACCAACTCAGCAATGCTGTTATTGATTAGCCAGTCCGAGCAAGACACCTTCT<br>CCATGCTGACTTTGTCAAATGCACACACATACACAC
LG16	ref-59402_17	50.407			GGTTGCCTTTCGAAAC[C/T]GTTGCCTTCCATTGT
LG16	ref-5686_4	50.916			AGA[A/C]AGCCCGCGAACAGTTTGCTGAAGACAAG
LG16	ref-56721	51.031			ATTTATACAGCGACGAATATGCAGTGTCATT

LG16	ref-5686_10	51.395			AGAAAGCCC[A/G]CGAACAGTTTGCTGAAGACAAG
LG16	ref-6280_9	51.71			AGAATACT[A/G]CCGAATGGCTTGCTTCCACAAAT
LG16	ref-17812_5	51.867			TGCAATGGAAAGGTTCCATAAATGTTAAAGGGTTAGTTCACCCAAAAATAAAAATTCTGTCATTAATTACTCACCCTCATGTCCTTCCAAACCTGTAAGACTTTCATTCATC<br>CTCACTCTATTGTTGATGACAGGGGTGACGTGCTTGGTCTGCTGCCTGGTCGGGTTTATATTTGTGCAGGGCTCAGGGAACTACTGGCTAGCC[C/T]TGTTCGACAGCTTTGC<br>CGGCTCGATTCCTCTCCTCATCATTGCTTTTTCTGAGATGATTGGAGTTGTGTACGTCTATGGGATAGACAGGTAGGTCAGAGCCTAGTACTTTTGTTTTTAACTCTTACAGC<br>ACTTCTCGAAGAGACAATGAGAACTGGTTATAGGGCATAAACAAGATAAAGAAAATAAATGATCAGTATCATCACTACACACAGTATCAGCAATTCA
LG16	ref-22034	52.279			TGAAGCCATACGATAGCTTTGCACAACACAT
LG16	ref-18643	52.344	Chr16	15372571	CTAATCCACCGACCTCCTGCTTGATAACTTTGACACATCTTTTGCGGTTCGCAATCGCTTCGGGCACTTGTGAATCCGTAAAAGGAGTCCATACTCCGGCTTTGGGACTGTT<br>CGTGGGGTCCGGCTGGTCGTAGGAGCCGTTGGTGGTGATGCCGTTCAGGTTAACGCTGGTGTCTGCCGCGGGCTCCTCGCAGCTGAGCGTCAGCGACTCGTCGCTTAAACT<br>CGCCAGCACCGCGTGCCATCGCTCCCGTACGAGCACCTCCAAACATGCACTTTTCTGCACTCTGTTTCCACAAACCGCCATCTTTGAAGCATTCTTATAATGCCATGTGATT<br>TCATAGACACGGACAGGACTTTACCCCCGACTAG
LG16	ref-53895_5	52.692	Chr9	26150463	GTTTTTACTGTATTTTTAATTAAATAAATGCAGCCTTGGTGAGCAGATGAAACTTCTTTTAAAAACATTAAAAATCTTACAGATTCCAAACTTTTGACCGGTAGTGTATATA<br>TATGTAAGCAATAAGGTACGAGAGGATGTGCTGTATCATGAATAAGTCATGGCTGAAGGGT[C/G]ATACAGCACTAGCCTCGAGTTGCTTTTATAAAACAACCATATAAAA<br>CAACCTTCCTTCCGCCGGAAAAAATAGTCCCTGACTGTGAACAGCAACAGAAGTTACATTTTCACGCCATTAGATGGCGGCAAAGACTGTCTTCATGAGTGTCAGTCAGTA<br>GCGAAGACTGAATTGTTGTGAATACGGAACAAAACGCTT
LG16	ref-67237_26	53.128			TTCTTCATCTCGATGCGCCTGCAAA[C/T]GCACCG
LG16	ref-33306_2	53.213			GTAACTCTGCCTGGTGGATGGGACTTGAATCAGGAGTAGAGGGGTTTGTTGAAGGGGTTCCAGACTGAGGGGAACTTTGAGGTGTAGTTCCGGAAATCTTCAAATGGTTCT<br>CACTGTTATTTAGATTTATAGTAAAGGTATATTAGTAACTTTTTCATGATTACCATTCAGATCACAGAGTAGCATCTCTTAAGCTGGGTG[C/T]GTACTGTACGATTTCTATG<br>CTAGCTATGCATAAGTCTTAGATCACATAACGCTGGCTTACACTACACAACTTAAACCCAGACTTTTCCGCAAATTGCAGTTGGGACAAGTCAATGCTAGTTGCCTTGAGTC<br>AGAGTTAATCTACACATTTTGGGAGCTCGGAGTTAACAGATTTCACATTGTGAATGATGGTTTCAGCCATCTAGTCTGAGGATTTAGTTGTACAGTTCA
LG16	ref-23796_1	53.617			CATGCTTGACTGTAGGCAAGACACAATTTTCTTGGTACTCCTCACCAGGGTGTCACCACACATGCTGGACACCATCTGAGCCAAACAAGTTTATCTCTATCAGACCACAGA<br>ACCTGGTTCCAGTAATTCATACTCTTGGACAGGTTGTCTTCAGCAAACTGTTTGGGGGCTTTCTTGTGAGCCAGCTTCAGAAGAGGCTT[A/C]CTTCTGGGACGACGGCCAT<br>GCAAAACTGACTTGTTGCAGTGTGCGACGTATGGTCTGAGCACGGACAAGCTGAACTTCTGCTTCTGCAACCTCTAAAGCAATGCTGTCAGCACACATGCATCTCTTTTTGG<br>AAGCCAGCTTCTGCACCTGACGCACCGCACGAGGACTCAACATCTTTGATTCAGCTTCTTTGGCCTGTTCCGAGTGGAACCTGTCTTGGAAAACCTCTGT
LG16	ref-31742_9	53.911	Chr16	37535041	AGTGACGCACAGTGACACAGTCACAAACATGTCTTAAGGTAACAAACAGTTACAGTGATGGAGTCAGGTATATCACAATAGCAAACATGCAGCGCTGACAGCTATTCAAG<br>TGCTACAATGGTGCCGAGAGTGACACGCGGCGCAGTGGGGGAGTTAACGACACTGCTCCGGAGACGCAGGCCTTCCGAGATTTGAGGGGGCTTTACTC[C/T]GCGACAGA<br>GATGCAATGTCAGATACAGAGGATTATGCATGCACTTCACATCAAAGACAGCTTTGTTCTCACAAGAGAACATAGACAGGCGGAAAAAGAGCGACTACATTCATTCATTC<br>GCTATTACAGACCCCTTTGATTGAAGTTTGTACACAAAATGGCCTTAAACCGACGCGGCACAAAGCCCCAAAAAGTCTTTAAAAGAGCTTTCTTGTACGAAATTG
LG16	ref-15731_23	53.988			TCTGGGTTCAAAAATGTCAAATTTTGGTCAATTCTGTAAACCTCATTTAAATGAATACAAAATAAAATGGATCAGCTCAAAAATTTAGATTGACGATCAACATTTTTAACTT<br>TTAGTCAAAGTATTTTAAGGTCTTTCTGCTGTTATAAGAAAAAAAAAAAAATGTGAGAGAACGCACCTGCATATTCGTCGA[A/C]CCCTCTATAATTTTTTTCGCCTGTGTAA<br>ACAAAAGAGGATTTAAAAAAAAAAAAAAAAAATGTTTGTAACAAACAAAAAGCAACAACTGTAATTTCTCAAAATATTTTTTGCATTTCACAGAAGTCAGTCATACAATTT<br>TGGAACAAATGGGTGAGCACATGATGACAGAATTTTCA
LG16	ref-54863	54.303			GCGGCAAATTCGAGCATGTTGCATGTTGTCA
LG16	ref-37971	54.428			AATGCGAAATGTTACATGATATCAGTTTTTGCGTGAAGTAATACACCGACTTTGTAATGCATGTATTTCCTTTCATTTTTACTTTGTCAAAAAAAAAAAAAAAAAAAAGTATC<br>AAGGTTACTGTAGTTCCATAGTTACTGAAGAATATTTTTACTACAAAAATAGCTATGTGATTAATTTGCACAGCCGTCCTCATCGCATAGTAAATTATTATTAATACTAATA<br>AAGAAATTTTGCATATTAATATGCATTTGATATGGTATTTCAATAATTCAATGTTTTGTTGTTTATTAAGTCACGTTTATGTTAAGCACATTTAAAAACATCAGAAATTGCAA<br>AAGTGCTTTCAAAATAATTACAAATAGCCTAA
LG16	ref-57844	54.84			GACCGGACTCCGACATGCTTGCTTGCTAAAT
LG16	ref-72673	55.343			TTTGAGCATTCGACCCTGATGCGGGGAACCG

LG16	ref-52556_29	55.912	Chr16	24346075	CCACAGTGTTAACAGAAACACTTGAAATATGATCCTGCGGAATGATTTCAACCAATAAAAATATCTAATATTAACTTTACACAAGGACTCCTCTTGTAAAGTTGCTGTAAA<br>AGATGAAAGGGCTTTACTCTATAAAAAGACAATTGAATTCAAATAAACTTTAGTGTATTGCTGTTGTTACCCACAGTGAGTTTGTATCAGACAATGTGTCGAGACACTTGC<br>ATCTGT[A/T]AGCAGCATTTTAATAGCAAATTTTGATTTAAAATTCAGTAATAAACAGTATTTTTGCTACCATTCAGAATTTTTTTATGCTACCATTCAAACTTTTTAATGATA<br>GTGTGTCAAAATTGTTTAAAATAATTAAATACATTGTCCTCATGTGTGTTTATTATAATTATTATTCCTCTCACATTGGCCGATTCAAAATCAATTGTA
LG16	ref-61416	56.709			GTGCACCTTGCGACATCACTGCACTGTGTCT
LG16	ref-23622_15	57.392	Chr16	29927374	GAAATGCATGATCAACACACACAAATGTACATACATATAAAATTCAACCAAATCATGTCCATTTGTTGCAAGCCTTTCAGAAGAATTTCTGCACATAACGTTGACACACGT<br>CTCCTGAAGGCTCGTTCCCCCCCTCTCCGTGATTCCCTCCGTCCCAGAGCTCACAGGCTCCCCCTGGAGCAG[C/T]ACAATCGCTCTGATACCAACATCAGGCTGGAGCTGC<br>AGACAGCCATTGTGTGCTGCAGACACATGTCTGCTGATGACACGTCCTTCTCACCGCAACACACACAGCCATCCCTGTTCTCTCCCTTTCCCTCAGCCTGTCTCTTTCCATAC<br>GCATCTATTTCCTTTGTCTATCTGCGTGGGTGTGAGT
LG16	ref-52975	58.164	Chr16	34648983	CTGTAAAAAGAAACAGCATTTACAAAACAAGCTTTGCAAGTATTTTATTACATATGACATCATCAGAGACCAAAAGCCCCAAACCCAAGACCTTTTCCTCCACCTGTTGCA<br>ATGCTTTGTTGCCTCCTTCACTACAATAAAAGTCTGTTGACACTTTTCTCATCCTACAGAGATGAAACAATATGATCGGCATAAGTACCACCTACTTTCCGATGGCACTGCT<br>TTGTGTCTGCCCACTACACAATCAATGTTCGTGAAATTCTTAACATTACAGGGCTCATGCTGGGGGAAGTAAGCATTGAGGCCATTGTTCAGTGTGTGGGTGCATGGCTTG<br>GCAGCGCTGACTACTTTGTCTCTTTGTTTGGTGCGCCGTGACTTTCGCTTCAATGAGCTTATGCCGAAGCTGAGCTGCTCTGCTCAGTAAGTGATAG
LG16	ref-54283_28	58.36			AAGACGTGCTCTTTTTTTTGGGGAGACAAAAAGCCTTGCAAGATGTCATCTCCTTCAGAGCCAAGCAGTCATTTAAAATGTAACAGACAAATGAAAGCTTCCTTGGTTTGT<br>TTACTGAACACCTGCTGAAAAAGAGATCTGTGGTTCCTTTGACATGAATGAATTGGATACTGTAAATGAGACATTTAACATTTAGTGAACGTACAAATACGAGTTGGCTGC<br>AGTAC[A/T]GAGGACAGTTGTTGTACACTTGCTGGAAGAACTCCAAAATTTCAATGCATCGTGAAATTTTTTTCATAGTGCAAAAATAAACATTTTAGTTGTGCCAGAACTT<br>GAAAATATCCTGCATAATGCTGACATGATATTATGAGTTTTCTCAACATTTCATTTTTTACAGTGTGACCTGAGAATTAGTTTGAAGTAAACCCTGTTATT
LG16	ref-64291	59.038			ATTATAAAGCTTAGATGCATCAGAATATATATTATTATTTCTGTGATTATGTTCATCAGAAAGAAGAAAGTCATATACACCTAGGATGGCTTGAGGGTGAGTAAAGCTTGG<br>GCTAATTTTCATTTTAAAGTGAACTAATCCTTTAAAGCTACGCAATGTAACATTTGTCCCTCTAGCGGTTAAAAATAAAACTGCATGCACCTTGTGGAACGATTGTTTTGCT<br>TGTGCTCTGCTCCTCTGTGCGGATGAATGTGGTTTGAAGCACAGGTGTACACATGGATACAGGACATCTTGTAAGAGTGGATGGGCAATCAAGGAATGTTTTCATTAAAAG<br>GATATTTCATTTTATTATAGTTTTTAATTTTTTTGTTTTTACTTTTTATTTTCTATTTATCATTTTTTACTTATTGTAATTTTTTTTTATATGTTGT
LG16	ref-31077_23	59.523			CATCATCAGTCTGTGACATCATTGTTGTCTGCTGGTGCTGTTAAAACTGGCTTATGGGCCGTTCACACAGAATGCATTTTTTCACTCCACTGTGCTTCTTTTCCACTGTTTTC<br>AGTGTGAAGATGAGCTAGACGGACGTGTTTGACCGATGTGCTTGCG[A/G]CTTGCGCATGACCAGGCATTCTAAAAACACGGGGCTGGACGTGTTTGACCGATGTGCTTGT<br>ATCTTGTGCATGACCAGGCATTCTAAAAACACGGGGCTCAACTTAAAATAAGTTCAACTTTTAAAAAAGAATATGTCTTCAGACCTTGCACTTTTTTCCATTCCATTGACCT<br>GCGTCTCATTTTTTTAACAGCAAAAACGCATTCTGTGTAAACCCAACCTGCCCTTAGGGACTGTTCACACAGAACACATTTTGCATTCAACTGCACTT
LG16	ref-31077_29	59.544			CGTGTTTGACCGATGTGCTTGCATCTTG[C/T]GCA
LG16	ref-54566_1	61.521	Chr16	44176831	GACTGTACCAGTGTACAGTACAGTAAATACAAAATACGATTGAAGGCTTTCAGTCGGTGCAAAGCAATCTTTGTGCATTTTGTTTGATGTGGTACTTTCTGTTATCTCATTC<br>GACAAGCATGACAGCTTTTGAATAGCAGAAATGAGCTTGAGTCATTAAACTGTTGAC[C/T]GAGGCGTTAGCATGCAAATCGATCTGCGACGATTTCCTTCTGATATGTCCA<br>GGAACTGAGGTCTGAGTTATCTAACAGCTCTCACAGCTCCGTTACTAGAGCGTTCATAATATCAGGGTATTGTCTCTGGCTCCGAAAGGTCACGCGGAGCCTATAGTTCCTC<br>TCTCCGGCACTTCAGGAAGAGGAAGAGAGAGGAAGAG
LG16	ref-15594_9	61.977			AGTGGGATAATTATACAACAGTTTAATAAACAAACAAGTTAATACTATGTAACTTACATTTTAGACACAGTAGTGTCTCGCCAGTTAGCCTACTTGTGTATTTCTGTCCCGC<br>CAATGAAAATAGTTCCAAACTAAGCAACAGTCGAAATGTTGCGCCGCGCGTCTCCAAGCAACACAC[A/G]GCAGCTGTTTCGTCCCTGAATGAATCTTTGCTTTGGAGCGA<br>ATCGTTTGAGTGAATGATTAATTGAATCACTCATAATGACTCACTTGTTTAATGCCTGAATGAATCTTTCTGAATTCGGAACAGCATTCTACTTTTACTATTCCTGCCATGGA<br>GATATGGTAGAGTAGAAAGAGTAGTATTCTAGTAGCC
LG16	ref-13955_30	62.383			GAACTACCTGCGACAGACATGCGAGACGA[C/T]TC
LG16	ref-18360_4	62.557			GTC[C/G]TACCTACGATGTTTGTGCACTGTGGCGC
LG16	ref-26237	63.254	Chr16	46034304	CTGTCAACATGATTTCAACCTAAAACAGATCCGGACTAACAGGACAAATGGTGTATTTGGAAGAGGTTTAGTGGTGAAGGTTTGATGTTGATGTCCATGTTACTCGGATGG<br>TCCGCGTTTTGTCTGCATCACTTTCACACATTTCTGCGTCCGTACTATGTTAACATATCCAACTAAACGCGAAATCATCGCTAAAGCAACTCTAACCTATTTTTCACACAGA<br>GGACGGGCTTTTCCACCGCATTTGTTTACAGCCCCTTCGCCATAGTGAAATGACACGGGTGTTACTGAGTAACAAAAACCAGTACCGAGTCTCTTAAAGGGCCAGTCCCGC<br>GCAACACACTCAATGCGCTTCTGTGCGCTGCTTCC
LG16	ref-23745	63.295			GGACATTCTACGAACTACATGCAAGTGCTGA

LG16	ref-15221	64.422			CTTGCGAGGACACCAAGCTGTTAAACACCATCAGCTTAACTGCTCATCCTGTCAACGATGGAGAGCTCAGCCTAAGGTACCACAGATGGCAGACTTGCCACCAAAGCGGCTCAGGCTTCTCTCTCCACCATTTTATTCAACAGGAGTAGACTGCTTCGGCCCTTATCATGTCAAGATAGGCAGAAGGATCAAAAAACGTTATGGTGTAATTTTCAAATGCCTCACCACAACAGCTGTCAACATAGAGCTTCTCAACTCCATGGCTGTGGATGCTTTCTTGCTTGCTCTTCGACAATTCATAGCCAAGTGTGGCAGGCCAAAGGAGATCAGGTCAGATTGTGGCACAAACTTTTGTGGTGCTGAACGAGA
LG16	ref-46938	64.617			TTATAAACTTCTACACACTGCCAGTCAAAATTTTGGATAACTTGACTACTCCTTGTAAAGACTTTTGCCATAATATTTAGAAAAATATACATTTTATGGAAGTGTTAGGTTTTCTGGAAGGAAGGACCCAAAAGCTGACAGATGCTGGGGTGAGCCACCTGGCTCCAGGCCAACTCTAAGATGGGTAAATCAAAAGAGGGATCAGACAGGCGAGGGTTATGCTGCCTTCACGTGCTAGCGGAAATTTGATAAATCCCACTTTTGAAGTTGTGATTACGAGCTCATCACATTCAAGTTTCAAAATGGGAGGGTATTTATGTGCAATTTTTATCTAGGAAACTCGTATTTACGATAATTCCAAGAGCATGTGAAGGCAGCATTTGACTGGAGGGACAGTCCATACAATGACCAGATCCAGA
LG16	ref-18363	64.71			CTTAAATACAAAACCACTTACATGTATTAGTAAAATCTATGTAGTGTTTCTCATGTTAACCTGTGAAATGTGCTTATGTGAAGTGATTATGTATGTAGCCACAAGATGTCACTTTGGTTCATGTAGGTTCCTTGTTTTTGTGAAGCAAAATCTGTCTACCATTTTATCACAAGTTTACTGCTTATGGTTAGGGGTTTTGAAAAACAGATACAAGCTGCTTGCCTTAGCAAACGCCAAAAACAGCACGACATCATTCGCTTTTTTATGCAACGTTATTTTTTAATCTTAGTCAAATCTAGGTAGTGCTTCTCATGTTAACCTAAGTTGAAATTATTCAGACACGTTTCCTCTTAACTGAGACTAAATTATAATTCAGGTGGGTCTTCCAAGAATAATATAATCATGTGGTTTGCATTGGCTGTCAAATTGA
LG16	ref-24639	65.346	Chr18	19802325	ATCTGGTGATATTCATTACATGCAAAATGAATTTCTGAATAATAGTTTTAAATAAGTTGCAGTCTGAACTGACTCACAAAAATAAAAAAAATCAAACTTACCAACTCTAAAGGCCCATTCACACCAAGAACGATAACTATAAAGATAATTATAATGATAACGATATTAGCGTTTATTGTGCAGCAATTTCGTTGTGCCACTTTAAAGGGGACCTTTTATGCAAAATTCATTTTTACATGGTGTTTGAACATAAATGTGTGTCGGCAGTGTGTGTACACAACCACCCTTATAATGGTAAAAATCCATCCACTTCTTTTTTATATTCCCCATAAATCATAAACTGTCTCAAAATGAGTCCTTCGTTTTT
LG16	ref-40264	65.579			CAGCTTTCCCGAAGAAGAGAGGATGCTTTTTGATTGAAATGAGGTTAAAGTTACAAATAATCGTGCACTTGATGAAAATGTAAAAAAGGAACTTTTAAGAGAGACTGGCCCATGAGATAAAATGCATAAATACGTTGTGCAAGAAAGAAAACATCTCAAGGTCGGTTTGCATAATTAGGGGAGAGGAAACTAAAAACTAACAACTAGGAACGACGCTCCTACATGTTAATAAAGAAAACGCAACTGGGACGAAAATAAATTAAGCACATTTTAAAATAAAGAGCAGTAGAGCACTGAAGAGGGAGAGCTTTGAAAGAAACAGCGTGTTGTCATATATATATCAACAAGCAATAGGCGAAAATGAGCAAAAATGTCATTATCGGTATAGAAAACATTGACATTTTTTTTCTTAAAACATAGATTAGCCTAA
LG16	ref-30547	66.421			GTGTTTTCCCCGATCTTATTGCTAGCTGTGC
LG16	ref-50843	67.169			GTCCTCTCACTGTAAGCAGAAGCATATATTATCTTCTCTTTTTTCAAATTTTCAGAGTATGTCTCCAATCCATCAGGCACAAAAGCTCAGGCGAAACATTAGAAGGACCATAATGATGATTTGTGTTCATTGCAATGATTATGACTCCAGGTTTTCATCCATTCCACAGGGCTCTCACAGTAATGTGCTGGATGAGATCTTCAGCAATGACGATGGCATTGCTGAGCCATCACTGAGAAATGAACAAGAAGCACTGAAGCCTGAATCCTGGCACACTGGCTTTCCATTCTACAGTCACAGTCATATGCAGACGAGGCCATTCCGGCTCCAGCAGGCAGCAGTCACCTGAATTCACACTAGTAAGAGGGAAACTAAGAGGTGCTCTAGGATCTAATCAACTATTCATACAGATGACATATTTAAATGAGAAC
LG16	ref-60655_14	68.3			CTAATGTACATCTTGAGACAAAACAATGGCAGTGACATATTTTAAGATAAGTTGCTGTCAGCTAAAACAGCTCAAACATGCATTTCACTCTGGAACCATAGGCTTAATCCTGGTCTGTGAAACCGGAGGATTGAGTTTGTTTCAGTAAGGTTTTTGAGCTATTTATTTTCTGCCATCCAGCA[C/T]TAGCGTCGTCTTTTAAGAACCCCAAAATCGGTCAACCAACAAGTGATGTCGTAAGGAGCTGTTCACACAGGAGATGTTCTTGCAATCATTTTTGCAGTGTTTTTTTTTTTTTTTGTACACATCCGCTGTTGTTCACATACAGTATCTGTCCTTTTTTTAAGATGCAGAATCAAGTTAAAAGTAGTTCC
LG16	ref-10725	68.572			TGTAGCATGTTTGGTACTTTGTGGCATATTGAAATGTGTTTCTGGGATTGAATTACAGTGTGAAGCATGCCATTTCCTGTTGCCAGCAGGTGGCGCTATGACTAACTGAATATTGCATATAGATGTCTTTATGCCAGGACTCTTAGTTTGGGGCAGATCGGACTCGGTATGCCTGACTTACAACAGCTTCGTCTTTCATGGCGAAACATCAGACTTTATCAGGCTGCCACGGACAGCAAAAACTGAAGATCTTCACAATTTAACATCGCAAAGGCCTTAAGATTAGACCAAATATGATGTTGATCTGGTTAAATCTCCATTAGGAGTTAATCACTGTGCAAAACATGTCATTTCCTGTTGCCCGCAGGT
LG16	ref-28294	69.101			AAATACAAAACGAAAGTAATGCCGGTGTTGT
LG16	ref-38057	70.194			AGGAAGAGCTCGACATTAATGCTTGTCTGCT
LG16	ref-54946_18	70.762			TCTAGAGCGCCGAATGT[C/T]GTGCAGTGTTGACG
LG16	ref-21183_14	71.434			CTTTCCCCCCCGA[C/T]TGCTCTGCTCCCAAGAGT
LG16	ref-19897_27	72.147			AGCTAATGGTGCTTGAGTGATGGGCAGGTCCAACTGCTATATATCTTGGCGCCAAGCGCCAATACCTTCACTTCGAAGGCTACTTTGCAGTGGAGAACGCTAAAGAAGCGATCCTACAAGGATGTTACGTGTGTGGTGGGCCTATTGCCATTTTTTGGAGGATCTGCCAATCAAGACACTCAGTGCGAGGCTTGCTGTTGTCCTTAACAGCGACCCACCTGCAGTT[A/G]CACCTCCCCTTCCCACAGGTGAGGAGGCTGTGACGAGCTCACCCATGTGCTCATCATTCTCCGGAGCCCCCAGCCTCTTATATTGAGGACAGCCTTCACCCTTCTCCTGAAGCATGGAACCTGGTTTCCTTCGGTTCTGGAGAGGATGATGCCATGTCAACTAAAGCCTCTGCCCTAGGTGCTTGGTCGGAAACCCAGAAGGTTG

LG16	ref-24301_16	73.421			ATATACCATACATTTAGTATGTATTTTACTATATATTTACCTTATATTAGTACTACCTTCATGATTATACTAAGAATTTTGTTTTAGTTTATACAAAATGCTGATGGACAACCATTTTATTTATTGTTATTTTAATCCTCAGCATCATTCAAATGGATGTGCATGATTTTACAGCACATGTATATGTGAAATACCATATATATGAGTGACCGATC[C/T]ACATGCTTTTTAACCCTTCATTTGCCTATTTTTTATAATGTATCATTTTCAACTGGTTTTTCATTTGCTGATGCTTCATAGAGCAACTCTGAAATCTCTTTTTGCTGTTTGTTTAGTGTCCTATTATGATACTTTGTTTTGATCAGCATTTCCTTGCTAGCGCTGTCAGTGTTCAGGGTAATGCCTTGCATTATGGAAATAAGGTACATGCACAAA
LG16	ref-35952_10	73.467			TATGTTCAA[C/T]CGATGGACCTGCTTGCAGGGTC
LG16	ref-70371	74.696			ATGACATGACACAAGAAACCTGTAACAATGTCAAAGTAAATCACAATTTAAATGTTCAATGTCAAAAGATCCTGAAGCAAATGTTGAGAACTACTACTAGGTCTACTACTATTAAAATTGGTTATAAATAGCACTGTAAACAAACAACAACAACAAAAATATTTGTGTTACTTGCCACTTAAGATTGTAAACCACTGAGAAAGTGTAGGTCGATGCACCTGCCATGGGCGTGTTGCTTGCAGTTACTGTGGACCATAAAAAGAGTTCCTCTTTACCGCAACTGTTCACCATAAAAAGCCAAGGTGCAGAGCAAAGACTCACAATATACATTTGACCAGAAAAACATAGAGTCATATCTGAAGAAGAAAGGTGGAAGTGATCAGTCATGGCCCAGACTCCAGTCTATTCTCAGGGCGGTTATGCTGGAAATC
LG16	ref-35952_16	74.788			TATGTTCAACCGATG[A/G]ACCTGCTTGCAGGGTC
LG16	ref-51443_30	75.447			GCACCAGATGCGACGCAAGTGCTTTTTGC[A/T]AG
LG16	ref-36499	77.857			GCCTGTCAATCGAACTCCCTGCGAAGGGTCA
LG16	ref-38194	82.13			GTCGGTGCCGTCGGCCGAGGGTTCACAGGGCGGCAGCTGGAGCAGCAGTTTGGAGACAGTGAGACGCAGCAGCTCCACTGAACCGGACGGGCTGGTGATGTCATCACAGAGCGCCACGCTCGCCTCGTTCAGCAGAATCCGGAAGGACAGCATCTGATTGGACAGCCTGCGAGAAACATGCATGGTCATCGTGACATCAAAATAGGTTATTGTTTTAAATTCATGTGTCTCGTAATCTTGAATCAGAATAACTCTTGCAGCATCTCTTCTCTTCTCTGATGATGAGGGCGGGGCAACCTGAGATCCACCATATCCACCAATAGCAAACCACAACCATCCACTCAATTCCCCACAGACAACATCAAGCCC
LG16	ref-1900_2	86.016			TGCAGCATTCCAAATTTGTCAATATTGGTAGCAAATTTTTGATGAGTCTGAACCAATGAAATGAGATTTTCAAGCTCATGCTGATGTAAACAAATCGATCTTACTCGCGCTGACTGACATATACACAGAATTACAGTAGGCCCGCTTCAAAATATTGAAGTAAAACAAAAAAGTGGAATATATATATTTTCCTATAGATAT[G/T]TGGTTTTGCGAAGTGTGTGCCAAATTAGGTGTTATTAACAGGGATTTTAAGGTGTATTTGCTTGGTGATTTTGTTTCATCATAAATTTTAACTTGATTTTTAACTCAACATCATAACATCATAAATTTTAACTCAAAATTGACTTTGCTCACTCTATCCACTTCAAACAGGTGTAGCTCAGTAATTTTTTTGTCGGATTCCAACAAATCATACATCATTTTTGAAGGTTT
LG16	ref-30277	87.349			AAACCTGTGCCGAAATTTATGCATTTGGCAT
LG16	ref-8590	87.721			ATTTTTTAGCTTATCAATTTTCTTAACAGTTTAAATCCATTGAAATAATAATAGGATAATATTAGTAACAATACACTTATAACTTGAGTCATTCTTGAACTATATTAACTTATATTACAGAAACCCCATACGAATTCATAATATTAAACAAAACTTACGTTATATTTTAAAAGCCAAAGCAAATAACAAGGAATCAGATGAAATATGAGACGATATTGCCGCCTGCTGACCATTAGTAGAACTGCAAAACAAACAGATAGTTCATCCAAAAATAGAAATTCTGTAAATATGTACTCAGCTTCATTACAAACCTGTATTAGTACAATCAATACAAAATTATAACAGACAATGTAACAGACATTGCAAATCGTTTTGCACACGTACTGCACATATTGTTATGTAAATATCGTTTGTTCCTTTGCAATGTTACT
LG16	ref-28649_25	89.742			AGTTATTGACAGCCTCCTGCTGGTCATATATGTAACTGCATGTTTGCGTATTCTGATCTCTCAGGATCTGGAGTGTAATGTGTCACTGGTTGATGACAACAGGCAGGAGTGGGTCTTTACCCTCTATGATTTTGACAACAGCGGAAAAGTAACGAAGGAGGTAAAAAAATTGCATTTTATGTATTTTTTATTTTAGCAGTCCTGCAGGGGCGATGTGTCTGCACT[C/T]TTAAGTGAAGCAGCAGTGTTTCATGCACAAGTGTGCTGCATGCTTGTTCATGCAGTATGAATGTTGTAACCTGTTAATATACTGTATGCAAAAATGATTATGCGCTGCAGATGTGTGAAACAGGCCTGCCGTAAACAAACATGCTATGCAACTGACATTCATTCCCTTCAATGAAATGCATGAGAATGTTTTTATGATGTTGGTGT
LG16	ref-38534_29	94.274			GACAGCCATCCGAGTCTGATGCATGTGT[A/T]CAT
LG16	ref-21067	99.816			ATATCTCCTGCGAAGTTGTTGCAAATGTCGC
LG17	ref-27750	0			AACTACGCACCGAATGCCGTGCGCACGCTCA
LG17	ref-69463	3.131			ATTATATTTTAGTTTCAGTATTCGACTACACTTTATCCATATTTTAAAGTCAACAGATGTTATTATGAAGTTACATATGAAGATGTACTTAAGTCAAAAGTCAAAAAAAGCACTCTTAAGTTAAGAATTAGATCAATAATTGGATTTAATATAAATTTAAACTAAAATACATTTTCATTTAATTGCAAATATTATACAACAGCAAGTGTTCGAAAACATTGCACTTAAGTATATTCTTAAGTATTATATCCGTAAAAAGTATTGTTTTTATGACACAAAGGATTTTCTTTTGGACCCCATTGATTAATACTGCATGGACAAAAAAATAGAAATATTCAAAATATCTTCTTTTGTGTTTCACAGAAGAAAAAAAGTAATAATATTGTGTGCATTTAATGCTTAAAAATCATTTGAATATAGCTGAAATATTC
LG17	ref-51632	7.866	Chr14	28632407	GTTGGTGTGAATGTGTGATTTCTCCAAAAGTCACAGCACATGTGAGTTGAGTACGAAGTTCAGGAGATGTGCCAGGATCACGAGCTCCCATATCAGAGTTTGATAATATCGCTTTGCAAGAAATAAAAACTTGTAAAAAGCTATATAATTATCAAATCTATTTGATGGGACAGCATCTGGCAGACATGTCGCTGAAGAGAACACACTTTAATGGGCAGATGCTATTTACAGCCACCCTGTTTGGCCAAATTAAAAGAAAGAGATACAACCACAGTCCCACATAGGTCACTGTGTTTGTCACGCAACAACATGTCCAAAAGTTAGCCCACTAAAAACTTTCTTTCTGTAGCCGCCCATCTTTCTTTTCAG

LG17	ref-13892_10	9.525	Chr14	30041494	AGCATGTAATTAATTGACACTTTGCTTCTGCTGATGTGTTTGCCTCACATGCAGTCTGCTGGGTTCCATTTTCCCAATGCCGCGTGTAATCTATGCTATGGCACAGGATGGGGTGCTTTTCAAAGTGTTAGCCCAAATCAATCCTAAGACGAAGACCCCTCTTATTGCTACAATGTCTTCAGGTGTAGTTGCAGGTGAGAGATGGAAGCA[A/T]GATTGCTGTGCAAACCACCAATGTGATCCACCAGGCAAATGTGAAAAAGAGTGGAAATGCGAAATATAAGAAATTATTCTATGGGAACATACTTCAAATTCAGGGTTACAGGGTTCCAACATTCACAAAAAAAAAAAACCTGGAAATCTCAGGAATAAAGCCAGGATAAATCAGGGAAATTAATAAAACCCTAAAAAGTCATGGAAAACAATGCAGTTCATT
LG17	ref-23912_18	12.066	Chr14	31644389	TGAATCCTGTCACTGGATTAGATCTGTGACATTAGCCCTTCTCTTCACATCAGGTCACAGGCTCCATGTCACATGACACCAGGGCCTTGACCCCTCTGTGGTGACATGTTACTCTAGGGTCAGTTGGGTCACTCAATTAGCAAAGGAGACAAAGACCTGGGCGTAGAGGAGGTGAGAGATCAGCCATCCCATCCAAAGAAATTCAACCACCGAGCTC[C/T]CTGCCGCACGCCTGCTTCTGCTCGCCTGGCCAAGCCCTCATTTAGCAGTTCACTGACTGGAGACGTGCCCGCTGCTCTTCACAGAATTTGCGGTGACAGCCAAGAGTCACTAAAAAGCTGAAGAGTCACTCCTGTGCCACATGGCGGGACCGTCGGCGAGATTATGATCTTACATCCATGAGTCAAGAATCTGGTCTTGAATTGACTGGCACGC
LG17	ref-44967	15.485			TGCCAATTTTCGACCCCCCTGCCAGATTATT
LG17	ref-61443	16.356	Chr14	36262368	GAATGTAGTATTGTTGAGTAGGTACCAACAGTAGGTACCAGCCTGCTTATTGTGAGACAGAGAAAAAATATGACTGGTAAAACAAAAACAAACAAAAAAATATGACTGGCAAAATTATTATTTGTACAGTCAAAAAAGAGCATATATGGCCAATAAATGTGAATATGACTCAAATCGCAGCAGTGTGATCGAACAACTTAACAAAGACTTGAAATTAAGTAGCAATAGTGCTCACAGACTTTTACGGCAGAGTAAACGTAAGTCTCTGCAGGCCATTGCCGGGGAGCAGATGTTTTATAATGGGCTCGTAAACTGAACGCTTCACGTTTGATTACAGCCAGAGAGAGACTCTCGTTTAACTAATGACCG
LG17	ref-19234	17.978			TAATCAGCACAACAGTTTTCAACATTGATTATAATAAGAACTTTCTAGAATTATTTCTGAAGAATCATGTGACGTGCTGAAAATTCAGTTTTGCCATCACAGGAAAAAAATTACATTTTAAAATATATTTAAATATATTTTACTGTATTTTGGCTCAAATAAATGCAGCCATGGTGAGTGCAAGAGATTCTTTCAAAAACATTAAAAAATCTTACCGACCCCAAACTTCTGAATGGTAGTGTTTCTCCATGTGGTTAGGATTAAAGTTGTTGATATACTTGATTGTTCATTATCAGTGTTTATGTCCTGTTTTTCATTCATGTTTATTTCTCTATAATCATTTTCTGCTGTATAATAGGAAACTCTGTC
LG17	ref-45377	18.733	Chr14	36728534	TTTCAGCCCTGCATGCATTGAAGAAAGAAGAATTGACAGCAGACAATTCATCTGCTCAGTGTGAGTGTGTATATGTGTGTGTGTGTGTGTTGGGGGGGGAGGTGTATGGGTGTGACTGATGGTAGGTTTATAATTTAATGCACTGCTTTCACTACTTAATATCACCCTCTTTCCTGTCGCAGAGTTTAAATTTCCACTGTACCTGTCACTCGAGTGGGGAGCCAATTAACCGCCTTGTCTGCCTTTCCCATCACAATATGCATGCAAGGATTATTAGGTTTCAGGAAAATGTGTGTACTTGGCAGTGTGTCTTTGTGTTTGAGTGTGTGATAAGAGAAATGGAGAGAGAGAGACACTTTAGTTACTATCACAGAGATTCATTGCAGGCTTTTTTTTTTCTTTTTTTTTTTTTTTTGAGATTTGCAATAC
LG17	ref-26206_27	18.996			TCACCCTAGTGACCCTACATCAATGCATGTACAGTAGATAGGCTACATCCAATCTGTCACATTAATAGCACAATTGCATAATGCAGCAACAGCGTCATGTCTGCCCAATTTGCACAAATCTCTTCAAATTATATCCAACACTGTAAATAATTTCTCTGTAAAATAACAGTAAAACAGTAGCAACAGAGTCGCCAGC[A/G]ATCCGAACAGGAATAGGAAGCTCGTTCCCAAACTCCTGTGAAACACCCGTTCAGTGCCATTGTGCTATTACTTAAAAACTATGCATTGCATAATACAGTACAAAACCATAATACATAAATTTAGATTACTATTGAAAATGGGCTGTAAATTAACAGCAGTTGCTTACCGTGAA
LG17	ref-20940	19.428			AAATAATGCACGATGATTGTGCGCTGGATAT
LG17	ref-21388	20.305	Chr14	36231588	ACAATCAGGCATGACTTCATGGTTATGTAATATGTAAAACATTTGTGTAAAATTTCCAGCAAAAACAACTATAATAACATATCAGTACAGTCATACTGTATAAAGATTTATGTCATAGGCCCAATAAATAAAGACACATACTGATAATGTTCATATGATCTGAATCTGTTTATGCAGAATAAAAATTCATAATTTCATTGCATTTCATCACGATTGTTAAGCACGCATATAGACATAACCTCAATGTGTCAGGCTGCAAACATTATGAATTTCTAACAGCAGGCATTCTGCAATACACCTGTCTATCCTTAGATATTCGTGCCTAATGTGAAAGTGTCACATTCAATTTGGCTCTCAGCTAATGCAAACACAGTGACGCCTAATTGAAATCCACTGGATTTATAACCACTGATTTACTGTGCAGTGTTGGT
LG17	ref-43480	21.47	Chr14	41300009	TGCATCTAATGTATCTTGTGTTAGAGAAATTATGTACCTGGCACAGAGTTGAAAATGCCTAAGGATACAAACAGCTGTAACCAGAGCTGTTCTTATCTGCCTGTCTGTCTGTGTGCACAGGAGACAGTGTTATTGTCTGCCTGTCTGTCTGAAAGAGCTGCAAACTGTTTCAGCAAAATGCACCACTCTTGTTGAAGAGAGAAATGTCAGTATGTAGTTCACAGAATATTTCTTTTGAGAGGAAGATTGTTACATGTGTGTGTTAGCATGCATGTGTACATGTGTTTGTAATTTGGCTGGGATTCAGAGATAATTATGATGTTGACGGCAGTGATTTCACTATTAACACATTTTCTTGCTGAAGTTTGT
LG17	ref-33851	22.417			AAATAAAGGACAAAAGTCAGATGTATATATTTATTTATTTTTAAGTTGGGGGAAAGAGCAAGGTATCTTTTCTTGCCCTTCTTTCCTCACTGGGAGATCAGATTGTGTGTGTATATATATGCGTGTGTGTGTGTGTGTTTATCCGGGTTTTTTTTTTGGACGCAAGGTGATGACAACCTTAGCAGAGCATTTGTGAAAGAAGTGTTTATGAGGTAGACAAGTCTCTTTCCATCTCTCTCACAAACATTCCATGCAGCCTTTCATTTTCTAACTTTTATAATAGTGGAAATGCATCGTCTGCTGGTATAAGAAACCATTTCCTAGCTATTGCATTCCACGGTGACAAACATGAACTTTAAATCTTTATGAGT
LG17	ref-47376	22.867			GATTGCTACACGAAAGCTGTGCATGCTCGTG
LG17	ref-60429	23.452			TCATGTGTCGCGAGTCATGTGCTTAGACTCC

LG17	ref-40191	24.666			TAATTTGAGGCGCCATTTAAAATAATAAAAATATAATACGAACTATTGCTCAATATTTAGTTATTGCTTAAGATGCATGATATTTTCATTTGCTACAAGTAGGATAAACAAGAAGCACGTTCAACATGGATGCGCAGAGGGTTGATGTGGTAACAAACACCACAGCAACCGCTTGCTGAGCACGCATCTCGTATACATAAAGCATTTTTAGGGGGCCTTTGGCTTTTGAGGGCCCAAGGCAGTCACCTAATGGTAAGATCACCATGAATGTTGTAACACTCAGCCCTACTGGCAGATGAAACACAATTAAAAAGAGAATTGATAAAAATACTTGTCCATTGGGTTTAAATATGAAGACAAGAAAACTAT
LG17	ref-33584_14	24.677			GACCTCAACCCGA[C/T]AGAACTGCCTGACCTCAC
LG17	ref-33584_25	24.677			GACCTCAACCCGATAGAACTGCCT[G/T]ACCTCAC
LG17	ref-32550	28.48			AATACTTGGACGAATAGTCTGCAGATTTGGA
LG17	ref-31809	29.831			AAATTTCAAATTCCAAGGTGACTCACAGTCATTCAAATCTTTTCGCTGGCTTTGGGCCTTTGACTCATGATTAATGGATGCGATCCCTGTCTAGAGCCCACCTTTTCTACATCTGAAGAGCATTACGGCGGCACTTGAGCTCATGGAATGAAATTTAATGAGGGTGGAGCCTCAGGGGTGCAGAGCTATCGGTTATCTCCCCATGACCCAAAAGAGAGTAGCCATCCTCTCTTTTATAACACACAGGAAACTCAAACGGACTGCCCTGAACGAACTGAGGTGTGCTTGTTCTAGTGGAGCAGACAACGTCCATGATTGATTCAGAAGATGTGACCTGCGCATTCAGAGCGAGACTCGGCTTTAAATAAC
LG17	ref-56802_1	32.707			TCTGAGCGGGCTGGGCGCAGGCGGATGGAGGCTGCTGCTTTGCAGACTTAGCAGGGGTGGAGGACAATGCAGGCGGGTGCCCTTGGCAACGAGCAGACTGAGGTTGCGCAGCCGGCGGTGGGGTGGAGGCAGCAGCGGACTGCCAGGGCAGGATATGTTTGATGGCCTCAGTCTGCTTCTGTGTGGCCGAGAACTGCTGG[C/G]CAAAGCTCTCGACCATGTTGCTGAATACGGACACGGGGGAGTCCAGGAACCGATACTTATCTGTCTCCCTCATGTCCACCAAGGTCAGCCATAGGTGGCGTTGCTGGACCATAAGGATAGCCATCTCTTGACCAACCGAGCGAGCCGTCACCAGTGGGGCATAGGGGAGGGGACCCCTGCTCCCTGAGAACCAAGGTATGAGAGTCTAGACCTCAAAACTGAGATCACGTT
LG17	ref-26080	34.554			GAGACAAATCCGAAGCGCGTGCACAAAGACT
LG17	ref-26967	36.528			ATTTCAGTCATATGTGTCCAAACGTCACAGTTTTCTGGCTGTTTCATCCTGCTGTGAAGTTTGTGCTTTTACAGGTGAAGCAAAATTCCCCTCGTTCAAAGCCTAAATGATATTTTAATCAGCCTGTGAAAGCTGACGATGGTCCTCTACGGCTACGACGAGTGAATCAGAGATTCTATCTTTCTATTTCAATAGTTTTCTAACAAGCTGCGAGACATTTGCCGGAATCGGCAAGAAACCTCACGTCACCGTTTGCTCTTTCTCTTTTTTTCCCCTCCCGTCTTGTTTTGCATCAGAATTCTGAGGTACAGGAACCGAAAACAGGGGGTTGAGCGTATCTAACATGTAACATTTACCCAGATGCACACTGGAACTTGCATGTGTAGACAGATAGTTTGCAGTGCGCTTGCAAACAGGAACTAGGAAAAGCC
LG17	ref-72558	38.625			CTTCTTGTGCTATCAGATTCAGAATGATCGTAAATACCATGTTTTCTACTGTAGGTAAAAACTGGACATAAATGCTATAAACTTTAAACATGGTGGAAGGGAGAACGATTGCTGCTCTGACTGCTATTCTTTATTAGTTTTTTCCACAACAGCTAAATGCCTTGATTTGTCATTACAGTAGTGCATATTTACATTTATACAACTGGTTCTCGAATCTGATGCGACATTCCCCTAGTATCAGCACTGGAACCGTTTCACCGTTTCACTCCGCTTGTGGCGACTGTCATGGCGGTCGAGTAAATCCACCATATTTTTTACAAATACTACTGTTGTTGTTGTCACGAAATGTAGTTTTAAGGTGAGAATGTAGTTATTTAGAGCTTAAATATGTGACTAATTTAAACATAGTGTCTATTTTACAATTTGTATAG
LG17	ref-56424	38.803			CAACTCCAGCTCAGCTGAATCTGACTCTGAAGAGGGAATTTTACAGGAATACGCCAACATGAGCCCTGGCAGTGCCCTTATTGAAAATGCCACTTTAGGGCGAGTGCATTTCTCTAAGGGTTCCTCATCCAGACAGTCTAATAATAATCAGCACTGTTAGCTACATGACACAATAAACACAACAGCAGCCGCGACAGAAGAACCCGGAGACAATTCCAATGCAAGAGATCAGACAAAAAAACAATCTTCCTGAACCTCTCAGGGAAACAAAAATATTTAAAGAGCGGCCCTAAAAACATATTTGGGCAGCAAAGCTCCACTTATAAATGTCTGAATGTCACTGTATTATTAGAAAACAAAATATCAATCCAGATCTTTAACCACGAGGCCACAGAATAACATCATCATTTCAGGCCAGTAAAAAAAATGTT
LG17	ref-24283	40.241			CAGAAGAGTCAAAACAGAAGAGACGACTGCTGTAAATGTGGCTAATGTTGGCAAATAATCACATACCTGATTAAACTCCCCATCAATGTTTGTCTCTTGTTCGCGAACAGTGACTATGAGCACATGACTAATTGCTGCTGTCTCGCGATAGGCTTGTCCGAGATGCATCAGTGCTGCGCAGACTGATCGGCGTATGACATCAAAGTCCCGCGAGAGCGATTCAAAAGCATAACGAGACGTATGCTCTCCAAACGCTCTCGCGGTACTTTGATGTCACGCCGATCAGTCTGCGCAGCGCCGATGCATCTCGAACAAGCCTATCGGTGCAGTCGGGGAGGAGCTGAAAACGGAGACGTGAAGTCGGACACTTTCTGCTTCCCGTAGTGACGCTTGCGCTGCGTTTTGCATATTATATCACAGTTTAAGTGAAA
LG17	ref-50363_15	40.999	Chr14	6048738	CAATATACTTATGCTAACAGAATCTCACTGGTTAAGCCGTTTCACCATCTTTCTGTTGAAGCTGATTTACCAAGAACTCTTTGATAGTCAAGTAACCCATGCTGTAAACTAAAACCTTTTCAGGTTGTTATACCTGCTAATTGAGCATTTGGGTACCGTGCAGGTCCTGATCGTGACGTGCAC[C/T]GCATTCGCTGGGGTGTTAGCACTGAACTATCAGAGAGATTACACCGAGTGGGTCCTGCCGCTCATCATCGTGTGTCTGTTCGCGTTCCTGGTGGCACACTGCTTCCTGTCCATCTTCGAGATCGTAGTGGACGTGCTCTTCCTCTGCTTTGCCATCGACACCAAATACAACAATGGCACACCTGGG
LG17	ref-48274	41.81			CATACCTGTTTTTTTCCATGTCATAGTCGCAATGTTGCCCATCACAGTTCGGTACAGGCATCAGAACCGCGTCTCGTAGCAGATCTTCATTTGTGTGTGACGGAGGGATTTGACTCATTTACACATTTTATAAGCTCTTCACATGTTCTCAGCTGGTTAATACCATCATATGTAGCTATGCACATACAACGAGCGCATTTAGCTCAGCACACAGCATTGTTTTGGATGTTGGTTAAGTTCCGTCGCAAAATACACTACCGGTCAAAAGTTTGGAATCTGTAAGATTTTTAATGTTTTTAAAAGAAGTTTCNNNNNNNNNNNNNNNNNNNNNNNNNNNNNNNNNNNNNNNNNNNNNNNNNNNNNNNNNNNNN

LG17	ref-13765_9	42.153	Chr14	7990324	GACACGTTTCGAAATGCATTTGAGATTGCATCGTTTGCGTTTGTTTATGTGTTTGCATAGAGTATGTTAAAGGCCTTAGTGATTCAGAAGATGTAAAACACAGTTTGAGTGAGTGTCTTTGCCAATCTTAAACTCAGGTTGTGTTAATATGCTGTGATGAGGTGCTTATGTAGTGGAAGTGATGCAGTGTAAGCTCTTCTCATTTGGCT[G/T]CGATAGACCTGCTGTGTCAACTTTTTCTTTCTTTTTTTGTTTTAATTAGCAGCTGGTTTTCCTCTGCGGGCTCCATTGGGACTCCATATGTTATGGAGTATAGATCCCATGTGTAAAGCACACGCTGCACCTCATCTTACACTATTTTACAAAAATAGGACCTATAGTTTCCACCAAAACACCACAGTTACTACATGTACCTGTACTCTTACTGTAAAATGA
LG17	ref-6409	43.05			TACACTCATTCGAAGCGCGTGCACAGCGCAC
LG17	ref-23057_14	43.38	Chr14	11554030	TGTGAAGCAGATTTGCACAGTTATTGACAATAGACTCAATTTTGCCAACATCCTCTTCTGCTGCCTCTAGGTCTCATCGTGTCTTATTAAATTCTTCAGCTAAATCATTAAGACTACTAAAAAAAGTCCACAGATCTGAGCTGTTCCACTCCTTGTCCTCCTCTCTGCTCTCATCCTCTGAGTGCATTAAAAATGAGTCAAGTTTGTGATCGA[A/G]CGCAGTGCATTAGAGCTCATGAATTTAGCATGCTTTACTATGCAGAAGAAAGACTGTTTAATCTGAACTTTACTGAGTGATATTCATGAACTCGTGCTATTTATAGCTCAGGTCAAACCACCACATGGAACATTGATGGGTTGGTCTATGATGTCATGGAGCATTACTTCACTGTGAAAAATGAAGAACTGACAGAGTAAAGGCATACACTTTTGGTA
LG17	ref-46971	43.778			AACAACAAAACGAAACTGCTGCGGCCAACTG
LG17	ref-54679	44.719	Chr20	1542278	AGGCTACAACCTATAACGTCAGTTGCTAGAGCATCTCTTGAGATCAGGGGAGTGAGGTTTACACGCACAGCTCTTACTAGCCTACGTCTGTTACACTCACCCCCCTAAACCTCACTCCCATCCGGGTCACGGCACCAATGTAGCCCTTCCAGTTCGCACCTGCCTGCTCCAAGCGGGACGCATGGGAGTCGGATGAGTTACTGTATGTTTTTACATATTGTGTGGAAAAGAGCAGCATGAACATCCTGGTGAATTTCTGTTTTTTTCTTCCACAGAATGAAAAAAAAAAAAAAACTAACAGATTTGGAATGACATGAGGGTGAGAATTTCTTCATTTTTGGCTGAGCTATTTCTTCACATTAAATAAAA
LG17	ref-57082	45.2			CAGAGTGCACCGAAAGGGCTGCCGATTTCAC
LG17	ref-50339_19	45.519			TGGTTTGTGCCGACCCCA[A/G]TGCATTTTTTTGT
LG17	ref-42421	45.885	Chr14	42792828	TGCTGTTGGTCTGTGTGGGCTCCTGTGATCATGATGGCTTTTCAAAGCAGACCTCAAAAACAAGGAGCATGATGGGAAGCGACTGAGCGTTAAGAGGGAGCGCCAGGGGCGACACGTTAGCAAAGAGGGCCACAAAACTCTGCATTCCAGTTGCCAAGGCAACCAGCCGAACGTTCTGCGTGTATACAAAAAGGCAAGCAGGTCTGACTGCGAGGCAAATGCACAACTCAAATACTCAGTTATGCAGAGCAGCGTATGGATGATTATTTAGAGACTAAAAAAAGAGTCTGAAATTGCTCTGCAGCTCTAATTGTGAATTTCAGGGAACATAAACTTCAGAGTCATTTTCGGAAAGCTACTGTAATTGTGACAAAGCGGAAAACAGAACTGAGGGAGCTTGCTTAAAATAGATGCTGCATTTAGTAATTAAT
LG17	ref-67883_4	46.352			ATACATTGATTTATTTGTGGCGGCCAGCCACAATATCAACATTGACCGCCACAAATAGATTTCAGTGCTCCCCATTCATTAACTAGACCCAGCCCGAAAATATATTTAAACTTCTCATAATAACATAAAAGGTCTTTAATGCCGTGTTTTGCGCCACCGCTGTCAAACAAGC[C/T]CGCAGTGCAGTACAGTCGTACCACATACCAGCTGCTTATCAGGTTAACTGACTCGTAACGTGTGCAACGGCTCACGGCCTCCTGAGTGTCCGAACTCGCACTCAGTACCTACAGGGACTGAGGTCCATCCACCAGACTTTAACAAAATCATGTGGGAAACACTGAATTATCTCTTCATTGACTTACCCTCATGTCGT
LG17	ref-15641_4	46.504			TGTATACAGTAGCCAGCACAAATGTCAACTTACATAATTTTACGTAAAGCACTATCACTTCTTTAATGTGCCTTACCAGACATACATGCACAAGTTGTTGGAAAATAGGGTGATCAGGGACAGATCTTCTGATGACGCTGGAGACGAGCAAGAGGCTCGTGATACTGATCAGGAGGCAAGTGATCAGGCTGAGGATGAGTGCA[C/T]TGACAACGACATAGTTGCTGAATCTGAGTCAGAACCAGAAATTGAACCAGTCACTGCGGTTATCTCTGAAAGGCAGAAGCCAAAGCCCAAAGCGGTTGCGCCTATTAGTCAGAGATCCCGTAGTAAAAGACCTGTGAAGCCAGTTATAAGACTGACTTATGATGAACCAGGAAGAAGTAGTGACCACCTAATTGAAATAGTCTATAGAGGGGTCATTATTAAATTAGG
LG17	ref-47840	46.507			AATAGTAACGCGACAACAATGCACTGAAACA
LG17	ref-56212_14	46.726			TTCATCATAGACGTCCCACTCTTGGTCAGAGGAACTTTGAAGGGCCGGCCATTCGATGTTGAGGTGAGCGGCCGCTCGTTCGCAAAACTCAAAGAAGACCGAAAGCAGCGGGAGCGTTCATCTGAGGCGTCACAGATGACGGGATGACATCCCCGTCTTCCGAGCCGTCAAGAAACCCAGCA[A/G]CATCCTCGTCCCCAGCACCCGGGTGGCTCATCCATAAAAGGGGTTTCAACACCGCAAAAGAGGCCACACCGCGGGAGTCCCCTGTGTGCGCACCTGCTTCGCCTTTTTTTCCCATCAGAGTTGGACAGACATAAATCAGAGCGCCATGCTACAGAGCGCAGAGAAATCAACAGCCTCTTCGGCGTGT
LG17	ref-23391	46.88			CACACACAAACGATCTGCTTGCCTGTGATGG
LG17	ref-51409	46.901			ATAATGGGAGGTGTTGTCTTCACAGCCGGTGGAAAAGAATCTGACAGGACTTGGGCAGAAATCATATTCATGGATGAGCTAATGTTCTAAAGATTTATTAACATTATTGTAGTATGAAGCAGGGTGTGGTTGAAAACCACTGGAGCAGAATGAGGCCACTGGAGCAATTGCTAATGAGAGACAAGTGTGACACACTTATTATGCTGCAGACGAGTGCTTCGCTTCTTCCGGTTATGAGTATGTGGGGTAACGCAGCACTGTTTTATCATATTAAATACATTTGCGTGTTGAAAGTTGTTATAATGCTACACTGTGCATTCGCTTGGCGACTACTATGAGACACTTCAGTAAGCTAGATCAATATTAGTCATGGTAAAACATGGCACTCTTGGTAAATCAAGAAAACTAGATTTAAACTATAAGACTTACTG
LG17	ref-55284	46.958			AGCCGTGGCGCGACATTGTTGCAAATGATAT

LG17	ref-54642_24	47.074			CGTTGGTGCTGGGAACGGATAGAAATACGGTTGTCAACCTAGATAGCAAGATCGTCCAAACCGTTGAGAGAAGGGGGTAATTCCAAGGAATTAATCTCGTCCTTGATGTGTTCCACTAATCCAAGCAGAAAGTGGTCCCACTGTGCTTTAGAATTCCACCCACAAGATGCTGCGAGTGTACGAAACTCGATGGAATATTCCGACACAGATCGAGCTCCTTGCCG[A/G]AACAGCGACAATGCTCGTGCTGCCTCGGAGCCCAATGCTGAATGGTCAAAAACCTTACGCAGCTCCTGAGAAAAATCCTCGTAGGATCTGCAACAGTCATGTTCATTATCCCACATGGCCGTTCCCCACTCCTTCGCAAGACCGGCCAGGTGCATGATGACGAAAGCCACTCTGGATTTCTCCATCAAAAAACAGAAGGGCTGTAGT
LG17	ref-54794_6	47.105	Chr13	18458741	TTACTACATCTTTTTTATAGTCTATGGTATAAATACAAAGTTGTGTCATGAAACTCTAGATGGTGCAGGCTAATAGGTCCTCTAACTCATCATCATTATCTATAGGGCACAGCTGATTGGTTTCCTGCTGTATCAGTAGCCAATGAGCTTGCGTGCTCAAACTTTAAAATACTT[C/T]GGTAGCATTTGCTTCGCAGTGCAACAAGCTTTCAGAAACCCTCCACCTTCCCCAGCTCCACCTGTATAGATCTGCTATGGGTAATTTATGTACATTGTACAGCAGCAGCATGACTTACTTGCACATAGCACTGTGTTGTGTATTTATTGGCAGTAGCAAGTCCCGTACTTGCTCTGCAGCAGCAGATATAACCTA
LG17	ref-11210	47.417			TGTAGACTTAAAAACAGAGGGAGCAAATTAACGTTTGGAGGGACTGTCGTGATATAATAAGAAATTGTCTAAAAGTATTAGCCTGTTTTCATCTGGTCTCTGAGTTGCCTTTTCATTTGTGTGTGTGTGTTCAGTGACTTCCAGCAGGGTTTAAATGAGCCTTCGTAATTGGGTACACGGCATACAGTTCAAGCGCAAAGTTGAGACGCACAATATCCTCTCACTTTCTTACGTTCATTTTGGTGTACTTAAATCCCAAGTTAATTCACTTCTGTTTTCATTTCCTCAGTTGGGGACAGACCTCTGCCATGCAGGGCTGCAACCAAATTAAAAAATGACCTTTGGAGTTAATAGCTTCCTTTCACAGTG
LG17	ref-41209	47.684			TGGCCAAGGGCGAAGGTTGTGCCTGTTAATT
LG17	ref-27871	48.085			TCCAAAGAATCTCACAAAGACATGCCACTGTCATGCCTTTCCCCACTGACCTTATGAAAGAGCCATCATCACATATTAGCACAACAGTGAGCAAGTGTGTTTTGATGGCAGACTTCAAAAACCATAGCAGCTGTTATCTCCTGTGGCAGACGTTAGGATGTGAGCATCCCCTCAAGACCTTTCTTCCATTCTACCAACCTGGAGCCAATACGAAACCACTACACACCACAGGGTCATTACCTCACACCAACCACCCTCTATCCAACTGCATGTGTCATCAGCCTCTCCACCACTGTATACATGACTGGGATTGAAATAAGTGTTTATAAGCCTATGTACACTAACAAAAGCTGACATGTCTCAATGTTTGTTATTTGTTTGGGACTGTCTGTTTGGCAGTCAAGCTGTACTAGTCACAGTATTTCAGAGAG
LG17	ref-18388	48.375			CCGGCCAGGTGCATGATGACGAAAGCCACTCTGGATTTCTCCATCAAAAAACAGAAGGGCTGTAGTGAGAAAGTCAGTGAGCACTGTGACAGAAATGAGCGACATGAATTTGGCTCACCAGAGTAAGGAGCAGGTGGATTGAGGCGAGGTTCGGATTGACAGGGAACGACCACCTCAGCGTCGGGTAGAAGTGGTGCAGTAGGAGCCATGCGAGGATTATGCTGAAGCTATTTGAGCCGCTGAGTAACCTCAGCTAGCTGGCGTGACATCTCAGAGATGGCCTGATGAGAAGCCGCAAGTTCCTCCTGCTGCCGTCCCAAAAGAATGCCTTGTTGTGCAATGGCTCGACGCAACTCAAGTTCATCCACTGAGTCCATAGTGGTCAGCTGATTCTGTCAGGAATGTCAGATAAATGGAGGACTCAAATGCAG
LG17	ref-8912	48.748			GTTGCATAAACTTAGGCTTTGGATTGTTTCTTAAGAAGTAGTTTGACCAACTAACAGTTAGACAAGAGGTAATCCTTTTTTCTTTTCCGATTCAAATTAGACCAGTCTACCTTTTATGTAGCTGACTTCGTTATAACCAGTCTTGATAAATAAACAAAAAATAGATGACTATCTTTTAAGAATAGTCTATTAGAAGTTTATGAAACCAGCCAACAATGTTGCCTCTTCTGTGGCCTTCAGCACTGGGCTAAAGTGTAGTTTCCACCCTGAACTACTCAGAGAGATCTGAGAAAGTTATTTCATCATGTGATTCTGCTGTGACCTCCAACCACCTGACACTAACATCGGTCTCAGCCCACCAGACCTGTTGAAAATGCACTTTAAAGCCGACGTGTAATTTTTGATGTTAAAATACCTTTTTCCCAGCTTAA
LG17	ref-171	49.291			AGGAAAAAGACGACCTCTGTGCTTCAGGAGT
LG17	ref-48463	49.3	Chr14	34931414	TGTCTAACAGAGTTCATGTTGCCTCATTCTCTGTGGTGCCACCTGTGATATCTAAACCGCTAAATGCAGCCTCCGTCATCCATTAGGTCCCTCCGACCCTCTCATTAGTCCTACTCACATTCACAGCAAATTACCCGCAGCAATTAGAATAAATATCAAACACAACATGCGCCTCGGCCGCAGCACATTCGGGTTCATGCTGGAATACCTACTGTGGGTGTTCTGATTAAGGTGAAACTTACATCAAAGCCCTGCAGTGCTGCCTGGGTAAAACACAGTGTTAAATGAACAGTGTCACCACTGTATTTCTGTCCAGTCAGGATTGCACCTTTTGCATTGTATGACACAGAGACTGTGAGTAAAATTAAA
LG17	ref-26376	49.332			ACAATAGCAGTTACTGAAATGGTGCAGGACTCTTTAAAAGTATGCAGTCTGCCACTTTTGAACCAATCACCTGAGCTAATCATTCTGGAAAATGACACGCTTATAAGCTGATAGCACCATTAAAACATGCAAAAATATAAGTACATTCTTAAAAATAATGGTTCCAAAAGAAAGGCTTTGCAGTGATGCCGTAGGCTGACCCGAAGAACTTTTCAGTGAACAGTTCTTAAAATCGTAGGCTGACCCAAAGGATTTTCAGTGAACAGTTCTTAAAATAATGATGTTTTTTCTTAGTATGAAGAAAATCTTCTTCATGACACTGTACTGTAGATGCCAGTAAATCAACTTCTATAACATAATAAAATATAA
LG17	ref-12045_30	49.358	Chr14	34216088	TTTATAGCACATGTTTGCTAGAGTAATAAACTTTTGGAATCACTGGCCGATGCAGGTGCGAGCTGGTACGGAGGACTGACTGATCCAGAAAGATACCCATGACAGAACCACCAGCAATGTTGAGGGCACATACGTCTCCAGAATGAAGAACAGCACATTTCTACGCAAAGCAAAGTAGAGCACTAGCTTCGGGTAAGA[C/T]CCTGCAACAGATCCAGAATACTGCAGGTGATTCATACATTCAACTTTTACAGAAAAATGCTATAAAAATGGTACATTAAAAGTGGTTAATTTGTTAACTGCAAGTTACTTGAAAACTGTTACGGTGAAAACTGTAAATGGTTTATAATTCTTACCATATACGGTGAAAACT
LG17	ref-47802_14	49.414	Chr19	5255198	TCTGCTTTGATTCTGTACAGACGACAATGTTGTTAAAACGATCCTCGTTCACACTGATCTGCGAAAGCGACTAAGAATGCTGTATTACGCATGCTAGGCCAGTAGTTGGCGATGTCACTTTGTTAAGAAACACTATGCGCCTATAGACTGAACATTTATTTAATGTTTACATGGAGACAGCAA[C/T]GGTGTCGCTTTCAAAAACTTGCACTTTGAAAGCTGTTCAAAAGTTTGTGTTTTCAGGGCCCCAAATCACTGTTGTATATGGCCCAACTTTGTATTACAAGGAATAAAAAAGAAAGTTTGTAACAACATGAGGAAATGATCACGATTTT

					TGATGCTAGCTAAATGCTGCTAGCTTTGATTTTCCAAA
LG17	ref-72377_10	50.454			AACCATCGC[A/G]CGAATAATTTGCACATCAAAAT
LG17	ref-70515	51.642			TCCAAGAATACAATCTGTGATTCCTAAGTACAGTATTGACCGGTGCAGTGATTGACAGCCACACATCAACATTAGATTCATCCACGCTGAGGAGTCATGCCGATGCACAACCCACGTAAAGATAATATTTCCGCAAATAACTGCAATTGTAGGTTTCAAACAGAGATGGCAACAAAGAGGCAATACTTACGGACTGCAGCTTTAAGGTTATGTGCTATTTTTAGATCAAGTTTGTGACTGTGTCTAGTTAGTGACATTGATTTCTCAACTCTTTGTACAGAACTTGTCAGGTTTTTGTCATTTATCATTCATTTTTTCAAAACCCAATTGACTTTCTGTCTTCCACACAACACCAAATGAGAAAAAATG
LG17	ref-12267	52.936	Chr14	38971237	TATTGGCTGGGAAAGCTTGCTACTGCATCCTCATTTGCAAAGTCAGTACAAACTCTGAGAACATCATCTGCTGAGGAGAACAGGACGGGGAAAACAAACTAATGAAGGAGTCTTTTTCCGCATGATTTTCCTTCAAATTTTACCCACTTTGCACTAGTTCGCTCTGTTTATCTTTTTCGGCATTACAGTCGGCTGCCACCCCCTTAACACCGGCTTCCCGGTTCTCCATTAATATGGAAGTGAAGCATCTGCTATGGTCTCACTGAACAATTAAAGAGCACATTCTCCTTATTAAAACAGAGCTCCAGAGGAGTGCCAGGAAGAGACCACCTCTTGCTATCACACCGCCGTGTTCTCGCACTTAATAAA
LG17	ref-12951_24	54.942			TGTTCCTATACTGTAGGAATTGCCGCAATGTGACACATTCCCTCCCATGTGGCACAGATCAGATATGTAAACATGTAAACAGGAAAAAGGCGCATGGATTCTGATATTCTCCGATCGGATTCATATGTGGAAATAAATCTGATATGAATCAGATACATGCATCTGCGCCTGCCGTGTCAGCAGACAGATCGG[G/T]TATTCACATCAATGCGAGTTGTGTCATTGAAAAAGTGACTTTTTTGAAACATTTCGCGGTACAGATGTATCTAATAACAAATGAAACATCTTTAGTTGACTAGCAGAGTATTAATTTAGTTCATTTGTGTTAAATAAAAGGTGATCTGGTGCTGAAATGTGTTGCTTTTGAAATCAT
LG17	ref-26412	55.419			CCGATTTCTTCGATAATAGTGCAAGAGGGAT
LG17	ref-8210_29	56.048	Chr14	2088835	GAGATTCAAGACATCAATGATCATGCACCCGTTTTTTCTAAGAGAGAAATAATTTTAGAGATTAGCGAGTCCACGCCTATAGGCACCATTTTCTTGTTAGATACCGCAGCTGATCCCGATGTTGGATTAAATTCTCTTCAGAGCTACAGTCTGAAACCTACTGATCATTTTGATTTAAAGCAGCACAGTCGCTCAGA[G/T]GGCAGCAAATATGCAGAGATGGTGTTGCAGTCAGTGTTAGACAGAGAGAAACAGAGCGAACATTCACTTATATTAACGATTAGATACCGCAACTGATCCCGATGTTGGGTTGAATTCTCTTCAGAGCTACAGTCTGAAACCTACTGATAATTTTGCTTTAAAGCAGCACAGT
LG17	ref-45574_30	57.94			ATGTATTTCCCGACTATCCTGCCGCAGTC[C/G]AG
LG17	ref-64887	58.793			CTAAAGGGCACGAGCGGCTTGCAGTCCAATT
LG17	ref-63061_32	59.003	Chr14	14652539	ATTTACACTAAGTCTAAATAACATCTAAGTAACACTATGTACTATTTATTTTATTTGCTATTTACACTGTGTATATAGCATCTAATGTTATTTGCAAACAGTAGAAATCGCCTCTACCTAATTAATAATAAATTACACTGTGGCTCACTGTCATGGCTTTTTCTTGATCACGTGTCTTAAGTTCATATGTAAATGTGGTTCTGTCCAAAGCGATGCATCTGCCATGACGAG[A/T]GCATGCAAACAGTGCTGTGGACAGCAGATTAATAGCAAACTTTCTTTACACTCTGGCTGAAGATCAGGAAGCGTCGAGCACTTACAGGAAGAGATAACATTAAAACCACACAGAGATATAGAACCTCAGCAGCTATGAGGGTCATGATTTCTGCTTCATAACTCCAGAATTATTTTGCTCAAAAGGACCAGGCTTCACAG
LG17	ref-65524	59.317	Chr15	36672409	ATGTCCAAATCCAAGGTTAGCTTCACTTCCTGTCTCAAGAGATGCCTTGTTTTGATTGATTTTTGAAGGCAGCAGATGTATCCTTCGCTTCCTTTGATATCCCACAATCCTGTGCTTTCTAACAACGATGTTCTAAAAATGGAAAAGTTTGTCCTTTTTTTCGCATACAGACGTCAACGTCGTCAAAATGATCCCGTTCACGTGGATTCACGAAAAAGATGCTGTGTTATTCATGCCAGGCCAGTAGTTGGCGATGTCACTGTGTAAAAAATTTGGAGACCAACCATGTAGTAAGTACATATGCGCATGACGTCACCGTTTCACAAATTCAAATTTTTGTAGTTTACATGTGGGCAATAATGCTTTCGTTTTAAAAACATGCACTTTGCAACCCGTTTTCAAAGGTTTGCATTTTCAGGCCCCCAAAATGC
LG17	ref-54436_8	59.866			ACGTTCACACACAGTTCAGTTATTTAAGGGAGTGACAACTGCATTCTATAACCGAACTCACACCTGTAAGTTTTCAGGACTCACAACTGTATTCTGTAACCCAACTCACAGCCGTAAAGTTTCAGGATTCACAACTGCATTCAGCGGAAAACACACGCTGTGTGAACGGAACCAGACTGGACAGCTAGTTGTCTGTCACGTCATACA[G/T]TGCGAGTAGGCTGCCCAAACGCATGTCTACCATGTGTTGCGGGGTTTTGTGTGTGGTTTTGGTTTCATTAACTTATAGCGACAGAGATTTGAGATGTGTGTCATCTGAAGGTTTTAGATCCAGTCACTGTTTTCCACTGGAGCTGCTCTAGTCAGTTTTGTGTTCTACACATGACTCAAATGCTCCGTTCTCTGCCCAGATATAAAAGCTGCTGTCGGTTAATG
LG17	ref-65553	60.219			TACCTTCACACGAAGAGCCTGCAAATTGTTA
LG17	ref-41671_2	60.253			TATTCAGTTGTTTTTTCTCAACAAAGTGTCAGTAAAAGAAATGCTGTTGGCCTACCACAGTGAAGTCTCATGAAAACTGTTGACAAACAAGTAAAAACAAATGTTTAGAACTTTTAGAAGTTACAAGAGAATCATTTTACAAATATCAGTTAATAACTGCTGTAGTTTCG[C/T]CAGTAAGAGCAGTGAGATCGCAGACATTAATCAGTCAGACATTTAATATTTTGTGTTACATAATAGATTTATGCGTTTATTTCTTCAAAAAGTGACAGGACATCAGTTTGTTTCATGTTCGTGGCAGTGCCGGTTTGATCAGCAGAGTAAGATGTAAAACATTTGAAAGATGAATTCTGTCCAAACTCACGATGGTTTG
LG17	ref-5729_23	60.662			GTCTGAGCTTTTGTTTATTAGTATGGTGTGTTGTGTTGCTATTACTAAATTTAGATCTTGAGGCAAAACAATTTGAGAACATTCTTTGCAGATATAGTAGATGTTTTCGGAACCTATACAGGACTTTTTACAAGATCTTGGTTTTCTTTCAACTCACCTTAAATAGGATTATTCATAGACTAATCAGGTTATTGCAGTGTGATGATGGTGCGACATCAGTGCT[A/T]GGAAAGAATTAGTCTCCATTTTAATCTTCCTCAGTGTCTTCAACATGATACTCATTCTGATTCATTGACAAAACCGCAGTGTTACAATTCTCCTAGTAGGAATGATATGAAT

					CTGTTGTTGATTGTCTCATGCATCTCTTCTTATTGATTCATGTCACGCTAGAGATGAAGGGGCTATGGTATCCGCTACAAGATGATTGGAGAGACG
LG17	ref-59878	61.41	Chr14	16893397	GCCGGTCGAGATTCACCAGGCCACGGAAGGCGTTCTCAGACAGGACGCGGATTCGATTGCCATGCAGGAACAGGTGGGTGAGGTTGACCAGGTCGGAGAAGAGCCCGTC GGGAAGGTGGCTCAGCTGGTTCTCCTGCAGGTAGAGGAATTGCAAGCTGTAGAGCTTGTGGAAGAGGTCGGCGGGCAGCTCGGTCAGGTGGCAGCGGTGCATGTGAAGGC TCTGCAGACGTTCCAGCCCTCTGAACGCACCTCCGTCCAGCCTGCGCAGTGAGGGGTTATCACTCAGATCCAGTTCCTCTAGGACACGCAGATTGCTGAATGCACCTGCTTC GATCCATGTGATGTTATTGGAGTACAGCCACAAGACCT
LG17	ref-1508	61.525			ACCGAACCCACGAATTAAATGCTACAGTACA
LG17	ref-37555	63.62			TAAATTTCACATGTGAAATCACCAATGAAATACTTGTTTCTTATACTTAATAAAGCTTATTTTTTAGGCTTAATCCGCATGTGCAGTCCTATGCGCGCATTTGGCTCATTTAT ATAGAAGGTGGGATTTATTCCGCCATATTATGCGTTGCACTTTCTTCCATTCACAGTAACATGAGTGCACCGTCTTCATGTATATAAAGTCTTTGCTAATGCCTGCATTGGC AGAACCTCATAAATGACTGATTTCGGACTCCATGCAACTCCATGAACCACACAAATAAAGTTAGCATGTTGCTAAGCGAACAGCATGATACTCTAATAAGCTCAAATAGA CACAGCACAAACAAATACTGAGTAAAATAATAGA
LG17	ref-71406_8	63.742	Chr14	24661883	TGTTATGCTGGTGTAATGTCCTTGACATGACAGCTCCCTCACATTATCACAGGGGGAAACTGAAGTGGAAGAATCACCGCACCATAATTACCCTTAATAACCTTAATACAT AAATGTCCGAATGCAGCATGCTGTGATCGCTAAACCTTTTCACCCGTTAAACACCTGCAATGCTG[A/C]GAGCATCACTATCGGTGAGTTGGGTAGTGGTAATCTCTGTTAA ATTTTTTCATCACTATTTAATATTTGCTCAGATTTGGAGACGAGCAGCACGTAAAGGCCAGGAGACAACACACTTAGCTTACAATTCATGTCAGAACATCGCACAGTAACA AACTCTGCTTGGTGCTAAAGCAATGGCTTAAAGATGAGG
LG17	ref-58470	64.21			GATTTTTTTGCGATTACCATGCCTCACCATT
LG17	ref-29397	65.647			ATCAAGCTTTCGACAGATCTGCCTCACCTCT
LG17	ref-46555_29	65.81			AAGTCAGTATCGATCTTGGTGCTCGCGC[C/T]GTA
LG17	ref-59367_17	67.41	Chr14	5509724	TTTTCAGGCATAGTGAATGGTGAAGTCTGCACTGACATAAAAGCTTCTTCTGCTATCTGAGACAAGGACTTTTTCATCTTTTTGGTGACTTTAGCCAGTTTAACCTTTTGAAC AATGGCTTATTTACTAGTTTTACTAGTACTGATGCTGGTTTCAGTATCCGAGCCATTCCATCTGGAGCACCT[A/C]AGTCGTTTTGACTGGCAGAAGAGCCTTCGACCAGGTG AGGGATGCCCTAAAAAGTGCCACCCTGAGAGGTGCCCAGATGTTAGGCAGCTGCAGGGCTGTCCAGCCGGGCTGGTTCGTGACCAGTGCGGGTGTTGCTGGGAATGTGGA AACGACGAGGGGCAACTGTGTGACCCAGAGCCCCGGT
LG17	ref-44637	68.112			AACAATGCCTCGACACGGCTGCAAGCTGCAT
LG17	ref-48157_29	68.261			GCCTTGCTTCTGGATTAAGATTTTAGTCTTTGACGCCCTAAAGAAAGAGAGAGAGAGATTAAAATTAAAGTTTGAAAGTGACTGTAATCCGTTGAAAGAGATTAGAGATTA AAGATTAAAGTTTGAAAGTGACTGTAATCCGTTTTTAAACCTACAGTACTTAAACTCACTCATTGGCAGCACAGAGTTCGCATTCA[C/T]TGCTGTATGTTTTTCCATCAGAC CCACACACGGGTGAATAGTTCATTGGGCATATGTTAGACGGGTTAGGCTTGCAGTTAAGCTGATCACATAAAAAAAAAAAAAAAACAACAACATTGATTTATTTGCAAAT TGAGGGAAGTAATTCTAAAGCCATTACACATTCAGATTA
LG17	ref-68986	68.998			ATCTACAGGCCGAGAATGATGCTGGTGCAAC
LG17	ref-40692	69.35			AATTCTGATAGATTAGAACTGTTATGTATTAGGCTTTCTGAGTAGCTTCAATATTAAGAATGTAAGTTTGATGCTTTCTGAGATGATGTTGTACTGCACAGCTGATCCTAAA TTAACAACAGTGTTAAGCAGACAAAGCAAAAGTCTTTCTGAAAAAGTACTATTGTAAACGTTAGCATAGATAGATATATAAATGATAGAGTGGCCTGGCAAACCTGTTGC TTTGTCAAACCAATGTATGCTGTTTGATTTTAATTGAAGGTTACGATATGTCTAAATGTGCTTCACTGTGAAGTGTTTGCTATAATACTTTGTATGAATCTTTTAGCCAAGGT TTATTTTTAATGCAGACCTTTTCTTGTTAGAGATATTCAGATAATATTGTTTCATTGGAGTTGCAAGGCACAAGGCCTGGGGCCTGATGTATAAAT
LG17	ref-44530	71.973			ATACAGGTCGGATAACTGTTATAGGTGATATTGATTTTGAAAAAGAAAGGCGGTTTGAAATAAGAGTTAAGGCCAAAGACCAAGGGGGTTTGACGAGCACTAGTAAACTT ATAATTGATGTAACTGACATAAATGATAATGCACCTGCGATAAACGTGATGTCTCTCTCCAGCACTATAGCAGAAGATTCGCCCCCTAGTACTACTATTGCTATAATTCATG TGAAAGACGCGGACTCAGGAAGAAATGGGCAGATTACGTGTTCTATTGACAAAAACCTACCGTTCAGTATCAAATCATCGCTCTCAAACTATTATAATTTAATCACAGATG CCTTTCTTGACCGTGAAACTCAGTCAGAATATAATA
LG17	ref-51266	72.538			ATGATGCTTTTTGATGTTTTATAAGATGCCCGAGAAAATGTGTGATATTAAGCCTCAAAATAACGTATGCAAAGGACAAAAAATAGTTTCTTTAAAATACAGAGAATGAGA ATAAATTAAAATAATTAAAACATTAAGGGAGGAAAAATCCTCTTATATTTTCACAACCTGAAAAACAAGCATTATGGTCGAGCAGGGCGCATGCACGACCTCTTCTCTAAA AATCTAAAAAGGAAATTTTGCGTGAAATGTTACGTAAATCTCTCTTTGATTATTGATGTGGATTTTCTC
LG17	ref-17983_5	74.015			CTGTACATGTTGCACTTAACTGTATTTTCCTTTTTGAAGTGATTTCAATCATATCTCAGCCAAATTTTAATATATTGGCCAAATTTTATTATCAGGCCGACTGATATGGTGGC CGATACATCATGCATCACTAATTTTAACTTTATATTCTTCAAAGAATCTACCAGGCGAATATTTTAAGTCACATGTTTTGAGGAATCACTA[A/C]TGTAGCGAAACATTTGCA

					AGGTGAGTTGTGTGCCGAAATTTAAACAAGATTGAACAAACCCCTAATCTCAAGTATTAATCTATGTCATGGAACTCATCAAGCAATGTTTGTACTGCATGAGTGCTACCTGAAATCATCCTTTGTTGAAATCCATAACCCTGTGTAGGATATGAATAGTGCCACTAAACACATCTCTAGGCCCTGTTTCCACCTGGTATTAAGATGCG
LG17	ref-23150_32	75.131			ATCCAACGTAGTGCAAGTTTCTTCGTTAACATAATAAGCCATACCATCCCAAAACACACTGCTTTGATCTGCTGTGAAGTTTTACCAATTGCAAATGCACAAGCTGTGACTGTGTGTGTTTCTCTGTCCCCATAATAAAGCGCGCGTCTGTCCGACAGATCCACTGCGCCACGTGCACGTGAGCGCGCGCAAACACGGATCAGACTAGCGCGAGATATCTGCTCACTGCATC[C/T]GAGCGGGTGCCATTCACTGTCGTTTAACTCAAACCTTATGGTATTTTTGATCAATTAATTTATAAGTTGTATCTGAATCTGTCTGTTTAATGCGCATGCACATTTATTTTTTTATGCTGTTGATGCAATATATATGCAGGAAACTTAAACTCTTAAATGTTTTGGTCTAATAAGAAAAGGTCAAACAGATTAATCCAT
LG17	ref-1434	75.827			ACTTTGAATTCGATTTTTGTGCCGACGGCTT
LG17	ref-56300	79.638	Chr24	4849520	AGTATGATAACTATAACAATAAAGATATAGCTCTAAAAATAGTTCTAAAATATAAAAGAATATCAGAGTCTACACCACAACTATAACGATAATAACGACACAGAGAAACGATACCGCTGTTTTTTTTTTCCATCTTATGAATTATAAAAACATTGACAGCCAATCAGAATCCATCCTGCTTTAAAGAGCTCGACCATTTAAAGCAACAGACAACAAAACTGCAGCGCTCTGGAAGCTAATATGGTTATTGTTATCTTTATAGTTATCATTCCTAGTGTGAACGGGCCATAACAATAAATATATAATTTCAAAAATCAGATCAGTATAGTTGGAATTACTTTTAAGGCCAGTTCACACCAAGAATGATAACTATAAAGATAACTATATTAGCATCCACACCAGCGGATGGAATCATTCTGTTTATTATGCTG
LG17	ref-25275	80.587	Chr14	15451003	CCTGACCCGTGTTCGCGTGTGCATTGGAATCCTACACTGGGAATCACACCGCATAGAATCAGTGTTTCCAATTGTTCCGGCACAGGCGTCTGAATGAGCTTATTTAGTGCCGTCAGACAGCAGGCGGGGTGGTCAGAGGAGCATGGGCTCTGCCGAAGCCCTTCAGAGACCACAATAGGAGCAGCTGCAAGGCGTTTGGGAAAAGTGTGACGACTATAGTGCACTCAGCGTTCGGCCAAGCACACGGTATACTGTGCCTCAGCCTTTGTGTCGTGTAAGATGGTGTTTTCACAAGCCTCAATCATGGTGGCTGTAATTAGCACAACACTGGCAGCGATACGAGCCTTGAACAAGAGGCAGTTCAGATTGTAATGATCCAGTGAATCTGGGAAGTTGACAAATAACATGGACATTAACTTTTTAAATGAAA
LG17	ref-45096_4	81.228			GAA[C/T]TTGCATCGATGATTGTGCCAATATAATC
LG17	ref-45096_3	81.453			ACCTTCAACCTGTTGAACCCCAGTGAAGTCCACTATATGGAGAAAAATCCTGGAATGTTTTCATCAAAAACCTTCATTTCTTTTTTACTGAAGAAAGAAAGACATGAACATCTTGGATGGGGTGAGTAAATTATCAGGAAATTTTGGTTTTTAGCCTATGCAAGCCTACATTTTTTAAGCACTGCAAAATCAAACTTACTGA[A/T]CTTGCATCGATGATTGTGCCAATATAATCTAATTTATTTTCTACATTAAAGTTTATATTTTTAACTACACCTCATTCTTAATATTTTTACTATCATTACCGAAATCAGCAACCTCATTTCTGTAACTTATGTATCATTACCGCAACAGGTGATTATTTAATGTTTATTTAAAAAATGGAAAAATAGTAGTTTGCTTTTAAATGCATGTGCAGAATCTATACAATATGTT
LG17	ref-34698	84.354			CAGGCAAAAAGTGTATTTTTTTAGCCCAGTGCAGCAGAACAAAGGGAAGTGGTAACTAAGGTTAGGAACTGAGAACCAGTTCCTACCCAGAACGAAGTGCATATCCTTTAATAGAGATGTCTCACCTCAAGAACATTACAACAATGAATGCACTGAAAAGCCCTCGTGCTACTCATACGTCAATGCAGAGAGAGCGAGAGAGCGAGAGAGAGAGAGAGGGTTGCACAAAACTGTACGATTAATCTTTAAAAGATTGCGTTCTTGATTTCATCACACATATGATCCAATTCCTAAATGACAACGATTCGCCTGTGTAGGCTATATTACATCTTTGACAAAAATAAAATTGCGACATTCAAATCTGCTTCACATACAAATATTCCAGTTATCACAGATGTATACAAAAGTGTATTATTCAGATAAACGCTGATG
LG17	ref-57927	85.291			AGGACATTAATGATTGATGGGCGAGGCCATAAACAGAGGGGCGTTTTTGAGAGCGTTACCTGATTTGCCCACATCAGAGTGTGGGAAGGGTTTAAAGGTGGGGTCGGGTGAAGGGGATCATTTTCATTGCATGGTATATAAACACCCACCAGTTTGAAAACACCCACAAATACAGAAACGCCCACTTTTGTTCCTCAGAGACCTCATATTCGAAATCCCTGCCCTGCAACCAATTTCATTGGCTGAGGTTACAGTGACGGGTAGGTTTAGGGATCAGGGTTGGGTGTAGGCAATCATTACTGTGATTGACATGGCCAATGAAATGTTTAGAATCGGCTCCAGGGCGGGATTTCCGCTGAACTAAAACAGACCAGAACGCATGCAAGCTACAGCGACGATGGAGGCCTACATAGATGAGAGACTGTGTGAAG
LG17	ref-64134_26	85.36	Chr14	16145066	GATCCAGGACCCAGACGCCGTCAAACCCGAGGACTGGTGAGACACAGAGAGAGTGTGTGTTGTACTGTGGATTGAAGTCATCACTTGTTGTCCACTTCCTCATTGTTTGGTGTGTTTGTGATTCAGGGATGAAGATGCTCCCGCTAAGATCGCTGATGAAGACTCTGTGAAACCTGACGGCTGGTTGGATGATGAGCCGGAGTACATCAGCGACCCCGATGCCCT[C/G]AAGCCAGAGGACTGGTAATGCATTCTCCATTACATTACAAGAAAACCCTGTTCTTAATATTAACTCTTTTCCGCCAACGTTTTTGAAAAAAGTTGCCCGCCAGGATTTTTGATAGTTTTCACTGAATTTTCATTTTGTCGTATGAATATCTGAACATGCAATAATTTAAAATAAAGAACAGAGCCTCTACTTGTAAACAAAAAAAA
LG17	ref-52436	86.459			ATATTCATTCATTCATCTGAGTGTTTTATAATGGATCTGACTGACTCTGCTCTTCCAGATGTTTCATACTTACATTAACACAAAACCTAATGAATGATCGTTTTTCTTTTGAGAGTCAAAGAGCATGTGGATTGTGATGTTGACATATAAAATGTTAGATGATTAGAGAAAGGCTGTTCTGGGTGGTTGCCAGAGTGTTGCTAAGGTGTTCTAAGTGGTTGCTAGGGCGTTGCTGAAGTGGTTGCTAAAATAACAGTGTAATCGTTCTCTGTAGCAAAACTAGTGTGGGATGAATTATGCATTAAATTTCAATACTTAACTCTTTTCATTTTCTCCAAACTTTCATGACCTCCAGAATATTATGTTGAATGAATATCTGAACATGCAGTAGTATATCAAAAGAAAGAAGAAAAGAGCCTCTGCTTAAACCA
LG17	ref-26139	88.07			TCACTGATTGTTTTACATCTTAACGGAAGTTACTTTTACTTTTCTCAAGTAACTAATAAAGTAAATTACTTTTCAAATTTACAACAAAATATCTGAGTTACTTTTTCAAATAAGTTTGTTTTCCCATTTATTGACTGACAGCTCTCCTGTCACCATGTTGAGAGAAATCGAAAGTGCGTGCAGAGGCGTCGTGTGTGTGTGTGTGTGTGTGTGTGTGTGTGTGAACATTGTAGTTCTATACTAAATGTGAGCTTGCATTCACTCATCTCACTTGCACAAAAACAGATTCATTATTTCTCAAAATTAATAAAAACACTGAAATGCAAACTCGTAAATGTATGCAAACCTGCAATAATTATATAAAACAAATG

LG17	ref-59181	88.698			ACATTGTGTTTGTGGCTCATGGCAGAATTGTCGACCAATTTATGCCAGCAGGTGGTCTGGTGCCCACACCTACCATCAGAGACACCATTCTTTACTCTCCGTGGAACTGCGC<br>TATTGATTCTCACGCTGCATTTGGAATTGCTCAGGGGTTCATTCAAGTGACAAACAGAGAGTTTGTCAATATGAATGACAATATGCTTTACTGTGAGCCGAACCCTTTGCCA<br>CGTCACTGGAATAGCATGCGAGGATCTCGCCACAACATCCCAGTGATCCTTCTATCCCCTGTAACCCCTGAGGAAGAAGCATGGGCCTTTTTTCACCAGTTTTGGATGAAT<br>AGAAGCATGGAAATAGAAGACCGTATTATTATCCCATATCTTGTCCCACAAAATATGGTAAACGCATTTAGAGAGATCCCCCTGTACATGTACATA
LG17	ref-59057	89.997			GTTATAGTGCAGTTTACAGAGATCACGTGCTCTTCTATAGCTGGACGTCACTGCTGTGTGTCTGATGCTCATGTTGTGTGTGTTTGTGTTTCAGGCTGCTGGTGAGATGAATT<br>CAGACACACACATTGTTACAGGTAAAACCTCAAGAACAACAATTGTGGCACGAGCAAACCTTCCTTTATATACTAACTATAAAGATGTGGATGTTTGCAACATGGTTGCTG<br>TGGTGGTTTGACCTTTCTGCAAAATTACTTTATTGTCTGGCAGGTGAAATTGTTTAGTGCACTAGATTATAAATGATCGTAAAGGTGCAATATGTAAGAATTTTGCAGTAAA<br>ATATACAAAAGCCACTAGGCCAGTGTTATATATTTTGTTCACTTGAGTACTTACAACATCCCAAATGTTTGCAACTATTTGTAAATCGTGAGAAA
LG18	ref-63025	0			TAGAGAGTCCCGAGAAATGTGCCAGTCACAG
LG18	ref-22373	0.466			TTGGGGAACTCGATAGTTTTGCAAGCGAACA
LG18	ref-10209	0.606			ACACTGCACACGTGTGAAAACTGATGACCGGAAAGATGGCAAGCGACACGGGACTTATTCTTGTTAGAAAATAAAATTTAAAAAACATAACGCATGCATCGATAACGCAG<br>AGCCTCTTAGGGCTTCTGTAGGGTAATGGTGCCGTTACATCTGATTTGGCATACGGTTCAGTTGTTCATGCAGATTTGCCGTGTTGTGCATAAGAGAGCTACTTGATTTGAA<br>AGTCGTGTGTGAGCCGTGATTCATACATCACTACATCTATTTTTAGAAAAGAAAAACCACAACATTTCACTAACAAGACCACATATTTACAAGGGTTAGCCACTACTGTGA<br>TACGGTGAGAATCGTAATGCAGATCTATCAGAGGAG
LG18	ref-24268	3.989			GTTTCTACCGCGACCACGTTGCACTATCTTG
LG18	ref-25024	5.753			GGGACATTTAATGAAAATGCAATTTAATTTAAAATAACATTTAAATGATCATTTTGCTACAGTTTTTGCTTAAACATATCTAGCCATTTCTGTAAAACACTGTCATTTTTTCA<br>CTGCGCATCTTTTGACTTTTGACATTCCCCTGACACATTAGAATGAATAACGAACATAGATGAAGTGCACTGATGTCGTAAGTGATCATTGTCAAGGTGAATTATTGACAGT<br>GAATCGTAAGTGATCGTTGTCAAGGTGAATTATTGACAGTGAATGTCATCACTGAGGTGTTAATGCACACATGCATATAGGGTATGTTAGCTACATGTACGTCTGTTTATTT<br>TATTTTATCTAATAAAAAACTATGGGCTCTGT
LG18	ref-57319	6.662			ATGTGGGCCAGATCTGGGCCAACACTATGTTGCTGTCTGGGAAACCAGGACCCCTGTATGCTTTGAACTATATGTCCATATGTGGAGATTTTCTAAGTTTATCTATGTCTCA<br>ACCAATCAGAATGATTCAACCTGAAGTAATGACGTAGTTTAGTGACCTGTGTTTGAGTATAACTGCTGTTGTATTGAGTGTGTATGGAGGGTGGCCTACAAACTTCTTGCTG<br>AGTGTTGAACCTGACTTCTGCTGATGAAACTGCAAACTATTGCATCTCTGACTCCTGGTCTTCATTCATCATATCTTGTCCTCAGGTCGTAACTGTAAATTCCCCTACAAAAC<br>AACTAAATGACATGGGCGAGAAAGCAGCAGTTCAGAAAGCATCTGATCATAGCGATATGGATATGCTTGCACAAGCTCTGCAGTTCAGCAATTA
LG18	ref-1826	7.791			ATTCTGTTTTTTTCTTCTTGATTTGAGGTTTCTGTTATTTCATAAGTTAACTCTCATTTCACTCTGCAGATGAAAGTGCATTCTGTATAAATTTGGTATACCGGCCATTTAAAG<br>TGGTTAACGTTTGGCCGTGTTAACCACAGACTAATATATATATATACACACACACACACACGCGCACACACCTCGTTAGCAACTGCTATGAGCCGCTATGCGTAAGCTG<br>TCTGCCATATTGGAACAGTCAAAGCCGGTCCATGAACACTCAAGAGGCTCAAGTTGACTGTGCTTCTGCAACCGTAGTTATACGCCTATGTATCTATATCTGCAATAGACTT<br>CGGCTGATGATTTTCATAAGATTTGGTTGATC
LG18	ref-15983	8.356			GTGACACAAACGAGCCTAATGCTTCAAATCA
LG18	ref-67276	8.735	Chr21	45546825	CTGTGTCGTCCTTTCAGCTGAAATCCTGGAGCTGGCAGGAAATGCAGCCAGAGACAACAAGAAGGGCCGTGTGACGCCCAGACACATCTTACTGGCCATCGCCAATGACG<br>AAGAGCTTCATCAGGTACATTCACAGACGCCAGCCTTCTCACGTGCTTCTGGTCGCCGGTGGAACTCGAGTGGTCAGATCAGACATGCCTTACATGACCACGACAAACATT<br>CAAAAACACTCCAGACACTGACTTCAGTTGCTATATCTTTACAAACTGATCCGACCGTTTTTTCTATGCTAACATGCTAAATAATGCTAACAACATGCTAATCATGCTAACA<br>AAATGCTAAATCATGTTAGCAACTAGAGTTGGGCCGATAGATAATGCCATTTCCATCGCCGATGGCTGACAGACATCATGATGTTGAGCAGGCAACAT
LG18	ref-41909_1	10.091			TTGTGCACTTAATGTAATAAACATCATACAGTAGTGAAAATTAGTCAAGATAATAACTATAAGACACTATAATTTTTGATCAGGCATTACCGCCTGGATCCTAAAGGAGAC<br>CCAATTCCTGATTCACCTCAGATCTTGAAAAATAAATGAAGGGAATGTATTTTTAATAATGGTAAAAAGTTGAAATATATATTAATTCG[A/T]TTTTTATTGCGAGTGCAGT<br>GCACTTGCAGAGAGACTTCGCCCATGCACTCTTCTGATTGGCTGTGATTTTTGATTGATTTTGGCGCGTCTCGTAGTCATGTTGCATGTGGTGTGGACAGACAAACTCCTTG<br>TCACTGGAATCTTATCGCATCACGTCTGGTTAGGACTCAAAAAAGTATCTTAAAATGTGTCAATTAAGTACTGCTGCTTATATTAATTCATATTCATTCA
LG18	ref-48044	11.497			ATCCTCTATTCGAGCTACATGCCCAGCCCAT
LG18	ref-24138	11.602			GACAGCAAAGCGAAACAGGTGCACAACAATA
LG18	ref-45220	12.782			TGTCTGTCTGTGTACACTGAATTAAATCAGTTTAACACAGTTCAGTACAGTGATAGTCATGAACACTTAAAAACAGAGTGTTTTCAGAGGAAAATATCAGTGTTTTGTGTTC<br>CAGTCTCATTCTGAATCATCGACTGACAGTGAAGAAGAACATTCTGCCGGTTAGAAGAGAAAAACTGCTGCTTTCTGAAGAGAAAAACACAATGTTAGAAATTAAAA

LG18	ref-40995_6	13.002			GGTTGTTTTTCATGTCCGCGGGTTGAAGCGACCTTCAATAACATGATATTTAGCCCCTGGAATATGAAATTTACCAGGGGAACACCGCCAAAAAAAAATGCAATTTCCCCCCGGAACGTGATTTTTACCGGGGTACCCCCCAAAATGTGATTAGGGTAGTTTTGGGCTAGTTTT[G/T]CGTAGCAATTTTGTCGTGAACACCTGGCAACCGTGTTTCGCACTCTCTCTCTCTCTTTACGTGCATGAGAAACACAGTGCTCTCAGCAAAGCGATGGCGAGTCATGTGCCTTCACACTAGAGTTTGCGGTATGTCAAATACAGGTATGCTGCGCATTCATTGAGCTGAACTGAAATAGTACAGATCGCTTGACAG
LG18	ref-59922_24	15.073	Chr9	49795373	GCCCCTCCAGTTCGAACTGCCTGCTCTAAGCGAGACTTGAACCCGGGCACAGCTCTTACCGGCCTACGTCCGTTACACTCACCCCCCTAAACTTCAAACCTATCCTGGTCACGGCACCAAATGTAGTCCCTTCAGTTCGAACCACCCGCTCTAAGCGAGACTTGAACCTGTTCCTGTCTGCATGGGAGTCGG[A/G]CGCTTTAACAAGGAGGCTAAAGACCGCAGTCTCTAGAGCACATCTTGAGATTAGGGTAGTGAGGTTTACACGCACAGCTCTTACCAGCCTACGTCCGTTACACTCACCCCCCTAAACTTCACTTCCATCCAGGTCACGGCACCAATGTAGCCCCTCCAGTTTGAACCGACCACTCTA
LG18	ref-59922_30	15.073			TTAAAGCGCCCGACTCCCATGCAGACAGG[A/C]AC
LG18	ref-50805	15.319	Chr20	32695475	ATATACTATTATAAAAATATAATAACTCAATGGTACTAAAATAACACTGGTTTTGACTAATATAATTCATTATTAAGTGTGAAAACAAACATTTTCTTAATTTTTTAATGATTAAGGGGCCATTCACATATCGCGTCTTTTTCAAATCTAGGCGCGCGGCAAGTGCGATCATAATTGAAGCAATGCGGTCGTGACACGCATGCGGTGCGGCACGCTCGGTTTTTCCAGGCGCGCCTATACCGCGGCGAGATGAAAACATCTCAACTTTTCAGAATGCCGCAAGCGCACCGCAGGTCATGTGACAAGAACCAACCAATCAGCTTCGGCCTTTCCACACGTTTTTAGACGCGACATGTGAACGGCCCCTAA
LG18	ref-61259_17	17.039	Chr9	22771514	CAATGTGGGTACAGTGTAATATTATATAGGTATAAATTCTGTTTTAATTGATTATTAAATTGTTTAATTATACAAAGTTTCCATGAGTTCAAGGTAAATCTTTTTATCTTACTACTGGAGCTGCCAGTTTGCAGCGTTTCATATGATTATTAAACAGCAGCACAAACTATGCAAAAGCAGCAACC[A/G]TTTCGGCGCACCCAGTGTCTGAATTCACTTATTCATTTTCATTCACTCCCTCAAGTGGACTGTATTAGTGGACTAATGTAGGGAATAGTGAATGAGGGTATAGAGGGCGATTTCATCACAACCACACTCTCGCACTCACCAGTATACTGATCACACTCACCTACAGCTTGTTATCCGCTTTTTT
LG18	ref-60675_1	18.661			TATACAAGCCGAATTTAAATTCCTCCGTTATCTTGACACCTCAATGTGGAGATATAGAAGGAATAAAAGGCTCACAAACTTAAATGCGGGACAGTGGAGGAGGCAGATATTAACACCTGCCTCAACTCTCTCTGCATCTAATTCCTCAGAATTAGATACGTCACATTAATCAGTCAGGTCAGAAGTGGCCACAGAGTGGGT[C/G]CCTGAACCGACATGAATGCAACATAGCAAGAGAAACCCAAAAGGAAAAAGTCCCATCAGCCTCTTCCCTATGCTCCGCCTACAATTCCACCCTGCCACCTTTGCGACTGAAGCCCAAAAGGAAAAAGTCCCATCAGGCTCTTCCCTATGCTCCACCTACAATTCCACCCTGCCACCTTTGCGACCACAGGACCTGAACCACGAGGTCAAACTCTAATCGCTCAAGAAAG
LG18	ref-15488	20.301			TTCCTGCCTTCGAAGATGATGCAGCTCACAA
LG18	ref-41896_1	20.813	Chr21	36203065	TCCCATCTACAGGGCGATGGGTTGTGGTTAGCTGAACCTCCAATGAAAAACAGACCTGGTGGCAAATCAAAACATCCTCCACAGAATTAAACATATTACGACAGGAATAAACAATCCGCTCCGATGACCAGGGATAACGGTCTGGGCTCGGCACGCTGATTGAAAGCAA[A/G]GATATCGCTGCATTGGCATCGTGAACCCTTCTTTTTTTTCAGAGGAAGAGGATTTGACGGAGTGCAGGAAGGACAGAGGGAATGGAGTGGTTGGGGGTGTCAGTTGTAAGACCAAACTCTGCATGCTTCCTTATGAATGACAAGGGCCCATGTGTAACTATCAGTAAATAAAAAGTGGCTGTAAAATTAACTACAAGACA
LG18	ref-13921_10	21.958			GCTAGGCGACTAAATAATGTCTATCATTAGTCATTGGCTAATAAATTGTTAGATTTCACTGGCCAGTCTTTGAGTTAGATGCTAAGTACAGTATAAGTTTAGCATAGATATTTTCACTCGCCTTGTCTTGAGTTTCGATCTCCGTCAAGCACACTATTTCAATTCATCTCAATTGATGT[C/T]CAGTGTATTCGACGTACCGTAAACTCTAGTGTGAAGATGCACGACTCGCTTAGAGAGCGCTTCACAGAGTCTTACACACTACATAGAATTGACGAACTACATGTAAATGATATTCTCTGTTGTATTTTCATGTCAAAAAAACTGAGTTCATTCGGAATTATTCAGAGTTTAAGAACAAGCAGAAGCCACT
LG18	ref-48806_31	30.549			TTCGATATGTATCGATACTGAAATTATTCCAGTACTAATTTTCCATAGTATTGATACACTGACAGCTGCGGAATCTTACTCTCTCTTCTATTTGACGGCGAATTGACAGTTTGACACACACACCCGCCTCCTCTCACTCACTCGTCTCATTTGCGCCGGCTGAATAGTGTTGTTACAGGGCACAAATTTCGGCATGTGG[A/T]TGTGGGTATTGCACATAATTTTACTCATTCCTGCAGGTTCGCACTAATCTACTGTATATAAGTGGCATACACACAGAGCCGCCTCTCAAACAGCTCGTGAGTACCAAATTTAGTTATTTTCCATGGCTTATTGCAATATGTAAGATTTTTGCAGTAGTTCTTTTCCATGG
LG18	ref-48806_32	30.736			AACCACATGCCGAAATTTGTGCCCTGTAACA[A/G]
LG18	ref-8005_31	32.527			TGGTCCATCATGCAAATGTGCCAGATTTAGCCATACAGGCTAGTTTCCATCTGTAGTCCCTGGCAGGTTAATGGTAAGAAAGTACATTAACACTTAAAGGGATAGTTCACCCAAAAATGGATATTCCCTCATCATTTACTTACCCTCATTCCAAGTAATTATATTGTCTGTGAGTCCATATAATGCCACTGAATGGCTGCCGCTCTGCAACGAAGTTCATGCTGCATAAC[A/G]AAACTAGATCCTAAAATGTTGGCCTGGTCCTCTTTCATCCTCCGTGGGACTCGAGCCCTCGATAGAGGTCTGATCACCAGGAGCACCAGTCCTACCACCACTAATTAGCTATAATTACAAATATATTTAATATAACTAAATAATTAGCTACTGTTATTGGATTATAGAAGTGTGAATATAGAAACATTATTATGGGGATCC

LG18	ref-62839	33.602			GATATATTAGGTGATATTTGTAAATTGTCTTTCTAAATGTTTCGTTAGCATGTTGCTAATGTGCTGTTAAATGTGGTTAAAGTTACCACATTTATGTTTCATCATGTTTCAAATAACATCATTATTTTTATTTTTCAACCCGAAAATGCTTGTAGTCTGTATAATTCATAAACGAATCTGCATTCTTATCGAGTCTGTGTGTAACTGTGTGTAGCTTTAACCATGTTAGCTGTCGTGCAGCACTATCAAACTCATTCAGAATCAAATGTTAGCAAACATCCACAAAATACATGTCATACTTATGTGATCTGATATGCTGCATCACAAACAGTTTGTAAAGATCCATTTTGAGAGTTATATTGTGAACTT
LG18	ref-35932_23	34.548			TTAGAGCGCCCGAGACCCATGC[C/T]GGTGGACTG
LG18	ref-58881	34.977			AGCGCACAGTCGAATGCACTGCCTTGCAAAG
LG18	ref-72818_24	41.197	Chr6	25222012	CCTAAAACTGATCTGGCAAATCGCCCTGATGGTGGCGGTACAGCGCTTTTGAATTTTTTAAAAAAACTACTTTTGCGAACTAGTCCTAGGTTTTTCACCCAATCAGAATCAAACAAGTGCAGAAAGATTATCTGTTGTCTGATTGTCAATATCAAAAAAAGTCTAAATTTCTATTCATGGCAAAACATTCGA[A/G]GTGGGCCTGGGCACTTACTAAAATGGCTATCACTCTTGTATGGAATGAGATCATTTCACCAAACTCGGTACACCTATGTACAGGCTCAGTCTGTGGTCTCCTGAAAAAGGACGCAGCAACTGGCCACTTGGTGGCGCTATAAATTAAAAAAAAAACCCATGAAAACAGCTATAGCTAT
LG18	ref-60373_17	42.821	Chr21	35484318	CAATACGTCTCCAAATCAAGAAAGCAGCTTAATATTTTATTTTTGTAAGTGGATGCAGGAAGCAGACAGAAGTTTGATGAGATTGTTGAAGGTTAACAGTCTACACCGAAACTAGACTCTTGGATATCTCCCAGACGATGTCTAGCCATTTAAGGCTATGACACTCCCCATTCTTAAGCGAAAGACACTTAAGCTCAGGCTTTGAATAACCGACAT[A/G]CTTGCCAAGTGACACTACAGAAGACACTGTGCAGATTGCAGTGTGAAAAACCATTAGGCAACATACACCTTTGGAGCTCATACTGATGACGCAAACTGCTGTAAGTCTTAGAAGATGGCTACTTATCAGGCTTTTGAACCAGAATAGAAAAGTGCCCCTGAAACAAGAGACAAAACTTTCAGCTTCCAGTAATCTCTACATTGAGCAATGATAAA
LG18	ref-41997_2	44.133	Chr21	35312617	CTCTGACCGCACATGTCCAGTGGGGGGCAGTGTTGTTTCAAGATTGATTTTAGTCCCGTGCTCTACTATACATCCAATTTCACATTCACATTCTTCTCTCTCATCAGGGCTGGTGGAGTTGGATGAGGGCAGGAAGATAGTGTTTGCTCCGGGCCAGTCTATTCCCCTGACGATTGTCAAATCAGATGGAGGCTATACTTA[C/T]GATACCTCCGATCTGGCTGCCATCAAAAATCGTCTGTTTGACGAGAAAGCTGACATCATCATCTACGTGACGGACAGTGGCCAGGTGCGTGGGTGTAGGTCTGGATATATGGGTTTGTAATGGAGAGTGTTGGGGAAGTCTTGAACGGCACAAACCTTCCCACAGTTATCTGATGCGTATGAGGACACAATGCAGCAGGGGTCAAGGCCACTGGGCGAGACACAAAAACA
LG18	ref-38816_3	45.839			TA[A/G]CCCTAAACGAACAAACTGCTCTACAGAGC
LG18	ref-66653	46.074			AGTCAGTTCTCGAGGGAGCTGCCTGTGCTGA
LG18	ref-3009_30	47.861			GATTCAGTAACGAAACTGTTGCCCGAAGA[C/T]GC
LG18	ref-2296	48.769			TCAACCCTGTTGTGTTTTGATCTTTAAAAAAAGGTCAAGAAAATAGATTGATATATTGAGATATGAAACGCCAAACTACAAAGAAATCTTGGTGAGCATGATACATATTGTTTTTATTGTATCTCTATGGCTGTTGATAAAAATTTAAATTGCATACCTTGATCAAAAATGTCTTGCATGCAGGTCCATCATGATAACACATATTTAAAAATTAATTATGACAAATGTACAGTACCATTAAATTAGTGTGAAAATATGTAATGAAAAGTAGATGTATAAATAAAGATAAGTATATACTTTCCTCGATTAAATCTGGTCAGTGTTGTGCTTTTAATTTTTCTTTTCAAGCATTGTAAGGCTAAACTATAT
LG18	ref-10458	49.716			GAATGACACTCGAAACCTGTGCAAGGGGTTA
LG18	ref-17470	50.794			TGTAGAAACTCGAATGTGATGCGTAACACCA
LG18	ref-41178_10	51.874			AAACCTGAGTCGTTCAATAAGGTAGCTCAATTTATGCTAGCTAATCAAGTTCAAGTTTGATGTGTACATTTTCTTGTATTCTGTAATTCGCTTTAGCATTCTGACAATTTTTTTAAACTTACGCATAGGTCGAAAACCACAAGTGCAGTGGCCCAGTGGAAACATCTGTCGAAAGAATTCAGAATCCTCAGGTAACAGAACAAGGTATG[C/T]CGAGGCCCCTGCCAGAATTACAGACTCCTCCTCCAGAAGACCTGTCTCGGAGCACTCCTTCAATAAATACCACACCATGTCAAGAGACTCAGTTGTTGTCTGTTGACACGCTAACTCCACCTCCAGAAACAATCGCATCACCATGTGCTGAAATGTGCCATAGGGAGCAGCTTCAAAGACAAAATGCTTTAGCCATTGGTGAGTACCTTTTTGAAGGTTTAC
LG18	ref-40313	52.292			AGGATTTCCATGATTAATGGATGGCTCTATTGTTTAGCCCGGTCGATCCATATTCAGTTGCATCTGAGATGTGACTTCAGATCGATGTTTAAATTGTAATATGAGGGTTGAGGAATTTGGGCTGCGATCCAATGCGTTTTTGAGCCAAAAGTGTCATTTGCACTGCAAGACATTCCTGTGCAGGAGACTGCACCTTGTAACCAGCGAATGCGAGCACGCCGCAGACAAAGGGGTGACCTGATGCAAAGCGACCTTACGGTTCAAACCACAATATGCGGGAATCCTCCTGCATCAGCTGAGTGTTAAAAGCATTCGCAGAAGCCCTCAAAAATAAAGGAATACTTAGATCAATATTTTCAGTGCATGCATATTTCTCTACTGTATAAGGGGAGAGGAAAAATACATAGTGAAATATAATTTCCCTTAATATT
LG18	ref-49193	53.11	Chr20	25419272	GAGCGCTGGGAGAGAACCGCACCTACCCCCACCTCTGAAGCATCGACCTCCACCACAAACTGACGTGTAGGATCAGGGGCAATGAGAATGGGAGCTGAAACAAAGCGGCTCTTGAGGTTGGTAAATGCAGCTTCAGCTGCACTAGACCACCTGAACGTCACCTTGGTGGAGGTCAAGGCGGTCAGAGGAGCGGCTAGTTGGCTGTAGTTGCGAATAAAACGCCGGTAGAAATTGGCAAACCCCAGAAACCTCAGCAGGGCCTTGCGGGAATCTGGGGTTGGCCAATCCACCACAGCTCTAACCTTATCGGGATCCATGCGTGTTCCCTCGGACGAAATGATGTACCCCAAAAACGGAACCGACTGTGCATGGAATGTGCACTTCTCCGCCTTGACAAAAAGCCCATTCTCGAGCAGCCTTTGGAGCACCCG
LG18	ref-32438	53.6			TGTTCTCTGTCGACAGTAGTGCTGTTCAATT

LG18	ref-29620_28	54.042			GCAAGGAATGGTAATTCTCACCTGCTTAAGAACAATCACACTCTAACCCTCTTCACACGCAAAAGTGATAGGAATGCTGTCACACCCTGTTCTGAAAGGAGGATGAACAG<br>ACTTTTATGTTGTTGTGTTAATCACTAGATCACCATTCTTTAACTGCCTGTTTCAGAGGTAATGATAAACTTTGACAGTTTTGCTTAAGGAAATGGCTTGCGATTTTGATGCT<br>AATT[A/G]GCTGCTGCCAGGTAACGCTCACACATATGCATAGTTTACTGGCATATACATGTACTGGCACAATGGTTGTGCTGAGTTGGTAATTTCATTGTTGAGCAGAAGTA<br>ATCATAAGATACTGTAATCCCTCCTCTAGACCCCAAAACTTGTGCAGATGTGGAGCTGGACAGCACAGAATGGGAGATCAAGACCATCACCAGTGCAATA
LG18	ref-22975	54.437			CTGTTTCTTTAACAGTTTTCTCATCCAACAGGACTTGCTGTTCCAGCATACCTCTCTCTGCCAGCTGACCCAGACTCCTCAGCAGGGTCGGGATGGACTGACTCAAACCCTG<br>GCAGTGATGCTTCAAGAGCGTGCACACAAAGTCAACGTAGATGGAGGTGGTGGTTTCCAGTCCACTTGTTATATCTGCACCGGTTTTGAGTCGTTCTTTGATAGTTGTGCAG<br>ATGATCCAGCAGACGACAGGGATGGAACAGGCGGTGATGAGGGTTTCAATCGTCTTCACAAGTGTGTAAGCCTTCCTGAAAAGTTCCTCATTTTGAAAAAACTTCTGGAAG<br>TACTCCTCTACCCCCTTTTCTGAAAATCCCATAATCTCAGAGAAACGCTGAGGTCCTTTGAGCAGTTTACTCAGTGTGTCTGTAGCTGTAGATCTA
LG18	ref-63533_10	54.552	Chr21	33547998	ATGCTGCAGTTAAAATCACAGGTTTAAATGCTAATTAGGAAGGTTTGGAGGCCGAGGAGGACTTGATTTTAACCCGACAAGATTGCAAACCAGGTTGGCTCGAATCATTAT<br>TATTTGCCATTATGCTTAATGAAGAGGGCCTTCATTGACAGAGGAAACATTCACCCCCGTGTAGCTC[C/G]GCATAACAATCGGCACAAAAAAAGAACCCGTGCACCAGGA<br>AAAAAGTTTCCCCATTGTCTGTATTAATTTAAGTCTCGTTTAACGACTGTCGGAATCACACGTTCCGCCGAGGAAGTAGGTGATGAAATACGCCGCTCTGCCGACAAACAA<br>ACAAGGAGAAAGGGGGAGAGAAGAAAGGGATGAAAGCGAA
LG18	ref-8854	54.633	Chr21	14228237	GGTGTATATGACTTTCTTCTTTCAGTCAAACACAATCGGAGTTATATTTAAAACCATCCTGGCTCTTCCAAGCTTTATAATGGTAGTGAAGCCCAAAAAGTGAAACAATGG<br>GTTTTTGTAAGAAAAATATCCATATTTAAAACTTTATAATCACTGGCTTCCGGTAGCGGCCGTACGCATTGAGTTCCGGCGGAAGAGTGACCTCTGACCCGACCATGACGC<br>AGGATGTAGGATGTAGGAGTAGCGTAAGCGTAGACGCTTCTTGCGGTTCAAACAAATAGGGCTGGGCAGCAAACTCCAGCTCCTCTTCTCTTATATTGAAATCCTCTGACA<br>TTTCTCTTTAAAAAATTCTCATTTTAGACTTCTAATTCATGACTGGTGTTTTGTTTTGCTCTATCCTCTGTGCTTCCGTGTTCATCAATATGTCATGC
LG18	ref-15965_26	54.971			GTGAAACAAAACCAGTGAAGAAAACACAAGCACGTCGTCCAGAAGCTATAAGCTCTGTTGATTGACAAGTATAGACAGACAGGTAAACTAAAGGGTATCAGGACGTTTT<br>GATAACACTGACTGCATTCCTATTCAATATTTTGGTCTGTGTCTTTGTTGCAAGTTGCATTCTTACTGTCGCACGCTCTTCGTCTT[A/G]TGGACCTCATATGCATTTGCAGGC<br>CACGTGGGACGCCATTTGCATTAGAAAGCGGAATACAATCACTTTAATTTTTAACAAGACATTCGGAATGTGCCACAAAGGCAGATTCTCGGCAACTGACGGAGCCCACG<br>AAAGGAAAGAAAAAAAGAATTATTATGCAGGGAATTAAAG
LG18	ref-34477	55.455			AAGGTCTGTCTTTGTGAAGGATACCCTCTGTCCACTGTCTAATGTAGTTTATGTCTTCACCTCTGCCTCCGCACTGGTTTGAAGGACTTTAGTTTAGGAAAATATCTGGCATA<br>GCATTGCAAATCTCTTATCCTACACTGTTGTGGAGAATATTGGATATACTGCAAAGGATGATGCATGCATCTCAGTCAAATTCTGTATTGTACATACTAAAATAATAGCAAT<br>GGTTCTTCACTGGTTTTGATTCACAACCTTGACATTTTATAACCTTGTCTTGTCATAAAATTAATAAAAATATTATAATAATAATAATAATACATTAATACTTCTAATATAAT<br>AATTCTATTATTAATTTATTTTTTATTATTC
LG18	ref-50017	55.743			TGTTCTCATATCCATATATTTGTTCTAATGTCACAAGTAATAATCTCAAGGTTTACTGGGATTTAAACGATTGTTTTTGCAGTACCTTTCTTCTTCTCACCGTTGCTTACTGC<br>CGATCTAGAGAACAATTTCTCTGAGTCGCCCCCTGTCGTTTCAAAGAACAGATGTCGTTTCATAAGCACCTCGTTCGTTTGGGGAGATGTGCGCGCAGTCAGCGGAGGCTC<br>GCGGTGCAGAGCCAGGTCCAGCTTTAGTTGGCCAACGAACAAAAACTACTCGTGTGTGATGGCATGCACGTGTATTACGTGTTTGGTCTATAGGCATGCATGTTTGGCATG<br>AGTGCTCTTTAAAAGAATGCGTATGTGTGCGTC
LG18	ref-34397	55.795			TATTTTTATTTGCTCAATTGCTCATAATTCCACAAACCTGTTGTAAGAGTCCATAATTGGTACCAAACATTGGTACTGGCATTTGTGTCAGTGACTCATTTCATCTGTTTTCA<br>TTAGTGCTCTGTATTAACCTTATTTTACCCAAGCTTAGCTATGACAGCAAGGAATACGACTGTGTCAAATGCATTATGAGATATCGTAGGCATGGCACGATACATATGCTTT<br>CTTCAAAAATGTCTTCCAGTCAGATGTTACACAAACATCTGATCTGGATGTGGCTTGATGGCCTTTTTAAGGTTTCTGACACAGCCTATGTTTCAATTCCTTGTCAACCAGAT<br>GGTCTATAAATATAATGACCATTTGGCTTCGTTTTTCATGTGTTACAATTAGAACCAGGTTATGTGAATAGCTGAACATTGACGTGTTTAACC
LG18	ref-37113_28	55.954			TACAGTGCACATGACGCGAATTTCATCATCAGAAGAGTGCGCACGTACTGTATGTGCACCGCCAGATTTTTGTCACATACTGTACGTGCACCGCCAGATTTTTGTACTTTGT<br>ACACGCAGATCGCATATGCGCATTTATTTATTTATTTATTTGCAGTAATTAATTTATCCACGAGGTTGCAACTGTTTCGATTCAC[C/T]AGGTAGTCGAAGAAAATCTGCATA<br>AACACAACAGAACGGAACGCATGCAAGCTACAGCGACAATGGAGGCCTATATAGATGACAGATTGTGCAAAGAGATTAGAAAGTAGCCTAATTTGTACAACTCTACCATG<br>AAAGAATACAAATATATTTACATGGGTTGTAACTCATG
LG18	ref-22010	56.141	Chr21	37699041	CAGCTCCAGGAGGACAGACGAAATGACAAGAAACCTCCACCATACAAACATATTAAGGTACATGACCATACAAGACATGCTATGAGATTGATTGCTAGCTTTATATTATG<br>ACGGTACCTGTCTTATCTTGGTCAATCCAATAGGTGAACAAACCAATTGGCAAAGTGCTGATCATCTCGGCAGACCTTTCAGAGATTCCCCGCTGCAACTGCAAAGCATCA<br>GATGAGAACCCCTGCGGTATGGATTCAGAGTGCATCAACCGCATGCTGCTGTATGAATGTCATCCACAGGTTTGTCCCGCAGGCGAGCGCTGCCAGAACCAGTGCTTCACC<br>AAACGGCAGTACTGCCAGGTGGAGATCTTCAGGACAC

LG18	ref-35184	56.439			TAGCAGACCGCAATAGACGCCTGCTTTCAAATGCTCCCGTGTGTTTCTGTGTAAGTGCTAAGTGAAGAGCGGTTTTTGAAGTGTTGATCATTGAAGCCAATCACAGATATATCCGATAAGCCGTGAACACAATGGCCAAGAGGCATTTACGAATCCACTCAACAGCGCTCAACGTGTCACATTTTTAAAACTTCAGTACCAATATGATACCGAAGTCAGTACTTTTGTCAACTCTAGACCTATAAGCTGCGCCCTCTAGAGTTTCATGACTGAACTAGATATTAATGCGGTTTTGATAGTAAATCATTTAATATTTAAAATATATTCTGTAGAAAGTAATATTCTTTGTATAACAAAAAGTACAATAGTACTGCCATTGTATTTTGGACATAGGATCATGATAATGGTTAAACAATGGCATGTAATAATAT
LG18	ref-323	56.508	Chr21	33309187	TTCACCCACAGCACGCAGAGAATTCCTAATAGTACCGCACATTAAGAGAGCGGAACAGACTACTCAGCGAATCAGATAATTGAGTTGAACAGCAGCTAGGGAGGAAAAGAGTGAGAGAGAAGAGAGAGAAGCAGTAGCATCTCCGCTGAACGTTACAGGGGGGAATATATTTCCACCCAGCATCCCAGTCGACGGCTCTTTTCTCTGTCTCCACCCTACTTGGAGAACATTCGAGCACTTGAAGCATGCTAGCACATGTGGCCTGCTGGTGATTGCCCTGGATCGTGAAGAGAGCGTACTCAGATTGTTGAGACGGCTTATTGTTCTGGGACTGCGAAAGGAACATGAGGGCCTGCCAGGAAAACAGGG
LG18	ref-37250	57.039			ACACACATTGCGAAATGCATGCTTTAGTGAA
LG18	ref-51988_10	57.267			AACCAACATGAAGGCTAAGAAAGTTTTTTTTTTTATTATTTTAAGTCCTATAATTTATGTATTTCATTGTGTTTCCACCTGACCTCTAAAAAAGCTTGTTGGCAGGGACAAAATCCCAGTCGCCTCTCTCTCCAGTTCACAGTTGACAGTTTTTCTATTGTCTCTGAGAAACCTTATTGCATGACCACGTCACATTTAGTTTAGCTGAT[C/T]CGAAGGGGCTGCTCTCTCATATCCCCCTTTCATCTTGCTTCCCAGACCCTGACACGTTTATGTCAACATCCATTTCATAGCACCTTATCCATAACATTGAATAAGGCTTTCACACTGGCAGTCTTCACACGCAAACCACGATCATGGTCAAAACCAAGAGGATGCAGTGCAAAGAAATACTTCAGTTATGGATATAGATGAGCAATCAATTTATAGATATAT
LG18	ref-48902	57.471			CAGGCAATGGCGACAATTATGCCCACCTAAA
LG18	ref-52880	58.697	Chr21	23974859	CAGTCAGGTCATTTGAGGGAGTTGATGTACGGCTGCAGAGATACAACAGTGTTGACTAGTGTTTGAAGGAGGCATCTCTCATTCCGCTCTCAATTGTTCTGCCTTAAGGCTGACTGATGCATCACCGTGAAAGTCGAGGCGTCTCCCTAGCAACAGAGAAGTTACAACAGCTCTTGTCAGCAAGCATTTCGATCACTTCTGGCTCCCCAGTGAGGGCCGACGTTGCCTCCGTCTCACCTAGCACAAGGCTTCAGCCAGGCCCCCTGTATAGGGCTAAACCTGGGCACCGAGACACAAAGCCTTCAGAGATTTACACCACCCCCACGTCGCCCCGGAGCATCGGCTGTTGTATGAGATGGATAGATAGAT
LG18	ref-18516_6	58.95	Chr21	22449092	CAAATCTACAGAAAGCAAGAAATAAGCCAAAGGAGTGAGGGATCTTTACAATAACCATCACATTTTTTTCTGGGTACAAAACATGTCACACCACAGGAGATTCGGGGAGTTATCTAGACCTACCGGATCTTTCTCTTGAGCATAGAGCACATCTCCCGTGAGAGAGGCATTGGG[C/G]GGCTGCAGTCCACTCGGGGAGTGGAGGCTATCTGACTCAAAACTCTGTGACTATGTGTTCACAACCTTAAGAGCATGTGACTGACAAAACCTTCTCAATTTAGCATCCGTGAAGGGGCCTTTGTGCTCAGGAATTTGCAGAGGACACAAACAGACCCAAACAAGACCCTACCCTTTCTCAGAATTGAGAATCTTA
LG18	ref-30142_31	59.073			TGTTATAAAGCGACGAGAATGCTTTGTGCG[C/T]G
LG18	ref-48041_6	59.35	Chr21	18367359	CTTTGATAAGTGCCACAAATTCCTCTGCAAGGACACACCAACCGTTAATACAAAGAATAAATCAAAGGGCAGCCCATGAAAGTGTAAGGGCCGGTTCGTTTTAAAAGTCTTGTGCAAAGGTAAAGGGTTATGCAATGCATATTATGCATTTAGCTTATGAATTTCATCTGGATTTGAATCACTTCAGCTACTAGCAGATCCCTCC[A/G]ATTGCGACGCCTATGCTGGAGTGCTTTTGAAAATGAATTTTCCCCCATGGCCCAGTTTACATACTATTATCATATCAGTCCCATCTGCTCAGATGTCCAGATGTTAGCAGTGTGAGACAGACCACAGCCGTGATACTCAAAGGTGAACAACAGCTTAGTAGCAATAATATGAAATGGTGGTCAGTAAACTGACCCGCCACTGAGTTTGACCCATTTTTATGACCTA
LG18	ref-50157_15	59.4	Chr21	26446835	TGACACCTCAGAAATTGACAAAACTTCCTAAATATGATTCTGAAACAAATTAACCTCTACTTTCTCGATAAAGACAAAAATAGGCGTATCGAGAGGGCGGGAATTTGTGACAAGCGTGCCCGCGTCCAATCGCCTTTACTAAATGATGTTTCCTGGCGTTAAATCTTAAAGCCCTCTTGTAAACCAACCTTGACGTCGCGTTCTTTCGCTCGAG[C/T]AGCGTGCTTTGCAGCAAATCAGGTTCCAGTATTTAACTCTCGTGCCCAATAGCCTTCGCATATTTTAAACAGGGGCGGAACAATACACAAATGGAACGAAGTAGGGTAGGTTGTTAGAAAGTTAATGGCTTGGGGGAAAGAGATTCTCTTCTCGCTTCAGGAGAGTTTGAGGATTTTTCTGAAGTAAAACGTAATTATTTTATAACAATATAACCCC
LG18	ref-65202	59.498			GGTTTTGCAACGATCACACTGCTGTGGGAAT
LG18	ref-160_25	60.372			GTGGTCCTTTCGACCTGTGTGCTA[C/T]ATCCCTA
LG18	ref-160_28	60.704			GTGGTCCTTTCGACCTGTGTGCTACAT[C/T]CCTA
LG18	ref-43359	62.704			TGTTCTTCCACGAGGCTTGTGCTGTTTGCTG
LG18	ref-50611	62.95			AATAATTTCTTTCAAAAAAAAAAAACTTACTTAACCTAAACTTCTGAACAGCAGCGTACATACCAGTTATTAAAGTCTATGTGTCTAATCTATAGTATTTGCAGCAAATTCATCTGTCATTTGGTAGAAGCAAAAACAGGCAGATGCCTACATGGACATGTTGCATCACATTCCATTATTCATGAACAACATTACACAAAGTTCACATTTGATTTGCATGCAATTCCGATTAAACATGTAGTTGTTTCCTGCTGGGAATATGACTGTTTACTGATGCTACTTGATAATGTAAAATTTAGGTGTTGAAGCAGAACCAGTTGGCAGTTTCTCTAGTGCTCTAAACATGAACCAGCACAGTGTATGCACATGTATTTAATCAGGTCTTCTTCAAATGAAATTTATGTGCAAAAAAGGACACTGATACAGTGAT

LG18	ref-58268	63.005			GAATAAAATACCTCTTTAGGGCATTAAAATGAATTAAAAAAAATATATCTTTCAGGACTCTTTAAAGTTCTTGTCATATTCTTTTAACTCTCCAATAGTATGACATTATTCC<br>AACTCATACGAATGGGAATTCAGGATTGATTTAGTTTTATTAAGATATTGGATAATATTGAAGTACTACAGTTTTATCGCTGATGTCTGGAGTGTGTGAGTGTGTTCAGGTT<br>TTTAAATAATGCTCACTATATGTAGTATTATGAGGAAATCAAACACATTGTCATGTTTATTTATTGAAGAGAACCTGCACTTGTTCACCGACAGGGGGAGCCCTGTGATTGT<br>GCCTTTTCAGCAGCTTCTAGTGTCGGTTGAGGG
LG18	ref-69325	63.513			GCGCACAGCCCGAAACACGTGCACGCACAGA
LG18	ref-21758_29	64.928			GGCATTGCTACATTGTTTCTTCAGTGTTTGGTTGGTTCTAGGGCATTACTAAATAGTTGCTAGGTAGTTCAGGGAGGCTGCTAAGGCATTGCTATGCAGTTGCTATGGTGTT<br>CTGGTTGTTAAGGCATTGCTACATTGTTTCTTAGGTGTTCTGTTCTAAGGCATTACTATGCAGTTGCTAGGGAATTCAGGGAGGCTGCTAAGGCATTGCGATGCAGTTGCTC<br>GGAT[C/T]GTCAACATGGATGCTAGGGTGTTTTGGATGGTTGATATGGTGTTCTGGTTGCTAGGTCATTGATACATTGTTTCTTACAATAGGTGTTCTGGTTGGTTCTAGGGC<br>ATTACTATGCAGTTGCTAGAGAGTTCAGGGAGGTTGCTAAAGCATTGCTATGCAGTTGCTAGGGTGCTCCACATGGGTGCTAGATTGTTTTGGGTGGT
LG18	ref-21758_8	64.928			TAAGGCA[C/T]TGCGATGCAGTTGCTCGGATTGTC
LG18	ref-51046	66.324			ATCATGAGGTCGAAGGAACTGCCTGAAGAGC
LG18	ref-51525	67.154			CGCTTGCTCACGAGCCGCCTGCAAAGCACCT
LG18	ref-36653_30	67.171			TCATGGTCATCGAATGGTTTGCCATCCTT[C/T]CT
LG18	ref-42272_19	67.249			ACTGACTGACACACTCATAAAGACAGTCTTTGCCGCCATCTAATGGCGTAATAATGTAACTTCTGTTGCTGTTCACGGTCAGGGACTATATTTTCCGGTGGAAGGAAGGCTT<br>TTAGTGAAAGTTTACTTCATGAAAGTTGCATTGATACATATTTTAGGGTTTAATATTTGTATTGTGTGGTAACCGTTTTCTAAAAGCAATAAGGTACTCGAGGCTA[C/T]TGC<br>TGTACGCTTTGCGTCGTGCCTAACAACGCCCTTCAGCTGTGACTTATTCACGATTCAGCACAGCCTCTCGTACCTTATTGCTTGCGTACATATATATATATATATGGACATTT<br>CCTTGAATATTATTAAGACAAATGAAACAATATTTTACATATATAAAACAAAAATATTCTAAAAGGCAAATGGACATGAAAAAAATGGAAAATATTT
LG18	ref-42272_6	67.249			ATAAG[G/T]TACTCGAGGCTATTGCTGTACGCTTT
LG18	ref-26259_1	67.267			TTGAGAATGGTGAGCGGCAAGAATTTCTTAAATTTCCAAATAGTCAAGTCAAGTCAACTTTATTTATATAGCACTTTTTACTGCTCTAGAAGAAAATGGTGTCATTATCCAG<br>CTTAAGTAAGTTCAGTATTGATTCATTCCATTGTAAAAATACCTGCAGTACTCCTGACTTTCTGCCCCAAAGTAAAAGTGAGTACGCC[G/T]GCTTGGAACCGAATAAGGTG<br>CTTAGCATTTTTCTTCATCAAAGTCCATTTTTATAAATCTGAATGTTGTCTGGATTTTAGTGTATGCACACTCTAAGATCAAATCTATGTGTACACATGTTTTATAAATGAGG<br>CCCCAGATATCTGCTGCCACCTATTTCTAAACGCCATTAAAAAAATTACATGCAGGCTAATAACCTACGAAAAACAAACATTGCTGAGTTATTTTGAT
LG18	ref-18895_2	67.737			A[A/G]AGGAATGTCGAAGGCCATGCATTCAAACAC
LG18	ref-70373	68.272	Chr21	6858102	GCCTTTTCTGTTATCCAAGCCTCTTTTGTGTGCGTTCACGCCCTTATTGGTCCGCCTTCCCAACGCAAGTGAATTACTCTGGATGGATTACAACTGCACACGCCGACCGCTCT<br>CCGTGGATGAGAAAGGAGGCGGTGCCACGCGCTCCTATATAAGGAGAGCGCGAAGCGCCCCTGTGGACACGAGACTCGCCACCAGGGATTTTCACTTGACAACGGATCGA<br>GTTAATGGCCCGATATTGTCATAGCCACGCGCCAGGAAGGTTGTAAACGAAACATACCAATGTCACGCCCCATTCCAGGTCAGCGTGAGTGAGTAATGATCACATTTCTTC<br>CGTGCTCTCCCTCTTACTATTCCCATATTAACTGT
LG18	ref-65687	68.747			AGGATTGATACGACGTGTGTGCAAGTGAGCT
LG18	ref-56787_1	69.151			TTCAGAACACAAACGAGATCCCATTAGATCTAGATTCATTTATTAGAATTCAGATTCAATTAAAGTATATTCAAAGGCCTGGCATGAGTCAGATTCAGATTTTTCTATGTAT<br>AGTCATGTTCATGGACATGGTACATACAGTATGCAATTGCTGCTCTGTAAAGATGATGA[C/T]AGTGCCAGGCAATAGTCTCGAAGCAGTGAAAGAAAAATTTATAACTCA<br>CGCAAGCAATAATAAAGTCTGCATTAGCTGTCAATCCTCCCTCTTTACCTCACACATACGTCTCTCTCCTCCTGCCTGAATCTATCCACTTGAAACTGTGCAGCAATCTTGTC<br>GAAAGGACCTCAGTGAAAACACTACGATTTAAGCAACC
LG18	ref-14415_1	69.66			GTACTAAAACAACGCAGACAGCACGTGCAATTAAACGCCACTTTCAGTGGCCGCAGTTCATTCTTAGTGAGTCCTCTATGAGCTGACTGATATGATTAGAGACAAATTGTA<br>TGTGTCTGTTTAAAATTCCTCCTGAATTCTGCAGTGGTTGGACATGATTAGCTGCAGAAACCCTCTCTAATATTCAACAGAGAGATTCA[C/G]AGCTACCGTCGAATGACCT<br>GCAAACTCAAAGCCATGTTTTAATGTGTTCACTGAGGTGATTAAAGTCAGCTTGTCAAATTCCTGGCTGACAGTAAAAGTCCCTCGGGTGGCTTTTAGAAGGAACAGGTAC<br>AGAGTTGTGGTGACGGGGGGCATTCTGTTCCATTTGTCCTAGCTTTGTGGAACCGGAATCAATTTTCTTTGCTCTCAATGAACAAAGTCGTGGCACACTGG
LG18	ref-40514	70.299	Chr21	16516520	GTCTTCACATTTCCCCTCTGTCCTGCCCTTCCTTCACAAATCTCGGCTCTTTCTCACACACTTGTGACCTGTGGGAGAGACAACCCTGGCAAATAAAAAAGAAACGTTCGTC<br>ACACATCGAGACACCGGATGGCTGCAGTATGATAATCTGGCTTTAACAAATGAAAATGACGACACTGAGACAGTGTGGCTCACTGTGGCCGATGCGCCCGAGGTCAGCGC<br>AGTCTTTTATTTTCCAATTAGCGAAAGAGAGGTGAGACGAAGCCTGCCAGGGTCTCTCTCGTCGAGAAAATGTGCGGTCTCCCGCTATAGCTGTTCCTTCCGCTCCCTCATA<br>TAATTCCATTCCGTTCTGCGATTTACATGCAAATGAGAGCAAATTACTATTCATTCAGAAGTGAAATTGGGAATGTGCATGCTAATGCTAAGAGACC

LG18	ref-39384	71.399	Chr21	15744263	ACGCTCAAATTCAGTGCCTTGAATCCCATCATAGCCCTGTGTCACAACAAACCAATCACAATCAGAGGTGGCATTTACGATAGTCTGGTCAGTTTCCCCTACATAGGTATTTTGCTTGCGGATGGCTAAAGTTTTGCCCACTTCCACAGGATCCATTTCATATATTGCTGTGCAGTTAATGCCATATTTTACTGTCAGCCGCAGCCTTTCAGACAATTCTGCTTCCTGTCCTCCTCCTTCATTTAGTCTTAAAATTATGATGATAACACAGCATATAATGAGTGCTAGTGTAAAACTGACAAAACAGATATTTTTCAGTTTGAAGTGAAGCCACAATCTTTTCATTCTGTAGGGAGAAATAAAGGCACATTACAAATTATGCACACTGTAATTTAAACAGAGAAACAAGAATGAGCTTTTGATGTTGCTTT
LG18	ref-28411	71.694			ATCACATAATAATGCACCTTATAGTGTTTTTAAATTCAAGTTCGTTTACTGAGTTATTTTGGATGTATTGGTGTATTCTCAGTTGAATCATGTTTCTGCTACACAAATTATTTGAGTTATTTTTATGAGGAAAATTGAGTGAACAATGTTGAGCTGTTGTGTGTAAATGATGACAACTGTGTTCTTTTGCAGCTTGTGTTTACTGTTCTGCGACATAAGTGCATCACAGTGTGTGTTTTAGTGAGTGAGAATGTGTTTGGAGTTTTGCAAAAATAGTGCATGAGATTGAGCGTACTGCATATTGTCACCTCTGCTGTGTTGTGTTTCTTTGACCTAGTTGCTGTCTGTCAGTCTGTTAATTATGTAATTAGTTTCATGTGTTCACTTACTCCCTCATTAGTTCCTTTGTTTGGTCAGTGTATTTATATTCT
LG18	ref-5400	71.846			TCATTCAGCGCGATTTGCATGCCTCTAAATA
LG18	ref-30067	71.908			AACTTAATCACGAGGACGTTGCACGTGCAAC
LG18	ref-44334	73.58			AACATAAAAAGATTCAACAACTGAGACAAAAACTTAACAATGTTAACAGACATTTGGCAAACAGAAATGGAATAATGAGTCCATGAACAAATGGCAGGTCAATATCAAAAGTAGCAGTCAGTTTGTTGTGTGTCCACCATCTGCTTTAAGCTCTAAAGTGCATCTCATCTTCATGGACTGCAGTAGATTCACCAGTTCTTGCTGTGAGATGTTACTCCACTCTTCCACCAAGGCACTTGCAAGTTCTCGAACATGTCTGGGGGGAACAGAAGGTCACATGTTGTTTGCCACTGTGAGGATGATCAGCTGTCCTTCCTGTCTCCCTGTAGTTCTGTTTTAGGCGTCTTACTATTGGAACTGTGACCATGA
LG18	ref-17817	74.782			CGGAGATGTTTGACACATGCAGATGAGCAGTGATGTTATGCATAATTGTCAGAACATCTCAATTAATCCCTGATGGGCCATTGTGTAATCTGATGGTTGAACCTGTATGTTAAACTGTGATTTACACTGTCTGCATAGCTACCATCATTAGCGTCTTTGTGCGGTTGTGTTGTGGGTTTCATAAACCCTAATGAGGAGAAGTCAGCCAGACGACTACCATACGTTTGTCTCCCAGAGGACTTCAGTGATGTCTCAGACTCGAGCCTCGAGGCGGTCTGGAGTCGAGACCACGTCACAACTCGCATCTCAAATACGTCCTCTAGAGCCAATTAAAAACCAGTTAAACATTAAAAAGATGAGAAGTTTTTAAGTGATGCTGTTAATTTATCTAAATGGTATTTACAAAGTTTAATTGGTTTTAAAATTTGCAT
LG18	ref-10033	76.268			GTAAATGTAACGAAGTAAATGCAGCCTACTT
LG18	ref-52771_17	79.298	Chr21	9964826	TGCTGTGGTGGTCCCAGGGCCGCTGGGGAGGCTCCCTGTCTGGAGTTCCTGGGCTTTCAGTGCTGCTGGGTAACGTACTAGTGCCAGACGACTCATACACAGTCTCGTTCTGTTGGAGCTGCATGGCCTCACGGGTGGCCAGGAAACACTGCAGCACCTCTGCGGGGAGGAGGAAACATGCAATTG[A/G]TTCGCAATTACACAACACCAGAGTAAGTATTTTAGAGCAAATAATCCCACGTATTAATCGCAACCATGACACATCTACACCATACAAAATTCATTTTCAAAGCTATTGGAGTGTTTTACAAGAAAATACAGTATCATTCTTTCTACTTCAAAGTTTCACATTTAATTCAATGTGTTACTTACT
LG18	ref-44837	82.478			GGAAGGGAGGGTCTGATTTACGAGGAGCCTCAGTTCAAAATTACGTCTGGTTGGGCCAGTAAGGTGCAACCATCAAGACCTGCTCCTTGTCCTCCCTTACCTTGCACAGTGTCTGTGTGAGCAGGCTCACTGGTGGTAATGCATACTTGTGTAGGCCCTGGGGCCAGCTGTGTGCCAACGCATCCATGCCGAGGGTCCCCTAGGTCAGGGAATAGAACAACTGGCAGTTGAGGACTCCTGGGAGGCAACCGAATTGACTCCAGATCAGCTGGATCACGTGGGGGTGGAGTCTCCATTCTCCCAGAATGTGAGAGAACGTCAGCCGCATGATTGAGCTTGCCCGGGATGTGAACAGCGACTTGAGCCGCT
LG18	ref-41809_25	84.385	Chr21	3500999	GGGTGGTGGTTCTGGCTTTGGCTGGCGTGTCCTTCTCAGCGTAATACATCTGCAGATCCCAGTTAATGAACTTACTCTGACCCAGACGGATCACCTCCACGCTGAAGGACAAGGAGAGAAAAACGAATGAATGATATGACAATCAGTGCGCTCAGCGACTGTTGTGTGTGGATGTTCGAGCATTGATATCGTAC[A/G]TCACATAAAGAGAGATGGGCACCATGGTGTTGAGGATGATGATGTAGCCCCAGAAACTGAGAAACCCTCGGTAAGATGACGATTGGTCCAGGCCGTCGAAAAGATACCAGGCCTTGGAGCCGATGCTCTCGTACCAGTACGTGTGTCCGATGGCCAGACCAGCACACAACAAAAC
LG18	ref-48578_29	85.467			TCTGGAACCTTCATTTTTAAGAGTTTAATTTAATTAACCTAGTTTGATCAGGTCACTTGATACAGTATCTGTGTCAGATGAGCCTCATGCTGTGACATGTTTATGCAGCTTGTTATGGAATGCTATTATGACTACAAAATAAGTATTAAATTAAATAAAATCATCACCTGATGTTGTTTGCAGCTGTGTCGTCCCAG[C/T]TCCCTTGGTAGGGTTCCACTCTCAGCTGAAAAGATGTCAGGAATCCATTCGAGCACCATTTTATATCTGTCCATTTACCCCAACTGTGAAATGAATGTGATAAGGAACATTAGTGGTTTAACCAGTTACCTGTTAGTTTGAAGAACACGTCACAATATTCCTCATTTAAATA
LG18	ref-38695	86.054			ACTAGATTAGCGACTCCCTTGCTCTTTTTGA
LG18	ref-12146	87.73			ATAATCTTGAATCAAAGGCTTGCTCGAGATGCATCGGCGCTGCACAGACCGATTGGCGTATGACATCAAAGTACCGCGAGAGCAATTTAAAAGCATAAGGAGTGGTATGCTCTCACGGTACTTTGATGTCATCTCAAACAAGCCTAAAATAAGGCTTGTTCGAGATGCATCAGCACTAAGCAGACCAATCGTTGTATGACCGATCAAAAGCACAAGGAATCGTATGCTCTCCAAACGTGGTAGATTTATGTCATACGCCGATCTGTCTGCACAGCGCCGATGCATCTCGCACAAACCTAATCACTTGCAGCGCCATCTCTTCTCTGATGACGTGTTTACTGGGGCAAGGGCGGGGCAACCTGTCACTCA
LG18	ref-43859	89.329			TTTGACAGAACGACAGGTTTGCTGTGTTTAG

LG18	ref-18182	91.734	Chr8	16032121	AAACTGCATGGTTCAAGTGACCCAATTCCGTTTTTTTCCTCCCATGTGGCACAGATCGGATATGAGCCACGACCGTGTAAGCAGGAAAAAAGCACGAGGATACCCATATTTACCGGTTTCAGGCCTCTTTCATACGTGGAAATATATCAGATGAATCGGATACATGCATTTGCACCTGTCGTGTAAGCTGACAGATCCAACGCTATCTCGCGACCAATTTGCATGTATGTTACGAGGTGGCTAATCCGTACAACCTCACTCGTAGGAATTCATTCAAATTTGGCAATTCATAAAATATGTATATAAGTATAAAATATATAAGTGCAAATTCGTACGAATTAGCCACCTCGTAAAATACGTACAAATTGCCGTGAGATTGAGTTGGACAGATTCGATATTCCCTGTAAATGCGACTCCTACGTCATTGAAAA
LG18	ref-18461_26	94.99			ACAGACACACACACGCCTGACAGAACACAGCGTCATGGTGCAAGTGTTTGTCAGGGAACTGGCTGCTATGCAGACAGCAGAAGAATAAAAAACCAGAACAGAAAGTCCACTGCCTCCGTTTAATGATGGGAAAGGAGTCTGTGTGTCTGTGGTGGAGGGAGTTCATCCAGAAACACACTCTACACACACAAACCTCAGAGCTCAAAGGGGCGATTTGATTGCATTT[C/T]GTCAGATGTGTTATTACTCTGGTGTCAACATGCTCAGCAAAATTCTCTTCAAACTGATCATACAAGAAGAAAGTTTGTGCTAATGTCCACTATAGTGCCTCTTTTGGCAATGCTGAGAATGCGCAAGTAATCATGTTCTGACAATCTTCTGAAGCAATGACGTGTTAAATCAAGCATTTACATACTTGCATACTTAAGTATTGCT
LG18	ref-29906	95.178	Chr21	4351667	TTCTGTGTCTGTTCTGTAAACAGCAACCAGGGATTGGTGGAGAAGGTAAGACTCGCCTACATCTTTAACCAATGAGAAAGCGATACAAAAACACTTTGTTGACCTTTGACCCTTAACTTCAGTGACACCTCACCAGGACGCCACCTTCCTTTACACGCAGCCTCTGGGAAGGGTGATGGGCGTATGGATCGCTCTGGAAGACGCTACAATCGAGAATGGCTGCCTGTGGTTCATGCCGGGATCACACAACGGTAATTTCAAACGCATTAAGCAGATTCATGGCATTTTACATAATTTGGGCATGCATGCTATTGGAGTGAAGCACAAATTTGTGTATTTAAAATGAGTTGGTGTGGCTCTTTTCATGTG
LG18	ref-54671	96.862			TCAATATGCCAGATAATTAAGAAAAAGAAAAATCATGCTGCAAAGTTTGAATTTCTGCACTTTTATTGATAAATGTCAATTTTAAAGAAAGTTCAGAATTGTTTTCGTCCCCATTCAGTTGCGATATGCATATGCCATGCAGAAACATTTTGGAGAATAATACACTGTACTGTAGAGCTGCACGATAATCGTTAAACGATACTGATCGTGATTCGAACATCCACGTGATCTTATTGTGTCTATTAAAGTTTGACATTCCAGACAGAGCAGGGTATCGATAACATGGAAAAGTGTAGTGCTGCCTTAGTTTTTGATGAATCAGTTTCACTCCTTATTTACTTCAGTGTTCTTTCAACATAACCTTTATGG
LG18	ref-51492_16	98.042	Chr21	399533	CAGCAGTCCTGGGGTTGGGGGTGCCTGCTTTATGTACTTTGCTGTTACCTTAATCTCTGGATGTCGCTTTTGGTAGTGGACCAGAACGCCCACAACAGAGCGGTTGTTGTAGACACAGTGTTTGCAGTAATAAACATCTTCATCCAGAGCCACAGGTAGGATGGCGTTCGAAGACTTGGGGGTGCTGGTTAGGCTGTAGGTTGAATTGCTCGACA[A/G]CTGTGCTCGGTCAACAGATATGACCCGCATTGTTTTCTGAATGCGGAAGTACGAGGCCTTTTGCTCTGGGTGTTTTTTCTGATAATGGACCAGTACTGAGTGCATGTTGGGACTTGAGAAAGAACAGACATCACAGTCATACACCACCACATTACCACCCACACCTTCTCGAAAAGCTTCAGCCTCAGTGTTTGCTTCTGGCGTTTTGCAGATGCT
LG18	ref-7302	98.815			TGTGTGTGTCCGAAGTGTGTGCATGCATGTG
LG18	ref-10469	99.074			AAAGCAATGACGAACTGGCTGCCATGTCAAA
LG18	ref-64632	99.449	Chr21	594742	ATGATTTTATTTCTGCAGTGATGATAGTAACTGGTGTGTTTTCGTCACATTCAGTGTCCATATGGTGTAACGGATCCATAATGCTCACAATGACACTGTATTCTGTGTCTCGGACACCTGAAACCCTGGATTCACCTGACAGCTTCAGACAACATGATTCACCAAACAAAAGACTTTAAACTCTCACGGATCAGAAACTCTCCCAGCGAACGAAAGCCATGGCGGTCTGAGAGTGTGTGTTCAGAGACAGGCCTCGACTCGACGGACAGATGAAGGAGTGTTTTCACACATGTCTTCCTGCTTTTATTGTGCAGTTTCCTTTCCTTCGCTGCCAAAACACTAAACGGAACATTCTCTCAAAGTTACGAACAAGCGTTCGTTCAAAGTTATCTGGTTTTCAATAATGTTCTGAAAACGTTAGCGCAAAAACA
LG19	ref-38727_23	0			CCATAATACCCACCGGCTGCAACTCTTTGCCATCTGTAACCATGGTGACCTGCTGCCCAGTGCGGCCCTGGTGGGCGGGGACAGCGATGGTGGTTCCGTCCTGAGTTACCATGGTGACGGCCGTGCCGAGGACCTGCAGGTGTGAGTTGATGATGTCGTCCGTTTCCATGGTAACTTCAGCATCACGTTCG[C/T]TCTGCTCAGTGCCCTGCGGCGACGCCTGAAAAACCTCTGCGTCTACATACAAAACCATTCTTTGCAAACTGTGCAAAAATCTGAAGTGAAATAATGCGTACGGTAGACAGAAAGTCGTTCTCACCGTCCTCGGCGGTGTCGAAGTCTCCGTGCGCGGTGCGCTTGTGCATGGCCAGCG
LG19	ref-3584	1.429	Chr10	10709633	TCAGGGAGATCAGAGATAGTGATATGTTAAAGTTGTGGATTCTTATTTTTGGCCTCTTTCTCTTTATTTTTAAAAAAATCCAAAAATGGTGTTTTAAGTTCGAATTTCTTGAACTTTTACATTACAAGAAAGATTTCTGTGTGGTCGGTTGTGTTTATCTGTGTTGTGAGTGTGTGTTTGCACACATGTTGTAATTAAAGCTACATGTGATGAATATGTTGTGATGTGGCAACATATTCTTCATACCGTGATCTAATTTATCTGTCCCCATGGGAGGTTTGTGAGGCAGACTGCACCACACTAATGGATGTTTGCCTTCATTGAATTACAGTGACGTGTGCACATGAGCGCTGTGCGTAAATGCTGTTT
LG19	ref-20096	2.617			TGCTAAACTACAACTGGCTGCAAGTTTTGCAGGGGCAACCACTTGAAACACCCTAACAACCACTTGAACACCCTAGTAACCACCTAGCAACCACCCAAAACACCCAAAACACCCCAACAACCACCGAACAACCATTTGAACACCCCAGTAACTACCTAGCAACCACCCAAAACAAACTAACAACCGCTCAGATAGGAAGAGAAACCAAAACAACACTCTTGCAACTGCAAAACTACAACTGGCTGCAAGTTTTGCAGGGGCAACCACTTAAAACACACTAACAACCACCTAGCAACCACTTGAACACCATAGTAACCACCTAGCAACCACCTAAAACACCCTAACAACCACTTGAACACCCTGGTAACCACCTAGCAACCACTTGAACACCCCAACAACCACCTAGCAACCACTTGAACACCATAGTAACC
LG19	ref-45544	2.98			GGCGAGCAGCCGAACGGGCTGCGATGTGGGC

LG19	ref-27089	3.747			TATACCAAAACTATGTTACAGTACAGTAGGGCTATTGCTAACAAAAACAAACAAGTCCAGAGAAATTTCATGTCGGCCATTATTTGAGCAGACTTTGCTTTTGTGGTGAGCTTGAGTGGTACACGAGTGTGGGCCGTGAGCGACTGATCAGAGAGCGCACACACACAGCAAAACAATGAGCAGTCACATCGAAGTTCTGGAGATGTTCTGACCCAGTCGTCTAGCCATCACAATTTGGTCCTTGTCAAACTCACTCAAATCCTTACGCTCGCCCATTTTTCCTGCTTCTAACACATCAACTTTGAGGACAAAATGTTCACTTGCTGCCTGATATATCCACCCACTAACAGGTGCCGTGATGAAGAGATA
LG19	ref-2335	4.388			GAGGGAAAAACGACTGACGTGCAAGTTATGA
LG19	ref-2813	4.851			AAGTTTGATGCGAGCTATTTGCTAACTGGTA
LG19	ref-31308	4.905			AGTATGTGAGCGATCAAACTGCAAAGGTAGC
LG19	ref-6968	5.011	Chr10	33470557	AGAAAACAAGGGAAGAGGACAAGAGATGGTCATTTCATGGCTGTTTGGGCACAAGTAATTGAATTTGAATTTAGTTGATTTGGTATTAAAGGGATAGTTCACCCAAAAATGAAACCTGTGTCATCATTTACTCACCCTAATGTTGTTTCAAATGAATTTTTACAGTTTTTGGGTGAACTATCCTTTTAAGAGTGATTCTCACCCCATTCCCGATGTATGTGCTCTGAACAAACCTCATTAAGCTCATTTGTGTTAGCCAGCATTTCCTGAAGGGCCCGCACAGATAACGACTGCTCCAGGATAAAGCAGCATATCGCCAGATCAGGAGGAACATTCTAGAATGAAAGGGAAAAAAAAAAAAGTGTACAGAACATTGGGAATCCAAAACATCACACAGTATTTCATACATTCAGAACGCCATGTAAGAAAAG
LG19	ref-34577	5.157			TTTTATAGGCTACTGGGGGTCCCTGTGTAGGCCCAGGCAGAGAGTTTGGGTGGGGGTGTCCCTGCTGGTTCAGAGCTTTACAGCAGCGGCTCCTCTAAAATTCGCTCGTCTAACCGCATCAGGTCAGTCCGTGTTTTACACGTTGCCTGAGAACAACAACAACGCCGTTTTAATACTCTGCACGACTCTCACTCATTTCTCTCTAACGTCTACAACAGGAAGTACAGACACAAAAAAGTCGTCCAAAACTACAGAAGATTAAAACATGCAATGAAAACAGTCTTTTATAATCGACAGATAGCGTTCAGCTGCGGGGAAGAACCGGTGAGAGTATCCAGTCAGGCATCACCGGACTCTCTTCAGTGGAAG
LG19	ref-72566	5.601			TGTGAATTTGTGTGAACGGATTTAAAAACAGAGGGTACGGTTTGCAAAATCTGAGCGAACGGGTTTGAAATTCATATGTACGGTTTATTAATCTGTGGGCACGATTTGTTAAACCGAGGGAACAGTTTAAGAATTTGTGAGCACGGTTTATAAACGGAGCGAACGGATTGCAAAACCGTCGGCACGGATTGTTTTTTTTTTCTCCTATAGGTGACGTGCCGGGCTCCGTACTACCTAATGACTCATTTGAACAGATTTATTTTAATGAATCATGAAAACAACTGATCTGTCCACACGGTCTATAACGAATCATCACAACATTTATTTCCATCAAGAGGTGCATCATTTTTTGGTCAAGATCTTGTATTGACAATGCAAGA
LG19	ref-48577	6.295			TGAAAGGAGCATAATAGGTCCTCTTTAAATTCATGTTCCTTGTAATCTTGAATCAGCATCTCTTCTCTGATGATGAGGGCGAGGCAACCTGTCACTCACATGAGATTCACCAATAGCAAGCCACAACCATCCAATCAATTCCCCACAGACAAAATCAAGCCCCGCCCTACATTTGTTCTTGTTTGAGAAGCTGTTTCACTCGGATATATACGAAAAGACTGGCGCACCATTCGACTTTAATACATTTCACATAAATCCAGCTTCTGATCAGATCGTACGTTTTTAGGAAAGCTCCTCTTTGAACGGATAGATATTGAGTAACATCTCTATTGGGTGGTCTGCAAGTGATTCCGACTGATTTTACCCACCAATGCAAACATTTTTCGAAAGACTTGCGCGCTTCAAAATAACAGCGAGCACCGAAGCAGGAA
LG19	ref-7032	6.407			GAAGGCTCACTGGGGGAAACGCATATTGGTCGCATATTTGATTGCCAGGGCATCCGTGCCGAGCGTGCCGTCGGTCAGGGAATAGAACAACTGGCAGTGGGCAGTCTCCGGGGAGGCGAACAGATCTACCTGTGCCTCGCCGAAGCGCTGCCAGATCAGCTGGACCACCTGGGGATGGAGTCTCCATTCGCCCGGAAGGCGGGGAAAAAG
LG19	ref-23251	6.535			TCTCGTCTGAGCGCACGGGCTGTGTGTTCATTCAGTTGTCAGTTCTCTTTTACGCTTACATTGTTTTTCTGTTCAGTTTATCCTGTAATCCTGAGTTTCTTTATTAAATATCTGTTACTGCTGCATTTAGATCCTGCTTCCTCTTTGTTTGCAACAGCGTGTTACGTTTCCATGTCCTTTTCGTTTTTTTTTTTCATGTCCCCTCCACCTCGAAGTATTTGCTAACATGTGCATATAATATCCAGATTTACCAAGTTAATTTGCATCTGGATTGGATGAACACTTCCATACACATTTTATGACCCTGTCAGACTCATTTCCACAGATTGGTTTGAGTCGCAGTGTGTTATTATATCATCTTATCACAAATGAGACACACAGATAGAAATGTTGTTCATTGTGTTGTTTACTGTAACAATTGTCTATGCACA
LG19	ref-39315	7.571			AATGTACCTGTAGTGTGCAGTGCACTAGTATTGCATTCTGAAAGCTCAAATGACCTTTTGACCCCAACAATTTGACCTCTTATAAACAGCCCAATCCACTAAACTCAGGTAAGACCTGTAAGAGCAGAGGATCATGGGACTGGAAGCAGGTGCTTGAAGAGGTTACTGAAGTGCCCATGGCAACAGCATCGCTGATGTCACTTCCTTACCGGAGTGAGGTTAATGAGTTCATTAGAGAGAGTAACTCTGCTAGTTAGACCCGTCGACTAATCAAATAACGGTTGTATGACTAAACAATGAGCAGTTTACATCAGAACACACATCATCGTAACACACATAAACCAGCAGCATGAAGACAACTAACCAACC
LG19	ref-46248	7.947			TCCTAAAACACGAATGCAATGCGCAGTGACG
LG19	ref-69171	7.964			TGTCACAAACCGAGGGTGCTGCTCATATGTC
LG19	ref-31848_1	8.624			TAAACAGAAAAGCACATGTATGCCCAACAATATTTCAGACCCCAGGAAGAGAAACTGGATTACTTATGTCTACAGAAATCAACATGATGTGAAAATTCAAGCTATGAATTTTGTGAAAGTCGAATCCTGTTTAGCCGAATTGGGTTAATTATGTGGTATACTGTTGGCTAAACAGTCTTTATCTATGTAAACAATATCAG[A/T]GAGATGCCACGACATGTTTGCTTTACATTCCCTATCATGGCTTGAATTTGCATGGGGCCTAAGTCCGAACCTTGAGCCACACCTCTCCTATATTTACAATGCCAATATTGTTGATTTTCAAACTGCCGTTGCTCTTATTACACATTCAATACAACCAAATATTTCAGCTTTTAAAGTTTTTATGTCATAATAATGTAAGCATTTCTTTGCGAAATGGACAAGAACAATGTA

LG19	ref-28710_18	9.402	Chr22	6914088	CTGTATGGAGTTTATTTTCCCCCTGAAGATCATCCAGCAGCTGTTGGTTTGTGTTTCAGATGTCTCTCCTGCTGATCGAGATGAAATGAAGAGAAAGTCAGTGAAGGAGGG<br>AGAATCTGTCACATTGGATCCTGGTGAGATAAACGGCTCGAATGATGTAATGACATGGTGTTTTGAAGGCAGGCC[C/T]ATCGCTCAGATCAGAAATCAGAGTAAGATCTT<br>TACAGTAAAACAGGGTGTTGATGATGATGCGAAATCAGTCAAATACAGCCTGAACATGAATCAGACCGGATCTCTGACCATCACAAACACCACAATCACAGACTCTGGAC<br>TCTATCAACTACAGATCATCAGCAGCATCAGATTCAGCATC
LG19	ref-13489	10.546			TATGCGATACCGAGGGTGGTGCACTTGGACA
LG19	ref-54318	10.926	Chr21	5275106	TCCCCAAAGCACTGGCCGAGATGAAGCTGTTCTCGGCGGGTACACGAGCACTGAACGGCCGCTGACGGCCTGGAGCTCCCATCTCCGAAAACGTCTAAACAAATTTTAAA<br>ATAGGCACGATGATTAAATATGAGTCACATATTTCAGGTCTAAACTACATTCTTCCCTCCACAGTTCATGGTACAACAAGTGTAGTATTTGTAAAAACTACGATGGATTTGC<br>TCAACCGTCATGACAGTCGCTTCAAGCAATTTCAGATTTGAGAACCAGAAAGAACTGTTGTATAAAATGTAATATTATATGCAATATTTATATAATATACAAATAACATAT<br>ATGTACAGAGCATATTTATTTATAGATAGGTACTTTTATTAGTACATTATATTGGCCACAGCTAGTAAAGTATACAGCATTAAAGCTCTTTCTTCGAT
LG19	ref-2458	11.619	Chr10	39221682	GTTTTGCTGAATATATAGAGCTTCAGTTTTCTCTTACAGCCAGTTTTAGCAACACATCCATATCTCCCTCTGGGACACACGCACAATAAACAAACTGAGACAAGTGCAAGG<br>TACCAAGCAGTGCTGCCTTTTTATTGGTTGTTTAAATGTTTGTCTAAACAATAAGGAGTGGATCTGGGCCTTCTTGCATAAGAAATGGGGACAGGCAAGCGATTATGATGC<br>TCATAAAAGGATCTTCAGAAAGATTAAACCATTGATCTTAGAAAACGTAAAGGAGTTTTGGTTATTTGACAGCATTTTGGAGAACATTATGAATGAATCTCATGAAATCAT<br>GTCCATAATGAAAAGATTTGTTTGCATTATGCCATTAATTTCATGATAAATAAGCACATATATACATTTATATATAATATGAATATATACAAATATTT
LG19	ref-17558_2	12.726			C[A/G]CACTCAGACGACACTGATGCCCATTCCAGC
LG19	ref-4297	13.922	Chr10	21851516	GCAGGAATCTTTTGTGTTATGTCTTTCAGATGTTAATGATAACCCCCCAGTCTTTTCACAGCCTTCTTACTCTGTAGACATAGCCGAAAACAATGCCCCAAATGCTCCCCTTC<br>TGTCCGTGTCTGCATCAGACCCAGATGTGGGTGAGAATTCTACAGTTTTTTTCTCAATTCTGGAGAGCGAGGCTCTCGGTATGTCTGCGTCCTCATATGTATATATAAACCC<br>AGATAGTGGGCAGATATATGCCATGAGAAAGTTTGACTATGAGCAATTGAATGCGTTTCAATTCGTAGTGCAAGTTCTTGATAGAGGAACTCCAGCTCAAAGCTCCAACAC<br>CACAGTGCATGTGTTTATTAAAGATCTGAACGA
LG19	ref-48858	14.055			CTAACAAAGACGAGGTACTTGCCGAGACAGG
LG19	ref-26915_14	14.175			TCTTAAAATCAAAGTGGGTAGTGTGATTTGAGGACTCCTACAGTATGTAGGCCCCTTAATTTCTTGAGTATTGAATCTGCTCCAAGGGCTAAAGGTAAAGTGTTTTTGTTGT<br>TTTGCATAGGGCTGGAAATGTATTCATGTTTAGAATACATTAACACACCTAACCTAAGTTGTTTATTTGCACCATAGCCATGAACTGAGCCTTTCGTCCGAA[A/G]GCTGTG<br>CGTCTGTCTTACACTGTTTCACTTTTATTTTAAAATGACCTGTGATTGTTTTTGTATGGATATTTTTTTTTTTGTTTGGTTTTTGGTTATTGGGATTTTACTGTGCTCTGATTTC<br>GATAATACTAACATACCGCAGATGTTATATAACTTGTAACAATGACAGAAGAGGGATATATTCTTATGATATATGAACTACCATCTTCGATACTGT
LG19	ref-11407	14.548	Chr10	23985206	GTGGTTTGAATGTCTTTTGTGTGTGTCTGAGCTCAGCAGCTCAAAGGGACTGAAAGGACTGCATTTGTGTGCGTGACATTTTGTGCCTCTCCCATGTGTGCATATTAATGAC<br>ACGTGGTATTTCTGAAGCCCAATAAAATATGCCCCTTGATGGCGTTGTGTGGATGGTGTGGGACATGGCGGGGGGTTCGGAGGGAGAAAGGGTTTCAAAGGCGACCTGCC<br>TCTTGTTTGGTCTGGGAGAACAAGCACAGTGTGATGGGGTGTGTGCCTTGAATAGCAGCAGACATCAGTGTGGATTAAACTCACTGGACATACACAGCCTGTGAGAGTTTC<br>AACAGTTTTTTTTTATTCTGAACAATAGTTTGAAAC
LG19	ref-36462	16			AACTACATCACGAAATGTATGCGTGCACTCA
LG19	ref-49686_18	16.292	Chr10	33757609	GGTCAAACTAGATAGATTTCTGCACAGATAATACAGTTTCACTCACAAACTGAACTGAACTGATATCGGTTCACATATTGCGTCTTAAAACGCGTGGAAAGCGCGGCAGCG<br>CCGCTTTCTTCTCCTTTCCACAAGCCCTTGCGCTCCAGTGGCGTCTGTCGTTGCTATGCAACCATGAACCGCGCTCTCCACGATGACGCATGCGCGTTGCGACCGC[A/G]TTG<br>CTTTTATGAGCATCTACATTTGAAATAACGAACTTGCGTGCGCAAAAGATGCGATATGTGAACGGCCCCTTATTTTAGAGTCAAATTTTTTTTAATTAAATCATTTATTTTAT<br>ATACTTCGTTCACAAAGATTTAATATCAAAAGCAATTTTATGATAGTATTAGACATTTTTAACATTAAACTTTTTTTTTACACACTCTTAAAACAGA
LG19	ref-7916	16.481			CCTTCTGGGACGACAGCCATGCACCGTAAAA
LG19	ref-9879	18.596			AGGAGAGTCGCGACCACAATGCAAGCATATA
LG19	ref-7194	22.713			GTGATGTGAGCGATGGGATTGCCATTAAAAT
LG19	ref-67130	25.822			TCTGAACACACGAAAATGCTGCAAGTCAGAA
LG19	ref-70741	27.06			GAGAAAATAACGAAAGTTTTGCCTGTGTTCA
LG19	ref-54062_30	27.714			ATCTGAAATCCGAGTTGTATGCATACCGA[C/T]GG
LG19	ref-54062_27	27.759			ATCTGAAATCCGAGTTGTATGCATAC[C/T]GATGG

LG19	ref-66662_19	28.686			GTGAGTCTCATTGATCTCTAGAAATTCAGAATTCTGTTTTATGTGCAATTTACAGTGTTCATTGTAGATCAGACGGACAAACTGATTAACACTGAAGGCTCGTTCACAGCGA<br>GAACAATGCAGTGATAACTATATTATTGTCCACACCAGCGGACGATATCGTTCTGTTTATTCTAAGCGCATTGCAG[A/G]CGTGCGTCATTATTGGCCGGCTCCTGCGTCAG<br>CATCACACGTGAACAGCGTCGGCCAATCCCGAGCCGGCATTCATATGCAAACAGAAGCTCTGCACTGTGTTTACAACAACAGCTGAATAGGAGAATCACAGGGAAGAGAA<br>GAAACTGTTCAATAAAGTCGTTATTTTGTTTTGTTTTTG
LG19	ref-46383	29.123	Chr11	21112599	TTATATATATTGTTTATATTCTGTGTATATTATGCTGTACTGTATTGTGCTATATATTATATGACTCCATACATTGTTTTGGTGCAACATTTTATAATGTTTTGTATTGCATAA<br>TAAAGTTTGAGGTCCTACTAGATATTTCTGAAGAAAGTGTGCTTAGTCATACAAATTTGCCACGGTAATCTGTTCTGAGTGGTTACCAAGGCGTTGCTATGTGGTTGCTAAG<br>GTGTTCTGAGTGAATTTAAATGTTTACTTTGAAGGCCTGTTCACATCAAGGACGTTCTAAATATGAAATATAATATAAAATATAATATAAAAGTTCTAAATATGAAAGAAT<br>TGCAGAGTACACATCACAACTATAACGATAACGATATAGAGAAATGATATCGTTGGAATCACTTACAGAACGATTTTTTTTCCAGCTGATGAAC
LG19	ref-4678_28	29.79			CTTTGCGATCCGATGGGAATGCTGATT[A/C]CTGT
LG19	ref-67744	31.469			GGTACTGGCTCGATGGCTTTGCTGAAAGTGG
LG19	ref-61237	32.548			AAGAATGTCACGATAGGGTTGCAAATACGGA
LG19	ref-37834	33.531			CAATCCTGCACGAGTTTCTTGCAGATTGACA
LG19	ref-37958	33.565			GGGGGGGACTCGATGTCACTGCCATTACACA
LG19	ref-9447	33.653			TGCCGCCGCCCGAGCCCGCTGCTCCTGCCGC
LG19	ref-15551	33.659			TCGCTCTGGTCGACGTGTATGCGCGACTTAC
LG19	ref-63321	34.575			CACAGAAAAACGACGCTAATGCAGTTGAAAC
LG19	ref-57634_23	35.996			ATGACTGAGACGAGAGGCGTGC[A/G]GATCCATCT
LG19	ref-40678_16	36.152			AGCACTTATCAATATAAAGCATCTTGAGGAAATGACATCAAACAACATCTTTTATATTTCGCTAAAATGTTAAGATTAGTTATCCAGAACTTTAATTTAAGTTTAACCACTT<br>TTTTTTTTTTTTACAAAATATTTATCTCCAGCACATGTTCATGAAACAACGTCTGCCTCTTGAGTCAGCACA[C/T]GTGTCGTGATGCTCTCGTGAATGTGCATTGGAGATTA<br>ATATTTTGTAAATAAAGTGGTAAATTATGTTGTTGTTGTTGTTGTTTTTAGCACAAAGCACTTACATCGCTTCAAAAAATTGCTTCATAAAATTGATTAAACCACTGGAGTC<br>GCATGGATTATTTTAAAGGGATCCTTGATTATGAAT
LG19	ref-675	36.675			CCAAATCATTGCTGGTCTAGAATAGCTTGTGTCAGGGGATGGAGGCGGGGTTACCATGACCAACAAGATGAACAGAAACTTGTGACCGCCATTTTTGAAATTGCAGCTAAT<br>CAAGCTAATAGCAGCTCGATTGTACTCTCCATCTATACAAATTAGGCATTGGATTCGCCATGTTTGGAATGCGATGTAGGTTCGCAAAGTCATGAGCATCAATAGCAATGC<br>AACGTTTGCCATTGTTGATGGAAGAGTGAAGTTTGGAAGTTGGAGTGGTATAAGTCCTGGGCAGACCGATCGGCGTGTGGCATCGGGGTGCCGCAAGAGCATTTGGAGAG<br>CATACATCTCCTTGTGCTTTTGGATCGCTCTTGCGGTGCTTTGGTGTTGTGCGCCGATTGGTCTGTGCGGTGCGAATAGCACTGGCTCTGAACTAGCAC
LG19	ref-659	36.976			GCAGATAACACTCACAGATGGGTAAACAAGGCAGGCAGCAGAGAATCGTAAACGGGTAAGCAGCAGGTTCAGTAACAAACAATCAACAGGGGAAAACGCTCAGAAATG<br>TACACCGTGACAATACAATACTTCGCGAAGCGTGAAGGTGACTGAGAGTCTTTTATAGTCCGGGTTATGGGAAACAGCTGGCGAGATAATCAGTCCAAGGCACGGGATTA<br>GTGCGGGTGCGTATGTGTGTATGTTTGTATTCGGGCGAGGGCTCCCTCCGATGGTCAGAGGAGGGTATCACGGAGCTCGCCCTTGTGACAATATTCAATGTGCCCCGTTATT<br>AGAACACTGTTCTATGATGTTAGCGATGTTTTTCACATTAAACTTTCATGAATTCTACTGAGAAACCACAAGAAATAGGCAAATAAAGACTGGGAGTCAAT
LG19	ref-41731_8	37.571	Chr10	2156335	TGTAACCCAAGGCTGTTTGGCCATGATAGTTAACCTGACAGACGGTTCTCCTTAGAAGAAAACAGCGCTGGCCTTTGACACTCATGTTCCCGCTCTCTGCCGCTCGACCTCC<br>TCGGTATTGTTTATTAGCCTTTATACCTGTGAGGACATGTGATATGTCTATTTAATAGTCCAGTAGTTGCAGTGAGAGAAGTCTGTCAGCGGTGC[A/G]CTCGACTGTGATGC<br>TCATGTGAGCATGACATCAGACACCTGAATGGAGCCAGTAAAAACAGGGTTTATGTTTCCCAGCTGCTGTTCAACAATTAGCCTCCGATTCTTTGATTATATTTTCGTTGTT<br>GGAAGCTGAAGACATAAAGACCCATTTGGGTCATATTTCACCTCAAAATTGAGAAAATTTTATTTACCTTCAAGTTCTCATTATTGTAATGTGAGTAA
LG19	ref-29024	37.997			ATTGCTGTTGCGACGCGTTTGCATCTAGCTA
LG19	ref-19895	38.371	Chr10	2940488	ACTAGAAGTCTGATTGGGTAATCATACACTACTAACTCTGCATCAACAGCATAAAATAAGGAAAACAACAATCTCCATGTTATGTTCTTCCTTAAGCATAGTTTACCTAAT<br>GAGAGAAAAGTATAACAAGTATTACAATTAAGGAAAAACAAAATACATTTTATAGAATACCTTTCACACGCAGTCACTCAGCAAGACTTACATTGGCCTTGAGTTCAGTG<br>CGTAAAACTTCATTGGATAAATGTGAATTCAAAGGCCTGAAAGACAATAACAAGAAGTGTACAAGAAGTTAACTATTATTAAAAGCAATAAACACATTAATGAAAAGCCA<br>AAAATACCTGTATGTACATCATATGAAACTAACAAAAAAAGTACTAAAAAGTAACAGTCTCCAAAGTGTTTTGGAGACACCATGTTTTCCAGACCTCACA
LG19	ref-46204	38.489			GTATCGACAACGATCTGTTTGCCATGTCCGT
LG19	ref-50344	39.349			GGTCTCTATACGATCAACTTGCACTACTCTT

LG19	ref-3452_6	39.982			TATTGCAGTGCAAATAATCAAATGTGAAACTTTTCCAGGTGTGTTTGATCCAGACGCAGATAAAATAAAGCCAGTGTCGGTGATGGAGGGAGATCCTGTCATTCTGAACACTGATGTTAAACTACACAAAGATGATCTGATGCTGTGGAGGTTTGCACAAGCCACAAAACGCTGCATATATAATCCGCTTCATCATAACCCGTGT[A/C]TAAGCGACGCCACTGCCATTGCTAAAATTGATGGAGAAACTCGGGAGGTCTCATTAGATGCTGGTGTCAGTGAGATATTTAATAACAGATTGAAGATGGACAAAATGAGCGGATCTCTGACCATCACAAACGTCAGAACTGAACACTCTGGGTTTTATACACTGCAGATCAGCAACAACACTGGGACCAAATACAGGAGATTCAACGTTACTGTCCGTGGTGAGAA
LG19	ref-30801	40			CGCGGTGGCACGATTGTCATGCCGACCGTCA
LG19	ref-46261_30	40.175			GAACCAAATACGAGACTGATGCCATCCAA[C/T]GC
LG19	ref-5661	40.46			ATACGAGTGGCGAAGCGTATGCAGAGTTGAA
LG19	ref-44167_25	40.663			GTGAGATATACGATACCAGTGCGG[A/C]GCGTAGG
LG19	ref-44167_26	40.797			GTGAGATATACGATACCAGTGCGGC[G/T]CGTAGG
LG19	ref-34214	41.104			ATGCTTGACTTGTTCAGGTGAATGATGTAGATATTTTTTTTTATTCATTTGCTGCTGATAAATGAATGGCAAAAATACAGTCCTGCAGGGCAGCTCATTTGCATATGTTTCCATTCCTCTTGTGTGTCTTATGACTCTTGATTTAAATGACACTTGAAAAGGTATATCATTTTTTTTTTCACCGTTTGTGGCATGTTCATTAATTTATGTGACAACATTGCCTGTGCCATATAAGAGCAGAGAATGATAGCACATAATAGGATGAATTGAAGTAGAGCTGAATTGCCTTGTTGAAATCATCTCATGCAAAGAACACAAACACAAACCAGCACTGACTGTTTACACATGATTATCAAAGGTAGACCTCTGACATTTTCCAATGCAACACAGGATGTGCTGCTTACTTTGATGCATGAAATTTGAAATATGC
LG19	ref-70935	41.349			TAAAGACGCTTCAAGAACTGTACTGAACACTATAATAGGAGGACTATAGGGGCGGGGCGGGGCGGGTTTTTTCCAGACAAAGTGACCGAAATCTGTAAATAATGAAGTAGTGTTTTGGGTTCTGTATTTCTTCTTTTTTTCTGCAAAAACGTGCCTTTTTTAACGGATGGATGAATCGCGAATAGGCCTAGACTATTTTAATGATCATTCGAAGCTCATGCACATTGTAACGGGCTTTATCCCATCTTGAGGAGCGAAACGATACTGAATTCACTGCTTTCAGTAGCACTAACGTAGGCCTATTTACTTAATTCTTCGAAATGGTTTAAGGTTACTTCGTAGGTTACTTCGATTTTCATAGTTTTGGGGAAAAAAGGCGGTTTACTAAATCATGAGGAGTTTTTTGATCGGATATTTAAGGAATTGTGAA
LG19	ref-2658	41.684			CACTGGCCACCGATGTCACTGCCTCACGTGA
LG19	ref-26093	41.964	Chr10	2378850	GCGCTTTATTTGGGATCCCAAGTCGTCGGTCACTACAGACGTATGTTGAACGTTACCGACTGAAAGGGAACGTCTCGGTTACATATGTAACCCTCGTTCCCTGAAGGAGGGAACGGAGACGTACGTCCCGTCACCACAGTTCCTGTGCCTTCGCTGTAGTGCGAAAAACAGGGTGCGATTGCATCTGCTTCCGTATTTATACCCTCCTAACGGGGGCGGTGCGCATTATGCAAATATCGCACGCCAGTTCCATTGGCTTGTTTTTATACACTCGAAGCTGATAGGGCTCTCTAGTGATATCCCAATTCGTCGGTCACTACTGATGTACGTCTCCGTTCCCTCCTTCAGGGAACGAGGGTTACATACGTAACCGAGACGGTAACTTTCAGGATATGTAAGTAGCCATAGCAAATTACTCTCTGAACATAACC
LG19	ref-44888	42.294			AGGAAATGGCCGATATATGTGCAAATAAATG
LG19	ref-43239	42.557			AAAGCGAAACCGAAGTGTATGCGTACACTCA
LG19	ref-25345	42.831			ACAGGTGTTTAGGCTGAGTTTCTGTTGCATTTACAACATTCAGACCAGCAGGCGCACATGTTTAAGAACACAGTGCCACGCTTCAATCTGAAACACGCAGCGCATATATTTATTTCATTAAATCACAGCCTTTGTGATTGGATAATTGCAGTGTGCCATATCGCAATTCGGTTAATCATGCAGCCCTATTGTAAGATCCTATGCTGTCTGTTTTTACTGCATTAAAGAATTATTTCTTTATATCCCAGATGTGGTGTTTAAACTGGATGATGGTACCGTTCAGGCCCATAAACCTCTACTCATCTCTAGCTGTGATTGGATGGCAGCCATGTTTGGTGGGCCTTTTGTCGAGAGCTGTACTAAAGAGGT
LG19	ref-65931	43.378			AGGCCAAGTTTGCTGCTGCCATCTCTCTGAACTGAGGTCAAAGGTTATAGCAGGATTATGATGATTAATTTGTTTGCTGCATTCGTACAATGGAAGAACGCTAGTGTTGAGAATCTTAAAGCACACTTAAAACGTTTAGATAATCTGTGTTTTAAATTCCAACATGAGGTTTGCAAATTGCAGGCATATTGTGACATGATGAAAAGCTAATAATTAAATCATAAAATGTACAATTAAAATGAATAAATATTTTTAAAATAAAAACAATC
LG19	ref-64929	43.527			ACATACCATTCGATGAGGTTGCCCGGCGGAG
LG19	ref-50626	44.035			AGAAATGTGGCGAGAAATGTGCCGAGACGGC
LG19	ref-15697	44.933			GCGGTTGAGTACAAGCGGTACAAGAATGGCAGGACTTTGAACTGATAGGTTTGATCCTGAAAGTTGAATCAGAGAAACTTCCTGTGGCGGGGAGCAATGGGAATATGGAAATAAGCGTCCTTTAAGTCGACACTTACAAACCAGTCTCGTTCCATCACAGACCGAAGAATGTCGACTGTGCATAACATTCAAAAATGAAGAGACTTTAGGAACACATTTAAACGTCCCAGATCCAGGATCGGGCGAAACCCGCCGTCTTTCTTTGGAACTAGGAAGTAAGTGGAGTAAAAACCGTCCCTCTGACTGAGGGGAGGAACTTTTTCTATGGCTATAGTGAACGAATTTCTAGAGAAAGGGCCATTGAACTCG
LG19	ref-47552	45.639			AAATCATTCTCAATATTAAAGAATAGCAGAATTCACACCACAGCTATAACGTTAACGCCACAGAGAAACAATATCGTTGGAATCACTTTCAGAACGATTTTATCAAGCTGATGAACGATAAAAACATTGACAGCCAATCAGAATCCATCCTGCTGTAACAACTTCGAGCATTTAAAGTGATAGACAAGCACACACTTAGAATAAACAGAACGATATCATTGCTGGTGTGGACACTAATATAGTTTCTTTATAGATATATTTATAGTTATAGTTCTTGGTGTGAATGGTCCTTTATTCCTTGGCTTAGAGCCCTTTGAAGCTGCATTGAATCTG

					CAGTTTAGACCTTCAACCCACTGGTAGCCGTTGAAGTCCACAATATGGAGAAAAATCCTGGAATGTTTTCTTTTCGACTGAAGAAAGAAAGATATTA
LG19	ref-73372	45.664			GTGGTTTTTGAAAAATAACCGCCGTCACGGTAGTCATAACACCAGCACCAGACCGGCCTGCCCTTAATTAGGGAAGCACGTCGCTCTGCGATAAGATGAGGAGGTGCTGTGTCTTATGTGACTTTGCTGTTCCATCACTCTCGCTGACACGGCTTGGAGCTGTTTGCACATGGAAACAGACATGTTCATCGGTCTTTGTGCGACAGACAGGTGCACTCTGCAGACTCACACACACATACTACATATTTTTTTGGTAACACTTTACAATAAGGTTCATTAGTTAACATTAGTTAACTACATTAGTTAACATGAACTAATAATGAACTGCACTTACACAGCATTTATGAATCTTTGTTAATGTTAATTTCA
LG19	ref-2864_8	46.306			TATTGCTTATTTGTGGCTTGATTCTGCTGTTTAGAATGTGTTTAATATTGCAAGTGCCCAAAACGTGCATTTTTTTTTTTTTTTTTACAGTGACAGCATTAGAAAATAATGAACGGAAATCATATTTGAGGTCATGTGCGTTGTATCCATGTCAATTAGTTGTGCATCTCTGTATG[A/C]ATGCATATAGCTCGCTGTTTGCCATAGTTTATTCTCGATGATCTTTGAAATGATTAATTATGCAAAGAATGTACATTCTTAGTGGCATAAACCTGATCTGAGGTCAAAATGGGATAAAGTAGTGACCATTAGGGCAGCCAGAATTAGGAAGGCCTTGCGATATTCATGCCTGCGTGTGAATGGGACTTCAGTT
LG19	ref-20560	46.319			TACAACGATACGAAACTTGTGCTGTGGGAAG
LG19	ref-44686_29	46.676			AATATTCAGGCGACAAATATGCAGCAAC[C/T]GTG
LG19	ref-72272	47.216			GACAAGCTGTCACTTCATGTCACACATGGCTTGTGAATGTCTGTGGTTTGGACTGCTAAAGGTCTAGAGGTTTTATGACTGGTACACACTATAGTTGAGATTTTGCTTACTTTAAATGTATATATTTGGCAAATTTTTTGCAAACATTTTTGTTCTTAGATGACTCTTTCTTACCAAAGCGAATCATATAAAAAAAAATGCACAAATCTGTGACAGACTTGCATGGTTTGTTTCCTCTACAATAAAATCTTGTTCAATATTTTATTTATATGTTGACAGGAACTTTTGCTTGTAGCACAAAGCCCATTCCAGGTTACAGTTTATTCTGGCCACTTAAGCAGAAAATATACTTCGTTTTTTTTACTACGTGTACTAGTATACTTCATGCATGCACATTGTGACAAATTGCAATCCCTCTATGAGTTACAACC
LG19	ref-3150_23	47.459			GCAGTACAGATGACAGAGACACTGCTGTTTGCGCTATACTGGCTAAATAGCAATGTTATCAAGCAACTCCCCAGAATCCTATTCATTGCAGAATACATTTTATTTTTCTGAGTCTAATTAACTAGTCTTCAGATAAGTAGTGTTGCATAGTTACACCCACAGACTACAATTGTATCATTGCATCCCCGTCG[A/G]TTACAGCATTTTATTATGCTGATTGTGTATTATAATTAGACAGCCTGATTAGGTAGTTGATGACGTATGCCTATGTGAAGTGTGCTTATATCACTCTGTGTTGTCCGAGTGGAAGGTTCAATCATGGTCGTGATATAATTGAGAGCCATTCATTATGTTGCCAAGCCATGTTTTGAAT
LG19	ref-66417_31	47.911			AGCAACAAGCCGAGATGCCTGCAGAACATC[G/T]G
LG19	ref-62414_16	47.913	Chr10	18476512	TAACTCATTCAATACTGAAATCTACACAAAGTTGAACCACTGGCTTGCTTCTCATGTGGAATACTATTCATTTGCATGACATGTCGAGTTGCAAAGCATCTCAAACCAGTAACTCAAACCGGTTTTGTTTCAGTTACGATGCTGCCCTTGAAGGAAACTGTCTATGTTGGCAGTAGACAGCAAGG[G/T]TCTCGAAGAAAGATGCTGAAAGAAGTTGGCTAAGGTCTGGCATGAAGGAATGGGGAATGAGGAAGAACGAAAGGGAGAGGCATGTCCTAACTCAGTCTGGCTCTCCTGGGGAAGGTCTTAAATTACACTCGGATGTGGACAGCTTGTCAATTGCGAGGCCAGGGCCCTGGCTCCAGAGAAACGC
LG19	ref-5475	48.233			CGTTCACATGCACAGCCAACGTGTCACTCATGCAGCAGCGTTTGCATTGAATTCAAAGTTGTTAATTTTGTTTAAACACTATGCATTGATCAAATAAACTCAGGTATTGATTCATGCACACGGATAAGCGTTCATATAAGCTGTCAGGCCTGAAAGCCACCTTGCTAGTGAGGTCTAAATGATCCTCGGCTTCCCCGTTCTGGTTGTCGCCGGCGTCGCTGCGCTCAGCGCCGGGCGAATCCTCCGCGCAACCCGGGTCAGGTGCGAACATACATGCCGGCACCAGCGACTCCACCGCGGGGCTTAAACTGGACTCCGTCGCCGCCATGTACTCCTCCAACTTGGGAATGGAATAGGGATTGATCCAGAGAAGTTTTTTTCTTTTTCTTTTTCGTTGGTTCTGAAACTAGAGTAAGTTGCGTTTAACCTTA
LG19	ref-59798_23	48.541	Chr10	18461261	TAGATTAGCAATAGCAGATTTACAACTCGTATTGACCACTTTTATGATGGTTTTGCATCCTTTATGAAATCTAAAACCTTCAGTCCTCATTCAATGTAATTGCATTGGAGGGGGGTCATGACAGAATTTTTGGTTGAACTATATCTTTAAGAAATGTTTAAGAAGCATTTATGTTAGGGAGATTATGGATGTATGTACAGCCTAAACGGTCGATGAACGTGCG[A/G]ATTGATATTACTGATAAACATATTTTGTGGGCAAGAATGTGTCTTTTCATGTGTCAGTGTGTGTTTGTAGTTGTTTTACCAGTTAGCCTCGTGGGATGAAGCAGCGGTATTCAGATCCTCTTATTTAGATGCAAGTCCTAGCGTGGTTGAACAGTGCTGGGAGATGGAGCGAGGTGGGAGGGAAATGGGGGGATTGGCTGTCCTTGTC
LG19	ref-7668	48.737			ATTAGCAGTGCGATTGTAATGCTTTTTTCTC
LG19	ref-71951_19	48.922			TTCTCAAATGGAAGATTGCATTAGGCTTTGTCCTTCCTAGTACAGTCCTTCCGAGGCTTGACGGTCTAGTAAATGCACTTGGTCATTAAAGTATAGTTAATCCTATTTTGATGGAGCTCGATGACATGGCAGCTGAGATATGCAGCCTTACTAGGACGTAGCCATCTGATTGAGAAGCACCAATCGCTACTCCAGTTGATGATTCTGTCACGAACGAT[C/T]TGCCTAGTTTTTGAGTTTCATTCAGACTCAAACCAGTAACTTTAATTAAGGGGGCATATTCATTTGGTTGGTACAACCAATGAAATGCTCCTTCACTGTCAGCAAAGGAATATTATTGGCTAGTCAGTCAGGCTGCATCCGAAATACTTCCCTACTATATAGGTGAAAAGCAGTGTGACAAAAGAAGTATGTCCGCATTCACAATACTCTTAA
LG19	ref-23132	49.39			CCCTCCACCTCGAAGTATTTGCTAACGTGCA
LG19	ref-14156	49.509			GTCTCGACACCGAAGTAGCTGCAGCAACAAG

LG19	ref-15593	49.615	Chr10	18339684	CACCAAGAGACATGCACACATTCCAACATCCCTCATTCCCTCATCGTTCTCAGCCCACTCGCGACTTCCTCTTTAAGCCCTGCTTCCGTCTGCATTTAGAAGCTGCCTCGTGG<br>CGCCTCGTTTTCAAAGAGGCACCGCGCTGGTAGGGGTAATAGCTTTTTACACCGAGTGCAAACCCAGCATCTGTCTTGCTTCCCAGTGTGTGTGCTGTGCTCACTGGTGACT<br>CACTCTATGTATACACAAGTGGCAGAACACACACATCCGCACACACACACACACACACACACAGACAGACACACAAACCCCCGCATAGGCTTCTGCAGCTATTCCTATGA<br>TAAGTGACACAAGCAAATATGCCTTTCCTTTTCA
LG19	ref-25882	49.68	Chr22	23074463	ATTTTGAACTCTTTATTTCATCAGGAGCGTAGAGTTTTATGGTAAGTTACAGTATCAAAAGTGTGTAAACTGAACAGTTCTTTTCGGTCCAGAAAACGCAAACTTTCCTTGC<br>AAGAAGTTATGATGCATAACTCCACACGTTTTGGTTGTGACAGTATTTCTTGCACTTCGCTGGATCGCATTTCACACGGTTTACAATCAAAACAAGGTCGATAGTATTGCGT<br>AGGGATATCATTTATAGATTCTGAAGTGCCTACATCTTTTTTTATCTGCTTCTTCGGTTATACAAAGGTCCTGAGCTGTCAGATGTGTTCATCTTATCTCAAGCTTGTTCTGA<br>ATTAACGGGTAAAATAAACTAACCTTTTTTTATATATACATATTTCTCTTTGGACATGAACCATGTCTTTTCCCNNNNNNNNNNNNNNNNNN
LG19	ref-72056	50.379			AGCACGAGTGCCATTTTTTTTTTTTACAACAGTTCAATAAATAAGTGCGTTATGTTTTAGACATTTAGATGTTTGCTCAATTTTGCCACTGAATATATTACCTATAAAGCCAG<br>AGCAGAACTGTTGTGCTTCTCCAAGCAACACAAAGTGGTGCTTTGTTCTGAATTAATCCACACTAAGCGAATCAATCGGGTGAAGCAATGATTCACTGGCCCATTCACAAA<br>GAGAGCCTTTACTTCATTTCTGAATGAATCAGCCATTTGAACTAATCAAATGAATGAATGACTCAATGACTTACTCATTTAGACATTTACGGCCACCTAATGACAGCAAAG<br>AAAATTGTCATCATTTACTCACACTCATGGCCAT
LG19	ref-7675	50.613			ATTCAGGATACGACGCGTATGCAGAGGTGGG
LG19	ref-16211	50.706			CGAGGCGAGGCGAGGCTTGTGCTCTCGTAGT
LG19	ref-60283	50.959			GCCTTCTGGTCATTTTTGTGGTTGCAAAACGCTGTCAGTGTGAATGTAGCTTAAGTGGGGTGTTTGCGTACAGAAAGTCAACAGGCTCTCCCGCCCGAGGTGATGACTACTT<br>AAAAAAGCCCTCTCTGTATCATCATTTTCATAATTGCTTTCACGTCGTTTACATGAAGGCCTTTAGTTATGCGTACTTAATGATAGTGCTATTCCCTCCGAGCTCCACGCAAC<br>CTTTGAGGCTAGAAAAGTTCACACAGGTAAGCACTTGAAAAGTGCTATTGAAAATGAGGCCGGTGTGCTTATGCATTAAACATTAAGTCCCAGTAGACGACGAAAGAGAA<br>AAACACTCATTTAATCGAGTCCATTTTCCAAAGAATTCCAATTAAATATCTAAAATGAAATAAAATCCTTTACGGCATGTGAAATCAAAGCCCATC
LG19	ref-54139_32	51.085	Chr14	28706018	CAGTAGCTTATAATGTTACTACAGTAAATGAATGCGTATTAATACAAAGAGTAAACCAGGGGAAAAAGACGAAGGTGCCTCCTTGATTGTCTCCTTAAAATGACACTGAA<br>GTGCGTTCCAAAAAAACACCCTACACCCTTCATCCCTTTGAAGCTCTCACTCCGGAGGGTAAACCGTTTGAAGGGATTAGGGCATAGGGATGAGCCCTTCCGAATAGAATG<br>CAGGATCTGC[A/G]TTTTGATCATTCATTTACACGACAACCGTATTTTGGGGGCCTGAAAACACATACTTTTGAAAACAATGCTGTTATCGTCTCCATGTAAACTACAAAAA<br>CATAAATTTGTGAAAACAGTCTTCATACGCATGCATGTGTATTACGTGTTGAGTCTATAGGCGTGTTGTGTTTCCTTACAAAGTGACATCGCCATCTACTGG
LG19	ref-41252_5	51.193			TGTT[C/G]TGTCACGAGCCACTTGCACTGTTTGCT
LG19	ref-53022	51.437			TTGAGCCCTCCGATGACATTGCATTAGAAAC
LG19	ref-22171	51.476	Chr22	31935984	AACACCTTATTGCGCATCGGTGTCCATTGTGAATGTGAGAAATAAGCAGTGAAATGAATATGTGGGGACTTTGGTCCCTTGTGATTGCGACTGGTGATATCATGACGTTGC<br>AGGATGAGTGTGTTAGCACTATAGGCAAGAAAGACGATATCAGTGTAATCACACACTAGCTTCTTTTACATACTGTCCCATCCTCCACACCGCGCCACACAACTCTACTGC<br>TCCTCGTGATGCTACAAGACCAGGCTTTGTCTTCGACAAGGCCTCTGCTGCCTACTGATTCACCACAAGACCCAGTGATAGAGATTGAGTCATTGGGGAAGGACTGTTTTT<br>AAGAGTATTTAATGCCTATTATTTTATATTATTACACAGCACTCTACAATAACCATTTCTAATAGGTCAAGCATTCAGCAGCATTCAGCAGTCAAATG
LG19	ref-56898	51.585			TGAGTGAACCGGAGCCACCACACACACGTACACTTGAAGCTGTAAAAAAAAAAAAAAGCCTTTGCGCTCCTTCACAGACAGTTTTGGGCGCCAATTAGGCGATATTAAAAA<br>GGCAAAACTCTTCAGTAATGAGAAACCGGAGCGCAGCTTTGAAACTCAGGAAAAGAGGACTAGCTTCCGGCAAATGACTCGATACACTGTTCATTTAACAACTACACTAG<br>TCACAATGGGCATTAGCCATCGAGTTCATCTAAAAGTTAGGGATTTTGAGGGGTTTCGTGTTTTTTTTTTTTTTTTGGAGAGGGAAAAGCAGGTAAAGGAGGATAAAAGGA<br>GATTTGAGATTAAACTGGCAAAACAGCACCCCTTTAAA
LG19	ref-16256	51.716			TGTACCATCTCGATGACTTTGCTCGCTTTGA
LG19	ref-13412_6	51.783	Chr10	9967176	ATATTTGGTTTTAGAGATGCATTTCTAACGAGAACAGTTAATGCTAGAAAAGCTAAAATCGTGATAAGGAACAGTATGGCTCGCTATTTTCTTTGAAGCCTGGCAGTGATT<br>TGGAGGGCGTGAACTAGTTTGTTTGTCCTATTATTCTGCAACAAAGTGGGACTGTAATTGTTT[C/T]CAGGGCAGCAGTGTCGCCTCAGGCTGTCTTCAGGAAGGCCAGACT<br>GTCCTGGTGACAGAATCCAGTTAAACCCAACATCTCACTCATGCTTTTCTAATACTTCTATTATCTAAAACTGCTAGAGGGAACCAAAAAATGATTAAAGGATTGACATCA<br>GTCAGAAAGAAGGCGGTGAAGGTCGGTCAAGGGAGCAGT
LG19	ref-61635	51.954			GTTTCCGAAACAAAACGAAAACACAGTAAAAGAAAGGCACAAGTATAACATATAAGTTGAGGAACATTGAAACAAAGGTAGTCCCAATTCCAGAGAAGTGCTTCCTGTG<br>AATAGCGGCATTTCTGTAACAATACTGAAGAGGATCGCTGGTTAAGGAGAATTGCGAATGTTTATGCTCAGCATGGCGTTCGCACTATCACAGGGGTGGCGTTTACGTCTG<br>TGGGCATTCAGTGCTCAGATCCAGGCTAGCCTCAGTGGTGTCCATCATGCTGCAGTCCAAACAGTCTCTAGGTGGTGCGCACTGCCTCACTCTGTCCACTTGCGACTCGCGG<br>GCAGGTTCAGATTCTGCAGATACCACAGCTTCTTTGG

LG19	ref-23629	51.985			TTACCTGGGCTTAAGCCCAGGCCAGGCCCCGGGCTGAGGGAGGGCCCCGGTGATGCCGGAAAAAAATATAACCGTATTTTATAATGTGATGACTGTCCCTTTAACATCGCTTTGCTTTGAAGACATGTGCGGGTGTTTCAAGTCCAGCGCGTGAAGTGCGTCAAGACGCAAACAACTCACAGTATCAGTATCTGGACAAGGCGGACAGACCGATGGCGCTGCCTGCACATCCACTCATAGGGCTCGTTCACACAGAACGCGTTTTTGCTTTGAAAAACGCGAGACGCGGGCAGTGGAATGGGGGAAACCGCAAGGTCTCGAGACCTTATCATATGTTTAATATTCTCCAGATGTTATTGACATCAACTTGTCTATTATTAGGAATTCAACGCGAGTAGCTACGTCAGGTGAGTTCAACAGGCGAATCATTA
LG19	ref-29737	52.049			CATGACAGTGCGAACAGCATGCTTGCTTTAT
LG19	ref-55154	52.094			ATACAGTTCAGACTGCAAAAAAAAAAAAAAAAAGAAAATACAAAGACTATGAAAAAAACAGATTTTGATCGTTGTTTCCAGAGACATACACTGCTGTGCTAACTTTCAGTTGCTCTTAAGAAATAAGAAACATGATGAGTGCTGAAGGCGAGCAGTATGCAGCAGGATTAATAAATAACAGCACTGGGCTCATACTCACTCTTTGGCAGAGCTATTTCATATTCAGTACCTGTAGCTGGATTTCTCTAGCAGAGACAGCTGGAGTTTGGGCTCTGACAGCTGTGTGAATCTGGGTTGGTCTGGCAGCTGATCTGTATACAAAAATTAAATATCGTAACGCCAAGAAATTTTGCGACGTCATCTCATTTCA
LG19	ref-52439	52.191			CGGGTGCTCTCGAAATGCTTGCTCAAAATTT
LG19	ref-16421_31	52.248			GATGAAGCTCTTAGGGATCCGATCGACAGGACCGGCCTTTCGCCTGTTGAAAGTGTTGCACGTTTGTTTCCGCACCCTTTCGATATGGCGGGACCCACTCTTTCTTCAAAAAGGGGTCAACATGGGTGCTGTTTTCTGTTGGCAAATGATCACAATGGACTCCTCTCTGACCGGCTGGGGCACAGTCTTCGAGGGCAGG[A/G]CAGCGTGTGGAGTGTGGACGGGAGAGTTCCTCTCTTGGCACATAACAGTTTGGAGCTAAAGGCTGTATCCCTGGCCCTGCTGCAATTTCTCCCCTTTCTCAGGGGATGCCACGTCATTGTCAGGATGTACAACATGGCGGTGGTGTCTCACATAAATCGCCAAGGGGGCT
LG19	ref-15331_32	52.329			ATAATTTTGAAGGTATTTTAATGGAGAATGTGAAAATATATTTATAAAACATATTGTCACAATCGTGCGTTTATTGTCTTCAGCAATCCATAATGTTTGGATAGCCTCGTTTTGGATAAGATTAAAATGACAGGCCATGGATAGACTATCTTGTCTCTCTGAATTTAAATCTGGATTTCTTCACATTGGCCTCTACGTGAACACATTTGCCGAAAAAAGTGCAGGGGACGT[A/G]TTTACATTTTATTAAAACTGTGGGGGATGCGTCCGTCCTCTGCGTATTCTAAGCCCATATGTAGATGTATGAAGCAATGCTCATTTCAATGGAATGAACCTTAAAATGAACCTTAAAAGGTTTTAATATTTAATTTAAATATTTACATTCTTAATAAACTTTTCTGGTTTTATTATGACCGATTCATCATCCAACTGTAA
LG19	ref-49728	52.356			AATTATTATTATTATTACAAGCCAATAAAATTTTTTTGAACTAAAAGCATGTATATATTTATTTATTACCTGATCCGTTAATTCATCAAGTTTATTTATATTATTTTAAAAGGATTTGATAATCTACCAAACCATTGTTCTAGGTTTGTGCCAGCTCTGTTCTCTTTCAGTATAGCTATGAACAGGAGGATCTTCGCTGATGCTAAGCTGCAAGTAGTGTGCTGTCTGGAAGAGTAGTGTAACAATGAAGTTGCACAGAGCTGTCACAGCATCACAGGAACAACTGCTGTGGGACAGTAATGCAGGCCGGCAGTCATTTAACACAGTCTGAGAAGATTAAATTAATATGACAAAAGTCATGAATTAATATTCTAATTTGAATTTTTTAATCATACCCAAATACTGTAATGAGTACTAGGCCTATACCTTAA
LG19	ref-9869	52.486			GTGCAGGCCACGAAATGGCTGCTTCTTAGCC
LG19	ref-52895_9	52.569			TGAACAATTTTGGGGTTATTGCACTGTTAAATTAACAAAAAAAATACAAATCTTTAAAACAACCAAAAGCACAAGAAAAAAACTGCAACAACCTCTAAGAATGCTCAACAATTTCTCAATGTTAGATCACTAAAAATCACATCATCGAAATTCAACACGTATCTGTCTTGCGTACAA[C/T]AGCACATCAATCGCACCAGTCTCCTGTAATGTCACACCAGAAATATCAAAGCTCTTTTACTCCTCACACTTTCACACATAATATATTCTGCATTCAGAGAGGAAAGAATATATTAGCCTCCTGAGATGTCTGACCAAAACATTTAAAACAGCGCTAAATCAAAAAACCTGGCAGCCACTTCGCAATCTATGG
LG19	ref-24895_4	52.618	Chr22	33216847	GTACATGAAAGAACTTAAACCTCATCTCCGGTTACGGATAAAGACGGCCTTTCAGCTCTCAGGAATGGCGGGCTGATCTCAAAAAGGTGGAAAAAGGGGCCTATCACAGCATTCCTCCCTTGATCAGCCCGGCACAAGAAGCTGCATTGGTCACAGGAACCCTGCCGCCAGA[A/G]TCACTAGCAATAGACTCGGACCCTTTCAGCAGTCAAAGTCCAGAGAAGTCTTTGAAATGGACCATTCTGTTCCAAAGACAAAATGGAAATCATTTCATGCGTGGGAAAAAAATGAGAATCCTGAAGGCCAGTCTCTACATGCCAGCAAAACTACAGAGGGCCATTGTGTTGGAGGGCTGAAATAGTCCACTGGACGC
LG19	ref-62948	52.865			AGTTTGACACCGAGCACTTTGCTGAGGCAAA
LG19	ref-7365	53.005			ATTTGTCAACCGATTGGATTGCTTATGAACC
LG19	ref-7320	53.032			ATAGTGTGCTTACATAGTTGTGAATCGGTCCTTGATTTGTTAAACTTCCACTTAGCGCCTCTTTGTGGTTTTTCTACCCAGAATCCAGTGGTGCACTTTGATCTCCCGCTGCGATTGGAGCATTGGTTTCCACTGAATCAGGTGCGCAGGAAGAGGGAGGAGCTACGGGAGCTGGTGCTGCAGCTGTACCGGAGACAAGGGGGCGGAGCTGAGCAGAGCCTCAAATAAAAACAGTGGGAAAATGTCCTGTCAGTAAACGAACAACAATTAGGGCATTTTGGGCTTTTTTTTGTTTTGTTTTGTTTTTTACTGATTAAAAGATAAAGGATATATTTATAATTTAACTTTGTATTTATTTGTATTAAAAT
LG19	ref-68617	53.232			ATGGGAAATCCGAATTGCTTGCGAATTTGCT
LG19	ref-47443	53.39			CATTTTCATACGATCTGCTTGCGCCCCAGTG

LG19	ref-42313	53.549	Chr22	16600878	CTGCACCTGCACGTCGCTCTTGATTATAACCTTCCCCACATATGCCACTGCCTTATCAACATCAAAGACCGATCGTACCTGTTTACCAGACCCATTTGGCTCTCAGTTTGAATAATTAATGGCAATTTATTATTATTTTTCAGCCTAAGATGTTTGTAGTATTAGACTTCAAAGGCAATGCAGCCCCTTTGTTTGCATTACGCTGTTTAATTACCTTGACGTGGCCTTGTCATTCATCTCTGTCCATTCATAACGCAGCCTTGATGGAGCCGAATGAAAATCCCTTGAGGACAGACCCTTCCCATAGACCTAATTGTATTGGTGCACAATATGCAACAAGCAAGTTTAGCTCGTAACATGTTGTTTGGG
LG19	ref-13745_17	53.726	Chr22	17359659	TTAATCTTGTTCATGCATAATACATGCCAGCGCGGTGTGTGATCGCATTGACGGCCGGTTATTAACTGTGTCACAGTGCGAAAACACACCCGAATCAACAGACCTCACATGCATATACAATCCACGGCTAAACTACTGCAAAAGACCGAAGACTTTGTGCAAAAGGCGATAAAAAACCTGTAATCATCAAAAGCTGGGGACTGACTGACTCGAGGC[A/G]TATGCTAACTTGAGTTTAAAAATGCAGTATTGAATACTTGAAGATGCAAATTTGATGATATCCCTTCCATTTGATCTAAATGTACACATTTTCTGGATTACGAGTTTCACAACTTTCACCAAATGTTGAGACTGTGTCACAAAGTTCATTAAAAACCAGAGAGCCTGCGGAGCTCCGTTCTGTCATGGCTCTTCGAGGATTTTTAGGTCTCCCGA
LG19	ref-27727	53.895			AAAAATCTAAATTAAAAAAAATTATAGCAAAGCAGGTATTAAAATACGAACAAATTAAGTCTTATTTTAAACTGCTAGAATCTTGAGAGAATTTTACTCAATGTAGCCTATTTTGACACTCAATTTTATACATTCCACAACCATTTGTTGAGACAAAGTGAAAATAAAATCAATATGCCACTGTAGTGTGTGTAGTAGAAACTAGCTTAATGACGAAACTGCCTAACCGACCTGTATTATACTCCTCCCCCTACTTACAGGTACATGATCAGGTGCTTTATAGATGCAAGGCAATATAACGAAACCTTATTGATAAACTGCAGGTACCAGGCCCTGTAAGGCCTCCAATACGGAATTAACTGTGAATTTTAGTATTAGTTTTAAAGGGGACGCATAATAAGGTATGTGAGTTGAGAGTTTTCTTTCCTCC
LG19	ref-41252_30	54.204			CTGTCAAAAGGATACATTTTCTGCCTGATGTTTTACTTAATCTTTGTCATCTGGTAACCATGTGCACGCGCTCTCTCTACACTGTGGAAGAGTAGCGGATGATCTGAAAACACAGACAAACAAGTAAGCTGGACTTTTCTATTTGAAAATATTCTATTTGAGATATTCTGCTTAAATGGAAGTGTCATTCAGCCAGTCTTTGTTCTGTCACGAGCCACTTGCACTGTTT[A/G]CTGAGACTAAATCTACGGTCAAACCTTCTCAGTGATGAACTGTAATAAATCCAAACATGGATCTCGACTATATTGTTTGCGATTGTGGTTGCTGAATAAATGCACTTATGCAACCTCTGTGCAGCAATACAAAGTATTTTTGGATGTTTTTTAACAGACATTATTGTAATTTGGATTATTGGAAGTACCAAATTTCACTGTT
LG19	ref-61481	54.755			GTGACTATGACGAGAGTATTGCCGTTTTTTA
LG19	ref-8305	55.121	Chr24	4849519	CATGGACGATAATTATAACTTTAAAGTTTCAATGATTGTCCAATTCTAGATTGTCTAATGATTTTCAGTAGCGATGTCCACAGCTATAATGATGACACAGAGGAACGATGTCATTAGAATCACTTTTTGAATGTTTTTTTTTTTTCTCTCCAGTTGATGAATAATTAAAAACATTGGCAGCTAATCAGAATCCACACATAAAGAAGCAGACGACAAAACTGCAGCGAATGCTTATAATTAACAGAATGTTATTGCTTGTTGGTTTGTACGCTAATATAGTTAGTATCGTTATTGGTGTGAACAGGCCTTTAAGTATAGGTGGAAACACTTTCAGAAAGATTTTTTTTTCCAGGTGATAAATGATAAAAACATTGACAGCCAATCAGAATCCATCCTGCTTTAAAATTTTATGTGGCAGGTGACAAAACTAC
LG19	ref-19924	55.333			ACGTGATGACGTTCTCCATATGCATTCAAACGCAGTGTAGAGTTCCCTTTCGAAAGGGAATACAAAACAGGAGTGGGAGGGAATGGGAGTCAATTTTCTGGGATTGGGACAGGACGGGATATTTTTGTGGGAGTGGGACGGGAGAGGTTTGAAAATCCACTCCCGTGTCACCCTCTAGTGCAGACCCATCGGTGTATGACATCAAAGTAAAGTACAGCGAGAGCGTTTGGAGAGCATACGACTCCTTGTGCTTTTGAATGGCTCTCGCGGTACTTTGATGTCATAGGGCAGGGGTCGGCAATTAAGTTTGGCATCGGGCCAAAAAAAAATTTCACCACTAGATGGCGGGCCAGAACATAGTCAAAGGAT
LG19	ref-23711	55.546			CCACCTAAGCCACCACTGCAAAGGTCTCATGTACAGCAGGCCAAAAGGTACCACGTTGGACGCTGCTGCCATGAGGCTTAACAGTCTCTGAAACTGTTTCACAGTGATGCATTGGACTAGCTTTAGCTTGTTTACGGCTACTAGTATCGACTCAATACGAGTAGGGGAAAGTGTTGCCTGCATCAATATCGAGTCCCACACTACACCTAAAAAAGTGGTCTTCTGAGCTCGAACGAGCACACTTTTCTTGGAATTGAGCTTTAAAGGCACAGTGTTTTTTTTGCCGCTAAACTCTTCAAAATAATAACAAAGGCATAGATCGATTGTGCAGTGAATGAGCGTAGGCTCATGGAACTTGTTGTCATTCCG
LG19	ref-36127	55.584			CTAAAGCGGACGACACTAGTGCACTTACGGT
LG19	ref-49035	55.866			ATTCTCCAACGCACGTTCATACAACAACGTCTGCTTTCGCGTCAACACAACACACATGCATCATGGTGTTCTCGTGAATGTGCACACATGCATGTTGTGTTGACGCAAGAGCAGACATTGCGTGAACGGCTGTTGGAGATTAATATTTTGTAAATAAAGTGGTAAATTGTGTTTTTTGTGCAAAGGACTCGTGTCACTTCATGAAACTGAGGTTAAATCATTGTAGTCACATGGATTACTTTAATGATGTCTTTACTACCTTTCTAGGGCTTGAAAGTGGTAATTGCGTAGACTGTCAATGGAGGGGCTGAAAGCTCTCAGATTTCATTAAAAAGATCCTCTTTTGTTTTCCNNNNNNNNNNNNNNNNN
LG19	ref-18025	55.898			ATATGTTTACCGATTTTGGTGCCTGTAGAGT
LG19	ref-37124_23	55.933			GAGACTCAGACAGAGGAATACAATTAAACAGATGTCGTCATTGACAGCAACAGTGCAGATTGGATGATGGCAGGTAAGTAGGTACTTCAAATGATACTTTGACTGTATAAATGATGATAATGATAGAATTTCTTTTCTTACAGGAGATCATGGATGGACCAGAGGGAAGACAAATGGATGCATACACCTCG[A/G]TAGTATAGGTGAGTATGGCTGACATGGAGACTTGGATAAAGGAAGGAAGACGCTGGAGTGGAGAACGGGAGAACACACTGGAGATAACGCAGGAGGGATAATGGAGAATAGTCGCTTGGAGAAACACTGGAGAGAGTCCTCTTCGTTGGAACGAGACTGGGCCATGAGTGAAGTGTGC

LG19	ref-43598	56.571			ACGACATCTCGATGCCGAACTGCCATCTCCTCTGATTGAGCTAGAATGAGCCAGTCGTCCTCCAAGCTTCTACATATTCTACTAAGGGGACAGTCTCTCAAGACTGACCTCGAGTGTCATGAGTGAGATAAGTTCGGTGCCCTGAAGCGGCTGGTCAGCAGGGGAAACTCGATCAATATTTTCTGCAGGCCTCATATGAGGGAGCGAGCTTGATAGGGCTGCAGCGAGATCAGGCGTACACTTCGATTTGCGTTCGCTGGAAACTGATGCATCCCGAAGCGCACAAGGCGGCGGGAAGCAAACATCGATTTCCTGAGGGCTTCGCAGAGGAGCGAGATGTTGTCGCTCCACCACAGGGGGGACTGCCCTCAGAGCAAGCCTTCCTCGCCAGAATGGCAGTCCTCAGATCTGCCTTCGGCTTCGAAGACTTG
LG19	ref-49711	57.103			GCAATGTCTGCGATGGTGGTGCCGGAGAAGC
LG19	ref-21231_25	57.647			CAAGTTGTACCGACCAAAGTGCAT[A/G]TTCAGTG
LG19	ref-70396_28	58.181			CTTAGGATGTCGACTGTCATGCTTCTC[A/G]CATC
LG19	ref-70396_14	58.207			TGATGAAATGCAACGGTACTCACGGAGATGGAATCTCAATACAGGGGGTCCCAGAGCACGACGGAGAAGATGTGAGAAGCA[C/T]GACAGTCGACATCCTAAGAAACATGGTCCCTAGTATCCAGAACAAGCTGGATGATGTAGTTGATGTTGTACACCGCCTATAGGGCAATGGAGATCCGACGGAGCACCTCGTAACATC
LG19	ref-28417	59.032			CATACATCAAGAATCAGCCTATGAATCTCAACAATGGTGACATGCTTAATGCCGCAATGCATTCTGGGGGCCATAGATGAGTTTTGCATGATACACCCAGAATGCATTGTGGCATGAAGCATGTCACCATTGCTGAGATTCATATGCTGATTCTAGATGTCTGTATGTGAGGAAAGTTTGCAGGGGCTTCGAAGTGTTTAGTGTACAGTGTGTTTTAAAACTAATTCAGCTTTTTTGATTAAAACTAAAGATCCAACACAAACTTTTGCTCCTACAAAGCTCAGTCATTTGTAAAATATATTGTAAAGTATATTTGTAAATGTATAGTATAATGCAATGTAAGTCGCTCTGGATAAAAGCGTCTGCCAA
LG19	ref-50145_9	59.192			TGATCATGCCAATACAGTACATTTGCTTTATCAAATAAAGCATTCAGAATGAAGTCCATTTTAAAAAGCAAATTCTTCCCAGTATCCGCACCTTTACAGCTGATACTTGAACTTTAATAAATGATTAATGCAAAGGCCTCACTTGCATTGCAGTAACATATAAATATTAGCACATTCTCTATGACATCATAGTGCCTTGTTCTGAAAG[C/T]GCGAAGCGCGTGCCTTTCTTCCTGTAAACAAAAAGGTTTTTTTAAGAGATTGGCAACAATTACATCATTCAGTTGGTTTACATTCAACACAACCGGTGTGTGTAATAGCATCCTGAAAAAGGTACGTTTACAAATGACTTTTACAGTGCAACACATGAGTCCGTTTGGCCTTGTACAATTCTGCTATGTTCACGAAAACTTTTGACCTCTTCCAGACTGTAA
LG19	ref-4551	59.682	Chr22	26926813	TGCTTTCCGGTCAGCCGCAAGGTGCGCACCTACCCGGCCAGCCCATGGCTAATGCACTCAGTAATCAGGTGTGCTCACCGGCTCCCGTACAGTCGCCTAGACCTCCATCGCGGCAACTTCCTCCACATTCCAGTCCTTCCCCACAGGTTCAGCCACAGCCATCACCGCAGCATGTGCCGCCTCACACAGGATCACCACACCCTGGTCTGACAACACCCATGCCTGGCTCCATGGAGCAGGGACACCTGGGTACACCTGAGCAGAGTGCAATGCTCCCGCAGCTTAACACGCCCAACCGCGGAGGGCTGCCCAGTGACTTAGGTATGGTGGGAGACACAACGGGAGACACGCTGGAGAAATTTGTGGAGGGATTGTAGCGTTTAATGGGAGACTCCACCCACCATAGTCTCCCCATTCCTCTTTTTTGTGGC
LG19	ref-44167_1	61.39			CGGCACCAGCGTAACGAAGTTAGTAGTTCAGGGAAGAAGGAGGCGGGAACCGGCGAATGTTTCAAACACTTTAATCCTATAAAATAAACAAACACAAAACGAAAGTAAAGACAAACAAAACACAACATAAATAGTCCAGGCCTGGTCCTCTCTTGTCCTTCACTGTCGTCGCTCCTCCTTTTATGCTTAAGAGCTCCTCC[A/G]TGAGATATACGATACCAGTGCGGCGCGTAGGTGACACTCATCAATCACGCCACTGGCCTCGCACCGTTCTCACGGCCCTCAGCCTCGTCCTGCTTGCCACATTTTGCAATATTCATAAGGCGGTTAGAGAATGATCTTTAGGTTTCGACACAAGTCAGTTTGACAGACTGGTTTGTTAATATTTTTGAAATGCATGTTGTTTGTTGTAATGCTAGATGTTTTTAATGTGAC
LG19	ref-41329_10	61.717	Chr22	11112854	TAAATAAATAAATAAATAAAAAGTCTCAGAAGGACTTTTTTGGGATCCTTGTAACCCTTACAATTCATACTGGACATCTACATTACCATTACACTACATTCCCCAATTTTGTTAAAACACGAATCGTTCGCCTGTCAGTGTAGACTCTGAGCACGTTCCCGAGCTCTTTAAGCGCGTTCCCCAGCGCTGTGACCTCCAGGTCGCCTCCC[C/T]CGAGGATGATGCTTGCGTGTGGGGGAAGGGTTTGCCCGTCAGGCTGTGGCCAATACATCCTCCGTCAAAGCCCCGAGCAATCCTGTTACACACGCAGCTCGCGCGATTATCCTGCTCCTCTCCTTGGCGCGACAAAAACAGCCTCTCGCGCAGAGCGGACACTAACATCACTCATATTAGTATATTTACCAATATTCTACTTTAATTCTTTTTGAAATCCAC
LG19	ref-15216	61.743			TCATTTGTGTGTTTAAAAGAGGAATTCCTGCTGCTGTTTTGACTCTTTGTTTTGGATGTTTGGTAAGTTCAGTCACAAAATATATGTCATAAAAGACGTTTTTTGTTTATTTGTTTGATATCTTTAGGTTTGGTGTTAAAAATGACAGTGTGAGGGATAAACAAACCAGGACTAAGGCAGCATTTACACCGCAGGTCTTGATGCCTAATTCCAATTTGTTGATTATACAGTATATCTGATTTTTTTTGAAGACCAGCTCACATCTTCTATTAAAAGTGTGACCCATGTCAAATATCTTGATTTACTATTTTATTATACATTTGGAAAAACAACCCAAATTAGACGTACTGACCGGAAAACCTTAGGCCA
LG19	ref-41348	62.13			GTGATTTCCGCGATGCGAGTGCCATTAAAAT
LG19	ref-4481	62.362			TAAAAGCCACCGCAATGCTTCAGTGCTAAACATAATCAACCATAACCGCTCAGGTTTATACATGAAAGCATAGCATACAATCCTCAACAGTGGTGACAATAATTATTCATACTGCAGTGCATGATGGGAGTTTTGTCCTATGGTGATACCAAGCACGCATTGCAGCGTGAAGCGTTTTTTCACCATTGTTGAGATTCATATGCTGATTCTTGATGTCTGTGCGTGTCTAAGTGCACATTTAATGTGATATTATGTAAACAATTTAAGAATGTATTATTGACTTTTATTTTTGGTGCTTGTGAACAAAGTGTGATCATAACTGAACTTTGTTCAATTGGTTCATCTATATATATTTTTATGATTAAAAAGTTTTTTTTACTACAATTCTTAAGTTGTTAGTGTATGGACCTTATATGTACTGTATTGCAGTG
LG19	ref-16303	62.418			CATAAGCGAACGAAGCGGCTGCTTAGGGCCC
LG19	ref-7483_4	62.506			AAT[G/T]TGATGTCGATCTGACTGCAAGGGATTAT

LG19	ref-7483_1	62.546			CAGATTCTCAAACAAGAAAAAAAAGTAGGGCCCCTACTTTTTAAATTCCGCCCCTGCAAGCTCACATTCATCTGCCTCCACTTTTGCATTCCATGCCCACATCTGAACATTTAATAAACAATTGATATATAGAATCAAGCCACTGCCTTACTTTTTTTTTTTTTTTTT[A/G]TAATCCCTTGCAGTCAGATCGACATCACATTAGGGGAAAAAAAATCGCGACTTAAACTGAAAGAAAAAAAATATTGCTCAAATGTTGTTCATTCATAGCTCAAGAGACACAATAGAGTTCTAATAGACATTCAACCTGAGCTGTCTCATAGCCTTCTAGGAAAAACTATTAAGATTTTGAGTTGAGATAATATTTGCAAA
LG19	ref-67172	62.738			TGTTTTCCAACGATGTCCTTGCAGATTTCGC
LG19	ref-53935	63.054			CGCAGCGAAGCGATGCTTGTGCTCACGCCTC
LG19	ref-18321	63.104			GGATAACCTTTGAATCACTATAATGAATATAGCGTATAGTGAATGATGAATAATGAATTTATGAATTCACTACATTCGTGTTGTTTACATTATATGCACTTGTAGGTGCTTTTTGCCAACAAAACAGACATGCAGTTGATGCAGTTTTATTCACCACCTGCAGTTACGACTCTTGACCGGCATCATTATCGCTGTGACGCTCCATCTTTCAGTTTCAAACGATCCGTAAATCCAGCGTAGAACTGGGCCTTGTTTATGAAACCATAAGCGCTGATCCTGAGGGCTCGAGCAGCCACGGAAAAACACAAGATATTCTCCATTCATTTTAACCAATAAAAAAATGATTGCAACTATCAGTTACTATGGTTA
LG19	ref-64807_29	63.14			GAGCCAAATGCGATTTGCTTGCCACAAT[C/T]TCC
LG19	ref-59014	63.375			TTTCAGTTTTGTTCTGCACCAACTCACTGATCTACAGAGTTTTTTCTCCCATTATAATCAACAGTGGCATAGACCAGATATTTATTATACACAGGAATGTATTATGGATTGGGGCTAAAGCCCAGGGGTCCAGGGGCTCATTTCCTTGCTTCTTTTATTTAGCATCTTTAGGATGGGTTGCACCAAAATCGAGTTGTGTTGCACCACTTAATTTTAAACTCAGATTAACAAATCAGAGATTAGAATTCTAACTGGTGAATAAAACTTGCATTTCCATTAATTTTCTCCACCATGACAGATTCGCCATTTTAATTAACAGAGCAACATTAGGCTAACAATGTTAATATTCCGTATCCTTCTTAATTGAAC
LG19	ref-39661	63.546			ACAAAAACAGCGAAAAGCCTGCCTGCAAAAG
LG19	ref-6927	63.823			CATCAGGTAGCGACTCCATTGCGGTTGGTTC
LG19	ref-1825	63.996			GTTAAGCGAGCGATGATGATGCCTTCATCTC
LG19	ref-1989	64.261			CTCTAGGGCCCGAAGCAGCTGCTTAGTTCGC
LG19	ref-69998_31	64.623			GTAAAATACGGACAAGTTTTCAAGTGGGTGTCCGTTTTTAACGCGTCTTGACTGTCGTACAGTCTGACATAAATGACAGCTGAGATCCCACAGTGTGACATGAGGATCATGTTCGTACAGTCTGACAAGCAACCATCGTAAAGGACTATTATAAATCGCACAGTGTGCACTCGGCTTTAGCAACAGCGTCGATTACGCA[A/G]ACTCACAGAAAATGTCAGAAACTGTTCAGCCTTTTATGTTCAAATCGGAGTTGGAAAATGATGGAGAGACTCAAGAAGAAGTGACAACATGTAGAATGCAACAGGATGTTTCTAAATGGTTAGCTGATTAATTTATGTAGCTGATGTGGAGTTAGCTATCTTAAGATCTT
LG19	ref-2491	64.676			AAAAGCCTTGCGATGAACTTGCCATCCTATC
LG19	ref-58010	65.807	Chr22	6087429	ATGATCCATAGAAAAAAATTCACAGCCCTATTGTATTGTTTTTCTTATGTTAACTGTATCTTATCCAGGTGTGTCTGGTGTTGATACACATGAAGTGTCAGTGATGGAGGGAGATTCAGTCACTCTACACACTGGTGTTTTAACAAACCAACATGATGATATTAGATGGTATTTTAATGACACTCTCATCGCTTGCATCAATAGTAAAATCTGTACAGATGTTCAGTGTAATAATGACACTGAGAGATTCAGAGACAGACTGAAGCTGGACAATCAGACTGGATCTCTGACCATCACAAACATCACAAACACAGACTCTGGAGAATATCAACTACAGATCTTCAGTAGCAGAAGCAAGAGTGAAAAGAT
LG19	ref-4653	65.974			GGCTTTCAGATTATGACGCGTGTCGCTCTGTCTCTTATCTGTGGTCACATGATTTCGCATCAGCTTCTTGCTCTCTCGTCCTTCAGCTTGTCCCTTACTGCGCATGCGCATACAAATATTGCGTCTTGTTTTCTTTTAAATATTTGCATTGTTTTAAGGGTAGGTTTAGATTAGGGGTACGTATGGATGTTAATAAAGCCTCAAAGCACACGAATATGGTGCTGGTAGCACAATCTGATAGGTAGCATTTTTCGCTGTCGAGTGCTGCTGTTTTTATGGGAGGTTTAACTGGCGATTCACACCGCCAATTCAAGAACAGGAAAAACTAAATAAATAAATGCCGTACAAAAATACACAACACGGAACATCGTGACTTTTTATATTACTAAACAGGCCTATACTAAATTATTTAAAACTTCAACCAATACAAG
LG19	ref-72689	66.688			TGCTTAAAGACGAGGCGTTTGCCTTTTCATA
LG19	ref-10811_7	66.887	Chr9	52347809	CGCGCGCTGTGACTTTAATTTTGAAAAAAGACGGATAAGGAAATGTTCAATGGGTCTCAACGCGGGAAAACAATAAAAGTGAAAGTAAAGGTGCTAAAAATAAAACGATTTTTATTAACCATTTTTAGTGATGGTGGAAACAAAGCTTTTCAAAGCTTTGAATCAATTGAACCAATTACTTCGCAAAATGATTCACTGTTTCGAAT[C/T]GTTCGAATGTTTTGCCGCTGGGGACGATCTCAGTGGGTTCACAGAGATCGCCCCCCAGCGATGAACCATTGAATTTTTTTTATTTATTTATTTTATTGCAATTGCAAACAATTCAAAGATGACATTAATGAAATACAACTTTTTAAGAACATAAGGAAAGCCAGGGACAGAACAAAAACAAAACCATTGAAATTTCGAAACGTTTCTAAACAGCTGTGACGTAAC
LG19	ref-50494_30	67.208			AGACCCGGTCCGACTAAAATGCAACTTTA[C/T]CC
LG19	ref-43994	67.66	Chr22	7106984	AGCCAAATCAGATTATAGATTCAGCCAAATGTTGTAAACTAGTAACAGCTTGTTTGACCGTCCTGTTATTACTTAACATTCTGCATACTAAACCCAAGCCTCAGACACTATAGTGTATGCTGTAGTGATCTGGAAAGCTGCCTATATCAGCACCCAGGCTGAAGCCAAGTGTCCTCTGCAGAGAGTCTGCAGCTGTGGAAGAGACGATGACGACTCCCTCGC

					GCGATAAAAGAGATAATTCAGCCCTGACGATGGCAGACGAGCTTCAGTTTTATAGAAAGAATAGTGAACGAGAGGAGGAGAAAGAGAGAGAAATAAATAAATAAAATACTAGACAATGCAGCAGTGGGGGAGAAGGGAGTATAAATCATGGTCAAAGCCAGAAACAGAAAACATGAGCAAGCAGGAGAGAGAGACAGTGACTTTTCTCT
LG19	ref-38056	68.004			GTTTCCAGTGTTGAGGGTATCATATTGCATGTAACGTGAATTACATAATCAGATTACTTTTGTCAAGTAACCAGTAAAGTAACACATTACTTTCAAATTTAAAAGTAACTCAAGTAACTTTGTTTTCCTGTTTATTGACTGACATCTCTCCTGTCCCCATGTTAAGAGAAATCATAAGTGCAGAGGCGTTGTGTGTGCTGTGTAAACATAATGGTTATTTTAGTTCTGGACTAAATGTGAGAAGGCATTTTCCAGACCTACAAACTCACAAGAGGTGAAAAAGGTAACATGTTTACGAATTAAATTGTTGTAGGGGGGTCGGGGGGCATCGTCCCCCACAAGAAAATTTTTGTGATTTTAACATGTAAA
LG19	ref-3227_14	69.896			AATGCATACCTGCCGCGTCCAACATTAACTTGTTAATTTCGGGATGGATGGATGAATGGATAGATAGATAGATAGTTGTTGTTAGAAAATGCACAAGTTTAACTTTTATATTAACTTTTATTTACTTGCCAAAGCCAATATTTACTTGCATTTAGTGCTTGCTTTTAACTTTATTCCCTGCAT[C/T]CTCGTCGTTCCTCACTCTTTAAACATGTAAGTATACCCATTAGGATAAGCTTGTTAAGGACTTTGGATGGTTTCAAGTGGGTTCTGTGGTTTATCTGTGTGCGAGTGTGTGTAAAGAAGCTGTGGGCAGCCGTGGGGCAGTGCAGGGGCCACCTGCGTGCCAAGGCAACATAACCACTTACATAGC
LG19	ref-44749	71.681			TTTTTATTCTTTCCTCATACTAGAATACAATACTTAAGAATGTGCTTAATTTTGAAAAATTTAAATGATATATTATCACAAGTCTTTTCCACAAAAAACACCAGTCACATTTTCTTTTTTACAAAATGTAAAAATCGATTTAGTTTTATTATAAAGTTATTAACTGTGGACTAAAGAACCATCTGAGGCTCTTATCTGTCCCTTCACTGTTGATCCAAGTGCTTATTTTGAAGCAAAATGTGAAGGTTCGCTGTCAGTGTGCTTTATCTATTTTTTTCTAACAATCCTAACATGTCCGTCCTTTTTCACGCATCGAACCAATGACCGTTCAGATTGGAGCGACGCGGTTGCCACCGGACCAATCACAGTGCTCTGACTCGCTTCCTCAATTCATATTCATAGCCTTTCGCGCGCGCTTTGTGATCACGTGG
LG19	ref-70983_29	73.121	Chr22	37293753	TGTAAATGCTAGAATGAATAAATGAAATCAGTGGAAATAACAATCTTCTGCACCTGATATTGCTATCTAATCTCTCTCCACTGCTCCTTCAGTATGGCACAAACCTCATCACTGCTGATTCTGGACTTGTTTCAGTGTTGGATTAAAATGTTTATCAATTATCACTCCCATCAGGATGAGCACTCCATTCGCTCTCC[C/T]GCTAATTGAATCATTGAATAATTTCTGCTTCCTTAGGGGATGCAGTCGTTTGCAAACAGCGCCATTCTTTTTCCTCCAGAGCCTTCTCGATTGCCAGATATGTTTATTAATTTTCCCGACCCCTTGGTGTTTGGAGGCTACACAGAGAACTGTCACATACAGCCATGAGCCC
LG19	ref-46501_30	73.818			GCGCTATTGACGAGCCAACTGCAGAGGAC[C/T]GC
LG19	ref-20182	73.853			CCTCATGTGACGATGTAAATGCAAGTAATGA
LG19	ref-46501_31	74.453			GCGCTATTGACGAGCCAACTGCAGAGGACT[A/G]C
LG19	ref-20421_23	75.041			ATAAACCAACATCATAATCTTGTGTCAGATGGCCGTTATTTGCACCATAACATTCCTTTTTGCCTCATTTTCTACCGTCCACAGAAGTTTATCAGTCATTTTCCCCAATTAAAATCTCTTTACAGCTCCAAATATTTCAGAGAGCACCAAGATATTTCCAATTAAATATGAGTATATTAAGATAAATTAGATTTTGATCAGGACGGTGGCCGAGAGAGCTGC[A/G]ACTGAAGAAAACACATGCAAATAGAAAAAAACACCAGCAAATTAAGAAAATGTCTTCATCAGTTTGACAACATGTGCTGCAAATGATCACAACAACCAAATACATAAACACGCTGGAAATAGAACAGACGACAAAGGAAATGTTTCAAGCGGACCCCAGTGACAAACCTGGCTGGAACATACTTATTGTTCAATGTCTACTAAAAGGTG
LG19	ref-60513	75.639	Chr22	36372823	GCCTGAGTCTTGTACAGTACATATGATATATATGATGATATGATGAGCACAAGTAGTGTTGCATGGCCCACAACCAGACAGACAAATTATTCACTAGCTCCATAACATTTAAGCACCATACAGAGGCCCTACATTCATCTCAGCACTCGCTTAGTGTATTTCAGAACTTCATCAATCAAGCAAACGGCTCATGGATCGTGTGTACAAAAAATGCAGCCTTGTGGAGATGGATATTACCATAAAGTGTTTTTGTCAAGACAATATATTTCATTAATAATAAATTTTGCACTAAATTACTACAAAATATTCAGAAAGTACCATAAAATAAAATACATTTTGAAACTTCGGTAAGGTTTTTCGTTTTTTTTA
LG19	ref-3802	76.97			CTTCTGATGCCGATGCCGCTGCCGCGCAGAG
LG19	ref-20245	77.342			AGGCCAGGAACGAAACTTTTGCAGACATCAA
LG19	ref-31738	77.464			AAACAAGTTTTGAAATATATTTTATTCTGCCATCTCATATTTTTTCCACCACCATTAAGGGCTCCGTACTATCAGTGCTTTTGCATTCACTCACAAAAGTTTTTGAGAAAAAAAAAGTTTTGGGGCGAATCGTAAAACCATTGACATAATTTTTGCCACAAGAAACTTTGCTTTCACTAGCAAAGAACTCAAAGTTTCTCGGGGGAGCACAAACATTTCAAACCTTTAGTCCCTTTAGGGGCTCCGTACATTTTAGTATATTTATTAAATTTAACTGTATATAAACACAAGCTGGAAATGTTTTTGTTTTTTGTTTCAGAAACAAAAAAATCTAAAAACAAAATCACTTTTTCTACAGTTCTTTTATTA
LG19	ref-5431	78.634			GATGTTGTGTTTTTGTGTTGTAAAATGTGCCTGTATTCATAATATGACACAAGTATGCAGTGCATTGCAACAGGACCACAAAGTCCCGTTACTGTTTTGTTCGCAAAAAACTAACGAAAGTGGCATTTTGTTGAATGTTTAAAAGGCGAGGACGCTCGATTTTGTGTGTGTTTTTGTGTAGTAAAATGTGTCTGCATTCATAATACGATACGACAAGTATGCATTGGAACAGGAACTCAAAATGTTGTCTTTGTGATAGAGTAGCAAAAATGGTATTTCGTTGCACATTTTCAATGCGAGGATGCTTGATTTTTTTGTTTTTGCATAGTAAAATGTGCCTGCATTTGTAAAACGACACAAAAAGTATGCATGGCATCGTAACAGGAGCTCAAAGTCCCGTTACTGTTTTGTTCGCATTAAACTAGCGAAAA

LG19	ref-13029	79.32			ATAGTTCTTTAACTGACAAGCTCCCATAGAAATTTGTCCCTGCTGCAAACACAATGTGTTCTCATCTCATAAACTTGACCATTGTGGCTGCAACTGAAAACCGCATATAATTTCTTTCCTTTAGTGAACTCCTGATAAGATAAAGCAAGAGTAAATACTGTCATTTTAGACAATGATTGGCAGATTGCTCGGATATTGCTTCGGGATGTCTATGGAAAAGTGATTTCACATGGATCTGCATTGTTTTGCAACATCAGTCTCTCCTAACTCATTTCATTATCAGTGTTTATTTCAAGCACCCGGACTTGTAATTTACTAAAGAGAATGACAGCAGTCTGTCCATATCTGTGCCAAAAACCAAAGCAAATT
LG19	ref-10649	79.471			AAAACCACCCCGATAAACGTGCAAAGCGCCG
LG19	ref-4678_18	79.693			CTTTGCGATCCGATGGG[A/G]ATGCTGATTACTGT
LG19	ref-46752	80.184			TCAGATTGGTCAGATGACAAACTTCCTCTAATTTATAAAATTCTATTTAAGGTTGTTTCATGACTTCATCTGACGGCCACGATCACTGTCTCACATGCCTGGGCAGACAGCATGCTGAGAGAGCGTCTGTGGCCGTCAGATGGAATCTGCAAGAGCATGTCCATGTTGGCACTGCGGTCGTGACTCTCCTTCTTTAAAATGGAGGAGAGTCGAGGGGTTTGCCCAGCAGTTCTTGGCAGTCCAAAAGCAGATGGAGGCCATAAAACACATCTTGCCACACACTGCCGTGTCTTGAATTGGGCCCTTCACAAGCTCCTGTTGAAGATGTTCACGCAGAAACACATCCTCACGTGCACTGGCTACCAGGACATGTTTTGCAATGATTGACCTGAAGGACTTGTACATCTATGTCTCACTTTTACCTCGACAGA
LG19	ref-15633	81.095			CCTGGGACACCCCCTCGTCCTCCTTACGGCTCCGCAAGGCTCCCTCGTCCTGCCAACTCCACCTCGGTCAGTCGTCGCTCTCGCCACAAACTTCCGGGTCTTCAGCTGCACCTCGTCCCTCCATCTCTATGGATCTGTTGGGCTCCTCCTTCCCTCCGACTCCACCTCTGTCCTCTCTTGCACCACCATCGCTGCAGTCTCCCAGCACCCAGGCTCCACCTCGGTCTCCCAGATTCTCAGCGTCACTTGGTCTCTCCAGCTCTCCGCCTGGGTCTCCACTTCTTTTGGCTCCATCTCCATTGCTCGTCCCCATGGTGTCATTGAGGGCATCTCCACCCTGGCTCCTCCCTCCCTTGACTCCACCGTGGG
LG19	ref-30981_27	81.664			TCGTTGATTTCACATGGATTTCAGAGCAGTAAACAGGACTTCAACTTTGGGCCGTGGACAGTCACAGCTGTGAAGACTCATATCATGAAGTCTAAAGACATTGAGAGGTGAGTGCATGCATGTTTTGATGTTGAAGAAATTATATTTCCTATGATAATATGAGCAGTTGAGTCTGATTCTGACTGCGATTCTGTCACAGGTTGGCAGAGACGATGCATATGCCGGC[A/T]TTGCCAGAGATGCTGTTCGGTGATAATGTGTTGCGCATTCAGCACACTGATGGTTTTGGGATTGAATTCAATGCGATCGACGCTCTCAAACGTGTCAACAACATGCAAGACTCTGTGAAAGTGGCTTGTGCCCAAGAATGGCAGGAAAGCAGGTAGAGCTTTGTGTTATTCATTATAGTCTGATTAGATGCTTTCATTCAGGAGG
LG19	ref-50473	82.624			AGCGAGGAAGCGAGTTCAATGCTGGATTCAC
LG19	ref-47238	85.761	Chr22	38907325	GCCAAAGGGGAGTGAGGCTGACCAGTGAAAACATACATTCAAATCCTTAAACTCAGTTGCAGATTTAGCACTTCCTGGGTCTGACATTTCAAGACTGAAACCAAGAAAAACAACTTGGGGAAATTTCTCCCCTCTCTGAGGCTTCAGGTTCAACTTTATGAGAAAAAGGTAAAAATTGTGCTCTAACCTCGACAGCCTCAACAAGATTTTGGCCACTATCAGCAGCCTCGGTCTGGCTTAATTTTCTTATTCATTCTTCTGAAATGAACTTTCCACATACATAAACACTGAACGCAACCCCCCACAGGTTCACAGTTTAGCAGAGATATAAAAGTATGCACAGTATTTGAGAGAACATGCCAGCAGAAG
LG19	ref-53968	86.217			CGTATGTATAATGTTTGACCGAATAGGGTGCGCGAGGCTAAGTAAAACATGTTGACAGGCAGGTAAGACATCCTATCGTATCCCTCAGACAGAGGGTTAGGGTTAGTCTGTAAAAAAGGGTTAGGGTTAGTCTGTAAAAATACCCCTCAGATTTGTGTTAAAGCTATTTGTACAGTGTGCAAACACATTGGGGTGAAAAAGGCGTGGAAGATGCGAGACATTCCATAAACATAACCCGCCAAGTGATTTCACATCGTTACCCTTTACAGTTTTTTACCCACATTTGGTGGATGTTCATTTCAAACCCCATCATTATGAACAGAACAGAACATTTTGTGTGTAAAAGCAAACTGTTTAATTATGTTGT
LG19	ref-49901_30	86.97			TGTATCGTTTCGAAGGCTGTGCCCTCCTT[A/G]GG
LG19	ref-49901_28	87.491			TCATAACTACTGCCTCATCAGCAACTGATAAGCTGTGTCTCTTTTCAAAGGCTGTGCCCTCCAGAGGTCACATTTGTTAATTACTGTAATTTCTTTCTTATTTTGGAATGTAATGCTTACTTTTCTGAAATGAGGCATCCTTGTTGACATATGTGGCCGACAAATGCGACCCAAGGAGGGCACAGCCTTCGAAACG[G/T]TACAACGAAATAATGTTGGAATATAGCTGATATAAAACTGTACCAAGCTACAAGCCACTCATAATTTATAGCAGTTCAATGAAACATAGTACTCTTTTGTACTACTCTTTCTCATACTCTTTTGAAACATAGTTGGCTATATTACTAAGACTTAAAAGGATTTTTCACCCAAA
LG19	ref-11905	87.681	Chr23	42947764	GTGCTACTTATGAACCACTTTTCCTGGTTCAGAGCTGGTGCTTTGGGTGTCAAAAGACAAAGAACTGATTTGAAACTAGGCTCTGGCTCTGAACCAGTACTCAAACTGCCTTGGTGGAAAAGGTGTAATAGAGATGCATTCGCGTTTGTTCTTGTGTGACAGAATCTGTCATTTGAACTTGCACGCTTGAGTTTAAATGTGTGCTTTTTGCGATGTGGTCGCAGTGAATGGTTCCACCTGGGCTTGACTCTCTTTTAAGGGATGAGAGTTGAGCGGCTGCACCCCGCGGTTTGGGTCCCGCGGCTACAAATTCGTTGAGGGGGGATTCAGCCAGGGGCTAAGTTGCTAGGCTTGCTGTAAGCCTCCTTGGCTACTGCACTTAAATCAGAACGTTGCTAGCCTCCTCTCGTTATAGAGAGTTGTTCTGTTAG
LG19	ref-40666	87.872			AGCCCTGGCCCGAAATCACTGCTGCGGCAGT
LG19	ref-26543	88.487			TGCGGATACACGAAGATGTTGCTGGGACATG
LG19	ref-29941	88.597			CGCAATACCAGCTCTGAAATTCAGGCCCTGAATAGTAACATATTTCTGTCACAATCTCTTCAAGTTAAACTGAGTTCTTTGAGTTTCTGTGTGTATATGATATTTTTCTCAAATTAAATGGTTAAAATCTGGTCAAGTTAACTCTCAGAGCAGCTCTGGAGATGAAGTTCATGTCCTCATATGGATAGACGACAGATGCTGAAAACGCCACGAGTGTCATGCATTCTAAACTACACCGCTCATTCACACAGAGACAGGCAGGACTTGCAGATATTTTCACTTTAATATTCACAGACACTAGTTAATATTAAGATTTGGTGAAAAGTGTACAGTCC

					GAGTTTTAATTTAGCTATTCATCCAAAATTTCCAAATTCTGTGACATTCCTCATTACATAGCAAATTACATTTTTACAAATTGCATTTTGCCTAGA
LG19	ref-49345_32	89.476			TAAAACTCTTCTAATTTGACGCCATGTTTTATACACGTCAGAAATACATTGTTTAGGTAACTAAATAGTTTACTCTATTCTTCTCCTAGTTCACCGGCCACTTACTTGCTTGTCTTTCGGGATAAGTGGATGAATTTGCATGTTGTAAATCCAACAACTCTGATTCCCCGCCCTGAAGAGCAAACTATTCGGATGGCCGATC[C/T]CCCGTTATCATCTAACATGATCATTTTTTTAAAAGACAAAATACAATCACTAACACATATTTGAGAATCAGAATATCAGTTTTCAAAATATAGATAAAGAAGACAATATTGAAGACAATTTACAAAATTAAGACTCTTGATATATCCAAAATCAAGAAGTATACATTG
LG19	ref-36478_5	91.029	Chr10	5396356	CTGTCATTTTCTTCTAGAACGTACGAGAAGGGCTTCTGCCAGCCGTACCGAGGCATTGCATGTGCTCGCTTCATAGGAAACAGGAGTATCTACGTGGAGTCTCTGCAGATGCAGGGAGAAAGTGAAAACCGCATCACTGGTGAGCATTGCTTTCTTGAAACCAGCGCTGTAAA[C/T]ACTGAGCACAGTTATCGTGATAGCTCTGCACGATATTGGAAAAAACTGATTCGGCGATATATATTGCGAAGTGAAAAAATACAGGAAGTTTCACTAGATGACTTGAATAGCTCTATTTGGAAAGAATGAATCATTGTAGAATAATTCTTTTGAATTGGTTCATCTGTGTAAATTCAGTTATTTTGTCTTGTGGATT
LG19	ref-35087	93.287	Chr10	3711963	CTCGGTTTACAACATCCGTCTCATCAGCCTCATTTTCTCTAATGGCGTCGGCTCGTTTTAATGGCAGATATTCATTTGGTATCGTAACAACAGTGGCACCCTTGGGACTCAGCCGTAAACAGGGGTGATGGGAAAATCCTCGACATTTGTCAGAGCAGCGCAGTGTGTTTGACAATATATGAATTATTGATTAAACTGTTGAATTTTAAACGAGGGTTGTGTCGCGTTGAGGCCCCGCAACATAAATGCAAAAGTATGCATATTCTGGAAAAGATATTGACTGGGAAGTCTGTGTTTTACTTTTCAAGAATAAACCTGATTTGTTTGCTCTATATTGAACTATTCAAGTGAACACAAACCCACGCATCCGCTAAAAACAACAATAGTACAATCAGATCGACCCGATCGAAGGCTGTTTTTATGGCCTGATG
LG19	ref-40734	93.51			CACACTGTTGTACATCGGGAAGGGTTGCTGAACTGCCACACAGGCATTGATATATAAATGTGAGCAGTCATACGTTAATGAGCTGCTGGTCATGATAAGCCCATACGTGAGGGTCAGCGTACAGTGCGAGTGAAGTGAATTTTGTCATCAGAAGAGTATGCGCATACTGTACATGCACGGCCGGATTTTTGTAACCATGCGTACATTGCACGAGCGGGTTGCACATGCGCATTTAATTTTTTTGCAGTAAAGTGTCAACCGTGCTGTGTGGGTGTCGATTCACAATGTAGTCGAAGAAAAACTGCATAAATAGAACAGATTGGAACACATGCAAGCTACAGCAACAATGGAGACCAACATAGACCAGAGATTGTGCTAAGAGGTTAAAAAGTACTCTCAGTACCCTCTAGCATGAAAGAATACAAAGATGT
LG19	ref-67829	93.901			TTCCTCACCTCGATCGCGGTGCATTTATAGA
LG19	ref-1115	94.419			CATGGGATTCACTCATGTTTTTCATTAGATATGTATGTATGCTGTACCAGTTGGCACTAGAGGAAAGTTTTGTATGATTGACGTGATGAACTCCAGAATCTACACATCCTTAAACTCATCACTATGACATGTTCAGACACTAATGTTGTGGTTTTTATACGTCAGTAGAGGTGGATTGAAATTTCTTACTCCATAGAGTGTCGTGTGTGTTGATGTTTATGCTCAGTAAACATTTGTTCTGCGTTTGAATGGTTGAGATGTTTCAACACAGGAAACATAAGTGTTATATTCACATCTAAATGAGTGTGCTCAAATCTGCCCTCCGCAATATACAAGTTACACTTTACGTGTACGACTAACTAACTAACTAACCAATTGGTAAAATAGTGTAAGACCTTTTGAGGTGCCTTTTTCGGGTAATTGCCGTATTA
LG19	ref-4255_6	95.364			GAGGACTCCAGCGATGGTAATGGGACAAAGTGCAACGGGACGCTCCTATATGATCAGTAGCCTGGAAGGGGGAATGAAAAGGCGAAACCGCCATCCCTTACAACTTAGCCCTTTTGAGAGCAGTGCTGATGCCAACAACAGGGCACTCAGAAACAAAGTGTGATGATGACACTG[G/T]CCAAGCAAATGACTCGCTGCCTACACAGACTAGTGAAGGATGAAGGGTAACAAGGTCTGGTAGAGTGAGTAAAGCAGTTGAGAAACTGAATTTGTAATACTGTATATCTTTCAAGTAGTGTAAGAACTGTGGTTAACAGACTGTTCAAGTGTCAAGGTTAGTCGTTTAAGTAAAGTGAATAAAATGTTAGTAAAT
LG19	ref-30072_9	95.728	Chr10	3687540	TATGAGTCATTTGCATACTGTATTCATTGAATCATTCATAAAATCGTCTTCTTAATGAGTAATTTGTGTCGTTTGCAGTAAAACAATAGCACTGCTCTGGATTCCCAGCCCGAACTGTCCTCCTGTCCGCTCTGATCATTTGCAGTGTGTGTTCAGTGAACTCTGCTCAGGTTTGTT[A/G]AGCACATCTCTCGTCTCGTTCACTTTCCACTGAGAGGTTTGGCTGCAGTCGTGGCGGTGAGTTGATCAAAATGAACATGTCACCTAGACGAGCGGGCCTGACGGACGGCCTCTGACTGCGATTGGCTGTCAGGCCCGGCGAGTGGGCGGAGCCGTGATGTGATTGATGATTGGTGTGCCGGTGAAGAGCGGG
LG19	ref-28975	97.783			TCAGATGGTCCTGTACTTAGAAGAGATCTAACGGTCTTGTGCAATGTGTAAAGGTTATGTTTTCAGTTTAATATAAAACCTGGGTGTAACTTCGTTTAAGAAAAATAGTTTAATTTATATCTTGTGCATGCATGCCTGTTTGACATGAATCATTCTAATGTTGTATCCCACAATGCATTGCACTCCACTTGACCTTCTCTTTGCTGTTTGACAAGTGATATCACCTGTGATTTCTTCCTTCTGAGAACTTTATTTACAATCAAGTGGTCTAATCTAAATCCAAGACTGCTGAAGAGACACAATGGCTTTATACGATGAACAGATTTACAATGCGTATATACTGAAGTAAACACAGACATTCAAATGATT
LG19	ref-25558	99.091			GCCATATGTTCGACACCTGTGCTTTAGCAGA
LG20	ref-23192	0			ATAGCAGGTGCGACGCAAGTGCTTTTTTGCT
LG20	ref-13027	0.316			AGTCATGCCTATGACAAACAGATCTTCACTGGTGTAAGTGATGTACACACACCATGTACCGGCCTCCAAAGACGAAGCCTCTGTTCCCGATGGTGGCGCAGGCGTGAGCGGCCCGCGGCGTCGGAGCGGTTCCCTGTACGGACACACACAGCATTTGAGCGCATCGCACGGCTTCTGCCGCAACACCATCATAAATTACAGGTTAAGCACCTTTGTGATTGGCTGACTCCACGACGACGTGTCCAGATCCAGAATGTGTATGTGATTGTTCCAGCCTCGCCCTGCGTGGTTGCCCTGAAAAACATCACATACTGAGTGTCTCATCCATGTCACAAATGGCGTCTGCCGATGAACACGGACTCACCATGA
LG20	ref-69517	3.436			AGTTAGAAGTCGAATGTTGTGCAGGATGTTC

LG20	ref-43438	4.215			TGTCTGTGATCGACATCACTGCAACCTTTCA
LG20	ref-3050	5.819			CAAAACACAACCATTACTCATTAAAAGAATAGGTACAACCCTTTTGGACTATACGCCCGGCCATTCGCCGACCATTTTTCCGTCGTTAAACTAGCAGAAGAGGATTCGGAC ACGCCTTAAGTGCACTTTCACCTGCACTTCAGACCATGTGCTTGGATCGTTAAAAATAGGGCCCAAAGGCACAAAACTCGACTTTTGAATTTATGTGGACTTTAAAGGAAC ACCTAAAAAAAAATTCTGTCATTATATAGTCTTATTTCATATCAAATCTGCATGCTGTATTTGTTTGGAACACAAAAAAATATGTTAAAAGATTCCTCATGTACCATATTACA ATTGCACTGACCTGTAGGTTGTCAAACCTGAGTCC
LG20	ref-15775_5	7.989	Chr17	50691268	CTCCAGAAACCCCTGCTGGAGTAACCAACACCTCCTTAGTACATCAGACTGCTGGAGACTCTGGGTGGTCTTCCATGTCCCACCTCCCCGTAACAGACGCCCACTCTATATT TACATTATGTGAAGACAGTGAGGCGTCCTGAGGTGTTGTCACTCAACATTTATGAGGGTCT[C/G]AAATAGCAGGGTCATCGTTGGCGAAAGGGCCGGTGAAAACAAACGC AGCACTGTACAAATGTGGCAGCGGCAGCCAAACGTTTGCACACTGCGGATGCTACGCAGTCAGGCACAGAGCCCATCCAATTCATTAACAGCACGAGAGGACCTGAATGA AGACTGCAGACTGTCAAACGCCAGGCTTACACAGCAATCC
LG20	ref-12469	8.648			ATTGTGCCTTTAAACTTTCTATTCATCAAACAATCCTGAAAGTCATGTATCATGATTTCCAAATAAGAAATATGGCTTTTCACACTGCGCTTAACCCAGGGTTATCGTCGTTC TAAACGCTACTTTTAACCTTGGGCAAAGGAACGTTCCACACTTGTAATTTAGAAGCGGGGTTGGCAGCACCCCTTTCGCGGTGTTAACCCCGCATTGCGGTGCCAAACATC TACAGTGTGAAACAACGCGGTGATACAATGTCAGAGCCAGGCGCATTCTCATATTTGCCTGATTTGGGCAGTCACGAAAACACTTCGCATTGTACATAAACAGTCAATTCA GCGTTTGAGAGGAAAAATGTGAAATGCTGACAGA
LG20	ref-23363	9.924			CATTCTCACGCGAAACTATTGCATTCTCACA
LG20	ref-19574	11.987			GCAATATTACAATATTGCGTTACTCCCTTAAAAAGTAACTAATTGCATTACTAATGTGTTATGTTACTTTTGCGTTACTTTTTCTGGGCTGGTCTGTTTGTTTGTTTGTTTGTT TTTATAACAACAAGTTTCTATTTTTGGTAAATGTTATTTTTGGCCCTTTCACACTATGGGCCCTATCATACACCCAGCGCAATGCGGCGGCAGGTGCGACGCAAGTGCTTTT TTGCTAGTTTCAGCCTGACAGTTATGATTTTCACGTCCTGCGCCACATTTAAATAATACAATTTGTGCGCCCATGGGCGTACTAGTCTGAAAATGAGGTGTGTTCAGGCGCA TTGTTGGCACGTTGCTATTTTGAGGCAACTAAAATAGACTGCGCCTTTGACCAACTGAATGGCACAATATTTGTTTTGTTATTTAAAGAGTGC
LG20	ref-32307	17.917			AGACACCAGACGAGAAAAGTGCAGTGATGAT
LG20	ref-12928	18.267			GGTGAGAGCACGACACATATGCTCTTCTCGT
LG20	ref-30741	18.921			CTCTTCCACACGAGGCATTTGCCCATAACCA
LG20	ref-67781	20.651			AAATAAGAAGCGATTATGGTGCTGCAATGTG
LG20	ref-47606_32	22.35			CCTTCGTGGTCGAGGTCGATGCATCCACTAC[A/T]
LG20	ref-39889_14	22.659			ATTAATTACGATTGATCGCACAGCCCACAGCCCTAACTGAGCCAGCTGAGATTTCAGTATGGAGATAAAGTTTAGGATCACGATTTGTCAGTAGTTCTACAGCAGTGGTCT CCAACCCTGCTCCTGGAGAGCCACTGTCTTGCAGATTTCAGCTCCAACCCAACTCAAACACACCTGAACCAGCTAATCAAGGTGTTCAGAGCTAATTACCGAC[C/T]GTTGT GCTTGAGCAGGGTTGGAACTGAAGTCTGCAGGACAGTAGCTCTCCAGGAGCCGGGTTGGAGACCCCTGCTCTACAGCAAACCATTTAGCACCGTTTAGGGCTTTTCACAC TTGAACTAGTTAATCTTGGGTCATTCCAAACCCCGCATAAAAGCAGAGCTCTCCTAAGCAGGGTTTCATAACCCTGGGTAAAATGCGGTGCTTAACCCACT
LG20	ref-31576	23.098			CACTCTGCTAGTGACGTCATGAACTGTTTTCGGCCAATAAAATTGGTGTGTTTGTCACACATGCTTCAGATGCCAGTCACACTAATGGCATTACCCATAGCATCAATCCAAG ATGCAGTGTCTTGTTCCCCCATCTTAGGGAACCATGGTTACAATTGTAACCTGAGACATTTTGTTGAGCAAGACTGTTGCTTTATAAACTGTCGCTATAAAGTGTGTCGCTA TATACCACTAAATTAGTGGTATTTTTTGCAATTTTAACCATATAAAAAATTTATAGAAAGGTAAAAGTTTTCTTCATCACAAGTACAACAAAATCTGTACAAACCAAACTGT AAAGTCAACATCCGTAATAAAACCTAAACATGT
LG20	ref-16732_9	24.91			CGGGGGTT[A/C]ACGAGGGAATTGCAAGTTGATGA
LG20	ref-16732_10	24.927			ATGATCATTATAAAGTTGTTTAAACAACAAAACACATTCACTTCCACATAGGCTATTTGACAATCGGGATATAATTCAGGTTTGTGAATATTTTAAATAAAAAAAGGAAAA ACGAAATAAACGCGATACCGTTCATAGTGTGGCTGCATGACGCGTCGCCGAGTAATTTAAAATAATGTTTTAAAACAGAGTTTCCCAAACGGGGGTTC[A/G]CGAGGGAAT TGCAAGTTGATGACAAGCAATTAATTAAATTATAATTAAATCATTAAAAATCAAACAAATAGGCTAAATAATATTCAAATAAAAAAATCTAAATTTGGTAACAAAAAATA ATATTTTATTTCTTTATTCTGCATGTCATGTGACTATTCAGTTACAGACCTGAACGCGTCTGCTCAATTCAGTGGGCTGAACGAGAGTCACAGCACACATCAG
LG20	ref-38285	26.336			CATTGTTGCCCGAAAGCCTTGCATAGTACAC
LG20	ref-34982	26.808			AAGTTAGCGGCGATCCTCCTGCTTCAGCGTG

LG20	ref-23052_23	26.987	Chr15	44299009	TGATAGCAATCAATAATAGAAATGGTCTAAATATTTAAAAAAAAAAAAAATGTACCTTTATCTTCTGTGGAAGTCTCAGGACCAAAAAATGTTCGTCCAATCATTGTATTAGGTCTAAATGCAATGAAACATTGTCCTATCTGCCCGTCAACATATGCATTTCCATATCTCCGTCTTTGCGCACCCTGCTCGT[A/G]TAGACTATACATGTCATCAGAGCGTTAAAAGGGATACTTCACCCGGTAACACTTCAGTATAGGGAACATGTATTCACTATTAACTACGACTTTTCCCTCAATAAACTCCTAATTTACTGCTTATTAATAGTTAGGAAGTTAGTTGTTAAGTTTAGGTATTGGATAGGATTAAGAATG
LG20	ref-22381	27.327			ATTCATCTCTCGATTTGGGTGCTCTTTGCGC
LG20	ref-60846	27.595			CTATGCTTTCCATTAATTTTGTGAATTGGTAAGTTTTGTCAGGGTGCGAAGAAATATAGAACAGTGAAAACGCCATGCTTCCTAATGATATTTCCCAAGGGTAGCATGTACAGAGTGAAAAGCAACGGTCCTAGTACTGAGCCTTGCAGTACTCCAAACTGAACGTGTGATCGATATGACATCTCTTTGTTTACTGTTACAAACTGATAATGACCTGTATGCCTCTCCAGAAACCACCCACTGCCAGTTGTTTTATTCCCTGAACGGGAGGACACTCTGTACGGATGCACTGGCATATAGCTGGCCCCGGGGCCAGGAGCAGGTGGACGAGGAGCAGGCATTTTCGGTCGATGAATCGTGCCTAGAGTTCGGGCTGGGTGACGCCCATGTGGTACTGAGACCTCAGCCTGGCTACGTGCCCAAGGTTCCTA
LG20	ref-56627	27.682			AGCAAGGAATCATTTGCAGGAGAAATAGGCTTTTATTAATAATAAAACAAAACAAGAACAAACAAAAACTACCCCAAGGGGGAAAACAAGACTTGACTTAGACTTAGACTTAGACTTAGACTTAGACTTAGACTTGGACTCGACTCGACTCGACTTGACTTGACTTGACTTGACTTGACAGGGAAGCAAACACGAGGTGACATTCAACAACGATCCAGCTGCAAACACAAGGAGAATAAATAGAAG
LG20	ref-46139	27.799	Chr17	42797684	AAAATAATCTGTGTCCCTGACTCTTTAAGTCAACAAGATAAGAATTGCCTGCTCATACTGTTAAAAAAAAATCCAAGATGTACATTTTTTTCCATTTATTTATTTATTTATTTTCTTTGGATGTTCTGTTTACTCACTCAGATCTTAGTTGTGGGCGAAACAGCCCTGTGTGGGTTGAGAAAGTTCGCTCCGGCCCAAACAGAGTCACTTCGAGGCACTCGCTCACTGTGAGTCACTTTGTGGCAGATGTGATTGTGGCTATAATAAGTGCCATCAAAACATATTCCTCACCCTTGACCTTTACACCACTGAAAGAGAGAGGAACATACACAGTGTACATACACTAAGAGCCATTTCCTCTCCAGTTACTGTTTTCACAGTGAATTCTGTGAGCGCCCGTCACTCTACCGTTTATCGAGGAGAATACATAA
LG20	ref-16553	27.95			ATACCAGTCTCGAGTTTGATGCAGCTCGGTC
LG20	ref-47606_2	28.365			C[C/T]TTCGTGGTCGAGGTCGATGCATCCACTACA
LG20	ref-6384_23	28.798			CGTTGATCCACGACCATGGTGC[C/G]CTGTAGTCC
LG20	ref-52732	29.348			GTAAGTCTGACGAGACCAGTGCAGGAAGTGA
LG20	ref-13620	29.567			TGTTCTCACACGAAATATGTGCAATCTGCAC
LG20	ref-39794_27	30.046			CCCAACTAATCACAAGCACTTTTGGCATATTTTAAACTGGTAAGAGAGAGAGACTGAGAGAAAGTGGTGATGGACTTAACCTTATTGGTACAAATACAATACTCTTGTGGAGTTAAAACTACAAACTTAAAAGAAAATATTATACAGTATCAACATTAGTCTGTTTACAGTCACTGGTAAAAAGTCTGACACTCCAGTCCAAATCTAAAGTTCGACCTAAATGCTGCA[C/T]GTCCTGTGAGAAAAGTTTTCTTTTTTATAGTTTTTACTGCCATCTTTGCATCATAAAACTGTTTCAGAAGAGCAAGATCACACAACAAAAGTTGGATGATGTTGACAAATTTATACATTTTAATATATAAATTAAAATAAACTCCCTCCTTGAGGAACAAGTGATTATGCAGTGTAATATTGCACATCTCCTTGTAAAAGTTTAA
LG20	ref-47719_31	30.208			GGTTTCCCCTAAAATCCATCTCTACTTTTTTGTGCGTGTTCAGCTCCAGGTTGTTATGACTGAACCAGACTGCCAGCTGTTCAACCTCCTGCGCATATGCAGACAAGTCACCATTGTGTATGAGGCCGATGAAAGTAGTGTCGTCTGCAGACTTCAGGAGTTTGACAGAGAAGTCTTTAGCAGTGCAGTCGTTCGTGTC[C/T]AGTGAGAAGAGCAGCGGGGAAAGCACACATCCCTAGGGGGCACCAGTTCTGATTGTGTGGGTGTTAGATGTGAATTACTCCATTCTCACTAGCTGTTGAAAGCTGGTGATGTTGACTGCATCATCCACTGACTTGTTTGCTCGATAAGCAAACTGCAGGGGCTCCAGCAA
LG20	ref-10307	30.594			GTTAAACAGTTTATTTCTCAAATGTGCTGCAATGTTTTAATGCAGGAATTTTCATCTAGGCATTGAGACACAAACCAAGCCTGCACAAGTCCTGAAAAGTGAAGCAAAAAGCATCTCGATCGTCCCCGGGTGGTTGGTCCCAGTATAGGTCATAAACCCCGCCCTCTCCGTGTAATTAAGGGTGCGTACACATTTGTAGTTCGGTTCGTTTGGTCCGGACCAAAAAAGAAAACAAAAATTTAGTCCTGGTCCGATTAGCGTTCAGATTGGCAATTTTATCACCGAACCTAAAGGTATGGA
LG20	ref-25967	31.095	Chr17	13355787	TGTCACATTTTTTAGAAATCCAGAAACCTCCAACATTTTATGAATTATATATGGGCTGCATGTCTTTAAGCTTGCAGGTGGCTGGTGAAAAATGCAAGATGTTGTTATCTTCTGTATACTGTCTGTAGTGTCTCTGCTTCACGAGTCATGTTCTTGCTTTGCCAATTACGCAGATTTAATTAATGACTTTGCAATCACCTGCAATAGTGATGACCTCTGTGCCTTTGAATTGTCCAGTTTTGAGCAACTATACAAATGCTCCTCCATTCTTTTCTCTTTTTTCTTTTCTTTTGCTCACAAAATTCCCATCTTGCGCACTTATATCTGCACATGGCAAAGGTGGCAGGTAATTTTAGTCCTTCCCCTAGAGATTCTAATTGATTATCTCCCAGTCTGCTGATGAGCAGCATTTGCATTTAACACAAGCAGTT
LG20	ref-8550_3	32.076			TGGATGTTCAGGCCCATGACTCTGAGATCCTGTGTCTGGAGTACTCCAAACCTGAGACCGGTGAGTGCTGAGAACGCAGAGGGTGTTTTCTTTATTGGTCTGTAGCCGATTAAATCCTGCACTAAGGTGCTGCCTAATACCCCAGTAAACAGGAAGTGCTGATCCAGCTCA[C/G]ATGCTAAGCAGATGCTTCGTAACCTTGATATCACTTCACACATATGTCACCAGCAGCATTTAAAGATAGTGTTTACAGTTTAAACTTCACATCTCAAACTGTAACCTGTTTCAGCTTAAACTTGAAGTCATATTTTCTATGTTAATATACCGTCTCCTGTCCCAACTTGATGTGCAGTGACAACAAAAAACAAGTCAT

LG20	ref-58965_27	32.324	Chr17	10748152	TAAGAAATGGTCACTAACCTTTGACCTTTCTTGCAGCATTGAGCACAGTGCCACAGCTTTACACAAAGACAGGAGGTGAGTTGTATTGAATATAAATCAATCATTCATATTGTCCTTCAACGTAAGGATCAAAGGTCACGCTTCTAGTCTCAGGCGGTTTAAATACTCAAGCATTTATTGCACCCCTTGTTTATGGCGAGGTGAGACGGCCGACCAGAATGCTGAGT[C/T]AGTAGTAGAGATTGTTATGCTTGTGCAACCTGAGATTACACACACAGATCCTCATGTGGGAAAGGTCAGCGTGCTGAGTGTGTCTGAGAGAGTGTAGTGTCTTTGCTATTCCTCCTTGAAATATCGTCTCTTCCCCCAATGCGGCAGTCATTTGTCACCCTGGCACGCTGTCCCGATGTCCTGTTCATATTTATGTGCTGGCCA
LG20	ref-66711	32.8	Chr17	22547600	TCTGTTCAGAGCAGGTTTCAGCTTGTCTGAGGGAGAACTGCTAGGGCTGTTGGAGACATCTGAATCTCTCTCCTTTTCCTTCAAAGAGAATGAAAAAAGAAAAGAGATAATCATATTTTCTAGGGAAAATGTGAGTGGTGTTTGCCCATGCTAAACCTAGCGGGTGTTTGTGGGTGGTTGCAAGGGCGTCGCTTACTGCATAAGGCAAAAGAGACCATGACATTGTTGGGGTCCCTAGATATTAGAAATTTCTGATCAATACCGATAAATGAATTATTTACATGTTATGGCTGATATAAATAAATGACAGATAATAAATTCTATACTAATTATGTCTAATTAAATAACAAATAATAAAAATCAATAATG
LG20	ref-49480_31	33.567	Chr15	6584414	ATATCTTCTTAAATGATGACAGAATTTTCATTTTTGGGTGAACTATACCTTTAATGTACTAACACCAAAAATGTACTAATACATGATTAAAATCAAACGTTAACATTACAATCTGTTAACATTAGTTAATGAACCTAAACTAACCTGAGCTAGCAAAGAACAATTGTATTTTTATTCATTAATGTTAACAAAAATTAGTAAGTTCTGTAGCGAGTGTATTGCTCATTGTT[A/G]GTTAATATTAGTTGATGCATCAACAAACCGTAACTAACGTAACTTTTTGTTAAATATATTCATGAGATCATAGCCCCCCAGTGGTGAACCATTGAAATTTCGAAACGTTTCGAAACACGTATGACGTAATGAAGCCTCCTTTAATGAAATCATGTGACTTTGGCAGTTTGATACACGCTCCGAACCACTGATTCAAAACAA
LG20	ref-29539	33.839	Chr17	8042732	ATTGTGCATATAGAAGTACCTGCTTTACCATCCATTTTGGGTCCATAATGACAAACTCTGGTCGCAGTTTGCTAATGCGACGGCCCTTGAGAATAGGTACCAGACCTCCCAGGTGTTCCAGATAGAGGGTGTAACAGGGGCCTCCTGCCTCTGGGTTCTCCTCTGAACGTTTCATAGTGCAGTGCAGCCGTTCATTCCCTGCTGTGGGATAGCTCACAAAGTCTCTAATGTTGTTCGGATCAGGGATGGGGGGCTTGCGAGATGAAAAAGATTATGCATTAATGCATATATTAGCATACATCAAACTGTGATGATCACTTGCTGCTTCTGTACCTTGATAGTTGGAATGAATGCAGTGGTAACGTAGG
LG20	ref-19034	33.965	Chr17	8388467	GCAGAGCAGAAAAAGATGTGGATCTGAGATAGAAAACAAGACACTATTTTGATAAGCTTTGTTTTTCCATTGCTGGCATGTATTTTGATGAAAAATAAGGCATAGAAAATACAAAGAACTCGCTGACAGGTACATTTTTAGCACCACTAGTGTTTTGTTTTTAAGAGGGAGTGTCAAATTAAAAGTAGCTCAGGTTGGAAAAACACGTCTCGAGTCCCCTGCATTCAGTTCCTCTCTTGCTGTTTTTGAACTCCAGGATCTGTCATGTGTGAACGACCCCTAATTCTGCACATTTTTGTGTGCATTCAGATTGAAATAATTGTAATGGTGTGTTGTTTTTCTGTAAGACCCCCTGTAGTTTCTACAGAACTGCTCTTTTTGAGTTGGACTCAAATCTCTTTCACCCTCCCCAATTCTCTCCGTCTTCCTTA
LG20	ref-66826	34.943			AGGAAGCTTTATGCATTAAATTGGAAGCAGAGCGGCAGAATATCAAGAGTTATTAAATACCTGACCTAGAAGGGCCAGGATCATAATCTCTGCACAGAAAGAGAAAGTTCTATCTTTTATTCCCATGATGGAAAGAATCGTGTGCTTCTCTGCTTGGTTCACCTGTTTGGAGCCGTCTGGAGTAAAGAGCAGTTCTAAAACTAAACTGTTCGAACGTTTAGCGGCACCTGCTCTGATATATGACTGTGATTTTCTTCAATCTCTCAGACACGCCTCCTGTCAGTTTGCATGTTTCAAGGTAGATGGCATGGGATTTATTATAGTATTACAGTATTATTATATACTTTTATTATATATTATTGTCCCGTCACTTATCTGTCCTGTCACTTATCAAGGTGTTCATTGGCTATAGTCTATAGTCCTGGTTTTATGT
LG20	ref-43986	35.124			TAATTTTCAGTCAGGCCACAGTGAATATCTCTAGGCTTCTTTGTCTGTCCTCCATAGTATACTCGTCTTTGATTCTGTTTTTCATTATTGCATTTTTTTTTTTTTCATTCCTGCAATATTCCAGATTTTATTTTGTTCCATTTCCTGCAGGATTTAACCGTTTTAGCTTGAGGTTACATGGCAGGACCATCGCTACAACATGCGACACGCATTTTGATTTTATGCCAGCATAAAGATAATATAAAATCCAAATTTTGTTCCTTACAGTTTCTGGACTAAATAAACGACTCATTGGTGCATGTTATTTAAAACAAAACTACAAACAGAAATGTAACTTGTTCCACCTCCTATAGCATTCTGATGGATGAAA
LG20	ref-6719	35.414			GAGTTGATTTCCCTGAAAAGTGTTAATGCTTATAAAAGACACTTTAAAGAACCCTTCAAATAGAATTGGACACTGGTAAAAAACATCTACAATAATGCAATCTTTTCCATTTTAAATGTTGTTTGCAACATTCAAAGAATGTTCTGCACTCTCTGCAGTCAATACTTGACAAGGGAGAAGCATGCTTCTCGAAGCCAGATGGTGTAGTTACGGCCAGAGGGGGCTACAGGGCCATGATAACCATTCAGTGACAACGTAGGCTCAACAAATGACTGGTATTTGGGGCTGGACTATCTGTTTACCGACCAGTTGGTCAGGAGTATACAAAAGCATATTTTTTGTGACCCTACTGGTGATAAAATTACACAT
LG20	ref-6384_10	35.727			CGTTGATCC[A/G]CGACCATGGTGCGCTGTAGTCC
LG20	ref-7820_30	36.352			AAAAAGCAGTAGTGTCTGATTCTGTGAGTAAGCACATTAGTTACTGTATTTGAAAACCAAAGAGCGCCTCTTCATCTTAAAATGGTTTGTAATTGCGCTTCTCTGTGCAAGAATGCTGTCCCGACGAAGCCTTGTTTCCAGCTCTTCTTGCAATATAGCATTCATACAGGCTTGTGTTTGCATGTGTCTCGACACTAT[A/C]CAGCGCTGGGAGATCCAGAGAGAGTGCGTTTGAATTTGTCGCAGTATTAATACGAATGTATTAATAGTATATAATATATAATAGATACTATATAATTTAACAGCGGGAAAGATGGCTACATTTGTATAGTGTTTGCATGTCTCGACAGTTAATAAAGCCACAATAAGCTCA
LG20	ref-46520_5	36.621			ATATAAAATATAAATATAATCTACAGAGATGTTTACTTGCAGTGAGCTTTCGATGTTGTTATTGAAAGGCCAAAGCTGATCAATTTGTTCTTGTCACATGACCTGCGCTGTGCTTGCGACATTCTGAAAAGTTGAGAACTTTTCATCCTGCTGCACCAGGAAAAATCGAGCAT[C/T]GCACAGCATGTGCATCGTGACCACGTCACTTCTATTATGACTGCGTTTGCCACGTGCCTACATTTGAAATAACTTGACTGGTGTTTTGTTGTGTTCTATCCTCTGCTGTTCCACATTTGTCAATGCGTCATGTGTCAGGTCAGAGATTACTCTTTTGGCGTAAGTCGATTTGCATAGGCTATCTATCGAAAGCTAG

LG20	ref-10447_5	37.007	Chr12	43276256	TTGTGAACAATATTTGAGAGAAATAGGCCTTTTGTGTACATAGAAAAAGTCTTAGATCTTTGAGTTCAGCTTAAGAAAATGGGGGCAAAAACAAAAGTGTTGCTATTATAA<br>TTTTGTTCAGTGTAATTAGTCCTTTTGTCCCTAGGCAGTGAACATTTGGCTGGATAAATTAG[C/T]GGCAGCAGTTCATTCGTGAACGTAGAGAGGAGCAAGAGAAGCACC<br>CCAGAGCTCACCTTGCCGTCTTTGCCAGAAAAAATATATCTTAAATCGTAAGTGACACTATTTGCTAACTTTTTACATTAAAAAAACTGCTGATACAGTGGAGGCCGGGGC<br>TAGTTGTTACATAAAGAGGTTGTCCCAAGGGTGTATATA
LG20	ref-53617	37.445	Chr17	29511728	CTTTAGGCTGTAAAATAATGAAGGTACCACTGGAAGTTCTTTGGTAAATGACCTGTAACCTTTATTTAGGAGCAAATGATCTTCTAATGTTTTTAATTATACTGATAGGTGG<br>GAGCGAGAGAAGTCCAGGCATGTTACGGCAGGCCAGTTGTTTAGATTCTGGTGTGTAAGGCATAAAAGAACTCAGACCGCAGTGTGTGTATGCGTATGTGAGAGGTTTGC<br>TGAGCTTTCATCTGTTTCCTGTGCTATCAGTGCATCAGTACATTAACTGTGTGAGAGAAGGACAGCTTAATGACTATTGATCATAGCACAGAGGTCAAGATATCAACATTTT<br>GGAAAAGGCAAATTTTTCTGCAGCTCGTGAGGTTTCCTTTAGAGTACAGAGGGGAAAATATTCAATCAATCAGTTCTGTTTTTTATCTCCATGAGC
LG20	ref-22293	37.665			GCTTGTGCCTCGAATACAATGCCAAAAACGA
LG20	ref-61616_3	38.598			CA[A/G]TCCCTCTCGAGAGGTTTGCATTGATGTAT
LG20	ref-26341	38.893	Chr14	22073083	AAGCTGTTTTCCTCATGGTAAACAAAAATGGTCCCCACAAGGACAAGGATTTTGGATATTGCCATCTTTGTGGAAAGATTTTGTCTATATAACATACCTGAAAGGTATACTT<br>GGAATTTTTCCTCTGCATTTAACCCATTAAATGGGGAGCAATTGGGGGTTAGGTGCCTTGCTCAAGGGCACCTCAGTCGGTATTGAGAATTGAACCCGCAACCTTTGAGTT<br>ACAAGTCAGACTCTATAACCATTAGGTCACAACCGCCTAACCGCAATGCACACAGCTTCCAAACTGAAGCAAAGTTTTCTCAATCCCATTTGCAAGTCAAATGGCCAAACT<br>ATCTTTATCAGCTCAAGCTCAGCAGCAACGCCAAA
LG20	ref-8730_3	39.138			ACATCTCATTTCAAAATGTTCTACTTCATTAAGTTCCAAGGCAGCTCCCTAAGCATAGGGCAACTCAGTGAGTTTATACATGTCCATTGTCTTGTCTTAGGTATCTCTCTGTG<br>GCAAGCAGGGCGGGGCTGAGAGCCGTTGGCATGGAGCGAGGCCGGTGACACTCATTAG[G/T]GATCGCAGCACTGGCCTCGCTCTGTTCCTCGCAGGCCGGGGGGTACCCT<br>TGAGACTGCTCTACAACCCCCGCCCCCCATCTCCCCTCTGGGGGGGCCAGTTACGGCCCCCGCAGCACCTGGGGGTAAGGACAGATGAGGCCAGAGACAGACTACTGAAG<br>GGTTGTGCGTTAATAAAGTTTTAATTTTGTGATGTGTAT
LG20	ref-11273	39.706			GAAGGTGGCCTGGTCTGATGAATCATGTTTTCTTTTACATCACGTGATAGATGGCCCGGTGCGTGTACGTCGCTTACCTGGGGAACACATGGCACCAGGATGCACTATGGG<br>AAGAAGGCAAGCCGGCGGAGGCAGTGTGATGCTTTGGGCAATGTTCTGCTGGGAATCCTTGGGTCCTGACATCCACGTCGATGTTACTTTGACACGTACCACCTACCTAAG<br>CATATTGCATGTACACCCTTTCATGGAAATGGTATTCCTTTGTGACTGTGGCCTCTTTCAAATGGTTCAGGAATGGTTTGAGGAGCACAACAACTTGGCCTCCAAATTCCCC<br>AGATCTCAATCCAATCAAGCATCTGTGGGATGTAC
LG20	ref-22669	40.302			ACATCTGTGCAGCCTCAGTGGCACTTTGTAGTGACGCGATGACTGAATCTTGTCATCAGGTGAATGTTGGTGCCTCAGAAAGCCCTACAGCTGTAACCTTTCCTACACATAT<br>TTCGCGCTGGGGGTCTCACCCACCACAGCTACAATCCAAAACATTGGCTCCTGTGCCCTTTCGTAGTGCACTACTGTCGACATCCAAATTTAGAGATTCAGCTCTGCAGTTT<br>GCACGCTACTGCCACAGAAAAACCCCTGAATTTGATTTGAAAATACCACATACAGATGCACAATCATCCAGCTTTGATGGAACCTCTGATCAGTTGACCCACACTGATGCA<br>CAGGGTAAAGCCTCCATGGTCAATGTGGGCTTTA
LG20	ref-54867	42.486			TATCTGACATTTTATGTCTCTACCATTTTTCTGTTTTTACATTAAAGGCGTTTTACATTATTTTAAGGTGGGGCAGAATTAAATGAATGTTAAAACTCAGCATTTTGTTCGCA<br>TTCCCTCTCTGTCACCTCACATAGGCTACTAGAGCAACCACTAATTGGCTGGTCCATTTGGCAGATGCTTCAGATTCGAGGTGTCTGAGAAAAAATGTCGTTTGTGCACAGA<br>TTTTCTGTAAGAGGTCTAAATCTTACATCTGCACCTTTGACAGGCAGCTAGACAGTGGCCATTAGCAGCACTAGGGAAAGCATGACCATTATACAGTACATCCCATAAATA<br>CATCTATTTGTCAGAGATATGTATGCCTCACAA
LG20	ref-62342_28	43.428			TAGGCATATTCGAACGCAGTGCTGAAA[A/G]CTAG
LG20	ref-62342_15	43.987			GTATGAATGTAAACTGGATGTATACATCCTCCATGTTGGCACTGTCATGTGACCTAAGGCATCAGTTGCGTTGCTTACCTGGCATTCATGACTCCTCTCCTATAAAATAATA<br>TACACTACAGAATCAGTAGTAGGTCATCTGGGTACTTTTCGTCTACTGTTTTATGAATACTACTGTTTTTGCATACTATTTAGAGAAGTAGGCATATTCGAA[C/T]GCAGTGC<br>TGAAAACTAGTTATAAAGGAAACAGGAACATGAGAGACAGGAAACAATGAACAAGGCAACAGGAACTAGGAACAAGAACTTCAACATGAAAGCACATAAAATAATAAA<br>ATAAAAGACCTAATTCAAAAACACGAAGCCAGAACAAAACTAAACATAAGTGACAGTAAGCTAGACTGTTCCTTGAGTGTTTGTGTTAATAATAAATATGTA
LG20	ref-67318	43.996			TATCTTCAGACTCCTTCTCTTATTAAGGCTTGTTTGTGAAATGACGCTTGACGAGTTAAAACTAAGCTTATATTTTTGTTGTTGTTGGAACCTAACTGCTCACTGTGCCCAGA<br>GTGCAACGTGAGTTTACAAACCTAGCTCCGCCTTCGCGGCTGACTTAAATGGCTGTTACTAGGGGAACAGCCTAATCGTCACCTGCTTGACGATGCAAGATCCGCCTTAGA<br>TTTGCATATCGCCTTCTGATTGGTGCAGAGTTAAACCCTTGCGAATGTAAACGTGACTCTAAAATGTGGTGTGAATTTTTCACACTGTGTGAAATCTGCACTGCAGCGTCAG<br>GAGTGAATCTAGCACGCGCTGCTGCGTCATCAG

LG20	ref-8081_25	44.331			GTTTTGAACATGAAATTAATGCATTTTCAGAAAATCATATGATGGTTTAGTCTGTTCAGTTATGAACAACAAAACCGTACTCAGGAATCCTCTCCAAATTTAAAATGTCTTAACTTCTTTAAAGAGGCATTCAGTAGTTCTTTAGCTAGCATTGAGTTCTTTAAGGATACCACAACGCTGCACATCTATCGAC[C/G]TGGTTCATTATGATTGCAATTTTTATGTAAAATTACACTAGCTATACAATGTGCATTTTGGGATATCACATAAAAAACGCTGGATGGAAATGCCAAAATGGATGTGCCAAAATTCTAAAAAATGCTCATAAAAATGCTTAAATGAATCGAAAACATTATTTTATCCGATAAGACAT
LG20	ref-34936	46.81			GAGATCCAGCCGATTGAACTGCTTTTTAATC
LG20	ref-12288	47.687			TGGGTCACTATAGGATGTTAACCTTTTTGATTTTAAGCCACTCTATAGTGACTTCAGCTCTATGTGGCTGGTCTGCTGGAAGATGAATTGTTTCATGCGCTCTGTGCTCTTGGGACTGCAACACTATTTCCTGAAGGTTTTTTTCTGCATCCATCAATTTTTCCTTCTATCTCAATGAATTTTCCAGTCCCTGTTGTAAACAAGCATCCCCGAACACAATGCTGCTTCACCTTGTTTCAAATGTAACTTATATGTCCTTTCCATTCATGTCAGATTTATGAATAGTGCATGCAATTCAATTTCTGTAGATGGCTTCCTATTTAAAAATCTGTGTTAAACAGCCTTTTTTTCTGACATTTTCTTACAAACTTTTCCTGGTTTTATTGTTCCTTAATTGTTCCTGTAGTGTGCCATGGGACTGCTTGAGATAT
LG20	ref-57341	48.983			GGGAGGGGTGCGAGGGGTGTGCCGTTAGTTC
LG20	ref-53370_6	49.444			TGCATTTTAGTCTGGGACTAGGCTTAAGCTTTGTCTGTGAAACTGGGATAAATGTAATAAAATCAGAGTAATTTTAGCATTTTGGTGATTCCCTAGTTATGTTTTTGTTATTGGCAATTTAATCAGGCTACTTGTCTGATTGGGCAAGTAAAATTCTCTTTCACTTGCCCCTTC[A/G]AAAAGCATTAATGTCGAGCCCTGTTATTCCATATATATTGTCTTCAGATTTGGATCAGACTTTAGCAAAAAAAAAAAAAAGGTATGGCTGAATTTTACTGTTTTTTTTTTTTGTTTTTTTTTTTTAACAATTCCTCGTGTGCAATGGCAAAACTGAAAATTATTTTAATATGCAAAACTGTTACCCAATCATTAGA
LG20	ref-21778_4	49.76	Chr17	32905893	GTTAGGCCGGCTTTAGCCACATCTGCGCTCCAGACTGGCCCAGCCATTTCCTCTGCAAATTAACGGCTTTATAATTTATCAAAAATGAAGATGCATGTGGGGCTAAATCAATGGGCCCCCACAAGGTCAATTAAGAGGTTCACCCAGCGCTTGCCTACCCCCCTTCGCTATA[C/T]AGCATGGCACCGTCATCGCAGCTCGCACTAAATACGGTACCTCACAGTGTCTGTTATGTAAAACAGTAAGCACCGGAACAGTCGCAGCAATAAACGTTGTACAACTGTAAATGTATGAAACTATAAAATTGACTGTCTGTGCTATTTGCAATGTTGAGCCTCTCTAATAATAGTGTGTATGTAGATTGGCGTGTTCC
LG20	ref-39077	51.371			CATGAATCATGGGATAGTACAGTGTTCATCATTTGCACACTTCAGAATCTCGTCGGAAGAAGTATTTTATCCAACTCTTTTATGAAGAGTATTTATTTACACTGTGAACTCGGACATACTTCTTTTGTCACTTTTGGTTTTTCGCCTACTATATAGTAGGGAAGTATGCGATTTCAGATGTAGCCATCTCGTGAAGTTCTCTAAGCACTTCCTCTTGGATGAATCCCCGGCGCCATTTTGAAGAGCGTTCAACTTTGTCAAGTGGGCGAGGGAAGTTTATTTGGACAAACCCTCGATTTTGACAGAAGGAGCAAGTCTACTCAATATGATGGCTGCATCCAAAAGCTTGAAAAAGCTGCCTTCGGAGGA
LG20	ref-43214	51.543			TAAAACAATTCGATTAACCTGCCAACAACCT
LG20	ref-27081	52.047			AAAAAAGTTTTGCAAATATTAATAAGCATATAATATAAATACATTATAATCATAAATATCAGTTGGCGCCAATAGCCTTGTGGGCAGCGCATCGACATTTAGCACAGTTGCGCTTTAGACACCCTTACTGACAATCGTAATTTTGGCTGCGTGAGATTCTCCAGCTTTGTTGTTGTTGAGCAACTGTATCGCGAGCTCTTAAAGCTCCGCCCTCTTCTGGAAAGCTCATTTGCATTTAAAGGGACACACAAAAAAATGGCGTGTTTACACACAAATAGGCTCAAATTTGACAAACTATAATAAATGATCAGAGACTTATATTACATCTTGTAAAAGGGGCATTATAGATCCCCTTTCAAGAATTTGAAG
LG20	ref-18733_2	53.278	Chr17	33826634	ATTGAAGTTGCACGTATTTGAATGCCTGTGTATTTTACCAGGATTGGCCCTGCTGCAGATGCTCAAGTTGCTTAGCAACAGCTTTTAGTGGTCTTCAGGAAATGTTGGCTGAGGGGATTGTTTGCTTGACAGTGCTTATAGGGTCTTTCTTCACTAAATACCAATAAAGT[A/G]AGCTCATGGCATCACTGTCGAGAACTCTCGCATGTTCTTATTCATATGGCAAGGGTGATGGGGCTTTTCATGAAATCTGACATTACTGTACTGTGAGCTTACAATGAAAATAGTACTTAAGTAATTTCCTGTCTTATCTCAATAGAGCGTTGTGGCAGAAAACACATTACATAATGGGTTGATAAGATTAACCAAAGAT
LG20	ref-59851_28	58.497	Chr20	21312319	CCGTGATTTCTTGAAAAAATGTAATATTGAACTTGCGCATCCTCACTCACAATGAACTCTCACGCACAAAGAAAATGACAGCATAGGCTAGGCACAACACGTTAATCAAACAGCCGCTGTATTTTACTGCGCAGCATTGGTCAATCTATCAATTAGAGTAACAAATCAACAATGTACATGCACTGAACTCGTAACGC[C/T]CCATGACCCCAAAACTGAGGTACGTACCGAACCATGAACTTCTGTGTACCATTACACCCCTAGAAAACAATATAGAGATGGCTCAAAAGAAGAAGGAGCTCAGAGATGTACCTGCCCAACACTCTCACAGGAGTAGGAGGAGGTGCGCTGCACCTTGTGCTCGGGCTCTGAG
LG20	ref-32470	58.65	Chr17	35667551	AATTAATTCTCTGACAAGCATCACTGATTTGCTAAATCAGTGAGAATGGCATGCGCTGATCAAAAGAGAAACAAGAGGAGAGATGTTTGAACACATGGACTCAGAGATGTCATTCACTGGGCTCTCACAACTGGCCTTGAGTCGCAGTGGCAGATTCAAGGACGTGAGCGGAATATACATTCAATTCAAACACAAACGACATATGCCGACCAATACACATGCCTAATGCACAGCAGCGCACACTATGCCTCAAAGACATGCTCAAACATACATGCACACATGCACATAGATTAGATTCCAAGACATTTATAATGCACATGCAGATAGATATTAATTCATGGGCAAAAACTGGGTTTGTTAAAATAACATCCTTTACAAATGTGCTTCCTCTATTGAATGAGTGATATACTGTATGCACAAGGAGAATTAAC

LG20	ref-55961	60.627			GATGTAAATTCATTGTTAGTTAATGTTAGTTAAAGGGGCTATATGTAAATATTTTAATGTATTTATCTTTTTTTTTTACTGCCAATATTTGAACAACTTGTCATGAAAATTTAAAAACGACACCTTCCAGTTTCACAACTTCCTAGGTTGTGTATAAAAGCCTGTAGACTGATTTTTATGCAAAGGACTCGGGTCGTTTTTGCTGGAAAATTCAAAAGGATGTGACGTTTATGAGTCACTTTTCCAATCTATGACAGTTTTCCGCGGAATTGGGCTACTTTAACACTGTTGCCACAGGTTGTTTTTATGTCCGCGGTTTGAAGCTAGCCCAAATAACATATTTAGCCCCTGTAATGCGAATTTTACCAG
LG20	ref-26676_17	60.81			GCAACTGATACGAGTG[C/T]TGTGCTAAAGCTGGG
LG20	ref-26676_23	60.81			GCAACTGATACGAGTGCTGTGC[G/T]AAAGCTGGG
LG20	ref-14477	62.143			TTTTCTTGTGGTTTTCACATCTCCGGCCTCCCACTGCGTCTGAGTCTTTTCCCTTTTTTGCCTGTCCACATCTTGCTCTGTCTTTCACTTGCTATGGTGTCAAACGTCTAACTGACAGCTGTCAGCAAGATGATGAAGTTTGCAGGAGGAGCGTTGCTCAGCATTTATCTGCAATGAAAAGTGCGCCACATGCCCACACAACAAGACCTGCGAGGCACATGCATCTCTAAAGAGCCGCTCGGCTTTGAATTAGTGTCAGAGCAGTCAAGTGTATTAAAATGCTGGGGTTGTTCTCAGGTGAAGATAACATTTGATTTTAGTGCTTTTAAGCTGAAAGTGGTATTCTTTTCCCATTCTTTTGTTTAGATTCTACTCCAGTATTCAGCAGTTAGCAAATACATTGAGATTCAAAGGTTTGAAACCCCCAGTAA
LG20	ref-59851_8	62.332			TGGTGCG[A/T]TACGAGTTCAGTGCATGTACATTG
LG20	ref-4311_23	62.952			CCCTCCTGTTTGAACCGCCCGCTCTAAGCGGGACTCGAAACTGGGACCACCGGGATGTACTGGCCTACGTCCGTTACACTCACCCCCCTAAACCTCACTCCCATCCAGGTCACGGCACCACTGTAGCCCCTCCTGTTCGAACCACCCGCTCTAAGCGGGACTCGAACCCGGGTCCGCCAGCATGGGAGTCG[C/T]GCGCTACGTCCTATGGCCTACATCATTACACTCAGCCCAGTTTGGGAACCACTGCTTTAGGTAGTTTTTTCCCCCTTTTTGTTAGCTGTTAATCACTTTATGTGTTCCCATTTGATACCGTAATGGAGATCCTTAATGTTTTGTGCCAAAAAAAACTTTATTTTATTACTTGTAGTG
LG20	ref-65907	63.797			TGAGAGTGCACGATCTGCATGCTACTGTCTG
LG20	ref-48970_6	63.85	Chr17	3966262	CATCCGGTCTATGACAGATCTCATTTCCTGTCCCAGAGGACAGACGGTAACCATATCCACTTGACCTCTGCCATCACTGTCTGAAGATATGACACCATTGTTCTCGCCATATTCAGTCATTTACCATTGTAAAAGACAATGACTGTGGGTATTAGAAAGGATTATGGATTTTTTTTTGTCCACCATGATTGTGTTTTCTTCTTTTC[C/T]AGTCGAGCATTGTGCCTGGTGCTGTCGAGCTAATAAACGCCATTTAAATATTCATGCAATGTTCATTACAGTGAACTCTAATGTCACTCCACAGCAGTCTACTGAACTCGAACAATCACGAGAAGTGAATAAACAAATTGAACCAGGGTCGTGACCTGCGGTGTATGCAACAGGTTTGGATATAAACATCAGGTCCTGTTTCAGGTGAGCTACAGGTACATCACA
LG20	ref-4187_29	64.867			TATAGTTTTTAAAATACATTGTATTCAGTAACAAGTGAAACAGAAAGTACAATAATAACAAGTCAGCAGTGCATTCGCTGTTA[G/T]AACTGCAATAAAACATTAAAAACAATCAATGTAAATAGAGCTATCGCACATTGACATATCTAAATATTTCACGTTTAAATTCTGTTCTTGTTTTCCAGGAAGTGATCGAAGCTATAGCGGAGTGCGCTTTCAAAACGTCTCCTTTCCCTGTCATCCTGTCCTTTGAGAATCA
LG20	ref-65072	65.747			AACGAACGTTTGTGCTGGTTTGGTTTGGAGATATTGAACATTATTTTTTAGTTTGTGTGACGTAACTTTCCATCCATGTTGTTCAAATCACTCCAAACCGGTCCCATTCGTTTGTGCTCAATTTGTGCCCGTTTGTGCCGTTAAATTAACACTGTACCCACTTCCCCATCTTAAGCCTAAAGTATACTTTTTTACACGTACGCTATCGCTCGACACATGTGCTTTTGAAAGTTTCATCTTTGTCCAGTATCTGTACTATTTTTCTCCAAAAGTTTTGACCGATAACAATGTCATTGTATTCTTTTAAACTAAAGTTGTGGCTCTCTCATAACCCCCTCACACAAGCGTTTGTCAATGTGGGGGTCTGTTGTTGTTGCAGTCTCCGGTATTTTTTTCACTTTAATTTAGTTTTCTACTGTGAAACAATGGCC
LG20	ref-7587	66.463	Chr25	4118087	CTATTGACTCGCAACATAGCACACTGAGGACATCTGCTGGTTAAAGCCGTGTAACTGCAACAAGAACAGCAAAAAACATGTTGTACACACTTTGAAGCCGCAGCGCGAGCTTACAATAAACAGACCGTTATCGTTCGTTGGTGCTGAACGCAAATATCTTTTGACGCATCGCGTCCACGGCATAGTTATCCTTCTTGGTGTGAACGGGCCTTAAAGGGACAGTTCACCCAAAAATGAAAATTCTGTCATCATTTACTCACCCTCGAGTTGTTCCAAGCCTGTACACATTTCTTTCTTCTGCTGAACATAAAGGAAGATATTTTGAAGAATACTAAACTCAGGCTTTATTTTCTTAAAGAAAAATATAAT
LG20	ref-73411	66.666			TGATATATGCCGATGCCGATGCCAATACCGA
LG20	ref-19573_27	67.359			GGTCGCTGACCGACAGTCCTGCATTA[C/T]CATAG
LG20	ref-1129_17	68.195			TAATAATTAAACAATAAACTTTTATTAAAGTGCTTATTAATAAATTTTCACCTTTTGATTATTTATTTTTGTCTCTAGTAATTATGTGCCTGACTTTTAATTTCCAACCTATTTTGGGTTTATTTAAGCCACTCATATAGTGATTTTTAAACAATAGTTGGGTTAAATAAAACTGCCCAGCAACTC[A/G]ATCGCTGGGTTTGTCCATTTTCAACCCAACTTGGATTGTTTTTAACCCAGCATTTTTTAGAGTGTGGATATCATAAATATTTTGTATTTGAACATGTGCAGTGACTGATTATTATTAACATCAAAGTTTTAGATACATTGTATGTATGTTTAGAAGAAAAATATGACTCGTCATATTTCTTTT
LG20	ref-58745	68.258			TGTTTGTGGACTCTGCTAACGGCGCATCCAACATCTCACAGCTTTTGGCCACATGTCCTTTATCACAGCGCATGTGGAGAGAGATTTGAGCCTGTTTGTGTTGTAAAGTCAAGCGCTGGAACGGTCCAGCACATGTGAGAGATGCCAGTAATCATGAGGAACGACTCTCTGCTTCATTAGCGCTGATGTCGATCCCTCACACTGGGAACGCGGGTCTCATGCAGAACTGAACGCTAATAAGCAAAATCAAGGGGATTTTCACAGTTTTTATCTTCAGTTCTACAATGTGAACGCAGACTAACCAATCAAAACAAAATTTCATAATACTGTACGACACAGCATGTGATACTGACCTGCGACACGTCATAG

LG20	ref-1750	68.459			GTTAACATCCCGACATCCATGCTGCTGAGAG
LG20	ref-67003_3	68.741			TTCCTAGATAAACTTTGCATTCATTCGCAAAACTTTTGTTTCCTAGAGAGCGTTAGTTTGCAAAACTTTTATGTTTCCCAGAGAAACTTTGTGTTAGTTCGCAAAACTTTTGT<br>TTCCTAGAAAAACTATGTGATGGAAGTAATATGAGGTCAGAGAAAAAACTTTATATTAATGCTTCTGCATGTGAATTCAAAGTTTCTCA[A/G]GAGAACACGATACTTTTGC<br>AAGAGAACACAAATGTTTTACGTGTGAATGCAAAGTTCCTCGGGGGAACAGTACTTATGTGAGAGCACGTAGAAGTTTTGAGAGTGAATGCGTGAGCATTTTTTTGATCAC<br>CATGACCACTTAGTGGCTCCGTAGTAATTGTTGGTCACTGTCAGTGAGGACAGACGTGTTATTTGGCACTGGAAACCTGCTGCATCATGGCTGGTCAGG
LG20	ref-57612	71.001			CTTCAAGCACCGAACCCCTTGCATAAACGCA
LG20	ref-66982	71.209	Chr14	34082897	TATGCCGAAAATGTGTAAGTTTCCATTTACTAAAACGAGACTAAAATGCTCAGACATTTAGTCGACTAAAACTTGACTAAACAAAATCAGGACAAGGTTGGCTAAACATG<br>ATCAAAACTAAGGGCGCTATTTTAACGATCTAAGCACATGGTCTGAAGCGCAGGTGCACTTTTAACAATGCACACACCTCGTTTTCAGACCAGCACGCCCATGGGTGAACA<br>GATGGATGCAAGTGCATTTGATATTTAAACAACATGGCGCAGGACATGAAAATGATAACCGTGTCAGGCTGAATCTAGCAAAAAACACTTGTATCACGTCTGGTGCCACA<br>TTGCGGCAGGTGTATGATAGGACCCTCAAAGTTACATT
LG20	ref-19020_32	71.315			CTTTTCAAACTGACCATGACCTGTTTCGCAGAATGTCTAGCTGATGAAAGGATGTCAGATATATTGGGACTGAGACCTTGGCTGCAGTGGAACACAACATATAACAGATGA<br>ATGAACCAATGTGAACAACACGTTTCATCACCAAAGAATGTGTCCCTGCATCTGAGTCATACACGACACCTTCAGTAACACTGCTCAGATTGTGATGCACGACATCTGTGC<br>AGGTACAAC[A/G]ATATTACCTAGAGCTTTTTTATTTTCAGTAGAATTGTTACTTCGTATTTTGTATTTTATATATTGAGCCAGCTTCAACATAAACCTTAGAGACATGACAT<br>TTTGACAAGTAGGTCTGGCCCAGTGAATGCACCATTGAGCTGTATTCCTCTATTAAATTTTGATGAAGTCATATATATGATAATAATAGAAATGAGCCTT
LG20	ref-42031_30	72.025			AAGTGCTCCACGAAGCGCTTGCCCAGGAA[C/T]GG
LG20	ref-52717	75.247			TGACACAATGCGATACATATGCCACAATTCA
LG20	ref-56742_27	77.965			AACTTTTGATGGTTTCGAATATTTAGCTTCCCAATCATTACGAGCAGTGTTGAATCTTGGCATAAATACTATTCCAACAGGATTCATTTTCTAAACGTAGTTTGAATTACAAT<br>TTTTAAATAAAATAGGATGCGTTCCATTTCATAAACTAATACCGGTGAGTTTGAGATCACTGTCCTTTTGCCAAGTTGTGAGTGTTGGAAGATACAACGATCCCATTGCTCT<br>GT[G/T]TGTTTGCATCATGAAATCCATTGAAATAGGCTTTTTTTGTTTTATTTTGTTTGCCAATGGGCTATAATTAAACATCTGGATGCTTCCTTGTTTAATATTCTTTCATAG<br>AAATGGCTAGTTTTACATAGTTTTGCTAGCTTTGTATATTAACAACTGTCAGAATTAACCTCTTAAACTCTTGCTGCATATTTTGGATTTCGATTA
LG20	ref-22317	78.929			TCGCTGACACTGCATTTACAACTCCGCATGCACCGCCTCTTATGTGCTTTATCTTATCCGTTACTGGGAAGATTCTGTAAGTAAATGTAGGCAAAAGCTATTTCTTGTATTAG<br>CTTTTTGAGAACATAAACACGGTACATCAGCAGAAAGGGGCTGATTTATGCGTTTAAATGTATTGCGCAAATGTTTCGTACAGTCTTGCTGCTTACATGCCCATTAAAAATC<br>ACTGTTTGTTTGTTATTCAGTATATCGCATCCACTATCATTCACACCGGTATTACAGTGCAGTTGTACTCACTGCAGAGTCAAATGACGTCCAGCTCCTCCTCTCTGGACGTC<br>TAATGGGGTGGAGAGATAGAGTTGTGGGTAG
LG20	ref-37064	87.099			CCCCCCTTGGCGACGGGCCTGCATTGTCATC
LG20	ref-1857_18	87.799			TAAACAGAATCGAAATG[C/T]GTGCATCGTATCGA
LG20	ref-39243_18	89.856			GACGGACTGGAGCCGAAAGTTAGATTCTCTGAGTTTGGGTTTCTGGAATTATGTTTTGAAAGAATCATCTGAACGATTCATTTGTCTGGTTCATCCATGGTTCCTACACCCT<br>GATAAGCATGTTGGTGAGGTTAGGCCCTTGAAGTAACTGACCATTCAAAGAAAATCCTTGGTAAGATGCATTAC[A/G]GTCGAAGACAACACAGATTTTCTTCTTCTTCTGA<br>TGATAAACCCCGTGGTGAGGAATGTACCACACCTTTCCATCACCACGGTGCAAATTCTCGTCAGGTATTTTCTCAGCATAACCTTTCTCAATGATGTTGTTCATAAATCTGG<br>TGTAGTCCTCATGGAACTCAGGATTCCTTTTGAGTCT
LG20	ref-73459	90.321			CGCATTAAAACGAAGCTGCTGCAATCATTTG
LG20	ref-27147	90.34			ATAATTGGGGCGACCACATTGCAAAACAGAA
LG20	ref-35379	91.968			ACCAAACATGCGATAAAGCTGCCTCAAAACT
LG20	ref-42331	99.26	Chr17	103865	TTGATCATAGTTTTATCACATGACTGGTTTGAAGGGAAGTGGGTACGGCTAATAAAGTTTTGAAGCCATATAATCAGACTTTTTCTTTAGTTGAGTGGGTGTGTTTTGTATT<br>CCAGATGTGGTAAATGGAGCTCTGAATAGGTTTAAAACAGGTAGAACCCCAACAAATACCCCTCAGTCTGATGGAGCAGGTGGGTAAATGTTCTCATCAGACTTCACTGC<br>AGCTTGAGTTTCTGATCTGATCTTCACATCCTTAATGAACATGCTTCTTTGAGTTTGAGATGGCTTTGAGTGTGCCCACTTTCGTGCGTCATGTCTCCACTTGTTCTTGTTTTT<br>CTCGTGACACGGCATGGATAAATACAGTTTCTCTTTCGGCGCTTACGAGAATGTTTCTTCTACTTTTGTTTAAATGATTTCTGCTCTATTCTCAG
LG21	ref-18237_4	0			GTG[A/G]AGTGTGCGATAAAGCTGCATTATGCCTG
LG21	ref-18237_8	0.745			GTGGAGT[C/G]TGCGATAAAGCTGCATTATGCCTG
LG21	ref-25453	1.004			TACGCAAGAACGAAACAAATGCAAAAGAATG

LG21	ref-40085	1.873			CCATTATTGACGAGATAATTGCAAACTATCG
LG21	ref-19590	3.376	Chr25	23376009	AGGTCTTGTTGACGAACAAATGACTTAAAATGCAAAGCGACCTGCTGTGATGACTGCAAGGAAAAATACATGCTCGTGTTAAGATACAAAGTCATGGTAGCACACCCACAACGGAAAAGGACAGTATTTCTTTGTTTTGACAAGTACGTAAAAAGGATAAAGTGTGAATCACATTTTTACAGAATTCTGAGAAAACATTTGTGGTGTTTTCGAAGAATTTGTGAATGCATGGCTCAAGAAAATTGAAGTAGTCCTATAATTTATGATAATAAGACATCACATGTAAGAAGTTACAGGTTACCTTGGAGTGTTGTAATCCATTTAAATCTGTTGCAGATTTAGTATAAACAAACCAAGGGTACGTTTACACGACAACGATGCACAGATGACACTTCTGTCAAAACGATCCGCAAAAATGGCTAAAAACGCTG
LG21	ref-62163_5	4.162			ACAGAAACATTGTTTTCATTTTATTATTCTGTTTTTCTAATTAATTTTAATTAATAAAGCTTTTTCCCTTTTGAGTTAAGCTATGGTTGTGTGTGTCCCTTTTTGTTGCTTCCCTCTGAGCCAAGGGTTGTAACAGCACAATTAGTTCGTGGACAACTTCTGTGGCCCTAACA[A/C]TGCAGGCAGAAAGTTCGTGAATGGCCTCTGTGGCCATAGCATGACAAGCAGAGAGTTCGGGAATGGCCTCCGTGGCCATGACATGAAAGTTATTATTAATTTTTGTAATTCTTTTTATTTTATTTGATGGTATTGTACAACACTGAGGTCAAGTATGTTGTTTTATTATGTTGTTTATTTATAAATAAATTTGGAT
LG21	ref-40377_5	4.722			NNNNNNNNNNNNNNNNNNNNNCTCATGTGCCTTGAGCAGGGAGAAAGCTCCCTCGAGAAACATTTAGAAAAATTTATGGATCTTGCCCATCAGACCACCTTTCGGACTCAATGCCGCCACATGTGCACAGCTGTCCAGGGAGGGTCCCCGAGGGAGCTTCGCTTCCTATGTGGAGTGGGTGCTGGCATCCTGCGGATCAGCATT[C/T]ATCGTCGAGGTTGCTGCCAGCCCCACTCCCCATCCAGTGCCCAGCCAGAACCATCCAGACGTCGAGGATGAACAACCTGAGGCCACCGCAGGTAGTGCGATGGAGACCGGCGCGACTGATGTGCCCACTAGAGCGATCGGGGGTTACATCGCCATGGAGCCTGAGCAGCATGCACCTGACCAGGTGTGTGAGCCGGCCGAGCCATCCATCGCCGAGGGAGAGTTGGT
LG21	ref-2445	5.46			TGGGATGATGCGACCCCTTTGCTTTGCCAAG
LG21	ref-35873	6.236			ATAATAGAGGCTATTTACACTAACTCAGCCCACCACTGTCTGGTTGGGCCAGACAAAAGTACAAAAAAAAACACCTGTGACCTGAAGTAAGGCGCGTTGCAGATACTTTTGACGACCCGAGTTCGAATCCTCCTTTAGCAGAACTCATTCTTCTCCCTTTTCACATCGCAAATCAGATTGGAAAGGCATCGATTTTCAATAAATATTCAGACAATATGTGAAAAGTGGAATTAAAGTGTCTAACGGTTATTTAATAATAAAGTGTTTATTGGTAGGGTTAGGGGTAAGTGTAGGAAAGGCTTTATTGTCCCAATAATGCTGCATATTTTTAAAACAACACTCAATATGTTATTATTGCATACTGTTTTA
LG21	ref-37999	6.318			ATGAAGCAGGCGATGACGCTGCTGAACAGCT
LG21	ref-47416	7.306			TTGCTTTGAACGAACTTTATGCGGCTATTAG
LG21	ref-66546_2	7.801			AGCAGAAGCTGATGCCTGGTTATGGACAGCTAGCAGAGCGGAAAGTGTTCTGTATGCCTGGCATCCTGCTCTCTCGCCAACTGCCCCTCCAAATAGTTAGTTGGAGGCAATGGTGTAGACATGATTATGTCGGGTATAGAGCCTGGTAGATGTGAGATGAGGTGGAAAGA[A/G]TCACGAGAGCAACCCACTCGTTCATTTGTCACCCTCCCTAATTGATCAGACTGGGGATTAAAGCAGGGGTATACTGCCTTGTGGATATCTTACAAGTATAGAGCTTGGCAGGTGAGAGCTGAAGTGGACCGAGTTGCAAGAGGTGTTACCTTGCACACTCCCTTTGGTAGGGCAGCACCTTAAGATCTTTACAGGTAAG
LG21	ref-54498_3	8.058			CT[C/T]GACACGACGATCCATTTGCTCAGATGGAA
LG21	ref-18996	8.495			TGCCGTCAAAAAGATGAAGAACAGAAAGGCCCAAGGACCCGATGACATTCCCACCAAGGCGTGGAAGCTACTTGGATGCTGAGGCGCTAAGGTCCTCGCTCTACTGTCCAACAAGATCATCGACAAGGGCACCACTCCCTCAGCGTACGCTGCTAGCATTACAGTCCTGATCTAGAAAGGCAAAAGCATCGTTGGCAAATGCAACACCTGCCGACCGATCCGCCTGCCGTGCCATGAAGATATTCGAGTGTGTAATTGATGCCCATCTCTGGAAGATCATCTCCATCAGCCCCAATTAGTGTGGATTTGTTAGAGGAAGCGGGACTACTGATGCTATTCACGCCATGAGGCTGCTGCTGGAGAGGCACC
LG21	ref-73419_32	8.704			GGGATGCGCACGACAGTGGTGCCGCTTTCTG[A/C]
LG21	ref-55014	9.261			ATATACATCATGAGTCTCACCTGGATCAAGGGAGAGCATCATGTCATATTCAGCTGAGTGGTGTCAGAAGTGAGACTGCTCTTCCTCAATGAACGCAGCTTGTGGGGGCAGGACAACATATACAGAGATCCAGCATAATAAACAGAAAGCATATGTCATGTTCCGCTGAGCATAAGTGAGCATTGAATTCGCCTTTAAAGTAAGCGTATACCGTTCTCAATGAACGCAGCTTGCAGGGGCTGGCAAGCGACGCGCTGAGAGGCTTGACATTTTAAGAAAACACCAATAAAATATGCTCTTGCGTTCTCATTGTGAGAACGCTCATTCTCAATGTACGCAGCTTGCGGGACGAAACCGGAAAGCGACGCG
LG21	ref-28577_23	9.526			ATGGCCTTTAAAGACATCTCCATTAAAGACATCTGTGAGGTGGCCGGCTGGTCCTCACTGTCCACGTTTGTCAGGTTCTATAACTTGGACGTTCCGGCGTTGCACGCCCGGGTCCTTTCAGTGTGATTTGACGGCCTCTACGTAGCCCGTCGGTCGTGCTGTTATCTCATGGGAGCCAGCTCCCTCTTAGAATGTGTAACCATGAGGGTACGACGGCTTTGC[A/C]TACCTACTTCGGTGCCTAAGATAGGTTCAGTCATTCCCTCCCATGGCACAGTGTGATTGTATTCATTCCCCATATGCAGTATGAGTGAAGTATCGAAAGGGAATGTACTCGGTTACATATGTAACCTCGGTTCCCTGAGATACGGGAATGAGTACTGCGTTTGCTGCCGTGCCATGGAGCTGTACGACTCAGTGTCGTCGCTTCAATCA
LG21	ref-28577_25	9.655			CATGAGGGTACGACGGCTTTGCAT[A/T]CCTACTT
LG21	ref-47593	9.968			ATCCCTGAATCGAAGGTTTTGCCGCACAAAA

LG21	ref-53397_29	10.51	TTCATTTGCGAGTGATATTTTTGCTGAGGTCGCCATTGGTGAGGTTATATTTGCGTGTTGCAGTAGCCTATCACATCATTTGCTGTGTTGATGTAATTAAACAAGATGCTGTGTGTGAAAGTTTCACTGAGTTAGAGCGGCGAAATATGAACAGGTAGGGAAAAATTTCACACACCAAACAGACACCAAAGAACTAGAGCTGATGAAAGCCGACTGTGTTGCCGCCTC[A/G]CATCGCGATACTCCCTAATGTGATCATTATTAATTTCATTAAATAAATGTGAGCGCTGCATGTTTTAAAGTCAACAGATGGCGCTGTCGCGCGTTCTTCAGAGCAGCTCGCATTCAATGATTAGCACACATTTGTCAACCGATTGCTTATGAACTAATGCTGATCAGTTATCACAAAGTTTACTTTATATTATTTATATTATA
LG21	ref-61786	10.623	TGTCGACTTGTGACTGTACACAGTGAATCAGGATTTACTTTGTGTATTTATTTGATATGACACTGTGTTAAAGGTACATGGAAAAGGCATCTGTGGAACATCAACATGGGCTGCAGCCAGGAAGACCTCTAAGAAGACAAACACCAAATGTGATGAAGAAGGTCTAGAGGTGGCAGTTTGCAGACACTGCGTATTGCTTAGAGGTCTGAACATGTTTAAGGGGTAGATATTTGCGTACCCTCTGATTTTGCAAAACGAGCTGGCCACAAAAAACAAAACTGCAAGTTCTTCTGCAATGACATCATGTGTCAATATTGGCCCTATCTGCAAAAGGTGGCTCAAGCATTCCCAGAAATGAAAAACTTGACT
LG21	ref-19712	10.79	ACCTTAGATCTCGTAGGTGTCTGATCCACTACGAGAAGAGAGAACTGTCTGACTTGCCTGCACTTTTTTCATTCCTCTCCTCACTGCAGCTGCCCACTCTTGCCTAGGTCTCAGGCGGGGCGAGGCGCATCTCAAATGAGCCTAATTTCTCCTCGAGAGGCTGTGCGGGCCTCAACAGTGCAACAGAGTCGTTCATTTTGGTGATTCCCTAGTTATTTATTGTTTTTGACGTCTTAGTCAGGCTTCTTGTCTGTTCGAGCAAGTAAAATTCACTTTCATTGTCTTCAGTATTTATTCAATAAACATTGAATAGCAGTATTAGCAGTACTGGAGGGAGGGCAATCCACACAAAAAATGGGTCGCATCATC
LG21	ref-41680_26	11.239	TACTTACAAGCGATGCTGTTGCAAG[A/G]TTAAAG
LG21	ref-41680_30	11.239	TACTTACAAGCGATGCTGTTGCAAGGTTA[A/C]AG
LG21	ref-54696	11.487	GTCTTCTGCCTCTCTGGGCCAGACACCCAGAACTTGGTCAATCCGCTTTTGTCTCTCTGGAGTCGTTACTATCTCGTTGGACGAAAACTGAATGTAGTGTTTGTTAATGTCTCTTTTCTTCACAGGCTGGAGGCCCAGAACGTGGTTGTATCTGTTGCTGCCTCACAAGCTGTAGACTCCGATCATGGGATCTGTAGTTGTCTCTCTGGACGAGCCAGAGGCTCAGAATGTTACAATGTATCAGCTTCACATGCTAGGACTCAGAACGGCATATCTGTTATGTCTCACCCGATGGGAGACTCTGAGTGTCTGTTGAAAGGGTACCTTTATCCCTTTCCGATGAGGAGGAGATTGCAATTTGGCGCTTCTCCATTCAAGTGCTGTGATTGGCTGCTGGCATCACTTTCCAATCCACGCGTGCTGTCCGAGGA
LG21	ref-70309_30	11.578	TCATCCCCTCATGTTGGCTGACTTTTTGAGCTCACACAACCAGTAGAGTACAAATAACTTTATTGCGATAACTTTGCAATTACCAGATGTTTTTAGTTTTGTGCAACAAATATTTTATTTCCAGAAATAAAAACATTCATTTTCTCCATTGGAAAAATTGATTTTGAAAGGTAACTTAGAAACCTTTAAAGACAGACCTACTGTGAGCTACGATGTTGTTGCGGGCTGA[C/T]GGCTTCAGCAGTAGCAAATACCATCGCTTATTTCAGATCATTTTTAAAATAATTGTGTTTAACAGTGAATTTCCTGGTGGAAACACTACATTACGTGCTGAAATTACACCAATCAGAGTAAAAAAAAAAAAAAGCAACATGCTCCATAATAGGTCTTTAGATCCACCCACTCCAATGAAGCACTGTCATTGTATGACGTAATT
LG21	ref-15435	11.595	GTTTCCCCGTCGACCATCGTGCAGCTCTTCT
LG21	ref-73419_28	11.979	CCAGACAGTAAATACTCAGTAAAGTTAGCGTCAGGGTGGTAAGACAATTTGGCAGACAGACGAGAAATATTCACAGGAGTCGACAAGTAATTTTTTTAATTTTTATTTTGTGATCTTTTAACAGGGCATATGATGCGGGAATGAGCCCCTCCACAGTAATTGCAGATGTTCAGAAAGCGGCACCACTGTCGTGCGCAT[C/T]CCCACGAGTTGAAGTTAATGCAAACTTGATCGCGGGAGTTGAACGTGATTTTCTTAAAATCCTTTGTTGAAGCGTCGAAATTTGCTGGTCGTGGGACGTAACTAGTCAATCTTTCCTGTGATGGAACTGGGCAAGGGGAATATATGATATGGTGGAGTGAGCGAGTGATCTG
LG21	ref-19546_1	12.125	TTGTATTGCTTCCTAGACAACTGCAATAAATTACAATAAATTAAACAATGAGCATCTCTTCTCATCCTGGATTATTCATCTCTCTTCCACCCTCTTTGATGTTTGTTTTTTCAGTACCCGATGTTCAGGAGGTCTGATGAGAGGGGGCTTATAATAGAGATGTGCATGT[A/C]CAGTAGGCTGCATCACGGTCGTCCAAACACTTTAATATCAGAGACGTGATAAACAACACATAAACACAGATGCATTCCAGAACATGACTGTGAGAAACTGAGAATTTGTGAGACAGTTTCCATGTATTATTGAGGACTTCAATCCATATTTTCATTCCCTCATCCTGTTTATGTGTTTTTCACTTCTGCAATTGCATGTA
LG21	ref-51093	12.451	TGATAACATTCGAAGCGGGTGCAGTTACTAG
LG21	ref-11467_8	12.526	CACAGGATCCCAACCTCTCGCCGCCCATTGGCTTGTATGGGAGGCCATACCAGGAGTGTCAGATAGGGTCCTGAACACGATCAAATGAGGCTACTCACTCTAGTTTCTCAGACCCCCCTGAGACAACCCCATCCCTTTCCCCCTGAGACAGAAGGGGATGCGGATCCTGAACTACCTTGATGATTGGCTTGTTATGGCCCAGTCGAG[C/T]GACGACCTATGTGCACACAGGTCTCTGCTCCTCAGACACTTAGAATCAACCTGACAAAGAGCTTACTTTCACCCAGCCAGTGAATATCCTTGTTGTGTGCAGTATTAGACTCTACTTAGATGTGAGCCTGGCTGTTGGAGCGCTTGCTGAAAATTCAGGTACTCGTGAAATCTTTCAAAGCCAGGGGGCCTTTGTCCCCTCAAGGTGTTTGGGAGATTGCTGGG
LG21	ref-64041	12.783	TGACGGGTCCCGAAGCAGGTGCGTATATATG
LG21	ref-14713_30	13.233	GAATGACAACTGACCAAGGGGGAACTGCTGGGATGTGGTAACCCAGTGGAGCCGAAAGGCCGAGGGTTTCTGGTAGAGCTGAAGGTGGGAGGAGCCAAGGTGGAGCTGACAGGTTGACAGGCCTAGACGGAGCAAGACGATCTGAGGCTGGAGGCAGAGCCACTGGATCTATCCACTCAGGCACTGATAGTGATTGTAGGACCTGAGGCTCGACATGACTGCTTGGCAT[C/T]GTAGGAGATGGTGAAGGGCTGACAACCTCCAGCAGAGACGGGGCTGGAGGACTGGCTGAGCAGGGCGGTGATGGAGGCAGATGAAGGCTAGACAGGATTGAAGGTAACACAACATTGTCCATGCAGGTGTTTGTGCTGTCTAAAATCAGCGATGAAGGTTCAGAAGGAACAAATAGAAGTTCAAACAATCCATGGTGCTGTC

LG21	ref-58175	13.485			TTTGTCTCAGCCTGTTGTCCTACAACTCAATCCGAAGTGGAATAATTCCACTTTTGAGTGGCGATGATGGCTTTTAGAAGTGTTTGCCTACTATGAGTACGGCTAAGCTTCCATCTAAGCTTCCTTTTTTACATGGCTTTCTGTAATGCGGCTTCTGCCTTGCACACTATGACGATTTTGCAAGCCTATCAAGCTCATTTCAAGAGGGACTTGAATGAAGGCACAGCAGTGGATGCCGAGGCTGTTGGTGCTGTAGACTTGGCACTGAGGGCTACCAAGCGCGTAGCTTGAAGAATGATCGTCGATCCTGTTCACTGCCTTGTTGGTTCGTACTGAAATTTGTACAGGAGCATTCTTGGAGCAGATGCA
LG21	ref-34492	13.684			TACATCTACACGAACCAATTGCCGTGCAGTT
LG21	ref-55123_7	13.794			TTCCCC[A/G]GTCCGAAGCCTCTGCAGCAGCAGCA
LG21	ref-49051	14.592			TCGGTCTTAAAGGGACAGTAGCCTAATTAAATATTTGTCTGTCATTAATGTTAATAAAACAACAAAAGATGTTAAATAAATGTATACATGGTAAATAAACCTACTGTATCTGAAAAACAAGTTTATTTAATTTGTATCTTGTGAAGTAATGTTTGTTTCATTAAAGGTATCTTCATTGTGCATTCTGCTCTTTTTGGATCGTTTATACATGCTAAGTGTGACAATATCCTATATTTCTCATTCATTCTCTTATCATGTGCTGATATAACACTGTTTAAAGAAATATACGTATTTCATGAGATGTGAAAACTACTTCATTCCTCCAAAGCACAAGAGTATATGATTTAAATAGAGAGAGTCACTTTGC
LG21	ref-10754	14.633			TGTTAACATGTTGCTAAGCTAAATACTTGAGACTCAAATACACATAATACAATACATAGCACACACATTTGGTAAAATATTATGAACCTACTCTTTTTACACACTTCGGCTCTGCTTGATCTGCTATCTTGGATTGGGTTTTTCCACCGATCTTGTCTCAGTGATTTCTGGGATTTTGGGCATCATGTGCGTAGCAAAGTGGTGTGGAAGTTATTATTAAACAATTCTAATATTTTGGGTTTGCCAATTATATCTGTATGTCATATCCGTTGTGAAAATAAACAATTCTAACACTTTGGGTATGCCAGTTATATCTGTATGTCATATCCGTTGTGAAAAAGAAGTGTCCTCAGTAGTATATTTTTTAAA
LG21	ref-72476	16.129			CCATACTCCACGAGAGGCCTGCTTTAAGGCC
LG21	ref-21483	17.025	Chr17	22664820	AACGTGCAAAGACTTAAAGTCAAAACGGCCTTTACAAAAAAAAGGTAAAACAACAATGTCGGATGATTTTGAAGTAGGAGAAAATGAGATTGAGTTTTTAGCCCTACCGCAGTACTTCCGCCTATGTCACGTGTGATCTTTCCAACATGATTATGTCATGTGTGGAGCAGTGCATACGCGCATACGCATCGCAGAGCAGTGCAAGACGAGTATTGAGATTAGTGTGGTCAAAAAGTATATCAATTTTAATATTTTTTTTTAGAAAATGACTGATCATTTTTCTAGATAAGACCCTTATTCCTCGGCTGGGATTGTGTAGAGCCCTTTGAAGCTCCATTGAAACTGCAATTTGGACCTTCAACCCATTGG
LG21	ref-68044_23	19.569			AGCTGGACATCGATGTTGGTGC[C/T]GTGAAGGAC
LG21	ref-48774	19.977			CTGTTGTTTCTTAGCCCCAGGCAGATCTATGCTTATGTGTTAGCCTTATAGGCCCGCAGCTATAGTTGGCCTGGGCTAGAACTAGGGTTTAGGCGATCTGCTAGTAGCATTTTACGCCCTTTTTATACTACCCCTGTTATGTGAGTGTAGTTTTTCCCTAGTTCTGGCCTCCTCTTATCAGAGTTGTCAGGCCAAGGCCGATTGTCCCTTCGGTGTAGGTGCAAAATAGATAGCAGCTAAGGTAGCAGACTATTGCTAAGTCCCCCTGGTGCCGTTGTCACAGGCGGTGTAGGCCCCTTTTCACTATGTGAATAGAAACTTTTTTGCTGTGGCCCTGCTCCCCCAGAACTATCTAGTGGTACTTTGACTTCAGTTTTTCCCCTGAATCCCTTGAACTATGTTCAAGCTTGAGTGATATAGTTGTAGCTTT
LG21	ref-26681	21.906	Chr5	13494938	GCCTCAACAAGTCATGTGACTAAAGCATTGGCTCAGATGATTGGCTACTACTGAACACCCCTTGCAACCACATATCAATGCCATAGCAACTAAGCAGAACACTTTAGAAACTGCATATCAACGCCTTGGCAACCCAAACAACCACATAGAAACACCCAAACAACAATCAAGAACACCCTAGCAACCACCTCGAACACTCTAGTGAGTAGCTAACAACCATCCATTTACATTTATGCATTTGGCAGACGCTTTTATCCAAAGCGACTTACATTGCATTATACTATACATTTGTATCTGAGTATGTGCAATCCCTGGGATCAAACCCACAACCTTGGCATTGCTAGTGCCATGCTCTAACCACTGAGCTACA
LG21	ref-1993	24.109			AAGTTTCCCCGCGACTTTCCTCAATGATTAAACTCTTTAAACTGTAGCACTGATGTGAAAGGTGTGAGCTCTTGCTCAAGGTGGAGCTGACTGCTGTCACCAGAGTTAAATCTTTCTGTTCCGCCTCTTCTTTATTTGGCTGAACGGAACGGGCGTTGCAAAACTCATCGCTTCTATAATCGCATCGCTTCACTCCATCACCAAAACTAACGACTGCTATTCCGCCCCCTCAGTCCAAACAGGAAGTTATAATGTCCACTATTTTAAACAGGCCACTGTAGAACTTCATACTTCACACACGTTATTTTTTTTCTTCATCTTTTGGCAACACCCGTATATA
LG21	ref-14704	24.856			TTGCCTCAGCCTGCTTCTGGACAGCTGAGAACTTTGGCCTGGTAGACTTGCAGGGTGGCCACAGCATGCAGGGCAGAGGAGTGGGAGGTCCACGGGAATATGTCCGGCAGAGAAGCTCCCACTGTAATCCTTGGTCGGCTCTGATTTTACACACAAATGGTGGCTGGTCAACAGCAGAGGAGCTCAAGCCTTTATGAGAAAGGTGAGATGCGAACAGCGTGCCGACATGGAACCACCCACAAACACCGTCTCATCATGCGTATGCCCAGGCGTGTGAGATAGCAATCACGGCCATCAGATGAGGACAGATAATGGCCGCATTCAGAAATGCATCCAGAAATGCTTGCTCTTTTATGGAAAAATGCTCTCTTAGTGCTGAAGCAGACAGGGACAGTCGCTGCACGGCATGCAAGACCCACTTCAGGCAGTCT
LG21	ref-46221	26.084			ATGCAATGTCTGCATTATTCGATTCATCTCAATGAAAAACAAATGCTAACTGATAGCTAAATGGGCTGGTCATGATTTATGGGGCTGCGCCTTCTCTGCTCCGTTCAATCCACACGAATTATTCGATTATTATGATTATTCTACTGATTTATTAGTGTGTTTAGGTTTACAACATAGTTGCAAAAAAGTCGCATTGCAGCAGTCTGATTTAAATAGGGATATAAAGGTCATGGGAATTTTAAGGGGATTTTTAAATTGTTTTCCAGGCATGTAAAGGTCATCAAAATAAATTTAATATTTGAAAGGATTGGAATTTATTTTAGCGATTATATATTTGTCTATCTCTGCTCTGCTCTGCTCTTAAAATAA

LG21	ref-58156_18	26.65			AAATGTCCTTTGAGATGTGATGGAAATGACATTTGTACAGACAAATAGAGCGTGCAGGTATGACGTCAGAACCCGGAAAAGTAATTCGCCACTGGTTTTTTCAAGATTTTC<br>CTATGGGGTTTTATGCTGCCTTCACGTGCTATCGGAAATTTGATAATTCCCACTTCTGAAGTCGTGATGCAAGCT[C/T]ATCGCATTCAAGTTTCAAAATGGAAGGGCGTTCA<br>TGTGCAATTTCTACGTAGGAAACTCGTATTTACAATAATTCCGAGAGCACGTAGTAAAAAGCAGCATTACAAAGGGAGTTTTATCAATTTATTAATAAAATAAAGCCTGTG<br>GTAAACATAACTTGACGATACTTTGACGTTTTGTCTAC
LG21	ref-9004	27.563	Chr25	12299241	TGAATTGTGATTGCATTATTCAACATTTTCTATCGCATTCTTCCTTCTTTTGTGTTCCTATTATGCGTCGCGTTGACCTGTCCCCATCGCAAATGATCCACATTGCTCCGGGTA<br>TGAGAGAATTAATTAGATAAAGAGAGAGAAGAACTAAAACATTTACCACAACAAAATTGGTTGCCGACAGAGATGTTATGCCATGCTCTTTCAGTCCGACCTCGTCGCGA<br>GCTTTAATAGCTGTCATTGTTGTGGCTGTTAGAAAGAGACAGTGTGTCTTTCATTGCAGTCTTTATCGCTCTGGTTTAAAAGGTGTTACTGAACCCGAACCATCCATCATGG<br>GCACGCTTTGTAAAACATGGGATATCTGTGTGTTGTCAATTGAAAACCTGTGCTTTCTCTCCAACCTATAGTGACTTATTAGAAGGAACATTTAA
LG21	ref-17802	28.091	Chr25	18503481	GTAGCGTGTAGCATAGGCCACATGTGTTTTGCAAAGGCCCAAATTGACATAAGGGTTTCATCATATCTGCCTCTTAATGTCAGGAAGGACGGCTCATGCCAGCTACTGATA<br>ATAAGCCATTTGAACTATGTGTTAATGTCTTCCAAGATACTAAAGCCCTGTTTCCACCTGGTATTAAGATGCGTTGTGATTGATCGGATCACAAGTAGACGAGGGAGACGC<br>ATTCCTGTCCACACCTGGTGTTTTAATCTCTTTTGACCATTTCTGTCCTGATTTCTTCAAGGGGAGGTGCCTAAAGGTGGGTAATTGAATGGGCTTTTTCAGATCTTTCCATC<br>TAATAGACGAAATAAGCTTACGCAATTTACATATGAACCCAACCGGAGACAATGGAAAACATAAGGAGAGCAGGCAGTCTTTTGAACTTCGATCAC
LG21	ref-13634	29.449	Chr25	14150388	CACACATGCAAACGTACACACACACAGGCTCATATTGCATTCCGCCAGTCTATATTAGATATTGGGGCCGCTGGGAAATATAAAACACCATCTTGAGTTTTAATCATGGTT<br>TATAAACAAGACCAAATTCAAAGCATGACAGAGTGATGGATTTCAGTTCACCACCCAAGTTCAAACCTGCGCCAGCTTCGATCGAATAGGTTGAGCTCTTTAGTTTGAGGT<br>ACACTTTCAAGTGCAATATAATGACTATTGATTTATTGAGACAGTGGATACCAGCTGCGCTGGCCGGCCCCTCGGAGCCCTGCGGAATTGTTTGTTATGTATAAGAGTCCTT<br>AATTGGGTGAAAATGTCTATTCATCTTTAGCTCCG
LG21	ref-20288	29.722			GTGATGGCCACGAGATGTGTGCAGCTGCGTC
LG21	ref-30382	29.803	Chr25	14147646	AATCTATCGAAATCCATTGGGTTTTGTTTGTTTACGTGCGCCAGCCACCATGCCTGTATGTTTAAAACAAGCACTGTAGTTATGTATGTAAATGTACATCCCATTTAATGCC<br>ACCCGTTGGTTACCATGGTTCCCAAATCCGCACAATAATTGGCTTAAGGGATGTGAGTTTGCCTTGTGCGCACTTGTCGCGGTTGAGAAGCCAAGGAGGTCTTGGAGCCTG<br>GCTTATTATCGACTTGGTAAAACATTTGAAATGGCGCAGATTTCAAAGAGCACTCAAATATTGTGTGAATTATGGGAAGGTATGCATGCACACATGCAAACGTACACACAC<br>ACAGGCTCATATTGCATTCCGCCAGTCTATATTAG
LG21	ref-17195	30.162	Chr25	13714437	CAACTTGATGTCACTGCAGTGTGAGGTCGTCTCAGTGTACTAAACTCCTGAACCCTGCGATTAACATCACTGAGCGCGCTCACAAATTCACTCAGAGGGGCTTAGGGGGCG<br>AGACCCCCCCGCATTCTTAAGACGCTTCATTGCGTAACTGTGGAGCCGTAAATGTTGCGTCACAGAGTGCAGTTGAGTGGAAGAGCCTTTTGGTGGCAACATCTAGTCAAA<br>ATAAGAAGATGCCTCGATGGCAAGAATCCATTGATAAAATGATAGAAATTAGTATCAGAATATTTCAATATCATAATATTTAAAGATTTCAAAATATAAAATACTTAAAAT<br>ATTAAATTAAATTGACCCCCTTAATCCTTTATTGAA
LG21	ref-24509	30.65			CCACAAGAGGATAGAGAAAGGTAGGCACACAGGTGGGACCCCCACTGACTTTCTCCAGCGACTGTGGACTAGCTACTGACTTTCGACTTCGAGCCTGGGCAGAGTTCATA<br>GCTGGCCTGCTCATTAAGCATCTCATGAATAATTCATGAGCAATAACTCAGCAGTCACTGCTGATCTGAGCACCTGTTTCGCGCGGCGGTTCAAGTGCTGCAAACGTTGTA<br>ATACTGTGTTTGTGTCGTTTTTTTTTTTAGTTTATTTCTTCACACTAAAGTTTGTTTTTTTTTATTCTAAATTACATTTATGCCACGCGGCGGTTCAAGTGCTGCAAACGTTCCA<br>ATACTGTGTTTGTGTCGTTTTTTTTTTAGTTTATT
LG21	ref-41323_31	31.605			AAGACCCTGTCGATGTTTGTGCATGCTTGC[A/G]C
LG21	ref-38760_30	31.68	Chr18	14180136	TTCACGTCCTCAGACTCTGAATTATGCGTTTCCATGTCAACAGTATCAACACCGACATAAATCTGCAGCAGTCATGTACAAGATCTCTAAGTTGTTTATGTTCATTATTATTG<br>CCGAGAGCAGCTGTGTATGTGTGCATTCAAGTGTTTTGGAGGAGGCGTGGCTTTGGGCGGCGATTTGGAGGGAGATCGTATGCTTT[C/T]AATGCTAGCAGGCTAATGTTAG<br>CATTCCTAGATCACCTACTGCACCTTTAACTAAGAAAATGAACAGATACTATTTAAAAACTTTGATTGCCAAGTTTGTTTAATATGGGGCCTCACTTAGCGTTTTTATGCAA<br>ACAAACTTGTGTAAAAACATTTATATGTAAATTTCG
LG21	ref-34627	32.188			CGATTGGTTTTGCTGGATTACGAGATCTTAATGACAGTCAGTTTAATTCGGTTATGATGTTTCAGTGACAGTTTTGTTGGGTTATGAGATCTCAATGATGGTCGGTTTTATTT<br>GGTCATGAGATCTCAATGACGGTTGGTTTTGCTGGGTTATGAGATCTCAATGACTGTTAGTGTTGCGCGTAACGGTCGGTTACGAGATCTCAATATAGTATGTTTTGTCCTT<br>TTACCAGATTTTAATGACAGTTGTTTTTTTTCAATAACGCAAACACATTACCACAACAGCTGGAAAAATCAAATATACACTTAAAGTTAAGAGGCCAACTAATGGAAACAC<br>CAATCACCATCATGGTAACCTCAAATGAAACAA
LG21	ref-58644	32.485			GGCTTGTGTGCGATATGTGTGCGTGTGTTAT

LG21	ref-11263	32.671	Chr25	12067520	GATTGAGTTTATGAAATGCAATTTTTCATTCTGATTTCTGGAGCTGTGAGTAGAAAAGAATGTCTTGTGCTGAAATGCATGCCATGTCTCTGCAGTGGCCACTAACAAGGGT GATTGCATTGCCAGCGTTTAGTGTGTGTGTGTGTATTTATGTGTGAGGGATGTAATCAGTGTGTTTTGATCCTCGTTCAGTGGAATGTAAGTGTTTGACGCTGAAAGTGCAG CCGTTGATCAGGCAACTGGAAGAAGCCAGTGTAGTTTAGGGAGTTTGTAACACAGTCAGACAAGTAAATAGTGGTGGAATGGTTTGTTTTATATAGAGCTGCACATTTAAT CGAAATTAAACCGTATGCGATTTGGCCAAGGCTGCGTTATTTTTTTATGCGCAGATTGTCAGTGCAGCATGGCTCTGTGATCAGTTGTAAATGCT
LG21	ref-55207	32.982			CAAAGCTAAACGATTATCTTGCCACTCGCAA
LG21	ref-37292_5	33.092	Chr25	21459684	GTAATTTTTAATTTTCAGTCTTTTTGATGTGTGCAACTGTGATGCATAATAATAAAAATATTTTTTTAATTTTCCATTTTTATGATCTGTGTATCTGTGTTGCATAATAATAAT AATAATAATAATAATAATAATAATAATTTCAATTTTCAGTTTTTAAAGATGTGTGTAAC[A/G]GTGTTGCAGAGGCGTCGTACACTCTCCTGCTGTACGACGAGCTGCTGGA GTGGACCGAAAGGCCTCTCAGAGAGTTTCTCACGTACCCCATGCAGAGCGAGTGGCAGAGGAAGGAATGCCTCCATCTCACCATCATACACAACTTTGACAGAGGGAAAG TGAGTCCATCTCTCATTTTAAGATGCACTCCACCCAC
LG21	ref-53323_7	33.14	Chr25	10071047	TCCAGCCACAGCTTTGGATGAAATCAACTGAAGATAAAATGAACTTCAAAAGTTTGATTTCAATTGGATTAATTTCAAGTGTTTGTTTAACTGAAATCATGCCAGTTTTTAC ATAGTGTAGTATGAGTTCAGCCAGTCTGGCATATTAATAGATTAAAGACTAAAGTAGACATACTGTATTTGATATTACGGTGTATTCAACCCAG[C/G]CAACGAACATAATG CAAATATGGGATCATGGAAAACGTTAACAACGTACATTACATTTTTAGAATGGATGAGGGAACAAATAACAAGAAACGGCAAACTCAAACCACACAAAGTGTGAAAACA GCAAAACATAGTCCAAACTATTTATCATCAATATAGTTACAACAGACACCCACCAATTTCAAAACTGCAATGACACAAATCATCCAAATTAGTCAATGTCAT
LG21	ref-73077_32	33.413			TTATGGTGTGGGTATTTGTTTTGCATCATTGCATATGAGCCAGTCATTAAACGTGCCACTGACTGGCTTATTGCACAACATATAGTATAGTGTTTTAATAAACTACAGCACG CCATAAACATAATTGCATACATGAGTGTTTCTTCATTCTAGACACTTTGATGTGTTTTATTAAAACTTTTTATTGAAATTTTTTTTCGTTTTGTCATACGAAGGCTCTGCCTTG GAAACT[G/T]TTTACTATTTATTTGCACATGAATCTGTGGAGAAAGACAAGATAAATATCAAAATTTTTATTGTGTGTCATGTCCAATCAAGCTGTAATTTGGTAGAACTAT GCAAAAAATTATGTTATGTGCATTTAGGAAACACACATTGCTAGCTTTCAAATTAGCATTAGCAAGACCAATTCTAACAATTCCACCATGTCATTT
LG21	ref-53323_9	33.729			ACCCAGGC[A/G]ACGAACATAATGCAAATATGGGA
LG21	ref-53270	34.151			CAGACCGGTACGATATCTTTGCCAGTCTTCA
LG21	ref-33022_9	34.624			GTGCGTGTTAGAAATCAGGAACGGTGACAGAACATTGTGCTTGATACGTTTTTTGTCGTTTTTTTGTCTTTAGCCGACAAAGTTTAAATTCCATATAGCTAGCGCGCTTCCC ATAAAAGTAGGCGGTCTTTTATGGCAGTTTGAACGCATTTCACCACATCAGCATTTACACTATGA[A/T]AGCAATCCAGTCGAATGACTACCTTTGGAAGTGGTCTAAAGTG GACAAGCTCAAAGCATTTTAGACCTCGTTTACACATGTATTTAGCGTTGTCCACTTGTGATCCGATTGACCAAAATCTGACTTATGACCTGTAACGCTGCCTGAATCATAAT CATGCACTGCTCCTGGTTGGCCAGAGGAGAACTGGCC
LG21	ref-38402_4	34.692			CAATTATTCTAAAACGGTTCTACAAACACACAATACAGTTTAAAAGACTAGTAAAATCAGTGTTGTTTTATATGGTCAAGCAGCTCTTAATTTAACACACCCGATAGATAT ATTTATATATTATTATTTCAACAATGCGGCTTTCATGACTGAATCAGCACATCACATTGTCAGATGCGCTCGGCCTCTTACAAAACGTGTTT[A/G]AATGAGCGAACCTGCTG CTCCTCGCTGCTCGCCTCCACTTCACCGGACATCTGGTCAAATTAAATGCAAAAAACAAAAGAAAACAAAACACAAAAAACGGAACACTTGGCTTTACTTCGCCCTCAGA GAGCCGTCAGGCGATATCGTTAACTGGCCGTCCGCTGCTGCTGCTGTCTTGAAAATCCGAAATGTTCTTCTTCGAGAGAAACAGAGAGCGGCGCACACACT
LG21	ref-42704	35.207			CACTGCTCTGCGATGCGTATGCACTGCTCCA
LG21	ref-38311_26	35.406			ACAATTAAAGAACTCCAACGTTTCTTCGGCTTCTCCAATTTCCTCTGTCACTTCATCAAGAATTACAGCTCCATCGCCAATCCACTCACCAGCTTCCTCCGCAATAAGCCCA AGTCTCTGTCCTGGACCCCTGAAGCCACCAACGCCTTCAACGCCCTCAAGGAAGCCTTGGAGTTGGAGCAGTGTTGTCCCAGCA[A/G]CAGGGGTCCCCAAG
LG21	ref-18211_8	35.479			AATTATGCAAAACACCAAAAATACAACATTTTCTGCACAATATATCAATACCATATCGGTTATAGACCGATATTAGAGTTTTCTTTTAGTTATTGTTTGATATTTGACATAT AATTGAAAACCAACTAACAAATATAGCTGCAAGCAGCGATGAAGGGCCCAACCTCGGTGACATTGCCACTCTGGTGGCTTCAGGGCAACTGTGCA[C/T]GACGAGCTATAT GCATTTAAACTGGGTAAATGTAAAAAGACTATTTTAAAATCATTTGAATATGCCACATTTACTGCAGCAGCTGGTTCCACCATGACCACAAGCCACAATAGCCACACCCAT GATGTAATTTAAATTCAGATGAATTTCATGTCCAGTATTTATCTCTAGGTCTTTTTTATATGTTTATTAACATAATTATTAAGCTACCTAGTTGACCTAAG
LG21	ref-31303	35.824	Chr25	32324288	TGAAGCAGATGAGCTGCTGGTGGTGGTTTAATGGTGTAGGGGATGTTTTCTTGGCACACTTGGGGCCCCTTAGTGCCAATTGAGCATCTTTTAAACACCAATGCCTACCTGA GTATTGTTGCTGACCATGTCCACCCCTTTATGAACACAGTGGGCCCATCTTCTGATGGCTACTTCCAGCAGATAATTTGCATGTCACAAAGCTCAAATCATCTCAATCTGGT TTCTTGAGCATGGCAATGAGTTCACTATACTCAAATGGCCCCCACAGTCACCAGTTCTCAATCCAACAGAGCAGCTTTGGGATGTGGTGGAATGGGAGATTTGCATCATGG ATGTGCAGCCGACAAATCTGCAGCAACTGCGTAT
LG21	ref-22288_10	36.156			CGAAAAAAA[A/C]CGAAAGATTTGCAAACAGCTTT
LG21	ref-31698	36.392			AACCTGGAATCGAAACTTTTGCTAACATCAG

LG21	ref-19884	36.779	Chr23	9522725	ATGGGGCGTTCACACCAGACCCGAATAAATCGCGCTATTCGCGCATAGTTGGACTCTTGAACATTTTGAGTTTACTCGCTTCATTCGCGTGTGAAATTCAGCAACACAAGATCCTAGGTTTCACACAGGTCCTTTACAGCCCATTTTTCACTTTTAGTTATGGGGCGTTCACACCAGACGCAAATAAATCGCGCTATTCGCGCGTAGTTGGACTCTTGAACATTTTGAGTTTAATCGCTTCATTCGCGTGTGAAATTCAGTAGGAATTCAACTCGTCAGCGGGAAAGTATTTGTAAAATACGGCGAATAAGCTCAATTTGCCGGCTTTAGCTAGCTTGTAGTCTCAATATATATATTAAAATTTAGTAA
LG21	ref-47389	37.076			AGATGGCCGCCGAGTGAAATGCTTTGTCTTA
LG21	ref-72303_16	37.171	Chr25	5492901	CTCATAATCAGCGCTGAGCAGTCTGGTTTAAAAATAAGACTCTGGTAATTGTGTCCGCGACACTCGGCTGGTACCTGCTGGGACTTCAAGCAGCTGAGATTTGGCGGGTGGAAAGAGACGATTTTCACACACTGTTGGCTTGGACTCCACAGCCAACCGTTCTTTTCCTCCGCACGACTGCACT[A/G]CGATCGCCTGGTGCATCTACACAATCAATAACGCATTACAACACAACGCTTCTCACGCAATTAAATACAACAATATTACAATACAACACTTGTGTTCAGGGAAAACATGACTGTCTTTACACTGGAATTTTGTTTACTGTAAATAATAGGATAAGAATCATTAATTCAATTAGTTCAATTTTAAT
LG21	ref-48307	37.87	Chr25	31173641	CTCGATTTATTCCCCTCTTTTGTAAAGTCATAGAAATGCTTTTATGGATTTTTTTCTTTTGCTACCATTCTAGTAGTTTACTCAACTATGACAACTTCTGATGAATCCCAGAAATGTGAAAAGAGTCCAAGTTGTACTTACGCCTGCAGCCTTGTGGCCCGAAAAGGAAGGTAGCCGGGGCGCACTTGTCGCAGTTTCTGCCAACCACGTTGGGGCGACACTGGCACTGCCCACCGCTCGGGTCACAAACAGTGCTGAGAGAGCCCTGCGGGTCACACTGGCAGGCTGAGAGAAACAGAGGACAAAAAGAGGAACTTAACTTTTTTTTTAATTGTCAGGATTGATCTGTTTCCCGTACAATGACGTAAC
LG21	ref-61734	38.651	Chr25	14412190	TCTGGGCAGCCACACACAGACCCGTCGTTGACTTAAGCATGCTGCTGATAGGCATTTACCAGTCATGGACTAATTCCCCTATTAGCTTCTATTCAGTGTTTCAATATTCTCACAAGACCACATAGCGTGTGGGGAGTCTTCACGGGATGTCCAAGCAAAAGTTTTGGTCTCATGTAGCAGCATGAAAATTGCTTAACAGGGTGTAGTAGTCTTAATTAGGCTACTGTGTTCTCAAGTAGGCTAGTAGCAATATAAATGGATTGTATAGTTACTCAAAATGTACTCTAAACAATTTAATGATTACAACTACTGTGTTTGTATGTTGGTTAACGCTAAATCTATTACGTATACCTCAGAGAAAACCAATAA
LG21	ref-15830	38.934			TATTGGGATGCCAGAATGTTCCCAGACAGGAGATTTATATGATGCAGGCAGCAGCTCTATCAGTGGCTTGTCTCCTACACTTGCCATGCTGAACTGCAACGCACCAACTGTGATGTCAGCAATAGATTTACATATTAACAGATGATAGAACAGCTGCTCTCGCGTGAAATGCATGCAGAGCATATCCATCGACAGAAGATACATTAGCGGTCTGTTTTATGCGTTGTTGTTTGTTTTAACAAAACGTATCATAGCTGCTTTGTCAGGTTTGAGATTATCCGCGGTGCAAATGTGTGCTGAACCTTGGGTGGAGAAGGGTATGGTTCGATTTTAACCATTACACCCCTAATATATCTCAACTAGCTGGTT
LG21	ref-50704	39.422	Chr25	14469609	AACAACTGAGATATTAAAGATGCAAATCATACAGTCAAATCTCCACATATCATAAACAGACACCCATATGAGATGCTTTGAATAAAAACGTCAGAAACCTTCTCTCTGTCTGACCTGCTTTCTTTTTCCACCCACAATCTCTCACCACCAATACCAACAACATTCACCAATATTATGAATGGGCAAATTTAATTAATTAGCAGTGACAGAAGACGCAGGTGCGACACAACCGAGTCACCAAAACAACCTGTCATCTATCCCACCATAGTGAAACACGCGCATGCAGAAACACCGGCACCCGTCCCCTCCCCCCTCTGAGACATTAGATTAGTGTCAAGACCCAAAGAAGTAAAAGCATATGGGGGGGGGATCCACAGGGCAGGAAATTGGGTTTTTTTCTAAAGACCTGTTATTAGAAGAGCAGTGTGGAG
LG21	ref-16210	39.462	Chr25	14413431	TCAGGTAAAAATTTGGTTCCAAAACCGAAGAATGAAATGGCGCAATTCCAAAGAAAGAGAGCTTCTGTCGTCTGGAGGTTGCAGGGAACAAACATTGCCGACGAAAATGAACCCCAATCCTGACCTTAGCGACGTTGGAAAAAAGTTTGAAAATGACGCGGCTCTGAGAGAAAGCCCGCGCGCGCCTATCTGTCAGGCGGGCAGAGATCACGAGCTCAACGCAGATTTCCATTTCAATTCGCCTTCCATTTCCAGCAAGCACTCAGACTTCTCAGAATCAGAGGACGAGGAAATAAACGTGTCATAATCATTAAACAGACTATGTAATTTCCAATGAGTTGTTCATGCACAAAACTGAAGAAAAACTTTGTCTTGTTTGACACGGTAGCTGTGTGAACTTTATTGATTGGAAAGCTTTCAGTTGAAGAGAG
LG21	ref-59192_7	40.347	Chr25	24342206	CAAGCACACGGAGGCAAGAGATTGCAGCTCAGCACAAATGTCATAGGCAGCGATGGGGTGGTGGCCATAAGCCTATTAGAGAGTTAATAAATCAGGCCCATAGCTGTGCCAGGAGCTGCTGTTGAGCCCGGCTTTATGAGCACTGAGCAGATTATGGCCATTGAGTTTCCAGTTT[C/T]GCTGCATCCATATCGATGGAGTGAATGAGGAAAAACATTGACCAGAGACTCAATTTCATTTTGGCTCCTTCCAGATCAAGGTTTGTTTATCTGACCGCCACCTGTGAAATGACAAAGTTTCATGCCAATTAATTTCAAAATCATTTTAAAAAGAATACTGTGCTAAAGGAATATTTTGGGATATAATGTGCTGT
LG21	ref-2607	42.152			TCAAGCTCCACGACCATCTTGCCTATCATCT
LG21	ref-39824	42.497			TGTTGAAGCTCAGTCCAAGGACCTGAAGATTAGGTAACGCGTATTTCCTTTGTGCGAAGCAGACTTGTTAATAGGGATTCACTGTTGGATCTTTCACCTGAGGCTTAAAGGAAGAAGCTGACGGCAGTAATCTGATAAATAAACACAGAGGGTAGGATCAGATGGCCCTAAAAAATTCAGCACAAGTGTCCAATGCAGGATTTTAGACTCTGTGCTGAGAGTCTTAATAGGTGCCTAAAAGCACTATTAGGCCATATGTGTCGAAAACACATGGCCTTTTGCATTACGGTGCTGATTATGTTTCAGACAACTTTTATTGAAATGTTGGTAACACTTTACAATAAGGTCTGATTTGTTAACATTAGTTCT
LG21	ref-9497	42.627			GTCTCTGTGTCGATTGCGCTGCCTACTTCTC
LG21	ref-13178	44.004			ATGTAAGAAACGACAGGTTTGCCAAATGAAG

LG21	ref-59625_23	44.674	Chr25	12030531	AATCATTTGTTGTTAAGTGCCAGAGATAAATAAGCGATTAAGACTCGGGATTCCTTTAGGAGTGTGGCAAAATGGCCTCGATTTGCTAGCCACCAGATGCTAAGCGGGACT<br>TCCTGTCAGTGGACGAGAGGGGAGACCTCTGGGAATTGCATTTCCACGACAGCAAACTACAAGTGCATGCATTTGCATCG[A/G]TGACAAGCCAAATGCTCCAGAGGATGT<br>GGCAGCCCTCTCCTCAATTAAACGCTTTGTCATTAGCTTAGGTGTAATTGCATTGTAGGTAATTTAAACTAGTCGTTTGGGAGTAGCAGACGAGTAATGGTGAACTACAGTC<br>AATGTTGATCTCATTAATGAAATTAGAAATGTTTGTTGA
LG21	ref-5308	45.696			ATTTTTGCGTCGATAGTGCTGCCATTGAAAC
LG21	ref-53161	46.079	Chr25	10456396	TCGTCTGTCCTGGGCTCTGTGCAGCTCAACTGGGGCGAGGGAGCGAGCTTCAAGAGTTACGCTTACAGGGCATCTAAGGTGAAACACCTGTTAGTTGCTTTGTGGGGAAAA<br>TGATTCAGTTGCAGTTCAAATTAAGTACTTAATTTTGCTTTTTGAATGTTTTTTTTTAATAAAGAGCGCCTTGAATATGGTTACAAGGTGTTTGGCTACCGATCTGGAAGCTG<br>AGGGGATCTTGTGCGTCGCCCTTCACCCTGGTTGGGTGCGCACTGATATGGGCGGACCAATGGTGAGTGTATAAATGTGTTTTTTAGGGCTGTGTATGGATTCCTTGATTTC<br>AATCGATTCTCTTTGTGATTAACTGACCTCGATTCTTAAATCCCAATAATCAACCTTTTAGCCTTTGTTTTCACTTGGTTGAACAAAACTTCAAT
LG21	ref-1335_15	51.245	Chr16	45481583	CAGATTTAGAAAGGAGTGAACTGGAGTGAATATTTATTTATTCATGACATTTTGGAAGGAATAAATTTAACAGAGATCATCATGACCTTATTATGGTCAATAACAATGACA<br>AATGCCACCATGCATGTATGCCGGTTAGTTTTTATGTTAACGCAACTGTGTTATCCAGTTTCAGTGACAATTGTATCCTATTATGGTCACTTACTGGGACGAT[G/T]TTAGTG<br>CATAGTGTGACAGAATATTGTTGAAGATGGCGAAAAGTTGTTACAGGCACAATATGTACGATTTTTGGATTGAAATATCCAAAAACCACTAGAACAGTGTTATATATTTTG<br>TTGACTTATGTACTAACATTATCCCAAATGTTTCCAAGAATGTTTAAATCCAGAGAAATAAGCAATTTTAACCAGAACACGTTGCCTATGAATGACAGCA
LG21	ref-47596_6	54.453	Chr25	2706640	CAGACCACAGGTTTGAACTATAGGTGAAAGAATCCGAAAGTGTTTTCCCAATCATCCATTACATCGCTGTGGACCAGAACGTTAGTGTTGTCATTTTTCCACTGCCAGAGTT<br>CATGGAGTCATAGAGTCTTTTGGAAACAAACAGGAGGGAAAGTTGGTTGAACTTCAGCAGGCG[C/T]GGGGCAATTGCTTCGCATCACATTTCACACTGTTGAGCCTTTGCC<br>ACGTTTGCTTGTAACAGTGGGGCTTTATTATTGTAATTTGATTAATGGCAAAGTCTTTGTCGGAGCCTCTTTCAATAGCGCCATTGTGTCAATGGACCAGAAGTGACTTCCC<br>CCTCCTGCTGGGTCTCGAAGACTGCTGAAACCGTCGC
LG21	ref-19870	58.533			GAGGTCGGAGGGATTGTTCTGGCAAGACATTTATAAACGTCTACATTCAAGTTTTGTACTTCCTAGCCTTTCCCTATGACTCATATGAATGCTGCCAGGTTTCCATTTTAAAT<br>AAAAAATGGTTCCTAATTGGGCACTGATTGAATTATGCTAAAAAATGAACTGAATAAATATGTTTTTGCAATATGATTTTACAATTTAAAAATGTACAAGTGAGCTGCCTA<br>TATAGACAGCATTTTAAGGCATATATATATATAAAAATAATACATATGCATTTTTTTTTCATTTTACATTATACATTTATTTCATGTTTTTTTATATATNNNNNNNNNNNNNN<br>NNNNNNNNNNNNNNNNNNNNNNNNNNNNNNNNNNNNNNNNNNNNNNNNNNNNNNNNNNNNNNNNNNNNNNNNNNNNNNNNNNNNNNNNNNNNNNNNNNNNNNNNNNNNNNNNNNNNNNNNNNN
LG21	ref-2610	60.962			TTAAACAGAGCGAAGGCATTGCGACACTTAT
LG21	ref-4526_17	63.19			TTCATTTCTTATGAAGTCACTCAACTATTATGCAAACTTCACCAGTTTGGTTTATTTACAGAAGTGGATTCTCTCACTGGGATTTAACTTTCTACTCAAGAGGAAATCAGATT<br>CCAAATGGAACTCCTGAAGGTCGGGTCGATTCCGCAGCCATCTTCAGTACTGGATTAAAGGTCACAGCATCAA[C/T]CTCGTCATGATGACATCAAATAAGGAGGACGTGA<br>GGCCTGAAATATCCTTTTTCCAGGCATTGTATTTGGCGGCGTTTGCTTTCAATTAAAGAATGTGTGGAAGGATGTACCTTTAACCACAAATATACATTCCAAGGTTCAGACG<br>AGGACTGACATTGTTGTCCAGTAGCCACTATTGTGTC
LG21	ref-58776_5	63.278			ATAC[A/G]CGAATCGATGAATATGCTAATTAGCTC
LG21	ref-34396	66.746			AGAAACACACTGTTAGCTCTGCAGACAGACAGGCGTTATGCACTGTTAATTTGGTTCAATTGGCCACACGAGCAGAGCGCGGCGTAGGCAGAATATCAATTTTGGAAAGC<br>TTGATGTTCCGCATCGATGAAAACAAGAAGGGAAAAGGTTCAACCACAAACAATGACACCATCTAAGAGGCATCGGCATCGAAGGAGCCAATCGTAGTGTTTGCTAACAG<br>ACGGAGCTCGTTGCCAATCAGATGTAGCGGTTGATTGAGTGGGCGGAAGGCCAAATGGAGCCCCGTGGGAAATGGGATCATCGCAGGGAGTTTCCCTTCATCCGACGGCC<br>CTTAGCAACAGCCTCTCAGTGAGAAATCACCTGTTGCTA
LG21	ref-39835	75.614			GCAGGATTTACGACAGACATGCAACACCACA
LG21	ref-60893	79.096			TGTTTTTAATATATCTTAATTTAGTTTGTATATATACATGAAATAAAAAAAATATTACACTTATATATAAAATTTTTTGTGTATGTTTATTTTAATTATACTAGTCAAACGTCT<br>GGCTACACGAGACTAGAAGCCAAGTGTCCTTGTTACACAAGTAATAAATCTGGTGGAAATCCTGAATTATATTTGAGCAGGAGCTGACGGTCCAAGCGCTTTAAGTGCTCA<br>GCTCATTTCCAAACATTCAAAGCAGCCAGAACAGAGTAAAAGATCTCCTCACAACGGCACAGCAATTCATTAATTTGACACAAGCCCCTACAAATCCTGCTCTTTCATTTC<br>AGTTTTCATGAAAAGAGCTTTCACTGAGCCTGTCTGGTTAAACCGACTCTAAAACCTGATCTGAGTTCAGCTTTTATGACTAAACGGCCACTTTG
LG21	ref-42635	79.722			TCAGCCGAGTCGATTGCAGTGCGATCTCTAG
LG21	ref-15281	80.489			CTGTTGAAGACGATGGAGGTGCGGATGACGT
LG21	ref-849	80.557			ACTCCTTTAACGATGAAGATGCAATTTTCGC

LG21	ref-67967	81.035	Chr25	2089063	GCTACCGTTATAGTTGTGCTGTGATTCCGCTATTCTTTTATATTTAGACCGATTTTTTTAGACTTATATTGTTATCGTTATCATCCAAGGTGTGAACGGGCCTTTAATCTTAGA GCAAAAGGAGTAAAACAGGTTTGTTTGCTATTAGTAAGCAATGCTAGTCTTACACAACAGGCCCAGGATTGTGTTCGTACCAGTTACTGCTGCTCCCCTGCTTGGGTTTGGC ATAAAACACCATCCATATTGCTGTGTAAAAATGAATTATGAACTATATTGCATAACTGTACCCAACGCGCACAAATTTCCATGCCACCTCCTGTAGAAGCGCAGCACACAA ATCTCCCAGAGTTCCTCTCCCACGAACCTGTG
LG21	ref-47831	81.677			ATGGCAGCAGGTGTTGCAGTAATGAGAAAATCAGTCAAAATCAAATAAAATGTTAATTTAAAAATATATATTATTTCAGAGTTTCATGTAACTGTCACAGACACACCAGGC TCCTCCATCACCCAATCACAGCGCACTCCTTCACCCGAATACTGATCACGTACACCTGCACCTCATCAGCACGGCAATCACCAGCAGCATAAAAGACACACTCACTCACAC AGTCATCGTCCGGTCTCATTAGCATCTAGACTTACCTTCATGCTCACCTTAAGGACTCCTTTAAGCTACTTACCTGTCTCCAGCGTGTTCCCAAGTCTCCTTCGTTGTTCCTTG TCTCGTATGAGTTCCCCTGTGTCAATCTCCAGC
LG21	ref-69862	83.801			ACACTTTCACCGAGACTCGTGCTGTTGTGAT
LG21	ref-36509_30	85.906			GCCGTTTGCATTCAGTGATCCACATTTATTGTTTTTGGACCAGACAAAATTCATTTCTGTTGTGTGAAATGTGATTTATACCACATCAGTAATATCTGTGGTTTTCAGTTTGA TCGACGTTGTGCTGCTGATTCACTGAAGTTTGTTCTGCATCGACCGTCAGAAACGTCACCATCTTTGCAAAGTCCTCGTACTGGA[A/G]TTGTCCGTCAAACGCCACGCCGG CTTCACTCAGCAGCTCGTCGACTGAGACATAAACATCATTATTATGAGTAATTAGCTAAGCAAGTCATTTCTATGCTGATGTGTTCTGAACTGTTCCTGGACTGCAGATGTT TTCAATATGCAGTCTTGCATTTGACATAAAAAGTTG
LG21	ref-13907	88.553			TTGTTTCAGCCGAGTCGATTGCAGTGCGATC
LG21	ref-24130	93.175			TTAAGAAATCATTCATGTCACAAACTCTGTAGGACGATGCACACACTAAACTTTAAAACTTGAGGCCACAATAGCAAAATTTTGCATGCAAAAAGGTCTAGTCTCAGTAGC ATAGCGATGTATGTAGTGTTTCTTTTCAAACCGCCTGCTTTAAGTGGGACTCGAACCCAGGTCCGCAGGCATGAGAGTCGCTTTAACAAGGAGGCTAAAGACCGCAGTCTC TAGAGATCAGGGGAATGAGGTTTACACACATGGCTCTTACCAGCCTACATCCGTTACACTCACCCCCTAAACCTCACTCCCATCCAGGTCACGGCACCAATGGGACCCCTC TTGTTCGATCCGCCCACTCTAAGCGGGACTCGAACC
LG21	ref-33125	97.551			AGCTTACACACGATGAAGATGCTTTATGATC
LG22	ref-25591_5	0			ACCACTCACATCGATCCTTACTTAGTGTTGTTTAAAAATCATCTTTATAATTCTGTTAATAACAGTTCAACAGAAAAAAAAAACAAGCAAAAATAAACAAAATACATTAAA AATATATCATCGTTGTCCAATACTTGGTTCAAACATCAATGGCTCATGTAGATGATTCAGTATATTTTCAAATACATATTAACCAACATCTCAA[A/G]AGCACAAGCCATGT GCAGTCCAGTGTGTGTTAAACCCAGAGTAATTTTACCTGATTACTTTAATATTATTTTACTGGATTTGTACTTTTCTCAAGTGCAATTTAAACTGAATAATTGTAATTTTTCTT AATTACATTTCTGAAGCAAACAAACAGTAGCCTACATTTTACTCTTACTTTTCACTCCTGCAAATTATTTTTATTTGAAAAGGTTTCATTTCATGCAG
LG22	ref-689_32	0.468			TGCTGGTAATCGAGTGGTATGCTCTCCAATC[A/G]
LG22	ref-5542	1.64			GAAGAGCTGCCGAAGAGATTGCAGAGCGCTT
LG22	ref-38908	1.936			GAAGTATTACCGCACATTCTGACCATAGCTATTGTTATAATACAGTACTCATCTTTTCCAGTGACTCAGATCAAAATGATGTAATTCTGGTCAGTAGATACTGAAAATTCAG ACGTCTGTACATGGCCTCCTGCTGTTCTTTGGTTTAGAGCTTAGTATTTAATGCATAATAGGGTGCAGCATCACTGTCACTACTTCCTTTCGTCGAACAGAGTGTGAAGCTG TGGTCTTTCATCTCTCTTCTACAACAGAGCTCTCTACAAATAACTCAGAGGACATTACACACTGTGTCATTTCGAAACAGGAACTGTGCATGTGGTTATTCATTTAGCTCTA CATATTTACTTGTATATAAAATCAGAAAGGATC
LG22	ref-17907	2.418			TCCATCTGACCGATCTGGATGCAGGTTTCAC
LG22	ref-20484_32	3.02			TAAAACACTCCCACACACACAGACACACACACACACACTGTTCTCAGAAACTCAGGGTTAGGAACAATGACACGCAACCTTGAGGCAGTTTCTCTGCTGTTACTTCAGTGA CAAATGTCAAGAGCACATTACTGTAACAAGAGAGAGCGTCCATGTAATGCACGAGCACGAATCTCTCTGCATTTCCTTCGCTCTGGCAAAA[C/T]ACTGGACGTGCGCTCTC AAAAACACATTGTTCTCTTATGAATCGGCTCTGCTCTCGCTCAGATATCGTGTGCGTTCTCAAACGTCCCGTGCGCTCGCCAATCTGCTCTAGAATTCTCTCTGCATAAACTT AGTCACAATATAGTAAGACTGTGTCTTATGAACTAATC
LG22	ref-689_30	3.042			TGCTGGTAATCGAGTGGTATGCTCTCCAA[A/T]CG
LG22	ref-34428	4.228			AGGGGAGGAGCGAAAAAAGTGCAGCAAGATG
LG22	ref-47208	4.606			TGAAACTGGCGGTTATGGTAAGAGGCGTGGCATTTCCCAAACACGTTAGAAGAACTTGACCAATCACAACACACTGGTCCAGCCGACCAATCAGAGCACATTGTGCTTTTC AGAAGGAGGGGCTTCATAGAGACAGGAACTAAACAGAGCATTACTGACAGACTGGGAAGAGAGGAGCTGCAACAATGTAGCTTCCATAGTGTCTTGCTGATCCAAGCTG GAAAACTTATTAAATATTTGAAAGTTAATGGATTATCACATAATGATCAATATTGATATGCAGTAAACTCTTGATATTATTAAAATACATGCTCAGGAACTCCCATTCAAGC TGTAATAAGGTGAAGAATGCTAACATTTTATTATTTA

LG22	ref-35756_1	6.933	Chr2	41272594	ATCAAACACAATGACAGGTGAATGAAAAGTAAACTCATGCAAAACATTCTTATAAACGTATGCATTACTTAGCTAATTCACTAAAGTAATGTCTCTCTTCTCAAAATCTCC TGATCTTGTACAAATTAACTCAAACAA[A/G]TGATGTTTGCAACTTGTTCGCTTAAACACCGCAAGCCAAATTAACATTTTTATTTACAACACGCAGCGGCGGCCTGAAGAA AGCCTGTCATTGATATCATGATTATTACGCCTGGTGACCGGCTGTATAATGAAGCTGTGCAACTCCTTCAGGAAGATGAATTGTGAAGTTGTTGTGTGTTGTCAATGCATAG TATACT
LG22	ref-42919	8.258			GTTGATCGCATGAAAGCAGTGATCTCGTCTTTCTGCACACTAACAAATACTTTTTATTTTTTTATTTATTATTTACTTTTTGTTAGTTACCTTTCGTAACTAACAGTGCAAACG TGATATAATT
LG22	ref-11081_1	9.123			GTGCCAGTGCTTTGTTAGGGGGTTGCTAGGGTTTTCTGTTTGGTTGCTAGGGCATAGCTAAGTGGTTTCTATAGAGTCACTATGCATTTGCTAGGGTGTTGTTAGGGTGTTTT GGATGATTGTCAGTGCTTTGTTAGTGGGGAGGCTAGGGTTTTCTGTTTCGTTGCTAGGGTATAGCTAGTTGTTTTCTAGGGAGTCAC[A/T]ATGCATTTGCGAGGGTGTTGCT AGGGTGTTTTGGGTGGTTGCTAGGGCATCACTATTCAGTTGCTAGGTTGTTAGGGTATTTTGGATGGTTGTCAGTGCTTTGTTAGTGAGGAGGCTAGGGTTTTCTGTTTGGTT GCTAGGGCATAGCTAGGTGGTTTCTAAGGAGTCACTATGCGGTTGCTAGGGTGTTGTTAGGGTGTTTTGGATGGTTGTCAGTGCTTTGTTAATGGG
LG22	ref-11081_2	9.123			T[A/C]TGCATTTGCGAGGGTGTTGCTAGGGTGTTT
LG22	ref-32892	9.314			GAAGAAATTGCGATGTCAGTGCCGATAGCCT
LG22	ref-59218	10.127			ATTAAAATGACGAGATCACTGCTTTCACGCA
LG22	ref-4950	10.128			ATAGCCAATGCGATAGTACTGCAGCTATCTG
LG22	ref-8502	10.813			TGATGATGGACGACAGCTGTGCTCTCTTCGT
LG22	ref-35756_23	10.885			AGTGTTTAAGCGAACAAGTTGC[A/G]AACATCATT
LG22	ref-41687	11.216			TGAAGTTTCTTTCTTTAAAAATGGCACTCCTGACGGCTTTGGCATCTGTTAAGAGGGTCGGGGGCCTGCAGGCATTTTCAATTGACGAAGCATGCCTGGAATTCGGGCCGG CCAACTTTCACGTTATACGTGCCCAAAGTTCCCACCCCTTTTTTAGTGTTCAGGTGGTGAACTTGCAAGCATTGCCCTCGGATGTGCTTACGTAGACAGAACTCAGTGCTTT AGGATCTCAGACCAGCTCTTTGTTTGTTATGGAGGCCAGCAGAAGGGAAAGGCTGTCTCCAAGCAGAGGTTGTCCCACTGGATAGTGGACGCTTTTGTCTTGGCTTATCAG GTTCAAGACTTGCCATGCCCCCCAGGGGTGAAAAC
LG22	ref-22546	11.225	Chr2	57393016	TAAAGTATTTGCAGTGAATTAACATCTGTGGGTTTGTGCAGAAAACCCTCAGACCCCAGAGGTATTTTGCTACATAAAAAATGATATTGAATGGCCTTCTCTATATGAAAG CTTTTCATCCGTGAATAAATAAAACCACAAACGGCCAGGATCGGTTTAAGCATTTTATGTTCTGCTGCAGCAGGTTCTGCAAACACTTTACAAGTGCAAAGATATTGATGC ACCGAAGGCTTTTGTAAATCCATCTTGTGCTGTGAAAACATCAATTTAGTATAAGCCTCTAATAAGGCTCTTTCACGGACACCAAGGGGGAATTTTGGCCAAATAAGAGGC TGTTTAATAACACATCTGAAATGTGAGGACAGTTGTGTTAAGCTCTGTGCTTAAGGAGAGTGAATCAGAGAGCGCTGGTGAAGGACACTCGAGCGTAA
LG22	ref-15579	11.468			CCAACTGCAGCGAGCCCTGTGCAGACAGCAG
LG22	ref-33920	11.507			GTGTTAAGACCCACAGACCACAGGGTGAGCACCCCCTGCTGGCCTCATTAACACCTCTTCAAGCAGCAACTTAAGTTTTCCCAGGAGGTCTCCCATCGAGGTACTAACCAG GCTCAGCCCTGCTTAGCTTTAGTGGGCACCAGTCTTGGGCTACAGGGTAATAGCTGTAGAGGTCTACAGCAGATTCTTCGAGCTGGAGAGACTCCATTCAACCACATCATC AGCAGTTATTCATAAACTGAAGGCCATATTCACCAGGCATGGTGTACCTGAAAGAATTATTTCAGCTAATGGCCCACAGTACAGCTCGCATGACTTTGCAATTCAGTGGGA CTTTGCGCATATAACATCTAGTCCGCACTACCCACA
LG22	ref-52967	11.69			TGTACATGGTCACTAGTGGTCCGTTTATGGTGCTGCTTTCTGTGACACACCACTGTGTGACACATGTGACACAGCAGAGCTCAGTTGTGGTCCAGTTTCACGTGCGGGCAGA AATCACAGAGTCTGTGGAAACATGTCAACATCAGTCTCTGTCTTCCATGACAGAGTGGACTACCTGTTGCTTGCCTGCTTGTTTGCCTTGACTCTCTTCGAAACAGGTGCTT GGAAATTTTCATTGTCACATCTGTAAAAGAAAAGAAAAAAAAAAAAAACGCTAGCATGCTGAGAACACTATCATTACCAAAACTGAATGAAATATATCACGAATAGAGAGT ATCACATTACAAACGTTTACACTTGAGCATATATACACAAATATTTGTAAGTGACATTATCACCTTTATTTATATAGCGCTTT
LG22	ref-10823	11.835	Chr2	9089650	TTTCAAATATGAAATTTAATCAAAAGCTTGGCAAACAGCTTTAGAGAATTTGATGTTTCCCCATTCAAAGAGATAGGAGCTGCACTTGGATGCCCGAGAGGCATTTCAAAG ATGGCCACCGAGTGAAATGACTTGTCTTAAAGGGACTTTGATCAAACTTCCACAATTAAGGTGGAGCAGCACCTCTCTCGAGTAGTGCATCCGACCGTAGTCTTTGAAATC ATAGGCATCACAACCACGCCATAGATTAAACAGAGTCCACATGGCAATGAGGCACATAAAATCCATTTTTGTATAAGCGCCTTGTCAAACATAGAGTTCACTAAAATAACT TAAAAGTTAAATCAAAAGAGTGAAGACATAAAACAC
LG22	ref-12978	11.882			CACAGGAACACCCATGTTGATGGATTTAAGTCTCCCCTAACAATTGCTGATGAGTAGACGTTTTCGTTCCATCCTGCCTATGTCAGGAAGACTGCAGCCTCAGGTTGTCGAT AAAAAAACTGGTGAGTGCGAAAAGGAAACTAAAACAAGCAAAACAGAGGCAGTATTATGATCGCACTGCACAGTCATCGTCAGCATTGGCACAAGGGGTTGGATGCCCG CCACTGTATTGCAATCTATGGACACACAACGGTCATACCTTATCTCCCCTCATTATGACCAGGTGTTCCGCCGCAATCGCCGACACTTAATGGAAACCAATTAAATAACTTC

					AAAAGTAAAGGATCCAGCACCTGGGAACATGCACAT
LG22	ref-48441	11.914			CCGCCTGCCCCGACCCCTATGCCTGTTTTCT
LG22	ref-47673	12.072			GGAGAGATTGGTGTTGAGATCCCTTCCCTTGGCCACCAGACAGAAACCTATCCTCTAGTTTTGAGCGTTTGGGGGTCTCTTTTTCCTGTGGCCAGTCTAGCTGTAATCTGGC CACAGCTTGAGTTACTACCTCTAGTAATTCCTCAATGGATTTATCATCACGTGAGGAATGCTCAGAGGCAGCACACTCGTAGAGAGGTTTGTCACCCCCCCTCGCGAACTG AAGAGGTACCAGAGTGCGCTTCAGGATCACGCAAAGGGACGCATGGATCAGGTGACAGAACGGGCGAATGGGCAGGACCCATCTCCCGCTCGTCAGCCAAATCCATTCG CGAGCCCATGAATGTACAGTGGGTGCTGACTCTTTGA
LG22	ref-21083	12.086			CTCCACGGATCGACACTTCTGCAAATCACCC
LG22	ref-47848_18	12.157			TGAGTTTCTGGAGCTCTTGGAGCTCCCATAGTTCATCGGGCATCTCCTCCAGACCTCGCATGGCCAGGCTGAGCGTGCTGTAGCCCAAATGTCTGGTGGTATGTTTGCGGAT CCGGTCGACCGAGGATAAATTTTCCACGTAACTGGTCTTCCGACGCAATGGCCTCTTTGAGTCCTCGGAGTTGGACTTCCGTCCGGCGGATCTTGGTGCGACCTCG[A/G]TG CTGCTTGCGTGCTGCTTTGAGATGTTTTGCCTTAAGAGAAACATCTTTCTGATAGAAGTCTGATAAGTTTGCCAACATGAAGCATTTGCACGTACTATAAGTTCTCAAAGTT TCATCCCATGCTCTTGTTTGTTCACTTTAGGTCCAGTTTTGAGGTCTGAATCTTTGTGCGGGCTGGTTAAGGATGAGAATACGAGCGTTCAGAGTGACG
LG22	ref-6321	12.48	Chr2	40955709	TATTATTATTGTCATCATTCCAACATAAATCATAAGCGAACCGGCACTGAAGTAAAGGAGTTTTGTACACTAAATCCTATAAAAATCTTTTAATCCATCAAGCTTGTGAAGT TATTAGTGGTACAACCTGTGATGGAAGGAAGTGAGAGATTTCAGAAACTGTGGGCTTCACAGAACAGATGGACGGGTCTCAGCTTCACCTCCACCTTCCGATTCCTGTGCT CCTCCTAATGATTTCAGAAGATTTTCTCAAAGCTGTGATGGACATGAAAGCAGGAAGGGTGTCCGTGGAGGTGGCGGACCCCCCTCTGATAGCCGGTGGATTGATGTCTGG TTGATCTGAGGGACAGATGAGGTCTTCTGCCATGGCTTGTCCTTTGCAAAAAGGGGGAACAGAGTTAGATTTATACTTGGTAAATAGGGGTGTAACG
LG22	ref-64022	13.204			TGCACTGGATCGACTACACTGCAAAAATGAT
LG22	ref-73383	13.767			ACAGTCTGATCGACTCCATTGCTGCAGTAAT
LG22	ref-56066	14.499			TGTGGTGCATCGATGAGAATGCACAAAGTTC
LG22	ref-58555	15.704			TTAAGAGCACCGAGAGATTTGCGATTGCACA
LG22	ref-49055_29	16.145			TGTAGTGAATGAGACAGTCCACTAGATGTCGCAAATGTGCTTCATTGTGTACTGAGTGTCACATGACTCTTCAGTGTTGGTACAGACACTAACGAACCGCCTGTTAATGTG GGTCACTTTCACATCCTTCATCACTAAGATACACAAACACATCAGGAAGCTCACAGTTCAGATGTTCTGCATCCTCCTCGCCAGCG[C/T]TGAGTCTTATGACCTGATAATTA TTTGTCTAAATGTGCGTTTTCAATAGCCAGAGAAATCTGAAACATTTAAGATGAGATGAAATGAATGTGCATGATGGATCAGGACAGCTCTGGTGCTTTTAATTATCCTCTC AAAACTATGAACAAAAGAGGAATAGAGGAAAGAAATG
LG22	ref-66352	16.27			AAATGAATGGAGGCACATTGGAAAGAGTTACCTTCCTTGAAGGGCTCGACAGCGGCAAAACAGGCAAAAAAACATCGCCAACAGATAAGCCAACTTCCACTAAATAGTG AACCATAGACTCTTCCCTCACAAATACAACTACAGCTTTATCCATTCTAGATGCTGACAAAATGTTCCGAGCACCTATTTCGCTACTAATAGCCTTCAAACAGTCTTCAACA GTGATTGATTTGTCGTAAACATTTAAAACCATGCCGGGCTGTTAAGGTAGCAAACCCATCAGAATCTGACGGCATTTTCACAACCCCCAACTAATACCAAATGACAGCAAA CTAAACAACACTAACCTTTCAAAGTAATTGAAAAAAA
LG22	ref-72561	16.86			GAGTAAAACACGACTGCTTTGCAACAGTGTC
LG22	ref-9000	17.387			AGAACTGAATCGACTCTTCTGCAGGTTTTTG
LG22	ref-41733	17.807			TTTAACTTCACGAATCAATTGCCATGCAGTT
LG22	ref-58649	20.768	Chr2	39169032	TACATTATCAACAGTCAAAGAACACGAGGTTCCTGAGTTTTGTAGCAATTCACGAGGCATTTTCTCAGCCTGCTGTCAGGTAGCTATTCGTGCAGACTTTCAAAGCCGGCA CTCAGTGCTTAAGGATTTTTTTTTTTTTTCAGGATAGAGGATGATAAGCACTTCCTTTGGCAGAAAAGGACCCAGTCAAATACAAATCTGAGCAAAACTCAAAACAAGTGC TGACAGCCAGACCACATCCTATACTCCAATCCTATTTTATTCTCCTCTTTTTTTCTCTGTCTCTTCTGTTTGTCTTAAATTATATTTCTACACAGACCAGTACTATAGTGCTAC CAAGGTACAGTTATGGTATTGGATGATAATAATACCATGGTACTTTGAGATATACCATGGTATGTCATGATTATCATATTCATATACCATAGTTT
LG22	ref-56761	21.634	Chr8	48510220	CACACAAAAAGTATTCTTGTTGCTTCATAACATTAAGATTGAACCACTGTAGTCACATGAACTGTTTTAAATACGTCTTTAGTAGCTTTCTGGGCATCTGAAAGTGTTAATT ATCTTGCTGTCAATGGAGGCCTCACTGAGCCATCGGATTTCATCAAAAATATCTTAGTTTGTGTTCCGAAGATGAACGAAGGTCTTACGGGTGTGGAACGACATGCTTGCTT ACAATTCCTCACACACATACACCACTCGAACCTTCCGCAAAATAATCATTCATAAAGTTCGGAAAGGTTGAAGCAAATTATAAAGGACATTATAGCGGGCTTCATTGCACA GGATGGCCGAAATTATCGAAATGACAATATCACGTGATTGATTGCTGGTTTGAAAAAATTGGCGCTTCACGAGCCCACCTGATGCATAAGAGGGAA
LG22	ref-35194	22.617			CTGAAGGCCGCGATGCGCATGCGGGAATGAA

LG22	ref-10534_29	28.861	Chr2	37991508	GCTCATGAGAGTCCCTGGCCACAAAGTAGATGTCCATTCCAATAAAAAGCCCCGTCAAAACACCTGTAGATATACTAGCAATCTGCACGGCGCGTGCTGCAGTTGTTCCCGCTGCATTGGCTATCTGCATCACACGCATAATCTCATTGGCGTTTGTTAGAATAGCTTTGCCCGCCATGGCACCATCCTCGTAGATA[A/G]TGTCGAAACAAGGGAAGTCTTGATTGTAGGCATGCTTCTTAATCTTATTCAGGTTGAACTTGCGTAGGTTTGTAATGCCTTGCTTGATGAACTTCATGCATTTGTTAAGGTCAGCCATCTTCGCCTGGTAGTCTTCTACAATGCGCTCCACCTTCTTGCGATCCATAGAGTT
LG22	ref-44417	32.187	Chr2	33140618	TCATTCAAAACAAACAAAAGGGGCTAATAGCCCCAGCATCTGTGTCCAAAGCGGTGGCAGGTCGATATACACACTGATAGATGTGAGCCACAAAATTGCTGGTTATTATGGCAGTGAAAGGGCCTTGCTCGGTTTAAGTGGTCAGTAGCGATCATATCTCACTCCCTGAGATTATCCACCATAAGGCAGAGCTGCATTCACTTATCGGAACGAAGACTGCGCTTGAGAAATGTTCTGATAAGATATATTGGGTTATAGCAGAGCTGTGAGGAAGCAAATATCTAATATTATATTCCAGTTATTACACTATTTTGGCTGTACGGCATTTATCTGAATCCATTTTTGTATCTGTTGTACTGGCCTCCAAATGTCATTCCCCATAGTGCACATATTCCTCACCGGTGCTGCTCGCTGTCTTTATTGAAAAACAC
LG22	ref-61845	34.583			TTTATCTTCAGTTGATTTCATCTAAGGCTGTGGCTGAATTCAGTTGTAGCTCTTGTGTTTCTGTGTTTAGTGATTCTGCTTGTTAACAGGCTTGTTAATGCTCAGATGTTTTTATAAATAATATATAAAGATTTTGTGGCTCAGATAAGGTCTAGTTCGGGTTTATTTCTTTGCTCTTGGCAGACATGTTGCATTGCTATGGCCTGCTTATGGCTCAGATCTGGCAAACAGGAGTGCTCCGCCCAAGTGCCATCATTTCACGCCACATGTGGCCCAGATCATCATACCACACGTGGCAGATGTAGGCCGGATCTGTGCTGGCACAAAGTTACTATCTGGGTATGAGGAGTTATTTAAATAAACCTGTAA
LG22	ref-19539	35.928	Chr2	29836152	TAGTAGTGCTTTATTTTGGCAAACCACATGTGCTTGTATGATTTAATTTCAGGAGCAGTGTTTTGGCTTTGTCTGCCTCGCTTTCAATAGACTGACATTGGCATAATGAGAAAGGGGAGTGTTGCTCACCTCATTTGCAGTAAAGATGTTATTTTTGTCATATACTTTCACACATGCTGGCAAAGTGATCGTTTCATGCATTAGAGAGCAGTAAAGCAGTCATCTCTGTTTAATCTGAAGTTTTGTCCTCTAGTAGGAAATAAAATGTCTGATAGCTTTATGGTCTGTTAAAAACACTGCAGCTAGATGCAACAAAGCCCTTCACTATGAGGTTTTTGTTGTTGTTGTTGGGGTTTTATTTTTTTATTT
LG22	ref-20931	37.407			GGACTACTCTCGACTCCTCTGCACTGAGGTG
LG22	ref-19553	38.274			AAGTGTTCAGCCCATAGTCTAGCTATCAAGGTATTAAATAGGGACAAATATACAGTGGGATGACAATTTTTGGAAATGTTTATACACTTTAGAAGCAAAATATTAAACCAAGTGGCATTTGGAAACAAATGCTGCTAGAGCTTTTACCAGAATTTTCCCATTTATTTAATAACTTTTAAGCAATGGCATCAAGGTGATTTTAGTGGATAAATGAACTCAGTTCTCCCCATTAGAGTAACATTTTTATAGAGCTGCACGATTAATCGTTAAAAAATCGTGATATCAATTCGAACCCCCACAATCTCATTCCTAAATGACAACGATTCGGCTGCATCTATTAAACCTTTGACAAAATCACACCGGAACATT
LG22	ref-42993_32	39.454			TTCCTCTTCTTTTGTTCTTATCCCCTACAGACTGCAGAGAGCACACTGGAAAGGGAGATGGACAGGCGGATGATCTGAAATGGCCTCCACTAATGGCTTCAAGAAGGAATTAATATCATTCTTTCACACCGGTGACCCACACTTACCGCAACTTTGTCAAAACACATTCAAGCGCCCCTGCAACACAATCGGACAAAAAGAA[C/T]CAAATAAATGAAATTAGAGAAGCCTAAGGAAGCTGAGTAAAACCTGGCACTGATGCTGGCCTCTTTTCCCCTTTAAAGTTCAGTGTTACCACAGAGGCAGTCTGCCTCATCACCCTCTGCCTGCTTATTATCTATCAGACTCCATTTAATGAGGCATCAGAAGAAAGG
LG22	ref-54081	39.785			CACAAGTGGACGAGGGAGATGCCCATTTACA
LG22	ref-52933	41.577			GGTATGACAGTGAGGGTTATCGGCTTTCCAAATGTTAAAGTGTCAGTAGCTGAAACTGCACGGCTATCGTAACCCTTACAAAAACACTCCTGACCCTACATCTGCATTACGACTGACAAACTGACCTAACTGAGCCCTTTTGACGACAACCAACTATGCAAAGGTTATGTTTTCACAAAACCAGGTGCATGGGTTAAAAAACTGCATTATAATAATATTGAAAGTGGATTTACTTACTTGGTTCTAC
LG22	ref-71997	42.168			TCTGAGTCTGCGACGGAGTTGCAGCGACGAC
LG22	ref-52168_31	43.612	Chr2	36255933	GATGTGTTTCCTGTCACTGTGGGCTGCAGGGCGCAGTAATACAGAGCAGAGTCTGATACTGCAGCAGAGGAGATCTTCAGATACACTTGTTTATCATTCATACTCATTTCTCCATCCATTCCGGGGATACTGGGATCTGCATATGTTACATGTTTCGTTTGTGACTCCATGACGAGGAGCACAAATTCAGGTCTGGATCCAGGATACTGTCGATACCAATGCAGACTGTT[G/T]GCACTGCCATTATATTTACATGAGAGAGTGACCGGCTCCTTCTCAGTGACATGAACATTGTATTGCAATGGTCTTATTGACTGCGCAAATGTATCTGCATAGAATAGAAAACATTCAGTTAGGATGAACACATTCAGAAGAAGTAAATCAAATGGCAACTTAATACTATGAACTCTCATAAAATACAATTAATTACTATCC
LG22	ref-37198	44.938	Chr2	34823128	AGACACACACACACAGGAATTTACAGAGTGTGTTCTTCAAATACGGTACACTGAGTGTGGGTTTCCCCCTGGAAGTTGTGCTCGAGGCTGTGCTACAGTCTTTATCCAGAAAGTCCCTATGGTACTGCTGCCAAAACCCTATAGCTTGTTGAACAGCGGTCAGTCAGCTTTTTTAATAGCAGCGCCATATCTGTGTACCATAGCTTTTGTCGAGCAGAGTGCAAAGAAATTAATATTATTCAAACATTTTGTTGACATCTTCATTAAATACTATACACAGGCTTTTATAGGGCATTCCAAACAGTGTACCCTTGCCTACTTCCACCCCCATATATTGTGTGCATTACCAGGATTTCATTTTGATTTCTGTCTTATCATCATGAGACTTTCCACCTAAAAACACGTGCCCTTTCCTAATTACTTTCTCTCTC
LG22	ref-40562	48.722			ATATTAATAATGATGCAACAAAATTTTGGCTACCAGAAATATTTTTTGGTTTTAAAAGGCTGAAACCAATGACCAAAACAGTGACTTTTAATTAGCGCTTTTCCAATTACACATTTTGACCATATCAGTGTCTATTTTGTGCATGCTATTTGGATACGCTACAACAGGTAAACATGACAGCAGTCACATTGTGGACGCACTTCACCATAACCAAAAAGGATGCTAGAGTAGCTAAATGTAAAATTTGTTTGGCTGAAATATCGAGAGGTAGCTTGTTGCTGGGTTAATTTATCACCTGAGAAAACAACACCCAGAACAGCATATATTAGCCAAA

					TTTAATTACTAAATTTAAGCTAGTTGTGCAACATC
LG22	ref-46998_8	49.426	Chr2	32600495	GCTTTTTGGATAAAAGCTAAATGCATAAATTTGTGCCACAAACTGCATGCTCAGATTCTACGAGTGCATTTTTGTATAAAACTCATGTGCCATTTTCTCTCATGGCAGATTTCCTGCTGTGGATCGACAGCACAGGCCGTGTCCTCCCCATCAGAACAGGCACACCAGCAGCAACAT[A/G]AGCAGCAGGATCGACTCCGTGTGTAGTACTGAACCTTAAATCTCCAGATGGGTCCGGCTCAGTCACGGCCCTCACTACTGACTTCCAGAAGTTCTCTCCCTTTGCCCCGTCACTGGAGGAAGGGGCTGACAAACAAGGCGGAGAGGAGCCTGCCTTCCTCTCTGCTCACATGCGTCCTCCAGCAGCGGCAGG
LG22	ref-27194_24	50.347			AATGAAGCAATGGAACTGGCAGGTGCCTCTTGCCAAGAACCCCAAGAGGCAAAGAACAAAGCTAAAAAGGTTGCTGGAGTAAAGCAAGCAGATACCCTCAGTACTAAGTCTTAATGTAAACCAAGAGATATAGACCTGGAAGATTTTTAAGAAGCTTTTGGAAGATGATACCTAAAGAAGCAATCACATCGA[C/T]TCTGCAGAAGGTTTTTAGGAATGCTACTGATCCCCATTCTGCTGCACTGGGACTACACCAGGTTTGAGAGGCCTTATTCTTTTGCAATGACCGAAGATATAGGACAGATGAAAATAAAGACAGAGAAAACCAACTGTGAGAAGGCAGTGCTTCTGACTCATGGAAGAAGTAGATTCT
LG22	ref-30410_1	52.666			TGTCCTGGGGGCATCGATTCACAAGGTAGTCAAAGAAAAACTGCATAAACAGAACAGACTGGAACGCATGCAAGCTACAGCGACAATGGAGGCCTACGAGAGATTTGTACAACTGTAGCATGAAAGAATACAAAGATGTTTACATGGGTTTTAACTCATGGTGAGAGATTG[C/T]TCAAAACCGCACATGCGTCGAATGCAGATAATGAACGAACGACAAGGAAGCACAAAAAATGAACGTACAGTACACAAGAGTAAATACAAATAAGAGTTTGTTGTTAATCGCCAACAAAACAGCAGCTCCAGACAATCAAAACCCAGTATTACTTACATGAAAAGGAATCAAAGCAGCCTCCACGTCTGTTTTCAGGCCT
LG22	ref-33594	54.481			AGGTCCTGCGACGCCGTTTTCTGTCCAGTGAAGAGTCTGGGCCTGATGCCGATTGGAAAGAGGCAGATCTATACGAGTTCTGTAAATGCGTAATGTTTCAGGCCACATTTAACACGCTATATGGACATTCGTCAAATCTGTGTCTGGATCAGCTGCGAGAGGACTTTGAGAAGTTTGATGCCTTTTTCCCTTTACTAATAGCCCATGTGCCAATTGGCTTGCTGGGAAAAACCAAAGAAATTAGAGAGAAGCTGACAAGATTTTTCTACCCTCATAAAATGGCAGAATGGGAGGCTCCAAGTGAGTTCATACAGAGCCGAACAGCCCTGTTCCAGCAGTATGATACGCTTCAGGACAGAGATAAAGCAGGTGATTCTCTCACATGCACACACACACACAAACGTACAGTACACAATCAGAAAGTCTCAGAT
LG22	ref-37479	55.42			TGCGGTTTAAAGGGGGTTGGGAAAGACTAGACCACGTGCTTTGCAATCTGCATTGGGAATTCTCTTTGCATTCACTTGTGAAAGGGTTGTTCGATCACTATGGAAAACCGCTATCAATATTGATCGCACAAATCAATAGATCAGCGCAAAAAGCATCATTGCTGCTCAAAGTAGCTGCGTGCGTTCAACTCGACGCTTTCCTGCACATCCCCGGCTACACAGATAGCAGCCGGTAGCCATGGTTACAGCGCGGTAGTATATTACATCATAGCGTTAAGTACTCATAAACAGCGGCAATATCACAAAACAACAAGTCAATTTTATTTAAAATAAAACTCACTATCAACAAAAGAATGAAGTTGCATAACCA
LG22	ref-1965_25	55.586			TTTATGTCTCAAAAAGTTCATAATGAGATTATAAAACTAATTTTCGTTTTTGGGTGAACTAACAATTTAATAGATTACACTTTCGTAAAAGTATTTGCAATTAGTTTGTAAAAGCTGTCATTATAGCCTATATGCTTGGGCTAAATTTTCATTGCATTTATGTGTTCAACTTGAGCTGTGCACACCAATCGGT[A/C]CATGAGTTTTTCAAGATGCCACAACATTGTGCAGCCTCTGGTTGTAGAAACTTATGAGATTTAAATTCCTGAAGAAATGGGTTAACCTTTCACAGGTGAGAATGTGTTGGTTTGTGTTTTTATGTATATGGCCTATTAACTCAATATTACAGTTTTTTAAACTATAGCAACGTTTT
LG22	ref-49577	56.196	Chr8	22294871	CATCAGATTGAAGATAGGAGATGTATCTATTTCATGTAGGCCTATTTGTTTCTATTATTGTGTATGTATCTATTTGTATCTATATGTATCTATTGTATCCTCATAGTAACGTGCCTGAACACACCTCGTTTTCAGACCAGCACGCCCATGGGCGCACAAATGGGCACAAATGCATTTGCTATTTAAACAACGTGGCGCAGGACGTGGAAACGATAACCGCGCCGGGCTGAAACTAGCAAAAAACACTTGCGTCGCATTGCGCCAGGTGTATGATAGGGGCTCAAAATCTCCATTCTGAAATATCAAATGTCCAGACAGTGACTCAGCTTTTATGAATCATTGAAATATTGGTGTATGGGTATAAATAGTGTTCACAAACAGCTGTTAATATACTGTATTATTCTCAATTGGCTTTATTTTAGTATATCTTAC
LG22	ref-9847	56.428			TTCGTTGGCCAATGGTGCGGTCAGGGACGTAGCACAAGAGTGTCATCAAGACCATACATATGCATCTGAAATAAACGTCATCAGCTTCGTCTTCAGGAATCCGCATGTTTGAGTGTATGTGTGAAAGAAAGAAAGATAGTGTTTGTCTACCCTGTTCATTTGAATGAGTAAAATGTCAGAGGACAACTTTTCTTGCAGTGGTGTGGGACTCGACCTCCATGCAGGCACTTTTGTCTCCTGCTCGGGTCGAGACCATATACAGACATTAAAAGGTTAGTTCACCCAAAAATTAACATTATGTCATTAATTACTCACTCCCATGTTGTTCCGTAAGACCTTCGTTCATCTTCGGAACACAAATTAAGATGTTCTGATGTAGAACCTGGAAATGCTGAAAGTCAGACAGCTCCAGGATTTCATCAAAAAATATC
LG22	ref-68578	56.53			AAGGACGTTAAGTAGTCCTGTAAGCGAGCAGGCTGAGTGTGCGATCTGGTAGGCCTGCCTTGCAATGCTGAAGATGGTGGTAGGGTTGTTGCTTGTGGAGTCACTGGATGCCGTGCTGGGATCTGAGGGCATGAAGAACTGAGCAGAGAGCAAGGAAGCACAGCGTCCTGGGTGTGATCTGCATCAGAAGGGCACAGTACTTTCCTCAGTCGATTGGCATGCCAACGGGAACCATCTGGAATGTAACCCGCACCCAGTCCAATGGGAAGATCCTGGGAATCCAAGCCTTTTTCTTTGCATTGAATCTGCTCTGTGCTTTGAGCTGTTGTCCACATACCCTGGCTTTCACCACAGGGTTTGCACCGTGAGAAGTGGTAGGGCAAAGTCTGTGCAAGGGTAACTCCAGCTCTCGCCCCAGCATCAGTTTTGCT
LG22	ref-23272_28	56.588			ATTTACCAAAAATAACCATAGAAACATATTCATCAAATATAAAATACTTCAGAAATATTATCCATGATCAGTTATACAATACGGATTAATAACATGACACAGCTGAAACGAAAAAACTTATCTTACCGTTTCGGAGATCAGGACAAAATGGCGTCTACTAAACGAGAAAGGTGCTCTTTGCAAACATGTCGCAGCA[C/G]CGCTAGTGTTTAGGATACCCTCCTTCAACAACTGTATATACCACAACACTTATCACATCAGCCTAACACAAACATAACGCTTCATAAACCATACATAATCTTAATTCCTATACTAGTGTAGCTAATTTACAGGTATTTAATCAATTAGCTCAAATTATGGGTAATATATATAT

LG22	ref-40981	56.887			AGGAAGTTCATGGGGCACGAGGCTGGATTCTGGGACCGGGGCAAACTCTGGAGTGGACTCATGGACAAGAGCGGGCTCTGAAGAGGATACATAGGTTGGAGAGGGCTTTGGAGCGGACTCAGGGGCTGGAGCGGGCTCTGGAGCAGACTCATGGGATGGAGCGGGCTTTGGAATGGATTCACGGGCTTGAGCAGACTAGGGGACTGGAGCCGATATGTGTGCAGCCAAACACACACCAAATGGCTGTGGCCATAATGGCCAGCACTGCAATCTCTGAGGGCTCAGGCGTGGCAGCCATCTTGGCTGAGGGCTCAGGCGTGGCAGCCATCTTGGCTGAGGGCTCAGGCGTGGGAACTATAGGGCAGGAAGATGCATAAAGCACAGGGCTAGCAACCCTTAAAACAGGACATGCTGGTGATAAAGCTGGGCCA
LG22	ref-24053	57.006			TGCTCGCGCATTTGATGCGCTCTCGACATTTGTCAGTAAAATAACGCAAATGAAACCACCTTAAATGAGCTATTAAAGTGAGAAGAAACTCATGTCTGAAAGTTGCAATCAATTTTTTTATTGGTTAAAATGAATGGAGAATATCTCGTGTTTTTTCGTAGGTGCTCGACCACTTCCGTGCGTGGCACTCGAGCATCCACGGGATCGCGCATGTTCGGTGGAAATATTTGCCATTAGGATGGATGATTACATGCTTTAATTTGGGCAGCTTTGAGCAGCTCTTGAGGTTCTTATCCAGTATGTGAAATGAAATCCTTGCTGGTGTATAGTTGTGTTTACCGACTTTGCTGAGGCCCGCACTTAGCTATT
LG22	ref-71765	57.011			GATCCTTCGTCAACAGCACAGGCATGTGCCATGGTGCTCATGTGCACAGCGTCAGCCAATACCGAGCCAGAATTATGACGTAGAACCTGGAAGCGCTAAATAAAGTCGTTATTGTCATTATTCTCATTGCTTCATAACATTAAGGTTGAACCACTGTAGTCACATGGACTATTTTAACGATGTCTTTTCTATCTTTCTGGGCCTTGAAAATGACTTTGCTGCCTATGTGTGGTTTAGATACCTCTCAGATTTGATCAAAAATATGGGTTTGGAACAGAGGGTGAGTAATTTATTGACAGAAATTTCATTTTTGGGTGAACTAACCCTTTAATGTTTCAGGATTTCAGAATGAGCCATTTTTGCTGCTTAGTTTTATTATTGGTCTTTTTTTCAATGGGTGGAACGTTCAGAAAGTTCATGGCACATTTTAT
LG22	ref-3034	57.137	Chr6	10043079	AAATATAATACAGTAAGTGCTCATTTACAAAAAAAGATATCAAAACTCTGGAATAACAATTGGTAAGTACACTCTGCTTTTTGTTATCATGCTGTGAGGAGCTGGCTTTCAAAGTTCGGGGATTATTCAGTTGGAATAATAATACACTATATAGGTTGTTGCTAATAAAACTAGTGTCAGCAAAAACTTTGCACTGCAGAGATGACAGAAGTTGCATCTGAAATAGCATTTAGCCATTATTTACAGAGACTATTGGGTAACACTTTAGTATAGGGAACATGTATTCACTATTAATTACAACTTTTCCCTTAATAAACACCTAATTTACTGCTTATTAATAGTTAGTAAGTTAGTTGTTAAATGTAAGTA
LG22	ref-3030	57.139			GCGATTTTTTGCTTTTATGATACTAACAATGCTTTGTTTTTTTGATGCTATGATCTTTTTTATGCTAATAATCAGGTGTTTTTAAAGAAGGAAGACAAAGGGTACTTCTCAAATGGAAGGCTGCATCTTATACGTAGGCTGCATATCTCGGCTGCTACGTCATCAAACGTCGCTGAAGGGCATCTCAGTCGGAAGGATCCTTGGAAGGTAGCCTTCGTTTCATGTCCTTCTTTCGGCCTGTTTTGAAGGATGCATTGGATGTATCCTTTGTGGACATAAATGACCCACAATCCTTTGCACAAGAAAATAAACTGACAGTAAATGAGTCCGTACTGAGCTTGTCTGAGTTTATTATGAAATGTAAGACCA
LG22	ref-68826_29	57.41			TACCAAGACAGAGACTAGTAAACACTAATAAACTTGACCATGTACTGCAGCCAAGGATTTAATCTCTTAATATCTGTTTGTGATGATTTTGTGTTTGTTTATTTTACAGATCAATGCTGAATTTTCTGAGAATTACAACCTTAAACCATGATTCCTGGCTTTCTTAGACAGGCACCACAGCAAGCTGATCGAGATCA[A/G]CAGAAACAAGGGAGGAACTGGCAGAGAGGTCTTAATATTAAGTCAAACAGCTCTATCAGCCTCATCAAAATGCACTTCTGCACTAACATATTACAACAAAGTAAACTGCTGAAAATGTCATACTAATGCATAGGCCAGCCAACATGTTGTTTGCTCATTGTAATAAAACCAA
LG22	ref-45313	57.65			AAACTGTGAACGACTCTCATGCTAACAGTTA
LG22	ref-62557	57.752			AGCTGAGACTCGAACGTGTTGCCTTGTGAGC
LG22	ref-21913_9	57.96			TTTCTGTATGTAATCTAATGTAATAACAACAGAGGATTTTCATGCTACTACTGTAAAACAATGTGAAGCTGACTGTTTAGACATTGGCTTGATTCACTGATACATAAACAACAATAAATGATTTTGTGACAAAGAATGTCTGACTTAATGTCTATCCTCGCATATCAGAAATCACTA[C/G]GGCATTACTGTCGGGTGCATATATTTGCATATGCATATGTATCAGTCTGTCTGAAATGTCTGCATTGAATTGCGACTGGTTTACAAAGGAATCCACAGTGAAATGAACCAACCAAACAAAGAATGCTCTACTGAGACATTCACATAGATGAAAATAAACAGCTACTTTCCTAGCATTAGCAACCTTTAGAAG
LG22	ref-15837	58.173			TGACTTTTGAACGTTAGTGTATATTAGTAAATCTGGGGATGGCGATTTAATGCATAAATAAAAAGAGTACAATTATGATTATGATCAATCAAACCACAATTATCCAGTGAGTAAAGAGTGATTCACAGTCATCAGCCTCATGCTCATTCCTTCTCCAACAACTAGCATAAATTGCTCCTGCACTACAATCCAGATGCACACAAGGTACTTTTAAATCTTACTGTGATTAACAGATTATATAAGAATTATATTTTTCATTCTGGTTAAGCTATTATGTACTGAATGAAACTGACAAAATCTGGATTGAGTAGAAGAGACGGAAGAGCTTCTGGGTGGAGATATTGGAAATTTGAGATGATCTCTATAATC
LG22	ref-36005	58.327			GCATCTGACACGAAGTGCTTGCTAATTACAG
LG22	ref-61415	58.642			ACTTTCCCCCCGATTGCTCTGCTATCAGGAG
LG22	ref-71853_2	58.688			T[A/T]AAGAAGTTCGATGTGTATGCTTCGGGGTGT
LG22	ref-38147	58.699			AGCGCCGAGCGTGCTTACGGTGTTCTTCCTTCTGCGGCCGCAGTTAGAAGCTAAAAGAGCATTCTGCACGCTGATGATGGGCACAGTGAATGCGTGCAATGTCTGGGCAAAATCCCACGCAGAAGCCGCGCTCACTGAATTGACCTGCTATGAGACTATGAGTCTCTCCTCTTTGCGCTCGCAGATAGCGTTCTTTTCAGAACGCGACTCCGCCCCTCGTGCCCTCCCGTTTTCTTCCTCCCAGGAGCCTGTGAGGAAAAAACAGCGGGGCAGAGGATCTCAGCGCTCGGATGAAAGTGATCTCACGTCGGCTCAGGTCCCATGTGCCTCACTTTCTCCACAAAGAGAGCCTTCCCCCTCCACCAACTCGAGCGACCTGGTCTCGTTCGGGGGATCTGAAGAAGAGCCACTGGATGGCAGCATGTCCCTAG

LG22	ref-20064_6	58.842			GAAGCGGCCAAAGGGTATGCGGGGATACCCCCAGTGGAGCGCTTGGTTGTGATGCAGTTGTGTTCTCAGAGCACCTCCACCTGGAGTGGTGATCCCAAGCTCCCCTCCAAAGCCTGTAAGTTCTCGTCCTCGCTTGTGGCCAAGGCTTACAGGGCTGCTTACAGAACACACCCTCCAGGTCTATCAGGCCAAGGTGCTCAAGGAG[C/T]TACACGAGAGCAGTGCTGACCCGGGAGTCTTGCAGGAGGTGCGCACTGTTACTCACTGCATGCTCCTTGGGTCAGGTGATGTCCACTTTGGTGGTCCAGGAGCGCCACCTATGGCTGAACCTGGCGGACATGAGAGAGTCTGATCGGACTCAGCTCTTCAACTCTTCGACCTCTTTGGCGAAGCGGTCGAGAGTTTTGCCCAGCAATTCTTGGCCGCCCAGAAGCA
LG22	ref-26077	59.017			ATTTGAAATCCGAACCTTGTGCTGTGTTCCC
LG22	ref-41216	59.276			TGCACAACCGCGATCAGCTTGCCAGGAACTG
LG22	ref-24400	59.331			TCATGAAGGTCGAGTGCAGTGCCACGCAGAA
LG22	ref-71296	59.439			GCTTCTGTGCCTGCAGCAAGCACTATATGTGAAATCGTGTGTCCTATTTATTGTTGTCTGTCTATCAGTTACAAGCACAACATATATTCATTATGACAAGCAAACAACAATTCAGAAAGTGTGTTCATCCCTGCCTTCGTTTTATTACAGGTGGGGATACACACCAGATGTGTGCTGTTTGTTTGGGAGTGCAGCACGCTCAACACCTTTCGAGGGGGCTGCTTGTGAGCACTGTGAGCTTCTCCCACTGATCCCTCTTTGAATTGCTCTGCGAATTTCTTCGCCTCTGGTTGAGACAGCCACAGTACAGTTATCCAGTTCTGAGGAATTGGATGTTGTTAGTGTGGCAACAGGAGAAACTGAAGACTCGTCACTTGTGGAGGTTTTGACTTGTGCTGTGGCCAAATTAAACACTGAATGGCCAGCCGAGA
LG22	ref-62672	59.572			TGCCAATGTCTATATTGTAACCAATGCAAATTTTTCTAAACTAATTTGTGCGTAAACAGGCACGTGTTTTCTCCTAAAATACATTCGAATAGGAAAGTTTGATAACCGTGTACATTGTTTTTGAGTATATTCAATTGTCTTCATGCCTTTTATTTATGCAAGTCTTTTGTAAATGTGTGAAACAAACAAGCTAAATGTGTATTTGTGCTACGAATAGCATGCACCAGGCCAGAATTCCAACAGTGCATCTTCTCCACTTGAAATGTTTGAGCTAGGATTTTAAAAGTTCAAGGAAATATGACAAAGAAACTACACTGTGACTGCTAGGAGGAGACTCAAAAACTCCACATGCACATGCTTAACCTGATAATGCATGTGGTCACGTAAACGCGTTAACCCGTTTCTTTACAGTCTAAAGTCATAATCAGCCA
LG22	ref-6554	59.624			GGCGCCTCAGCGAGAGCGCTGCATCACATCA
LG22	ref-71853_14	59.996			TATTTAACCTAAGATATTTCAAATTGATCCGAGAAAGCTAATTTAAATAGGTACGTTTTAAGACGTGATTTAAAAGTAGCTAGACAATCACTTTCTTGTAAAGATTGTGAAAGAGAATTCCAGAGATGAGGAGCAGCACAGCTGAACGCCCTGGACCCCATAGTGACTAAACGAACAGAGGGTACAACAAGAGAAACAGATAAAGAAGTTCGAA[A/T]TGTATGCTTCGGGGTGTAGATATGAAACAATTCGGAGAGATATGAGGGTGCAAGATTGTGAAGAGCTTTGAAAGTGAGAAGCAGAATCTTGTACTTGTAGAGGGCACATAAGTCTGAAGTAGTGAGTTTAGGTATAGGGGTGCAGAGCCAGTGGTCCGTCTGTAAGCAAACATCAGTGCCTTCAATTTGATGTGAGCGTCTATTGATGAAGAGAGGT
LG22	ref-4496_28	60.536			AAATATATAAATGATTAAAGAAATCAATAACATGTATTAGAATTCATAATGAAGAGATTAGTAAGATTCATAAAAGGAAAAATTAATAATCGAAATAAATGAAAGACATTTCTTTAGTATAATTAATAAAAACTCCTGCATTTCTGTTCAGTGATTGATTGCTAGTCTATAAACGTGCAATATGCAAATACAAAACGTGAATAATTGATCCGAGGCATATGCATAGA[C/G]TTGGTAAGATACATAGACTAAAGTTAGTCTAATGTATGCTTCTGAAATGAAATGGGGAGTTCACCCCCCCCCCCAAAAAAAGAGATCTCTGTTATCATTTACTCACCTTCATGTTGTTCCAAACCTGCGTGACTTTCTTTCTTCTGTGGAACTTACAAGAAGATACTTTGAAAAAATTCACAAAATGGTTTATTTTTTTGTCCA
LG22	ref-62029	61.129	Chr2	51352616	ACCTGTGCTGGGCAGGAACACCATGCTGGCTGATGTGGTGGAGAAACTAAAGAAGACAGGACTGCATGCTGGGCCCACCTCATCCGATCCAACCCAAGCTGAACCCGGGGATGTCGAATGTGATGTGTGCTCTGGGAAGAAAAACAAAGCCTTTAAATCCTGCCTGGTCTGTCTGGCGTCTTATTGCGAGACTCACCTTCAACCTCACTACGAGTCTCCTGTCTTTCAGAAACACAAGCTGGTTTCTCCTTCAAAGAAGATACAAGAACAGCTCTGCTCTCGGCATGATAAATTGCTAGAGGTGTTTTGCCGCACAGATCAGCAATGTATTTGTTACCTTTGTTTAACGGATGAGCACAAAGGACATGACACTGTTCTAGCTACAGCAGAGATGAATGAGAAAAAGGTAAAATTGTAGACCATTTCTAGAG
LG22	ref-35444	61.964			TAACAACACCTCATCTATTATGATGATGCCAGCCTGTGTGTGTTTTTCCTCAAGGGACTTTCCATGAGTACGTAGAGCGGTTATTCCTCAACAACAGTTCTCCATTCACCATCTGCGAGAGTGAGGAGGAAAACAGCGAGCCAGCCGCTGCCACCAGCCCAGCGACAGCGACGCCTGAGCCCACCACAGATCATGAGCCTGAGTCTGCCTTGATAGAGGTGCCTGAGCCGAAGCCAGAGCCACAGATTGCCCCGGATTATGAAGGGGTGAAGTGGAGAAACGCTGATCGACTTCAAGAGTGAGTTTGTCATGCCTGTTTCCCCATGCACGGACCCTCTTTCGTCACTCAGCACAGTGCTATTGATGTCTGTGTTCCCGCCCTGCACTCCCACCGCCTCAGCCTGTTCCGCGTCGGCCAAGAGAGACATTCA
LG22	ref-16202	62.826			GTCTGCTCTTTGTACTCATCAGCAATATTGTAATATCCAGTTCACTTGATTTATCTTCATGGCATCTGTGTTCACTAGAATTCAAACACCCAACCTGTGCCTCTTGAGTCACTTTTGAAATTATTCATTCATCTGCTCAAGGGAGAGATTGATGCTTGTATTTATTAATAAAGCTGAATGCAATCATATCGACCGGCACATCTCACCAGAGCATGGACAGCAGTGAGCATGAAAGCAGAGTGAAACCACGAAACACAAACTGTGATTATTATTTCTTTTAGTTAATTTTCTTTCACTGCCAATGCATCAAAGTCTTGATTTTGTTTTTGGGTGAACTAGAATATTTGGGTCTATTAAAAGATTTACAGG
LG22	ref-13338_4	63.407			CCT[C/T]AAACTGCGAAGAGCCTGCAGTCAAAAAG
LG22	ref-70964_5	63.767			GTGT[G/T]ATAATCGACACAGATGCTGCCCATAAA
LG22	ref-35957	64.523			CAGAAAATGTCGAGAAAAATGCCCTACGTTT

LG22	ref-1667	65.039			ATACTTTTTTCTTCAAATTCCCATTGTTTGTTGTACACACAGTAATGCTAAAATAAAATAATAATGAAAATATACAATAAATTACACAAATAGAACCAAACAAAATGGTCATATATAATTTCAAGCTTTTTTCCCCATGCCTCTAAATCTGCTTTCTTGGATATTACCCACAACTCCTTGCTCCACGAAAGAGGAGGTGTATAAGACAGCCGATGCGGATGCATTGCGTGTTTTGGGGTTGTGCCTAGAAGAACTGAGGCAAAATTTCATAATAAAATAACAGTAAATTAATTTGCTACTTATCAGATTGTATTTTATTAACGACTACACCTACCCCCAACCCTAAACCTACCCTTACAATACTGCAAATACAGTTATTAAATAAAATAAATAAATATTAAAATGTCCAGTGGCCGTATAGCTGTATCCCT
LG22	ref-37485_2	65.19			T[G/T]TCGAACCTCGAAAACGCTGCAGACCCCGTT
LG22	ref-4448_10	66.717			CATAGAGGACACCTTAATGAACACATGAGAATTCACACTGGAGAGAAGCCTTTCATATGCCATCAGTGTGGACAGGGTTTCACACATAAAGGCAGCCTGACATGGCACATTAAAATTCACACTAAAGATGGACTTTCCACATGCCATCAAAGTGGGAAGAATTTTATAAATGAAAGAA[A/G]GCATAAGCGTCGCACAACAAGTCACTCTGTAGACAAGCCGTATGCATGCCATCAGTGTGGAAAACACTTCATTCATAGAGGACACCTTAATGAACACATGAGAATACACACTGGAGAGAAGCCATATACATGTCCTCAGTGTGGACAGAGTTTCCGACATAAAGGAAACCTGACGTGGCATATGAGAACTC
LG22	ref-44085	66.914			CTGAACTTCAAATGTGATTCATATATGTCCCGTGTCGCTGCATACTGATATCAGTCATGCTGAAGTCTTGTGGGTGTATATTAATGTGTGTATCCAAATGTTTTATTCAGACAATTAATTTCTGTTAATGCTGCATATGTTAACAACAAAGTTCAAAACCATTTTTCGGGACCCATCTGATCTGGCCGTATGAGGACGATATAAACCATGCGATGTGATCGCATACGTTTCTGACTCTCTAGCACAACTCGGGAAAGTTTTTATGAGTTTTATATGCTAAACTATGTAACCAGTGATTGTAACACCAGTACTTTTTTTAATTTCGTATAATTTAGTCTAAACTAAGCAGTCGTGCATGTCCAAACCAAAATGCATACAGTCAGTCTTCTCTCTCTCTCTGCAGCTATTAAAAGTGTTTCAAATCAACAGCT
LG22	ref-9386	68.24			GATGGCAATCCGAAATCCTTGCCGTTGTGGG
LG22	ref-32524	69.975			ATGTAAATTAATAAGTATATCCTTTTATATAAAAGTATATCCTTTTATATCCGGCAAGTTTGAGCGCGGAATCTTGGGACATGTGGTCTTCACCTCACAGCTGATGGAAACAATCGGGATAGGACTCGGGAAGAAATCATGTTCAAGGATGAGATTATTAACGTTACTGTAGTATGAAGCAGAGCAGGACCGAGTGTTGTGGGAGCTGAACGAGGCGATTGCACAATACATGCCTCACGAGCAGCAGAACTTTTATTATGTCACAGTCAACGGCACCGCTTCCGCTTTTCCGGTCATGAGTATGAGGTAACACAGCTCTGTTGATCATATTAGATACATTTGAGTGTGTTGAAAATTATGTTATAACATTACACTGTGCGTTCGCTCGGCGGCTGCTGTGAGACAGTTGTTTGAGACACACTGCAGTAAGC
LG22	ref-17772	70.771			CGCCCAGACTCGAGGCTCATGCAGGCTCAGC
LG22	ref-3791_1	71.013			CTTGCCTTGAGAAGTGCTCTACAGAGGAGCTGTCTGATTATCTTCTGGCTTTGTACAGTTCCTTCAGCGCCTGCTAACACATGCATCTTACCTTCATGAGCTGAGTACAGAGTAACCCAATGTGTTGTATGTATATATATATAGTAGAGATGCACCGATACTGAATTTCTCTGCCAAAACAGATAACCGATTATTCAGAA[C/T]GATATCGGCCGATGCCGATGCTGATAACATTGGAAAACATTCCTGAGGTAAATGTGACATTATGTGTCATATTGTTTACTTATTGACGTTTTTTCGAGGTTCACAGATTAAGTGATTACATAAGACATGAGACATTCTGGTAAGTTGCACTGTATCTTTCAACATACCTTTTGATGTTCATGCATGTTTTTCGAGATATAACTTGTATTAAAGTGGAAAAAAAGACTCGCG
LG22	ref-7726_17	71.672			TCAGAGAACGTTTAAGGTCCTAAAAGTGTCAACAGAATAATTCGAAAATCACTTCTGGGTCTGTGACCATGTAAATCAGAGAACGTTTAATTTAATAATATAGTAACACATATGGATGAAAACAATTCAGACGATTTAAAAGAGTTTTATGTTTACATGAATGTTTTCAACCCTCATTCTCGTGCCCAGCTCTTCTGTAACGTCATTACACGAGACG[A/G]CTGCCAAGCACTGAATCAATCTGCGTCAGTCATGTGAACAGCAACATGTTCAGAGAACAAAGAGTCACAAAAAAAATTCTGATTAAAATAATAAAACCCTTTGGTACAAAATCAGTGATTTTTAGCCAATGCATGAACTGTTATGGCAAGGTTTTCACCCACTCACTCATAACAGACCTTCACATAAGTTTAGTTTCATGCTGCTAACTATCCCA
LG22	ref-66955_28	72.36	Chr2	1260720	CATGCAGCACTATTTTATGTAATGAATGAACTTATGTCATTAACGCTGAACTTGACACTAACAGCGCATATAAGGTATTACATATGTATAATAATGGCATATATTTGTACAATACCAGTGATATAATCGAAGAGTTCAGAGTCGATCTGTGGAAACTCGTTCTTCAGTATCTCCACATACGCCGCCATCATACACACACCGGGCCGGAAACGAGGCGCATGCGCACA[G/T]GCGCATCAATAACACGTGGACGAACGCCGCAAGAATAACTCGAGAAGTGCGTGTTCCTCCAGTGAGCGATCTGCGGCTCAGGAAGAGTTGTTAACATTCAAATCTCACGACATTGTGCGCTAGATAGTGCACTCGTCTAATGTACTAAATCCAAAATTGGAGACTGACATACAAGTGGAATGTAATAATTCTCTATAAACGCCT
LG22	ref-46703	72.678	Chr2	57217488	CGGACGAGTGAATTACGACTTTAGAGCCATTGCCGTGACAACCACGATCCGGGGTGGGGGGGTCACAGGCTTGTGTCAGCAAATTCAATAAAACGACAAGGGACAACACAACAGCGCACACTGTGAGTACACACACTACTGACAGAGCAGAACCAGCAAAACAGGAAAGAATTATTCAAGCACTACCGTCGCTCTACTGCAGACGAGCTGCTCGCACACAATGAGGACGGAAAGTTCATTACGAGCAGCGTATTTATCCAAACACTCAACAATAACGCCACTTCCTGTAGCCAGTGGATGATCATCTAGAGGATCGCAGCCATAAGAAAGGCTCTAGTGAGCTCCCTACTGAGACAGCATCCACAGAAT
LG22	ref-52850	73.218	Chr2	57719999	ATTATTGTGAAGTGTGAGACACAGTTTACACTCATAAGACCCCAAGTGGTTTTTCATGAAGTATGGATCTTTATTGATGTCTATCGTCTCGAGCGCGAGCTGCCGCAGTCGCTCACGCCGGTCTCGGTTGCTCTCAGACGACGAGGCGACGCCGCCGCTGCCCGTTTTCCCTCCAGCTCTGTGCTGGAAATCCATGATGGCGGATCCTCACAATACTGCTGCTGCTGAAACCAGCAAAGAGGGAAACACATTTAAAGGGGATGGAAGAATATGATGGATTCTTGATCTCAATTTCATGCACGGTCCACTCAGTCACTGAATCGGAAATCAAATACTTCTCTACAAGAGAAAAACAGTATGTGACAAAAGTAGTACGTCCAAATACATTGGCTGCATCCGAAATCGAATACTTCTCTATTATATAGTATGCG

LG22	ref-34366	74.185	Chr9	10957919	AAGAGCGATAACTATACAGATAACTATAAAGATAGTAACGATAATAGCATCCACATCAACGTAGGCTACCAATGTCCTGTTTAATATATGCGTACGTGGCGCGCCGCAGTCTGCAAATGATCGACTTGTGGGCTGGGGGAAAAAATCGTTCTGAAAGTGATTCCAACTATATCGCTTCTGTATTGTTATCGTTATGGTGTGGACTATAAGAGAACTTGGTGGGGCTTGTCTTTTTTGTAAATAAAGTAAAGACTATCCCACGTAGACTGTTTTCTTAAAGCTGCAGTCCTTAAGTTTTTCCTCTTTGTCGCCATCTCAGTTTGAAACCTGCAATTGCAGTTATTTGCAGAATTATCATCTTTACTTGGG
LG22	ref-23608	74.83			AAAATCGCTCCTCTTACAAGCAAGCGGTAAGAGGAGCCTCTGTTCAGCAACCGGTTGGCTACCCAATTACCACCTCATCGGCTGCTTTCTGGAGCTACTCTGTCCAAAGGTACAGTTTACCCATTGTCCATCCCGGAGCAGAAGGCCATGGAGGAGTACATCCAGGACGCACTACAACAGCAATTCATTCAACCTTCCACATCCCCTGCCGCTTCCAGCTTCTTCTTCTTTGTCACCAAAAAGGACGGAGGCTTGTGTCCATGTATAGATTACAGAGCCCTTAACTCCCAGATGGTGAAGTACAGTTATCCTCTTCCTCTGGTCCCTGCAGCATTAGAACAGCTGTGTGGGGCTCTCATCTTCTCCAAG
LG22	ref-3576_23	77.177			CAGATTAGCTCGAGAACAGTGC[A/G]TAGAGAGCT
LG22	ref-39120	77.552			GTTAAGACTTCATTTCATGACATTTCATTTCAAGATTTCATGACATGATGTCTATAGGCTACTCACGGTGATATAGCTATATGCTGACGTTTGGTACTCTAGCAATTATTAAACAAAACCGTATGTAGGCTATCTTGTATCCATCTATGTAGCCTATCTGTGGTACTTTGCATCAGTTGGCATCCCATTCGTCTAAAATAGTCCGAATATAAACACTTATAGGCGTACCGTAGTGATTCAGAATAAGAAATATACACGCTTTGGAAGATGAATTAATGAAAAAAGTTAAAAAAAAAAATTAGTGTTAAAAGTTAGTGTTCATTTTACATGGTTAAATGACAGTGATAATTCAAATAATAATTAATGTGT
LG22	ref-47936	78.776			GGAGTGAAGTCGATCTGGTTGCCATGGAGAC
LG22	ref-70089	79.454			ATAAACCATTAAAGACAGATCTACTGTAAGCTACAAGGTTGTTAGTTGATGGCATGCTTTTGTTGAAGTCATCAGCCTCCATTATTTCAACTTATTTTAAAATAATGTTTAACAATGGAATCCTGAAACTAAATTACCAATGATTCTGCAAAGAAATCTCCACCAATCAGAGAATCGAGACAAGCAAGTCGCACCAAAAGACATCTTTAACGCTAACCCATTCCCATGAAGAATTGCAAATGATGTAATTGAGTCAATCTCCCTATGTTCCCAAACCACTTAATATTTGTGCATTTCTTCATGGGATTGTAGTTCTTTCCTTCATCAAAGCCATTAAGTACACAGCCCTATTGTCAAATATTTTTAAGT
LG22	ref-15803	79.808			TAGAAATGTATTCATATTTAACAAGTTTATAAAGGAAAATATCTAGCTTCCGCCAGACCATCTTCCGTATTCAACTTACGAAAAAGTGTAATGCCTCTCGCAGATCAAAACGCTTACGCTACATCCTACGCCTTCCGTGTTCAACGTATGAAACAAGCGTAATTGACGCGACGTCAGTTGCACTTTCTTCGTAAGGCGAATACGGAAGGTGGTCTGGCGGAAGCTAGATATTTTACTTTATAGCTTGTTAAATAGGGATATTTTTCTAACACAAATGCATCGTTTTGCTTCAGAAAGCCTTTATTAACTCCCTGTAGCCATGTGAAGTACGTTTATGATGGATGGATGCACTTTTTGAGCTTCATACTC
LG22	ref-34258	81.559			GCAATGTCAGCGAGGCAGCTGCCTAGGTTTT
LG22	ref-51985	82.392			TGCCCAGAGACGACCACAATGCATCCATGCA
LG22	ref-37968	82.697			TTGGTTGTTACGACGTCATTGCCTGTAGAGG
LG22	ref-8293	84.22			TGGTTTATGACGATGCCTTTGCTGTAATCGA
LG22	ref-66812	84.262			AGAATTCGCCCGAGCCCCCTGCAGATGGAGA
LG22	ref-20518_10	85.11	Chr3	48325475	TGTTCATGTTGATTTTTATTCATACAGTAAAAGCATATGTAGAACAGGGGCTGGGCTGTCAGTGTCCAAAATTTGTTCTTTTCCTAACACAAAGCTATATATGAAGTCATATGGCTTCAGATGATTTGAATTATTCTATGGTCTGTCTGAATACTTGATTCTAATTGGCTGGATGGTG[A/C]GCAATAGAATCGTTTAATGCACAGGTAGTTCCAAGTCAGTTTAATCACCGTTCTATATTAATCACTCGCATCAACACACACTCTCACACAGAAACTCAAACAAATGTAATCTTACACACTACTTTATCTGTTTGTCTGATTTAAAGTGGGCAGAGAGCTACATCTAATGAGCTGTAACTGATGAAAATAAC
LG22	ref-29824	85.306			GATTGACAGTCGATCTCAATGCCTCACGTTC
LG22	ref-14112	85.444			ACGCTGCAAACGATGGACATGCACACCTGGA
LG22	ref-46031	85.482			CGGGGTAGACCGAAGGTAGTGCAGAACTCCG
LG22	ref-13071	85.557			GTGAGAGATACGAGGCCGGTGCAGCACAAGG
LG22	ref-61765	85.637			GCCAGTTTCACGAGGGAGATGCTTATGTGGT
LG22	ref-33980	85.728			AGGGACAGGACGAACTGGTTGCAATCGAGGG
LG22	ref-3432	87.909	Chr4	41088559	GGTGCTGTGAGCTGTCAAGAACACGACGGGCACCGACTTCACCCTTGCCAAGCAGGTCCCCTGTTTCCGGACTGGCACATTATCAGAGGGGAAAAGAGGCAGTGCTAAACAGTCACCAGCTCCCTCTGAAGAATCATACCTCCCCACTAAGCTAAAAGAACTTGCTCCAGCCAGCCACAGATCATCTCATGGCTTGGACCTCAGAGACCTCGACAAGCTTGCCAGGAACATTGGCAATTTCACCCCAAATGTGCCGGGTAGTCCAAATGTTCAGAGTTATCTGCAAGATATCGATTTCCATCTGGAAATGAGACCCAATGTCACTGACAAAG

					ATTTGCTCCGAGCCACATCCAGCTCTGAAGTGCGCAGCTTCCTGGACCGGCAGCCTGCCCATACAAAGACTGATGCTCCAACTGATACAACCTGCTCCG
LG22	ref-32544_31	89.536			AATGTTGCGGAAATTTGCTCTTTAGAAATGGCTCTTTAGTAAAGCAACTGTGTTGTAAAAGGATACACCGAAGTGCACTCACTTGACGCTGCAGGCGGCTTGGGGGCGAAG<br>TGCTGACCAGGCTGCAGAGTTGCTTGAAAGCGGCGAGCTGATCTGGCTGCTGTGAAGCAGCGGGCAATCAGCGCTGCACGAGAGCGGCAATCTGAGCGGCGAGAGAGCT<br>GCAGGCTGCTC[A/C]TAGGCAGCGGCTGCTAGAGAGCAGCGAGCTGCTTGAGAGCAGCGAGCAGAATAAAGAGCGGCGAGCAGGTAGGCAAGCTGACCAAACTGCTGCA<br>GAGCGGCGAGTTGATCAGCGGCGAATTCCAGCTGCTTGCAGGTGAAGGCGGCTTCAAGTCTTGTAGATCAAGTGAAGAGATGATATCTGCGTCGTTGAAGGAGAA
LG22	ref-53360	90.751			TTATGTAGTTGCTGTGGAGTTGATTCAACTCATCGACTAGCATGTGACATCATGTTAATCTTTTGTTTGTTTGTTTTTGTTTGTTTGTGAAGCAATCCGGCGTAAAATGACAG<br>CATGGCAACAACAGTCTACTACAGTGGTCGGCAATAGGTGGCCCGTGGGCCAAAACTGGCCCGCCAGCAATAATATCGTGGCGCCGTTTAATATTATCACCGTGTCTATAC<br>CAGACACAACTTTTTTCGAGTCGCATTGCGCCGCAACAGCTAAAAGCTGTCTATACTGAACGCGACAAACCAACCGATGCAAAGCACTTTGACTACGTCAGAAACAGAAT<br>GGGGAATCCGTTTACTGACGGGAATGTGTTGTGTC
LG22	ref-17667	99.839	Chr2	53012617	AATTTATTTGAAATATATTACTTTTTATTAGGGTTCTCTTAGAACATGTTTTGTTTTTTATTAACTCCCCTCATCTTCTGTTATTCAATTAAACTGGGATATGTGAAAGCATTT<br>TAACATCTGAAACACGTCACATTTATAAAGTTTATTGCACCTTCTGGCTGCTTTTCATATTGGAGCAAACAAATGATTCTTCCTACTGTTTCAGTGTGACTTTCATGCCATCT<br>GGAAATGGCTCTCAGCGCCCTTGATAAAGCCAAGAAATTTTTTGCTTTTTGGCTCTAACGATATTTTTTAATGAGAATATTTGGCAGAAGTGTGACAGGTCTTTAGAGCTAG<br>TTGATTCAAAGTCCAGTTGATTCAGGCACGCTCTCATTACCTTTCCACCCACTTCTCTCTCTCAGCAATGAGCATTTCATGATCAAAGGATG
LG23	ref-38331	0			AGCCTCATTTTCACCTCAAACCTGTGATCATCTGATGTTCTCCCTCAGTGTTTCTGTCTTGTTTTCCAGTAACATTATTAAATCTTGTCTTTGTGAAAAACTGACCTGTTCCTG<br>CTGTTTTCCTAAAAGAGAAAGACGGCAGATCCCGGCGAGGAGTTTGGCAGGCGTGTGCCGGTACGGCAGCAGACTCGAGTGTTGTTATGGATGGAAGAAGAACAGTAAA<br>GGACACTGTGAAGGTGAACAAGTCCAAATTAACAACTCTTTTAATTAAGAATGCAAGTGTCAGTGATGGAGAATTTTTCAGTTGAATGTAAAACATGGAAACAATGGGAA<br>AATATTCCAACCGTACACTCTTGGTTTGGTGCTGTT
LG23	ref-17934_6	0.423			AACTT[C/T]GTGCCGAGCGTGATGCATGTGCACTG
LG23	ref-8754	1.504			AAAGTTGTCAGAGTTACAGTTCACGATAACTCCCAGGTGGATTAAACAACAGAAAAGCAACAGAGAATCTTCTTTTGCACAGCACAGCGAGAGTATATATATATCCATCA<br>CATGAGCATGCTTTATCTGAACTACATTACCTATGATGCATTTCACTAAGGACTACAACACCCGTAGATATGGTGCGTTCATGTCGGAAAGATCGTATTACGAGCTGAATG<br>CACATGAACGCCACCACAATATCGTAAATACGAGTGGGGAGCTCGGGATTTTCTTTAAGCCCCGATCTGAACGAGTTGGGGGCGTGTCAGTGAGAAACATGGCGAAATAC<br>CACAATACTTATAGGTAATTAGCAGTGACATTGTCAGGCTTTTCTATTTACAACAAAATAAGCAGATTCCCAGCAAAAAATACGATAGCATCATAATATA
LG23	ref-29550	1.985			GTGTCCAAACTTTTGACTGCTAGTGTATGTGGCAAGGTATTTGCTCTATTGTAGACTTTAAAACAACTAGTCCAGTGCCTCAATCCTGTACTGCCACTATGCCCGATAAGTT<br>TAATCACT
LG23	ref-70593	1.989			AGCAGAAAGAGGTCATGGGGTTCAGACCCTCTCCTCATTTCGCAAATCGCTTTATTTAAATATTCCCTCATGATATTCCCTGCAAATCGTGGATAAGCAGTTGTTCCTTCCC<br>TTGCACTGGAATAATGTAAAAAGGAGCACGTTCGGGACGTTTTGATGACGTATCACGCACGTTGTCCGCGGTAAAATCGCTTTTTCTCCCAGTCACAACCGGCGAGCAGTC<br>GGGAAAAACTCTGAAGTGAAGTTCCAGCTCTGAGGAGGATCTGAGTGACAGCTGCAGGTTCACGCGCATCACTGCGCCTCTTCCACGCGCGATGGTTTCTGCTGTTTTTAC<br>TCCCATATAGAGTGTCCGGAGGATTCGGCGCGAGT
LG23	ref-45844	3.095			TGGAACTGTGCTTTGTCGCTCGGTAAGCCATCACAGCATAAGGAACCATAAGATCCCATTCCCAATGACATCGCTCCAAGGTTGTGGCTAGGATTCTGTGTAGAGTGGCAT<br>TGAATCGCTCAACTTGCCCATCCGACTGAGGCTGGAAAGGTGTTGTACGGGTCTTGTCAATGTCTAGCAACTCACACATTGTTTGGAAGACCTCCGAATCGAAATTGGTGC<br>CTTGGTCACTATGGAGTGTAAACAGTACCCCATAACGGCAGACCCATTCAGCGACTATGACCTCTGCTACAGTGACAGCCTGGTCGTTGGGCAGGGGGTAGGCTTCCACCC<br>ATTTGGTAAAATAATCGTACACCACAAGAATGTAATGATTGAACCATTCTGTTTCATTCATGGGGCCCATCGAATCGACAGCAATCCTTTCCATGGGG
LG23	ref-68827	4.511			AACATCTTTTATGTGAAATATTTTATTCAGGAAAGTACTAAAAAAAAATGAATTTTTAATGTTGATCATATATTATACGAGCGAAAATGATTTTTTAACAAACATTTTGCAG<br>ATTCCGCAAGGAGTATGTGAAATTTTCTGTATTCTTAACAGATTCCTTGTCCTCTAATGCAGTGGTTGCATAGAGATCGATTTAAGACTCAAA
LG23	ref-53341_6	6.893			CTAGCCACATATATGTTGCTGTTATATTGCAGTATATTGTTTTATCATTATTGTTTATATTCATCAGCCAATCAGTGTGATGATGTCATCTTCCTGTGTCTCCAGAGTGATTG<br>ATCACATGACATTATTGTCTCAGGTGATTGGCTCAAAGCAGTGGCTGCAGATCCGAGCATCCGTGAGGAAAGATGGGAGATGATCTTCAGAAC[C/T]GGACGATACGGATG<br>CTGTCTCAGGTTGGTAAATGTGTGCAGATCATTAACCACTAGTTTAATCTTCACTGGTCGAGTGCAGTGCATTGTGGGATACATTAGTATTGTGTTGTATTCTGCACATGTA<br>AACAATAAATAGCAGTAAGTCATTCGGGTGTACAAAAGAATCTGTTTTAATTAATTTTGTTTTAATTTGTTCTTAATTTTGATGAAATTTAATGAGTTA
LG23	ref-3759	20.29			TCAGGCGCTGTATTAAAAACAGGACAAGTCAGAAAACGTCCCAAGCGTTTAGCGTGCACCATCTGACCTAATAGAAGAAGAATGCGCATTTAATTGAGTGCAGCGCTGCA<br>GGCTGATGGAGGAGACACTGCTCTTGTCCGCATGGGGCAATAAATCCACACCAACAGCGATGAGGAGATGCACTCGGATCAGAGAGAGGCTGATCTGTTCAATATTAATG<br>AAAGAAGGATTTATTGTTGAGCTGCATTTCAAATGTGCGGCATGAAACACTTGTTTATTGAACGTCTCTGGCTGCAAATGCATCATTTGTCTTGGTTGTGTCCATCACTGCT

					TTGACAGCTTTTATATAGACATTCATTGACTAATAAA
LG23	ref-68527	20.835			AGACCGTGCACGACACAAGTGCTAGTTTCAG
LG23	ref-72911	23.901			CGGAGGCTGCCGACATCAGTGCCTGACCTCA
LG23	ref-47126	33.285			TATTTTATACCGAGCGGGATGCCAACCGCGG
LG23	ref-55996	35.832			GAAATCTAAACGAGAGTTCTGCGGCTTTTAG
LG23	ref-44738	38.001			GTCGAATATGCCTAATCTAAAATATCAGATTTGAGAAGAAAATTGGATTCATGCCAAATTGAAGCATAATCATTATTAGGGGCCAAGCACCGAAGTTGCGTAGGCACTTAT TGTAATCGTTGGCGTTCTTCTTCCGTTTCTGCTCTGGGAGTCTGCTTGCGGGACAGTTATGAAATTTGGCACACAGATCGAGGCCAATTTGAACTGTGTCCATAGCAAATTT GGAGTCTCTAACTCAATCCCTCTAGCGCCACCAACTGTCCAAAGTTGCACTCACGTTTATGCTAATAACTTTTGAACCGTAATAACTTTTTCCGCCATTTTGAATTTTCCGAA AAACCTACTTTTTCGAACTCCTCCTAGGCCGTT
LG23	ref-38832	40.871	Chr9	14811125	ATCCCTCCTAGGAGTATAAAACCATGCTCATGTCAGTCATCAATGGCGACAGCTGCCCACGCCGGCGCCCACCTCACCTTTACGAGCGCTGTGATCTGGAGATAGGTTTAT TTCTGTGAAAGGGTATGGTCGTCTCAGTCTTCAGTTCACCATGGTAACGGTATCACCAGGAGGTTCATGCATGCGGTTTGAGTGGAGGAATACAGTAGCTAACAGGCCGCC GCTGTTCTCTCCTCCATCTACATATGAATCACTAAAAGGCCTTTTTAATGGAAGTTCAATCATCTCCCCTTTGATTCCCAAGATGCAATGTCCTGATTAAGATGATTGGATG AATGGAAAATGGCATGAATATGTGATTGGGTCTGT
LG23	ref-65353_25	42.833			TAAAATCGATCGATCTTACTGCAG[C/T]GTGCAAC
LG23	ref-39695	43.188			GATGATGAAGGTGATGATGATGGCAGCACTGGTTTCTGCTGCCATTTATCCTCTCTGTCTGTTGATCTCATGGGAACAGAAGTGACTGATGCTTCTAGAAATACCAACACC AAGTATGAGCATATAACCGAATTGTAGCGATCATGTAAATCTCACACTTTGCATGTTTCTCACATTGTATCTCACACTTACGCATTGACATACTTGGGTCGAATCCTGTGCT TGTACCTGAGACTTTTGAGATCGGTCTGTGAGGAACTGTTGATTGAGACGTGCTTGGGGACTCTGATGTGGCTTTCTGCTCACCTGTCTTTTGAAATCAGTAAAAAGGAAAT TTGAATCAATTCACAAAACAAAACACCACAAACACAATATACACAACTGTACTAGATAGTATGAGTCCTTTAATTTTAATTTATAAATGTATAATT
LG23	ref-69008	43.977			AGTGTGGATATACGTAAAATGCACAGGGGAACTTGTGATGAATCAAACATCAAATAAAGCAAAATGACATAAACAGTGAAGTAGTCATTAGGTGCCATGTTCGTTACAGC AGCATCTCGGAAAAGATTAATTAGCAGAAGTTATTACTCCTCCACCTGCGCGTGATGTCATTAATGACAGTCCTATATTGTTGAACCAGTTTGGTTTAAACAACAGAGGTG CAACCATGTTAATACAGTTTCAGGAAACAGTCATGACCGGTTGGTTAGTTTCTCCAATGATGCATCATACTATGGTAGTTAATCAGCGAGTTACATCATTGTACGGGAAAC GCAGCCCTGATCACATTTAAACATTGTTTCCAAGAGAACTGATAAAGTTTTATAAAACCATCCAGATTAGAGCTTGCCAGATAAAGAAAGTTCCTGCCA
LG23	ref-73247	44.213	Chr9	50785266	CGCTTTTGATCTCAGGATGTATGTCAAAGAAAGTAACAGTAATGAAAAAAAAAATGTCAAAAAGAAAACAAAGCGAGCAAATGGAAGAGAGAAACTGAGAATAAGAAA AAAAAAACTCTTGGCAGACAAATTCACAGAGCTTAAGGGTATATGATTAACCAACCTCTCCATTCTCTGAGTCCCAGGAGAGACGCGGAGCCTTCCAGGAGTCGGAGGAG CTGCGGTGAGAAGCCTGTGTAGTTGGAAGAACAAGCAAGGTTAATGAGTAGAAGTAAAAAGAGCAGAACTGACATTAGTTTAAAACAAAGAGTAGAAAGAACAGAGTCC GTTTGGAGAAGACGAGCGCTCGCTAGTCCTGTCGCATCAGAAATAGACACGGCTCATTCTCTTTGATTCAACTCCCTCATGACAGTTTAGCCGCTGTCTCAGA
LG23	ref-60593_28	45.478			ATGGCATTATATTTAGATCATGGCATGAAAGGTTGCAGAGATGAAAACTGTGTAGCCGATCACAGACATGTTGAGGGCATGAAAGCAGTGATCTCGTCATTGCAACTCAA TTTCTGTACACTAACAAATGCTTTTATCTTTACTATCAGTGCAAACGCAATATAACTAAGAGATTCGTTGAATTAAACTGGAGTTTTGCACTTCAGTCACGATAAACTTGCG CTGTTT[C/T]ATTGACAGCTTCAGCGCGTGCTGCTCCAGCAGCTCTCAAAGGGAGCTTTCTCTGATCACTTTCTTTATATAATTTAATCACCGTTAAATAACAGATTATTTTCA TCCTACTAAGAGAAACAACGCAAGTTGACGTTTAGTCACGGGTTTGATTTACTGACACACAGACAAAGAACATCTGACTGTACATGAATCATTAATAT
LG23	ref-9392	46.384			ATCCTAGGATCGAGCAGAATGCCACCTGAAT
LG23	ref-18085_23	46.668			CACTTGGAGGCGACTGAGATGC[A/T]TTGGCTTTA
LG23	ref-194	46.716			GCCGGTTTTGAATAGCAATTGGGCTGGTTTTGTTATGCAAATCTGGCAACCCTGATTCTAAGTGCGCACGGCAGTTTTGTCATCTACCACTTTAAGGGGCCATTCACATATT GCGTCTTTTGCACGCTCAAATTCGTTATTTCAAAGGTAGGCACGCAGCAAGCGCGCTCATAATGGAAGCGACGGGGTCGCGACACGAATGCGGTGCATTTTCCAGGTGCAT CGAGATGAAAAAATTTTCAACTTTTCAGAATGCCGCAAACACACCGCAGGTCATGTGACAAGAACCAAGCAATCAGCTTCGGCCTTTCTGTAACAAAACATCGAAAGCTC AGCCGAACAGCTGATCATAGCTGTACAGGGCGGGTT
LG23	ref-36021	46.904			CAGATCCACACGATATGCATGCCATTTGATA
LG23	ref-51794	47.783			GATGTGCTGCCGAATCCTTTGCTCTTGATAC

LG23	ref-18085_14	49.138	Chr9	23478264	GACATCACACACCCGTTTCATAGCATCAAATAACTAACTAAAACCAAACTTATTTAAAAAAAAACGAACACTTGAACTTACATCAGTGTGATAAAAACTGCCTAAAATGA<br>CAGTAACCATCTTTGGCAAAAAAAAAAAATATTTTAAGTATAATTTGATTGTTTAGTTTGGCGGGGTTTATGACCTATACTGCATCCAGCCACTTGGAGGCGAT[C/T]GAGA<br>TGCATTGGCTTTACTTTTCAGGACTTGTGCGGTACACCAAAGACTATAGATATAGAAAGACAGTTCACTCCTATTAGTTATGAATTGGAGAAACTGCAATGCGCAATATAG<br>CGGAATATGTCCCGCCTTCTAAGTATAAGAGCCAATCGCTGATTGATAAAGTCATTGCGTCACTGCAGCTGCTCTTAGAAGCTCCGGTTCCTATAGAAACAT
LG23	ref-39915	49.769			TTTCAGCTGCCGATAAAAGTGCAGTACTCCC
LG23	ref-29308	50.845	Chr9	16846527	CCTTGTCTGTAACATTAAACATGCTATGCAATAAAGCTCATTCTGATTCTGAAGAGAGTAAACCAAACCATTGTGCAAAGTAACTTGAAAACAGTCACGTGAATACGAACC<br>ACTATAAACACATTTGGTCCTAGTTAGTACTGACAATGTAACTGAGCTGAAATGTACAGTAGGTGGCTGCAACTTACTTGTAAGGCACTGGCCAGACCTGCTTTCCACTAA<br>CCCTAGAAACGACCAACCCCACAATCCAACAGTGAAGCACCGCGTAAATGAAGTGAACGGAATACTTCATCTCAGCACAACCAAAAACACTAAAGTGCTGTCTTTCTTGC<br>AGCTCTATATATTGTTTTGTCTCACATCCTTGTTTTA
LG23	ref-55857	51.023			TAGTTGATTTAATTATAGAATATCAAATAGGTTGTGATTGTCGTCTTAAAAAAAAAAAATCCTCATGTTTTTAATGGTGACACGTTCATGTAACCAAAGAGTAGCGCGCGCT<br>GCCAATAACGCACCTAAAAAGCTGTCAATCAACTCTTAGACAACAGTAGAACACAGACTAGCGTGGCATATAACAGAGACGTTAAAATCAGTGTTCACACGACACAGGTG<br>CCATTCAGGCTGTTCACAATGTTATAACACGTTTAGGCAGCCTAAAATCAATATGGCTTGAGGTAGGCTTGCTGCGATAGTCGGTGTTGCCGGTGTTAAGGAACAACACCA<br>GTATCACCACTTTCCCACCGCTCCCAAATTGGTGCTAATTTGAAAGTGAAAGTAAAATCCCCTCGGTCATTCATGGTGTGTGTCCACTGGTGAAGAGCG
LG23	ref-23182	51.548			ATTTGTGCATCGATTGTCGTGCAGTGTTCAT
LG23	ref-20490	51.666			TAAGTAAACAGCACTCACTGACTGCCTTGTAGTTGTCATTCAGAAATTGACTAATACAATCCAGGGGAGGGGGAAACAAATGTTGGACTGGTTAGCATGATTCAGCTACTG<br>GTATGTCCAAACCAAATCATCTTGCTGCAAGATGATGTTTATGTATTTTATGGATTGTTGTCATGATGCTTTTTTGGCATTTATGGAGACTGCAAGTCTCGAATTGGGTGTAT<br>CCGATGAGGTAGACATACAAAATGTGCAGTGGTAGGGAGTCCCCAGATCAGGTTTGAGAAGCACTGTTATAGGACACTTTGGTGGGGGAAATCTGTATGTTAAAAAAAAA<br>AAAAAAAAATGGTAAAAGCCTATTTTATTCTATTTTTAAGCAGTAAGGTAGAAGAGGATGCGACTATACTGTAGTGGTGTTGTTAGCTTTTTACCCC
LG23	ref-68183	53.174			TCGCATACTCAAGCAGTTCATACTTGTCTGAACTTAAAATGTTGTAAAAAGTGCAAGCATTTTCTTTGACACAATTTGTTGAGAAGATAAGAACTAAGTGGTAGACTACTG<br>GAAGAGTTTTTCTATGAAAAGCTGCATAGAAAGCAGTACTCAAGAACATAAGCATGGTTGTTTTGCATGCAAAATACTTGAGAGTTTGTCATAGTTGTATAAACACAGGAT<br>GGAGTAGGAGTTTCCCATGAGTGAGTAAAGGGGAAAATCAGACACAGATGATGTCGTGACTCATTTCTTTAAGTCCTGAGTCAGTGACTTGTGTCTGTCATTAACCTTACT<br>AACATGCGCTTTGGGGAGTTATGAGATCCTCCTTCT
LG23	ref-7127	54.044			CTTACATTATCCCAAAAGTTCCCAAAAATTTGTAAATCTAGAGAAATCCCCATTTTAAATTATGGCTCGTCCCTTGTCATTGTCATCTATCAATGACGTAATATCCGCGCTAT<br>CCTTGGTTTCCGCTTGTAGAAACCATGGCAACTCACACGGACAAATGCGGACATGGTGGTTATACAGCATCTAGCTTGTTGTGTCGTTCAGTCATCGTGGATCACGATTATT<br>CATTGGAAAATACAGAAAATCCAAAACGTAGATCAACTGAGGTCTCATATCTCTCACAGAGGAGCTTCGGAAGCATAAAAGGAGAACCGACCACAGTGAAGGGATAGAG<br>AGTACCAGGCCTTGACTAAAATTTAAACGTAGATC
LG23	ref-60208	54.078	Chr9	44324510	ACATTGATATGCAGGGGAGAGAATCTACTATAATAAAGCAAAGCTACATTTTTTCACGTTTCTGTAGCCATTCTTAATGGAATCTCATCACTCACCGAGAGTAAACAGTGT<br>CTGTGCAGGGATACACACTATAGCAAACATCATGGGACCATATGCCACAGCATTCAGATGGTCTCATAGCATCAGTATCAGCGTGTGTCTAACAAGAAAAAGCTTTATCAC<br>AAAATCACCTGCACGGAGAACAGAATTGATCAAAATAGAAAGGCTGCCTTGTTTATGGAATGCATAAGTCCATTTAAAGGAACCATTCACCCAAAATGCAAATCCAAATC<br>TGTATACTGTGTCTATTGAAAAATGGCTTTTTTGCGT
LG23	ref-8684_9	54.553	Chr15	43152812	AGGAATCAGACTACACTGGTCACGCTGTATGATTTTGATTCACTAAAAAGTGAACGAGTGATTCAATCAAAAGAGACACTTGCTGAGGTCGGAACCGCATACTACTTTTAA<br>TATTTCTGCTATAGAAAGATATGATAATGGTAATAGCAGTAGTACACTAGTATGCAGTTCCGAATT[C/G]AGCAATTCGGTCGGGAATCACACCAGTGTTGCCAGATGTACG<br>ATAATTATCGTATTTGTACGATAATTTTGACCTCTATACGATGTACGATCAATAATGAAAAAAAAATCCCATGATGTACGATAATTTCAGTATTTTGTGACACTTCAAATGA<br>TGGTCGTTATAAATCGGCTCATTGATCTAGATGTGTGG
LG23	ref-46283	54.656	Chr9	43707798	CACTAACCACAGCTAATTGACGGGAAATCTGAAGAACCTAGAAGTCTAGGTGAGGACAGACACCTCGTGCCAGTAGCCAATCAAAAGGAGATCAGCGAAAGCCCAGTTG<br>AGACAGGAGTTTTAAGAGGCAGTGCTGTGGGCAACCTGATTATGGAACCGAATTAGCGGGTGCAGACTCAGCATTTTACTCGAACTGCTTTCTAAATGTTAGAAACGAATG<br>AATATAAATTAGATTTTATTTCACATTCATGTCATATCCTATGCACCAAGGCAAAATGAAAAACATAAATCAGCAGGACCAATATTATGAGGTCATAAATAAAAAAATAA<br>ATAAATACTCTATTGTATATACACTACCATTCAGAGGTT
LG23	ref-63406	55.235	Chr9	38883390	GCGGTTGGCTAGTAAGGGACTTGAAAACCGGGTCATCAACTGTGGTGGCTACATCTTCAAGCACTGGGGGTGCAGTAGTCAGGCCCCGAATGGTCGTCTCCTCCACTGTAT<br>GGGTTGGAGACCCTTCGGATTCCTCACGGCCAGTGGAGCTGACAGATCCGGTTGAAAACTCAGTGGAAGCACCAGTATCCACTGAATAAGTCACTGTGGAATCACTGCTCT<br>GTTGGGATGTGCTAGTGGCATTGGTGTGTTCGCCAGAGGTAAAGGGTGGGATAGACGAAACAGAGTCACCGGTTTCTGTATAAGATGTCTCACTGCTCACCAATGTGACAT

					CTCTATTGATCACTGTAAGAGGGGGTGTTGAATTTG
LG23	ref-37852_18	55.561	Chr9	39748153	ATGGTGTTGCTATATGTTTACACTCTCTCTTTTAAATCTCTCTAAATCTCTCTTTGTCTAGTCTGTCCTCCTCCTCCTCTCTTGTTCTCAACCCCTCTGTTGTCGTCTCTCAACT<br>GGAACAGGTGGAGTATTAATTATGCATTAAAATATACAGAGGACCAACTATGATCCACTCGACTGCAGCCC[A/G]ATCGTTCGCCTCATCCTCATCTCTCTGTCCATCTTTCT<br>TTCTCCTCATCCCTGTTTCTCTCTCCATTCCACTCTTGTTTGTGAATTGCAGGAGTTTATTTCCACACTGAGCACGTCTCAGGGAGTGAAAGGGGATTTTGTTTGTGCGGCAC<br>ACTTCCTCTACCCCGACACTCTACTGCTGAGA
LG23	ref-69365_32	55.689			GTAATACAAACGAGTCAGTTGCCTTGGTAAT[C/G]
LG23	ref-66839	55.696	Chr9	21063555	AGACCTAGACCACTATTATTCTGTTGTGCTTACAGCTAAAACAGGAGGTTAAAAGGTTAAAGTTCAAACATCTAAGAGAAGGCCTGATGTCTCCAACATGCTAAATATTCA<br>GTCATTATCAATGGGTTATGTTTAAATTAATGTGCTTTTCCATAAAAACACAGCTGTGTCTGATTAGTGCATCCTGTTCGATGAGGATTAACACAGAACATGTTCTCAGGCG<br>TTTAACTTCAAACTTTTTCTGAGGAATTTTTAATATTGGTAGGTGTGACATTTATAGCTTTGAGACCCAGACTGGTCTCATAAAATGCTGCAAGCCTTTTTACGGCTTTATAA<br>ATATACACTTCATTTGCTTTTTTCATTACTGTT
LG23	ref-23548_23	55.924	Chr9	23024523	AGGTCTGTCCACAGTCCAGATCCACTTGTTCTTGGTGGCCTCGTACTTCTTCTTGTAGTCAAGCTACAAGGAGAAGAAACACAAATAATGATACGCTTGCTTGTGATTCAAC<br>TTCACAGATGCTCAATGCAGATCAATGTAGACTCACATTGCTTATGTTCTTGTAGGCCTCCTTGGCGGCACGGATGTCG[C/T]GAGCGTCAGGTGCCATGTTGATGTGAGCC<br>TTGTTTCTTTCATACACTTCCTTGTATTTCAGCTGTAAAAAAAACAACAGAAGTTTGAAATGAAATATACAGACATAAAAAGTCTTGAATTTGGTTCCTTTCTGCAAATGAA<br>AAGAAGGGTAAACTTACATTGCTGGTGATCTCCTTGA
LG23	ref-22345_5	56.73	Chr9	24014438	CGACATGAAGGTGAGTAATAAATGACATAATTTTCATTTTTGGGTAAACTAACCATTTAATGTTTATTCAATAAAAAAAACAATATGTTTACAAATGCGCATGCTCCCGTTG<br>AGCTTCGCTTTTTTGAGAATATGCTAATTATTCATTTTCAGGTGCCAATCAGTTGAGGCGTT[A/G]TGACGCATGGCACTCGGGCAGCGCTTGGGAGGCGGAGTCAAGTGCT<br>GAACGCAGAATAAAACCACTGCAGCCCGCGAGCAGATCTCAGTGTGAGGAGAATCATGGACGTAAACTAAACAGAAAGTTACCATGATAAAAGTTAGGCATTTCTTTCAA<br>ATTACAGCAACAACACCCGTTTCCCTTGTAAATCAACAA
LG23	ref-9359_27	57.221	Chr9	24523353	CCCTCTTGTTGGGTCTGGAAATAGCTTAGTCTCTGAAATGTTTTTCACAAAGGCAACCAGGGTGAATCTGTGTCTTGTTTGTGATAAGTGGAATATTAAGTCATTTCCATTTT<br>CAATGTTATTTTCAATTTTGTTTTTCAATGTTATTTTCAGTTATGTTATTTACTCAATTATATTACTACATACTACCTCCTAGCTATCTACCAACTACGAACAGCATGCTACCG<br>[A/G]GCTAAATAATAGCTCATTGACTAAAGCAATGTTCTTTATGGACCAGAAACTGTTCTAAATCAAATCATAACTGGACATTAAAGCCTACTACTATAACTTATAGAATGT<br>TTGTAACCAAGCAGATTTATTTCGGGGTAAACTATCCATTTAACTTGGTTAAACTGAAGCTACAAACAGAAAAAAACATTTTTTTGCACTTTATGA
LG23	ref-22008_3	57.255			TCTATAAATTTATATTCAGTTTTTTGTGTTTTTGCCTTTATTATGACAGGACAGTACATTGACAGGAAGCGGAGTTGGAAAGAGAGAGGGGGAAGGGAGTGCGGAGTTGGA<br>AAGAGAGAGGGGGAAGGGATCTGAAATGGTCCACGAGCCAGGACTCAAACTCAGTACGCCCGAAGCGCAACTGTGCTATACATGTCGGCGC[A/G]CTGCCGACGAGGCTA<br>TTGCCACCGACATATAAAGTTAGATTCAGTTTATGAACTTTTTAAGAATTTTGTTCGTTCATTTTTATATTTTATGAAGATTTTACATAAAACTGTGGGTTAATGACATCAAA<br>AAGTGTCAAAAATGTTTCGTCAGAATACACTACAAATTTTATTTTTCAAAATCGTAAAACAAACTGTGTGGTGGCTTTACATGTCGATCTGGCAGACCTTC
LG23	ref-18359	57.356	Chr9	25990786	ACCAAACAATATTATCATAGAAACGTCTATCCATTTATATTTCCTTACTTTTTAAACATGCTGAGATGTGAAATCTGCGCTCAGAAATGTAGATCCAAAAATGAACTTCACT<br>AACAGGGCATGCTTCTGTGCCAGCACGCATAAATTACATCACATACAAACCAGTTAATATTTCACAAGGGAATAAAAAAAATAAATAAATAAATGGACCGAATTCTATGC<br>CCTGGACCAGTGCAAGCCAGTTGCAGAGATTACAATACATAGTAAGGTCTGAAATGTGTTAGATGTGAAACAAAGGGTTCCCTGCAGTCAGCCTCTCGGAGGTGAAAGGT<br>CAACCCGGCACATCTGACATACTGCACATTAAGATGTGTGGGCCAGGGAGCCTTGAGGTACTATTTGCCACCTGGCAAAGATCAAGTCGTAATGCTTTG
LG23	ref-19862	57.532			TGTGAAGCATTTTTTTTTTATCACTATTGTTGAGATTCATATGCTGATTCTTGATGTAATGTCTGTATGTGTGTCTAAGTGATGCAGTAATTCAAAAATTTGTATGCACTACA<br>CGGTTTTAAAATATTTCAATACACCTGTTTGTTGCAACACTTTGTTTTAAATGACAAAACATATGAGCAGCTGCTTTGGATGCAATCCAAAAGTCTCACAATGACTAGATTA<br>TCACATAAAAACAGCTAATGTCTGCTCACTGTAAGGCACTGATCTCACTGCTTTACAACAAAAACCTATGCATTGTTGCACAGAAAAAATCTAACACAGAAATTCTAGGGT<br>CAAGAGCCTAACTTAAGCAAACGCTGATCCTCA
LG23	ref-16952_30	58.074			GGGGTACTCTGAGGTTCCACAGTTGGAAGAATGTGATTTTGGTGCACTTGAGCCCCCAAAACACTGCCCCCGGGAGAAATTGTCAAAGCCTTTCATCCAGGGCATGCAAGT<br>TTACCTCATCTCTGAAGGTGAGGGCTTACAATGCCATTGGTCAGACCGCCTCCGCCTTGTATGCAAAGGCCATCCTGCAAGTTTATCAGGCCAAAGTACCGAAAGATCTGC<br>ATGAGGG[C/T]AGTCCCGACCCAGAAATGATGCCGGAGCTGCGCTTAGCAACTGACTATGTCATACAGACAACAAAGGTCACAGTGTTCCTGCATAATTACTGAGTTGGGA<br>CTGAGTTGGGCGCTGAGGTAGGTGATGTCCACACTTGTGGTCCAGGAGCGAAACCTGTGCCTCAACCTTGCAGAGGACTGGGGATGGGCCTCACTCCTGGTT
LG23	ref-26125_8	58.311			GAAAAAAATCCTGGTCCAGTTCCCAAAATGCAGAGCCGCTTGATGCTCCACCAGCCAGGAGAACTTCTTTGCGATGCCTGATGGTGGCACTGGATGCTCTAGGTGGACGGC<br>CTTATTCTTCCTCCATCCGGCAGCGAGGCCCCATTCCTACAGCACCTGCTCCTCCCCATGCTGGCAGCTGGATGCAAGCCCTGACATCGACACCTT[C/T]TTCGACCCGACTG<br>CAGCCGCCCTTCCATGGTTTGGTGGACAGTATATACGTGGAGGCGAGTATATCCGTCCTCTCGCCTAGCCTTCCAGCTCCACCACCTTGAGCGTCCTTGGCGGCAGATTCTG

					CACAATTCAACTCGATGGCACCGCGACTTCCCTACAGCAGAAATGGAGATGACAGCGCATCACTTCTTCCTTCTGGGCACCAATGTAACAATGTTTGAA
LG23	ref-66368	58.698			CTTCAGCCAACGAGCAAACTGCCGCAAATGA
LG23	ref-8287_9	59.023	Chr9	29852843	CTAAACGAATCATCGGGGTTTGCTGGTCAAATAGTCACAAAGCGGTGCCAAATTTTAAGTCATTATCCGAATGATGCAGTCGGCAGTAATCAGAAAGGGGCGGATTTGAC AGATGGGGCGCAGTTTCTTTCTTCTCCCAATCAACCTTCACGCTGCTGGTGGTGTGAGTGACGCAGC[A/C]CGCAGTTCTCTCGCTGTCTGCTGCTGTAGTAAAGCACAGGG TCTGTTGTCAAGCTACTTGTAGTGTGGTCATTTGTTTCGTCTTCAAGGGGAACATCGACTAACGTTCCGTTCATTAAATGCATTCATCTTCAAACTTGGTCTTCATAAATGAA TCTTAAACGCAATAGCGTTTCATTTCCTCTTAAAAAAC
LG23	ref-19953_17	59.43	Chr9	33709172	TCTTTCTAGAGATTAGATTGTGCCTTTAAGAGTAAAAAATAAGGTGGTAAATTGTATAGTATTGTCGGAAGTAGAATTTGAAACTACTTACGTCAGTTGCCGTGTTGTGTAT AGTCCTTTATTAGTTTCACATGCTGTTTTATTGCTGAATGACATACATACTTGCTTATTGTGAGAGAGCACAA[C/T]GCTCGTCCTCTGTCTTTCTGCCTCTCTGTAAAAAGAC ACAAAGGTGACTTGACTGGCTCAGAAATGTAGACGTTAAGCAGCACTGAGACAAACAATGTTTCTCTGTTAGCGGGATCAGCACTGTAATCAGAGCTGTGCCATTAAAGT GCTTAGCAGTGTAGAAGTGCCATTGTAATCAAGTACT
LG23	ref-46675_31	59.601	Chr9	26335140	ACACTTTGGATTGTGGAATCCACCCCAGGAGTGTGTGTGTGAGTGTCTGTGTGTGCGTGTGCGTGCCCAGAACCCAACAGAGATCAGAAGAGCATCACTCTCAAGTTAAGA TCGATATCTTACCCCCCACCCTTCTGAACTTCTACACCTCATAACACACACACACAGATGGATATTGTGCATGCTGCTCGTGAGCACC[C/T]TATCTCAGGATTAGACTGAAT ACGAGGACAAAAATTGTTGTCGTGAAAATGAAATAATAAGGAACGGGAAGAAAAAACTGGATGGGTGTAATTGCAATTTATTTCCACTTCAGAGCCTGTGGGGTAAAAGA GAGAGAGAGAGAGAGAAATCCTTTAATATTCACAGCCTT
LG23	ref-2507_3	59.761			AT[A/G]CAGAGCTCGAACCTCCTGCTGTGTAGCCT
LG23	ref-71508_1	60.037	Chr9	31928512	ACATTTCAGTGAAAAATTGTTTGGCATAAGAAATCTTGGAGTATAGAGGCAACATCATATTGACTTCTTTTAAGGAGCTTTTCTTTTGTTACTTTTGGATGGTGAATAGTGA ATGTTCCTTTTCAGGTGAACTATTTCTTTAAACTAATTCCTTTTGCTTAGTTTTTATTGGACAGTCACAACCTGCTATCCATTTTTTTG[C/T]CAGTACCCGATCAAGGTGCAA AACTGAAACCCTGAGTATGTGCCACACTCTAAAACAGATTGTGTTTTTTTGTCCTCAGTGGGTTCATTCTCTCCTACGGGCCTGTGCTATAATCAGTGTCATTTCGGTCTGTA TGAATACACCCAAGACCTTTGAGCACTACCCGCCCCTGCAGTATGTCACCTTCACCCTGGACACACTGCTGATGTTCCTCTACACTGCAGAGATG
LG23	ref-6737	60.319			GGTTTTAAAACATAATATGTCCAATCACATTTTCAGCATTGTATGCTTCAGCATACGTGGAGCAGAAACTCGTCAGGTCCTTTGCACAACTATGAATGAATGAACAACAAA AATTCAGCTAATACAACTCAAGATACAACATTATTAACCTGATAACGGACTATTAGGCTCTCACAAGAGCACAGGGTTTGAATCGCTGTACATGTTGCGGATTAGACACTG CCTGTCAAGGCCTCGTTCAGTTATCAGATAAATCATCTTGGTTTAAGCCGGTCAAATAATTACAGTTTCAAAAGATCACATATATTTAACGACACGAAGCCAAACAACTCG TGGTTTTTCATACAACTACCAAGGTTTTCAGAATGT
LG23	ref-63101	60.878			ATGTGCAGCTCGATTCAATTGCCTTTTAAAG
LG23	ref-9473	61.053	Chr9	35993735	TCTTTTCTACAGCTTGTCCATGTAGAGGGACCCGGACTACTGGGATGAGCTGCGGTAATGGGGAGCTGGATGTGATGAGATGTCAGGTCAGACTCCTTCTCTAGAGATGTC ACAAGACTTGTCCTGCTAGGATTGCCGGTGACAGGGCCAGTGGGCTGCACCCAAGCAAGAGGCAGGTAATGGGACTGGCTTAATGGGACTGTCCTCAGGCGAAGTCATCG CAAAGGTCTCACTGAAACACAAACAAAGAAATACGTACACTCGATGGACGATGGACACAGGAATATCTGAGAAATCTAGTTCTGTAGCATAAACACCAAAACTATCAGTA CAGATGCAGAAGCAAATAAATGTGCGACCCAGACTAGCCTAAAGCCAAACCACATGTGATGACTTGTATTTATAAAATTGCAGGGGCTGCACAATCGATA
LG23	ref-5830	61.894			CATTCTGACACGACATGAATGCACTGAGTTG
LG23	ref-57380	62.225			CAATGTGGTGCGACCATTTTGCTATGGTTTC
LG23	ref-27068_32	62.322			ACTTAGTACCCGAAAGCACTGCACTGTGTTT[A/T]
LG23	ref-46385	62.572			TCCTCCGAAACGAGTTTGCTGCACAAGTCAC
LG23	ref-65214_6	62.801	Chr9	14453681	AAGTGAACCCTCTATGAGGTTGGAGTGTGAGGACTCTCACACACACACACCTCCTGCGAGACGTTGTCACTGTTAATATAATACAGAAAGAGAAGAGAGAATTTATGGTC GCATAAAAGTGGACACGATCTCCCACAATGCCTGACTGCTCAGGCTGGGTTACAGAAAGCCACA[C/T]GACCGCACTGGGGTCGCTGGAGAAGAAAGAGGTGTTGCGTTCC CCGCCCTGTCCATCTTTCCCCGTGTCTGTCTTTTTGTCTTTCGTTCCCTCCATCTCTCGCTGCTTTGCATTGGCTCGCTGCCACACTTTTTTTTTTTCTCTCAGACTGATGGGGC CGCTGGGAAATGTGGTCAGCGTTCACAGCTCTGCTCG
LG23	ref-27068_18	62.929			ACTTAGTACCCGAAAGC[A/G]CTGCACTGTGTTTT
LG23	ref-54099_18	62.997			ACGAGGTCCTTTTGGGGAGTTATTGACGGCGAAGGTGGGGTGTTGGGTAGGGTTAGTATTTGTTGTAGCATTGTGTAGGAAATCAGCACTGTGATTGACATGACCAGTGAA ATCTTTGGAATGTCCTGCGGGGCGAAGTTTGCGCTGAACTGGTTTGTTTTACGGGGGGAAAAAATCATGCATATGTG[C/T]CGTGTTGTGTTTGTCATGCCCCGTTTTGTAGT TCCTCATTCTCTAGCGGTTGTCAGTGCGCAGTGGTTTACCTGCACAACTCTCACTAGCTGCCCACTGTAACACACGCAATGCGAGAGTCGCTGTCTGTTTATCACAGCTGAC

					GTGCAACAAAATGCTTCATGAAAAATAAAGCGCAGCTG
LG23	ref-54099_32	62.997			AAACACAACACGACACACATGCATGATTTTT[C/T]
LG23	ref-65214_8	63.273			TCTTCTC[C/T]AGCGACCCCAGTGCGGTCGTGTGG
LG23	ref-32696_15	63.881			AATAAGCCTTAGGAACAGCAGACATATGGGCCCCTATCTGTTGTACAACACTTGGATAAGAGACACAACCTGCTCTTTTCTCCACAGACATTGCCTTGCAGAGAGCTCGGA<br>CACCCGTTGCTGGAATTTCCTGCCACTCATCATCTGTGCTAAGGTGGGTATGTGGACGTCTGGACAATGCAT[G/T]AGAGTCGATGTTCATTCGGCCTGGCCTGTATTTCAGA<br>CTGAAATTGTAGGTGGTAAGGGCTGACAACCAACGGTGACCAGTCGCATCAAGTTTCGCTGACTTCATTATATAGGTTAGAGGATTGTTATCAGTTCTAACCTCGAACTGG<br>ACACCATAGAGATAGTCGTGCAGTCTATCCACAACAGC
LG23	ref-32696_4	63.895			GAA[A/T]GAACATCGACTCTGATGCATTGTCCAGA
LG23	ref-67455_32	64.146			CTTCACATGCTTGTATAACGTAATTACAGCGGGTCAACTCGTAATTTTCTGTGAGCTACGACTTGTACCATGTGGCCGCTGTACCACCTGACCACTGCAGATTTAAGGTGTG<br>TTCGACTTGAAGTGGCGCTGTGCCAACCAATCGGTGTATGACATCAAAGCACCGCGAGAGTGATTTGAAAGCATTCAGAGCTTTATTTACGGTAATTACGACATGGCTGCA<br>TGACTTAT[A/C]GTTTAAAACATGTAAGTACTAGTAGCCTGATGTTAGCCACTTTACATGGCCGCTTGCTTTTACGTTAACGATAACCTAGATAAATGTGCACTAATATTAGG<br>CGCATATCCAAGTGTTCTTGCGGAGTGTGGAAGCTAAATTAGTAGCACGCTTACTGCACGACATGTTTAACTTAAATTCCATGTCCATGCCATCAGAAT
LG23	ref-33266	64.434			CATGATTCTAGTCATAAAAGTGTATTTCTTTAACTACACTTAATTTGCCTTTTAATTCATTAATATTATATCATCTGCAAGTACAGTTTTTTTAAAAATATACTTTTAAGATTC<br>CAAATGCACTACAAGTTCACATTAAATACAACTAATTGTGCTTTTTTTTTTTTTTCAAAAGGGTAGGCAGATCACTAGGTTTTGGAACAGAGCTGCCAAGCACTGTGCTTTG<br>AACCTGGGATTTGATTAACACAGTGGAAGAGGTTATAACTTCACACAATCCACAGTCCTACATTCTACACATGGCATCTGTGTTTCTACACTCATGCTGAACACAATGAAC<br>AAATAGAAGAACAAAGGAGCCCTCTGCTAATTAGGCCCATGAATAATAGCAGAAAGGGAAATGCGTGACACATCCCAAAATCAGTTACAGGAAA
LG23	ref-24605	64.791			CAGGTCTTAGGATTTTGAAAAACTGAAAAGCTTGTAGGTAAAATATTACAGATGAACTTTCAATATATGGACTAAAAGGTGTTTCAAAATTGATAACTGCATAATCTATAA<br>AACACATATACCCACGTATAGGCATATTCAATGGTATGCCTCAGATATAGTCAAAACAATTTTGTAATAATTAATTTTTTTGCATGAACATCTGTAGAGCGATCTGTACGCA<br>GCACAATATGAGCAATATTGTGAGCATATACAGACCACAATCATCATGTTTACAGATGAAAGAGGGAATGATGCTCACTCAGAGGATGCTGCTGTTGCCATGGTTACTCTG<br>GGTTGTTCTTTGCGAAGGATAGTGCAGATATAAAGGGGGATGTAAATTCCAAGTATTCATCATACATCGCATGCCATCTTCTGACACCTCCATTGTC
LG23	ref-18100	65.071			CTGCAGAATCCGATGAGGCTGCCTCGCACGT
LG23	ref-14028_18	65.531			TCTCTTATATCGAAATCTGCTGACATTTCCGTTTAAAATTTCTCGTTTTAGACTTCTAATTCATGACCGGTGTTTTGCTGACTTCGTCAAAATAGTCCCTTCATCATTGCATCC<br>AGGTCAAAGGTCGCTCTTTGCACATTCTACGTACGGTCGTTGCAGCCTTTAGCCTCCTTGTTAGAGCGTCTGACTTCCACGCCGGAGAGCCAGGTTCGATGCC[C/T]GTGCA<br>GAGCGTGGTGAGTAGGACCTGGGGAGAGGGGTTGTGTTTATACAATTTTAAATATGGATATTTTTGTTACAAAAACGCATCGTTTCGCTTCAGAAGGCCTTTATTAACCCA<br>CTGGAGTCGTATGGATTACTTTTTTTTTTTTTTGGATGGATGGATGGATGGATGGATGCATTATTTTTTACTTCAAAACGCAGTCCCCCATTCACAAC
LG23	ref-19813	65.545			ATTGATCTATCTTACTGCTGTGTGCAGCAAGTGTCTCATAGCAGCCACCAAGGGAACGCACGGAGAAACGCTATAACATCATTTTCAACACAGTCTTTAATATGACAAACA<br>GTGCTGCTTTACCCCACATACTCTTGACTGGAAGAAGCTGAAGCGGCGACTGTGGCATAATAAAAGTTGCGCTGGTCTCGAGACGCGAGTCGCACTCGTCTCTCATTAGCA<br>ATTGCTCCAGCGGCCTTGTTTCTGCTCGCACAGCACTCGGCCCTGCTCTGGTTCATACTACATTAATGTTAATAATCTCATCATGAACATGATTTCTGCCCAAGTCACGTTGG<br>ATTCTATCCCCTGGCTGTGAGGTGAAGACGTCAT
LG23	ref-49298	65.977			AAAGCTGCGAATTTTTAGTGCGAGTGCATACAGGCATGTCTGCTTCATAATACAACATAAAAGCTACCTTTTAGTGAGCAAATGTGTCGCCCTCGCCGTGTGGTGTCTTGG<br>ATTTCCAAGGGGAATCGGATATGCGTATTTTGACGTAGGTCGCATGTCCAGAGATCGGATACGTATTTGGAAGTGGCTCGGATCGGATGCGAAAAAAATCGGATCTTGTGC<br>GGATTTTTGTGTTTACACCTGTGCAACATATTCCGATCTGTGTCAGATGTGAGCAAAAAATCTGATATTTTGTGCCAGTGTAAATGCAGCCAACGAGTGCCAGCCCCGCCTA<br>AGTACGGTGTTGATTCACTCAACTATGTTTTGGCT
LG23	ref-17467	66.341	Chr9	17372029	CAAGCAAACTGTGAATGATTACCTGCGTACGGGCTGGGCAATTTTACTCCGTGTGGCCCCACTTGTCCCAACAAAGGAAGCAGGTCGAGGTAGAGCGCTGCGACTGGAGG<br>AGGGGGTGCTGGGTTTGCTGGGGAGGGGGATACTGCTGCTGGAGAGACAGTTGAGACTAACAGAGGAAGGCATTGTGGTAGAACTCTGTATCGTAGGGCTCACATACGCT<br>GTGGCTTTCAGAGGTGGCCGTGTTGTCACGGAGAAATTCCCCACGCTCTGGACCCGCTGCTGTAACTGGCCAGGAGGGGGAACTGAAATTCAACAGAGAGCAAAACTTGT<br>GTTATATAATCCACTACAAGATAATTATCATTATATTTA
LG23	ref-69351	66.449			CGCCCGATACCGATACCGGTGCAACCCTAAT
LG23	ref-59696	66.611			GCGTACTAAACGACCAGCCTGCAGCGAAGGT
LG23	ref-20011	66.789			TCATCAGATTCGATGACGGTGCCACAAGACC

LG23	ref-17922	66.841			TAAAGAGAACCGAAGGGATTGCATGCTGTTA
LG23	ref-3419	67.166			TGGTCGTATTTAATGATTAGTTAAAGACAAAAATTCCTTTCCCTTTTAATGGTCCTCAATGAAGATGATGTTGATTTTGTTGAGCTAGTGCTCCGTACTAGTGGTTCCTCTTT<br>CTTGCCATGGACATAGAGCCTGAGGCCACACCCACTTCAGGCCCAGAGCCAAAGCCCATGCCCACCACAGACCTCCTGCCTGTGGAGATTTCCGTCCCGGAGCTAGTGCCT<br>GAAGCCATGTTCCGCCCCCTCTGCTGGATCCTCCCATTTGCCTGGCTCGTCTTTGTCCTCCTGAGTTGCCGGCTCTCCTCGGCCCTTCGACCTGTCAGCTCCACCATGATCCA<br>TAATCCCTCTGGCATCACCGATGTCCATCACACTCCTGGCTCCACCATGGACTTCCGGGCCTTTGGCTGCACCTGGGCCCTCAATTACTTCGGT
LG23	ref-7862	67.331			TTACAGGAATCGAACGGCATGCATCCCTCTC
LG23	ref-40161	67.421			TAAACCCAAACGAACGAAGTGCTCTTGCTCT
LG23	ref-35612_19	67.503			ATATTTTTTTCACAGAGTAACTGTTTACAAACAGTGTCATTTTCTATACCGCAAACTATGCGGTCCATAATGAGTTCGTCTTGCAGCGTCTTGTATATGCTTGCACTTTCTTT<br>GATTTATCCAACAGGCCCGCATAGCATTAGATGTGTCATTCCTTTTCAAACTTTCTCCAGTTATCAGCAATGTT[C/T]TCGTCAAAAACCAGCGGATCGATACGGCGAAGTC<br>CCTGGACCATTTTCTCCCACTTCTTACACCATGTATGTTTTTGTTATAAAAGGAATCACATCGAATCAGCCTCGATCCAAAACCACCGTACTCTCTTTTATTTCACGCTCCTC<br>TATCTTAAAGTGAAACCACACATAATGCTACAAGT
LG23	ref-39198_9	67.521			CTTCAAGA[A/G]CCGATCTTGGTGCTCTTCAAGAG
LG23	ref-25886	67.709			AAATGTCCCCCGACGCCACTGCCTTCAGCGT
LG23	ref-54025_15	68.174			TGTAACCAAATTACACGAAAAAAATTCTGAATTGGGGTTTAAAAGACAATTAATATTCAGTAATATTTTCAATCTGACTGTACTGTGGCCCTGTTTCCACCTGCGATTTGGT<br>TGATTGGATCACAAGTTGACGACACTAAATTCAGGTGTAAACGCAGGTCTAAAACGTTTTGAGCTTGTCCACTTTCAATTACTTCCAGAGGTAGCATTCGAC[C/T]GGATTG<br>CTTTTGTAGTGTAAACGCTCATGTGATCGAATGTGTTTGAACGGCCACAAAAGACGCCTACTGAATGCGCTCTAGCCATACGGGATTTAAACTTTGTCGGCTGAAGACCCA<br>AGTTTGGTTTGAAGATTAAAAAAACGTACCAAGCACAATGATCTCTCATCATTCCTGATTTCTAACACACACTCACAGCGGTCGCCTGTTGTGTGAAATA
LG23	ref-54025_26	68.185			AGGTAGCATTCGACTGGATTGCTTT[C/T]GTAGTG
LG23	ref-2629_30	68.245	Chr9	44902283	TTATGCTAATCTAACCACCAACAGCTCTTCAGTGTGATTGTGAAATAAAGAGGCTGAATTCATTCCTCTTAATTGCTCTTCATTTGGCTATAAGACAAAATCTTTAATTGCTT<br>TCCTCGCAGCCATCCAGCACGGTTGGCAACCAAAACAACAGCGAGCAAATTAGCCCTCAATCTGCAGCATCCCAATCGACAACGC[C/T]GAGAGGCCTCGTAATGAATTCC<br>ACCGGCTTTGTTCTGCCCACTCAACCCCTTTACGTCCAAGAGGCTTGAAATAAAATAGTAAGATTCAAATCATTATAATGCATTCTCTAAAGAAACAAGACCTCTGATGAC<br>ACTCTATTAAATATTGTACAGGGGTTGCCATGACAAAG
LG23	ref-10182	68.845			ATTTCCTGTGCGATTTTTGTGCAGTTTCAGG
LG23	ref-47690	69.431			ACGTCATCAGCGAGTTGCATGCTATGCAGAC
LG23	ref-47415	69.874	Chr17	49099704	TACTCTTAAAGGAACATATCATAGGTCATATCCAGGGCTTGACATTAACACCCGCCAACCCGCCAAATGCGGGTAGATTTCAGCTTTGGCGGGTAAGACAGCCCCTCCCTA<br>GCCACTTTGGCGGGTTAAATATAATTTCAGCCTACAGCCTAATCAAAGGTAGCCGAGCTTGTTGTAGCACAAAATTACAATTCAATCACAATTCGTTTTCGATGAACGTGC<br>AGGGATAGCCGGAATAGCGCTGAATGACACAACCGAATCCGACCCGATTGGATACAACACGATCAGTTCTCCTTGCGCCTGAATAGTCAAATAAACGCACACACAGATGT<br>CAAAACGTTCCTCGTGTGGAGTATCTCGCGTGAATACAGTCGGTTATGGCTTAAGTGGACGTAAACAGGTGGGTAAAAACTGGATATGTGTCAGTATAT
LG23	ref-52189_3	70.44			TC[C/T]GCAGTTACGACTGTAATGCACTCCGCAAC
LG23	ref-29357	70.713			GATTGCTTCCCGAGTCCAATGCCTATTCTTT
LG23	ref-28771_15	71.417	Chr9	47028046	TAGAATTATAAGGGTGACATTTGACAAAAAAGTTGAGTAGTTTGCATCACTAATTGCATACTACCACACACTCATTCACCTGTTTACTTTTTGTTCAAAAACAGATTAATTC<br>AGGAAAGGTAGCTGAGAGACACGCAATGGTTCTGCAGTGACTTTGTTTCTCACTATTTCCGTTGGCGGCAT[A/T]AAAATCGATTGACAATGTTAATTAATGCTAAAAAAAA<br>TGGATCAAATTTAAATATTCATGTTATACAATAAAAGAAACAACAGTCTTACCTGCTTGCGTTTGAAGAAGCCCTTCCTGTCACAGTTAGGCAGGTAGAGGGAAAGTGAAG<br>TGTCTTTCATACTTTGAATGATTCCATCCAGCTTCCTT
LG23	ref-54099_30	72.23			AAACACAACACGACACACATGCATGATTT[C/T]TC
LG23	ref-54099_31	72.364			AAACACAACACGACACACATGCATGATTTT[C/T]C
LG23	ref-26335_30	73.786			CACCGAAGCTCAGTCACTATTCGATTGTTTCAGAACTGCAGCTTCCCGGTTTGGGCTCACTATTGCTCTAAAGAAGACAGTCATGCTTCAAGTGGTGAACAAGTTAAACTCT<br>CATCCGCTGCTAGTTACTGCAGGTGACACTTTGCTGACAGCTGTTGACAAGTTCTGCTGCCTCGGTAGGATTCTCGCAGCTGATGCGACAGCGGACTCCGACATCAGTGCC<br>CGCATC[A/G]CAAAAGCTAGTGTTACCTTTGGTCAACTGTTCAAGTGAATATGGGACGAGCACACCAACTGGTTGGACACAAAGGTGGCAGTATATAAAGCAGTAATCCTC<br>ACAGTTCTGTTGTATGGATCTGAGTCTTGGATCACTTATCGCCAACAGA

LG23	ref-25596	74.055			TGCCATGGTTCGATTCTTATGCCTCTAATGT
LG23	ref-13093_26	74.455			GCTCAGGGTTATTTGATGAACTGAAAAAAAACTTTTTAAAACAGAAATGTTTTGTAACATTATAAAATGTCTTTACTGTCACTTTTGATCAGTTTAATACAGAGTCCCTAAA GGGACGTGAGGATGGAAAAAATATAAGATGGGAGGAAAAATTACATTTCAAAGCTTTTACATTCTTTCCGCGAGAACGCAAAGTTTCTTGGAGGAACACGAAACATTTGC GAG[C/T]GAACGTAAAAGCTTTGAAATTTAATTTTTTCTCCCATCTCATATTTTTCAATTTAGGGGCTGGGTAATTTAATGCATCCATGCTTACCGATGTCAAACTTCTGAAC GATAGTGTATATACTGATCGTGTCATATGGCTTGTTGCTGGCATTTTACAGAACAAATGGAGGCACATGTGTAGTCATACGGCTGTGGTAAAATCACTGC
LG23	ref-57329	74.663			GTGTTAGTTTCGAGTCGAGTGCAGCTTGAGT
LG23	ref-12705	74.777			AAGCCCAGACCGACATGACTGCAGATGCAGC
LG23	ref-41884	75.081			GAATAGAGAACGACTTTAATGCAAGCCTGTG
LG23	ref-62100	75.639			AGGAGACGACTAGATGGATGACGCTGCCCCTGTCTTTAATGGGAAGAATAAATGTGATTAAGATGAACATATTACCAAGATTTTTTATATATCTTTCAGTTGTTACCACTTC CCCCTCCTCCTGCTTTTTTCCAAAAAGTAAGTAAGCTTCTTTCTAATTTTATTTGGAGTAATAGGAAGCCTAGACTTCGACTTTCCCTTCTGTACCTGCCTTATGACAGGGGG AGGGTTACAATTACCAAATTTGTTATGGTATTATTGGGCTGCTCAAATGAGGGCTGCAATGTTCTGGTTCTCAAAAGATACAAATATACCTTGGTTACAGGTTGAAAAGTT GGCTGCAAAAGGCTTGACAGCTTTTTATATTCA
LG23	ref-23764_1	75.99			CACATTTTCCCATATCTGGTGTGTTTGTGATTGACACTGATGATTGTTTGTTGTTCTGCAGTGTGGGAGTCGTTGAAGATGCCTGTGGGATACGGTGTGGTCGCTGCTGTGAT CGTTGCTGTCTTCTCAGTGCTGCTGGGTCTTGCTGTCGCCCACTGCTGTTTCTCCA[A/G]GAACCCCATGCACACGCCTCGCCCCCGAAACAAGCTTTTGGACATGGAGATGG ATTAAAACAGTCCACCATCCTAGCGCTGTGACCCGACCCGAAACCACTTCAACCAACTTTGGGACTGGATGTGGATATGCACATTGCTTAACACGAGATGGAGAACCTGTT TATATGCACTCCGAGGATTGGGCCACTCTGACTGAC
LG23	ref-65002	76.658			GTTTAAATTTATATTAAATATAATTAGCTGAACTTTAGAGTGCTTGGACTTAAATATATCTTTATGTAATTTCATAATTACTTCTGTTGAAATATGGTTAAAGTGTACTGCTG AATGTACTGACAAGCGTTTATGGTAAACTAAAATATACTTTAATGTCCTTTCTACTGAAACTTGTCATATTTAAATATATATGTAAATGCACATCTGCAATGAAGTTGCAAT TTAGTACATTTAAAATATATGAACTTTAAATGTAACATAAAATAACACTAAAGTGAAAATTATAATCAAGTACTTTTACATGTGCTTTAGTATGATAGTCAACACATCAAA ATAAGTGTACTTCTTTAAAGCACAACAAAAGATTATTAAAACATAATGATTTAAAATTTACTTTAAAATAAAACTTTTAATTTGACATTATTACA
LG23	ref-8009	76.893	Chr4	64827513	AAACGGAACTTTAACGCCTGGTTTTGGTTGTGCATATTATTGGCCTGCTCTGCTGGGTCAAATTGTATCCCCCGGCAACAGATATGGCTGAAACAAACAAGGTCAACTCTA AGCCTAGGCTTAACCCTGACTTTGCCCGCACAAGGATTTCCTCGGCGAAATGTGAGGCTCAGCTCCACGCGCACGGAATTTAGACACTGATAAAATCTGCGACCAGCCCGC AGACAGGCTCGGCACCCGCCCGGCTAGCTCAGTCGGTAGAGCATGAGACTCTTAATCTCAGGGTCGTGGGTTCGAGCCCCACGTTGGGCGTATCTATTTTCTCTGTCTCTCT TCCCAGCGGCTTCTCGGTGTTCCAGCGTGCAAAGCAGCGCGATACTGGCTGGCTCACCAGCGGTCTCTGTGGCGCAATCGGTTAGCGCGTTCGGCTG
LG23	ref-46592	77.566			ACAATCCGCGCGATCAAGCTGCCAAAATAAA
LG23	ref-20657	77.665			ATATAAACATCGATGTTAATGCAACCGCAAT
LG23	ref-54738	78.241			GATCTTCACGTGCTTCTGCTGCTGCTGCTGCTCGCCCTCACACGCGACGCGTCAACTCATACATGCGCTGCTCCTCGTGCCCGGCCGGCGCGCGCTGATGACGCCACGGAG GCGCGTCGCAGGTCTCGTGAACGCCACTGGATGGCAGCACGAGCACAGTTCTGTGTTGCAGTCAAGAGGCTCGATCAGAACGAGCCTTTTCGGAACTTCGAATCAGTTGCT GCTCAAAATGATTCAGTTTTTGGAAGCGCTCGAATCTTCTGCTAGCCAGAACTGTGTGAACTTCACCAAAACATGCATAAAACACCTTTACAGACCAATCGCAAATGTTTT ATTGAAAGTGCAATCTATTAATTTGAAAATAACTAAGCATTAAATTTGAAGTGTCTTTATTATAAGGGTTCTTTTATTAGTTTTCAGGGCTTGTTTT
LG23	ref-26593	78.705			CTAGCCCTGGATTTCTAGATCTGCTAGCATCTGGGTCGAGTAGGTCCTTAAAGAGGTCATGAACTGTGTTGTATTATTGTTTTATAATGTTTCTGGGGTGCACTTAT
LG23	ref-59488	79.925	Chr4	67089835	ACAGAGGAGCAATTCTCACTGGAATTTCATAACGAACAGGCTTCTGCTTCAGGAGTCTAACAGTGCACTTAGCAGCTCTTGCCATGGAGGAGCCGTGCACCATCACCGTGA GACTTGCGTCTCTTGAGAAATCAGAATCAGTGGTCCTCCAGAAATGCAACGTTTCATAGCTCCGATATGCACGTTTCTCTGCAAACCGTAACGCTGCGCCAAGCGAAGGCC GTGAGGTCATCGGGGGCCACCGAAGGGATGGCCAAAGGCCTTGGATGGCACAACTCTCATCCCAGAAAGCTAAAGCAAAAGGGGCAAGCCGGCCCGTAGTCGGCAGGGT TCGAACCTGCGCGGGGAGACCCCAATGGATTTCAAGTC
LG23	ref-2887	80.545			TTTATTAATTTGAACTATTGGAGATTGCCAAAGGTGTCCCCCAAGGATCTGTGTTGGGGCCCCCTTTTATTTACTATTTACATAAATAGTCTTGATTATGACATTCAGGATGC TAATTTTCACTTTTTTGCAGACGACACTGTCATGTATTGTAGTGCTTCCTCAAAACAGCAAGCCCTGCATAAGCTTCGATTAGCTTTTGATGTCATTCAGTCGTGACTTTATA AGTTAAAACTTGTTTTGAATGCTGATAAAACCAAGCATATGTTATTTTCAAGTTCACAGAAAATGGTAAATAATCTAATTTCTCTCCAGACCTTATGTGATTGAGTCAGTAA AACAGTATAAATACCTTGGCATCATTGTTGA

LG23	ref-72508_19	82.166			GGCATGTATTTCACAGCACTGACTGTGATATTTGACCTCCATCAAGTTCTCCTCTATTGGCTCCAGACATTCAATATGGCAAGCAATGCCATTTTCTTGCACTTCTTTCGCTCCTCTGGCTGCCAGCTGACAAAAGCATATTGACCAGCCGGCCTTCTTGAGCTCTGACTTATGGTTCTGCATGACG[A/G]TCGTAGTCGTTCTTTTCTCCGACGAGCTAATGTGTTGTTTCCATGTTCATCACAGGTTTTAACACTACCTATTTTTGTTCCATCAGCCGGAGCATCAGTAAAGCTGTCTGGGTACTGTTTAACAATGTTCTGCTCTACAATCTGACACATATCTTAAAATATGTCACTGCCATTGTTTTGTC
LG23	ref-57670	82.695			ACAATTTTGGCGATTTGCATGCATGCATATT
LG23	ref-6326	82.983			GGGACCAGGCCGAAGTGTGTGCTATTTGGGA
LG23	ref-41267	84.033			AGGAGCTCGACGACGGCTTTGCAACGCAGAG
LG23	ref-20268	84.656			AGACTTTACCCGACATGGCTGCTGCCATATT
LG23	ref-42207_4	86.408			CGC[A/G]ATTCTACGAAGAGCATGCCAAAATGGAC
LG23	ref-42207_3	86.573			CG[C/T]AATTCTACGAAGAGCATGCCAAAATGGAC
LG23	ref-29298	89.039			ACGCTTATTTCGACACGCGTGCATTTCGATG
LG23	ref-55867	89.248			CTTTGAGGGCTGGTAGTGCTCACACCATCTACAACGTCACCAAGGTTTGCCCCAATCGGCCTGACGGGGGCGCTACAGAGGTCAAAAGTACGAAATGGCTCTTAACTCCTAAAGCATTAGGCGTAGGCTCAAGTGTCTTATATCATTGGTGGCCTTGGCTCAAGACGGACAAGATGCATGCCTTGGATTCGTTCGTCATTCTTGGCGAAATTTTCGAGTATTTTGGATTTTTTGAAAACCCTACTTTTGCGAAATAGTCCTAGTTTTTTTACCCAATCGGAACCAAACCAGTGCAGCGAGATTCTCTTGCACGAAGGGGCTAATGTTCAAAGTGGGTGGAGCCACTTTTACTAAAATAGCTATAACTCG
LG23	ref-38677_3	89.895	Chr9	1020674	AAGTTTACATTTTTATGTATATTTTTCTTGTGTAAAAATTATGTAATAAGTGTATAAACATTGTATGTAATATCTGTATATAGAGTGAGAAATTTTGATCTTGTTTTTTGATGTAAGAATTAAAATTGTGTGAATTTGCTCAGTTGTCACTGCCGTTCGATTGGTTCAGCTGTTATCAACACACTCAAGCCCTTTCAGCAGC[A/G]GGTTAAACGACACTGGTGCTTCCTCAAAAGATACTAATATGGGTCATCGTAGTGGTGATTTCTCAGAAATGTATGTTAGTCTGATTCGAACTTTAGCACAGATTTATAGTTGTATAATCCCACAGGACCTTCTTTTACATAAGACCGCATTAAAATGCCGTTATCATTCAGGTGCAATCAAAAGTGACTCAGAACTACTGGAGAAACAGGAAGCACAACAAAAATGGTT
LG23	ref-25363	90.401			TTTGTTTGGGCGAGGAGCGTGCACGTTCAGT
LG23	ref-66904	91.124			AATTTAAGATGAGAGTATCCTGAAAGTAGCATTCACATTTCAGGGAGATGAAGAGTGCAACAGCCTGGAAGAACTAAAAAGTCGTACTGAGCAGGCTGATGAAAGGCACAATTTTGCCAAGCAATTAGAGTCCATAGTATCAAGGGCCCTACATTTACAAAATCTTAAAAGAATGGATAGCACAGTGTTCGAGTCAGAATGTAGAGATAAAGCCCCTAACACCTGCAGTGCTTAAAAATATAGCGTGAAACAGCAGAAAAAAATCTCGCCGTCATCATAATGAAATACTCAGTCTTCAGAAGATGGTGGAGAAGAGAACCGATTCTGGCAAAGTTTTCCAGACGGTGCTCCTGCATCCTCGAGGAGTGA
LG23	ref-18028	91.171			AGCCGCTATGCGACTTTGATGCGCACTTCAG
LG23	ref-22375	92.949			AGGGTCATCTGCGTCTCCCATCCCAGGCCTGTAAGTTCTCTGCTACACTCACAGCCAAGGCTTACAGTGCTGCTGGACAGGCCACCTCTCTGCCCTGCATGCCATGGCCTTCTTGCATACCTTCTTGTATACCAGGCCAAAGCACTAAAAGAGCTGCATGAGGGTAGTTCTGACCCTGGGCTGACAGAGGAGCTGCATACAGCGACGGAGCGAAGCCAATGCTCGATCCCTGAGAAAGACAATGTCCATGCTAATGGTTCAGGAACATCATCTCTGGCTGAACCTGGCCGAGATTAAGGAGGTCAACAAAGCTTGCTTTCTCTAGTCCCCCATCTCCCAGGCCGGCCTCTTCGCCGGAGCTGTCAAGAACTTTGCCCAACAGTTCTTGGCAGTACAGAAGCAGATGGAGACAATCAAACACATCCTGCCCC
LG23	ref-30007	93.453			TGATGAAAGCCGACTGTGTTGCAGAGATGGA
LG23	ref-19426	93.845			ATGGAAATGTAAAAAGATCCCCTTGAGACAAAACAGGTAAGGCCATGAGTTCTTTAGTTCAGACAGTGGCAGAGGTGGAGTGGCATGTATTTCACAGCACTGACTGTGATATTTGACCTCCATCAAGTTCTCCTCTATTGGCTCCAGACATTCAATATGGCAAGCAATGCCATTTTCTTGCACTTCTTTCGCTCCTCTGGCTGCCAGCTGACAAAAGCATATTGACCAGCCGGCCTTCTTGAGCTCTGACTTATGGTTCTGCATGACGATCGTAGTCGTTCTTTTCTCCGACGAGCTAATGTGTTGTTTCCATGTTCATCACAGGTTTTAACACTACCTATTTTTGTTCCATCAGCCGGAGCATCAGTA
LG23	ref-1927	94.004			AGAGGCTTTCACCTACAGGGAAAAACTATTAAATGAAAGGAATAGTAATGATGAACACTTGTCCTAAACTCTGTTTTTAACTATCTAAAGTAATAATGAATCAGATATCTTGATCTGATCTTGATCTTGGTAAAAGGATGAATGTCTCAGTTTGTTGCTCACTGGCTGTAATTGTACTAGCATGCCTTTCGGTCCGATTTTATATTAAGTGTCAAAATAGTTATGTACTAACACATAACAGGATCAGGTATGTTTAACACCCCACAGGGTCCAATCCTGCTCCTGGAGGGCCACTATCCAGCAGAGTTTAGCTTCAATCCTAATTAAACGCACCTGATCCAGCTAATCAAGGTCTTCAGGGTTACTAGA

LG23	ref-71269	94.263			GCCTACTCCATCCACAATCAAAGAGCTTCAAAGATTCCTCGGGTTTGCAAACTTCTATAGAAGATTCATCCAGAACTACAGCTCCATAGCATACCCTCTGACAAGCCTCCTC<br>CGAGGCAAGCCCAAGTCTCTGTCCTGGTCACCAACTGCCAACGAAGCTTTCAACACCCTCAAGGAAGCCTTCACCAGCGCACCGCTCCTCGTACACCCCGATCCAGGTGCT<br>TTCTCAGCAGCAGGGGAATCCAGCCCAACTCCATCCATGCGCCTTCTTCTCCCGCAAACTCAGCCTGGCGGAGAGTAACTATGACATCGGCAACCGGGAACTCCTAGCCAT<br>CAAACTGGCTCTGGAAGTGTGGAGGCATTGGTTAGAGGGTGCCAAGCAGTACTCACTGACCACAAGAACTTGAAGTACTTAAGAGACTAAATTAAAC
LG23	ref-43155	94.327			CAAGCAAGCTGTTCAGGTGTGTGGCCTTCACAGGCCTCCCTTTAAGCTTCCTCTTCCCGTTTCTGGGTGTTCTCTAAGCAGACTAGCGCATGCTGGCCTTTAGCAAGGCTGC<br>TGTAATATATTTCTACACATGTAGAATTTGTGTCATGTGGCAGGTCCCCTCTTGGGCCTCCATGATTTTGTGCCTACAGTATGCTTTATCTGTTATGTCGAGCTCTGTACTCC<br>CCTCGTTTGAAGGTTGTTCTGCATAGTGCTTAGCTCCCTAAGCTGGTCAGTGGTTGAAGGATCACTCTGAGGCCTCCCTATTGAGACAGACAGGTTGTTGGCCAAGACTAG<br>GTTTCTGTAAAGTAGACCAGGTCAGGCCTCCCTGGTGGCAATCAGGGTCGAGTACGTTCCCCTGACAAGTGACTGGCTAGGGTTCCCTCGGGCCC
LG23	ref-25719	94.6			CACAAGCGTTCGAGAGGTATGCATGTTAGTT
LG23	ref-10029	94.817			CTGGTCTTTTAAGCTGCACAGATTCCTGACAGACACTTGATGTGACAAAAACATGGGACAATTTGACTTCACCTATTCACACTAATGTGTTAATTTGGAAATTATCCAAGAC<br>AGTAAGTCATGAGTTAACCCCTCACATGAACATTTATGAACAGGATGCATTAGATAAGTCCGCAACAATTTAGCTTTTTTCCTGTTAAAGTTTTGGCACGATCTTCATGCTG<br>AAGTGGGAGTGTGAGGGGATTGTGTAGCATGGCTCAAGCACTTTTAACATATTTTTAAACCCATTGTTTGACACAATGGAGTATGGCCTCATGTCTGCAGCGATAAAAATC<br>CCGATAGATTTGGTAATCGCTTTAGCCTGTTCTGATTCTGCTGCAAAGAGCTGTTTAAATGCAGCAGTGAGAAGCTGCTGTGTTTTGGGTCCTGTC
LG23	ref-47877_28	95.083			CCCTAACATCCTTTTTAACATCATGAATCACGTTAAGAACGCTGTCAATTTTTGTAGCAAAGACACTTCTCATCATGTCCAACTTTTGAATCACATCTAGATTCTGGCCAGT<br>GTTGGTACAGATTTCTTCGGTTAGGGGAGCGCTGCATGCATTACCTGTTGAGCTAGCCACACTCCTAGCATCCCACTCGTCATCT[C/G]GTTCCCTGAGTGGCGAAGAGTCT<br>GATTTGTGTTTTATTTTCGATGGCATTTCGATCCTTAGTTCACCTCAGAAAGCTAGTGAAATGAATTAAACTTGTATAAACAAAAAGACGGATAACATACAGGATTTTAAG<br>AGAATAACGCCAGGAGCTCACAAAAAGACGTGCACTCA
LG23	ref-37411	95.612			TTAGTCTTTGCGAGGGACTTGCTCATCGTCA
LG23	ref-20437	96.253			GGCTTCATGGCGAGCACAGTGCCATACACTC
LG23	ref-63066	97.417	Chr4	1799980	GTTGAAGCTTTATTTCTCGTTAATTTAGCCACTGTATCAAATAAATACCTAGGGCTGCTTTTGTTTTCATCTAAAAGAGTTGAATAGTAAGCAGATCTAGCAGTTTTTCAGG<br>CCTTTCTGTAGGCAGAAACACTATCCTTCCATGCATTGCAAAATATCTCTAGTTTTGATTTCTTCCAGCAGGGGTGTCCAAACTCGGTCCTGAAGGGCCACTGTCCTGCAGA<br>GGTTAGCTCCAACTTCACGGAAAGTTTCTAGTATGCCTAGTAAGACCTTGATTATCTAGTTCAGGAGTGTTTATTTGGGGTTGGAGCTGAACTCTGCGGGACAGTGAACATC<br>CAGACACCACTGGTCTATAGTTTCTGTTACAAA
LG23	ref-49506	98.481			CCTTCTGAAGCGAAGCGTTTGCGTAAAAAAA
LG23	ref-61422	99.935			ATGTTTTTACCGAGTACATTGCCTGTTGGAA
LG24	ref-44382_10	0	Chr13	18501911	GGGTCGCTCTCAGGAGCTCAGTGAATTCATAAGTCCATTATAAGTCTTCGTGAAATTTCCTCACTACTAAATATTCCGCGGTCAACTGTTAGTGGTATCATAACAATGTGGA<br>AGCAATTGGCAACAACAGCAACTCAGCCACGAGGTGGTAGGCCACTTAAAATCACAGAGCGGGGTC[C/G]GCACAGAAGTCGCCAACTTTATGCAGAGTCAATAGCTGCA<br>GACCTCCAAACTTTGTGTGGCCTTCAGATTAGCTCAAGAACAATGCGTAGAGAGCTTCATGGAATGGGTTTCCATGGCCAAGCAGCTGCATCCAAGCCTTACATCACCAAG<br>NNNNNNNNNNNNNNNNNNNNNNNNNNNNNNNNNNNNNNNN
LG24	ref-49159	0.377			TCATGTGAACGCAATCGTACTAAATCGCAGAAGTAAACCACTATTAATGATGTATGATTTGATAAAAATGTGTGTTTGTGTGTGTTTCACTCATTTTTCTGAAGTGTGTGGC<br>TGGCATAATGCAAGCAGAGAGCTGTATGTGTCGTTTTTGCTCGCCTTCAGCGATCTTTAGAGCATTTGCAGTACATTCGCGGCAGAACAAAACTATAAAAGTGAGGTGAGC<br>AGGTAATCAAACCAAGTGTGAAACCACCCTAAAATCAACTTAACTCACAATGCTTTTGAGAAACAGCCCATACTGAATACTAAAAAATAAAATTGCAATGATTTTGGACT<br>ATAGGCAGAGACAGACAGCACGACACACACAAAAAA
LG24	ref-44382_2	0.894			A[G/T]AAAGTTGGCGACTTCTGTGCTGACCCCGCT
LG24	ref-10225	11.596			ACAGATTTTGCGAATGGGATGCAAATCGGTG
LG24	ref-1553	12.993			CAACTCAATGGCACAGCGACCAAATGGGCACTCGTGTTTATCTTCTCGACTGCTGTTTTCTAGAACAATCTGAAAAACGAAGCAAACAGGCCAAAGGCATTATCAATGGG<br>GACTGTGAAAGAGGACAGAAGGGAGAATAGGAAGAGGAGATGAAGATACAAGTGACAGCAGACACCAGAGCTTGTGCATCGCCAACATCATAATGAAATAAAGCAGAA<br>TGAATTTAACATCTGTATTCCCAGCTTCACACAAACAGAAACTGAAACATGATTGCTTGATGGACTTGATTGCGCATCAAGCATGTTTGATCTTTCGTTTACCATATTTGAT<br>AAGCTATTAAATTAAAATCTATCCATCATATAAACTGAT
LG24	ref-62574	14.167			AGCATGTAGGCGATTGTTGTGCAGTGAGCAG

LG24	ref-60535	17.364			GGTAAGCCTCCGACTGACCTGCAAGGAGCAT
LG24	ref-65177	17.579			TAATGTTTGGCGAGCCCAGTGCCCCTGGCGT
LG24	ref-6999	20.646			ATGGTCGAGTAGAATCTCATGATCAACAGTGTCACACTTTTATGTTCTAAAACCCTGGTCATCACATATATGCCTTCTGAGAATGGGCAAAGTGAAATTATATTGGTGCTTT TAAATCAAGAAGGCCTACAATGCCTGGAGAAAGTTATCCATTAGTGTCCCTGCTGATCTCCCACTTGCTTTACAAGCAAGAAATCTTCCAGAAGTAGACAACCCCCTTGCT GCCATTTTTTGTCATTTGAGGCAAATATCTACTAACCACCCTAGAATAGTGGCCAGTAATTCTATAGGAACAAGAAAATTACCTTATGACATGCTGAGATTGCTGATGATA CCCCACAAGAACATGCAGTCTTAAACCCTTTACCATTCTGAGCTGTGATGTCAGAAAAAAAAATTGTCTTGGGGATATGGACGTGCAATGTTCTTGC
LG24	ref-60994	23.472			TTAAAAATATCCTGGCTCTTCCAAGCTTTATAATGGTATGCATCCATCCATCATAAAAATAATCCATACGGCTCCAGGGGTTTAATAAAGGCCTTCTGAAGTGAAGTGATG GGTTTTTGTAAGAAAAATATCCATATTTAGAACTTTATTAACTAAAATAATTAGCTTCCGGCAGACGGGCGTATGCATCGATTTGCAAAACACTAGTCAATAATTAGAAGT CTAAGATGAGAAATTTTAAAAAGAAATGTCAGAGAATTTCAATATAAGAAAAGAGGAGCTTGAGTTTGTCCAAGCGTTTAGTCTTAAAGTGACAGTAGCCTAAAATACCT GTCTGTGTCATTAATGTTAAACAACAAAAGATGTTAA
LG24	ref-25087_5	26.498	Chr11	2943217	GATACACTTTGAGCTCATTATACAATCCATATAGTTGAGTGTTGCTTGCTTTTGAAGACTGTTTTTACCTACTGTAACTAGACATGTCCTGATTTTAAACGCAAGCATTAAAT TATTCAACATCTTTTCAAAAAAAGCTTGAAAGGGATGTGGTGCAAAGATCAAACTTGTGTTGATTTGTATTTGTGCTTCTGGGAGACTTTT[C/G]AGCGGCGAGGGGAATGC TGTGATGTTTTCCAGGTGCGCTCTGGGGTGGAGCTCTCTTGCATTGTTCCTTTATGATTTTGTAAATACTTTATCTGTAAAGTGAAAGATTCACTGGAATATGAGACTCATTG TTGATATTTGCCTCCAGAGATCGTACCAGGTTTATTACCCATGCCGCTCAAGAGATAAAAGTCAACATACGTGCAATACGGTTCAATATGCAAAGAA
LG24	ref-48229	28.554			CATGGCCTCCCGAGTGGCCTGCACCTTATTA
LG24	ref-41841	30.94			AGAGCCCAAAGACAAAATCCAAACTTTGAAATAAGTCCATAATGGGTCCCCTTTATGTCTGTCTTTCTGATTGTGAAGTTCCTGTTTATGTGCGCTAAATATCTTGAACTGA GAGAGTGAAGAGTGTCATTGTTCTCTACAGGTTGATTAATTGTGGTGTGACAGGTGAAGGTTGTGCTGCGCTGGCTTCGGCTCTGAGATCAAACCCCTCACACTTGAGACA ACTGGATCTGAACAATAATAAACTAGGAGACTCAGGAGTCAGTTTGCTCTCTGATGAATTGAAGAATCCTCAGTGTAAACTGGAAACACTGTGGTAAGGTTTTATAAATGT GCTTCCTCTATTTTCTTCCCGTGGTGAATAATGAA
LG24	ref-56151	34.005			GTCTGGAGCTCGAGCTTTATGCGTTATATTG
LG24	ref-55821	36.097			AGAACGCGCCCGATGCTCGTGCACCTTATCG
LG24	ref-22698_3	38.813			CAACTGTAAAACACACAAACTGTAAATGTGTTTTATTTCCTCTTTAACTTGCTAGTCTCTTAAATGCTGAATGTACCAAGTCATGATCTTGCACTGTCATTTGTGGCGTCATA CCAATCACTGAGCAGTCGAGCAATCCATCCAGTCCGTTTCCATAGTGATGAATAGGCTGAAAGCAGGATGAATGGAGGAAAGTGAAGAA[C/T]AGATTGCCGACACCTGT GCTTGAAAAAAGAGCCTAATTGCAGTACAGCATATGTCTGTATTCTGTTAATTATGTCATTGTGTCATGACACTAATCAATATTAATCTTAAAATGTTGAAAAAGTCAATAG GTTCTCTACAACACAGCTCCAGCATGATTTGTGATAAAAACCAGATGATTAAATGTCATTAAACGTAGGTACTGTCACGTATTGGTGGTTACAATGATAA
LG24	ref-53333	42.043			TGAAGCTTCCCGAAGCACCTGCCCATTGTAA
LG24	ref-46694	45.945			AAAGAGGTCCTGACGCTCTTTGCAGGCCTCTGAGGGCAGTCCCCGCTGTGGTGGAGCGACAATATCTCGCTTTTCCGCGGAGCCCTCAGGAAATCGATGTTTGCTTCCCAC TGCCTTGTGCACTTCGGGATGCATTGGTTTCCAGTGAACGCAAATCGAAGTGTCCGCCTGAAGTCATTGCAGCCCTGTCGAGTTCGTTACCTCAGATGAGGCCTGTAGAAA ATCTTGTTTGGTTTTCCCCTGACGACCAGCCGCTTCAGGGCACCAAACTTATTTCACACACAACACCCGAGGTCAGTCTCGAAAAACTGATCCCCCTAGTAGAGTATGTAA AAGCTTGTAGGAGTCTTCAAAATATATCTCAGCGGG
LG24	ref-59758	48.544			CTTTTTAGCTACGATGCCATCCCTAGAAAGCTCCTGCCTACTTGGTTTGAAGCTTTATTTGAACTTTTATTTTATTGCATTATATCAGTCATCATTGAACACTGATCTCTTAGA GGGTTAGTTCACCCAGAAATGAAATTTCTGTCATTAATTACTCACCCTCATGTCGTTCCAAACCCGTAAGACCTTCGTTGAATAAAGTCGTTATTTCTGTTTTGTTTTTGTGC ACAAAAAGTATTCTCGTCTCTTCATAACATTAAGGTTGAACCACTGCAGTCACATGACTGTTTTATCAATGTCTTTAGTACCTTTCTGGACCTTGAATGTGGTAATTTCCTTG CTTTTTATGGGGGATGAAAAAAAAACCAC
LG24	ref-50914_1	50.177			ATGTGTCTTTAGCCATTTCTTGTTAAAGACTAAAGGCTAAAGGGTTGAGTTAGGCAGGTCATTCCACCAGCAGGGACCAATCCAGGTAAAGGTATGTAAAAGTGCCTCTTT GGAATGGCACCACAAGATGCCATGCACTTGTAGAACGTGAAGCTTCTGGAAGGCACATATGTCTGTAGTGGTAAGTTCTATAGGCAAAC[A/T]TCACTGCCTCGAATTAGA TGCAAGCGGCTATTGGGAACCAGTGGAAATTGATGAAGAGGTGTGACGTTCATTAAAGACCATTAAAGAAGAGAGGTGTGACATCTAATAAAGTTGCAGAGGTTTGACAG TACACACTGGAAGGCCTGCCAAGAGAGTATTACAATAGTCCAGTCTGGATAGAACAAGTGCTTGGACAAGGAGTTGTGTAGCATGCTCCGATAGGAAGGGCCT
LG24	ref-50914_31	50.317			ATCACTGCCTCGAATTAGATGCAAGCGGCT[A/T]T

LG24	ref-43404	50.564			AGATAATATGAAAAGCTCACAGTTTACCCATATGTGAACTGAGGGCGAGCTACAACCTGCCACTAACTTGACAGAGATTGGTGTGCCGTTCACATCTGACCGCGGGACACGATAGCAAATACTGAGAAACCCTCAGAGCGGCTTTGATCTGAAGCAGATGTCGGCGGCGGGGGGCTGATGCACCGGCTCCGCTTATTCTGTATTCAGAGAGCAGATCAGGGCTGCGTTTCCCAAAAAACAGTAGATGCTTTCGGGAAACGCAGGCCAGGTCTATCTGCTAAAAGTCTGAAGGACGATGGACACAAATACATGCTTTCTTTCTTTGTGATGAAAAAGTAAAGGTACGCAAGAGAAAGCACTCGAAGCATT
LG24	ref-72663	51.305			ACAGTCTTTACGAGACAAATGCTCATTAGCC
LG24	ref-28381	57.924			GTTATGAAACATGGACGCGCATTTACGCCGACCCCTAAACTCACTCCAACGTACTACCCAATAGTAGCATATGTGTCACTTCTGTATGTCACGATGGATTAGAAGTGGAACAAGGCCAACAAACGCAACGTCAAAATTGATGGACTCTCAGAGTGCGCATGCGCTCCTCCTTCAGGTGAGCAGAAAGTTCGAATTAGTAACGCATAAAAGTTATTAGCTGTCTACTTGGTTATTTGCGTTTTTTTCCCCCCGAAAGAGCCTATCGGTATTTTTTTCTGTGAGTCAGTTTACGTCAACATGCAGCTGCTATAGGCCTACTTGGAGCTGTGGTTATCACGTAAACAAATAGCATAAACAAGCGAACAACTT
LG24	ref-45716	61.48	Chr11	17630830	GTTTATTGAGCACCAAATCAGCATATTAGAATAACTTCTGAAGGTATCCTATGATACTGAGGATTTTAAAATAGAAAACAGTTGTTTTAAATTAGTATAATATTTCATAATATTACTGTAGTTTTAATCAAGTAAATGCAGCCTCTGTGAGCATAAGAGAATTCTTTCAAAAAATCTTACCGACCCTAATCTTTTGAACGGCAGTGCAGGCGATGGTTATGCTGTAATTTTTCCTCTTCAACAGAGGTTACAAGAAGCAGAGACCAGAAACCAGGAACTTAGCCAAAGTGTTACGTCAGCAACACGGCCCCTCCTGCGGCAGATAGAGAACCTCCAGGCCACACTCGGTGCCCAGACAGCATCATGGGAAAAACTAGAGAAGAACATCTCTGACCGCTTAGGTGAGACTTGCTTGTTTTGAATGAGCATCA
LG24	ref-55042_18	62.161			AGGTCATGACCGATACA[A/G]CTGCAAGGTACACT
LG24	ref-55042_14	62.179	Chr9	45427355	AGGTTTCAGGTTCAACCTTTGCTCCATTTTCTTTGAGCACTTGAACAAAGTAAAATCATTTCCGAACATCATTTACAACTATATCCTACTGCTTCTCCATTTAACCATTGCTGCTGATACGTACCTTCTGTTTTTCTTTACATTTGGCCCTAAAGCTCAGAACTTCCTGAGTGTACCTTGCA[C/T]TTGTATCGGTCATGACCTTCACATCTGCTAGAACCTTTGCTAACCTAACAAGTAAATAAAAAACAAGAAATAAACACAAGAAAACTGTTTATAATATTTAATACATTACTGTTAAAAAAAATTTGGTAACACTTTAGTTTAGGGGCCAATTCTCACTATTAACTAGTTGCTTATTAGCATGCATATT
LG24	ref-42871_16	63.173			GTATTAATTTCTTACTGAACCCAGACATTTGAACAGCAGTGTATGTTGACCAGCATTAGATTAAAAAAAAAAATAAATAAATAAATTAGTTTGTGATCTGCAATTTAAGAGGGAGCATCTATCATTCTGGTGTGACATTCTTGTGCAAGAACATAATAGCATATTATTTTCACATTTGTGCAATG[A/C]CATCGTGTTGTACGAACACCTTCCGTTTAATCACTGGAAAGGTTATGAAAGAGAATTACTCTAAGATGCCATATGGTGAACTGCACTGAACATGCCTGCTCAGCTGTGCAGCTCGTACCTGTTAGCTACTCCCCAGGGTGAGATGCACGATTCCTGTGGCTGTCTTGTTTTCCACCTGCATATC
LG24	ref-17344_27	64.023	Chr11	9740766	TACTTTTCAAATGCCTTTTATGTATCTTAGAATCTTGTGTATCTTAGGGGTGCTCTCCTACACCATGGATCCACAGGAATCCTGGCTGCTCCCTGGCCCTTCCACCATTAGACACAGAGCCTGCGAGCATGTACGCGGCAGCCAGCCAGAGTGTCACAAAGAGAAAGTGTTGCTCGGCGGCAGCGCTCTCGCGAA[A/G]GTCATTTAAATTCATCAGCGGCCGACAGGCAAACACACACGCACGGACAGACAAACAAATCGCACACAGAAGCAAACAAACAACTCCAGACTCATTTTGATAAGCGAAATTAGGAGGAAGGCATGGGTGTTTTGTGCAACTATCATAACACGAAAGGGAGAAAGACAAGCAGAG
LG24	ref-12964	64.761			AATCTGAAAACGACATCTTTGCGTTTCCATC
LG24	ref-52404	65.658	Chr11	11049886	TGGCCCTTCACCAGCTGCCCCATAAATCCTCCACTGTTGCAGAACACCCTTCTTTACCACCACTTTTGAATCCACCACCGCTAGCTTATTATTCATCGCTCCGTACAACATGCTTCCCCCACAGCGCCACCACATGGTTGGGAGGGAAACATCACACTCAAAGATGCCCACGTCAGGCTGCATCTTGGTTGTGTTGATCCCTAGACATCGATTGGTGCCCAGATTGTAGAGACGGTGCCTGGATACCCATTTCCAAAGCAGGCCGGCAGATGGGGGGTCACAGCTGGTCAGAGATAGACCGGATGAGTTTGCTGAGAGGCAACGCTCCTGCGATATACTTTCCAACCGGAAAACACCTTTACCTGAGTC
LG24	ref-45570	66.261	Chr11	10736578	AAGCCATATGTGGCTACTCTTTTCCTTTCTAACCTGTTTGAGCGGCGCTGCATCCTAAATGGCACCGTCATGCTGGACATGAGGGCCAACTCACTGGAGGAGATCGCAGGTACAGAGTCAGAAAACACTGCATCCAAAGCAATAAGCCTTTTGATTATGAAAATATTGTGCTACAGGTGCTGCTTGATATACTTTTTAGGGTGGATTTTTTGAGATCTGTGCATGTCTTTCAGCTAATGTGCTAATGAATGCCAACAACTCCAAACGTAACAGAAAAGGAGTTTGTGATGGTTCTCTTTCAGCCCTGAATCTAAAAATTAAATAATCATAATAAACTTTAACAGTGAAATTAGCTAGATGCTAGTGTTGTAGTCAAGACCACCTAAACCGAGACCAAGACCAGTGTGTGTCGAGACCAAGACAAGACTAAG
LG24	ref-4302_16	67.368	Chr11	10661588	ACAGAGTGAGAACGAAGATAGTGAAGTTGGCATTATTGAGCGTACAGTATTTGTCACTATATGACATCCATATCAGATATACCACACACTGAGATCCTCTTAAAATAGAGTCCAGTAATCAGATTACAGCAGATGCTGCTTTTATCACGCAGAAATTATCTGAAAACACAATACACCTTGCACA[G/T]CTGTCGGTTTTGTCTCATACTGTAAGTAAGTAATATTAGATCTGTCTGTCTGCCTGTCTGTCTTATTTATTTAGCATTAATAATAATAATGGCAAGTAATAATGGTAAATAAATAAATAAAATAAAATAAAATAAAATAATCCCATGATTTCCAGATATACAGCATAACATTTTTAACATTTTTA

LG24	ref-5578	68.016	Chr11	7729026	ACCTTGGATGCAGGTGTGCTTTCTGGGTGAATATTGATTATGAATTGCCTTGCTGATGGCGAGAGAGTACCAAGCGACTAAACGAATGGGTGATCAGGGCAAATGATTTCT CTGTGGTTTATTTGGCCTTATTGAGATTCAGGACGAGTGTTACATGTAGGGTTATAATATAGGGCATCAGGGACTGCTCAATTTATATGACCACAGTTACGAATAACGTGC ATTTGAATGATCTTTTTACACAATTATATGAAGAAATTCAAAGATTGAAAAGTACTTCAGGGTGAATCGAACATTCACAGGAGTAACAAAGCTCAGATTTTTTCCTTTCAA ATATTCAGCAAATAAATGATTTTTTAGTCTGCACATTGATCGCAGTGTCGAGATAAAAGACAACTCTAAATTAGAACATCATCAAAGCGAAATCTTC
LG24	ref-39438_2	68.541			G[A/G]CAAAAAAACGATTTTGCTGCTATGTGAAGA
LG24	ref-72002_10	68.656			TTCGGGGAGGCACAGTAGACCTGTTCGTCTCACAGGAATGCTTTGGAGATCAGCAGAAGGCAAAGGCTGTCTCCAAACAAAGAATGGCCCATTGGATTGTGGATGCCATT CTCTGGGCTTATCAGGCCCAGGGAGTATCATGCCCACTGGGAGTGAGGGCTCACTCCACCAGGGGTAT[C/T]GCAGCCATGTCGGACTCTGGTGAACGATGCTTCATTAAA AGACATATATAAAGCTGCGTGCTGGGCTTTACCCATCACTTTTGCGAGATTTAATAATCTCTGCATGGAAACAGTTTTGTCTCGAGTCTTAGGTAATAATGGGTAAAACTCG GACTAACCGCCCTCTACAAGGCGCGCTTATGCTTTGAACT
LG24	ref-33267	68.882			CTCTCTCTGTCTCTTTCTCTCTATAAAACACTTTCATACCTCTGTTCCCCTCCCTTTAGTTTTATGGCTGTAATCTGACACAGTTTCTGTGTCCTATCAGCACCAATGACAAAT AAGCCCAACAGCAAACCACAGTATGTCAGTAAATTTCTCTGTCAAAGCTAAGCCAATAGGCGGCGAGCATTAAATCACACATGTCACATTGGTGGCCAATGGCATTGCGA AGGCAATATATCTGTCAGGAAGCAGAGCCTCTTAATGAGTGCTGCATTGCTGCTGGAGGAAGCATGAAGAAGGAAAACGGGTGAAAACATCCACTGCAGTTTGGCTTGTG TTTTTCTCTTGTGGTTTAAAGGTTGAGCTTATTTGATCCAGTGTGTGCCTAATAACAACAAAGTTAGGATTAGAAAACCACAGAAGTGTTTCCACTT
LG24	ref-70918_9	69.091	Chr4	50725650	ACTTAATGCACAAGTGTTTGTAGTACATAAACCAATCACAAAATTGTCAAAAATATAGGGGAAAATATTAGATATTGGTTATTGTTCAGCAGTGTATTTGATTTCTTATTAA AAGCAAATAGAATTCAAATTAAAAGCAAGAGATGAGATTTAAAAAAAATCTATCAGTTAGACAAAGGTCAGATGAAAAGTGGTATTACACCCGACA[C/G]CCGACATTTC TGCCCCCATCACTTCTGAAATGATGGCTACGCCCCTGTTCATGTGCTATCAGAATTATCGTAAATTGGAAGCTGAGGGATATTGAAAACTGAAAGTTGAAGTTATCTGTTTA CTCTGAGCAAACTTACCCTGGTATGTCACATAACCTGCTTTCTGGAATACCCCCTAGGTGTTTCTAGTGCTACTGTTTGAGCTACAGAAATCTGGAGAGAG
LG24	ref-51901_19	69.363	Chr11	17073927	ACAATGTTAACATATTTATGGAGGGGGAAGCTGAGATTATGGCCAACTCATGAGTCCTGGTGACAGGTACTCGTTTAGACCATCTCCTCTGCCATTACTACCCCTCCCTGAT CAAATGGAGACCATCTGTCTGCATAGCGACACAAACACACACGCAGACAGATGTAAATGCCCAAACAGCACGGCC[A/G]TCGATGATGAGTCTCTGCATCTCCCAAGAGC CGCATCTGCAAATAATGATAGATGCTGTTCATTTTTTCAGGGATTGATCCTGTGCATCTTCTCTAGGCCGGTCCTGGCTACAGCCTCTAAGCACTCTGTTCTTCGGCCCCATG GCTATCACACTCGATCCTGAATAATAAATACCAGCTAG
LG24	ref-31786	69.479			AGAAGGGCTCCGATAGCGATGCCATGCAAAG
LG24	ref-22450	70.142			TGAATGCGCGCGATCCATCTGCACGCTACTG
LG24	ref-13195_26	70.364			TCTGGCCCGCGCCCGCAGCCATGTTATTTTGAAAGCGGCTGGTTCTGAAATAGATCTTCGTCTCGTGGTGCCGTCTAATATTATCTTAATATTGTCTACACCAGATGTGACTT TTTTCGCGTCACATTGCGCCGCAACAGCTAAAAGCTGTCTACACTGAATGCGACAAACCAACCGTTGCAAAGCACTCGGATT[A/G]CGTCAAAAACAGAATGGGGAATCCG TTTTCTGTCGGGAACGTGTTGTGTCGCGTGACTTGCGTTGCACCGCGCCGCATCCAGTGTATAGTCAATATCACTGATTATAATGGGTTCTATTATCTTATGACGCGTCGCA CCGCGCCACTCGCGTCAGTGTTATATTCTTTGGAAAG
LG24	ref-71891	71.215	Chr11	41622950	AGAAGATCAAATACTTACTGTATGATTATTTTATCCTGTATTTCTACTGACGAGCAAATGTTGATTTTGGAGAATGTGACAGTGTTTACAGAAAAAATGGTAAGTAGGGCA AGCAGGGTGGGGCCGAGAGCCTTGAGAAAGGAGCAAGGCCAGTGGCGTGAATGCTAATGAGCATCACATGTGTGCCACAACAAAGTCCCATGGAGGCCCCGACCTGCTT GCCACAGTAACACTTTATTATAAGCACCCATTAGTTAACTAACTAACAATGAGCAATACATTTATTACAGTATTTATTCATTTTATTTAAAAATACAAATTCATGTTAGCTC AGGTGCTTAGAAGCTTTACGAATCTTTTGTTTCGAATCAGTGGTTAGGAGCGCATATCAAACTGCCAAAGTCACGCCCCCCAGTGGTAAACCATTGAAA
LG24	ref-65806_18	71.468	Chr11	24821304	AGTGTTAATCACATAATATTAACCTCGTATGACCCTCACCATCCTTTGGTTGAACAGCTGAGTACTAGATGTCTTCAGTAGTGCTGGTTGGATCTCTCCATAATAGTGTTGTT CATTCTTCAGCACTCTCTCAGTGTTGTGAGATCGGTTTTCCAACAACGACTCAAACTGTGTCTTCAGCACAAG[G/T]CTCGGCTCCAATACTTGGTAGAGTGTTTGTGGCTTT TGGTAGCAGTGCTCACTGCTAATGTAGTCCTGGAAGGATGAAGAGAAGGGCTCTTTACGTAAAATGGCTTCTGAGGATGCTGATGAAGAAACTGTTTTTGCGTCTGGAAGA ACCATGCTTTTCATCCTGGATTTCTCAGTCAGCTTC
LG24	ref-65806_24	71.687			GTATTGGAGCCGAGGCTTGTGCT[A/G]AAGACACA
LG24	ref-42386_1	72.669	Chr11	26376946	TTTGAAATGGGTCAAAGGGTCAGAATAGTGTTTACAGTAGTGCTGTGGTTTGCACTGTAGTGTCACATAGACAAGAATCAACGGTTTTATTTCGATTCATACCAGCAAGGG AGGAACAGTGATGATGGAGAGAGAGAAAGAACGGGTACGTCTGGGTGAGCCTGCTTCCTGTTGCTGTTTTTCCCTCTCTCTACTATCCCTC[C/T]CTCAAAGGTCGAGTGGCCT GCTTAGCAACCAAAGGTCTCCTGGGAGGCGTAAACCATGTAGAGGAAGCCATCTTCATCTCGCTCTTGTTCGTAGATCTCAGAAATGGGGGTGGACACACTGACCATGCTG TGCTGATTGACAAGAAGGAAAAAGGCCTGGGTGGGGTTGAGCTGCAGTCTACGCCTGGAAAAGAGAGTCATTTTGATAACTGATTATAATGACAAAAACAT

LG24	ref-6252	72.873	Chr11	31693787	CAATATGTAGTTGTTTTGTTTGTGACAATATCCGTTTCAATTCAAGAGATTAAATCTTGTGTAGAGGGGCGGACACAACATCGATCTGAGAAAAGTTTAAATAAAGAGTGATTGATGACTTCTAACTGGTTGTGCCACTATACTGAACGGTAAATTGATACAAAAGCTGTTTAGAATTGGCAATACAGTCGCAGCAACACAGATGGTATGCGTGTTTGTGCATGGAATTACCACTACTCCAAACTGCTCCGGCTCAGAGAGGTTGGCATATTCACTCTTGTACTTGACCAGTGTGTTCAGCTGCTCCTGTTCAGGCAGGTGCTTTACAAGATTCTGGAGGGAAGAACAAGACAAAGAACTGTTACAAAG
LG24	ref-57230_23	73.381	Chr11	22158200	CTCAGAAGTCCACCGAAGAGTCAGAGTTGGGTGTTCTCTTATCTTGTATCATTGTTGTATCAACAAAACATTTCAGGCTCAGCCCTGAACATAGTGAGTGACGTATATATTTTGGCATATACTGACGCCGTTCTGTGCGTATACACCACCTCCTCCTGTGACGTTGTTCTCCCACACCGCCCATCAGCGAATGCCAGGCATACACTGCCCCGATGGAGATGC[A/T]TCCAAACAGAGCTAGCTATCAAAGTGCCCCATTGTCACAGTAGCCAGTGAATTCATGGCATCTCTACATCAAAGGATGAAGAAAAAATAGAAGAGGCCACAAAGCTTATGCACATACACACAAACACACCTGCATGCTTATAAAGTCTCATAGCATTCGGTATATCACTATGCTTGTGTGCCGCCTTTACACAGCAAAGCTCAATACTG
LG24	ref-14667_3	73.652			GG[A/G]ATGGAGTCGAACTGTTTGCATGAGCCGTG
LG24	ref-9069_27	73.75			TCAAGACCTTTCCTTTGTGCAACGAAGTCTAGGCCTCAAATGGGACCTTGTTGCAGATACACTTACTTTCCAAGTGGCTAATGTTGAAAAACCATATACTCGACGTGGTGTGTCATCTGCAATCAATAGCCTATTCAACCCGCTTGGGTTTGCTGCTCCTGTGAGTGTTCAAGGTCGAAAAATTCTAAGAGATCTGACCAGTGATGTCTGCGATTGGGATGCACCA[C/T]TCCCTGAACACAAGTTCCAGAAATGGCGAAAATGGTGTATTTCTTTACAAGATCTGAGTGTGGTGAAGATTCCAAGAATGTACACATCAATTCCTCATTCACAAGCTCAGGAAGGAGAAGTTTGTATATTCTGTGATGCATCAACTAAAGCTATTGCTGCTGTTGCCTATTTAAAGGCTACAGATGCTCAAGGACAGGCTGAGCT
LG24	ref-27498_10	73.961			CCACTAATGGAATTGAGAGAAAGCTCAAAATTAAAAAAGATTTATGACATTTTGCTCCTGAACTTTTTCCAGTCGTCTTTTTTGTTCCCCCCTCTCTCTCTCTCTCTCTCTCTCTCTCTCTCCTTCCAACTAACTCCATTAATTCCCTCTATATTTGATTAGAATGTAAGGGCTGCAT[A/C]GCAAACCATTCGGTGAGAGGTTGAGTTGTTCTCGCCTGGAGTGTCAGTGTGTTACAGCTATTCACTCGAGAAATGGCTACCAGTACTAACTTACTTACTATTTTAGAGCCCAGCGGGGCTCATGTTTTATATCTTAGCTACAGTATTCACACCAGGTATTAAAACATGACAAAAAAGCACCATATGAAATG
LG24	ref-51369_5	74.008	Chr11	34757658	TTCTGTATTTCTGTGTTAATGTGCATTATTTTTTAGTGGATCTGTTTATTTGTTTGGTTTATAGGATGATGCCCGTCAGTTGTTTGCCCTGGCCGGGGCGGCAGAGGAACAGGGCATCCTGCCTGATGACCTGGCAAATGTCATCACCAGACTGTGGGCTGATGGAGGGGTACAGAACTGCTTCACCCGCGCTCGAGAGTACCA[A/G]CTCAACGACTCCGCTGCATAGTGAGTTCTCGTTCCATTGTTGGTCAGAAATGATATTGACCATTTGTCAGTGACCATTCATCAATATTCATGGCAAGCTAAAATCATAACTCATAATGCGCAGCCTGTCAGGTGATAAATATGCATTTACAGATGGAACAGGGTGGTGCACTATAGCATTGTGGGTATTTTTCTTGAAATATAAACATATTCAAATATTCAAAGGTT
LG24	ref-14034_16	74.311			CGCGTGCTGAAATAGCCTTCCTCGTTTCCGCGAGGGATGCTGATGATGATGTTGTCGCCCGAGTAGGCGGGTGAGGGTCCGATGCGGAAGATCTGAGCTGGGATGATGATGCTGGTCTGGAAGCTCAGCTGGTAGTACGTGATGCGTAATGGCATAGTCTGGCATTGCTGGAAGTTGGGGCAAC[A/G]CATTCGCTCACAGCGTCTAAAAACATGGGAGAGGTGAGAGTTTTGGTCTATCGAATGTGCAAGAATTTGTTCTGCAAAGACCCTGGCCGAAGTTGTGTTTTAAAGTCAAACATCTAAAATGGTACTTATGAAATTAACAGACACTTCTAAAGGGATAGTTTAACCAATAATGAAAATTCTCATGT
LG24	ref-32333_1	74.418	Chr11	32537753	ATAATCCAGGATAAAGTTTAAAAGTCATTTTTTCAGTGTTCTGGGCCAAGATTGTCTGCTAATCACAGATCCAATCTGGAATCTGTGGCTTACTCCAGTAAGGTCTTTATGGATGCCGTTGCTACGGATTGTTCTAAAGCTCCCACGGCAACAGCCTCCATGCAACCTGCATGCAACCGGCCTGAGCAGAATTCCAAATT[G/T]ACCTTGGAGCGATGAGATTGCCATAGGCATTTTTATACCCCACCTGGCCAGAATTGGGCCTATAACAGGGCAGTGTGAGAAAATGCTTTGCTTTTGATCCACTGACACTGTCTGCCATGGCTTTGATCTCACTGTAATAATCTCATTGTTTATGGAAATGGAATTTTGTTTTCCAGGAAAGTTGTCACTGTGCTTTCAAATGAACTGCGCTGAGAATGTGTGTCCTGGAGG
LG24	ref-50694	76.079	Chr11	36325262	TTTGATTTTCCTAAAGTCTGTTAATGTTTCAGTTATTTTTGGCACATTTAAATGTTACTTTTACAAAACTACCTTTCATCTTTCTGAGTCTGTGAGTCTACAAAAAATGTGTTTGCCCCAGATCTGCTGTCCATGTGGTTTTATGATGTACGTTCATCTTCATAGAACTGACTGCACAAGCATTAGTTTCCTGTGGTCTGTTTCCTAATCCACACATTGTGTAAGTCTCCTCTGTTTTCTCACTCGCTGTAATGGAGTTGTTCATCTTCATTGCTTTTCAGCTTCTTATGGAGGTTCAGTCTTACAGTAAGTGATCTCTGCTGTTTAATGCTGATTAAATACTGTATGTCCACATTTGTAGTTAAAAAT
LG24	ref-31409	76.399			CATTTGTATACGAGCAGGCTGCTTTATGGAA
LG24	ref-55760_30	76.623	Chr11	38246922	GGGTAAAAGTTTTGCTTGTTAATGATGTAGGCAATGCATTTATTTAAATGTGCAGAAATGCATTGAGGGTGTTTTTTGCTTTCGTTGGATGTTTTTCCTACTCTTATAAATAATTCATGACCTCTTTTACTCTAACCAATCACGTACTTCGTTTTGGAGCGAGCCCCCGCCACGCAACCAATCGGAAGCCAGGAATCGGATCCGTGCCCTCGAGTACATTGCGATGCGT[A/G]TCATGTGACAGGACTCTGAGAGAAATGCCAACATGGAACCGTGGCAAGGACGATGTTCTTCCCAACAATAACACTGAAAGAGACATCTGGATTCATCCTATGTCGTACACCCACAAGCTGGATATTTCAATCCATATTTACACAAGGTAATGAGACCTTATAAATATATCGTTTTACTTTCGATAAACCAAATCGAAGGGGT
LG24	ref-70081	76.903			ACCTAGACAACGATATCTTTGCGTGGGAACG

LG24	ref-52236	77.612			GAGTCAAGCCTGTATTCTTTTATCAGTAACCATTGCAAAAATGACCAAAGTAATATCAAAGATGGCGATATCCATAAAACTGAAAAGTCTTATCACTTTTGACATGACAAGTCCAATCGAGCTGTGAGTTCATCTGTCAAATCTTCACTAACCTACAGCAGCTTTTATATCTGGGTGGAGCATTTCAGTCGTGTGTAGAACACAAAACTGACGGGAGCAGCTCTGATTGGTGGAGAACAGTGACAGGATCTTAAACCTTCAGATGACACACAGCTCATATCTCTGCTGCTGTAAGTTACCAAAACACACATGGAAGCCTGCAGCACACGGTAGACATGTGTTTGTGCAGTGCAGAGTGACTCTGTTGCA
LG24	ref-66697	77.932			GGTGTACTTGTGAAAGATGGGCTGTTGACTTTGTTTGTTGATGGGAAACATAAGGGCTGCAGAGGGACTGATGGGGGTCCAAAGCACTCAGAGCAGTCAATATATCATTACGGTCACTCATGAGGGGGTCCATGAGAGCCTGCGTGTCTATAACTCTAGCATGGGCAGGATTACAAGCGGCAAGCTTGTCGTTTTAATTCCTTTAGCTTCACAGCTTTCTTCACGGCCATTGTACCATTCAGAGGGTGGGAGGCCGGATCGTTTTTAGAGGGTATTTCTCTCAACATGAGAGCAGAAAGTCAATAGTTTAACTTTACAGAGGTCAGTGCATAGACATGTTCTCACTAAATGCATGGTACTGTTACTGTA
LG24	ref-16966_18	78.078	Chr11	39141072	TTGAGAGGTTTCCTTGGCGATAAGGTTCACTGACATCTTTTGGACTGTGCGATAGCTCATGCTAAACGGGAGGGGATGACGGATGGTGCTAGCCTTAGATAGCTTTGTCAGTGTCACAGCCCCCGTTCCCCGCTGGGTCCCGAGCAGCCCGGACCGGGCGGCACTGGGCACGGAAAGAAGCACGTG[C/T]CTCGAGTGTCTGGAGAGACACCGGTGACAAAAAGCAAGCTCTCGATCTCCTAACTCAAATAACTACTTGCCTGTATCCCATATTTCTCCGTGACCTTAAAGGAAATGACAGCTCTCATCTGGGAGTTGGTGCCAGGTCAAAATACAATTGTTTTTTTTTCTCTCGCTACCCTTCTCCTCTAC
LG24	ref-7617	79.462			CTGCCGCCGCCGAGCCCGCTGCCCCTCTTCA
LG24	ref-25418	80.781			ACAAAAAAAACTTTTGTTGTGACTTTTGTGCTGTTCCACAATCCAGTAAGCTGCCTTTTTGCATACTGTCATAATAAGAATGAAACGAAAACAGATCATTCTTATTGACTACGTATTATAAATGATTTATCCATGCACATGATATGCCCTTGTAATCTTTATTCAAAATAACCCCCCTTGCAATGACTTCGCTTCTCTGATGACATCAGTGTTGACAATTGCTTTCAGCTGAAAGTAGCTAAAACTGGTCGCTTAATGCCGCTAGATGATGTCATATTGGCGAATCCACTGATCTGTATTAATATAAATATAGATCAGTGGGCGAGTCACGGAATCTAGATAAGGTTTGTCCAAGAAGTTGCTAGATTT
LG24	ref-40257_17	81.484			GGAGTCTGGCTGACTCTGGCTGTCCGATGAAGTCATGGAGGGCTCCTTCTGGCACTTGTCGGCGTGTAATAGACACTGATGATCAAGTATGCCGTTCTCTGAGGAATCCTGCACTAGCTGAAGAGAACAAGAGATGGGAAAGATGAAACGAGGATGTGTGGAATACTGTATCCCACAGTGCAATG[C/G]GTTCGTTTTGAAATTCCATTACTATCTTTTTCCACATTATTAGTAACACCGTGTCCTAACCAGACGCGCTGCGACGCCACAACACGCGATAAAAGGTATCTTTTCCATTTTAATCAGAGTTTTTGTCCTGGCTGGAACAACAACACTGATAATCTCAGGTCATGTTTATGTGCTTTATTCAATG
LG24	ref-42901_28	82.322	Chr12	43798526	TAATCTGTAAATTGTATCTATGTATGATTTTTTCCAACAGTCCTCAATGAGTAAGCTCCTTCAGTAGTTTTAATACATTAAGTTCTAGACAACTTTTAAAGTCACCATGAAATCAAAATTGACATTTCTTATTTATCTATGGAACATATTTTTATATATATGTGTGCCCTCGTAATCTTTAGTCAAAATAACTTCCCCTTCCTCTTGCAGCGACGTCACTGCTCTGAA[A/T]AAGTGTTTACTGGAGCGAGGGCGGGGCAACCTGTCACTCAAATTACATCACAGCAATAGCAAACCACAACCATCCAATCAATTCCCAATGGACAAAATCAAGTCCCGCCCTACATCTTTTCTTGCTCGAGAAGCGTTTCATTCGGATCAGAGCTAACGGCTCTGATTCGGATATGTGTCATGCAGAATAGGTAAGAAAAGACT
LG24	ref-5686_7	83.96			AGAAAG[C/T]CCGCGAACAGTTTGCTGAAGACAAG
LG24	ref-66423	84.799			TCCCTCACACGTCACTGGATTTCGCCCGCAAAATTTTTCACCTGAAATGGTGCATGTAAACGCAAACTAAGGCACAAAGCTATTTATTTAAAATAAATGCAGCATATAGCGATAAAATTGTATATGAATAAACTTTGGCGTAACTTTGTGTTCAAAGTCTCTGTGATATTCTGGTCTGTACATATGTCTCGAGTCTCTACTTCAATTATTATTATTATTTGTGCTTCATGTCCGTTTTCACTGCATGAACGATAGTGATACTGATGCTTATGTACGCCAGTCTGAGAACCTATGAATGGTGGATCCATAATGGCCTCCATAGATGAACACCACTGACCTCAATTTGCGCTGTGTGTTTGGCATAGTACT
LG24	ref-35355	86.04			ACTTAGACGACGACACCTCTGCAAGGCTAGC
LG24	ref-20068	88.003	Chr11	36630517	AACCCAAATGTCTTGTATTTTATGATGGTCCAAGCTGGTGTACATGCATGTCCATTCTGGGTCCTAGTGTAACTAATAAACACATTTATTTAATTAAAAATTAGCCTTTTTTAGCCATATATATCATTAATGTTAAAAAAAAAAGACAGATGTCTGTCTGCAACTGAAAACTGAAACAGATTTAGATTCATTATAAATTGGCAATATAACCGATCCATCTGGTTGGGGTGTCTGCAATTTTTTTTTCCATGACTAGAGTTCTTCCTAGAGTTTTTTTTTTTTTTTTTTAAAGGTGTTTTCTGAAGTGTTGTGTGCTGTGAGATGATCTTCAAGTCTGCCGGGTTGAATCGTTCTGAGCTGATGACATTATTAAGTAAAACGGTTACAAGGACAGAATGAAAAACTATGAGAGAGGAAGTGATTCAGACTGA
LG24	ref-49879	90.19			CTTAATATTAATGAGCCAACCCATCACCATGAAGGATTGTCACGATACCTCATTTATTTTCATGAGCCAAGCTTTTACCATTTTCATGAAGCACGGCTTCGCCATAGTAGCGAGGTCTGTTGCGTCCTAAACCTGAAAAGCAAAAACTGGAAATAAAACTGGTTTTACGGACAGCCCAACCATCACTTTCGAGTCTCGCATGTTTGTGAGTATTTAAAATAAGAATTCCATAATGAACTTATCTAAAATTTTGCGTTTTGCCTATTGATTTAGTTTTGACGCATTGCAATATATGTAATTTTCCTGTTTAAGATTGTTACGAATCTAAACAAATATCGCATTTAAAAACTTTGTAGTCTATAACTTTAT
LG24	ref-37592_32	98.95			TGAACACTAGCGAAATGGCTGCTAAAAAAAA[A/C]
LG24	ref-37592_31	98.989			TGAACACTAGCGAAATGGCTGCTAAAAAAA[A/C]C

Supplementary Table A3 The markers within confidence intervals of QTLs for growth traits in silver carp and the potential candidate genes detected from the genome of zebrafish.

Linkage group	QTL	Marker	Map position (cM)	LOD	Potential candidate gene
LG14	qBW1-a	ref-5067_7	55.7	5.02*	
LG16	qBH1-a	ref-24639	65.346	4.21*	
		ref-40264	65.579	4.19*	
	qBW1-b	ref-67451_31	37.391	3.78*	
		ref-36807_32	37.784	3.86*	
		ref-36807_30	37.961	3.87*	
		ref-33817_9	38.207	3.89*	
		ref-29748_27	38.258	4.17*	
		ref-68349_2	38.692	3.96*	*Hepcidin*
		ref-48846	38.73	3.95*	
		ref-37915	39.35	3.71*	
		ref-15392	39.393	3.73*	
		ref-11795	49.731	4.07*	Phosphodiesterase 11a, like
LG18	qBL1-a	ref-16553	27.9	8.28**	
	qBW1-c			5.94*	
LG22	qBL1-b	ref-46998_8	49.4	5.03*	Fas-activated serine/threonine kinase
	qBH1-b			5.48*	
	qHL1-a			5.51*	
	qBW1-d			4.43*	
LG18	qBL2-a	ref-16553	27.9	6.71*	
	qBW2-a			5.63*	
LG22	qBL2-b	ref-46998_8	49.4	5.81*	
	qBH2-a			6.75*	
	qHL2-a			6.72*	
	qBW2-b			6.09*	
LG8	qBL3-a	ref-68151	64.2	5.33*	
	qHL3-a			4.96*	
LG14	qBL3-b	ref-30367_1	55	5.04*	Solute carrier family 35, member F3a
LG19	qHL3-b	ref-29737	52	4.08*	
	qBW3-a	ref-13029	79.3	3.95*	
		ref-10649	79.5	4*	

a BL, body length; BH, body height; HL, head length; BW, body weight. The digits of suffix (1, 2 and 3) indicate the growth traits measured at 6 months, 12 months and 18 months after hatch, respectively.

b *, chromosome-wide (CW) LOD significance; **, genome-wide (GW) significance.

Supplementary Table A4 Primers for polymorphism identification and expression analysis of *hepcidin* in silver carp.

Primer name	Primer sequence (forward)	Primer sequence (reverse)	Usage
Hy-hepcidin-SNP-1	CGGGAAGAGTCAAGCAGGTC	TTAGGGGTTAGGGTTCGGGA	SNP identification and genotyping
Hy-hepcidin-SNP-2	TAGAAATGTGTGCCTTGGA	CAGCAGGGATTTTGAAGAC	SNP identification and genotyping
Hy-hepcidin-qPCR	ATCCTTCAGACCGCAGCCGT	AATGCCAGGGGGTTTGTTTG	qRT-PCR
Hy-β-actin-qPCR	TCACCGAGAGAGGCTACAG	TACCGCAAGACTCCATACC	qRT-PCR

Supplementary Table A5 Association analysis between genotypes of hepcidin-g.752C>T and growth traits in two silver carp populations.

Populations	Age	Genotypes (Numbers)	Alleles (Frequency)	BL (mm)	BH (mm)	HL (mm)	BW (g)
Family A	6 months	CC (21)	C (76.3%)	174.5±8.4	$51.6±2.1^{a}$	51.1±2.4	$97.2±14.5^{a}$
		CT (19)	T (23.7%)	171.1±12.4	$49.5±3.3^{b}$	50.1±3.2	$85.4±17.5^{b}$
	12 months	CC (21)	C (76.3%)	221.9±11.2	66.3±2.9	68.1±3.3	195.7±26.4
		CT (19)	T (23.7%)	217.1±15.4	64.5±4.5	66.4±4.2	181.6±31.9
	18 months	CC (10)	C (76.3%)	342.3±19.8	95.8±4.4	104.1±4.7	677.4±91.9
		CT (9)	T (23.7%)	341.3±13.9	95.2±4.7	103.2±3.8	674.4±77.4
Population B	6 months	CC (34)	C (94.7%)	$165.5±33.1^{A}$	$50.5±11.8^{a}$	$48.9±9.4^{A}$	$96.8±49.7^{a}$
		CT (4)	T (5.3%)	$115±14.4^{B}$	$35.2±4.3^{b}$	$35.5±4.2^{B}$	$30.5±10.4^{b}$

BL, body length; BH, body height; HL, head length; BW, body weight; a,b, the different superscript lowercase letter within a column means significant difference at $P < 0.05$; A,B, the different superscript uppercase letter within a column means significant difference at $P < 0.01$.
